# A near-complete genome assembly of the bearded dragon *Pogona vitticeps* provides insights into the origin of *Pogona* sex chromosomes

**DOI:** 10.1093/gigascience/giaf079

**Published:** 2025-08-19

**Authors:** Qunfei Guo, Youliang Pan, Wei Dai, Fei Guo, Tao Zeng, Wanyi Chen, Yaping Mi, Yanshu Zhang, Shuaizhen Shi, Wei Jiang, Huimin Cai, Beiying Wu, Yang Zhou, Ying Wang, Chentao Yang, Xiao Shi, Xu Yan, Junyi Chen, Chongyang Cai, Jingnan Yang, Xun Xu, Ying Gu, Yuliang Dong, Qiye Li

**Affiliations:** BGI Research, Wuhan 430074, China; State Key Laboratory of Genome and Multi-omics Technologies, BGI Research, Shenzhen 518083, China; BGI Research, Wuhan 430074, China; BGI Research, Wuhan 430074, China; BGI Research, Shenzhen 518083, China; BGI Hangzhou CycloneSEQ Technology Co., Ltd, Hangzhou 310030, China; BGI Research, Shenzhen 518083, China; BGI Hangzhou CycloneSEQ Technology Co., Ltd, Hangzhou 310030, China; BGI Research, Shenzhen 518083, China; School of Life Sciences, Southwest University, Chongqing 400715, China; BGI Research, Wuhan 430074, China; College of Life Sciences, Northwest University, Xi'an 710069, China; BGI Research, Wuhan 430074, China; College of Future Technology, University of Chinese Academy of Sciences, Beijing 100049, China; BGI Research, Wuhan 430074, China; College of Life Sciences, University of Chinese Academy of Sciences, Beijing 100049, China; BGI Research, Shenzhen 518083, China; Women's Hospital, Zhejiang University School of Medicine, Hangzhou 310058, China; Center for Evolutionary & Organismal Biology, Zhejiang University School of Medicine, Hangzhou 310058, China; BGI Research, Wuhan 430074, China; School of Biology and Biological Engineering, South China University of Technology, 510006 Guangzhou, China; State Key Laboratory of Genome and Multi-omics Technologies, BGI Research, Shenzhen 518083, China; BGI Research, Wuhan 430074, China; State Key Laboratory of Genome and Multi-omics Technologies, BGI Research, Shenzhen 518083, China; BGI Research, Shenzhen 518083, China; BGI Research, Shenzhen 518083, China; BGI Research, Shenzhen 518083, China; BGI Research, Shenzhen 518083, China; BGI Research, Shenzhen 518083, China; State Key Laboratory of Genome and Multi-omics Technologies, BGI Research, Shenzhen 518083, China; Guangdong Provincial Key Laboratory of Genome Read and Write, Shenzhen 518083, China; State Key Laboratory of Genome and Multi-omics Technologies, BGI Research, Shenzhen 518083, China; State Key Laboratory of Genome and Multi-omics Technologies, BGI Research, Shenzhen 518083, China; BGI Hangzhou CycloneSEQ Technology Co., Ltd, Hangzhou 310030, China; BGI Research, Wuhan 430074, China; State Key Laboratory of Genome and Multi-omics Technologies, BGI Research, Shenzhen 518083, China; College of Life Sciences, University of Chinese Academy of Sciences, Beijing 100049, China

**Keywords:** Pogona vitticeps, CycloneSEQ, genome assembly, sex-determining gene

## Abstract

**Background:**

Vertebrate sex is typically determined either by genetic factors, such as sex chromosomes, or by environmental cues like temperature. Therefore, the agamid dragon lizard *Pogona vitticeps* is remarkable in this regard, as it exhibits both ZZ/ZW genetic and temperature-dependent sex determination. However, complete sequence and full gene content of *P. vitticeps* sex chromosomes remain unclear, hindering the investigation of sex-determining cascade in this model lizard.

**Results:**

Using CycloneSEQ and DNBSEQ sequencing technologies, we generated a near-complete chromosome-scale genome assembly for a ZZ male *P. vitticeps*. Compared with previous reference genome (GCF_900067755.1/Pvi1.1), this ∼1.8-Gb new assembly displayed >5,700-fold improvement in contiguity (contig N50: 202.5 Mb vs. 35.5 kb) and achieved complete chromosome anchoring (16 vs. 13,749 scaffolds). We found that over 80% of the *P. vitticeps* Z chromosome remains as a pseudo-autosomal region, where recombination is not suppressed. The sexually differentiated region (SDR) is small and occupied mostly by transposons, yet it aggregates genes involved in male development, such as *AMH, AMHR2*, and *BMPR1A*. Finally, by tracking the evolutionary origin and developmental expression of SDR genes, we proposed a model for the origin of *P. vitticeps* sex chromosomes that considered the Z-linked *AMH* as the master sex-determining gene.

**Conclusions:**

In this study, we fully characterized the Z sex chromosome of *P. vitticeps*, identified *AMH* as the candidate sex-determining gene, and proposed a new model for the origin of *P. vitticeps* sex chromosomes. The near-complete *P. vitticeps* reference genome will also benefit future study of reptile evolution.

## Introduction

Sex determination is a fundamental process in sexual organisms, influencing reproductive strategies, population dynamics, and genetic diversity [1]. Among vertebrates, the mechanisms of sex determination are diverse, ranging from genetic sex determination (GSD) to those determined by environmental factors such as temperature-dependent sex determination (TSD) [2–5]. The central bearded dragon *Pogona vitticeps* (NCBI:txid103695) represents a fantastic model in studying the molecular cascades of sex determination, as this species possesses a unique ZZ/ZW GSD system that is influenced by temperature. Normally, ZW embryos of *P. vitticeps* develop as females and ZZ embryos as males. However, high incubation temperatures can induce functional male-to-female sex reversal in genetically male (ZZ) individuals [6]. The capacity of a ZZ embryo to develop as a normal female without the help of the W chromosome suggests that sexual fate is most likely determined by a dosage-sensitive gene on the Z chromosome [7], making the complete Z chromosome sequence particularly crucial for deciphering the sex-determining cascade in this species.

While many studies involving *P. vitticeps* in the past decade tightly rely on its genome assembly and annotation, the current reference genome (GCF_900067755.1/Pvi1.1) is still quite fragmented. This reference genome was constructed with a wild-caught ZZ male individual, based on Illumina short-read sequencing data generated from gradient libraries with an insert size ranging from 250 bp to 40 kb [8]. Due to the limitation of short-read sequencing, the contig N50 of this assembly version is merely 35.5 kb in length, lagging far behind the common standard of a reference genome (>1 Mb), as proposed by the Earth BioGenome Project (EBP) [9] and the Vertebrate Genomes Project (VGP) [10] in recent years. Furthermore, telomeres, which are essential for chromosome stability and composed of thousands of telomeric repeat units (TRUs) (TTAGGG)_n_ [11] in vertebrates, are almost absent in Pvi1.1 due to the limitation of short reads in assembling highly repetitive regions. Additionally, *P. vitticeps* possesses microchromosomes [12], which, like those in birds and some reptiles, present significant assembly challenges due to their high gene density, elevated GC content, and intense interchromosomal interaction signals [13]. These characteristics make the assembly of a complete *P. vitticeps* genome particularly challenging. Although subsequent scaffolding efforts anchored ∼42% of the genomic sequences to chromosomes [14], the low anchoring percentage still limits its use in chromosome-scale investigation. For example, the sex chromosomes of *P. vitticeps* are well known to be heteromorphic based on cytogenetic evidence, implying the existence of sequence divergence between Z and W due to recombination suppression [15]. However, the PAR and SDR of both sex chromosomes remain undefined so far, which in turn hamper the search for the master sex-determining gene.

In this study, we generated a chromosome-scale genome assembly for a ZZ male *P. vitticeps*, via a combination of long- and short-read whole-genome sequencing (WGS), as well as long-range sequencing technologies. With this near-complete genome assembly, we fully characterized the Z sex chromosome of *P. vitticeps* and demarcated the PAR and SDR on Z, tracked the evolutionary origin and developmental expression of the Z-linked SDR genes, and proposed an alternative model for explaining the origin of sex chromosomes with the Z-linked *AMH* as the candidate of master sex-determining gene in *P. vitticeps*.

## Results

### The construction of a near-complete *P. vitticeps* genome

To reduce interference caused by allelic variation, all sequencing data for genome assembly were collected from a single captive-bred individual. The sex of this individual was verified as a ZZ male, as demonstrated by the anatomical presence of testes and the PCR examination of sex-linked markers (Supplementary Fig. S1A). High molecular weight (HMW) DNA was extracted from the muscle and lung tissues, which was subjected to WGS with the CycloneSEQ [16] long-read and DNBSEQ short-read technologies, respectively (Supplementary Tables S1, S2, and S3). In addition, we generated long-range sequencing data from the liver tissue by integrating chromatin conformation capture technique with CycloneSEQ (see Methods for details; hereafter referred as CycloneSEQ-based Pore-C). The *k*-mer analysis with the short-read data confirmed that *P. vitticeps* has a heterozygous diploid genome (heterozygosity ∼1.63%) with a haploid size of ∼1.70 Gb [8] (Supplementary Table S4; Supplementary Fig. S1B).

**Figure 1:**
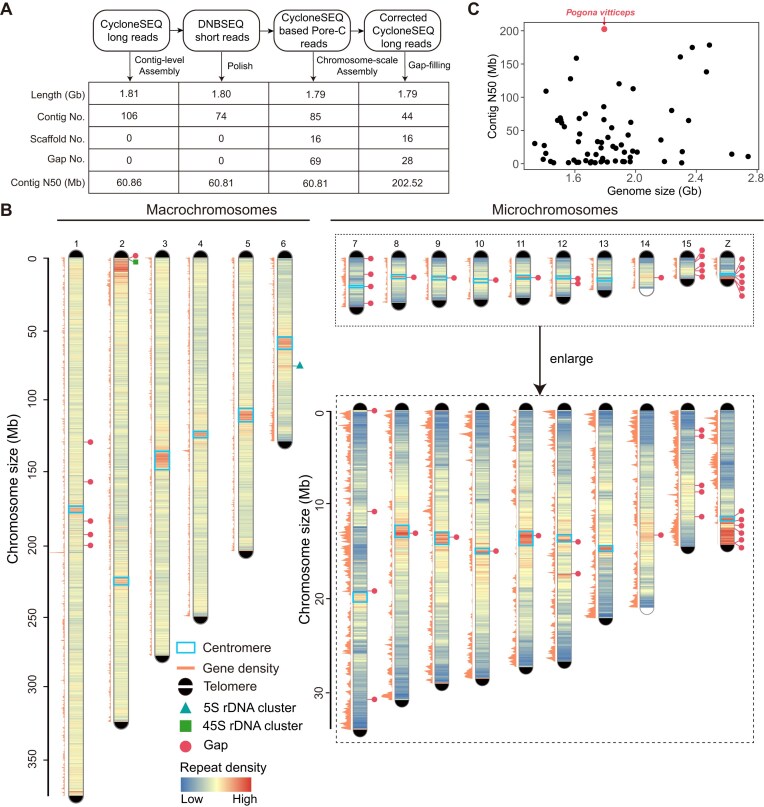
The near-complete genome assembly of *P. vitticeps*. (A) The CycloneSEQ long reads were initially utilized for *de novo* contig assembly. Thereafter, DNBSEQ short reads were employed to polish the assembled contigs. Subsequently, CycloneSEQ-based Pore-C reads were implemented for scaffolding the contigs into chromosomal sequences. Finally, gaps were filled with corrected CycloneSEQ long reads. The table presents the assembly metrics for each step. (B) An overview of the genome features. The distributions of repeat density and gene density were calculated in 100-kb windows. All the gaps in Pvit2024 are shown in red circles. All chromosomes, except for chromosomes 14 and 15, had identified centromeric regions, as marked by light blue boxes. (C) The comparison of genome size and contig N50 between Pvit2024 and 66 publicly available squamate genomes assembled by long-read data.

We obtained a total of ∼265 Gb CycloneSEQ WGS reads, with over 40% (∼105 Gb) achieving a sequence length longer than 40 kb (Supplementary Table S1;  Supplementary Fig. S1C, D). As the 40-kb+ reads alone had already covered the *P. vitticeps* haploid genome ∼62 times, fully satisfying the requirements for long read–based *de novo* assembly, we therefore assembled the 40-kb+ CycloneSEQ WGS reads using NextDenovo [17] in the first step (Fig. 1A, Supplementary Fig. S1E). This resulted in a 1.81-Gb primary assembly with merely 106 contigs (Supplementary Table S5). The contig N50 was 60.9 Mb, surpassing the majority of squamate genomes published to date (Supplementary Table S6). Before further scaffolding by long-range data, we removed false duplications in the primary assembly with Purge_Haplotigs [18] and corrected sequence errors with short-read data using NextPolish [19]. After polishing, a 1.80-Gb assembly with 74 contigs was scaffolded by ∼33× long-range CycloneSEQ-based Pore-C data with YaHS [20] (Supplementary Table S7). The order and orientation of each contig were also manually examined in Juicebox [21] to avoid misplacements. The final chromatin contact map clearly sorted all the contigs into 16 chromosomes, including 6 macrochromosomes and 10 microchromosomes (Fig. 1B).

To interrogate the integrity of chromosome ends, we next examined the presence of TRUs (TTAGGG)_n_. Only 8 of the 32 expected telomeres were presented in the initial chromosome-scale *P. vitticeps* assembly (Supplementary Table S8). To resolve this limitation, we developed an in-house pipeline to conduct local telomere assembly with the TRU-containing reads and assign the assembled telomeres to their corresponding chromosome ends (see Methods for details). As a result, 31 of the 32 expected telomeres with a mean copy number of 1,661 TRUs were patched to corresponding chromosome ends, except 1 on chromosome 14 (Fig. 1B).

After telomere repair, the chromosome-scale assembly still contained 69 unclosed gaps. These gaps were subjected to gap-filling with the corrected long reads (generated by NextDenovo during assembly) using TGS-GapCloser [22]. The correctness of each closed gap was further examined by long-read coverage with an in-house pipeline. This led to the solid closure of 41 gaps inside the chromosomes and helped 4 of the 6 macrochromosomes (chromosomes 3, 4, 5, and 6) and 1 microchromosome (chromosome 13) achieve telomere-to-telomere (T2T) gapless assembly. The remaining 28 gaps were mainly located in microchromosomes and colocalized with repeat-dense regions (Fig. 1B). Of note, a local region (0.5–11 Mb) with a particularly high density of repeats was clearly observed in almost all chromosomes, presumably corresponding to the position of centromeres (Fig. 1B; Supplementary Tables S9 and S10).

In summary, the final *P. vitticeps* genome assembly (hereafter referred as Pvit2024) was 1.79 Gb in length, with all contigs anchored into 16 chromosomes, a final contig N50 of 202.5 Mb, and the presence of all but 1 of the 32 telomeres. Although there were still 28 inner gaps remaining unclosed, our Pvit2024 assembly outperformed all other squamate genomes reported so far in terms of continuity (Fig. 1C; Supplementary Table S11).

### Quality validation of the genome assembly

**Figure 2:**
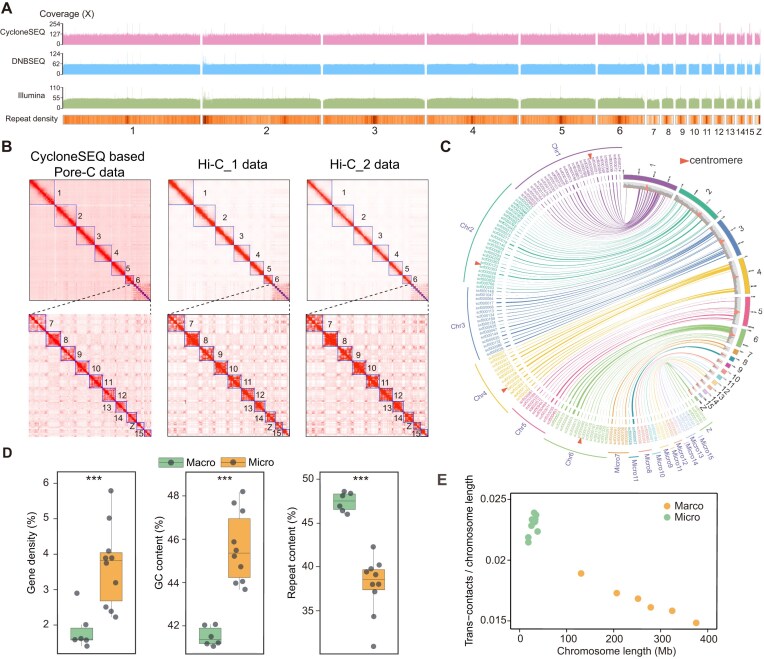
Quality validation of the genome assembly. (A) Genome-wide coverages calculated in 10-kb windows. The values on the y-axis for each coverage track denote the mean coverage as well as 2 times the mean coverage for each dataset. Tracks in the bottom panel show repeat density of Pvit2024. (B) Heatmaps of chromosomes contact matrices for the CycloneSEQ-based Pore-C library and the 2 traditional Hi-C libraries. (C) The circos plot illustrating the synteny between the Pvit2024 chromosomes (on the right) and the scaffolds anchored to chromosomes by BACs from Deakin et al. [14] (on the left). The gray tracks for Pvit2024 chromosomes show repeat density, with red indicating the positions of centromeres. The red triangles on the left mark the centromere positions determined by Deakin et al. [14]. (D) The comparisons of gene density, GC content, and repeat content between macrochromosomes and microchromosomes. Asterisks indicate the significance of differences based on Student’s *t*-test: ***P < 0.001. (E) Trans-contacts scaled by chromosome size for each chromosome, which were calculated by ∼33× CycloneSEQ-based Pore-C data. Each dot represents 1 chromosome (yellow for macrochromosomes, green for microchromosomes).

To assess the completeness of Pvit2024, we first aligned 3 sets of WGS reads to the Pvit2024 assembly, that is, the CycloneSEQ long reads and DNBSEQ short reads collected in this study, as well as a set of Illumina short reads generated from another ZZ male individual that were not used for our genome assembly. All 3 read sets revealed a mapping rate greater than 98% (Supplementary Table S5), and the aligned reads showed uniform coverage across the genome with only a few exceptions in repeat-dense regions (Fig. 2A). Second, we conducted BUSCO [23] and Compleasm [24] assessments for Pvit2024 with the Sauropsida_odb10 dataset (*n* = 7,480 genes), which represents conserved single-copy orthologs broadly present across Sauropsida (including birds, squamates, and turtles). The BUSCO and Compleasm complete scores were 97.6% and 98.63%, respectively, comparable to or even higher than other squamate genomes assembled by long-read data (Supplementary Fig. S2A; Supplementary Table S6). The consensus quality value (QV) of Pvit2024, as estimated by short-read data, was 36.4, corresponding to a single-base error rate of merely 0.0229%.

Next, we assessed the accuracy of chromosomal sorting and scaffolding by different strategies. From a technical perspective, 2 traditional Hi-C libraries were constructed and sequenced with the DNBSEQ technology to serve as independent validations of the long-range information provided by CycloneSEQ-based Pore-C. These 2 additional Hi-C contact maps consistently supported the scaffolding result of Pvit2024 (Fig. 2B). In addition, we aligned those chromosome-anchored sequences from Deakin et al. [14] to Pvit2024 and observed high consistency (i.e., sequences were mapped to the expected position in the expected order; Fig. 2C). Furthermore, Deakin et al. [14] have also located the genomic regions harboring the centromeres in chromosomes 1, 2, 4, and 6 [14]. All these 4 centromere-containing regions showed perfect overlaps with the putative centromeric regions identified in Pvit2024 (Fig. 2C), supporting the feasibility of centromere positioning based on local repeat abundance in this species. From a biological perspective, the concurrent presence of macrochromosomes and microchromosomes is the hallmark of many vertebrate genomes [13, 25–27]. According to the lengths of the assembled chromosomes, we could clearly define 6 of the 16 Pvit2024 chromosomes as macrochromosomes and the remaining 10 as microchromosomes, in line with the reported karyotype of a male *P. vitticeps* (Supplementary Fig. S2C) [28]. In addition to chromosomal lengths, we also observed other recognized features that separate microchromosomes from macrochromosomes, including higher gene density, higher GC content, lower repeat content, and more frequent interchromosomal interaction (Fig. 2D, E; Supplementary Fig. S2B) [29, 30]. These multiple lines of evidence together highlight the reliability of the Pvit2024 chromosomal assembly.

### The new reference genome recovers ∼120-Mb missing sequences with numerous genes and regulatory elements

**Figure 3:**
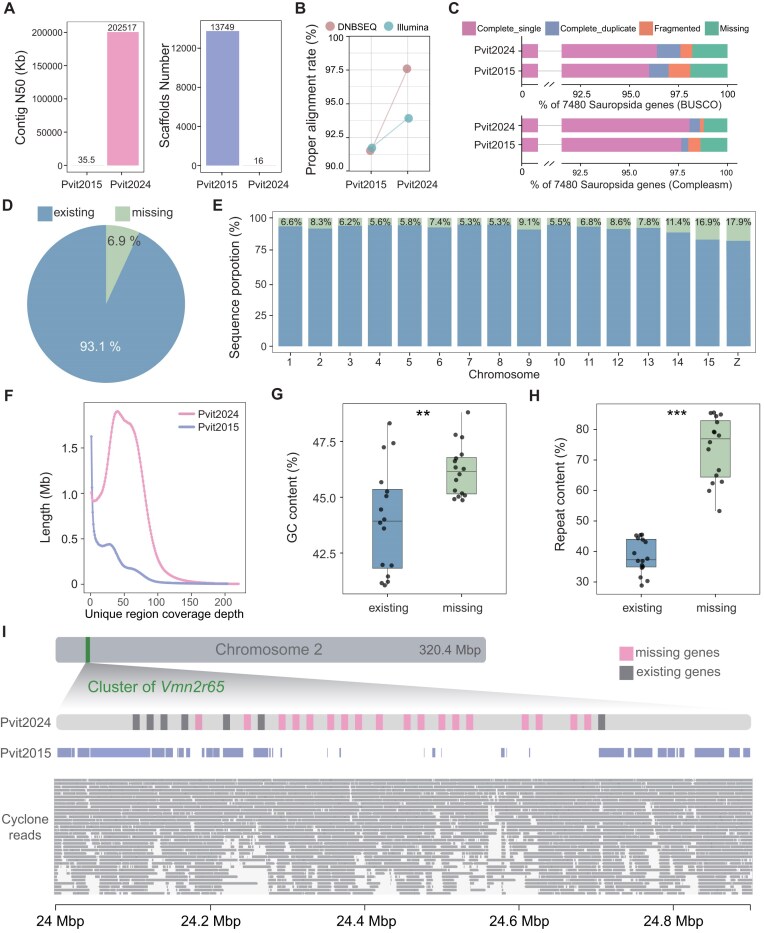
Comparison between Pvit2015 and Pvit2024. (A) The comparison of contig N50 and scaffold number. (B) The proper alignment rate of DNBSEQ and Illumina WGS data. (C) Gene completeness assessment of Pvit2015 and Pvit2024 based on BUSCO and Compleasm analyses. (D) Proportion of sequences existing and missing in the Pvit2015 assembly compared to the Pvit2024. (E) The missing rate of Pvit2015 assembly across chromosomes in Pvit2024 is depicted, with blue and green bars representing the existing and missing ratios of Pvit2015, respectively. (F) Base coverage depth distribution of unique sequences in Pvit2015 and Pvit2024. (G, H) Comparison of GC content (G) and repeat element content (H) between existing and missing sequences in Pvit2015 across each chromosome. Significance level by Student’s *t*-test: *P < 0.05, **P < 0.01, ***P < 0.001. (I) Distribution of *Vmn2r65* genes along the chromosome. The blue block shows the sequence of Pvit2015 that can be aligned back to Pvit2024. The gray strip in the bottom panel shows the coverage of CycloneSEQ reads.

Compared with the previous reference genome (GCF_900067755.1/pvi1.1) [8] (hereafter referred as Pvit2015), the contiguity of Pvit2024 was increased by over 5,700-fold in terms of contig N50 (202.5 Mb vs. 35.5 kb), and the number of scaffolds was remarkably reduced from 13,749 to 16 (Fig. 3A). Additionally, the genomic completeness, as assessed by read alignments and BUSCO and Compleasm analyses, all showed improvements (Fig. 3B, C).

To further uncover the differences between the 2 genome assemblies, we conducted reciprocal whole-genome alignment to identify missing sequences in one assembly relative to the other. Briefly, we defined genomic sequences in one assembly with no alignment to the other one by Winnowmap2 [31] and Minimap2 [32] as missing sequences in the former one. In this way, 124 Mb of the Pvit2024 sequences were detected as missing in Pvit2015, accounting for 6.9% of the Pvit2024 assembly size and affecting all the 16 chromosomes to a different degree (Fig. 3D, E; Supplementary Fig. S3A). Read coverages of these Pvit2015-missing regions were uniform and close to that of genome average, indicating that they are not assembly errors (Fig. 3F; Supplementary Fig. S3H). Conversely, only 36 Mb of Pvit2015 sequences were not aligned to Pvit2024, and the read coverage of these regions was quite low, suggesting that these Pvit2015-specific sequences likely resulted from assembly errors (Fig. 3F).

We next focused on the annotation of the 124-Mb sequences that were missing in a prior assembly, especially for the possibility of carrying functional genes or regulatory elements. The missing sequences revealed higher GC content than the genome average, with most missing sequences (∼73.6%) annotated as repetitive elements in Pvit2024, highlighting the advantages of long-read sequencing, especially for accurately sequencing regions with high GC content and repetitive sequences (Fig. 3G, H; Supplementary Fig. S3B) [33]. Nevertheless, it is noteworthy that these Pvit2015-missing sequences also harbored the intact copies of up to 576 protein-coding and 312 long noncoding RNA (lncRNA) genes—namely, they were completely absent in a prior assembly (Supplementary Fig. S3C, D). A representative example was the *Vmn2r65* (vomeronasal 2 receptor) gene family, of which 25 copies were located on a tandem array in chromosome 2 of Pvit2024 (Fig. 3I); only 7 copies of *Vmn2r65* were identified and distributed on 6 distinct scaffolds in Pvit2015. In addition, the missing sequences in Pvit2015 also led to a partial absence of 1 or more exons for up to 1,319 protein-coding and 1,651 lncRNA genes (Supplementary Fig. S3C, D), which may affect the accuracy of expression quantification by RNA sequencing. In terms of regulatory elements, we first examined the completeness of gene promoters, the region upstream of genes where the RNA polymerase binds to initiate transcription [34], and found that 2,504 (11.5%) protein-coding and 1,281 (8.1%) lncRNA genes contained >500-bp missing sequences in their putative promoter regions, respectively. We then examined the CpG islands, which are widespread in vertebrate genomes and play important roles in transcriptional regulation [35]. Consistent with the difficulty of short-read technology to sequence through high GC regions, we observed that 32,864 of the annotated 216,126 (15.2%) CpG islands were completely missing, and 7,361 (3.4%) were partially missing in the prior Pvit2015 assembly (Supplementary Fig. S3E–G).

Taken together, our genome-wide comparative analysis indicated a notable proportion (6.9%) of genomic sequences missing in the prior *P. vitticeps* reference genome. These missing sequences tend to be GC-rich and repeat-rich, and more importantly, they contain numerous genes and regulatory regions that are not accessible based on prior reference genome.

### Improving genome annotation by long-read RNA sequencing

Besides genome assembly, comprehensive and accurate annotation of gene model is necessary to realize the value of a reference genome [36]. To facilitate a comprehensive gene annotation for Pvit2024, we performed high-depth long-read RNA sequencing (RNA-seq) for 8 different tissues (brain, eye, heart, kidney, liver, lung, muscle, and testis) with the CycloneSEQ technology (Supplementary Table S2). We also generated paired-end short-read RNA-seq data for these 8 tissues with the DNBSEQ technology (Supplementary Table S3). All the long-read and short-read RNA-seq data were generated from the same individual as that used for genome sequencing in this study.

**Figure 4:**
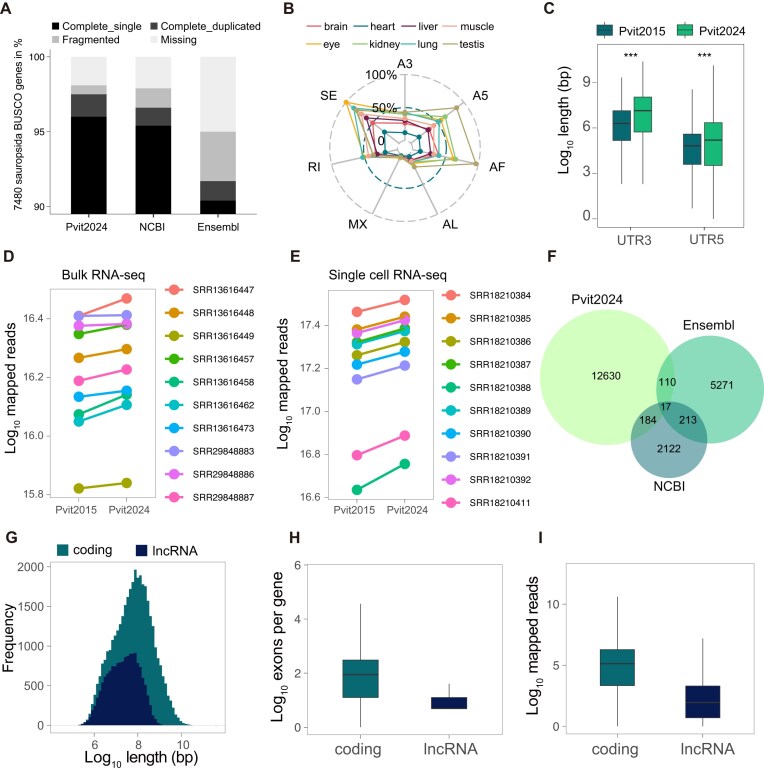
Enhancement of genome annotation by long-read RNA sequencing. (A) Completeness of Sauropsida BUSCO genes in annotations generated by this study (Pvit2024), NCBI (Pvit2015), and Ensembl (Pvit2015). (B) Radar chart showing relative splicing abundance of 8 tissues for each event type (SE–skipping exon; RI–retained intron; MX-mutually exclusive exons; A5–alternative 5’ splice site; A3–alternative 3’ splice site; AF-alternative first exons; AL- alternative last exons). Normalization here employs the maximum abundance value of splicing events as the denominator. (C) Length distributions of 5′-UTR and 3′-UTR for the Pvit2024 and NCBI annotations; Pvit2024 has longer regions than NCBI. Significance level of Student’s *t*-test: *P < 0.05, **P < 0.01, ***P < 0.001. (D, E) Connected dot-line showing the variance of mapped reads in Pvit2024 and NCBI RefSeq annotations for bulk (D) and single-cell (E) RNA-seq data. (F) Venn diagram showing lncRNA counts for Pvit2024, Ensembl, and NCBI RefSeq annotations. (G) Histogram showing the distribution of gene length (exon regions only) for coding genes and lncRNAs. (H) Distribution of the exon counts per gene in coding genes and lncRNAs. (I) Distribution of the read counts for transcriptomes in coding genes and lncRNAs.

With the abundant RNA-seq data, we first conducted protein-coding gene annotation with a combination of transcriptomic, homologous, and *ab initio* prediction evidence and obtained a total of 21,783 nonredundant protein-coding gene models (Supplementary Table S12). Up to 92% (20,002) of the protein-coding loci were supported by the RNA-seq signal (transcripts per million [TPM] >5) in at least 1 tissue. BUSCO assessment with the Sauropsida conserved genes revealed a complete score of 97.5%, consistent with that estimated for Pvit2024 genome assembly and slightly higher than the commonly used NCBI annotation (96.6%) and Ensembl annotation (91.7%) based on Pvit2015 (Fig. 4A).

Then, we leveraged the long-read RNA-seq data to refine isoform and UTR annotations for the protein-coding genes. After strict filtering steps to exclude artificial and low-quality sequences, we finally assigned 53,272 transcripts to 21,783 protein-coding loci, with a mean of 2.5 isoforms detected per gene. Up to 97% of the splicing junctions derived from the assigned transcripts were also supported by short-read alignments, further supporting the reliability of these transcript models. In addition, all major types of alternative splicing events could be identified in all 8 tissues, with exon skipping and alternative first exon usage being the most dominant events (Fig. 4B). By discriminating coding regions from noncoding parts, we could identify the UTRs for most transcripts, with 17,110 (31.1%) having a 5′-UTR longer than 30 bp and 19,161 (34.8%) having a 3′-UTR longer than 50 bp. Of note, while the lengths of coding regions were comparable between our and the NCBI/Ensembl annotations, the 5′-UTRs and 3′-UTRs in our annotation were significantly longer (Fig. 4C), corroborating a more accurate definition of transcription boundaries owing to the assistance of long-read RNA-seq. To evaluate the effect of the new annotation on future RNA-seq studies, we calculated the read mapping rate and number of expressed genes with a bulk and a single-cell RNA-seq dataset that were not used for gene annotation in this study. Both datasets revealed a significant improvement in both metrics, especially for the single-cell dataset (Fig. 4D, E).

lncRNAs are another important class of RNA molecules that engage in numerous biological processes [37], yet the lncRNA repertoire is understudied in squamate reptiles. By identifying transcripts that are longer than 200 nt and lack coding potential, we annotated 13,269 high-confidence lncRNAs in Pvit2024 (Fig. 4F). When compared with the protein-coding genes, the *P. vitticeps* lncRNAs were generally shorter in length, had fewer exons, and were expressed at lower levels (Fig. 4G–I), consistent with previous observations in other organisms [38]. Interestingly, we found that many lncRNAs were expressed specifically in 1 or a few tissues, especially the testis (Supplementary Fig. S4). The large amount of tissue-specific lncRNAs uncovered in *P. vitticeps* implies their potential importance in squamate biology that is worth further attention.

### The demarcation of PAR and SDR along the *P. vitticeps* Z sex chromosome

**Figure 5:**
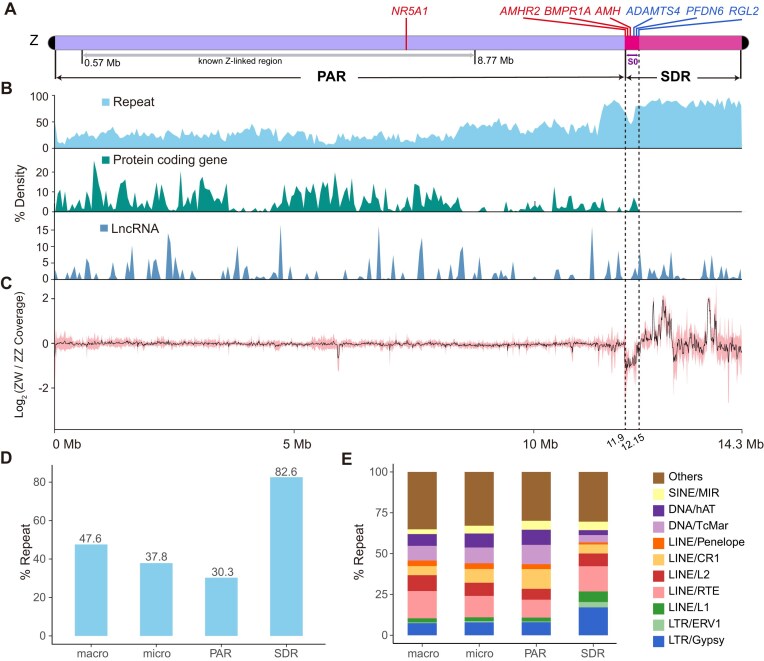
The characteristics of the Z sex chromosome. (A) The schematic of the Z chromosome, which is composed of PAR and SDR. The locations of protein-coding genes potentially involved in sex determination/differentiation are marked in red, while pseudogenes are marked in bule. Known Z-linked regions were identified by mapping known Z-linked scaffolds identified by Deakin *et al*. [14]. (B) Densities of repeats, protein-coding genes and lncRNAs across the Z chromosome. All the densities were calculated in 50 kb windows. (C) The distribution of the ratio of ZW/ZZ read coverage calculated in 10 kb windows across the Z chromosome. The black line denotes the mean values for all ZW-ZZ pairs based on the WGS data from twelve ZW females and eight ZZ males. The pink area denotes the confidence interval for each window. (D) Repeat content for macrochromosomes, microchromosomes, PAR region, and SDR region. (E) Relative proportions for the ten most abundant transposon families and the rest repetitive elements.

The identity of the *P. vitticeps* Z chromosome was ascertained by mapping known Z-linked scaffolds identified by Deakin et al. [14] to our Pvit2024 assembly. Specifically, all the Z-linked scaffolds were aligned to a ∼14.3-Mb microchromosome in expected order, spanning a continuous region of ∼8.2 Mb on the first half of this microchromosome (Fig. 5A; Supplementary Fig. S5A). The uncovered portion comprised ∼6.1-Mb genomic sequences that were newly identified to be Z-linked, accounting for ∼43% of the whole Z chromosome (Fig. 5A). These newly identified Z-linked sequences harbored 47 protein-coding genes and 65 lncRNA genes that were either missing or unknown to be Z-linked in a prior Pvit2015 assembly (Supplementary Table S13). The overall Z chromosome showed an uneven distribution of genomic elements, with an increase of repeat content accompanied by depletion of protein-coding genes toward the chromosomal end that enriched newly anchored sequences (Fig. 5B).

We next attempted to demarcate the SDR, where Z-W sequence divergence is accumulated, and the PAR, where Z and W remain identical, by comparing WGS read coverage between ZW and ZZ individuals. In principle, the PAR is expected to display comparable read coverage in both sexes, while the SDR would exhibit halved coverage in ZW females relative to ZZ males [39]. According to this principle, we analyzed the WGS data from 12 ZW females and 8 ZZ males (Supplementary Table S14). The data of 5 individuals were sourced from published studies, and those of the remaining 15 individuals were newly collected in this study after sex validation with sex-specific markers (Supplementary Fig. S5C). To exclude the potential confounding effects of relatedness, we conducted principal component analysis and kinship analysis for all the 20 individuals and confirmed that all were from different families (Supplementary Fig. S5D, E). Based on this curated dataset, up to 83% (∼11.9 Mb) of the Z chromosome displayed a comparable read coverage in both sexes, suggesting that the majority of Z remains as PAR that persists recombination. Nevertheless, there was a ∼250-kb region exhibiting roughly halved coverage in ZW females relative to ZZ males (Fig. 5C), fulfilling the expectation of an evolutionarily old stratum (hereafter referred as S0) where Z and W substantially diverged. For the region ranging from the downstream of S0 to the chromosome end, the read coverage in ZW females was even higher than that in ZZ males (Fig. 5C). However, this region lacked protein-coding genes and instead was occupied exclusively by repetitive elements (Fig. 5B). We speculated that this unusual coverage pattern was attributed to the much higher abundance of repeats accumulated in the W counterpart of this region, as previous cytogenetic studies have suggested that the short arm of W is much longer than Z [12]. If so, this downstream region of S0 (∼2.18 Mb in length) might also represent a stratum that was fully degenerated due to transposon invasion, and therefore, we tentatively grouped it into SDR in this study.

Together, we could conservatively define a large, continuous PAR and a small SDR on the *P. vitticeps* Z chromosome, with the PAR-SDR boundary delimited at ∼12 Mb in coordinates (Fig. 5A). Compared with PAR, SDR was apparently characterized by a remarkably high repeat content (>80%) (Fig. 5D). However, the repetitive element composition of SDR was generally similar to those of PAR and other chromosomes, although several classes of transposon, such as LTR/Gypsy, LTR/ERV1, and LINE/L1, were relatively more abundant in SDR (Fig. 5E).

### The evolutionary origin and developmental expression of the Z-linked SDR genes

**Figure 6:**
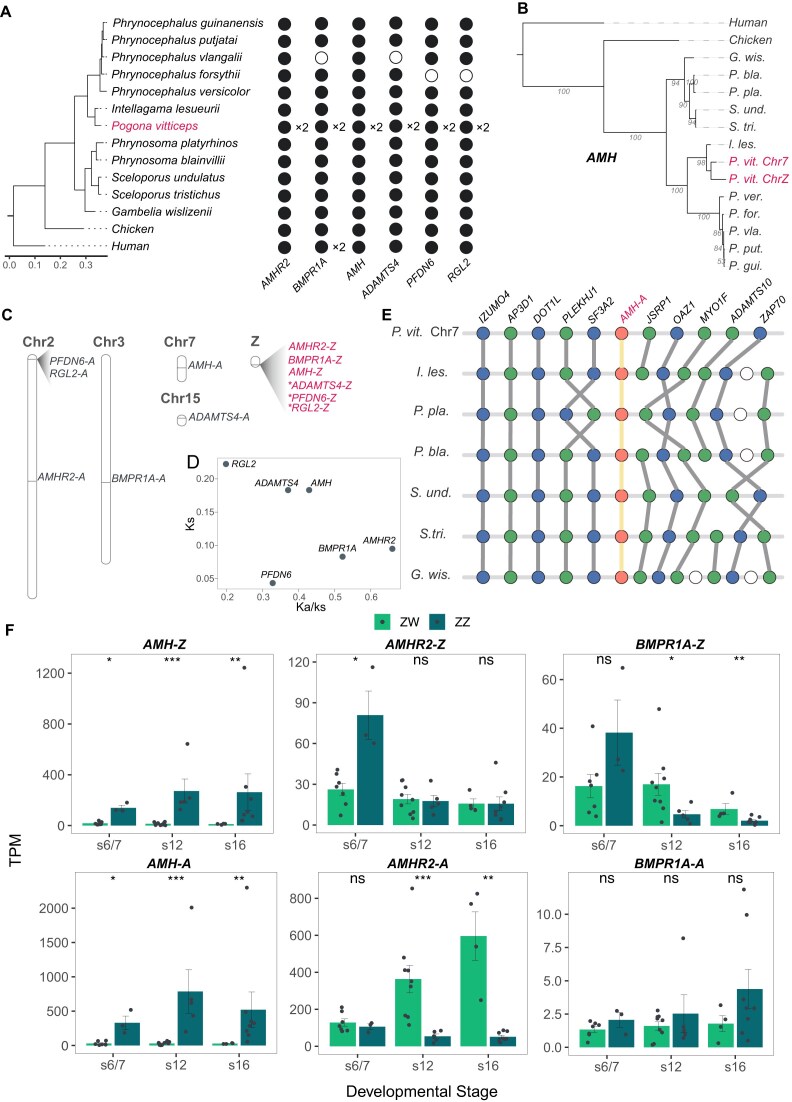
Z-linked SDR genes. (A) Copy number of SDR genes in different species. A black circle indicates the presence of a copy, a number preceded by 'x' denotes copy number, and a while circle indicates absence. (B) Phylogenetic relationship of *AMH* genes across different species. The pink font denotes the 2 copies of *AMH* in *P. vitticeps*. (C) Ideogram showing the gene model of SDR genes and their autosomal counterparts in the Pvit2024 assembly. Pseudogenes are indicated with an asterisk. “-A” indicates autosomal genes, while “-Z” indicates Z-linked genes. (D) Ka/Ks analysis of the SDR genes. For each gene, Ka and Ks were estimated between the Z-linked copy and its autosomal counterpart. (E) Synteny relationships of *AMH-A* and its flanking 5 genes in closely related species. *AMH* is highlighted in orange, and surrounding genes are shown in gray. (F) Differential expression of SDR genes between ZZ and ZW gonads in different developmental stages. Asterisks represent degree of significance with the Benjamini–Hochberg method: ns (not significant), *P < 0.05, **P < 0.01, ***P < 0.001.

Although being predominantly occupied by repetitive elements, we found that the *P. vitticeps* SDR carried 3 intact protein-coding genes (*AMHR2, BMPR1A*, and *AMH*) and 3 pseudogenes (*ADAMTS4, PFDN6*, and *RGL2*), of which all were located in a ∼150-kb region within S0 (Fig. 5A). Notably, the 3 intact genes are all well-known players in driving sexual differentiation—namely, *AMH* (anti-Müllerian hormone) [40] and its 2 receptors, *AMHR2* (anti-Müllerian hormone receptor type 2) [41] and *BMPR1A* (bone morphogenetic protein receptor type 1A) [42]. It is also noteworthy that each of these 6 SDR genes had a counterpart (i.e., paralog) on autosomes, while all other investigated lizards encoded only 1 copy of each in their genomes (Fig. 6A), indicating that lineage-specific gene duplication events occurred in *P. vitticeps* (and probably in other closely related agamid lizards as well). This was also supported by gene phylogenetic analyses, which preferentially clustered the *P. vitticeps* paralogs together among the proteins from multiple species (Fig. 6B; Supplementary Fig. S6A). In contrast, while the PAR maintained up to 334 protein-coding genes, only 21 of them (6.3%) had paralogs in autosomes. Hereafter, we designated the 6 SDR genes as *AMHR2-Z, BMPR1A-Z, AMH-Z, ADAMTS4-Z, PFDN6-Z*, and *RGL2-Z*, and their autosomal counterparts as *AMHR2-A, BMPR1A-A, AMH-A, ADAMTS4-A, PFDN6-A*, and *RGL2-A*, respectively (Fig. 6C).

We next asked, for these 6 paralog pairs in *P. vitticeps*, whether the SDR or the autosomal copy was the original copy that gave rise to the other one. By examining gene synteny information across species, we found that none of the SDR copies could be deemed to be original—namely, they all arose from the duplication of their autosomal counterparts (Fig. 6D; Supplementary Fig. S6B). But it is noted that *PFDN6* and *RGL2* were probably duplicated as a whole, because their gene order and the transcriptional direction in SDR were maintained the same as their original copies in chromosome 2 (Fig. 6C; Supplementary Fig. S6B). We then estimated the synonymous nucleotide substitution rate (Ks) between the paralog pairs to date the order of their translocation into SDR. As expected, the pseudogenes displayed the largest Ks, probably due to relaxed selection. But it is interesting that, among the 3 intact genes in SDR, *AMH* displayed the largest Ks, suggesting that *AMH* is likely among the earliest ones to be integrated into the SDR (Fig. 6D).

The master sex-determining gene that initiates sex differentiation remains unknown in *P. vitticeps*, yet it is expected to be a gene that locates in SDR and reveals differential expression between the ZZ male and the ZW female at an early stage of gonad differentiation. Therefore, we examined the expression dynamics of the SDR genes along gonadal development with the RNA-seq data from Wagner et al. [43]. This dataset comprised gonadal transcriptomes of both sexes collected at 3 embryonic developmental stages (stage 6/7, stage 12, and 16; Supplementary Table S15). In *P. vitticeps*, stage 6/7 represents the earliest stage at which a consolidated gonad is recognizable and begins to differentiate, stage 12 represents an early stage of differentiation, and the gonads at stage 16 have been fully differentiated [7, 43]. Of note, all 3 intact SDR genes (*AMH-Z, AMHR2-Z*, and *BMPR1A-Z*) were identified as differentially expressed genes (DEGs) between sexes at 1 or more developmental stages (DESeq2 false discovery rate [FDR] < 0.05; Fig. 6F; Supplementary Table S16), while none of the pseudogenes and lncRNAs in SDR were significant DEGs (Supplementary Fig. S6C). In addition, we found that *AMH-Z* (FDR = 0.00005; Fold Change (FC) = 5.54) and its potential receptor *AMHR2-Z* (FDR = 0.05; FC = 2.22) manifested a significant male-biased expression pattern at stage 6/7, the earliest developmental stage examined. Moreover, *AMH-Z* maintained and even amplified the differential expression pattern throughout gonadal differentiation, reinforcing its central role in driving male development in vertebrates. Together with its early integration into SDR, *AHM-Z* could be considered a strong candidate of the master sex-determining gene in *P. vitticeps*.

Meanwhile, we also examined the expression of the autosomal paralogs of the SDR genes, to explore whether the expression pattern had diverged after gene duplication. Interestingly, *AMH-A* displayed almost the same expression pattern as *AMH-Z*, maintaining a high expression level in males while repressing its expression in females throughout embryonic gonadal development (Fig. 6F). However, the expression dynamics of *AMHR2-A* and *BMPR1A-A*apparently diverged from that of their SDR counterparts. Specifically, while *AMHR2-Z* displayed transient male-biased expression at stage 6/7, *AMHR2-A* was not differentially expressed at stage 6/7 but became significantly female-biased at stages 12 and 16 due to its upregulation in ZW females. In terms of *BMPR1A*, the expression of *BMPR1A-Z* was a bit male-biased at stage 6/7, but the following sharp decrease of *BMPR1A-Z* expression in ZZ males made it become significantly female-biased at later stages; however, *BMPR1A-A* did not show significant sex-biased expression throughout development, although a slight trend of male-biased expression was observed (Fig. 6F).

## Discussion

### The first chromosome-scale reference genome for Amphibolurinae (Squamata: Agamidae)

Squamate reptiles are a species-rich clade of amniote vertebrates that have adapted to most terrestrial ecosystems [44]. With over 12,000 extant species that make up a significant part of the vertebrate tree of life, reference genomes for squamates are particularly scarce when compared with other amniote clades such as mammals and birds [45]. Specifically, according to the latest data deposited in NCBI (accessed in July 2024), only 104 squamate species have a genome assembly with a contig N50 over 30 kb, in sharp contrast with ∼630 for birds and ∼730 for mammals. This unevenness has drawn the concern of the biodiversity genomics community, which calls for more attention to be paid to this neglected vertebrate group in the genomic era [9, 10]. Thanks to technological advances in long-read sequencing, constructing a near-complete reference genome has become feasible for many organisms, especially for those able to supply sufficient HMW DNA from a single individual, such as the reptiles, as showcased in this study. By leveraging a newly released long-read sequencing technique called CycloneSEQ, we achieved a near-complete genome assembly of a ZZ male *P. vitticeps*, which represents the first chromosome-scale reference genome for the Amphibolurinae subfamily of the agamid lizards (Agamidae).

In the meantime, our assembly pipeline resolved a persistent challenge regarding telomere assembly. Unlike most published squamate genomes, which often lack telomeric repeats (TTAGGG)_n_ at their chromosomal ends, our initial genome assembly captured only 8 of 32 expected telomeres. However, raw CycloneSEQ reads contained abundant TRUs, clearly demonstrating that the absence of telomeres in the initial assembly stemmed from a computational limitation rather than a sequencing omission. Our optimized assembly pipeline successfully recovered almost all (31/32) of the *P. vitticeps* telomeres from raw sequencing data and is expected to facilitate T2T genome assembly of other species as well.

### AMH signaling as an upstream driver of sexual differentiation in *P. vitticeps*

Although several unclosed gaps remained in repeat-rich regions, our Pvit2024 Z chromosomal assembly has approached the T2T level, as indicated by the presence of telomeric sequences on both chromosomal ends. Additionally, the Pvit2024 Z almost doubled the known Z-linked sequences, from 8.3 Mb to 14.3 Mb. More importantly, the newly anchored Z-linked sequences carried the whole SDR, a region that hopefully harbors the master sex-determining gene.

The Z/W-resided *nr5a1*, which encodes the steroidogenic factor 1 (SF1) required for male development, was once regarded as the most promising sex-determining candidate in *P. vitticeps* [46]. However, the location of *nr5a1* in PAR with no W-specific mutations, as uncovered in the population dataset (Supplementary Table S17), has almost ruled it out as the master sex-determining gene. Instead, the aggregation of *AMH* signaling related genes (i.e., *AMH, AHMR2*, and *BMPR1A*) in the SDR is of particular interest. *AMH*, which encodes a hormone of the transforming growth factor–β (TGF-β) superfamily, is critical for testis development and has been identified as a master sex-determining gene in a growing number of vertebrates, including several lineages of teleost fish [47] and monotreme mammals [48]. In *P. vitticeps*, we found that the SDR-resided *AMH* (*AHM-Z*) was highly expressed in all examined ZZ embryos and maintained over a 5-fold expression difference between ZZ and ZW embryos since the beginning of gonadal differentiation (stage 6/7). This prominent male-biased expression pattern suggests *AMH-Z* as a strong sex-determining candidate, which deserves further functional experimental validation in future studies.

AMH signals through binding to its receptors, such as the type II receptor encoded by *AMHR2* and the type I receptor encoded by *BMPR1A* [42]. Therefore, our finding of transient upregulation of the SDR-resided *AMHR2* and *BMPR1A* in ZZ embryos at stage 6/7 is particularly notable. We hypothesize that such a transient upregulation of the *AHM* receptors may serve as an amplifier of the *AHM* signaling cascade at the bipotential gonads, which is essential for tipping the sex differentiation network toward male development at this critical time window. Another notable finding is the upregulation of the autosome-resided *AMH* (*AMH-A*) since stage 6/7. Given the high protein similarity of *AHM-Z* and *AMH-A*, which implies their functional conservation, this parallel upregulation of *AMH-A* may serve as another booster of AMH signaling during early sex differentiation. Collectively, all this evidence points to one conclusion: AMH signaling is essential and likely serves as an upstream signal driving *P. vitticeps* sex differentiation. Such a pattern of duplicated gene functioning in upstream sex determination has previously been identified mainly in fish [49]. Interestingly, all these duplications, including our current finding in the *Pogona* lizard, seem to direct gonad development into testis.

However, it is also notable that the gonadal transcriptome profiles of ZZ and ZW embryos diverged at stage 6/7 [43]. This suggests that the initial elevation of AMH signaling likely occurs even earlier, before stage 6/7. Accordingly, future studies may focus on when and where (e.g., what cell types) AMH signaling initially diverges between ZZ and ZW embryos.

### A proposed model for the origin and evolution of *P. vitticeps* sex chromosomes

The origin of the *P. vitticeps* ZW microchromosomes is presumably associated with the fusion of a fragment derived from chromosome 2, as both the Z and W chromosomes share homology with the terminal region of the long arm of chromosome 2 (chr2qter), as revealed by physical mapping of a BAC clone (Pv151P16) [50]. Our result also supports this homology according to the finding of an additional copy of the chr2qter-resided *PFDN6-RGL2* syntenic block in SDR (Fig. 6C). However, we also found that the homology with chr2qter is limited to the terminal region of the *P. vitticeps* Z chromosome, which lacks intact protein-coding genes, thus arguing for the role of the chr2qter fusion event in driving ZW formation. Indeed, the fusion of chr2qter to *P. vitticeps* ZW microchromosomes seemed to occur early during Amphibolurinae evolution, probably before the differentiation of Z and W, because the concurrent mapping of Pv151P16 to chr2qter and a pair of microchromosomes also have been observed in other Amphibolurinae species, including GSD species with homomorphic sex chromosomes and TSD species without sex chromosomes [50].

However, it is notable that the Ks of *AMH*-Z is higher than that of *PFDN6-Z* and almost comparable to that of *RGL2-Z*, despite *PFDN6-Z* and *RGL2-Z* becoming pseudogenes (Fig. 6C). This suggests that the integration of *AMH* and the fusion of chr2qter to the proto-sex chromosomes might occur within a very narrow time window. We thus propose that the duplication and translocation of the autosomal *AMH* to the proto-sex chromosomes might represent the real milestone in triggering the formation of Z and W chromosomes in the ancestor of *P. vitticeps* and its sister taxa. After *AMH* integration, the subsequent aggregation of other male-biased genes, including *AMHR2* and *BMPR1A*, further consolidated the role of the proto-Z in sex determination and promoted ZW differentiation. We anticipate that chromosome-scale genome assemblies from other closely related dragon lizards will provide crucial evidence for testing this hypothesis.

## Methods

The DNBSEQ library construction and sequencing protocols used in this study are gathered in a protocols.io collection [51].

### Sample collection

This study was performed in accordance with the guidelines of the national and organizational stipulations. All analyses were performed in accordance with the scope of the ZJU20240302 research protocol. An adult lizard *P. vitticeps* in captivity was collected at Zhejiang University, under permit ZJU20240342. This lizard was euthanized after being anesthetized by diethyl ether. All the tissue samples were frozen via liquid nitrogen and stored at −80°C immediately after dissection. Muscle and lung tissues were selected for genome sequencing, which included CycloneSEQ long-read WGS sequencing, DNBSEQ short-read WGS sequencing, CycloneSEQ-based Pore-C sequencing, and Hi-C sequencing. Furthermore, 8 tissue samples, including liver, lung, eye, muscle, testis, kidney, brain, and heart, were subjected to transcriptome sequencing, which included bulk RNA-seq and CycloneSEQ long-read RNA-seq.

### Genomic DNA CTAB extraction

HMW genomic DNA was extracted from muscle and lung tissues using the CTAB method as follows: preheat the CTAB lysate in a water bath. Collect 20 to 30 mg of fresh or frozen animal tissue, and freeze in liquid nitrogen. Grind the tissue using a mortar and pestle in the presence of liquid nitrogen until the tissue is finely ground. Transfer the ground animal tissue to 2-mL polypropylene centrifuge tubes. Add 1 mL CTAB lysis buffer and 50 µL protease K (20 mg/mL). Swirl and mix, then incubate at 50°C (with shaking) for 1 hour. Cool the tube to 37°C. Add 20 µL RNase A (10 mg/mL) per 1 mL of lysis buffer, mix by inversion, and incubate for 10 minutes. Add an equal volume of phenol-chloroform-isoamyl alcohol (25:24:1 ratio) with pH >7.8, mix by inversion, and spin at 5,000 rpm for 10 minutes in a tabletop centrifuge at room temperature (RT). Transfer the top aqueous solution to new 2-mL centrifuge tubes using a wide-bore pipette tip. Add an equal volume of chloroform-isoamyl alcohol (24:1 ratio), mix by inversion, and spin at 5,000 rpm for 10 minutes in a tabletop centrifuge at RT. Transfer the top aqueous solution to new 1.5-mL centrifuge tubes using a wide-bore pipette tip. Add two-thirds volume of isoamyl alcohol and mix by inversion to form an emulsion, and centrifuge at 5,000 rpm for 2 minutes at 4°C. Discard the supernatant. Add 1 mL 75% ethanol and mix slowly to resuspend the precipitation. Centrifuge at 5,000 rpm for 2 minutes at 4°C, and discard the supernatant. Repeat the previous step. Discard the remaining supernatant, and dry at RT for 3 minutes. Dissolve in 200 to 400 µL TE Buffer, incubate at 37°C for 1 h, and incubate at RT overnight. Store the extracted DNA at −80°C.

### CycloneSEQ library construction and sequencing

In this study, 3 CycloneSEQ-associated library preparation and sequencing approaches were employed: CycloneSEQ long-read WGS library preparation and sequencing [52], CycloneSEQ-based Pore-C library preparation and sequencing [53], and CycloneSEQ long-read RNA-seq library preparation and sequencing [54]. Detailed protocols for all methods have been deposited to protocol.io.

### Other library preparation and sequencing

For the Hi-C library construction, the muscle sample was crosslinked with formaldehyde, and 2 Hi-C libraries were constructed by using the dpnII restriction endonuclease. The libraries were sequenced on the DNBSEQ platform with 150-bp paired-end sequencing strategy.

For bulk RNA-seq library construction, the isolated RNA was then fragmented into 200 to 400 bp and then reverse-transcribed to cDNA for library preparation. Quantity and quality of the genome’s DNA and RNA were assessed by pulsed field gel electrophoresis, Qubit 3.0 (Invitrogen) and Qseq 400 (Bioptic). A total of 12 short-insert paired-end (PE) libraries (4 for genomic DNA and 8 for cDNA) were constructed and sequenced on the DNBSEQ platform (MGI), using the manufacturer’s instructions.

### Sex identification

Organ anatomical examination and sex-linked marker analysis were employed for sex identification in *P. vitticeps*. For the anatomical assessment, testes were dissected from the individual. Molecular analysis of sex-linked markers was conducted using a protocol adapted from Holleley et al. [6]. Genotypic sexing was performed utilizing 2 PCR primers: H2, GCCCATATCTCACTAGTTCCCCTCC; F, CAGTTCCTTCTACCTGGGAGTGC, which flanked 2 W chromosome–specific deletions, measuring 150 bp and 14 bp, respectively. PCR was conducted using Platinum High-Fidelity ReadyMix(2x) (GCATbio), with a range of primer concentrations and genomic DNA quantities to establish experimental groups, and a no-genomic DNA control group. Cycling conditions were 95°C for 5 minutes; (95°C for 20 seconds, 70∼65°C for 20 seconds, 72°C for 1 minute) × 10 cycles with annealing temperature decreased 0.5°C per cycle; (95°C for 20 seconds, 65°C for 20 seconds, 72°C for 1 minute) × 30 cycles; 72°C for 10 minutes. The PCR products were resolved on a 1.5% agarose gel and visualized using GelStain Blue (Transgen). The presence of 2 bands indicated ZW individuals, while a single band confirmed ZZ individuals.

### Genome survey

The DNBSEQ short-read WGS data were cleaned by SOAPnuke v1.5.6 (RRID: SCR_015025) [55] to exclude reads characterized by low quality and the presence of adapter sequences and poly-N regions with parameters -Q 2 -G -d -l 20 -q 0.2 -5 1 -t 5,0,5,0. To accurately assess genomic characteristics, including genome size and heterozygosity rate, all clean data were analyzed using *k*-mer–based methods by using Jellyfish and GenomeScope tools. The haploid genome size was estimated according to *k*-mer analysis frequency distributions generated by Jellyfish v2.2.6 (RRID: SCR_005491) [56] using a series of *k* values (19, 21, 23, 25, 27) with the -C setting, which was calculated as the number of effective *k*-mers (i.e., total *k*-mer − erroneous *k*-mer) divided by the homozygous peak depth. The rate of heterozygosity was estimated by GenomeScope v2.0.0 (RRID: SCR_017014) [57] with the *k*-mer frequency distributions generated by Jellyfish as inputs.

### Genome assembly

The initial process was to retain CycloneSEQ long reads longer than 40 kb, and the chimeric reads were removed using Yacrd v1.0.0 [58] with parameters -c 5 -n 0.6. Clean reads were then assembled into contigs using NextDenovo v2.5.0 (RRID:SCR_025033) [17] with the following parameters: read_cutoff = 35k, genome_size = 1.7 g. In response to the high heterozygosity detected in the initial assembly, DNBSEQ short reads were adopted to remove heterozygous contigs by purge_haplotigs v1.1.2 (RRID:SCR_017616) [18] with parameters -l 10 -m 53 -h 120. Next, 2 rounds of polishing were performed using NextPolish v1.4.1 (RRID:SCR_025232) [19] with recommended parameters, utilizing DNBSEQ short reads. Subsequently, CycloneSEQ-based Pore-C reads were used as the main body to anchor the assembly to pseudo-chromosomes, while 2 additional libraries of Hi-C reads served as controls to assess the reliability of the anchoring. The specific process of anchoring was as follows: CycloneSEQ-based Pore-C reads were first aligned to the contig-level genome using wf-pore-c v1.1.0 [59] with default parameters. The unaligned fragments were subsequently filtered out from the wf-pore-c output file “null.ns.bam” using the command “samtools view -F 4 -bh.” Adjacent fragment pairs were then extracted from the filtered BAM file into BED format with a custom script [60]. In parallel, 2 additional libraries of Hi-C reads were aligned using Chromap v0.2.3-r407 [61] with default parameters. The alignment results were converted from the .sam format to a .bed file using bedtools v2.29.2 [62]. Both sets of BED files, derived from CycloneSEQ-based Pore-C and Hi-C reads, respectively, were utilized to assemble contigs into scaffolds using YaHS v1.2a.1 [20] with the parameters –no-contig-ec –no-scaffold-ec. Following the scaffolding process, we proceeded to generate a .hic file for further manual examination and curation using JuiceBox (JBAT) [21], adhering to the guidelines [63].

To avoid telomeric repeat sequences being incorrectly trimmed by the assembler, by using known vertebrate 6-base telomere repeats (“TTAGGG”) as a sequence query, we developed an in-house pipeline to correct telomeric regions as follows: First, we extracted those reads from CycloneSEQ WGS raw reads containing at least 100 consecutive copies of the telomere units. Then, all of these reads were aligned to the assembly using minimap2 v2.23-r1116-dirty (RRID:SCR_018550) [32] with the parameter -x map-ont. Subsequently, based on the alignment results, for each chromosome’s 2 ends, the read with the highest alignment quality and the largest number of copies of the telomere unit was selected and used to replace the corresponding end [60].

After telomere repair, unclosed gaps in the chromosome-scale assembly were initially filled using TGS-GapCloser v1.2.1 (RRID:SCR_017633) [22] with the corrected long reads (generated by NextDenovo during assembly). The correctness of each closed gap was further examined by long-read coverage with an in-house pipeline as follows: the 5 kb of upstream and downstream regions was extracted and aligned to the corrected long read to identify any alignment. These alignments were further visualized and manually examined using IGV. Only alignments with proper size and consistent orientation without conflicting alignments were used for the purpose of gap filling within the genomic assembly.

Finally, the assembled genome was evaluated using BUSCO v5.7.1 (RRID:SCR_015008) [23], Compleasm v0.2.6 [24], and Merqury v1.3 (RRID:SCR_015811) [64] using default parameters. Short reads were aligned to the genome using BWA-MEM v0.7.17-r1198-dirty (RRID:SCR_012940) [65], and SAMtools v1.15.1 (RRID:SCR_002164) [66] was used to count them as properly paired. Long reads were mapped to the genome using minimap2 v2.23-r1116-dirty (RRID:SCR_015008) [32] with the parameter -ax map-ont.

### Reconstruction of NOR 45S rDNA cluster

According to the comprehensive cytogenetic map, *P. vitticeps* has an active nucleolus organizer region (NOR) rich in the 45S rDNA cluster, comprising 18S, 5.8S, and 28S rRNAs, at the subtelomeric region of chromosome 2 [12]. However, the sequencing depth of the NOR region, identified as the 45S rDNA-rich region near the telomere on Chr2 from the *de novo* assembly of the whole genome, was significantly higher than that of adjacent regions. Additionally, the length of the NOR region was much shorter than those found in closely related species. These findings indicated that the NOR region was not fully assembled. To address this, we reconstructed the NOR 45S rDNA cluster. First, the 45S rDNA sequence from the NOR region was used to identify all CycloneSEQ reads containing the 45S rDNA sequence feature. Second, these reads were assembled using NextDenovo v2.5.0 [17] with the following parameters: read_cutoff = 1k, genome_size = 0.005 g, minimap2_options_raw = -I 6 G –step 2 –dual=yes -t 4 -x ava-ont -k 17 -w 17 –minlen 2000 –maxhan1 5000. Finally, we obtained a 282.2-kb NOR region rich in the 45S rDNA cluster and replaced this segment with the genomic NOR position where the assembly had collapsed.

### Repetitive element annotation

We annotated the lizard whole-genome repeat sequences based on *de novo* predictions and homology annotations. For *de novo*, RepeatModeler v2.0.4 (RRID:SCR_015027) [67] was used to identify the custom repeats from the assembly sequence, with subsequent annotation and masking using RepeatMasker v4.1.5 (RRID:SCR_015027) [68]. For homology annotations, we first identified known transposable elements in the Pvit2024 genome using RepeatMasker by searching against the RepeatModeler (v2.0.4) transposable element library. Then, the transposable element proteins were searched based on RepeatProteinMask database [69], which is part of RepeatMasker. Tandem repeats were also extracted using TRF v4.09 [70] via *ab initio* prediction, with the following parameters: “Match = 2, Mismatch = 7, Delta = 7, PM = 80, PI = 10, Minscore = 50, and MaxPeriod = 2000.”

### Protein-coding gene annotation

We integrated 3 types of evidence for predicting protein-coding genes: transcriptome data, homology evidence, and *ab initio* prediction. For the transcriptome evidence, we first cleaned the DNBSEQ RNA-seq data using SOAPnuke v1.5.6 (RRID:SCR_015978) [55] with the following command: filter -n 0.03 -l 20 -q 0.3 -p 1 -Q 2 -G -5 1 -t 10,0,10,0 -E 70. Next, we aligned the cleaned short reads to the genome using HISAT2 v2.2.1 (RRID:SCR_015530) [71] with the –dta option. Finally, the transcripts were reconstructed using StringTie2 v2.2.3 (RRID:SCR_016323) [72], and coding sequences were predicted with TransDecoder v5.7.1 (RRID:SCR:015534) [73]. For *ab initio* prediction, we randomly selected 700 high-quality genes from the transcriptome predictions for training and used AUGUSTUS v3.4.0 (RRID:SCR_008417) [74] for the *ab initio* annotation of coding genes. For homology-based prediction, homologous data from 5 closely related species, including *Ahaetulla prasina* (GCF_028640845.1), *Furcifer pardalis* (GCA_030440675.1), *Hemicordylus capensis* (GCF_027244095.1), *Rhineura floridana* (GCF_030035675.1), and *Zootoca vivipara* (GCF_963506605.1), were collected with NCBI RefSeq. To summarize, we integrated the above 3 types of evidence and short read splice information data as input to the GeMoMa v1.9 (RRID:SCR_017646) [75] workflow for a comprehensive prediction of coding genes. For the annotation results obtained from the GeMoMa, we further filtered based on the repeat sequence annotation file, removing predictions suspected to be repeat elements, ultimately obtaining the final protein-coding gene annotation set.

### Isoform detection using CycloneSEQ long-read RNA sequencing

The process of CycloneSEQ long-read RNA-seq data was as follows: we filtered the TSO (template switch oligo sequence)/RTP (reverse transcription primer) and split chimeric reads based on artificial sequence. The clean data were mapped to the assembly using winnowmap2 v2.03 [31] with the following command: winnowmap -u b -K 4 G -G 135k -ax splice ref fastq. We then reconstructed transcripts based on the reference annotation using Isoquant v3.5.0 [76] with the following command: *python3 isoquant.py –gene_quantification all –reference genome.fa –data_type nanopore –genedb genes.gtf –threads 16 –count_exons –bam input.bam –label sample*. Subsequently, we merged the transcripts from 8 tissues and eliminated redundancy based on an all-exon overlap threshold of greater than 0.95 between isoforms. We then performed TransDecoder v5.7.1 to predict the coding potential of all transcripts using the *–single_best_only* option. Finally, isoforms of coding genes were assigned to the corresponding loci.

### LncRNA annotation

LncRNA identification was also based on long sequencing reads of CycloneSEQ RNA-seq. The remaining transcripts assembled by Isoquant from the isoform detection steps, which were filtered by length (≥200 nt), were used as the foundational dataset for lncRNA detection. We then utilized 2 tools of FEELnc v0.2.1 [77] and CPC2 v1.0.1 (Coding Potential Calculator, RRID:SCR_002764) [78] to filter potential coding genes. The candidates of lncRNA were obtained by taking the intersection of the results from both tools. We further mapped the corresponding DNBSEQ sequencing data from the same library with CycloneSEQ RNA-seq using HISAT2 v2.2.1 for alignment to the assembly. Expression detection of candidate lncRNAs was performed using FeatureCounts v2.0.1 (RRID:SCR_012919) [79]. We ultimately considered lncRNAs with TPM >1 to be true and reliable.

### *De novo* prediction for tRNA and rRNA

Two types of noncoding RNAs were predicted: transfer RNAs (tRNAs) and ribosomal RNAs (rRNAs). To identify putative tRNA genes in the *P. vitticeps* genome, we employed tRNAscan-SE v2.0.10 (RRID:SCR_008637) [80] with parameters optimized for eukaryotic genomes. Subsequently, we filtered out “pseudo” and “undet” from the output-generated tRNAscan-SE and extracted high-confidence tRNA genes from the BED file, producing a refined tRNA set. For rRNA prediction, we employed the barrnap v0.9 (RRID:SCR_015995) [81] program to identify rRNA genes in the genome, using the following command: barrnap –quiet –kingdom euk genome.fa –threads 20.

### Chromosome contact analyses

In the above scaffolding procedure, the .hic files were derived from the processing of CycloneSEQ-based Pore-C reads and Hi-C reads. Subsequently, the interaction contacts within the .hic files were binned to construct the genome-wide interaction matrix at resolutions of 5 kb, 10 kb, 100 kb, 500 kb, 100 kb, 500 kb, and 1Mb. Following this, iterative correction and eigenvector decomposition (ICE) normalization was then employed to normalize the interaction matrix. Then, *cis*-contacts and *trans*-contacts for each 100-kb window were calculated using the 100-kb normalized interaction matrix.

### Missing sequence identification

We compared the Pvit2024 genome assembly with the previous (Pvit2015) assembly generated by Illumina sequencing. The Pvit2015 assembly was downloaded from NCBI RefSeq by searching for GCF_900067755.1. We excluded the mitochondrial genome from the assembly to prevent misalignment between mitochondrial and nuclear genomes. We then aligned the Pvit2015 assembly to Pvit2024 ref by minimap2 v2.23-r1116-dirty (RRID:SCR_018550) [32] with the following command: *minimap2 -ax asm20 -k14 -K 8 G –secondary=no -s 80 -t 16 Pivt2015 Pvit2024*, and then we used paftools to obtain the aligned regions by minimap2. Additionally, we also performed alignments using the Winnowmap2 tool, which requires the creation of a database with Meryl v1.4.1 [64]. The database was created using the following commands: *meryl count k=19 output merylDB Pvit2024* and *meryl print greater-than distinct=0.9998 merylDB > repetitive_k19.txt*. The alignment was then executed with the following command: *winnowmap -t 16 -W repetitive_k19.txt -K 8 G –secondary=no -ax asm20 -s 80 Pvit2024 Pvit2015*. Similarly, we used paftools to obtain the alignment files for the 2 genomes. We further utilized a Python script [60] to extract unaligned regions from the alignment files. The final set of missing regions was determined by taking the intersection of the results obtained from both methods.

### Missing sequences in genomic elements

We identified the proportions of coding genes, lncRNAs, and promoters within the missing regions based on coordinate overlap information. To identify missing coding genes and lncRNA, we considered a locus span and exon region with an overlap greater than 0.8 with the missing region as a missing gene in the Pvit2015 assembly. In contrast, an overlap between 0.1 and 0.8 was classified as an incomplete gene. Additionally, for the identification of promoters, we defined the 2,000-bp upstream region of coding genes and lncRNAs as potential regulatory regions. An overlap greater than 0.7 between these regions and the missing region was considered a missing promoter, while an overlap between 0.1 and 0.7 was classified an incomplete promoter.

### Calculation of GC content and repeat content

GC content was determined by calculating the total number of Gs and Cs divided by the length of the given coordinates, excluding ambiguous nucleotides (N), using a custom Python script. Repeat content was assessed based on preannotated repeat files, with a Python script used to extract the coordinates of repeat regions and count the number of repeat nucleotides.

### Identification of S0 region

To identify regions with differential sequencing coverage between the Z and W chromosomes of *P. vitticeps*, the WGS datasets from 12 ZW females and 8 ZZ males were aligned to the assembled genome using BWA-MEM v0.7.17-r1198-dirty (RRID:SCR_012940) [65] (Supplementary Table S14). The depth of coverage was extracted using SAMtools v1.15.1 (RRID:SCR_002164) [66]. Median depth was calculated using a nonoverlapping sliding window of 10 kb.

### Searches for genes in SDR regions of closely related species

The genome assemblies of all closely related species, including *Gambelia wislizenii* (GCA_030847615.1), *Intellagama lesueurii* (GCA_037013535.1) [82], *Phrynocephalus forsythia* (GCA_029282475.1) [83], *Phrynocephalus guinanensis* (GCA_037367245.1) [84], *Phrynocephalus putjatai* (GCA_037367255.1) [84], *Phrynocephalus versicolor* (GCA_023846285.1) [84], *Phrynocephalus vlangalii* (GCA_037367305.1) [84], *Phrynosoma blainvillii* (GCA_026167975.1) [85], *Phrynosoma platyrhinos* (GCA_020142125.1) [86], *Sceloporus tristichus* (GCA_016801415.1) [87], *Sceloporus undulatus* (GCA_019175285.1) [88], and the outgroup species *Gallus gallus* (GCF_016699485.2) and *Homo sapiens* (GCF_009914755.1) [89], were downloaded from NCBI RefSeq. We used the protein sequences of *AMHR2, BMPR1A, AMH, ADAMTS4, PFDN6*, and *RGL2* from human, chicken, and green anole, which were downloaded from Ensembl, as query sequences. We further conducted tblastn of blast v2.11.0 [90] searches using these query sequences against the whole genomes of closely related species, with search parameters set to *-evalue 1e-5 -num_threads 16*. For the potential loci obtained from the tblastn searches, we extended 2,000 bp upstream and downstream of each locus. Subsequently, we performed detailed predictions using GeneWise v3 (RRID:SCR_015054) [91], which allowed us to accurately identify the coding regions of coding genes. Finally, we compared the predicted proteins against the nonredundant protein databases of human, chicken, and green anole to confirm the reliability of our predictions.

### Gene synteny analysis

Based on the annotation methods previously applied to *P. vitticeps*, we rapidly performed batch predictions of coding genes on the genome assemblies of 9 closely related species using GeMoMa. The annotation GFF3 files and coding sequences were collected. Syntenic gene pairs between *P. vitticeps* and related species were identified using the Python version of MCScan, JCVI v1.1.16, with the following command: *python3 -m jcvi.compara.catalog ortholog –score=0.95 –no_strip_names*. Syntenic blocks were filtered using the following command: *python3 -m jcvi.compara.synteny mcscan –iter=1*. We combined all species’ syntenic gene pairs based on *P. vitticeps* loci using the following command: *python3 -m jcvi.formats.base join –boheader*. For the synteny plot of each target gene, we selected the target gene and its flanking 10 genes from the *P. vitticeps* locus and generated the plot using the following command: *python3 -m jcvi.graphics.synteny*.

### Phylogenetic analysis and gene evolution

The protein sequences of each orthologous group of sex-determining genes were aligned using MAFFT v7.471 (RRID:SCR_011811) [92] with the following parameters: mafft –anysymbol –maxiterate 1000 –localpair pep.fa. The multiple alignment sequences were then processed to remove spurious sequences or poorly aligned regions using trimAl v1.4.rev15 (RRID:SCR_017334) [93] with the following command: trimal -in pep.fa.aln -out pep.fa.aln.trimal -gt 1. Maximum likelihood inference of phylogenetic relationships was performed using IQ-TREE v2.2.0.3 (RRID_SCR_017254) [94] with the following parameters: iq-tree -m TEST -bb 1000 -bnni. The ratios of nonsynonymous to synonymous substitutions within 6 sex-determining gene pairs were calculated using KaKs_Calculator v2.0 (RRID:SCR_022068) [95] with its default settings.

### Transcriptome source across different sexes and developmental stages

For the male and female gonadal transcriptome data, we used previously published datasets, which included *P. vitticeps* ZZ and ZW individuals at 4 developmental stages (6/7, 12, 16, and adult) [8, 43]. The early-stage gonadal RNA-seq source accession number is SRP304423. The adult source accession numbers used for testes of ZZ male are ERR753529 and ERR413070, and for ovaries of ZW female, they are ERR753530 and ERR413082.

### RNA-seq and differential expression analysis

Paired-end raw reads underwent adapter trimming using SOAPnuke v1.5.6 (RRID: SCR_015025) [55] with *filter -n 0.03 -l 20 -q 0.3 -p 1 -Q 2 -G -5 1 -t 10,0,10,0 -E 70*. Quality control of the sequencing reads was conducted using FastQC v0.12.1 (RRID:SCR_014583). The clean data were mapped to the genome using STAR v2.7.11a (RRID:SCR_004463) [96]. Counts of reads mapping to genes were obtained using FeatureCounts v2.0.1 (RRID:SCR_012919) [79] against the top-length isoform annotation with the following command: -T 16 -g gene_id -t exon. To investigate gene expression changes from stage 6/7 to adult, differential gene expression analysis was conducted between these stages for each sex, ZZ and ZW. Differential expression was performed in R v4.0.2 using the DESeq2 package v1.28.1 (RRID:SCR_015687) [97]. Additionally, we processed the raw expression matrix using TPM counts to ensure comparability between samples and ultimately used these values to generate histograms of gene expression.

## Availability of Source Code and Requirements

Project name: Pvit_T2T

Project homepage: https://github.com/guoqunfei/Pvit_T2T

Operating system(s): Platform independent

Programming language: Python

Other requirements: Python ≥ 3.7

License: MIT

A version of the record snapshot of the GitHub repository has been archived in the Software Heritage Library with the PID swh:1:snp:6b86dd54dbc73e2f086220e8ee0feee27b3403d9 [98].

**Editors’ Note** Another near-T2T bearded dragon genome assembly is published alongside this work, sequenced using different sequencing technology and approaches [99]

## Additional Files

**Supplementary Fig. S1**. Description of sequencing data, genome assembly, and sex identification.

**Supplementary Fig. S2**. Quality validation of the genome assembly.

**Supplementary Fig. S3**. Analysis of missing sequences and related genomic features.

**Supplementary Fig. S4**. Expression matrix of 15,758 lncRNAs across 8 different tissues.

**Supplementary Fig. S5**. Analysis of sex chromosome.

**Supplementary Fig. S6**. Analysis of SDR genes in *P. vitticeps* and related species.

**Supplementary Table S1**. The summary statistics of sequencing data generated for genome assembly and annotation of *Pogona vitticeps*.

**Supplementary Table S2**. The detailed statistics of CycloneSEQ long-read RNA-seq data.

**Supplementary Table S3**. Detailed statistics for DNBSEQ short-read RNA-seq data from 8 tissues.

**Supplementary Table S4**. Estimation of genome size and heterozygosity of *Pogona vitticeps* by *k*-mer analysis.

**Supplementary Table S5**. Improvement in continuity and completeness of genome assembly generated by each of the 7 assembly steps as stated in the main text.

**Supplementary Table S6**. Summary of comprehensive assessment of genome completeness for 67 long-read sequenced Squamata species using BUSCO and Compleasm.

**Supplementary Table S7**. Quality control of long-range sequencing data from 2 platforms.

**Supplementary Table S8**. Comparison of telomeric region characteristics between *Pogona vitticeps* assembly and 28 squamate species whose genome reached chromosome level.

**Supplementary Table S9**. Summary of telomere and centromere location information of *Pogona vitticeps* genome.

**Supplementary Table S10**. Annotation of repeat sequences.

**Supplementary Table S11**. Genome features between Pvit2024 and Pvit2015.

**Supplementary Table S12**. Gene model and function annotation.

**Supplementary Table S13**. Forty-seven protein-coding genes and 65 lncRNA genes that are missing or unknown to be Z-linked in the prior Pvit2015 assembly.

**Supplementary Table S14**. Twenty samples (8 ZZ males and 12 ZW females) of WGS data used for demarcating the SDR and PAR regions.

**Supplementary Table S15**. Overview of all sequenced embryonic gonad samples included in this study.

**Supplementary Table S16**. Differential expression profiles of Z-link genes across various developmental stages.

**Supplementary Table S17**. The distribution of the depths of ATGC at each site of the *nr5a1* gene locus (26,763 bp in length) across 20 samples.

giaf079_Supplementary_Files

giaf079_Authors_Response_To_Reviewer_Comments_Original_Submission

giaf079_Authors_Response_To_Reviewer_Comments_Revision_1

giaf079_GIGA-D-24-00422_Original_Submission

giaf079_GIGA-D-24-00422_Revision_1

giaf079_GIGA-D-24-00422_Revision_2

giaf079_GIGA-D-24-00422_Revision_3

giaf079_Reviewer_1_Report_Original_Submission Heiner Kuhl -- 11/15/2024

giaf079_Reviewer_1_Report_Revision_1 Heiner Kuhl -- 5/2/2025

giaf079_Reviewer_2_Report_Original_Submission Nazila Koochekian -- 12/8/2024

giaf079_Reviewer_2_Report_Revision_1 Nazila Koochekian -- 5/2/2025

## Abbreviations

A3: alternative 3’ splice site; A5: alternative 5’ splice site; AF: alternative first exon; AL: alternative last exon; BUSCO: Benchmarking Universal Single-Copy Orthologs; chr2qter: the terminal region of the long arm of chromosome 2; EBP: Earth BioGenome Project; GSD: genetic sex determination; HMW: high molecular weight; LncRNA: long noncoding RNA; MX: mutually exclusive exons; NCBI: National Center for Biotechnology Information; NOR: nucleolus organizer region; PAR: pseudo-autosomal region; QV: consensus quality value; RI: retained intron; RNA-seq: RNA sequencing; rRNA: ribosomal RNA; RTP: reverse transcription primer; SDR: sexually differentiated region; SE: skipped exon; T2T: telomere-to-telomere; TPM: transcripts per million; tRNA: transfer RNA; TRU: telomeric repeat unit; TSD: temperature-dependent sex determination; TSO: template switch oligo sequence; UTR: untranslated region; VGP: Vertebrate Genomes Project; WGS: whole-genome sequencing.

## Data Availability

CycloneSEQ long-read WGS data, DNBSEQ short-read WGS data, CycloneSEQ long-read RNA-seq data, DNBSEQ short-read RNA-seq data, CycloneSEQ based Pore-C data, and Hi-C data in this study are deposited in NCBI Sequence Read Archive (SRA) under BioProject accession no. PRJNA1184386 and in the CNGB Nucleotide Sequence Archive (CNSA) of the China National Gene Bank DataBase (CNGBdb) under accession no. CNP0005509. Genome assembly, annotation, other supporting data, and material are available in the *GigaScience* database, GigaDB [100].
