# Supplementary material for: A near-complete genome assembly of the bearded dragon Pogona vitticeps provides insights into the origin of Pogona sex chromosomes
Source: Gigascience. 2025 Aug 19;14:giaf079. doi: 10.1093/gigascience/giaf079 (PMC12360845; doi:10.1093/gigascience/giaf079)
Supplement: giaf079_Supplementary_Files [file giaf079_supplementary_files.zip › Figure S1.pdf]

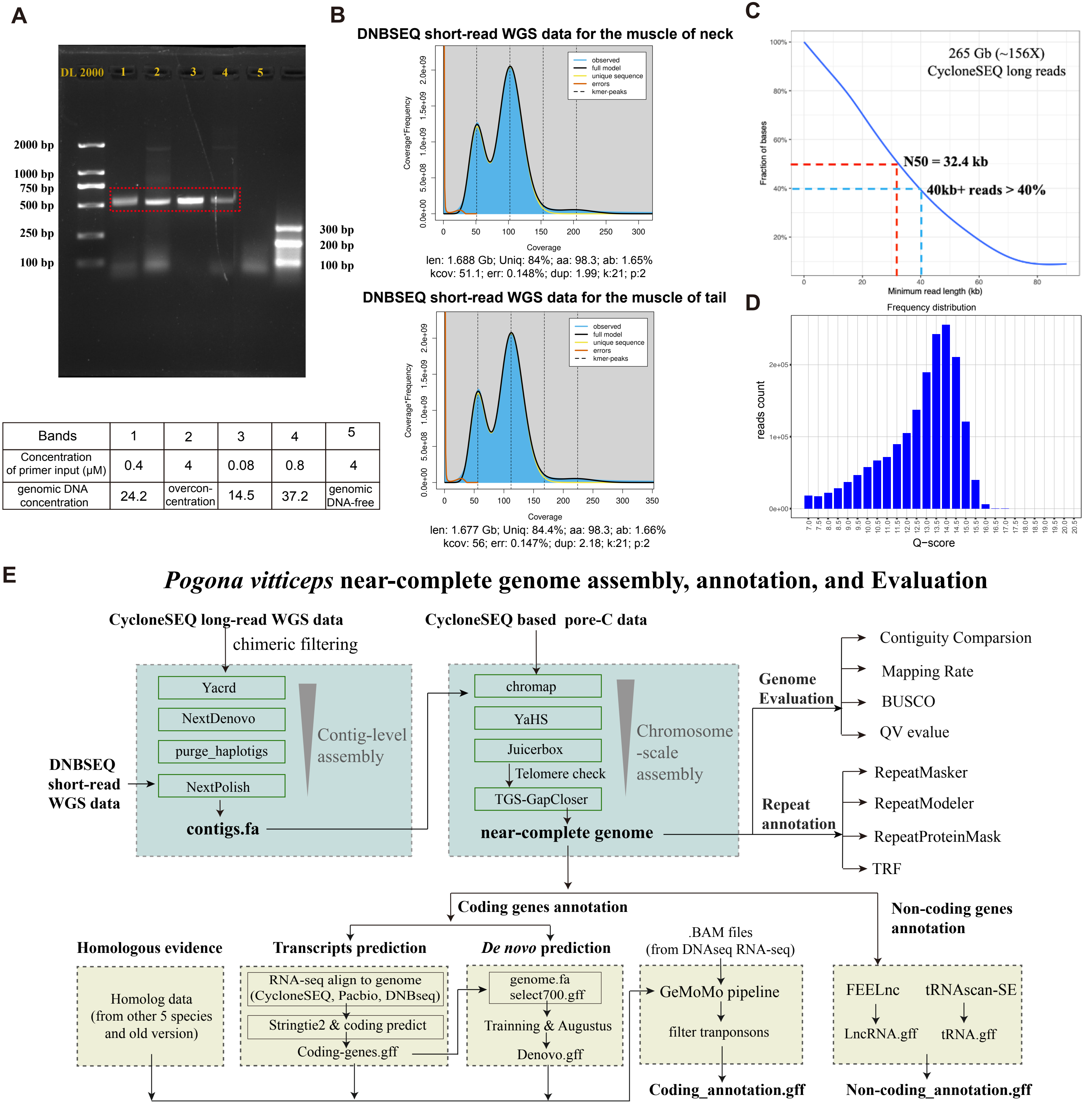

Bands	1	2	3	4	5
Concentration of primer input (μM)	0.4	4	0.08	0.8	4
genomic DNA concentration	24.2	overcon-centration	14.5	37.2	genomic DNA-free
