# Supplementary material for: A near-complete genome assembly of the bearded dragon Pogona vitticeps provides insights into the origin of Pogona sex chromosomes
Source: Gigascience. 2025 Aug 19;14:giaf079. doi: 10.1093/gigascience/giaf079 (PMC12360845; doi:10.1093/gigascience/giaf079)
Supplement: giaf079_Supplementary_Files [file giaf079_supplementary_files.zip › Figure S6.pdf]

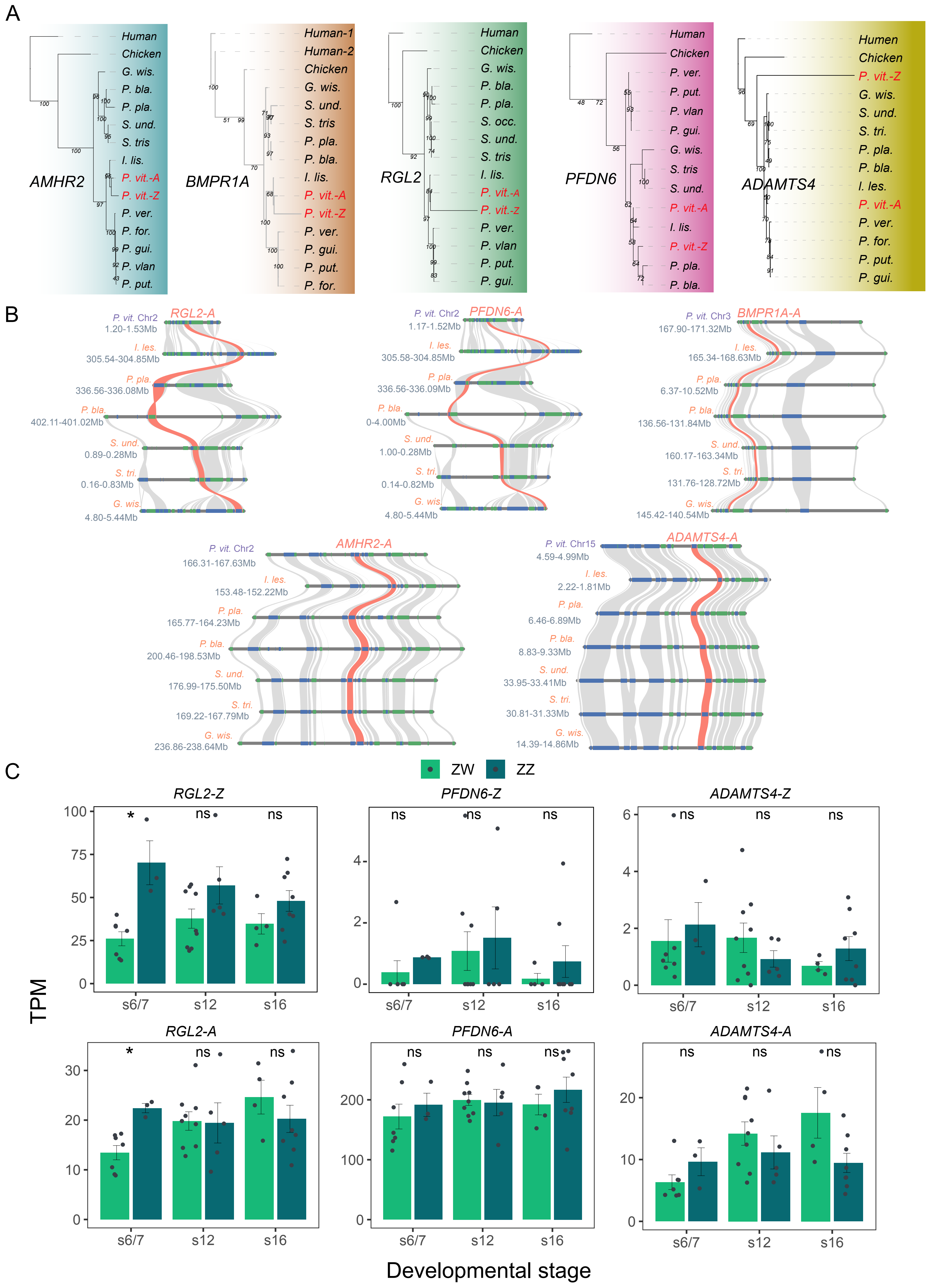

A
AMHR2
Human
Chicken
G. wis.
P. bla.
P. pla.
S. und.
S. tris
I. lis.
P. vit.-A
P. vit.-Z
P. ver.
P. for.
P. gui.
P. vlan
P. put.
BMPR1A
Human-1
Human-2
Chicken
G. wis.
S. und.
S. tris
P. pla.
P. bla.
I. lis.
P. vit.-A
P. vit.-Z
P. ver.
P. gui.
P. put.
P. for.
RGL2
Human
Chicken
G. wis.
P. bla.
P. pla.
S. occ.
S. und.
S. tris
I. lis.
P. vit.-A
P. vit.-Z
P. ver.
P. vlan
P. put.
P. gui.
PFDN6
Human
Chicken
P. ver.
P. put.
P. vlan
P. gui.
G. wis.
S. tris
S. und.
P. vit.-A
I. lis.
P. vit.-Z
P. pla.
P. bla.
ADAMTS4
Humen
Chicken
P. vit.-Z
G. wis.
S. und.
S. tri.
P. pla.
P. bla.
I. les.
P. vit.-A
P. ver.
P. for.
P. put.
P. gui.
B
RGL2-A
P. vit. Chr2 1.20-1.53Mb
I. les. 305.54-304.85Mb
P. pla. 336.56-336.08Mb
P. bla. 402.11-401.02Mb
S. und. 0.89-0.28Mb
S. tri. 0.16-0.83Mb
G. wis. 4.80-5.44Mb
PFDN6-A
P. vit. Chr2 1.17-1.52Mb
I. les. 305.58-304.85Mb
P. pla. 336.56-336.09Mb
P. bla. 0-4.00Mb
S. und. 1.00-0.28Mb
S. tri. 0.14-0.82Mb
G. wis. 4.80-5.44Mb
BMPR1A-A
P. vit. Chr3 167.90-171.32Mb
I. les. 165.34-168.63Mb
P. pla. 6.37-10.52Mb
P. bla. 136.56-131.84Mb
S. und. 160.17-163.34Mb
S. tri. 131.76-128.72Mb
G. wis. 145.42-140.54Mb
AMHR2-A
P. vit. Chr2 166.31-167.63Mb
I. les. 153.48-152.22Mb
P. pla. 165.77-164.23Mb
P. bla. 200.46-198.53Mb
S. und. 176.99-175.50Mb
S. tri. 169.22-167.79Mb
G. wis. 236.86-238.64Mb
ADAMTS4-A
P. vit. Chr15 4.59-4.99Mb
I. les. 2.22-1.81Mb
P. pla. 6.46-6.89Mb
P. bla. 8.83-9.33Mb
S. und. 33.95-33.41Mb
S. tri. 30.81-31.33Mb
G. wis. 14.39-14.86Mb
C
ZW
ZZ
RGL2-Z
PFDN6-Z
ADAMTS4-Z
RGL2-A
PFDN6-A
ADAMTS4-A
TPM
s6/7
s12
s16
Developmental stage
