# Supplementary figures and images for: A near-complete genome assembly of the bearded dragon Pogona vitticeps provides insights into the origin of Pogona sex chromosomes
Source: Gigascience. 2025 Aug 19;14:giaf079. doi: 10.1093/gigascience/giaf079 (PMC12360845; doi:10.1093/gigascience/giaf079)

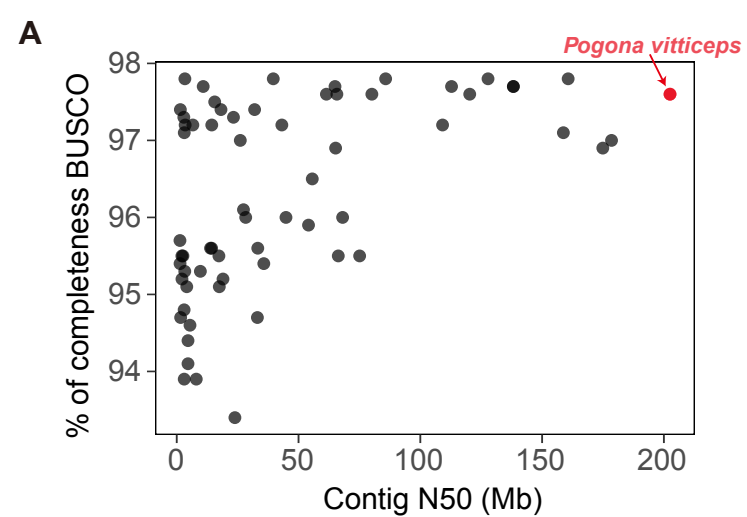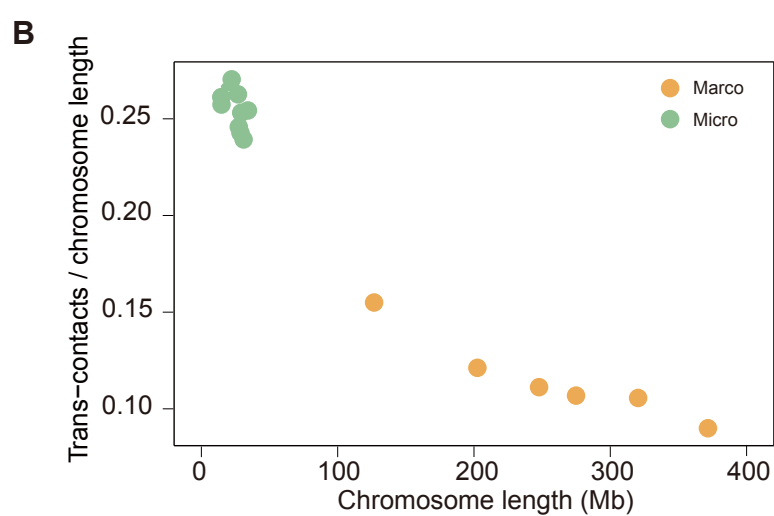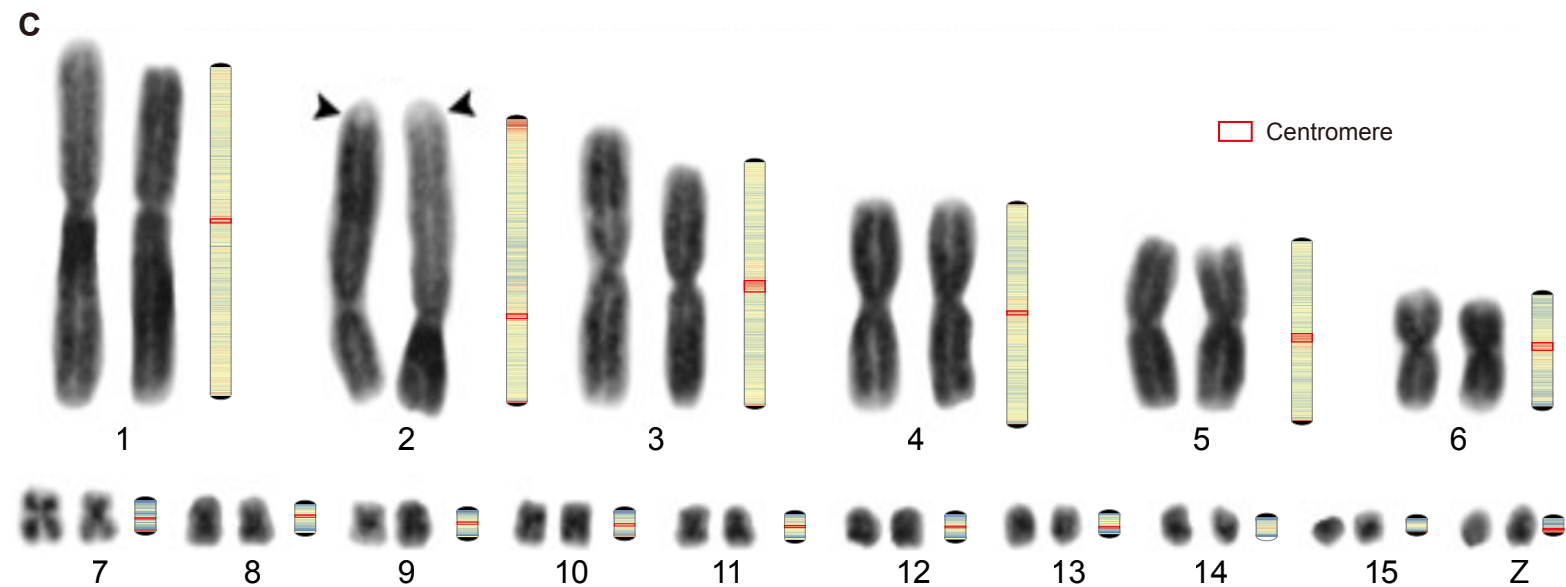

Supplement: giaf079_Supplementary_Files [file giaf079_supplementary_files.zip › Figure S2.pdf]

A

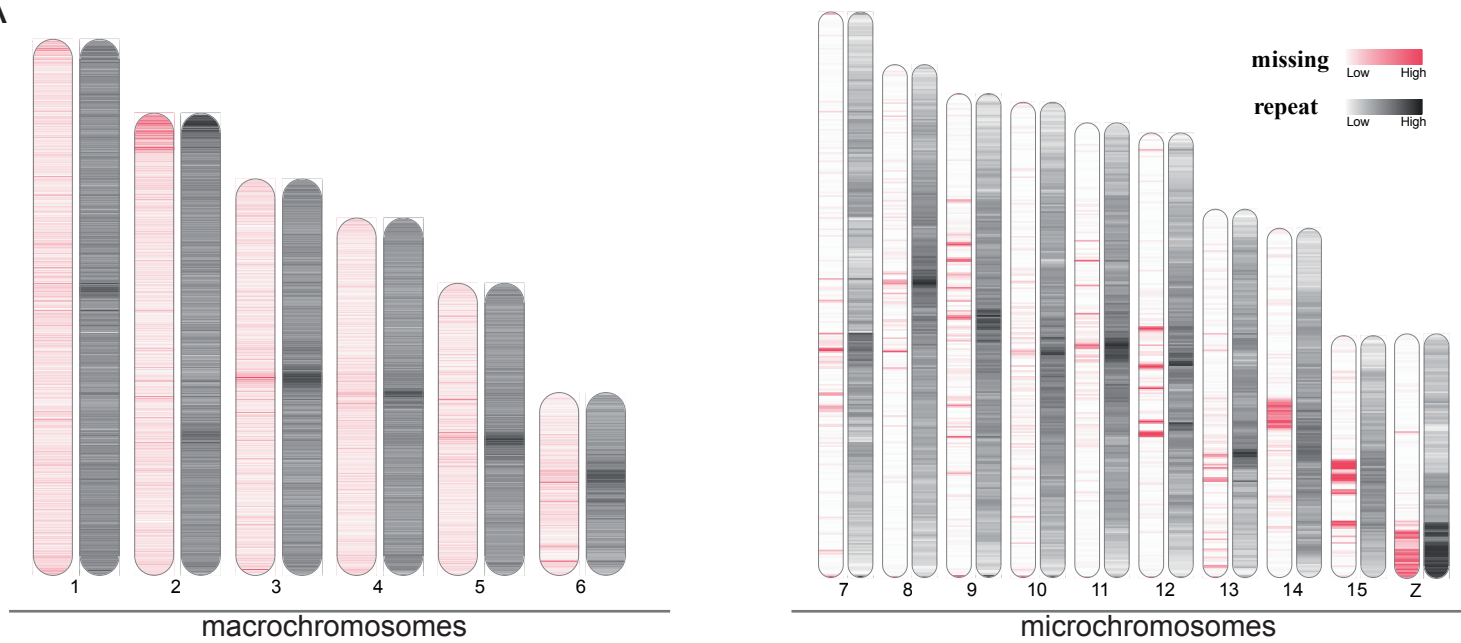

B

missing sequence

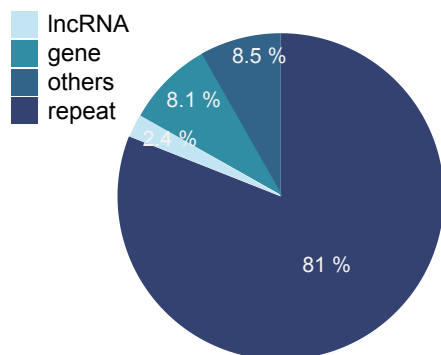

C

coding genes

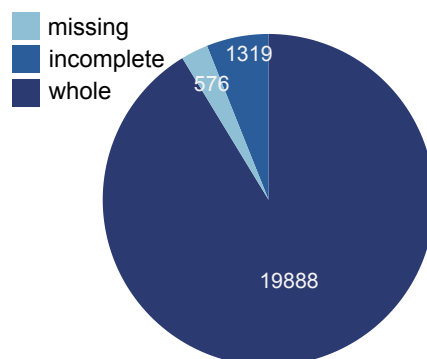

D

lncRNA

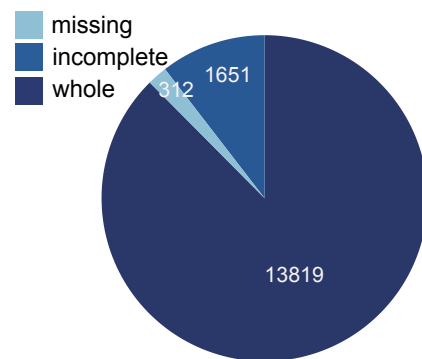

E

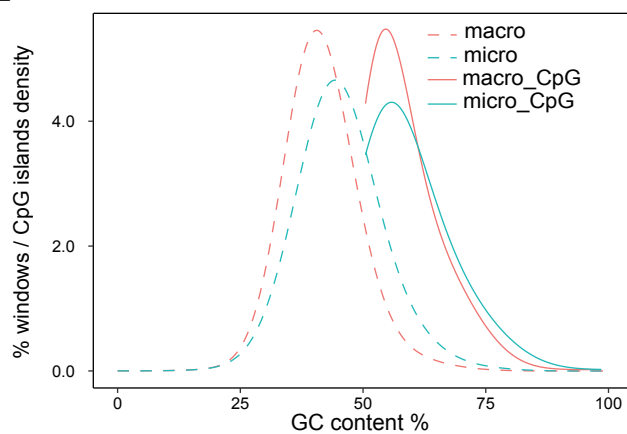

F

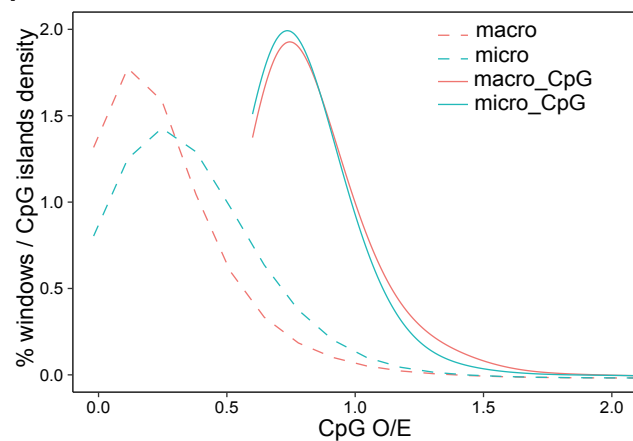

G

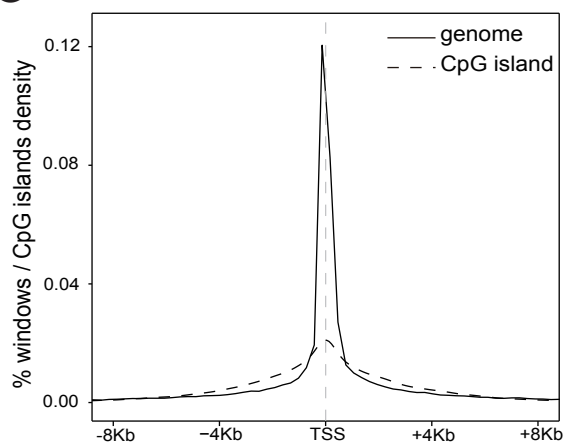

H

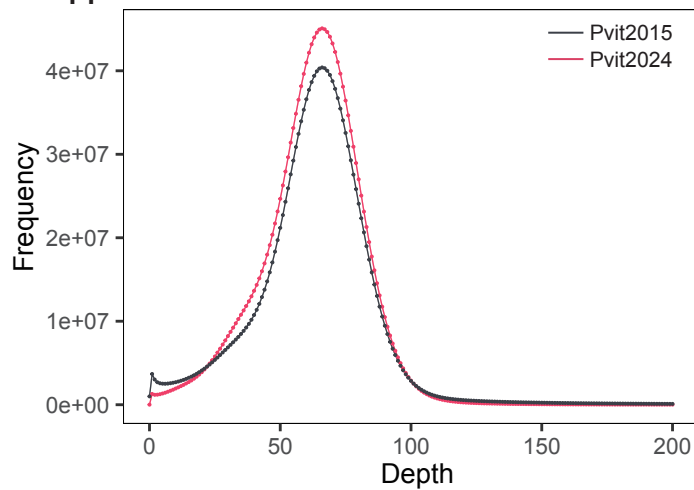

Supplement: giaf079_Supplementary_Files [file giaf079_supplementary_files.zip › Figure S3.pdf]

**lncRNAs expression matrix**

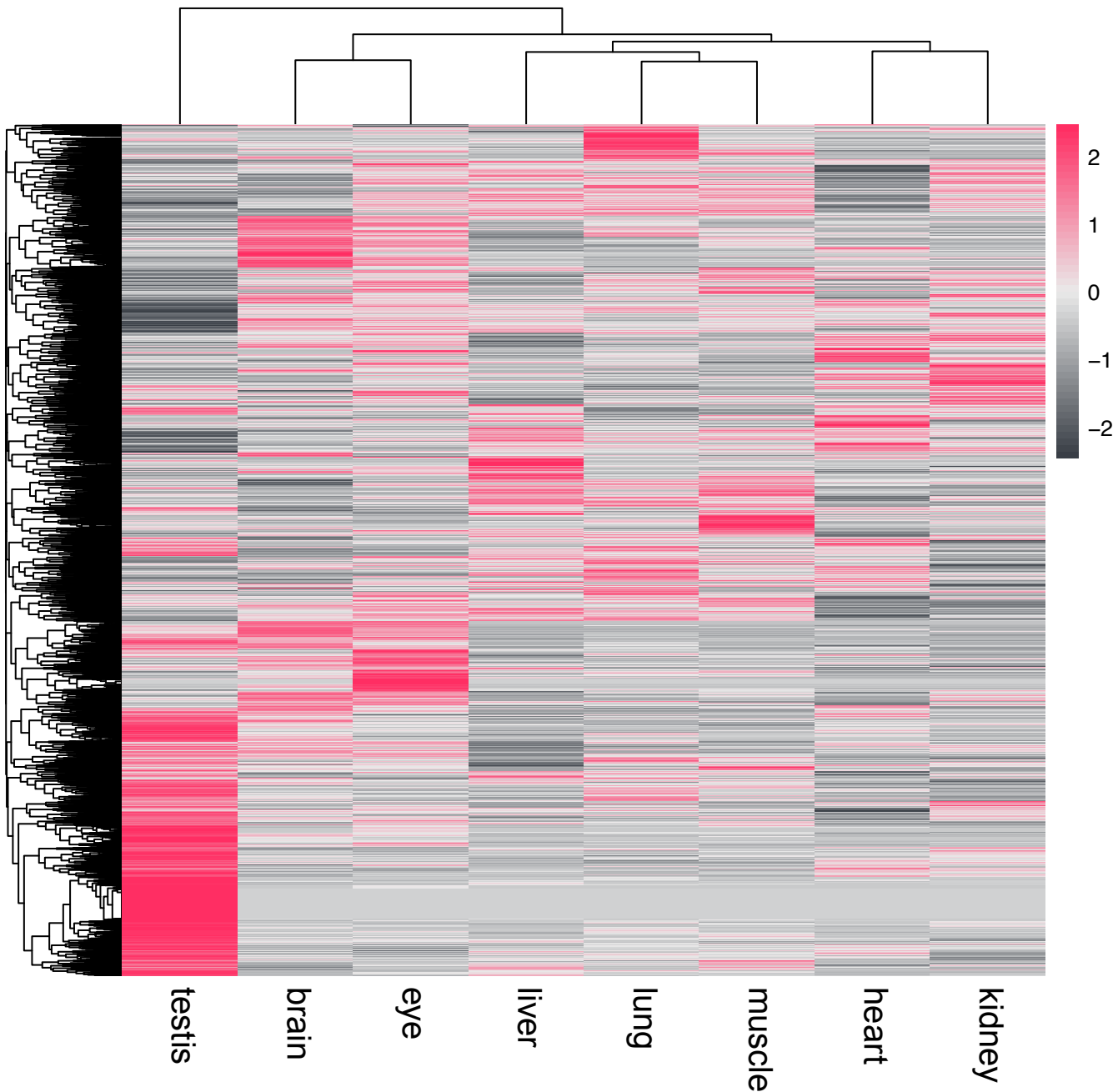

Supplement: giaf079_Supplementary_Files [file giaf079_supplementary_files.zip › Figure S4.pdf]

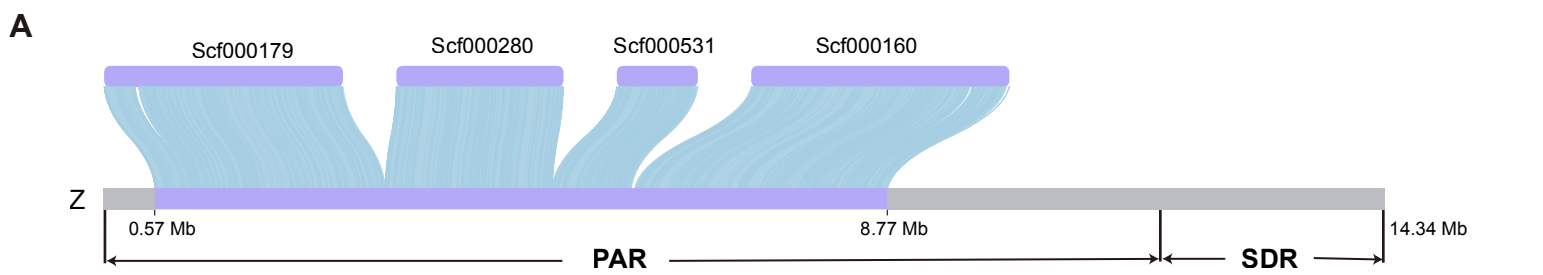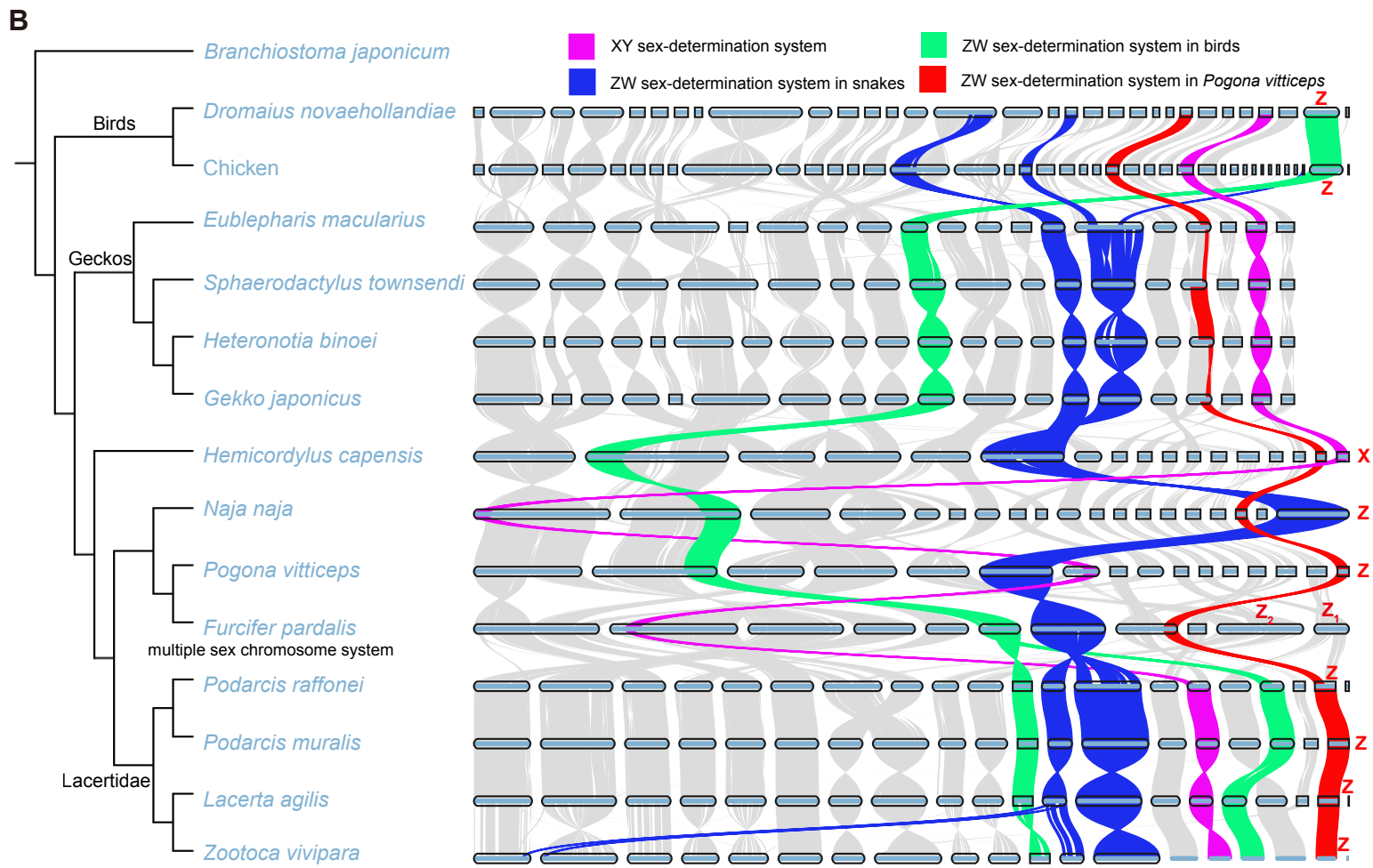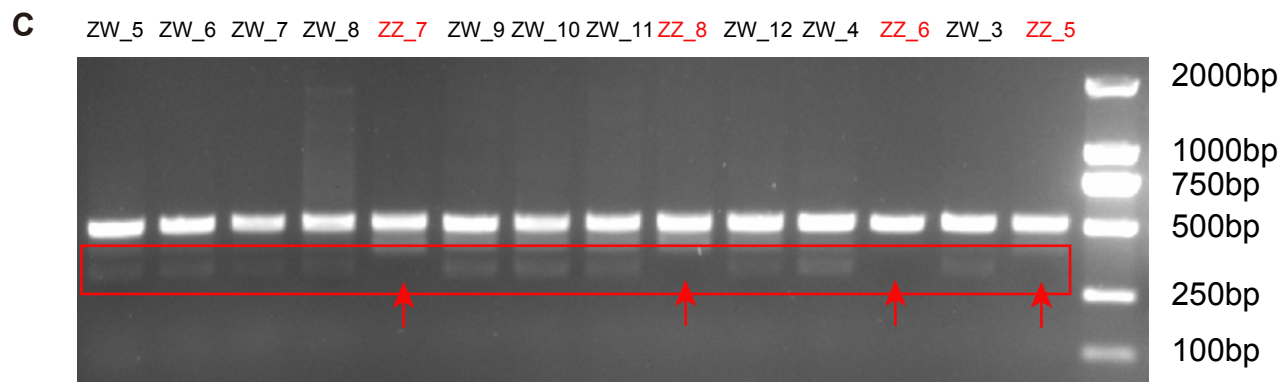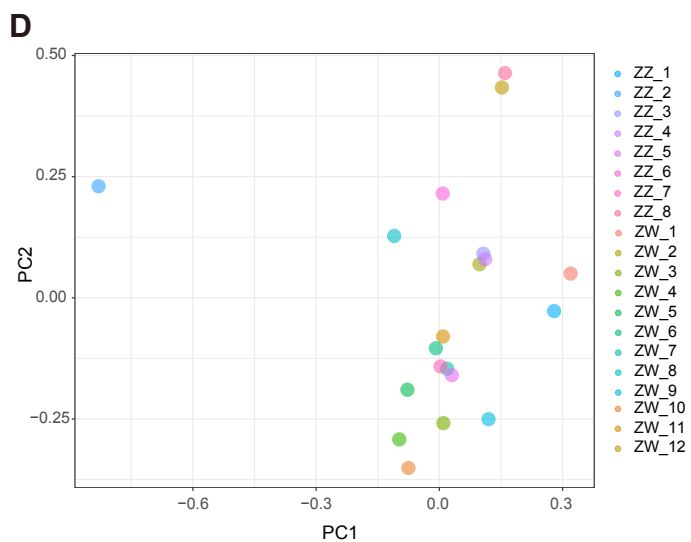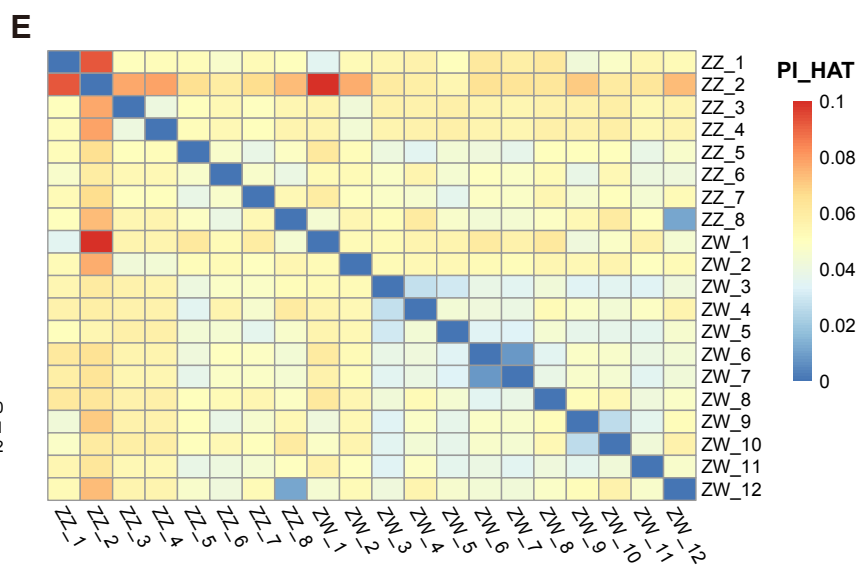

Supplement: giaf079_Supplementary_Files [file giaf079_supplementary_files.zip › Figure S5.pdf]
