# Supplementary material for: A near-complete genome assembly of the bearded dragon Pogona vitticeps provides insights into the origin of Pogona sex chromosomes
Source: Gigascience. 2025 Aug 19;14:giaf079. doi: 10.1093/gigascience/giaf079 (PMC12360845; doi:10.1093/gigascience/giaf079)
Supplement: giaf079_GIGA-D-24-00422_Revision_1 [file giaf079_giga-d-24-00422_revision_1.pdf]

GigaScience

A near-complete genome assembly of the bearded dragon Pogona vitticeps provides insights into the origin of Pogona sex chromosomes

--Manuscript Draft--

**Manuscript Number:**	GIGA-D-24-00422R1
**Full Title:**	A near-complete genome assembly of the bearded dragon Pogona vitticeps provides insights into the origin of Pogona sex chromosomes
**Article Type:**	Research
**Funding Information:**	National Natural Science Foundation of China (32370666) — N/A Qiye Li
**Abstract:**	Background<br><br>The agamid dragon lizard Pogona vitticeps is one of the most popular domesticated reptiles to be kept as pets worldwide. The capacity of breeding in captivity also makes it emerging as a model species for a range of scientific research, especially for the studies of sex chromosome origin and sex determination mechanisms.<br><br>Results<br><br>By leveraging the CycloneSEQ and DNBSEQ sequencing technologies, we conducted whole genome and long-range sequencing for a captive-bred ZZ male to construct a chromosome-scale reference genome for P. vitticeps. The new reference genome is ~1.8 Gb in length, with a contig N50 of 202.5 Mb and all contigs anchored onto 16 chromosomes. Genome annotation assisted by long-read RNA sequencing greatly expanded the P. vitticeps lncRNA catalog. With the chromosome-scale genome, we were able to characterize the whole Z sex chromosome for the first time. We found that over 80% of the Z chromosome remains as pseudo-autosomal region (PAR) where recombination is not suppressed. The sexually differentiated region (SDR) is small and occupied mostly by transposons, yet it aggregates genes involved in male development, such as AMH, AMHR2 and BMPR1A. Finally, by tracking the evolutionary origin and developmental expression of the SDR genes, we proposed a model for the origin of P. vitticeps sex chromosomes which considered the Z-linked AMH as the master sex-determining gene.<br><br>Conclusions<br><br>Our study provides novel insights into the sex chromosome origin and sex determination of this model lizard. The near-complete P. vitticeps reference genome will also benefit future study of amniote evolution and may facilitate genome-assisted breeding.
**Corresponding Author:**	Qunfei Guo<br>BGI Group<br>Wuhan, CHINA
**Corresponding Author Secondary Information:**	
**Corresponding Author's Institution:**	BGI Group
**Corresponding Author's Secondary Institution:**	
**First Author:**	Qunfei Guo
**First Author Secondary Information:**	
**Order of Authors:**	Qunfei Guo
	Youliang Pan
	Wei Dai

Powered by Editorial Manager® and ProduXion Manager® from Aries Systems Corporation

	Fei Guo
	Tao Zeng
	Wanyi Chen
	Yaping Mi
	Yanshu Zhang
	Shuaizhen Shi
	Wei Jiang
	Huimin Cai
	Beiying Wu
	Yang Zhou
	Ying Wang
	Chentao Yang
	Xiao Shi
	Xu Yan
	Junyi Chen
	Chongyang Cai
	Jingnan Yang
	Xun Xu
	Ying Gu
	Yuliang Dong
	Qiye Li
**Order of Authors Secondary Information:**	
**Response to Reviewers:**	Point to point response for Reviewers' Comments:<br><br>In this report, we have addressed all the questions and comments from the two reviewers (marked in italic black), by our point by point responses (marked in blue). All references cited in this document have been placed at the end.<br><br>Point to point response for Reviewer #1<br>Reviewer #1: Guo et al. present a new reference genome for Pogona vitticeps, a widespread reptile model organism that is also common as a domestic animal worldwide. The genome assembly shows much improvement over an older assembly from 2017. There are two points that make this manuscript outstanding from common genome assembly papers:<br>1. The authors find a new sex determination locus in this species.<br>2. the authors use a new nanopore sequencing technology ("CycloneSEQ"), which has so far only described in a preprint:<br>(https://www.biorxiv.org/content/10.1101/2024.08.19.608720v1).<br><br>In my opinion this deserves a publication in Gigascience, but both points must be focused more on a revised manuscript.<br><br>Response<br>We are sincerely grateful for your summary of our work and your recognition of its significance. We also appreciate the numerous constructive suggestions provided, which have significantly enhanced our manuscript. We have carefully revised the manuscript based on your comments to ensure that it includes sufficient evidence and details, and all the revised parts in the manuscript have been highlighted in dark red. We believe that the revised version represents a substantial improvement.

Powered by Editorial Manager® and ProduXion Manager® from Aries Systems Corporation

Below, I will explain these points:
1-1 The authors have sequenced a male individual (ZZ), which means the long-read reference assembly is missing the W-chromosome. PAR and SDR regions are deduced from the Z sequence, by analysis of sequencing coverage of only a few sexed samples (2 females and 4 males). It is unclear if these individuals are from the same family, which could mean that the newly found SD-region could just be a family specific variation. To make the whole story more intriguing and statistical sound the authors should at least test 15 males and 15 females from different P. vitticeps populations for W-specific markers near the proposed AMH deletion. The authors should also show that the prior proposed SD locus (nr5a1) does not carry W-specific mutations in these 15+15 individuals. Furthermore, a phased assembly of a female (ZW) Pogona vitticeps individual, could enable the assembly of the missing W-chr and should be included, it would even improve analysis of W-specific sequences in the proposed additional individuals.

Response
Thank you for this constructive suggestion. Based on your comment, I have broken them down into the following four points and will address each one in turn:

Point 1: PAR and SDR regions are deduced from the Z sequence, by analysis of sequencing coverage of only a few sexed samples (2 females and 4 males). To make the whole story more intriguing and statistical sound the authors should at least test 15 males and 15 females from different P. vitticeps populations for W-specific markers near the proposed AMH deletion.
Response
We sincerely appreciate your insightful suggestion to expand the sample size for validating the PAR/SDR regions. While logistical constraints (e.g., difficulties in obtaining specimens from diverse wild populations due to import restrictions) limited our ability to reach the proposed 15 males and 15 females, we made significant efforts to enhance our cohort.
We collected an additional 14 individuals and rigorously validated their genetic sex using W-specific molecular markers, as described in the Methods section (Sex identification). These markers were analyzed via agarose gel electrophoresis, where ZZ males exhibited a single band and ZW females showed two distinct bands (Figure R1.1; Supplementary Fig. S13) [1]. Combined with the original 6 samples, our final dataset now includes 8 ZZ males and 12 ZW females (Table R1.2).
Although this sample size falls short of the reviewer's recommendation, we note that it aligns with established practices in sex chromosome studies [2]. For instance, recent work on Hippocampus erectus sex determination demonstrated robust PAR/SDR demarcation with comparable sample size (10 females + 10 males) [2]. Notably, our re-analysis incorporating the expanded dataset confirmed consistent PAR and SDR boundaries (see updated Fig. 5C), reinforcing the reliability of our initial findings.
We acknowledge that larger, population-diverse cohorts could further strengthen statistical confidence. However, the current evidence – supported by methodological rigor, consistency across analyses, and congruence with published precedents – provides and compelling foundation for our conclusions regarding Z chromosome architecture in P. vitticeps.

Figure R1.1 The agarose gels for the additional 14 individuals.

Table R1.2 8 ZZ male and 12 ZW female individuals' information used for PAR/SDR demarcation.

Sample ID	Genotype	Total clean data (Gb)	Genome coverage (X)
ZW_1	ZW female	63.19	35.3
ZW_2	ZW female	66.05	36.9
ZZ_1	ZZ male	68.56	38.3
ZZ_2	ZZ male	112.23	62.7
ZZ_3	ZZ male	100.60	56.2
ZZ_4	ZZ male	98.27	54.9
ZW_3	ZW female	246.48	137.70
ZW_4	ZW female	149.29	83.40
ZW_5	ZW female	153.76	85.90
ZW_6	ZW female	165.04	92.20
ZW_7	ZW female	167.01	93.30

Powered by Editorial Manager® and ProduXion Manager® from Aries Systems Corporation

ZW_8ZW female205.85 115.00
ZW_9ZW female185.62 103.70
ZW_10ZW female176.49 98.60
ZW_11ZW female148.75 83.10
ZW_12ZW female147.85 82.60
ZZ_5ZZ male161.82 90.40
ZZ_6ZZ male257.04 143.60
ZZ_7ZZ male187.41 104.70
ZZ_8ZZ male121.36 67.80

Point 2: PAR and SDR regions are deduced from the Z sequence, by analysis of sequencing coverage of only a few sexed samples (2 females and 4 males). It is unclear if these individuals are from the same family, which could mean that the newly found SD-region could just be a family specific variation.
Response
We thank you for highlighting this critical concern. To preclude familial bias in PAR/SDR analysis, our cohort integrates: (1) five geographically/temporally diverse samples from public datasets (Supplementary Table S13), (2) one newly collected sample with no lineage overlap to published data, and (3) 14 additional samples collected from different locations. All individuals were verified as unrelated through breeder records and genomic validation. Besides this, to empirically rule out familial relatedness, we conducted two complementary genomic analyses:
1.Principal Component Analysis (PCA) was performed using Plink v1.07 (RRID:SCR_001757) [3] on genome-wide SNPs called using FreeBayes v0.9.21 (RRID:SCR_010761) [4]. The resulting PCA plot (Figure R1.3) revealed no clustering patterns indicative of shared ancestry, with samples broadly dispersed across genetic space.
2.Kinship analysis was conducted using PLINK's pairwise PI_HAT estimates, which quantify the proportion of alleles shared identical-by-descent (IBD). All pairwise values fell within a narrow range of 0.012 ~ 0.100 (Figure R1.4), well below the threshold for even distant relatedness (PI_HAT > 0.1) [5]. Critically, no sample pairs approached values suggestive of first- or second-degree relationships (PI_HAT > 0.25), confirming the absence of parent-offspring, sibling, or avuncular connects.
The combination of samples from different sources, PCA-based population structure validation, and kinship coefficient analysis provides robust evidence that our cohort is devoid of familial relatedness. This ensures that the observed Z-W coverage patterns and the identification of the SDR region reflect species-level genomic architecture rather than lineage-specific artifacts.

Figure R1.3 PCA analysis of 20 samples.

Figure R1.4 kinship analysis of 20 samples.

Point 3: The authors should also show that the prior proposed SD locus (nr5a1) does not carry W-specific mutations in these 15+15 individuals.
Response
We conducted a comprehensive analysis to evaluate potential W-specific mutations in the nr5a1 locus. First, we compared genetic diversity patterns between the nr5a1 region and autosomes (Chr1~Chr15). Comparative analysis of SNP density (per Mb) and average sequencing coverage between the nr5a1 gene region and autosomes (Chr1~ Chr15) (Figure R1.5 and Figure R1.6) revealed no evidence of a two-fold depth reduction across all 20 samples.
Building on these global patterns, we performed detailed nucleotide-resolution analysis of sequencing coverage across all sites within the nr5a1 region (Supplementary Table S17). Among 26,763 interrogated loci, 24,072 (89.95%) exhibited consistent homozygosity across all samples. None of the 2691 retained loci exhibited mutations specific to ZW individuals, with any observed mutation being consistently shared across all ZW individuals. This systematic evaluation conclusively excludes W-linked mutational signatures in nr5a1.
Collectively, our multilayered analysis – spanning regional diversity metrics, depth profiles, and allele-specific patterns – confirms the absence of W-specific mutations in nr5a1. These findings support its characterization as a conserved sex-determining gene on the Z chromosome, rather than a W-derived element.

Powered by Editorial Manager® and ProduXion Manager® from Aries Systems Corporation

Figure R1.5 SNP density per Mb between nr5a1 region and Autosomes (Chr1~Chr15).
Figure R1.6 Average sequencing coverage (X) between nr5a1 region and Autosomes (Chr1~Chr15).

Point4: Furthermore, a phased assembly of a female (ZW) Pogona vitticeps individual, could enable the assembly of the missing W-chr and should be included, it would even improve analysis of W-specific sequences in the proposed additional individuals.
Response
We fully acknowledge the importance of resolving the W chromosome to strengthen our conclusions. However, due to limitations in project funding, achieving W chromosome assembly would require collecting and sequencing data from parents and F1 ZW females, as well as performing haploid assembly. Such a task is beyond the scope of the current project. Nevertheless, we hope to address this in future work.

1-2 A technology aware reader would like to see more information on the specifics of the CycloneSEQ data quality and handling and maybe a comparison to competing technologies.
Response
We thank you for emphasizing the importance of comparing emerging sequencing technologies with other sequencing platforms. However, since we did not sequence data from the PacBio and ONT platforms in this study, direct comparisons are not feasible. For more detailed sequencing performance metrics of this sequencing technology, please refer to the preprint article “A single-molecule nanopore sequencing platform” [6].

1-3 Which enzymes and buffers were used to prepare the library? In the sections on the methods, there are only superficial descriptions such as (DNA repair buffer/enzyme, DNA clean beads, wash buffer for long fragments). Is it a kit or were the enzymes and buffers purchased individually?
Response
We thank you for highlighting the need for methodological transparency. The CycloneSEQ library was prepared using the CycloneSEQ Universal DNA Library Prep Set kit (H940-000013), a commercially available kit optimized for long-read sequencing. The full protocol and reagent specifications are available in the MGI Technical Documentation:
(https://en.mgitech.cn/Home/Products/instruments_info/id/65.html).

1-4 I cannot find the procedure for preparation and sequencing of the long-read cDNA libraries.
Response
We apologize for any ambiguity in the original manuscript and appreciate your request for clarification. The long-read cDNA libraries were prepared using the MGI CycloneSEQ RNA Library Prep Kit, following the manufacturer’s optimized protocol. For specific and detailed methodological descriptions, please refer to the Methods section “CycloneSEQ long-read RNA-seq library preparation and sequencing”. (Lines 587-596)

1-5 How many flowcells were needed to generate the different datasets?
Response
We thank you for raising this important technical detail. The number of flowcells used to generate each dataset has been thoroughly documented in Table S1 of the Supplementary Materials (Table R1.7).

Table R1.7 The number of flowcells for different datasets.

Data type	Sample/Tissue	Flowcell number
CycloneSEQ long-read WGS data	muscle and lung	36
CycloneSEQ based Pore-C data	liver	6
CycloneSEQ long-read RNA-seq data	eye	3
	kidney	4
	live	3
	lung	3
	muscle	4
	testis	3

Powered by Editorial Manager® and ProduXion Manager® from Aries Systems Corporation

brain5
heart3

1-6 How do the read-length distributions look like (statistics over all reads not only selected 40Kb+)?
Response
We thank you for requesting clarity on read-length distributions. As show in Figure R1.8, the full distribution of all raw CycloneSEQ reads (prior to size selection), with a N50 of 32.4 kb and over 40% of reads exceed 40 kb.

Figure R1.8 Frequency distribution of read lengths in all data.

1-7 How was the variability between those runs, especially cumulative output over time?
Response
We thank you for the highlighting the importance of sequencing run consistency. The sequencing for this study were primarily conducted using the WT platform of CycloneSEQ, a platform optimized for moderate-throughput applications. For the majority of run, the WT platform consistently generated 15~20 Gb of data per flowcell, with minimal variability across experimental batches. The WT platform’s reliability in delivering stable yields within the 15~20 Gb range ensured robust data generation for the core assembly and sex chromosome analyses.

1-8 What hardware was needed to run the basecalling and what was the runtime?
Response
Basecalling for CycloneSEQ runs was performed in real-time during sequencing using the integrated computational module of the WT instrument. The process utilized a dedicated hardware configuration comprising an Intel i7/i9 CPU (24 cores), 64 GB DDR4 RAM, a NVIDIA RTX 4090D GPU, and a 512 GB NVMe system drive, which collectively enabled simultaneous signal acquisition and basecalling without delaying the sequencing workflow. Each run on the WT platform completed within 24 hours, including both sequencing and Basecalling. This integrated approach ensured rapid data availability while maintaining high accuracy.

1-9 How is the Q-Value distribution of the reads?
Response
The Q-value distribution of raw CycloneSEQ reads in shown in Figure R1.9.

Figure R1.9 Frequency count distribution of Q-value for reads in all data.

1-10 Why is the consensus accuracy of the assembly low (Q36.4)? can it be improved? Typically reference quality genomes should have Q40+.
Response:
We thank you for raising the critical question regarding the consensus accuracy (QV=36.4) of our assembly. The observed QV value primarily stems from the challenges inherent to assembling this species’ highly heterozygous genome, which exhibits extensive allelic divergence between homologous chromosomes. During the assembly process, regions of high heterozygosity often led to erroneous merging of divergent haplotypes into single contigs. These artifacts, misinterpreted as sequencing errors during quality assessment, disproportionately inflated base-level discordance and reduced the overall QV score.

1-11 Which regions of the genome display lower consensus accuracies (is it random or sequence specific)?
Response:
By assembly-specific 21-bp k-mers distribution feature, we find that the assembly errors are random rather than sequence specific. As demonstrated in Figure R1.10, we systematically mapped assembly-specific 21-bp k-mers (entirely absent in raw WGS data) across the genome. Their uniform spatial distribution provides definitive evidence that assembly errors are randomly distributed. This pattern strongly argues against locus-specific technical biases in genome reconstruction.

Figure R1.10 The frequency distribution of assembly-specific 21-bp kmers (absent in raw WGS data) within 500-kb windows (Right) and the repeat density (Left).

Minor comments:
1-12 L.900: PRJNAxxxxxx looks like a placeholder, insert the true number,please.
Response
Thanks. We have replaced the placeholder “PRJNAxxxxxx” with PRJNA1184386 in Line 923. Morever, all sequencing data of 14 additional samples used for sex determination analysis have been deposited in public databases (NCBI: PRJNA1184386; CNSA: CNP0005509) to ensure transparency and reproducibility.

Point to point response for Reviewer #2
Reviewer #2: Impressive work but needs major revision to be accepted. The authors compressed everything in the result section and did not put enough effort into the other sections. Introduction and discussion need major changes and more details regarding many aspects of the study that comes in the results. Methods need rearrangement. It's common to keep the order of methods such as first DNA extraction, then sequencing and so on. The data availability needs to be completed. Biosamples for each sequenced tissue, all the reads, and even intermediate assemblies need to be submitted to the database and reported in the manuscript. More specific comments are on the copy of the manuscript attached for the authors.
Response
We sincerely thank you for the positive assessment of our work and for the thoughtful, detailed feedback that has significantly strengthened the manuscript. We will proceed to reply to each comment marked by the reviewer in the manuscript one by one. Finally, all the revised parts in the manuscript have been highlighted in dark red.

2-1 Software and database versions are missing throughout the manuscript.
Response
Thank you for highlighting the importance of software and database version transparency for reproducibility. We have rigorously updated the Methods section to include full version details for all software and databases used in this study, aligning with GigaScience’s reporting standards.

2-2 The background description is too poor.
Response
Thank you for your feedback. Upon review, we agree that it was insufficient. We have now rewritten the background section to provide a more comprehensive and detailed description.

2-3 Lines 29 “N50 of 202.5 Mb and all contigs anchored”: Explain if it's any better than previous genomes published for this organism.
Response
We appreciate your suggestion. We have added key assembly improvement metrics in the Abstract: “The new reference genome is ~1.8 Gb, shows a 5,700-fold improvement in contiguity (N50 = 202.5 Mb vs 35.5 kb) and complete chromosomal anchoring (16 scaffolds vs. 13,749).” (Lines 30-32)

2-4 The conclusions are too short.
Response
Thank you for your feedback. Due to word count limitations (not exceed 250 words), the conclusions were kept concise. However, we will expand on the key findings and implications in the revised manuscript to provide a more comprehensive summary.

2-5 Introduction does not include many info that is needed.
Response
Thank you for your feedback. As per your suggestion, we have added more relevant information and removed unnecessary details. We have also reorganized the paragraphs accordingly, with significant revisions made to the first paragraph of the Introduction to better align with your expectations.

2-6 Lines 46 “In the wild, P. vitticeps has a natural distribution in the eastern and central Australia.” Ambiguous
Response
Thank you for your feedback. Following your suggestion regarding the Introduction, we

	have reviewed the sentence in Line 46 and agree that it is ambiguous and not essential to the main content. Therefore, we have removed it from the revised manuscript.<br><br>2-7 Lines 48-50 “It is one of the most popular reptile pets nowadays, owing to ease of breeding in captivity, presence of morphs varying in scale coloration, and docility to their owners.” Unrelated<br>Response<br>Thank you for your feedback. We have removed the sentence in the revised manuscript to ensure the content remains focused and aligned with the main theme.<br><br>2-8 Lines 54-56 “as it possesses a ZZ/ZW genetic sex determination system whereas the final sex is also influenced by environmental temperature during embryonic development.” Break it into a separate sentence<br>Response<br>Thank you for your feedback. We have addressed the sentence in Lines 54-56 by splitting it and reorganizing the content for better clarity and flow.<br><br>2-9 Lines 109-114 “probably due to the well-known challenge of assembling the highly repetitive telomeric regions [7] which was also evidenced by the prevalent absence of telomeric sequences in the chromosomal ends of most squamate genomes published so far (Supplementary Table S9). However, we were able to find many CycloneSEQ reads containing TRUs before assembling, suggesting that the missing of telomeres are mainly due to computational drawback rather than sequencing omission.” move to discussion<br>Response<br>We thank you for the constructive suggestion. As recommended, the highlighted text has been relocated to the Discussion section (now Lines 438-446) to better contextualize telomere assembly challenges within broader genome assembly limitations. This adjustment improves the manuscript’s logical flow by positioning technical limitations within comparative biological interpretations.<br><br>2-10 Lines 132-133“our Pvit2024 assembly outperformed all other squamate genomes reported so far in terms of continuity” add discussion<br>Response<br>We appreciate this suggestion. The statement regarding assembly continuity has been incorporated into the Discussion (Lines 436-438).<br><br>2-11 Lines 152 “7,480 Sauropsida conserved genes” more details. did you find them?<br>Response<br>Thank you for raising this critical point. We have clarified (Lines 152-153) that the 7480 conserved genes derive from the Sauropsida_odb10 BUSCO dataset, representing single-copy orthologs shared across birds, squamates, and turtles.<br><br>2-12 Lines 162 “good agreements” What does this mean? "Good" needs to be replaced.<br>Response<br>We sincerely thank you for the precise suggestion to improve terminology clarity. In response, the phrase “good agreements” has been replaced with “high consistency” to objectively describe the Hi-C validation results.<br><br>2-13 Lines 169 cite more studies that published microchromosomes in lizards.<br>Response<br>We thank you for emphasizing the importance of contextualizing our findings within the broader literature on reptilian microchromosomes. In response, we have added three key references to the revised manuscript that explicitly discuss microchromosomes in squamates [8-10].<br><br>2-14 Lines 174 “GC content, lower repeat content, and more frequent inter-chromosomal interaction” Why do we care?<br>Response<br>We thank you for raising this important point. The three microchromosomal features highlighted in Lines 174 — GC content, repeat content, and inter-chromosomal interaction patterns — serve as established quality metrics in Sauropsida genome

Powered by Editorial Manager® and ProduXion Manager® from Aries Systems Corporation

assembly studies. Here, we report these parameters to objectively validate the completeness and accuracy of our assembly, rather than to explore their biological implications.

2-15 Lines 175-176 “These multiple lines of evidence together highlight the reliability of the Pvit2024 chromosomal assembly.” Move to discussion
Response
We thank you for this suggestion. The sentence highlighting assembly reliability has been moved to the Discussion (Lines 447-450) and expanded with supporting validation data. This revision consolidates evidence for assembly quality within the broader genomic context.

2-16 Microchromosome background info: add background for any concept that comes to result section.
Response
Thank you for your suggestion. Detailed background information on “Microchromosome” has been added to the Introduction section to enhance clarity and provide context.

2-17 Lines 439-445 “P. vitticeps possesses a ZZ male/ZW female genetic sex determination (GSD) system. However, the default sexual fate of ZZ embryos can be overwritten by high incubation temperature, leading to sex reversal of ZZ males to ZZ females [11]. The capacity of a ZZ embryo to develop as a normal female without the help of W chromosome suggests that sexual fate is most likely determined by a dosage-sensitive gene on the Z chromosome [12]. Therefore, the achievement of a complete Z chromosome sequence represents a particularly important beginning for disclosing the sex-determining cascade in P. vitticeps. " This needs to go to introduction.
Response
Thank you for your suggestion. We have incorporated the content from Lines 439-445 into the first paragraph of the Introduction to provide a more comprehensive background and context for the study.

2-18 Lines 516-517 BioSample ID?
Response
Thank you for your attention! The BioSample information for the corresponding samples is presented in the table below (Table R2.1). Additionally, we have included this information in the supplementary materials for readers’ reference. Please note that there are some issues with the CycloneSEQ data upload to NCBI, which are currently being resolved. However, the corresponding data are fully accessible and available for download in the CNGBdb database.
BioSample ID information have been added to Supplementary Table S1.

Table R2.1 BioSample ID for all uploaded datasets.
Data typeInstrument/PlatformSample/TissueNCBI
Biosample No.NCBI
Accession No.CNSA
Accession No.
CycloneSEQ long-read RNA-seq
dataCycloneSEQeyeSAMN44670089***CNS1198628
kidneySAMN44670090***CNS1198630
liveSAMN44670091***CNS1198631
lungSAMN44670092***CNS1198632
muscleSAMN44670093***CNS1198633
testisSAMN44670094***CNS1198634
brainSAMN44670095SRR31361862CNS1198627
heartSAMN44670096SRR31361861CNS1198629
DNBSEQ short-read RNA-seq
dataDNBSEQbrainSAMN44670097SRR31361884CNS1065805
eyeSAMN44670098SRR31361883CNS1065806
heartSAMN44670099SRR31361882CNS1065807
kidneySAMN44670100SRR31361881CNS1065808
liverSAMN44670101SRR31361880CNS1065809
lungSAMN44670102SRR31361879CNS1065810

	muscleSAMN44670103SRR31361878CNS1065811<br>testisSAMN44670104SRR31361877CNS1065812<br><br>2-19 Lines 533 Keep the order of the sections in a rational order. First extraction.<br>Response<br>We thank you for emphasizing the importance of methodological clarity. The Methods section has been restructured to follow a logical workflow, beginning with Sample collection, followed by different kinds of library preparation and sequencing (include CycloneSEQ long-read WGS data, CycloneSEQ based Pore-C data and CycloneSEQ long-read RNA-seq data). This reorganization aligns with best practices in genomics reporting and enhances reproducibility.<br><br>2-20 Lines 614 All reads and intermediate assemblies need their database information. (SRR?)<br>Response<br>We thank you for emphasizing the importance of open data access. All sequencing data have been deposited in NCBI and CNSA. The detail information can be found in Table R2.1 and Supplementary Table S1. And the intermediate assemblies have been deposited in GigaDB database.<br><br>Table R2.2 The accession ID of NCBI and CNSA for all uploaded datasets<br>Data typeInstrument/PlatformSample/TissueAccession (NCBI)Accession (CNSA)<br><br>CycloneSEQ long-read WGS dataCycloneSEQmuscle and lungSAMN44670087CNS1198625<br>DNBSEQ short-read WGS dataDNBSEQmuscle of neckSRR31361869/SRR31361868CNS1065816<br>muscle of tailSRR31361871/SRR31361870CNS1065815<br>CycloneSEQ based Pore-C dataCycloneSEQliverSRR31361885<br>CNS1198626<br>Hi-C dataDNBSEQmuscle (Hi-C_1 data)SRR31361875/SRR31361876<br>CNS1065813<br>muscle (Hi-C_2 data)SRR31361872/SRR31361873<br>CNS1065814<br>CycloneSEQ long-read RNA-seq dataCycloneSEQeyeSAMN44670089CNS1198628<br>kidneySAMN44670090CNS1198630<br>liveSAMN44670091CNS1198631<br>lungSAMN44670092CNS1198632<br>muscleSAMN44670093CNS1198633<br>testisSAMN44670094CNS1198634<br>brainSRR31361862CNS1198627<br>heartSRR31361861CNS1198629<br>DNBSEQ short-read RNA-seq dataDNBSEQbrainSRR31361884CNS1065805<br>eyeSRR31361883CNS1065806<br>heartSRR31361882CNS1065807<br>kidneySRR31361881CNS1065808<br>liverSRR31361880CNS1065809<br>lungSRR31361879CNS1065810<br>muscleSRR31361878CNS1065811<br>testisSRR31361877CNS1065812<br><br>2-21 Lines 681 “homologous” more details.<br>Response<br>We thank you for highlighting the need for methodological precision. The revised manuscript (Methods – Repetitive element annotation, Lines 698-702) now provides comprehensive details on the homology-based annotation pipeline.<br><br>2-22 Lines 709 “winnowmap -u b -K 4G -G 135k -ax splice ref fastq.” No italic<br>Response<br>Thanks. We have revised it accordingly (Line 729).<br><br>2-23 Lines 900 PRJNAxxxxxx<br>Response<br>Thanks. We have replaced the placeholder “PRJNAxxxxxx” with PRJNA1184386 in

Powered by Editorial Manager® and ProduXion Manager® from Aries Systems Corporation

	Line 923.<br><br>References:<br>1.Quinn, A.E., et al., Extension, single-locus conversion and physical mapping of sex chromosome sequences identify the Z microchromosome and pseudo-autosomal region in a dragon lizard, Pogona vitticeps. Heredity, 2010. 104(4): p. 410-417.<br>2.Long, X., et al., Independent evolution of sex chromosomes and male pregnancy–related genes in two seahorse species. Molecular Biology and Evolution, 2023. 40(1): p. msac279.<br>3.Purcell, S., et al., PLINK: a tool set for whole-genome association and population-based linkage analyses. The American journal of human genetics, 2007. 81(3): p. 559-575.<br>4.Garrison, E. and G. Marth, Haplotype-based variant detection from short-read sequencing. arXiv preprint arXiv:1207.3907, 2012.<br>5.Manichaikul, A., et al., Robust relationship inference in genome-wide association studies. Bioinformatics, 2010. 26(22): p. 2867-2873.<br>6.Zhang, J.-Y., et al., A single-molecule nanopore sequencing platform. bioRxiv, 2024: p. 2024.08. 19.608720.<br>7.Bresler, M.a., et al., Telescoper: de novo assembly of highly repetitive regions. Bioinformatics, 2012. 28(18): p. i311-i317.<br>8.Pinto, B.J., et al., A lizard is never late: squamate genomics as a recent catalyst for understanding sex chromosome and microchromosome evolution. Journal of Heredity, 2023. 114(5): p. 445-458.<br>9.Waters, P.D., et al., Microchromosomes are building blocks of bird, reptile, and mammal chromosomes. Proceedings of the National Academy of Sciences, 2021. 118(45): p. e2112494118.<br>10.Lisachov, A.P. and P.M. Borodin, Microchromosome polymorphism in the sand lizard, Lacerta agilis Linnaeus, 1758 (Reptilia, Squamata). Comparative Cytogenetics, 2016. 10(3): p. 387.<br>11.Whiteley, S.L., et al., Two transcriptionally distinct pathways drive female development in a reptile with both genetic and temperature dependent sex determination. PLoS Genetics, 2021. 17(4): p. e1009465.<br>12.Quinn, A.E., et al., Temperature sex reversal implies sex gene dosage in a reptile. Science, 2007. 316(5823): p. 411-411.
**Additional Information:**	
**Question**	**Response**
Are you submitting this manuscript to a special series or article collection?	No
**Experimental design and statistics**<br><br>Full details of the experimental design and statistical methods used should be given in the Methods section, as detailed in our Minimum Standards Reporting Checklist. Information essential to interpreting the data presented should be made available in the figure legends.<br><br>Have you included all the information requested in your manuscript?	Yes
**Resources**	Yes

A description of all resources used, including antibodies, cell lines, animals and software tools, with enough information to allow them to be uniquely identified, should be included in the Methods section. Authors are strongly encouraged to cite Research Resource Identifiers (RRIDs) for antibodies, model organisms and tools, where possible.<br><br>Have you included the information requested as detailed in our Minimum Standards Reporting Checklist?	
**Availability of data and materials**<br><br>All datasets and code on which the conclusions of the paper rely must be either included in your submission or deposited in publicly available repositories (where available and ethically appropriate), referencing such data using a unique identifier in the references and in the “Availability of Data and Materials” section of your manuscript.<br><br>Have you have met the above requirement as detailed in our Minimum Standards Reporting Checklist?	Yes

Manuscript Click here to access/download;Manuscript;Pogona vitticeps manuscript 0930.docx

A near-complete genome assembly of the bearded dragon *Pogona vitticeps* provides insights into the origin of *Pogona* sex chromosomes

Qunfei Guo[1,9], Youliang Pan[1,9], Wei Dai[1,9], Fei Guo[2], Tao Zeng[2], Wanyi Chen[2,3], Yaping Mi[1,4], Yanshu Zhang[1,5], Shuaizhen Shi[1,6], Wei Jiang[2], Huimin Cai[7], Beiying Wu[1,8], Yang Zhou[2], Ying Wang[1], Chentao Yang[2], Xiao Shi[2], Xu Yan[2], Junyi Chen[2], Chongyang Cai[2], Jingnan Yang[2], Xun Xu[2], Ying Gu[2*], Yuliang Dong[2*], Qiye Li[1,2*]

[1]BGI Research, Wuhan 430074, China

[2]BGI Research, Shenzhen 518083, China

[3]School of Life Sciences, Southwest University, Chongqing 400715, China

[4]College of Life Sciences, Northwest University, Xi'an 710069, China

[5]College of Future Technology, University of Chinese Academy of Sciences, Beijing 100049, China

[6]College of Life Sciences, University of Chinese Academy of Sciences, Beijing 100049, China

[7]Women's Hospital, School of Medicine, Zhejiang University, Hangzhou, 310006, China

[8]School of Biology and Biological Engineering, South China University of Technology, 510006 Guangzhou, China

[9]These authors contributed equally

[*]Corresponding authors:

guying@genomics.cn (Y.G.); dongyuliang@genomics.cn (Y.D.); liqiye@genomics.cn (Q.L.)

Abstract

Background: The agamid dragon lizard *Pogona vitticeps* is one of the most popular domesticated reptiles to be kept as pets worldwide. The capacity of breeding in captivity also makes it emerging as a model species for a range of scientific research, especially for the studies of sex chromosome origin and sex determination mechanisms.

Results: By leveraging the CycloneSEQ and DNBSEQ sequencing technologies, we conducted whole genome and long-range sequencing for a captive-bred ZZ male to construct a chromosome-scale reference genome for *P. vitticeps*. The new reference genome is ~1.8 Gb in length, with a contig N50 of 202.5 Mb and all contigs anchored onto 16 chromosomes. Genome annotation assisted by long-read RNA sequencing greatly expanded the *P. vitticeps* lncRNA catalog. With the chromosome-scale genome, we were able to characterize the whole Z sex chromosome for the first time. We found that over 80% of the Z chromosome remains as pseudo-autosomal region (PAR) where recombination is not suppressed. The sexually differentiated region (SDR) is small and occupied mostly by transposons, yet it aggregates genes involved in male development, such as *AMH*, *AMHR2* and *BMPR1A*. Finally, by tracking the evolutionary origin and developmental expression of the SDR genes, we proposed a model for the origin of *P. vitticeps* sex chromosomes which considered the Z-linked *AMH* as the master sex-determining gene.

Conclusions: Our study provides novel insights into the sex chromosome origin and sex determination of this model lizard. The near-complete *P. vitticeps* reference genome will also benefit future study of amniote evolution and may facilitate genome-assisted breeding.

Introduction

The central bearded dragon *Pogona vitticeps*, first described by German zoologist Ernst Ahl about a century ago, is an omnivorous lizard species belonging to the squamate family Agamidae [1]. In the wild, *P. vitticeps* has a natural distribution in the eastern and central Australia, where these lizards have adapted to the arid to semiarid habitats of the Australian inlands [2]. Yet, as a pet species, *P. vitticeps* is indeed commonly found around the world. It is one of the most popular reptile pets nowadays, owing to ease of breeding in captivity, presence of morphs varying in scale coloration, and docility to their owners [3]. And thanks to its easy accessibility and maintenance, *P. vitticeps* is also becoming a popular reptile model for a variety of research topics, such as amniote brain function and evolution [4, 5], chromosome rearrangement [6, 7], germline mutation rate [8], gene regulation of hibernation [9, 10], and especially, the mechanisms for sex chromosome origin and sex determination [11, 12], as it possesses an ZZ/ZW genetic sex determination system whereas the final sex is also influenced by environmental temperature during embryonic development [13].

While many studies involving *P. vitticeps* in the past decade tightly rely on its genome assembly and annotation, the current reference genome is still quite fragmented. This reference genome was constructed with a wild-caught ZZ male individual, based on Illumina short-read sequencing data generated from gradient libraries with insert size ranging from 250 bp to 40 kb [14]. Due to the limitation of short-read sequencing, the contig N50 of this assembly version is merely 35.5 kb in length, lag far behind the common standard of a reference genome (> 1 Mb) as proposed by the Earth BioGenome Project (EBP) [15] and the Vertebrate Genomes Project (VGP) [16] in recent years. Although this draft genome assembly was later scaffolded by different approaches that finally made ~42 % of the genomic sequences anchored to chromosomes [6], the low anchoring percentage still limits its use in chromosome-scale investigation. For example, the sex chromosomes of *P. vitticeps* are well-known to be heteromorphic based on cytogenetic evidence, implying the existence of sequence divergence between Z and W due to recombination suppression [17]. However, the PAR and SDR of both sex chromosomes remain undefined so far, which in turn hamper the search for the master sex-determining gene.

Motivated by the increasing demand of *P. vitticeps* in academic research and pet industry, we decided to upgrade the genome assembly of this model lizard, via a combination of long- and

short-read whole genome sequencing (WGS) as well as long-range sequencing technologies. The new *P. vitticeps* reference genome has a contig N50 of 202.5 Mb, with all contigs anchored onto 16 chromosomes and only few gaps remained. With this near complete chromosome-scale genome assembly, we fully characterized the Z sex chromosome and demarcated the PAR and SDR on Z, tracked the evolutionary origin and developmental expression of the Z-linked SDR genes, and proposed an alternative model for explaining the origin of sex chromosomes with the Z-linked *AMH* as the candidate of master sex-determining gene in *P. vitticeps*.

Results

The construction of a near-complete *P. vitticeps* genome

To reduce interference caused by allelic variation, all sequencing data for genome assembly were collected from a single captive-bred individual. The sex of this individual was verified as a ZZ male, as demonstrated by the anatomical presence of testes and the polymerase chain reaction (PCR) examination of sex-linked markers (Supplementary Fig. S1A). High molecular weight (HMW) DNA was extracted from the muscle and lung tissues, which was subjected to WGS with the CycloneSEQ [18] long-read and DNBSEQ short-read technologies, respectively (Supplementary Tables S1). In addition, we generated long-range sequencing data from the liver tissue by integrating chromatin conformation capture technique with CycloneSEQ (see Methods for details; hereafter referred as CycloneSEQ based Pore-C). *K*-mer analysis with the short-read data confirmed that *P. vitticeps* has a heterozygous diploid genome (heterozygosity ~1.63%) with a haploid size of ~1.7 Gb [14] (Supplementary Table S4; Supplementary Fig. S1B).

We obtained a total of ~265 Gb CycloneSEQ WGS reads, with over a quarter (~105 Gb) achieving a sequence length longer than 40 kb (Supplementary Table S1). As the 40 kb+ reads alone had already covered the *P. vitticeps* haploid genome for ~62 times, fully satisfying the requirements for long read-based *de novo* assembly, we therefore assembled the 40 kb+ CycloneSEQ WGS reads using NextDenovo [19] in the first step (Fig. 1A; Supplementary Fig. S1C). This resulted in a 1.81 Gb primary assembly with merely 106 contigs. The contig N50 was 60.9 Mb, surpassing the majority of squamate genomes published to date (Supplementary Table S7). Before further scaffolding by long-range data, we removed false duplications in the primary assembly with Purge_Haplotigs [20], and corrected sequence errors with short-read data using NextPolish [21]. After polishing, a 1.80 Gb assembly with 74 contigs were scaffolded by ~33X long-range

CycloneSEQ based Pore-C data with YaHS [22]. The order and orientation of each contig was also manually examined in Juicebox [23] to avoid misplacements. The final chromatin contact map clearly sorted all the contigs into 16 chromosomes, including six macrochromosomes and 10 microchromosomes (Fig. 1B).

To interrogate the integrity of chromosome ends, we next examined the presence of vertebrate telomeric repeat units (TRUs), i.e., $(TTAGGG)_n$ [24]. Only eight of the 32 expected telomeres were presented in the initial chromosome-scale *P. vitticeps* assembly, probably due to the well-known challenge of assembling the highly repetitive telomeric regions [25] which was also evidenced by the prevalent absence of telomeric sequences in the chromosomal ends of most squamate genomes published so far (Supplementary Table S9). However, we were able to find many CycloneSEQ reads containing TRUs before assembling, suggesting that the missing of telomeres are mainly due to computational drawback rather than sequencing omission. We thus developed an in-house pipeline to conduct local telomere assembly with the TRU-containing reads and assign the assembled telomeres to their corresponding chromosome ends (see Methods for details). As a result, 31 of the 32 expected telomeres with a mean copy number of 1661 TRUs were patched to corresponding chromosome ends, except one on chromosome 14 (Fig. 1B).

After telomere repair, the chromosome-scale assembly still contains 69 unclosed gaps. These gaps were subjected to gap-filling with the corrected long reads (generated by NextDenovo during assembly) using TGS-GapCloser [26]. The correctness of each closed gap was further examined by long-read coverage with an in-house pipeline. This led to the solid closure of 41 gaps inside the chromosomes, and made four of the six macrochromosomes (chromosomes 3, 4, 5 and 6) as well as one microchromosome (chromosome 13) achieving telomere-to-telomere (T2T) gapless assembly. The remaining 28 gaps were mainly located in microchromosomes and co-localized with repeat-dense regions (Fig. 1B). Of note, a local region (0.5 to 11 Mb) with a particularly high density of repeats was clearly observed in almost all chromosomes, assumably corresponding to the position of centromeres (Fig. 1B; Supplementary Table S8).

In summary, the final *P. vitticeps* genome assembly (hereafter referred as Pvit2024) was 1.79 Gb in length, with all contigs anchored into 16 chromosomes, a final contig N50 of 202.5 Mb, and the presence of all but one of the 32 telomeres. Although there were still 28 inner gaps remaining

unclosed, our Pvit2024 assembly outperformed all other squamate genomes reported so far in terms of continuity (Fig. 1C).

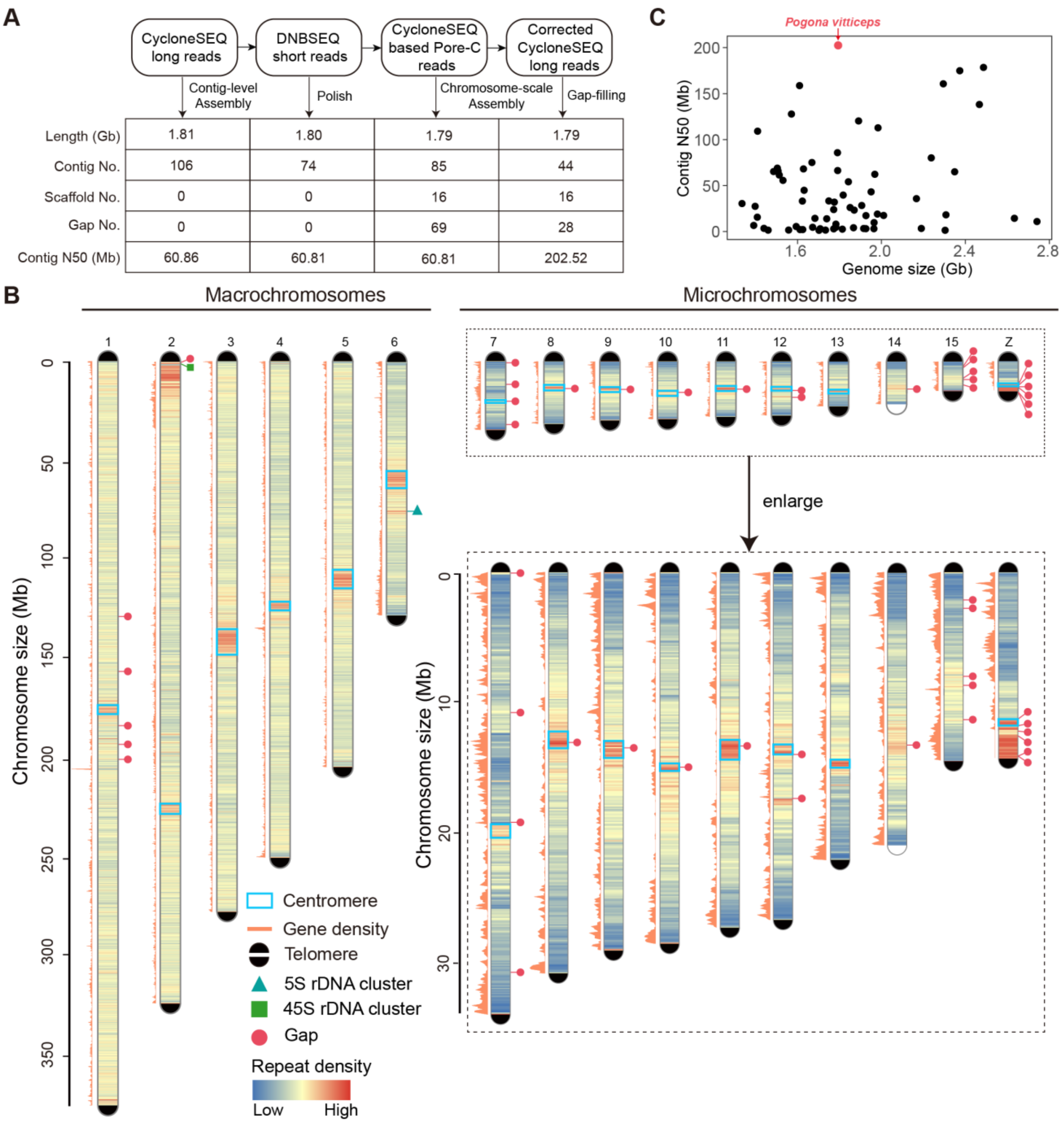

Length (Gb)	1.81	1.80	1.79	1.79
Contig No.	106	74	85	44
Scaffold No.	0	0	16	16
Gap No.	0	0	69	28
Contig N50 (Mb)	60.86	60.81	60.81	202.52

Figure 1: The near-complete genome assembly of *P. vitticeps*. (A) The CycloneSEQ long reads were initially utilized for *de novo* contig assembly. Thereafter, DNBSEQ short reads were employed to polish the assembled contigs. Subsequently, CycloneSEQ based Pore-C reads were implemented for scaffolding the contigs into chromosomal sequences. Finally, gaps were filled with corrected CycloneSEQ long reads. The table presents the

genomic features for each step. (B) An overview of the genome features. The distribution of all repeat density and gene density were calculated in 100 kb windows. All the gaps in Pvit2024 were show in read circles. All chromosomes, except for chromosomes 14 and 15, had been identified with centromeric regions marked by light blue boxes. (C) The comparison of genome size and contig N50 between Pvit2024 and 66 publicly available squamate genomes assembled by long-read data.

Quality validation of the genome assembly

To assess the completeness of Pvit2024, we first aligned three sets of WGS reads to the Pvit2024 assembly, that is, the CycloneSEQ long reads and DNBSEQ short reads collected in this study as well as a set of Illumina short reads generated from another ZZ male individual that were not used for our genome assembly. All the three read sets revealed a mapping rate greater than 98% (Supplementary Table S5), and the aligned reads showed uniform coverage across the genome with only a few exceptions in repeat-dense regions (Fig. 2A). Secondly, we conducted Benchmarking Universal Single-Copy Orthologs (BUSCO) [27] and Compleasm [28] assessments for Pvit2024 with 7,480 Sauropsida conserved genes. The BUSCO and Compleasm complete score were 97.6% and 98.63%, respectively, comparable to or even higher than other squamate genomes assembled by long-read data (Supplementary Fig. S2A; Supplementary Table S7). The consensus quality value (QV) of Pvit2024 as estimated by short-read data was 36.4, corresponding to a single-base error rate of merely 0.0229%.

Next, we assessed the accuracy of chromosomal sorting and scaffolding by different strategies. From a technical perspective, two traditional Hi-C libraries were constructed and sequenced with the DNBSEQ technology to serve as independent validations of the long-range information provided by CycloneSEQ based Pore-C. Both these two additional Hi-C contact maps consistently supported the scaffolding result of Pvit2024 (Fig. 2B). Besides, we aligned those chromosome-anchored sequences from Deakin et al. [6] to Pvit2024 and observed good agreements (i.e., sequences were mapped to expected position in expected order; Fig. 2C). Furthermore, Deakin et al. have also located the genomic regions harboring the centromeres in chromosomes 1, 2, 4 and 6 [6]. All these four centromere-containing regions showed perfect overlaps with the putative centromeric regions identified in Pvit2024 (Fig. 2C), supporting the feasibility of centromere positioning based on local repeat abundance in this species. From a biological perspective, the concurrent presence of macrochromosomes and microchromosomes is the hallmark of many vertebrate genomes [29]. According to the lengths of the assembled chromosomes, we could

clearly define six of the sixteen Pvit2024 chromosomes as macrochromosomes and the remaining 10 as microchromosomes, in line with the reported karyotype of a male *P. vitticeps* (Supplementary Fig. S2C) [30]. In addition to chromosomal lengths, we also observed other recognized features that separate microchromosomes from macrochromosomes, including higher gene density, higher GC content, lower repeat content, and more frequent inter-chromosomal interaction (Fig. 2D, E; Supplementary Fig. S2B) [31, 32]. These multiple lines of evidence together highlight the reliability of the Pvit2024 chromosomal assembly.

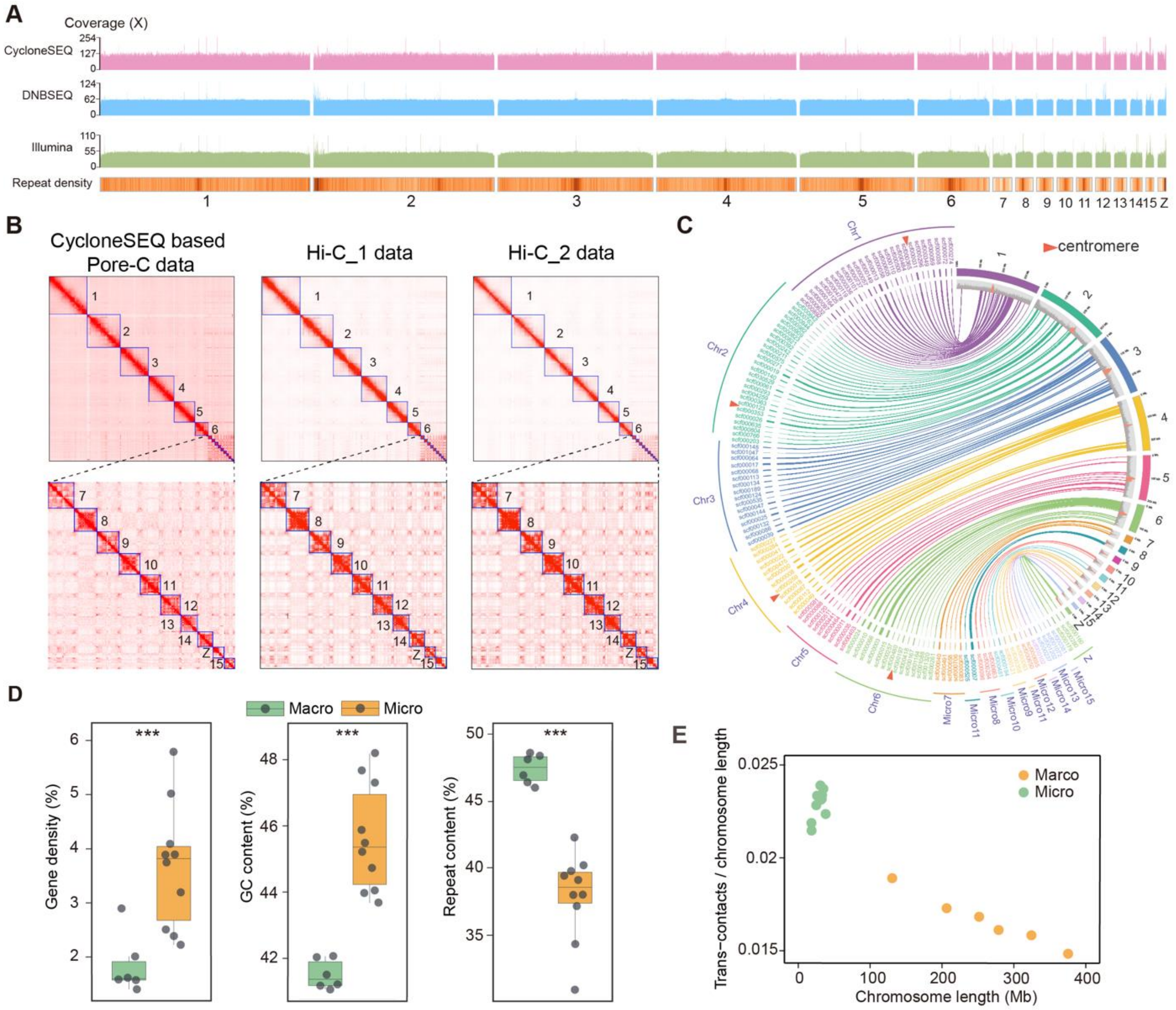

Figure 2: Quality validation of the genome assembly. (A**)** Genome-wide coverage based on various data was computed over 10 kb windows. Numbers on the y axis of each coverage track indicate the double average and average values of sequencing coverage for each track. Genomic data types are color coded. Pink: CycloneSEQ long-read WGS data. Light blue: DNBSEQ short-read WGS data. Light green: Illumina short-read WGS data.

Additional tracks in the bottom panel show repeat density of Pvit2024. (B) The heatmap for one CycloneSEQ based Pore-C library and two traditional Hi-C libraries chromosomes contact matrices. (C) The circos plot illustrates genomic synteny, with Pvit2024 chromosomes on the right and scaffolds anchored to chromosomes by BACs from Deakin et al. The second right circle shows repeat density, red indicating centromeres. The red triangle marks the centromere position as determined by Deakin et al. (D) The comparison of gene density, GC content, and repeat content between macrochromosomes and microchromosomes. Asterisks indicate the significance of differences: *** represents $p < 0.001$. (E) Trans-contacts scaled by chromosome size for each chromosome were calculated by ~33X CycloneSEQ based Pore-C data. Each dot represents one chromosome (yellow for macrochromosomes, green for microchromosomes).

The new reference genome recovers ~120 Mb missing sequences with numerous genes and regulatory elements

Compared with the previous reference genome (GCF_900067755.1/ pvi1.1) [14] (hereafter referred as Pvit2015), the contiguity of Pvit2024 was increased by over 5700 folds in terms of contig N50 (202.5 Mb vs 35.5 kb), and the number of scaffolds was remarkably reduced from 13,749 to 16 (Fig. 3A). Additionally, the genomic completeness as assessed by read alignments and BUSCO and Compleasm analyses all showed improvements (Fig. 3B, C).

To further uncover the differences between the two genome assemblies, we conducted reciprocal whole-genome alignment to identify missing sequences in one assembly relative to the other. Briefly, we defined genomic sequences in one assembly with no alignment to the other one by Winnowmap2 [33] and Minimap2 [34] as missing sequences in the former one. In this way, 124 Mb of the Pvit2024 sequences were detected as missing in Pvit2015, accounting for 6.9% of the Pvit2024 assembly size and affecting all the 16 chromosomes in a different degree (Fig. 3D, E; Supplementary Fig. S3A). Read coverages of these Pvit2015-missing regions were uniform and close to that of genome average, indicating that they are not assembly errors (Fig. 3F; Supplementary Fig. S3H). Conversely, only 36 Mb of Pvit2015 sequences were not aligned to Pvit2024, and the read coverage of these regions were quite low, suggesting that these Pvit2015-speficic sequences likely resulted from assembly errors (Fig. 3F).

We next focused on the annotation of the 124 Mb sequences that were missing in prior assembly, especially for the possibility of carrying functional genes or regulatory elements. The missing sequences revealed higher GC content than genome average, and most missing sequences (~73.6%) was annotated as repetitive elements in Pvit2024, reminding the advantage of the long-read

sequencing to sequence through high GC content regions and repeat regions (Fig. 3G, H; Supplementary Fig. S3B) [35]. Nevertheless, it is noteworthy that these Pvit2015-missing sequences also harbored the intact copies of up to 576 protein-coding and 312 lncRNA genes, namely, they are completely absent in prior assembly (Supplementary Fig. S3C, D). A representative example was the *Vmn2r65* (Vomeronasal 2 Receptor) gene family, of which 25 copies were located on a tandem array in chromosome 2 of Pvit2024 (Fig. 3I); only seven copies of *Vmn2r65* were identified and distributed on 6 distinct scaffolds in Pvit2015. In addition, the missing sequences in Pvit2015 also led to partial absence of one or more exons for up to 1,319 protein-coding and 1,651 lncRNA genes (Supplementary Fig. S3C, D), which may affect the accuracy of expression quantification by RNA sequencing. In terms of regulatory elements, we first examined the completeness of gene promoters, the region upstream of genes where the RNA polymerase binds to initiate transcription [36] and found that 2,504 (11.5%) protein-coding and 1,281 (8.1%) lncRNA genes contain >500 bp missing sequences in their putative promoter regions, respectively. We then examined the CpG islands, which are widespread in vertebrate genomes and play important roles in transcriptional regulation [37]. Consistent with the difficulty of short-read technology to sequence through high GC regions, we observed that 32,864 of the annotated 216,126 (15.2%) CpG islands were completely missing and 7,361 (3.4%) were partial missing in prior Pvit2015 assembly (Supplementary Fig. S3E, F, G).

Taken together, our genome-wide comparative analysis indicated a notable proportion (6.9%) of genomic sequences missing in the prior *P. vitticeps* reference genome. These missing sequences tend to be GC-rich and repeat-rich, and more importantly, they contain numerous genes and regulatory regions that are not accessible based on prior reference genome.

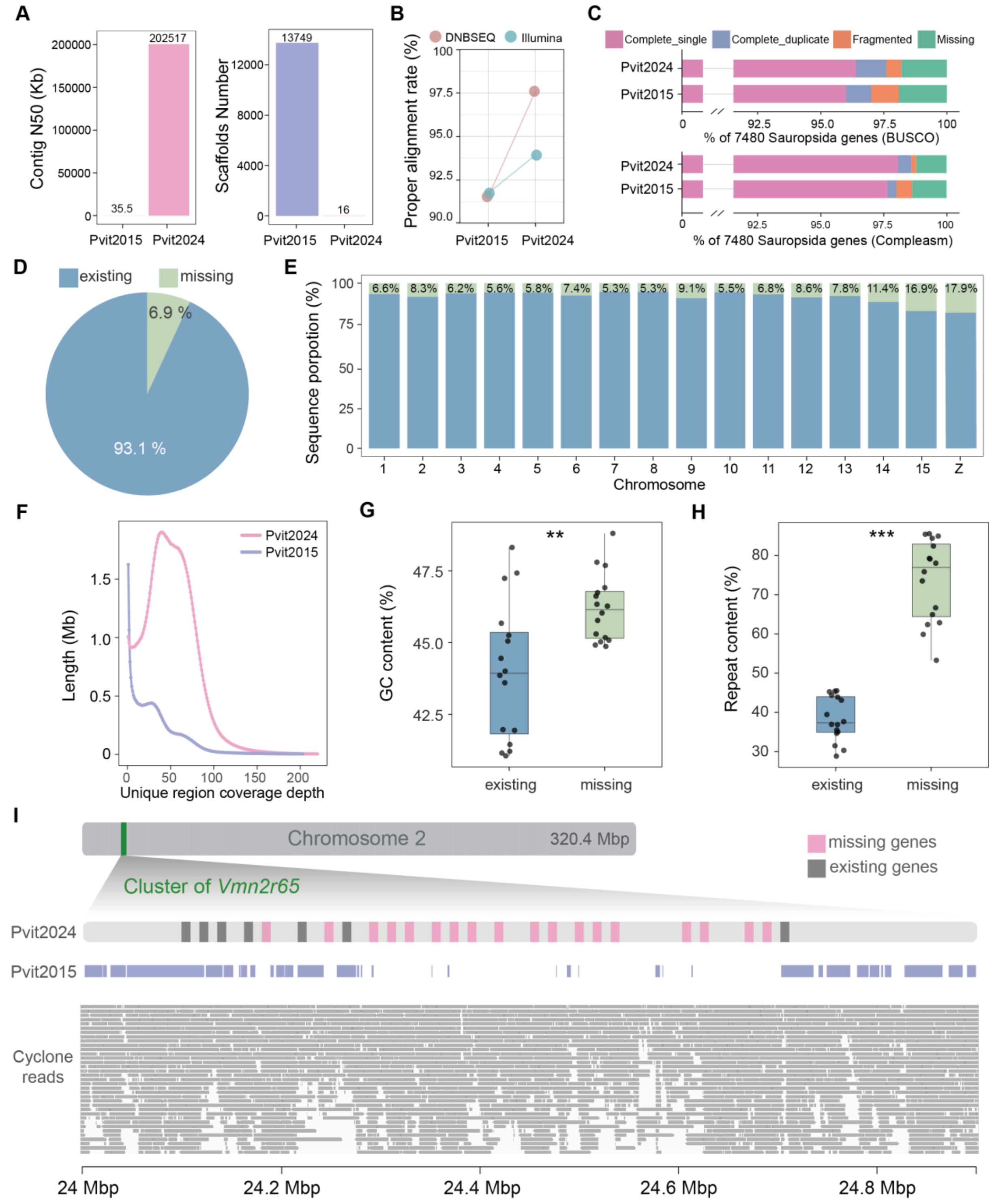

Figure 3: Comparison between Pvit2015 and Pvit2024. (A) The comparison of contig N50 and scaffold number. (B) The proper alignment rate of DNBSEQ and Illumina WGS data. (C) Gene completeness assessment of Pvit2015 and Pvit2024 based on BUSCO and Compleasm analyses. (D) Proportion of sequences existing and

missing in the Pvit2015 assembly compared to the Pvit2024. (E) The missing rate of Pvit2015 assembly across chromosomes in Pvit2024 is depicted, with blue and green bars representing the existing and missing ratios of Pvit2015, respectively. (F) Base coverage depth distribution of unique sequences in Pvit2015 and Pvit2024, the DNBSEQ genomics data were used. (G and H) Comparison of GC content (G) and repeat element content (H) between existing and missing sequences in Pvit2015 across each chromosome. T-test analysis of variance; $^{*}P<0.05$, $^{**}P<0.01$, $^{***}P<0.001$. (I) Distribution of *Vmn2r65* gene family along the chromosome, determined using ChromoMap of R packages. The blue block show sequence of Pvit2015 that can be aligned back to Pvit2024. The grey strip of bottom shows the coverage of CycloneSEQ reads.

Improving genome annotation by long-read RNA sequencing

Besides genome assembly, comprehensive and accurate annotation of gene model is the necessity to realize the value of a reference genome [38]. To facilitate a comprehensive gene annotation for Pvit2024, we performed high-depth long-read RNA sequencing (RNA-seq) for eight different tissues (brain, eye, heart, kidney, liver, lung, muscle, and testis) with the CycloneSEQ technology (Supplementary Table S2). In the meanwhile, we also generated paired-end short-read RNA-seq data for these eight tissues with the DNBSEQ technology (Supplementary Table S3). All the long-read and short-read RNA-seq data were generated from the same individual as that used for genome sequencing in this study.

With the abundant RNA-seq data, we first conducted protein-coding gene annotation with a combination of transcriptomic, homologous and *ab initio* prediction evidence, and obtained a total of 21,783 non-redundant protein-coding gene models (Supplementary Table S11). Up to 92% (20,002) of the protein-coding loci are supported by RNA-seq signal (TPM > 5) in at least one tissue. BUSCO assessment with the Sauropsida conserved genes revealed a complete score of 97.5%, consistent with that estimated for Pvit2024 genome assembly and slightly higher than the commonly used NCBI annotation (96.6%) and Ensembl annotation (91.7%) based on Pvit2015 (Fig. 4A).

Then, we leveraged the long-read RNA-seq data to refine isoform and UTR annotations for the protein-coding genes. After strict filtering steps to exclude artificial and low-quality sequences, we finally assigned 53,272 transcripts to 21,783 protein-coding loci, with a mean of 2.5 isoforms detected per gene. Up to 97% of the splicing junctions derived from the assigned transcripts were also supported by short-read alignments, further supporting the reliability of these transcript models. In addition, all the major types of alternative splicing events could be identified in all the

eight tissues, with exon skipping and alternative first exon usage being the most dominant events (Fig. 4B). By discriminating coding regions from non-coding parts, we could identify the UTRs for most transcripts, with 17,110 (31.1%) having a 5'-UTR longer than 30 bp and 19,161 (34.8%) having a 3'-UTR longer 50 bp. Of note, while the lengths of coding regions were comparable between our and the NCBI/Ensembl annotations, the 5'-UTRs and 3'-UTRs in our annotation were significantly longer (Fig. 4C), corroborating a more accurate definition of transcription boundaries owing to the assistance of long-read RNA-seq. To evaluate the effect of the new annotation on future RNA-seq studies, we calculated the read mapping rate and number of expressed genes with a bulk and a single-cell RNA-seq dataset that were not used for gene annotation in this study. Both datasets revealed a significant improvement on both metrics, especially for the single-cell dataset (Fig. 4D, E).

Long non-coding RNAs (lncRNAs) are another important class of RNA molecules that engage in numerous biological processes [39], yet the lncRNA repertoire is understudied in squamate reptiles. By identifying transcripts that are longer than 200 nt and lack coding potential, we annotated 13,269 high-confidence lncRNAs in Pvit2024 (Fig. 4F). When compared with the protein-coding genes, the *P. vitticeps* lncRNAs were generally shorter in length, have fewer exons, and were expressed at lower levels (Fig. 4G, H, I), consistent with previous observations in other organisms [40]. Interestingly, we found that many lncRNAs were expressed specifically in one or a few tissues, especially the testis (Supplementary Fig. S4). The large amount of tissue-specific lncRNAs in uncovered in *P. vitticeps* implied their potential importance in squamate biology that worth further attention.

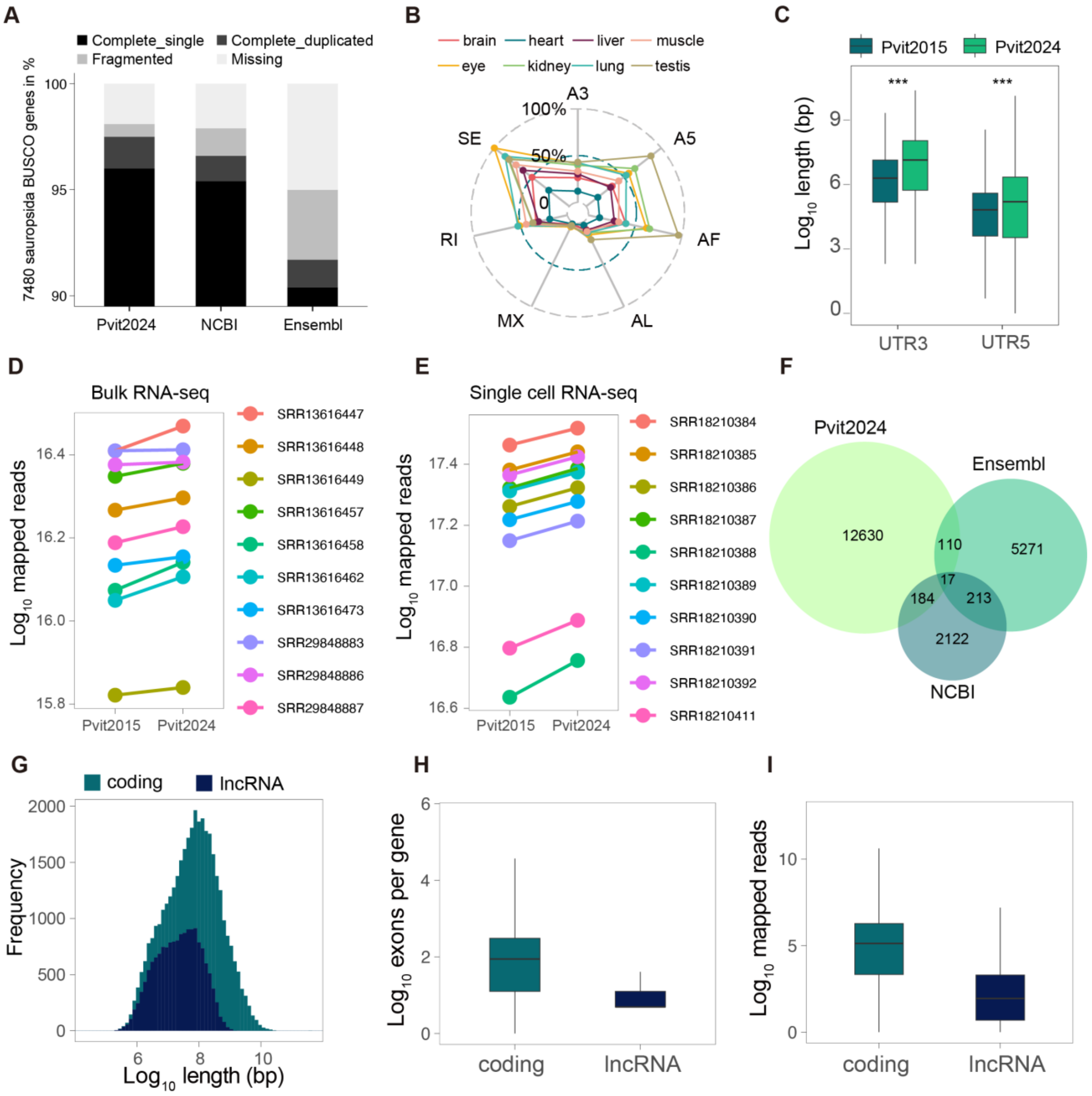

Figure 4: Enhancement of the genome annotation for *P. vitticeps*. (A) Completeness of Sauropsida BUSCO genes in annotations produced by Pvit2024, NCBI (Pvit2015), and Ensembl (Pvit2015). (B) Radar chart showing relative splicing abundance of eight tissues for each event type (SE–skipping exon; RI–retained intron; MX-mutually exclusive exons; A5–alternative 5' splice site; A3–alternative 3' splice site; AF-alternative first exons; AL- alternative last exons). Normalization here employs the maximum abundance value of splicing events as the denominator. (C) UTRs length distributions between the Pvit2024 and NCBI, Pvit2024 has longer than NCBI. T-test analysis of variance; *P<0.05, **P<0.01, ***P<0.001. (D and E) Connected dot-line showing the variance of mapping reads in Pvit2024 and NCBI RefSeq annotations for bulk (D) and single-cell (E)

transcriptomes. (F) Venn diagram showing lncRNA counts for Pvit2024, Ensembl and NCBI RefSeq annotations. (G) Histogram shows the distribution of gene length (exon regions) for coding gene and lncRNA. (H) Distribution of the exon counts per gene in coding gene and lncRNA. (I) Distribution of the read counts for transcriptomes in coding genes and lncRNAs.

The demarcation of PAR and SDR along the *P. vitticeps* Z sex chromosome

The identity of *P. vitticeps* Z chromosome was ascertained by mapping known Z-linked scaffolds identified by Deakin et al. [6] to our Pvit2024 assembly. Specifically, all the Z-linked scaffolds were aligned to a ~14.3 Mb microchromosome in expected order, spanning a continuous region of ~8.2 Mb on the first half of this microchromosome (Fig. 5A; Supplementary Fig. S5A). The uncovered portion comprised ~6.1 Mb genomic sequences that were newly identified to be Z-linked, accounting for ~43% of the whole Z chromosome (Fig. 5A). These newly identified Z-linked sequences harbored 47 protein-coding genes and 65 lncRNA genes that are either missing or unknown to be Z-linked in prior Pvit2015 assembly (Supplementary Table S12). The overall Z chromosome showed an uneven distribution of genomic elements, with an increase of repeat content accompanied by depletion of protein-coding genes toward the chromosomal end that enriched newly anchored sequences (Fig. 5B).

We next attempted to demarcate the SDR, where Z-W sequence divergence is accumulated, and the PAR, where Z and W remains identical, by comparing WGS read coverage between ZW and ZZ individuals. In principle, a PAR is expected to display comparable read coverage in both genders, while a SDR would exhibit halved coverage in ZW females relative to ZZ males [41]. Guided by this basic idea, we analyzed the WGS data collected from two ZW females and four ZZ males. Up to 83% (~11.9 Mb) of the Z chromosome displayed a comparable read coverage in both genders, suggesting that the vast majority of Z remains as PAR that persists recombination. Nevertheless, there was a ~250 kb region exhibiting roughly halved coverage in ZW females relative to ZZ males (Fig. 5C), fulfilling the expectation of an evolutionarily old stratum (hereafter referred as S0) where Z and W have been substantially diverged. For the region ranging from the downstream of S0 to the chromosome end, the read coverage in ZW females was even higher than that in ZZ males (Fig. 5C). However, this region lacked protein-coding genes and instead was occupied exclusively by repetitive elements (Fig. 5B). We speculated that this unusual coverage pattern was attributed to the much higher abundance of repeats accumulated in the W counterpart of this region, as previous cytogenetic studies has suggested that the short arm of W is much longer

than Z [42]. If so, this downstream region of S0 (~2.18 Mb in length) might also represent a stratum that was fully degenerated due to transposon invasion, and therefore, we tentatively grouped it into SDR in this study.

Together, we could conservatively define a large, continuous PAR and a small SDR on the *P. vitticeps* Z chromosome, with the PAR-SDR boundary delimited at ~12 Mb in coordinate (Fig. 5A). Compared with PAR, SDR was apparently characterized by a remarkably high repeat content (>80%) (Fig. 5D). However, the repetitive element composition of SDR was generally similar to those of PAR and other chromosomes, although several classes of transposons such as LTR/Gypsy, LTR/ERV1 and LINE/L1 were relatively more abundant in SDR (Fig. 5E).

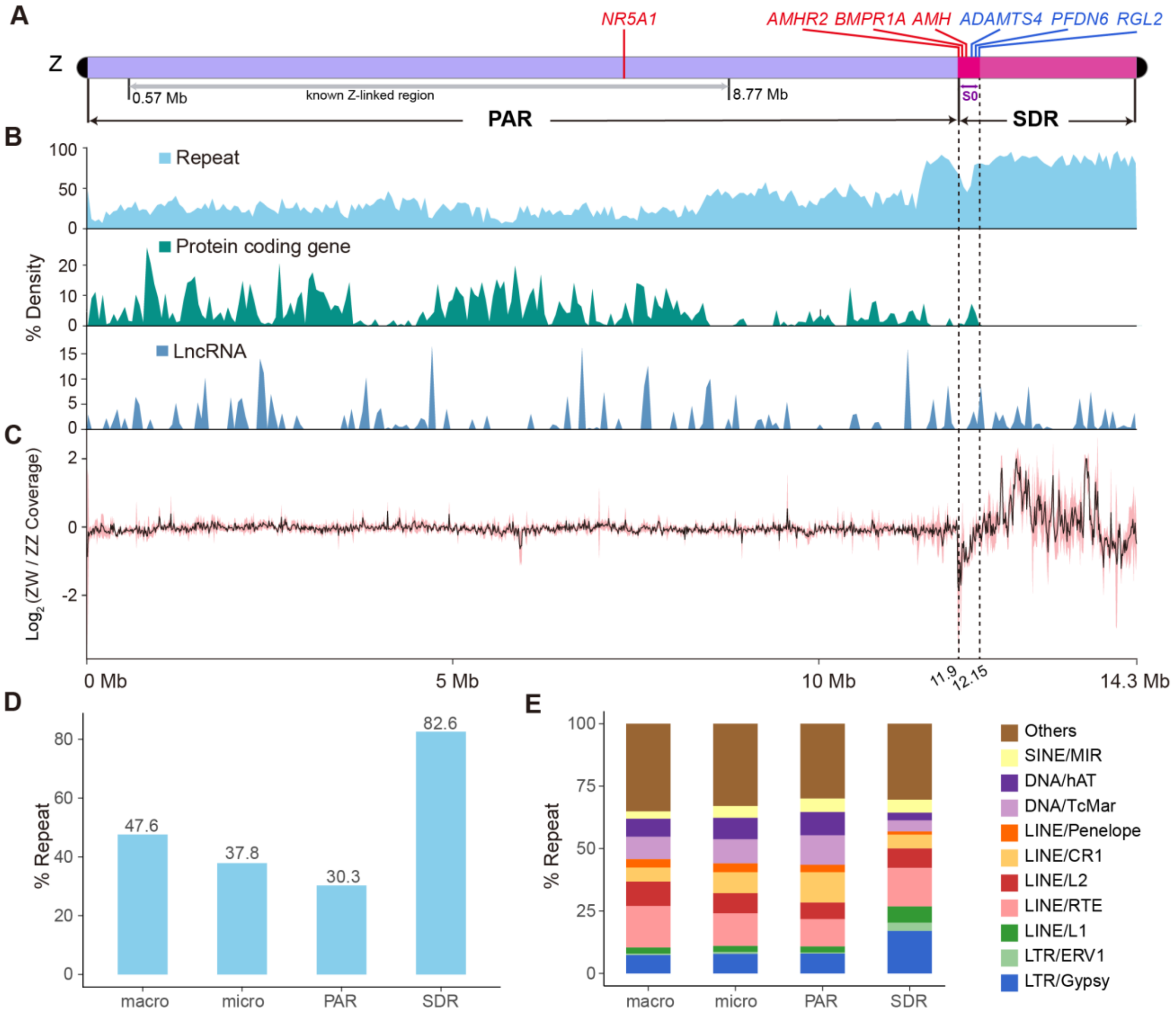

Figure 5: Z sex chromosome identification and the composition of PAR and SDR. (A) The Z chromosome can be divided into two regions: PAR and SDR. Sex-determination genes are marked in red, while pseudogenes are in bule. (B) Repeat density, protein coding gene density, and lncRNA density are calculated in 50 kb windows. (C) The distribution shows the ratio of ZW to ZZ read coverage in 10 kb windows. The depth (black line) is calculated as the average ratio of read coverage for ZW females to ZZ males between each pair of ZW females and ZZ males, based on the WGS data from two ZW females and four ZZ males. The pink areas are the confidence intervals for each window. (D) Repeat content for macrochromosomes, microchromosomes, PAR region, and SDR region. (E) Relative proportions for ten most abundant transposons families and other elements.

The evolutionary origin and developmental expression of the Z-linked SDR genes

Although being predominantly occupied by repetitive elements, we found that the *P. vitticeps* SDR carried three intact protein-coding genes (*AMHR2*, *BMPR1A* and *AMH*) and three pseudogenes (*ADAMTS4*, *PFDN6* and *RGL2*), of which all located in a ~150 kb region within S0 (Fig. 5A). Notably, the three intact genes are all well-known players in driving sexual differentiation, namely, *AMH* (anti-Mullerian hormone) [43] and its two receptors, *AMHR2* (anti-Mullerian hormone receptor type 2) [44] and *BMPR1A* (bone morphogenetic protein receptor type 1A) [45]. It is also noteworthy that each of these six SDR genes had a counterpart (i.e., paralog) on autosomes, while all other investigated lizards encoded only one copy of each in their genomes (Fig. 6A), indicating that lineage-specific gene duplication events occurred in *P. vitticeps* (and probably in other closely related agamid lizards as well). This was also supported by gene phylogenetic analyses, which preferentially clustered the *P. vitticeps* paralogs together among the proteins from multiple species (Fig. 6B, Supplementary Fig. S6A). In contrast, while the PAR maintained up to 334 protein-coding genes, only 21 of them (6.3%) had paralogs in autosomes. Hereafter, we designated the six SDR genes as *AMHR2-Z*, *BMPR1A-Z*, *AMH-Z*, *ADAMTS4-Z*, *PFDN6-Z* and *RGL2-Z*, and their autosomal counterparts as *AMHR2-A*, *BMPR1A-A*, *AMH-A*, *ADAMTS4-A*, *PFDN6-A* and *RGL2-A*, respectively (Fig. 6C).

We next asked, for these six paralog pairs in *P. vitticeps*, whether the SDR or the autosomal copy was the original copy that gave rise to the other one. By examining gene synteny information across species, we found that none of the SDR copies could be deemed to be original, namely, they all arose from the duplication of their autosomal counterparts (Fig. 6D, Supplementary Fig. S6B). But it is noted that *PFDN6* and *RGL2* were probably duplicated as a whole, because their gene order as well as the transcriptional direction in SDR maintained the same as their original copies

in chromosome 2 (Fig. 6C; Supplementary Fig. S6B). We then estimated the synonymous nucleotide substitution rate (Ks) between the paralog pairs to date the order of their translocation into SDR. As expected, the pseudogenes displayed the largest Ks, probably due to relaxed selection. But it is interesting that, among the three intact genes in SDR, *AMH* displayed the largest Ks, suggesting that *AMH* is likely among the earliest ones to be integrated into the SDR (Fig. 6D).

The master sex-determining gene that initiates sex differentiation remains unknown in *P. vitticeps*, yet it is expected to be a gene that locates in SDR and reveals differential expression between ZZ male and ZW female at an early stage of gonad differentiation. Therefore, we examined the expression dynamics of the SDR genes along gonadal development with the RNA-seq data from Wagner et al. [46]. This dataset comprised gonadal transcriptomes of both genders collected at three embryonic developmental stages (stage 6/7, stage 12 and 16). In *P. vitticeps*, stage 6/7 represents the earliest stage at which a consolidated gonad is recognizable and begins to differentiate, stage 12 represents an early stage of differentiation, while the gonads at stage 16 have been fully differentiated [13, 46]. Of note, all the three intact SDR genes (*AMH-Z*, *AMHR2-Z* and *BMPR1A-Z*) were identified as differentially expressed genes (DEGs) between genders at one or more developmental stages (DESeq2 FDR < 0.05; Fig. 6F), while none of the pseudogenes and lncRNAs in SDR were significant DEGs (Supplementary Fig. S6C). In addition, we found that *AMH-Z* (FDR = 0.00005; FC = 5.54) and its potential receptor *AMHR2-Z* (FDR = 0.05; FC = 2.22) manifested a significant male-biased expression pattern at stage 6/7, the earliest developmental stage examined. Moreover, *AMH-Z* maintained and even amplified the differential expression pattern throughout gonadal differentiation, reinforcing its central role in driving male development in vertebrates. Together with its early integration into SDR, *AHM-Z* could be considered as a strong candidate of the master sex-determining gene in *P. vitticeps*.

Meanwhile, we also examined the expression of the autosomal paralogs of the SDR genes, to explore whether the expression pattern has been diverged after gene duplication. Interestingly, *AMH-A* displayed almost the same expression pattern as *AMH-Z*, maintaining a high expression level in males while repressing its expression in females throughout embryonic gonadal development (Fig. 6F). However, the expression dynamics of *AMHR2-A* and *BMPR1A-A* were apparently diverged from that of their SDR counterparts. Specifically, while *AMHR2-Z* displayed transient male-biased expression at stage 6/7, *AMHR2-A* was not differentially expressed at stage 6/7 but turned to be significantly female-biased at stages 12 and 16 due to its upregulation in ZW

females. In terms of *BMPR1A*, the expression of *BMPR1A-Z* was a bit male-biased at stage 6/7, but the following sharp decrease of *BMPR1A-Z* expression in ZZ males made it turn to be significantly female-biased at later stages; however, *BMPR1A-A* did not show significant sex-biased expression throughout development, although a slight trend of male-biased expression was observed (Fig. 6F).

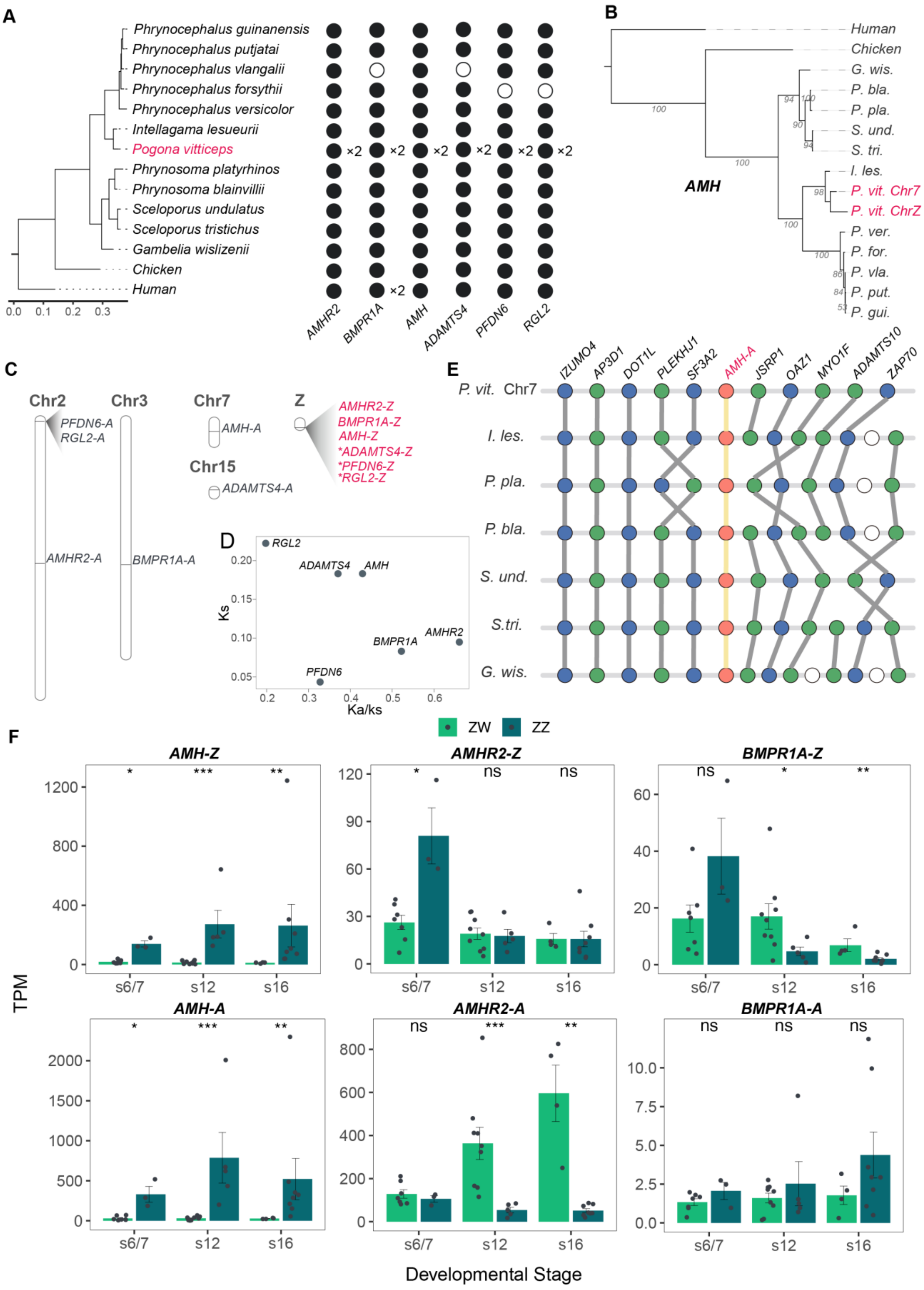
A
Phrynocephalus guinanensis
Phrynocephalus putjatai
Phrynocephalus vlangalii
Phrynocephalus forsythii
Phrynocephalus versicolor
Intellagama lesueurii
Pogona vitticeps
Phrynosoma platyrhinos
Phrynosoma blainvillii
Sceloporus undulatus
Sceloporus tristichus
Gambelia wislizenii
Chicken
Human
AMHR2 BMPR1A AMH ADAMTS4 PFDN6 RGL2
B
AMH
C
Chr2 Chr3 Chr7 Z Chr15
D
Ks
Ka/ks
E
F
ZW ZZ
AMH-Z AMHR2-Z BMPR1A-Z AMH-A AMHR2-A BMPR1A-A
TPM
Developmental Stage

Figure 6: Z-linked SDR genes. (A) Copy number of SDR genes in cross-species. A dot (black) indicated a copy, digital followed by an "×" indicated copy number, and circles indicated absence. (B) Phylogenetic relationships of *AMH* genes across different species. The pink font showing the two copies of *AMH* in *P. vitticeps*. (C) Ideogram showing the gene model of SDR genes and their autosomal counterparts in the Pvit2024 assembly. Pseudogenes were indicated with an asterisk. "-A" indicated autosomal genes, while "-Z" indicated sex-linked genes. (D) Ka/Ks analysis of SDR genes showing order of gene occurrence in Z-linked SDR. (E) Synteny relationships of *AMH-A* and its flanking 5 genes in closely related species. *AMH* is highlighted in orange, and surrounding genes are shown in grey. (F) Differential expression of SDR genes between ZZ and ZW during developmental stages. Benjamini-Hochberg analysis of variance: ns (not significant), *P<0.05, **P<0.01, ***P<0.001.

Discussion

The first chromosome-scale reference genome for Amphibolurinae (Squamata: Agamidae)

Squamate reptiles are a species-rich clade of amniote vertebrates that have adapted to most terrestrial ecosystems [47]. With over 12,000 extant species that make up a significant part of the vertebrate tree of life, reference genomes for squamates are particularly scarce when compared with other amniote clades such as mammals and birds [48]. Specifically, according to the latest data deposited in NCBI (accessed in July 2024), only 104 squamate species have a genome assembly with contig N50 over 30 kb, in sharp contrast with ~630 for birds and ~730 for mammals. This unevenness has drawn the concern of the biodiversity genomics community who call for more attention for this neglected vertebrate group in the genomic era [15, 16]. Thanks to technological advances in long-read sequencing, constructing a near-complete reference genome has become feasible for many organisms, especially for those able to supply sufficient HMW DNA from a single individual, such as the reptiles as showcased in this study. By leveraging a newly released long-read sequencing technique called CycloneSEQ, we upgraded the genome assembly of the model lizard *P. vitticeps* to one of the most continuous squamate genomes so far. This new *P. vitticeps* genome represents the first chromosome-scale reference genome for the Amphibolurinae subfamily of the agamid lizards (Agamidae), highlighting its potential value in evolutionary genomics studies. With this near-complete *P. vitticeps* reference genome, we also provided novel insights into the sex chromosome origin and sex determination of this species.

AMH signaling as an upstream driver of sexual differentiation in *P. vitticeps*

P. vitticeps possesses a ZZ male/ZW female genetic sex determination (GSD) system. However, the default sexual fate of ZZ embryos can be overwritten by high incubation temperature, leading to sex reversal of ZZ males to ZZ females [49]. The capacity of a ZZ embryo to develop as a normal female without the help of W chromosome suggests that sexual fate is most likely determined by a dosage-sensitive gene on the Z chromosome [12]. Therefore, the achievement of a complete Z chromosome sequence represents a particularly important beginning for disclosing the sex-determining cascade in *P. vitticeps*. Although there are several unclosed gaps remained in repeat-rich regions, our Pvit2024 Z chromosomal assembly is approaching T2T level as indicated by the presence of telomeric sequences on both chromosomal ends. Additionally, the Pvit2024 Z has almost doubled the known Z-linked sequences, from 8.3 Mb to 14.3 Mb. More importantly, the newly anchored Z-linked sequences carry the whole SDR, a region that hopefully harbors the master sex-determining gene.

The Z/W-resided *nr5a1*, which encodes the steroidogenic factor 1 (SF1) required for male development, was once regarded as the most promising sex-determining candidate in *P. vitticeps* [50]. However, the location of *nr5a1* in PAR as uncovered in our study has almost ruled out it as the master sex-determining gene. Instead, the aggregation of *AMH* signaling related genes (i.e., *AHM*, *AHMR2* and *BMPR1A*) in the SDR is of particular interest. *AMH*, which encodes a hormone of the transforming growth factor-β (TGF-β) superfamily, is critical for testis development and has been identified as a master sex-determining gene in a growing number of vertebrates, including several lineages of teleost fish [51] and monotreme mammals [52]. In *P. vitticeps*, we found that the SDR-resided *AMH* (*AHM-Z*) was highly expressed in all examined ZZ embryos and maintained over 5-fold expression difference between ZZ and ZW embryos since the beginning of gonadal differentiation (stage 6/7). This prominent male-biased expression pattern suggests *AMH-Z* as a strong sex-determining candidate, which deserves further functional experimental validation in future studies.

AMH signals through binding to its receptors, such as the type II receptor encoded by *AMHR2* and the type I receptor encoded by *BMPR1A* [45]. Therefore, our finding of transient up-regulation of the SDR-resided *AMHR2* and *BMPR1A* in ZZ embryos at stage 6/7 is particularly notable. We hypothesize that such a transient up-regulation of the *AHM* receptors may serve as an amplifier of the *AHM* signaling cascade at the bipotential gonads, which is essential for tipping the sex differentiation network towards male development at this critical time window. Another notable

finding is the up-regulation of the autosome-resided *AMH* (*AMH-A*) since stage 6/7. Given the high protein similarity of *AHM-Z* and *AMH-A* that implies their functional conservation, this parallel up-regulation of *AMH-A* may serve as another booster of AMH signaling during early sex differentiation. Collectively, all this evidence points to one conclusion, that is, the AMH signaling is essential and likely serve as an upstream signal driving *P. vitticeps* sex differentiation. Such pattern of duplicated gene functioning in upstream sex determination have previously been identified mainly in fish [53]. And interestingly, all these duplications, including our current finding in the *Pogona* lizard, seems to direct gonad development into testis.

However, it is also notable that the gonadal transcriptome profiles of ZZ and ZW embryos has been diverged at stage 6/7 [46]. This suggests that the initial elevation of AMH signaling likely occurs even earlier, before stage 6/7. Interestingly, in teleost fish Patagonian pejerrey (*Odontesthes hatcheri*) a convergent gene duplication event was found as *AMH* was duplicated onto the Y chromosome and may also serve as the candidate sex determinant gene, indicative by an earlier expression than the autosomal *AMH* [54], Future studies are recommended to focus on when and where (e.g., what cell types) the AMH signaling is initially diverged between ZZ and ZW embryos.

A proposed model for the origin and evolution of *P. vitticeps* sex chromosomes

The origin of the *P. vitticeps* ZW microchromosomes is presumably associated with the fusion of a fragment derived from chromosome 2, as both the Z and W chromosomes share homology with the terminal region of the long arm of chromosome 2 (chr2qter) as revealed by physical mapping of a BAC clone (Pv151P16) [55]. Our result also supports this homology according to the finding of an additional copy of the chr2qter-resided *PFDN6-RGL2* syntenic block in SDR (Fig. 6C). However, we also found that the homology with chr2qter is limited to the terminal region of the *P. vitticeps* Z chromosome which lacks intact protein-coding genes, thus arguing the role of the chr2qter fusion event in driving ZW formation. Indeed, the fusion of chr2qter to *P. vitticeps* ZW microchromosomes seems to occur early during Amphibolurinae evolution, probably before the differentiation of Z and W, because the concurrent mapping of Pv151P16 to chr2qter and a pair of microchromosomes are also observed in other Amphibolurinae species, including GSD species with homomorphic sex chromosomes and TSD species without sex chromosomes [55].

However, it is notable that the *Ks* of *AMH*-Z is higher than that of *PFDN6-Z* and almost comparable to that of *RGL2-Z*, despite that *PFDN6-Z* and *RGL2-Z* have become pseudogenes (Fig. 6C). This

suggests that the integration of *AMH* and the fusion of chr2qter to the proto-sex chromosomes might occur within a very narrow time window. We thus propose that the duplication and translocation of the autosomal *AMH* to the proto-sex chromosomes might represent the real milestone in triggering the formation of Z and W chromosomes in the ancestor of *P. vitticeps* and its sister taxa. After *AMH* integration, the subsequent aggregation of other male-biased genes including *AMHR2* and *BMPR1A* further consolidated the role of the proto-Z in sex determination and promoted ZW differentiation. We anticipate that chromosome-scale genome assemblies from other closely related dragon lizards will provide crucial evidence for testing this hypothesis.

Methods

Sample collection

This study was performed in accordance with the guideline of the national and organizational stipulation. An adult lizard *Pogona vitticeps* in captivity was collected at Zhejiang University, under the permit ZJU20240342. This lizard was euthanized after anesthetized by diethyl ether. All the tissue samples were frozen via liquid nitrogen and stored at -80°C refrigerator immediately after dissection. Muscle and lung tissues were selected for genome sequencing, which included CycloneSEQ long-read WGS sequencing, DNBSEQ short-read WGS sequencing, CycloneSEQ based Pore-C sequencing, and Hi-C sequencing. Furthermore, eight tissue samples, including liver, lung, eye, muscle, testis, kidney, brain, and heart, were subjected for transcriptome sequencing.

Genome and Transcriptome sequencing

The High-molecular-weight genomic DNA was extracted using the cetyl trimethylammonium bromide method (see "Genomic DNA CTAB extraction" for details) from muscle and lung samples. Eight tissue samples were used for RNA extraction with TRlzol reagent (Invitrogen, USA) following the manufacturer's guidelines. The isolated RNA was then fragmented into 200-400 bp, and then reverse-transcribed to cDNA for library preparation. Quantity and quality of the genome's DNA and RNA were assessed by pulsed field gel electrophoresis, Qubit 3.0 (Invitrogen, USA) and Qseq 400 (Bioptic, China). A total of 12 short-insert paired-end (PE) libraries (4 for genomic DNA and 8 for cDNA) were constructed and sequenced on DNBSEQ platform (MGI, Shenzhen), with the manufacturer's instructions. The long-read libraries were constructed as CycloneSEQ library and sequenced via CycloneSEQ WuTong02 platform (see "CycloneSEQ library preparation and

sequencing" for details). For the Hi-C library construction, the muscle sample was crosslinked with formaldehyde and 2 Hi-C libraries were constructed by using the dpnII restriction endonuclease. The libraries were sequenced on the DNBSEQ platform with 150-bp paired-end sequencing strategy.

Genomic DNA CTAB extraction

Preheat the CTAB lysate in a water bath. Collect 20-30 mg of fresh or frozen animal tissue, freeze in liquid nitrogen. Grind tissue using mortar and pestle in the presence of liquid nitrogen until finely ground. Transfer the ground animal tissue to 2 mL polypropylene centrifuge tubes. Add 1 mL of CTAB lysis buffer and 50 μL protease K (20 mg/mL). swirl and mix, incubate at 50 °C (with shaking) for 1 h. Cool the tube to 37 °C. Add 20 μL RNase A (10 mg/mL) for every 1 mL of lysis buffer, mix by inversion and incubate for 10 min. Add equal volume phenol-chloroform-isoamyl alcohol (25:24:1 ratio) with pH > 7.8, mix by inversion and spin at 5000 rpm for 10 minutes in a tabletop centrifuge at room temperature (RT). Transfer top aqueous solution to new 2 mL centrifuge tubes using wide-bore pipette tip. Add equal volume chloroform-isoamyl alcohol (24:1 ratio), mix by inversion and spin at 5000 rpm for 10 minutes in a tabletop centrifuge at RT. Transfer top aqueous solution to new 1.5 mL centrifuge tubes using wide-bore pipette tip. Add 2/3 volume of isoamyl alcohol and mix by inversion to form an emulsion, centrifuge at 5000 rpm for 2 minutes at 4°C. Discard the supernatant. Add 1mL 75% ethanol and mix slowly to resuspend the precipitation. Centrifuge at 5,000 ×g for 2 min at 4°C and discard the supernatant. Repeat the previous step. Discard the remaining supernatant, dry at RT for 3 min. Dissolve in 200-400 μL TE Buffer and incubate at 37 °C for 1 h, incubate at RT overnight. Store the extracted DNA at -80 °C.

CycloneSEQ library preparation and sequencing

The preparation and sequencing of CycloneSEQ library was proceeded following the manufacturer's guidelines. Each sample, comprising 2 μg of input DNA (≥23 ng/μL), was initially diluted with nuclease-free water to a total volume of 44 μL, followed by mixing with 6 μL DNA repair buffer 1, 3 μL DNA repair buffer 2, 3 μL DNA repair enzyme 1, and 4 μL DNA repair enzyme 2. The mixes were then incubated in a thermocycler through the following steps: 10 min at 20°C, 10 min at 65°C, and held at 4°C. After incubation, the mixes were purified using a 1.0x volume of DNA clean beads, and DNAs were eluted with 60 μL of nuclease-free water. Next, the purified end-repaired samples were mixed with 10 μL sequencing adaptors, 25 μL 4x ligation

buffer, 10 μL DNA ligase, and 2.5 μL nuclease-free water, before incubating at 25°C for 30 minutes to complete the adaptor ligation. The ligated products were again purified with a volume of 1.0x DNA clean beads and long fragment wash buffer was applied to gently resuspend the beads. After removing the supernatant, the libraries were recovered into 17 μL of elution buffer and quantified on a Qubit fluorometer. Each prepared library was sequenced on the CycloneSEQ WuTong02 platform according to the protocol.

CycloneSEQ based Pore-C sequencing

About 100 mg tissues were homogenized with pestle and washed with 2000 μL 1X PBS (Phosphate-Buffered Saline). After being spun down, the tissues were fixed by 10ml of 1% formaldehyde 1X PBS solution and incubated for 10 minutes at room temperature (RT). To quench formaldehyde crosslinking, 527 μL of 2.5 M glycine was added and incubated for 5 minutes at RT, followed by 10 minutes on ice. Fixed tissues were pelleted by centrifugation at 2000 ×g for 10 minutes at 4 °C. The pellet was resuspended in 550 μL of ice-cold lysis buffer (10 mM Tris-HCl pH 8.0, 10 mM NaCl, 0.2% Igepal-CA630, 50 μL of protease inhibitor cocktail) and incubated on ice for 15 minutes. Nuclei were pelleted at 2000 ×g for 10 minutes at 4 °C and the supernatant was discarded. The pellet was resuspended in 200 μL of the prepared chilled 1.5X digestion reaction buffer and was pelleted by centrifugation at 2000 ×g for 10 minutes at 4 °C. The pellet was resuspended in 300 μL of 1.5X digestion reaction buffer and added 33.5 μl 1% SDS, followed by incubate in a thermomixer at 300 rpm at 65°C for 10 minutes. The reaction was stopped by adding 37.5 μl of 10% (v/v) Triton X-100. Then, 45 μL of 10 U/μL NlaIII restriction enzyme (NEB, R0125L) and 34μL water were added, and the sample was rotated at 37 °C for 18 hours. NlaIII was then heat-inactivated at 65 °C for 20 minutes. A total of 550 μL of ligation master mix was added: 100 μL of 10× NEB T4 DNA ligase buffer with 10 mM ATP (NEB, B0202), 10 μL of 10 mg/mL BSA (Thermo Fisher, AM2616), 50 μL of 400 U/μL T4 DNA Ligase (NEB, M0202), and 390 μL of water. The reactions were rotated at RT for 5 hours. Then, the sample was added a master mix (100 μl of 10% SDS, 500 μL of 20% tween-20, 100 μl of 20 mg/ml proteinase K, 300 μL of water) and incubated at 56 °C for 18 hours. The sample was extracted with equal volume of phenol: chloroform: isoamyl alcohol (25:24:1) and precipitated with 0.7 X isopropanol. DNA was size selected (>1.5 kb) using DNA Clean beads and constructed to CycloneSEQ library.

Sex identification

Organ anatomical examination and sex-linked markers analysis were employed for sex identification in *P. vitticeps*. For the anatomical assessment, testes were dissected from the individual. Molecular analysis of sex-linked markers was conducted using a protocol adapted from Holleley et al. [11], Genotypic sexing was performed utilizing two PCR primers: H2, GCCCATATCTCACTAGTTCCCCTCC; F, CAGTTCCTTCTACCTGGGAGTGC, which was flanking two W-chromosome-specific deletions, measuring 150 base pairs and 14 base pairs, respectively. PCR was conducted using Platinum High-Fidelity ReadyMix(2x) (GCATbio), with a range of primer concentrations and genomic DNA quantities to establish experimental groups, and a no-genomic DNA control group. Cycling conditions were 95℃ for 5 min; (95℃ for 20 s, 70~65℃ for 20 s, 72℃ for 1 min) ×10 cycles with annealing temperature decreased 0.5℃ per cycle; (95℃ for 20 s, 65℃ for 20 s, 72℃ for 1 min) ×30 cycles; 72℃ for 10 min. The PCR products were resolved on a 1.5% agarose gel and visualized using GelStain Blue (Transgen). The presence of two bands indicated ZW individuals, while a single band confirmed ZZ individuals.

Genome survey

The DNBSEQ short-read WGS data was cleaned by SOAPnuke v1.5.6 (RRID: SCR_015025) [56] to exclude reads characterized by low quality and the presence of adapter sequences and poly-N regions with parameters -Q 2 -G -d -l 20 -q 0.2 -5 1 -t 5,0,5,0. To accurately assess genomic characteristics, including genome size and heterozygosity rate, all clean data were adopted *k*-mer based methods by using Jellyfish and GenomeScope tools. The haploid genome size was estimated according to *k*-mer analysis frequency distributions generated by Jellyfish v2.2.6 (RRID: SCR_005491) [57] using a series of *k* value (19, 21, 23, 25, 27) with the -C setting, which was calculated as the number of effective *k*-mers (ie. total *k*-mer – erroneous *k*-mer) divided by the homozygous peak depth. The rate of heterozygosity was estimated by GenomeScope v2.0.0 (RRID: SCR_017014) [58] with the *k*-mer frequency distributions generated by Jellyfish as inputs.

Genome assembly

The initial process was to retain CycloneSEQ long reads longer than 40 kb, the chimeric reads were removed using Yacrd v1.0.0 [59] with parameters -c 5 -n 0.6. Cleaned reads were then assembled into contigs using NextDenovo v2.5.0 (RRID:SCR_025033) [19] with the following parameters: read_cutoff = 35k, genome_size = 1.7g. In response to the high heterozygosity

detected in the initial assembly, DNBSEQ short reads were adopted to remove heterozygous contigs by purge_haplotigs v1.1.2 (RRID:SCR_017616) [20] with parameters -l 10 -m 53 -h 120. Next, two rounds of polishing were performed using NextPolish v1.4.1 (RRID:SCR_025232) [21] with recommended parameters, utilizing DNBSEQ short reads. Subsequently, CycloneSEQ based Pore-C reads were used as the main body to anchor the assembly to pseudo-chromosomes, while two additional libraries of Hi-C reads served as controls to assess the reliability of the anchoring. The specific process of anchoring was as follows: CycloneSEQ based Pore-C reads were first aligned to the contig-level genome using wf-pore-c v1.1.0 (https://github.com/epi2me-labs/wf-pore-c) with default parameters. The unaligned fragments were subsequently filtered out from the wf-pore-c output file 'null.ns.bam' using the command 'samtools view -F 4 -bh'. Adjacent fragment pairs were then extracted from the filtered BAM file into BED format with a custom script (https://github.com/guoqunfei/Pvit_T2T). In parallel, two additional libraries of Hi-C reads were aligned using Chromap v0.2.3-r407 [60] with default parameters. The alignment results were converted from the .sam format to a .bed file using bedtools v2.29.2 [61]. Both sets of BED files, derived from CycloneSEQ based Pore-C and Hi-C reads respectively, were utilized to assemble contigs into scaffolds using YaHS v1.2a.1 [22] with the parameters –no-contig-ec –no-scaffold-ec. Following the scaffolding process, we proceeded to generate a .hic file for further manual examination and curation using JuiceBox (JBAT) [23], adhering to the guidelines provided at https://github.com/c-zhou/yahs.

To avoid telomeric repeat sequences being incorrectly trimmed by the assembler, by using known vertebrate six base telomere repeats (‘TTAGGG’) as a sequence query, we developed an in-house pipeline to correct telomeric regions as follows: First, we extracted those reads from CycloneSEQ WGS raw reads containing at least 100 consecutive copies of the telomere units. Then, all of these reads were aligned to the assembly using minimap2 v2.23-r1116-dirty (RRID:SCR_018550) [34] with the parameter: -x map-ont. Subsequently, based on the alignment results, for each chromosome’s two ends, the read with the highest alignment quality and the largest number of copies of the telomere unit was selected and used to replace the corresponding end. (The specific implementation code can be seen at https://github.com/guoqunfei/Pvit_T2T).

After telomere repair, unclosed gaps in the chromosome-scale assembly were initially filled using TGS-GapCloser v1.2.1 (RRID:SCR_017633) [26] with the corrected long reads (generated by NextDenovo during assembly). The correctness of each closed gap was further examined by long-

read coverage with an in-house pipeline as follows: the 5 kb of upstream and downstream regions were extracted and aligned to the corrected long read to identify any alignment. These alignments were further visualized and manually examined using IGV. Only alignments with proper size and consistent orientation without conflicting alignments were used for the purpose of gap filling within the genomic assembly.

Finally, the assembled genome was evaluated using BUSCO v5.7.1 (RRID:SCR_015008) [27], Compleasm v0.2.6 [28], Merqury v1.3 (RRID:SCR_015811) [62] using default parameters. Short reads were aligned to the genome using BWA-MEM v0.7.17-r1198-dirty (RRID:SCR_012940) [63], and SAMtools v1.15.1 (RRID:SCR_002164) [64] were counted as properly paired. Long reads were mapped to the genome using minimap2 v2.23-r1116-dirty (RRID:SCR_015008) [34] with the parameter: -ax map-ont.

Reconstruction of NOR 45S rDNA cluster

According to the comprehensive cytogenetic map, *P. vitticeps* has an active nucleolus organizer region (NOR) rich in 45S rDNA cluster, comprising of 18S, 5.8S, and 28S rRNAs, at the sub-telomeric region of Chromosome 2 [42]. However, the sequencing depth of the NOR region, identified as the 45S rDNA-rich region near the telomere on Chr2 from the *de novo* assembly of whole genome, was significantly higher than that of adjacent regions. Additionally, the length of the NOR region was much shorter than those found in closely related species. These findings indicated that the NOR region was not fully assembled. To address this, we reconstructed the NOR 45S rDNA cluster. Firstly, the 45S rDNA sequence from NOR region was used to identify all CycloneSEQ reads containing the 45S rDNA sequence feature. Secondly, these reads were assembled using NextDenovo v2.5.0 [19] with the following parameters: read_cutoff = 1k, genome_size = 0.005g, minimap2_options_raw = -I 6G --step 2 --dual=yes -t 4 -x ava-ont -k 17 -w 17 --minlen 2000 –maxhan1 5000. Finally, we obtained a 282.2 kb NOR region rich in 45S rDNA cluster and replaced this segment with the genomic NOR position where the assembly had collapsed.

Repetitive element annotation

We annotated the lizard whole-genome repeat sequences based on *de novo* predictions and homology annotations. For *de novo*, the RepeatModeler v2.0.4 (RRID:SCR_015027) [65] was used to identification the custom repeats from the assembly sequence, with subsequent annotation

and masking using RepeatMasker v4.1.5 (RRID:SCR_015027) [66]. For homology annotations, the homologous repeat elements in the genome were identified and classified using RepeatMasker. The transposable element proteins were searched based on RepeatProteinMask database (http://www.repeatmasker.org) that part of RepeatMasker, and the tandem repeats were extracted using TRF v4.09 [67] via ab initio prediction.

Protein coding gene annotation

We integrated three types of evidence for predicting protein-coding genes: transcriptome data, homology evidence, and ab initio prediction. For the transcriptome evidence, we first cleaned the DNBSEQ RNA-seq data using SOAPnuke v1.5.6 (RRID:SCR_015978) [56] with the command: filter -n 0.03 -l 20 -q 0.3 -p 1 -Q 2 -G -5 1 -t 10,0,10,0 -E 70. Next, we aligned the cleaned short reads to the genome using HISAT2 v2.2.1 (RRID:SCR_015530) [68] with the –dta option. Finally, the transcripts were reconstructed using StringTie2 v2.2.3 (RRID:SCR_016323) [69] and coding sequences were predicted with TransDecoder v5.7.1 (RRID:SCR:015534) (https://github.com/TransDecoder/TransDecoder). For the ab initio prediction, we randomly selected 700 high-quality genes from the transcriptome predictions for training and used AUGUSTUS v3.4.0 (RRID:SCR_008417) [70] for the ab initio annotation of coding genes. For homology-based prediction, homologous data from five closely related species including *Ahaetulla prasina* (GCF_028640845.1), *Furcifer pardalis* (GCA_030440675.1), *Hemicordylus capensis* (GCF_027244095.1), *Rhineura floridana* (GCF_030035675.1), *and Zootoca vivipara* (GCF_963506605.1), were collected by NCBI RefSeq. To sum up, we integrated the above three types of evidence and short reads splice information data as input to GeMoMa v1.9 (RRID:SCR_017646) [71] workflow for a comprehensive prediction of coding genes. For the annotation results obtained from the GeMoMa, we further filtered based on the repeat sequence annotation file, removing predictions suspected to be repeat elements, ultimately obtaining the final protein coding gene annotation set.

Isoform detection by long reads

The process of CycloneSEQ long-read RNA-seq data was as follows: we filtered the TSO (template switch oligo sequence)/RTP (reverse transcription primer) and split chimeric reads based on artificial sequence. The clean data was mapping to assembly using winnowmap2 v2.03 [33] with the command: *winnowmap -u b -K 4G -G 135k -ax splice ref fastq*. We then reconstructed

transcripts based on the reference annotation using Isoquant v3.5.0 [72] with the command: *python3 isoquant.py –gene_quantification all –reference genome.fa –data_type nanopore –genedb genes.gtf –threads 16 –count_exons –bam input.bam –label sample*. Subsequently, we merged the transcripts from eight tissues, and eliminate redundancy based on an all-exon overlap threshold of greater than 0.95 between isoforms. We then performed TransDecoder v5.7.1 to predict the coding potential of all transcripts using the *–single_best_only* option. Finally, isoforms of coding gene were assigned to the correspond loci.

LncRNA annotation

LncRNA identification also based on long sequencing reads of CycloneSEQ RNA-seq. The remaining transcripts assembled by Isoquant from the isoform detection steps, which filtering by length (⩾200 nt), were used as the foundational dataset for lncRNA detection. We then utilized two tools of FEELnc v0.2.1 [73] and CPC2 v1.0.1 (Coding Potential Calculator, RRID:SCR_002764) [74] to filter potential coding genes. The candidates of lncRNA were obtained by taking the intersection of the results from both tools. We further mapped the corresponding DNBSEQ sequencing data from the same library with CycloneSEQ RNA-seq using HISAT2 v2.2.1 for alignment to the assembly. Expression detection of candidate lncRNAs was performed using FeatureCounts v2.0.1 (RRID:SCR_012919) [75]. We ultimately considered lncRNAs with TPM > 1 to be true and reliable.

***De novo* prediction for tRNA and rRNA**

Two types of noncoding RNAs were predicted, namely transfer RNAs (tRNAs) and ribosomal RNAs (rRNAs). To identify putative tRNA genes in the *P. vitticeps* genome, we employed tRNAscan-SE v2.0.10 (RRID:SCR_008637) [76] with parameters optimized for eukaryotic genomes. Subsequently, we filtered out “pseudo” and “undet” from the output generated tRNAscan-SE, and extract high-confidence tRNA genes from the BED file, producing a refined tRNA set. For rRNA prediction, we employed the barrnap v0.9 (RRID:SCR_015995) (https://github.com/tseemann/barrnap) program to identify rRNA genes in the genome, using the following command: barrnap –quiet –kingdom euk genome.fa –threads 20.

Chromosome contact analyses

In the above scaffolding procedure, the .hic files were derived from the processing of CycloneSEQ based Pore-C reads and Hi-C reads. Subsequently, the interaction contacts within the .hic files

were binned to construct the genome-wide interaction matrix at resolutions of 5 kb, 10 kb, 100 kb-, 500 kb, 100 kb, 500 kb and 1Mb. Following this, the ICE (iterative correction and eigenvector decomposition) normalization was then employed to normalize the interaction matrix. Then, cis-contacts and trans-contacts for each 100-kb window were calculated using the 100-kb normalized interaction matrix.

Missing sequence identification

We compare the Pvit2024 genome assembly with a previous (Pvit2015) assembly generated using Illumina sequencing. The Pvit2015 assembly was downloaded from NCBI RefSeq by searching for GCF_900067755.1. We excluded the mitochondrial genome from the assembly to prevent misalignment between mitochondrial and nuclear genomes. We then aligned the Pvit2015 assembly to Pvit2024 ref by minimap2 v2.23-r1116-dirty (RRID:SCR_018550) [34] with the following command: *minimap2 -ax asm20 -k14 -K 8G –secondary=no -s 80 -t 16 Pivt2015 Pvit2024*, and with paftools to obtain the aligned regions by minimap2. Additionally, we also performed alignments using the Winnowmap2 tool, which requires the creation of a database with Meryl v1.4.1 [62]. The database was created using the following commands: *meryl count k=19 output merylDB Pvit2024* and *meryl print greater-than distinct=0.9998 merylDB > repetitive_k19.txt*. The alignment was then executed with the command: *winnowmap -t 16 -W repetitive_k19.txt -K 8G –secondary=no -ax asm20 -s 80 Pvit2024 Pvit2015*. Similarly, we used paftools to obtain the alignment files for the two genomes. We further utilized a Python scripts (https://github.com/guoqunfei/Pvit_T2T) to extract unaligned regions from the alignment files. The final set of missing regions was determined by taking the intersection of the results obtained from both methods.

Missing sequences in genomic elements

We identified the proportions of coding genes, lncRNAs, and promoter within the missing regions based on coordinate overlap information. To identify missing coding genes and lncRNA, we considered a locus span and exon region with an overlap greater than 0.8 with the missing region as a missing gene in the Pvit2015 assembly. In contrast, an overlap between 0.1 and 0.8 was classified as an incomplete gene. Additionally, for the identification of promoters, we defined the 2000 bp upstream region of coding genes and lncRNAs as potential regulatory regions. An overlap greater than 0.7 between these regions and the missing region was considered a missing promoter,

while an overlap between 0.1 and 0.7 was classified as an incomplete promoter.

Calculation of GC content and repeat content

GC content was determined by calculating the total number of Gs and Cs divided by the length of the given coordinates, excluding ambiguous nucleotides (N), using a custom Python script. Repeat content was assessed based on pre-annotated repeat files, with a Python script used to extract the coordinates of repeat regions and count the number of repeat nucleotides.

Identification of S0 region

To identify regions with differential sequencing coverage between the Z and W chromosomes of *P. vitticeps*, two ZW female WGS data and four ZZ male WGS data were aligned to the assembled genome using BWA-MEM v0.7.17-r1198-dirty (RRID:SCR_012940) [63] (Supplementary Table S13). Depth of coverage was extracted using SAMtools v1.15.1 (RRID:SCR_002164) [64]. Median depth was calculated using a non-overlapping sliding window of 10 kb.

Searches for genes in SDR regions of closely related species

The genome assemblies of all closely related species, including *Gambelia wislizenii* (GCA_030847615.1), *Intellagama lesueurii* (GCA_037013535.1), *Phrynocephalus forsythia* (GCA_029282475.1), *Phrynocephalus guinanensis* (GCA_037367245.1), *Phrynocephalus putjatai* (GCA_037367255.1), *Phrynocephalus versicolor* (GCA_023846285.1), *Phrynocephalus vlangalii* (GCA_037367305.1), *Phrynosoma blainvillii* (GCA_026167975.1), *Phrynosoma platyrhinos* (GCA_020142125.1), *Sceloporus tristichus* (GCA_016801415.1), *Sceloporus undulatus* (GCA_019175285.1), as well as the outgroup species *Gallus gallus* (GCF_016699485.2) and *Homo sapiens* (GCF_009914755.1), were downloaded from NCBI RefSeq. We used the protein sequences of *AMHR2*, *BMPR1A*, *AMH*, *ADAMTS4*, *PFDN6*, and *RGL2* from human, chicken, and green anole, which were downloaded from Ensembl, as query sequences. We further conducted tblastn of blast v2.11.0 [77] searches using these query sequences against the whole genomes of closely related species, with search parameters set to *-evalue 1e-5 -num_threads 16*. For the potential loci obtained from the tblastn searches, we extended 2000 bp upstream and downstream of each locus. Subsequently, we performed detailed predictions using GeneWise v3 (RRID:SCR_015054) [78], which allowed us to accurately identify the coding regions of coding genes. Finally, we compared the predicted proteins against the non-redundant protein databases of human, chicken, and green anole to confirm the reliability of our predictions.

Gene synteny analysis

Based on the annotation methods previously applied to *P. vitticeps*, we rapidly performed batch predictions of coding genes on the genome assemblies of nine closely related species using GeMoMa. The annotation GFF3 files and coding sequences were collected. Syntenic gene pairs between *P. vitticeps* and related species were identified using the Python version of MCScan, JCVI v1.1.16, with the following command: *python3 -m jcvi.compara.catalog ortholog --score=0.95 --no_strip_names*. Syntenic blocks were filtered using the command: *python3 -m jcvi.compara.synteny mcscan --iter=1*. We combined all species' syntenic gene pairs based on *P. vitticeps* loci using the command: *python3 -m jcvi.formats.base join --boheader*. For the synteny plot of each target gene, we selected the target gene and its flanking 10 genes from the *P. vitticeps* locus and generated the plot using the command: *python3 -m jcvi.graphics.synteny*.

Phylogenetic analysis and gene evolution

The protein sequences of each orthologous group of sex-determining genes were aligned using MAFFT v7.471 (RRID:SCR_011811) [79] with the parameters: mafft --anysymbol --maxiterate 1000 --localpair pep.fa. The multiple alignment sequences were then processed to remove spurious sequences or poorly aligned regions using trimAl v1.4.rev15 (RRID:SCR_017334) [80] with the command: trimal -in pep.fa.aln -out pep.fa.aln.trimal -gt 1. Maximum likelihood inference of phylogenetic relationships was performed using IQ-TREE v2.2.0.3 (RRID_SCR_017254) [81] with the parameters: iq-tree -m TEST -bb 1000 -bnni. The ratios of non-synonymous to synonymous substitutions within six sex-determining gene pairs were calculated using KaKs_Calculator v2.0 (RRID:SCR_022068) [82] with its default settings.

Transcriptome source across different sexes and developmental stages

For the male and female gonadal transcriptome data, we used previously published datasets, which included *P. vitticeps* ZZ and ZW individuals at four developmental stages (6/7, 12, 16, and adult) [14, 46]. The early-stage gonadal RNA-seq source accession numbers are SRP304423. The adult source accession numbers used for testes of ZZ male are ERR753529 and ERR413070, and for ovaries of ZW female are ERR753530 and ERR413082.

RNA-seq and differential expression analysis

Paired-end raw reads underwent adapter trimming using SOAPnuke v1.5.6 (RRID: SCR_015025) [56] with *filter -n 0.03 -l 20 -q 0.3 -p 1 -Q 2 -G -5 1 -t 10,0,10,0 -E 70*. Quality control of the

sequencing reads was conducted using FastQC v0.12.1 (RRID:SCR_014583) . The clean data were mapped to genome using STAR v2.7.11a (RRID:SCR_004463) [83]. Counts of reads mapping to genes were obtained using FeatureCounts v2.0.1 (RRID:SCR_012919) [75] against the top-length isoform annotation with the following command: -T 16 -g gene_id -t exon. To investigate gene expression changes from stage 6/7 to adult, differential gene expression analysis was conducted between these stages for each sex, ZZ and ZW. Differential expression was performed in R v4.0.2 using the DESeq2 package v1.28.1 (RRID:SCR_015687) [84]. Additionally, we processed the raw expression matrix using TPM (Transcripts Per Million) counts to ensure comparability between samples, and ultimately used these values to generate histograms of gene expression.

Additional Files

Supplementary Fig. S1. Sex identification and additional description of genome assembly.

Supplementary Fig. S2. Quality validation of the genome assembly.

Supplementary Fig. S3. Analysis of missing sequences and related genomic features.

Supplementary Fig. S4. Expression matrix of 15,758 lncRNAs across 8 different tissues.

Supplementary Fig. S5. Analysis of sex chromosome.

Supplementary Fig. S6. Analysis of SDR genes in *P. vitticeps* and related species

Supplementary Table S1. Summary statistics of sequencing data for the genome assembly and annotation of *Pogona vitticeps*.

Supplementary Table S2. Detailed statistics for CycloneSEQ long-read RNA-seq data from eight tissues.

Supplementary Table S3. Detailed statistics for DNBSEQ short-read RNA-seq data from eight tissues.

Supplementary Table S4. Estimation of genome size and heterozygosity of *Pogona vitticeps* by *k*-mer analysis.

Supplementary Table S5. Improvement in continuity and completeness of genome assembly generated by each of the seven assembly steps as stated in main text.

Supplementary Table S6. Genome features between Pvit2024 and Pvit2015.

Supplementary Table S7. Summary of comprehensive assessment of genome completeness for 67 long-read sequenced Squamata species using BUSCO and Compleasm.

Supplementary Table S8. Summary of telomere and centromere location information of *Pogona vitticeps* genome.

Supplementary Table S9. Comparison of telomeric region characteristics between *Pogona vitticeps* assembly and 28 squamate species whose genome reached chromosome level.

Supplementary Table S10. Annotation of repeat sequences.

Supplementary Table S11. Gene model and function annotation.

Supplementary Table S12. 47 protein-coding genes and 65 lncRNA genes that are missing or unknown to be Z-linked in prior Pvit2015 assembly.

Supplementary Table S13. Data information for identification of S0 region.

Supplementary Table S14. Overview of all sequenced embryonic gonad samples included in this study.

Supplementary Table S15. Differential expression profiles of Z-link genes across various developmental stages.

Supplementary Table S16. Quality control of long-range sequencing data from two platforms.

Abbreviations

LncRNA: Long non-coding RNA; PAR: pseudo-autosomal region; SDR: sexually differentiated region; EBP: Earth BioGenome Project; VGP: Vertebrate Genomes Project; WGS: whole genome sequencing; PCR: polymerase chain reaction; HMW: high molecular weight; TRUs: telomeric repeat units; T2T: telomere-to-telomere; BUSCO: Benchmarking Universal Single-Copy Orthologs; QV: consensus quality value; RNA-seq: RNA-sequencing; TPM: Transcripts Per Million; NCBI: The National Center for Biotechnology information; UTR: Untranslated Region; 5'-UTRs: five prime untranslated regions; 3'-UTR: three prime untranslated regions; A3: alternative 3' splice site; A5: alternative 5' splice site; AF: alternative first exon; AL: alternative last exon; MX: mutually exclusive exons; RI: retained intron; SE: skipped exon; GSD: genetic sex determination; chr2qter: the terminal region of the long arm of chromosome 2; TSD: Temperature-dependent sex determination; TSO: template switch oligo sequence; RTP: reverse transcription primer ; NOR: nucleolus organizer region; tRNAs: transfer RNAs; rRNAs: ribosomal RNAs.

Code availability

The bioinformatic tools used in this work, including version, setting and parameters, had been described in the Methods section. Default parameters were applied if no parameters were mentioned for a tool. The pipeline for genome *de novo* assembly, annotation, and chromosome contact analysis, and the custom code and scripts for sex chromosomes analysis in this work had been deposited at GitHub (https://github.com/guoqunfei/Pvit_T2T). All figures were plotted in R.

Data Availability

CycloneSEQ long-read WGS data, DNBSEQ short-read WGS data, CycloneSEQ long-read RNA-seq data, DNBSEQ short-read RNA-seq data, CycloneSEQ based Pore-C data, and Hi-C data in this study are deposited in NCBI Sequence Read Archive (SRA) under BioProject accession no. PRJNAxxxxxx and in the CNGB Nucleotide Sequence Archive (CNSA) of China National Gene Bank DataBase (CNGBdb) under accession no. CNP0005509. Genome assembly, annotation, other supporting data, and material are available in the *GigaScience* GigaDB database.

Competing interests

All authors are employees of the BGI Group. They declare that they don't have stock, patents, or direct financial incentives from CycloneSEQ technology, and they have no competing interests.

Funding

The work was supported by the National Natural Science Foundation of China (grant no. 32370666 to Q.L.)

Authors' contributions

Q.L., Y.D., and Y.G. conceived the study. W.D., W.J., and Y.W. performed sample collection and tissue dissection. F.G., T.Z., X.S., and X.Y. conducted the CycloneSEQ long-read WGS experiments and sequencing. J.C., C.C., and J.Y. performed CycloneSEQ based Pore-C experiments and sequencing. Q.G., Y.P., W.C., H.C., Y.Z., and C.Y. performed genome assembly and annotation. Q.G., Y.P., Y.M., Y.Z., S.S., and B.W. conducted comparative analyses, combined the results, and draw figures. X.X. contributed computing resources. Q.L. wrote the manuscript with the inputs from all authors. All authors read and approved the final manuscript.

Acknowledgements

We thank Professor Arthur Georges and Dr. Sarah L. Whiteley from the University of Canberra for their providing transcriptome data of *P. vitticeps* across early developmental stages. We also thank the China National GeneBank for providing computing resources.

Supplementary Figures

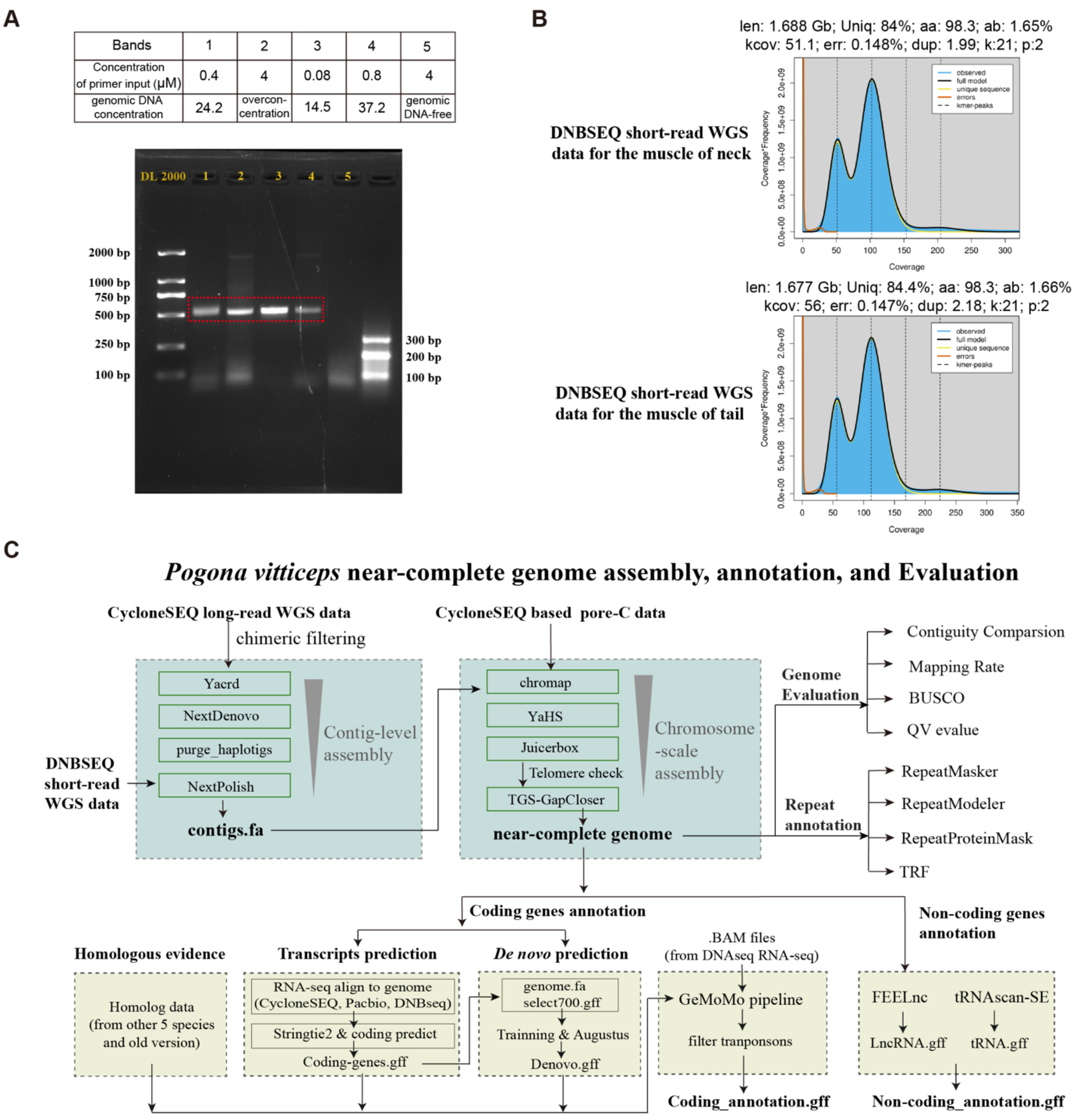

Figure S1: Sex identification and additional description of genome assembly (related to Fig. 1). (A) Amplification of the DNA fragment of the PCR primers that flank two W-chromosome-specific deletions. Band 1 to 4 were made using different concentrations of primers and genomic DNA. The fifth band without genomic DNA was the control group. (B) The 21-mer frequency distribution of two libraries of DNBSEQ short-read WGS data. (C) Comprehensive assembly and annotation workflow for the near-complete genome of *P. vitticeps*.

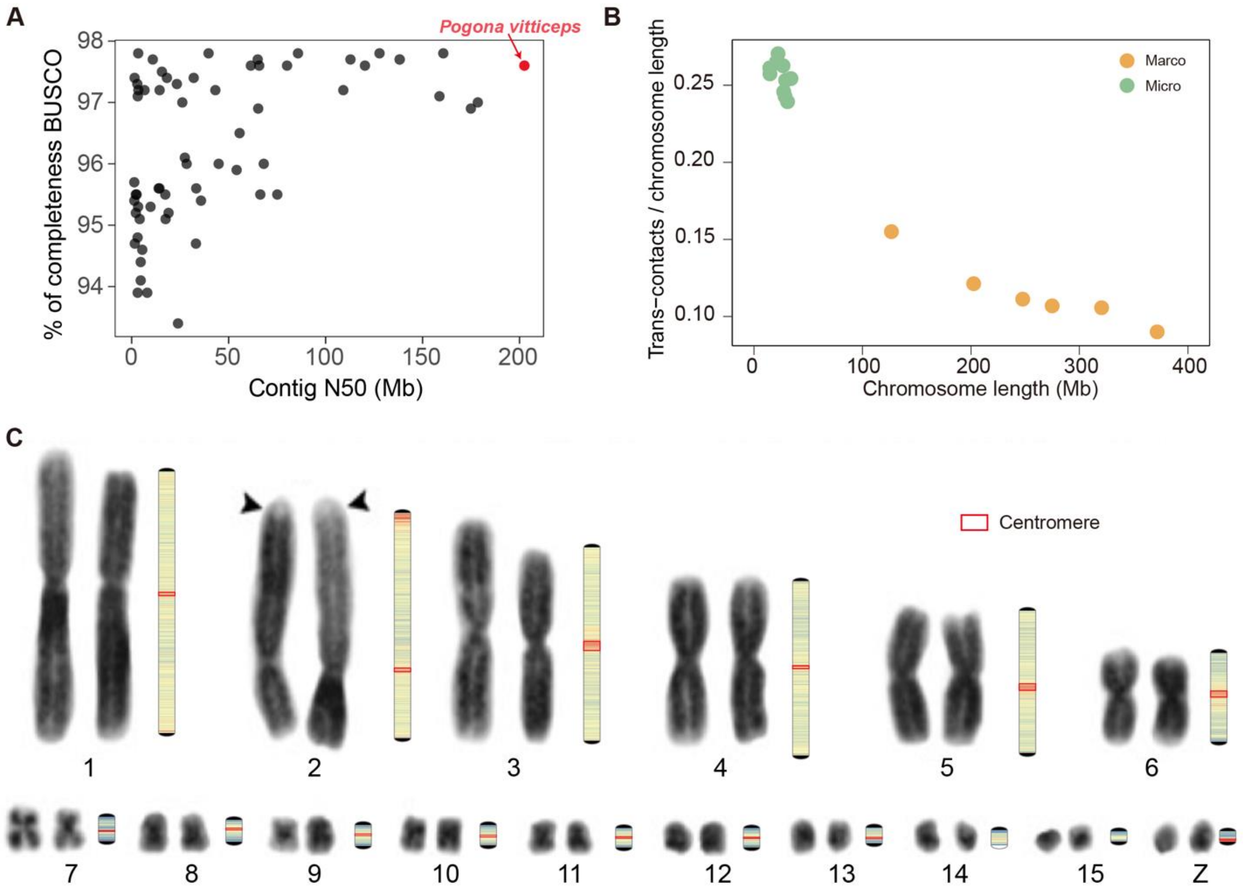

Figure S2: Quality validation of the genome assembly (related to Fig. 2). (A) The comparison of BUSCO complete score based on 7,480 Sauropsida conserved genes and contig N50 between Pvit2024 and 66 publicly available squamate genomes assembled by long-read data. (B) Trans-contact for each chromosome were calculated by ~153X Hi-C_1 data. (C) A high degree of consistency between the location of the centromere region identified by the near-complete genome with the depressed region in the karyotype map from Young et al. , which is generally believed to be the centromere.

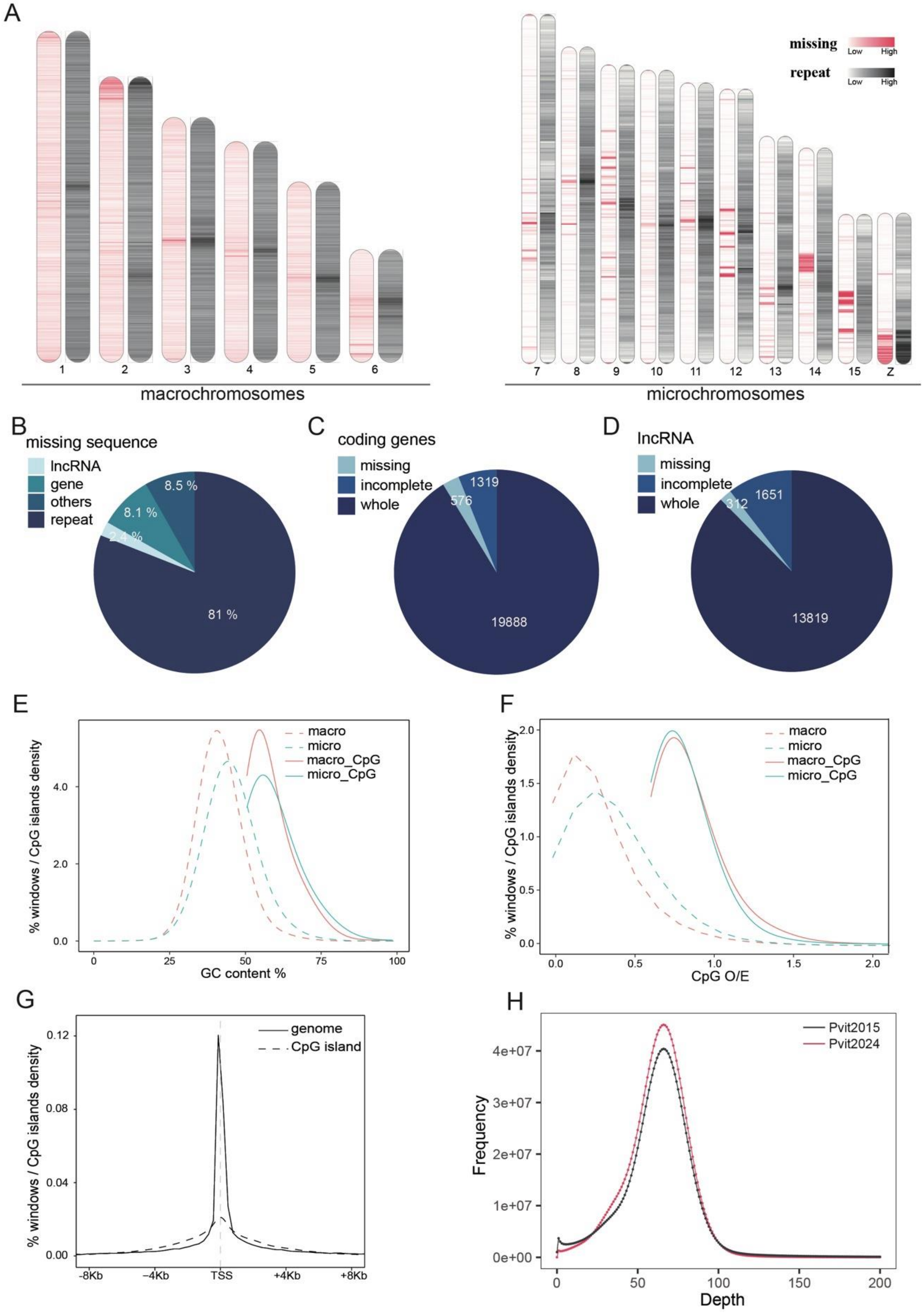
A
missing
Low High
repeat
Low High
1 2 3 4 5 6
macrochromosomes
7 8 9 10 11 12 13 14 15 Z
microchromosomes
B
missing sequence
lncRNA
gene
others
repeat
8.5 %
8.1 %
2.4 %
81 %
C
coding genes
missing
incomplete
whole
1319
576
19888
D
lncRNA
missing
incomplete
whole
1651
312
13819
E
macro
micro
macro_CpG
micro_CpG
% windows / CpG islands density
GC content %
F
macro
micro
macro_CpG
micro_CpG
% windows / CpG islands density
CpG O/E
G
genome
CpG island
% windows / CpG islands density
-8Kb
-4Kb
TSS
+4Kb
+8Kb
H
Pvit2015
Pvit2024
Frequency
Depth

Figure S3: Analysis of missing sequences and related genomic features (related to Fig. 3). (A) Overview of missing and repeat sequences distribution across all chromosomes. The red range indicates missing sequences, and the black range represents repeat sequences. (B) Proportion of various types of elements within the missing sequences. (C) Types and proportions of missing coding genes in the Pvit2015. (D) Types and proportions of missing lncRNAs in the Pvit2015. (E) In the genome, use a non-overlapping sliding window method to scan the genome, and the window size was set to 200bp. By calculating the GC content and CpG O/E value of each window, windows that satisfied the GC content ≥ 50% and CpG O/E ≥ 0.6 were identified as CpG island. The GC content density distribution maps of all windows and CpG islands in the genome were obtained, in which macro- and micro- chromosomes were displayed separately due to different genomic characteristics. (F) CpG O/E value density distribution of all windows and CpG islands in the genome, macrochromosomes and microchromosomes are displayed separately. (G) According to the genome annotation file, TSS (transcription start site) was located, and the CpG island closest to each TSS was found within 8 kb upstream and downstream, and its relative distance was calculated. For comparative analysis, 200 bp windows equal to the CpG islands were randomly selected in the genome, and the closest relative distance between these random windows and each TSS were also counted. The density distribution maps of the closest distances between TSS and the random windows of the genome and CpG islands were obtained to compare the distribution characteristics. (H) Distribution of base coverage depth after mapping DNBSEQ reads to the Pvit2015 and Pvit2024 genomes.

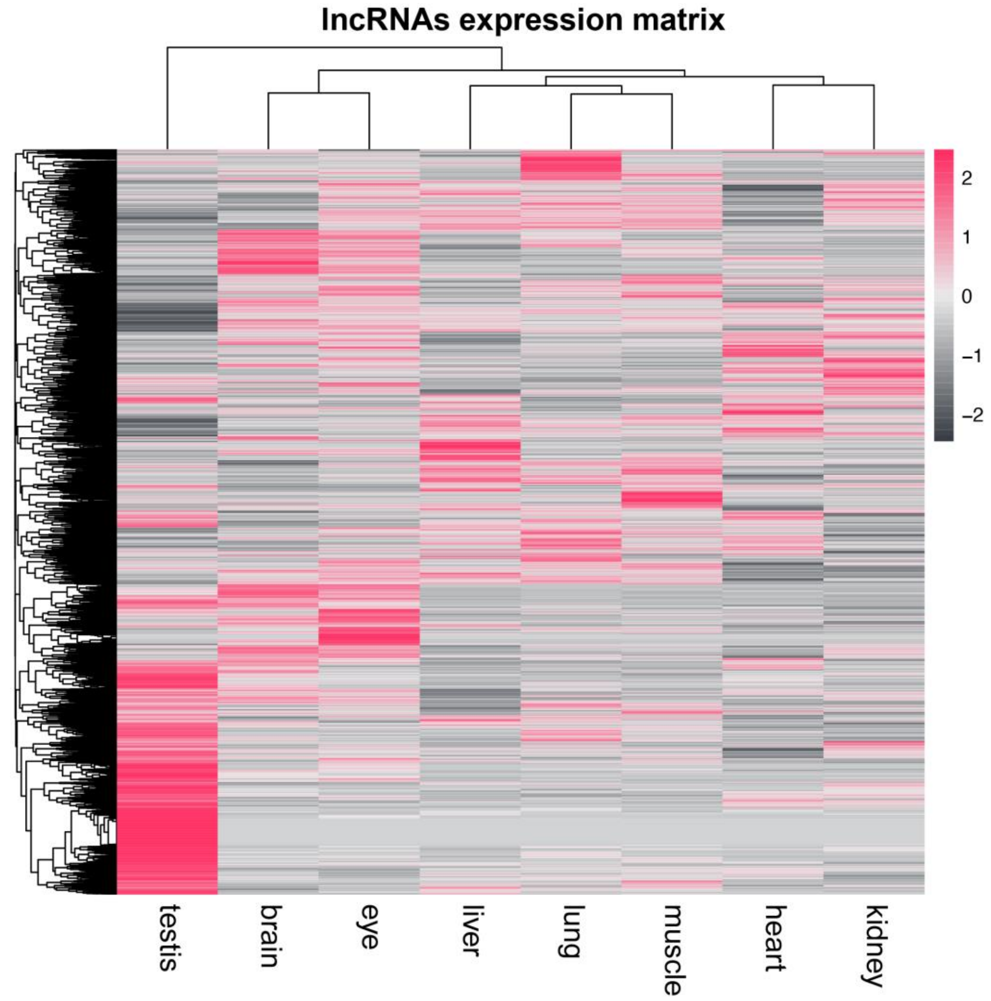

Figure S4: Expression matrix of 15,758 lncRNAs across 8 different tissues (related to Fig. 4).

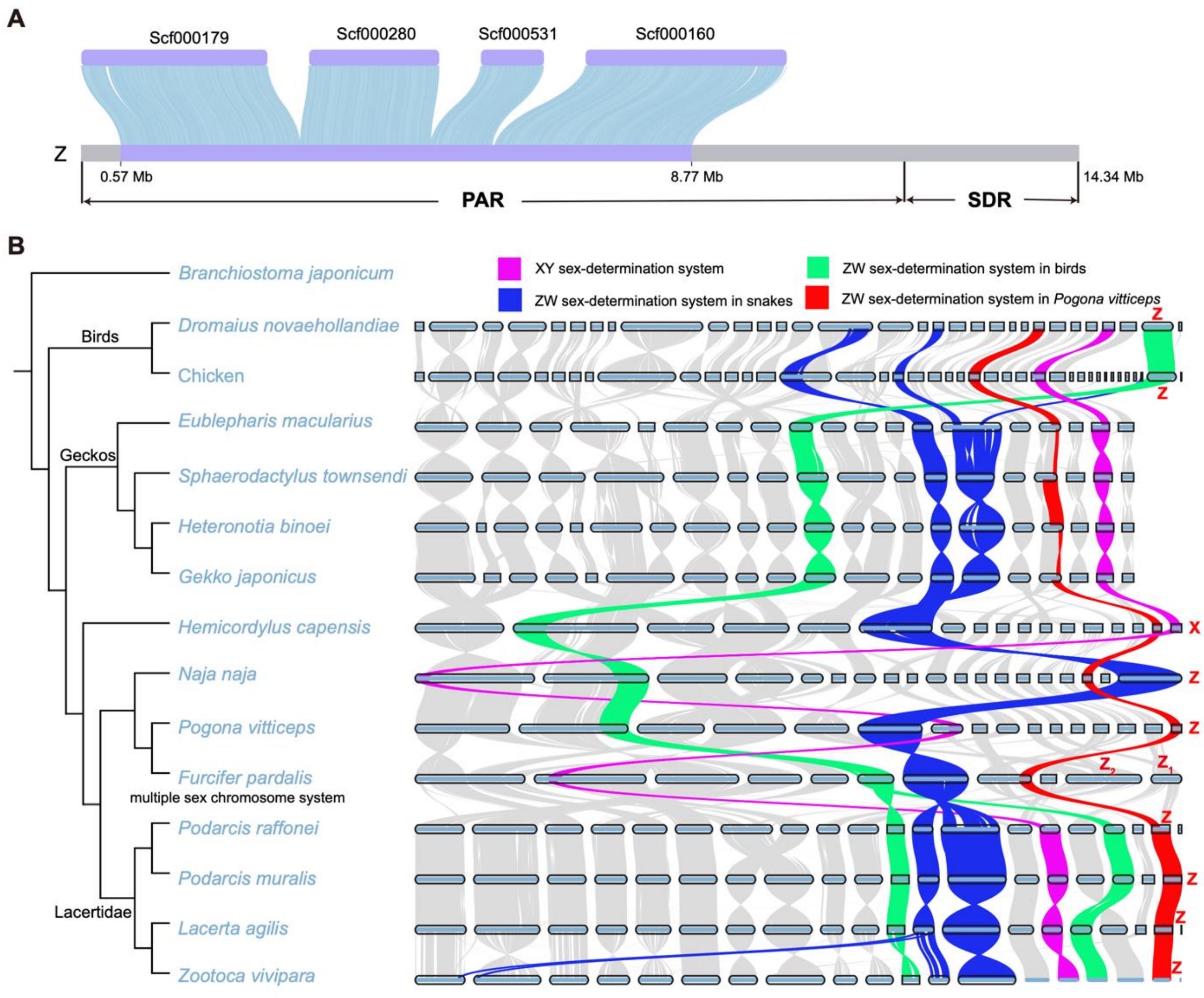

Figure S5: Analysis of sex chromosome (related to Fig. 5). (A) Collinearity between the four known Z-linked scaffolds identified by Deakin et al. and the Z chromosome of Pvit2024. (B) The construction of sex chromosome homologous regions between the identified sex chromosomes of squamate species and the Z chromosomes of two closely related species, the chicken (*Gallus gallus*) and the emu (*Dromaius novaehollandiae*), based on comprehensive gene collinearity analysis.

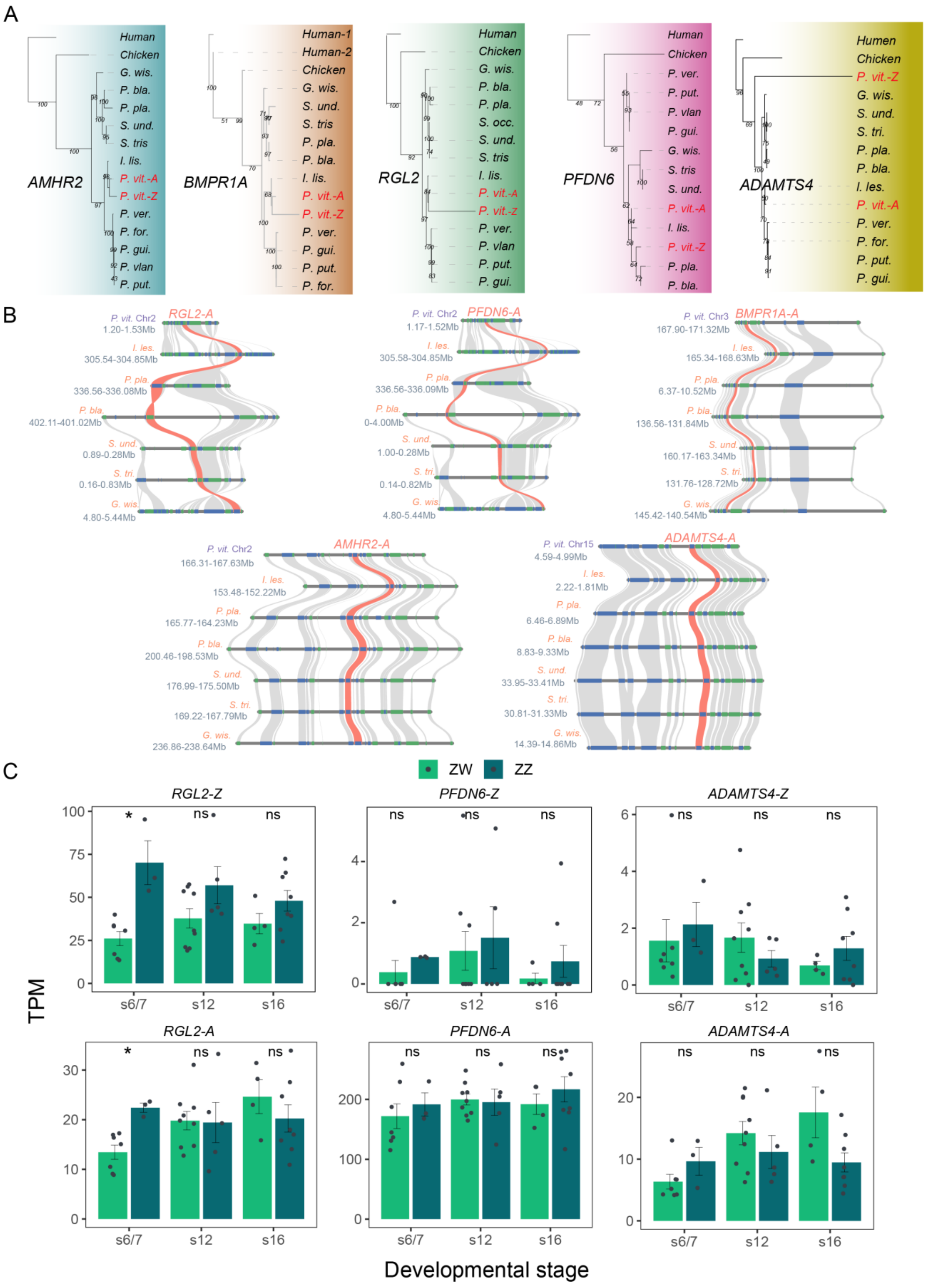
A
AMHR2
BMPR1A
RGL2
PFDN6
ADAMTS4
B
RGL2-A
PFDN6-A
BMPR1A-A
AMHR2-A
ADAMTS4-A
C
ZW
ZZ
RGL2-Z
PFDN6-Z
ADAMTS4-Z
RGL2-A
PFDN6-A
ADAMTS4-A
TPM
Developmental stage

Figure S6: Analysis of SDR genes in *P. vitticeps* (related to Fig. 6). (A) Phylogenetic relationships of five genes in different species. Light red font indicates the two copies of SDR-related genes in *P. vitticeps*. (B) Synteny relationships of autosome SDR genes and their flanking 10 genes in closely related species. The target genes are highlighted in orange, and the surrounding genes are shown in grey. (C) Differential expression of SDR gene pairs between ZZ and ZW during different developmental stages. Benjamini-Hochberg analysis of variance: ns (not significant), *$P<0.05$, **$P<0.01$, ***$P<0.001$.

References

1. Ahl, E., *Neue eidechsen und amphibien.* Zoologischer Anzeiger, 1926. **67**(7/8): p. 186-192.
2. Grenard, S., *The Bearded Dragon: An Owner's Guide to a Happy Healthy Pet*. 1999: Howell Book House.
3. Sollom, H. and H. Baron, *Clinical presentation and disease prevalence of captive central bearded dragons (Pogona vitticeps) at veterinary clinics in Australia.* Australian Veterinary Journal, 2023. **101**(5): p. 200-207.
4. Norimoto, H., et al., *A claustrum in reptiles and its role in slow-wave sleep.* Nature, 2020. **578**(7795): p. 413-418.
5. Hain, D., et al., *Molecular diversity and evolution of neuron types in the amniote brain. Science 377, eabp8202*. 2022.
6. Deakin, J.E., et al., *Anchoring genome sequence to chromosomes of the central bearded dragon (Pogona vitticeps) enables reconstruction of ancestral squamate macrochromosomes and identifies sequence content of the Z chromosome.* BMC genomics, 2016. **17**: p. 1-15.
7. Alam, S.M.I., et al., *Cross-species BAC mapping highlights conservation of chromosome synteny across dragon lizards (Squamata: Agamidae).* Genes, 2020. **11**(6): p. 698.
8. Bergeron, L.A., et al., *Evolution of the germline mutation rate across vertebrates.* Nature, 2023. **615**(7951): p. 285-291.
9. Capraro, A., et al., *Waking the sleeping dragon: gene expression profiling reveals adaptive strategies of the hibernating reptile Pogona vitticeps.* BMC genomics, 2019. **20**: p. 1-16.
10. Capraro, A., et al., *MicroRNA dynamics during hibernation of the Australian central bearded dragon (Pogona vitticeps).* Scientific Reports, 2020. **10**(1): p. 17854.
11. Holleley, C.E., et al., *Sex reversal triggers the rapid transition from genetic to temperature-dependent sex.* Nature, 2015. **523**(7558): p. 79-82.
12. Quinn, A.E., et al., *Temperature sex reversal implies sex gene dosage in a reptile.* Science, 2007. **316**(5823): p. 411-411.
13. Whiteley, S.L., et al., *Sex determination mode does not affect body or genital development of the central bearded dragon (Pogona vitticeps).* Evodevo, 2017. **8**: p. 1-15.
14. Georges, A., et al., *High-coverage sequencing and annotated assembly of the genome of the Australian dragon lizard Pogona vitticeps.* Gigascience, 2015. **4**(1): p. s13742-015-0085-2.
15. Lawniczak, M.K., et al., *Standards recommendations for the earth BioGenome project.* Proceedings of the National Academy of Sciences, 2022. **119**(4): p. e2115639118.
16. Rhie, A., et al., *Towards complete and error-free genome assemblies of all vertebrate species.* Nature, 2021. **592**(7856): p. 737-746.

17. Quinn, A.E., et al., *Extension, single-locus conversion and physical mapping of sex chromosome sequences identify the Z microchromosome and pseudo-autosomal region in a dragon lizard, Pogona vitticeps.* Heredity, 2010. **104**(4): p. 410-417.
18. Zhang, J.-Y., et al., *A single-molecule nanopore sequencing platform.* bioRxiv, 2024: p. 2024.08. 19.608720.
19. Hu, J., et al., *NextDenovo: an efficient error correction and accurate assembly tool for noisy long reads.* Genome Biology, 2024. **25**(1): p. 107.
20. Roach, M.J., S.A. Schmidt, and A.R. Borneman, *Purge Haplotigs: allelic contig reassignment for third-gen diploid genome assemblies.* BMC bioinformatics, 2018. **19**(1): p. 1-10.
21. Hu, J., et al., *NextPolish: a fast and efficient genome polishing tool for long-read assembly.* Bioinformatics, 2020. **36**(7): p. 2253-2255.
22. Zhou, C., S.A. McCarthy, and R. Durbin, *YaHS: yet another Hi-C scaffolding tool.* Bioinformatics, 2023. **39**(1): p. btac808.
23. Durand, N.C., et al., *Juicer provides a one-click system for analyzing loop-resolution Hi-C experiments.* Cell systems, 2016. **3**(1): p. 95-98.
24. Zhou, Y., et al., *Profiles of telomeric repeats in Insecta reveal diverse forms of telomeric motifs in Hymenopterans.* Life Science Alliance, 2022. **5**(7).
25. Bresler, M.a., et al., *Telescoper: de novo assembly of highly repetitive regions.* Bioinformatics, 2012. **28**(18): p. i311-i317.
26. Xu, M., et al., *TGS-GapCloser: a fast and accurate gap closer for large genomes with low coverage of error-prone long reads.* GigaScience, 2020. **9**(9): p. giaa094.
27. Simão, F.A., et al., *BUSCO: assessing genome assembly and annotation completeness with single-copy orthologs.* Bioinformatics, 2015. **31**(19): p. 3210-3212.
28. Huang, N. and H. Li, *compleasm: a faster and more accurate reimplementation of BUSCO.* Bioinformatics, 2023. **39**(10): p. btad595.
29. Srikulnath, K., et al., *Why do some vertebrates have microchromosomes?* Cells, 2021. **10**(9): p. 2182.
30. Ezaz, T., et al., *The dragon lizard Pogona vitticeps has ZZ/ZW micro-sex chromosomes.* Chromosome Research, 2005. **13**: p. 763-776.
31. Perry, B.W., et al., *Microchromosomes exhibit distinct features of vertebrate chromosome structure and function with underappreciated ramifications for genome evolution.* Molecular Biology and Evolution, 2021. **38**(3): p. 904-910.
32. Liu, J., et al., *A new emu genome illuminates the evolution of genome configuration and nuclear architecture of avian chromosomes.* Genome Research, 2021. **31**(3): p. 497-511.
33. Jain, C., et al., *Long-read mapping to repetitive reference sequences using Winnowmap2.* Nature Methods, 2022. **19**(6): p. 705-710.
34. Li, H., *Minimap2: pairwise alignment for nucleotide sequences.* Bioinformatics, 2018. **34**(18): p. 3094-3100.
35. Shin, S.C., et al., *Advantages of single-molecule real-time sequencing in high-GC content genomes.* PloS one, 2013. **8**(7): p. e68824.
36. Schramm, L. and N. Hernandez, *Recruitment of RNA polymerase III to its target promoters.* Genes & development, 2002. **16**(20): p. 2593-2620.
37. Pachano, T., et al., *Orphan CpG islands amplify poised enhancer regulatory activity and determine target gene responsiveness.* Nature genetics, 2021. **53**(7): p. 1036-1049.

38. Guigó, R., *Genome annotation: From human genetics to biodiversity genomics.* Cell Genomics, 2023. **3**(8).
39. Yao, Z.T., et al., *New insights into the interplay between long non‐coding RNAs and RNA‐binding proteins in cancer.* Cancer communications, 2022. **42**(2): p. 117-140.
40. Xu, Q., et al., *Systematic comparison of lncRNAs with protein coding mRNAs in population expression and their response to environmental change.* BMC Plant Biology, 2017. **17**: p. 1-15.
41. Palmer, D.H., et al., *How to identify sex chromosomes and their turnover.* Molecular ecology, 2019. **28**(21): p. 4709-4724.
42. Young, M., et al., *Molecular cytogenetic map of the central bearded dragon, Pogona vitticeps (Squamata: Agamidae).* Chromosome Research, 2013. **21**: p. 361-374.
43. Jarmińska-Jackowiak, T., A. Warenik-Szymankiewicz, and W. Trzeciak, *Anti-Mullerian hormone. Structure and role in sexual differentiation.* Ginekologia Polska, 1995. **66**(1): p. 51-58.
44. Josso, N., et al., *AMH and AMH receptor defects in persistent Müllerian duct syndrome.* Human reproduction update, 2005. **11**(4): p. 351-356.
45. Jamin, S.P., et al., *Requirement of Bmpr1a for Müllerian duct regression during male sexual development.* Nature genetics, 2002. **32**(3): p. 408-410.
46. Wagner, S., et al., *Gene expression of male pathway genes sox9 and amh during early sex differentiation in a reptile departs from the classical amniote model.* BMC genomics, 2023. **24**(1): p. 243.
47. Gable, S.M., et al., *The state of squamate genomics: past, present, and future of genome research in the most speciose terrestrial vertebrate order.* Genes, 2023. **14**(7): p. 1387.
48. *reptile-database.org*. 6 October 2021; Available from: http://www.reptile-database.org/db-info/SpeciesStat.html.
49. Whiteley, S.L., et al., *Two transcriptionally distinct pathways drive female development in a reptile with both genetic and temperature dependent sex determination.* PLoS Genetics, 2021. **17**(4): p. e1009465.
50. Zhang, X., et al., *Sex-specific splicing of Z-and W-borne nr5a1 alleles suggests sex determination is controlled by chromosome conformation.* Proceedings of the National Academy of Sciences, 2022. **119**(4): p. e2116475119.
51. Song, W., et al., *A duplicated amh is the master sex-determining gene for Sebastes rockfish in the Northwest Pacific.* Open Biology, 2021. **11**(7): p. 210063.
52. Cortez, D., et al., *Origins and functional evolution of Y chromosomes across mammals.* Nature, 2014. **508**(7497): p. 488-493.
53. Martínez, P., et al., *Genetic architecture of sex determination in fish: applications to sex ratio control in aquaculture.* Frontiers in genetics, 2014. **5**: p. 340.
54. Hattori, R.S., et al., *A Y-linked anti-Müllerian hormone duplication takes over a critical role in sex determination.* Proceedings of the National Academy of Sciences, 2012. **109**(8): p. 2955-2959.
55. Matsubara, K., et al., *ZW sex chromosomes in Australian dragon lizards (Agamidae) originated from a combination of duplication and translocation in the nucleolar organising region.* Genes, 2019. **10**(11): p. 861.
56. Chen, Y., et al., *SOAPnuke: a MapReduce acceleration-supported software for integrated quality control and preprocessing of high-throughput sequencing data.* Gigascience, 2018. **7**(1): p. gix120.

57. Marçais, G. and C. Kingsford, *A fast, lock-free approach for efficient parallel counting of occurrences of k-mers.* Bioinformatics, 2011. **27**(6): p. 764-770.
58. Vurture, G.W., et al., *GenomeScope: fast reference-free genome profiling from short reads.* Bioinformatics, 2017. **33**(14): p. 2202-2204.
59. Marijon, P., R. Chikhi, and J.-S. Varré, *yacrd and fpa: upstream tools for long-read genome assembly.* Bioinformatics, 2020. **36**(12): p. 3894-3896.
60. Zhang, H., et al., *Fast alignment and preprocessing of chromatin profiles with Chromap.* Nature communications, 2021. **12**(1): p. 6566.
61. Quinlan, A.R. and I.M. Hall, *BEDTools: a flexible suite of utilities for comparing genomic features.* Bioinformatics, 2010. **26**(6): p. 841-842.
62. Rhie, A., et al., *Merqury: reference-free quality, completeness, and phasing assessment for genome assemblies.* Genome biology, 2020. **21**: p. 1-27.
63. Li, H., *Aligning sequence reads, clone sequences and assembly contigs with BWA-MEM.* arXiv preprint arXiv:1303.3997, 2013.
64. Li, H., et al., *The sequence alignment/map format and SAMtools.* bioinformatics, 2009. **25**(16): p. 2078-2079.
65. Flynn, J.M., et al., *RepeatModeler2 for automated genomic discovery of transposable element families.* Proceedings of the National Academy of Sciences, 2020. **117**(17): p. 9451-9457.
66. Tarailo‐Graovac, M. and N. Chen, *Using RepeatMasker to identify repetitive elements in genomic sequences.* Current protocols in bioinformatics, 2009. **25**(1): p. 4.10. 1-4.10. 14.
67. Benson, G., *Tandem repeats finder: a program to analyze DNA sequences.* Nucleic acids research, 1999. **27**(2): p. 573-580.
68. Kim, D., et al., *Graph-based genome alignment and genotyping with HISAT2 and HISAT-genotype.* Nature biotechnology, 2019. **37**(8): p. 907-915.
69. Kovaka, S., et al., *Transcriptome assembly from long-read RNA-seq alignments with StringTie2.* Genome biology, 2019. **20**: p. 1-13.
70. Stanke, M., et al., *AUGUSTUS: ab initio prediction of alternative transcripts.* Nucleic acids research, 2006. **34**(suppl_2): p. W435-W439.
71. Keilwagen, J., F. Hartung, and J. Grau, *GeMoMa: homology-based gene prediction utilizing intron position conservation and RNA-seq data.* Gene prediction: Methods and protocols, 2019: p. 161-177.
72. Prjibelski, A.D., et al., *Accurate isoform discovery with IsoQuant using long reads.* Nature Biotechnology, 2023. **41**(7): p. 915-918.
73. Wucher, V., et al., *FEELnc: a tool for long non-coding RNA annotation and its application to the dog transcriptome.* Nucleic acids research, 2017. **45**(8): p. e57-e57.
74. Kang, Y.-J., et al., *CPC2: a fast and accurate coding potential calculator based on sequence intrinsic features.* Nucleic acids research, 2017. **45**(W1): p. W12-W16.
75. Liao, Y., G.K. Smyth, and W. Shi, *featureCounts: an efficient general purpose program for assigning sequence reads to genomic features.* Bioinformatics, 2014. **30**(7): p. 923-930.
76. Chan, P.P. and T.M. Lowe, *tRNAscan-SE: searching for tRNA genes in genomic sequences*. 2019: Springer.
77. McGinnis, S. and T.L. Madden, *BLAST: at the core of a powerful and diverse set of sequence analysis tools.* Nucleic acids research, 2004. **32**(suppl_2): p. W20-W25.
78. Birney, E., M. Clamp, and R. Durbin, *GeneWise and genomewise.* Genome research, 2004. **14**(5): p. 988-995.

79. Katoh, K., et al., *MAFFT: a novel method for rapid multiple sequence alignment based on fast Fourier transform.* Nucleic acids research, 2002. **30**(14): p. 3059-3066.
80. Capella-Gutiérrez, S., J.M. Silla-Martínez, and T. Gabaldón, *trimAl: a tool for automated alignment trimming in large-scale phylogenetic analyses.* Bioinformatics, 2009. **25**(15): p. 1972-1973.
81. Nguyen, L.-T., et al., *IQ-TREE: a fast and effective stochastic algorithm for estimating maximum-likelihood phylogenies.* Molecular biology and evolution, 2015. **32**(1): p. 268-274.
82. Zhang, Z., et al., *KaKs_Calculator: calculating Ka and Ks through model selection and model averaging.* Genomics, proteomics and bioinformatics, 2006. **4**(4): p. 259-263.
83. Dobin, A., et al., *STAR: ultrafast universal RNA-seq aligner.* Bioinformatics, 2013. **29**(1): p. 15-21.
84. Love, M.I., W. Huber, and S. Anders, *Moderated estimation of fold change and dispersion for RNA-seq data with DESeq2.* Genome biology, 2014. **15**: p. 1-21.

The responses for comments
Click here to access/download;Manuscript;Point to point response for Reviewers.clean.20250415.docx

Point to point response for Reviewers' Comments:

In this report, we have addressed all the questions and comments from the two reviewers (marked in *italic black*), by our point by point responses (marked in blue). All references cited in this document have been placed at the end.

Point to point response for Reviewer #1

Reviewer #1*: Guo et al. present a new reference genome for Pogona vitticeps, a widespread reptile model organism that is also common as a domestic animal worldwide. The genome assembly shows much improvement over an older assembly from 2017. There are two points that make this manuscript outstanding from common genome assembly papers:*

1. The authors find a new sex determination locus in this species.

2. the authors use a new nanopore sequencing technology ("CycloneSEQ"), which has so far only described in a preprint:

(https://www.biorxiv.org/content/10.1101/2024.08.19.608720v1).

In my opinion this deserves a publication in Gigascience, but both points must be focused more on a revised manuscript.

Response

We are sincerely grateful for your summary of our work and your recognition of its significance. We also appreciate the numerous constructive suggestions provided, which have significantly enhanced our manuscript. We have carefully revised the manuscript based on your comments to ensure that it includes sufficient evidence and details, and all the revised parts in the manuscript have been highlighted in dark red. We believe that the revised version represents a substantial improvement.

Below, I will explain these points:

1-1 *The authors have sequenced a male individual (ZZ), which means the long-read reference assembly is missing the W-chromosome. PAR and SDR regions are deduced from the Z sequence, by analysis of sequencing coverage of only a few sexed samples (2 females and 4 males). It is unclear if these individuals are from the same family, which could mean that the newly found SD-region could just be a family specific variation. To make the whole story more intriguing and statistical sound the authors should at least test 15 males and 15 females from different P. vitticeps populations for W-specific markers near the proposed AMH deletion. The authors should also show that the prior proposed SD locus (nr5a1) does not carry W-specific mutations in these 15+15 individuals. Furthermore, a phased assembly of a female (ZW) Pogona vitticeps individual, could enable the assembly of the missing W-chr and should be included, it would even improve analysis of W-specific sequences in the proposed additional individuals.*

Response

Thank you for this constructive suggestion. Based on your comment, I have broken them down into the following four points and will address each one in turn:

Point 1: *PAR and SDR regions are deduced from the Z sequence, by analysis of sequencing coverage of only a few sexed samples (2 females and 4 males). To make the whole story more intriguing and statistical sound the authors should at least test 15 males and 15 females from different P. vitticeps populations for W-specific markers near the proposed AMH deletion.*

Response

We sincerely appreciate your insightful suggestion to expand the sample size for validating the PAR/SDR regions. While logistical constraints (e.g., difficulties in obtaining specimens from diverse wild populations due to import restrictions) limited our ability to reach the proposed 15 males and 15 females, we made significant efforts to enhance our cohort.

We collected an additional 14 individuals and rigorously validated their genetic sex using W-specific molecular markers, as described in the Methods section (Sex identification). These markers were analyzed via agarose gel electrophoresis, where ZZ males exhibited a single band and ZW females showed two distinct bands (**Figure R1.1**; **Supplementary Fig. S13**) [1]. Combined with the original 6 samples, our final dataset now includes 8 ZZ males and 12 ZW females (**Table R1.2**).

Although this sample size falls short of the reviewer's recommendation, we note that it aligns with established practices in sex chromosome studies [2]. For instance, recent work on *Hippocampus erectus* sex determination demonstrated robust PAR/SDR demarcation with comparable sample size (10 females + 10 males) [2]. Notably, our re-analysis incorporating the expanded dataset confirmed consistent PAR and SDR boundaries (see **updated Fig. 5C**), reinforcing the reliability of our initial findings.

We acknowledge that larger, population-diverse cohorts could further strengthen statistical confidence. However, the current evidence – supported by methodological rigor, consistency across analyses, and congruence with published precedents – provides and compelling foundation for our conclusions regarding Z chromosome architecture in *P. vitticeps*.

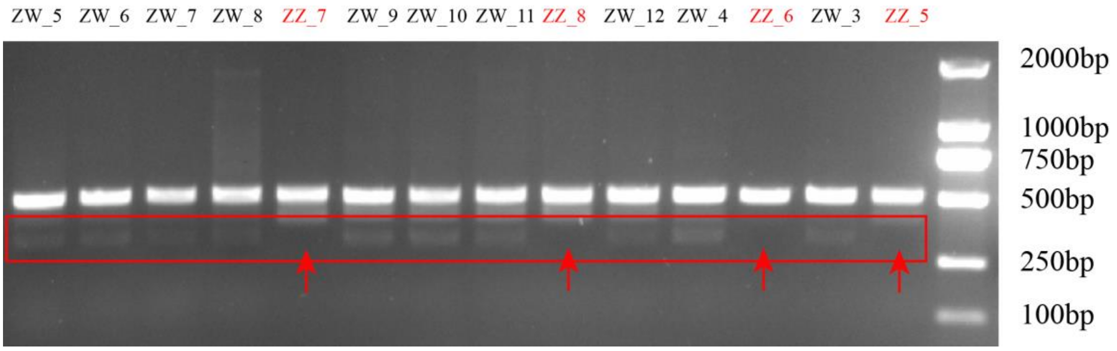

Figure R1.1 The agarose gels for the additional 14 individuals.

Table R1.2 8 ZZ male and 12 ZW female individuals' information used for PAR/SDR demarcation.

Sample ID	Genotype	Total clean data (Gb)	Genome coverage (X)
ZW_1	ZW female	63.19	35.3

ZW_2	ZW female	66.05	36.9
ZZ_1	ZZ male	68.56	38.3
ZZ_2	ZZ male	112.23	62.7
ZZ_3	ZZ male	100.60	56.2
ZZ_4	ZZ male	98.27	54.9
ZW_3	ZW female	246.48	137.70
ZW_4	ZW female	149.29	83.40
ZW_5	ZW female	153.76	85.90
ZW_6	ZW female	165.04	92.20
ZW_7	ZW female	167.01	93.30
ZW_8	ZW female	205.85	115.00
ZW_9	ZW female	185.62	103.70
ZW_10	ZW female	176.49	98.60
ZW_11	ZW female	148.75	83.10
ZW_12	ZW female	147.85	82.60
ZZ_5	ZZ male	161.82	90.40
ZZ_6	ZZ male	257.04	143.60
ZZ_7	ZZ male	187.41	104.70
ZZ_8	ZZ male	121.36	67.80

Point 2: *PAR and SDR regions are deduced from the Z sequence, by analysis of sequencing coverage of only a few sexed samples (2 females and 4 males). It is unclear if these individuals are from the same family, which could mean that the newly found SD-region could just be a family specific variation.*

Response

We thank you for highlighting this critical concern. To preclude familial bias in PAR/SDR analysis, our cohort integrates: (1) five geographically/temporally diverse samples from public datasets (Supplementary Table S13), (2) one newly collected sample with no lineage overlap to published data, and (3) 14 additional samples collected from different locations. All individuals were verified as unrelated through breeder records and genomic validation. Besides this, to empirically rule out familial relatedness, we conducted two complementary genomic analyses:

1. Principal Component Analysis (PCA) was performed using Plink v1.07 (RRID:SCR_001757) [3] on genome-wide SNPs called using FreeBayes v0.9.21 (RRID:SCR_010761) [4]. The resulting PCA plot (**Figure R1.3**) revealed no

clustering patterns indicative of shared ancestry, with samples broadly dispersed across genetic space.

2. Kinship analysis was conducted using PLINK's pairwise PI_HAT estimates, which quantify the proportion of alleles shared identical-by-descent (IBD). All pairwise values fell within a narrow range of 0.012 ~ 0.100 (**Figure R1.4**), well below the threshold for even distant relatedness (PI_HAT > 0.1) [5]. Critically, no sample pairs approached values suggestive of first- or second-degree relationships (PI_HAT > 0.25), confirming the absence of parent-offspring, sibling, or avuncular connects.

The combination of samples from different sources, PCA-based population structure validation, and kinship coefficient analysis provides robust evidence that our cohort is devoid of familial relatedness. This ensures that the observed Z-W coverage patterns and the identification of the SDR region reflect species-level genomic architecture rather than lineage-specific artifacts.

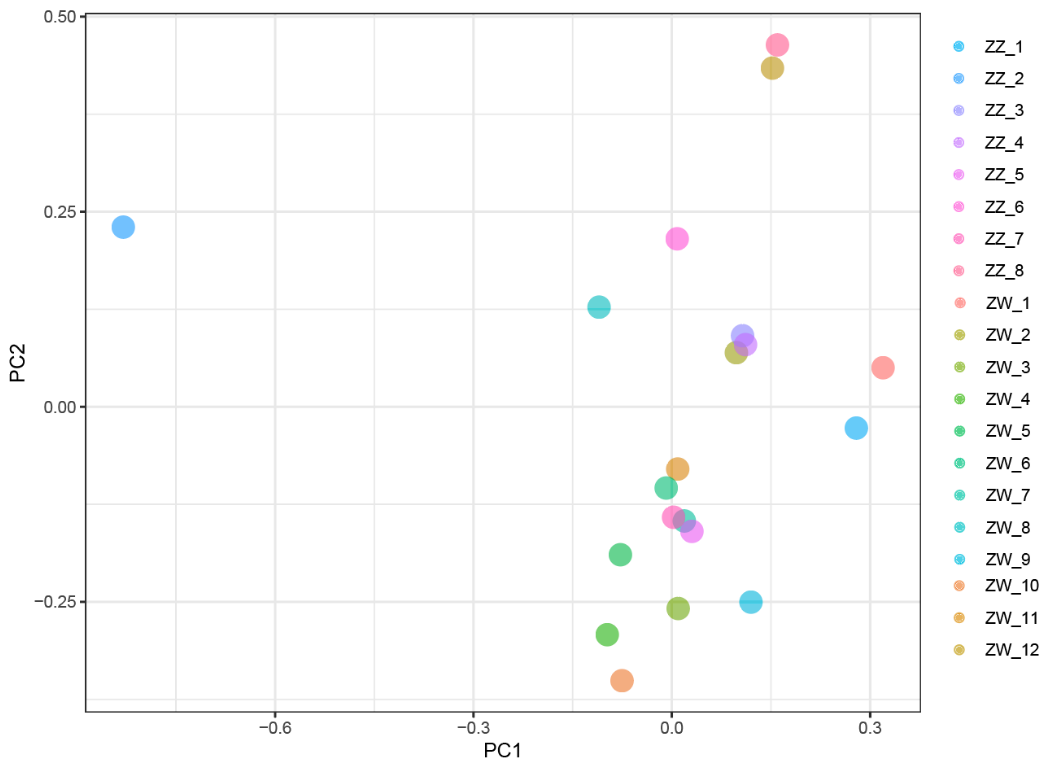

Figure R1.3 PCA analysis of 20 samples.

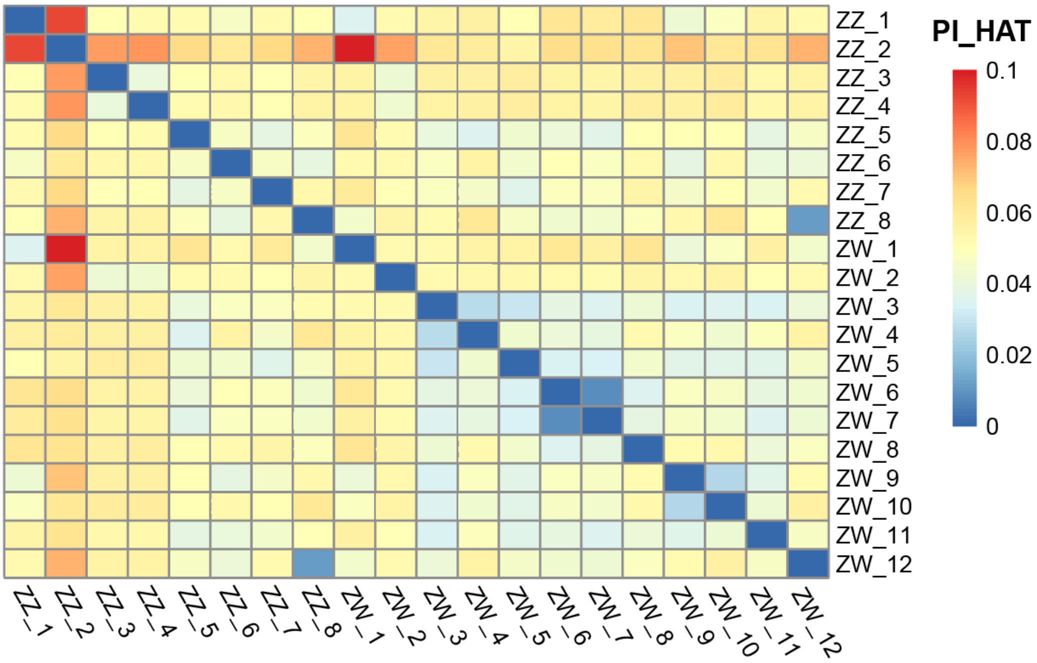

Figure R1.4 kinship analysis of 20 samples.

Point 3: *The authors should also show that the prior proposed SD locus (nr5a1) does not carry W-specific mutations in these 15+15 individuals.*

Response

We conducted a comprehensive analysis to evaluate potential W-specific mutations in the *nr5a1* locus. First, we compared genetic diversity patterns between the *nr5a1* region and autosomes (Chr1~Chr15). Comparative analysis of SNP density (per Mb) and average sequencing coverage between the *nr5a1* gene region and autosomes (Chr1~ Chr15) (**Figure R1.5 and Figure R1.6**) revealed no evidence of a two-fold depth reduction across all 20 samples.

Building on these global patterns, we performed detailed nucleotide-resolution analysis of sequencing coverage across all sites within the *nr5a1* region (**Supplementary Table S17**). Among 26,763 interrogated loci, 24,072 (89.95%) exhibited consistent homozygosity across all samples. None of the 2691 retained loci exhibited mutations specific to ZW individuals, with any observed mutation being consistently shared across all ZW individuals. This systematic evaluation conclusively excludes W-linked mutational signatures in *nr5a1*.

Collectively, our multilayered analysis – spanning regional diversity metrics, depth profiles, and allele-specific patterns – confirms the absence of W-specific mutations in *nr5a1*. These findings support its characterization as a conserved sex-determining gene on the Z chromosome, rather than a W-derived element.

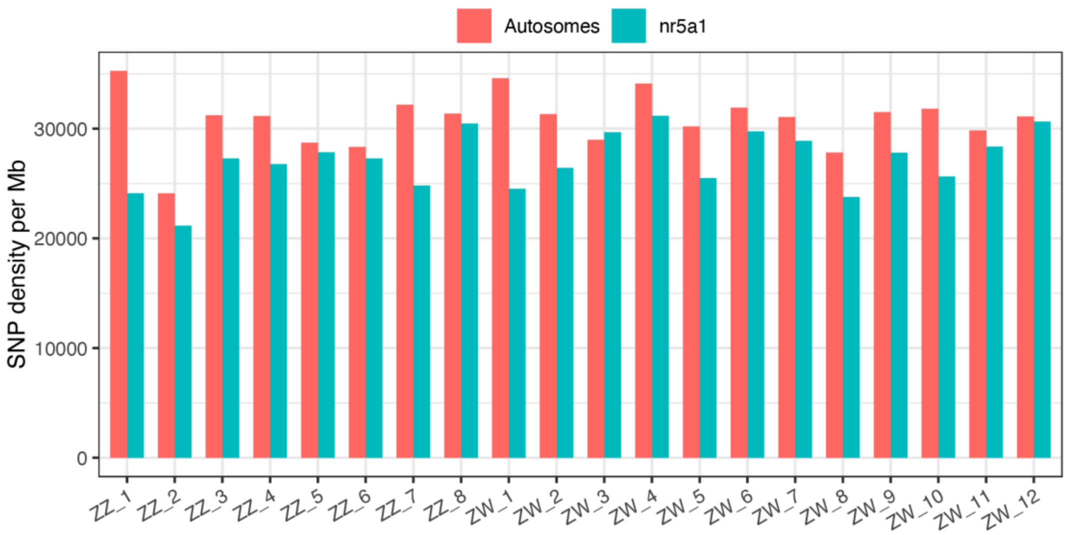

Figure R1.5 SNP density per Mb between *nr5a1* region and Autosomes (Chr1~Chr15).

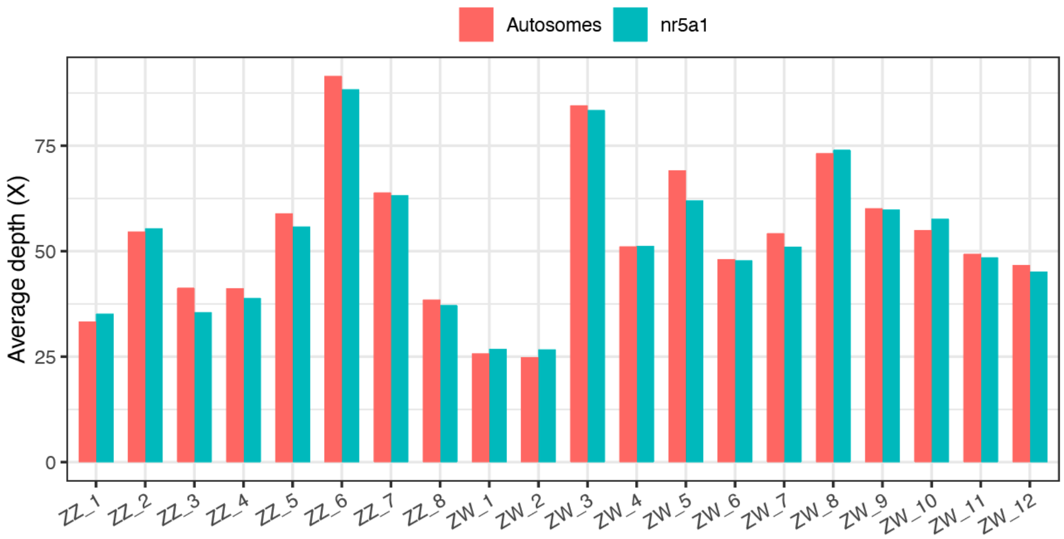

Figure R1.6 Average sequencing coverage (X) between *nr5a1* region and Autosomes (Chr1~Chr15).

Point4: *Furthermore, a phased assembly of a female (ZW) Pogona vitticeps individual, could enable the assembly of the missing W-chr and should be included, it would even improve analysis of W-specific sequences in the proposed additional individuals.*

Response

We fully acknowledge the importance of resolving the W chromosome to strengthen our conclusions. However, due to limitations in project funding, achieving W chromosome assembly would require collecting and sequencing data from parents and F1 ZW females, as well as performing haploid assembly. Such a task is beyond the scope of the current project. Nevertheless, we hope to address this in future work.

1-2 *A technology aware reader would like to see more information on the specifics of the CycloneSEQ data quality and handling and maybe a comparison to competing technologies.*

Response

We thank you for emphasizing the importance of comparing emerging sequencing technologies with other sequencing platforms. However, since we did not sequence data from the PacBio and ONT platforms in this study, direct comparisons are not feasible. For more detailed sequencing performance metrics of this sequencing technology, please refer to the preprint article "A single-molecule nanopore sequencing platform" [6].

1-3 *Which enzymes and buffers were used to prepare the library? In the sections on the methods, there are only superficial descriptions such as (DNA repair buffer/enzyme, DNA clean beads, wash buffer for long fragments). Is it a kit or were the enzymes and buffers purchased individually?*

Response

We thank you for highlighting the need for methodological transparency. The CycloneSEQ library was prepared using the CycloneSEQ Universal DNA Library Prep Set kit (H940-000013), a commercially available kit optimized for long-read

sequencing. The full protocol and reagent specifications are available in the MGI Technical Documentation:
(https://en.mgitech.cn/Home/Products/instruments_info/id/65.html).

1-4 *I cannot find the procedure for preparation and sequencing of the long-read cDNA libraries.*

Response

We apologize for any ambiguity in the original manuscript and appreciate your request for clarification. The long-read cDNA libraries were prepared using the MGI CycloneSEQ RNA Library Prep Kit, following the manufacturer's optimized protocol. For specific and detailed methodological descriptions, please refer to the Methods section "CycloneSEQ long-read RNA-seq library preparation and sequencing". (**Lines 587-596**)

1-5 *How many flowcells were needed to generate the different datasets?*

Response

We thank you for raising this important technical detail. The number of flowcells used to generate each dataset has been thoroughly documented in Table S1 of the Supplementary Materials (Table **R1.7**).

Table R1.7 The number of flowcells for different datasets.

Data type	Sample/Tissue	Flowcell number
CycloneSEQ long-read WGS data	muscle and lung	36
CycloneSEQ based Pore-C data	liver	6
CycloneSEQ long-read RNA-seq data	eye	3
	kidney	4
	live	3
	lung	3
	muscle	4
	testis	3
	brain	5
	heart	3

1-6 How do the read-length distributions look like (statistics over all reads not only selected 40Kb+)?

Response

We thank you for requesting clarity on read-length distributions. As show in **Figure R1.8**, the full distribution of all raw CycloneSEQ reads (prior to size selection), with a N50 of 32.4 kb and over 40% of reads exceed 40 kb.

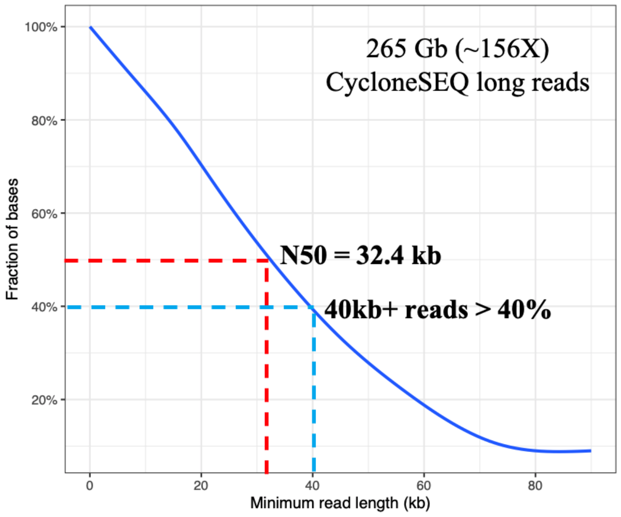

Figure R1.8 Frequency distribution of read lengths in all data.

1-7 How was the variability between those runs, especially cumulative output over time?

Response

We thank you for the highlighting the importance of sequencing run consistency. The sequencing for this study were primarily conducted using the WT platform of CycloneSEQ, a platform optimized for moderate-throughput applications. For the majority of run, the WT platform consistently generated 15~20 Gb of data per flowcell, with minimal variability across experimental batches. The WT platform's reliability in delivering stable yields within the 15~20 Gb range ensured robust data generation for the core assembly and sex chromosome analyses.

1-8 What hardware was needed to run the basecalling and what was the runtime?

Response

Basecalling for CycloneSEQ runs was performed in real-time during sequencing using the integrated computational module of the WT instrument. The process utilized a dedicated hardware configuration comprising an Intel i7/i9 CPU (24 cores), 64 GB DDR4 RAM, a NVIDIA RTX 4090D GPU, and a 512 GB NVMe system drive, which collectively enabled simultaneous signal acquisition and basecalling without delaying the sequencing workflow. Each run on the WT platform completed within 24 hours, including both sequencing and Basecalling. This integrated approach ensured rapid data availability while maintaining high accuracy.

1-9 *How is the Q-Value distribution of the reads?*

Response

The Q-value distribution of raw CycloneSEQ reads in shown in **Figure R1.9**.

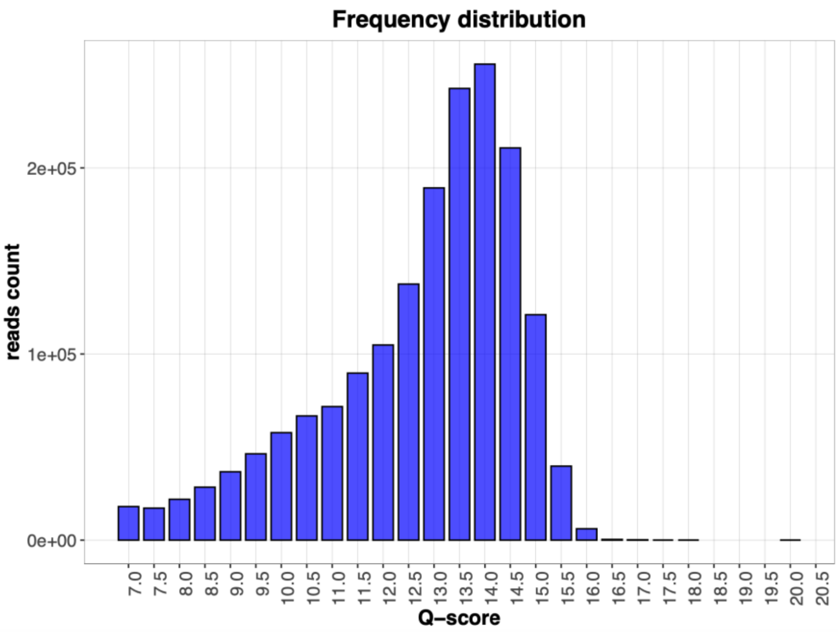

Figure R1.9 Frequency count distribution of Q-value for reads in all data.

1-10 *Why is the consensus accuracy of the assembly low (Q36.4)? can it be improved? Typically reference quality genomes should have Q40+.*

Response:

We thank you for raising the critical question regarding the consensus accuracy (QV=36.4) of our assembly. The observed QV value primarily stems from the challenges inherent to assembling this species' highly heterozygous genome, which exhibits extensive allelic divergence between homologous chromosomes. During the assembly process, regions of high heterozygosity often led to erroneous merging of divergent haplotypes into single contigs. These artifacts, misinterpreted as sequencing errors during quality assessment, disproportionately inflated base-level discordance and reduced the overall QV score.

1-11 *Which regions of the genome display lower consensus accuracies (is it random or sequence specific)?*

Response:

By assembly-specific 21-bp k-mers distribution feature, we find that the assembly errors are random rather than sequence specific. As demonstrated in Figure R1.10, we systematically mapped assembly-specific 21-bp k-mers (entirely absent in raw WGS data) across the genome. Their uniform spatial distribution provides definitive evidence that assembly errors are randomly distributed. This pattern strongly argues against locus-specific technical biases in genome reconstruction.

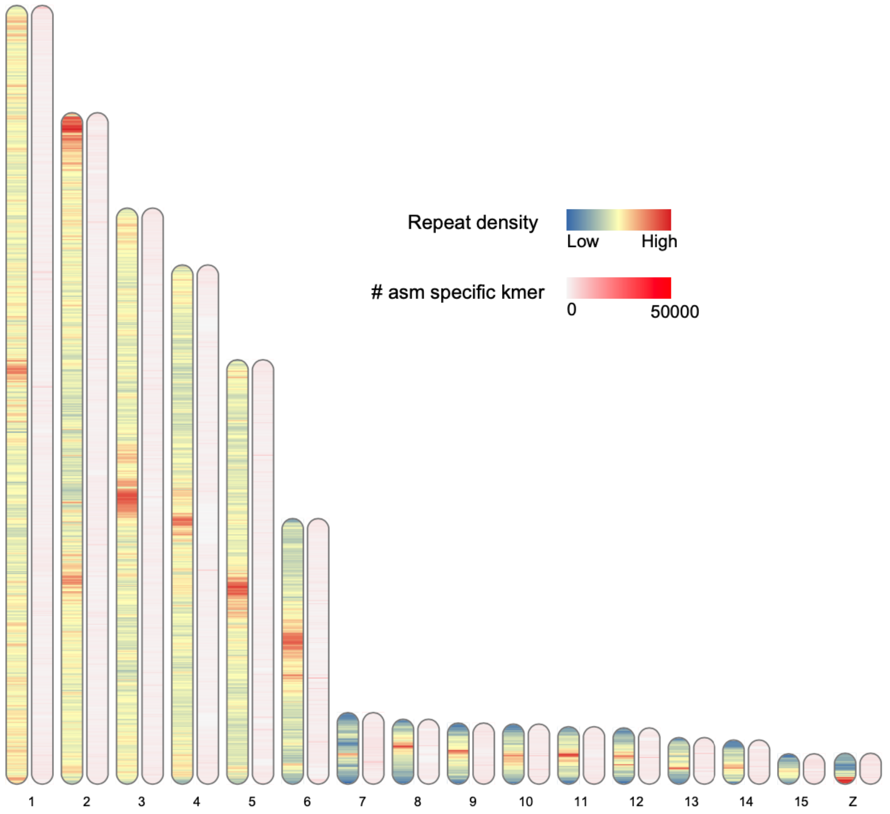

Figure R1.10 The frequency distribution of assembly-specific 21-bp kmers (absent in raw WGS data) within 500-kb windows (Right) and the repeat density (Left).

Minor comments:

1-12 *L.900: PRJNAxxxxxx looks like a placeholder, insert the true number,please.*

Response

Thanks. We have replaced the placeholder "PRJNAxxxxxx" with **PRJNA1184386** in **Line 923**. Morever, all sequencing data of 14 additional samples used for sex determination analysis have been deposited in public databases (NCBI: PRJNA1184386; CNSA: CNP0005509) to ensure transparency and reproducibility.

Point to point response for Reviewer #2

Reviewer #2: *Impressive work but needs major revision to be accepted. The authors compressed everything in the result section and did not put enough effort into the other sections. Introduction and discussion need major changes and more details regarding*

many aspects of the study that comes in the results. Methods need rearrangement. It's common to keep the order of methods such as first DNA extraction, then sequencing and so on. The data availability needs to be completed. Biosamples for each sequenced tissue, all the reads, and even intermediate assemblies need to be submitted to the database and reported in the manuscript. More specific comments are on the copy of the manuscript attached for the authors.

Response

We sincerely thank you for the positive assessment of our work and for the thoughtful, detailed feedback that has significantly strengthened the manuscript. We will proceed to reply to each comment marked by the reviewer in the manuscript one by one. Finally, all the revised parts in the manuscript have been highlighted in dark red.

2-1 *Software and database versions are missing throughout the manuscript.*

Response

Thank you for highlighting the importance of software and database version transparency for reproducibility. We have rigorously updated the Methods section to include full version details for all software and databases used in this study, aligning with GigaScience's reporting standards.

2-2 *The background description is too poor.*

Response

Thank you for your feedback. Upon review, we agree that it was insufficient. We have now rewritten the background section to provide a more comprehensive and detailed description.

2-3 *Lines 29 "N50 of 202.5 Mb and all contigs anchored": Explain if it's any better than previous genomes published for this organism.*

Response

We appreciate your suggestion. We have added key assembly improvement metrics in the Abstract: "The new reference genome is ~1.8 Gb, shows a 5,700-fold improvement

in contiguity (N50 = 202.5 Mb vs 35.5 kb) and complete chromosomal anchoring (16 scaffolds vs. 13,749).” (**Lines 30-32**)

2-4 *The conclusions are too short.*

Response

Thank you for your feedback. Due to word count limitations (not exceed 250 words), the conclusions were kept concise. However, we will expand on the key findings and implications in the revised manuscript to provide a more comprehensive summary.

2-5 *Introduction does not include many info that is needed.*

Response

Thank you for your feedback. As per your suggestion, we have added more relevant information and removed unnecessary details. We have also reorganized the paragraphs accordingly, with significant revisions made to the first paragraph of the Introduction to better align with your expectations.

2-6 *Lines 46 “In the wild, P. vitticeps has a natural distribution in the eastern and central Australia.” Ambiguous*

Response

Thank you for your feedback. Following your suggestion regarding the Introduction, we have reviewed the sentence in **Line 46** and agree that it is ambiguous and not essential to the main content. Therefore, we have removed it from the revised manuscript.

2-7 *Lines 48-50 “It is one of the most popular reptile pets nowadays, owing to ease of breeding in captivity, presence of morphs varying in scale coloration, and docility to their owners.” Unrelated*

Response

Thank you for your feedback. We have removed the sentence in the revised manuscript to ensure the content remains focused and aligned with the main theme.

2-8 *Lines 54-56 "as it possesses a ZZ/ZW genetic sex determination system whereas the final sex is also influenced by environmental temperature during embryonic development." Break it into a separate sentence*

Response

Thank you for your feedback. We have addressed the sentence in **Lines 54-56** by splitting it and reorganizing the content for better clarity and flow.

2-9 *Lines 109-114 "probably due to the well-known challenge of assembling the highly repetitive telomeric regions [7] which was also evidenced by the prevalent absence of telomeric sequences in the chromosomal ends of most squamate genomes published so far (Supplementary Table S9). However, we were able to find many CycloneSEQ reads containing TRUs before assembling, suggesting that the missing of telomeres are mainly due to computational drawback rather than sequencing omission." move to discussion*

Response

We thank you for the constructive suggestion. As recommended, the highlighted text has been relocated to the Discussion section (now **Lines 438-446**) to better contextualize telomere assembly challenges within broader genome assembly limitations. This adjustment improves the manuscript's logical flow by positioning technical limitations within comparative biological interpretations.

2-10 *Lines 132-133 "our Pvit2024 assembly outperformed all other squamate genomes reported so far in terms of continuity" add discussion*

Response

We appreciate this suggestion. The statement regarding assembly continuity has been incorporated into the Discussion (**Lines 436-438**).

2-11 *Lines 152 "7,480 Sauropsida conserved genes" more details. did you find them?*

Response

Thank you for raising this critical point. We have clarified (**Lines 152-153**) that the 7480 conserved genes derive from the Sauropsida_odb10 BUSCO dataset, representing single-copy orthologs shared across birds, squamates, and turtles.

2-12 *Lines 162 "good agreements" What does this mean? "Good" needs to be replaced.*

Response

We sincerely thank you for the precise suggestion to improve terminology clarity. In response, the phrase "good agreements" has been replaced with "high consistency" to objectively describe the Hi-C validation results.

2-13 *Lines 169 cite more studies that published microchromosomes in lizards.*

Response

We thank you for emphasizing the importance of contextualizing our findings within the broader literature on reptilian microchromosomes. In response, we have added three key references to the revised manuscript that explicitly discuss microchromosomes in squamates [8-10].

2-14 *Lines 174 "GC content, lower repeat content, and more frequent inter-chromosomal interaction" Why do we care?*

Response

We thank you for raising this important point. The three microchromosomal features highlighted in Lines 174 — GC content, repeat content, and inter-chromosomal interaction patterns — serve as established quality metrics in Sauropsida genome assembly studies. Here, we report these parameters to objectively validate the completeness and accuracy of our assembly, rather than to explore their biological implications.

2-15 *Lines 175-176 "These multiple lines of evidence together highlight the reliability of the Pvit2024 chromosomal assembly." Move to discussion*

Response

We thank you for this suggestion. The sentence highlighting assembly reliability has been moved to the Discussion (**Lines 447-450**) and expanded with supporting validation data. This revision consolidates evidence for assembly quality within the broader genomic context.

2-16 Microchromosome background info: add background for any concept that comes to result section.

Response

Thank you for your suggestion. Detailed background information on “Microchromosome” has been added to the Introduction section to enhance clarity and provide context.

2-17 Lines 439-445 “P. vitticeps possesses a ZZ male/ZW female genetic sex determination (GSD) system. However, the default sexual fate of ZZ embryos can be overwritten by high incubation temperature, leading to sex reversal of ZZ males to ZZ females [11]. The capacity of a ZZ embryo to develop as a normal female without the help of W chromosome suggests that sexual fate is most likely determined by a dosage-sensitive gene on the Z chromosome [12]. Therefore, the achievement of a complete Z chromosome sequence represents a particularly important beginning for disclosing the sex-determining cascade in P. vitticeps. " This needs to go to introduction.

Response

Thank you for your suggestion. We have incorporated the content from **Lines 439-445** into the first paragraph of the Introduction to provide a more comprehensive background and context for the study.

2-18 Lines 516-517 BioSample ID?

Response

Thank you for your attention! The BioSample information for the corresponding samples is presented in the table below (**Table R2.1**). Additionally, we have included this information in the supplementary materials for readers’ reference. Please note that

there are some issues with the CycloneSEQ data upload to NCBI, which are currently being resolved. However, the corresponding data are fully accessible and available for download in the CNGBdb database.

BioSample ID information have been added to **Supplementary Table S1**.

Table R2.1 BioSample ID for all uploaded datasets.

Data type	Instrument/ Platform	Sample/Tissue	NCBI Biosample No.	NCBI Accession No.	CNSA Accession No.
CycloneSEQ long-read RNA-seq data	CycloneSEQ	eye	SAMN44670089	***	CNS1198628
		kidney	SAMN44670090	***	CNS1198630
		live	SAMN44670091	***	CNS1198631
		lung	SAMN44670092	***	CNS1198632
		muscle	SAMN44670093	***	CNS1198633
		testis	SAMN44670094	***	CNS1198634
		brain	SAMN44670095	SRR31361862	CNS1198627
		heart	SAMN44670096	SRR31361861	CNS1198629
DNBSEQ short-read RNA-seq data	DNBSEQ	brain	SAMN44670097	SRR31361884	CNS1065805
		eye	SAMN44670098	SRR31361883	CNS1065806
		heart	SAMN44670099	SRR31361882	CNS1065807
		kidney	SAMN44670100	SRR31361881	CNS1065808
		liver	SAMN44670101	SRR31361880	CNS1065809
		lung	SAMN44670102	SRR31361879	CNS1065810
		muscle	SAMN44670103	SRR31361878	CNS1065811
		testis	SAMN44670104	SRR31361877	CNS1065812

2-19 Lines 533 Keep the order of the sections in a rational order. First extraction.

Response

We thank you for emphasizing the importance of methodological clarity. The Methods section has been restructured to follow a logical workflow, beginning with Sample collection, followed by different kinds of library preparation and sequencing (include CycloneSEQ long-read WGS data, CycloneSEQ based Pore-C data and CycloneSEQ long-read RNA-seq data). This reorganization aligns with best practices in genomics reporting and enhances reproducibility.

2-20 Lines 614 All reads and intermediate assemblies need their database information. (SRR?)

Response

We thank you for emphasizing the importance of open data access. All sequencing data have been deposited in NCBI and CNSA. The detail information can be found in **Table R2.1** and **Supplementary Table S1**. And the intermediate assemblies have been deposited in GigaDB database.

Table R2.2 The accession ID of NCBI and CNSA for all uploaded datasets

Data type	Instrument/Platform	Sample/Tissue	Accession (NCBI)	Accession (CNSA)
CycloneSEQ long-read	CycloneSEQ	muscle and lung	SAMN44670087	CNS1198625
DNBSEQ short-read	DNBSEQ	muscle of neck	SRR31361869/SRR31361868	CNS1065816
		muscle of tail	SRR31361871/SRR31361870	CNS1065815
CycloneSEQ	CycloneSEQ	liver	SRR31361885	CNS1198626
Hi-C data	DNBSEQ	muscle (Hi-C_1 data)	SRR31361875/SRR31361876	CNS1065813
		muscle (Hi-C_2 data)	SRR31361872/SRR31361873	CNS1065814
CycloneSEQ long-read RNA-seq data	CycloneSEQ	eye	SAMN44670089	CNS1198628
		kidney	SAMN44670090	CNS1198630
		live	SAMN44670091	CNS1198631
		lung	SAMN44670092	CNS1198632
		muscle	SAMN44670093	CNS1198633
		testis	SAMN44670094	CNS1198634
		brain	SRR31361862	CNS1198627
		heart	SRR31361861	CNS1198629
DNBSEQ short-read RNA-seq data	DNBSEQ	brain	SRR31361884	CNS1065805
		eye	SRR31361883	CNS1065806
		heart	SRR31361882	CNS1065807
		kidney	SRR31361881	CNS1065808
		liver	SRR31361880	CNS1065809
		lung	SRR31361879	CNS1065810
		muscle	SRR31361878	CNS1065811
		testis	SRR31361877	CNS1065812

2-21 Lines 681 "homologous" more details.

Response

We thank you for highlighting the need for methodological precision. The revised manuscript (Methods – Repetitive element annotation, **Lines 698-702**) now provides comprehensive details on the homology-based annotation pipeline.

2-22 Lines 709 "winnowmap -u b -K 4G -G 135k -ax splice ref fastq." No italic

Response

Thanks. We have revised it accordingly (**Line 729**).

2-23 Lines 900 PRJNAxxxxxx

Response

Thanks. We have replaced the placeholder "PRJNAxxxxxx" with **PRJNA1184386** in **Line 923**.

References:

1. Quinn, A.E., et al., *Extension, single-locus conversion and physical mapping of sex chromosome sequences identify the Z microchromosome and pseudo-autosomal region in a dragon lizard, Pogona vitticeps.* Heredity, 2010. **104**(4): p. 410-417.
2. Long, X., et al., *Independent evolution of sex chromosomes and male pregnancy–related genes in two seahorse species.* Molecular Biology and Evolution, 2023. **40**(1): p. msac279.
3. Purcell, S., et al., *PLINK: a tool set for whole-genome association and population-based linkage analyses.* The American journal of human genetics, 2007. **81**(3): p. 559-575.
4. Garrison, E. and G. Marth, *Haplotype-based variant detection from short-read sequencing.* arXiv preprint arXiv:1207.3907, 2012.
5. Manichaikul, A., et al., *Robust relationship inference in genome-wide association studies.* Bioinformatics, 2010. **26**(22): p. 2867-2873.
6. Zhang, J.-Y., et al., *A single-molecule nanopore sequencing platform.* bioRxiv, 2024: p. 2024.08. 19.608720.
7. Bresler, M.a., et al., *Telescoper: de novo assembly of highly repetitive regions.* Bioinformatics, 2012. **28**(18): p. i311-i317.
8. Pinto, B.J., et al., *A lizard is never late: squamate genomics as a recent catalyst for understanding sex chromosome and microchromosome evolution.* Journal of Heredity, 2023. **114**(5): p. 445-458.

9. Waters, P.D., et al., *Microchromosomes are building blocks of bird, reptile, and mammal chromosomes.* Proceedings of the National Academy of Sciences, 2021. **118**(45): p. e2112494118.
10. Lisachov, A.P. and P.M. Borodin, *Microchromosome polymorphism in the sand lizard, Lacerta agilis Linnaeus, 1758 (Reptilia, Squamata).* Comparative Cytogenetics, 2016. **10**(3): p. 387.
11. Whiteley, S.L., et al., *Two transcriptionally distinct pathways drive female development in a reptile with both genetic and temperature dependent sex determination.* PLoS Genetics, 2021. **17**(4): p. e1009465.
12. Quinn, A.E., et al., *Temperature sex reversal implies sex gene dosage in a reptile.* Science, 2007. **316**(5823): p. 411-411.

Manuscript

Click here to access/download;Manuscript;Pogona vitticeps manuscript.clean.20250415.docx

A near-complete genome assembly of the bearded dragon *Pogona vitticeps* provides insights into the origin of *Pogona* sex chromosomes

Qunfei Guo[1,9], Youliang Pan[1,9], Wei Dai[1,9], Fei Guo[2], Tao Zeng[2], Wanyi Chen[2,3], Yaping Mi[1,4], Yanshu Zhang[1,5], Shuaizhen Shi[1,6], Wei Jiang[2], Huimin Cai[7], Beiying Wu[1,8], Yang Zhou[2], Ying Wang[1], Chentao Yang[2], Xiao Shi[2], Xu Yan[2], Junyi Chen[2], Chongyang Cai[2], Jingnan Yang[2], Xun Xu[2], Ying Gu[2*], Yuliang Dong[2*], Qiye Li[1,2*]

[1]BGI Research, Wuhan 430074, China

[2]BGI Research, Shenzhen 518083, China

[3]School of Life Sciences, Southwest University, Chongqing 400715, China

[4]College of Life Sciences, Northwest University, Xi'an 710069, China

[5]College of Future Technology, University of Chinese Academy of Sciences, Beijing 100049, China

[6]College of Life Sciences, University of Chinese Academy of Sciences, Beijing 100049, China

[7]Women's Hospital, School of Medicine, Zhejiang University, Hangzhou, 310006, China

[8]School of Biology and Biological Engineering, South China University of Technology, 510006 Guangzhou, China

[9]These authors contributed equally

[*]Corresponding authors:

guying@genomics.cn (Y.G.); dongyuliang@genomics.cn (Y.D.); liqiye@genomics.cn (Q.L.)

Abstract

Background: Sex determination in reptiles is a complex process, ranging from complete genetic determination to environmentally determined. The central bearded dragon *Pogona vitticeps* exhibits both genetic and temperature-dependent sex determination, however, the features and origin of its sex chromosome remain unclear due to the absence of a chromosome-level genome. Here, we generated a near complete genome of a ZZ male *P. vitticeps* and first deciphered the structure of its Z chromosome.

Results: Using CycloneSEQ and DNBSEQ sequencing technologies, we generated a chromosome-scale genome for a ZZ male *P. vitticeps*. The ~1.8 Gb assembly achieved a 5,700-fold improvement in contiguity (N50 = 202.5 Mb vs. 35.5 kb) and complete chromosome anchoring (16 vs. 13,749 scaffolds) compared to previous reference genome (pvi1.1). Long-read RNA sequencing enhanced genome annotation, expanding the lncRNA catalog. We fully characterized the Z chromosome, revealing over 80% as a pseudo-autosomal region (PAR). The small sexually differentiated region (SDR) is transposon-rich and contains key male development genes, including *AMH*, *AMHR2*, and *BMPR1A*. By tracing SDR gene origin and expression, we proposed a model for *P. vitticeps* sex chromosome origin, identifying the Z-linked *AMH* as the master sex-determining gene.

Conclusions: By a near complete genome assembly, we characterized the PAR and SDR region of the Z chromosome in *P. vitticeps*, identifies *AMH* as a pivotal sex-determining gene, and proposes a new model for sex chromosome origin of *P. vitticeps*. This work advances understanding of sex determination in *P. vitticeps* and provides a molecular foundation for studying sex determination in reptiles.

Introduction

Sex determination is a fundamental process in vertebrate evolutionary biology, influencing reproductive strategies, population dynamics, and genetic diversity [1]. Among reptiles, the mechanisms of sex determination are diverse, ranging from genetic sex determination (GSD) to

environmental factors such as temperature-dependent sex determination (TSD) [2-5]. The central bearded dragon *Pogona vitticeps* has gained recognition as a pivotal reptilian model in biological research due to its accessibility, low maintenance requirements, and particularly reproductive biology. This species exhibits a unique ZZ/ZW GSD system and is additionally influenced by TSD, demonstrating sexual fate plasticity in which ZW embryos develop as females and ZZ embryos as males under standard conditions, while high incubation temperatures induce functional female sex reversal in genetically male (ZZ) individuals [6]. This temperature-dependent plasticity suggests that sexual fate is ultimately determined by a dosage-sensitive gene on the Z chromosome [7], making the complete Z chromosome sequence particularly crucial for deciphering the sex-determining cascade in this species.

While many studies involving *P. vitticeps* in the past decade tightly rely on its genome assembly and annotation, the current reference genome is still quite fragmented. This reference genome was constructed with a wild-caught ZZ male individual, based on Illumina short-read sequencing data generated from gradient libraries with insert size ranging from 250 bp to 40 kb [8]. Due to the limitation of short-read sequencing, the contig N50 of this assembly version is merely 35.5 kb in length, lag far behind the common standard of a reference genome (> 1 Mb) as proposed by the Earth BioGenome Project (EBP) [9] and the Vertebrate Genomes Project (VGP) [10] in recent years. Additionally, *P. vitticeps* possesses microchromosomes [11], which, like those in birds and some reptilian species, present significant assembly challenges due to their high gene density, elevated GC content, and intense inter-chromosomal interaction signals [12]. These characteristics make the assembly of *P. vitticeps* particularly complex. Although subsequent scaffolding efforts anchored ~42 % of the genomic sequences to chromosomes [13], the low anchoring percentage still limits its use in chromosome-scale investigation. For example, the sex chromosomes of *P. vitticeps* are well-known to be heteromorphic based on cytogenetic evidence, implying the existence of sequence divergence between Z and W due to recombination suppression [14]. However, the PAR and SDR of both sex chromosomes remain undefined so far, which in turn hamper the search for the master sex-determining gene.

In this study, we generate a new genome assembly for a ZZ male *P. vitticeps*, via a combination of long- and short-read whole genome sequencing (WGS) as well as long-range sequencing technologies. The new *P. vitticeps* reference genome has a contig N50 of 202.5 Mb, with all contigs

anchored onto 16 chromosomes and only few gaps remained. With this near complete chromosome-scale genome assembly, we fully characterized the Z sex chromosome and demarcated the PAR and SDR on Z, tracked the evolutionary origin and developmental expression of the Z-linked SDR genes, and proposed an alternative model for explaining the origin of sex chromosomes with the Z-linked AMH as the candidate of master sex-determining gene in *P. vitticeps*.

Results

The construction of a near-complete *P. vitticeps* genome

To reduce interference caused by allelic variation, all sequencing data for genome assembly were collected from a single captive-bred individual. The sex of this individual was verified as a ZZ male, as demonstrated by the anatomical presence of testes and the polymerase chain reaction (PCR) examination of sex-linked markers (Supplementary Fig. S1A). High molecular weight (HMW) DNA was extracted from the muscle and lung tissues, which was subjected to WGS with the CycloneSEQ [15] long-read and DNBSEQ short-read technologies, respectively (Supplementary Tables S1). In addition, we generated long-range sequencing data from the liver tissue by integrating chromatin conformation capture technique with CycloneSEQ (see Methods for details; hereafter referred as CycloneSEQ based Pore-C). *K*-mer analysis with the short-read data confirmed that *P. vitticeps* has a heterozygous diploid genome (heterozygosity ~1.63%) with a haploid size of ~1.7 Gb [8] (Supplementary Table S4; Supplementary Fig. S1B).

We obtained a total of ~265 Gb CycloneSEQ WGS reads, with over a quarter (~105 Gb) achieving a sequence length longer than 40 kb (Supplementary Table S1). As the 40 kb+ reads alone had already covered the *P. vitticeps* haploid genome for ~62 times, fully satisfying the requirements for long read-based *de novo* assembly, we therefore assembled the 40 kb+ CycloneSEQ WGS reads using NextDenovo [16] in the first step (Fig. 1A; Supplementary Fig. S1C). This resulted in a 1.81 Gb primary assembly with merely 106 contigs. The contig N50 was 60.9 Mb, surpassing the majority of squamate genomes published to date (Supplementary Table S7). Before further scaffolding by long-range data, we removed false duplications in the primary assembly with Purge_Haplotigs [17], and corrected sequence errors with short-read data using NextPolish [18].

After polishing, a 1.80 Gb assembly with 74 contigs were scaffolded by ~33X long-range CycloneSEQ based Pore-C data with YaHS [19]. The order and orientation of each contig was also manually examined in Juicebox [20] to avoid misplacements. The final chromatin contact map clearly sorted all the contigs into 16 chromosomes, including six macrochromosomes and 10 microchromosomes (Fig. 1B).

To interrogate the integrity of chromosome ends, we next examined the presence of vertebrate telomeric repeat units (TRUs), i.e., $(TTAGGG)_n$ [21]. Only eight of the 32 expected telomeres were presented in the initial chromosome-scale *P. vitticeps* assembly (Supplementary Table S9). To resolve this limitation, we developed an in-house pipeline to conduct local telomere assembly with the TRU-containing reads and assign the assembled telomeres to their corresponding chromosome ends (see Methods for details). As a result, 31 of the 32 expected telomeres with a mean copy number of 1661 TRUs were patched to corresponding chromosome ends, except one on chromosome 14 (Fig. 1B).

After telomere repair, the chromosome-scale assembly still contains 69 unclosed gaps. These gaps were subjected to gap-filling with the corrected long reads (generated by NextDenovo during assembly) using TGS-GapCloser [22]. The correctness of each closed gap was further examined by long-read coverage with an in-house pipeline. This led to the solid closure of 41 gaps inside the chromosomes, and made four of the six macrochromosomes (chromosomes 3, 4, 5 and 6) as well as one microchromosome (chromosome 13) achieving telomere-to-telomere (T2T) gapless assembly. The remaining 28 gaps were mainly located in microchromosomes and co-localized with repeat-dense regions (Fig. 1B). Of note, a local region (0.5 to 11 Mb) with a particularly high density of repeats was clearly observed in almost all chromosomes, assumably corresponding to the position of centromeres (Fig. 1B; Supplementary Table S8).

In summary, the final *P. vitticeps* genome assembly (hereafter referred as Pvit2024) was 1.79 Gb in length, with all contigs anchored into 16 chromosomes, a final contig N50 of 202.5 Mb, and the presence of all but one of the 32 telomeres. Although there were still 28 inner gaps remaining unclosed, our Pvit2024 assembly outperformed all other squamate genomes reported so far in terms of continuity (Fig. 1C).

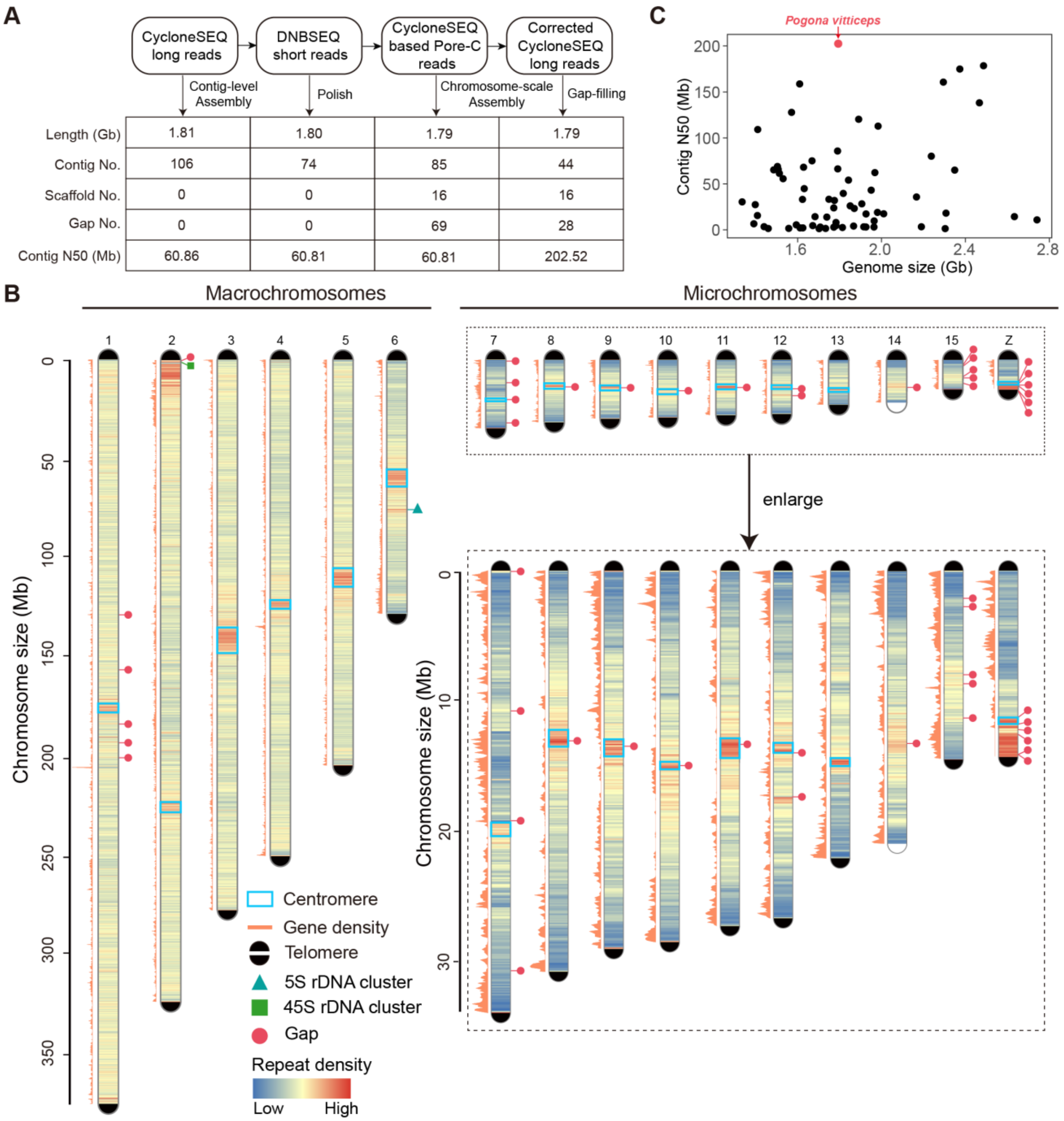

Figure 1: The near-complete genome assembly of *P. vitticeps*. (A) The CycloneSEQ long reads were initially utilized for *de novo* contig assembly. Thereafter, DNBSEQ short reads were employed to polish the assembled contigs. Subsequently, CycloneSEQ based Pore-C reads were implemented for scaffolding the contigs into chromosomal sequences. Finally, gaps were filled with corrected CycloneSEQ long reads. The table presents the genomic features for each step. (B) An overview of the genome features. The distribution of all repeat density and gene density were calculated in 100 kb windows. All the gaps in Pvit2024 were show in read circles. All chromosomes, except for chromosomes 14 and 15, had been identified with centromeric regions marked by light

blue boxes. (C) The comparison of genome size and contig N50 between Pvit2024 and 66 publicly available squamate genomes assembled by long-read data.

Quality validation of the genome assembly

To assess the completeness of Pvit2024, we first aligned three sets of WGS reads to the Pvit2024 assembly, that is, the CycloneSEQ long reads and DNBSEQ short reads collected in this study as well as a set of Illumina short reads generated from another ZZ male individual that were not used for our genome assembly. All the three read sets revealed a mapping rate greater than 98% (Supplementary Table S5), and the aligned reads showed uniform coverage across the genome with only a few exceptions in repeat-dense regions (Fig. 2A). Secondly, we conducted Benchmarking Universal Single-Copy Orthologs (BUSCO) [23] and Compleasm [24] assessments for Pvit2024 with the Sauropsida_odb10 dataset (n = 7,480 genes), which represents conserved single-copy orthologs broadly present across Sauropsida (including birds, squamates, and turtles). The BUSCO and Compleasm complete score were 97.6% and 98.63%, respectively, comparable to or even higher than other squamate genomes assembled by long-read data (Supplementary Fig. S2A; Supplementary Table S7). The consensus quality value (QV) of Pvit2024 as estimated by short-read data was 36.4, corresponding to a single-base error rate of merely 0.0229%.

Next, we assessed the accuracy of chromosomal sorting and scaffolding by different strategies. From a technical perspective, two traditional Hi-C libraries were constructed and sequenced with the DNBSEQ technology to serve as independent validations of the long-range information provided by CycloneSEQ based Pore-C. Both these two additional Hi-C contact maps consistently supported the scaffolding result of Pvit2024 (Fig. 2B). Besides, we aligned those chromosome-anchored sequences from Deakin et al. [13] to Pvit2024 and observed high consistency (i.e., sequences were mapped to expected position in expected order; Fig. 2C). Furthermore, Deakin et al. have also located the genomic regions harboring the centromeres in chromosomes 1, 2, 4 and 6 [13]. All these four centromere-containing regions showed perfect overlaps with the putative centromeric regions identified in Pvit2024 (Fig. 2C), supporting the feasibility of centromere positioning based on local repeat abundance in this species. From a biological perspective, the concurrent presence of macrochromosomes and microchromosomes is the hallmark of many vertebrate genomes [12, 25-28]. According to the lengths of the assembled chromosomes, we could

clearly define six of the sixteen Pvit2024 chromosomes as macrochromosomes and the remaining 10 as microchromosomes, in line with the reported karyotype of a male *P. vitticeps* (Supplementary Fig. S2C) [29]. In addition to chromosomal lengths, we also observed other recognized features that separate microchromosomes from macrochromosomes, including higher gene density, higher GC content, lower repeat content, and more frequent inter-chromosomal interaction (Fig. 2D, E; Supplementary Fig. S2B) [30, 31]. These multiple lines of evidence together highlight the reliability of the Pvit2024 chromosomal assembly.

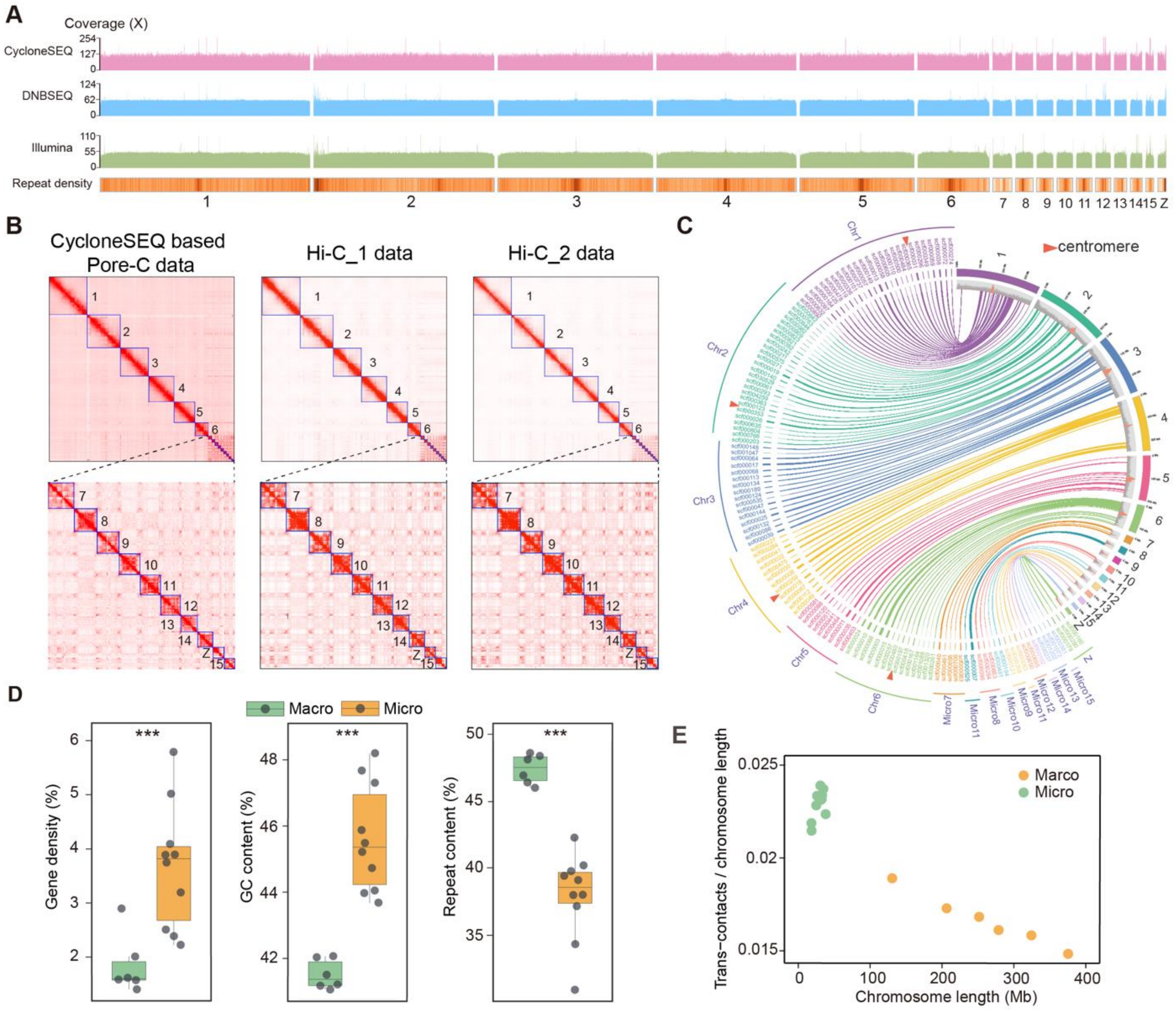

Figure 2: Quality validation of the genome assembly. (A**)** Genome-wide coverage based on various data was computed over 10 kb windows. Numbers on the y axis of each coverage track indicate the double average and average values of sequencing coverage for each track. Genomic data types are color coded. Pink: CycloneSEQ long-read WGS data. Light blue: DNBSEQ short-read WGS data. Light green: Illumina short-read WGS data.

Additional tracks in the bottom panel show repeat density of Pvit2024. (B**)** The heatmap for one CycloneSEQ based Pore-C library and two traditional Hi-C libraries chromosomes contact matrices. (C) The circos plot illustrates genomic synteny, with Pvit2024 chromosomes on the right and scaffolds anchored to chromosomes by BACs from Deakin et al. The second right circle shows repeat density, red indicating centromeres. The red triangle marks the centromere position as determined by Deakin et al. (D) The comparison of gene density, GC content, and repeat content between macrochromosomes and microchromosomes. Asterisks indicate the significance of differences: *** represents $p < 0.001$. (E) Trans-contacts scaled by chromosome size for each chromosome were calculated by ~33X CycloneSEQ based Pore-C data. Each dot represents one chromosome (yellow for macrochromosomes, green for microchromosomes).

The new reference genome recovers ~120 Mb missing sequences with numerous genes and regulatory elements

Compared with the previous reference genome (GCF_900067755.1/ pvi1.1) [8] (hereafter referred as Pvit2015), the contiguity of Pvit2024 was increased by over 5700 folds in terms of contig N50 (202.5 Mb vs 35.5 kb), and the number of scaffolds was remarkably reduced from 13,749 to 16 (Fig. 3A). Additionally, the genomic completeness as assessed by read alignments and BUSCO and Compleasm analyses all showed improvements (Fig. 3B, C).

To further uncover the differences between the two genome assemblies, we conducted reciprocal whole-genome alignment to identify missing sequences in one assembly relative to the other. Briefly, we defined genomic sequences in one assembly with no alignment to the other one by Winnowmap2 [32] and Minimap2 [33] as missing sequences in the former one. In this way, 124 Mb of the Pvit2024 sequences were detected as missing in Pvit2015, accounting for 6.9% of the Pvit2024 assembly size and affecting all the 16 chromosomes in a different degree (Fig. 3D, E; Supplementary Fig. S3A). Read coverages of these Pvit2015-missing regions were uniform and close to that of genome average, indicating that they are not assembly errors (Fig. 3F; Supplementary Fig. S3H). Conversely, only 36 Mb of Pvit2015 sequences were not aligned to Pvit2024, and the read coverage of these regions were quite low, suggesting that these Pvit2015-speficic sequences likely resulted from assembly errors (Fig. 3F).

We next focused on the annotation of the 124 Mb sequences that were missing in prior assembly, especially for the possibility of carrying functional genes or regulatory elements. The missing sequences revealed higher GC content than genome average, and most missing sequences (~73.6%)

was annotated as repetitive elements in Pvit2024, reminding the advantage of the long-read sequencing to sequence through high GC content regions and repeat regions (Fig. 3G, H; Supplementary Fig. S3B) [34]. Nevertheless, it is noteworthy that these Pvit2015-missing sequences also harbored the intact copies of up to 576 protein-coding and 312 lncRNA genes, namely, they are completely absent in prior assembly (Supplementary Fig. S3C, D). A representative example was the *Vmn2r65* (Vomeronasal 2 Receptor) gene family, of which 25 copies were located on a tandem array in chromosome 2 of Pvit2024 (Fig. 3I); only seven copies of *Vmn2r65* were identified and distributed on 6 distinct scaffolds in Pvit2015. In addition, the missing sequences in Pvit2015 also led to partial absence of one or more exons for up to 1,319 protein-coding and 1,651 lncRNA genes (Supplementary Fig. S3C, D), which may affect the accuracy of expression quantification by RNA sequencing. In terms of regulatory elements, we first examined the completeness of gene promoters, the region upstream of genes where the RNA polymerase binds to initiate transcription [35] and found that 2,504 (11.5%) protein-coding and 1,281 (8.1%) lncRNA genes contain >500 bp missing sequences in their putative promoter regions, respectively. We then examined the CpG islands, which are widespread in vertebrate genomes and play important roles in transcriptional regulation [36]. Consistent with the difficulty of short-read technology to sequence through high GC regions, we observed that 32,864 of the annotated 216,126 (15.2%) CpG islands were completely missing and 7,361 (3.4%) were partial missing in prior Pvit2015 assembly (Supplementary Fig. S3E, F, G).

Taken together, our genome-wide comparative analysis indicated a notable proportion (6.9%) of genomic sequences missing in the prior *P. vitticeps* reference genome. These missing sequences tend to be GC-rich and repeat-rich, and more importantly, they contain numerous genes and regulatory regions that are not accessible based on prior reference genome.

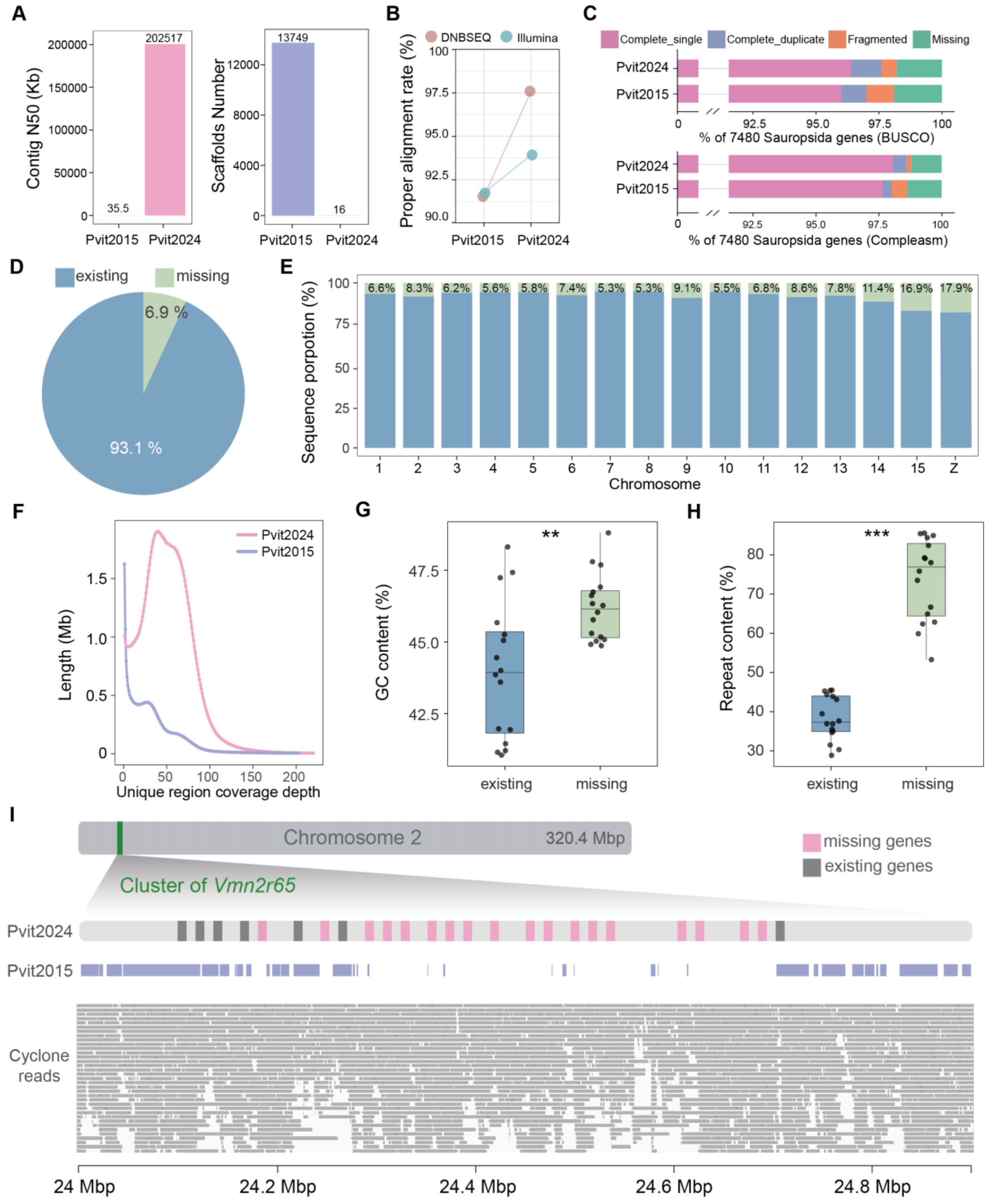

Figure 3: Comparison between Pvit2015 and Pvit2024. (A) The comparison of contig N50 and scaffold number. (B) The proper alignment rate of DNBSEQ and Illumina WGS data. (C) Gene completeness assessment of Pvit2015 and Pvit2024 based on BUSCO and Compleasm analyses. (D) Proportion of sequences existing and

missing in the Pvit2015 assembly compared to the Pvit2024. (E) The missing rate of Pvit2015 assembly across chromosomes in Pvit2024 is depicted, with blue and green bars representing the existing and missing ratios of Pvit2015, respectively. (F) Base coverage depth distribution of unique sequences in Pvit2015 and Pvit2024, the DNBSEQ genomics data were used. (G and H) Comparison of GC content (G) and repeat element content (H) between existing and missing sequences in Pvit2015 across each chromosome. T-test analysis of variance; *$P<0.05$, **$P<0.01$, ***$P<0.001$. (I) Distribution of *Vmn2r65* gene family along the chromosome, determined using ChromoMap of R packages. The blue block show sequence of Pvit2015 that can be aligned back to Pvit2024. The grey strip of bottom shows the coverage of CycloneSEQ reads.

Improving genome annotation by long-read RNA sequencing

Besides genome assembly, comprehensive and accurate annotation of gene model is the necessity to realize the value of a reference genome [37]. To facilitate a comprehensive gene annotation for Pvit2024, we performed high-depth long-read RNA sequencing (RNA-seq) for eight different tissues (brain, eye, heart, kidney, liver, lung, muscle, and testis) with the CycloneSEQ technology (Supplementary Table S2). In the meanwhile, we also generated paired-end short-read RNA-seq data for these eight tissues with the DNBSEQ technology (Supplementary Table S3). All the long-read and short-read RNA-seq data were generated from the same individual as that used for genome sequencing in this study.

With the abundant RNA-seq data, we first conducted protein-coding gene annotation with a combination of transcriptomic, homologous and *ab initio* prediction evidence, and obtained a total of 21,783 non-redundant protein-coding gene models (Supplementary Table S11). Up to 92% (20,002) of the protein-coding loci are supported by RNA-seq signal (TPM > 5) in at least one tissue. BUSCO assessment with the Sauropsida conserved genes revealed a complete score of 97.5%, consistent with that estimated for Pvit2024 genome assembly and slightly higher than the commonly used NCBI annotation (96.6%) and Ensembl annotation (91.7%) based on Pvit2015 (Fig. 4A).

Then, we leveraged the long-read RNA-seq data to refine isoform and UTR annotations for the protein-coding genes. After strict filtering steps to exclude artificial and low-quality sequences, we finally assigned 53,272 transcripts to 21,783 protein-coding loci, with a mean of 2.5 isoforms detected per gene. Up to 97% of the splicing junctions derived from the assigned transcripts were

also supported by short-read alignments, further supporting the reliability of these transcript models. In addition, all the major types of alternative splicing events could be identified in all the eight tissues, with exon skipping and alternative first exon usage being the most dominant events (Fig. 4B). By discriminating coding regions from non-coding parts, we could identify the UTRs for most transcripts, with 17,110 (31.1%) having a 5'-UTR longer than 30 bp and 19,161 (34.8%) having a 3'-UTR longer 50 bp. Of note, while the lengths of coding regions were comparable between our and the NCBI/Ensembl annotations, the 5'-UTRs and 3'-UTRs in our annotation were significantly longer (Fig. 4C), corroborating a more accurate definition of transcription boundaries owing to the assistance of long-read RNA-seq. To evaluate the effect of the new annotation on future RNA-seq studies, we calculated the read mapping rate and number of expressed genes with a bulk and a single-cell RNA-seq dataset that were not used for gene annotation in this study. Both datasets revealed a significant improvement on both metrics, especially for the single-cell dataset (Fig. 4D, E).

Long non-coding RNAs (lncRNAs) are another important class of RNA molecules that engage in numerous biological processes [38], yet the lncRNA repertoire is understudied in squamate reptiles. By identifying transcripts that are longer than 200 nt and lack coding potential, we annotated 13,269 high-confidence lncRNAs in Pvit2024 (Fig. 4F). When compared with the protein-coding genes, the *P. vitticeps* lncRNAs were generally shorter in length, have fewer exons, and were expressed at lower levels (Fig. 4G, H, I), consistent with previous observations in other organisms [39]. Interestingly, we found that many lncRNAs were expressed specifically in one or a few tissues, especially the testis (Supplementary Fig. S4). The large amount of tissue-specific lncRNAs in uncovered in *P. vitticeps* implied their potential importance in squamate biology that worth further attention.

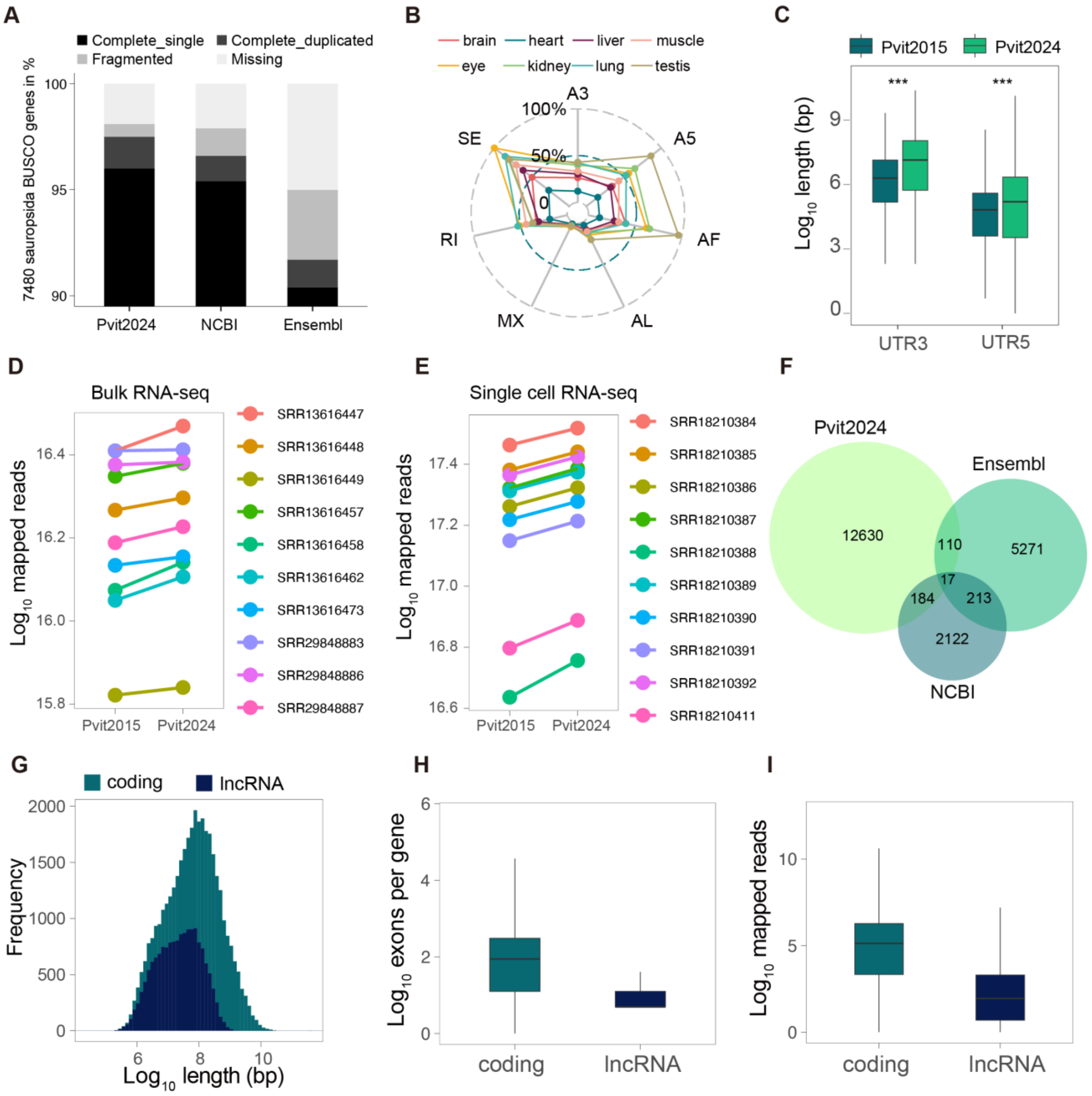

Figure 4: Enhancement of the genome annotation for *P. vitticeps*. (A) Completeness of Sauropsida BUSCO genes in annotations produced by Pvit2024, NCBI (Pvit2015), and Ensembl (Pvit2015). (B) Radar chart showing relative splicing abundance of eight tissues for each event type (SE–skipping exon; RI–retained intron; MX-mutually exclusive exons; A5–alternative 5' splice site; A3–alternative 3' splice site; AF-alternative first exons; AL- alternative last exons). Normalization here employs the maximum abundance value of splicing events as the denominator. (C) UTRs length distributions between the Pvit2024 and NCBI, Pvit2024 has longer than NCBI. T-test analysis of variance; **P*<0.05, ***P*<0.01, ****P*<0.001. (D and E) Connected dot-line showing the variance of mapping reads in Pvit2024 and NCBI RefSeq annotations for bulk (D) and single-cell (E)

transcriptomes. (F) Venn diagram showing lncRNA counts for Pvit2024, Ensembl and NCBI RefSeq annotations. (G) Histogram shows the distribution of gene length (exon regions) for coding gene and lncRNA. (H) Distribution of the exon counts per gene in coding gene and lncRNA. (I) Distribution of the read counts for transcriptomes in coding genes and lncRNAs.

The demarcation of PAR and SDR along the *P. vitticeps* Z sex chromosome

The identity of *P. vitticeps* Z chromosome was ascertained by mapping known Z-linked scaffolds identified by Deakin et al. [13] to our Pvit2024 assembly. Specifically, all the Z-linked scaffolds were aligned to a ~14.3 Mb microchromosome in expected order, spanning a continuous region of ~8.2 Mb on the first half of this microchromosome (Fig. 5A; Supplementary Fig. S5A). The uncovered portion comprised ~6.1 Mb genomic sequences that were newly identified to be Z-linked, accounting for ~43% of the whole Z chromosome (Fig. 5A). These newly identified Z-linked sequences harbored 47 protein-coding genes and 65 lncRNA genes that are either missing or unknown to be Z-linked in prior Pvit2015 assembly (Supplementary Table S12). The overall Z chromosome showed an uneven distribution of genomic elements, with an increase of repeat content accompanied by depletion of protein-coding genes toward the chromosomal end that enriched newly anchored sequences (Fig. 5B).

We next attempted to demarcate the SDR, where Z-W sequence divergence is accumulated, and the PAR, where Z and W remain identical, by comparing WGS read coverage between ZW and ZZ individuals. In principle, the PAR is expected to display comparable read coverage in both genders, while a SDR would exhibit halved coverage in ZW females relative to ZZ males [40]. To apply this principle, we analyzed WGS data from twelve ZW females and eight ZZ males (Supplementary Table S13). Of these, five individuals were sourced from published studies, and the remaining fifteen samples were newly collected and validated for gender using sex-specific markers (Fig. S5C). To exclude potential confounding effects of relatedness, PCA and kinship analyses confirmed that all individuals were sourced from different lineages (Fig. S5D, E). Based on this curated dataset, up to 83% (~11.9 Mb) of the Z chromosome displayed a comparable read coverage in both genders, suggesting that the vast majority of Z remains as PAR that persists recombination. Nevertheless, there was a ~250 kb region exhibiting roughly halved coverage in ZW females relative to ZZ males (Fig. 5C), fulfilling the expectation of an evolutionarily old stratum (hereafter referred as S0) where Z and W have been substantially diverged. For the region

ranging from the downstream of S0 to the chromosome end, the read coverage in ZW females was even higher than that in ZZ males (Fig. 5C). However, this region lacked protein-coding genes and instead was occupied exclusively by repetitive elements (Fig. 5B). We speculated that this unusual coverage pattern was attributed to the much higher abundance of repeats accumulated in the W counterpart of this region, as previous cytogenetic studies have suggested that the short arm of W is much longer than Z [11]. If so, this downstream region of S0 (~2.18 Mb in length) might also represent a stratum that was fully degenerated due to transposon invasion, and therefore, we tentatively grouped it into SDR in this study.

Together, we could conservatively define a large, continuous PAR and a small SDR on the *P. vitticeps* Z chromosome, with the PAR-SDR boundary delimited at ~12 Mb in coordinate (Fig. 5A). Compared with PAR, SDR was apparently characterized by a remarkably high repeat content (>80%) (Fig. 5D). However, the repetitive element composition of SDR was generally similar to those of PAR and other chromosomes, although several classes of transposons such as LTR/Gypsy, LTR/ERV1 and LINE/L1 were relatively more abundant in SDR (Fig. 5E).

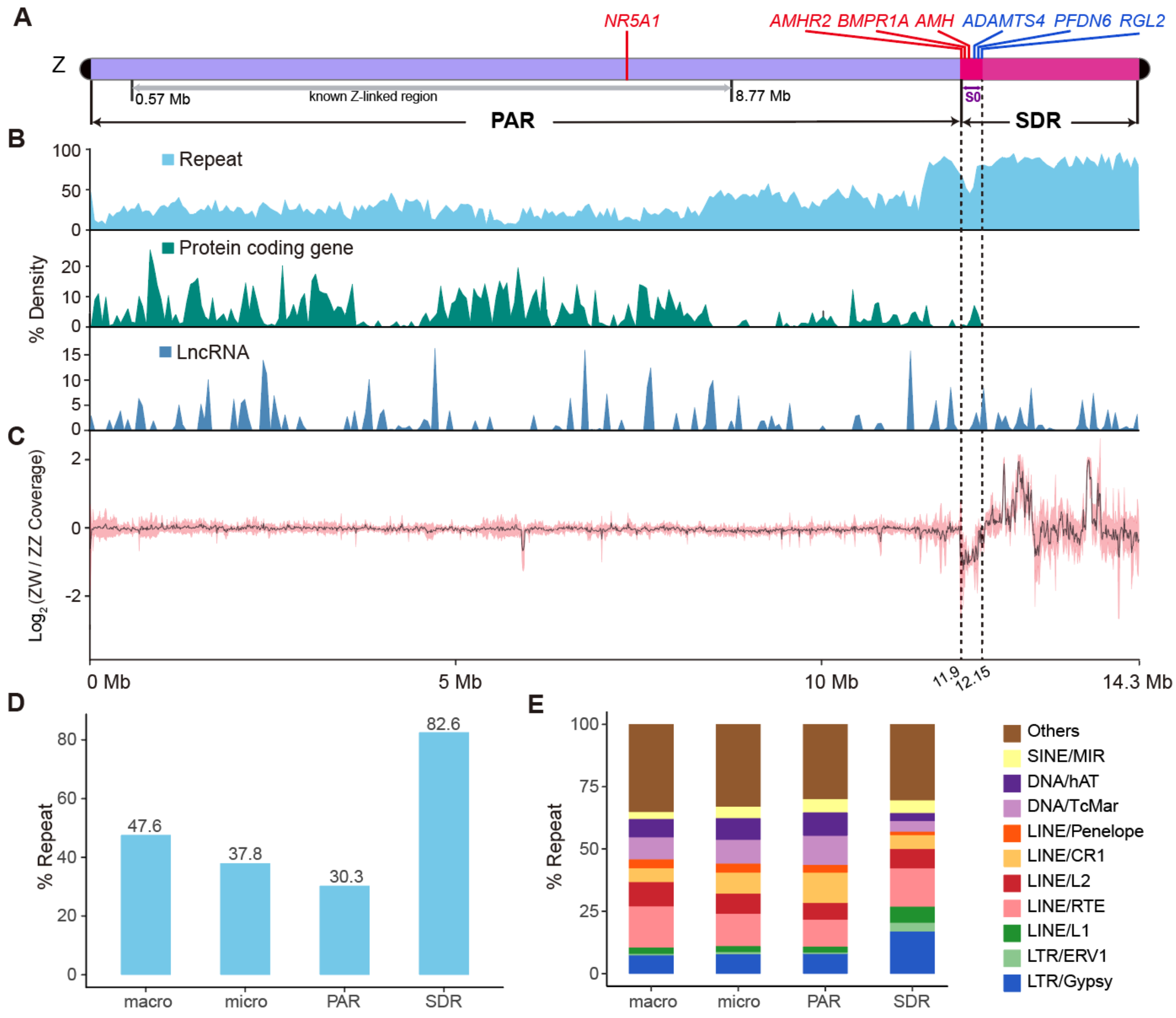

Figure 5: Z sex chromosome identification and the composition of PAR and SDR. (A) The Z chromosome can be divided into two regions: PAR and SDR. Sex-determination genes are marked in red, while pseudogenes are in bule. (B) Repeat density, protein coding gene density, and lncRNA density are calculated in 50 kb windows. (C) The distribution shows the ratio of ZW to ZZ read coverage in 10 kb windows. The depth (black line) is calculated as the average ratio of read coverage for ZW females to ZZ males between each pair of ZW females and ZZ males, based on the WGS data from twelve ZW females and eight ZZ males. The pink areas are the confidence intervals for each window. (D) Repeat content for macrochromosomes, microchromosomes, PAR region, and SDR region. (E) Relative proportions for ten most abundant transposons families and other elements.

The evolutionary origin and developmental expression of the Z-linked SDR genes

Although being predominantly occupied by repetitive elements, we found that the *P. vitticeps* SDR carried three intact protein-coding genes (*AMHR2*, *BMPR1A* and *AMH*) and three pseudogenes

(*ADAMTS4*, *PFDN6* and *RGL2*), of which all located in a ~150 kb region within S0 (Fig. 5A). Notably, the three intact genes are all well-known players in driving sexual differentiation, namely, *AMH* (anti-Mullerian hormone) [41] and its two receptors, *AMHR2* (anti-Mullerian hormone receptor type 2) [42] and *BMPR1A* (bone morphogenetic protein receptor type 1A) [43]. It is also noteworthy that each of these six SDR genes had a counterpart (i.e., paralog) on autosomes, while all other investigated lizards encoded only one copy of each in their genomes (Fig. 6A), indicating that lineage-specific gene duplication events occurred in *P. vitticeps* (and probably in other closely related agamid lizards as well). This was also supported by gene phylogenetic analyses, which preferentially clustered the *P. vitticeps* paralogs together among the proteins from multiple species (Fig. 6B, Supplementary Fig. S6A). In contrast, while the PAR maintained up to 334 protein-coding genes, only 21 of them (6.3%) had paralogs in autosomes. Hereafter, we designated the six SDR genes as *AMHR2-Z*, *BMPR1A-Z*, *AMH-Z*, *ADAMTS4-Z*, *PFDN6-Z* and *RGL2-Z*, and their autosomal counterparts as *AMHR2-A*, *BMPR1A-A*, *AMH-A*, *ADAMTS4-A*, *PFDN6-A* and *RGL2-A*, respectively (Fig. 6C).

We next asked, for these six paralog pairs in *P. vitticeps*, whether the SDR or the autosomal copy was the original copy that gave rise to the other one. By examining gene synteny information across species, we found that none of the SDR copies could be deemed to be original, namely, they all arose from the duplication of their autosomal counterparts (Fig. 6D, Supplementary Fig. S6B). But it is noted that *PFDN6* and *RGL2* were probably duplicated as a whole, because their gene order as well as the transcriptional direction in SDR maintained the same as their original copies in chromosome 2 (Fig. 6C; Supplementary Fig. S6B). We then estimated the synonymous nucleotide substitution rate (Ks) between the paralog pairs to date the order of their translocation into SDR. As expected, the pseudogenes displayed the largest Ks, probably due to relaxed selection. But it is interesting that, among the three intact genes in SDR, *AMH* displayed the largest Ks, suggesting that *AMH* is likely among the earliest ones to be integrated into the SDR (Fig. 6D).

The master sex-determining gene that initiates sex differentiation remains unknown in *P. vitticeps*, yet it is expected to be a gene that locates in SDR and reveals differential expression between ZZ male and ZW female at an early stage of gonad differentiation. Therefore, we examined the expression dynamics of the SDR genes along gonadal development with the RNA-seq data from Wagner et al. [44]. This dataset comprised gonadal transcriptomes of both genders collected at

three embryonic developmental stages (stage 6/7, stage 12 and 16). In *P. vitticeps*, stage 6/7 represents the earliest stage at which a consolidated gonad is recognizable and begins to differentiate, stage 12 represents an early stage of differentiation, while the gonads at stage 16 have been fully differentiated [7, 44]. Of note, all the three intact SDR genes (*AMH-Z*, *AMHR2-Z* and *BMPR1A-Z*) were identified as differentially expressed genes (DEGs) between genders at one or more developmental stages (DESeq2 FDR < 0.05; Fig. 6F), while none of the pseudogenes and lncRNAs in SDR were significant DEGs (Supplementary Fig. S6C). In addition, we found that *AMH-Z* (FDR = 0.00005; FC = 5.54) and its potential receptor *AMHR2-Z* (FDR = 0.05; FC = 2.22) manifested a significant male-biased expression pattern at stage 6/7, the earliest developmental stage examined. Moreover, *AMH-Z* maintained and even amplified the differential expression pattern throughout gonadal differentiation, reinforcing its central role in driving male development in vertebrates. Together with its early integration into SDR, *AHM-Z* could be considered as a strong candidate of the master sex-determining gene in *P. vitticeps*.

Meanwhile, we also examined the expression of the autosomal paralogs of the SDR genes, to explore whether the expression pattern has been diverged after gene duplication. Interestingly, *AMH-A* displayed almost the same expression pattern as *AMH-Z*, maintaining a high expression level in males while repressing its expression in females throughout embryonic gonadal development (Fig. 6F). However, the expression dynamics of *AMHR2-A* and *BMPR1A-A* were apparently diverged from that of their SDR counterparts. Specifically, while *AMHR2-Z* displayed transient male-biased expression at stage 6/7, *AMHR2-A* was not differentially expressed at stage 6/7 but turned to be significantly female-biased at stages 12 and 16 due to its upregulation in ZW females. In terms of *BMPR1A*, the expression of *BMPR1A-Z* was a bit male-biased at stage 6/7, but the following sharp decrease of *BMPR1A-Z* expression in ZZ males made it turn to be significantly female-biased at later stages; however, *BMPR1A-A* did not show significant sex-biased expression throughout development, although a slight trend of male-biased expression was observed (Fig. 6F).

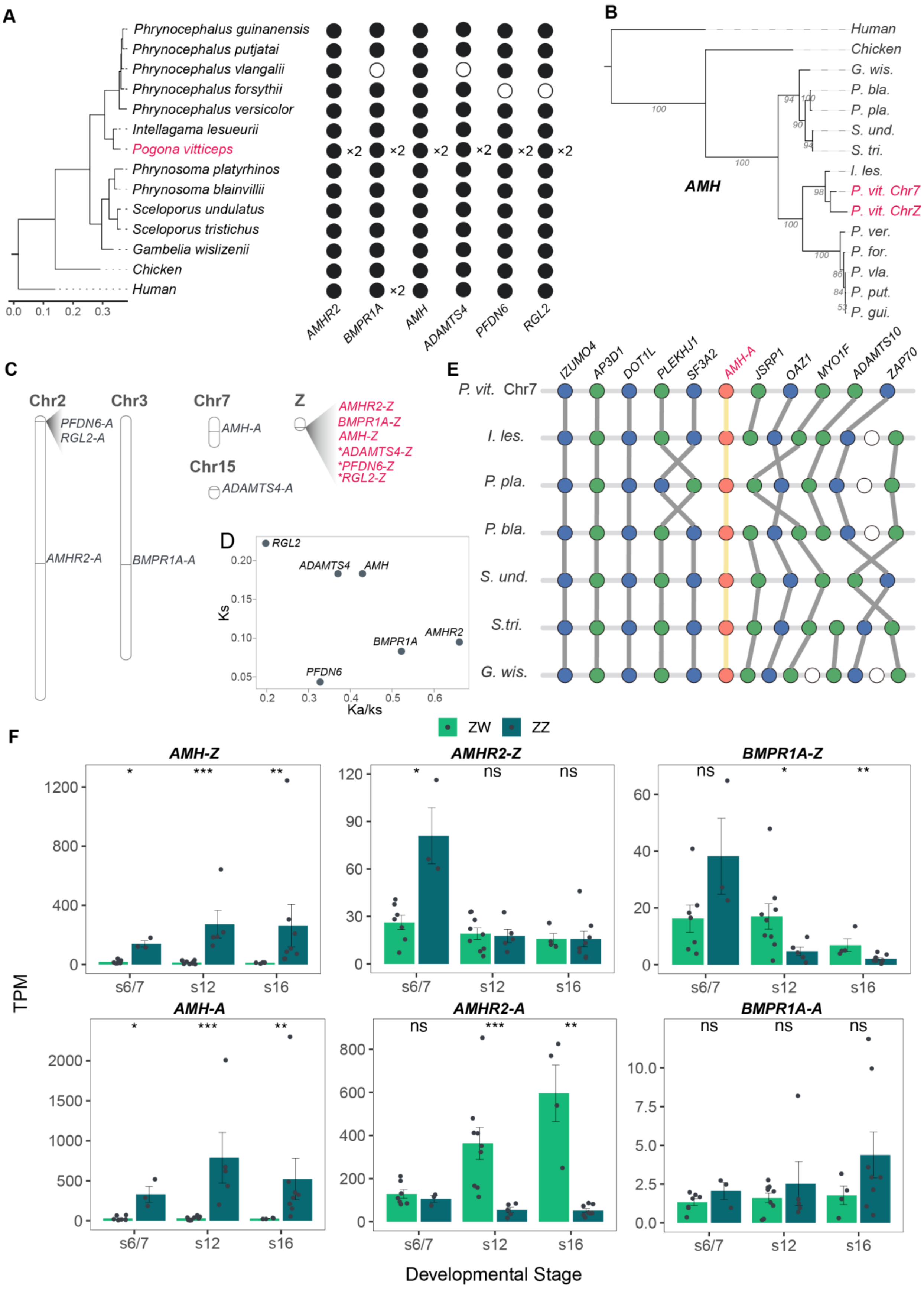

A
Phrynocephalus guinanensis
Phrynocephalus putjatai
Phrynocephalus vlangalii
Phrynocephalus forsythii
Phrynocephalus versicolor
Intellagama lesueurii
Pogona vitticeps
Phrynosoma platyrhinos
Phrynosoma blainvillii
Sceloporus undulatus
Sceloporus tristichus
Gambelia wislizenii
Chicken
Human
AMHR2
BMPR1A
AMH
ADAMTS4
PFDN6
RGL2
B
AMH
C
Chr2
Chr3
Chr7
Z
Chr15
PFDN6-A
RGL2-A
AMHR2-A
BMPR1A-A
AMH-A
ADAMTS4-A
AMHR2-Z
BMPR1A-Z
AMH-Z
*ADAMTS4-Z
*PFDN6-Z
*RGL2-Z
D
Ks
Ka/ks
E
P. vit. Chr7
I. les.
P. pla.
P. bla.
S. und.
S.tri.
G. wis.
IZUMO4
AP3D1
DOT1L
PLEKHJ1
SF3A2
AMH-A
JSRP1
OAZ1
MYO1F
ADAMTS10
ZAP70
F
ZW
ZZ
AMH-Z
AMHR2-Z
BMPR1A-Z
AMH-A
AMHR2-A
BMPR1A-A
TPM
Developmental Stage

Figure 6: Z-linked SDR genes. (A) Copy number of SDR genes in cross-species. A dot (black) indicated a copy, digital followed by an "×" indicated copy number, and circles indicated absence. (B) Phylogenetic relationships of *AMH* genes across different species. The pink font showing the two copies of *AMH* in *P. vitticeps*. (C) Ideogram showing the gene model of SDR genes and their autosomal counterparts in the Pvit2024 assembly. Pseudogenes were indicated with an asterisk. "-A" indicated autosomal genes, while "-Z" indicated sex-linked genes. (D) Ka/Ks analysis of SDR genes showing order of gene occurrence in Z-linked SDR. (E) Synteny relationships of *AMH-A* and its flanking 5 genes in closely related species. *AMH* is highlighted in orange, and surrounding genes are shown in grey. (F) Differential expression of SDR genes between ZZ and ZW during developmental stages. Benjamini-Hochberg analysis of variance: ns (not significant), *P<0.05, **P<0.01, ***P<0.001.

Discussion

The first chromosome-scale reference genome for Amphibolurinae (Squamata: Agamidae)

Squamate reptiles are a species-rich clade of amniote vertebrates that have adapted to most terrestrial ecosystems [45]. With over 12,000 extant species that make up a significant part of the vertebrate tree of life, reference genomes for squamates are particularly scarce when compared with other amniote clades such as mammals and birds [46]. Specifically, according to the latest data deposited in NCBI (accessed in July 2024), only 104 squamate species have a genome assembly with contig N50 over 30 kb, in sharp contrast with ~630 for birds and ~730 for mammals. This unevenness has drawn the concern of the biodiversity genomics community who call for more attention for this neglected vertebrate group in the genomic era [9, 10]. Thanks to technological advances in long-read sequencing, constructing a near-complete reference genome has become feasible for many organisms, especially for those able to supply sufficient HMW DNA from a single individual, such as the reptiles as showcased in this study. By leveraging a newly released long-read sequencing technique called CycloneSEQ, we generated the *P. vitticeps* Pvit2024 assembly, currently among the most continuous squamate genome. However, this advance simultaneously resolved a persistent challenge: the difficulty in telomere assembly has led to the prevalent absence of telomeric repeats (TTAGGG)n at the chromosomal ends of most published squamate genomes. While our initial assembly captured only 8 of 32 expected telomeres, raw CycloneSEQ reads contained abundant TRUs, clearly demonstrating that these omissions stemmed from computational limitations rather than insufficient sequencing depth. Our pipeline specifically

addresses the critical challenge of telomere sequence recovery from raw sequencing data, which could potentially enhance telomere-to-telomere (T2T) genome assembly efforts across diverse taxonomic groups in future studies.

The reliability of Pvit2024 is further evidenced by multiple lines: Hi-C scaffolding confirmed chromosomal synteny, BUSCO analyses revealed near-complete gene representation (98.5%), and the length, gene density, GC content, repeat content, and inter-chromosomal interactions of microchromosomes also match the recognized features published before. This new *P. vitticeps* genome represents the first chromosome-scale reference genome for the Amphibolurinae subfamily of the agamid lizards (Agamidae), highlighting its potential value in evolutionary genomics studies. With this near-complete *P. vitticeps* reference genome, we also provided novel insights into the sex chromosome origin and sex determination of this species.

AMH signaling as an upstream driver of sexual differentiation in *P. vitticeps*

Our Pvit2024 Z chromosomal assembly, despite unresolved gaps in repeat-rich regions, is approaching T2T level as indicated by the presence of telomeric sequences on both chromosomal ends. Additionally, the Pvit2024 Z has almost doubled the known Z-linked sequences, from 8.3 Mb to 14.3 Mb. More importantly, the newly anchored Z-linked sequences carry the whole SDR, a region that hopefully harbors the master sex-determining gene.

The Z/W-resided *nr5a1*, which encodes the steroidogenic factor 1 (SF1) required for male development, was once regarded as the most promising sex-determining candidate in *P. vitticeps* [47]. However, the location of *nr5a1* in PAR with no W-specific mutations as uncovered in population datasets (Supplementary Table S17) has almost ruled out it as the master sex-determining gene. Instead, the aggregation of *AMH* signaling related genes (i.e., *AMH*, *AHMR2* and *BMPR1A*) in the SDR is of particular interest. *AMH*, which encodes a hormone of the transforming growth factor-β (TGF-β) superfamily, is critical for testis development and has been identified as a master sex-determining gene in a growing number of vertebrates, including several lineages of teleost fish [48] and monotreme mammals [49]. In *P. vitticeps*, we found that the SDR-resided *AMH* (*AHM-Z*) was highly expressed in all examined ZZ embryos and maintained over 5-fold expression difference between ZZ and ZW embryos since the beginning of gonadal

differentiation (stage 6/7). This prominent male-biased expression pattern suggests *AMH-Z* as a strong sex-determining candidate, which deserves further functional experimental validation in future studies.

AMH signals through binding to its receptors, such as the type II receptor encoded by *AMHR2* and the type I receptor encoded by *BMPR1A* [43]. Therefore, our finding of transient up-regulation of the SDR-resided *AMHR2* and *BMPR1A* in ZZ embryos at stage 6/7 is particularly notable. We hypothesize that such a transient up-regulation of the *AHM* receptors may serve as an amplifier of the *AHM* signaling cascade at the bipotential gonads, which is essential for tipping the sex differentiation network towards male development at this critical time window. Another notable finding is the up-regulation of the autosome-resided *AMH* (*AMH-A*) since stage 6/7. Given the high protein similarity of *AHM-Z* and *AMH-A* that implies their functional conservation, this parallel up-regulation of *AMH-A* may serve as another booster of AMH signaling during early sex differentiation. Collectively, all this evidence points to one conclusion, that is, the AMH signaling is essential and likely serve as an upstream signal driving *P. vitticeps* sex differentiation. Such pattern of duplicated gene functioning in upstream sex determination have previously been identified mainly in fish [50]. And interestingly, all these duplications, including our current finding in the *Pogona* lizard, seems to direct gonad development into testis.

However, it is also notable that the gonadal transcriptome profiles of ZZ and ZW embryos has been diverged at stage 6/7 [44]. This suggests that the initial elevation of AMH signaling likely occurs even earlier, before stage 6/7. Interestingly, in teleost fish Patagonian pejerrey (*Odontesthes hatcheri*) a convergent gene duplication event was found as *AMH* was duplicated onto the Y chromosome and may also serve as the candidate sex determinant gene, indicative by an earlier expression than the autosomal *AMH* [51], Future studies are recommended to focus on when and where (e.g., what cell types) the AMH signaling is initially diverged between ZZ and ZW embryos.

A proposed model for the origin and evolution of *P. vitticeps* sex chromosomes

The origin of the *P. vitticeps* ZW microchromosomes is presumably associated with the fusion of a fragment derived from chromosome 2, as both the Z and W chromosomes share homology with the terminal region of the long arm of chromosome 2 (chr2qter) as revealed by physical mapping

of a BAC clone (Pv151P16) [52]. Our result also supports this homology according to the finding of an additional copy of the chr2qter-resided *PFDN6-RGL2* syntenic block in SDR (Fig. 6C). However, we also found that the homology with chr2qter is limited to the terminal region of the *P. vitticeps* Z chromosome which lacks intact protein-coding genes, thus arguing the role of the chr2qter fusion event in driving ZW formation. Indeed, the fusion of chr2qter to *P. vitticeps* ZW microchromosomes seems to occur early during Amphibolurinae evolution, probably before the differentiation of Z and W, because the concurrent mapping of Pv151P16 to chr2qter and a pair of microchromosomes are also observed in other Amphibolurinae species, including GSD species with homomorphic sex chromosomes and TSD species without sex chromosomes [52].

However, it is notable that the *Ks* of *AMH*-Z is higher than that of *PFDN6-Z* and almost comparable to that of *RGL2-Z*, despite that *PFDN6-Z* and *RGL2-Z* have become pseudogenes (Fig. 6C). This suggests that the integration of *AMH* and the fusion of chr2qter to the proto-sex chromosomes might occur within a very narrow time window. We thus propose that the duplication and translocation of the autosomal *AMH* to the proto-sex chromosomes might represent the real milestone in triggering the formation of Z and W chromosomes in the ancestor of *P. vitticeps* and its sister taxa. After *AMH* integration, the subsequent aggregation of other male-biased genes including *AMHR2* and *BMPR1A* further consolidated the role of the proto-Z in sex determination and promoted ZW differentiation. We anticipate that chromosome-scale genome assemblies from other closely related dragon lizards will provide crucial evidence for testing this hypothesis.

Methods

Sample collection

This study was performed in accordance with the guideline of the national and organizational stipulation. An adult lizard *Pogona vitticeps* in captivity was collected at Zhejiang University, under the permit ZJU20240342. This lizard was euthanized after anesthetized by diethyl ether. All the tissue samples were frozen via liquid nitrogen and stored at -80°C refrigerator immediately after dissection. Muscle and lung tissues were selected for genome sequencing, which included CycloneSEQ long-read WGS sequencing, DNBSEQ short-read WGS sequencing, CycloneSEQ based Pore-C sequencing, and Hi-C sequencing. Furthermore, eight tissue samples, including liver,

lung, eye, muscle, testis, kidney, brain, and heart, were subjected for transcriptome sequencing, which included bulk RNA-seq and CycloneSEQ long-read RNA-seq.

Genomic DNA CTAB extraction

The High-molecular-weight (HMW) genomic DNA was extracted from muscle and lung tissues using the CTAB method as follows: Preheat the CTAB lysate in a water bath. Collect 20-30 mg of fresh or frozen animal tissue, freeze in liquid nitrogen. Grind tissue using mortar and pestle in the presence of liquid nitrogen until finely ground. Transfer the ground animal tissue to 2 mL polypropylene centrifuge tubes. Add 1 mL of CTAB lysis buffer and 50 μL protease K (20 mg/mL). swirl and mix, incubate at 50 °C (with shaking) for 1 h. Cool the tube to 37 °C. Add 20 μL RNase A (10 mg/mL) for every 1 mL of lysis buffer, mix by inversion and incubate for 10 min. Add equal volume phenol-chloroform-isoamyl alcohol (25:24:1 ratio) with pH > 7.8, mix by inversion and spin at 5000 rpm for 10 minutes in a tabletop centrifuge at room temperature (RT). Transfer top aqueous solution to new 2 mL centrifuge tubes using wide-bore pipette tip. Add equal volume chloroform-isoamyl alcohol (24:1 ratio), mix by inversion and spin at 5000 rpm for 10 minutes in a tabletop centrifuge at RT. Transfer top aqueous solution to new 1.5 mL centrifuge tubes using wide-bore pipette tip. Add 2/3 volume of isoamyl alcohol and mix by inversion to form an emulsion, centrifuge at 5000 rpm for 2 minutes at 4°C. Discard the supernatant. Add 1mL 75% ethanol and mix slowly to resuspend the precipitation. Centrifuge at 5,000 ×g for 2 min at 4°C and discard the supernatant. Repeat the previous step. Discard the remaining supernatant, dry at RT for 3 min. Dissolve in 200-400 μL TE Buffer and incubate at 37 °C for 1 h, incubate at RT overnight. Store the extracted DNA at -80 °C.

CycloneSEQ long-read WGS library preparation and sequencing

The preparation and sequencing of CycloneSEQ long-read WGS library was proceeded following the manufacturer's guidelines. Each sample, comprising 2 μg of input DNA (≥23 ng/μL), was initially diluted with nuclease-free water to a total volume of 44 μL, followed by mixing with 6 μL DNA repair buffer 1, 3 μL DNA repair buffer 2, 3 μL DNA repair enzyme 1, and 4 μL DNA repair enzyme 2. The mixes were then incubated in a thermocycler through the following steps: 10 min at 20°C, 10 min at 65°C, and held at 4°C. After incubation, the mixes were purified using a 1.0x

volume of DNA clean beads, and DNAs were eluted with 60 μL of nuclease-free water. Next, the purified end-repaired samples were mixed with 10 μL sequencing adaptors, 25 μL 4x ligation buffer, 10 μL DNA ligase, and 2.5 μL nuclease-free water, before incubating at 25°C for 30 minutes to complete the adaptor ligation. The ligated products were again purified with a volume of 1.0x DNA clean beads and long fragment wash buffer was applied to gently resuspend the beads. After removing the supernatant, the libraries were recovered into 17 μL of elution buffer and quantified on a Qubit fluorometer. Each prepared library was sequenced on the CycloneSEQ WuTong02 platform according to the protocol.

CycloneSEQ based Pore-C library preparation and sequencing

About 100 mg tissues were homogenized with pestle and washed with 2000 μL 1X PBS (Phosphate-Buffered Saline). After being spun down, the tissues were fixed by 10ml of 1% formaldehyde 1X PBS solution and incubated for 10 minutes at room temperature (RT). To quench formaldehyde crosslinking, 527 μL of 2.5 M glycine was added and incubated for 5 minutes at RT, followed by 10 minutes on ice. Fixed tissues were pelleted by centrifugation at 2000 ×g for 10 minutes at 4 °C. The pellet was resuspended in 550 μL of ice-cold lysis buffer (10 mM Tris-HCl pH 8.0, 10 mM NaCl, 0.2% Igepal-CA630, 50 μL of protease inhibitor cocktail) and incubated on ice for 15 minutes. Nuclei were pelleted at 2000 ×g for 10 minutes at 4 °C and the supernatant was discarded. The pellet was resuspended in 200 μL of the prepared chilled 1.5X digestion reaction buffer and was pelleted by centrifugation at 2000 ×g for 10 minutes at 4 °C. The pellet was resuspended in 300 μL of 1.5X digestion reaction buffer and added 33.5 μl 1% SDS, followed by incubate in a thermomixer at 300 rpm at 65°C for 10 minutes. The reaction was stopped by adding 37.5 μl of 10% (v/v) Triton X-100. Then, 45 μL of 10 U/μL NlaIII restriction enzyme (NEB, R0125L) and 34μL water were added, and the sample was rotated at 37 °C for 18 hours. NlaIII was then heat-inactivated at 65 °C for 20 minutes. A total of 550 μL of ligation master mix was added: 100 μL of 10× NEB T4 DNA ligase buffer with 10 mM ATP (NEB, B0202), 10 μL of 10 mg/mL BSA (Thermo Fisher, AM2616), 50 μL of 400 U/μL T4 DNA Ligase (NEB, M0202), and 390 μL of water. The reactions were rotated at RT for 5 hours. Then, the sample was added a master mix (100 μl of 10% SDS, 500 μL of 20% tween-20, 100 μl of 20 mg/ml proteinase K, 300 μL of water) and incubated at 56 °C for 18 hours. The sample was extracted with equal volume of phenol:

chloroform: isoamyl alcohol (25:24:1) and precipitated with 0.7 X isopropanol. DNA was size selected (>1.5 kb) using DNA Clean beads and constructed to CycloneSEQ library.

CycloneSEQ long-read RNA-seq library preparation and sequencing

CycloneSEQ long-read RNA sequencing libraries were prepared from eight tissues using the Hifair ® AdvanceFast 1st Strand cDNA Synthesis Kit and the CycloneSEQ Universal Library Prep Set. Total RNA was treated with 5x gDNA Digester Mix (42 °C, 2min) to eliminate genomic DNA. Reverse transcription was performed in 20 µL reactions containing 4x Hifair® SuperMix, Random Primers (50 µM), and RNA template, with incubation at 72°C (5 min) and the heat inactivation at 85°C (5 sec). For high-GC templates, incubation temperature was increased to 60°C. The resulting cDNA was processed into libraries using CycloneSEQ protocols (DNA repair, adapter ligation), mirroring the whole-genome sequencing workflow. Finally, libraries were sequenced on the CycloneSEQ WuTong02 platform.

Other library preparation and sequencing

For the Hi-C library construction, the muscle sample was crosslinked with formaldehyde and 2 Hi-C libraries were constructed by using the dpnII restriction endonuclease. The libraries were sequenced on the DNBSEQ platform with 150-bp paired-end sequencing strategy.

For bulk RNA-seq library construction, the isolated RNA was then fragmented into 200-400 bp, and then reverse-transcribed to cDNA for library preparation. Quantity and quality of the genome’s DNA and RNA were assessed by pulsed field gel electrophoresis, Qubit 3.0 (Invitrogen, USA) and Qseq 400 (Bioptic, China). A total of 12 short-insert paired-end (PE) libraries (4 for genomic DNA and 8 for cDNA) were constructed and sequenced on DNBSEQ platform (MGI, Shenzhen), with the manufacturer’s instructions.

Sex identification

Organ anatomical examination and sex-linked markers analysis were employed for sex identification in *P. vitticeps*. For the anatomical assessment, testes were dissected from the individual. Molecular analysis of sex-linked markers was conducted using a protocol adapted from

Holleley et al. [6], Genotypic sexing was performed utilizing two PCR primers: H2, GCCCATATCTCACTAGTTCCCCTCC; F, CAGTTCCTTCTACCTGGGAGTGC, which was flanking two W-chromosome-specific deletions, measuring 150 base pairs and 14 base pairs, respectively. PCR was conducted using Platinum High-Fidelity ReadyMix(2x) (GCATbio), with a range of primer concentrations and genomic DNA quantities to establish experimental groups, and a no-genomic DNA control group. Cycling conditions were 95℃ for 5 min; (95℃ for 20 s, 70~65℃ for 20 s, 72℃ for 1 min) ×10 cycles with annealing temperature decreased 0.5℃ per cycle; (95℃ for 20 s, 65℃ for 20 s, 72℃ for 1 min) ×30 cycles; 72℃ for 10 min. The PCR products were resolved on a 1.5% agarose gel and visualized using GelStain Blue (Transgen). The presence of two bands indicated ZW individuals, while a single band confirmed ZZ individuals.

Genome survey

The DNBSEQ short-read WGS data was cleaned by SOAPnuke v1.5.6 (RRID: SCR_015025) [53] to exclude reads characterized by low quality and the presence of adapter sequences and poly-N regions with parameters -Q 2 -G -d -l 20 -q 0.2 -5 1 -t 5,0,5,0. To accurately assess genomic characteristics, including genome size and heterozygosity rate, all clean data were adopted *k*-mer based methods by using Jellyfish and GenomeScope tools. The haploid genome size was estimated according to *k*-mer analysis frequency distributions generated by Jellyfish v2.2.6 (RRID: SCR_005491) [54] using a series of *k* value (19, 21, 23, 25, 27) with the -C setting, which was calculated as the number of effective *k*-mers (ie. total *k*-mer – erroneous *k*-mer) divided by the homozygous peak depth. The rate of heterozygosity was estimated by GenomeScope v2.0.0 (RRID: SCR_017014) [55] with the *k*-mer frequency distributions generated by Jellyfish as inputs.

Genome assembly

The initial process was to retain CycloneSEQ long reads longer than 40 kb, the chimeric reads were removed using Yacrd v1.0.0 [56] with parameters -c 5 -n 0.6. Cleaned reads were then assembled into contigs using NextDenovo v2.5.0 (RRID:SCR_025033) [16] with the following parameters: read_cutoff = 35k, genome_size = 1.7g. In response to the high heterozygosity detected in the initial assembly, DNBSEQ short reads were adopted to remove heterozygous contigs by purge_haplotigs v1.1.2 (RRID:SCR_017616) [17] with parameters -l 10 -m 53 -h 120.

Next, two rounds of polishing were performed using NextPolish v1.4.1 (RRID:SCR_025232) [18] with recommended parameters, utilizing DNBSEQ short reads. Subsequently, CycloneSEQ based Pore-C reads were used as the main body to anchor the assembly to pseudo-chromosomes, while two additional libraries of Hi-C reads served as controls to assess the reliability of the anchoring. The specific process of anchoring was as follows: CycloneSEQ based Pore-C reads were first aligned to the contig-level genome using wf-pore-c v1.1.0 (https://github.com/epi2me-labs/wf-pore-c) with default parameters. The unaligned fragments were subsequently filtered out from the wf-pore-c output file 'null.ns.bam' using the command 'samtools view -F 4 -bh'. Adjacent fragment pairs were then extracted from the filtered BAM file into BED format with a custom script (https://github.com/guoqunfei/Pvit_T2T). In parallel, two additional libraries of Hi-C reads were aligned using Chromap v0.2.3-r407 [57] with default parameters. The alignment results were converted from the .sam format to a .bed file using bedtools v2.29.2 [58]. Both sets of BED files, derived from CycloneSEQ based Pore-C and Hi-C reads respectively, were utilized to assemble contigs into scaffolds using YaHS v1.2a.1 [19] with the parameters –no-contig-ec –no-scaffold-ec. Following the scaffolding process, we proceeded to generate a .hic file for further manual examination and curation using JuiceBox (JBAT) [20], adhering to the guidelines provided at https://github.com/c-zhou/yahs.

To avoid telomeric repeat sequences being incorrectly trimmed by the assembler, by using known vertebrate six base telomere repeats (‘TTAGGG’) as a sequence query, we developed an in-house pipeline to correct telomeric regions as follows: First, we extracted those reads from CycloneSEQ WGS raw reads containing at least 100 consecutive copies of the telomere units. Then, all of these reads were aligned to the assembly using minimap2 v2.23-r1116-dirty (RRID:SCR_018550) [33] with the parameter: -x map-ont. Subsequently, based on the alignment results, for each chromosome’s two ends, the read with the highest alignment quality and the largest number of copies of the telomere unit was selected and used to replace the corresponding end. (The specific implementation code can be seen at https://github.com/guoqunfei/Pvit_T2T).

After telomere repair, unclosed gaps in the chromosome-scale assembly were initially filled using TGS-GapCloser v1.2.1 (RRID:SCR_017633) [22] with the corrected long reads (generated by NextDenovo during assembly). The correctness of each closed gap was further examined by long-read coverage with an in-house pipeline as follows: the 5 kb of upstream and downstream regions

were extracted and aligned to the corrected long read to identify any alignment. These alignments were further visualized and manually examined using IGV. Only alignments with proper size and consistent orientation without conflicting alignments were used for the purpose of gap filling within the genomic assembly.

Finally, the assembled genome was evaluated using BUSCO v5.7.1 (RRID:SCR_015008) [23], Compleasm v0.2.6 [24], Merqury v1.3 (RRID:SCR_015811) [59] using default parameters. Short reads were aligned to the genome using BWA-MEM v0.7.17-r1198-dirty (RRID:SCR_012940) [60], and SAMtools v1.15.1 (RRID:SCR_002164) [61] were counted as properly paired. Long reads were mapped to the genome using minimap2 v2.23-r1116-dirty (RRID:SCR_015008) [33] with the parameter: -ax map-ont.

Reconstruction of NOR 45S rDNA cluster

According to the comprehensive cytogenetic map, *P. vitticeps* has an active nucleolus organizer region (NOR) rich in 45S rDNA cluster, comprising of 18S, 5.8S, and 28S rRNAs, at the sub-telomeric region of Chromosome 2 [11]. However, the sequencing depth of the NOR region, identified as the 45S rDNA-rich region near the telomere on Chr2 from the *de novo* assembly of whole genome, was significantly higher than that of adjacent regions. Additionally, the length of the NOR region was much shorter than those found in closely related species. These findings indicated that the NOR region was not fully assembled. To address this, we reconstructed the NOR 45S rDNA cluster. Firstly, the 45S rDNA sequence from NOR region was used to identify all CycloneSEQ reads containing the 45S rDNA sequence feature. Secondly, these reads were assembled using NextDenovo v2.5.0 [16] with the following parameters: read_cutoff = 1k, genome_size = 0.005g, minimap2_options_raw = -I 6G --step 2 --dual=yes -t 4 -x ava-ont -k 17 -w 17 --minlen 2000 –maxhan1 5000. Finally, we obtained a 282.2 kb NOR region rich in 45S rDNA cluster and replaced this segment with the genomic NOR position where the assembly had collapsed.

Repetitive element annotation

We annotated the lizard whole-genome repeat sequences based on *de novo* predictions and homology annotations. For *de novo*, the RepeatModeler v2.0.4 (RRID:SCR_015027) [62] was

used to identification the custom repeats from the assembly sequence, with subsequent annotation and masking using RepeatMasker v4.1.5 (RRID:SCR_015027) [63]. For homology annotations, we first identified known transposable elements in the Pvit2024 genome using RepeatMasker by searching against the RepeatModeler (v2.0.4) transposable element library. Then, the transposable element proteins were searched based on RepeatProteinMask database (http://www.repeatmasker.org) that part of RepeatMasker. Tandem repeats were also extracted using TRF v4.09 [64] via ab initio prediction, with the parameters following: “Match = 2, Mismatch = 7, Delta = 7, PM = 80, PI = 10, Minscore = 50, and MaxPeriod = 2000”.

Protein coding gene annotation

We integrated three types of evidence for predicting protein-coding genes: transcriptome data, homology evidence, and ab initio prediction. For the transcriptome evidence, we first cleaned the DNBSEQ RNA-seq data using SOAPnuke v1.5.6 (RRID:SCR_015978) [53] with the command: filter -n 0.03 -l 20 -q 0.3 -p 1 -Q 2 -G -5 1 -t 10,0,10,0 -E 70. Next, we aligned the cleaned short reads to the genome using HISAT2 v2.2.1 (RRID:SCR_015530) [65] with the –dta option. Finally, the transcripts were reconstructed using StringTie2 v2.2.3 (RRID:SCR_016323) [66] and coding sequences were predicted with TransDecoder v5.7.1 (RRID:SCR:015534) (https://github.com/TransDecoder/TransDecoder). For the ab initio prediction, we randomly selected 700 high-quality genes from the transcriptome predictions for training and used AUGUSTUS v3.4.0 (RRID:SCR_008417) [67] for the ab initio annotation of coding genes. For homology-based prediction, homologous data from five closely related species including *Ahaetulla prasina* (GCF_028640845.1), *Furcifer pardalis* (GCA_030440675.1), *Hemicordylus capensis* (GCF_027244095.1), *Rhineura floridana* (GCF_030035675.1), *and Zootoca vivipara* (GCF_963506605.1), were collected by NCBI RefSeq. To sum up, we integrated the above three types of evidence and short reads splice information data as input to GeMoMa v1.9 (RRID:SCR_017646) [68] workflow for a comprehensive prediction of coding genes. For the annotation results obtained from the GeMoMa, we further filtered based on the repeat sequence annotation file, removing predictions suspected to be repeat elements, ultimately obtaining the final protein coding gene annotation set.

Isoform detection by long reads

The process of CycloneSEQ long-read RNA-seq data was as follows: we filtered the TSO (template switch oligo sequence)/RTP (reverse transcription primer) and split chimeric reads based on artificial sequence. The clean data was mapping to assembly using winnowmap2 v2.03 [32] with the command: winnowmap -u b -K 4G -G 135k -ax splice ref fastq. We then reconstructed transcripts based on the reference annotation using Isoquant v3.5.0 [69] with the command: *python3 isoquant.py –gene_quantification all –reference genome.fa –data_type nanopore –genedb genes.gtf –threads 16 –count_exons –bam input.bam –label sample*. Subsequently, we merged the transcripts from eight tissues, and eliminate redundancy based on an all-exon overlap threshold of greater than 0.95 between isoforms. We then performed TransDecoder v5.7.1 to predict the coding potential of all transcripts using the *–single_best_only* option. Finally, isoforms of coding gene were assigned to the correspond loci.

LncRNA annotation

LncRNA identification also based on long sequencing reads of CycloneSEQ RNA-seq. The remaining transcripts assembled by Isoquant from the isoform detection steps, which filtering by length (⩾200 nt), were used as the foundational dataset for lncRNA detection. We then utilized two tools of FEELnc v0.2.1 [70] and CPC2 v1.0.1 (Coding Potential Calculator, RRID:SCR_002764) [71] to filter potential coding genes. The candidates of lncRNA were obtained by taking the intersection of the results from both tools. We further mapped the corresponding DNBSEQ sequencing data from the same library with CycloneSEQ RNA-seq using HISAT2 v2.2.1 for alignment to the assembly. Expression detection of candidate lncRNAs was performed using FeatureCounts v2.0.1 (RRID:SCR_012919) [72]. We ultimately considered lncRNAs with TPM > 1 to be true and reliable.

De novo prediction for tRNA and rRNA

Two types of noncoding RNAs were predicted, namely transfer RNAs (tRNAs) and ribosomal RNAs (rRNAs). To identify putative tRNA genes in the *P. vitticeps* genome, we employed tRNAscan-SE v2.0.10 (RRID:SCR_008637) [73] with parameters optimized for eukaryotic genomes. Subsequently, we filtered out “pseudo” and “undet” from the output generated tRNAscan-SE, and extract high-confidence tRNA genes from the BED file, producing a refined

tRNA set. For rRNA prediction, we employed the barrnap v0.9 (RRID:SCR_015995) (https://github.com/tseemann/barrnap) program to identify rRNA genes in the genome, using the following command: barrnap –quiet –kingdom euk genome.fa –threads 20.

Chromosome contact analyses

In the above scaffolding procedure, the .hic files were derived from the processing of CycloneSEQ based Pore-C reads and Hi-C reads. Subsequently, the interaction contacts within the .hic files were binned to construct the genome-wide interaction matrix at resolutions of 5 kb, 10 kb, 100 kb-, 500 kb, 100 kb, 500 kb and 1Mb. Following this, the ICE (iterative correction and eigenvector decomposition) normalization was then employed to normalize the interaction matrix. Then, cis-contacts and trans-contacts for each 100-kb window were calculated using the 100-kb normalized interaction matrix.

Missing sequence identification

We compare the Pvit2024 genome assembly with a previous (Pvit2015) assembly generated using Illumina sequencing. The Pvit2015 assembly was downloaded from NCBI RefSeq by searching for GCF_900067755.1. We excluded the mitochondrial genome from the assembly to prevent misalignment between mitochondrial and nuclear genomes. We then aligned the Pvit2015 assembly to Pvit2024 ref by minimap2 v2.23-r1116-dirty (RRID:SCR_018550) [33] with the following command: *minimap2 -ax asm20 -k14 -K 8G –secondary=no -s 80 -t 16 Pivt2015 Pvit2024*, and with paftools to obtain the aligned regions by minimap2. Additionally, we also performed alignments using the Winnowmap2 tool, which requires the creation of a database with Meryl v1.4.1 [59]. The database was created using the following commands: *meryl count k=19 output merylDB Pvit2024* and *meryl print greater-than distinct=0.9998 merylDB > repetitive_k19.txt*. The alignment was then executed with the command: *winnowmap -t 16 -W repetitive_k19.txt -K 8G –secondary=no -ax asm20 -s 80 Pvit2024 Pvit2015*. Similarly, we used paftools to obtain the alignment files for the two genomes. We further utilized a Python scripts (https://github.com/guoqunfei/Pvit_T2T) to extract unaligned regions from the alignment files. The final set of missing regions was determined by taking the intersection of the results obtained from both methods.

Missing sequences in genomic elements

We identified the proportions of coding genes, lncRNAs, and promoter within the missing regions based on coordinate overlap information. To identify missing coding genes and lncRNA, we considered a locus span and exon region with an overlap greater than 0.8 with the missing region as a missing gene in the Pvit2015 assembly. In contrast, an overlap between 0.1 and 0.8 was classified as an incomplete gene. Additionally, for the identification of promoters, we defined the 2000 bp upstream region of coding genes and lncRNAs as potential regulatory regions. An overlap greater than 0.7 between these regions and the missing region was considered a missing promoter, while an overlap between 0.1 and 0.7 was classified as an incomplete promoter.

Calculation of GC content and repeat content

GC content was determined by calculating the total number of Gs and Cs divided by the length of the given coordinates, excluding ambiguous nucleotides (N), using a custom Python script. Repeat content was assessed based on pre-annotated repeat files, with a Python script used to extract the coordinates of repeat regions and count the number of repeat nucleotides.

Identification of S0 region

To identify regions with differential sequencing coverage between the Z and W chromosomes of *P. vitticeps,* two ZW female WGS data and four ZZ male WGS data were aligned to the assembled genome using BWA-MEM v0.7.17-r1198-dirty (RRID:SCR_012940) [60] (Supplementary Table S13). Depth of coverage was extracted using SAMtools v1.15.1 (RRID:SCR_002164) [61]. Median depth was calculated using a non-overlapping sliding window of 10 kb.

Searches for genes in SDR regions of closely related species

The genome assemblies of all closely related species, including *Gambelia wislizenii* (GCA_030847615.1) , *Intellagama lesueurii* (GCA_037013535.1) [74], *Phrynocephalus forsythia* (GCA_029282475.1) [75], *Phrynocephalus guinanensis* (GCA_037367245.1) [76], *Phrynocephalus putjatai* (GCA_037367255.1) [76], *Phrynocephalus versicolor* (GCA_023846285.1) [76], *Phrynocephalus vlangalii* (GCA_037367305.1) [76], *Phrynosoma*

blainvillii (GCA_026167975.1) [77], *Phrynosoma platyrhinos* (GCA_020142125.1) [78], *Sceloporus tristichus* (GCA_016801415.1) [79], *Sceloporus undulatus* (GCA_019175285.1) [28], as well as the outgroup species *Gallus gallus* (GCF_016699485.2) and *Homo sapiens* (GCF_009914755.1) [80], were downloaded from NCBI RefSeq. We used the protein sequences of *AMHR2*, *BMPR1A*, *AMH*, *ADAMTS4*, *PFDN6*, and *RGL2* from human, chicken, and green anole, which were downloaded from Ensembl, as query sequences. We further conducted tblastn of blast v2.11.0 [81] searches using these query sequences against the whole genomes of closely related species, with search parameters set to *-evalue 1e-5 -num_threads 16*. For the potential loci obtained from the tblastn searches, we extended 2000 bp upstream and downstream of each locus. Subsequently, we performed detailed predictions using GeneWise v3 (RRID:SCR_015054) [82], which allowed us to accurately identify the coding regions of coding genes. Finally, we compared the predicted proteins against the non-redundant protein databases of human, chicken, and green anole to confirm the reliability of our predictions.

Gene synteny analysis

Based on the annotation methods previously applied to *P. vitticeps*, we rapidly performed batch predictions of coding genes on the genome assemblies of nine closely related species using GeMoMa. The annotation GFF3 files and coding sequences were collected. Syntenic gene pairs between *P. vitticeps* and related species were identified using the Python version of MCScan, JCVI v1.1.16, with the following command: *python3 -m jcvi.compara.catalog ortholog --score=0.95 --no_strip_names*. Syntenic blocks were filtered using the command: *python3 -m jcvi.compara.synteny mcscan --iter=1*. We combined all species' syntenic gene pairs based on *P. vitticeps* loci using the command: *python3 -m jcvi.formats.base join --boheader*. For the synteny plot of each target gene, we selected the target gene and its flanking 10 genes from the *P. vitticeps* locus and generated the plot using the command: *python3 -m jcvi.graphics.synteny*.

Phylogenetic analysis and gene evolution

The protein sequences of each orthologous group of sex-determining genes were aligned using MAFFT v7.471 (RRID:SCR_011811) [83] with the parameters: mafft --anysymbol --maxiterate 1000 --localpair pep.fa. The multiple alignment sequences were then processed to remove spurious

sequences or poorly aligned regions using trimAl v1.4.rev15 (RRID:SCR_017334) [84] with the command: trimal -in pep.fa.aln -out pep.fa.aln.trimal -gt 1. Maximum likelihood inference of phylogenetic relationships was performed using IQ-TREE v2.2.0.3 (RRID_SCR_017254) [85] with the parameters: iq-tree -m TEST -bb 1000 -bnni. The ratios of non-synonymous to synonymous substitutions within six sex-determining gene pairs were calculated using KaKs_Calculator v2.0 (RRID:SCR_022068) [86] with its default settings.

Transcriptome source across different sexes and developmental stages

For the male and female gonadal transcriptome data, we used previously published datasets, which included *P. vitticeps* ZZ and ZW individuals at four developmental stages (6/7, 12, 16, and adult) [8, 44]. The early-stage gonadal RNA-seq source accession numbers are SRP304423. The adult source accession numbers used for testes of ZZ male are ERR753529 and ERR413070, and for ovaries of ZW female are ERR753530 and ERR413082.

RNA-seq and differential expression analysis

Paired-end raw reads underwent adapter trimming using SOAPnuke v1.5.6 (RRID: SCR_015025) [53] with *filter -n 0.03 -l 20 -q 0.3 -p 1 -Q 2 -G -5 1 -t 10,0,10,0 -E 70.* Quality control of the sequencing reads was conducted using FastQC v0.12.1 (RRID:SCR_014583) . The clean data were mapped to genome using STAR v2.7.11a (RRID:SCR_004463) [87]. Counts of reads mapping to genes were obtained using FeatureCounts v2.0.1 (RRID:SCR_012919) [72] against the top-length isoform annotation with the following command: -T 16 -g gene_id -t exon. To investigate gene expression changes from stage 6/7 to adult, differential gene expression analysis was conducted between these stages for each sex, ZZ and ZW. Differential expression was performed in R v4.0.2 using the DESeq2 package v1.28.1 (RRID:SCR_015687) [88]. Additionally, we processed the raw expression matrix using TPM (Transcripts Per Million) counts to ensure comparability between samples, and ultimately used these values to generate histograms of gene expression.

Additional Files

Supplementary Fig. S1. Description of sequencing data, genome assembly and sex identification.

Supplementary Fig. S2. Quality validation of the genome assembly.

Supplementary Fig. S3. Analysis of missing sequences and related genomic features.

Supplementary Fig. S4. Expression matrix of 15,758 lncRNAs across 8 different tissues.

Supplementary Fig. S5. Analysis of sex chromosome.

Supplementary Fig. S6. Analysis of SDR genes in *P. vitticeps* and related species

Supplementary Table S1. Summary statistics of sequencing data for the genome assembly and annotation of *Pogona vitticeps*.

Supplementary Table S2. Detailed statistics for CycloneSEQ long-read RNA-seq data from eight tissues.

Supplementary Table S3. Detailed statistics for DNBSEQ short-read RNA-seq data from eight tissues.

Supplementary Table S4. Estimation of genome size and heterozygosity of *Pogona vitticeps* by *k*-mer analysis.

Supplementary Table S5. Improvement in continuity and completeness of genome assembly generated by each of the seven assembly steps as stated in main text.

Supplementary Table S6. Genome features between Pvit2024 and Pvit2015.

Supplementary Table S7. Summary of comprehensive assessment of genome completeness for 67 long-read sequenced Squamata species using BUSCO and Compleasm.

Supplementary Table S8. Summary of telomere and centromere location information of *Pogona vitticeps* genome.

Supplementary Table S9. Comparison of telomeric region characteristics between *Pogona vitticeps* assembly and 28 squamate species whose genome reached chromosome level.

Supplementary Table S10. Annotation of repeat sequences.

Supplementary Table S11. Gene model and function annotation.

Supplementary Table S12. 47 protein-coding genes and 65 lncRNA genes that are missing or unknown to be Z-linked in prior Pvit2015 assembly.

Supplementary Table S13. Data information for identification of S0 region.

Supplementary Table S14. Overview of all sequenced embryonic gonad samples included in this study.

Supplementary Table S15. Differential expression profiles of Z-link genes across various developmental stages.

Supplementary Table S16. Quality control of long-range sequencing data from two platforms.

Supplementary Table S17. The distribution of the depths of ATGC at each site of the *nr5a1* gene locus (26,763 bp in length) across 20 samples.

Abbreviations

LncRNA: Long non-coding RNA; PAR: pseudo-autosomal region; SDR: sexually differentiated region; EBP: Earth BioGenome Project; VGP: Vertebrate Genomes Project; WGS: whole genome sequencing; PCR: polymerase chain reaction; HMW: high molecular weight; TRUs: telomeric repeat units; T2T: telomere-to-telomere; BUSCO: Benchmarking Universal Single-Copy Orthologs; QV: consensus quality value; RNA-seq: RNA-sequencing; TPM: Transcripts Per Million; NCBI: The National Center for Biotechnology information; UTR: Untranslated Region; 5'-UTRs: five prime untranslated regions; 3'-UTR: three prime untranslated regions; A3: alternative 3' splice site; A5: alternative 5' splice site; AF: alternative first exon; AL: alternative last exon; MX: mutually exclusive exons; RI: retained intron; SE: skipped exon; GSD: genetic sex determination; chr2qter: the terminal region of the long arm of chromosome 2; TSD: Temperature-

dependent sex determination; TSO: template switch oligo sequence; RTP: reverse transcription primer ; NOR: nucleolus organizer region; tRNAs: transfer RNAs; rRNAs: ribosomal RNAs.

Code availability

The bioinformatic tools used in this work, including version, setting and parameters, had been described in the Methods section. Default parameters were applied if no parameters were mentioned for a tool. The pipeline for genome *de novo* assembly, annotation, and chromosome contact analysis, and the custom code and scripts for sex chromosomes analysis in this work had been deposited at GitHub (https://github.com/guoqunfei/Pvit_T2T). All figures were plotted in R.

Data Availability

CycloneSEQ long-read WGS data, DNBSEQ short-read WGS data, CycloneSEQ long-read RNA-seq data, DNBSEQ short-read RNA-seq data, CycloneSEQ based Pore-C data, and Hi-C data in this study are deposited in NCBI Sequence Read Archive (SRA) under BioProject accession no. PRJNA1184386 and in the CNGB Nucleotide Sequence Archive (CNSA) of China National Gene Bank DataBase (CNGBdb) under accession no. CNP0005509. Genome assembly, annotation, other supporting data, and material are available in the *GigaScience* GigaDB database.

Competing interests

All authors are employees of the BGI Group. They declare that they don’t have stock, patents, or direct financial incentives from CycloneSEQ technology, and they have no competing interests.

Funding

The work was supported by the National Natural Science Foundation of China (grant no. 32370666 to Q.L.)

Authors' contributions

Q.L., Y.D., and Y.G. conceived the study. W.D., W.J., and Y.W. performed sample collection and tissue dissection. F.G., T.Z., X.S., and X.Y. conducted the CycloneSEQ long-read WGS experiments and sequencing. J.C., C.C., and J.Y. performed CycloneSEQ based Pore-C experiments and sequencing. Q.G., Y.P., W.C., H.C., Y.Z., and C.Y. performed genome assembly and annotation. Q.G., Y.P., Y.M., Y.Z., S.S., and B.W. conducted comparative analyses, combined the results, and draw figures. X.X. contributed computing resources. Q.L. wrote the manuscript with the inputs from all authors. All authors read and approved the final manuscript.

Acknowledgements

We thank Professor Arthur Georges and Dr. Sarah L. Whiteley from the University of Canberra for their providing transcriptome data of *P. vitticeps* across early developmental stages. We also thank the China National GeneBank for providing computing resources.

Supplementary Figures

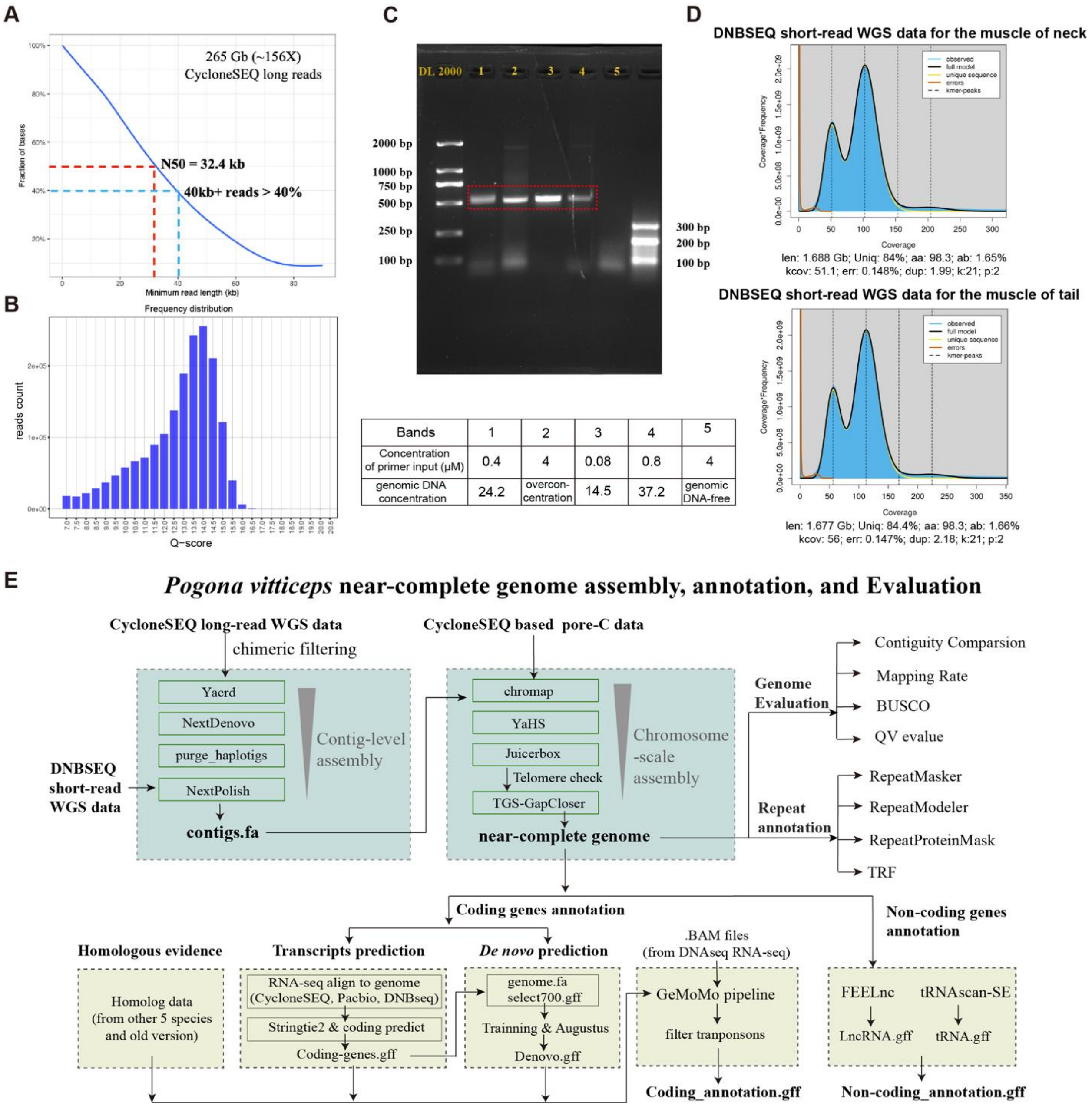

Figure S1: Description of sequencing data, genome assembly and sex identification (related to Fig. 1). (A) Read length distribution of CycloneSEQ data. The curve plots cumulative base fraction (y-axis) against minimum read length thresholds (0-80 kb, x-axis). Key metrics: N50=32.4 kb (red dash), >40% bases from reads >40 kb (blue dash). Total output: 265 Gb (~156× coverage). (B) Blue histogram shows read counts (y-axis, $0\text{-}2\times10^5$) across Q-scores (x-axis, 7.0-20.5), peaking at 13.5–14.5. (C) Amplification of the DNA fragment of the PCR primers that flank two W-chromosome-specific deletions. Band 1 to 4 were made using different concentrations of

primers and genomic DNA. The fifth band without genomic DNA was the control group. (D) The 21-mer frequency distribution of two libraries of DNBSEQ short-read WGS data. (E) Comprehensive assembly and annotation workflow for the near-complete genome of *P. vitticeps*.

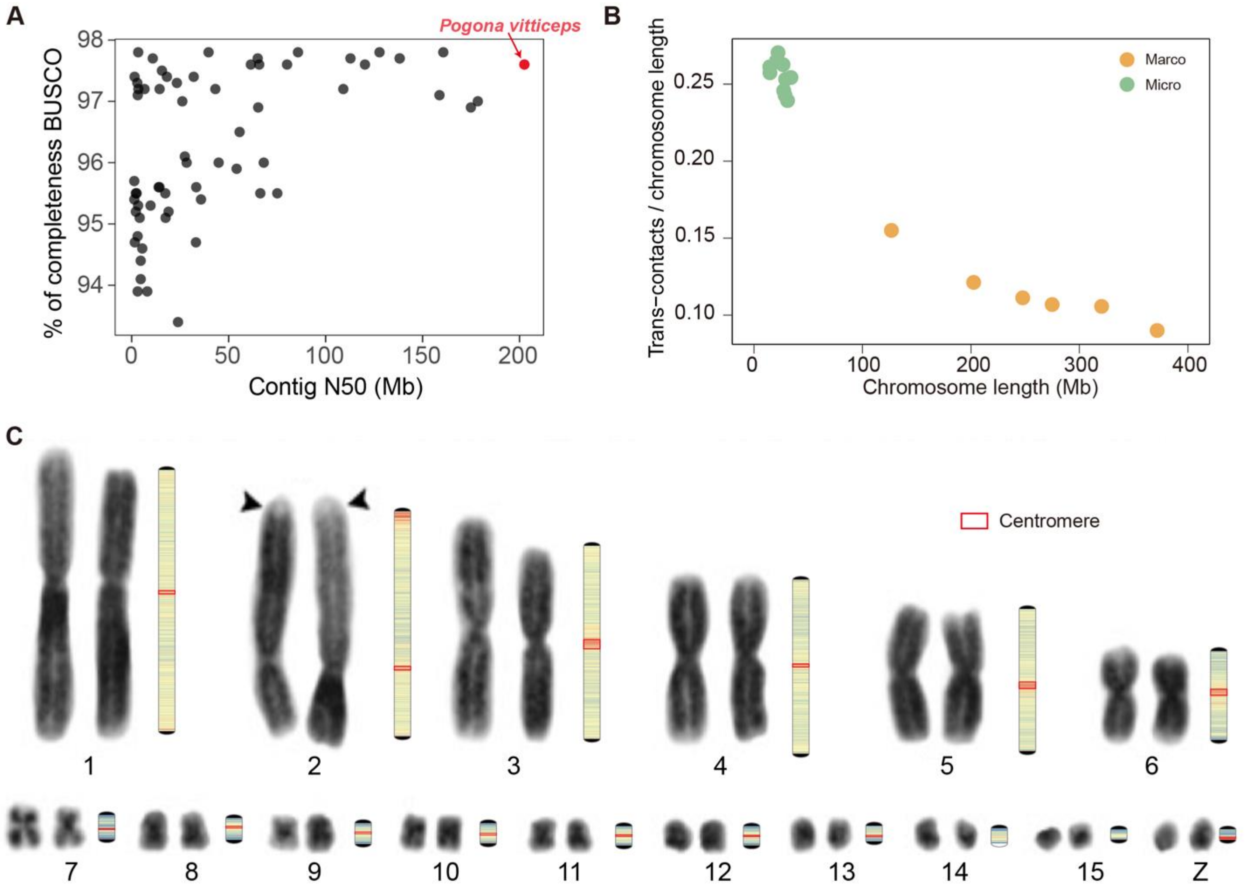

Figure S2: Quality validation of the genome assembly (related to Fig. 2). (A) The comparison of BUSCO complete score based on 7,480 Sauropsida conserved genes and contig N50 between Pvit2024 and 66 publicly available squamate genomes assembled by long-read data. (B) Trans-contact for each chromosome were calculated by ~153X Hi-C_1 data. (C) A high degree of consistency between the location of the centromere region identified by the near-complete genome with the depressed region in the karyotype map from Young et al. , which is generally believed to be the centromere.

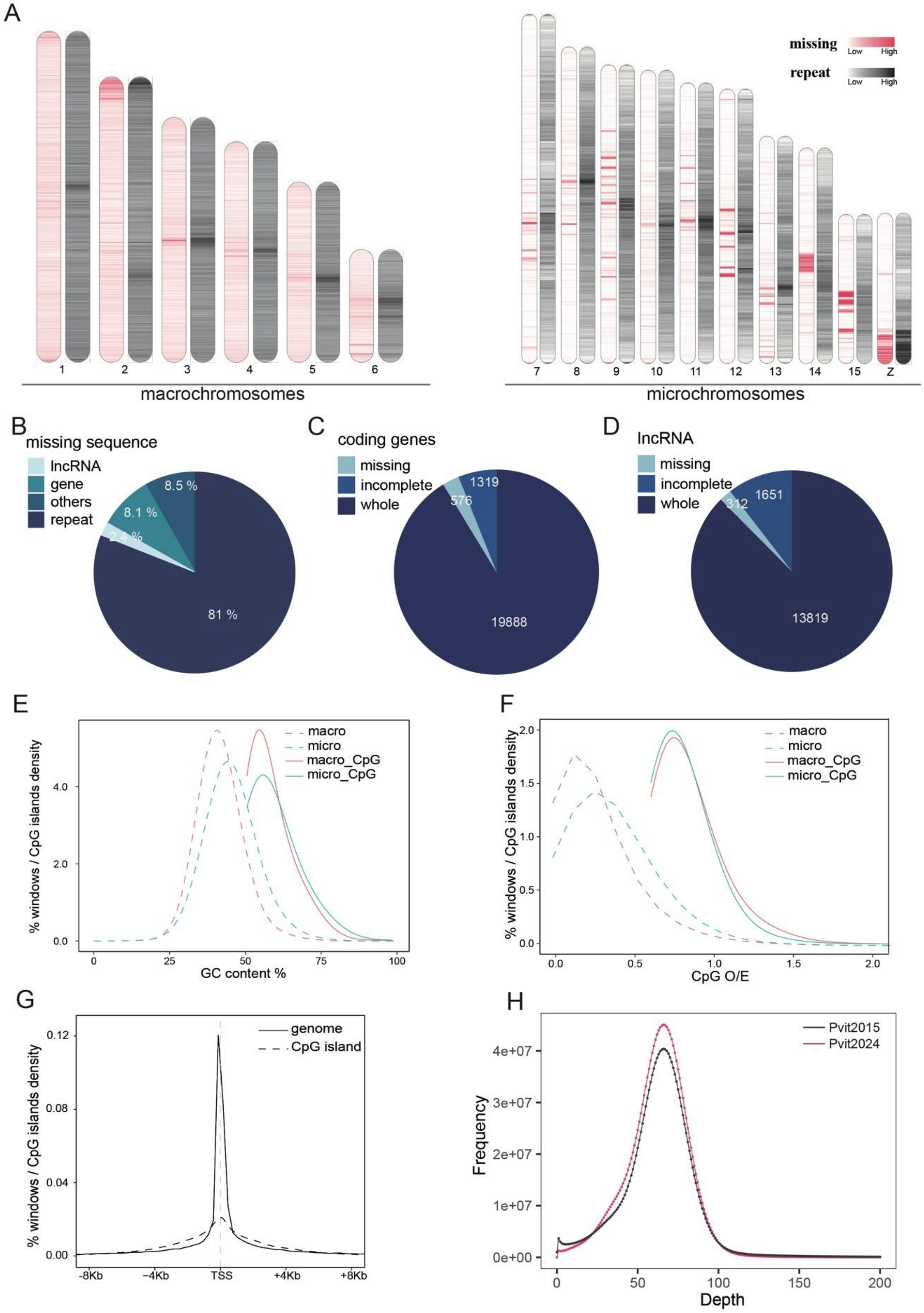

A
missing
Low
High
repeat
Low
High
1
2
3
4
5
6
macrochromosomes
7
8
9
10
11
12
13
14
15
Z
microchromosomes
B
missing sequence
lncRNA
gene
others
repeat
8.5 %
8.1 %
2.4 %
81 %
C
coding genes
missing
incomplete
whole
1319
576
19888
D
lncRNA
missing
incomplete
whole
1651
312
13819
E
macro
micro
macro_CpG
micro_CpG
% windows / CpG islands density
GC content %
F
macro
micro
macro_CpG
micro_CpG
% windows / CpG islands density
CpG O/E
G
genome
CpG island
% windows / CpG islands density
-8Kb
−4Kb
TSS
+4Kb
+8Kb
H
Pvit2015
Pvit2024
Frequency
Depth

Figure S3: Analysis of missing sequences and related genomic features (related to Fig. 3). (A) Overview of missing and repeat sequences distribution across all chromosomes. The red range indicates missing sequences, and the black range represents repeat sequences. (B) Proportion of various types of elements within the missing sequences. (C) Types and proportions of missing coding genes in the Pvit2015. (D) Types and proportions of missing lncRNAs in the Pvit2015. (E) In the genome, use a non-overlapping sliding window method to scan the genome, and the window size was set to 200bp. By calculating the GC content and CpG O/E value of each window, windows that satisfied the GC content $\geq$ 50% and CpG O/E $\geq$ 0.6 were identified as CpG island. The GC content density distribution maps of all windows and CpG islands in the genome were obtained, in which macro- and micro- chromosomes were displayed separately due to different genomic characteristics. (F) CpG O/E value density distribution of all windows and CpG islands in the genome, macrochromosomes and microchromosomes are displayed separately. (G) According to the genome annotation file, TSS (transcription start site) was located, and the CpG island closest to each TSS was found within 8 kb upstream and downstream, and its relative distance was calculated. For comparative analysis, 200 bp windows equal to the CpG islands were randomly selected in the genome, and the closest relative distance between these random windows and each TSS were also counted. The density distribution maps of the closest distances between TSS and the random windows of the genome and CpG islands were obtained to compare the distribution characteristics. (H) Distribution of base coverage depth after mapping DNBSEQ reads to the Pvit2015 and Pvit2024 genomes.

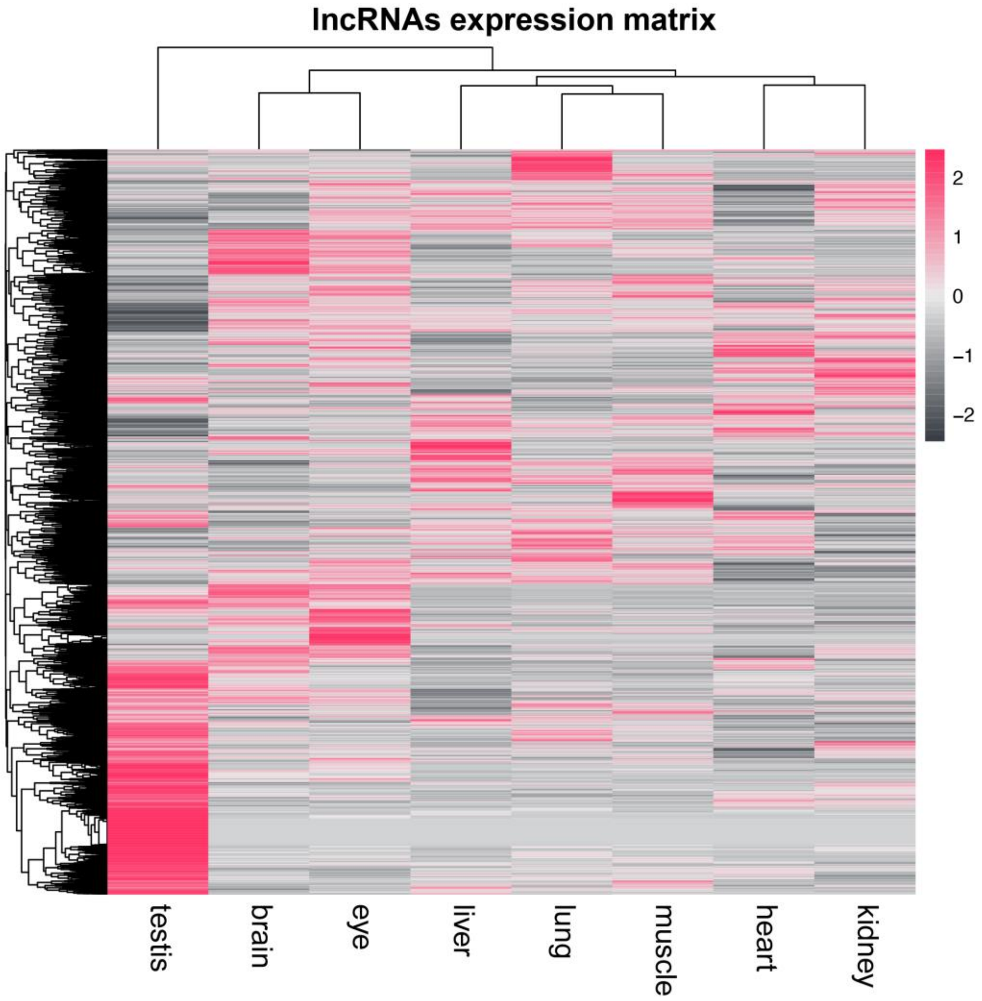

Figure S4: Expression matrix of 15,758 lncRNAs across 8 different tissues (related to Fig. 4).

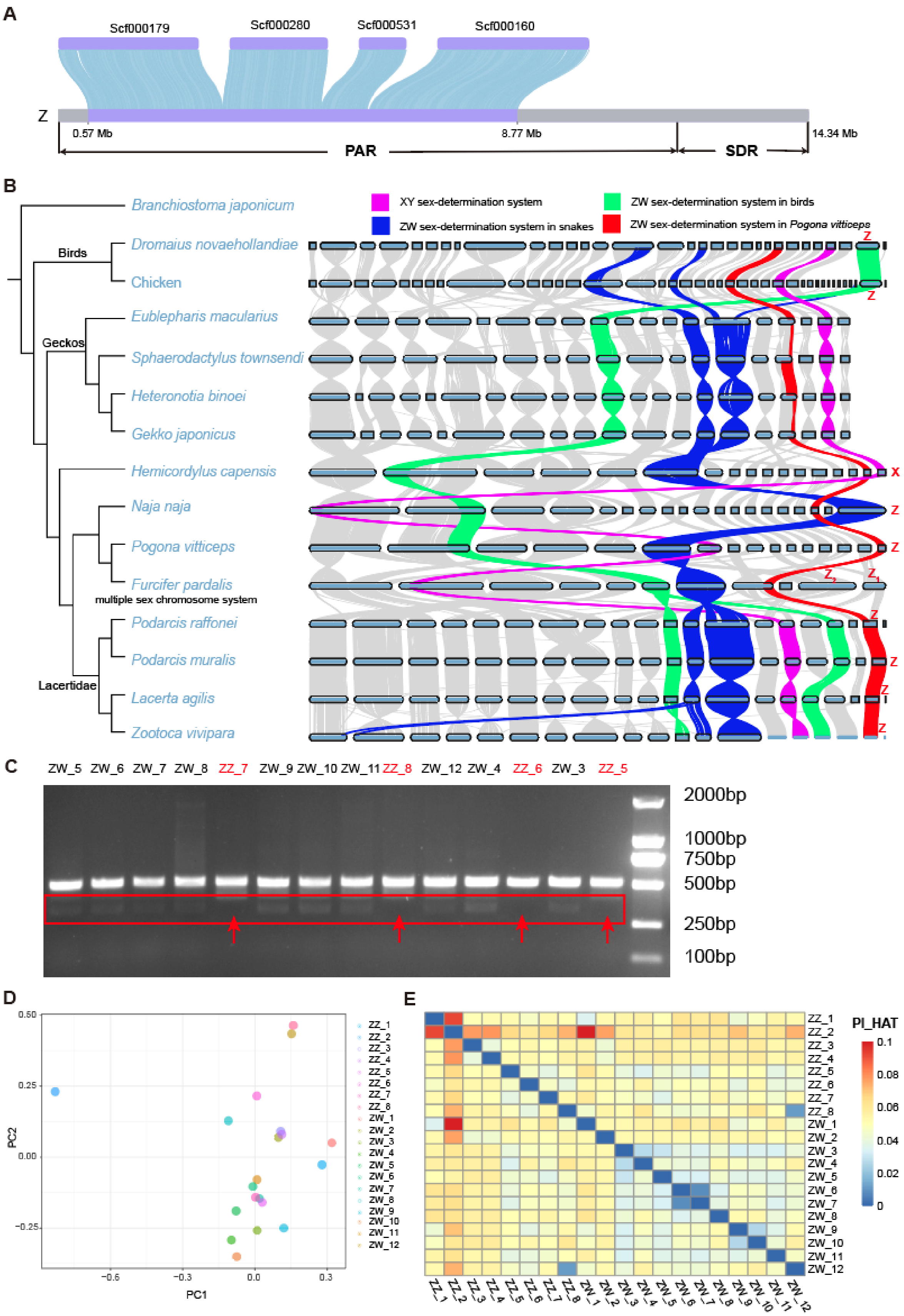

A
Scf000179
Scf000280
Scf000531
Scf000160
Z
0.57 Mb
8.77 Mb
14.34 Mb
PAR
SDR
B
Branchiostoma japonicum
Birds
Dromaius novaehollandiae
Chicken
Geckos
Eublepharis macularius
Sphaerodactylus townsendi
Heteronotia binoei
Gekko japonicus
Hemicordylus capensis
Naja naja
Pogona vitticeps
Furcifer pardalis
multiple sex chromosome system
Podarcis raffonei
Podarcis muralis
Lacertidae
Lacerta agilis
Zootoca vivipara
XY sex-determination system
ZW sex-determination system in snakes
ZW sex-determination system in birds
ZW sex-determination system in Pogona vitticeps
C
ZW_5 ZW_6 ZW_7 ZW_8 ZZ_7 ZW_9 ZW_10 ZW_11 ZZ_8 ZW_12 ZW_4 ZZ_6 ZW_3 ZZ_5
2000bp
1000bp
750bp
500bp
250bp
100bp
D
PC1
PC2
E
PI_HAT

Figure S5: Analysis of sex chromosomes (related to Fig. 5). (A) Collinearity between the four known Z-linked scaffolds identified by Deakin et al. and the Z chromosome of Pvit2024. (B) Identification of homologous regions between the sex chromosomes of squamate species and the Z chromosomes of two closely related avian species, chicken (*Gallus gallus*) and emu (*Dromaius novaehollandiae*), based on comprehensive gene collinearity analysis. (C) Agarose gels electrophoresis results for 14 additional individuals. ZZ males exhibited a single band, whereas ZW females showed two distinct bands. (D) Principal Component Analysis (PCA) of 20 individuals. Genome-wide SNP-based PCA revealed no clustering patterns indicative of share ancestry, with samples broadly dispersed across genetic space. (E) Heatmap of pairwise PI_HAT values from kinship analysis. All values ranged narrowly from 0.012 to 0.100, well below the threshold for distant relatedness (PI_HAT > 0.1).

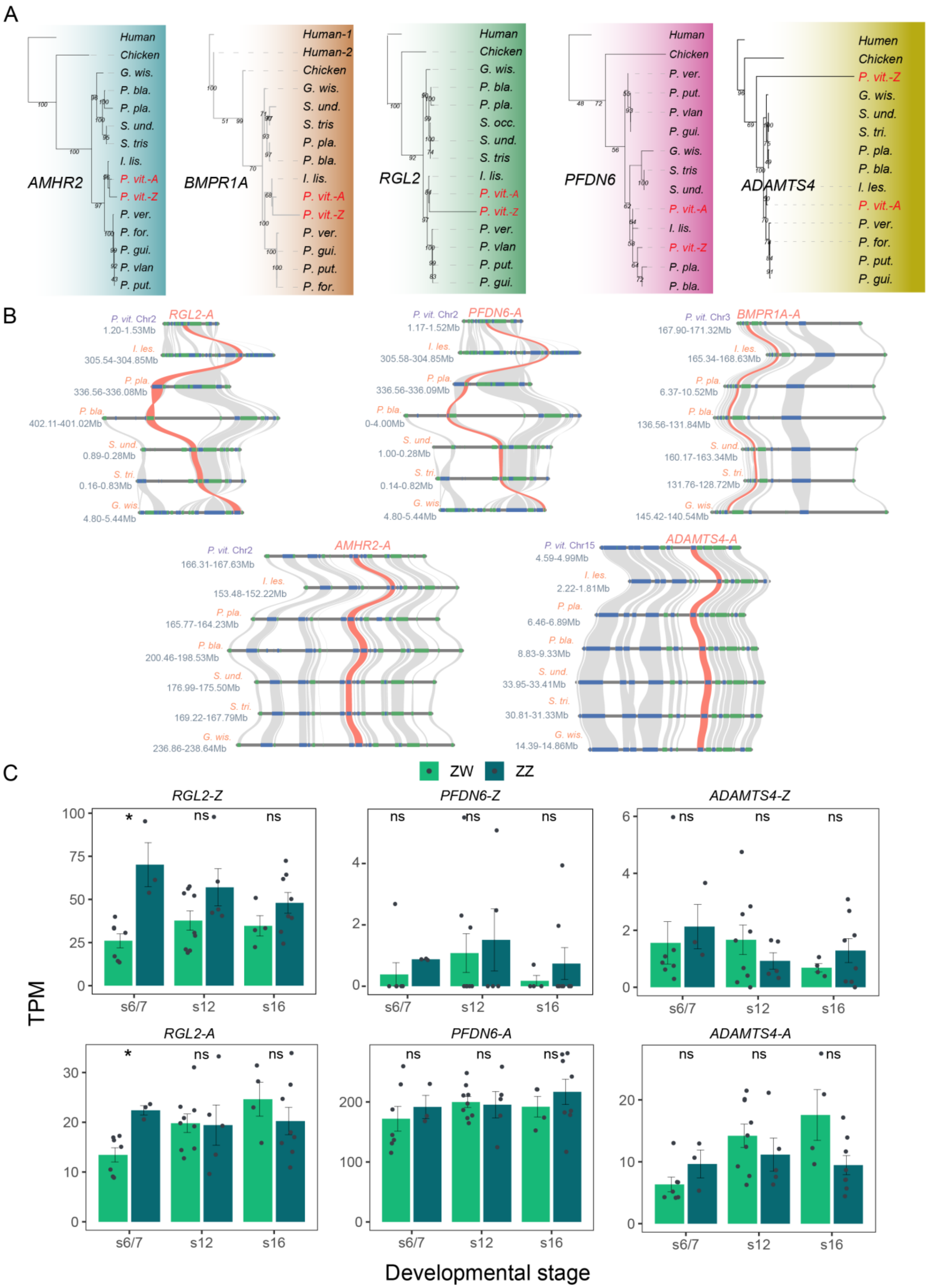

A
AMHR2
BMPR1A
RGL2
PFDN6
ADAMTS4
Human
Chicken
Human-1
Human-2
Humen
B
RGL2-A
PFDN6-A
BMPR1A-A
AMHR2-A
ADAMTS4-A
C
ZW
ZZ
RGL2-Z
PFDN6-Z
ADAMTS4-Z
RGL2-A
PFDN6-A
ADAMTS4-A
TPM
s6/7
s12
s16
Developmental stage

Figure S6: Analysis of SDR genes in *P. vitticeps* (related to Fig. 6). (A) Phylogenetic relationships of five genes in different species. Light red font indicates the two copies of SDR-related genes in *P. vitticeps*. (B) Synteny relationships of autosome SDR genes and their flanking 10 genes in closely related species. The target genes are highlighted in orange, and the surrounding genes are shown in grey. (C) Differential expression of SDR gene pairs between ZZ and ZW during different developmental stages. Benjamini-Hochberg analysis of variance: ns (not significant), *$P<0.05$, **$P<0.01$, ***$P<0.001$.

References

1. Capel, B., *Vertebrate sex determination: evolutionary plasticity of a fundamental switch.* Nature Reviews Genetics, 2017. **18**(11): p. 675-689.
2. Wang, W., et al., *Genomic imprinting-like monoallelic paternal expression determines sex of channel catfish.* Science Advances, 2022. **8**(51): p. eadc8786.
3. Anderson, J.L., et al., *Multiple sex-associated regions and a putative sex chromosome in zebrafish revealed by RAD mapping and population genomics.* PloS one, 2012. **7**(7): p. e40701.
4. Matsumoto, Y. and D. Crews, *Molecular mechanisms of temperature-dependent sex determination in the context of ecological developmental biology.* Molecular and cellular endocrinology, 2012. **354**(1-2): p. 103-110.
5. Li, X.-Y. and J.-F. Gui, *Diverse and variable sex determination mechanisms in vertebrates.* Science China Life Sciences, 2018. **61**: p. 1503-1514.
6. Holleley, C.E., et al., *Sex reversal triggers the rapid transition from genetic to temperature-dependent sex.* Nature, 2015. **523**(7558): p. 79-82.
7. Whiteley, S.L., et al., *Sex determination mode does not affect body or genital development of the central bearded dragon (Pogona vitticeps).* Evodevo, 2017. **8**: p. 1-15.
8. Georges, A., et al., *High-coverage sequencing and annotated assembly of the genome of the Australian dragon lizard Pogona vitticeps.* Gigascience, 2015. **4**(1): p. s13742-015-0085-2.
9. Lawniczak, M.K., et al., *Standards recommendations for the earth BioGenome project.* Proceedings of the National Academy of Sciences, 2022. **119**(4): p. e2115639118.
10. Rhie, A., et al., *Towards complete and error-free genome assemblies of all vertebrate species.* Nature, 2021. **592**(7856): p. 737-746.
11. Young, M., et al., *Molecular cytogenetic map of the central bearded dragon, Pogona vitticeps (Squamata: Agamidae).* Chromosome Research, 2013. **21**: p. 361-374.
12. Waters, P.D., et al., *Microchromosomes are building blocks of bird, reptile, and mammal chromosomes.* Proceedings of the National Academy of Sciences, 2021. **118**(45): p. e2112494118.
13. Deakin, J.E., et al., *Anchoring genome sequence to chromosomes of the central bearded dragon (Pogona vitticeps) enables reconstruction of ancestral squamate macrochromosomes and identifies sequence content of the Z chromosome.* BMC genomics, 2016. **17**: p. 1-15.

14. Quinn, A.E., et al., *Extension, single-locus conversion and physical mapping of sex chromosome sequences identify the Z microchromosome and pseudo-autosomal region in a dragon lizard, Pogona vitticeps.* Heredity, 2010. **104**(4): p. 410-417.
15. Zhang, J.-Y., et al., *A single-molecule nanopore sequencing platform.* bioRxiv, 2024: p. 2024.08. 19.608720.
16. Hu, J., et al., *NextDenovo: an efficient error correction and accurate assembly tool for noisy long reads.* Genome Biology, 2024. **25**(1): p. 107.
17. Roach, M.J., S.A. Schmidt, and A.R. Borneman, *Purge Haplotigs: allelic contig reassignment for third-gen diploid genome assemblies.* BMC bioinformatics, 2018. **19**(1): p. 1-10.
18. Hu, J., et al., *NextPolish: a fast and efficient genome polishing tool for long-read assembly.* Bioinformatics, 2020. **36**(7): p. 2253-2255.
19. Zhou, C., S.A. McCarthy, and R. Durbin, *YaHS: yet another Hi-C scaffolding tool.* Bioinformatics, 2023. **39**(1): p. btac808.
20. Durand, N.C., et al., *Juicer provides a one-click system for analyzing loop-resolution Hi-C experiments.* Cell systems, 2016. **3**(1): p. 95-98.
21. Zhou, Y., et al., *Profiles of telomeric repeats in Insecta reveal diverse forms of telomeric motifs in Hymenopterans.* Life Science Alliance, 2022. **5**(7).
22. Xu, M., et al., *TGS-GapCloser: a fast and accurate gap closer for large genomes with low coverage of error-prone long reads.* GigaScience, 2020. **9**(9): p. giaa094.
23. Simão, F.A., et al., *BUSCO: assessing genome assembly and annotation completeness with single-copy orthologs.* Bioinformatics, 2015. **31**(19): p. 3210-3212.
24. Huang, N. and H. Li, *compleasm: a faster and more accurate reimplementation of BUSCO.* Bioinformatics, 2023. **39**(10): p. btad595.
25. Srikulnath, K., et al., *Why do some vertebrates have microchromosomes?* Cells, 2021. **10**(9): p. 2182.
26. Pinto, B.J., et al., *A lizard is never late: squamate genomics as a recent catalyst for understanding sex chromosome and microchromosome evolution.* Journal of Heredity, 2023. **114**(5): p. 445-458.
27. Lisachov, A.P. and P.M. Borodin, *Microchromosome polymorphism in the sand lizard, Lacerta agilis Linnaeus, 1758 (Reptilia, Squamata).* Comparative Cytogenetics, 2016. **10**(3): p. 387.
28. Westfall, A.K., et al., *A chromosome-level genome assembly for the eastern fence lizard (Sceloporus undulatus), a reptile model for physiological and evolutionary ecology.* GigaScience, 2021. **10**(10): p. giab066.
29. Ezaz, T., et al., *The dragon lizard Pogona vitticeps has ZZ/ZW micro-sex chromosomes.* Chromosome Research, 2005. **13**: p. 763-776.
30. Perry, B.W., et al., *Microchromosomes exhibit distinct features of vertebrate chromosome structure and function with underappreciated ramifications for genome evolution.* Molecular Biology and Evolution, 2021. **38**(3): p. 904-910.
31. Liu, J., et al., *A new emu genome illuminates the evolution of genome configuration and nuclear architecture of avian chromosomes.* Genome Research, 2021. **31**(3): p. 497-511.
32. Jain, C., et al., *Long-read mapping to repetitive reference sequences using Winnowmap2.* Nature Methods, 2022. **19**(6): p. 705-710.
33. Li, H., *Minimap2: pairwise alignment for nucleotide sequences.* Bioinformatics, 2018. **34**(18): p. 3094-3100.

34. Shin, S.C., et al., *Advantages of single-molecule real-time sequencing in high-GC content genomes.* PloS one, 2013. **8**(7): p. e68824.
35. Schramm, L. and N. Hernandez, *Recruitment of RNA polymerase III to its target promoters.* Genes & development, 2002. **16**(20): p. 2593-2620.
36. Pachano, T., et al., *Orphan CpG islands amplify poised enhancer regulatory activity and determine target gene responsiveness.* Nature genetics, 2021. **53**(7): p. 1036-1049.
37. Guigó, R., *Genome annotation: From human genetics to biodiversity genomics.* Cell Genomics, 2023. **3**(8).
38. Yao, Z.T., et al., *New insights into the interplay between long non‐coding RNAs and RNA‐binding proteins in cancer.* Cancer communications, 2022. **42**(2): p. 117-140.
39. Xu, Q., et al., *Systematic comparison of lncRNAs with protein coding mRNAs in population expression and their response to environmental change.* BMC Plant Biology, 2017. **17**: p. 1-15.
40. Palmer, D.H., et al., *How to identify sex chromosomes and their turnover.* Molecular ecology, 2019. **28**(21): p. 4709-4724.
41. Jarmińska-Jackowiak, T., A. Warenik-Szymankiewicz, and W. Trzeciak, *Anti-Mullerian hormone. Structure and role in sexual differentiation.* Ginekologia Polska, 1995. **66**(1): p. 51-58.
42. Josso, N., et al., *AMH and AMH receptor defects in persistent Müllerian duct syndrome.* Human reproduction update, 2005. **11**(4): p. 351-356.
43. Jamin, S.P., et al., *Requirement of Bmpr1a for Müllerian duct regression during male sexual development.* Nature genetics, 2002. **32**(3): p. 408-410.
44. Wagner, S., et al., *Gene expression of male pathway genes sox9 and amh during early sex differentiation in a reptile departs from the classical amniote model.* BMC genomics, 2023. **24**(1): p. 243.
45. Gable, S.M., et al., *The state of squamate genomics: past, present, and future of genome research in the most speciose terrestrial vertebrate order.* Genes, 2023. **14**(7): p. 1387.
46. *reptile-database.org*. 6 October 2021; Available from: http://www.reptile-database.org/db-info/SpeciesStat.html.
47. Zhang, X., et al., *Sex-specific splicing of Z-and W-borne nr5a1 alleles suggests sex determination is controlled by chromosome conformation.* Proceedings of the National Academy of Sciences, 2022. **119**(4): p. e2116475119.
48. Song, W., et al., *A duplicated amh is the master sex-determining gene for Sebastes rockfish in the Northwest Pacific.* Open Biology, 2021. **11**(7): p. 210063.
49. Cortez, D., et al., *Origins and functional evolution of Y chromosomes across mammals.* Nature, 2014. **508**(7497): p. 488-493.
50. Martínez, P., et al., *Genetic architecture of sex determination in fish: applications to sex ratio control in aquaculture.* Frontiers in genetics, 2014. **5**: p. 340.
51. Hattori, R.S., et al., *A Y-linked anti-Müllerian hormone duplication takes over a critical role in sex determination.* Proceedings of the National Academy of Sciences, 2012. **109**(8): p. 2955-2959.
52. Matsubara, K., et al., *ZW sex chromosomes in Australian dragon lizards (Agamidae) originated from a combination of duplication and translocation in the nucleolar organising region.* Genes, 2019. **10**(11): p. 861.

53. Chen, Y., et al., *SOAPnuke: a MapReduce acceleration-supported software for integrated quality control and preprocessing of high-throughput sequencing data.* Gigascience, 2018. **7**(1): p. gix120.
54. Marçais, G. and C. Kingsford, *A fast, lock-free approach for efficient parallel counting of occurrences of k-mers.* Bioinformatics, 2011. **27**(6): p. 764-770.
55. Vurture, G.W., et al., *GenomeScope: fast reference-free genome profiling from short reads.* Bioinformatics, 2017. **33**(14): p. 2202-2204.
56. Marijon, P., R. Chikhi, and J.-S. Varré, *yacrd and fpa: upstream tools for long-read genome assembly.* Bioinformatics, 2020. **36**(12): p. 3894-3896.
57. Zhang, H., et al., *Fast alignment and preprocessing of chromatin profiles with Chromap.* Nature communications, 2021. **12**(1): p. 6566.
58. Quinlan, A.R. and I.M. Hall, *BEDTools: a flexible suite of utilities for comparing genomic features.* Bioinformatics, 2010. **26**(6): p. 841-842.
59. Rhie, A., et al., *Merqury: reference-free quality, completeness, and phasing assessment for genome assemblies.* Genome biology, 2020. **21**: p. 1-27.
60. Li, H., *Aligning sequence reads, clone sequences and assembly contigs with BWA-MEM.* arXiv preprint arXiv:1303.3997, 2013.
61. Li, H., et al., *The sequence alignment/map format and SAMtools.* bioinformatics, 2009. **25**(16): p. 2078-2079.
62. Flynn, J.M., et al., *RepeatModeler2 for automated genomic discovery of transposable element families.* Proceedings of the National Academy of Sciences, 2020. **117**(17): p. 9451-9457.
63. Tarailo - Graovac, M. and N. Chen, *Using RepeatMasker to identify repetitive elements in genomic sequences.* Current protocols in bioinformatics, 2009. **25**(1): p. 4.10. 1-4.10. 14.
64. Benson, G., *Tandem repeats finder: a program to analyze DNA sequences.* Nucleic acids research, 1999. **27**(2): p. 573-580.
65. Kim, D., et al., *Graph-based genome alignment and genotyping with HISAT2 and HISAT-genotype.* Nature biotechnology, 2019. **37**(8): p. 907-915.
66. Kovaka, S., et al., *Transcriptome assembly from long-read RNA-seq alignments with StringTie2.* Genome biology, 2019. **20**: p. 1-13.
67. Stanke, M., et al., *AUGUSTUS: ab initio prediction of alternative transcripts.* Nucleic acids research, 2006. **34**(suppl_2): p. W435-W439.
68. Keilwagen, J., F. Hartung, and J. Grau, *GeMoMa: homology-based gene prediction utilizing intron position conservation and RNA-seq data.* Gene prediction: Methods and protocols, 2019: p. 161-177.
69. Prjibelski, A.D., et al., *Accurate isoform discovery with IsoQuant using long reads.* Nature Biotechnology, 2023. **41**(7): p. 915-918.
70. Wucher, V., et al., *FEELnc: a tool for long non-coding RNA annotation and its application to the dog transcriptome.* Nucleic acids research, 2017. **45**(8): p. e57-e57.
71. Kang, Y.-J., et al., *CPC2: a fast and accurate coding potential calculator based on sequence intrinsic features.* Nucleic acids research, 2017. **45**(W1): p. W12-W16.
72. Liao, Y., G.K. Smyth, and W. Shi, *featureCounts: an efficient general purpose program for assigning sequence reads to genomic features.* Bioinformatics, 2014. **30**(7): p. 923-930.
73. Chan, P.P. and T.M. Lowe, *tRNAscan-SE: searching for tRNA genes in genomic sequences*. 2019: Springer.

74. Powell, D., et al., *The genome of the Australian water dragon (Intellagama lesueurii), an agamid model for urban adaptation.* Journal of Heredity, 2024: p. esae054.
75. Qi, Y., et al., *Chromosome-level genome assembly of Phrynocephalus forsythii using third-generation DNA sequencing and Hi-C analysis.* DNA Research, 2023. **30**(2): p. dsad003.
76. Lu, B., et al., *Genetic basis and evolutionary forces of sexually dimorphic color variation in a toad-headed agamid lizard.* Molecular Biology and Evolution, 2024. **41**(3): p. msae054.
77. Richmond, J.Q., et al., *Reference genome of an iconic lizard in western North America, Blainville's horned lizard Phrynosoma blainvillii.* Journal of Heredity, 2023. **114**(4): p. 410-417.
78. Koochekian, N., et al., *A chromosome-level genome assembly and annotation of the desert horned lizard, Phrynosoma platyrhinos, provides insight into chromosomal rearrangements among reptiles.* GigaScience, 2022. **11**: p. giab098.
79. Bedoya, A.M. and A.D. Leaché, *Characterization of a pericentric inversion in plateau fence lizards (Sceloporus tristichus): evidence from chromosome-scale genomes.* G3, 2021. **11**(2): p. jkab036.
80. Nurk, S., et al., *The complete sequence of a human genome.* Science, 2022. **376**(6588): p. 44-53.
81. McGinnis, S. and T.L. Madden, *BLAST: at the core of a powerful and diverse set of sequence analysis tools.* Nucleic acids research, 2004. **32**(suppl_2): p. W20-W25.
82. Birney, E., M. Clamp, and R. Durbin, *GeneWise and genomewise.* Genome research, 2004. **14**(5): p. 988-995.
83. Katoh, K., et al., *MAFFT: a novel method for rapid multiple sequence alignment based on fast Fourier transform.* Nucleic acids research, 2002. **30**(14): p. 3059-3066.
84. Capella-Gutiérrez, S., J.M. Silla-Martínez, and T. Gabaldón, *trimAl: a tool for automated alignment trimming in large-scale phylogenetic analyses.* Bioinformatics, 2009. **25**(15): p. 1972-1973.
85. Nguyen, L.-T., et al., *IQ-TREE: a fast and effective stochastic algorithm for estimating maximum-likelihood phylogenies.* Molecular biology and evolution, 2015. **32**(1): p. 268-274.
86. Zhang, Z., et al., *KaKs_Calculator: calculating Ka and Ks through model selection and model averaging.* Genomics, proteomics and bioinformatics, 2006. **4**(4): p. 259-263.
87. Dobin, A., et al., *STAR: ultrafast universal RNA-seq aligner.* Bioinformatics, 2013. **29**(1): p. 15-21.
88. Love, M.I., W. Huber, and S. Anders, *Moderated estimation of fold change and dispersion for RNA-seq data with DESeq2.* Genome biology, 2014. **15**: p. 1-21.

 Click here to access/download;Table;Supplementary Tables.xlsx

Table S1. The summary statistics of sequencing data generated for [illegible]

Data type	Instrument/Platform
CycloneSEQ long-read WGS data	CycloneSEQ
DNBSEQ short-read WGS data	DNBSEQ
CycloneSEQ based Pore-C data	CycloneSEQ
Hi-C data	DNBSEQ
CycloneSEQ long-read RNA-seq data	CycloneSEQ
DNBSEQ short-read RNA-seq data	DNBSEQ

genome assembly and annotation of *Pogona vitticeps*.

Sample/Tissue	No.of Libraries	Flowcell numbers
muscle and lung	3	36
muscle of neck	2	2
muscle of tail	2	2
muscle	1	1
muscle	1	1
muscle	1	1
muscle	1	1
muscle	1	1
liver	1	1
liver	1	1
liver	1	1
liver	1	1
liver	1	1
liver	1	1
muscle	1	1
muscle	1	1
muscle	1	1
liver	6	6
muscle (Hi-C_1 data)	1	1
muscle (Hi-C_2 data)	1	1
eye	3	3
kidney	4	4
live	3	3
lung	4	3
muscle	3	4
testis	3	3
brain	5	5
heart	3	3
brain	1	1
eye	1	1
heart	1	1
kidney	1	1
liver	1	1
lung	1	1
muscle	1	1
testis	1	1

Accession (NCBI)	Accession (CNSA)
SAMN44670087	CNS1198625
SRR31361869/SRR31361868	CNS1065816
SRR31361871/SRR31361870	CNS1065815
	CNS1317895
	CNS1317896
	CNS1317897
	CNS1317898
	CNS1317899
	CNS1317889
	CNS1317890
	CNS1317891
	CNS1317892
	CNS1317893
	CNS1317894
	CNS1317887
	CNS1317888
	CNS1317885
SRR31361885	CNS1198626
SRR31361875/SRR31361876	CNS1065813
SRR31361872/SRR31361873	CNS1065814
SAMN44670089	CNS1198628
SAMN44670090	CNS1198630
SAMN44670091	CNS1198631
SAMN44670092	CNS1198632
SAMN44670093	CNS1198633
SAMN44670094	CNS1198634
SRR31361862	CNS1198627
SRR31361861	CNS1198629
SRR31361884	CNS1065805
SRR31361883	CNS1065806
SRR31361882	CNS1065807
SRR31361881	CNS1065808
SRR31361880	CNS1065809
SRR31361879	CNS1065810
SRR31361878	CNS1065811
SRR31361877	CNS1065812

Mean length (bp)	Reads N50 (bp)	Total raw Bases (Gb)
13,499	26,582	398.19
-	-	242.40
-	-	264.20
-	-	154.12
-	-	165.34
-	-	167.40
-	-	206.20
-	-	149.58
-	-	187.72
-	-	186.02
-	-	176.90
-	-	149.00
-	-	121.54
-	-	148.06
-	-	257.58
-	-	246.94
-	-	162.12
1,681	2,793	59.42
-	-	288.24
-	-	323.33
-	-	23.92
-	-	25.13
-	-	21.83
-	-	27.80
-	-	22.26
-	-	25.28
-	-	73.88
-	-	45.91
-	-	259.94
-	-	276.56
-	-	276.42
-	-	251.82
-	-	249.12
-	-	243.84
-	-	168.49
-	-	254.40

Total clean bases (Gb)	Clean data coverage (X)	Application
104.55	61.50	Genome assembly
218.40	128.47	Genome survey
238.60	140.35	
153.76	85.90	Sex Determination Analysis
165.04	92.20	
167.01	93.30	
205.85	115.00	
149.29	83.40	
187.41	104.70	
185.62	103.70	
176.49	98.60	
148.75	83.10	
121.36	67.80	
147.85	82.60	
257.04	143.60	
246.48	137.70	
161.82	90.40	
-	-	Chromosome construction
259.44	152.61	Chromosome construction
291.26	171.33	
-	-	Genome annotation
-	-	
-	-	
-	-	
-	-	
-	-	
-	-	
-	-	
233.46	-	Genome annotation
248.37	-	
248.07	-	
226.11	-	
223.86	-	
219.01	-	
151.27	-	
228.58	-	

Table S2. The detailed statistics of CycloneSEQ long-read RNA-seq data.

Tissue	Chip ID		Reads number	Max read length (bp)
brain	WTB400000E2A	SRR31361862	21,738,733	540,305
	WTB400000F31		20,691,877	368,665
	WTB400000F3B		23,866,521	384,843
	WTB40000101B		13,810,418	272,924
	WTB4000010B6		10,382,528	436,488
eye	WT32H0000594		7,847,488	478,946
	WT32H0000964		6,667,141	789,917
	WTB320000202		6,203,658	349,259
heart	WTB400000F97	SRR31361861	22,931,313	248,932
	WTB400000FFB		21,830,092	530,098
	WTB400001298		15,648,151	180,443
kidney	WT32H0000068		6,298,454	607,030
	WT32H0000224		5,915,303	497,078
	WT32H0000829		4,964,764	576,127
	WTB32000014A		5,929,059	544,970
liver	WT32H0000294		6,954,810	545,516
	WT32H0000608		7,451,290	536,080
	WT32H0000794		7,864,264	613,703
lung	WT32H0000102		9,371,690	472,699
	WT32H0000830		11,223,664	577,115
	WTB32000023D		7,627,271	649,700
muscle	WT32H0000189		4,736,247	532,007
	WT32H0000307		5,193,256	554,077
	WTB320000178		6,081,954	486,518
	WTB32000023F		6,410,900	577,431
testis	WTB32000019A		6,947,043	461,551
	WTB3200002BF		7,549,256	539,719
	WTB320000302		5,548,677	461,802

Reads N50 (bp)	Reads mean length (bp)	Reads median length (bp	Total bases (Gb)
1,093	813	597	17.68
1,073	800	586	16.55
1,095	815	599	19.46
1,106	827	607	11.42
1,127	845	630	8.77
1,550	1,147	880	9.00
1,552	1,143	872	7.62
1,585	1,178	903	7.31
972	758	569	17.39
965	755	565	16.48
988	770	576	12.04
1,459	1,055	808	6.65
1,516	1,109	858	6.56
1,545	1,129	871	5.60
1,478	1,067	816	6.32
1,290	976	727	6.79
1,267	969	723	7.22
1,308	994	740	7.82
1,259	986	729	9.24
1,249	982	730	11.02
1,257	988	733	7.54
1,209	943	691	4.47
1,290	1,006	735	5.22
1,296	999	724	6.08
1,272	1,012	747	6.49
1,701	1,262	991	8.77
1,690	1,258	989	9.50
1,704	1,264	993	7.02

GC content (%)
41
41
41
41
41
41
41
41
40
40
40
42
42
42
42
41
41
41
41
41
41
40
40
40
39
41
41
41

Table S3. Detailed statistics for DNBSEQ short-read RNA-seq da

Tissue	NCBI accession No.	Raw reads number
**brain**	**SRR31361884**	2,599,363,998
**eye**	**SRR31361883**	2,765,574,142
**heart**	**SRR31361882**	2,764,172,400
**kidney**	**SRR31361881**	2,518,157,150
**liver**	**SRR31361880**	2,491,237,606
**lung**	**SRR31361879**	2,438,409,538
**muscle**	**SRR31361878**	1,684,864,416
**testis**	**SRR31361877**	2,543,966,726

ata from eight tissues.

Clean reads number	Total raw bases (Gb)	Total clean bases (Gb)
2,596,609,506	259.94	233.46
2,762,708,832	276.56	248.37
2,760,293,230	276.42	248.07
2,515,310,708	251.82	226.11
2,489,409,124	249.12	223.86
2,435,575,540	243.84	219.01
1,682,409,186	168.49	151.27
2,541,618,112	254.40	228.58

Raw data Q20 (%)	Clean data Q20 (%)	Raw data Q30 (%)	an data Q30 (%)
97	97	91	90
97	97	91	91
97	97	90	90
97	97	90	90
98	98	92	91
97	97	91	91
97	97	91	91
97	97	91	91

Table S4. Estimation of genome size and heterozygosity of *Pogona*

Data	*k*	Total number of *k* -mers
**DNBSEQ short-read WGS data of the muscle of neck**	19	177,020,064,126
	21	172,421,809,376
	23	167,823,570,585
	25	163,225,346,245
	27	158,627,135,903
**DNBSEQ short-read WGS data of the muscle of tail**	19	193,321,192,868
	21	188,299,515,148
	23	183,277,854,245
	25	178,256,208,188
	27	173,234,576,379
**Illumina short-read WGS data (NCBI accession number : ERR409943, ERR409944, ERR409945, ERR409946, and ERR409947)**	19	82,801,106,820
	21	81,067,927,867
	23	79,355,856,174
	25	77,664,892,605
	27	75,973,931,659

***vitticeps* by *k*-mer analysis.**

Minimum coverage (X)	Number of erroneous *k*-mers	Homozygous peak
13	3,075,586,360	103
12	3,618,094,304	100
11	3,922,322,682	97
11	4,166,773,555	94
10	4,374,295,026	91
14	3,218,696,282	113
13	3,798,750,876	110
12	4,124,369,191	106
12	4,386,007,441	103
11	4,608,644,448	100
6	1,820,204,988	48
6	2,102,198,339	46
6	2,295,441,241	45
5	2,415,163,433	44
5	2,555,019,877	43

Estimated genome size (Gb)	GenomeScope2	
	Estimated genome size (Gb)	Estimated heterozygosity (%)
1.69	1.64	1.64
1.69	1.64	1.66
1.69	1.64	1.64
1.69	1.64	1.62
1.70	1.63	1.60
1.68	1.64	1.65
1.68	1.64	1.67
1.69	1.64	1.65
1.69	1.64	1.63
1.69	1.64	1.61
1.69	1.61	1.67
1.72	1.61	1.69
1.71	1.61	1.67
1.71	1.60	1.64
1.71	1.60	1.61

% Repeat
30.66
24.21
21.86
20.30
19.05
21.78
15.63
13.93
12.97
12.26
31.30
25.21
22.94
21.39
20.14

Table S5. Improvement in continuity and completeness of genome assembly generat

Assembly step	Main purpose
1	Contig level assembly by NextDenovo
2	Remove heterozygous contigs by purge_haplotigs
3	Two rounds of polishing by NextPolish
4	Anchor the assembly to pseudo-chromosomes by Cyclone based Pore-C reads
5	Correct telomeric regions
6	Gap filling by GapCloser
7	Examine the correctness of each closed gaps

ted by each of the seven assembly steps as stated in main text.

Assembly size (bp)	Number of sequences	Number of gaps	Gap length (bp)
1,813,085,128	106	0	0
1,796,337,866	74	0	0
1,795,080,159	74	0	0
1,793,030,482	16	67	6,700
1,793,295,301	16	69	6,900
1,793,931,292	16	20	2,000
1,793,642,912	16	28	2,800

Assembly statistics			
Contig N50 (bp)	Contig N90 (bp)	Scaffold N50 (bp)	Scaffold N90 (bp)
60,857,226	12,375,454	60,857,226	12,375,454
60,857,226	13,185,222	60,857,226	13,185,222
60,808,468	13,173,302	60,808,468	13,173,302
60,808,468	12,373,276	274,777,790	29,148,224
60,808,468	12,373,276	274,780,347	29,177,152
271,395,404	27,452,928	274,873,614	29,217,309
202,516,942	13,986,680	274,873,533	29,176,711

		BU
**Consensus quality value (QV)**	**Completeness based on *k* -mer**	**Complete BUSCOs (%)**
29.6648	78.2779	91.9
29.6859	78.1057	91.8
36.5281	79.7766	97.7
36.5653	79.7450	97.6
36.3514	79.7210	97.6
36.3531	79.7386	97.6
36.3611	79.7373	97.6

SCO assessment (Sauropsida conserved genes num = 7,480)		
Duplicate BUSCOs (%)	Fragmented BUSCOs (%)	Missing BUSCOs (%)
1.3	2.2	5.9
1.2	2.2	6.0
1.2	1.6	1.7
1.2	0.6	1.8
1.2	0.6	1.8
1.2	0.6	1.8
1.2	0.6	1.8

DNBSEQ short-read WGS mapping rate (%)
99.52
99.49
99.50
99.47
99.47
99.47
99.47

DNBSEQ short-read WGS mapping rate in proper pairs (%)
97.44
97.34
97.28
97.25
97.25
97.25
97.25

Reads m
**CycloneSEQ long-read WGS mapping rate (%)**
98.51
98.23
98.25
98.20
98.20
98.20
98.20

napping assessment
Illumina short-read WGS mapping rate in proper pairs (%)
95.57
95.54
95.55
95.52
95.52
95.51
95.52

DNBSEQ short-read RNA-seq mapping rate (%)
75.79
75.66
78.49
78.48
78.44
78.44
78.44

CycloneSEQ long-read RNA-seq mapping rate (%)
49.81
49.81
50.18
50.23
50.21
50.23
50.23

Table S6. Genome features between Pvit2024 and Pvit2015.

Version	Pvit2024 in this study	Pvit2015 from Georges *et al.*
Number of contigs	43	104,295
Number of scaffolds	16	13,749
Number of chromosomes	16	-
Assembly size (bp)	1,793,642,912	1,716,675,060
Number of gaps	28	90,546
Gap length (bp)	2,800	68,427,839
Longest (bp)	319,961,577	14,681,335
contig N50 (bp)	202,516,942	33,316
contig L50	4	14,807
contig N90 (bp)	13,986,680	8,854
contig L90	15	50,668
BUSCOs (N = 7,480)	97.60%	97.00%

Table S7. Summary of comprehensive assessment of genome completenes

Species	GenBank assembly	Genome size (bp)
Pogona vitticeps	-	1,793,642,912
Ahaetulla prasina	GCA_028640845.1	1,772,420,080
Anniella stebbinsi	GCA_029215795.1	1,891,076,299
Anolis carolinensis	GCA_035594765.1	1,850,740,401
Anolis sagrei	GCA_037176765.1	1,950,830,970
Arizona elegans	GCA_022577455.1	1,842,551,953
Aspidoscelis tigris	GCA_023333525.1	1,335,668,279
Azemiops feae	GCA_023970755.1	1,557,776,103
Bothrops alternatus	GCA_034064705.1	1,739,305,912
Bradypodion pumilum	GCA_035047305.1	2,486,266,449
Bradypodion ventrale	GCA_035047345.1	2,373,187,502
Bungarus multicinctus	GCA_023653725.1	1,593,755,901
Candoia aspera	GCA_035149785.1	1,530,810,380
Coluber constrictor foxii	GCA_038048715.1	1,668,806,547
Crotalus helleri	GCA_024509165.1	1,628,461,744
Crotalus tigris	GCA_016545835.1	1,612,002,213
Cryptoblepharus egeriae	GCA_030015325.1	1,409,969,067
Cyclura pinguis	GCA_030412105.1	1,983,631,412
Darevskia armeniaca	GCA_034641395.1	1,925,748,154
Darevskia mixta	GCA_034641385.1	1,408,516,084
Darevskia nairensis	GCA_034642065.1	1,772,584,127
Darevskia unisexualis	GCA_032766585.1	1,398,207,223
Diadophis punctatus	GCA_023053685.1	1,783,023,707
Elgaria multicarinata	GCA_023053635.2	1,790,500,004
Erythrolamprus reginae	GCA_031021105.1	1,964,049,041
Eublepharis macularius	GCA_028583425.1	2,237,397,408
Euleptes europaea	GCA_029931755.1	1,775,520,415
Furcifer pardalis	GCA_030440675.1	1,609,777,135
Gambelia wislizenii	GCA_030847615.1	2,466,589,883
Gekko gecko	GCA_029375565.1	2,741,107,181
Heloderma charlesbogerti	GCA_026122225.1	2,303,480,078
Hemicordylus capensis	GCA_027244095.1	2,294,751,221
Heteronotia binoei	GCA_032191835.1	2,633,472,656
Hydrophis curtus	GCA_019472885.1	1,964,827,820
Hydrophis cyanocinctus	GCA_019473425.1	1,980,712,740
Hydrophis elegans	GCA_033807725.1	2,010,336,133
Hydrophis major	GCA_033807585.1	2,166,703,382
Hydrophis ornatus	GCA_037055225.1	1,926,171,689
Intellagama lesueurii	GCA_037013535.1	1,817,723,846
Lacerta agilis	GCA_009819535.1	1,391,404,169
Lepidodactylus listeri	GCA_029233225.1	2,348,847,456

Liasis olivaceus	GCA_030867105.1	1,486,119,866
Masticophis lateralis	GCA_030761175.1	1,504,000,112
Natrix natrix natrix	GCA_029891615.1	1,733,453,643
Natrix helvetica	GCA_029890345.1	1,782,847,860
Pantherophis gloydi	GCA_037215945.1	1,906,104,767
Pantherophis guttatus	GCA_029531705.1	1,708,105,946
Pantherophis spiloides	GCA_037575465.1	1,791,426,338
Phrynocephalus guinanensis	GCA_037367245.1	1,813,735,221
Phrynocephalus putjatai	GCA_037367255.1	1,915,366,425
Phrynocephalus vlangalii	GCA_037367305.1	1,866,892,976
Phrynosoma blainvillii	GCA_026167975.1	1,968,358,621
Pituophis catenifer	GCA_029215655.1	1,749,255,673
Plestiodon gilberti	GCA_026170595.1	1,571,222,493
Podarcis cretensis	GCA_951804945.1	1,507,627,055
Podarcis lilfordi	GCA_947686815.1	1,460,103,102
Podarcis raffonei	GCA_027172205.1	1,513,131,503
Rhineura floridana	GCA_030035675.1	2,307,419,594
Shinisaurus crocodilurus	GCA_021292165.1	2,189,995,079
Thamnophis elegans	GCA_009769535.1	1,672,190,305
Thamnophis sirtalis	GCA_029207655.1	1,683,046,358
Tiliqua scincoides	GCA_035046505.1	1,623,332,312
Urosaurus nigricaudus	GCA_034509955.1	1,870,054,407
Varanus salvator	GCA_023646645.1	1,702,541,867
Vipera latastei	GCA_024294585.1	1,631,568,913
Vipera ursinii	GCA_947247035.1	1,625,023,540
Zootoca vivipara	GCA_963506605.1	1,440,239,386

s for 67 long-read sequenced Squamata species using BUSCO and Compleas

Number of scaffolds	Scaffold N50 (bp)	Scaffold L50	Number of contigs
16	274,873,533	3	43
748	231,952,326	3	1,079
91	276,533,474	3	122
211	288,964,324	3	1,052
29	249,210,491	4	220
140	105,945,816	5	396
74	93,690,953	5	113
-	-	-	4,303
-	-	-	1,557
21	389,612,775	3	39
113	374,919,295	3	135
448	135,406,522	5	2,079
149	209,480,725	3	250
30	141,372,050	4	570
340	68,097,879	9	374
-	-	-	4,228
-	-	-	74
30	291,724,653	3	58
-	-	-	1,656
-	-	-	402
-	-	-	1,805
-	-	-	152
444	83,654,930	5	1,167
34	121,985,326	6	107
400	259,117,534	3	1,791
76	145,573,841	6	137
381	101,915,064	7	558
11	184,669,281	4	23
69	380,060,318	3	96
4,208	190,862,615	6	5,480
-	-	-	3,543
44	359,646,233	3	86
661	150,619,664	7	2,269
711	266,229,956	3	2,109
1,163	264,245,889	3	2,399
3,120	17,836,126	34	3,121
1,320	268,276,564	3	1,797
546	270,035,755	3	973
556	269,041,945	3	877
29	86,565,987	7	408
402	119,873,534	8	461

43	203,662,644	3	116
120	214,958,471	3	297
12,760	1,579,101	295	12,847
-	-	-	1,221
198	160,296,173	4	629
466	63,228,677	10	4,019
-	-	-	369
50	132,691,761	6	1,270
-	-	-	7,767
-	-	-	6,566
52	352,551,559	3	116
426	161,302,193	4	1,029
39	231,322,181	3	54
51	94,621,998	7	98
2,149	89,641,981	7	4,470
27	93,623,253	7	57
76	188,683,971	5	318
1,553	296,945,371	4	2,405
365	100,851,885	6	1,883
226	83,410,609	7	629
350	242,160,784	3	722
327	279,651,831	3	531
858	71,461,993	9	13,056
56	222,489,854	3	825
384	212,821,320	3	2,203
48	96,056,580	7	731

m.

Contig N50 (bp)	Contig L50	Chromosome Number
202,516,942	4	16
23,873,608	23	18
120,289,678	6	20
26,091,274	21	8
43,146,625	14	15
54,101,938	11	-
30,424,254	11	-
1,596,756	258	-
13,867,869	32	18
178,505,980	6	17
174,922,266	6	17
5,437,538	68	-
55,640,180	9	18
75,089,467	7	-
68,097,879	9	-
2,106,662	194	-
109,130,949	4	-
112,814,430	7	20
3,089,098	175	19
15,581,688	25	19
2,900,118	139	19
27,396,532	18	-
8,013,852	52	-
85,762,895	8	23
3,064,756	172	14
80,105,973	9	20
31,964,801	19	21
158,731,830	4	11
138,191,064	6	-
10,858,724	69	19
1,358,783	516	-
160,691,000	6	17
14,376,057	52	22
9,704,100	50	17
18,985,681	27	18
17,470,302	34	-
35,743,180	16	16
17,311,133	37	-
39,629,392	16	-
6,613,248	57	21
64,972,995	12	-

65,226,870	8	17
68,931,034	8	-
1,528,028	307	-
4,622,328	105	-
28,315,120	13	-
3,031,475	142	-
66,322,725	7	-
2,541,092	239	24
3,298,286	152	-
4,113,497	108	-
62,272,472	10	17
33,264,011	14	-
127,798,174	5	-
65,743,000	8	20
1,481,499	231	21
61,439,401	10	20
18,156,940	38	22
3,338,364	196	16
4,620,601	83	18
14,321,763	33	-
33,113,817	14	16
23,281,897	24	14
1,316,942	87	20
44,839,567	11	18
2,137,891	215	20
3,447,840	122	19

Sequencing technology
CycloneSEQ; DNBSEQ
MGISEQ-2000;OxfordNanoporePromethION;IlluminaHiSeq
PacBioSequelIIe;IlluminaNovaSeq;DovetailOmniC
PacBioSequel
PacBioSequelIIHiFi;ArimaHi-Cv2GCA_037176765.1PacBioSequelIIHiFi;ArimaH
PacBioSequelII;PacBioSequelIIe;DovetailOmniC;IlluminaNovaSeq
PacBioSequelIIe;DovetailsOmniC;IlluminaNovaSeq
IlluminaHiSeq;PacBioSequel
PacBioHiFi
PacBioSequel;IlluminaHiSeq
PacBioSequel;IlluminaHiSeq
PacBioSequel
PacBioSequelIIHiFi;ArimaHi-Cv2
PacBioSequel;IlluminaNovaSeq
PacBioSequelII;PacBioSequelIIe;DovetailsOmniC;IlluminaNovaSeq
PacBioSequel;Illumina
PacBioHiFi
PacBioSequelIIHiFi;BionanoGenomicsDLS;DovetailOmniC
PacBioHiFi
PacBioHiFi
PacBioHiFi
PacBioHiFi
PacBioSequelIIe;DovetailsOmniC;IlluminaNovaSeq
IlluminaNovaSeq6000;PacBioSequelII;DovetailOmniC
PacBioSequelIIHiFi;ArimaHi-Cv2
PacBioSequel
PacBioSequelIIHiFi;BionanoGenomicsDLS;ArimaHi-Cv2
PacBioSequelIIe;DNBSEQ-T7
IlluminaNovaSeq6000;PacbioSequelIIe;DovetailOmniC
OxfordNanopore;IlluminaNextSeq
PacBioSequelII
PacBioSequelIIe;DovetailOmni-C
PacBioSequel
PacBioSequel;IlluminaHiSeq
PacBioSequel;IlluminaHiSeq
OxfordNanoporePromethION;MGIG400
PacBioRSII
OxfordNanoporePromethION;IlluminaNovaseq
PacBioSequel
PacBioSequelICLR;IlluminaNovaSeq;ArimaGenomicsHi-C;BionanoGenomicsDL
PacBioHiFi;IlluminaNovaSeq6000

PacBioSequelIIHiFi;BionanoGenomicsDLS;ArimaHi-Cv2
IlluminaNovaSeq6000;PacBioSequelIIe;DovetailOmniC
PacBioSequel;IlluminaNovaSeq
PacBioSequel;IlluminaNovaSeq
PacBioSequel;IlluminaNovaSeq
PacBio
PacBioRevio
PacBioSequel
PacBioSequel
PacBioSequel
PacBioSequelII;IlluminaNovaSeq;DovetailGenomicsOmni-C
IlluminaNovaSeq;DovetailOmniC;PacBioSequelIIe
PacBioSequelII;IlluminaNovaSeq;DovetailsOmniC
PacBio,Arima2
ONT,10Xlinkedreads,omniC
PacBioSequelIIHiFi;BionanoDLS;ArimaHi-Cv2
PacBioSequelIIHiFi;BionanoDLS;ArimaHi-Cv2
PacBio;IlluminaHiSeq
PacBioSequelICLR;IlluminaNovaSeq;ArimaGenomicsHi-C;BionanoGenomicsDL[illegible]
IlluminaNovaSeq;DovetailOmniC;PacBioSequelII
PacBioSequelIIHiFi;ArimaHi-Cv2
PacBioSequel
IlluminaNovaSeq;PacBioSequel
PacBioSequelIIHiFi;BionanoDLE1;ArimaHi-C
PacBio,Arima2
PacBio,Arima2

Comprehensive Evaluation of Genomic Completeness Using BUSCO Analysis (N:7480)
C:97.6%[S:96.4%;D:1.2%];F:0.6%;M:1.8%;
C:93.4%[S:92.4%;D:1.0%];F:0.9%;M:5.7%;
C:97.6%[S:96.5%;D:1.1%];F:0.6%;M:1.8%;
C:97.0%[S:95.3%;D:1.7%];F:0.7%;M:2.3%;
C:97.2%[S:93.4%;D:3.8%];F:0.6%;M:2.2%;
C:95.9%[S:94.5%;D:1.4%];F:0.7%;M:3.4%;
C:81.4%[S:80.6%;D:0.8%];F:0.7%;M:17.9%;
C:94.7%[S:92.7%;D:2.0%];F:1.3%;M:4.0%;
C:95.6%[S:87.2%;D:8.4%];F:0.9%;M:3.5%;
C:97.0%[S:95.9%;D:1.1%];F:0.6%;M:2.4%;
C:96.9%[S:95.4%;D:1.5%];F:0.7%;M:2.4%;
C:94.6%[S:92.8%;D:1.8%];F:0.8%;M:4.6%;
C:96.5%[S:95.7%;D:0.8%];F:0.7%;M:2.8%;
C:95.5%[S:94.2%;D:1.3%];F:0.7%;M:3.8%;
C:96.0%[S:94.5%;D:1.5%];F:0.7%;M:3.3%;
C:95.5%[S:93.6%;D:1.9%];F:0.8%;M:3.7%;
C:97.2%[S:95.9%;D:1.3%];F:0.7%;M:2.1%;
C:97.7%[S:96.5%;D:1.2%];F:0.6%;M:1.7%;
C:97.1%[S:56.9%;D:40.2%];F:0.7%;M:2.2%;
C:97.5%[S:94.0%;D:3.5%];F:0.6%;M:1.9%;
C:97.3%[S:76.7%;D:20.6%];F:0.6%;M:2.1%;
C:96.1%[S:94.4%;D:1.7%];F:0.7%;M:3.2%;
C:93.9%[S:92.4%;D:1.5%];F:0.8%;M:5.3%;
C:97.8%[S:96.7%;D:1.1%];F:0.5%;M:1.7%;
C:93.9%[S:91.3%;D:2.6%];F:0.9%;M:5.2%;
C:97.6%[S:95.8%;D:1.8%];F:0.7%;M:1.7%;
C:97.4%[S:95.7%;D:1.7%];F:0.7%;M:1.9%;
C:97.1%[S:96.4%;D:0.7%];F:0.5%;M:2.4%;
C:97.7%[S:96.7%;D:1.0%];F:0.6%;M:1.7%;
C:97.7%[S:95.9%;D:1.8%];F:0.7%;M:1.6%;
C:95.4%[S:93.2%;D:2.2%];F:1.4%;M:3.2%;
C:97.8%[S:96.2%;D:1.6%];F:0.6%;M:1.6%;
C:97.2%[S:93.7%;D:3.5%];F:0.8%;M:2.0%;
C:95.3%[S:92.8%;D:2.5%];F:0.8%;M:3.9%;
C:95.2%[S:93.0%;D:2.2%];F:0.9%;M:3.9%;
C:95.1%[S:94.1%;D:1.0%];F:0.7%;M:4.2%;
C:95.4%[S:94.2%;D:1.2%];F:0.8%;M:3.8%;
C:95.5%[S:94.6%;D:0.9%];F:0.7%;M:3.8%;
C:97.8%[S:96.7%;D:1.1%];F:0.7%;M:1.5%;
C:97.2%[S:94.7%;D:2.5%];F:0.6%;M:2.2%;
C:97.7%[S:95.8%;D:1.9%];F:0.6%;M:1.7%;

C:96.9%[S:96.2%;D:0.7%];F:0.6%;M:2.5%;
C:90.9%[S:89.9%;D:1.0%];F:0.7%;M:8.4%;
C:94.4%[S:92.8%;D:1.6%];F:1.6%;M:4.0%;
C:95.4%[S:92.8%;D:2.6%];F:0.7%;M:3.9%;
C:96.0%[S:94.1%;D:1.9%];F:0.7%;M:3.3%;
C:94.8%[S:90.6%;D:4.2%];F:0.9%;M:4.3%;
C:95.5%[S:94.0%;D:1.5%];F:0.7%;M:3.8%;
C:95.5%[S:92.1%;D:3.4%];F:0.8%;M:3.7%;
C:95.3%[S:93.4%;D:1.9%];F:1.0%;M:3.7%;
C:95.1%[S:93.3%;D:1.8%];F:1.0%;M:3.9%;
C:87.8%[S:86.5%;D:1.3%];F:0.6%;M:11.6%;
C:95.6%[S:94.5%;D:1.1%];F:0.8%;M:3.6%;
C:97.8%[S:96.8%;D:1.0%];F:0.5%;M:1.7%;
C:97.6%[S:96.1%;D:1.5%];F:0.5%;M:1.9%;
C:97.4%[S:95.8%;D:1.6%];F:0.6%;M:2.0%;
C:97.6%[S:95.8%;D:1.8%];F:0.5%;M:1.9%;
C:97.4%[S:95.7%;D:1.7%];F:0.6%;M:2.0%;
C:97.8%[S:90.8%;D:7.0%];F:0.6%;M:1.6%;
C:94.1%[S:91.3%;D:2.8%];F:0.9%;M:5.0%;
C:95.6%[S:94.3%;D:1.3%];F:0.7%;M:3.7%;
C:94.7%[S:93.6%;D:1.1%];F:0.7%;M:4.6%;
C:97.3%[S:95.9%;D:1.4%];F:0.7%;M:2.0%;
C:95.7%[S:86.8%;D:8.9%];F:0.9%;M:3.4%;
C:96.0%[S:94.7%;D:1.3%];F:0.6%;M:3.4%;
C:95.2%[S:93.8%;D:1.4%];F:0.9%;M:3.9%;
C:97.2%[S:95.4%;D:1.8%];F:0.7%;M:2.1%;

Comprehensive Evaluation of Genomic Completeness Using Compleasm Analysis (N:7480)
C:98.63%[S:98.11%;D:0.52%;];F:0.19%;M:1.18%;
C:94.47%[S:93.98%;D:0.49%;];F:0.33%;M:5.19%;
C:98.54%[S:98.13%;D:0.41%;];F:0.11%;M:1.35%;
C:98.04%[S:97.29%;D:0.75%;];F:0.32%;M:1.64%;
C:98.10%[S:95.15%;D:2.95%;];F:0.25%;M:1.64%;
C:96.98%[S:96.23%;D:0.75%;];F:0.24%;M:2.78%;
C:82.34%[S:81.93%;D:0.41%;];F:0.39%;M:17.27%;
C:95.60%[S:94.26%;D:1.34%;];F:0.86%;M:3.54%;
C:96.66%[S:89.28%;D:7.38%;];F:0.32%;M:3.02%;
C:97.83%[S:97.46%;D:0.37%;];F:0.24%;M:1.93%;
C:97.80%[S:97.01%;D:0.79%;];F:0.28%;M:1.93%;
C:95.82%[S:94.71%;D:1.11%;];F:0.28%;M:3.90%;
C:97.61%[S:97.42%;D:0.19%;];F:0.23%;M:2.17%;
C:96.56%[S:95.92%;D:0.64%;];F:0.24%;M:3.20%;
C:97.00%[S:96.10%;D:0.90%;];F:0.28%;M:2.73%;
C:96.58%[S:95.35%;D:1.23%;];F:0.35%;M:3.06%;
C:98.21%[S:97.42%;D:0.79%;];F:0.19%;M:1.60%;
C:98.68%[S:98.17%;D:0.51%;];F:0.16%;M:1.16%;
C:98.11%[S:58.48%;D:39.63%;];F:0.29%;M:1.59%;
C:98.49%[S:95.96%;D:2.53%;];F:0.23%;M:1.27%;
C:98.07%[S:78.38%;D:19.69%;];F:0.24%;M:1.68%;
C:97.05%[S:96.15%;D:0.90%;];F:0.27%;M:2.69%;
C:95.07%[S:94.24%;D:0.83%;];F:0.29%;M:4.64%;
C:98.71%[S:98.30%;D:0.41%;];F:0.15%;M:1.14%;
C:95.02%[S:92.91%;D:2.11%;];F:0.41%;M:4.56%;
C:98.72%[S:97.77%;D:0.95%;];F:0.12%;M:1.16%;
C:98.50%[S:97.55%;D:0.95%;];F:0.16%;M:1.34%;
C:98.02%[S:97.82%;D:0.20%;];F:0.21%;M:1.76%;
C:98.73%[S:98.36%;D:0.37%;];F:0.21%;M:1.06%;
C:98.77%[S:97.77%;D:1.00%;];F:0.21%;M:1.02%;
C:96.50%[S:94.84%;D:1.66%;];F:0.80%;M:2.69%;
C:98.74%[S:97.87%;D:0.87%;];F:0.15%;M:1.11%;
C:98.23%[S:95.53%;D:2.70%;];F:0.24%;M:1.52%;
C:96.44%[S:94.51%;D:1.93%;];F:0.28%;M:3.29%;
C:96.45%[S:94.71%;D:1.74%;];F:0.31%;M:3.25%;
C:96.31%[S:95.82%;D:0.49%;];F:0.24%;M:3.45%;
C:96.65%[S:95.87%;D:0.78%;];F:0.25%;M:3.10%;
C:96.75%[S:96.34%;D:0.41%;];F:0.20%;M:3.05%;
C:98.75%[S:98.28%;D:0.47%;];F:0.25%;M:1.00%;
C:98.13%[S:96.74%;D:1.39%;];F:0.23%;M:1.64%;
C:98.68%[S:97.53%;D:1.15%;];F:0.17%;M:1.15%;

C:97.88%[S:97.63%;D:0.25%;];F:0.15%;M:1.97%;
C:91.90%[S:91.34%;D:0.56%;];F:0.33%;M:7.77%;
C:95.61%[S:94.59%;D:1.02%;];F:1.07%;M:3.32%;
C:96.53%[S:94.69%;D:1.84%;];F:0.24%;M:3.22%;
C:96.94%[S:95.70%;D:1.24%;];F:0.27%;M:2.79%;
C:95.89%[S:92.31%;D:3.58%;];F:0.43%;M:3.68%;
C:96.55%[S:95.68%;D:0.87%;];F:0.28%;M:3.17%;
C:96.41%[S:93.64%;D:2.77%;];F:0.40%;M:3.20%;
C:96.25%[S:95.25%;D:1.00%;];F:0.60%;M:3.13%;
C:95.94%[S:95.08%;D:0.86%;];F:0.60%;M:3.46%;
C:88.52%[S:87.89%;D:0.63%;];F:0.29%;M:11.19%;
C:96.81%[S:96.20%;D:0.61%;];F:0.24%;M:2.94%;
C:98.70%[S:98.26%;D:0.44%;];F:0.16%;M:1.14%;
C:98.51%[S:97.81%;D:0.70%;];F:0.17%;M:1.32%;
C:98.47%[S:97.55%;D:0.92%;];F:0.17%;M:1.35%;
C:98.54%[S:97.59%;D:0.95%;];F:0.15%;M:1.31%;
C:98.46%[S:97.47%;D:0.99%;];F:0.20%;M:1.34%;
C:98.73%[S:92.71%;D:6.02%;];F:0.23%;M:1.04%;
C:95.31%[S:93.45%;D:1.86%;];F:0.45%;M:4.24%;
C:96.77%[S:96.11%;D:0.66%;];F:0.23%;M:3.01%;
C:95.57%[S:94.97%;D:0.60%;];F:0.28%;M:4.14%;
C:98.23%[S:97.74%;D:0.49%;];F:0.28%;M:1.48%;
C:96.79%[S:88.38%;D:8.41%;];F:0.35%;M:2.86%;
C:96.86%[S:96.14%;D:0.72%;];F:0.24%;M:2.90%;
C:96.08%[S:95.33%;D:0.75%;];F:0.47%;M:3.44%;
C:98.18%[S:97.38%;D:0.80%;];F:0.27%;M:1.55%;

Table S8. Summary of telomere and centromere location information of *Pogona*

Chromosome	Chromosome Length (bp)	Telomere left start	Telomere left end
Chr1	371,574,468	1,169	17,320
Chr2	320,472,310	3	30,828
Chr3	274,873,533	3	12,882
Chr4	247,739,185	3	23,748
Chr5	202,516,942	904	19,902
Chr6	126,726,790	3	13,844
Chr7	34,151,048	12	23,665
Chr8	30,970,978	3	5,761
Chr9	29,176,711	7	2,710
Chr10	28,680,989	10	15,916
Chr11	27,431,910	5	5,276
Chr12	26,844,116	10	4,596
Chr13	22,233,288	3	20,727
Chr14	21,077,759	3	10,087
Chr15	14,522,058	3	16,563
ChrZ	14,335,630	3	10,867

***vitticeps* genome.**

Telomere left length (bp)	Telomere left TRUs num	Telomere right start
16,152	2,432	371,565,872
30,826	4,481	320,456,258
12,880	1,867	274,863,620
23,746	3,609	247,725,795
18,999	1,407	202,504,972
13,842	2,158	126,719,974
23,654	3,721	34,139,221
5,759	890	30,963,687
2,704	441	29,173,587
15,907	2,437	28,658,472
5,272	814	27,429,053
4,587	691	26,836,825
20,725	3,077	22,227,622
10,085	1,446	NA
16,561	2,505	14,511,754
10,865	1,681	14,286,002

Telomere right end	Telomere right length (bp)	Telomere right TRUs num
371,574,465	8,594	1,151
320,472,306	16,049	2,354
274,873,464	9,845	1,283
247,739,183	13,389	2,005
202,516,940	11,969	1,840
126,726,786	6,813	1,009
34,151,040	11,820	1,478
30,970,976	7,290	1,034
29,176,707	3,121	493
28,680,987	22,516	3,371
27,431,058	2,006	304
26,844,114	7,290	1,034
22,233,283	5,662	702
NA	NA	NA
14,521,151	9,398	1,441
14,295,687	9,686	1,491

Centromere start	Centromere end	Centromere length (bp)
172,000,000	178,000,000	6,000,000
219,000,000	225,600,000	6,600,000
134,500,000	145,500,000	11,000,000
119,500,000	124,500,000	5,000,000
104,000,000	113,000,000	9,000,000
54,500,000	63,000,000	8,500,000
19,500,000	20,500,000	1,000,000
12,500,000	14,000,000	1,500,000
13,000,000	14,500,000	1,500,000
15,000,000	15,500,000	500,000
12,500,000	14,500,000	2,000,000
13,000,000	14,000,000	1,000,000
14,500,000	15,500,000	1,000,000
NA	NA	NA
NA	NA	NA
11,500,000	12,000,000	500,000

Table S9. Comparison of telomeric region characteristics between *Pogona v*

Species	Chromosome num	Telomere num
Pogona vitticeps assembly before correcting telomeric regions	16	8
**Pvit2014**	16	31
Podarcis raffonei	20	33
Vipera latastei	18	30
Zootoca vivipara	19	25
Liasis olivaceus	17	25
Podarcis cretensis	19	21
Eublepharis macularius	19	24
Furcifer pardalis	11	20
Cyclura pinguis	19	19
Hemicordylus capensis	17	14
Heteronotia binoei	21	15
Bungarus multicinctus	18	3
Hydrophis major	16	12
Erythrolamprus reginae	40	10
Podarcis lilfordi	20	3
Thamnophis elegans	18	2
Podarcis muralis	19	2
Shinisaurus crocodilurus	16	0
Lacerta agilis	20	1
Hydrophis cyanocinctus	18	1
Hydrophis curtus	17	1
Sphaerodactylus townsendi	17	0
Sceloporus undulatus	11	0
Sceloporus tristichus	11	0
Podarcis muralis nigriventris	20	0
Phrynosoma platyrhinos	18	0
Naja naja	19	0
Crotalus viridis viridis	18	0
Ahaetulla prasina	18	0

itticeps assembly and 28 squamate species whose genome reached chromosome le

% total assembled telomeric regions num to expected num	Average TRUs num	Average telomeric region length (bp)
25.00	2,158	13,743
96.88	1,661	11,289
82.50	935	5,906
83.33	2,264	13,932
65.79	1,391	9,217
73.53	1,395	8,696
55.26	831	11,649
63.16	1,457	9,104
90.91	1,824	11,273
50.00	1,466	9,446
41.18	1,929	12,593
35.71	1,283	10,886
8.33	223	2,907
37.50	1,862	12,694
12.50	2,411	19,892
7.50	1,292	8,342
5.56	394	3,091
5.26	463	3,021
0.00	0	0
2.50	357	2,711
2.78	326	4,448
2.94	354	2,783
0.00	0	0
0.00	0	0
0.00	0	0
0.00	0	0
0.00	0	0
0.00	0	0
0.00	0	0
0.00	0	0

evel.

Table S10. Annotation of repeat sequences

Type	Pvit2024	
	Length (bp)	% of genome
**RepBase**	367,001,920	20.47
**ProteinMask**	17,316,349	0.97
**TRF**	42,964,525	2.40
**Denovo**	791,538,902	44.14
**Total**	829,017,046	46.23
**Category**		
**SINEs**	62,178,492	3.47
**LINEs**	322,002,884	17.96
**LTR elements**	106,912,258	5.96
**DNA elements**	158,916,449	8.86
**Satellite**	3,575,435	0.20
**Unknown**	228,552,495	12.75

Table S11. Gene model and function annotation.

Species Name	Pvit2024
total genes	21,783
total transcripts (isoforms)	54,998
number of genes with intact-orf	17,123
pre-terminated genes	0
single exon genes	2,402
mean length locus (bp)	36,714.80
mean length of coding region (bp)	1,641.58
mean exons per gene	9.4
mean length exons (bp)	492.48
mean length introns (bp)	3,821.53
number of genes with 5' utr	19,011
5' utr mean length per gene (bp)	661
number of genes with 3' utr	20,320
3' utr mean length per gene (bp)	2,428
BUSCO of sauropsid_odb10 (%)	98.2
% of UniProtKB/Swiss-Prot	90.7
% of NCBI nr database	92.7
% of InterProScan	61.5
% of KEGG	84.8
% of eggNOG-5.0 database	89
% of proteins with functional annotation	92.8

Table S12. 47 protein-coding genes and 65 lncRNA genes that are missin

Gene Type	Chromosome	Gene ID
		PvitG26974.1
		PvitG26975.1
		PvitG26976.1
		PvitG26977.1
		PvitG26978.3
		PvitG26979.1
		PvitG26981.3
		PvitG26982.1
		PvitG26983.1
		PvitG26984.1
		PvitG26985.1
		PvitG26986.1
		PvitG26987.1
		PvitG27287.1
		PvitG27291.1
		PvitG27292.1
		PvitG27295.1
		PvitG27296.1
		PvitG27297.1
		PvitG27298.2
		PvitG27299.1
		PvitG27300.1
		PvitG27301.1
Protein coding genes		PvitG27302.1
		PvitG27303.1
		PvitG27305.1
		PvitG27307.3
		PvitG27306.1
		PvitG27308.1
		PvitG27309.1
		PvitG27310.1
		PvitG27311.1
		PvitG27312.1
		PvitG27313.1
		PvitG27314.1
		PvitG27316.1
		PvitG27317.1
		PvitG27318.1
		PvitG02539.1_2
		PvitG02539.1_3
		PvitG27331.1

		PvitG27346.1
		PvitG27347.1
		PvitG27348.1
		ChrZG00347.1
		ChrZG00349.1
		ACAT00000003626.4_PvitG27353_RGL
		novel_gene28466.2
		novel_gene28474.3
		novel_gene28478.1
		PvitG26987.6
		novel_gene28479.1
		novel_gene28581.1
		novel_gene28583.2
		novel_gene28584.1
	ChrZ	novel_gene28586.1
		novel_gene28587.2
		PvitG27292.3
		novel_gene28588.1
		novel_gene28590.1
		novel_gene28592.2
		novel_gene28595.1
		novel_gene28596.1
		novel_gene28597.1
		novel_gene28600.1
		novel_gene28601.1
		novel_gene28602.1
		novel_gene28603.1
		novel_gene28606.1
		novel_gene28608.1
		novel_gene28611.2
		novel_gene28614.1
		novel_gene28615.1
		novel_gene28618.1
		novel_gene28619.3
		novel_gene28621.1
		novel_gene28623.1
		novel_gene28626.3
		novel_gene28628.1
lncRNAs		novel_gene28629.3
		novel_gene28634.1
		novel_gene28635.2
		novel_gene28636.2
		novel_gene28640.2

novel_gene28643.1
novel_gene28647.1
novel_gene28648.1
novel_gene28649.1
novel_gene28650.2
novel_gene28653.5
novel_gene28656.1
novel_gene28657.6
novel_gene28659.5
novel_gene28661.2
novel_gene28666.2
novel_gene28663.4
novel_gene28668.4
novel_gene28670.2
novel_gene28676.2
novel_gene28693.4
PvitG07790_1.1
novel_gene28695.2
novel_gene28698.1
novel_gene28703.2
novel_gene28705.3
novel_gene28707.8
PvitG27453.1
novel_gene28709.2
novel_gene28712.2
novel_gene28716.2
novel_gene28718.1
novel_gene28720.3

g or unknown to be Z-linked in prior Pvit2015 assembly.

Pos start	Pos end	Strand
48,814	69,207	+
72,229	73,458	-
92,686	99,840	-
92,686	109,854	+
104,631	133,846	-
135,540	208,712	-
212,772	229,055	-
225,002	233,487	+
234,708	241,939	+
247,416	251,592	+
339,299	365,698	+
365,728	369,644	+
424,951	536,711	+
8,816,524	8,904,111	+
9,346,662	9,356,930	-
9,467,121	9,935,222	+
9,829,500	9,833,018	-
9,945,789	9,951,319	+
9,945,789	10,102,971	-
10,331,238	10,403,948	+
10,423,427	10,437,608	+
10,465,434	10,478,654	+
10,502,831	10,519,246	-
10,528,336	10,540,547	-
10,571,160	10,663,216	+
10,682,967	10,717,483	+
10,713,757	10,759,128	-
10,721,811	10,725,072	+
10,769,156	10,777,533	+
10,816,491	10,817,484	+
10,827,770	10,864,590	+
10,903,746	10,980,529	+
11,007,341	11,260,439	+
11,264,716	11,271,705	+
11,332,529	11,363,620	+
11,362,419	11,367,514	-
11,366,864	11,374,090	+
11,436,221	11,439,949	-
11,720,704	11,721,387	-
11,725,734	11,726,420	-
11,866,136	11,875,750	-

11,948,426	11,995,446	-
12,003,763	12,026,527	+
12,034,575	12,040,740	+
12,071,495	12,083,434	+
12,087,155	12,088,467	+
12,091,150	12,100,215	-
15,931	17,922	-
279,298	283,324	+
372,252	373,370	+
435,645	438,442	+
534,433	536,978	-
8,882,480	8,886,153	-
9,163,795	9,229,091	-
9,205,163	9,206,585	+
9,341,447	9,343,314	-
9,388,000	9,404,419	-
9,584,507	9,587,600	+
10,040,306	10,040,975	+
10,491,846	10,497,260	+
10,492,022	10,506,013	+
10,502,945	10,506,013	+
10,799,081	10,800,134	+
10,803,914	10,804,979	+
11,201,999	11,206,908	-
11,220,898	11,222,215	-
11,223,038	11,225,709	-
11,248,916	11,250,227	-
11,511,714	11,514,102	-
11,594,314	11,598,086	+
11,707,913	11,761,545	+
11,752,669	11,761,178	-
11,761,318	11,772,008	+
11,790,362	11,792,151	+
11,799,058	11,813,694	-
11,799,628	11,800,884	-
12,050,518	12,052,350	+
12,128,587	12,145,184	-
12,140,716	12,143,783	-
12,157,502	12,162,257	-
12,187,730	12,200,579	+
12,203,429	12,206,009	+
12,203,571	12,210,294	+
12,403,170	12,418,140	+

12,426,275	12,440,482	+
12,483,700	12,484,700	-
12,503,439	12,565,768	-
12,526,450	12,528,171	+
12,660,890	12,682,103	-
12,724,642	12,767,940	+
12,733,404	12,738,770	+
12,845,785	12,873,059	-
12,863,525	12,874,076	-
12,874,444	12,878,515	+
12,881,743	12,883,785	+
12,882,380	12,990,692	+
12,950,978	12,970,591	+
13,031,694	13,039,152	+
13,139,700	13,201,140	+
13,511,346	13,531,137	-
13,577,135	13,590,510	+
13,577,861	13,613,773	-
13,648,594	13,708,383	-
13,732,630	13,733,584	-
13,747,774	13,796,277	-
13,864,650	13,914,873	-
13,914,878	13,949,381	+
13,915,131	13,922,360	+
14,012,174	14,050,621	-
14,120,145	14,165,991	-
14,239,977	14,278,468	-
14,305,145	14,333,310	-

Table S13. 20 samples (8 Z

Sample ID	Genotype
ZW_1	ZW female
ZW_2	ZW female
ZZ_1	ZZ male
ZZ_2	ZZ male
ZZ_3	ZZ male
ZZ_4	ZZ male
ZW_3	ZW female
ZW_4	ZW female
ZW_5	ZW female
ZW_6	ZW female
ZW_7	ZW female
ZW_8	ZW female
ZW_9	ZW female
ZW_10	ZW female
ZW_11	ZW female
ZW_12	ZW female
ZZ_5	ZZ male
ZZ_6	ZZ male
ZZ_7	ZZ male
ZZ_8	ZZ male

ZZ males and 12 ZW females) WGS data used for demarcating

Biosample Accession
SAMN21971475
SAMEA2300449
SAMN21971475
SAMN44670111/SAMN44670112/SAMN44670109/SAMN44670110
SAMEA2300448
SAMEA2300447
-
-
-
-
-
-
-
-
-
-
-
-
-
-

g the SDR and PAR regions.

Run Accession	Total clean bases used for this analysis (Gb)
SRR17072952	63.19
ERR409918, ERR409919, ERR409920	66.05
SRR17072953	68.56
**DNBSEQ WGS data in this study**	112.23
ERR409938, ERR409939, ERR409940, ERR409941, ERR409942	100.60
ERR409943, ERR409944, ERR409945, ERR409946, ERR409947	98.27
**DNBSEQ WGS data in this study**	246.48
**DNBSEQ WGS data in this study**	149.29
**DNBSEQ WGS data in this study**	153.76
**DNBSEQ WGS data in this study**	165.04
**DNBSEQ WGS data in this study**	167.01
**DNBSEQ WGS data in this study**	205.85
**DNBSEQ WGS data in this study**	185.62
**DNBSEQ WGS data in this study**	176.49
**DNBSEQ WGS data in this study**	148.75
**DNBSEQ WGS data in this study**	147.85
**DNBSEQ WGS data in this study**	161.82
**DNBSEQ WGS data in this study**	257.04
**DNBSEQ WGS data in this study**	187.41
**DNBSEQ WGS data in this study**	121.36

Genome coverage (X)
35.3
36.9
38.3
62.7
56.2
54.9
137.70
83.40
85.90
92.20
93.30
115.00
103.70
98.60
83.10
82.60
90.40
143.60
104.70
67.80

Table S14. Overview of all sequenced embryonic gonad samples i

Run accession	Sample ID	Developmental stage	Genotype
SRR13616458	ZW_s6/7_c1_a	stage6	ZW
SRR13616457	ZW_s6/7_c1_b	stage6	ZW
SRR13616473	ZW_s6/7_c1_c	stage6	ZW
SRR13616462	ZW_s6/7_c3_a	stage6	ZW
SRR13616449	ZW_s6/7_c3_b	stage6	ZW
SRR13616448	ZW_s6/7_c3_c	stage6	ZW
SRR13616447	ZW_s6/7_c6_a	stage6	ZW
SRR29848887	ZZ_s6/7_c2_a	stage6	ZZ
SRR29848883	ZZ_s6/7_c2_b	stage6	ZZ
SRR29848886	ZZ_s6/7_c6_b	stage6	ZZ
SRR13616480	ZW_s12_c1_b	stage12	ZW
SRR13616479	ZW_s12_c1_c	stage12	ZW
SRR13616478	ZW_s12_c1_d	stage12	ZW
SRR13616477	ZW_s12_c2_a	stage12	ZW
SRR13616476	ZW_s12_c2_c	stage12	ZW
SRR13616475	ZW_s12_c3_a	stage12	ZW
SRR13616474	ZW_s12_c3_c	stage12	ZW
SRR13616472	ZW_s12_c3_d	stage12	ZW
SRR13616471	ZW_s12_c6_b	stage12	ZW
SRR29848875	ZZ_s12_c1_a	stage12	ZZ
SRR29848878	ZZ_s12_c2_b	stage12	ZZ
SRR29848882	ZZ_s12_c3_b	stage12	ZZ
SRR29848879	ZZ_s12_c6_a	stage12	ZZ
SRR29848876	ZZ_s12_c6_c	stage12	ZZ
SRR13616464	ZW_s16_08	stage16	ZW
SRR13616463	ZW_s16_10	stage16	ZW
SRR13616461	ZW_s16_c1_c	stage16	ZW
SRR13616460	ZW_s16_c3_a	stage16	ZW
SRR29848874	ZZ_s16_c1_a	stage16	ZZ
SRR29848873	ZZ_s16_c1_b	stage16	ZZ
SRR29848877	ZZ_s16_c2_a	stage16	ZZ
SRR29848884	ZZ_s16_c2_b	stage16	ZZ
SRR29848880	ZZ_s16_c3_b	stage16	ZZ
SRR29848881	ZZ_s16_c3_c	stage16	ZZ
SRR29848872	ZZ_s16_c6_a	stage16	ZZ
SRR29848885	ZZ_s16_c6_b	stage16	ZZ

ncluded in this study.

Total raw bases (Gb)	Total clean bases (Gb)	Raw data Q30 (%)	Clean data Q30 (%)
6.69	5.38	90	94
6.91	5.73	92	96
5.90	4.97	93	96
6.06	5.12	94	96
5.69	4.67	93	96
6.29	5.26	92	95
7.59	6.07	89	94
7.52	6.14	91	95
6.72	5.60	92	95
7.69	6.39	91	95
7.95	6.57	91	95
6.27	5.27	93	96
6.31	5.34	93	96
6.53	5.49	93	96
7.02	5.73	90	95
6.86	5.68	91	95
5.58	4.61	91	95
6.39	5.36	92	95
5.82	4.79	91	96
5.57	4.59	93	96
6.24	5.27	93	96
6.58	5.38	91	95
6.01	5.07	93	96
5.49	4.57	93	96
6.53	5.46	92	95
6.52	5.38	91	95
14.70	12.19	90	93
14.78	12.30	91	93
6.06	5.07	93	96
6.59	5.35	90	95
6.19	5.21	93	96
7.24	5.77	89	95
5.46	4.37	90	95
6.13	4.11	83	95
6.06	5.10	93	96
6.52	5.37	91	95

Biosample_Accession
SAMN17765903
SAMN17765904
SAMN17765905
SAMN17765906
SAMN17765907
SAMN17765908
SAMN17765909
SAMN42536705
SAMN42536717
SAMN42536706
SAMN17765916
SAMN17765917
SAMN17765918
SAMN17765919
SAMN17765920
SAMN17765921
SAMN17765922
SAMN17765923
SAMN17765924
SAMN42536711
SAMN42536708
SAMN42536718
SAMN42536707
SAMN42536710
SAMN17765931
SAMN17765932
SAMN17765933
SAMN17765934
SAMN42536712
SAMN42536713
SAMN42536709
SAMN42536716
SAMN42536720
SAMN42536719
SAMN42536714
SAMN42536715

Table S15. Differential expression profiles of Z-link genes across various develop

Developmental stage	Gene ID	Log2 Fold Change	pvalue
stage6/7	PvitG005755	1.528083998	3.95E-06
stage6/7	PvitG005772	1.272967953	0.000588088
stage6/7	PvitG005872	1.840921903	8.50E-05
stage6/7	PvitG005878	-2.748115195	7.99E-05
stage6/7	PvitG005903	-4.115387101	0.000332648
stage6/7	PvitG005995	-1.686134416	0.001674141
stage6/7	PvitG006026	1.247394341	7.80E-06
stage6/7	PvitG006049	-3.004617267	0.001471015
stage6/7	PvitG006058	1.216070448	3.47E-07
stage6/7	PvitG006071	2.44388135	3.34E-07
stage12	PvitG005748	-1.612292304	1.75E-06
stage12	PvitG005754	-1.188083014	0.000117632
stage12	PvitG005755	2.074799081	1.51E-06
stage12	PvitG005764	2.154513884	2.11E-12
stage12	PvitG005769	2.230964926	1.28E-16
stage12	PvitG005795	-1.108070687	0.00111618
stage12	PvitG005811	2.068462842	1.44E-12
stage12	PvitG005885	-2.448076738	0.001380971
stage12	PvitG005890	1.799532183	1.24E-05
stage12	PvitG005894	1.063969495	3.02E-06
stage12	PvitG005900	1.09574239	0.000333059
stage12	PvitG005922	-1.53953962	0.00287047
stage12	PvitG005950	-2.178224652	1.05E-06
stage12	PvitG005952	-2.179761956	0.001291535
stage12	PvitG005964	-1.151183102	6.44E-08
stage12	PvitG005965	-1.728572832	2.00E-06
stage12	PvitG005994	-3.392925682	0.002230758
stage12	PvitG005995	-3.571723502	2.62E-23
stage12	PvitG005998	-1.688950274	0.000412841
stage12	PvitG006015	1.10089596	0.00193338
stage12	PvitG006043	-1.144731829	6.34E-05
stage12	PvitG006049	-1.860087733	2.54E-05
stage12	PvitG006058	2.398310967	2.67E-24
stage12	PvitG006070	-1.764061493	0.001320493
stage12	PvitG006071	4.361093601	4.80E-17
stage16	PvitG005748	-1.214253639	0.002290827
stage16	PvitG005755	1.965854146	5.56E-05
stage16	PvitG005764	2.755131395	4.86E-08
stage16	PvitG005769	1.881719721	0.001645918
stage16	PvitG005792	-1.961512266	2.65E-05
stage16	PvitG005811	2.171155905	2.89E-12

stage16	PvitG005884	1.04727797	0.001081267
stage16	PvitG005885	-3.066045219	1.02E-05
stage16	PvitG005890	1.809868122	3.94E-05
stage16	PvitG005894	1.25206201	0.000302533
stage16	PvitG005900	1.274474767	0.002495376
stage16	PvitG005938	-1.435335043	0.000101013
stage16	PvitG005951	4.0065347	1.35E-05
stage16	PvitG005964	-2.192354007	1.93E-08
stage16	PvitG005965	-2.130784065	6.13E-06
stage16	PvitG005994	-4.636387138	1.46E-05
stage16	PvitG005995	-4.679761465	1.48E-23
stage16	PvitG005998	-1.494520457	2.87E-06
stage16	PvitG006043	-1.383342972	3.45E-06
stage16	PvitG006049	-1.424482981	0.000218202
stage16	PvitG006058	1.942233051	6.64E-07
stage16	PvitG006071	4.502887786	1.48E-10

mental stages.

padj
0.000417008
0.022372293
0.005210789
0.00493921
0.014822154
0.045821423
0.0007206
0.042336342
5.09E-05
4.97E-05
5.47E-05
0.002235253
4.83E-05
1.86E-10
1.94E-14
0.014168168
1.31E-10
0.016610427
0.000314427
8.78E-05
0.005357196
0.030025146
3.47E-05
0.015811922
2.77E-06
6.21E-05
0.024467376
7.90E-21
0.006459666
0.021850195
0.001318592
0.000581578
8.89E-22
0.016052449
7.99E-15
0.041061823
0.002083427
4.68E-06
0.031526851
0.001093138
6.45E-10

0.023009643
0.000496958
0.001546997
0.008457567
0.043737049
0.003442423
0.000618206
2.05E-06
0.000316892
0.000660209
1.28E-20
0.000160338
0.000187468
0.006512445
4.57E-05
2.54E-08

Table S16. Quality control of long-range sequencing data from two platforms.

Data type	Hi-C_1 data	Hi-C_2 data	CycloneSEQ based Pore-C data
**pair reads number**	480,395,400	538,885,800	35,343,379
**Total bases (Gb)**	144	162	59,417,472,662
**total pairs**	311,477,361	334,885,244	134,790,785
**valid pairs ratio**	87.61%	87.71%	91.43%
**intra pairs ratio**	80.21%	80.21%	82.37%
**inter pairs ratio**	19.79%	19.79%	17.63%

* Simulate restriction enzyme digestion of the reference genome and extract adjacent fragments of CycloneSEQ based Pore-C reads as pairs. If the two reads in a pair come from different genomic digestion regions, they are a pair of valid fragments. The valid pairs ratio is the number of valid pairs divided by the total number of pairs.If the two reads in a valid pair come from the same chromosome, it is an intra pair, otherwise it is an inter pair.intra/inter pairs ratio is the number of intra/inter pairs divided by the total number of valid pairs.

Table S17. The distribution of the depths of ATGC at each site of

Pos	ZZ_1	ZZ_2
Z_7331670_G	71G	62G
Z_7331671_T	73T	59T
Z_7331672_C	69C	60C
Z_7331673_C	71C	60C
Z_7331674_C	67C	60C
Z_7331675_A	68A	60A
Z_7331676_G	70G	56G
Z_7331677_C	72C	58C
Z_7331678_T	71T	58T
Z_7331679_C	72C	58C
Z_7331680_C	68C	58C
Z_7331681_T	63T	58T
Z_7331682_G	63G	59G
Z_7331683_C	63C	59C
Z_7331684_C	62C	59C
Z_7331685_A	62A	61A
Z_7331686_A	59A	63A
Z_7331687_C	59C	63C
Z_7331688_C	62C	62C
Z_7331689_T	64T	63T
Z_7331690_A	64A	63A
Z_7331691_G	64G	62G
Z_7331692_C	64C	61C
Z_7331693_A	63A	64A
Z_7331694_G	61G	64G
Z_7331695_T	61T	62T
Z_7331696_T	58T	62T
Z_7331697_C	58C	65C
Z_7331698_A	55A	65A
Z_7331699_A	56A	66A
Z_7331700_A	58A	64A
Z_7331701_A	55A	63A
Z_7331702_G	54G	62G
Z_7331703_C	56C	62C
Z_7331704_A	54A	64A
Z_7331705_T	55T	64T
Z_7331706_G	55G	65G
Z_7331707_T	47T	63T
Z_7331708_A	51A	62A
Z_7331709_A	48A	62A
Z_7331710_A	48A	63A

Z_7331711_T	48T	62T
Z_7331712_G	49G	61G
Z_7331713_C	49C	62C
Z_7331714_G	48G	63G
Z_7331715_A	44A	64A
Z_7331716_G	46G	64G
Z_7331717_T	46T	61T
Z_7331718_A	43A	64A
Z_7331719_G	47G	66G
Z_7331720_A	47A	65A
Z_7331721_T	47T	65T
Z_7331722_A	44A	64A
Z_7331723_A	44A	64A
Z_7331724_A	35G	64A
Z_7331725_T	40T	64T
Z_7331726_A	41A	65A
Z_7331727_G	40G	66G
Z_7331728_G	39G	68G
Z_7331729_G	37T	68G
Z_7331730_A	36A	66A
Z_7331731_C	30G	67C
Z_7331732_C	34C	65C
Z_7331733_A	34A	65A
Z_7331734_C	35C	65C
Z_7331735_C	35C	66C
Z_7331736_A	32A	67A
Z_7331737_T	31T	66T
Z_7331738_A	30A	70A
Z_7331739_G	32G	70G
Z_7331740_T	32T	70T
Z_7331741_A	31G	70A
Z_7331742_G	31G	70G
Z_7331743_G	32G	69G
Z_7331744_A	32A	71A
Z_7331745_A	34A	71A
Z_7331746_G	34G	71G
Z_7331747_G	33G	72G
Z_7331748_T	36T	73T
Z_7331749_A	37A	72A
Z_7331750_A	37A	72A
Z_7331751_T	39T	73T
Z_7331752_G	38G	73G
Z_7331753_G	38G	73G

Z_7331754_C	37C	73C
Z_7331755_G	37G	73G
Z_7331756_T	38T	71T
Z_7331757_T	39T	71T
Z_7331758_C	40C	72C
Z_7331759_T	38T	72T
Z_7331760_A	39A	72A
Z_7331761_T	39T	73T
Z_7331762_G	39G	73G
Z_7331763_T	38T	72T
Z_7331764_C	42C	72C
Z_7331765_T	42T	72T
Z_7331766_A	43A	71A
Z_7331767_G	44G	70G
Z_7331768_T	44T	71T
Z_7331769_A	43A	71A
Z_7331770_G	43G	71G
Z_7331771_A	42A	71A
Z_7331772_A	42A	71A
Z_7331773_A	42A	71A
Z_7331774_C	45C	70C
Z_7331775_T	44T	70T
Z_7331776_G	44G	68G
Z_7331777_T	42T	69T
Z_7331778_C	42C	69C
Z_7331779_C	42C	69C
Z_7331780_A	42A	66A
Z_7331781_C	42C	66C
Z_7331782_G	42G	64G
Z_7331783_G	40G	64G
Z_7331784_A	39A	62A
Z_7331785_C	39C	65C
Z_7331786_A	40A	67A
Z_7331787_A	41A	69A
Z_7331788_A	41A	68A
Z_7331789_T	41T	63T
Z_7331790_G	43G	68G
Z_7331791_C	43C	68C
Z_7331792_C	42C	67C
Z_7331793_G	44G	68G
Z_7331794_G	40G	66G
Z_7331795_C	41C	27A_40C
Z_7331796_T	41T	69T

Z_7331797_C	41C	69C
Z_7331798_T	42T	27A_42T
Z_7331799_A	42A	69A
Z_7331800_C	42C	72C
Z_7331801_A	44A	71A
Z_7331802_G	46G	73G
Z_7331803_C	48C	74C
Z_7331804_T	49T	78T
Z_7331805_T	49T	77T
Z_7331806_G	49G	80G
Z_7331807_G	51G	81G
Z_7331808_A	52A	79A
Z_7331809_G	52G	80G
Z_7331810_A	52A	79A
Z_7331811_T	52T	80T
Z_7331812_G	55G	81G
Z_7331813_G	54G	79G
Z_7331814_G	55G	77G
Z_7331815_G	52G	78G
Z_7331816_A	54A	79A
Z_7331817_T	53T	79T
Z_7331818_G	52G	79G
Z_7331819_A	49A	78A
Z_7331820_G	38A_11G	79G
Z_7331821_C	48C	80C
Z_7331822_A	48A	78A
Z_7331823_C	47C	76C
Z_7331824_C	48C	77C
Z_7331825_G	50G	77G
Z_7331826_C	48C	77C
Z_7331827_G	47G	78G
Z_7331828_C	46C	77C
Z_7331829_C	46C	77C
Z_7331830_C	45C	76C
Z_7331831_T	47T	76T
Z_7331832_A	49A	69A
Z_7331833_G	49G	74G
Z_7331834_A	46A	75A
Z_7331835_G	46G	68G
Z_7331836_T	45T	76T
Z_7331837_C	46C	74C
Z_7331838_G	43G	77G
Z_7331839_G	44G	77G

Z_7331840_A	45A	75A
Z_7331841_C	45C	76C
Z_7331842_A	44A	75A
Z_7331843_C	43C	76C
Z_7331844_A	42A	69A
Z_7331845_A	41A	77A
Z_7331846_C	40C	78C
Z_7331847_T	43T	77T
Z_7331848_G	44G	76G
Z_7331849_G	44G	76G
Z_7331850_A	45A	75A
Z_7331851_C	46C	76C
Z_7331852_T	45T	76T
Z_7331853_A	45A	76A
Z_7331854_A	47A	77A
Z_7331855_A	49A	77A
Z_7331856_T	48T	45T_29C
Z_7331857_G	48G	30A_45G
Z_7331858_T	49T	76T
Z_7331859_C	49C	64C
Z_7331860_A	48A	69A
Z_7331861_A	47A	79A
Z_7331862_G	47G	78G
Z_7331863_G	47G	78G
Z_7331864_G	47G	78G
Z_7331865_G	47G	76G
Z_7331866_A	47A	77A
Z_7331867_A	47A	78A
Z_7331868_C	46C	78C
Z_7331869_C	48C	77C
Z_7331870_T	48T	78T
Z_7331871_T	46T	77T
Z_7331872_T	48T	72T
Z_7331873_A	46A	76A
Z_7331874_C	43C	75C
Z_7331875_C	45C	74C
Z_7331876_T	45T	74T
Z_7331877_T	47T	75T
Z_7331878_T	45T	74T
Z_7331879_A	45A	76A
Z_7331880_C	45C	74C
Z_7331881_C	45C	67C
Z_7331882_T	43T	70T

Z_7331883_A	41A	69A
Z_7331884_T	41T	65T
Z_7331885_A	39A	65A
Z_7331886_T	39T	67T
Z_7331887_C	39C	67C
Z_7331888_T	41T	68T
Z_7331889_G	43G	69G
Z_7331890_G	43G	68G
Z_7331891_C	45C	68C
Z_7331892_T	47T	70T
Z_7331893_A	48A	71A
Z_7331894_C	50C	71C
Z_7331895_T	49T	69T
Z_7331896_C	48C	72C
Z_7331897_T	49T	73T
Z_7331898_G	50G	74G
Z_7331899_C	50C	41T_33C
Z_7331900_C	48C	76C
Z_7331901_A	50A	73A
Z_7331902_C	48C	72C
Z_7331903_C	50C	72C
Z_7331904_C	51C	70C
Z_7331905_T	50T	70T
Z_7331906_C	51C	68C
Z_7331907_T	48T	68T
Z_7331908_C	48C	69C
Z_7331909_T	49T	70T
Z_7331910_C	49C	68C
Z_7331911_T	48T	68T
Z_7331912_A	48A	68A
Z_7331913_C	47C	69C
Z_7331914_C	48C	70C
Z_7331915_C	48C	70C
Z_7331916_T	49T	70T
Z_7331917_C	48C	71C
Z_7331918_C	48C	70C
Z_7331919_C	48C	32T_37C
Z_7331920_G	47G	70G
Z_7331921_G	47G	69G
Z_7331922_T	47T	69T
Z_7331923_G	46G	70G
Z_7331924_G	44G	72G
Z_7331925_G	43G	71G

Z_7331926_T	44T	71T
Z_7331927_G	43G	70G
Z_7331928_G	41G	69G
Z_7331929_A	38A	68A
Z_7331930_T	40T	67T
Z_7331931_T	40T	66T
Z_7331932_C	41C	66C
Z_7331933_T	41T	64T
Z_7331934_C	43C	61C
Z_7331935_G	43G	61G
Z_7331936_T	42T	62T
Z_7331937_A	43A	62A
Z_7331938_G	43G	61G
Z_7331939_T	41T	60T
Z_7331940_T	43T	60T
Z_7331941_C	42C	59C
Z_7331942_C	41C	60C
Z_7331943_T	42T	61T
Z_7331944_A	41A	61A
Z_7331945_C	41C	60C
Z_7331946_T	43T	61T
Z_7331947_C	41C	62C
Z_7331948_A	40A	61A
Z_7331949_C	40C	60C
Z_7331950_A	40A	60A
Z_7331951_C	39C	60C
Z_7331952_T	39T	60T
Z_7331953_G	39G	60G
Z_7331954_T	38T	60T
Z_7331955_C	38C	60C
Z_7331956_A	39A	58A
Z_7331957_A	38A	57A
Z_7331958_G	33G	58G
Z_7331959_T	34T	58T
Z_7331960_G	33G	58G
Z_7331961_A	31A	61A
Z_7331962_T	29T	61T
Z_7331963_A	28A	61A
Z_7331964_A	25A	62A
Z_7331965_T	27T	62T
Z_7331966_T	26T	63T
Z_7331967_C	27C	62C
Z_7331968_A	26A	62A

Z_7331969_T	26T	64T
Z_7331970_C	27C	63C
Z_7331971_C	27C	63C
Z_7331972_T	26T	62T
Z_7331973_T	27T	62T
Z_7331974_C	28C	60C
Z_7331975_A	28A	60A
Z_7331976_C	28C	60C
Z_7331977_A	29A	55A
Z_7331978_G	29G	55G
Z_7331979_T	29T	53T
Z_7331980_C	29C	53C
Z_7331981_T	31T	53T
Z_7331982_G	32G	52G
Z_7331983_A	32A	52A
Z_7331984_A	32A	52A
Z_7331985_T	33T	51T
Z_7331986_G	31G	53G
Z_7331987_C	30C	53C
Z_7331988_A	29A	51A
Z_7331989_A	29A	50A
Z_7331990_A	28A	49A
Z_7331991_G	29G	46G
Z_7331992_G	17A_29G	44G
Z_7331993_G	29A	15A_41G
Z_7331994_A	31A	43A
Z_7331995_A	30A	42A
Z_7331996_A	30A	42A
Z_7331997_A	31A	44A
Z_7331998_A	31A	44A
Z_7331999_A	31A	44A
Z_7332000_A	30A	44A
Z_7332001_A	31A	44A
Z_7332002_A	30A	44A
Z_7332003_A	30A	44A
Z_7332004_A	28A	43A
Z_7332005_A	28A	43A
Z_7332006_T	27T	42T
Z_7332007_A	26A	41A
Z_7332008_A	26A	41A
Z_7332009_G	26G	43G
Z_7332010_G	25G	42G
Z_7332011_G	26G	43G

Z_7332012_C	28C	46C
Z_7332013_C	29C	46C
Z_7332014_A	30A	47A
Z_7332015_G	32G	46G
Z_7332016_C	31C	47C
Z_7332017_T	32T	47T
Z_7332018_C	33C	46C
Z_7332019_A	31A	47A
Z_7332020_C	31C	47C
Z_7332021_T	33T	47T
Z_7332022_G	33G	49G
Z_7332023_G	33G	49G
Z_7332024_T	35T	51T
Z_7332025_C	36C	51C
Z_7332026_C	37C	51C
Z_7332027_C	38C	53C
Z_7332028_C	38C	53C
Z_7332029_A	39A	53A
Z_7332030_A	38A	54A
Z_7332031_G	39G	54G
Z_7332032_C	37C	53C
Z_7332033_A	38A	54A
Z_7332034_G	36G	54G
Z_7332035_C	37C	54C
Z_7332036_A	36A	54A
Z_7332037_A	35A	53A
Z_7332038_A	35A	53A
Z_7332039_G	35G	54G
Z_7332040_A	34A	55A
Z_7332041_T	35T	55T
Z_7332042_A	33A	56A
Z_7332043_A	32A	56A
Z_7332044_G	32G	56G
Z_7332045_G	32G	56G
Z_7332046_A	31A	55A
Z_7332047_C	31C	55C
Z_7332048_T	32T	55T
Z_7332049_C	32C	55C
Z_7332050_G	31G	56G
Z_7332051_G	30G	59G
Z_7332052_C	30C	58C
Z_7332053_A	30A	59A
Z_7332054_A	29A	59A

Z_7332055_A	29A	57A
Z_7332056_G	28G	55G
Z_7332057_C	28C	56C
Z_7332058_G	26G	57G
Z_7332059_G	29G	57G
Z_7332060_G	30G	56G
Z_7332061_G	29G	55G
Z_7332062_A	30A	56A
Z_7332063_G	30G	57G
Z_7332064_A	29A	55A
Z_7332065_A	30A	55A
Z_7332066_A	28A	57A
Z_7332067_A	28A	58A
Z_7332068_C	28C	58C
Z_7332069_C	29C	58C
Z_7332070_C	29C	58C
Z_7332071_A	28A	59A
Z_7332072_C	29C	60C
Z_7332073_A	29A	59A
Z_7332074_C	29C	60C
Z_7332075_T	29T	59T
Z_7332076_T	29T	60T
Z_7332077_G	30G	60G
Z_7332078_A	30A	59A
Z_7332079_C	30C	60C
Z_7332080_T	31T	60T
Z_7332081_A	31A	59A
Z_7332082_T	29T	57T
Z_7332083_A	28A	57A
Z_7332084_A	26A	58A
Z_7332085_G	25G	58G
Z_7332086_T	26T	59T
Z_7332087_G	24G	59G
Z_7332088_A	24A	63A
Z_7332089_A	24A	62A
Z_7332090_T	24T	61T
Z_7332091_C	22C	63C
Z_7332092_G	22G	62G
Z_7332093_T	23T	63T
Z_7332094_A	22A	64A
Z_7332095_A	21A	64A
Z_7332096_A	22A	64A
Z_7332097_A	23A	65A

Z_7332098_T	23T	65T
Z_7332099_T	23T	65T
Z_7332100_T	23T	66T
Z_7332101_A	24A	66A
Z_7332102_T	25T	66T
Z_7332103_T	25T	64T
Z_7332104_G	27G	64G
Z_7332105_G	27G	64G
Z_7332106_A	27A	63A
Z_7332107_A	27A	60A
Z_7332108_T	27T	60T
Z_7332109_G	26G	59G
Z_7332110_T	26T	62T
Z_7332111_C	26C	63C
Z_7332112_T	26T	63T
Z_7332113_T	27T	62T
Z_7332114_C	28C	65C
Z_7332115_C	29C	66C
Z_7332116_A	30A	66A
Z_7332117_T	30T	64T
Z_7332118_T	30T	64T
Z_7332119_T	31T	61T
Z_7332120_G	30G	61G
Z_7332121_G	30G	61G
Z_7332122_T	30T	59T
Z_7332123_A	30A	59A
Z_7332124_A	30A	60A
Z_7332125_G	31G	59G
Z_7332126_A	31A	58A
Z_7332127_G	31G	61G
Z_7332128_A	32A	62A
Z_7332129_G	29G	62G
Z_7332130_C	28C	61C
Z_7332131_T	28T	61T
Z_7332132_C	29C	63C
Z_7332133_A	28A	62A
Z_7332134_T	28T	61T
Z_7332135_C	30C	60C
Z_7332136_T	30T	62T
Z_7332137_T	30T	60T
Z_7332138_A	30A	61A
Z_7332139_A	30A	63A
Z_7332140_C	30C	63C

Z_7332141_C	30C	64C
Z_7332142_C	28C	64C
Z_7332143_C	29C	64C
Z_7332144_C	29C	64C
Z_7332145_T	29T	63T
Z_7332146_C	29C	60C
Z_7332147_T	29T	60T
Z_7332148_G	31G	59G
Z_7332149_G	32G	60G
Z_7332150_G	31G	60G
Z_7332151_G	31G	61G
Z_7332152_A	31A	60A
Z_7332153_G	31G	59G
Z_7332154_T	31T	58T
Z_7332155_A	31A	58A
Z_7332156_A	30A	58A
Z_7332157_A	30A	57A
Z_7332158_A	31A	58A
Z_7332159_G	31G	58G
Z_7332160_G	31G	58G
Z_7332161_G	31G	58G
Z_7332162_A	30A	57A
Z_7332163_A	30A	57A
Z_7332164_A	30A	57A
Z_7332165_C	30C	57C
Z_7332166_A	28A	56A
Z_7332167_C	26C	57C
Z_7332168_T	26T	57T
Z_7332169_T	25T	58T
Z_7332170_A	25A	58A
Z_7332171_A	24A	57A
Z_7332172_G	23G	57G
Z_7332173_G	23G	57G
Z_7332174_A	21A	58A
Z_7332175_C	21C	58C
Z_7332176_A	21A	58A
Z_7332177_C	22C	59C
Z_7332178_T	23T	61T
Z_7332179_T	24T	63T
Z_7332180_T	23T	63T
Z_7332181_T	24T	63T
Z_7332182_T	24T	64T
Z_7332183_C	24C	62C

Z_7332184_G	23G	63G
Z_7332185_T	22T	62T
Z_7332186_T	21T	61T
Z_7332187_T	23T	61T
Z_7332188_C	24C	60C
Z_7332189_G	23G	59G
Z_7332190_A	23A	60A
Z_7332191_G	24G	61G
Z_7332192_A	24A	61A
Z_7332193_A	25A	60A
Z_7332194_A	26A	60A
Z_7332195_G	25G	60G
Z_7332196_A	25A	61A
Z_7332197_A	25A	61A
Z_7332198_A	24A	61A
Z_7332199_G	24G	60G
Z_7332200_G	24G	60G
Z_7332201_A	23A	61A
Z_7332202_A	22A	60A
Z_7332203_A	22A	61A
Z_7332204_A	22A	62A
Z_7332205_A	20A	60A
Z_7332206_C	20C	58C
Z_7332207_C	20C	56C
Z_7332208_A	20A	56A
Z_7332209_A	20A	52A
Z_7332210_A	42A_21G_1C	52A
Z_7332211_A	21A	52A
Z_7332212_G	22G	52G
Z_7332213_A	21A	54A
Z_7332214_G	21G	55G
Z_7332215_A	21A	56A
Z_7332216_G	21G	56G
Z_7332217_A	22A	56A
Z_7332218_G	20G	57G
Z_7332219_A	20A	56A
Z_7332220_G	20G	56G
Z_7332221_A	22A	58A
Z_7332222_G	20G	55G
Z_7332223_A	20A	57A
Z_7332224_G	20G	58G
Z_7332225_A	19A	59A
Z_7332226_T	16T	56T

Z_7332227_C	17C	55C
Z_7332228_A	18A	57A
Z_7332229_C	17C	60C
Z_7332230_A	19A	62A
Z_7332231_G	20G	59G
Z_7332232_A	22A	62A
Z_7332233_G	21G	61G
Z_7332234_A	23A	60A
Z_7332235_G	23G	59G
Z_7332236_A	23A	59A
Z_7332237_G	23G	59G
Z_7332238_A	23A	60A
Z_7332239_G	23G	60G
Z_7332240_A	23A	60A
Z_7332241_G	22G	60G
Z_7332242_A	23A	60A
Z_7332243_A	26A_15G_1C	56A
Z_7332244_A	21A	89A_56G
Z_7332245_G	20G	33G
Z_7332246_A	20A	34A
Z_7332247_G	21G	31G
Z_7332248_A	22A	60A
Z_7332249_G	21G	62G
Z_7332250_A	21A	62A
Z_7332251_G	20G	62G
Z_7332252_A	20A	64A
Z_7332253_G	19G	62G
Z_7332254_A	18A	62A
Z_7332255_G	18G	61G
Z_7332256_A	18A	59A
Z_7332257_G	19G	61G
Z_7332258_A	16A	61A
Z_7332259_G	10T_20G	61G
Z_7332260_T	5A	26A_36T
Z_7332261_G	7G	63G
Z_7332262_T	7A	65T
Z_7332263_G	15G	67G
Z_7332264_T	16T	67T
Z_7332265_G	17G	68G
Z_7332266_A	17A	66A
Z_7332267_C	17C	66C
Z_7332268_A	18A	68A
Z_7332269_C	21C	69C

Z_7332270_A	21A	69A
Z_7332271_T	22T	71T
Z_7332272_T	22T	71T
Z_7332273_C	23C	73C
Z_7332274_A	23A	73A
Z_7332275_A	23A	74A
Z_7332276_A	23A	74A
Z_7332277_C	24C	73C
Z_7332278_T	24T	71T
Z_7332279_A	24A	71A
Z_7332280_T	24T	71T
Z_7332281_G	24G	72G
Z_7332282_T	25T	73T
Z_7332283_T	24T	74T
Z_7332284_C	24C	75C
Z_7332285_T	23T	76T
Z_7332286_C	23C	75C
Z_7332287_C	24C	75C
Z_7332288_A	25A	75A
Z_7332289_G	26G	75G
Z_7332290_C	26C	74C
Z_7332291_T	26T	74T
Z_7332292_A	25A	74A
Z_7332293_T	27T	75T
Z_7332294_T	28T	75T
Z_7332295_T	30T	76T
Z_7332296_C	31C	75C
Z_7332297_A	31A	76A
Z_7332298_A	31A	73A
Z_7332299_A	31A	75A
Z_7332300_C	30C	75C
Z_7332301_C	30C	77C
Z_7332302_A	32A	77A
Z_7332303_C	32C	77C
Z_7332304_T	32T	78T
Z_7332305_G	31G	78G
Z_7332306_C	30C	41T_37C
Z_7332307_A	31A	79A
Z_7332308_C	30C	79C
Z_7332309_T	33T	79T
Z_7332310_C	32C	80C
Z_7332311_A	32A	80A
Z_7332312_T	35T	79T

Z_7332313_G	35G	77G
Z_7332314_C	36C	78C
Z_7332315_A	36A	77A
Z_7332316_C	34C	78C
Z_7332317_G	36G	78G
Z_7332318_T	36T	76T
Z_7332319_C	36C	77C
Z_7332320_T	37T	76T
Z_7332321_A	35A	78A
Z_7332322_T	36T	76T
Z_7332323_G	35G	76G
Z_7332324_C	35C	72C
Z_7332325_C	36C	74C
Z_7332326_G	38G	73G
Z_7332327_A	38A	74A
Z_7332328_A	37A	74A
Z_7332329_C	37C	75C
Z_7332330_A	38A	76A
Z_7332331_G	36G	80G
Z_7332332_A	39A	82A
Z_7332333_G	39G	82G
Z_7332334_T	40T	82T
Z_7332335_C	41C	82C
Z_7332336_C	41C	80C
Z_7332337_A	40A	80A
Z_7332338_G	40G	80G
Z_7332339_A	38A	78A
Z_7332340_C	38C	78C
Z_7332341_G	37G	76G
Z_7332342_T	38T	76T
Z_7332343_C	38C	75C
Z_7332344_A	38A	78A
Z_7332345_C	38C	78C
Z_7332346_A	39A	78A
Z_7332347_G	38G	78G
Z_7332348_G	39G	78G
Z_7332349_A	39A	79A
Z_7332350_A	40A	79A
Z_7332351_C	40C	79C
Z_7332352_G	39A	76G
Z_7332353_G	39G	75G
Z_7332354_A	39A	73A
Z_7332355_A	38A	74A

Z_7332356_A	38A	76A
Z_7332357_A	36A	75A
Z_7332358_A	37A	74A
Z_7332359_A	37A	75A
Z_7332360_G	37G	76G
Z_7332361_G	37G	76G
Z_7332362_T	38T	76T
Z_7332363_A	36A	73A
Z_7332364_G	35G	73G
Z_7332365_C	33C	72C
Z_7332366_A	32A	70A
Z_7332367_A	32A	69A
Z_7332368_C	30C	67C
Z_7332369_A	32A	66A
Z_7332370_T	33T	66T
Z_7332371_G	32G	64G
Z_7332372_T	32T	64T
Z_7332373_T	32T	64T
Z_7332374_T	33T	64T
Z_7332375_A	33G	36A_30G
Z_7332376_T	34T	66T
Z_7332377_G	33G	67G
Z_7332378_G	34G	65G
Z_7332379_A	32A	64A
Z_7332380_G	32G	63G
Z_7332381_G	31G	63G
Z_7332382_T	30T	63T
Z_7332383_G	30G	62G
Z_7332384_C	30C	64C
Z_7332385_A	31A	65A
Z_7332386_G	31G	66G
Z_7332387_A	30A	69A
Z_7332388_A	30A	68A
Z_7332389_A	30A	68A
Z_7332390_G	31G	68G
Z_7332391_C	30C	69C
Z_7332392_C	28C	68C
Z_7332393_A	29A	70A
Z_7332394_T	29T	69T
Z_7332395_T	32T	69T
Z_7332396_G	33G	67G
Z_7332397_C	32C	67C
Z_7332398_T	33T	66T

Z_7332399_G	33G	65G
Z_7332400_C	31C	65C
Z_7332401_A	31G	30A_37G
Z_7332402_G	29G	66G
Z_7332403_T	29T	67T
Z_7332404_G	28G	66G
Z_7332405_A	25A	65A
Z_7332406_C	25C	65C
Z_7332407_C	27C	64C
Z_7332408_C	27C	65C
Z_7332409_T	26T	63T
Z_7332410_T	26T	65T
Z_7332411_G	25G	65G
Z_7332412_A	25A	65A
Z_7332413_T	24T	67T
Z_7332414_G	24G	66G
Z_7332415_G	24G	67G
Z_7332416_C	24C	64C
Z_7332417_A	24A	65A
Z_7332418_C	23C	65C
Z_7332419_A	23A	65A
Z_7332420_A	24A	62A
Z_7332421_A	24A	60A
Z_7332422_A	25A	62A
Z_7332423_A	25A	57A
Z_7332424_A	26A	37A_23T
Z_7332425_A	23A	60A
Z_7332426_G	26G	58G
Z_7332427_G	26G	57G
Z_7332428_G	25G	56G
Z_7332429_A	23G	32A_24G
Z_7332430_A	24A	57A
Z_7332431_T	24T	59T
Z_7332432_G	24G	59G
Z_7332433_G	25G	59G
Z_7332434_A	26A	60A
Z_7332435_A	26A	60A
Z_7332436_A	26A	60A
Z_7332437_T	28T	60T
Z_7332438_G	29G	61G
Z_7332439_C	29C	60C
Z_7332440_T	29T	59T
Z_7332441_C	28C	59C

Z_7332442_C	29C	61C
Z_7332443_T	29T	61T
Z_7332444_G	31G	62G
Z_7332445_A	30A	62A
Z_7332446_C	30C	63C
Z_7332447_A	30A	65A
Z_7332448_A	30A	66A
Z_7332449_A	32A	66A
Z_7332450_C	33C	65C
Z_7332451_C	34C	62C
Z_7332452_C	36C	61C
Z_7332453_A	35A	61A
Z_7332454_A	34A	60A
Z_7332455_C	34C	60C
Z_7332456_A	33A	60A
Z_7332457_C	33C	61C
Z_7332458_A	34A	61A
Z_7332459_A	35A	59A
Z_7332460_G	35G	60G
Z_7332461_G	35G	61G
Z_7332462_C	36C	61C
Z_7332463_A	37A	61A
Z_7332464_A	38A	30A_31T
Z_7332465_G	38G	62G
Z_7332466_G	34G	61G
Z_7332467_A	35A	61A
Z_7332468_G	35G	61G
Z_7332469_T	35T	60T
Z_7332470_A	35C	59A
Z_7332471_G	35G	58G
Z_7332472_A	35A	59A
Z_7332473_T	36T	61T
Z_7332474_C	35C	61C
Z_7332475_C	35C	61C
Z_7332476_G	36G	61G
Z_7332477_C	36C	61C
Z_7332478_T	36T	61T
Z_7332479_G	36G	61G
Z_7332480_C	38C	60C
Z_7332481_A	38A	59A
Z_7332482_G	38G	57G
Z_7332483_G	37G	56G
Z_7332484_A	38A	57A

Z_7332485_A	40A	56A
Z_7332486_C	41C	55C
Z_7332487_A	40A	56A
Z_7332488_A	38A	56A
Z_7332489_C	38C	54C
Z_7332490_A	39A	54A
Z_7332491_C	38C	55C
Z_7332492_T	39T	59T
Z_7332493_G	38G	58G
Z_7332494_T	38T	57T
Z_7332495_C	36C	56C
Z_7332496_C	37C	57C
Z_7332497_A	37A	56A
Z_7332498_G	37G	54G
Z_7332499_C	34G	55C
Z_7332500_T	33C	33T_24C
Z_7332501_C	38C	59C
Z_7332502_A	38A	61A
Z_7332503_G	38G	63G
Z_7332504_T	38T	62T
Z_7332505_T	38T	58T
Z_7332506_C	38C	60C
Z_7332507_A	36A	59A
Z_7332508_G	38G	61G
Z_7332509_G	32G	55G
Z_7332510_G	30A	21A_36G
Z_7332511_G	32A	21A_36G
Z_7332512_A	38A	61A
Z_7332513_A	38A	63A
Z_7332514_A	38A	63A
Z_7332515_A	38A	62A
Z_7332516_A	38A	64A
Z_7332517_A	38A	65A
Z_7332518_A	39A	66A
Z_7332519_T	36T	62T
Z_7332520_C	34C	62C
Z_7332521_A	35A	61A
Z_7332522_C	32C	64C
Z_7332523_C	34C	65C
Z_7332524_T	34T	67T
Z_7332525_G	33G	67G
Z_7332526_T	34T	65T
Z_7332527_C	37C	67C

Z_7332528_T	36T	68T
Z_7332529_G	33G	68G
Z_7332530_A	34A	71A
Z_7332531_T	33T	71T
Z_7332532_C	33C	75C
Z_7332533_T	33T	75T
Z_7332534_A	33A	75A
Z_7332535_C	32C	77C
Z_7332536_A	33A	77A
Z_7332537_T	31T	75T
Z_7332538_T	31T	73T
Z_7332539_G	31G	72G
Z_7332540_C	33C	72C
Z_7332541_A	33A	73A
Z_7332542_C	33C	71C
Z_7332543_A	33G	36A_36G
Z_7332544_T	34T	74T
Z_7332545_T	34T	76T
Z_7332546_T	34T	76T
Z_7332547_A	35A	77A
Z_7332548_T	35T	77T
Z_7332549_A	36A	77A
Z_7332550_G	34G	77G
Z_7332551_C	35C	78C
Z_7332552_C	36C	80C
Z_7332553_A	38A	80A
Z_7332554_T	38T	80T
Z_7332555_T	37T	80T
Z_7332556_T	36T	79T
Z_7332557_G	36G	80G
Z_7332558_A	37A	81A
Z_7332559_G	36G	79G
Z_7332560_A	35A	81A
Z_7332561_C	36C	84C
Z_7332562_C	35C	84C
Z_7332563_A	37A	83A
Z_7332564_C	37C	83C
Z_7332565_A	36T	44A_36T
Z_7332566_C	33C	36T_42C
Z_7332567_G	33G	80G
Z_7332568_A	34A	76A
Z_7332569_G	34T	38T_40G
Z_7332570_C	33C	79C

Z_7332571_A	35A	81A
Z_7332572_T	36T	81T
Z_7332573_A	36A	81A
Z_7332574_G	35G	81G
Z_7332575_C	36C	81C
Z_7332576_T	37T	81T
Z_7332577_C	36C	81C
Z_7332578_C	36C	81C
Z_7332579_A	36A	82A
Z_7332580_T	36T	82T
Z_7332581_T	34T	83T
Z_7332582_C	33C	82C
Z_7332583_C	33C	83C
Z_7332584_C	32C	84C
Z_7332585_C	33C	84C
Z_7332586_A	35A	83A
Z_7332587_A	36A	79A
Z_7332588_A	35A	80A
Z_7332589_A	35A	80A
Z_7332590_T	35T	81T
Z_7332591_C	35C	81C
Z_7332592_C	35C	83C
Z_7332593_T	34T	86T
Z_7332594_G	34G	88G
Z_7332595_G	34G	88G
Z_7332596_G	34G	86G
Z_7332597_A	32A	84A
Z_7332598_T	33T	83T
Z_7332599_T	33T	84T
Z_7332600_G	33G	84G
Z_7332601_G	34G	84G
Z_7332602_T	35T	79T
Z_7332603_A	34A	79A
Z_7332604_G	34G	80G
Z_7332605_A	35A	79A
Z_7332606_T	34T	81T
Z_7332607_A	34A	83A
Z_7332608_G	34G	82G
Z_7332609_G	34G	82G
Z_7332610_G	33G	84G
Z_7332611_G	34G	83G
Z_7332612_G	34G	86G
Z_7332613_T	35T	87T

Z_7332614_A	35A	89A
Z_7332615_T	34T	90T
Z_7332616_G	32G	90G
Z_7332617_T	33T	89T
Z_7332618_A	32A	88A
Z_7332619_G	31G	87G
Z_7332620_A	30A	87A
Z_7332621_A	29A	88A
Z_7332622_A	29A	87A
Z_7332623_T	29T	87T
Z_7332624_G	29G	85G
Z_7332625_T	29T	83T
Z_7332626_C	30C	84C
Z_7332627_T	31T	80T
Z_7332628_G	31G	79G
Z_7332629_C	33C	78C
Z_7332630_T	35T	77T
Z_7332631_A	34A	79A
Z_7332632_G	34G	79G
Z_7332633_C	33C	80C
Z_7332634_A	34A	81A
Z_7332635_A	34A	80A
Z_7332636_A	36A	80A
Z_7332637_G	36G	79G
Z_7332638_C	38C	78C
Z_7332639_C	37C	78C
Z_7332640_T	37T	76T
Z_7332641_C	38C	76C
Z_7332642_C	37C	76C
Z_7332643_C	38C	78C
Z_7332644_G	42G	80G
Z_7332645_A	43A	80A
Z_7332646_A	43A	78A
Z_7332647_C	44C	75C
Z_7332648_T	44T	75T
Z_7332649_C	44C	76C
Z_7332650_T	43T	77T
Z_7332651_G	44G	78G
Z_7332652_G	44G	77G
Z_7332653_C	44C	76C
Z_7332654_A	44A	77A
Z_7332655_G	45G	75G
Z_7332656_A	42A	73A

Z_7332657_G	42G	72G
Z_7332658_A	43A	75A
Z_7332659_A	42A	76A
Z_7332660_C	41C	76C
Z_7332661_T	42T	76T
Z_7332662_C	40C	74C
Z_7332663_T	40T	73T
Z_7332664_G	41T	78G
Z_7332665_A	40A	79A
Z_7332666_A	40A	77A
Z_7332667_G	40G	78G
Z_7332668_C	38C	83C
Z_7332669_C	38C	83C
Z_7332670_C	39C	83C
Z_7332671_G	36A	82G
Z_7332672_T	36T	84T
Z_7332673_C	35C	84C
Z_7332674_C	43C	83C
Z_7332675_C	42C	84C
Z_7332676_C	42C	83C
Z_7332677_A	42A	85A
Z_7332678_A	42A	85A
Z_7332679_A	42A	85A
Z_7332680_C	42C	85C
Z_7332681_T	43T	85T
Z_7332682_G	42G	85G
Z_7332683_C	42C	84C
Z_7332684_A	42A	84A
Z_7332685_A	41A	83A
Z_7332686_A	42A	84A
Z_7332687_C	41T	83C
Z_7332688_T	44T	82T
Z_7332689_C	40C	80C
Z_7332690_C	45C	79C
Z_7332691_A	46A	80A
Z_7332692_A	46A	81A
Z_7332693_T	45T	80T
Z_7332694_A	45A	80A
Z_7332695_C	44C	80C
Z_7332696_T	44T	80T
Z_7332697_A	44A	80A
Z_7332698_G	41G	81G
Z_7332699_A	42A	81A

Z_7332700_A	40A	81A
Z_7332701_C	39C	78C
Z_7332702_A	40A	76A
Z_7332703_T	40T	75T
Z_7332704_G	39G	75G
Z_7332705_C	37C	72C
Z_7332706_C	37C	74C
Z_7332707_A	35A	70A
Z_7332708_C	33T	31T_35C
Z_7332709_C	31C	64C
Z_7332710_A	31G	33A_29G
Z_7332711_A	32A	63A
Z_7332712_G	32G	65G
Z_7332713_A	33A	66A
Z_7332714_T	32T	67T
Z_7332715_T	29T	67T
Z_7332716_T	28T	66T
Z_7332717_T	29T	67T
Z_7332718_G	28G	68G
Z_7332719_C	28C	70C
Z_7332720_T	29C	39T_31C
Z_7332721_A	30A	69A
Z_7332722_T	31T	69T
Z_7332723_A	32A	70A
Z_7332724_C	31C	72C
Z_7332725_A	32A	72A
Z_7332726_T	33T	71T
Z_7332727_A	34A	71A
Z_7332728_C	34C	70C
Z_7332729_A	33A	70A
Z_7332730_A	33A	71A
Z_7332731_A	33A	69A
Z_7332732_T	30T	70T
Z_7332733_C	31C	70C
Z_7332734_C	31C	68C
Z_7332735_C	30C	68C
Z_7332736_A	32A	68A
Z_7332737_A	33A	67A
Z_7332738_G	34G	65G
Z_7332739_A	34A	65A
Z_7332740_T	33T	64T
Z_7332741_T	34T	65T
Z_7332742_T	34T	66T

Z_7332743_T	33T	67T
Z_7332744_G	33G	67G
Z_7332745_T	33T	68T
Z_7332746_T	34T	67T
Z_7332747_G	35G	67G
Z_7332748_A	35A	67A
Z_7332749_G	36G	66G
Z_7332750_A	36A	66A
Z_7332751_T	34T	67T
Z_7332752_G	33G	68G
Z_7332753_G	34G	64G
Z_7332754_A	39A	66A
Z_7332755_G	40G	67G
Z_7332756_C	39C	67C
Z_7332757_T	38T	68T
Z_7332758_G	37G	68G
Z_7332759_T	38T	63T
Z_7332760_G	39G	65G
Z_7332761_A	38A	65A
Z_7332762_T	40T	63T
Z_7332763_A	40A	59A
Z_7332764_A	40A	60A
Z_7332765_A	41A	62A
Z_7332766_T	42T	62T
Z_7332767_G	43G	60G
Z_7332768_G	42G	60G
Z_7332769_T	44T	59T
Z_7332770_A	45A	58A
Z_7332771_T	48T	58T
Z_7332772_C	48C	57C
Z_7332773_A	48A	56A
Z_7332774_A	48A	55A
Z_7332775_A	49A	56A
Z_7332776_C	49C	56C
Z_7332777_T	50T	57T
Z_7332778_G	49G	57G
Z_7332779_A	48A	58A
Z_7332780_C	49C	60C
Z_7332781_A	49A	59A
Z_7332782_C	51C	59C
Z_7332783_A	51A	59A
Z_7332784_A	51A	60A
Z_7332785_C	49C	61C

Z_7332786_C	49C	62C
Z_7332787_T	48T	62T
Z_7332788_G	48G	63G
Z_7332789_T	48T	62T
Z_7332790_G	48G	62G
Z_7332791_T	47T	63T
Z_7332792_C	45C	64C
Z_7332793_A	44A	63A
Z_7332794_T	45T	62T
Z_7332795_G	45G	62G
Z_7332796_T	43T	62T
Z_7332797_A	43A	63A
Z_7332798_G	44G	63G
Z_7332799_A	44A	63A
Z_7332800_G	44G	63G
Z_7332801_G	45G	63G
Z_7332802_G	45G	67G
Z_7332803_T	45T	68T
Z_7332804_C	44C	72C
Z_7332805_T	47T	72T
Z_7332806_C	45C	71C
Z_7332807_C	45C	70C
Z_7332808_A	44A	70A
Z_7332809_T	44T	69T
Z_7332810_C	46C	70C
Z_7332811_T	46T	72T
Z_7332812_C	46C	71C
Z_7332813_T	46T	70T
Z_7332814_C	47C	68C
Z_7332815_T	47T	68T
Z_7332816_T	48T	68T
Z_7332817_T	47T	70T
Z_7332818_C	47C	69C
Z_7332819_T	46T	67T
Z_7332820_T	48T	67T
Z_7332821_G	48G	67G
Z_7332822_A	48A	66A
Z_7332823_A	48A	68A
Z_7332824_T	43T	67T
Z_7332825_A	42A	67A
Z_7332826_T	43T	67T
Z_7332827_C	43C	64C
Z_7332828_C	43C	65C

Z_7332829_C	42C	65C
Z_7332830_A	41A	66A
Z_7332831_C	43C	67C
Z_7332832_C	40C	68C
Z_7332833_C	40C	69C
Z_7332834_A	42A	68A
Z_7332835_G	40G	68G
Z_7332836_T	41T	67T
Z_7332837_G	40G	67G
Z_7332838_G	42G	71G
Z_7332839_T	41T	69T
Z_7332840_T	38T	68T
Z_7332841_T	36T	69T
Z_7332842_C	35C	71C
Z_7332843_T	37T	72T
Z_7332844_C	40C	73C
Z_7332845_T	41T	72T
Z_7332846_C	43C	71C
Z_7332847_T	41T	70T
Z_7332848_T	42T	70T
Z_7332849_C	42C	69C
Z_7332850_C	42T	69C
Z_7332851_T	42T	70T
Z_7332852_T	38T	71T
Z_7332853_A	39A	72A
Z_7332854_C	39C	72C
Z_7332855_A	40A	70A
Z_7332856_T	40C	71T
Z_7332857_A	39A	70A
Z_7332858_C	41C	70C
Z_7332859_C	41C	69C
Z_7332860_C	42C	69C
Z_7332861_A	42A	69A
Z_7332862_C	42C	70C
Z_7332863_A	41A	71A
Z_7332864_C	41C	71C
Z_7332865_A	41A	73A
Z_7332866_A	41A	72A
Z_7332867_A	41A	74A
Z_7332868_C	39C	73C
Z_7332869_C	40C	75C
Z_7332870_T	40T	76T
Z_7332871_G	39G	76G

Z_7332872_G	38G	77G
Z_7332873_T	42T	77T
Z_7332874_C	40C	75C
Z_7332875_T	39T	74T
Z_7332876_G	40G	73G
Z_7332877_G	39G	73G
Z_7332878_A	40A	73A
Z_7332879_G	40G	72G
Z_7332880_A	38A	70A
Z_7332881_G	38G	68G
Z_7332882_A	40A	67A
Z_7332883_A	40A	66A
Z_7332884_C	38C	69C
Z_7332885_G	39G	70G
Z_7332886_C	37C	69C
Z_7332887_A	38A	70A
Z_7332888_C	37C	71C
Z_7332889_T	39T	72T
Z_7332890_C	38C	72C
Z_7332891_T	39T	72T
Z_7332892_T	40T	73T
Z_7332893_G	39G	73G
Z_7332894_T	41T	74T
Z_7332895_T	42T	76T
Z_7332896_T	42T	76T
Z_7332897_T	44T	73T
Z_7332898_T	44T	73T
Z_7332899_A	45A	71A
Z_7332900_A	45A	73A
Z_7332901_A	44A	79A
Z_7332902_C	44C	78C
Z_7332903_G	45G	79G
Z_7332904_G	45G	81G
Z_7332905_T	45T	79T
Z_7332906_T	45T	78T
Z_7332907_G	44G	77G
Z_7332908_T	43T	77T
Z_7332909_G	43G	76G
Z_7332910_G	34T_77G	30T_107G
Z_7332911_G	44G	77G
Z_7332912_T	44T	77T
Z_7332913_A	42G	45A_30G
Z_7332914_T	44T	76T

Z_7332915_C	42C	77C
Z_7332916_G	43G	77G
Z_7332917_G	41G	77G
Z_7332918_A	45A	78A
Z_7332919_G	45G	78G
Z_7332920_G	46G	79G
Z_7332921_T	47T	80T
Z_7332922_T	49T	80T
Z_7332923_T	49T	79T
Z_7332924_T	49T	79T
Z_7332925_T	49T	79T
Z_7332926_A	48A	79A
Z_7332927_T	47T	77T
Z_7332928_G	46G	76G
Z_7332929_G	45G	76G
Z_7332930_A	47A	77A
Z_7332931_C	47C	74C
Z_7332932_C	47C	74C
Z_7332933_A	48A	74A
Z_7332934_G	48G	75G
Z_7332935_G	46G	73G
Z_7332936_A	46A	73A
Z_7332937_T	46T	74T
Z_7332938_T	45T	74T
Z_7332939_T	46T	73T
Z_7332940_G	47G	73G
Z_7332941_T	45T	75T
Z_7332942_A	45A	77A
Z_7332943_C	43C	77C
Z_7332944_A	44A	77A
Z_7332945_G	43G	78G
Z_7332946_C	44C	79C
Z_7332947_C	42C	78C
Z_7332948_T	42T	78T
Z_7332949_A	41A	80A
Z_7332950_T	40T	80T
Z_7332951_A	42A	80A
Z_7332952_G	42G	80G
Z_7332953_C	42C	81C
Z_7332954_A	41A	81A
Z_7332955_A	40A	78A
Z_7332956_A	41A	76A
Z_7332957_G	41G	77G

Z_7332958_A	40A	74A
Z_7332959_T	41T	75T
Z_7332960_G	41G	74G
Z_7332961_C	42C	75C
Z_7332962_T	40T	77T
Z_7332963_G	38G	78G
Z_7332964_C	39C	79C
Z_7332965_C	36C	76C
Z_7332966_T	36T	76T
Z_7332967_T	35T	77T
Z_7332968_C	35C	77C
Z_7332969_A	36A	76A
Z_7332970_C	34C	76C
Z_7332971_G	32G	74G
Z_7332972_T	33T	74T
Z_7332973_C	33C	74C
Z_7332974_A	34A	75A
Z_7332975_A	35A	76A
Z_7332976_T	35T	76T
Z_7332977_G	33G	76G
Z_7332978_A	33A	79A
Z_7332979_A	32A	79A
Z_7332980_C	33C	79C
Z_7332981_A	33A	79A
Z_7332982_G	33G	79G
Z_7332983_A	32A	81A
Z_7332984_A	33A	81A
Z_7332985_A	32A	81A
Z_7332986_A	32A	82A
Z_7332987_G	32G	81G
Z_7332988_C	33C	80C
Z_7332989_A	35A	77A
Z_7332990_A	33A	76A
Z_7332991_C	31C	75C
Z_7332992_C	30C	73C
Z_7332993_T	30T	72T
Z_7332994_C	30C	33T_39C
Z_7332995_T	31T	71T
Z_7332996_T	32T	65T
Z_7332997_C	30C	64C
Z_7332998_T	35T	65T
Z_7332999_A	35A	66A
Z_7333000_A	34A	66A

Z_7333001_A	36A	66A
Z_7333002_G	36G	66G
Z_7333003_C	35C	65C
Z_7333004_A	36A	64A
Z_7333005_C	36C	64C
Z_7333006_A	36A	64A
Z_7333007_T	38T	61T
Z_7333008_C	37C	61C
Z_7333009_T	41T	62T
Z_7333010_G	40G	62G
Z_7333011_T	41T	62T
Z_7333012_G	41G	62G
Z_7333013_C	41C	61C
Z_7333014_A	41A	61A
Z_7333015_C	42C	63C
Z_7333016_A	42A	62A
Z_7333017_T	44T	62T
Z_7333018_C	44C	62C
Z_7333019_T	45T	61T
Z_7333020_G	44G	61G
Z_7333021_T	42T	61T
Z_7333022_G	42G	62G
Z_7333023_C	42C	62C
Z_7333024_A	43A	61A
Z_7333025_G	43G	61G
Z_7333026_G	41G	62G
Z_7333027_T	43T	62T
Z_7333028_G	43G	60G
Z_7333029_T	41T	60T
Z_7333030_T	38T	60T
Z_7333031_T	41T	61T
Z_7333032_G	41G	59G
Z_7333033_C	42C	59C
Z_7333034_A	42A	59A
Z_7333035_T	43T	60T
Z_7333036_T	43T	59T
Z_7333037_C	43C	58C
Z_7333038_A	43A	58A
Z_7333039_G	42G	56G
Z_7333040_T	41T	55T
Z_7333041_C	42C	53C
Z_7333042_A	40A	50A
Z_7333043_C	45C	52C

Z_7333044_A	45A	49A
Z_7333045_C	44C	50C
Z_7333046_A	44A	50A
Z_7333047_C	45C	51C
Z_7333048_A	46A	50A
Z_7333049_C	46C	50C
Z_7333050_A	45A	49A
Z_7333051_C	42C	50C
Z_7333052_A	43A	50A
Z_7333053_C	42C	50C
Z_7333054_A	41A	51A
Z_7333055_C	42C	50C
Z_7333056_A	43A	48A
Z_7333057_C	44C	47C
Z_7333058_G	41G	46G
Z_7333059_C	39C	44C
Z_7333060_A	40A	43A
Z_7333061_G	40G	43G
Z_7333062_A	40A	42A
Z_7333063_A	42A	43A
Z_7333064_G	43G	43G
Z_7333065_C	42C	42C
Z_7333066_C	42C	44C
Z_7333067_T	43T	44T
Z_7333068_A	41A	45A
Z_7333069_G	42G	44G
Z_7333070_A	41A	42A
Z_7333071_A	40A	44A
Z_7333072_A	41A	44A
Z_7333073_G	42G	41G
Z_7333074_G	40G	40G
Z_7333075_G	41G	38G
Z_7333076_T	42T	35T
Z_7333077_G	41G	34G
Z_7333078_G	40G	31G
Z_7333079_G	38G	31G
Z_7333080_C	38C	31C
Z_7333081_T	37T	30T
Z_7333082_T	38T	30T
Z_7333083_G	39G	31G
Z_7333084_T	38T	31T
Z_7333085_A	37A	31A
Z_7333086_G	36G	31G

Z_7333087_A	34A	31A
Z_7333088_A	33A	31A
Z_7333089_C	33T	11T_20C
Z_7333090_T	34T	33T
Z_7333091_G	37G	33G
Z_7333092_A	37A	30A
Z_7333093_T	39T	31T
Z_7333094_T	37T	30T
Z_7333095_C	37C	30C
Z_7333096_C	37C	31C
Z_7333097_A	38A	31A
Z_7333098_A	38A	31A
Z_7333099_A	38A	32A
Z_7333100_G	39G	33G
Z_7333101_G	35G	33G
Z_7333102_G	39G	33G
Z_7333103_T	39T	32T
Z_7333104_G	40G	35G
Z_7333105_G	39G	36G
Z_7333106_G	40G	34G
Z_7333107_A	40A	35A
Z_7333108_G	40G	35G
Z_7333109_C	40C	35C
Z_7333110_A	39A	34A
Z_7333111_A	40A	34A
Z_7333112_A	39A	35A
Z_7333113_A	37A	35A
Z_7333114_C	38C	12G_23C
Z_7333115_A	40A	35A
Z_7333116_G	41G	35G
Z_7333117_T	42T	35T
Z_7333118_C	41C	35C
Z_7333119_A	40A	36A
Z_7333120_G	41G	35G
Z_7333121_G	42G	35G
Z_7333122_G	42G	35G
Z_7333123_C	43C	36C
Z_7333124_T	43T	34T
Z_7333125_T	43T	34T
Z_7333126_T	41T	34T
Z_7333127_T	42T	33T
Z_7333128_A	42A	32A
Z_7333129_A	44A	32A

Z_7333130_A	43A	31A
Z_7333131_G	42G	30G
Z_7333132_C	42C	30C
Z_7333133_T	43T	31T
Z_7333134_G	43G	30G
Z_7333135_G	42G	32G
Z_7333136_T	43T	33T
Z_7333137_C	42C	33C
Z_7333138_C	42C	35C
Z_7333139_T	41T	36T
Z_7333140_A	41A	37A
Z_7333141_A	43A	35A
Z_7333142_A	43A	36A
Z_7333143_G	42G	36G
Z_7333144_A	42A	36A
Z_7333145_C	41C	37C
Z_7333146_C	42C	37C
Z_7333147_G	42G	37G
Z_7333148_G	42G	37G
Z_7333149_G	41G	39G
Z_7333150_A	42A	39A
Z_7333151_G	41G	39G
Z_7333152_G	41G	39G
Z_7333153_A	40A	40A
Z_7333154_A	40A	40A
Z_7333155_T	40T	40T
Z_7333156_C	40C	40C
Z_7333157_G	41G	41G
Z_7333158_T	42T	41T
Z_7333159_C	43C	42C
Z_7333160_C	42C	43C
Z_7333161_A	41A	43A
Z_7333162_A	40A	44A
Z_7333163_C	42C	43C
Z_7333164_T	42T	43T
Z_7333165_C	41C	45C
Z_7333166_C	41C	44C
Z_7333167_C	40C	44C
Z_7333168_A	43A	45A
Z_7333169_T	43T	45T
Z_7333170_C	41C	45C
Z_7333171_A	42A	45A
Z_7333172_C	42C	45C

Z_7333173_C	41C	45C
Z_7333174_C	39C	45C
Z_7333175_T	40T	45T
Z_7333176_C	39C	45C
Z_7333177_A	38A	45A
Z_7333178_C	38C	45C
Z_7333179_C	38C	48C
Z_7333180_A	41A	48A
Z_7333181_G	43G	48G
Z_7333182_C	42C	48C
Z_7333183_C	44C	48C
Z_7333184_A	43A	46A
Z_7333185_T	42T	44T
Z_7333186_G	41G	44G
Z_7333187_T	39T	44T
Z_7333188_T	39T	47T
Z_7333189_G	39G	47G
Z_7333190_G	38G	47G
Z_7333191_C	38C	47C
Z_7333192_T	39T	49T
Z_7333193_G	38G	49G
Z_7333194_A	40A	49A
Z_7333195_G	40G	51G
Z_7333196_G	41G	51G
Z_7333197_A	41A	51A
Z_7333198_C	40C	53C
Z_7333199_G	40G	54G
Z_7333200_A	39A	53A
Z_7333201_T	40T	54T
Z_7333202_G	41G	54G
Z_7333203_G	38G	54G
Z_7333204_G	38G	55G
Z_7333205_A	38A	53A
Z_7333206_G	40G	56G
Z_7333207_C	40C	55C
Z_7333208_T	39T	56T
Z_7333209_G	39G	56G
Z_7333210_T	39T	56T
Z_7333211_A	37A	55A
Z_7333212_G	38G	55G
Z_7333213_T	38T	56T
Z_7333214_G	39G	55G
Z_7333215_A	37A	54A

Z_7333216_G	40G	54G
Z_7333217_A	40A	55A
Z_7333218_A	40A	54A
Z_7333219_C	38C	54C
Z_7333220_T	36T	55T
Z_7333221_T	38T	55T
Z_7333222_C	37C	55C
Z_7333223_G	38G	55G
Z_7333224_G	40G	57G
Z_7333225_C	40C	58C
Z_7333226_A	41A	58A
Z_7333227_G	40G	58G
Z_7333228_G	40G	59G
Z_7333229_G	40G	59G
Z_7333230_T	41T	58T
Z_7333231_G	39G	57G
Z_7333232_C	38C	57C
Z_7333233_G	39G	54G
Z_7333234_A	40A	53A
Z_7333235_G	39G	57G
Z_7333236_G	40G	58G
Z_7333237_T	41T	57T
Z_7333238_T	36T	57T
Z_7333239_C	39C	56C
Z_7333240_A	38A	56A
Z_7333241_C	39C	57C
Z_7333242_C	38C	57C
Z_7333243_C	40C	57C
Z_7333244_A	40A	55A
Z_7333245_A	40A	54A
Z_7333246_G	39G	55G
Z_7333247_C	40C	56C
Z_7333248_G	40G	55G
Z_7333249_G	39G	55G
Z_7333250_C	37C	55C
Z_7333251_T	37T	57T
Z_7333252_T	35T	57T
Z_7333253_C	36C	59C
Z_7333254_A	38A	58A
Z_7333255_G	38A	26A_29G
Z_7333256_A	39A	55A
Z_7333257_A	40A	54A
Z_7333258_C	42C	25T_29C

Z_7333259_G	42G	54G
Z_7333260_A	42A	54A
Z_7333261_A	41A	54A
Z_7333262_A	40A	57A
Z_7333263_A	42A	57A
Z_7333264_C	42C	58C
Z_7333265_C	40C	61C
Z_7333266_C	40C	64C
Z_7333267_A	39A	63A
Z_7333268_A	42A	63A
Z_7333269_T	42T	63T
Z_7333270_G	44G	63G
Z_7333271_C	44C	63C
Z_7333272_A	44A	62A
Z_7333273_A	47A	63A
Z_7333274_C	47C	60C
Z_7333275_A	49A	59A
Z_7333276_C	50C	58C
Z_7333277_A	51A	59A
Z_7333278_G	51G	60G
Z_7333279_A	50A	59A
Z_7333280_G	50G	59G
Z_7333281_A	51A	59A
Z_7333282_G	51G	60G
Z_7333283_G	52G	58G
Z_7333284_G	50G	25A_31G
Z_7333285_A	48A	55A
Z_7333286_A	48A	55A
Z_7333287_C	48C	54C
Z_7333288_A	48A	54A
Z_7333289_T	51T	53T
Z_7333290_T	50T	53T
Z_7333291_T	51T	53T
Z_7333292_G	51G	54G
Z_7333293_T	50T	52T
Z_7333294_A	50A	51A
Z_7333295_C	48C	52C
Z_7333296_A	49A	52A
Z_7333297_A	48A	52A
Z_7333298_A	47A	52A
Z_7333299_G	47G	50G
Z_7333300_A	48A	53A
Z_7333301_A	48A	53A

Z_7333302_G	49G	53G
Z_7333303_T	47T	53T
Z_7333304_C	46C	55C
Z_7333305_T	46T	55T
Z_7333306_G	46G	55G
Z_7333307_G	46G	56G
Z_7333308_A	45A	58A
Z_7333309_G	47G	58G
Z_7333310_G	46G	59G
Z_7333311_G	46G	59G
Z_7333312_A	46A	58A
Z_7333313_T	45T	58T
Z_7333314_C	44C	59C
Z_7333315_C	44C	58C
Z_7333316_C	47C	58C
Z_7333317_A	48A	58A
Z_7333318_A	47A	58A
Z_7333319_A	47A	56A
Z_7333320_G	47G	55G
Z_7333321_G	46G	54G
Z_7333322_C	47C	55C
Z_7333323_A	47A	53A
Z_7333324_G	45G	55G
Z_7333325_G	44G	56G
Z_7333326_G	44G	57G
Z_7333327_C	43C	57C
Z_7333328_C	39C	57C
Z_7333329_C	43C	56C
Z_7333330_T	45T	53T
Z_7333331_G	44G	53G
Z_7333332_T	45T	53T
Z_7333333_T	43T	54T
Z_7333334_T	43T	54T
Z_7333335_G	44G	54G
Z_7333336_C	45C	54C
Z_7333337_T	46T	53T
Z_7333338_A	44A	53A
Z_7333339_A	44A	53A
Z_7333340_G	42G	53G
Z_7333341_G	42G	54G
Z_7333342_C	41C	53C
Z_7333343_T	34T	53T
Z_7333344_C	37C	54C

Z_7333345_A	36A	53A
Z_7333346_A	34A	52A
Z_7333347_A	36A	50A
Z_7333348_A	37A	48A
Z_7333349_A	37A	48A
Z_7333350_G	37G	48G
Z_7333351_A	39A	48A
Z_7333352_G	38G	48G
Z_7333353_G	38G	48G
Z_7333354_A	38A	48A
Z_7333355_G	38G	47G
Z_7333356_C	39C	46C
Z_7333357_T	37T	44T
Z_7333358_G	40G	42G
Z_7333359_C	39C	43C
Z_7333360_A	39A	42A
Z_7333361_G	40G	40G
Z_7333362_G	41G	41G
Z_7333363_A	41A	41A
Z_7333364_G	42G	41G
Z_7333365_A	43A	43A
Z_7333366_C	42C	45C
Z_7333367_A	42A	46A
Z_7333368_A	42A	46A
Z_7333369_A	42A	46A
Z_7333370_A	43A	46A
Z_7333371_C	43C	46C
Z_7333372_A	42A	47A
Z_7333373_G	41G	46G
Z_7333374_A	43A	45A
Z_7333375_G	45G	45G
Z_7333376_A	44A	45A
Z_7333377_G	46G	44G
Z_7333378_A	46A	44A
Z_7333379_A	43A	44A
Z_7333380_T	43T	45T
Z_7333381_C	43C	45C
Z_7333382_C	42C	46C
Z_7333383_A	42T	26A_20T
Z_7333384_A	41A	46A
Z_7333385_A	41A	46A
Z_7333386_G	40G	47G
Z_7333387_G	39G	47G

Z_7333388_T	40T	47T
Z_7333389_A	40A	47A
Z_7333390_C	39C	46C
Z_7333391_A	38A	46A
Z_7333392_C	38C	46C
Z_7333393_A	38A	46A
Z_7333394_A	40A	46A
Z_7333395_T	38T	43T
Z_7333396_A	39A	41A
Z_7333397_A	40A	42A
Z_7333398_T	40T	41T
Z_7333399_A	37A	40A
Z_7333400_G	37G	41G
Z_7333401_C	37C	41C
Z_7333402_A	37A	40A
Z_7333403_T	37T	37T
Z_7333404_C	35C	38C
Z_7333405_C	33C	37C
Z_7333406_C	32C	41C
Z_7333407_A	31A	43A
Z_7333408_A	31A	43A
Z_7333409_C	30C	44C
Z_7333410_T	31T	46T
Z_7333411_C	31C	46C
Z_7333412_T	31T	47T
Z_7333413_C	31C	48C
Z_7333414_A	32A	48A
Z_7333415_G	32G	48G
Z_7333416_C	31C	48C
Z_7333417_G	29G	48G
Z_7333418_G	29G	50G
Z_7333419_A	30A	48A
Z_7333420_G	29G	47G
Z_7333421_A	26A	45A
Z_7333422_C	27T	47C
Z_7333423_T	28T	48T
Z_7333424_C	27C	49C
Z_7333425_T	28T	52T
Z_7333426_G	27G	51G
Z_7333427_G	26G	52G
Z_7333428_G	24G	51G
Z_7333429_A	24A	51A
Z_7333430_A	24A	51A

Z_7333431_G	23G	51G
Z_7333432_A	22A	51A
Z_7333433_G	22G	53G
Z_7333434_C	20C	53C
Z_7333435_G	19G	53G
Z_7333436_T	19T	52T
Z_7333437_G	19G	54G
Z_7333438_T	20T	54T
Z_7333439_G	20G	53G
Z_7333440_C	18C	54C
Z_7333441_T	19T	54T
Z_7333442_T	20T	56T
Z_7333443_C	18C	56C
Z_7333444_G	18G	57G
Z_7333445_A	17A	57A
Z_7333446_A	19A	57A
Z_7333447_G	19G	57G
Z_7333448_C	21C	58C
Z_7333449_A	21A	59A
Z_7333450_G	21G	59G
Z_7333451_A	22A	60A
Z_7333452_G	22G	59G
Z_7333453_T	22T	60T
Z_7333454_T	23T	59T
Z_7333455_T	23T	60T
Z_7333456_A	25A	59A
Z_7333457_A	26A	58A
Z_7333458_A	25A	58A
Z_7333459_A	27A	58A
Z_7333460_A	27A	57A
Z_7333461_G	29G	55G
Z_7333462_A	30A	55A
Z_7333463_A	30A	54A
Z_7333464_C	28C	54C
Z_7333465_A	28A	54A
Z_7333466_G	27G	54G
Z_7333467_A	27A	52A
Z_7333468_G	28G	52G
Z_7333469_A	28A	52A
Z_7333470_G	28G	54G
Z_7333471_G	27G	54G
Z_7333472_A	27A	54A
Z_7333473_G	27G	54G

Z_7333474_G	27G	56G
Z_7333475_A	30A	55A
Z_7333476_C	30C	55C
Z_7333477_A	30A	54A
Z_7333478_G	31G	55G
Z_7333479_A	32A	55A
Z_7333480_A	31A	56A
Z_7333481_A	32A	55A
Z_7333482_G	33G	54G
Z_7333483_A	34A	53A
Z_7333484_C	33C	53C
Z_7333485_T	34T	53T
Z_7333486_A	34A	53A
Z_7333487_A	35A	53A
Z_7333488_A	34A	54A
Z_7333489_A	35A	55A
Z_7333490_G	37G	55G
Z_7333491_G	36G	56G
Z_7333492_G	37G	54G
Z_7333493_A	36A	55A
Z_7333494_G	36G	55G
Z_7333495_A	36A	54A
Z_7333496_G	36G	54G
Z_7333497_C	35T	54C
Z_7333498_T	36T	54T
Z_7333499_C	37C	54C
Z_7333500_G	37G	54G
Z_7333501_C	38C	52C
Z_7333502_T	39T	50T
Z_7333503_G	39G	48G
Z_7333504_G	38G	48G
Z_7333505_A	39A	46A
Z_7333506_A	39A	47A
Z_7333507_T	39T	46T
Z_7333508_T	40T	45T
Z_7333509_A	40A	47A
Z_7333510_G	39G	47G
Z_7333511_A	39A	48A
Z_7333512_G	38G	47G
Z_7333513_T	39T	45T
Z_7333514_G	40G	45G
Z_7333515_T	39T	45T
Z_7333516_A	37A	46A

Z_7333517_A	36A	48A
Z_7333518_G	35G	47G
Z_7333519_C	34C	44C
Z_7333520_C	35C	43C
Z_7333521_A	34A	44A
Z_7333522_A	82A	76A
Z_7333523_A	18A	32A
Z_7333524_A	17A	34A
Z_7333525_A	16A	36A
Z_7333526_C	15C	37C
Z_7333527_A	39A	48A
Z_7333528_A	33A	46A
Z_7333529_A	32A	47A
Z_7333530_A	32A	48A
Z_7333531_C	30C	48C
Z_7333532_A	30A	46A
Z_7333533_A	31A	48A
Z_7333534_A	32A	50A
Z_7333535_A	32A	50A
Z_7333536_C	31C	51C
Z_7333537_A	31A	50A
Z_7333538_A	30A	51A
Z_7333539_A	31A	50A
Z_7333540_A	32A	50A
Z_7333541_C	32C	52C
Z_7333542_A	33A	52A
Z_7333543_A	33A	50A
Z_7333544_A	33A	50A
Z_7333545_A	27A	46A
Z_7333546_C	28C	47C
Z_7333547_A	30A	48A
Z_7333548_A	30A	49A
Z_7333549_A	30A	49A
Z_7333550_G	31G	47G
Z_7333551_G	31G	47G
Z_7333552_A	33A	48A
Z_7333553_A	31A	49A
Z_7333554_A	33A	51A
Z_7333555_A	33A	51A
Z_7333556_A	33A	52A
Z_7333557_C	32C	21A_32C
Z_7333558_C	34C	53C
Z_7333559_A	34A	53A

Z_7333560_T	35T	54T
Z_7333561_A	35A	54A
Z_7333562_T	34T	54T
Z_7333563_A	33A	57A
Z_7333564_G	32G	58G
Z_7333565_G	31G	59G
Z_7333566_C	33C	60C
Z_7333567_T	33T	61T
Z_7333568_C	35C	63C
Z_7333569_A	36A	62A
Z_7333570_G	36G	61G
Z_7333571_C	35C	62C
Z_7333572_T	36T	61T
Z_7333573_C	36C	63C
Z_7333574_T	37T	62T
Z_7333575_G	38G	61G
Z_7333576_A	37A	61A
Z_7333577_A	36A	62A
Z_7333578_G	35G	61G
Z_7333579_C	36C	60C
Z_7333580_T	37T	59T
Z_7333581_C	37C	60C
Z_7333582_A	36A	60A
Z_7333583_A	35A	59A
Z_7333584_A	39A	58A
Z_7333585_G	38G	58G
Z_7333586_G	37G	58G
Z_7333587_G	37G	58G
Z_7333588_C	38C	58C
Z_7333589_A	36A	59A
Z_7333590_A	37A	59A
Z_7333591_A	36A	58A
Z_7333592_G	36G	58G
Z_7333593_A	35A	57A
Z_7333594_G	34G	56G
Z_7333595_A	32A	56A
Z_7333596_A	33A	55A
Z_7333597_A	34A	56A
Z_7333598_G	34G	56G
Z_7333599_A	32A	59A
Z_7333600_T	34T	60T
Z_7333601_C	34C	60C
Z_7333602_A	34A	60A

Z_7333603_T	33T	60T
Z_7333604_C	33C	59C
Z_7333605_C	33C	61C
Z_7333606_T	33T	59T
Z_7333607_A	34A	59A
Z_7333608_G	35G	59G
Z_7333609_G	34G	25A_33G
Z_7333610_A	33A	58A
Z_7333611_T	32T	58T
Z_7333612_G	32G	58G
Z_7333613_T	32T	58T
Z_7333614_C	33C	58C
Z_7333615_C	33C	58C
Z_7333616_C	33C	57C
Z_7333617_C	32C	57C
Z_7333618_A	33A	57A
Z_7333619_A	33A	55A
Z_7333620_A	31A	53A
Z_7333621_A	33A	51A
Z_7333622_A	31A	51A
Z_7333623_C	31C	51C
Z_7333624_T	31T	50T
Z_7333625_G	31G	48G
Z_7333626_A	30A	50A
Z_7333627_G	30G	51G
Z_7333628_C	31C	49C
Z_7333629_A	31A	47A
Z_7333630_C	29C	46C
Z_7333631_T	31T	45T
Z_7333632_C	33C	45C
Z_7333633_A	33A	44A
Z_7333634_A	32A	46A
Z_7333635_G	31G	46G
Z_7333636_C	30C	44C
Z_7333637_A	30A	44A
Z_7333638_A	28A	45A
Z_7333639_A	28A	45A
Z_7333640_G	30G	46G
Z_7333641_G	30G	45G
Z_7333642_G	32G	46G
Z_7333643_G	32G	45G
Z_7333644_G	30T	21T_23G
Z_7333645_T	31T	42T

Z_7333646_T	31T	42T
Z_7333647_T	31T	41T
Z_7333648_T	31T	41T
Z_7333649_T	31T	40T
Z_7333650_T	31T	41T
Z_7333651_G	31G	41G
Z_7333652_A	32A	40A
Z_7333653_G	34G	40G
Z_7333654_A	30A	40A
Z_7333655_C	28C	39C
Z_7333656_C	31C	40C
Z_7333657_C	32C	39C
Z_7333658_C	33C	37C
Z_7333659_T	33T	37T
Z_7333660_G	33G	35G
Z_7333661_G	33G	34G
Z_7333662_C	32C	32C
Z_7333663_A	34G	15A_15G
Z_7333664_G	33G	30G
Z_7333665_G	33G	32G
Z_7333666_A	34A	31A
Z_7333667_G	33G	33G
Z_7333668_A	34A	32A
Z_7333669_T	34T	32T
Z_7333670_A	37A	32A
Z_7333671_G	37G	32G
Z_7333672_A	35A	31A
Z_7333673_C	36C	32C
Z_7333674_A	36A	32A
Z_7333675_A	37A	31A
Z_7333676_A	37A	33A
Z_7333677_A	37A	33A
Z_7333678_G	37G	33G
Z_7333679_G	38G	33G
Z_7333680_G	39G	35G
Z_7333681_A	38A	35A
Z_7333682_T	39T	36T
Z_7333683_G	38G	15A_21G
Z_7333684_G	38G	35G
Z_7333685_G	38G	36G
Z_7333686_A	36A	36A
Z_7333687_G	37G	37G
Z_7333688_C	35C	37C

Z_7333689_A	38A	37A
Z_7333690_T	36T	36T
Z_7333691_C	36C	38C
Z_7333692_C	36C	39C
Z_7333693_T	37T	38T
Z_7333694_T	38T	37T
Z_7333695_C	37C	36C
Z_7333696_C	37C	35C
Z_7333697_C	37C	35C
Z_7333698_A	36A	34A
Z_7333699_G	35G	36G
Z_7333700_C	36C	36C
Z_7333701_C	34C	36C
Z_7333702_A	31A	38A
Z_7333703_C	31C	38C
Z_7333704_A	32A	38A
Z_7333705_G	32G	38G
Z_7333706_A	35A	38A
Z_7333707_G	35G	38G
Z_7333708_G	35G	39G
Z_7333709_A	36A	40A
Z_7333710_A	34A	40A
Z_7333711_A	34A	40A
Z_7333712_T	33T	41T
Z_7333713_T	32T	42T
Z_7333714_A	32A	44A
Z_7333715_A	31A	44A
Z_7333716_G	31G	45G
Z_7333717_G	30G	45G
Z_7333718_A	31A_25G	46A_22G
Z_7333719_G	33G	50G
Z_7333720_G	34G	51G
Z_7333721_G	34G	50G
Z_7333722_G	34G	51G
Z_7333723_G	32G	51G
Z_7333724_G	33G	53G
Z_7333725_G	33G	52G
Z_7333726_C	31C	52C
Z_7333727_A	31A	53A
Z_7333728_T	31T	53T
Z_7333729_A	30A	51A
Z_7333730_A	29A	51A
Z_7333731_T	31T	51T

Z_7333732_T	30T	51T
Z_7333733_T	31T	51T
Z_7333734_T	32T	53T
Z_7333735_C	32C	53C
Z_7333736_C	33C	54C
Z_7333737_C	33C	53C
Z_7333738_T	32T	54T
Z_7333739_T	30T	56T
Z_7333740_A	32A	56A
Z_7333741_A	32A	56A
Z_7333742_G	33G	55G
Z_7333743_T	33T	54T
Z_7333744_G	33G	54G
Z_7333745_G	33G	54G
Z_7333746_T	32T	54T
Z_7333747_T	32T	54T
Z_7333748_G	32G	54G
Z_7333749_T	34T	54T
Z_7333750_G	36G	54G
Z_7333751_C	36C	53C
Z_7333752_C	35C	53C
Z_7333753_C	36C	51C
Z_7333754_A	36A	51A
Z_7333755_A	37A	52A
Z_7333756_G	35G	53G
Z_7333757_C	37C	54C
Z_7333758_T	36T	55T
Z_7333759_T	37T	55T
Z_7333760_T	37T	51T
Z_7333761_C	37C	53C
Z_7333762_C	38C	53C
Z_7333763_C	38C	53C
Z_7333764_C	38C	53C
Z_7333765_C	41C	53C
Z_7333766_T	42T	55T
Z_7333767_A	41A	56A
Z_7333768_T	41T	55T
Z_7333769_T	41T	55T
Z_7333770_T	42T	55T
Z_7333771_T	43T	53T
Z_7333772_T	43T	53T
Z_7333773_A	43A	53A
Z_7333774_T	44T	54T

Z_7333775_A	42A	52A
Z_7333776_G	39G	51G
Z_7333777_G	41G	52G
Z_7333778_C	39C	52C
Z_7333779_C	39C	52C
Z_7333780_A	40A	52A
Z_7333781_G	39G	53G
Z_7333782_A	39A	55A
Z_7333783_A	38A	57A
Z_7333784_G	39G	57G
Z_7333785_C	40C	56C
Z_7333786_G	42G	56G
Z_7333787_G	43G	55G
Z_7333788_A	43A	52A
Z_7333789_C	43C	56C
Z_7333790_T	43T	56T
Z_7333791_G	42G	57G
Z_7333792_T	42T	57T
Z_7333793_T	40T	56T
Z_7333794_C	42C	57C
Z_7333795_A	41A	55A
Z_7333796_G	42G	54G
Z_7333797_G	40G	54G
Z_7333798_T	43T	53T
Z_7333799_A	43A	53A
Z_7333800_G	44G	54G
Z_7333801_T	44T	55T
Z_7333802_G	44G	55G
Z_7333803_A	42A	53A
Z_7333804_T	42T	53T
Z_7333805_G	42G	53G
Z_7333806_A	42A	53A
Z_7333807_T	41T	51T
Z_7333808_G	41G	49G
Z_7333809_A	40A	47A
Z_7333810_T	38T	48T
Z_7333811_G	38G	48G
Z_7333812_G	36G	49G
Z_7333813_C	38C	48C
Z_7333814_A	37A	46A
Z_7333815_G	37G	46G
Z_7333816_G	37G	45G
Z_7333817_A	37A	42A

Z_7333818_T	38T	43T
Z_7333819_G	35G	40G
Z_7333820_C	33C	42C
Z_7333821_T	34T	41T
Z_7333822_G	34G	42G
Z_7333823_C	34C	41C
Z_7333824_G	34G	41G
Z_7333825_C	33C	38C
Z_7333826_C	32C	42C
Z_7333827_C	31C	43C
Z_7333828_C	30C	41C
Z_7333829_C	30C	42C
Z_7333830_C	31C	40C
Z_7333831_C	31C	39C
Z_7333832_C	30C	38C
Z_7333833_T	27T	36T
Z_7333834_T	29T	34T
Z_7333835_T	25T	35T
Z_7333836_T	25T	33T
Z_7333837_C	24C	36C
Z_7333838_A	24A	37A
Z_7333839_G	25G	40G
Z_7333840_G	25G	41G
Z_7333841_A	24A	40A
Z_7333842_G	24G	41G
Z_7333843_G	25G	43G
Z_7333844_C	25C	43C
Z_7333845_A	24A	43A
Z_7333846_A	24A	43A
Z_7333847_C	26C	44C
Z_7333848_C	27C	45C
Z_7333849_C	26C	45C
Z_7333850_A	26A	45A
Z_7333851_C	26C	45C
Z_7333852_C	28C	46C
Z_7333853_C	28C	47C
Z_7333854_C	29C	47C
Z_7333855_T	27T	45T
Z_7333856_T	25T	48T
Z_7333857_T	25T	48T
Z_7333858_G	23G	48G
Z_7333859_C	22C	48C
Z_7333860_C	21C	47C

Z_7333861_C	21C	45C
Z_7333862_T	22T	45T
Z_7333863_G	20G	45G
Z_7333864_A	22A	44A
Z_7333865_A	21A	48A
Z_7333866_C	22C	49C
Z_7333867_T	23T	49T
Z_7333868_G	22G	50G
Z_7333869_C	23C	50C
Z_7333870_T	21T	52T
Z_7333871_T	23T	54T
Z_7333872_C	25C	54C
Z_7333873_C	24C	54C
Z_7333874_T	25T	54T
Z_7333875_G	27G	54G
Z_7333876_A	27A	53A
Z_7333877_G	27G	50G
Z_7333878_A	27A	51A
Z_7333879_A	27A	52A
Z_7333880_T	28T	55T
Z_7333881_C	28C	55C
Z_7333882_G	28G	54G
Z_7333883_G	27G	54G
Z_7333884_C	27C	54C
Z_7333885_C	28C	54C
Z_7333886_T	28T	53T
Z_7333887_C	26C	53C
Z_7333888_C	26C	54C
Z_7333889_A	26A	52A
Z_7333890_G	25G	52G
Z_7333891_A	26A	52A
Z_7333892_C	27C	53C
Z_7333893_A	27A	52A
Z_7333894_A	29A	52A
Z_7333895_T	29T	51T
Z_7333896_C	29C	52C
Z_7333897_T	30T	52T
Z_7333898_T	31T	52T
Z_7333899_C	32C	52C
Z_7333900_C	32C	53C
Z_7333901_A	33A	54A
Z_7333902_A	34A	54A
Z_7333903_G	34G	54G

Z_7333904_G	34G	56G
Z_7333905_C	37C	60C
Z_7333906_A	37A	59A
Z_7333907_G	37G	58G
Z_7333908_A	37C	33A_1T_24C
Z_7333909_C	37C	58C
Z_7333910_G	35G	58G
Z_7333911_C	37C	59C
Z_7333912_A	37A	59A
Z_7333913_T	37T	60T
Z_7333914_G	34G	60G
Z_7333915_G	35G	60G
Z_7333916_A	36A	61A
Z_7333917_G	34G	63G
Z_7333918_C	34C	64C
Z_7333919_C	34C	65C
Z_7333920_A	37A	66A
Z_7333921_A	36A	66A
Z_7333922_G	36G	65G
Z_7333923_C	36C	65C
Z_7333924_C	36C	65C
Z_7333925_T	37T	64T
Z_7333926_C	38C	68C
Z_7333927_A	37A	68A
Z_7333928_C	36C	71C
Z_7333929_T	36T	71T
Z_7333930_C	37C	72C
Z_7333931_T	37T	70T
Z_7333932_T	39T	70T
Z_7333933_T	39T	68T
Z_7333934_C	38C	66C
Z_7333935_C	39C	66C
Z_7333936_A	38A	65A
Z_7333937_A	37A	65A
Z_7333938_A	36A	63A
Z_7333939_T	36T	63T
Z_7333940_G	36G	62G
Z_7333941_G	33G	46A_86G
Z_7333942_G	33G	39G
Z_7333943_A	33A	39A
Z_7333944_A	35A	39A
Z_7333945_G	33G	60G
Z_7333946_G	33G	60G

Z_7333947_A	34A	59A
Z_7333948_A	34A	57A
Z_7333949_G	34G	57G
Z_7333950_G	33G	56G
Z_7333951_A	33A	56A
Z_7333952_A	33A	56A
Z_7333953_G	33G	55G
Z_7333954_G	32G	56G
Z_7333955_A	32A	56A
Z_7333956_A	33A	56A
Z_7333957_G	33G	57G
Z_7333958_G	33G	57G
Z_7333959_C	34C	56C
Z_7333960_C	33C	53C
Z_7333961_A	33A	53A
Z_7333962_A	32A	53A
Z_7333963_C	32C	52C
Z_7333964_C	30C	52C
Z_7333965_T	31T	52T
Z_7333966_A	31A	51A
Z_7333967_C	30C	50C
Z_7333968_A	29A	48A
Z_7333969_A	28A	48A
Z_7333970_A	27A	48A
Z_7333971_G	26G	48G
Z_7333972_C	26C	47C
Z_7333973_C	25C	47C
Z_7333974_C	25C	45C
Z_7333975_T	24T	44T
Z_7333976_T	24T	45T
Z_7333977_C	24C	42C
Z_7333978_C	24C	43C
Z_7333979_A	25A	44A
Z_7333980_G	26G	44G
Z_7333981_T	25T	44T
Z_7333982_C	26C	44C
Z_7333983_C	24C	43C
Z_7333984_C	26C	43C
Z_7333985_T	26T	42T
Z_7333986_C	27C	41C
Z_7333987_T	27T	40T
Z_7333988_G	28G	40G
Z_7333989_A	28A	40A

Z_7333990_G	25G	40G
Z_7333991_A	25A	38A
Z_7333992_A	23A	39A
Z_7333993_C	24C	39C
Z_7333994_A	23A	39A
Z_7333995_G	23G	38G
Z_7333996_G	21G	38G
Z_7333997_G	22G	38G
Z_7333998_A	22A	39A
Z_7333999_C	22C	38C
Z_7334000_C	23C	35C
Z_7334001_A	23A	35A
Z_7334002_G	21G	35G
Z_7334003_A	21A	36A
Z_7334004_A	20A	35A
Z_7334005_A	20A	35A
Z_7334006_G	20G	36G
Z_7334007_A	21A	38A
Z_7334008_G	19G	37G
Z_7334009_A	22A	36A
Z_7334010_A	21A	36A
Z_7334011_G	21G	33G
Z_7334012_G	21G	34G
Z_7334013_C	21C	33C
Z_7334014_G	18G	34G
Z_7334015_C	19C	34C
Z_7334016_T	17T	32T
Z_7334017_A	17A	32A
Z_7334018_A	17A	30A
Z_7334019_G	17G	29G
Z_7334020_C	17C	29C
Z_7334021_C	18C	28C
Z_7334022_A	18A	29A
Z_7334023_A	18A	31A
Z_7334024_G	19G	31G
Z_7334025_G	19G	30G
Z_7334026_A	17A	29A
Z_7334027_A	18A	29A
Z_7334028_C	16C	30C
Z_7334029_A	16A	29A
Z_7334030_G	15G	29G
Z_7334031_C	16C	29C
Z_7334032_C	17C	29C

Z_7334033_A	17A	29A
Z_7334034_G	15G_16C	29G_11C
Z_7334035_C	1A_3C	18C
Z_7334036_C	16C	28C
Z_7334037_C	16C	29C
Z_7334038_C	16C	29C
Z_7334039_C	16C	31C
Z_7334040_C	17C	31C
Z_7334041_C	17C	31C
Z_7334042_C	18C	32C
Z_7334043_A	18A	33A
Z_7334044_A	18A	33A
Z_7334045_T	18T	33T
Z_7334046_G	18G	33G
Z_7334047_C	17C	33C
Z_7334048_A	17A	33A
Z_7334049_G	18G	33G
Z_7334050_A	17A	33A
Z_7334051_A	17A	32A
Z_7334052_G	15G	31G
Z_7334053_A	16A	33A
Z_7334054_G	14G	33G
Z_7334055_C	15C	36C
Z_7334056_A	14A	36A
Z_7334057_G	14G	35G
Z_7334058_G	13G	37G
Z_7334059_G	13G	37G
Z_7334060_T	12T	36T
Z_7334061_T	12T	37T
Z_7334062_G	12G	37G
Z_7334063_T	13T	36T
Z_7334064_T	13T	37T
Z_7334065_G	13G	36G
Z_7334066_G	12G	37G
Z_7334067_C	12C	36C
Z_7334068_A	12A	35A
Z_7334069_C	13C	36C
Z_7334070_C	13C	36C
Z_7334071_C	13C	36C
Z_7334072_A	12A	35A
Z_7334073_C	12C	34C
Z_7334074_G	12G	14A_19G
Z_7334075_A	15A	19A_14G

Z_7334076_T	16T	35T
Z_7334077_A	16A	35A
Z_7334078_T	18T	36T
Z_7334079_C	18C	40C
Z_7334080_G	18G	44G
Z_7334081_G	17G	45G
Z_7334082_C	17C	45C
Z_7334083_C	17C	45C
Z_7334084_T	17T	45T
Z_7334085_G	17G	46G
Z_7334086_C	17C	46C
Z_7334087_A	17G	46A
Z_7334088_G	17G	47G
Z_7334089_C	17C	48C
Z_7334090_T	17T	48T
Z_7334091_T	17T	48T
Z_7334092_C	17C	49C
Z_7334093_T	17T	50T
Z_7334094_T	17T	51T
Z_7334095_C	17C	49C
Z_7334096_T	16T	48T
Z_7334097_G	16G	47G
Z_7334098_A	17A	44A
Z_7334099_G	17G	46G
Z_7334100_G	17G	46G
Z_7334101_G	16A	46G
Z_7334102_C	19C	44C
Z_7334103_G	19G	44G
Z_7334104_G	19G	43G
Z_7334105_A	20A	44A
Z_7334106_C	21C	45C
Z_7334107_G	20G	45G
Z_7334108_C	20C	45C
Z_7334109_C	21C	47C
Z_7334110_A	20A	47A
Z_7334111_A	20A	47A
Z_7334112_G	19G	47G
Z_7334113_G	20G	48G
Z_7334114_A	20A	48A
Z_7334115_T	19T	47T
Z_7334116_C	21C	49C
Z_7334117_C	21C	50C
Z_7334118_T	22T	50T

Z_7334119_G	21G	48G
Z_7334120_G	21G	46G
Z_7334121_G	21G	46G
Z_7334122_G	21G	46G
Z_7334123_C	21C	47C
Z_7334124_C	20C	48C
Z_7334125_A	21A	47A
Z_7334126_A	21A	48A
Z_7334127_C	21C	48C
Z_7334128_C	21C	48C
Z_7334129_G	20G	48G
Z_7334130_C	20C	49C
Z_7334131_C	20C	49C
Z_7334132_T	20T	48T
Z_7334133_T	18T	48T
Z_7334134_G	20G	46G
Z_7334135_G	19G	46G
Z_7334136_A	19A	45A
Z_7334137_G	21G	46G
Z_7334138_G	21G	45G
Z_7334139_C	20C	45C
Z_7334140_A	20A	44A
Z_7334141_G	21G	44G
Z_7334142_G	21G	44G
Z_7334143_G	21G	44G
Z_7334144_C	21C	13T_29C
Z_7334145_A	18A	29A_13G
Z_7334146_G	17G	42G
Z_7334147_C	18C	14T_29C
Z_7334148_C	16C	46C
Z_7334149_G	15G	45G
Z_7334150_T	14T	44T
Z_7334151_G	16G	44G
Z_7334152_A	16A	46A
Z_7334153_G	16G	47G
Z_7334154_G	16G	47G
Z_7334155_C	16C	46C
Z_7334156_A	17A	49A
Z_7334157_C	17C	49C
Z_7334158_T	17T	49T
Z_7334159_G	16G	50G
Z_7334160_C	18C	50C
Z_7334161_A	19A	49A

Z_7334162_C	20C	50C
Z_7334163_C	18C	51C
Z_7334164_C	19C	52C
Z_7334165_C	19C	53C
Z_7334166_C	19C	54C
Z_7334167_T	19T	52T
Z_7334168_T	18T	55T
Z_7334169_A	18A	55A
Z_7334170_C	17C	55C
Z_7334171_C	16C	54C
Z_7334172_C	16C	56C
Z_7334173_T	16T	54T
Z_7334174_G	18G	51G
Z_7334175_T	18T	48T
Z_7334176_G	16G	47G
Z_7334177_C	16C	46C
Z_7334178_C	17C	49C
Z_7334179_G	16G	48G
Z_7334180_T	16T	49T
Z_7334181_C	18C	52C
Z_7334182_T	18T	53T
Z_7334183_T	17T	52T
Z_7334184_G	17G	53G
Z_7334185_C	17C	52C
Z_7334186_C	17C	53C
Z_7334187_C	18C	52C
Z_7334188_C	17C	51C
Z_7334189_C	17C	50C
Z_7334190_T	14T_5C	48T
Z_7334191_C	19C	48C
Z_7334192_C	20C	50C
Z_7334193_A	20A	47A
Z_7334194_G	20G	50G
Z_7334195_G	18G	50G
Z_7334196_G	20G	49G
Z_7334197_C	20C	50C
Z_7334198_C	20C	50C
Z_7334199_C	20C	51C
Z_7334200_A	20A	49A
Z_7334201_A	20G	33A_17G
Z_7334202_T	20T	45T
Z_7334203_C	20C	47C
Z_7334204_C	21C	47C

Z_7334205_T	20T	45T
Z_7334206_C	20T	14T_31C
Z_7334207_A	20A	44A
Z_7334208_A	19A	46A
Z_7334209_T	19T	46T
Z_7334210_C	19C	46C
Z_7334211_C	19C	45C
Z_7334212_C	18C	44C
Z_7334213_A	18A	41A
Z_7334214_C	19C	43C
Z_7334215_C	19C	43C
Z_7334216_A	19A	43A
Z_7334217_C	19C	15A_27C
Z_7334218_C	18C	41C
Z_7334219_C	18C	41C
Z_7334220_A	18A	40A
Z_7334221_C	18T	39C
Z_7334222_G	18G	39G
Z_7334223_C	19C	39C
Z_7334224_C	19C	39C
Z_7334225_T	20T	36T
Z_7334226_G	20G	40G
Z_7334227_C	20C	42C
Z_7334228_C	20C	42C
Z_7334229_C	20C	42C
Z_7334230_C	20C	42C
Z_7334231_A	19A	41A
Z_7334232_G	20G	41G
Z_7334233_T	20T	41T
Z_7334234_C	20C	40C
Z_7334235_T	19T	40T
Z_7334236_C	21C	42C
Z_7334237_T	21T	42T
Z_7334238_C	20C	41C
Z_7334239_T	21T	40T
Z_7334240_A	22A	39A
Z_7334241_C	25C	41C
Z_7334242_T	25T	42T
Z_7334243_A	26A	39A
Z_7334244_C	25C	41C
Z_7334245_C	24C	41C
Z_7334246_C	22C	40C
Z_7334247_A	22A	39A

Z_7334248_G	22G	42G
Z_7334249_A	21A	41A
Z_7334250_A	23A	41A
Z_7334251_G	25G	40G
Z_7334252_G	24G	39G
Z_7334253_G	24G	38G
Z_7334254_A	23A	37A
Z_7334255_C	26C	35C
Z_7334256_G	25G	37G
Z_7334257_G	25G	36G
Z_7334258_G	25G	36G
Z_7334259_A	24A	34A
Z_7334260_G	23G	35G
Z_7334261_G	23G	37G
Z_7334262_G	22G	38G
Z_7334263_G	22G	36G
Z_7334264_G	22G	36G
Z_7334265_G	22G	36G
Z_7334266_G	23G	36G
Z_7334267_A	23A	34A
Z_7334268_G	22G	35G
Z_7334269_A	23A	35A
Z_7334270_G	23G	37G
Z_7334271_G	25G	37G
Z_7334272_G	26G	37G
Z_7334273_G	25G	38G
Z_7334274_C	25C	36C
Z_7334275_C	26C	35C
Z_7334276_C	26C	37C
Z_7334277_T	26T	37T
Z_7334278_T	27T	37T
Z_7334279_G	27G	36G
Z_7334280_T	28T	37T
Z_7334281_G	29G	38G
Z_7334282_C	30C	38C
Z_7334283_G	30G	37G
Z_7334284_C	29C	38C
Z_7334285_A	27A	39A
Z_7334286_A	30T	23A_16T
Z_7334287_G	30G	38G
Z_7334288_C	30C	38C
Z_7334289_G	30G	39G
Z_7334290_C	30C	39C

Z_7334291_A	30A	40A
Z_7334292_A	30A	39A
Z_7334293_C	30C	42C
Z_7334294_T	30T	41T
Z_7334295_C	29C	41C
Z_7334296_A	29A	46A
Z_7334297_A	29A	47A
Z_7334298_G	29G	47G
Z_7334299_T	29T	47T
Z_7334300_C	28C	47C
Z_7334301_C	29C	48C
Z_7334302_G	26G	49G
Z_7334303_C	26C	48C
Z_7334304_T	26T	48T
Z_7334305_T	28T	49T
Z_7334306_G	27G	49G
Z_7334307_G	27G	49G
Z_7334308_C	26C	51C
Z_7334309_A	28A	51A
Z_7334310_T	26T	51T
Z_7334311_G	24G	51G
Z_7334312_G	23G	51G
Z_7334313_A	23A	52A
Z_7334314_G	23G	51G
Z_7334315_C	23C	51C
Z_7334316_A	23A	54A
Z_7334317_T	24T	55T
Z_7334318_T	24T	55T
Z_7334319_T	25T	56T
Z_7334320_C	23C	55C
Z_7334321_G	21G	55G
Z_7334322_A	22A	52A
Z_7334323_T	22T	54T
Z_7334324_G	22G	55G
Z_7334325_A	20A	57A
Z_7334326_G	20G	59G
Z_7334327_C	20C	58C
Z_7334328_A	21A	59A
Z_7334329_A	21A	58A
Z_7334330_A	21A	59A
Z_7334331_T	21T	60T
Z_7334332_T	22T	60T
Z_7334333_G	22G	60G

Z_7334334_T	23T	58T
Z_7334335_T	23T	60T
Z_7334336_G	22G	60G
Z_7334337_C	23C	60C
Z_7334338_A	25A	61A
Z_7334339_A	25A	60A
Z_7334340_G	26G	61G
Z_7334341_G	23G	61G
Z_7334342_A	23A	61A
Z_7334343_A	23A	58A
Z_7334344_C	21C	59C
Z_7334345_C	22C	58C
Z_7334346_T	23T	59T
Z_7334347_C	23C	59C
Z_7334348_T	24T	59T
Z_7334349_C	26C	59C
Z_7334350_C	25C	62C
Z_7334351_G	25G	62G
Z_7334352_C	25C	62C
Z_7334353_T	24T	61T
Z_7334354_C	26C	62C
Z_7334355_A	27A	62A
Z_7334356_G	25G	62G
Z_7334357_G	25G	60G
Z_7334358_T	25T	61T
Z_7334359_G	24G	63G
Z_7334360_C	25C	65C
Z_7334361_T	28T	66T
Z_7334362_T	29T	65T
Z_7334363_G	29G	66G
Z_7334364_T	30T	63T
Z_7334365_G	30G	66G
Z_7334366_G	30G	65G
Z_7334367_T	29T	66T
Z_7334368_A	30A	66A
Z_7334369_C	30C	66C
Z_7334370_A	31A	65A
Z_7334371_G	31G	64G
Z_7334372_G	32G	63G
Z_7334373_T	32T	64T
Z_7334374_A	32A	64A
Z_7334375_C	31C	65C
Z_7334376_T	30T	65T

Z_7334377_C	30C	66C
Z_7334378_C	30C	66C
Z_7334379_T	30T	66T
Z_7334380_C	31C	65C
Z_7334381_G	32G	65G
Z_7334382_G	32G	66G
Z_7334383_C	33C	68C
Z_7334384_C	34C	66C
Z_7334385_T	34T	68T
Z_7334386_G	34G	67G
Z_7334387_C	36C	68C
Z_7334388_A	35A	65A
Z_7334389_T	35T	67T
Z_7334390_G	35G	68G
Z_7334391_C	37C	64C
Z_7334392_T	35T	63T
Z_7334393_G	35G	64G
Z_7334394_A	33A	64A
Z_7334395_G	35G	64G
Z_7334396_G	35G	61G
Z_7334397_G	38G	62G
Z_7334398_A	38A	62A
Z_7334399_C	38C	63C
Z_7334400_C	41C	63C
Z_7334401_G	41G	64G
Z_7334402_G	41G	63G
Z_7334403_A	41A	62A
Z_7334404_T	41T	62T
Z_7334405_G	41G	64G
Z_7334406_T	39T	65T
Z_7334407_C	42C	65C
Z_7334408_G	41G	64G
Z_7334409_G	42G	66G
Z_7334410_C	41C	68C
Z_7334411_C	44C	65C
Z_7334412_A	44A	65A
Z_7334413_G	44G	66G
Z_7334414_C	45C	65C
Z_7334415_C	44C	66C
Z_7334416_G	44G	66G
Z_7334417_G	44G	68G
Z_7334418_A	43A	67A
Z_7334419_G	41G	66G

Z_7334420_G	41G	63G
Z_7334421_A	42A	62A
Z_7334422_G	41G	62G
Z_7334423_C	41C	61C
Z_7334424_A	42A	61A
Z_7334425_G	42G	62G
Z_7334426_C	42C	61C
Z_7334427_T	42T	62T
Z_7334428_G	41G	63G
Z_7334429_C	41C	64C
Z_7334430_C	42C	64C
Z_7334431_G	41G	63G
Z_7334432_G	39G	66G
Z_7334433_A	40A	66A
Z_7334434_A	41A	66A
Z_7334435_T	41T	67T
Z_7334436_T	43T	67T
Z_7334437_T	43T	67T
Z_7334438_G	43G	68G
Z_7334439_T	43T	67T
Z_7334440_C	41C	65C
Z_7334441_C	42C	65C
Z_7334442_G	41G	65G
Z_7334443_T	43T	68T
Z_7334444_G	41G	68G
Z_7334445_G	42G	68G
Z_7334446_T	43T	67T
Z_7334447_G	43G	68G
Z_7334448_T	43T	69T
Z_7334449_G	40G	68G
Z_7334450_A	41A	70A
Z_7334451_G	39G	68G
Z_7334452_G	40G	68G
Z_7334453_A	39A	68A
Z_7334454_T	40T	67T
Z_7334455_A	40A	66A
Z_7334456_G	39G	65G
Z_7334457_T	38T	66T
Z_7334458_G	39G	66G
Z_7334459_G	39G	68G
Z_7334460_C	38C	66C
Z_7334461_A	39A	66A
Z_7334462_G	38G	66G

Z_7334463_A	40A	66A
Z_7334464_T	40T	69T
Z_7334465_C	40C	69C
Z_7334466_G	43G	69G
Z_7334467_T	41T	67T
Z_7334468_G	40G	70G
Z_7334469_T	41T	69T
Z_7334470_A	39A	68A
Z_7334471_C	39C	69C
Z_7334472_T	38T	68T
Z_7334473_C	37C	69C
Z_7334474_C	37C	69C
Z_7334475_A	37A	69A
Z_7334476_G	38G	68G
Z_7334477_C	38C	68C
Z_7334478_A	38A	67A
Z_7334479_G	38G	67G
Z_7334480_G	38G	67G
Z_7334481_G	34G	67G
Z_7334482_C	34C	66C
Z_7334483_G	35G	65G
Z_7334484_G	33G	64G
Z_7334485_C	34C	63C
Z_7334486_G	34A	62G
Z_7334487_T	34T	62T
Z_7334488_T	35T	62T
Z_7334489_G	35G	64G
Z_7334490_G	35G	65G
Z_7334491_C	35C	66C
Z_7334492_T	38T	70T
Z_7334493_T	40T	70T
Z_7334494_T	38T	70T
Z_7334495_C	37C	69C
Z_7334496_T	39T	68T
Z_7334497_C	38C	69C
Z_7334498_C	41C	69C
Z_7334499_T	42T	70T
Z_7334500_G	40G	68G
Z_7334501_G	40G	69G
Z_7334502_G	42G	69G
Z_7334503_C	39C	70C
Z_7334504_G	40G	69G
Z_7334505_T	40T	67T

Z_7334506_C	38C	66C
Z_7334507_T	39T	66T
Z_7334508_T	39T	68T
Z_7334509_T	40T	70T
Z_7334510_C	40C	71C
Z_7334511_A	40A	71A
Z_7334512_C	40C	69C
Z_7334513_G	39G	72G
Z_7334514_A	40A	72A
Z_7334515_G	41G	73G
Z_7334516_G	42G	74G
Z_7334517_C	44C	74C
Z_7334518_T	44T	74T
Z_7334519_C	44C	75C
Z_7334520_G	44G	73G
Z_7334521_A	43A	73A
Z_7334522_G	44G	73G
Z_7334523_T	45T	73T
Z_7334524_T	43T	74T
Z_7334525_C	43C	76C
Z_7334526_T	43T	77T
Z_7334527_C	42C	75C
Z_7334528_C	41C	75C
Z_7334529_A	41A	79A
Z_7334530_C	42C	79C
Z_7334531_G	41G	23A_58G
Z_7334532_T	41T	81T
Z_7334533_A	41A	80A
Z_7334534_T	41T	80T
Z_7334535_T	41T	80T
Z_7334536_T	38T	81T
Z_7334537_G	38G	82G
Z_7334538_A	38A	79A
Z_7334539_C	37C	79C
Z_7334540_A	35A	76A
Z_7334541_T	36T	79T
Z_7334542_C	36C	78C
Z_7334543_T	36T	78T
Z_7334544_G	36G	80G
Z_7334545_C	36T	79C
Z_7334546_A	35A	79A
Z_7334547_A	34A	79A
Z_7334548_C	33C	80C

Z_7334549_G	31G	79G
Z_7334550_A	32A	79A
Z_7334551_A	32A	81A
Z_7334552_A	32A	82A
Z_7334553_T	44A_22T_44G_3C	83T
Z_7334554_A	3A	82A
Z_7334555_A	3A	80A
Z_7334556_G	2G	81G
Z_7334557_G	1G	82G
Z_7334558_A	22C	83A
Z_7334559_A	29A	80A
Z_7334560_A	29A	83A
Z_7334561_G	31G	81G
Z_7334562_G	33G	83G
Z_7334563_G	34G	81G
Z_7334564_A	35A	81A
Z_7334565_G	36G	80G
Z_7334566_A	36A	78A
Z_7334567_G	37G	79G
Z_7334568_G	38G	79G
Z_7334569_C	39C	80C
Z_7334570_G	39G	81G
Z_7334571_G	37G	82G
Z_7334572_A	37A	82A
Z_7334573_G	34G	82G
Z_7334574_T	35T	82T
Z_7334575_G	36G	82G
Z_7334576_A	35A	82A
Z_7334577_G	36G	83G
Z_7334578_G	36G	83G
Z_7334579_C	36C	83C
Z_7334580_C	35C	83C
Z_7334581_T	39T	83T
Z_7334582_G	38G	83G
Z_7334583_A	37A	80A
Z_7334584_C	37C	80C
Z_7334585_A	37A	80A
Z_7334586_G	37G	79G
Z_7334587_G	38G	75G
Z_7334588_C	37C	75C
Z_7334589_G	36G	74G
Z_7334590_A	35A	74A
Z_7334591_G	35G	76G

Z_7334592_A	33A	75A
Z_7334593_G	34G	77G
Z_7334594_G	35G	76G
Z_7334595_C	35C	75C
Z_7334596_G	36G	74G
Z_7334597_G	35G	74G
Z_7334598_G	34G	74G
Z_7334599_C	36T	73C
Z_7334600_G	37G	25A_49G
Z_7334601_G	37G	75G
Z_7334602_T	37T	76T
Z_7334603_T	38T	74T
Z_7334604_T	37T	72T
Z_7334605_C	36C	69C
Z_7334606_C	37C	68C
Z_7334607_T	37T	67T
Z_7334608_G	36G	66G
Z_7334609_T	36T	66T
Z_7334610_A	36A	65A
Z_7334611_C	37C	23T_40C
Z_7334612_G	38G	65G
Z_7334613_C	38C	66C
Z_7334614_C	37C	66C
Z_7334615_A	37A	65A
Z_7334616_T	36T	65T
Z_7334617_C	35C	65C
Z_7334618_C	33C	64C
Z_7334619_A	33A	63A
Z_7334620_G	33G	62G
Z_7334621_G	34G	61G
Z_7334622_T	34T	60T
Z_7334623_G	34G	59G
Z_7334624_G	32G	56G
Z_7334625_T	32T	55T
Z_7334626_T	31T	53T
Z_7334627_T	31T	53T
Z_7334628_T	31T	54T
Z_7334629_T	30T	54T
Z_7334630_A	30A	55A
Z_7334631_A	29A	53A
Z_7334632_T	27T	52T
Z_7334633_T	26T	52T
Z_7334634_C	24C	54C

Z_7334635_T	24T	54T
Z_7334636_A	24A	52A
Z_7334637_A	23A	52A
Z_7334638_G	22G	50G
Z_7334639_A	21A	50A
Z_7334640_A	20A	51A
Z_7334641_C	null	25C
Z_7334642_G	null	27G
Z_7334643_A	null	27A
Z_7334644_A	null	26A
Z_7334645_A	null	25A
Z_7334646_A	null	25A
Z_7334647_A	null	24A
Z_7334648_A	null	23A
Z_7334649_A	null	23A
Z_7334650_G	null	23G
Z_7334651_A	null	23A
Z_7334652_G	null	22G
Z_7334653_C	null	21C
Z_7334654_A	null	21A
Z_7334655_T	null	21T
Z_7334656_C	null	21C
Z_7334657_T	null	20T
Z_7334658_T	null	20T
Z_7334659_T	null	21T
Z_7334660_T	null	20T
Z_7334661_C	null	20C
Z_7334662_C	null	20C
Z_7334663_T	null	20T
Z_7334664_G	null	21G
Z_7334665_A	null	21A
Z_7334666_G	null	22G
Z_7334667_G	3A	22G
Z_7334668_A	18A	22A
Z_7334669_G	18G	22G
Z_7334670_G	20G	22G
Z_7334671_G	20G	22G
Z_7334672_G	21G	22G
Z_7334673_A	21A	22A
Z_7334674_A	21A	22A
Z_7334675_A	21A	22A
Z_7334676_A	21A	22A
Z_7334677_A	21A	22A

Z_7334678_A	21A	22A
Z_7334679_G	21G	23G
Z_7334680_G	21G	23G
Z_7334681_G	21G	23G
Z_7334682_G	21G	24G
Z_7334683_A	21A	24A
Z_7334684_G	21G	24G
Z_7334685_T	21T	23T
Z_7334686_G	21G	22G
Z_7334687_T	21T	22T
Z_7334688_G	21G	22G
Z_7334689_G	21G	22G
Z_7334690_A	21A	22A
Z_7334691_A	21A	22A
Z_7334692_C	21C	23C
Z_7334693_C	1C	23C
Z_7334694_A	null	23A
Z_7334695_G	null	24G
Z_7334696_T	null	24T
Z_7334697_A	null	23A
Z_7334698_T	null	23T
Z_7334699_A	null	23A
Z_7334700_G	null	23G
Z_7334701_A	null	23A
Z_7334702_C	null	24C
Z_7334703_C	null	24C
Z_7334704_A	1A	24A
Z_7334705_G	1G	24G
Z_7334706_A	1A	24A
Z_7334707_C	1C	22C
Z_7334708_G	1G	21G
Z_7334709_G	1G	21G
Z_7334710_G	1G	21G
Z_7334711_C	1C	21C
Z_7334712_G	1G	21G
Z_7334713_G	1G	22G
Z_7334714_G	1G	21G
Z_7334715_A	1A	20A
Z_7334716_T	1T	20T
Z_7334717_A	1A	20A
Z_7334718_C	1C	20C
Z_7334719_A	2A	19A
Z_7334720_A	2A	19A

Z_7334721_A	2A	19A
Z_7334722_T	2T	20T
Z_7334723_C	2C	19C
Z_7334724_A	2A	19A
Z_7334725_A	2A	18A
Z_7334726_A	2A	18A
Z_7334727_T	2T	18T
Z_7334728_A	2A	18A
Z_7334729_A	2A	16A
Z_7334730_A	2A	19A
Z_7334731_T	2T	17T
Z_7334732_A	2A	17A
Z_7334733_A	2A	17A
Z_7334734_A	2A	16A
Z_7334735_T	2T	16T
Z_7334736_A	2A	16A
Z_7334737_A	2A	15A
Z_7334738_A	2A	15A
Z_7334739_T	2T	15T
Z_7334740_A	2A	15A
Z_7334741_A	2A	15A
Z_7334742_A	2A	15A
Z_7334743_T	2T	15T
Z_7334744_A	2A	16A
Z_7334745_A	2A	16A
Z_7334746_A	2A	15A
Z_7334747_T	2T	15T
Z_7334748_A	2A	16A
Z_7334749_A	2A	16A
Z_7334750_A	1A	16A
Z_7334751_T	1T	17T
Z_7334752_A	1A	18A
Z_7334753_A	1A	19A
Z_7334754_A	1A	18A
Z_7334755_T	1T	18T
Z_7334756_A	1A	19A
Z_7334757_A	1A	19A
Z_7334758_A	1A	19A
Z_7334759_T	1T	19T
Z_7334760_A	1A	19A
Z_7334761_A	1A	18A
Z_7334762_A	1A	18A
Z_7334763_A	1A	18A

Z_7334764_A	1A	19A
Z_7334765_T	1T	22T
Z_7334766_A	1A	22A
Z_7334767_A	null	22A
Z_7334768_A	null	23A
Z_7334769_T	null	24T
Z_7334770_A	1A	25A
Z_7334771_A	1C	24A
Z_7334772_A	1A	25A
Z_7334773_A	1A	27A
Z_7334774_C	1C	28C
Z_7334775_C	2T	27C
Z_7334776_G	14G	59G
Z_7334777_C	15C	60C
Z_7334778_A	16A	60A
Z_7334779_A	17A	60A
Z_7334780_A	17A	61A
Z_7334781_C	17T	33T_30C
Z_7334782_A	18A	64A
Z_7334783_G	18G	64G
Z_7334784_T	18T	65T
Z_7334785_G	18G	66G
Z_7334786_C	19C	66C
Z_7334787_T	19T	65T
Z_7334788_G	20G	67G
Z_7334789_T	21T	67T
Z_7334790_T	21T	67T
Z_7334791_C	20C	67C
Z_7334792_T	21T	66T
Z_7334793_C	22C	67C
Z_7334794_C	22T	68C
Z_7334795_G	22G	69G
Z_7334796_G	23G	71G
Z_7334797_A	23A	70A
Z_7334798_T	24T	70T
Z_7334799_A	25A	72A
Z_7334800_A	26A	72A
Z_7334801_A	27A	71A
Z_7334802_T	28T	73T
Z_7334803_G	29G	74G
Z_7334804_T	29T	74T
Z_7334805_A	30A	74A
Z_7334806_A	32A	76A

Z_7334807_A	32A	77A
Z_7334808_A	32A	78A
Z_7334809_A	33G	36A_43G
Z_7334810_T	34T	78T
Z_7334811_T	33T	78T
Z_7334812_C	34C	78C
Z_7334813_A	34A	80A
Z_7334814_A	35A	80A
Z_7334815_G	35G	80G
Z_7334816_A	38A	79A
Z_7334817_C	41C	78C
Z_7334818_A	41A	80A
Z_7334819_C	40C	80C
Z_7334820_A	42A	80A
Z_7334821_G	42G	82G
Z_7334822_G	46G	82G
Z_7334823_A	46A	82A
Z_7334824_A	46A	84A
Z_7334825_G	45G	82G
Z_7334826_C	44C	81C
Z_7334827_C	43C	81C
Z_7334828_T	44T	78T
Z_7334829_C	45C	82C
Z_7334830_C	44C	82C
Z_7334831_T	44T	81T
Z_7334832_A	44A	83A
Z_7334833_T	43T	84T
Z_7334834_T	44T	84T
Z_7334835_G	44G	85G
Z_7334836_A	44A	87A
Z_7334837_C	45C	87C
Z_7334838_T	45T	86T
Z_7334839_G	45G	85G
Z_7334840_T	44T	84T
Z_7334841_G	42G	84G
Z_7334842_G	42G	84G
Z_7334843_A	42A	83A
Z_7334844_G	43G	85G
Z_7334845_A	43A	86A
Z_7334846_T	43T	86T
Z_7334847_G	42G	87G
Z_7334848_C	41C	86C
Z_7334849_A	40A	87A

Z_7334850_G	40G	88G
Z_7334851_A	41A	85A
Z_7334852_A	40A	86A
Z_7334853_G	39G	87G
Z_7334854_G	40G	86G
Z_7334855_A	39A	87A
Z_7334856_C	38C	86C
Z_7334857_C	39C	85C
Z_7334858_C	40C	85C
Z_7334859_T	41T	84T
Z_7334860_T	42T	79T
Z_7334861_T	42T	81T
Z_7334862_G	43G	81G
Z_7334863_C	42C	78C
Z_7334864_C	40C	74C
Z_7334865_A	44A	73A
Z_7334866_C	42C	71C
Z_7334867_T	45T	71T
Z_7334868_G	44G	69G
Z_7334869_G	42G	67G
Z_7334870_C	43C	66C
Z_7334871_A	43A	67A
Z_7334872_G	43G	68G
Z_7334873_A	43A	68A
Z_7334874_T	44T	69T
Z_7334875_T	43T	70T
Z_7334876_A	41A	71A
Z_7334877_G	40G	70G
Z_7334878_C	39C	71C
Z_7334879_T	39T	70T
Z_7334880_G	39G	69G
Z_7334881_G	38G	69G
Z_7334882_T	38T	70T
Z_7334883_G	37G	69G
Z_7334884_A	39A	70A
Z_7334885_T	37T	71T
Z_7334886_G	35G	71G
Z_7334887_C	34C	71C
Z_7334888_C	34C	71C
Z_7334889_A	35A	71A
Z_7334890_C	36C	71C
Z_7334891_G	36G	73G
Z_7334892_G	32G	74G

Z_7334893_A	32A	74A
Z_7334894_A	32A	74A
Z_7334895_T	33T	74T
Z_7334896_G	34G	74G
Z_7334897_T	37T	73T
Z_7334898_T	36T	72T
Z_7334899_C	35C	73C
Z_7334900_A	36A	73A
Z_7334901_C	37C	72C
Z_7334902_G	37G	22A_49G
Z_7334903_A	38A	71A
Z_7334904_A	37A	71A
Z_7334905_C	37C	72C
Z_7334906_A	37A	72A
Z_7334907_T	35T	73T
Z_7334908_T	36T	71T
Z_7334909_T	35T	72T
Z_7334910_T	35T	72T
Z_7334911_A	35A	73A
Z_7334912_A	35A	72A
Z_7334913_T	36T	70T
Z_7334914_T	35T	70T
Z_7334915_T	36T	70T
Z_7334916_C	34C	68C
Z_7334917_C	35C	68C
Z_7334918_A	36A	65A
Z_7334919_A	36A	67A
Z_7334920_T	36T	66T
Z_7334921_G	37G	67G
Z_7334922_A	37A	69A
Z_7334923_G	36G	72G
Z_7334924_G	38G	70G
Z_7334925_T	38T	73T
Z_7334926_G	38G	74G
Z_7334927_G	37G	73G
Z_7334928_G	36G	75G
Z_7334929_G	35G	75G
Z_7334930_G	34G	74G
Z_7334931_A	35A	72A
Z_7334932_G	35G	71G
Z_7334933_A	38A	71A
Z_7334934_A	39A	71A
Z_7334935_A	37A	71A

Z_7334936_T	37T	71T
Z_7334937_C	37C	73C
Z_7334938_C	36C	74C
Z_7334939_T	37C	49T_24C
Z_7334940_T	39T	73T
Z_7334941_C	38C	72C
Z_7334942_C	36C	71C
Z_7334943_T	36T	70T
Z_7334944_C	36C	70C
Z_7334945_C	36C	68C
Z_7334946_T	37T	68T
Z_7334947_A	37A	67A
Z_7334948_A	40A	66A
Z_7334949_G	40G	66G
Z_7334950_C	40C	67C
Z_7334951_A	41A	71A
Z_7334952_G	42G	71G
Z_7334953_G	44G	74G
Z_7334954_A	43A	74A
Z_7334955_A	45A	74A
Z_7334956_A	46A	72A
Z_7334957_A	43A	74A
Z_7334958_A	44A	73A
Z_7334959_G	44G	71G
Z_7334960_G	42G	75G
Z_7334961_G	46G	76G
Z_7334962_G	45G	75G
Z_7334963_T	44T	75T
Z_7334964_T	45T	75T
Z_7334965_A	44A	75A
Z_7334966_G	44G	76G
Z_7334967_C	43C	33G_42C
Z_7334968_A	44A	77A
Z_7334969_C	44C	76C
Z_7334970_G	44G	76G
Z_7334971_G	44G	74G
Z_7334972_A	44A	76A
Z_7334973_A	42A	75A
Z_7334974_T	43T	74T
Z_7334975_A	42A	75A
Z_7334976_T	42T	75T
Z_7334977_T	43T	77T
Z_7334978_C	44C	75C

Z_7334979_A	45A	75A
Z_7334980_C	46C	78C
Z_7334981_A	46A	76A
Z_7334982_A	48A	76A
Z_7334983_A	47A	76A
Z_7334984_C	47C	75C
Z_7334985_T	46T	77T
Z_7334986_G	45G	76G
Z_7334987_T	46T	75T
Z_7334988_C	45C	77C
Z_7334989_C	45C	77C
Z_7334990_T	45T	77T
Z_7334991_G	44G	77G
Z_7334992_T	44T	79T
Z_7334993_T	45T	80T
Z_7334994_T	44T	82T
Z_7334995_T	45T	83T
Z_7334996_C	46C	82C
Z_7334997_T	46T	81T
Z_7334998_A	45A	80A
Z_7334999_A	45A	82A
Z_7335000_A	44A	81A
Z_7335001_G	44G	84G
Z_7335002_A	42A	85A
Z_7335003_A	39A	84A
Z_7335004_G	38G	85G
Z_7335005_G	39G	42G_43C
Z_7335006_C	40C	87C
Z_7335007_A	41A	87A
Z_7335008_A	42A	88A
Z_7335009_G	42G	88G
Z_7335010_T	42T	86T
Z_7335011_T	44T	86T
Z_7335012_A	44A	88A
Z_7335013_G	31A_43G_1C	65A_88G
Z_7335014_A	49A	91A
Z_7335015_A	48A	91A
Z_7335016_A	47A	92A
Z_7335017_A	49A	91A
Z_7335018_A	46A	91A
Z_7335019_A	46A	92A
Z_7335020_A	45A	89A
Z_7335021_A	44A	89A

Z_7335022_T	40T	89T
Z_7335023_C	39C	87C
Z_7335024_C	40C	87C
Z_7335025_T	39T	89T
Z_7335026_C	38C	90C
Z_7335027_A	38A	90A
Z_7335028_A	38A	92A
Z_7335029_G	38G	92G
Z_7335030_T	36T	88T
Z_7335031_G	36G	88G
Z_7335032_A	36A	86A
Z_7335033_C	36C	86C
Z_7335034_A	24A	80A
Z_7335035_G	25G	80G
Z_7335036_G	24G	80G
Z_7335037_A	24C	50A_30C
Z_7335038_G	24A	30A_49G
Z_7335039_A	25A	80A
Z_7335040_G	25G	83G
Z_7335041_C	25A	32A_51C
Z_7335042_G	36G	92G
Z_7335043_A	36A	94A
Z_7335044_G	36G	94G
Z_7335045_A	39A	94A
Z_7335046_A	39A	93A
Z_7335047_C	40C	92C
Z_7335048_A	41A	93A
Z_7335049_A	43A	92A
Z_7335050_G	44G	93G
Z_7335051_A	45A	94A
Z_7335052_C	46C	94C
Z_7335053_C	48C	95C
Z_7335054_C	49C	94C
Z_7335055_A	49A	93A
Z_7335056_T	49T	94T
Z_7335057_T	49T	94T
Z_7335058_A	50A	94A
Z_7335059_G	49G	94G
Z_7335060_C	50C	96C
Z_7335061_T	52T	93T
Z_7335062_G	51G	92G
Z_7335063_C	51C	91C
Z_7335064_A	50A	92A

Z_7335065_A	49A	92A
Z_7335066_A	49A	91A
Z_7335067_A	49A	91A
Z_7335068_C	48C	91C
Z_7335069_T	50T	92T
Z_7335070_C	51C	92C
Z_7335071_A	51A	95A
Z_7335072_T	52T	95T
Z_7335073_G	54G	95G
Z_7335074_T	54T	94T
Z_7335075_A	54A	93A
Z_7335076_G	54G	95G
Z_7335077_C	53C	94C
Z_7335078_C	53C	93C
Z_7335079_A	52A	93A
Z_7335080_T	51T	93T
Z_7335081_T	51T	90T
Z_7335082_G	50G	90G
Z_7335083_G	49G	90G
Z_7335084_T	49T	90T
Z_7335085_T	49T	91T
Z_7335086_G	48G	90G
Z_7335087_A	48A	88A
Z_7335088_T	47T	87T
Z_7335089_G	47G	85G
Z_7335090_G	46G	85G
Z_7335091_G	47G	85G
Z_7335092_C	48C	85C
Z_7335093_T	47T	41T_43G
Z_7335094_G	48G	83G
Z_7335095_T	48T	83T
Z_7335096_T	47T	82T
Z_7335097_A	46A	82A
Z_7335098_A	46A	84A
Z_7335099_T	47T	85T
Z_7335100_T	47T	84T
Z_7335101_C	48C	82C
Z_7335102_C	49C	81C
Z_7335103_A	49A	83A
Z_7335104_C	47C	83C
Z_7335105_T	47T	83T
Z_7335106_G	46G	83G
Z_7335107_A	48A	82A

Z_7335108_A	49A	81A
Z_7335109_A	49A	81A
Z_7335110_C	49C	81C
Z_7335111_A	50A	80A
Z_7335112_T	46T	78T
Z_7335113_C	48C	77C
Z_7335114_T	48T	74T
Z_7335115_C	45C	73C
Z_7335116_G	45G	73G
Z_7335117_T	45T	70T
Z_7335118_T	43T	70T
Z_7335119_T	42T	70T
Z_7335120_C	41C	68C
Z_7335121_T	40T	68T
Z_7335122_C	39C	66C
Z_7335123_A	38A	66A
Z_7335124_A	38A	66A
Z_7335125_G	36G	66G
Z_7335126_A	39A	68A
Z_7335127_G	42G	67G
Z_7335128_A	42A	69A
Z_7335129_A	43A	70A
Z_7335130_A	42A	70A
Z_7335131_G	41G	72G
Z_7335132_T	40T	72T
Z_7335133_G	42G	71G
Z_7335134_G	43G	72G
Z_7335135_G	43G	70G
Z_7335136_A	43A	70A
Z_7335137_A	43A	70A
Z_7335138_C	41A	30A_38C
Z_7335139_G	41G	69G
Z_7335140_T	44T	70T
Z_7335141_G	44G	70G
Z_7335142_T	42T	68T
Z_7335143_C	42C	69C
Z_7335144_A	42A	72A
Z_7335145_G	42G	72G
Z_7335146_A	40A	71A
Z_7335147_A	40A	71A
Z_7335148_C	39C	70C
Z_7335149_A	39A	69A
Z_7335150_T	38T	68T

Z_7335151_C	37C	67C
Z_7335152_A	36A	69A
Z_7335153_A	37A	68A
Z_7335154_C	36C	69C
Z_7335155_T	34C	38T_29C
Z_7335156_G	34G	68G
Z_7335157_A	35A	71A
Z_7335158_T	38T	71T
Z_7335159_A	41A	70A
Z_7335160_G	42G	69G
Z_7335161_C	44C	67C
Z_7335162_T	43T	68T
Z_7335163_C	41C	69C
Z_7335164_T	42T	68T
Z_7335165_T	43T	67T
Z_7335166_T	47T	64T
Z_7335167_C	47C	64C
Z_7335168_T	49T	65T
Z_7335169_C	46C	65C
Z_7335170_T	47T	65T
Z_7335171_T	46T	64T
Z_7335172_G	45G	64G
Z_7335173_G	46G	64G
Z_7335174_A	45A	64A
Z_7335175_T	46T	65T
Z_7335176_G	46G	66G
Z_7335177_A	47A	65A
Z_7335178_A	48A	63A
Z_7335179_T	47T	62T
Z_7335180_T	47T	61T
Z_7335181_A	47A	61A
Z_7335182_A	47A	61A
Z_7335183_A	46A	61A
Z_7335184_A	46T	37A_30T
Z_7335185_A	46A	66A
Z_7335186_G	45G	68G
Z_7335187_A	45A	68A
Z_7335188_T	46T	65T
Z_7335189_C	46C	69C
Z_7335190_A	46A	72A
Z_7335191_A	40C	70A
Z_7335192_G	40A	69G
Z_7335193_A	42A	66A

Z_7335194_C	42C	67C
Z_7335195_A	43A	67A
Z_7335196_A	44A	67A
Z_7335197_A	44A	67A
Z_7335198_G	43G	65G
Z_7335199_C	43C	65C
Z_7335200_A	44A	65A
Z_7335201_C	45C	65C
Z_7335202_A	46A	65A
Z_7335203_C	44C	64C
Z_7335204_A	44A	64A
Z_7335205_C	43C	62C
Z_7335206_A	44A	63A
Z_7335207_A	44A	64A
Z_7335208_A	45A	64A
Z_7335209_C	45C	64C
Z_7335210_C	42C	64C
Z_7335211_A	42A	66A
Z_7335212_C	41C	66C
Z_7335213_T	41T	66T
Z_7335214_G	42G	66G
Z_7335215_A	40A	65A
Z_7335216_T	42T	65T
Z_7335217_T	41T	64T
Z_7335218_T	44T	64T
Z_7335219_T	45G	62T
Z_7335220_G	46G	64G
Z_7335221_A	46A	62A
Z_7335222_G	45G	61G
Z_7335223_G	44G	60G
Z_7335224_G	45G	59G
Z_7335225_G	44G	58G
Z_7335226_G	45G	61G
Z_7335227_T	44T	62T
Z_7335228_G	42G	59G
Z_7335229_C	39C	58C
Z_7335230_A	38A	56A
Z_7335231_C	37C	56C
Z_7335232_A	38A	58A
Z_7335233_A	39A	59A
Z_7335234_A	41A	58A
Z_7335235_T	41T	35T_25C
Z_7335236_A	38A	60A

Z_7335237_C	36C	61C
Z_7335238_A	36A	60A
Z_7335239_A	38A	57A
Z_7335240_C	39C	56C
Z_7335241_T	39T	58T
Z_7335242_G	40G	58G
Z_7335243_T	40T	59T
Z_7335244_T	40T	59T
Z_7335245_C	40C	59C
Z_7335246_C	41C	59C
Z_7335247_T	39T	59T
Z_7335248_G	37G	60G
Z_7335249_C	37C	61C
Z_7335250_T	38T	61T
Z_7335251_G	36G	62G
Z_7335252_C	40C	58C
Z_7335253_T	39T	58T
Z_7335254_T	39T	60T
Z_7335255_T	39T	60T
Z_7335256_C	39C	60C
Z_7335257_A	38A	60A
Z_7335258_T	39T	59T
Z_7335259_A	39A	59A
Z_7335260_T	39T	59T
Z_7335261_C	42C	59C
Z_7335262_C	42C	60C
Z_7335263_A	46A	58A
Z_7335264_T	46T	58T
Z_7335265_T	45T	58T
Z_7335266_G	44G	58G
Z_7335267_G	44G	59G
Z_7335268_C	42C	59C
Z_7335269_T	44T	59T
Z_7335270_G	43G	59G
Z_7335271_C	44C	58C
Z_7335272_T	43T	58T
Z_7335273_C	43C	59C
Z_7335274_A	44A	59A
Z_7335275_G	45G	60G
Z_7335276_C	45C	61C
Z_7335277_T	44T	62T
Z_7335278_G	43G	62G
Z_7335279_C	43C	58C

Z_7335280_T	45T	59T
Z_7335281_G	46G	57G
Z_7335282_A	45A	57A
Z_7335283_T	47T	57T
Z_7335284_G	47G	55G
Z_7335285_C	47C	52C
Z_7335286_C	47C	52C
Z_7335287_A	49A	52A
Z_7335288_C	47C	52C
Z_7335289_G	47G	53G
Z_7335290_G	46G	53G
Z_7335291_C	46C	54C
Z_7335292_A	47A	54A
Z_7335293_T	46T	54T
Z_7335294_G	48G	55G
Z_7335295_T	48T	56T
Z_7335296_T	50T	56T
Z_7335297_C	49C	56C
Z_7335298_A	53A	56A
Z_7335299_T	55T	58T
Z_7335300_A	58A	60A
Z_7335301_A	58A	58A
Z_7335302_A	57A	58A
Z_7335303_C	55C	59C
Z_7335304_A	53A	59A
Z_7335305_T	52T	59T
Z_7335306_T	50T	58T
Z_7335307_C	53C	59C
Z_7335308_A	53A	59A
Z_7335309_C	52C	59C
Z_7335310_A	51A	61A
Z_7335311_T	52T	61T
Z_7335312_T	51T	62T
Z_7335313_C	50C	62C
Z_7335314_C	47C	63C
Z_7335315_C	49C	68C
Z_7335316_A	49A	69A
Z_7335317_A	48A	70A
Z_7335318_C	51C	71C
Z_7335319_A	51A	71A
Z_7335320_G	52G	71G
Z_7335321_C	54C	68C
Z_7335322_T	51T	66T

Z_7335323_T	52T	69T
Z_7335324_G	52G	69G
Z_7335325_G	53G	69G
Z_7335326_C	54C	70C
Z_7335327_A	56A	69A
Z_7335328_G	54G	70G
Z_7335329_A	55A	70A
Z_7335330_A	54A	69A
Z_7335331_T	52T	69T
Z_7335332_G	51G	68G
Z_7335333_G	47G	67G
Z_7335334_G	48G	69G
Z_7335335_A	48A	69A
Z_7335336_G	47G	67G
Z_7335337_A	47A	68A
Z_7335338_A	48A	68A
Z_7335339_A	48A	68A
Z_7335340_G	47G	69G
Z_7335341_A	46A	70A
Z_7335342_A	46A	70A
Z_7335343_G	46G	68G
Z_7335344_G	47G	67G
Z_7335345_A	46A	67A
Z_7335346_G	46G	68G
Z_7335347_C	47C	69C
Z_7335348_A	47A	70A
Z_7335349_C	47C	69C
Z_7335350_A	48A	70A
Z_7335351_A	48A	71A
Z_7335352_A	48A	70A
Z_7335353_A	47A	70A
Z_7335354_A	46A	71A
Z_7335355_A	47A	71A
Z_7335356_A	45A	73A
Z_7335357_A	43A	70A
Z_7335358_G	43G	72G
Z_7335359_C	42C	72C
Z_7335360_C	42C	73C
Z_7335361_A	42A	72A
Z_7335362_C	43C	72C
Z_7335363_C	44C	73C
Z_7335364_T	44T	74T
Z_7335365_A	45A	75A

Z_7335366_C	43C	76C
Z_7335367_G	45G	76G
Z_7335368_T	41T	77T
Z_7335369_G	39G	78G
Z_7335370_A	39A	78A
Z_7335371_C	38C	78C
Z_7335372_C	38C	77C
Z_7335373_A	39A	37A_39G
Z_7335374_C	39T	39T_36C
Z_7335375_A	39A	76A
Z_7335376_C	40C	76C
Z_7335377_C	39C	77C
Z_7335378_A	39A	77A
Z_7335379_A	41A	78A
Z_7335380_A	42A	79A
Z_7335381_A	41A	80A
Z_7335382_A	42A	81A
Z_7335383_T	42T	82T
Z_7335384_C	43C	81C
Z_7335385_C	44C	82C
Z_7335386_C	43C	81C
Z_7335387_T	42T	80T
Z_7335388_C	43C	82C
Z_7335389_C	42C	82C
Z_7335390_A	43A	80A
Z_7335391_T	43T	81T
Z_7335392_T	43T	81T
Z_7335393_T	43T	81T
Z_7335394_T	43T	79T
Z_7335395_T	43T	76T
Z_7335396_G	42G	77G
Z_7335397_T	41T	80T
Z_7335398_T	42T	80T
Z_7335399_T	41T	81T
Z_7335400_G	41G	81G
Z_7335401_G	41G	82G
Z_7335402_A	41A	82A
Z_7335403_T	42T	82T
Z_7335404_C	42C	83C
Z_7335405_T	41T	81T
Z_7335406_G	42G	81G
Z_7335407_T	42T	80T
Z_7335408_G	44G	80G

Z_7335409_T	44T	80T
Z_7335410_G	45G	74G
Z_7335411_C	46C	73C
Z_7335412_A	47A	72A
Z_7335413_A	48A	74A
Z_7335414_A	46A	75A
Z_7335415_T	47T	76T
Z_7335416_A	45A	76A
Z_7335417_T	46T	75T
Z_7335418_G	46G	74G
Z_7335419_G	46G	74G
Z_7335420_G	44G	74G
Z_7335421_T	44T	71T
Z_7335422_G	44G	74G
Z_7335423_A	44A	74A
Z_7335424_A	45A	74A
Z_7335425_C	44C	72C
Z_7335426_T	44T	73T
Z_7335427_T	44T	72T
Z_7335428_C	43C	73C
Z_7335429_C	42C	71C
Z_7335430_T	43T	73T
Z_7335431_T	42T	73T
Z_7335432_C	42C	73C
Z_7335433_T	41T	76T
Z_7335434_C	41C	77C
Z_7335435_A	42A	75A
Z_7335436_A	42A	75A
Z_7335437_G	40G	74G
Z_7335438_A	42A	75A
Z_7335439_G	41G	75G
Z_7335440_A	39A	75A
Z_7335441_A	39A	73A
Z_7335442_G	38G	74G
Z_7335443_A	39A	73A
Z_7335444_G	39G	72G
Z_7335445_G	39G	71G
Z_7335446_G	40G	72G
Z_7335447_A	39A	72A
Z_7335448_G	39G	74G
Z_7335449_T	36T	73T
Z_7335450_G	35G	74G
Z_7335451_A	36A	73A

Z_7335452_A	37A	74A
Z_7335453_C	37C	72C
Z_7335454_T	33T	72T
Z_7335455_C	35C	73C
Z_7335456_A	36A	74A
Z_7335457_A	35A	74A
Z_7335458_T	36T	74T
Z_7335459_T	37T	73T
Z_7335460_G	36G	73G
Z_7335461_G	36G	72G
Z_7335462_G	35G	75G
Z_7335463_G	35G	72G
Z_7335464_C	35C	72C
Z_7335465_A	37A	72A
Z_7335466_T	35T	72T
Z_7335467_A	36A	74A
Z_7335468_C	34C	74C
Z_7335469_A	35A	75A
Z_7335470_G	36G	75G
Z_7335471_G	36G	76G
Z_7335472_G	33G	79G
Z_7335473_A	35A	79A
Z_7335474_G	35G	78G
Z_7335475_G	35G	77G
Z_7335476_A	38A	76A
Z_7335477_A	38A	76A
Z_7335478_G	33G	76G
Z_7335479_G	35G	76G
Z_7335480_A	36A	74A
Z_7335481_G	35G	74G
Z_7335482_G	35G	73G
Z_7335483_C	36C	74C
Z_7335484_A	37A	73A
Z_7335485_A	39A	73A
Z_7335486_A	39A	74A
Z_7335487_G	38G	75G
Z_7335488_G	38G	76G
Z_7335489_A	40A	77A
Z_7335490_A	42A	80A
Z_7335491_C	41C	80C
Z_7335492_A	41A	79A
Z_7335493_T	40T	80T
Z_7335494_A	40A	80A

Z_7335495_G	40G	81G
Z_7335496_T	43T	79T
Z_7335497_A	45A	79A
Z_7335498_T	46T	79T
Z_7335499_G	46G	78G
Z_7335500_C	45T	36T_44C
Z_7335501_A	46A	83A
Z_7335502_T	46T	82T
Z_7335503_T	47T	81T
Z_7335504_T	46T	82T
Z_7335505_G	45G	82G
Z_7335506_G	46G	82G
Z_7335507_G	46G	82G
Z_7335508_C	47C	79C
Z_7335509_A	48A	82A
Z_7335510_G	47G	82G
Z_7335511_G	49G	80G
Z_7335512_A	51A	80A
Z_7335513_T	50T	82T
Z_7335514_C	52C	83C
Z_7335515_T	52T	83T
Z_7335516_T	53T	85T
Z_7335517_G	54G	86G
Z_7335518_C	53C	87C
Z_7335519_C	54C	89C
Z_7335520_C	55C	89C
Z_7335521_C	55C	89C
Z_7335522_T	53T	90T
Z_7335523_T	53T	89T
Z_7335524_T	54T	89T
Z_7335525_A	53A	88A
Z_7335526_G	53G	89G
Z_7335527_A	54A	90A
Z_7335528_G	53G	87G
Z_7335529_C	52C	86C
Z_7335530_C	54C	88C
Z_7335531_A	55A	89A
Z_7335532_T	54T	88T
Z_7335533_G	53G	88G
Z_7335534_C	54C	88C
Z_7335535_A	55A	88A
Z_7335536_A	55A	89A
Z_7335537_A	55A	87A

Z_7335538_C	54C	88C
Z_7335539_T	55T	88T
Z_7335540_C	54C	88C
Z_7335541_C	54C	88C
Z_7335542_A	54A	91A
Z_7335543_G	53G	91G
Z_7335544_G	53G	92G
Z_7335545_G	52G	90G
Z_7335546_C	50C	92C
Z_7335547_C	51C	93C
Z_7335548_C	51C	94C
Z_7335549_A	50A	95A
Z_7335550_A	50A	94A
Z_7335551_A	48A	92A
Z_7335552_C	48C	93C
Z_7335553_A	49A	92A
Z_7335554_C	49C	92C
Z_7335555_G	45G	91G
Z_7335556_G	46G	92G
Z_7335557_C	46C	89C
Z_7335558_T	46T	89T
Z_7335559_G	46G	90G
Z_7335560_C	45C	89C
Z_7335561_A	45A	88A
Z_7335562_G	44G	89G
Z_7335563_T	43T	94T
Z_7335564_T	44T	93T
Z_7335565_C	44C	92C
Z_7335566_G	41G	96G
Z_7335567_T	39T	92T
Z_7335568_T	39T	95T
Z_7335569_G	40G	95G
Z_7335570_T	40T	95T
Z_7335571_T	39T	95T
Z_7335572_C	41C	94C
Z_7335573_C	41C	96C
Z_7335574_T	40T	96T
Z_7335575_C	40C	97C
Z_7335576_T	40T	98T
Z_7335577_G	39G	98G
Z_7335578_T	38T	97T
Z_7335579_A	38A	97A
Z_7335580_G	37G	98G

Z_7335581_A	39A	97A
Z_7335582_T	39T	96T
Z_7335583_G	39G	95G
Z_7335584_A	35A	94A
Z_7335585_T	36T	91T
Z_7335586_G	33G	92G
Z_7335587_G	33G	90G
Z_7335588_A	36A	88A
Z_7335589_T	37T	87T
Z_7335590_G	36G	86G
Z_7335591_G	38G	86G
Z_7335592_A	38A	86A
Z_7335593_C	39C	88C
Z_7335594_A	39A	87A
Z_7335595_C	39C	87C
Z_7335596_A	38A	84A
Z_7335597_G	38G	84G
Z_7335598_A	36A	84A
Z_7335599_T	37T	83T
Z_7335600_A	32A	83A
Z_7335601_C	34C	88C
Z_7335602_C	36C	89C
Z_7335603_T	36T	90T
Z_7335604_T	37T	87T
Z_7335605_T	34T	86T
Z_7335606_A	36A	87A
Z_7335607_A	37A	89A
Z_7335608_T	39T	87T
Z_7335609_C	39C	86C
Z_7335610_T	40T	87T
Z_7335611_C	41C	86C
Z_7335612_C	41C	83C
Z_7335613_C	41C	80C
Z_7335614_T	41T	80T
Z_7335615_G	41G	81G
Z_7335616_C	41C	81C
Z_7335617_C	43C	80C
Z_7335618_G	42G	39A_43G
Z_7335619_T	43T	82T
Z_7335620_T	42T	82T
Z_7335621_C	42C	81C
Z_7335622_T	42T	80T
Z_7335623_C	41C	82C

Z_7335624_A	40A	82A
Z_7335625_A	40A	81A
Z_7335626_A	41A	80A
Z_7335627_G	41G	80G
Z_7335628_C	42C	81C
Z_7335629_A	42A	82A
Z_7335630_G	42G	82G
Z_7335631_C	42C	81C
Z_7335632_A	42A	83A
Z_7335633_A	41A	83A
Z_7335634_A	42A	83A
Z_7335635_C	43C	84C
Z_7335636_A	43A	83A
Z_7335637_G	43G	80G
Z_7335638_G	46G	79G
Z_7335639_C	46C	78C
Z_7335640_T	45T	79T
Z_7335641_C	46C	77C
Z_7335642_A	45A	78A
Z_7335643_G	43G	77G
Z_7335644_C	44C	78C
Z_7335645_T	47T	78T
Z_7335646_G	47G	78G
Z_7335647_A	49A	80A
Z_7335648_C	50C	80C
Z_7335649_A	52A	80A
Z_7335650_T	52T	81T
Z_7335651_G	50G	81G
Z_7335652_G	49G	81G
Z_7335653_A	49A	84A
Z_7335654_A	51A	85A
Z_7335655_A	51A	84A
Z_7335656_C	49C	84C
Z_7335657_C	49C	84C
Z_7335658_A	49A	83A
Z_7335659_T	50T	82T
Z_7335660_T	48T	82T
Z_7335661_A	48A	77A
Z_7335662_T	48T	76T
Z_7335663_C	48C	75C
Z_7335664_C	47C	75C
Z_7335665_C	47C	75C
Z_7335666_C	49C	74C

Z_7335667_A	49A	75A
Z_7335668_G	49G	73G
Z_7335669_C	51C	76C
Z_7335670_G	51G	77G
Z_7335671_C	51C	77C
Z_7335672_C	50C	78C
Z_7335673_C	50C	78C
Z_7335674_A	49A	80A
Z_7335675_G	50G	80G
Z_7335676_A	50A	82A
Z_7335677_G	50G	82G
Z_7335678_C	47C	83C
Z_7335679_C	47C	84C
Z_7335680_C	47C	83C
Z_7335681_T	48T	83T
Z_7335682_C	46C	83C
Z_7335683_T	48T	85T
Z_7335684_C	46C	86C
Z_7335685_C	47C	87C
Z_7335686_C	46C	87C
Z_7335687_T	47T	85T
Z_7335688_G	47G	84G
Z_7335689_G	46G	84G
Z_7335690_T	45C	45T_37C
Z_7335691_G	46G	82G
Z_7335692_T	43C	45T_37C
Z_7335693_G	48G	82G
Z_7335694_G	49G	82G
Z_7335695_G	48G	81G
Z_7335696_C	50C	77C
Z_7335697_A	50A	77A
Z_7335698_T	49T	76T
Z_7335699_G	47G	75G
Z_7335700_T	48T	76T
Z_7335701_T	48T	76T
Z_7335702_G	49G	74G
Z_7335703_T	50T	74T
Z_7335704_G	51G	74G
Z_7335705_G	50G	73G
Z_7335706_A	51A	72A
Z_7335707_A	51A	75A
Z_7335708_A	48A	75A
Z_7335709_A	47A	74A

Z_7335710_G	47G	74G
Z_7335711_A	47A	73A
Z_7335712_G	48G	74G
Z_7335713_G	49G	74G
Z_7335714_T	48T	73T
Z_7335715_C	46C	74C
Z_7335716_T	45T	78T
Z_7335717_T	44T	77T
Z_7335718_A	45A	76A
Z_7335719_G	43G	79G
Z_7335720_G	39G	79G
Z_7335721_A	41A	79A
Z_7335722_A	43A	81A
Z_7335723_C	43C	80C
Z_7335724_A	43A	78A
Z_7335725_C	43C	79C
Z_7335726_A	43A	81A
Z_7335727_A	43A	79A
Z_7335728_G	42G	79G
Z_7335729_G	39G	80G
Z_7335730_C	40C	78C
Z_7335731_G	41G	80G
Z_7335732_C	41C	83C
Z_7335733_A	40A	82A
Z_7335734_A	40A	84A
Z_7335735_A	39A	84A
Z_7335736_G	38G	85G
Z_7335737_G	37G	84G
Z_7335738_T	37T	84T
Z_7335739_T	36T	81T
Z_7335740_C	36C	84C
Z_7335741_T	34T	84T
Z_7335742_T	33T	83T
Z_7335743_T	32T	83T
Z_7335744_T	33T	82T
Z_7335745_G	32G	80G
Z_7335746_G	33G	79G
Z_7335747_T	32T	80T
Z_7335748_G	32G	77G
Z_7335749_C	31C	75C
Z_7335750_G	32G	75G
Z_7335751_A	32A	74A
Z_7335752_G	33G	75G

Z_7335753_A	32A	73A
Z_7335754_G	31G	75G
Z_7335755_G	32G	75G
Z_7335756_C	33C	75C
Z_7335757_C	32C	75C
Z_7335758_A	33A	75A
Z_7335759_T	31T	76T
Z_7335760_T	32T	79T
Z_7335761_A	32A	81A
Z_7335762_G	32G	80G
Z_7335763_C	34C	82C
Z_7335764_A	33A	83A
Z_7335765_G	33G	81G
Z_7335766_G	32G	81G
Z_7335767_T	32T	80T
Z_7335768_G	31G	81G
Z_7335769_T	32T	79T
Z_7335770_G	31G	79G
Z_7335771_A	31A	76A
Z_7335772_A	30A	74A
Z_7335773_G	29G	76G
Z_7335774_C	27C	75C
Z_7335775_C	28C	76C
Z_7335776_G	28G	77G
Z_7335777_G	30G	78G
Z_7335778_G	31G	76G
Z_7335779_G	31G	77G
Z_7335780_A	31A	77A
Z_7335781_G	30G	77G
Z_7335782_A	28A	80A
Z_7335783_T	29T	82T
Z_7335784_A	29A	82A
Z_7335785_A	29A	82A
Z_7335786_C	31C	84C
Z_7335787_C	32C	84C
Z_7335788_A	31A	85A
Z_7335789_G	30G	85G
Z_7335790_A	30A	85A
Z_7335791_T	30T	87T
Z_7335792_C	29C	86C
Z_7335793_C	29C	87C
Z_7335794_C	29C	40T_48C
Z_7335795_C	29C	87C

Z_7335796_T	29T	86T
Z_7335797_T	29T	89T
Z_7335798_T	28T	89T
Z_7335799_A	29A	89A
Z_7335800_A	30A	90A
Z_7335801_G	28G	90G
Z_7335802_C	28C	88C
Z_7335803_T	28T	88T
Z_7335804_G	28G	88G
Z_7335805_G	28G	87G
Z_7335806_C	27C	88C
Z_7335807_A	29A	89A
Z_7335808_G	29G	89G
Z_7335809_G	28G	93G
Z_7335810_C	28C	92C
Z_7335811_T	29T	88T
Z_7335812_G	29G	87G
Z_7335813_T	29T	86T
Z_7335814_T	28T	83T
Z_7335815_G	28G	85G
Z_7335816_T	27T	83T
Z_7335817_T	29T	82T
Z_7335818_G	30G	84G
Z_7335819_G	30G	84G
Z_7335820_G	30G	86G
Z_7335821_A	29A	85A
Z_7335822_A	28A	86A
Z_7335823_A	28A	85A
Z_7335824_A	29A	84A
Z_7335825_A	30A	87A
Z_7335826_A	29A	84A
Z_7335827_G	29G	82G
Z_7335828_T	30T	82T
Z_7335829_G	30G	81G
Z_7335830_G	29G	79G
Z_7335831_G	29G	79G
Z_7335832_T	29T	77T
Z_7335833_G	27G	77G
Z_7335834_A	26A	76A
Z_7335835_T	27T	74T
Z_7335836_T	28T	74T
Z_7335837_T	30T	72T
Z_7335838_A	31A	70A

Z_7335839_T	31T	73T
Z_7335840_C	33C	74C
Z_7335841_C	33C	76C
Z_7335842_T	34T	75T
Z_7335843_C	34C	77C
Z_7335844_T	34T	77T
Z_7335845_A	32A	79A
Z_7335846_A	32A	79A
Z_7335847_T	31T	78T
Z_7335848_A	31A	76A
Z_7335849_G	32G	75G
Z_7335850_G	32G	75G
Z_7335851_C	33C	75C
Z_7335852_T	33T	75T
Z_7335853_T	34T	74T
Z_7335854_G	34G	75G
Z_7335855_A	34A	72A
Z_7335856_C	33C	70C
Z_7335857_A	33A	69A
Z_7335858_A	32A	68A
Z_7335859_C	32C	64C
Z_7335860_C	31C	64C
Z_7335861_C	32C	64C
Z_7335862_C	31C	66C
Z_7335863_C	31C	67C
Z_7335864_A	31A	67A
Z_7335865_G	31G	67G
Z_7335866_G	32G	67G
Z_7335867_A	32A	67A
Z_7335868_G	33G	68G
Z_7335869_A	32A	68A
Z_7335870_G	31G	69G
Z_7335871_G	31G	68G
Z_7335872_A	30A	67A
Z_7335873_G	30G	67G
Z_7335874_C	29C	65C
Z_7335875_A	31A	65A
Z_7335876_A	32A	64A
Z_7335877_G	31G	64G
Z_7335878_C	32C	63C
Z_7335879_A	33A	64A
Z_7335880_G	33G	65G
Z_7335881_G	31G	64G

Z_7335882_C	32C	65C
Z_7335883_A	34A	65A
Z_7335884_A	34A	65A
Z_7335885_G	33G	64G
Z_7335886_T	32T	64T
Z_7335887_C	31C	64C
Z_7335888_A	31A	64A
Z_7335889_A	31A	63A
Z_7335890_G	30G	63G
Z_7335891_A	31A	63A
Z_7335892_G	32G	62G
Z_7335893_C	32C	62C
Z_7335894_C	32C	63C
Z_7335895_C	30C	62C
Z_7335896_C	32C	63C
Z_7335897_G	30G	62G
Z_7335898_G	31G	63G
Z_7335899_A	31A	63A
Z_7335900_C	30C	64C
Z_7335901_T	32T	62T
Z_7335902_C	33C	62C
Z_7335903_T	34T	60T
Z_7335904_G	34G	56G
Z_7335905_G	34G	56G
Z_7335906_T	33T	56T
Z_7335907_T	31T	55T
Z_7335908_G	30G	56G
Z_7335909_A	30A	56A
Z_7335910_G	28G	55G
Z_7335911_A	27A	56A
Z_7335912_G	27G	57G
Z_7335913_G	26G	56G
Z_7335914_T	27T	58T
Z_7335915_C	27C	57C
Z_7335916_A	26A	57A
Z_7335917_C	26C	57C
Z_7335918_G	25G	57G
Z_7335919_C	24C	57C
Z_7335920_G	25G	55G
Z_7335921_T	24G	33T_24G
Z_7335922_A	24A	58A
Z_7335923_G	25G	63G
Z_7335924_C	24C	62C

Z_7335925_G	23G	63G
Z_7335926_G	23G	64G
Z_7335927_C	22C	67C
Z_7335928_A	22A	67A
Z_7335929_A	23A	67A
Z_7335930_C	23C	68C
Z_7335931_A	24A	70A
Z_7335932_T	25T	73T
Z_7335933_T	25T	75T
Z_7335934_A	24A	73A
Z_7335935_A	24A	72A
Z_7335936_C	23C	70C
Z_7335937_C	22C	70C
Z_7335938_C	22C	69C
Z_7335939_T	23T	70T
Z_7335940_G	24G	69G
Z_7335941_G	26G	70G
Z_7335942_A	28A	69A
Z_7335943_A	28A	72A
Z_7335944_T	29T	73T
Z_7335945_G	28G	75G
Z_7335946_T	27T	75T
Z_7335947_T	26T	75T
Z_7335948_G	26G	78G
Z_7335949_A	26A	77A
Z_7335950_G	26G	77G
Z_7335951_G	26G	77G
Z_7335952_T	26T	73T
Z_7335953_T	24T	75T
Z_7335954_C	26C	76C
Z_7335955_C	26C	75C
Z_7335956_C	26C	75C
Z_7335957_C	26C	73C
Z_7335958_T	26T	73T
Z_7335959_T	26T	72T
Z_7335960_G	25G	72G
Z_7335961_A	26A	72A
Z_7335962_C	25C	72C
Z_7335963_A	25A	70A
Z_7335964_T	25T	70T
Z_7335965_T	25T	69T
Z_7335966_G	25G	71G
Z_7335967_A	27A	73A

Z_7335968_A	27A	35A_39G
Z_7335969_T	28T	74T
Z_7335970_C	28C	73C
Z_7335971_C	28C	74C
Z_7335972_A	27A	74A
Z_7335973_G	26G	73G
Z_7335974_T	26T	73T
Z_7335975_C	25C	72C
Z_7335976_G	27G	72G
Z_7335977_T	28T	72T
Z_7335978_G	28G	71G
Z_7335979_T	28T	70T
Z_7335980_C	28C	70C
Z_7335981_C	29C	73C
Z_7335982_G	30G	73G
Z_7335983_A	30A	71A
Z_7335984_C	30C	34T_72C
Z_7335985_T	30T	72T
Z_7335986_C	34C	71C
Z_7335987_T	34T	71T
Z_7335988_A	33A	73A
Z_7335989_G	34G	73G
Z_7335990_G	33G	75G
Z_7335991_G	33G	75G
Z_7335992_T	35T	40T_35C
Z_7335993_G	34G	75G
Z_7335994_T	34T	39T_35C
Z_7335995_G	34G	75G
Z_7335996_G	35G	77G
Z_7335997_T	36T	77T
Z_7335998_G	36G	77G
Z_7335999_C	35C	77C
Z_7336000_T	33T	78T
Z_7336001_C	34C	79C
Z_7336002_A	33A	79A
Z_7336003_T	33T_32C	79T_1G_32C
Z_7336004_C	34C	80C
Z_7336005_C	34C	78C
Z_7336006_C	34C	77C
Z_7336007_G	32G	76G
Z_7336008_T	31T	74T
Z_7336009_C	32C	74C
Z_7336010_T	28T	72T

Z_7336011_C	31C	74C
Z_7336012_C	32C	74C
Z_7336013_A	31A	74A
Z_7336014_A	32A	75A
Z_7336015_G	32G	75G
Z_7336016_C	32C	74C
Z_7336017_C	34C	69C
Z_7336018_G	34G	70G
Z_7336019_T	34T	69T
Z_7336020_G	34G	70G
Z_7336021_G	33G	68G
Z_7336022_A	34A	69A
Z_7336023_G	34G	72G
Z_7336024_C	34C	72C
Z_7336025_C	34C	73C
Z_7336026_A	36A	70A
Z_7336027_G	36G	69G
Z_7336028_C	38C	72C
Z_7336029_G	37G	73G
Z_7336030_T	36T	72T
Z_7336031_T	37T	72T
Z_7336032_T	37T	74T
Z_7336033_T	37T	75T
Z_7336034_C	34C	73C
Z_7336035_C	35C	73C
Z_7336036_A	33A	36A_37G
Z_7336037_T	32T	70T
Z_7336038_A	31A	68A
Z_7336039_G	31G	66G
Z_7336040_A	31A	34A_33C
Z_7336041_C	32C	68C
Z_7336042_A	32A	66A
Z_7336043_G	33G	66G
Z_7336044_T	33G	66T
Z_7336045_T	33T	66T
Z_7336046_T	32T	66T
Z_7336047_T	33T	66T
Z_7336048_C	33C	65C
Z_7336049_G	31G	64G
Z_7336050_T	32T	64T
Z_7336051_G	30G	65G
Z_7336052_G	31G	28A_35G
Z_7336053_T	30T	63T

Z_7336054_C	29C	63C
Z_7336055_A	27A	63A
Z_7336056_T	25C	36T_28C
Z_7336057_G	25G	64G
Z_7336058_T	25T	63T
Z_7336059_G	25G	66G
Z_7336060_A	24G	36A_28G
Z_7336061_C	25C	64C
Z_7336062_C	25C	65C
Z_7336063_A	25A	65A
Z_7336064_G	26G	63G
Z_7336065_C	26C	29T_35C
Z_7336066_G	23G	63G
Z_7336067_C	25C	63C
Z_7336068_G	25G	65G
Z_7336069_A	26A	65A
Z_7336070_C	27C	64C
Z_7336071_T	27T	64T
Z_7336072_A	27A	64A
Z_7336073_G	26G	63G
Z_7336074_A	28A	64A
Z_7336075_C	28C	60C
Z_7336076_A	29A	60A
Z_7336077_C	31C	60C
Z_7336078_G	32G	25A_35G
Z_7336079_G	34G	60G
Z_7336080_A	33A	60A
Z_7336081_A	32A	62A
Z_7336082_C	31C	62C
Z_7336083_G	31G	62G
Z_7336084_T	29T	61T
Z_7336085_C	30C	60C
Z_7336086_G	30G	63G
Z_7336087_T	28T	62T
Z_7336088_T	28T	63T
Z_7336089_A	28A	63A
Z_7336090_C	28C	63C
Z_7336091_C	28C	62C
Z_7336092_T	27T	62T
Z_7336093_T	27T	61T
Z_7336094_T	26T	62T
Z_7336095_C	26C	59C
Z_7336096_C	24C	60C

Z_7336097_C	24C	61C
Z_7336098_A	22A	61A
Z_7336099_C	22C	62C
Z_7336100_C	22T	62C
Z_7336101_G	21G	62G
Z_7336102_A	20A	62A
Z_7336103_G	20G	65G
Z_7336104_G	19G	64G
Z_7336105_T	20T	66T
Z_7336106_G	19G	64G
Z_7336107_G	19G	65G
Z_7336108_T	19T	67T
Z_7336109_C	17C	68C
Z_7336110_C	18C	67C
Z_7336111_C	16C	69C
Z_7336112_T	14A_38T_7G_14C	70T
Z_7336113_A	9A	69A
Z_7336114_T	8T	69T
Z_7336115_T	14T	70T
Z_7336116_T	14T	69T
Z_7336117_A	6A	72A
Z_7336118_C	6C	72C
Z_7336119_C	6C	71C
Z_7336120_G	6G	68G
Z_7336121_T	15T	69T
Z_7336122_A	15A	68A
Z_7336123_T	16T	65T
Z_7336124_T	16T	64T
Z_7336125_T	16T	66T
Z_7336126_T	16T	68T
Z_7336127_T	16T	67T
Z_7336128_C	9T_7C	67C
Z_7336129_G	18G	68G
Z_7336130_C	18C	68C
Z_7336131_T	12T_6G	69T
Z_7336132_C	18C	69C
Z_7336133_C	19C	71C
Z_7336134_A	19A	73A
Z_7336135_T	19T	74T
Z_7336136_A	19A	73A
Z_7336137_A	19A	73A
Z_7336138_G	20G	74G
Z_7336139_A	20A	75A

Z_7336140_T	7T_13C	75T
Z_7336141_G	25G	75G
Z_7336142_C	27C	77C
Z_7336143_A	27A	77A
Z_7336144_C	27C	78C
Z_7336145_C	27C	78C
Z_7336146_T	27T	78T
Z_7336147_C	29C	77C
Z_7336148_T	30T	75T
Z_7336149_C	30C	76C
Z_7336150_C	31C	76C
Z_7336151_A	33A	77A
Z_7336152_T	33T	76T
Z_7336153_A	33A	77A
Z_7336154_A	33A	78A
Z_7336155_G	32G	75G
Z_7336156_A	30A	76A
Z_7336157_C	29C	75C
Z_7336158_G	20A_7G	75G
Z_7336159_C	29C	76C
Z_7336160_A	29A	77A
Z_7336161_C	30C	78C
Z_7336162_C	31C	79C
Z_7336163_A	31A	81A
Z_7336164_A	33A	77A
Z_7336165_A	34A	78A
Z_7336166_T	34T	79T
Z_7336167_T	34T	78T
Z_7336168_T	34T	78T
Z_7336169_T	34T	80T
Z_7336170_T	35T	80T
Z_7336171_A	35A	82A
Z_7336172_A	7A_26G_1C	83A
Z_7336173_G	37G	84G
Z_7336174_A	36A	83A
Z_7336175_G	38G	84G
Z_7336176_A	37A	82A
Z_7336177_A	39A	82A
Z_7336178_G	36G	83G
Z_7336179_A	37A	81A
Z_7336180_A	37A	83A
Z_7336181_A	38A	80A
Z_7336182_A	37A	81A

Z_7336183_C	38C	79C
Z_7336184_A	39A	80A
Z_7336185_G	39G	80G
Z_7336186_G	40G	83G
Z_7336187_A	38A	83A
Z_7336188_A	40A	84A
Z_7336189_A	40A	87A
Z_7336190_A	41A	87A
Z_7336191_A	45A	87A
Z_7336192_A	46A	87A
Z_7336193_T	46T	86T
Z_7336194_A	46A	87A
Z_7336195_A	46A	87A
Z_7336196_T	46T	87T
Z_7336197_C	46C	87C
Z_7336198_T	43T	87T
Z_7336199_G	44G	85G
Z_7336200_T	42T	83T
Z_7336201_T	44T	83T
Z_7336202_T	43T	83T
Z_7336203_T	42T	81T
Z_7336204_C	43C	81C
Z_7336205_T	43T	80T
Z_7336206_T	42T	80T
Z_7336207_C	42C	80C
Z_7336208_A	31G	79A
Z_7336209_C	39C	83C
Z_7336210_T	40T	82T
Z_7336211_C	41C	82C
Z_7336212_C	40C	79C
Z_7336213_A	39A	76A
Z_7336214_T	43T	77T
Z_7336215_A	43A	78A
Z_7336216_A	45A	78A
Z_7336217_G	43G	78G
Z_7336218_A	43A	78A
Z_7336219_C	42C	78C
Z_7336220_G	42G	76G
Z_7336221_C	40C	75C
Z_7336222_A	41A	73A
Z_7336223_C	41C	74C
Z_7336224_A	42A	74A
Z_7336225_G	39G	75G

Z_7336226_A	38A	74A
Z_7336227_C	41C	75C
Z_7336228_T	42T	73T
Z_7336229_T	42T	73T
Z_7336230_T	43T	73T
Z_7336231_C	42C	72C
Z_7336232_C	43C	72C
Z_7336233_A	43A	70A
Z_7336234_C	41C	69C
Z_7336235_C	40C	72C
Z_7336236_T	39T	71T
Z_7336237_C	39C	73C
Z_7336238_C	39C	75C
Z_7336239_C	39C	74C
Z_7336240_T	38T	75T
Z_7336241_G	37G	75G
Z_7336242_T	37T	75T
Z_7336243_T	36T	76T
Z_7336244_T	36T	76T
Z_7336245_T	36T	76T
Z_7336246_G	35G	76G
Z_7336247_T	33T	75T
Z_7336248_G	32G	77G
Z_7336249_G	30G	76G
Z_7336250_G	30G	77G
Z_7336251_A	20G	77A
Z_7336252_A	20G	79A
Z_7336253_A	21G	78A
Z_7336254_A	28A	78A
Z_7336255_A	28A	76A
Z_7336256_A	29A	75A
Z_7336257_G	30G	74G
Z_7336258_T	30T	73T
Z_7336259_G	30G	72G
Z_7336260_C	30C	73C
Z_7336261_G	30G	74G
Z_7336262_T	29T	75T
Z_7336263_C	28C	76C
Z_7336264_T	31T	74T
Z_7336265_T	34T	74T
Z_7336266_A	34A	72A
Z_7336267_T	34T	71T
Z_7336268_A	35A	71A

Z_7336269_G	36G	71G
Z_7336270_T	36T	70T
Z_7336271_G	36G	71G
Z_7336272_C	38C	71C
Z_7336273_G	40G	70G
Z_7336274_A	38A	69A
Z_7336275_A	39A	68A
Z_7336276_A	39A	69A
Z_7336277_A	40A	69A
Z_7336278_A	40A	70A
Z_7336279_T	40T	69T
Z_7336280_C	40C	69C
Z_7336281_T	40T	66T
Z_7336282_A	40A	66A
Z_7336283_C	42C	65C
Z_7336284_T	41T	62T
Z_7336285_C	40C	63C
Z_7336286_G	40G	63G
Z_7336287_C	41C	62C
Z_7336288_A	40A	62A
Z_7336289_T	40T	61T
Z_7336290_T	39T	62T
Z_7336291_T	39T	63T
Z_7336292_T	39T	65T
Z_7336293_A	38A	63A
Z_7336294_A	37A	63A
Z_7336295_C	39C	65C
Z_7336296_A	40A	65A
Z_7336297_T	39T	65T
Z_7336298_G	42G	65G
Z_7336299_C	42C	66C
Z_7336300_T	41T	66T
Z_7336301_T	41T	65T
Z_7336302_T	37T	66T
Z_7336303_C	38C	65C
Z_7336304_T	38T	62T
Z_7336305_A	38A	62A
Z_7336306_A	38A	61A
Z_7336307_C	38C	62C
Z_7336308_T	30A_98T_90G_30C	61T
Z_7336309_G	8G	61G
Z_7336310_C	null	61C
Z_7336311_T	null	63T

Z_7336312_A	null	66A
Z_7336313_G	null	66G
Z_7336314_G	null	67G
Z_7336315_T	1C	67T
Z_7336316_T	39T	69T
Z_7336317_G	38G	69G
Z_7336318_G	39G	68G
Z_7336319_C	39C	68C
Z_7336320_A	40A	67A
Z_7336321_G	39G	68G
Z_7336322_G	40G	66G
Z_7336323_A	42A	65A
Z_7336324_G	42G	65G
Z_7336325_G	42G	66G
Z_7336326_T	43T	68T
Z_7336327_G	46G	73G
Z_7336328_G	47G	73G
Z_7336329_G	46A	74G
Z_7336330_A	50A	72A
Z_7336331_C	51C	72C
Z_7336332_A	51A	70A
Z_7336333_A	50A	68A
Z_7336334_G	50G	34A_35G
Z_7336335_C	51C	71C
Z_7336336_G	51G	71G
Z_7336337_A	50A	72A
Z_7336338_T	52T	72T
Z_7336339_G	52G	75G
Z_7336340_G	52G	80G
Z_7336341_G	54G	81G
Z_7336342_A	51A	82A
Z_7336343_G	55G	79G
Z_7336344_C	56C	81C
Z_7336345_T	58T	81T
Z_7336346_G	58G	80G
Z_7336347_A	55A	77A
Z_7336348_A	56A	78A
Z_7336349_T	56T	76T
Z_7336350_C	55C	77C
Z_7336351_T	53T	77T
Z_7336352_G	54G	76G
Z_7336353_T	54T	77T
Z_7336354_C	52C	77C

Z_7336355_A	52A	77A
Z_7336356_C	53C	77C
Z_7336357_G	53G	76G
Z_7336358_T	58T	78T
Z_7336359_G	57G	81G
Z_7336360_G	58G	83G
Z_7336361_A	57A	84A
Z_7336362_T	59T	84T
Z_7336363_T	59T	83T
Z_7336364_C	60C	84C
Z_7336365_G	60G	85G
Z_7336366_A	61A	85A
Z_7336367_T	62T	85T
Z_7336368_C	62C	85C
Z_7336369_T	64T	85T
Z_7336370_T	63T	86T
Z_7336371_A	63A	85A
Z_7336372_C	64C	83C
Z_7336373_A	64A	81A
Z_7336374_A	61A	81A
Z_7336375_T	55T	80T
Z_7336376_G	59G	80G
Z_7336377_G	61G	80G
Z_7336378_C	59C	80C
Z_7336379_T	60T	82T
Z_7336380_G	60G	81G
Z_7336381_G	60G	81G
Z_7336382_T	59T	82T
Z_7336383_C	60C	84C
Z_7336384_T	59T	84T
Z_7336385_T	58T	81T
Z_7336386_C	55C	82C
Z_7336387_C	56C	81C
Z_7336388_G	56G	81G
Z_7336389_A	57A	81A
Z_7336390_T	59T	80T
Z_7336391_C	59C	81C
Z_7336392_T	58T	81T
Z_7336393_T	58T	81T
Z_7336394_G	58G	79G
Z_7336395_C	58C	79C
Z_7336396_A	58A	79A
Z_7336397_G	54G	79G

Z_7336398_C	55C	79C
Z_7336399_C	55C	79C
Z_7336400_C	53C	77C
Z_7336401_A	52A	80A
Z_7336402_G	52G	80G
Z_7336403_A	52A	82A
Z_7336404_G	52G	83G
Z_7336405_G	51G	83G
Z_7336406_C	44C	81C
Z_7336407_T	54T	78T
Z_7336408_T	53T	80T
Z_7336409_C	52C	76C
Z_7336410_T	51T	80T
Z_7336411_G	49G	78G
Z_7336412_C	49C	78C
Z_7336413_G	45G	78G
Z_7336414_A	41A	77A
Z_7336415_T	41T	77T
Z_7336416_T	42T	78T
Z_7336417_T	42T	78T
Z_7336418_A	44A	83A
Z_7336419_A	43A	82A
Z_7336420_C	43C	81C
Z_7336421_C	45C	79C
Z_7336422_C	44C	75C
Z_7336423_G	42G	75G
Z_7336424_C	45C	74C
Z_7336425_A	43A	72A
Z_7336426_G	43G	73G
Z_7336427_C	41T	75C
Z_7336428_G	45G	76G
Z_7336429_C	45C	75C
Z_7336430_C	45C	73C
Z_7336431_A	43A	72A
Z_7336432_C	41C	72C
Z_7336433_C	41C	73C
Z_7336434_T	40T	70T
Z_7336435_C	28T_28G_67C	65C
Z_7336436_G	10G	64G
Z_7336437_T	9T	63T
Z_7336438_C	7C	65C
Z_7336439_C	41C	64C
Z_7336440_G	33G	63G

Z_7336441_T	35T	64T
Z_7336442_C	35C	64C
Z_7336443_C	36C	65C
Z_7336444_C	36C	64C
Z_7336445_C	36C	66C
Z_7336446_C	36T	68C
Z_7336447_T	37T	68T
Z_7336448_G	37G	67G
Z_7336449_G	38G	67G
Z_7336450_A	42A	66A
Z_7336451_A	42A	66A
Z_7336452_A	44A	66A
Z_7336453_G	44G	64G
Z_7336454_T	44T	62T
Z_7336455_T	44T	60T
Z_7336456_G	44G	59G
Z_7336457_A	42A	59A
Z_7336458_A	43A	59A
Z_7336459_C	43C	59C
Z_7336460_T	44T	59T
Z_7336461_A	44A	59A
Z_7336462_C	44C	58C
Z_7336463_C	43C	58C
Z_7336464_T	40T	57T
Z_7336465_C	41C	58C
Z_7336466_T	43T	62T
Z_7336467_C	41C	63C
Z_7336468_T	41T	64T
Z_7336469_T	41T	64T
Z_7336470_A	41A	66A
Z_7336471_A	42A	65A
Z_7336472_G	40G	65G
Z_7336473_G	42G	64G
Z_7336474_C	43C	63C
Z_7336475_A	43A	63A
Z_7336476_C	43C	63C
Z_7336477_T	43T	63T
Z_7336478_G	42G	63G
Z_7336479_G	42G	64G
Z_7336480_C	40C	64C
Z_7336481_A	40A	63A
Z_7336482_G	38G	63G
Z_7336483_G	39G	68G

Z_7336484_G	39G	67G
Z_7336485_A	39A	67A
Z_7336486_T	40C	68T
Z_7336487_A	39A	67A
Z_7336488_G	41G	67G
Z_7336489_T	41T	69T
Z_7336490_A	40A	72A
Z_7336491_A	41A	71A
Z_7336492_G	40G	70G
Z_7336493_A	39A	70A
Z_7336494_G	40G	70G
Z_7336495_C	37C	70C
Z_7336496_T	40T	70T
Z_7336497_A	40A	70A
Z_7336498_A	41A	68A
Z_7336499_A	42A	66A
Z_7336500_T	44T	67T
Z_7336501_T	45T	66T
Z_7336502_C	45C	68C
Z_7336503_A	44A	66A
Z_7336504_T	41T	65T
Z_7336505_C	41C	65C
Z_7336506_C	39C	66C
Z_7336507_T	39T	66T
Z_7336508_T	40T	66T
Z_7336509_G	41G	67G
Z_7336510_G	40G	68G
Z_7336511_G	41G	68G
Z_7336512_G	39G	69G
Z_7336513_A	42A	64A
Z_7336514_A	43A	65A
Z_7336515_T	43T	64T
Z_7336516_G	43G	64G
Z_7336517_T	44T	63T
Z_7336518_T	44T	66T
Z_7336519_G	42G	67G
Z_7336520_A	38A	68A
Z_7336521_A	40A	68A
Z_7336522_C	39C	66C
Z_7336523_C	41C	66C
Z_7336524_T	42T	68T
Z_7336525_C	41C	69C
Z_7336526_C	42C	70C

Z_7336527_A	41A	70A
Z_7336528_G	41G	69G
Z_7336529_C	41C	69C
Z_7336530_A	40A	69A
Z_7336531_T	42T	69T
Z_7336532_G	41G	71G
Z_7336533_G	44G	69G
Z_7336534_G	44G	71G
Z_7336535_G	43G	73G
Z_7336536_T	42T	72T
Z_7336537_G	43G	72G
Z_7336538_C	43C	71C
Z_7336539_A	43A	72A
Z_7336540_G	43G	70G
Z_7336541_C	42C	68C
Z_7336542_T	43T	69T
Z_7336543_A	42A	71A
Z_7336544_A	42A	72A
Z_7336545_T	42C	72T
Z_7336546_G	42G	71G
Z_7336547_G	41G	71G
Z_7336548_T	43T	72T
Z_7336549_C	43A	71C
Z_7336550_C	46C	72C
Z_7336551_C	45C	72C
Z_7336552_T	45T	74T
Z_7336553_C	44C	74C
Z_7336554_T	43T	75T
Z_7336555_G	43G	73G
Z_7336556_G	44G	74G
Z_7336557_T	45T	74T
Z_7336558_G	43G	76G
Z_7336559_C	44C	77C
Z_7336560_C	44C	76C
Z_7336561_A	43A	71A
Z_7336562_C	43C	71C
Z_7336563_T	43T	70T
Z_7336564_T	43T	70T
Z_7336565_C	43C	70C
Z_7336566_A	42A	71A
Z_7336567_C	40C	71C
Z_7336568_A	38G	73A
Z_7336569_C	38C	75C

Z_7336570_T	36T	75T
Z_7336571_G	36G	74G
Z_7336572_C	35C	71C
Z_7336573_A	36A	71A
Z_7336574_G	36G	72G
Z_7336575_T	38T	75T
Z_7336576_T	38T	75T
Z_7336577_T	39T	75T
Z_7336578_A	38A	70A
Z_7336579_C	38C	71C
Z_7336580_A	39A	71A
Z_7336581_G	39G	69G
Z_7336582_C	40C	68C
Z_7336583_A	39A	68A
Z_7336584_T	39T	68T
Z_7336585_C	39C	67C
Z_7336586_T	39T	69T
Z_7336587_G	38G	70G
Z_7336588_C	39C	72C
Z_7336589_C	39C	71C
Z_7336590_T	38T	71T
Z_7336591_T	37T	72T
Z_7336592_T	37T	73T
Z_7336593_T	36T	74T
Z_7336594_T	36T	75T
Z_7336595_A	36A	73A
Z_7336596_A	37A	74A
Z_7336597_A	37A	76A
Z_7336598_C	37C	78C
Z_7336599_T	37T	80T
Z_7336600_G	37G	81G
Z_7336601_T	36T	80T
Z_7336602_A	36A	79A
Z_7336603_T	34T	78T
Z_7336604_G	34G	79G
Z_7336605_A	33A	78A
Z_7336606_G	32G	77G
Z_7336607_C	34C	76C
Z_7336608_T	35T	76T
Z_7336609_T	37T	76T
Z_7336610_T	37T	77T
Z_7336611_G	37G	77G
Z_7336612_C	37C	81C

Z_7336613_A	37A	79A
Z_7336614_G	39G	79G
Z_7336615_C	37C	79C
Z_7336616_A	37A	80A
Z_7336617_G	38G	80G
Z_7336618_A	37A	79A
Z_7336619_G	36G	79G
Z_7336620_G	36G	79G
Z_7336621_C	36A	81C
Z_7336622_C	36C	81C
Z_7336623_T	37T	79T
Z_7336624_C	38C	79C
Z_7336625_G	36G	80G
Z_7336626_C	36C	80C
Z_7336627_C	33C	78C
Z_7336628_T	36T	78T
Z_7336629_G	34G	76G
Z_7336630_T	35T	75T
Z_7336631_T	35T	76T
Z_7336632_A	34A	76A
Z_7336633_T	34T	76T
Z_7336634_C	34C	77C
Z_7336635_C	33C	77C
Z_7336636_C	35C	77C
Z_7336637_T	35C	77T
Z_7336638_G	37G	77G
Z_7336639_G	35G	78G
Z_7336640_A	37A	78A
Z_7336641_T	36T	78T
Z_7336642_C	36C	78C
Z_7336643_A	35A	79A
Z_7336644_A	35A	81A
Z_7336645_G	33G	82G
Z_7336646_C	32C	84C
Z_7336647_C	31C	81C
Z_7336648_C	31C	83C
Z_7336649_C	31C	81C
Z_7336650_A	30A	83A
Z_7336651_G	31G	82G
Z_7336652_G	30G	82G
Z_7336653_T	31T	80T
Z_7336654_G	30G	79G
Z_7336655_C	31C	80C

Z_7336656_A	32A	81A
Z_7336657_C	33C	37T_45C
Z_7336658_G	34G	83G
Z_7336659_G	34G	83G
Z_7336660_A	34A	82A
Z_7336661_T	35T	83T
Z_7336662_A	35A	86A
Z_7336663_T	34T	84T
Z_7336664_T	33T	83T
Z_7336665_G	33G	84G
Z_7336666_G	32G	84G
Z_7336667_G	32G	85G
Z_7336668_A	32A	86A
Z_7336669_T	32T	87T
Z_7336670_G	34G	86G
Z_7336671_G	32G	87G
Z_7336672_G	32G	91G
Z_7336673_A	33A	91A
Z_7336674_T	33T	91T
Z_7336675_A	33A	91A
Z_7336676_A	34A	92A
Z_7336677_A	32A	92A
Z_7336678_A	32A	93A
Z_7336679_A	30A	91A
Z_7336680_C	30C	91C
Z_7336681_A	30A	90A
Z_7336682_G	29G	90G
Z_7336683_C	29C	88C
Z_7336684_A	26A	86A
Z_7336685_C	27C	89C
Z_7336686_C	27C	88C
Z_7336687_C	28C	87C
Z_7336688_A	28A	86A
Z_7336689_G	28G	87G
Z_7336690_T	28T	87T
Z_7336691_C	29C	87C
Z_7336692_C	29C	83C
Z_7336693_T	30T	82T
Z_7336694_T	29T	80T
Z_7336695_C	29C	81C
Z_7336696_A	31A	80A
Z_7336697_A	32A	80A
Z_7336698_A	31A	80A

Z_7336699_A	31A	81A
Z_7336700_G	31G	80G
Z_7336701_A	31A	81A
Z_7336702_C	31C	81C
Z_7336703_C	29C	82C
Z_7336704_A	30A	81A
Z_7336705_T	30T	80T
Z_7336706_C	30C	81C
Z_7336707_C	31C	78C
Z_7336708_A	30A	77A
Z_7336709_G	30G	79G
Z_7336710_C	29C	79C
Z_7336711_T	31T	78T
Z_7336712_T	31T	78T
Z_7336713_T	31T	79T
Z_7336714_G	31G	77G
Z_7336715_G	32G	75G
Z_7336716_G	31G	74G
Z_7336717_T	32T	75T
Z_7336718_T	31T	75T
Z_7336719_C	30C	75C
Z_7336720_A	31A	74A
Z_7336721_A	30A	74A
Z_7336722_T	29T	77T
Z_7336723_G	27G	77G
Z_7336724_G	28G	77G
Z_7336725_G	28G	77G
Z_7336726_A	27A	78A
Z_7336727_C	25C	78C
Z_7336728_A	24A	78A
Z_7336729_G	25G	76G
Z_7336730_A	27A	76A
Z_7336731_A	26A	76A
Z_7336732_G	26G	76G
Z_7336733_G	26G	75G
Z_7336734_C	26C	73C
Z_7336735_A	27A	76A
Z_7336736_G	28G	76G
Z_7336737_G	28G	76G
Z_7336738_C	28C	74C
Z_7336739_C	29C	73C
Z_7336740_A	27A	73A
Z_7336741_A	28A	73A

Z_7336742_C	28T	74C
Z_7336743_G	27G	73G
Z_7336744_C	27C	75C
Z_7336745_C	27C	75C
Z_7336746_A	26G	77A
Z_7336747_T	28T	76T
Z_7336748_C	28C	77C
Z_7336749_A	28A	78A
Z_7336750_A	28A	76A
Z_7336751_A	28A	76A
Z_7336752_C	27C	76C
Z_7336753_A	27A	75A
Z_7336754_G	28G	75G
Z_7336755_G	27G	76G
Z_7336756_A	27G	73A
Z_7336757_T	26T	70T
Z_7336758_C	26C	71C
Z_7336759_T	26T	70T
Z_7336760_G	27G	70G
Z_7336761_T	26T	70T
Z_7336762_G	25G	70G
Z_7336763_G	25G	68G
Z_7336764_C	25C	66C
Z_7336765_T	25T	64T
Z_7336766_G	23G	62G
Z_7336767_G	22G	58G
Z_7336768_G	22G	58G
Z_7336769_C	22C	58C
Z_7336770_A	21A	58A
Z_7336771_T	21T	53T
Z_7336772_T	21T	53T
Z_7336773_C	32A_21C	42A_66C
Z_7336774_A	10A	41A
Z_7336775_C	10C	40C
Z_7336776_A	10A	40A
Z_7336777_C	9A_8C	39C
Z_7336778_A	19A	41A
Z_7336779_C	19C	11A_44C
Z_7336780_A	20A	59A
Z_7336781_C	19C	58C
Z_7336782_A	19A	60A
Z_7336783_C	19C	60C
Z_7336784_A	19A	60A

Z_7336785_C	18C	62C
Z_7336786_A	18A	62A
Z_7336787_C	18C	62C
Z_7336788_A	20A	62A
Z_7336789_C	20C	62C
Z_7336790_A	14A	60A
Z_7336791_C	14C	59C
Z_7336792_A	15A	59A
Z_7336793_C	17C	60C
Z_7336794_A	17C	58A
Z_7336795_C	18C	58C
Z_7336796_A	18A	58A
Z_7336797_T	19T	59T
Z_7336798_C	19C	59C
Z_7336799_G	19G	58G
Z_7336800_C	19C	58C
Z_7336801_C	19C	59C
Z_7336802_A	20A	61A
Z_7336803_T	20T	62T
Z_7336804_A	19A	61A
Z_7336805_A	21A	60A
Z_7336806_C	21C	61C
Z_7336807_A	21A	61A
Z_7336808_T	22T	60T
Z_7336809_T	22T	60T
Z_7336810_T	21T	57T
Z_7336811_G	21G	58G
Z_7336812_G	19G	57G
Z_7336813_T	19T	59T
Z_7336814_G	19G	59G
Z_7336815_T	17T	60T
Z_7336816_T	16T	60T
Z_7336817_G	16G	59G
Z_7336818_A	16A	60A
Z_7336819_T	16T_26C	59T
Z_7336820_C	16C	62C
Z_7336821_C	15C	61C
Z_7336822_C	15C	61C
Z_7336823_C	16C	63C
Z_7336824_C	16C	63C
Z_7336825_C	16C	62C
Z_7336826_C	18C	62C
Z_7336827_C	17C	62C

Z_7336828_A	16A	63A
Z_7336829_A	16A	62A
Z_7336830_A	16A	61A
Z_7336831_C	16C	61C
Z_7336832_T	18T	61T
Z_7336833_T	18T	61T
Z_7336834_G	18G	63G
Z_7336835_C	17C	62C
Z_7336836_C	18C	61C
Z_7336837_A	18A	60A
Z_7336838_G	18G	61G
Z_7336839_C	18C	61C
Z_7336840_T	17T	59T
Z_7336841_T	18T	60T
Z_7336842_G	19G	60G
Z_7336843_G	19G	62G
Z_7336844_G	20G	61G
Z_7336845_A	20A	62A
Z_7336846_A	21A	63A
Z_7336847_G	21G	62G
Z_7336848_G	21G	62G
Z_7336849_G	22G	62G
Z_7336850_A	22A	62A
Z_7336851_G	22G	62G
Z_7336852_T	22T	63T
Z_7336853_T	22T	63T
Z_7336854_C	22C	63C
Z_7336855_T	22T	64T
Z_7336856_C	22C	63C
Z_7336857_A	20A	64A
Z_7336858_T	20T	64T
Z_7336859_T	20T	65T
Z_7336860_T	21T	65T
Z_7336861_C	21C	64C
Z_7336862_A	19A	64A
Z_7336863_G	19G	62G
Z_7336864_G	18G	63G
Z_7336865_C	20C	63C
Z_7336866_A	20A	66A
Z_7336867_A	20A	66A
Z_7336868_G	20G	66G
Z_7336869_T	20T	65T
Z_7336870_T	20T	66T

Z_7336871_G	21G	67G
Z_7336872_T	21T	67T
Z_7336873_G	22G	66G
Z_7336874_A	22A	63A
Z_7336875_C	23C	64C
Z_7336876_A	24A	64A
Z_7336877_G	24G	62G
Z_7336878_A	24A	63A
Z_7336879_A	24A	63A
Z_7336880_T	24T	61T
Z_7336881_C	24C	61C
Z_7336882_C	23C	61C
Z_7336883_C	26C	60C
Z_7336884_C	26C	61C
Z_7336885_C	26C	61C
Z_7336886_T	29T	60T
Z_7336887_T	29T	61T
Z_7336888_T	30T	60T
Z_7336889_C	30C	60C
Z_7336890_T	30T	60T
Z_7336891_C	30C	61C
Z_7336892_A	30A	61A
Z_7336893_G	29G	62G
Z_7336894_A	29A	62A
Z_7336895_G	28G	62G
Z_7336896_T	29T	62T
Z_7336897_A	28A	60A
Z_7336898_A	28A	59A
Z_7336899_C	28C	60C
Z_7336900_A	28A	61A
Z_7336901_T	28T	61T
Z_7336902_T	29T	61T
Z_7336903_T	27T	61T
Z_7336904_C	26C	61C
Z_7336905_T	27T	61T
Z_7336906_G	27G	60G
Z_7336907_C	27C	59C
Z_7336908_A	26A	58A
Z_7336909_A	25A	58A
Z_7336910_A	24A	56A
Z_7336911_T	25T	57T
Z_7336912_C	25C	55C
Z_7336913_T	25T	55T

Z_7336914_T	24T	56T
Z_7336915_A	25A	53A
Z_7336916_T	24T	53T
Z_7336917_A	25A	54A
Z_7336918_T	26T	52T
Z_7336919_T	26T	53T
Z_7336920_T	26T	53T
Z_7336921_A	24A	53A
Z_7336922_T	26T	54T
Z_7336923_A	27A	53A
Z_7336924_G	29G	54G
Z_7336925_A	29A	54A
Z_7336926_A	30A	54A
Z_7336927_T	30T	53T
Z_7336928_A	29A	53A
Z_7336929_A	29G	27A_25G
Z_7336930_G	28G	54G
Z_7336931_G	27G	53G
Z_7336932_G	28G	53G
Z_7336933_A	28A	52A
Z_7336934_G	30G	50G
Z_7336935_G	29G	49G
Z_7336936_A	28A	48A
Z_7336937_A	27A	48A
Z_7336938_A	28A	46A
Z_7336939_T	27T	46T
Z_7336940_G	30G	45G
Z_7336941_T	27T	44T
Z_7336942_T	28T	46T
Z_7336943_A	27A	46A
Z_7336944_C	27C	48C
Z_7336945_C	26C	49C
Z_7336946_T	27C	26T_23C
Z_7336947_T	26T	48T
Z_7336948_C	26C	47C
Z_7336949_C	26C	47C
Z_7336950_C	26C	47C
Z_7336951_T	26T	45T
Z_7336952_T	26C	24T_20C
Z_7336953_C	26C	44C
Z_7336954_C	25C	44C
Z_7336955_A	25A	44A
Z_7336956_T	25T	46T

Z_7336957_T	24T	48T
Z_7336958_C	25C	50C
Z_7336959_G	25G	50G
Z_7336960_G	25G	54G
Z_7336961_A	27A	32A_21G
Z_7336962_A	27A	54A
Z_7336963_A	27A	54A
Z_7336964_A	28A	54A
Z_7336965_A	30A	55A
Z_7336966_A	30A	54A
Z_7336967_A	31A	53A
Z_7336968_A	31A	53A
Z_7336969_A	31A	53A
Z_7336970_A	31A	52A
Z_7336971_C	31C	52C
Z_7336972_A	30A	52A
Z_7336973_A	31A	51A
Z_7336974_A	31A	51A
Z_7336975_A	31A	51A
Z_7336976_A	31A	50A
Z_7336977_A	32A	51A
Z_7336978_C	32C	51C
Z_7336979_A	32A	49A
Z_7336980_A	34A	52A
Z_7336981_A	35A	52A
Z_7336982_A	35A	52A
Z_7336983_C	34C	52C
Z_7336984_A	35A	53A
Z_7336985_C	34C	52C
Z_7336986_A	35A	52A
Z_7336987_A	35A	52A
Z_7336988_G	32G	51G
Z_7336989_C	33C	51C
Z_7336990_T	33T	51T
Z_7336991_A	34A	52A
Z_7336992_G	33G	51G
Z_7336993_G	32G	51G
Z_7336994_A	30A	50A
Z_7336995_G	30G	50G
Z_7336996_T	30T	50T
Z_7336997_T	30T	50T
Z_7336998_A	32A	50A
Z_7336999_C	33C	50C

Z_7337000_A	33A	51A
Z_7337001_T	32T	52T
Z_7337002_C	31C	52C
Z_7337003_G	32G	53G
Z_7337004_C	29C	53C
Z_7337005_T	30T	53T
Z_7337006_A	30A	52A
Z_7337007_A	31A	53A
Z_7337008_A	33A	54A
Z_7337009_T	33T	53T
Z_7337010_G	32G	52G
Z_7337011_T	32T	53T
Z_7337012_C	32C	52C
Z_7337013_A	34A	51A
Z_7337014_T	35T	51T
Z_7337015_A	35A	52A
Z_7337016_T	34T	51T
Z_7337017_C	34C	51C
Z_7337018_T	33T	51T
Z_7337019_C	34C	50C
Z_7337020_T	34T	50T
Z_7337021_C	33C	50C
Z_7337022_T	33T	51T
Z_7337023_C	32C	53C
Z_7337024_T	33T	54T
Z_7337025_T	34T	52T
Z_7337026_C	33C	52C
Z_7337027_C	33C	52C
Z_7337028_A	35A	52A
Z_7337029_C	36C	53C
Z_7337030_G	36G	53G
Z_7337031_C	36C	53C
Z_7337032_G	37G	53G
Z_7337033_G	38G	54G
Z_7337034_G	38G	54G
Z_7337035_C	35C	54C
Z_7337036_T	36T	54T
Z_7337037_T	35T	52T
Z_7337038_G	34G	52G
Z_7337039_A	34A	51A
Z_7337040_C	35C	52C
Z_7337041_A	33A	52A
Z_7337042_A	34A	52A

Z_7337043_T	32T	51T
Z_7337044_G	32G	53G
Z_7337045_A	32A	52A
Z_7337046_G	32G	54G
Z_7337047_G	30G	54G
Z_7337048_G	30G	54G
Z_7337049_C	31C	54C
Z_7337050_A	31A	52A
Z_7337051_G	32G	51G
Z_7337052_T	32T	50T
Z_7337053_T	31T	48T
Z_7337054_C	33C	48C
Z_7337055_A	35A	45A
Z_7337056_G	33G	44G
Z_7337057_C	34C	43C
Z_7337058_G	34G	43G
Z_7337059_C	34C	42C
Z_7337060_G	34G	42G
Z_7337061_A	34A	42A
Z_7337062_T	35T	41T
Z_7337063_G	36G	43G
Z_7337064_A	37A	43A
Z_7337065_A	37A	43A
Z_7337066_C	40C	44C
Z_7337067_T	39T	46T
Z_7337068_C	40C	46C
Z_7337069_A	39A	46A
Z_7337070_C	38C	46C
Z_7337071_C	39C	46C
Z_7337072_T	39T	45T
Z_7337073_G	38G	45G
Z_7337074_G	38G	47G
Z_7337075_T	39T	44T
Z_7337076_T	40T	43T
Z_7337077_T	39T	43T
Z_7337078_G	36G	46G
Z_7337079_G	38G	46G
Z_7337080_C	36C	46C
Z_7337081_A	37A	47A
Z_7337082_G	38G	46G
Z_7337083_A	36A	47A
Z_7337084_A	34A	48A
Z_7337085_G	35G	50G

Z_7337086_A	36A	48A
Z_7337087_A	35A	48A
Z_7337088_A	37A	48A
Z_7337089_C	38C	49C
Z_7337090_C	38C	49C
Z_7337091_C	41C	49C
Z_7337092_T	41T	48T
Z_7337093_A	41A	48A
Z_7337094_T	41T	49T
Z_7337095_T	40T	49T
Z_7337096_C	39C	48C
Z_7337097_A	39A	49A
Z_7337098_T	37T	48T
Z_7337099_G	35G	48G
Z_7337100_C	36C	49C
Z_7337101_T	36T	52T
Z_7337102_T	35T	51T
Z_7337103_T	35T	50T
Z_7337104_A	35A	50A
Z_7337105_A	34A	51A
Z_7337106_T	35T	51T
Z_7337107_T	35T	51T
Z_7337108_G	35G	51G
Z_7337109_A	36A	52A
Z_7337110_A	36A	52A
Z_7337111_T	36T	53T
Z_7337112_A	35A	53A
Z_7337113_T	36T	54T
Z_7337114_T	37T	54T
Z_7337115_T	38T	55T
Z_7337116_G	37G	55G
Z_7337117_A	36A	54A
Z_7337118_A	39A	52A
Z_7337119_T	38T	50T
Z_7337120_A	37A	51A
Z_7337121_A	36A	54A
Z_7337122_T	36T	53T
Z_7337123_C	36C	54C
Z_7337124_T	34T	54T
Z_7337125_T	32T	54T
Z_7337126_T	32T	54T
Z_7337127_T	31T	55T
Z_7337128_C	31C	54C

Z_7337129_A	33A	55A
Z_7337130_T	34T	55T
Z_7337131_T	36T	55T
Z_7337132_C	36C	54C
Z_7337133_C	36C	55C
Z_7337134_T	34T	54T
Z_7337135_G	36G	52G
Z_7337136_C	33C	52C
Z_7337137_G	35G	52G
Z_7337138_A	35A	52A
Z_7337139_A	35A	50A
Z_7337140_T	35T	52T
Z_7337141_G	34G	51G
Z_7337142_A	34A	52A
Z_7337143_A	35A	53A
Z_7337144_T	35T	54T
Z_7337145_G	35G	54G
Z_7337146_T	34T	53T
Z_7337147_T	36T	54T
Z_7337148_G	36G	55G
Z_7337149_T	36T	55T
Z_7337150_C	37C	54C
Z_7337151_C	36C	54C
Z_7337152_A	36A	55A
Z_7337153_A	36A	57A
Z_7337154_A	36A	58A
Z_7337155_T	37T	57T
Z_7337156_A	35A	57A
Z_7337157_A	36A	57A
Z_7337158_C	34C	55C
Z_7337159_A	33A	55A
Z_7337160_A	33A	55A
Z_7337161_C	32C	54C
Z_7337162_A	35A	50A
Z_7337163_T	35T	53T
Z_7337164_C	35C	59C
Z_7337165_G	35G	59G
Z_7337166_A	37A	60A
Z_7337167_C	35C	61C
Z_7337168_T	37T	61T
Z_7337169_C	37C	60C
Z_7337170_G	36G	60G
Z_7337171_C	37C	63C

Z_7337172_T	34T	63T
Z_7337173_C	35C	61C
Z_7337174_G	35G	62G
Z_7337175_C	34C	66C
Z_7337176_C	38C	66C
Z_7337177_G	38G	67G
Z_7337178_C	38C	66C
Z_7337179_A	38A	66A
Z_7337180_T	37T	65T
Z_7337181_T	38T	65T
Z_7337182_G	38G	67G
Z_7337183_A	35A	62A
Z_7337184_G	37G	66G
Z_7337185_A	36A	65A
Z_7337186_G	36G	64G
Z_7337187_A	36A	64A
Z_7337188_G	34G	63G
Z_7337189_A	37A	63A
Z_7337190_G	37G	62G
Z_7337191_A	38A	61A
Z_7337192_G	38G	62G
Z_7337193_A	38A	63A
Z_7337194_G	39G	63G
Z_7337195_A	39A	62A
Z_7337196_G	39G	59G
Z_7337197_A	40A	60A
Z_7337198_G	40G	60G
Z_7337199_A	38A	60A
Z_7337200_G	37G	59G
Z_7337201_A	34A	61A
Z_7337202_C	34C	61C
Z_7337203_C	32C	62C
Z_7337204_C	33C	60C
Z_7337205_A	31A	60A
Z_7337206_A	32A	60A
Z_7337207_G	31G	60G
Z_7337208_C	30C	60C
Z_7337209_A	30A	60A
Z_7337210_C	30C	59C
Z_7337211_G	31G	59G
Z_7337212_G	30G	58G
Z_7337213_C	29C	59C
Z_7337214_T	30T	61T

Z_7337215_G	30G	62G
Z_7337216_C	30C	61C
Z_7337217_G	29G	63G
Z_7337218_A	28A	62A
Z_7337219_A	28A	60A
Z_7337220_G	28G	61G
Z_7337221_A	28A	62A
Z_7337222_C	28C	61C
Z_7337223_T	27T	62T
Z_7337224_C	29C	60C
Z_7337225_C	27C	62C
Z_7337226_C	28C	64C
Z_7337227_T	30T	64T
Z_7337228_C	29C	63C
Z_7337229_C	30C	64C
Z_7337230_G	30G	64G
Z_7337231_A	29A	65A
Z_7337232_T	26T	65T
Z_7337233_G	26G	65G
Z_7337234_A	25A	66A
Z_7337235_C	26C	64C
Z_7337236_A	25A	68A
Z_7337237_A	24A	70A
Z_7337238_A	26A	68A
Z_7337239_A	28A	67A
Z_7337240_G	29G	69G
Z_7337241_C	27C	69C
Z_7337242_G	27G	70G
Z_7337243_C	27C	68C
Z_7337244_C	29C	69C
Z_7337245_G	29G	69G
Z_7337246_G	27A	36A_31G_1C
Z_7337247_G	27G	67G
Z_7337248_A	27A	66A
Z_7337249_G	27G	65G
Z_7337250_A	26A	65A
Z_7337251_A	27A	67A
Z_7337252_G	28G	67G
Z_7337253_G	28G	67G
Z_7337254_A	28A	67A
Z_7337255_G	28G	68G
Z_7337256_A	29A	68A
Z_7337257_G	29G	69G

Z_7337258_G	28G	71G
Z_7337259_A	25A	65A
Z_7337260_C	25C	64C
Z_7337261_A	25A	65A
Z_7337262_G	24G	64G
Z_7337263_C	25C	64C
Z_7337264_C	24C	63C
Z_7337265_A	24A	64A
Z_7337266_G	25G	63G
Z_7337267_A	25A	64A
Z_7337268_G	25G	63G
Z_7337269_G	26G	63G
Z_7337270_T	26T	59T
Z_7337271_G	27G	57G
Z_7337272_G	27G	57G
Z_7337273_G	27G	58G
Z_7337274_A	27A	57A
Z_7337275_G	27G	57G
Z_7337276_G	26G	57G
Z_7337277_G	25G	56G
Z_7337278_G	25G	56G
Z_7337279_G	25G	57G
Z_7337280_A	26A	57A
Z_7337281_C	25C	57C
Z_7337282_C	27C	57C
Z_7337283_G	28G	57G
Z_7337284_T	29T	58T
Z_7337285_T	30T	58T
Z_7337286_T	30T	58T
Z_7337287_T	29T	57T
Z_7337288_T	29T	56T
Z_7337289_C	29C	57C
Z_7337290_T	28T	60T
Z_7337291_A	28A	63A
Z_7337292_A	29A	61A
Z_7337293_G	30G	62G
Z_7337294_C	31C	62C
Z_7337295_C	31C	62C
Z_7337296_C	29C	62C
Z_7337297_C	28C	65C
Z_7337298_T	27T	65T
Z_7337299_C	27C	65C
Z_7337300_A	28A	65A

Z_7337301_A	28A	64A
Z_7337302_G	28G	65G
Z_7337303_T	28T	64T
Z_7337304_G	28G	64G
Z_7337305_T	26T	64T
Z_7337306_T	25T	64T
Z_7337307_C	27C	64C
Z_7337308_T	27T	66T
Z_7337309_T	25T	64T
Z_7337310_T	25T	63T
Z_7337311_G	26G	61G
Z_7337312_C	26C	61C
Z_7337313_T	27T	62T
Z_7337314_T	26T	62T
Z_7337315_T	26T	63T
Z_7337316_C	25C	63C
Z_7337317_A	25A	63A
Z_7337318_G	25G	62G
Z_7337319_C	25C	62C
Z_7337320_G	25G	60G
Z_7337321_C	24C	62C
Z_7337322_C	25C	63C
Z_7337323_G	25G	63G
Z_7337324_C	25C	63C
Z_7337325_T	25T	59T
Z_7337326_T	25T	62T
Z_7337327_T	26T	62T
Z_7337328_T	27T	62T
Z_7337329_G	28G	61G
Z_7337330_T	29T	61T
Z_7337331_T	31T	61T
Z_7337332_T	30T	58T
Z_7337333_G	31G	60G
Z_7337334_C	32C	61C
Z_7337335_C	32C	60C
Z_7337336_G	31G	61G
Z_7337337_G	32G	60G
Z_7337338_A	32A	60A
Z_7337339_G	30G	60G
Z_7337340_G	31G	60G
Z_7337341_C	31C	61C
Z_7337342_T	31T	62T
Z_7337343_T	29T	63T

Z_7337344_C	31C	62C
Z_7337345_T	32T	63T
Z_7337346_G	31G	61G
Z_7337347_A	33A	60A
Z_7337348_T	33T	61T
Z_7337349_A	31A	61A
Z_7337350_A	32A	61A
Z_7337351_A	30A	61A
Z_7337352_T	29T	60T
Z_7337353_T	29T	59T
Z_7337354_G	29G	60G
Z_7337355_C	29C	60C
Z_7337356_A	28A	62A
Z_7337357_A	29A	62A
Z_7337358_C	30C	64C
Z_7337359_C	31C	64C
Z_7337360_G	33G	65G
Z_7337361_C	34C	65C
Z_7337362_T	33T	66T
Z_7337363_T	30T	66T
Z_7337364_A	32A	65A
Z_7337365_A	32A	65A
Z_7337366_G	31G	65G
Z_7337367_G	32G	64G
Z_7337368_G	31G	63G
Z_7337369_A	32A	65A
Z_7337370_A	30A	67A
Z_7337371_C	30C	67C
Z_7337372_G	31G	68G
Z_7337373_C	32C	67C
Z_7337374_G	32G	67G
Z_7337375_A	33A	66A
Z_7337376_G	32G	67G
Z_7337377_G	31G	68G
Z_7337378_C	31C	68C
Z_7337379_G	30G	67G
Z_7337380_C	32C	68C
Z_7337381_A	31A	69A
Z_7337382_T	31T	71T
Z_7337383_A	31A	74A
Z_7337384_A	32A	73A
Z_7337385_A	32A	71A
Z_7337386_A	32A	67A

Z_7337387_G	32G	69G
Z_7337388_C	30C	70C
Z_7337389_C	31C	70C
Z_7337390_G	30G	69G
Z_7337391_C	30C	67C
Z_7337392_T	31T	64T
Z_7337393_C	30C	63C
Z_7337394_T	32T	63T
Z_7337395_T	32T	63T
Z_7337396_G	31G	63G
Z_7337397_C	29C	62C
Z_7337398_T	29T	62T
Z_7337399_C	26C	64C
Z_7337400_C	26C	64C
Z_7337401_C	23C	64C
Z_7337402_C	24C	64C
Z_7337403_C	23C	62C
Z_7337404_C	22C	62C
Z_7337405_T	21T	61T
Z_7337406_C	22C	62C
Z_7337407_C	20C	60C
Z_7337408_C	18C	59C
Z_7337409_C	22C	59C
Z_7337410_T	22T	59T
Z_7337411_C	21C	60C
Z_7337412_G	21G	27A_33G
Z_7337413_G	22G	61G
Z_7337414_A	21A	61A
Z_7337415_G	21G	61G
Z_7337416_T	21T	58T
Z_7337417_G	22G	58G
Z_7337418_G	22G	57G
Z_7337419_T	24T	55T
Z_7337420_T	24T	57T
Z_7337421_G	25G	57G
Z_7337422_T	25T	58T
Z_7337423_G	25G	61G
Z_7337424_T	24T	62T
Z_7337425_G	22G	63G
Z_7337426_A	22A	60A
Z_7337427_A	22A	62A
Z_7337428_T	22T	61T
Z_7337429_G	22G	59G

Z_7337430_G	22G	59G
Z_7337431_A	21A	56A
Z_7337432_A	21A	58A
Z_7337433_G	22G	57G
Z_7337434_T	20T	58T
Z_7337435_C	19C	58C
Z_7337436_A	20A	58A
Z_7337437_G	19G	57G
Z_7337438_G	18G	55G
Z_7337439_C	18C	55C
Z_7337440_G	18G	55G
Z_7337441_G	17G	58G
Z_7337442_G	18G	58G
Z_7337443_C	17C	58C
Z_7337444_G	17G	58G
Z_7337445_C	16C	58C
Z_7337446_A	16A	58A
Z_7337447_C	16C	57C
Z_7337448_G	14G	60G
Z_7337449_G	15G	61G
Z_7337450_G	14G	62G
Z_7337451_A	14A	57A
Z_7337452_G	13G	58G
Z_7337453_G	12G	57G
Z_7337454_C	13C	59C
Z_7337455_A	12A	58A
Z_7337456_G	14G	56G
Z_7337457_G	14G	54G
Z_7337458_A	13A	54A
Z_7337459_G	13G	55G
Z_7337460_G	13G	55G
Z_7337461_G	11G	56G
Z_7337462_G	13G	55G
Z_7337463_G	13G	55G
Z_7337464_T	12T	55T
Z_7337465_C	12C	54C
Z_7337466_A	12A	55A
Z_7337467_A	12A	53A
Z_7337468_T	12T	54T
Z_7337469_C	12C	53C
Z_7337470_C	13C	56C
Z_7337471_A	14A	57A
Z_7337472_T	15T	57T

Z_7337473_T	15T	58T
Z_7337474_A	16A	58A
Z_7337475_A	18A	57A
Z_7337476_G	18G	56G
Z_7337477_G	17G	54G
Z_7337478_T	18T	51T
Z_7337479_T	17T	52T
Z_7337480_C	17C	50C
Z_7337481_T	17T	50T
Z_7337482_A	15A	50A
Z_7337483_T	16T	51T
Z_7337484_T	16T	53T
Z_7337485_A	16A	54A
Z_7337486_A	16A	55A
Z_7337487_G	14G	57G
Z_7337488_T	15T	61T
Z_7337489_C	13C	63C
Z_7337490_C	15C	64C
Z_7337491_T	15T	64T
Z_7337492_C	14C	64C
Z_7337493_C	15C	64C
Z_7337494_T	15T	65T
Z_7337495_A	16A	66A
Z_7337496_G	15G	67G
Z_7337497_A	16A	68A
Z_7337498_G	16G	68G
Z_7337499_A	17A	68A
Z_7337500_A	15A	67A
Z_7337501_C	16C	69C
Z_7337502_A	16A	69A
Z_7337503_A	15A	69A
Z_7337504_A	16A	69A
Z_7337505_G	16G	67G
Z_7337506_C	17C	67C
Z_7337507_T	18T	67T
Z_7337508_A	19A	66A
Z_7337509_A	19A	66A
Z_7337510_C	19C	69C
Z_7337511_C	19C	69C
Z_7337512_T	19T	69T
Z_7337513_G	19G	69G
Z_7337514_T	19T	69T
Z_7337515_G	19G	69G

Z_7337516_C	19C	70C
Z_7337517_C	20C	69C
Z_7337518_A	21A	68A
Z_7337519_T	22T	68T
Z_7337520_C	22C	67C
Z_7337521_C	23C	67C
Z_7337522_T	24T	67T
Z_7337523_C	24C	66C
Z_7337524_A	24A	65A
Z_7337525_T	24T	66T
Z_7337526_A	24A	66A
Z_7337527_A	24A	66A
Z_7337528_A	24A	66A
Z_7337529_T	25T	65T
Z_7337530_A	25A	65A
Z_7337531_T	26T	66T
Z_7337532_T	26T	66T
Z_7337533_G	27G	67G
Z_7337534_C	27C	66C
Z_7337535_T	28T	66T
Z_7337536_G	28G	64G
Z_7337537_C	28C	65C
Z_7337538_A	28A	64A
Z_7337539_T	29T	66T
Z_7337540_G	28G	66G
Z_7337541_G	27G	67G
Z_7337542_A	27A	67A
Z_7337543_G	27G	63G
Z_7337544_G	26G	61G
Z_7337545_A	24A	60A
Z_7337546_G	24G	60G
Z_7337547_C	24C	60C
Z_7337548_C	24C	60C
Z_7337549_C	24C	57C
Z_7337550_C	24C	57C
Z_7337551_G	24G	57G
Z_7337552_A	23A	56A
Z_7337553_A	24A	57A
Z_7337554_T	24T	56T
Z_7337555_T	23T	56T
Z_7337556_T	23T	55T
Z_7337557_T	23T	55T
Z_7337558_A	24A	55A

Z_7337559_A	24A	54A
Z_7337560_T	23T	54T
Z_7337561_T	22T	53T
Z_7337562_A	22A	54A
Z_7337563_A	22A	54A
Z_7337564_A	21A	54A
Z_7337565_C	21C	52C
Z_7337566_C	21C	51C
Z_7337567_T	22T	50T
Z_7337568_A	22C	31A_18C
Z_7337569_T	21T	49T
Z_7337570_T	21T	49T
Z_7337571_T	20T	51T
Z_7337572_A	20A	51A
Z_7337573_C	20C	51C
Z_7337574_C	19C	51C
Z_7337575_T	19T	51T
Z_7337576_G	18G	51G
Z_7337577_C	16C	51C
Z_7337578_T	16T	49T
Z_7337579_C	17C	49C
Z_7337580_A	18A	48A
Z_7337581_G	18G	48G
Z_7337582_G	18G	45G
Z_7337583_A	19A	42A
Z_7337584_G	18G	39G
Z_7337585_A	20A	38A
Z_7337586_T	20T	37T
Z_7337587_A	19A	38A
Z_7337588_G	19G	37G
Z_7337589_A	18A	36A
Z_7337590_G	17G	35G
Z_7337591_G	18G	34G
Z_7337592_G	16G	34G
Z_7337593_G	17G	35G
Z_7337594_G	17G	35G
Z_7337595_G	17G	36G
Z_7337596_A	15A	34A
Z_7337597_G	16G	35G
Z_7337598_G	16G	35G
Z_7337599_A	15A	35A
Z_7337600_G	15G	35G
Z_7337601_A	14A	34A

Z_7337602_G	13G	34G
Z_7337603_A	13A	35A
Z_7337604_A	15A_6G	37A
Z_7337605_G	12G	34G
Z_7337606_A	12A	35A
Z_7337607_A	21A_31G	36A
Z_7337608_G	12G	36G
Z_7337609_G	11G	37G
Z_7337610_A	11A	37A
Z_7337611_G	12G	36G
Z_7337612_G	10G	38G
Z_7337613_G	11G	38G
Z_7337614_A	11A	37A
Z_7337615_G	13G	36G
Z_7337616_G	13G	35G
Z_7337617_G	13G	35G
Z_7337618_A	13A	34A
Z_7337619_G	13G	35G
Z_7337620_G	13G	35G
Z_7337621_G	13G	35G
Z_7337622_A	13A	36A
Z_7337623_G	13G	37G
Z_7337624_G	13G	39G
Z_7337625_G	13G	39G
Z_7337626_A	13A	39A
Z_7337627_G	13G	39G
Z_7337628_A	9A	40A
Z_7337629_G	10G	41G
Z_7337630_A	10A	41A
Z_7337631_A	10A	41A
Z_7337632_A	11A	41A
Z_7337633_T	11T	41T
Z_7337634_C	11C	40C
Z_7337635_T	11T	39T
Z_7337636_A	13A	38A
Z_7337637_T	14T	38T
Z_7337638_T	14T	38T
Z_7337639_T	15T	38T
Z_7337640_A	16A	39A
Z_7337641_G	15G	39G
Z_7337642_A	14A	40A
Z_7337643_C	14C	40C
Z_7337644_T	13T	41T

Z_7337645_T	13T	41T
Z_7337646_G	13G	42G
Z_7337647_A	12A	45A
Z_7337648_A	12A	45A
Z_7337649_C	12C	45C
Z_7337650_G	11G	46G
Z_7337651_T	12T	45T
Z_7337652_C	12C	46C
Z_7337653_G	12G	46G
Z_7337654_G	13G	46G
Z_7337655_T	13T	46T
Z_7337656_C	13C	48C
Z_7337657_A	13A	47A
Z_7337658_G	14G	47G
Z_7337659_C	15C	47C
Z_7337660_T	15T	48T
Z_7337661_T	15T	48T
Z_7337662_C	16C	48C
Z_7337663_T	18T	48T
Z_7337664_T	18T	49T
Z_7337665_G	18G	49G
Z_7337666_T	18T	52T
Z_7337667_G	19G	52G
Z_7337668_C	20C	52C
Z_7337669_T	18T	51T
Z_7337670_C	20C	51C
Z_7337671_A	20A	53A
Z_7337672_C	20C	54C
Z_7337673_T	20T	52T
Z_7337674_C	20C	56C
Z_7337675_A	21A	57A
Z_7337676_C	21C	57C
Z_7337677_A	21A	57A
Z_7337678_C	24C	56C
Z_7337679_A	24A	58A
Z_7337680_C	23C	58C
Z_7337681_A	24A	58A
Z_7337682_C	25C	56C
Z_7337683_A	25A	59A
Z_7337684_C	23C	64C
Z_7337685_A	22A	59A
Z_7337686_C	23C	59C
Z_7337687_A	23A	60A

Z_7337688_C	23C	58C
Z_7337689_A	23A	57A
Z_7337690_C	15A_8C	15A_41C
Z_7337691_A	23A	58A
Z_7337692_C	23C	57C
Z_7337693_A	24A	57A
Z_7337694_C	7A_17C	59C
Z_7337695_A	24A	58A
Z_7337696_C	25C	57C
Z_7337697_A	25A	58A
Z_7337698_C	25C	57C
Z_7337699_A	25A	54A
Z_7337700_C	24C	55C
Z_7337701_A	23A	53A
Z_7337702_C	25C	53C
Z_7337703_A	27A	52A
Z_7337704_C	5A_5G_33C	51C
Z_7337705_A	23A	50A
Z_7337706_C	22C	50C
Z_7337707_G	17A_1T_4G_3C	17A_31G_1C
Z_7337708_C	21C	16A_56C
Z_7337709_A	20A	40A
Z_7337710_C	20C	41C
Z_7337711_A	20A	39A
Z_7337712_C	20C	19A_20T_9G_58C
Z_7337713_G	19G	48G
Z_7337714_T	19T	48T
Z_7337715_G	21G	48G
Z_7337716_T	22T	47T
Z_7337717_G	22G	47G
Z_7337718_T	23T	45T
Z_7337719_G	24G_19C	45G
Z_7337720_C	5T	46C
Z_7337721_A	26A	46A
Z_7337722_T	27T	48T
Z_7337723_G	27G	48G
Z_7337724_T	27T	48T
Z_7337725_G	28G	47G
Z_7337726_T	28T	48T
Z_7337727_G	28G	49G
Z_7337728_T	28T	49T
Z_7337729_G	27G	47G
Z_7337730_C	24T	23T_25C

Z_7337731_G	25G	45G
Z_7337732_C	24T	48C
Z_7337733_G	25G	48G
Z_7337734_C	27C	47C
Z_7337735_G	27G	46G
Z_7337736_C	27C	46C
Z_7337737_A	27A	46A
Z_7337738_C	30C	46C
Z_7337739_G	29G	49G
Z_7337740_C	30C	50C
Z_7337741_A	28A	50A
Z_7337742_A	28A	51A
Z_7337743_C	26C	52C
Z_7337744_A	27A	53A
Z_7337745_G	25G	53G
Z_7337746_C	25C	54C
Z_7337747_A	24A	54A
Z_7337748_A	25A	54A
Z_7337749_T	25T	54T
Z_7337750_G	25G	54G
Z_7337751_T	25T	53T
Z_7337752_A	25A	54A
Z_7337753_C	25C	56C
Z_7337754_T	25T	56T
Z_7337755_G	25G	58G
Z_7337756_G	25G	58G
Z_7337757_G	25G	59G
Z_7337758_A	25A	60A
Z_7337759_T	25T	59T
Z_7337760_T	26T	60T
Z_7337761_G	26G	60G
Z_7337762_T	26T	58T
Z_7337763_G	27G	58G
Z_7337764_C	27C	58C
Z_7337765_A	27A	31A_26C
Z_7337766_A	26A	57A
Z_7337767_C	29C	56C
Z_7337768_A	28A	57A
Z_7337769_G	29G	56G
Z_7337770_C	29C	57C
Z_7337771_C	28C	56C
Z_7337772_A	29A	57A
Z_7337773_G	28G	59G

Z_7337774_G	26G	57G
Z_7337775_A	24A	57A
Z_7337776_A	23A	57A
Z_7337777_A	23A	58A
Z_7337778_G	22G	58G
Z_7337779_A	22A	58A
Z_7337780_G	22G	28A_30G
Z_7337781_G	22G	58G
Z_7337782_T	23T	56T
Z_7337783_G	24G	60G
Z_7337784_G	23G	60G
Z_7337785_T	24T	58T
Z_7337786_A	23A	58A
Z_7337787_T	25T	58T
Z_7337788_G	24G	60G
Z_7337789_G	22G	59G
Z_7337790_G	25G	57G
Z_7337791_G	24G	57G
Z_7337792_A	24A	59A
Z_7337793_G	25G	59G
Z_7337794_A	26A	59A
Z_7337795_G	27G	59G
Z_7337796_G	29G	58G
Z_7337797_T	28T	59T
Z_7337798_G	29G	59G
Z_7337799_C	27A	59C
Z_7337800_A	30A	59A
Z_7337801_A	30A	59A
Z_7337802_A	30A	58A
Z_7337803_T	31T	57T
Z_7337804_T	30T	57T
Z_7337805_C	30C	56C
Z_7337806_T	31T	56T
Z_7337807_C	31C	54C
Z_7337808_T	28T	55T
Z_7337809_A	28A	55A
Z_7337810_G	28G	55G
Z_7337811_T	27T	55T
Z_7337812_T	27T	56T
Z_7337813_A	27A	56A
Z_7337814_T	27T	56T
Z_7337815_T	27T	55T
Z_7337816_G	27G	55G

Z_7337817_G	29G	53G
Z_7337818_C	30C	53C
Z_7337819_A	33A	54A
Z_7337820_A	35A	55A
Z_7337821_T	35T	57T
Z_7337822_T	37T	57T
Z_7337823_A	40A	58A
Z_7337824_C	39C	60C
Z_7337825_A	40A	60A
Z_7337826_G	40G	61G
Z_7337827_C	40C	30A_34C
Z_7337828_C	41C	64C
Z_7337829_A	42A	64A
Z_7337830_A	41A	65A
Z_7337831_C	40C	65C
Z_7337832_A	41A	66A
Z_7337833_C	41C	67C
Z_7337834_A	42A	65A
Z_7337835_C	41C	65C
Z_7337836_A	41A	65A
Z_7337837_A	39A	62A
Z_7337838_T	42T	62T
Z_7337839_A	41A	62A
Z_7337840_A	41A	61A
Z_7337841_A	42A	61A
Z_7337842_G	42G	60G
Z_7337843_G	42G	61G
Z_7337844_C	41C	61C
Z_7337845_A	42A	32A_27G
Z_7337846_A	43A	59A
Z_7337847_C	42C	58C
Z_7337848_A	44A	57A
Z_7337849_G	44G	26A_31G
Z_7337850_T	44T	57T
Z_7337851_T	42T	60T
Z_7337852_C	42C	61C
Z_7337853_T	42T	59T
Z_7337854_C	42C	59C
Z_7337855_A	41A	61A
Z_7337856_G	41G	62G
Z_7337857_C	41C	62C
Z_7337858_A	41A	60A
Z_7337859_C	40C	62C

Z_7337860_C	40C	61C
Z_7337861_A	40G	32A_30G
Z_7337862_G	41G	63G
Z_7337863_A	42A	63A
Z_7337864_G	42G	65G
Z_7337865_G	41G	64G
Z_7337866_C	40C	64C
Z_7337867_T	40T	64T
Z_7337868_G	39G	62G
Z_7337869_T	39T	62T
Z_7337870_G	37G	62G
Z_7337871_A	38A	61A
Z_7337872_A	39A	62A
Z_7337873_G	39G	61G
Z_7337874_A	38A	61A
Z_7337875_T	38T	62T
Z_7337876_C	38C	63C
Z_7337877_A	39A	63A
Z_7337878_A	41A	65A
Z_7337879_A	40A	65A
Z_7337880_T	41T	66T
Z_7337881_C	41C	68C
Z_7337882_A	41A	66A
Z_7337883_C	42C	65C
Z_7337884_T	42T	64T
Z_7337885_T	42T	65T
Z_7337886_C	41C	66C
Z_7337887_C	39C	65C
Z_7337888_T	39T	64T
Z_7337889_G	35G	66G
Z_7337890_G	33G	64G
Z_7337891_T	34T	64T
Z_7337892_G	30G	64G
Z_7337893_C	30C	63C
Z_7337894_A	31A	64A
Z_7337895_C	29C	64C
Z_7337896_C	28C	64C
Z_7337897_A	29A	65A
Z_7337898_A	32A	65A
Z_7337899_G	30G	65G
Z_7337900_T	31T	65T
Z_7337901_C	29C	68C
Z_7337902_A	31A	69A

Z_7337903_G	30G	68G
Z_7337904_G	28G	70G
Z_7337905_T	29T	70T
Z_7337906_G	28G	70G
Z_7337907_G	31G	68G
Z_7337908_A	30A	69A
Z_7337909_G	30G	69G
Z_7337910_G	28G	69G
Z_7337911_G	30G	70G
Z_7337912_G	32G	70G
Z_7337913_G	31G	70G
Z_7337914_C	32C	70C
Z_7337915_C	32C	72C
Z_7337916_A	31A	70A
Z_7337917_T	33T	70T
Z_7337918_G	30G	69G
Z_7337919_G	32G	68G
Z_7337920_G	31G	68G
Z_7337921_C	33C	69C
Z_7337922_C	34C	66C
Z_7337923_A	34A	66A
Z_7337924_T	35T	66T
Z_7337925_G	35G	66G
Z_7337926_A	35A	65A
Z_7337927_G	34G	66G
Z_7337928_C	35C	66C
Z_7337929_A	34A	66A
Z_7337930_C	35C	66C
Z_7337931_C	33C	65C
Z_7337932_A	33A	65A
Z_7337933_G	32G	66G
Z_7337934_T	31T	65T
Z_7337935_T	30T	66T
Z_7337936_G	31G	65G
Z_7337937_C	32C	65C
Z_7337938_A	32A	64A
Z_7337939_G	31G	64G
Z_7337940_C	32C	64C
Z_7337941_C	32C	63C
Z_7337942_T	31T	62T
Z_7337943_C	30C	64C
Z_7337944_C	30C	62C
Z_7337945_T	30T	63T

Z_7337946_T	29T	60T
Z_7337947_T	30T	59T
Z_7337948_G	30G	59G
Z_7337949_G	28G	59G
Z_7337950_T	30T	57T
Z_7337951_T	29T	56T
Z_7337952_T	30T	57T
Z_7337953_T	30T	57T
Z_7337954_C	30C	56C
Z_7337955_T	30T	57T
Z_7337956_C	31C	58C
Z_7337957_T	31T	58T
Z_7337958_G	31G	58G
Z_7337959_G	31G	56G
Z_7337960_G	31G	56G
Z_7337961_A	32A	57A
Z_7337962_A	31A	56A
Z_7337963_G	31G	56G
Z_7337964_G	30G	56G
Z_7337965_T	30T	56T
Z_7337966_C	32C	57C
Z_7337967_T	33T	56T
Z_7337968_G	31G	56G
Z_7337969_G	32G	56G
Z_7337970_T	32T	56T
Z_7337971_G	32G	56G
Z_7337972_A	31A	56A
Z_7337973_A	34A	51A
Z_7337974_A	35A	51A
Z_7337975_G	36G	50G
Z_7337976_A	36A	48A
Z_7337977_A	34A	49A
Z_7337978_G	34G	49G
Z_7337979_A	33A	50A
Z_7337980_A	34A	48A
Z_7337981_G	32G	48G
Z_7337982_A	30A	48A
Z_7337983_A	32A	49A
Z_7337984_C	32C	48C
Z_7337985_C	32C	48C
Z_7337986_A	33A	48A
Z_7337987_C	31C	49C
Z_7337988_C	30C	51C

Z_7337989_A	31A	51A
Z_7337990_T	32T	53T
Z_7337991_C	33C	54C
Z_7337992_A	33A	54A
Z_7337993_C	32C	56C
Z_7337994_C	33C	56C
Z_7337995_A	35A	55A
Z_7337996_G	36G	54G
Z_7337997_G	36G	53G
Z_7337998_T	37T	56T
Z_7337999_G	36G	56G
Z_7338000_G	36G	56G
Z_7338001_A	37A	56A
Z_7338002_T	36T	57T
Z_7338003_G	36G	57G
Z_7338004_G	38G	58G
Z_7338005_A	39A	58A
Z_7338006_A	40A	58A
Z_7338007_A	39G	31A_27G
Z_7338008_C	40C	62C
Z_7338009_T	41T	63T
Z_7338010_T	41T	61T
Z_7338011_T	39T	60T
Z_7338012_C	40C	60C
Z_7338013_T	41T	60T
Z_7338014_A	42A	59A
Z_7338015_A	43A	60A
Z_7338016_C	43C	58C
Z_7338017_A	43A	58A
Z_7338018_C	43C	59C
Z_7338019_A	43A	60A
Z_7338020_T	43T	58T
Z_7338021_G	43G	59G
Z_7338022_C	44C	58C
Z_7338023_A	43A	58A
Z_7338024_C	44C	55C
Z_7338025_A	44A	55A
Z_7338026_T	45T	55T
Z_7338027_G	45G	55G
Z_7338028_C	43C	53C
Z_7338029_A	43A	52A
Z_7338030_T	44T	50T
Z_7338031_C	44C	50C

Z_7338032_C	45C	50C
Z_7338033_A	46A	51A
Z_7338034_T	47T	51T
Z_7338035_G	46G	52G
Z_7338036_A	48A	52A
Z_7338037_A	47A	52A
Z_7338038_G	47G	51G
Z_7338039_G	46G	51G
Z_7338040_G	47G	52G
Z_7338041_A	46A	52A
Z_7338042_A	46A	51A
Z_7338043_A	46A	51A
Z_7338044_A	45A	51A
Z_7338045_G	44G	50G
Z_7338046_A	46A	51A
Z_7338047_A	46A	51A
Z_7338048_C	45C	54C
Z_7338049_T	46T	54T
Z_7338050_G	46G	53G
Z_7338051_T	48T	53T
Z_7338052_C	49C	52C
Z_7338053_A	48A	51A
Z_7338054_C	48C	51C
Z_7338055_T	47T	52T
Z_7338056_A	47A	52A
Z_7338057_A	48A	52A
Z_7338058_A	47A	52A
Z_7338059_T	47T	53T
Z_7338060_T	45T	54T
Z_7338061_T	45T	53T
Z_7338062_C	45C	55C
Z_7338063_T	45T	55T
Z_7338064_T	43T	56T
Z_7338065_G	43G	54G
Z_7338066_A	40A	54A
Z_7338067_T	41T	55T
Z_7338068_C	40C	55C
Z_7338069_T	41T	55T
Z_7338070_T	41T	56T
Z_7338071_G	41G	57G
Z_7338072_A	40A	56A
Z_7338073_G	39G	55G
Z_7338074_T	41T	56T

Z_7338075_G	42G	56G
Z_7338076_T	42T	56T
Z_7338077_G	40G	55G
Z_7338078_A	40A	56A
Z_7338079_G	32A_40G_1C	14A_56G
Z_7338080_A	42A	58A
Z_7338081_A	43A	60A
Z_7338082_A	42A	60A
Z_7338083_A	42A	58A
Z_7338084_A	43A	58A
Z_7338085_A	41A	56A
Z_7338086_A	42A	57A
Z_7338087_A	41A	57A
Z_7338088_A	43A	55A
Z_7338089_A	42A	57A
Z_7338090_C	41C	57C
Z_7338091_A	41A	56A
Z_7338092_C	41C	57C
Z_7338093_A	41A	54A
Z_7338094_T	40T	52T
Z_7338095_G	39G	53G
Z_7338096_G	39G	55G
Z_7338097_A	41A	53A
Z_7338098_T	41T	52T
Z_7338099_T	41T	52T
Z_7338100_T	41T	52T
Z_7338101_T	41T	51T
Z_7338102_C	40C	50C
Z_7338103_T	39T	49T
Z_7338104_T	39T	48T
Z_7338105_T	37T	49T
Z_7338106_C	36C	48C
Z_7338107_C	38C	49C
Z_7338108_T	38T	49T
Z_7338109_T	38T	51T
Z_7338110_C	38C	55C
Z_7338111_A	37A	55A
Z_7338112_G	36G	56G
Z_7338113_C	37C	55C
Z_7338114_G	37G	55G
Z_7338115_C	36C	55C
Z_7338116_A	38A	55A
Z_7338117_A	38A	55A

Z_7338118_G	37G	54G
Z_7338119_A	37A	54A
Z_7338120_G	36G	56G
Z_7338121_T	36T	58T
Z_7338122_G	36G	59G
Z_7338123_A	36A	60A
Z_7338124_G	37G	60G
Z_7338125_A	36A	60A
Z_7338126_C	37C	60C
Z_7338127_A	37A	63A
Z_7338128_A	36A	62A
Z_7338129_G	36G	62G
Z_7338130_G	36G	65G
Z_7338131_A	36A	66A
Z_7338132_G	36G	67G
Z_7338133_C	36C	67C
Z_7338134_A	35A	67A
Z_7338135_A	35A	67A
Z_7338136_C	34C	68C
Z_7338137_T	34T	67T
Z_7338138_C	34C	71C
Z_7338139_T	33T	72T
Z_7338140_T	33T	72T
Z_7338141_G	33G	71G
Z_7338142_C	34C	69C
Z_7338143_T	30T	66T
Z_7338144_C	31C	68C
Z_7338145_C	29C	69C
Z_7338146_A	29A	68A
Z_7338147_G	28G	68G
Z_7338148_T	27T	68T
Z_7338149_C	28C	68C
Z_7338150_T	28T	66T
Z_7338151_C	26C	67C
Z_7338152_T	28T	69T
Z_7338153_G	28G	69G
Z_7338154_C	26C	70C
Z_7338155_A	28A	70A
Z_7338156_A	28A	70A
Z_7338157_T	28T	69T
Z_7338158_G	27G	69G
Z_7338159_C	29C	68C
Z_7338160_C	30C	68C

Z_7338161_T	31T	67T
Z_7338162_T	31T	67T
Z_7338163_T	30T	68T
Z_7338164_G	30G	67G
Z_7338165_C	32C	67C
Z_7338166_A	31A	66A
Z_7338167_C	30C	66C
Z_7338168_A	31A	66A
Z_7338169_A	32A	67A
Z_7338170_A	33A	68A
Z_7338171_A	32A	70A
Z_7338172_C	33C	70C
Z_7338173_A	32A	68A
Z_7338174_T	32T	69T
Z_7338175_G	33G	69G
Z_7338176_A	35A	68A
Z_7338177_A	35A	65A
Z_7338178_C	35C	67C
Z_7338179_C	37C	67C
Z_7338180_C	38C	68C
Z_7338181_T	38T	67T
Z_7338182_T	37T	66T
Z_7338183_T	36T	67T
Z_7338184_C	36C	36T_29C
Z_7338185_C	36C	66C
Z_7338186_T	33T	66T
Z_7338187_C	34C	67C
Z_7338188_A	34A	67A
Z_7338189_G	34G	67G
Z_7338190_A	32A	68A
Z_7338191_G	34G	68G
Z_7338192_G	34G	67G
Z_7338193_A	33A	68A
Z_7338194_G	33G	69G
Z_7338195_C	33C	69C
Z_7338196_A	32A	68A
Z_7338197_A	33A	68A
Z_7338198_A	33A	70A
Z_7338199_G	32G	71G
Z_7338200_C	33C	68C
Z_7338201_A	33A	70A
Z_7338202_C	35C	69C
Z_7338203_A	35A	69A

Z_7338204_C	35C	68C
Z_7338205_A	35A	63A
Z_7338206_A	35A	64A
Z_7338207_G	36G	63G
Z_7338208_G	35G	64G
Z_7338209_G	36G	65G
Z_7338210_C	35C	67C
Z_7338211_A	36A	67A
Z_7338212_G	33C	38G_29C
Z_7338213_C	36C	67C
Z_7338214_C	36C	67C
Z_7338215_T	36T	64T
Z_7338216_C	38C	63C
Z_7338217_A	39A	62A
Z_7338218_A	39A	62A
Z_7338219_G	38G	63G
Z_7338220_C	40C	65C
Z_7338221_A	41A	65A
Z_7338222_G	40G	64G
Z_7338223_A	40A	65A
Z_7338224_G	40G	64G
Z_7338225_A	38A	62A
Z_7338226_A	39A	63A
Z_7338227_A	39A	62A
Z_7338228_G	39G	61G
Z_7338229_G	35G	62G
Z_7338230_C	35C	62C
Z_7338231_C	36C	64C
Z_7338232_G	37A	28A_37G
Z_7338233_G	38G	64G
Z_7338234_G	38G	62G
Z_7338235_T	36T	62T
Z_7338236_G	36G	63G
Z_7338237_A	37A	64A
Z_7338238_T	36T	66T
Z_7338239_T	35T	68T
Z_7338240_T	34T	67T
Z_7338241_T	35T	68T
Z_7338242_T	34T	68T
Z_7338243_C	36C	67C
Z_7338244_A	37A	67A
Z_7338245_A	38A	67A
Z_7338246_A	36A	67A

Z_7338247_A	34A	66A
Z_7338248_A	34A	67A
Z_7338249_G	31G	64G
Z_7338250_G	32T	26T_38G
Z_7338251_T	33T	63T
Z_7338252_G	34G	64G
Z_7338253_C	36C	65C
Z_7338254_A	37A	66A
Z_7338255_A	36A	66A
Z_7338256_G	37G	67G
Z_7338257_C	36C	68C
Z_7338258_C	36C	68C
Z_7338259_A	36A	70A
Z_7338260_C	35C	70C
Z_7338261_C	34C	72C
Z_7338262_T	35T	71T
Z_7338263_G	37G	72G
Z_7338264_G	38T	28T_43G
Z_7338265_C	38C	71C
Z_7338266_A	38A	69A
Z_7338267_A	38A	70A
Z_7338268_A	40A	69A
Z_7338269_C	40C	69C
Z_7338270_A	39A	68A
Z_7338271_C	39T	1A_32T_37C
Z_7338272_A	37A	72A
Z_7338273_C	38C	71C
Z_7338274_T	38T	72T
Z_7338275_T	38T	72T
Z_7338276_A	38A	72A
Z_7338277_A	37A	72A
Z_7338278_T	38T	71T
Z_7338279_C	39C	72C
Z_7338280_C	38C	71C
Z_7338281_T	38T	72T
Z_7338282_T	38T	72T
Z_7338283_A	37A	73A
Z_7338284_A	37A	74A
Z_7338285_A	37A	72A
Z_7338286_A	35A	73A
Z_7338287_G	34G	73G
Z_7338288_T	34T	73T
Z_7338289_C	34C	74C

Z_7338290_C	31C	73C
Z_7338291_A	33A	74A
Z_7338292_T	33T	73T
Z_7338293_G	35G	71G
Z_7338294_C	34C	70C
Z_7338295_A	35A	69A
Z_7338296_A	35A	69A
Z_7338297_C	34C	70C
Z_7338298_G	33G	70G
Z_7338299_A	32A	70A
Z_7338300_A	34A	72A
Z_7338301_A	34A	71A
Z_7338302_C	32C	71C
Z_7338303_A	32A	71A
Z_7338304_G	32G	70G
Z_7338305_C	37C	70C
Z_7338306_A	37A	71A
Z_7338307_G	36G	71G
Z_7338308_C	35C	72C
Z_7338309_C	39C	72C
Z_7338310_A	38A	72A
Z_7338311_C	38C	71C
Z_7338312_T	38T	73T
Z_7338313_G	37G	74G
Z_7338314_T	37T	73T
Z_7338315_A	37A	72A
Z_7338316_A	39A	72A
Z_7338317_T	39T	70T
Z_7338318_T	39T	70T
Z_7338319_T	40T	70T
Z_7338320_T	39T	70T
Z_7338321_T	39T	69T
Z_7338322_C	38C	70C
Z_7338323_A	35A	70A
Z_7338324_C	35C	69C
Z_7338325_G	36G	68G
Z_7338326_A	37A	68A
Z_7338327_G	37G	67G
Z_7338328_T	36T	65T
Z_7338329_A	36A	65A
Z_7338330_C	36C	66C
Z_7338331_C	36C	64C
Z_7338332_T	36T	64T

Z_7338333_T	34T	62T
Z_7338334_G	34G	60G
Z_7338335_G	33G	61G
Z_7338336_A	35A	64A
Z_7338337_T	35T	64T
Z_7338338_T	34T	64T
Z_7338339_T	34T	64T
Z_7338340_A	34A	64A
Z_7338341_G	34G	64G
Z_7338342_C	34C	63C
Z_7338343_A	33A	63A
Z_7338344_C	34C	66C
Z_7338345_C	33C	67C
Z_7338346_A	35A	65A
Z_7338347_C	34C	66C
Z_7338348_G	33G	65G
Z_7338349_G	33G	39G_26C
Z_7338350_T	33T	66T
Z_7338351_C	34C	65C
Z_7338352_A	35A	65A
Z_7338353_A	36A	66A
Z_7338354_A	37A	65A
Z_7338355_A	36A	67A
Z_7338356_T	41T	66T
Z_7338357_T	42T	67T
Z_7338358_A	42A	68A
Z_7338359_A	41A	40A_28G
Z_7338360_T	41T	68T
Z_7338361_A	41A	67A
Z_7338362_G	39G	68G
Z_7338363_G	40G	67G
Z_7338364_G	40G	66G
Z_7338365_A	42A	65A
Z_7338366_G	42G	66G
Z_7338367_C	42C	64C
Z_7338368_T	42T	64T
Z_7338369_T	42T	63T
Z_7338370_G	42G	64G
Z_7338371_C	42T	65C
Z_7338372_G	42G	65G
Z_7338373_A	43A	65A
Z_7338374_G	43G	64G
Z_7338375_A	38A	65A

Z_7338376_G	40G	63G
Z_7338377_A	40A	62A
Z_7338378_G	41G	61G
Z_7338379_T	38T	60T
Z_7338380_G	39G	61G
Z_7338381_G	39G	62G
Z_7338382_G	39G	62G
Z_7338383_A	38A	61A
Z_7338384_A	38A	60A
Z_7338385_G	38G	60G
Z_7338386_A	35A	61A
Z_7338387_A	35A	61A
Z_7338388_G	35G	62G
Z_7338389_A	34A	62A
Z_7338390_T	35T	64T
Z_7338391_A	34A	63A
Z_7338392_A	34A	63A
Z_7338393_C	34C	62C
Z_7338394_C	34C	63C
Z_7338395_A	34A	62A
Z_7338396_T	35T	63T
Z_7338397_G	36G	64G
Z_7338398_C	37C	62C
Z_7338399_C	37C	62C
Z_7338400_T	37T	61T
Z_7338401_A	35A	62A
Z_7338402_G	36G	64G
Z_7338403_C	36C	63C
Z_7338404_T	35T	63T
Z_7338405_T	36T	63T
Z_7338406_T	35T	63T
Z_7338407_T	35T	60T
Z_7338408_A	34A	59A
Z_7338409_T	34T	59T
Z_7338410_T	35T	58T
Z_7338411_G	35G	58G
Z_7338412_G	36G	61G
Z_7338413_T	36T	62T
Z_7338414_G	35G	62G
Z_7338415_G	37G	61G
Z_7338416_C	36C	62C
Z_7338417_A	38A	61A
Z_7338418_G	38G	61G

Z_7338419_G	36G	62G
Z_7338420_A	36A	62A
Z_7338421_A	38A	60A
Z_7338422_T	37T	60T
Z_7338423_A	37A	61A
Z_7338424_C	37C	65C
Z_7338425_C	38C	63C
Z_7338426_T	35T	65T
Z_7338427_T	35T	66T
Z_7338428_T	35T	66T
Z_7338429_A	35A	66A
Z_7338430_T	37T	63T
Z_7338431_G	36G	64G
Z_7338432_T	37T	65T
Z_7338433_T	36T	65T
Z_7338434_C	37C	65C
Z_7338435_T	35T	66T
Z_7338436_G	35G	69G
Z_7338437_A	36A	69A
Z_7338438_G	37G	67G
Z_7338439_G	37G	68G
Z_7338440_A	37A	66A
Z_7338441_C	38C	67C
Z_7338442_C	37C	65C
Z_7338443_A	39A	65A
Z_7338444_A	40A	64A
Z_7338445_A	40A	36A_30G
Z_7338446_A	36A	67A
Z_7338447_C	37C	68C
Z_7338448_A	36A	69A
Z_7338449_C	38C	71C
Z_7338450_A	37A	69A
Z_7338451_A	38A	69A
Z_7338452_A	38A	69A
Z_7338453_A	37A	71A
Z_7338454_T	36T	72T
Z_7338455_C	38C	72C
Z_7338456_A	39A	72A
Z_7338457_G	41G	72G
Z_7338458_A	42A	71A
Z_7338459_G	43G	72G
Z_7338460_A	42A	72A
Z_7338461_A	42A	69A

Z_7338462_A	43A	69A
Z_7338463_T	42T	68T
Z_7338464_C	41C	67C
Z_7338465_C	42C	67C
Z_7338466_A	42A	65A
Z_7338467_G	41G	66G
Z_7338468_T	42T	67T
Z_7338469_T	41T	68T
Z_7338470_C	39C	68C
Z_7338471_T	42T	68T
Z_7338472_A	42A	68A
Z_7338473_G	42G	69G
Z_7338474_A	43A	67A
Z_7338475_A	42A	70A
Z_7338476_G	42G	68G
Z_7338477_A	41A	68A
Z_7338478_A	42A	68A
Z_7338479_G	43G	68G
Z_7338480_T	43T	68T
Z_7338481_G	45G	68G
Z_7338482_C	43C	69C
Z_7338483_T	44T	68T
Z_7338484_C	44C	66C
Z_7338485_T	42T	66T
Z_7338486_T	43T	65T
Z_7338487_T	40T	65T
Z_7338488_C	41C	65C
Z_7338489_A	41A	65A
Z_7338490_A	41A	65A
Z_7338491_G	39G	65G
Z_7338492_T	39T	63T
Z_7338493_G	38G	64G
Z_7338494_A	37A	65A
Z_7338495_C	35C	64C
Z_7338496_A	35A	62A
Z_7338497_A	34A	61A
Z_7338498_A	33A	62A
Z_7338499_A	34A	65A
Z_7338500_A	34A	67A
Z_7338501_A	35A	68A
Z_7338502_G	38G	68G
Z_7338503_G	37G	67G
Z_7338504_T	37T	67T

Z_7338505_G	38G	67G
Z_7338506_T	38T	68T
Z_7338507_A	38A	66A
Z_7338508_A	37A	64A
Z_7338509_T	36T	64T
Z_7338510_T	35T	69T
Z_7338511_A	34A	69A
Z_7338512_T	36T	72T
Z_7338513_A	34A	72A
Z_7338514_A	35A	73A
Z_7338515_A	37A	74A
Z_7338516_A	38A	74A
Z_7338517_T	38T	74T
Z_7338518_C	40C	73C
Z_7338519_A	38A	69A
Z_7338520_A	39A	71A
Z_7338521_A	39A	70A
Z_7338522_T	39T	71T
Z_7338523_T	41T	71T
Z_7338524_A	42A	73A
Z_7338525_A	40A	74A
Z_7338526_A	39A	73A
Z_7338527_A	39A	73A
Z_7338528_G	40G	74G
Z_7338529_G	39G	77G
Z_7338530_A	40A	76A
Z_7338531_G	40G	74G
Z_7338532_A	41A	74A
Z_7338533_G	41G	74G
Z_7338534_G	41G	73G
Z_7338535_T	41T	74T
Z_7338536_A	39A	74A
Z_7338537_G	38T	74G
Z_7338538_C	41C	75C
Z_7338539_G	41G	75G
Z_7338540_G	42G	75G
Z_7338541_T	42T	77T
Z_7338542_G	41G	76G
Z_7338543_T	41T	74T
Z_7338544_C	42C	71C
Z_7338545_G	44G	37A_34G
Z_7338546_T	45T	71T
Z_7338547_C	44C	70C

Z_7338548_T	45T	68T
Z_7338549_C	44C	68C
Z_7338550_C	43C	69C
Z_7338551_A	43A	69A
Z_7338552_T	44T	69T
Z_7338553_T	45T	69T
Z_7338554_G	45G	70G
Z_7338555_T	45T	71T
Z_7338556_C	45C	71C
Z_7338557_T	44T	72T
Z_7338558_C	45C	72C
Z_7338559_T	45T	74T
Z_7338560_G	44G	74G
Z_7338561_G	45G	75G
Z_7338562_A	45A	74A
Z_7338563_A	45A	73A
Z_7338564_G	45G	72G
Z_7338565_A	45A	72A
Z_7338566_C	45C	72C
Z_7338567_A	45A	73A
Z_7338568_A	46A	72A
Z_7338569_A	44A	72A
Z_7338570_C	44C	71C
Z_7338571_A	43A	70A
Z_7338572_A	40A	71A
Z_7338573_C	40C	74C
Z_7338574_A	41A	76A
Z_7338575_C	41C	77C
Z_7338576_C	42C	77C
Z_7338577_T	41T	76T
Z_7338578_T	41T	77T
Z_7338579_C	41C	77C
Z_7338580_C	42C	77C
Z_7338581_T	43T	77T
Z_7338582_G	42G	77G
Z_7338583_T	44T	77T
Z_7338584_G	43G	80G
Z_7338585_T	41T	80T
Z_7338586_A	40A	80A
Z_7338587_T	40T	82T
Z_7338588_A	38A	82A
Z_7338589_T	38T	82T
Z_7338590_G	37G	84G

Z_7338591_G	34G	85G
Z_7338592_A	34A	84A
Z_7338593_A	33A	82A
Z_7338594_T	33T	81T
Z_7338595_T	34T	79T
Z_7338596_C	34C	80C
Z_7338597_T	32T	80T
Z_7338598_G	29G	80G
Z_7338599_G	30G	79G
Z_7338600_A	29A	80A
Z_7338601_G	28G	80G
Z_7338602_T	30T	81T
Z_7338603_T	30T	81T
Z_7338604_G	30G	83G
Z_7338605_T	29T	80T
Z_7338606_G	28G	79G
Z_7338607_A	29A	76A
Z_7338608_A	28A	77A
Z_7338609_T	28T	75T
Z_7338610_A	27A	73A
Z_7338611_G	28G	75G
Z_7338612_C	29C	75C
Z_7338613_A	28A	75A
Z_7338614_A	28A	77A
Z_7338615_T	29T	76T
Z_7338616_A	28A	77A
Z_7338617_T	28T	76T
Z_7338618_A	28A	76A
Z_7338619_G	28G	74G
Z_7338620_A	27A	72A
Z_7338621_T	27T	71T
Z_7338622_C	26C	70C
Z_7338623_A	28A	69A
Z_7338624_C	28C	68C
Z_7338625_A	29A	67A
Z_7338626_T	28T	67T
Z_7338627_T	30T	67T
Z_7338628_G	30G	70G
Z_7338629_C	30C	68C
Z_7338630_T	31T	68T
Z_7338631_G	31G	69G
Z_7338632_G	30G	69G
Z_7338633_G	30G	68G

Z_7338634_G	29G	69G
Z_7338635_A	29A	67A
Z_7338636_G	29G	65G
Z_7338637_G	30A	66G
Z_7338638_A	29A	65A
Z_7338639_A	29A	66A
Z_7338640_A	31A	66A
Z_7338641_G	32G	65G
Z_7338642_C	33C	66C
Z_7338643_A	33A	65A
Z_7338644_G	32A	64G
Z_7338645_T	32T	63T
Z_7338646_A	32A	63A
Z_7338647_A	31A	62A
Z_7338648_A	31A	62A
Z_7338649_G	33G	61G
Z_7338650_A	32A	60A
Z_7338651_T	32T	61T
Z_7338652_A	32A	61A
Z_7338653_A	31A	62A
Z_7338654_A	32A	62A
Z_7338655_C	32C	62C
Z_7338656_C	34C	61C
Z_7338657_C	35C	62C
Z_7338658_G	35G	63G
Z_7338659_G	39G	61G
Z_7338660_T	39T	63T
Z_7338661_T	40T	63T
Z_7338662_C	39C	63C
Z_7338663_C	41C	63C
Z_7338664_T	41T	65T
Z_7338665_G	40G	64G
Z_7338666_A	40A	65A
Z_7338667_A	41A	62A
Z_7338668_C	42C	61C
Z_7338669_C	42C	59C
Z_7338670_A	42A	59A
Z_7338671_A	43A	58A
Z_7338672_G	40G	60G
Z_7338673_C	40C	61C
Z_7338674_A	41A	64A
Z_7338675_G	41G	64G
Z_7338676_C	40C	66C

Z_7338677_C	40C	66C
Z_7338678_C	38C	66C
Z_7338679_T	38T	63T
Z_7338680_G	39G	62G
Z_7338681_G	38G	62G
Z_7338682_T	39T	63T
Z_7338683_G	39G	63G
Z_7338684_A	39A	62A
Z_7338685_A	38A	60A
Z_7338686_A	38A	59A
Z_7338687_C	38C	60C
Z_7338688_T	37T	63T
Z_7338689_T	39T	63T
Z_7338690_A	41A	63A
Z_7338691_C	42C	61C
Z_7338692_C	43C	62C
Z_7338693_C	41C	62C
Z_7338694_A	41A	62A
Z_7338695_G	41G	62G
Z_7338696_A	41A	61A
Z_7338697_C	41C	60C
Z_7338698_T	41T	60T
Z_7338699_G	42G	59G
Z_7338700_A	42A	58A
Z_7338701_A	42A	59A
Z_7338702_G	42G	61G
Z_7338703_A	42A	60A
Z_7338704_G	42G	61G
Z_7338705_A	42A	62A
Z_7338706_A	44A	60A
Z_7338707_T	42T	61T
Z_7338708_A	43A	61A
Z_7338709_A	43A	60A
Z_7338710_G	41G	59G
Z_7338711_G	41G	57G
Z_7338712_A	37A	61A
Z_7338713_A	38A	61A
Z_7338714_T	38T	61T
Z_7338715_T	38T	62T
Z_7338716_T	37T	61T
Z_7338717_C	39C	62C
Z_7338718_A	39A	62A
Z_7338719_A	37A	60A

Z_7338720_G	39G	62G
Z_7338721_C	39C	63C
Z_7338722_A	40A	63A
Z_7338723_G	41G	60G
Z_7338724_A	42A	62A
Z_7338725_C	41C	62C
Z_7338726_A	40A	63A
Z_7338727_A	39A	63A
Z_7338728_A	38A	65A
Z_7338729_C	35C	64C
Z_7338730_T	37T	62T
Z_7338731_C	37C	61C
Z_7338732_C	38C	61C
Z_7338733_T	38T	61T
Z_7338734_G	36G	61G
Z_7338735_C	36C	60C
Z_7338736_T	37T	60T
Z_7338737_T	38T	61T
Z_7338738_G	38G	64G
Z_7338739_T	39T	65T
Z_7338740_C	40C	66C
Z_7338741_T	40T	72T
Z_7338742_A	42A	73A
Z_7338743_C	42C	74C
Z_7338744_T	41T	77T
Z_7338745_T	41T	78T
Z_7338746_G	41G	78G
Z_7338747_G	42G	77G
Z_7338748_A	44A	77A
Z_7338749_G	44G	75G
Z_7338750_G	42G	75G
Z_7338751_G	42G	76G
Z_7338752_A	40A	74A
Z_7338753_G	41G	75G
Z_7338754_T	41T	77T
Z_7338755_G	38G	74G
Z_7338756_C	39C	75C
Z_7338757_A	41A	75A
Z_7338758_A	41A	74A
Z_7338759_G	36G	72G
Z_7338760_T	36T	70T
Z_7338761_G	37G	71G
Z_7338762_A	38A	72A

Z_7338763_A	40A	72A
Z_7338764_G	40G	72G
Z_7338765_G	40G	70G
Z_7338766_A	39A	71A
Z_7338767_C	38C	70C
Z_7338768_C	38C	68C
Z_7338769_A	38A	65A
Z_7338770_G	38G	66G
Z_7338771_C	38C	64C
Z_7338772_T	39T	65T
Z_7338773_C	38C	65C
Z_7338774_T	38T	65T
Z_7338775_T	38T	63T
Z_7338776_G	37G	66G
Z_7338777_C	39C	64C
Z_7338778_G	39G	65G
Z_7338779_C	39C	65C
Z_7338780_C	40C	65C
Z_7338781_C	39C	66C
Z_7338782_G	44G	64G
Z_7338783_C	42C	62C
Z_7338784_A	45A	61A
Z_7338785_G	44G	62G
Z_7338786_C	45C	62C
Z_7338787_A	44A	64A
Z_7338788_C	44C	65C
Z_7338789_C	44C	65C
Z_7338790_A	42A	66A
Z_7338791_G	41G	68G
Z_7338792_G	39G	67G
Z_7338793_T	41T	67T
Z_7338794_T	39T	66T
Z_7338795_G	38G	64G
Z_7338796_T	40T	67T
Z_7338797_G	39G	65G
Z_7338798_C	40C	65C
Z_7338799_A	39A	64A
Z_7338800_G	38G	63G
Z_7338801_G	37G	63G
Z_7338802_A	36A	64A
Z_7338803_T	34T	65T
Z_7338804_G	35G	64G
Z_7338805_G	36G	64G

Z_7338806_A	35A	62A
Z_7338807_C	33C	61C
Z_7338808_C	33C	61C
Z_7338809_C	33C	61C
Z_7338810_G	31G	60G
Z_7338811_G	31G	62G
Z_7338812_C	29C	64C
Z_7338813_T	30T	67T
Z_7338814_T	30T	68T
Z_7338815_G	31G	67G
Z_7338816_G	32G	67G
Z_7338817_G	31G	66G
Z_7338818_T	33T	67T
Z_7338819_G	32G	66G
Z_7338820_G	33G	66G
Z_7338821_C	33C	66C
Z_7338822_A	32A	67A
Z_7338823_A	33A	67A
Z_7338824_C	33C	70C
Z_7338825_C	33C	70C
Z_7338826_G	35G	70G
Z_7338827_T	33T	71T
Z_7338828_T	33T	72T
Z_7338829_G	37G	71G
Z_7338830_A	35A	72A
Z_7338831_G	35G	73G
Z_7338832_A	34A	72A
Z_7338833_G	33G	71G
Z_7338834_G	32G	72G
Z_7338835_T	31T	70T
Z_7338836_C	34C	66C
Z_7338837_C	34C	67C
Z_7338838_A	34A	67A
Z_7338839_C	33C	65C
Z_7338840_C	33C	65C
Z_7338841_T	33T	64T
Z_7338842_A	32A	63A
Z_7338843_G	33G	64G
Z_7338844_G	32G	30T_35G
Z_7338845_T	32T	65T
Z_7338846_T	32T	64T
Z_7338847_G	31G	64G
Z_7338848_G	30G	66G

Z_7338849_G	31G	65G
Z_7338850_G	29G	65G
Z_7338851_A	29A	66A
Z_7338852_A	26A	65A
Z_7338853_G	26G	65G
Z_7338854_A	27A	65A
Z_7338855_A	28A	65A
Z_7338856_C	28C	65C
Z_7338857_A	29A	64A
Z_7338858_G	29G	64G
Z_7338859_A	30A	64A
Z_7338860_G	29G	64G
Z_7338861_A	30A	63A
Z_7338862_G	31G	63G
Z_7338863_G	31G	63G
Z_7338864_G	33G	61G
Z_7338865_G	33G	62G
Z_7338866_A	33A	62A
Z_7338867_G	33G	62G
Z_7338868_A	34A	63A
Z_7338869_T	36T	65T
Z_7338870_T	35T	65T
Z_7338871_C	36C	63C
Z_7338872_A	37A	63A
Z_7338873_G	35G	64G
Z_7338874_G	35G	64G
Z_7338875_A	37A	65A
Z_7338876_C	37C	64C
Z_7338877_T	37T	64T
Z_7338878_A	37A	66A
Z_7338879_C	38C	66C
Z_7338880_C	38C	68C
Z_7338881_C	41C	69C
Z_7338882_A	41A	66A
Z_7338883_A	40A	65A
Z_7338884_C	39C	65C
Z_7338885_C	41C	64C
Z_7338886_T	38T	62T
Z_7338887_C	38C	63C
Z_7338888_G	37G	64G
Z_7338889_T	37T	64T
Z_7338890_T	37T	66T
Z_7338891_G	38G	63G

Z_7338892_C	39C	63C
Z_7338893_C	40C	63C
Z_7338894_C	40C	64C
Z_7338895_T	41T	64T
Z_7338896_G	39G	63G
Z_7338897_G	39G	62G
Z_7338898_G	39G	62G
Z_7338899_G	37G	63G
Z_7338900_A	39A	64A
Z_7338901_A	38A	64A
Z_7338902_G	38G	63G
Z_7338903_G	37G	63G
Z_7338904_G	39G	63G
Z_7338905_C	40C	63C
Z_7338906_C	37C	60C
Z_7338907_G	38A	26A_33G
Z_7338908_G	38G	56G
Z_7338909_C	38C	55C
Z_7338910_C	36C	55C
Z_7338911_A	39A	55A
Z_7338912_T	38T	57T
Z_7338913_C	37C	58C
Z_7338914_A	38A	58A
Z_7338915_G	38G	57G
Z_7338916_A	39A	57A
Z_7338917_A	39A	56A
Z_7338918_A	39A	55A
Z_7338919_G	38G	53G
Z_7338920_A	40A	54A
Z_7338921_C	40C	54C
Z_7338922_C	40C	55C
Z_7338923_T	40T	57T
Z_7338924_C	39C	61C
Z_7338925_C	39C	60C
Z_7338926_C	38C	60C
Z_7338927_A	37A	60A
Z_7338928_T	38T	58T
Z_7338929_C	36C	56C
Z_7338930_G	37G	56G
Z_7338931_G	36G	56G
Z_7338932_A	36A	56A
Z_7338933_T	37T	54T
Z_7338934_G	35G	53G

Z_7338935_C	36C	54C
Z_7338936_A	35A	56A
Z_7338937_G	36G	58G
Z_7338938_G	34G	57G
Z_7338939_G	33G	57G
Z_7338940_T	32T	58T
Z_7338941_T	33T	59T
Z_7338942_T	32T	60T
Z_7338943_T	33T	59T
Z_7338944_A	34A	60A
Z_7338945_A	33A	61A
Z_7338946_A	33A	60A
Z_7338947_T	34T	60T
Z_7338948_T	36T	61T
Z_7338949_T	35T	63T
Z_7338950_T	35T	64T
Z_7338951_A	34A	64A
Z_7338952_G	34G	65G
Z_7338953_A	35A	66A
Z_7338954_A	35A	69A
Z_7338955_T	33T	70T
Z_7338956_G	33G	71G
Z_7338957_T	33T	72T
Z_7338958_A	33A	73A
Z_7338959_A	34A	74A
Z_7338960_A	34A	74A
Z_7338961_T	33T	75T
Z_7338962_C	33C	75C
Z_7338963_T	34T	74T
Z_7338964_A	32A	73A
Z_7338965_G	33G	73G
Z_7338966_C	33C	74C
Z_7338967_A	33A	74A
Z_7338968_G	31G	72G
Z_7338969_T	31T	73T
Z_7338970_T	29T	72T
Z_7338971_C	30C	72C
Z_7338972_A	30A	73A
Z_7338973_G	30G	71G
Z_7338974_G	29G	71G
Z_7338975_G	28G	68G
Z_7338976_A	28A	68A
Z_7338977_C	28C	68C

Z_7338978_C	29C	69C
Z_7338979_A	29A	70A
Z_7338980_G	29G	70G
Z_7338981_A	28A	70A
Z_7338982_C	29C	70C
Z_7338983_A	29A	69A
Z_7338984_T	29T	69T
Z_7338985_T	29T	67T
Z_7338986_T	27T	67T
Z_7338987_T	29T	70T
Z_7338988_C	29C	71C
Z_7338989_T	30T	70T
Z_7338990_C	29C	70C
Z_7338991_C	28C	71C
Z_7338992_C	29C	72C
Z_7338993_T	29T	72T
Z_7338994_C	28C	71C
Z_7338995_T	27T	70T
Z_7338996_C	28C	70C
Z_7338997_A	28A	71A
Z_7338998_G	28G	72G
Z_7338999_G	28G	73G
Z_7339000_G	29G	73G
Z_7339001_A	29A	72A
Z_7339002_T	28T	74T
Z_7339003_G	26G	74G
Z_7339004_T	27T	73T
Z_7339005_G	27G	73G
Z_7339006_T	27T	73T
Z_7339007_G	26G	71G
Z_7339008_T	27T	70T
Z_7339009_A	27A	70A
Z_7339010_T	28T	69T
Z_7339011_G	29G	69G
Z_7339012_T	29T	69T
Z_7339013_T	28T	68T
Z_7339014_T	27T	67T
Z_7339015_T	28T	51T
Z_7339016_G	28G	52G
Z_7339017_T	27T	51T
Z_7339018_G	25G	60G
Z_7339019_T	25T	56T
Z_7339020_A	25A	56A

Z_7339021_G	24G	56G
Z_7339022_G	24G	56G
Z_7339023_T	22T	57T
Z_7339024_T	24T	57T
Z_7339025_C	25C	57C
Z_7339026_C	26C	56C
Z_7339027_T	27T	55T
Z_7339028_C	26C	55C
Z_7339029_C	26C	55C
Z_7339030_C	26C	73C
Z_7339031_C	25C	61C
Z_7339032_T	25T	52T
Z_7339033_G	24G	54G
Z_7339034_G	24G	54G
Z_7339035_A	26A	53A
Z_7339036_C	27C	52C
Z_7339037_A	28A	52A
Z_7339038_A	28A	54A
Z_7339039_C	27C	52C
Z_7339040_A	28A	53A
Z_7339041_C	27C	53C
Z_7339042_C	28C	54C
Z_7339043_T	29T	56T
Z_7339044_T	29T	54T
Z_7339045_A	29A	53A
Z_7339046_C	30C	53C
Z_7339047_A	29A	52A
Z_7339048_A	33A	52A
Z_7339049_G	34G	50G
Z_7339050_A	35A	50A
Z_7339051_A	35A	49A
Z_7339052_T	34T	50T
Z_7339053_G	34G	51G
Z_7339054_G	34G	49G
Z_7339055_C	34C	52C
Z_7339056_A	33A	51A
Z_7339057_C	33C	51C
Z_7339058_A	34A	51A
Z_7339059_A	33A	51A
Z_7339060_C	33C	52C
Z_7339061_T	32T	52T
Z_7339062_G	32G	51G
Z_7339063_G	33G	51G

Z_7339064_A	33A	52A
Z_7339065_A	32A	53A
Z_7339066_G	32G	54G
Z_7339067_G	32G	53G
Z_7339068_A	33A	33A_19G
Z_7339069_C	32C	52C
Z_7339070_A	32A	32A_19C
Z_7339071_T	33T	52T
Z_7339072_A	34A	53A
Z_7339073_A	34A	55A
Z_7339074_A	34A	54A
Z_7339075_A	35A	55A
Z_7339076_A	35A	55A
Z_7339077_G	36G	55G
Z_7339078_T	35T	55T
Z_7339079_G	36G	55G
Z_7339080_T	37T	55T
Z_7339081_G	37G	56G
Z_7339082_C	36C	55C
Z_7339083_C	37C	55C
Z_7339084_T	38T	55T
Z_7339085_T	36T	55T
Z_7339086_T	36T	55T
Z_7339087_T	37T	55T
Z_7339088_T	38T	53T
Z_7339089_T	39T	53T
Z_7339090_G	38G	54G
Z_7339091_G	37G	56G
Z_7339092_A	38A	57A
Z_7339093_A	38A	55A
Z_7339094_A	36A	56A
Z_7339095_G	35G	57G
Z_7339096_G	33G	56G
Z_7339097_G	33G	56G
Z_7339098_G	34G	56G
Z_7339099_G	35G	57G
Z_7339100_C	36C	57C
Z_7339101_A	37A	58A
Z_7339102_T	39T	58T
Z_7339103_C	39C	59C
Z_7339104_C	40C	59C
Z_7339105_C	37C	62C
Z_7339106_G	35G	61G

Z_7339107_C	35C	62C
Z_7339108_A	36A	64A
Z_7339109_G	36G	65G
Z_7339110_A	36A	65A
Z_7339111_G	38G	65G
Z_7339112_G	37G	65G
Z_7339113_C	35C	66C
Z_7339114_C	36C	66C
Z_7339115_A	36A	68A
Z_7339116_C	36C	67C
Z_7339117_C	35C	67C
Z_7339118_T	32T	66T
Z_7339119_G	32G	65G
Z_7339120_G	31G	66G
Z_7339121_C	31C	66C
Z_7339122_C	32C	66C
Z_7339123_A	32A	66A
Z_7339124_A	32A	67A
Z_7339125_A	32A	65A
Z_7339126_G	32G	65G
Z_7339127_T	32T	65T
Z_7339128_G	31G	64G
Z_7339129_G	31G	63G
Z_7339130_A	30A	63A
Z_7339131_T	32T	63T
Z_7339132_T	34T	63T
Z_7339133_C	33C	61C
Z_7339134_T	34T	34T_25C
Z_7339135_C	32C	24G_33C
Z_7339136_A	34A	57A
Z_7339137_T	35T	56T
Z_7339138_G	34G	55G
Z_7339139_T	34T	56T
Z_7339140_T	34T	56T
Z_7339141_A	34A	56A
Z_7339142_T	33T	24A_32T
Z_7339143_C	33C	55C
Z_7339144_C	31C	55C
Z_7339145_A	31A	55A
Z_7339146_C	32C	57C
Z_7339147_C	31C	56C
Z_7339148_T	31T	55T
Z_7339149_C	30C	55C

Z_7339150_A	29A	53A
Z_7339151_G	29G	54G
Z_7339152_G	29G	44G
Z_7339153_C	30C	13T_32C
Z_7339154_C	29C	13T_32C
Z_7339155_G	30G	47G
Z_7339156_G	29G	48G
Z_7339157_A	30A	35A_13G
Z_7339158_G	30G	51G
Z_7339159_A	29A	50A
Z_7339160_A	29A	50A
Z_7339161_G	29G	50G
Z_7339162_T	29T	49T
Z_7339163_C	29C	50C
Z_7339164_A	29A	50A
Z_7339165_C	30C	50C
Z_7339166_T	29T	50T
Z_7339167_C	28C	49C
Z_7339168_C	28C	49C
Z_7339169_A	27A	49A
Z_7339170_C	28C	49C
Z_7339171_T	27T	48T
Z_7339172_C	24C	48C
Z_7339173_C	24C	48C
Z_7339174_T	23T	47T
Z_7339175_G	23G	48G
Z_7339176_G	23G	49G
Z_7339177_A	22A	44A
Z_7339178_G	24G_18C	45G
Z_7339179_C	1A_1T_4C	46C
Z_7339180_G	26G	48G
Z_7339181_C	25C	49C
Z_7339182_G	25G	47G
Z_7339183_C	24C	46C
Z_7339184_G	25G	46G
Z_7339185_C	26C	38C
Z_7339186_C	26C	39C
Z_7339187_C	26C	39C
Z_7339188_A	27A	32A
Z_7339189_C	29C	35C
Z_7339190_C	29C	34C
Z_7339191_C	29C	34C
Z_7339192_A	29A	34A

Z_7339193_C	32C	36C
Z_7339194_C	32C	36C
Z_7339195_C	32C	36C
Z_7339196_T	34T	36T
Z_7339197_G	35G	36G
Z_7339198_A	38A	35A
Z_7339199_A	38A	35A
Z_7339200_T	40T	36T
Z_7339201_A	40A	38A
Z_7339202_T	39T	37T
Z_7339203_A	38A	37A
Z_7339204_C	36C	38C
Z_7339205_G	37G	38G
Z_7339206_C	38C	37C
Z_7339207_C	37C	37C
Z_7339208_A	38A	37A
Z_7339209_C	38C	37C
Z_7339210_C	40C	38C
Z_7339211_C	40C	38C
Z_7339212_A	41A	38A
Z_7339213_C	41C	39C
Z_7339214_C	41C	39C
Z_7339215_T	42T	38T
Z_7339216_G	43G	38G
Z_7339217_C	43C	38C
Z_7339218_C	43C	38C
Z_7339219_C	43C	39C
Z_7339220_A	43A	40A
Z_7339221_T	42T	41T
Z_7339222_A	42A	42A
Z_7339223_T	41T	43T
Z_7339224_A	42A	43A
Z_7339225_G	42G	43G
Z_7339226_A	44A	28A_14G
Z_7339227_T	43T	42T
Z_7339228_C	42C	43C
Z_7339229_G	42G	44G
Z_7339230_T	41T	46T
Z_7339231_G	42G	47G
Z_7339232_C	41C	16T_31C
Z_7339233_T	42T	47T
Z_7339234_G	41G	46G
Z_7339235_A	43A	29A_16G_1C

Z_7339236_C	42C	49C
Z_7339237_A	42A	51A
Z_7339238_G	42G	51G
Z_7339239_G	44G	52G
Z_7339240_A	44A	52A
Z_7339241_A	44A	52A
Z_7339242_G	43G	52G
Z_7339243_T	43T	52T
Z_7339244_G	43G	51G
Z_7339245_T	44T	49T
Z_7339246_G	43G	51G
Z_7339247_A	42A	52A
Z_7339248_C	41C	54C
Z_7339249_G	42A	55G
Z_7339250_T	41T	52T
Z_7339251_C	42C	52C
Z_7339252_A	42A	53A
Z_7339253_A	42A	55A
Z_7339254_T	41T	55T
Z_7339255_T	42T	55T
Z_7339256_G	42G	53G
Z_7339257_T	42T	55T
Z_7339258_G	41G	56G
Z_7339259_A	40A	57A
Z_7339260_T	40C	35T_22C
Z_7339261_G	40G	58G
Z_7339262_A	41A	60A
Z_7339263_T	38T	60T
Z_7339264_G	37G	60G
Z_7339265_G	38G	58G
Z_7339266_A	37A	61A
Z_7339267_G	37G	63G
Z_7339268_A	34A	63A
Z_7339269_A	34A	62A
Z_7339270_A	33A	63A
Z_7339271_T	34T	62T
Z_7339272_G	36G	63G
Z_7339273_A	36A	63A
Z_7339274_G	35G	62G
Z_7339275_C	34C	61C
Z_7339276_A	32A	61A
Z_7339277_A	31C	36A_26C
Z_7339278_T	30T	62T

Z_7339279_C	31C	64C
Z_7339280_G	33G	64G
Z_7339281_G	33G	64G
Z_7339282_A	32A	65A
Z_7339283_G	31G	68G
Z_7339284_C	29T	69C
Z_7339285_T	30G	68T
Z_7339286_C	34C	69C
Z_7339287_T	35T	67T
Z_7339288_C	39C	65C
Z_7339289_T	40T	68T
Z_7339290_C	41C	33G_35C
Z_7339291_T	41T	64T
Z_7339292_C	42C	70C
Z_7339293_T	42T	74T
Z_7339294_T	41T	73T
Z_7339295_T	41T	72T
Z_7339296_A	40A	70A
Z_7339297_T	41T	72T
Z_7339298_C	41C	73C
Z_7339299_T	41T	71T
Z_7339300_A	41A	72A
Z_7339301_A	41A	71A
Z_7339302_G	41G	73G
Z_7339303_C	42C	73C
Z_7339304_A	42A	73A
Z_7339305_T	40T	71T
Z_7339306_C	42C	72C
Z_7339307_G	41G	72G
Z_7339308_C	43C	71C
Z_7339309_T	41T	71T
Z_7339310_G	41G	71G
Z_7339311_G	41G	69G
Z_7339312_A	40A	72A
Z_7339313_T	40T	72T
Z_7339314_T	39T	70T
Z_7339315_A	39A	70A
Z_7339316_G	39G	72G
Z_7339317_A	37A	71A
Z_7339318_A	37A	70A
Z_7339319_C	32G	22G_43C
Z_7339320_T	31C	43T_22C
Z_7339321_A	31A	65A

Z_7339322_C	30A	22A_43C
Z_7339323_C	30C	65C
Z_7339324_T	32C	42T_22C
Z_7339325_T	41T	76T
Z_7339326_T	42T	75T
Z_7339327_C	42C	75C
Z_7339328_T	43T	75T
Z_7339329_C	44C	75C
Z_7339330_T	44T	75T
Z_7339331_G	43G	77G
Z_7339332_G	44G	76G
Z_7339333_T	44T	76T
Z_7339334_C	45C	80C
Z_7339335_T	44T	81T
Z_7339336_A	43A	80A
Z_7339337_G	42G	79G
Z_7339338_C	43C	79C
Z_7339339_A	43A	80A
Z_7339340_A	43A	82A
Z_7339341_G	41G	82G
Z_7339342_G	40G	81G
Z_7339343_C	40C	80C
Z_7339344_C	40C	81C
Z_7339345_C	41C	81C
Z_7339346_T	41T	81T
Z_7339347_T	41T	82T
Z_7339348_C	41C	79C
Z_7339349_C	43C	81C
Z_7339350_T	40T	82T
Z_7339351_T	39T	82T
Z_7339352_A	40A	81A
Z_7339353_A	40A	81A
Z_7339354_C	40C	83C
Z_7339355_G	39A	83G
Z_7339356_T	40T	82T
Z_7339357_A	38A	81A
Z_7339358_T	35T	81T
Z_7339359_G	34G	81G
Z_7339360_A	33A	82A
Z_7339361_C	34C	81C
Z_7339362_T	34T	80T
Z_7339363_A	34A	81A
Z_7339364_A	34A	81A

Z_7339365_G	32G	81G
Z_7339366_G	32G	82G
Z_7339367_T	31T	82T
Z_7339368_G	29G	82G
Z_7339369_C	32C	81C
Z_7339370_A	33A	80A
Z_7339371_C	31C	79C
Z_7339372_A	33A	78A
Z_7339373_T	33T	78T
Z_7339374_T	33T	77T
Z_7339375_T	33T	77T
Z_7339376_T	31T	76T
Z_7339377_T	32T	75T
Z_7339378_T	32T	74T
Z_7339379_A	32A	72A
Z_7339380_A	33A	72A
Z_7339381_A	32A	72A
Z_7339382_A	32A	73A
Z_7339383_A	32A	74A
Z_7339384_A	32A	72A
Z_7339385_A	31A	73A
Z_7339386_C	30C	73C
Z_7339387_A	32A	72A
Z_7339388_G	33G	67G
Z_7339389_A	32A	67A
Z_7339390_T	29T	67T
Z_7339391_C	31C	67C
Z_7339392_T	32T	67T
Z_7339393_G	32G	67G
Z_7339394_T	31T	68T
Z_7339395_A	31A	67A
Z_7339396_T	32T	69T
Z_7339397_T	33T	68T
Z_7339398_C	33C	70C
Z_7339399_C	33C	72C
Z_7339400_T	33T	72T
Z_7339401_T	33T	73T
Z_7339402_C	34C	74C
Z_7339403_T	34T	74T
Z_7339404_T	34T	74T
Z_7339405_T	36T	70T
Z_7339406_C	36C	71C
Z_7339407_C	38C	68C

Z_7339408_C	38C	70C
Z_7339409_A	38A	70A
Z_7339410_C	37C	67C
Z_7339411_G	37G	64G
Z_7339412_G	38G	64G
Z_7339413_C	39C	65C
Z_7339414_C	38C	66C
Z_7339415_C	37T	64C
Z_7339416_C	38C	63C
Z_7339417_A	38A	63A
Z_7339418_G	38G	64G
Z_7339419_C	37C	64C
Z_7339420_T	37T	66T
Z_7339421_G	39G	66G
Z_7339422_T	38T	66T
Z_7339423_A	38A	66A
Z_7339424_C	37C	68C
Z_7339425_C	36C	68C
Z_7339426_T	39T	65T
Z_7339427_C	40C	67C
Z_7339428_C	39C	69C
Z_7339429_T	41T	65T
Z_7339430_G	40G	63G
Z_7339431_G	41G	64G
Z_7339432_C	41C	63C
Z_7339433_C	42C	63C
Z_7339434_G	42G	62G
Z_7339435_G	42G	60G
Z_7339436_T	42T	61T
Z_7339437_C	41C	60C
Z_7339438_A	42A	59A
Z_7339439_C	42C	59C
Z_7339440_C	42C	59C
Z_7339441_A	42A	59A
Z_7339442_G	42G	59G
Z_7339443_C	42C	60C
Z_7339444_A	42A	59A
Z_7339445_C	41C	58C
Z_7339446_C	42C	59C
Z_7339447_A	43A	60A
Z_7339448_T	43T	60T
Z_7339449_G	43G	59G
Z_7339450_C	42C	59C

Z_7339451_T	43T	58T
Z_7339452_G	44G	60G
Z_7339453_T	43T	60T
Z_7339454_G	43G	60G
Z_7339455_C	48C	60C
Z_7339456_T	51T	60T
Z_7339457_C	49C	61C
Z_7339458_C	49C	60C
Z_7339459_T	49T	60T
Z_7339460_T	49T	61T
Z_7339461_C	46C	60C
Z_7339462_C	48C	59C
Z_7339463_C	48C	60C
Z_7339464_A	47A	61A
Z_7339465_T	48T	63T
Z_7339466_A	47A	63A
Z_7339467_C	45C	64C
Z_7339468_T	45T	63T
Z_7339469_G	45G	62G
Z_7339470_C	45C	62C
Z_7339471_A	44A	60A
Z_7339472_C	45C	60C
Z_7339473_T	45T	60T
Z_7339474_T	45T	61T
Z_7339475_G	43G	60G
Z_7339476_G	41G	59G
Z_7339477_C	43C	59C
Z_7339478_G	42G	58G
Z_7339479_G	42G	57G
Z_7339480_A	42A	58A
Z_7339481_A	42A	59A
Z_7339482_G	42G	59G
Z_7339483_A	42A	60A
Z_7339484_T	42T	60T
Z_7339485_G	42G	62G
Z_7339486_T	43T	62T
Z_7339487_G	43G	60G
Z_7339488_G	43G	59G
Z_7339489_T	45T	58T
Z_7339490_C	46C	56C
Z_7339491_A	43A	55A
Z_7339492_A	43A	56A
Z_7339493_A	45A	55A

Z_7339494_A	44A	53A
Z_7339495_A	46A	54A
Z_7339496_C	43C	52C
Z_7339497_C	42C	51C
Z_7339498_A	42A	51A
Z_7339499_G	40G	53G
Z_7339500_C	42C	53C
Z_7339501_A	42A	53A
Z_7339502_G	42G	54G
Z_7339503_T	42T	52T
Z_7339504_T	43T	52T
Z_7339505_C	46C	51C
Z_7339506_G	45G	51G
Z_7339507_C	46C	51C
Z_7339508_T	48T	50T
Z_7339509_C	48C	49C
Z_7339510_C	47C	49C
Z_7339511_A	48A	48A
Z_7339512_G	47G	48G
Z_7339513_C	46C	48C
Z_7339514_A	47A	49A
Z_7339515_G	47G	50G
Z_7339516_T	48T	50T
Z_7339517_T	48T	51T
Z_7339518_C	48C	52C
Z_7339519_T	49T	50T
Z_7339520_G	49G	50G
Z_7339521_C	49C	50C
Z_7339522_A	48A	49A
Z_7339523_G	47G	47G
Z_7339524_C	47C	47C
Z_7339525_A	43A	47A
Z_7339526_G	39G	47G
Z_7339527_C	40C	49C
Z_7339528_T	38T	49T
Z_7339529_T	38T	50T
Z_7339530_C	39C	49C
Z_7339531_A	39A	48A
Z_7339532_T	38T	47T
Z_7339533_C	38C	48C
Z_7339534_T	38T	49T
Z_7339535_G	39G	48G
Z_7339536_G	38G	50G

Z_7339537_T	36T	48T
Z_7339538_C	37C	47C
Z_7339539_T	37T	49T
Z_7339540_C	36C	48C
Z_7339541_C	37C	48C
Z_7339542_G	36G	46G
Z_7339543_A	35A	46A
Z_7339544_C	36C	45C
Z_7339545_C	36C	45C
Z_7339546_T	35T	45T
Z_7339547_G	33G	45G
Z_7339548_G	33G	45G
Z_7339549_A	35A	45A
Z_7339550_A	35A	44A
Z_7339551_G	34G	44G
Z_7339552_A	34A	46A
Z_7339553_A	34A	46A
Z_7339554_G	33G	47G
Z_7339555_A	36A	46A
Z_7339556_A	34A	45A
Z_7339557_A	35A	46A
Z_7339558_G	32G	22A_23G
Z_7339559_G	34G	44G
Z_7339560_C	33C	42C
Z_7339561_A	33A	42A
Z_7339562_G	32G	42G
Z_7339563_G	30G	43G
Z_7339564_G	31G	43G
Z_7339565_A	30A	43A
Z_7339566_G	30G	44G
Z_7339567_C	32C	44C
Z_7339568_A	32A	45A
Z_7339569_A	32A	46A
Z_7339570_A	30A	48A
Z_7339571_G	29G	48G
Z_7339572_C	29C	47C
Z_7339573_C	28C	48C
Z_7339574_G	27G	49G
Z_7339575_G	24G	48G
Z_7339576_T	25T	47T
Z_7339577_G	26G	46G
Z_7339578_A	26A	45A
Z_7339579_G	25G	48G

Z_7339580_A	25A	46A
Z_7339581_A	24A	46A
Z_7339582_G	24G	47G
Z_7339583_C	22C	49C
Z_7339584_C	25C	50C
Z_7339585_G	26G	50G
Z_7339586_C	24C	52C
Z_7339587_T	25T	51T
Z_7339588_G	25G	51G
Z_7339589_G	24G	52G
Z_7339590_C	26C	52C
Z_7339591_C	26C	54C
Z_7339592_C	26C	55C
Z_7339593_C	28C	24T_31C
Z_7339594_A	27A	54A
Z_7339595_C	28C	56C
Z_7339596_A	30A	55A
Z_7339597_C	30C	56C
Z_7339598_C	30C	24T_32C
Z_7339599_G	30G	57G
Z_7339600_C	29C	57C
Z_7339601_A	29A	57A
Z_7339602_A	27A	57A
Z_7339603_G	29G	60G
Z_7339604_G	31G	60G
Z_7339605_G	31G	60G
Z_7339606_C	31C	65C
Z_7339607_C	32C	65C
Z_7339608_A	32A	64A
Z_7339609_G	32G	62G
Z_7339610_A	31A	60A
Z_7339611_G	32G	60G
Z_7339612_G	32G	56G
Z_7339613_C	35C	61C
Z_7339614_T	34T	63T
Z_7339615_G	34G	62G
Z_7339616_C	33C	63C
Z_7339617_C	34C	64C
Z_7339618_A	34A	66A
Z_7339619_T	32T	68T
Z_7339620_T	33T	24A_45T
Z_7339621_G	34G	68G
Z_7339622_C	34C	69C

Z_7339623_G	34A	25A_44G
Z_7339624_T	37T	68T
Z_7339625_G	34G	68G
Z_7339626_G	35G	70G
Z_7339627_A	34A	70A
Z_7339628_G	34G	68G
Z_7339629_A	33A	66A
Z_7339630_G	33G	66G
Z_7339631_C	35C	64C
Z_7339632_T	37T	65T
Z_7339633_T	37T	65T
Z_7339634_A	37A	63A
Z_7339635_A	37A	62A
Z_7339636_G	36G	63G
Z_7339637_G	35G	64G
Z_7339638_C	35C	64C
Z_7339639_A	37A	65A
Z_7339640_G	38G	66G
Z_7339641_A	38A	68A
Z_7339642_G	38G	66G
Z_7339643_G	39G	66G
Z_7339644_G	42G	66G
Z_7339645_G	42G	67G
Z_7339646_C	42C	66C
Z_7339647_G	40G	64G
Z_7339648_G	39G	65G
Z_7339649_G	39G	65G
Z_7339650_T	38T	66T
Z_7339651_C	38C	66C
Z_7339652_C	39C	66C
Z_7339653_C	40C	67C
Z_7339654_T	36T	68T
Z_7339655_C	37C	70C
Z_7339656_G	37G	70G
Z_7339657_G	35G	68G
Z_7339658_T	35T	69T
Z_7339659_G	34G	69G
Z_7339660_T	35T	67T
Z_7339661_C	36C	69C
Z_7339662_A	37A	70A
Z_7339663_G	36G	70G
Z_7339664_A	35A	68A
Z_7339665_C	35C	66C

Z_7339666_C	36C	66C
Z_7339667_C	37C	65C
Z_7339668_A	37A	65A
Z_7339669_C	38C	66C
Z_7339670_G	39G	66G
Z_7339671_C	40C	26T_40C
Z_7339672_G	40G	66G
Z_7339673_C	39C	66C
Z_7339674_C	36C	63C
Z_7339675_G	40G	65G
Z_7339676_T	40T	64T
Z_7339677_T	39T	63T
Z_7339678_T	40T	62T
Z_7339679_T	40T	61T
Z_7339680_A	41A	63A
Z_7339681_T	40T	61T
Z_7339682_T	42T	61T
Z_7339683_T	39T	61T
Z_7339684_T	40T	60T
Z_7339685_C	39C	32T_29C
Z_7339686_A	41A	59A
Z_7339687_A	44A	59A
Z_7339688_A	46A	59A
Z_7339689_A	46A	58A
Z_7339690_T	46T	56T
Z_7339691_A	44A	56A
Z_7339692_A	46A	57A
Z_7339693_G	46G	57G
Z_7339694_T	44T	29T_27C
Z_7339695_G	44G	56G
Z_7339696_A	44A	56A
Z_7339697_C	43C	55C
Z_7339698_A	44A	54A
Z_7339699_T	43T	53T
Z_7339700_G	42G	52G
Z_7339701_T	41T	27T
Z_7339702_T	39T	27T
Z_7339703_A	39A	30A
Z_7339704_T	41T	30T
Z_7339705_T	41T	30T
Z_7339706_T	41T	30T
Z_7339707_T	41T	30T
Z_7339708_G	43G	29G

Z_7339709_G	41G	28G
Z_7339710_C	41C	27C
Z_7339711_C	42C	27C
Z_7339712_G	42G	28G
Z_7339713_C	44C	27C
Z_7339714_A	43A	27A
Z_7339715_A	43A	27A
Z_7339716_A	44A	27A
Z_7339717_C	44C	26C
Z_7339718_A	43A	26A
Z_7339719_A	44A	29A
Z_7339720_G	44G	31G
Z_7339721_A	44A	31A
Z_7339722_T	45T	31T
Z_7339723_G	43G	31G
Z_7339724_T	45C	32T
Z_7339725_G	45G	32G
Z_7339726_T	45T	32T
Z_7339727_G	47G	32G
Z_7339728_G	47G	32G
Z_7339729_T	47T	32T
Z_7339730_T	46T	32T
Z_7339731_G	45G	32G
Z_7339732_G	41G	31G
Z_7339733_G	43G	31G
Z_7339734_A	44A	31A
Z_7339735_A	42A	31A
Z_7339736_C	42C	31C
Z_7339737_A	40A	31A
Z_7339738_T	39T	32T
Z_7339739_T	40C	30T
Z_7339740_G	39G	56G
Z_7339741_C	39C	55C
Z_7339742_G	38G	57G
Z_7339743_G	37G	56G
Z_7339744_C	39C	57C
Z_7339745_T	34T	57T
Z_7339746_C	36C	58C
Z_7339747_C	36C	57C
Z_7339748_C	35C	58C
Z_7339749_G	35G	57G
Z_7339750_G	36G	55G
Z_7339751_A	37A	57A

Z_7339752_A	36A	57A
Z_7339753_G	36G	57G
Z_7339754_C	35C	57C
Z_7339755_A	35A	57A
Z_7339756_T	34T	58T
Z_7339757_C	33C	59C
Z_7339758_A	31A	60A
Z_7339759_T	31T	60T
Z_7339760_T	30T	61T
Z_7339761_C	32C	61C
Z_7339762_C	30C	61C
Z_7339763_C	30C	60C
Z_7339764_C	29C	59C
Z_7339765_T	29T	59T
Z_7339766_A	29A	58A
Z_7339767_T	29T	58T
Z_7339768_T	29T	59T
Z_7339769_T	30T	59T
Z_7339770_A	31A	57A
Z_7339771_G	31G	57G
Z_7339772_T	30T	57T
Z_7339773_G	30G	57G
Z_7339774_A	27A	56A
Z_7339775_G	28G	53G
Z_7339776_G	28G	53G
Z_7339777_G	28G	52G
Z_7339778_G	25G	52G
Z_7339779_A	28A	52A
Z_7339780_G	26G	50G
Z_7339781_A	27A	49A
Z_7339782_A	27A	46A
Z_7339783_G	25G	45G
Z_7339784_A	26A	45A
Z_7339785_C	26C	45C
Z_7339786_A	25A	44A
Z_7339787_C	24C	42C
Z_7339788_A	25A	42A
Z_7339789_C	24C	42C
Z_7339790_A	24A	42A
Z_7339791_C	22C	42C
Z_7339792_C	25C	42C
Z_7339793_A	24A	40A
Z_7339794_G	24G	40G

Z_7339795_C	25C	40C
Z_7339796_C	24C	39C
Z_7339797_A	23A	39A
Z_7339798_C	23C	36C
Z_7339799_A	23A	36A
Z_7339800_A	24A	36A
Z_7339801_G	22G	35G
Z_7339802_A	23A	34A
Z_7339803_A	23A	33A
Z_7339804_T	22T	33T
Z_7339805_A	22A	32A
Z_7339806_C	1A_21T_19G_23C	32C
Z_7339807_G	25G	32G
Z_7339808_G	23G	32G
Z_7339809_G	25G	32G
Z_7339810_G	26G	34G
Z_7339811_G	26G	36G
Z_7339812_G	26G	66G
Z_7339813_G	26G	38G
Z_7339814_G	26G	38G
Z_7339815_T	25T	33T
Z_7339816_G	25G	33G
Z_7339817_C	25C	34C
Z_7339818_A	24A	33A
Z_7339819_T	23T	33T
Z_7339820_A	24A	33A
Z_7339821_T	24T	33T
Z_7339822_G	24G	33G
Z_7339823_G	24G	33G
Z_7339824_G	24G	34G
Z_7339825_C	22C	37C
Z_7339826_C	21C	38C
Z_7339827_C	23C	40C
Z_7339828_C	23C	41C
Z_7339829_A	21A	41A
Z_7339830_C	21C	41C
Z_7339831_A	21A	41A
Z_7339832_A	22A	41A
Z_7339833_A	22G	30A_10G
Z_7339834_T	22T	40T
Z_7339835_C	22C	40C
Z_7339836_A	23A	39A
Z_7339837_C	21C	40C

Z_7339838_C	22C	41C
Z_7339839_G	24G	40G
Z_7339840_G	23G	41G
Z_7339841_C	23C	42C
Z_7339842_C	23C	43C
Z_7339843_A	26A	42A
Z_7339844_C	26C	44C
Z_7339845_C	26C	45C
Z_7339846_C	25C	45C
Z_7339847_T	24T	45T
Z_7339848_T	26T	44T
Z_7339849_C	25C	45C
Z_7339850_C	24C	44C
Z_7339851_C	25C	44C
Z_7339852_T	23T	42T
Z_7339853_G	23G	42G
Z_7339854_A	22A	45A
Z_7339855_C	24C	45C
Z_7339856_C	24C	44C
Z_7339857_G	25G	47G
Z_7339858_A	24A	47A
Z_7339859_G	25G	48G
Z_7339860_A	23A	50A
Z_7339861_A	23A	50A
Z_7339862_G	23G	51G
Z_7339863_T	23T	50T
Z_7339864_A	25A	49A
Z_7339865_C	25C	50C
Z_7339866_A	25A	51A
Z_7339867_A	26A	54A
Z_7339868_A	26A	54A
Z_7339869_T	26T	55T
Z_7339870_C	26C	56C
Z_7339871_C	26C	56C
Z_7339872_A	26A	55A
Z_7339873_G	26G	57G
Z_7339874_A	26A	58A
Z_7339875_T	24T	59T
Z_7339876_C	23C	19A_1G_39C
Z_7339877_C	23C	59C
Z_7339878_G	23G	20A_1T_39G
Z_7339879_A	23A	60A
Z_7339880_A	23A	59A

Z_7339881_C	23C	58C
Z_7339882_A	23A	59A
Z_7339883_G	23G	61G
Z_7339884_A	24A	61A
Z_7339885_T	24T	61T
Z_7339886_G	25G	62G
Z_7339887_T	25T	63T
Z_7339888_G	25G	63G
Z_7339889_T	25T	64T
Z_7339890_A	24A	64A
Z_7339891_A	24A	64A
Z_7339892_A	26A	64A
Z_7339893_C	23C	65C
Z_7339894_A	27A	63A
Z_7339895_C	27C	63C
Z_7339896_A	26A	62A
Z_7339897_A	26A	63A
Z_7339898_A	26A	63A
Z_7339899_T	26T	64T
Z_7339900_T	26T	65T
Z_7339901_A	26A	67A
Z_7339902_A	25A	66A
Z_7339903_G	24G	65G
Z_7339904_G	25G	66G
Z_7339905_G	25C	28A_37G
Z_7339906_A	24A	64A
Z_7339907_C	24C	64C
Z_7339908_T	24T	64T
Z_7339909_T	22T	61T
Z_7339910_G	22C	35G_25C
Z_7339911_C	22C	60C
Z_7339912_G	22G	61G
Z_7339913_G	21G	63G
Z_7339914_T	23T	63T
Z_7339915_G	23G	64G
Z_7339916_A	23A	64A
Z_7339917_T	23T	65T
Z_7339918_G	22G	65G
Z_7339919_G	21G	63G
Z_7339920_A	22A	64A
Z_7339921_A	22A	63A
Z_7339922_C	23C	63C
Z_7339923_A	20A	62A

Z_7339924_G	21G	62G
Z_7339925_A	21A	63A
Z_7339926_G	20G	65G
Z_7339927_C	19C	65C
Z_7339928_A	22A	66A
Z_7339929_T	22T	66T
Z_7339930_A	23A	67A
Z_7339931_T	23T	68T
Z_7339932_T	24T	68T
Z_7339933_T	24T	67T
Z_7339934_T	23T	68T
Z_7339935_G	23G	66G
Z_7339936_G	23G	64G
Z_7339937_C	22C	63C
Z_7339938_T	21T	61T
Z_7339939_T	22T	61T
Z_7339940_C	22C	60C
Z_7339941_T	22T	32T_28C
Z_7339942_T	22T	62T
Z_7339943_A	22A	62A
Z_7339944_G	23G	61G
Z_7339945_A	23A	62A
Z_7339946_A	25A	62A
Z_7339947_A	27A	62A
Z_7339948_C	28C	63C
Z_7339949_T	28T	60T
Z_7339950_G	30G	59G
Z_7339951_G	31G	59G
Z_7339952_A	31A	57A
Z_7339953_G	31G	57G
Z_7339954_G	31G	56G
Z_7339955_G	32G	54G
Z_7339956_C	31C	55C
Z_7339957_A	30A	29A_25G
Z_7339958_A	30A	54A
Z_7339959_C	30C	54C
Z_7339960_A	32A	54A
Z_7339961_C	33C	54C
Z_7339962_C	30C	51C
Z_7339963_A	31A	51A
Z_7339964_A	30A	27A_23G
Z_7339965_G	30G	49G
Z_7339966_T	30T	48T

Z_7339967_A	30A	48A
Z_7339968_T	29T	47T
Z_7339969_A	30A	45A
Z_7339970_C	29C	45C
Z_7339971_T	30T	45T
Z_7339972_T	30T	45T
Z_7339973_T	29T	44T
Z_7339974_G	29G	44G
Z_7339975_C	29C	44C
Z_7339976_A	29A	44A
Z_7339977_G	28G	44G
Z_7339978_C	29C	41C
Z_7339979_T	30T	41T
Z_7339980_G	29G	40G
Z_7339981_C	30C	39C
Z_7339982_C	30C	38C
Z_7339983_C	29C	38C
Z_7339984_A	27A	38A
Z_7339985_A	27A	39A
Z_7339986_A	27A	39A
Z_7339987_A	27A	39A
Z_7339988_T	26T	39T
Z_7339989_C	25C	38C
Z_7339990_T	25T	38T
Z_7339991_G	25G	38G
Z_7339992_A	24A	37A
Z_7339993_G	24G	38G
Z_7339994_G	25G	37G
Z_7339995_A	23A	37A
Z_7339996_A	24A	34A
Z_7339997_G	24G	34G
Z_7339998_G	22G	34G
Z_7339999_C	22C	34C
Z_7340000_T	21T	34T
Z_7340001_G	21G	34G
Z_7340002_G	19G	34G
Z_7340003_T	18T	35T
Z_7340004_C	18C	37C
Z_7340005_T	18T	37T
Z_7340006_A	18A	37A
Z_7340007_C	18C	35C
Z_7340008_A	18A	33A
Z_7340009_A	18A	33A

Z_7340010_A	18A	31A
Z_7340011_T	18T	31T
Z_7340012_C	18C	30C
Z_7340013_C	18C	30C
Z_7340014_T	5A	30T
Z_7340015_T	5C	27T
Z_7340016_T	5C	26T
Z_7340017_T	5T	24T
Z_7340018_T	5T	24T
Z_7340019_T	5T	24T
Z_7340020_G	5G	23G
Z_7340021_A	5A	21A
Z_7340022_A	5A	21A
Z_7340023_A	5A	20A
Z_7340024_C	5C	18C
Z_7340025_G	4G	18G
Z_7340026_G	4G	17G
Z_7340027_G	4G	18G
Z_7340028_G	4G	17G
Z_7340029_G	4G	17G
Z_7340030_G	3G	17G
Z_7340031_G	2G	17G
Z_7340032_G	2G	17G
Z_7340033_G	2G	17G
Z_7340034_G	2G	17G
Z_7340035_G	2G	17G
Z_7340036_G	2G	18G
Z_7340037_G	2G	4A_14G
Z_7340038_A	2A	13A
Z_7340039_A	5A	10A
Z_7340040_T	8T	14T
Z_7340041_A	8A	16A
Z_7340042_T	12T	17T
Z_7340043_T	12T	18T
Z_7340044_T	12T	18T
Z_7340045_A	12A	18A
Z_7340046_T	12T	19T
Z_7340047_T	12T	21T
Z_7340048_T	12T	21T
Z_7340049_T	12T	21T
Z_7340050_A	15A	21A
Z_7340051_T	17T	20T
Z_7340052_A	17A	21A

Z_7340053_T	17T	21T
Z_7340054_T	18T	21T
Z_7340055_T	18T	21T
Z_7340056_A	18A	19A
Z_7340057_T	18T	21T
Z_7340058_T	18T	21T
Z_7340059_T	18T	22T
Z_7340060_A	19A	22A
Z_7340061_T	19T	22T
Z_7340062_T	19T	22T
Z_7340063_T	19T	22T
Z_7340064_A	19A	24A
Z_7340065_T	20T	25T
Z_7340066_T	20T	26T
Z_7340067_G	19G	26G
Z_7340068_G	21G	27G
Z_7340069_A	21A	27A
Z_7340070_C	21C	27C
Z_7340071_T	21T	29T
Z_7340072_T	21T	29T
Z_7340073_A	20A	30A
Z_7340074_T	19T	30T
Z_7340075_A	21A	32A
Z_7340076_T	22T	34T
Z_7340077_A	22A	34A
Z_7340078_C	22C	32C
Z_7340079_C	22C	34C
Z_7340080_G	26G	33G
Z_7340081_C	27C	33C
Z_7340082_C	27C	32C
Z_7340083_C	27C	31C
Z_7340084_C	29C	32C
Z_7340085_A	29A	34A
Z_7340086_T	29T	33T
Z_7340087_A	29A	35A
Z_7340088_G	29G	34G
Z_7340089_C	29C	34C
Z_7340090_G	26G	34G
Z_7340091_C	28C	35C
Z_7340092_T	28T	36T
Z_7340093_A	28A	36A
Z_7340094_C	26C	36C
Z_7340095_A	27A	38A

Z_7340096_A	27A	39A
Z_7340097_G	26G	38G
Z_7340098_C	25C	40C
Z_7340099_A	26A	41A
Z_7340100_C	26C	42C
Z_7340101_T	23T	41T
Z_7340102_C	21C	40C
Z_7340103_T	20T	40T
Z_7340104_C	20C	41C
Z_7340105_C	19C	41C
Z_7340106_G	20G	41G
Z_7340107_G	20G	41G
Z_7340108_G	19G	42G
Z_7340109_C	19C	41C
Z_7340110_G	18G	42G
Z_7340111_G	18G	42G
Z_7340112_T	18T	43T
Z_7340113_T	18T	44T
Z_7340114_T	18T	46T
Z_7340115_A	16A	48A
Z_7340116_C	17C	48C
Z_7340117_A	17A	48A
Z_7340118_A	16A	49A
Z_7340119_T	17T	49T
Z_7340120_T	16T	49T
Z_7340121_T	14T	48T
Z_7340122_A	14A	47A
Z_7340123_A	14A	48A
Z_7340124_T	14T	47T
Z_7340125_T	13T	47T
Z_7340126_A	14A	46A
Z_7340127_T	14T	47T
Z_7340128_A	10A_4G	47A
Z_7340129_C	14C	48C
Z_7340130_A	12A	48A
Z_7340131_G	10G	48G
Z_7340132_G	13G	48G
Z_7340133_C	14C	49C
Z_7340134_T	13T	49T
Z_7340135_A	12A	49A
Z_7340136_C	12C	48C
Z_7340137_A	12A	46A
Z_7340138_C	12C	46C

Z_7340139_A	13A	48A
Z_7340140_T	13T	49T
Z_7340141_T	13T	51T
Z_7340142_G	13G	51G
Z_7340143_C	12C	51C
Z_7340144_C	13C	51C
Z_7340145_C	11C	51C
Z_7340146_C	9C	51C
Z_7340147_C	9C	49C
Z_7340148_C	9C	50C
Z_7340149_C	8C	51C
Z_7340150_A	6A	51A
Z_7340151_G	6G	52G
Z_7340152_C	8C	53C
Z_7340153_A	8A	53A
Z_7340154_A	8A	52A
Z_7340155_G	8G	53G
Z_7340156_C	8C	53C
Z_7340157_T	8T	54T
Z_7340158_G	8G	54G
Z_7340159_G	8G	52G
Z_7340160_G	9G	50G
Z_7340161_T	9T	50T
Z_7340162_A	9A	30A_20C
Z_7340163_C	9C	49C
Z_7340164_T	9T	50T
Z_7340165_C	9C	51C
Z_7340166_A	11A	49A
Z_7340167_T	11T	49T
Z_7340168_T	11T	48T
Z_7340169_T	10T	49T
Z_7340170_T	10T	47T
Z_7340171_A	10A	45A
Z_7340172_C	10C	43C
Z_7340173_C	10C	46C
Z_7340174_G	10G	46G
Z_7340175_A	10A	45A
Z_7340176_C	10C	47C
Z_7340177_C	10C	49C
Z_7340178_T	11T	46T
Z_7340179_C	12C	48C
Z_7340180_G	12G	48G
Z_7340181_G	12G	47G

Z_7340182_A	13A	48A
Z_7340183_A	13A	48A
Z_7340184_G	13G	49G
Z_7340185_G	14G	50G
Z_7340186_A	14A	51A
Z_7340187_T	16T	53T
Z_7340188_G	16G	53G
Z_7340189_G	16G	52G
Z_7340190_A	18A	47A
Z_7340191_A	18A	50A
Z_7340192_G	18G	50G
Z_7340193_G	19G	48G
Z_7340194_C	19C	46C
Z_7340195_T	19T	46T
Z_7340196_G	19G	46G
Z_7340197_A	18A	46A
Z_7340198_G	21G	48G
Z_7340199_T	21T	46T
Z_7340200_C	21C	47C
Z_7340201_A	20A	47A
Z_7340202_A	22A	47A
Z_7340203_C	21C	47C
Z_7340204_C	20C	47C
Z_7340205_T	21T	47T
Z_7340206_T	21T	47T
Z_7340207_G	21G	47G
Z_7340208_A	21A	46A
Z_7340209_G	20G	46G
Z_7340210_C	20C	44C
Z_7340211_C	20C	44C
Z_7340212_A	20A	25A_20G
Z_7340213_G	20G	45G
Z_7340214_C	21C	45C
Z_7340215_T	22T	46T
Z_7340216_A	22A	47A
Z_7340217_C	24C	48C
Z_7340218_C	22C	49C
Z_7340219_T	24T	49T
Z_7340220_G	24G	49G
Z_7340221_G	24G	49G
Z_7340222_G	25G	50G
Z_7340223_A	25A	49A
Z_7340224_T	24T	49T

Z_7340225_T	24T	50T
Z_7340226_T	24T	50T
Z_7340227_G	24G	51G
Z_7340228_A	24A	49A
Z_7340229_A	24A	49A
Z_7340230_C	23C	48C
Z_7340231_C	23C	48C
Z_7340232_C	22C	49C
Z_7340233_C	23C	49C
Z_7340234_A	23A	47A
Z_7340235_G	23G	47G
Z_7340236_G	20G	44G
Z_7340237_T	20T	44T
Z_7340238_C	21C	44C
Z_7340239_G	23G	44G
Z_7340240_T	23T	44T
Z_7340241_G	23G	44G
Z_7340242_A	23A	43A
Z_7340243_G	6A_17G	43G
Z_7340244_C	23C	16A_42C
Z_7340245_A	23A	27A
Z_7340246_C	23C	41C
Z_7340247_A	23A	42A
Z_7340248_G	21G	42G
Z_7340249_T	21T	42T
Z_7340250_T	21T	42T
Z_7340251_T	21T	42T
Z_7340252_T	20T	42T
Z_7340253_A	20A	43A
Z_7340254_G	20G	44G
Z_7340255_C	18C	45C
Z_7340256_T	18T	46T
Z_7340257_T	17T	47T
Z_7340258_G	16G	48G
Z_7340259_C	16C	47C
Z_7340260_A	15A	46A
Z_7340261_G	14G	43G
Z_7340262_T	16T	46T
Z_7340263_A	15A	47A
Z_7340264_C	16C	46C
Z_7340265_A	18A	49A
Z_7340266_G	18G	49G
Z_7340267_C	16C	49C

Z_7340268_G	16G	50G
Z_7340269_T	15T	52T
Z_7340270_T	16T	52T
Z_7340271_T	16T	52T
Z_7340272_T	14T	51T
Z_7340273_A	14A	51A
Z_7340274_A	14A	50A
Z_7340275_C	14C	49C
Z_7340276_C	14C	49C
Z_7340277_A	14A	50A
Z_7340278_C	14C	51C
Z_7340279_T	14T	49T
Z_7340280_G	14G	47G
Z_7340281_C	14C	47C
Z_7340282_G	14G	47G
Z_7340283_C	14C	46C
Z_7340284_C	13C	45C
Z_7340285_A	12A	45A
Z_7340286_C	12C	45C
Z_7340287_G	10G	47G
Z_7340288_A	10A	46A
Z_7340289_G	10G	47G
Z_7340290_G	16G	47G
Z_7340291_C	16C	47C
Z_7340292_T	12T	49T
Z_7340293_G	13G	47G
Z_7340294_C	14C	47C
Z_7340295_T	15T	48T
Z_7340296_C	15C	48C
Z_7340297_T	10T	49T
Z_7340298_T	10T	48T
Z_7340299_G	12G	49G
Z_7340300_C	12C	49C
Z_7340301_T	13T	49T
Z_7340302_G	13G	49G
Z_7340303_A	13A	48A
Z_7340304_G	14G	48G
Z_7340305_C	14C	48C
Z_7340306_A	14A	47A
Z_7340307_C	15C	48C
Z_7340308_C	16C	49C
Z_7340309_C	16C	47C
Z_7340310_A	17A	46A

Z_7340311_G	19G	45G
Z_7340312_G	19G	46G
Z_7340313_G	19G	46G
Z_7340314_G	19G	45G
Z_7340315_A	19A	46A
Z_7340316_T	20T	47T
Z_7340317_G	20G	48G
Z_7340318_G	20G	48G
Z_7340319_C	20C	47C
Z_7340320_G	20A	47G
Z_7340321_G	20G	47G
Z_7340322_G	19G	47G
Z_7340323_C	19C	48C
Z_7340324_A	20A	47A
Z_7340325_A	20A	47A
Z_7340326_T	21T	48T
Z_7340327_G	22G	47G
Z_7340328_A	22A	47A
Z_7340329_G	23G	47G
Z_7340330_G	23G	48G
Z_7340331_G	23G	48G
Z_7340332_T	23T	47T
Z_7340333_C	23C	48C
Z_7340334_T	24C	29T_19C
Z_7340335_C	24C	48C
Z_7340336_A	22A	48A
Z_7340337_A	23A	49A
Z_7340338_G	23G	49G
Z_7340339_A	24A	50A
Z_7340340_G	24G	48G
Z_7340341_G	24G	48G
Z_7340342_G	24G	48G
Z_7340343_T	23T	48T
Z_7340344_C	24C	48C
Z_7340345_T	24T	48T
Z_7340346_A	24A	48A
Z_7340347_C	23C	47C
Z_7340348_A	24A	47A
Z_7340349_G	24G	46G
Z_7340350_G	24G	45G
Z_7340351_T	24T	43T
Z_7340352_A	24A	43A
Z_7340353_G	23G	43G

Z_7340354_G	23G	44G
Z_7340355_G	26G	45G
Z_7340356_G	26G	45G
Z_7340357_C	25C	45C
Z_7340358_T	23T	45T
Z_7340359_C	25C	44C
Z_7340360_A	25A	45A
Z_7340361_C	24C	47C
Z_7340362_C	25C	47C
Z_7340363_C	24C	44C
Z_7340364_C	22C	45C
Z_7340365_C	22C	45C
Z_7340366_C	23C	19A_41C
Z_7340367_A	24A	44A
Z_7340368_A	24A	44A
Z_7340369_A	24A	44A
Z_7340370_A	24A	45A
Z_7340371_A	23A	44A
Z_7340372_G	23G	44G
Z_7340373_C	21C	44C
Z_7340374_A	22A	46A
Z_7340375_A	23A	46A
Z_7340376_A	25A	46A
Z_7340377_T	24T	47T
Z_7340378_T	24T	48T
Z_7340379_T	25T	48T
Z_7340380_T	24T	48T
Z_7340381_C	23C	46C
Z_7340382_A	23A	49A
Z_7340383_T	22T	49T
Z_7340384_G	23G	48G
Z_7340385_C	23C	48C
Z_7340386_A	23A	47A
Z_7340387_G	23G	46G
Z_7340388_G	23G	45G
Z_7340389_T	25T	44T
Z_7340390_C	25C	44C
Z_7340391_T	26T	45T
Z_7340392_C	27C	45C
Z_7340393_T	27T	45T
Z_7340394_G	26G	46G
Z_7340395_A	27A	47A
Z_7340396_A	26A	48A

Z_7340397_A	26A	47A
Z_7340398_G	27G	49G
Z_7340399_A	26A	52A
Z_7340400_A	26A	56A
Z_7340401_A	26A	58A
Z_7340402_G	26G	57G
Z_7340403_T	26T	57T
Z_7340404_C	26C	58C
Z_7340405_C	27C	58C
Z_7340406_T	26T	58T
Z_7340407_A	27A	57A
Z_7340408_G	26G	57G
Z_7340409_A	26C	57A
Z_7340410_T	24T	59T
Z_7340411_C	25C	57C
Z_7340412_C	26C	58C
Z_7340413_A	26A	58A
Z_7340414_C	25C	58C
Z_7340415_A	26A	57A
Z_7340416_A	27A	58A
Z_7340417_G	27G	57G
Z_7340418_C	27C	57C
Z_7340419_G	27G	57G
Z_7340420_A	28A	57A
Z_7340421_C	29C	22T_36C
Z_7340422_C	29C	58C
Z_7340423_T	29T	58T
Z_7340424_C	31C	56C
Z_7340425_A	29A	57A
Z_7340426_G	30G	57G
Z_7340427_A	30A	57A
Z_7340428_G	31G	56G
Z_7340429_T	30T	57T
Z_7340430_C	30C	57C
Z_7340431_A	30A	56A_21T
Z_7340432_G	30G	55G
Z_7340433_G	30G	58G
Z_7340434_G	29G	57G
Z_7340435_G	31G	57G
Z_7340436_C	30C	56C
Z_7340437_C	30C	57C
Z_7340438_T	30G	57T
Z_7340439_C	28C	57C

Z_7340440_C	28C	59C
Z_7340441_T	28T	61T
Z_7340442_G	29G	62G
Z_7340443_G	29G	62G
Z_7340444_T	29T	62T
Z_7340445_T	28T	62T
Z_7340446_C	24C	62C
Z_7340447_C	25C	61C
Z_7340448_C	25C	60C
Z_7340449_T	24T	59T
Z_7340450_T	24T	59T
Z_7340451_T	23T	59T
Z_7340452_T	23T	58T
Z_7340453_C	24C	58C
Z_7340454_A	22A	55A
Z_7340455_G	22G	55G
Z_7340456_A	22A	53A
Z_7340457_C	22C	53C
Z_7340458_A	23A	54A
Z_7340459_G	24G	52G
Z_7340460_G	24G	52G
Z_7340461_C	24C	53C
Z_7340462_C	24C	54C
Z_7340463_T	23T	54T
Z_7340464_A	24A	52A
Z_7340465_C	23C	54C
Z_7340466_C	23C	54C
Z_7340467_G	22G	53G
Z_7340468_G	22G	52G
Z_7340469_T	22T	51T
Z_7340470_T	23T	52T
Z_7340471_C	23C	52C
Z_7340472_A	23A	51A
Z_7340473_C	23C	51C
Z_7340474_C	22C	51C
Z_7340475_G	21G	51G
Z_7340476_A	21A	51A
Z_7340477_G	20G	49G
Z_7340478_A	20A	50A
Z_7340479_C	20C	50C
Z_7340480_T	20T	49T
Z_7340481_G	21G	48G
Z_7340482_G	20G	49G

Z_7340483_G	21G	53G
Z_7340484_G	22G	54G
Z_7340485_A	21A	54A
Z_7340486_A	21A	52A
Z_7340487_A	21A	52A
Z_7340488_A	24A	51A
Z_7340489_G	24G	52G
Z_7340490_T	21T	51T
Z_7340491_T	23T	53T
Z_7340492_A	23A	55A
Z_7340493_C	23C	53C
Z_7340494_T	22T_41C	49T
Z_7340495_T	24T	49T
Z_7340496_T	25T	47T
Z_7340497_T	25T	48T
Z_7340498_T	26T	49T
Z_7340499_T	26T	48T
Z_7340500_T	27T	49T
Z_7340501_T	28T	48T
Z_7340502_T	28T	49T
Z_7340503_T	28T	49T
Z_7340504_C	29C	48C
Z_7340505_A	27A	46A
Z_7340506_T	29T	46T
Z_7340507_C	29C	47C
Z_7340508_C	28C	48C
Z_7340509_T	29T	48T
Z_7340510_C	28C	47C
Z_7340511_A	30A	48A
Z_7340512_T	30T	49T
Z_7340513_C	31C	49C
Z_7340514_C	31C	49C
Z_7340515_T	32T	48T
Z_7340516_C	31C	47C
Z_7340517_A	31A	47A
Z_7340518_C	30C	47C
Z_7340519_C	30C	48C
Z_7340520_A	31A	48A
Z_7340521_T	30T	49T
Z_7340522_C	29C	49C
Z_7340523_T	30T	50T
Z_7340524_T	31T	50T
Z_7340525_A	30A	50A

Z_7340526_G	29G	50G
Z_7340527_A	28A	48A
Z_7340528_A	28A	48A
Z_7340529_C	26C	49C
Z_7340530_T	26T	49T
Z_7340531_G	25G	50G
Z_7340532_C	25C	48C
Z_7340533_A	25A	48A
Z_7340534_G	24G	48G
Z_7340535_A	26A	47A
Z_7340536_G	25G	45G
Z_7340537_C	26C	41C
Z_7340538_T	25T	44T
Z_7340539_G	24G	44G
Z_7340540_G	23G	44G
Z_7340541_G	25G	44G
Z_7340542_A	26A	44A
Z_7340543_G	26G	44G
Z_7340544_G	24G	44G
Z_7340545_G	26G	44G
Z_7340546_A	25A	44A
Z_7340547_C	26C	44C
Z_7340548_C	26C	44C
Z_7340549_C	27C	44C
Z_7340550_T	25T	44T
Z_7340551_A	28A	44A
Z_7340552_T	28T	44T
Z_7340553_G	28G	43G
Z_7340554_G	27G	44G
Z_7340555_A	27A	45A
Z_7340556_A	24A	44A
Z_7340557_C	24C	43C
Z_7340558_A	24A	43A
Z_7340559_C	22C	45C
Z_7340560_A	22A	45A
Z_7340561_T	20T	44T
Z_7340562_C	20C	44C
Z_7340563_C	19C	43C
Z_7340564_G	17G	44G
Z_7340565_A	19A	42A
Z_7340566_G	18G	44G
Z_7340567_C	18C	43C
Z_7340568_G	16G	43G

Z_7340569_C	19C	44C
Z_7340570_T	18T	44T
Z_7340571_T	17T	44T
Z_7340572_G	17G	45G
Z_7340573_C	16C	46C
Z_7340574_C	15C	49C
Z_7340575_T	16T	49T
Z_7340576_T	15T	49T
Z_7340577_T	15T	48T
Z_7340578_C	15C	45C
Z_7340579_A	17A	39A
Z_7340580_C	16C	43C
Z_7340581_T	15T	43T
Z_7340582_T	15T	44T
Z_7340583_G	14G	44G
Z_7340584_C	14C	43C
Z_7340585_A	13A	43A
Z_7340586_T	13T	37T
Z_7340587_C	13C	37C
Z_7340588_T	13T	36T
Z_7340589_C	13C	37C
Z_7340590_C	13C	35C
Z_7340591_G	13G	32G
Z_7340592_T	13T	32T
Z_7340593_C	13C	33C
Z_7340594_C	12C	32C
Z_7340595_C	12C	30C
Z_7340596_C	13C	31C
Z_7340597_C	13C	30C
Z_7340598_C	15C	30C
Z_7340599_C	16C	30C
Z_7340600_C	17C	31C
Z_7340601_C	17C	30C
Z_7340602_C	17C	30C
Z_7340603_C	16C	29C
Z_7340604_A	16A	28A
Z_7340605_A	15A	31A
Z_7340606_A	15A	32A
Z_7340607_A	16A	32A
Z_7340608_A	16A	32A
Z_7340609_A	16A	33A
Z_7340610_A	16A	34A
Z_7340611_A	30A_14C	32A

Z_7340612_A	1A	19A_14C
Z_7340613_C	1A	34C
Z_7340614_A	17A	34A
Z_7340615_A	19A	34A
Z_7340616_T	19T	36T
Z_7340617_C	19C	37C
Z_7340618_A	19A	36A
Z_7340619_A	19A	36A
Z_7340620_T	21T	36T
Z_7340621_A	23A	36A
Z_7340622_A	22A	37A
Z_7340623_G	23G	38G
Z_7340624_G	21G	36G
Z_7340625_A	21A	37A
Z_7340626_T	21T	37T
Z_7340627_A	22A	37A
Z_7340628_A	22A	38A
Z_7340629_T	23T	37T
Z_7340630_A	24A	38A
Z_7340631_A	25A	39A
Z_7340632_C	25C	41C
Z_7340633_A	25A	40A
Z_7340634_A	25A	40A
Z_7340635_C	24C	41C
Z_7340636_A	24A	41A
Z_7340637_T	24T	41T
Z_7340638_G	26G	41G
Z_7340639_C	25C	43C
Z_7340640_T	26T	43T
Z_7340641_G	25G	44G
Z_7340642_G	26G	44G
Z_7340643_T	26T	44T
Z_7340644_C	26C	45C
Z_7340645_G	26G	45G
Z_7340646_G	27G	47G
Z_7340647_C	26C	48C
Z_7340648_A	27A	47A
Z_7340649_G	27G	49G
Z_7340650_G	27G	52G
Z_7340651_C	28C	53C
Z_7340652_T	28T	52T
Z_7340653_C	28C	52C
Z_7340654_T	28T	52T

Z_7340655_G	28G	50G
Z_7340656_T	29T	50T
Z_7340657_G	29G	52G
Z_7340658_A	29A	52A
Z_7340659_A	29A	51A
Z_7340660_A	29A	52A
Z_7340661_A	29A	52A
Z_7340662_C	28C	53C
Z_7340663_G	29G	52G
Z_7340664_T	30T	53T
Z_7340665_G	29G	52G
Z_7340666_T	29T	52T
Z_7340667_G	29G	52G
Z_7340668_C	30T	51C
Z_7340669_G	29G	46G
Z_7340670_A	30A	45A
Z_7340671_T	29T	46T
Z_7340672_T	29T	47T
Z_7340673_C	28C	46C
Z_7340674_G	28A	46G
Z_7340675_C	28C	47C
Z_7340676_C	29C	49C
Z_7340677_C	28C	49C
Z_7340678_C	28C	47C
Z_7340679_C	28C	48C
Z_7340680_T	28T	47T
Z_7340681_T	28T	47T
Z_7340682_C	28C	46C
Z_7340683_C	28C	47C
Z_7340684_C	28C	47C
Z_7340685_C	25C	47C
Z_7340686_G	26A	23A_24G
Z_7340687_C	28C	47C
Z_7340688_A	28A	47A
Z_7340689_G	30G	47G
Z_7340690_G	28G	48G
Z_7340691_G	26G	49G
Z_7340692_C	26C	50C
Z_7340693_A	26A	51A
Z_7340694_G	26G	52G
Z_7340695_G	26G	52G
Z_7340696_G	26G	53G
Z_7340697_G	25G	53G

Z_7340698_T	25T	53T
Z_7340699_C	24C	54C
Z_7340700_C	23C	53C
Z_7340701_A	22A	52A
Z_7340702_G	23G	51G
Z_7340703_A	23A	52A
Z_7340704_C	23G	52C
Z_7340705_G	24G	52G
Z_7340706_T	24T	51T
Z_7340707_C	25C	51C
Z_7340708_C	23C	51C
Z_7340709_A	24A	52A
Z_7340710_G	23G	54G
Z_7340711_A	23A	51A
Z_7340712_G	22G	51G
Z_7340713_A	23A	51A
Z_7340714_G	24G	50G
Z_7340715_C	24C	50C
Z_7340716_A	23A	50A
Z_7340717_C	23C	49C
Z_7340718_G	23G	48G
Z_7340719_G	23G	48G
Z_7340720_C	23C	48C
Z_7340721_T	21T	47T
Z_7340722_G	21G	47G
Z_7340723_G	21G	50G
Z_7340724_C	21C	51C
Z_7340725_T	21T	53T
Z_7340726_G	20G	52G
Z_7340727_T	20T	50T
Z_7340728_C	22C	52C
Z_7340729_A	22A	51A
Z_7340730_A	22A	51A
Z_7340731_G	23G	51G
Z_7340732_C	22C	51C
Z_7340733_C	23C	51C
Z_7340734_A	22A	50A
Z_7340735_G	22G	50G
Z_7340736_G	22G	48G
Z_7340737_C	23C	49C
Z_7340738_T	22T	49T
Z_7340739_G	23G	47G
Z_7340740_G	22G	49G

Z_7340741_A	23A	48A
Z_7340742_G	22G	47G
Z_7340743_A	22A	47A
Z_7340744_C	24C	47C
Z_7340745_C	23C	43C
Z_7340746_G	22G	43G
Z_7340747_C	22C	45C
Z_7340748_G	24C	24G_21C
Z_7340749_G	27G	45G
Z_7340750_G	27G	44G
Z_7340751_C	27C	44C
Z_7340752_T	27T	43T
Z_7340753_C	27C	44C
Z_7340754_C	25C	44C
Z_7340755_C	26C	42C
Z_7340756_C	27C	43C
Z_7340757_G	27G	42G
Z_7340758_C	27C	43C
Z_7340759_T	25T	43T
Z_7340760_C	27C	45C
Z_7340761_T	27T	47T
Z_7340762_A	27A	47A
Z_7340763_C	26C	48C
Z_7340764_C	25C	49C
Z_7340765_C	27C	48C
Z_7340766_C	27C	48C
Z_7340767_C	30C	50C
Z_7340768_T	30T	52T
Z_7340769_T	32T	52T
Z_7340770_T	32T	51T
Z_7340771_T	31T	52T
Z_7340772_T	32T	51T
Z_7340773_C	33C	51C
Z_7340774_T	32T	53T
Z_7340775_T	34T	54T
Z_7340776_C	33C	55C
Z_7340777_A	34A	54A
Z_7340778_A	34A	55A
Z_7340779_A	35A	55A
Z_7340780_T	35T	54T
Z_7340781_C	36C	55C
Z_7340782_A	36A	55A
Z_7340783_A	36A	56A

Z_7340784_G	34G	58G
Z_7340785_C	35C	58C
Z_7340786_A	36A	57A
Z_7340787_C	35C	58C
Z_7340788_C	35C	57C
Z_7340789_G	38G	58G
Z_7340790_C	39C	58C
Z_7340791_T	40T	57T
Z_7340792_T	39T	56T
Z_7340793_C	40C	56C
Z_7340794_C	38C	57C
Z_7340795_C	41C	58C
Z_7340796_A	41A	61A
Z_7340797_C	42C	61C
Z_7340798_A	40A	61A
Z_7340799_C	41C	62C
Z_7340800_G	41G	61G
Z_7340801_C	40C	63C
Z_7340802_A	41A	63A
Z_7340803_A	40A	64A
Z_7340804_A	41A	63A
Z_7340805_G	40G	60G
Z_7340806_G	40G	61G
Z_7340807_A	41A	62A
Z_7340808_A	42A	62A
Z_7340809_T	41T	63T
Z_7340810_G	41G	64G
Z_7340811_A	38A	64A
Z_7340812_G	41G	64G
Z_7340813_G	41G	64G
Z_7340814_G	38G	64G
Z_7340815_A	40A	63A
Z_7340816_G	40G	66G
Z_7340817_A	41A	67A
Z_7340818_T	40T	62T
Z_7340819_G	38G	64G
Z_7340820_T	38T	61T
Z_7340821_G	39G	62G
Z_7340822_C	39C	63C
Z_7340823_A	42A	62A
Z_7340824_A	40A	63A
Z_7340825_C	42C	63C
Z_7340826_T	42T	63T

Z_7340827_C	41C	63C
Z_7340828_A	41A	64A
Z_7340829_G	41G	64G
Z_7340830_C	40C	65C
Z_7340831_A	40A	65A
Z_7340832_G	39G	64G
Z_7340833_A	41A	66A
Z_7340834_G	42G	66G
Z_7340835_T	40T	67T
Z_7340836_C	39C	67C
Z_7340837_C	39C	68C
Z_7340838_G	39G	67G
Z_7340839_G	38G	67G
Z_7340840_C	38T	67C
Z_7340841_A	37A	67A
Z_7340842_G	38G	66G
Z_7340843_G	37G	66G
Z_7340844_G	37G	66G
Z_7340845_G	36G	67G
Z_7340846_A	36A	68A
Z_7340847_A	34A	69A
Z_7340848_C	37C	69C
Z_7340849_G	37G	70G
Z_7340850_C	35C	70C
Z_7340851_C	37C	69C
Z_7340852_A	37A	70A
Z_7340853_G	38G	69G
Z_7340854_A	38A	68A
Z_7340855_C	38C	67C
Z_7340856_C	37C	65C
Z_7340857_C	38C	65C
Z_7340858_A	38A	66A
Z_7340859_G	35G	66G
Z_7340860_G	36G	67G
Z_7340861_G	35G	69G
Z_7340862_C	35C	69C
Z_7340863_G	36G	69G
Z_7340864_T	37T	70T
Z_7340865_C	36C	72C
Z_7340866_C	35C	69C
Z_7340867_C	37C	70C
Z_7340868_C	38C	72C
Z_7340869_A	39A	71A

Z_7340870_G	39G	70G
Z_7340871_T	40T	71T
Z_7340872_C	40C	72C
Z_7340873_C	43C	71C
Z_7340874_G	44G	72G
Z_7340875_G	45G	73G
Z_7340876_C	45C	74C
Z_7340877_T	43T	75T
Z_7340878_C	45C	74C
Z_7340879_T	44T	74T
Z_7340880_T	45T	73T
Z_7340881_A	45A	73A
Z_7340882_T	46T	72T
Z_7340883_C	47C	72C
Z_7340884_T	49T	72T
Z_7340885_T	48T	71T
Z_7340886_C	47C	71C
Z_7340887_T	47T	71T
Z_7340888_C	46C	72C
Z_7340889_C	48C	71C
Z_7340890_C	47C	69C
Z_7340891_C	47C	67C
Z_7340892_G	46G	67G
Z_7340893_T	44T	37T_22G
Z_7340894_T	44T	59T
Z_7340895_C	47C	21T_38C
Z_7340896_A	47A	38A_21G
Z_7340897_A	47A	37A_21C
Z_7340898_C	48C	59C
Z_7340899_T	47T	59T
Z_7340900_C	47C	60C
Z_7340901_C	47C	62C
Z_7340902_T	46T	62T
Z_7340903_G	44G	62G
Z_7340904_G	43G	65G
Z_7340905_G	44G	68G
Z_7340906_A	47A	70A
Z_7340907_C	44C	71C
Z_7340908_A	45A	72A
Z_7340909_G	44G	73G
Z_7340910_A	45A	76A
Z_7340911_G	45G	75G
Z_7340912_A	44A	75A

Z_7340913_A	45A	76A
Z_7340914_T	45T	77T
Z_7340915_C	44C	78C
Z_7340916_T	47T	78T
Z_7340917_C	46C	80C
Z_7340918_G	44G	80G
Z_7340919_C	44C	81C
Z_7340920_C	46C	81C
Z_7340921_T	44T	81T
Z_7340922_G	44G	82G
Z_7340923_A	43A	82A
Z_7340924_T	43T	84T
Z_7340925_T	42T	84T
Z_7340926_C	42C	84C
Z_7340927_T	41T	85T
Z_7340928_T	41T	85T
Z_7340929_A	41A	83A
Z_7340930_A	43A	80A
Z_7340931_C	42C	81C
Z_7340932_A	43A	81A
Z_7340933_G	42G	81G
Z_7340934_A	41A	83A
Z_7340935_G	42G	84G
Z_7340936_T	44T	84T
Z_7340937_G	42G	86G
Z_7340938_T	42T	86T
Z_7340939_C	41C	87C
Z_7340940_T	43T	86T
Z_7340941_G	43G	84G
Z_7340942_T	44T	82T
Z_7340943_T	45T	82T
Z_7340944_G	45G	82G
Z_7340945_G	44G	82G
Z_7340946_G	44G	84G
Z_7340947_C	43C	83C
Z_7340948_C	43C	85C
Z_7340949_A	43A	86A
Z_7340950_G	43G	85G
Z_7340951_G	41G	89G
Z_7340952_G	41G	89G
Z_7340953_G	40G	87G
Z_7340954_A	40A	88A
Z_7340955_G	39G	90G

Z_7340956_G	40G	89G
Z_7340957_G	41G	89G
Z_7340958_A	43A	87A
Z_7340959_A	42A	87A
Z_7340960_A	43A	86A
Z_7340961_A	43A	86A
Z_7340962_C	42C	86C
Z_7340963_T	45T	85T
Z_7340964_G	43G	85G
Z_7340965_C	42C	85C
Z_7340966_C	39C	83C
Z_7340967_C	42C	82C
Z_7340968_C	41C	85C
Z_7340969_G	40G	88G
Z_7340970_T	42T	87T
Z_7340971_T	42T	88T
Z_7340972_G	43G	86G
Z_7340973_T	44T	88T
Z_7340974_T	48T	87T
Z_7340975_T	49T	88T
Z_7340976_G	49G	89G
Z_7340977_C	48C	89C
Z_7340978_C	47C	90C
Z_7340979_T	48T	90T
Z_7340980_G	47G	90G
Z_7340981_A	47A	90A
Z_7340982_C	46C	91C
Z_7340983_A	47A	90A
Z_7340984_A	47A	92A
Z_7340985_A	46A	93A
Z_7340986_A	43A	92A
Z_7340987_G	44G	93G
Z_7340988_A	44A	93A
Z_7340989_T	44T	91T
Z_7340990_G	42G	94G
Z_7340991_C	43C	95C
Z_7340992_A	43A	98A
Z_7340993_G	44G	97G
Z_7340994_T	44T	98T
Z_7340995_G	45G	98G
Z_7340996_T	44T	95T
Z_7340997_C	46C	98C
Z_7340998_T	45T	98T

Z_7340999_C	47C	98C
Z_7341000_C	45C	95C
Z_7341001_T	46T	93T
Z_7341002_A	48A	93A
Z_7341003_A	51A	93A
Z_7341004_G	50G	93G
Z_7341005_A	49A	89A
Z_7341006_T	48T	90T
Z_7341007_G	48G	92G
Z_7341008_C	45C	90C
Z_7341009_C	46C	90C
Z_7341010_G	45G	90G
Z_7341011_T	44T	89T
Z_7341012_T	42T	89T
Z_7341013_G	39G	89G
Z_7341014_T	39T	88T
Z_7341015_G	39G	91G
Z_7341016_G	39G	92G
Z_7341017_A	39A	91A
Z_7341018_T	39T	89T
Z_7341019_A	39A	87A
Z_7341020_C	38C	86C
Z_7341021_A	39A	86A
Z_7341022_T	39T	87T
Z_7341023_A	37A	87A
Z_7341024_T	37T	87T
Z_7341025_A	79A_126T	174A_116T
Z_7341026_T	45T	88T
Z_7341027_T	31T	79T
Z_7341028_T	31T	80T
Z_7341029_T	32T	80T
Z_7341030_T	31T	79T
Z_7341031_G	32G	78G
Z_7341032_C	33C	79C
Z_7341033_T	33T	80T
Z_7341034_C	34C	80C
Z_7341035_T	34T	80T
Z_7341036_C	37C	81C
Z_7341037_C	36C	81C
Z_7341038_C	34C	34T_48C
Z_7341039_G	35G	82G
Z_7341040_G	35G	82G
Z_7341041_G	35G	82G

Z_7341042_A	35A	80A
Z_7341043_G	36G	80G
Z_7341044_A	36A	80A
Z_7341045_T	36T	79T
Z_7341046_A	36A	78A
Z_7341047_C	37C	78C
Z_7341048_G	38G	79G
Z_7341049_G	39G	81G
Z_7341050_T	38T	80T
Z_7341051_C	38C	80C
Z_7341052_C	37C	81C
Z_7341053_C	37C	83C
Z_7341054_T	38T	84T
Z_7341055_T	38T	83T
Z_7341056_C	37C	80C
Z_7341057_C	37C	80C
Z_7341058_T	38T	79T
Z_7341059_C	38C	80C
Z_7341060_C	38C	79C
Z_7341061_G	37G	79G
Z_7341062_C	38C	79C
Z_7341063_C	37C	80C
Z_7341064_T	34T	77T
Z_7341065_T	32T	78T
Z_7341066_G	33G	76G
Z_7341067_T	34T	75T
Z_7341068_A	33A	74A
Z_7341069_A	33A	74A
Z_7341070_G	33G	73G
Z_7341071_A	31A	71A
Z_7341072_A	31A	71A
Z_7341073_T	32T	72T
Z_7341074_G	32G	72G
Z_7341075_C	32C	72C
Z_7341076_A	31A	73A
Z_7341077_G	31G	73G
Z_7341078_A	29A	70A
Z_7341079_T	30T	69T
Z_7341080_G	30G	68G
Z_7341081_G	30G	70G
Z_7341082_G	28G	69G
Z_7341083_G	5A_1T_22G	26A_41G
Z_7341084_A	28A	69A

Z_7341085_A	26A	66A
Z_7341086_A	26A	64A
Z_7341087_A	26A	62A
Z_7341088_A	24A	64A
Z_7341089_A	24A	64A
Z_7341090_G	24G	63G
Z_7341091_A	24A	64A
Z_7341092_A	25A	65A
Z_7341093_G	25G	65G
Z_7341094_G	25G	65G
Z_7341095_G	25G	63G
Z_7341096_A	25A	66A
Z_7341097_G	25G	68G
Z_7341098_A	25A	69A
Z_7341099_T	25T	43T_27C
Z_7341100_G	25G	70G
Z_7341101_G	25G	68G
Z_7341102_A	25A	67A
Z_7341103_C	25C	66C
Z_7341104_T	25T	66T
Z_7341105_C	23C	65C
Z_7341106_C	22C	64C
Z_7341107_C	22C	63C
Z_7341108_T	22T	64T
Z_7341109_T	22T	64T
Z_7341110_T	22T	64T
Z_7341111_A	22A	63A
Z_7341112_A	22A	62A
Z_7341113_C	22C	66C
Z_7341114_C	21C	67C
Z_7341115_C	21C	68C
Z_7341116_C	20C	69C
Z_7341117_C	22C	69C
Z_7341118_T	23T	71T
Z_7341119_G	22G	72G
Z_7341120_G	20G	75G
Z_7341121_G	21G	77G
Z_7341122_G	21G	78G
Z_7341123_A	21A	79A
Z_7341124_T	21T	78T
Z_7341125_A	23A	78A
Z_7341126_A	23A	79A
Z_7341127_G	23G	79G

Z_7341128_A	24A	80A
Z_7341129_A	23A	80A
Z_7341130_A	22A	82A
Z_7341131_G	22G	82G
Z_7341132_A	23A	83A
Z_7341133_A	24A	81A
Z_7341134_G	24G	83G
Z_7341135_G	25G	83G
Z_7341136_T	25T	82T
Z_7341137_G	24G	83G
Z_7341138_C	23C	83C
Z_7341139_T	23T	81T
Z_7341140_T	23T	84T
Z_7341141_C	24C	86C
Z_7341142_A	23A	88A
Z_7341143_T	24T	88T
Z_7341144_T	23T	88T
Z_7341145_C	23C	90C
Z_7341146_T	23T	90T
Z_7341147_C	25C	94C
Z_7341148_C	26C	91C
Z_7341149_T	25T	90T
Z_7341150_C	27C	90C
Z_7341151_T	26T	90T
Z_7341152_C	28C	89C
Z_7341153_T	30T	53T_36C
Z_7341154_G	30G	88G
Z_7341155_G	31G	89G
Z_7341156_G	31G	91G
Z_7341157_T	31T	88T
Z_7341158_T	31T	89T
Z_7341159_T	31T	90T
Z_7341160_T	30T	91T
Z_7341161_T	31T	91T
Z_7341162_C	30C	92C
Z_7341163_T	30T	92T
Z_7341164_A	30A	92A
Z_7341165_C	31C	95C
Z_7341166_A	30A	95A
Z_7341167_G	30G	95G
Z_7341168_T	30A	36A_59T
Z_7341169_A	30A	94A
Z_7341170_G	30G	95G

Z_7341171_G	31G	95G
Z_7341172_C	31C	95C
Z_7341173_G	30G	95G
Z_7341174_C	30C	94C
Z_7341175_C	30C	95C
Z_7341176_A	31A	92A
Z_7341177_A	29A	91A
Z_7341178_G	29G	91G
Z_7341179_A	30A	93A
Z_7341180_T	29T	93T
Z_7341181_G	29G	94G
Z_7341182_C	29C	96C
Z_7341183_G	31G	95G
Z_7341184_T	31T	95T
Z_7341185_T	32T	96T
Z_7341186_T	32T	92T
Z_7341187_G	30G	94G
Z_7341188_G	28G	95G
Z_7341189_A	28A	97A
Z_7341190_G	28G	97G
Z_7341191_A	30A	94A
Z_7341192_C	30C	92C
Z_7341193_C	30C	92C
Z_7341194_C	29C	91C
Z_7341195_T	26T	90T
Z_7341196_G	26G	91G
Z_7341197_G	26G	91G
Z_7341198_T	25T	90T
Z_7341199_C	26C	90C
Z_7341200_G	26G	90G
Z_7341201_T	26T	91T
Z_7341202_G	25G	91G
Z_7341203_T	24T	90T
Z_7341204_T	24T	91T
Z_7341205_T	23T	89T
Z_7341206_T	22T	90T
Z_7341207_G	22G	91G
Z_7341208_T	22T	89T
Z_7341209_T	22T	89T
Z_7341210_C	22C	88C
Z_7341211_A	23A	87A
Z_7341212_G	23G	85G
Z_7341213_G	22G	82G

Z_7341214_C	23C	82C
Z_7341215_G	23G	79G
Z_7341216_G	23G	77G
Z_7341217_T	22T	72T
Z_7341218_C	21C	75C
Z_7341219_C	20C	75C
Z_7341220_C	19C	77C
Z_7341221_C	20C	76C
Z_7341222_G	18G	76G
Z_7341223_C	16C	74C
Z_7341224_C	17C	73C
Z_7341225_G	18G	72G
Z_7341226_G	18G	71G
Z_7341227_G	18G	72G
Z_7341228_G	18G	72G
Z_7341229_A	18A	70A
Z_7341230_T	19T	70T
Z_7341231_C	18C	70C
Z_7341232_A	19A	69A
Z_7341233_A	19A	69A
Z_7341234_G	19G	69G
Z_7341235_A	18A	68A
Z_7341236_C	18C	66C
Z_7341237_C	18C	64C
Z_7341238_G	19G	63G
Z_7341239_A	20A	61A
Z_7341240_G	20G	60G
Z_7341241_G	20C	34G_25C
Z_7341242_A	21A	54A
Z_7341243_G	21G	54G
Z_7341244_A	21A	53A
Z_7341245_G	21G	52G
Z_7341246_C	22C	54C
Z_7341247_A	22A	55A
Z_7341248_G	22G	55G
Z_7341249_G	21G	54G
Z_7341250_T	22T	51T
Z_7341251_G	22G	50G
Z_7341252_G	21G	50G
Z_7341253_G	21G	50G
Z_7341254_T	21T	49T
Z_7341255_T	20T	48T
Z_7341256_G	20G	48G

Z_7341257_T	19T	47T
Z_7341258_T	19T	48T
Z_7341259_G	20G	48G
Z_7341260_T	20T	45T
Z_7341261_G	18G	46G
Z_7341262_A	19A	46A
Z_7341263_C	19C	46C
Z_7341264_C	19C	47C
Z_7341265_G	19G	49G
Z_7341266_T	19T	48T
Z_7341267_G	19G	50G
Z_7341268_T	19C	26T_24C
Z_7341269_G	18G	51G
Z_7341270_C	18C	50C
Z_7341271_C	18C	49C
Z_7341272_G	18G	49G
Z_7341273_A	18A	49A
Z_7341274_G	18G	49G
Z_7341275_A	18A	49A
Z_7341276_A	18A	47A
Z_7341277_A	18A	44A
Z_7341278_G	18G	43G
Z_7341279_A	18A	45A
Z_7341280_T	17T	45T
Z_7341281_C	16C	44C
Z_7341282_T	16T	43T
Z_7341283_C	16C	43C
Z_7341284_C	14C	41C
Z_7341285_C	14C	41C
Z_7341286_C	12C	42C
Z_7341287_C	20C	24A_1G_88C
Z_7341288_C	2C	38C
Z_7341289_T	2C	23T_14C
Z_7341290_C	2C	38C
Z_7341291_C	2C	38C
Z_7341292_A	2C	25A_13C
Z_7341293_C	2C	47C
Z_7341294_A	2C	47A
Z_7341295_A	8A	47A
Z_7341296_A	8A	47A
Z_7341297_A	9A	47A
Z_7341298_A	9A	48A
Z_7341299_A	10A	48A

Z_7341300_A	9A	50A
Z_7341301_A	10A	50A
Z_7341302_T	10T	51T
Z_7341303_G	10G	54G
Z_7341304_C	10C	54C
Z_7341305_A	10A	55A
Z_7341306_G	11G	56G
Z_7341307_T	13T	56T
Z_7341308_C	14C	58C
Z_7341309_C	14C	58C
Z_7341310_A	14A	58A
Z_7341311_A	15A	58A
Z_7341312_A	16A	58A
Z_7341313_G	16G	59G
Z_7341314_T	16T	60T
Z_7341315_T	15T	59T
Z_7341316_C	16C	59C
Z_7341317_C	15C	58C
Z_7341318_A	16A	61A
Z_7341319_C	15C	63C
Z_7341320_C	16C	63C
Z_7341321_T	18T	63T
Z_7341322_G	19G	58G
Z_7341323_G	20G	61G
Z_7341324_C	20C	61C
Z_7341325_C	20C	60C
Z_7341326_A	20A	60A
Z_7341327_G	22G	62G
Z_7341328_G	22G	61G
Z_7341329_G	22G	61G
Z_7341330_G	22G	60G
Z_7341331_C	21C	60C
Z_7341332_T	22T	60T
Z_7341333_A	23A	60A
Z_7341334_C	23C	61C
Z_7341335_A	23A	61A
Z_7341336_G	23G	60G
Z_7341337_G	25G	63G
Z_7341338_G	25G	63G
Z_7341339_C	25C	63C
Z_7341340_C	25C	63C
Z_7341341_T	26T	62T
Z_7341342_A	25A	61A

Z_7341343_C	25C	63C
Z_7341344_T	24T	63T
Z_7341345_T	25T	62T
Z_7341346_T	26T	62T
Z_7341347_A	26A	62A
Z_7341348_C	26C	62C
Z_7341349_A	26A	62A
Z_7341350_G	27G	60G
Z_7341351_G	27G	60G
Z_7341352_G	28G	61G
Z_7341353_C	28C	60C
Z_7341354_T	28T	60T
Z_7341355_G	29G	60G
Z_7341356_T	29T	63T
Z_7341357_T	29T	63T
Z_7341358_G	30G	63G
Z_7341359_T	30T	62T
Z_7341360_G	29G	61G
Z_7341361_C	28C	61C
Z_7341362_A	27A	62A
Z_7341363_A	27A	60A
Z_7341364_A	27A	59A
Z_7341365_T	28T	58T
Z_7341366_A	29A	57A
Z_7341367_G	28G	58G
Z_7341368_C	28C	58C
Z_7341369_C	27C	59C
Z_7341370_A	27A	59A
Z_7341371_C	27C	60C
Z_7341372_A	28A	60A
Z_7341373_A	28A	60A
Z_7341374_C	28C	59C
Z_7341375_A	28A	60A
Z_7341376_A	27A	58A
Z_7341377_G	25G	59G
Z_7341378_G	25G	60G
Z_7341379_C	26C	59C
Z_7341380_A	27A	60A
Z_7341381_T	26T	60T
Z_7341382_G	24G	62G
Z_7341383_T	25T	56T
Z_7341384_G	26G	60G
Z_7341385_C	27C	61C

Z_7341386_C	27C	61C
Z_7341387_C	27C	62C
Z_7341388_A	27A	59A
Z_7341389_A	27A	60A
Z_7341390_C	27C	61C
Z_7341391_G	25G	61G
Z_7341392_C	24C	62C
Z_7341393_C	25C	60C
Z_7341394_A	26A	60A
Z_7341395_C	28C	59C
Z_7341396_A	29A	59A
Z_7341397_A	27A	58A
Z_7341398_A	26A	53A
Z_7341399_C	28T	54C
Z_7341400_G	28G	54G
Z_7341401_C	28C	53C
Z_7341402_T	28T	52T
Z_7341403_C	28C	54C
Z_7341404_T	27T	55T
Z_7341405_T	26T	56T
Z_7341406_G	27G	56G
Z_7341407_T	25T	56T
Z_7341408_A	25A	56A
Z_7341409_C	25C	55C
Z_7341410_G	25G	55G
Z_7341411_A	24A	57A
Z_7341412_C	24C	57C
Z_7341413_G	24G	54G
Z_7341414_G	24G	52G
Z_7341415_C	26C	52C
Z_7341416_T	27T	52T
Z_7341417_T	27T	54T
Z_7341418_T	27T	54T
Z_7341419_T	26T	55T
Z_7341420_C	27C	54C
Z_7341421_A	27A	55A
Z_7341422_G	26G	53G
Z_7341423_G	27G	52G
Z_7341424_C	27C	52C
Z_7341425_C	27C	52C
Z_7341426_T	26T	53T
Z_7341427_T	25T	53T
Z_7341428_G	25G	54G

Z_7341429_T	25T	53T
Z_7341430_G	25G	53G
Z_7341431_G	25G	52G
Z_7341432_G	27G	50G
Z_7341433_T	27T	51T
Z_7341434_C	27C	51C
Z_7341435_T	26T	51T
Z_7341436_G	25G	51G
Z_7341437_G	26G	51G
Z_7341438_G	26G	50G
Z_7341439_C	25C	49C
Z_7341440_A	26A	49A
Z_7341441_G	27G	49G
Z_7341442_C	27C	49C
Z_7341443_C	27C	48C
Z_7341444_C	28C	50C
Z_7341445_A	28A	51A
Z_7341446_C	29T	23T_29C
Z_7341447_A	29A	50A
Z_7341448_C	29C	51C
Z_7341449_A	28A	51A
Z_7341450_A	28A	51A
Z_7341451_C	28C	46C
Z_7341452_G	27G	47G
Z_7341453_C	27C	47C
Z_7341454_T	26T	48T
Z_7341455_A	27A	48A
Z_7341456_G	28G	48G
Z_7341457_C	28C	48C
Z_7341458_T	27T	48T
Z_7341459_C	27C	50C
Z_7341460_C	29C	53C
Z_7341461_G	30G	53G
Z_7341462_G	31G	52G
Z_7341463_A	29A	52A
Z_7341464_T	28T	52T
Z_7341465_T	26T	52T
Z_7341466_C	28C	51C
Z_7341467_T	30T	51T
Z_7341468_G	31G	51G
Z_7341469_A	29A	51A
Z_7341470_G	30G	51G
Z_7341471_A	30A	51A

Z_7341472_G	32G	51G
Z_7341473_T	31T	48T
Z_7341474_G	31G	48G
Z_7341475_C	31C	47C
Z_7341476_T	30T	46T
Z_7341477_T	33T	13A_44T
Z_7341478_A	33A	44A
Z_7341479_A	33A	42A
Z_7341480_A	33A	41A
Z_7341481_A	33A	42A
Z_7341482_A	34A	41A
Z_7341483_A	82A_24G	41A
Z_7341484_A	10A	42A
Z_7341485_A	8A	43A
Z_7341486_G	1A	43G
Z_7341487_A	25A	44A
Z_7341488_A	25A	44A
Z_7341489_A	24A	45A
Z_7341490_A	25A	45A
Z_7341491_G	25G	45G
Z_7341492_A	26G	45A
Z_7341493_A	26A	47A
Z_7341494_A	27A	49A
Z_7341495_T	27T	49T
Z_7341496_A	29A	49A
Z_7341497_T	29T	49T
Z_7341498_T	31T	47T
Z_7341499_A	32A	44A
Z_7341500_C	33C	44C
Z_7341501_C	32C	43C
Z_7341502_A	33A	43A
Z_7341503_A	33A	42A
Z_7341504_A	33G	43A
Z_7341505_T	33T	44T
Z_7341506_T	33T	40T
Z_7341507_C	34C	41C
Z_7341508_T	35T	42T
Z_7341509_A	36A	43A
Z_7341510_A	36A	42A
Z_7341511_T	36T	41T
Z_7341512_C	35C	39C
Z_7341513_T	36T	38T
Z_7341514_A	37A	38A

Z_7341515_A	36A	40A
Z_7341516_C	35C	41C
Z_7341517_A	35A	42A
Z_7341518_C	34C	43C
Z_7341519_C	34C	43C
Z_7341520_A	34A	44A
Z_7341521_A	34A	45A
Z_7341522_G	35G	45G
Z_7341523_A	35A	45A
Z_7341524_C	33C	45C
Z_7341525_G	34G	45G
Z_7341526_G	35G	46G
Z_7341527_T	35T	45T
Z_7341528_G	35G	45G
Z_7341529_C	34C	45C
Z_7341530_C	33C	46C
Z_7341531_C	34C	46C
Z_7341532_G	33G	47G
Z_7341533_G	33G	47G
Z_7341534_A	32A	46A
Z_7341535_G	31G	47G
Z_7341536_T	30T	47T
Z_7341537_T	30T	47T
Z_7341538_G	32G	47G
Z_7341539_G	32G	46G
Z_7341540_C	28C	46C
Z_7341541_A	27G	23A_22G
Z_7341542_C	27C	45C
Z_7341543_T	26T	43T
Z_7341544_G	27G	43G
Z_7341545_A	27A	43A
Z_7341546_T	26T	43T
Z_7341547_T	26T	44T
Z_7341548_C	29C	43C
Z_7341549_C	29C	43C
Z_7341550_T	29T	43T
Z_7341551_G	27G	23G_21C
Z_7341552_G	28G	43G
Z_7341553_C	28C	43C
Z_7341554_A	29A	41A
Z_7341555_C	30C	39C
Z_7341556_G	31G	39G
Z_7341557_G	33G	39G

Z_7341558_G	33G	38G
Z_7341559_C	32C	38C
Z_7341560_A	33A	38A
Z_7341561_T	32T	39T
Z_7341562_C	33C	39C
Z_7341563_T	33T	38T
Z_7341564_A	32A	38A
Z_7341565_A	32A	37A
Z_7341566_A	30A	37A
Z_7341567_G	29G	38G
Z_7341568_G	29G	37G
Z_7341569_A	28A	40A
Z_7341570_A	28A	40A
Z_7341571_T	28T	41T
Z_7341572_C	28C	41C
Z_7341573_A	29A	41A
Z_7341574_A	29A	41A
Z_7341575_A	29A	41A
Z_7341576_G	28G	40G
Z_7341577_G	27G	40G
Z_7341578_T	27T	39T
Z_7341579_C	26C	39C
Z_7341580_A	26A	40A
Z_7341581_C	26C	39C
Z_7341582_T	26T	40T
Z_7341583_C	26C	40C
Z_7341584_A	25A	40A
Z_7341585_T	25T	40T
Z_7341586_T	25T	40T
Z_7341587_T	25T	41T
Z_7341588_C	25C	39C
Z_7341589_T	25T	37T
Z_7341590_G	24G	36G
Z_7341591_T	23T	36T
Z_7341592_G	22G	35G
Z_7341593_C	22C	35C
Z_7341594_T	22T	35T
Z_7341595_C	22C	35C
Z_7341596_T	21T	35T
Z_7341597_A	49A_28T_35G_56C	47A_12T_15G_24C
Z_7341598_G	14G	33G
Z_7341599_T	14T	32T
Z_7341600_G	1C	23G

Z_7341601_C	null	23C
Z_7341602_T	null	23T
Z_7341603_T	null	24T
Z_7341604_C	null	24C
Z_7341605_C	null	24C
Z_7341606_A	null	23A
Z_7341607_C	null	24C
Z_7341608_A	null	24A
Z_7341609_G	null	23G
Z_7341610_C	null	23C
Z_7341611_A	null	22A
Z_7341612_A	null	20A
Z_7341613_G	null	20G
Z_7341614_C	null	20C
Z_7341615_C	null	21C
Z_7341616_T	null	21T
Z_7341617_C	1C	21C
Z_7341618_G	1T	20G
Z_7341619_A	11A	26A
Z_7341620_G	11G	26G
Z_7341621_T	11T	26T
Z_7341622_C	11C	26C
Z_7341623_G	11G	26G
Z_7341624_T	12T	26T
Z_7341625_T	12T	26T
Z_7341626_T	11T	26T
Z_7341627_C	13C	24C
Z_7341628_C	13C	25C
Z_7341629_C	13C	25C
Z_7341630_C	13C	25C
Z_7341631_A	13A	25A
Z_7341632_G	13G	26G
Z_7341633_C	14C	27C
Z_7341634_T	14T	26T
Z_7341635_C	14C	26C
Z_7341636_T	14T	26T
Z_7341637_G	15G	26G
Z_7341638_A	14A	27A
Z_7341639_C	15C	27C
Z_7341640_G	15G	9A_18G
Z_7341641_C	15C	26C
Z_7341642_C	15C	27C
Z_7341643_T	15T	27T

Z_7341644_G	15G	28G
Z_7341645_G	15G	28G
Z_7341646_C	13C	27C
Z_7341647_A	14A	27A
Z_7341648_A	12A	27A
Z_7341649_C	11C	28C
Z_7341650_T	11T	28T
Z_7341651_T	12C	18T_10C
Z_7341652_T	12T	28T
Z_7341653_G	13G	28G
Z_7341654_C	13C	28C
Z_7341655_C	13C	28C
Z_7341656_C	13C	28C
Z_7341657_C	13C	27C
Z_7341658_A	13A	27A
Z_7341659_T	13C	18T_8C
Z_7341660_G	14G	27G
Z_7341661_G	13G	27G
Z_7341662_C	11C	28C
Z_7341663_T	13T	28T
Z_7341664_C	13C	25C
Z_7341665_T	12T	26T
Z_7341666_G	11G	25G
Z_7341667_C	11C	25C
Z_7341668_C	12C	25C
Z_7341669_A	13A	24A
Z_7341670_C	13C	23C
Z_7341671_C	12C	23C
Z_7341672_C	14C	23C
Z_7341673_T	14T	23T
Z_7341674_T	13T	23T
Z_7341675_G	13G	21G
Z_7341676_C	12C	21C
Z_7341677_C	11C	20C
Z_7341678_A	12A	20A
Z_7341679_A	11A	19A
Z_7341680_A	12A	19A
Z_7341681_G	12G	19G
Z_7341682_G	12G	18G
Z_7341683_C	12C	18C
Z_7341684_A	13A	18A
Z_7341685_A	13A	19A
Z_7341686_C	13C	19C

Z_7341687_T	12T	19T
Z_7341688_G	12G	19G
Z_7341689_A	12A	19A
Z_7341690_G	12G	19G
Z_7341691_C	12C	19C
Z_7341692_A	13A	18A
Z_7341693_T	14T	18T
Z_7341694_T	13T	18T
Z_7341695_C	13C	18C
Z_7341696_C	13C	18C
Z_7341697_T	12T	18T
Z_7341698_T	12T	17T
Z_7341699_C	12C	18C
Z_7341700_C	12C	18C
Z_7341701_C	12C	18C
Z_7341702_T	12T	18T
Z_7341703_C	11C	18C
Z_7341704_C	11C	18C
Z_7341705_A	11A	18A
Z_7341706_C	12C	17C
Z_7341707_T	11T	17T
Z_7341708_T	11T	17T
Z_7341709_C	11C	17C
Z_7341710_T	11T	16T
Z_7341711_C	12C	16C
Z_7341712_A	12A	16A
Z_7341713_G	12G	16G
Z_7341714_C	12C	16C
Z_7341715_C	12C	16C
Z_7341716_C	12C	15C
Z_7341717_T	11T	15T
Z_7341718_C	12C	15C
Z_7341719_T	12T	15T
Z_7341720_G	12G	14G
Z_7341721_C	11C	14C
Z_7341722_T	11T	14T
Z_7341723_C	10C	14C
Z_7341724_T	10T	14T
Z_7341725_G	10G	14G
Z_7341726_G	10G	15G
Z_7341727_A	10A	13A
Z_7341728_A	10A	12A
Z_7341729_A	9A	12A

Z_7341730_T	9T	12T
Z_7341731_C	8C	12C
Z_7341732_A	9A	12A
Z_7341733_T	6T_3G	10T
Z_7341734_C	6G_3C	3G_8C
Z_7341735_C	9C	11C
Z_7341736_C	9C	11C
Z_7341737_C	8C	10C
Z_7341738_C	8C	10C
Z_7341739_C	8C	9C
Z_7341740_C	7C	9C
Z_7341741_C	7C	9C
Z_7341742_C	6C	9C
Z_7341743_A	6A	9A
Z_7341744_C	6C	8C
Z_7341745_T	6T	8T
Z_7341746_C	6T	3T_5C
Z_7341747_C	6C	8C
Z_7341748_A	6A	8A
Z_7341749_A	6A	8A
Z_7341750_C	6C	8C
Z_7341751_C	6C	8C
Z_7341752_C	6C	8C
Z_7341753_A	6A	8A
Z_7341754_T	6T	8T
Z_7341755_C	5C	8C
Z_7341756_A	5A	7A
Z_7341757_G	5G	7G
Z_7341758_C	1G_4C	6C
Z_7341759_C	5C	7C
Z_7341760_T	5T_9C	5T_9C
Z_7341761_C	5C	6C
Z_7341762_C	5C	6C
Z_7341763_C	4C	6C
Z_7341764_C	4C	6C
Z_7341765_C	4C	7C
Z_7341766_C	4C	7C
Z_7341767_G	4G	8G
Z_7341768_C	4C	8C
Z_7341769_T	4T	7T
Z_7341770_T	4T	8T
Z_7341771_C	4C	8C
Z_7341772_A	4A	8A

Z_7341773_A	4A	8A
Z_7341774_A	3A	8A
Z_7341775_C	3C	8C
Z_7341776_A	3A	7A
Z_7341777_C	3C	8C
Z_7341778_T	2T	8T
Z_7341779_G	2G	8G
Z_7341780_C	2C	7C
Z_7341781_C	2C	7C
Z_7341782_A	2A	8A
Z_7341783_C	2C	8C
Z_7341784_C	2C	8C
Z_7341785_A	2A	8A
Z_7341786_T	1T	8T
Z_7341787_C	1C	8C
Z_7341788_T	1T	9T
Z_7341789_G	1G	9G
Z_7341790_T	1T	9T
Z_7341791_A	1A	9A
Z_7341792_T	1T	9T
Z_7341793_T	1T	10T
Z_7341794_C	1C	9C
Z_7341795_T	2T	9T
Z_7341796_C	2C	9C
Z_7341797_T	2T	9T
Z_7341798_C	2C	9C
Z_7341799_A	2A	9A
Z_7341800_C	2C	9C
Z_7341801_G	2G	9G
Z_7341802_C	2C	9C
Z_7341803_A	2A	9A
Z_7341804_C	2C	9C
Z_7341805_C	2C	10C
Z_7341806_A	2A	9A
Z_7341807_T	2C	6T_3C
Z_7341808_G	2G	9G
Z_7341809_G	2G	9G
Z_7341810_A	2A	9A
Z_7341811_G	2G	9G
Z_7341812_T	2T	8T_3C
Z_7341813_C	2C	7C
Z_7341814_C	2C	9C
Z_7341815_C	3C	9C

Z_7341816_C	3C	9C
Z_7341817_C	3C	9C
Z_7341818_C	3C	8C
Z_7341819_C	3C	8C
Z_7341820_C	3C	8C
Z_7341821_C	3C	9C
Z_7341822_C	3C	9C
Z_7341823_C	3C	9C
Z_7341824_C	3C	11C
Z_7341825_G	1G_1C	11G
Z_7341826_T	2T	11T
Z_7341827_T	2T	11T
Z_7341828_C	2C	11C
Z_7341829_A	2A	11A
Z_7341830_C	2C	11C
Z_7341831_T	2T	12T
Z_7341832_A	1A_1T	12A
Z_7341833_A	2A	13A
Z_7341834_C	2C	13C
Z_7341835_A	3A	13A
Z_7341836_C	3C	13C
Z_7341837_T	3T	14T
Z_7341838_T	4T	14T
Z_7341839_C	4C	14C
Z_7341840_C	5C	16C
Z_7341841_A	5A	16A
Z_7341842_T	5T	16T
Z_7341843_A	6A	17A
Z_7341844_G	6G	17G
Z_7341845_C	6C	17C
Z_7341846_G	6G	16G
Z_7341847_C	7C	17C
Z_7341848_A	6A	17A
Z_7341849_A	6A	17A
Z_7341850_C	6C	19C
Z_7341851_A	7A	19A
Z_7341852_A	7A	20A
Z_7341853_A	7A	21A
Z_7341854_A	7A	21A
Z_7341855_G	7G	21G
Z_7341856_A	8A	20A
Z_7341857_G	8G	21G
Z_7341858_A	9A	21A

Z_7341859_G	9G	21G
Z_7341860_C	8C	21C
Z_7341861_C	10C	21C
Z_7341862_G	10G	20G
Z_7341863_G	10G	19G
Z_7341864_G	10G	18G
Z_7341865_A	10A	20A
Z_7341866_T	10T	21T
Z_7341867_G	9G	22G
Z_7341868_A	10A	23A
Z_7341869_T	10T	24T
Z_7341870_G	10G	24G
Z_7341871_A	11A	23A
Z_7341872_T	13T	26T
Z_7341873_G	12G	26G
Z_7341874_G	12G	27G
Z_7341875_A	14A	27A
Z_7341876_C	14C	27C
Z_7341877_A	14A	26A
Z_7341878_A	14A	27A
Z_7341879_T	14T	28T
Z_7341880_G	14G	28G
Z_7341881_G	14G	29G
Z_7341882_A	14A	29A
Z_7341883_G	15G	29G
Z_7341884_A	15A	29A
Z_7341885_T	15T	30T
Z_7341886_G	15G	30G
Z_7341887_C	15C	31C
Z_7341888_A	15A	30A
Z_7341889_T	16T	30T
Z_7341890_C	16C	30C
Z_7341891_A	16A	31A
Z_7341892_T	16T	31T
Z_7341893_A	16A	32A
Z_7341894_C	16C	32C
Z_7341895_A	16A	32A
Z_7341896_C	16C	32C
Z_7341897_A	16A	32A
Z_7341898_C	16C	33C
Z_7341899_G	15G	33G
Z_7341900_C	19C	32C
Z_7341901_A	19A	31A

Z_7341902_C	19C	31C
Z_7341903_T	19T	31T
Z_7341904_C	20C	32C
Z_7341905_C	17C	32C
Z_7341906_A	17G	20A_12G
Z_7341907_A	18A	32A
Z_7341908_G	19G	32G
Z_7341909_C	17C	33C
Z_7341910_A	16A	34A
Z_7341911_C	16C	35C
Z_7341912_C	16C	35C
Z_7341913_C	15C	35C
Z_7341914_C	15C	35C
Z_7341915_T	15T	35T
Z_7341916_T	16T	34T
Z_7341917_A	15A	34A
Z_7341918_C	15C	35C
Z_7341919_A	15A	33A
Z_7341920_T	16T	33T
Z_7341921_A	16A	33A
Z_7341922_C	16C	33C
Z_7341923_A	16A	33A
Z_7341924_C	17C	34C
Z_7341925_A	18A	35A
Z_7341926_T	17T	34T
Z_7341927_A	17A	34A
Z_7341928_C	16T	33C
Z_7341929_C	15C	33C
Z_7341930_C	16C	35C
Z_7341931_T	15T	37T
Z_7341932_T	16T	36T
Z_7341933_C	16C	37C
Z_7341934_C	15C	40C
Z_7341935_A	15A	38A
Z_7341936_C	15C	38C
Z_7341937_A	16A	38A
Z_7341938_C	16C	37C
Z_7341939_T	16T	37T
Z_7341940_T	16T	36T
Z_7341941_C	15C	36C
Z_7341942_T	13T	36T
Z_7341943_A	13A	37A
Z_7341944_C	13C	37C

Z_7341945_C	13T	12T_23C
Z_7341946_G	13G	36G
Z_7341947_C	13C	34C
Z_7341948_C	13C	34C
Z_7341949_A	13A	34A
Z_7341950_T	13T	34T
Z_7341951_T	14T	34T
Z_7341952_G	14G	33G
Z_7341953_T	14T	33T
Z_7341954_G	14G	33G
Z_7341955_T	13T	33T
Z_7341956_G	13G	33G
Z_7341957_A	13C	33A
Z_7341958_G	13G	33G
Z_7341959_G	13G	33G
Z_7341960_C	13C	32C
Z_7341961_T	12T	31T
Z_7341962_C	13C	30C
Z_7341963_T	13T	29T
Z_7341964_T	13T	28T
Z_7341965_C	13C	29C
Z_7341966_T	13T	29T
Z_7341967_A	13A	27A
Z_7341968_A	13A	27A
Z_7341969_T	13T	26T
Z_7341970_G	11G	27G
Z_7341971_G	11G	27G
Z_7341972_A	12A	27A
Z_7341973_A	12A	26A
Z_7341974_A	13A	26A
Z_7341975_G	13G	28G
Z_7341976_A	13A	27A
Z_7341977_A	12A	26A
Z_7341978_G	13G	28G
Z_7341979_A	12G	29A
Z_7341980_C	13C	27C
Z_7341981_A	13A	28A
Z_7341982_G	13G	28G
Z_7341983_A	13A	28A
Z_7341984_T	13T	29T
Z_7341985_G	13G	30G
Z_7341986_T	12T	29T
Z_7341987_G	12G	29G

Z_7341988_G	12G	29G
Z_7341989_T	13T	30T
Z_7341990_G	12G	30G
Z_7341991_C	12C	30C
Z_7341992_T	12T	30T
Z_7341993_A	12A	29A
Z_7341994_A	11A	29A
Z_7341995_A	10A	30A
Z_7341996_A	10A	30A
Z_7341997_T	10T	30T
Z_7341998_A	11A	31A
Z_7341999_G	11G	30G
Z_7342000_T	11T	30T
Z_7342001_A	12A	33A
Z_7342002_A	11A	33A
Z_7342003_A	11A	34A
Z_7342004_C	13C	33C
Z_7342005_C	13C	32C
Z_7342006_A	13A	32A
Z_7342007_C	12C	32C
Z_7342008_A	12A	32A
Z_7342009_T	12T	34T
Z_7342010_G	12G	35G
Z_7342011_A	12A	35A
Z_7342012_T	12T	35T
Z_7342013_C	13C	35C
Z_7342014_C	13C	16T_35C
Z_7342015_C	12C	34C
Z_7342016_C	14C	34C
Z_7342017_C	14C	34C
Z_7342018_G	15G	47G
Z_7342019_G	15G	19G
Z_7342020_G	15G	36G
Z_7342021_G	16G	34G
Z_7342022_G	16G	36G
Z_7342023_A	15A	36A
Z_7342024_G	15G	35G
Z_7342025_A	15A	35A
Z_7342026_A	15A	34A
Z_7342027_A	15A	34A
Z_7342028_A	15A	34A
Z_7342029_A	14A	32A
Z_7342030_G	14G	30G

Z_7342031_G	14G	30G
Z_7342032_A	14A	31A
Z_7342033_A	14A	31A
Z_7342034_G	24A_14G_1C	31G
Z_7342035_G	11G	15A_16G
Z_7342036_G	10A	31G
Z_7342037_A	10A_10G	31A
Z_7342038_A	18A	34A
Z_7342039_A	18A	35A
Z_7342040_A	19A	37A
Z_7342041_A	20A	37A
Z_7342042_A	20A	36A
Z_7342043_A	20A	37A
Z_7342044_A	20A	37A
Z_7342045_A	20A	37A
Z_7342046_G	20G	37G
Z_7342047_G	19G	37G
Z_7342048_A	20A	37A
Z_7342049_A	21A	37A
Z_7342050_T	21T	38T
Z_7342051_C	20C	40C
Z_7342052_A	21A	40A
Z_7342053_A	21A	41A
Z_7342054_G	21G	41G
Z_7342055_C	21C	43C
Z_7342056_A	21A	46A
Z_7342057_A	21A	46A
Z_7342058_T	20T	47T
Z_7342059_C	20C	48C
Z_7342060_A	21A	47A
Z_7342061_A	21A	48A
Z_7342062_G	21G	50G
Z_7342063_C	20C	50C
Z_7342064_A	21A	50A
Z_7342065_A	21A	51A
Z_7342066_T	23T	51T
Z_7342067_G	22G	51G
Z_7342068_C	22C	51C
Z_7342069_T	23T	52T
Z_7342070_T	24T	53T
Z_7342071_A	22A	52A
Z_7342072_T	22T	52T
Z_7342073_G	22G	52G

Z_7342074_G	23G	50G
Z_7342075_A	23A	47A
Z_7342076_C	23C	49C
Z_7342077_C	23C	50C
Z_7342078_A	24A	49A
Z_7342079_A	24A	51A
Z_7342080_G	23G	51G
Z_7342081_G	23G	50G
Z_7342082_A	23A	49A
Z_7342083_A	22A	50A
Z_7342084_G	22G	50G
Z_7342085_A	21A	49A
Z_7342086_A	21A	49A
Z_7342087_A	21A	51A
Z_7342088_A	19A	51A
Z_7342089_G	19G	52G
Z_7342090_A	19A	52A
Z_7342091_A	20A	52A
Z_7342092_A	22A	52A
Z_7342093_G	21G	53G
Z_7342094_A	22A	53A
Z_7342095_A	23A	51A
Z_7342096_G	23G	53G
Z_7342097_G	23G	52G
Z_7342098_T	23T	50T
Z_7342099_T	24T	49T
Z_7342100_C	23C	54C
Z_7342101_T	22T	54T
Z_7342102_G	23G	55G
Z_7342103_G	22G	57G
Z_7342104_G	21G	57G
Z_7342105_G	21G	54G
Z_7342106_A	21A	53A
Z_7342107_G	21G	55G
Z_7342108_G	18G	55G
Z_7342109_A	19A	54A
Z_7342110_A	18G	31A_23G
Z_7342111_G	17G	56G
Z_7342112_G	18G	56G
Z_7342113_G	18G	56G
Z_7342114_G	19G	56G
Z_7342115_G	19G	53G
Z_7342116_G	20G	55G

Z_7342117_T	19G	33T_25G
Z_7342118_T	20T	60T
Z_7342119_T	19T	61T
Z_7342120_G	20G	63G
Z_7342121_G	20G	64G
Z_7342122_G	20G	66G
Z_7342123_G	20G	65G
Z_7342124_A	20A	66A
Z_7342125_G	21G	68G
Z_7342126_A	20A	70A
Z_7342127_G	19G	70G
Z_7342128_G	20G	70G
Z_7342129_T	21T	72T
Z_7342130_T	21T	69T
Z_7342131_T	21T	71T
Z_7342132_T	22T	74T
Z_7342133_A	23A	74A
Z_7342134_T	23T	75T
Z_7342135_G	23G	73G
Z_7342136_G	22G	73G
Z_7342137_C	21C	73C
Z_7342138_A	22A	73A
Z_7342139_T	23T	75T
Z_7342140_C	22C	76C
Z_7342141_T	23T	78T
Z_7342142_A	24A	78A
Z_7342143_T	25T	81T
Z_7342144_A	24A	80A
Z_7342145_G	26G	80G
Z_7342146_A	27A	77A
Z_7342147_G	27G	80G
Z_7342148_A	27A	81A
Z_7342149_G	27G	81G
Z_7342150_A	28A	79A
Z_7342151_G	26G	76G
Z_7342152_G	28G	75G
Z_7342153_A	29A	75A
Z_7342154_A	29A	74A
Z_7342155_G	27G	74G
Z_7342156_G	28G	73G
Z_7342157_C	30C	73C
Z_7342158_T	30T	73T
Z_7342159_A	30A	73A

Z_7342160_T	30T	72T
Z_7342161_G	29G	70G
Z_7342162_G	26G	70G
Z_7342163_G	25G	72G
Z_7342164_A	26A	71A
Z_7342165_T	25T	68T
Z_7342166_G	25G	67G
Z_7342167_G	25G	66G
Z_7342168_G	24G	67G
Z_7342169_A	24A	67A
Z_7342170_G	24G	68G
Z_7342171_T	23T	68T
Z_7342172_T	24T	67T
Z_7342173_C	24C	67C
Z_7342174_A	25A	65A
Z_7342175_A	25A	66A
Z_7342176_G	26G	66G
Z_7342177_A	26A	65A
Z_7342178_G	26G	66G
Z_7342179_A	26A	67A
Z_7342180_C	26C	70C
Z_7342181_T	26T	70T
Z_7342182_T	25T	69T
Z_7342183_G	26G	68G
Z_7342184_A	26A	67A
Z_7342185_G	26G	66G
Z_7342186_A	26A	66A
Z_7342187_A	27A	67A
Z_7342188_C	27C	67C
Z_7342189_A	28A	66A
Z_7342190_T	28T	65T
Z_7342191_T	30T	66T
Z_7342192_G	30G	65G
Z_7342193_A	29A	66A
Z_7342194_G	31G	65G
Z_7342195_T	29T	64T
Z_7342196_T	31T	63T
Z_7342197_G	30G	63G
Z_7342198_T	31T	61T
Z_7342199_T	30T	63T
Z_7342200_G	30G	64G
Z_7342201_C	30C	63C
Z_7342202_C	29C	64C

Z_7342203_A	29A	65A
Z_7342204_T	30T	64T
Z_7342205_T	28T	64T
Z_7342206_T	30T	66T
Z_7342207_T	30T	67T
Z_7342208_C	29C	67C
Z_7342209_A	28A	69A
Z_7342210_G	28G	69G
Z_7342211_G	27G	69G
Z_7342212_G	28G	67G
Z_7342213_A	26A	64A
Z_7342214_A	27A	64A
Z_7342215_T	26T	64T
Z_7342216_A	27A	63A
Z_7342217_A	27A	61A
Z_7342218_T	27T	61T
Z_7342219_C	28C	61C
Z_7342220_C	27C	61C
Z_7342221_G	30G	59G
Z_7342222_A	28A	58A
Z_7342223_A	28A	61A
Z_7342224_G	28G	59G
Z_7342225_A	26A	60A
Z_7342226_G	27G	62G
Z_7342227_C	29C	59C
Z_7342228_C	30C	59C
Z_7342229_G	29G	57G
Z_7342230_C	31C	58C
Z_7342231_C	31C	60C
Z_7342232_T	30T	60T
Z_7342233_C	31C	59C
Z_7342234_G	31G	58G
Z_7342235_G	30G	57G
Z_7342236_A	34A	56A
Z_7342237_T	35T	56T
Z_7342238_A	35A	53A
Z_7342239_A	34A	56A
Z_7342240_A	36A	55A
Z_7342241_G	36G	56G
Z_7342242_C	37C	55C
Z_7342243_G	37G	55G
Z_7342244_G	38G	55G
Z_7342245_C	37C	56C

Z_7342246_T	36T	55T
Z_7342247_C	35C	57C
Z_7342248_C	39C	57C
Z_7342249_C	36C	57C
Z_7342250_C	39C	59C
Z_7342251_A	40A	61A
Z_7342252_T	41T	59T
Z_7342253_C	41C	59C
Z_7342254_G	39G	61G
Z_7342255_T	40T	61T
Z_7342256_G	40G	62G
Z_7342257_G	41G	63G
Z_7342258_C	41C	62C
Z_7342259_T	42T	66T
Z_7342260_C	42C	67C
Z_7342261_A	41A	68A
Z_7342262_T	41T	68T
Z_7342263_G	41G	68G
Z_7342264_A	40A	67A
Z_7342265_T	43T	66T
Z_7342266_T	42T	67T
Z_7342267_C	41C	68C
Z_7342268_A	45A	69A
Z_7342269_C	45C	70C
Z_7342270_A	45A	69A
Z_7342271_C	44C	70C
Z_7342272_A	47A	70A
Z_7342273_A	47A	70A
Z_7342274_C	45C	70C
Z_7342275_T	46T	68T
Z_7342276_T	44T	68T
Z_7342277_C	45C	67C
Z_7342278_C	45C	67C
Z_7342279_C	46C	67C
Z_7342280_T	48T	69T
Z_7342281_G	48G	68G
Z_7342282_T	47T	67T
Z_7342283_A	47A	67A
Z_7342284_C	46C	67C
Z_7342285_T	47T	66T
Z_7342286_T	45T	66T
Z_7342287_T	45T	67T
Z_7342288_G	44G	65G

Z_7342289_C	42C	65C
Z_7342290_A	44A	68A
Z_7342291_T	40T	70T
Z_7342292_T	42T	71T
Z_7342293_G	43G	71G
Z_7342294_T	42T	70T
Z_7342295_G	44G	70G
Z_7342296_T	44T	70T
Z_7342297_T	42T	70T
Z_7342298_T	42T	72T
Z_7342299_G	42G	74G
Z_7342300_C	41C	75C
Z_7342301_G	41C	41G_34C
Z_7342302_T	41T	75T
Z_7342303_G	41G	77G
Z_7342304_G	42G	75G
Z_7342305_G	41G	75G
Z_7342306_C	40C	75C
Z_7342307_A	39A	75A
Z_7342308_G	40G	75G
Z_7342309_C	41C	75C
Z_7342310_A	41A	74A
Z_7342311_A	40A	75A
Z_7342312_A	41A	74A
Z_7342313_C	41C	75C
Z_7342314_C	40C	72C
Z_7342315_T	39T	72T
Z_7342316_G	39G	72G
Z_7342317_G	40G	72G
Z_7342318_G	39G	70G
Z_7342319_C	40C	70C
Z_7342320_G	40G	69G
Z_7342321_G	41G	67G
Z_7342322_G	40G	67G
Z_7342323_G	38G	67G
Z_7342324_A	65A_27T	91A_24T
Z_7342325_A	41A	68A
Z_7342326_T	41T	66T
Z_7342327_A	42A	66A
Z_7342328_T	42T	65T
Z_7342329_A	43A	65A
Z_7342330_G	38T	24T_36G
Z_7342331_A	40A	60A

Z_7342332_C	39C	61C
Z_7342333_T	39T	60T
Z_7342334_C	40C	62C
Z_7342335_C	40C	63C
Z_7342336_T	40T	63T
Z_7342337_G	41G	63G
Z_7342338_C	40C	61C
Z_7342339_T	41T	61T
Z_7342340_G	41G	62G
Z_7342341_T	41T	63T
Z_7342342_T	42T	66T
Z_7342343_G	43G	65G
Z_7342344_C	43C	66C
Z_7342345_C	43C	68C
Z_7342346_A	43A	67A
Z_7342347_T	42T	66T
Z_7342348_T	40T	67T
Z_7342349_T	40T	66T
Z_7342350_T	40T	70T
Z_7342351_A	41A	71A
Z_7342352_C	41C	70C
Z_7342353_A	40A	71A
Z_7342354_G	40G	70G
Z_7342355_A	40A	71A
Z_7342356_T	41T	70T
Z_7342357_T	43T	71T
Z_7342358_A	41A	72A
Z_7342359_A	43A	72A
Z_7342360_T	41T	72T
Z_7342361_T	43T	72T
Z_7342362_A	44A	74A
Z_7342363_A	42A	75A
Z_7342364_C	44C	78C
Z_7342365_A	46A	77A
Z_7342366_G	46G	76G
Z_7342367_A	45A	78A
Z_7342368_G	46G	78G
Z_7342369_G	45G	77G
Z_7342370_T	46T	78T
Z_7342371_G	46G	80G
Z_7342372_G	45G	80G
Z_7342373_A	44A	80A
Z_7342374_G	44G	83G

Z_7342375_G	44G	82G
Z_7342376_G	42G	82G
Z_7342377_C	40C	82C
Z_7342378_A	41A	83A
Z_7342379_A	39A	84A
Z_7342380_A	39A	84A
Z_7342381_T	40T	83T
Z_7342382_A	40A	82A
Z_7342383_G	40G	79G
Z_7342384_A	40A	79A
Z_7342385_C	37C	78C
Z_7342386_C	37C	79C
Z_7342387_G	38G	81G
Z_7342388_A	38A	80A
Z_7342389_T	37T	78T
Z_7342390_C	38C	79C
Z_7342391_A	40A	80A
Z_7342392_A	42A	80A
Z_7342393_A	40A	79A
Z_7342394_G	40G	77G
Z_7342395_C	40C	75C
Z_7342396_C	39C	74C
Z_7342397_C	38C	75C
Z_7342398_A	39A	73A
Z_7342399_A	37A	73A
Z_7342400_G	36G	74G
Z_7342401_C	33C	75C
Z_7342402_C	33C	75C
Z_7342403_A	34A	76A
Z_7342404_C	35C	76C
Z_7342405_C	36C	75C
Z_7342406_G	37G	75G
Z_7342407_A	36A	75A
Z_7342408_G	37G	76G
Z_7342409_C	37C	78C
Z_7342410_C	36C	78C
Z_7342411_A	35A	79A
Z_7342412_G	33G	79G
Z_7342413_A	33A	78A
Z_7342414_A	32A	79A
Z_7342415_G	32G	79G
Z_7342416_G	32G	78G
Z_7342417_C	34C	79C

Z_7342418_T	33T	78T
Z_7342419_G	35G	78G
Z_7342420_C	35C	76C
Z_7342421_A	36A	77A
Z_7342422_A	36A	77A
Z_7342423_G	37G	78G
Z_7342424_G	37G	78G
Z_7342425_G	38G	78G
Z_7342426_A	37A	79A
Z_7342427_A	35A	79A
Z_7342428_G	36G	80G
Z_7342429_C	35C	79C
Z_7342430_G	35A	32A_46G
Z_7342431_A	34A	78A
Z_7342432_G	33G	77G
Z_7342433_T	33T	77T
Z_7342434_C	31C	77C
Z_7342435_G	30G	75G
Z_7342436_G	30G	77G
Z_7342437_C	31C	74C
Z_7342438_T	30T	73T
Z_7342439_T	30T	72T
Z_7342440_C	30C	70C
Z_7342441_T	31T	72T
Z_7342442_C	32C	74C
Z_7342443_T	33T	73T
Z_7342444_G	35G	74G
Z_7342445_C	37C	71C
Z_7342446_T	39T	70T
Z_7342447_C	41C	69C
Z_7342448_T	43T	69T
Z_7342449_A	44A	68A
Z_7342450_G	44G	66G
Z_7342451_A	42A	64A
Z_7342452_C	45C	65C
Z_7342453_A	45A	64A
Z_7342454_A	45A	65A
Z_7342455_G	45G	65G
Z_7342456_G	45G	63G
Z_7342457_T	46T	60T
Z_7342458_A	45A	61A
Z_7342459_T	44T	57T
Z_7342460_A	44A	57A

Z_7342461_T	45T	57T
Z_7342462_A	43A	54A
Z_7342463_G	42G	55G
Z_7342464_A	44A	54A
Z_7342465_A	43A	53A
Z_7342466_G	43G	50G
Z_7342467_A	44A	54A
Z_7342468_G	43G	53G
Z_7342469_G	45G	52G
Z_7342470_A	44A	51A
Z_7342471_G	44G	54G
Z_7342472_G	45G	55G
Z_7342473_G	45G	54G
Z_7342474_C	43C	52C
Z_7342475_G	43G	52G
Z_7342476_G	42G	53G
Z_7342477_T	41T	51T
Z_7342478_G	41G	52G
Z_7342479_G	40G	52G
Z_7342480_T	40T	53T
Z_7342481_T	40T	49T
Z_7342482_A	32G	43A
Z_7342483_G	33G	50G
Z_7342484_G	33G	50G
Z_7342485_G	34G	51G
Z_7342486_G	1A_55T_62G	50G
Z_7342487_G	30G	22T_62G
Z_7342488_G	30T	49G
Z_7342489_G	44G	48G
Z_7342490_T	45T	49T
Z_7342491_G	46G	48G
Z_7342492_G	45G	48G
Z_7342493_C	45C	51C
Z_7342494_A	45A	52A
Z_7342495_C	46C	54C
Z_7342496_C	46C	54C
Z_7342497_T	45T	54T
Z_7342498_C	46C	54C
Z_7342499_T	46T	55T
Z_7342500_T	46T	55T
Z_7342501_C	44C	55C
Z_7342502_C	46C	54C
Z_7342503_T	48T	55T

Z_7342504_A	47A	55A
Z_7342505_G	47G	57G
Z_7342506_C	46C	59C
Z_7342507_C	48C	57C
Z_7342508_A	47A	58A
Z_7342509_A	48A	58A
Z_7342510_A	47A	58A
Z_7342511_A	45A	61A
Z_7342512_A	41T	36A_25T
Z_7342513_T	39T	62T
Z_7342514_G	36A	63G
Z_7342515_G	39G	63G
Z_7342516_T	39T	63T
Z_7342517_T	41T	63T
Z_7342518_C	44C	62C
Z_7342519_T	43T	63T
Z_7342520_G	43G	65G
Z_7342521_T	43T	65T
Z_7342522_T	43T	64T
Z_7342523_G	43G	63G
Z_7342524_C	43C	64C
Z_7342525_C	44C	64C
Z_7342526_A	44A	64A
Z_7342527_G	44G	64G
Z_7342528_A	42A	64A
Z_7342529_G	44G	64G
Z_7342530_C	46C	63C
Z_7342531_A	45A	62A
Z_7342532_G	44G	62G
Z_7342533_C	44C	62C
Z_7342534_C	43C	62C
Z_7342535_A	43A	63A
Z_7342536_A	45A	63A
Z_7342537_C	45C	60C
Z_7342538_C	46C	60C
Z_7342539_A	46A	59A
Z_7342540_G	46G	57G
Z_7342541_A	45A	58A
Z_7342542_T	44T	58T
Z_7342543_G	43G	59G
Z_7342544_C	42C	61C
Z_7342545_C	42C	61C
Z_7342546_C	40C	64C

Z_7342547_A	42A	64A
Z_7342548_C	41C	64C
Z_7342549_A	40G	32A_32G
Z_7342550_G	43G	66G
Z_7342551_G	42G	66G
Z_7342552_A	40A	68A
Z_7342553_T	41T	67T
Z_7342554_G	41G	68G
Z_7342555_T	42T	68T
Z_7342556_T	42T	69T
Z_7342557_C	42C	70C
Z_7342558_C	43C	70C
Z_7342559_T	43T	71T
Z_7342560_A	44A	73A
Z_7342561_A	43A	74A
Z_7342562_G	43T	73G
Z_7342563_C	44C	74C
Z_7342564_A	45A	73A
Z_7342565_G	44G	73G
Z_7342566_G	44G	68G
Z_7342567_A	47A	68A
Z_7342568_T	47T	69T
Z_7342569_G	47G	70G
Z_7342570_C	48C	72C
Z_7342571_G	49G	73G
Z_7342572_A	50A	72A
Z_7342573_G	47G	73G
Z_7342574_A	49A	75A
Z_7342575_T	49T	76T
Z_7342576_C	50C	76C
Z_7342577_A	50A	77A
Z_7342578_A	50A	76A
Z_7342579_C	50C	76C
Z_7342580_A	50A	76A
Z_7342581_A	49A	77A
Z_7342582_C	48C	77C
Z_7342583_A	48A	77A
Z_7342584_C	48C	77C
Z_7342585_G	48G	79G
Z_7342586_C	49C	80C
Z_7342587_T	48T	80T
Z_7342588_C	45C	82C
Z_7342589_A	46A	79A

Z_7342590_C	47C	80C
Z_7342591_T	48T	81T
Z_7342592_T	49T	82T
Z_7342593_T	50T	82T
Z_7342594_C	49C	81C
Z_7342595_C	48C	80C
Z_7342596_C	48C	82C
Z_7342597_C	46C	82C
Z_7342598_G	49G	83G
Z_7342599_G	46G	82G
Z_7342600_C	45C	83C
Z_7342601_A	47A	81A
Z_7342602_A	48A	82A
Z_7342603_C	48C	83C
Z_7342604_T	49T	83T
Z_7342605_T	47T	84T
Z_7342606_G	43G	81G
Z_7342607_C	42T	44T_36C
Z_7342608_A	43A	79A
Z_7342609_T	43C	34T_44C
Z_7342610_T	45T	77T
Z_7342611_A	46A	78A
Z_7342612_T	47T	78T
Z_7342613_G	47G	78G
Z_7342614_T	46T	79T
Z_7342615_G	47G	77G
Z_7342616_A	45A	77A
Z_7342617_A	46A	77A
Z_7342618_G	47G	80G
Z_7342619_C	48C	79C
Z_7342620_T	48T	79T
Z_7342621_T	47T	79T
Z_7342622_C	49C	80C
Z_7342623_A	48A	80A
Z_7342624_A	48A	80A
Z_7342625_A	47A	80A
Z_7342626_C	49C	80C
Z_7342627_A	49A	82A
Z_7342628_C	48C	81C
Z_7342629_C	47C	83C
Z_7342630_A	45A	86A
Z_7342631_A	45A	88A
Z_7342632_A	45A	88A

Z_7342633_G	45G	90G
Z_7342634_G	44G	91G
Z_7342635_G	43G	92G
Z_7342636_C	44C	90C
Z_7342637_A	42A	91A
Z_7342638_T	42T	90T
Z_7342639_A	42A	89A
Z_7342640_G	43G	90G
Z_7342641_G	42G	87G
Z_7342642_A	39A	88A
Z_7342643_T	40T	88T
Z_7342644_A	38A	90A
Z_7342645_T	38T	90T
Z_7342646_C	39C	90C
Z_7342647_A	37A	89A
Z_7342648_T	37T	90T
Z_7342649_C	38C	90C
Z_7342650_C	39C	91C
Z_7342651_T	40T	92T
Z_7342652_A	40A	91A
Z_7342653_T	39T	93T
Z_7342654_A	40A	92A
Z_7342655_A	41A	91A
Z_7342656_C	41C	90C
Z_7342657_C	41C	90C
Z_7342658_C	39C	41T_49C
Z_7342659_T	41T	95T
Z_7342660_C	42C	93C
Z_7342661_C	41C	93C
Z_7342662_C	42C	93C
Z_7342663_A	41A	92A
Z_7342664_A	41A	91A
Z_7342665_A	41A	91A
Z_7342666_G	41G	90G
Z_7342667_C	41C	91C
Z_7342668_T	42T	93T
Z_7342669_T	41T	93T
Z_7342670_G	43G	95G
Z_7342671_C	42C	96C
Z_7342672_C	41C	96C
Z_7342673_T	39T	96T
Z_7342674_C	40C	97C
Z_7342675_T	40T	93T

Z_7342676_C	41C	94C
Z_7342677_C	39C	93C
Z_7342678_A	39A	93A
Z_7342679_C	40C	46T_46C
Z_7342680_G	40G	90G
Z_7342681_A	38A	89A
Z_7342682_A	39A	90A
Z_7342683_G	38G	89G
Z_7342684_A	38A	88A
Z_7342685_A	38A	88A
Z_7342686_G	37G	89G
Z_7342687_C	36C	88C
Z_7342688_G	35A	93G
Z_7342689_T	34T	93T
Z_7342690_C	33C	92C
Z_7342691_T	32T	93T
Z_7342692_C	32C	43T_49C
Z_7342693_C	32C	91C
Z_7342694_T	31T	90T
Z_7342695_C	31C	89C
Z_7342696_T	29T	89T
Z_7342697_G	29G	87G
Z_7342698_A	28A	89A
Z_7342699_T	29T	90T
Z_7342700_G	29G	89G
Z_7342701_T	29T	88T
Z_7342702_C	29C	89C
Z_7342703_T	28T	90T
Z_7342704_T	28T	90T
Z_7342705_T	28T	90T
Z_7342706_T	28T	90T
Z_7342707_T	27T	90T
Z_7342708_A	26A	92A
Z_7342709_A	26A	90A
Z_7342710_A	24A	90A
Z_7342711_A	24A	91A
Z_7342712_G	24G	88G
Z_7342713_C	24C	88C
Z_7342714_C	24C	89C
Z_7342715_G	2A	89G
Z_7342716_C	null	88C
Z_7342717_C	null	89C
Z_7342718_A	null	89A

Z_7342719_G	null	90G
Z_7342720_G	null	89G
Z_7342721_G	null	90G
Z_7342722_C	null	89C
Z_7342723_T	null	91T
Z_7342724_G	null	89G
Z_7342725_G	null	84G
Z_7342726_C	null	82C
Z_7342727_A	null	82A
Z_7342728_G	null	80G
Z_7342729_C	null	35T_43C
Z_7342730_G	null	78G
Z_7342731_A	null	78A
Z_7342732_T	null	81T
Z_7342733_G	null	81G
Z_7342734_A	null	81A
Z_7342735_T	null	49T_33C
Z_7342736_G	null	83G
Z_7342737_G	null	32A_52G
Z_7342738_C	null	86C
Z_7342739_T	null	87T
Z_7342740_A	null	85A
Z_7342741_T	null	85T
Z_7342742_T	null	84T
Z_7342743_T	null	83T
Z_7342744_T	null	84T
Z_7342745_G	null	83G
Z_7342746_T	null	83T
Z_7342747_C	null	82C
Z_7342748_A	null	82A
Z_7342749_G	null	82G
Z_7342750_T	null	81T
Z_7342751_T	null	81T
Z_7342752_T	null	80T
Z_7342753_T	null	78T
Z_7342754_C	null	73C
Z_7342755_C	null	73C
Z_7342756_A	null	73A
Z_7342757_A	null	74A
Z_7342758_A	null	75A
Z_7342759_A	null	75A
Z_7342760_G	null	74G
Z_7342761_A	null	74A

Z_7342762_G	null	73G
Z_7342763_T	null	70T
Z_7342764_T	null	69T
Z_7342765_C	null	65C
Z_7342766_T	null	64T
Z_7342767_T	null	63T
Z_7342768_G	null	62G
Z_7342769_A	null	60A
Z_7342770_A	null	62A
Z_7342771_G	null	62G
Z_7342772_T	null	64T
Z_7342773_C	null	65C
Z_7342774_G	null	29A_35G
Z_7342775_T	null	65T
Z_7342776_T	null	66T
Z_7342777_A	null	66A
Z_7342778_G	null	66G
Z_7342779_A	null	38A_28G
Z_7342780_C	null	68C
Z_7342781_T	null	68T
Z_7342782_G	null	67G
Z_7342783_G	null	63G
Z_7342784_A	null	63A
Z_7342785_C	null	17T_39C
Z_7342786_T	null	58T
Z_7342787_C	null	17T_40C
Z_7342788_A	null	40A_17G
Z_7342789_T	null	41T_18C
Z_7342790_C	null	67C
Z_7342791_A	null	68A
Z_7342792_T	null	71T
Z_7342793_G	null	71G
Z_7342794_G	null	71G
Z_7342795_C	null	71C
Z_7342796_A	null	71A
Z_7342797_G	null	71G
Z_7342798_A	null	71A
Z_7342799_A	null	72A
Z_7342800_G	null	73G
Z_7342801_C	null	73C
Z_7342802_A	20A	73A
Z_7342803_G	21G	74G
Z_7342804_C	21C	76C

Z_7342805_C	22C	77C
Z_7342806_A	23A	80A
Z_7342807_A	23A	80A
Z_7342808_G	23G	80G
Z_7342809_C	23C	79C
Z_7342810_T	23T	80T
Z_7342811_G	23G	82G
Z_7342812_A	23A	81A
Z_7342813_C	23T	82C
Z_7342814_T	23T	84T
Z_7342815_C	23C	84C
Z_7342816_T	25T	84T
Z_7342817_T	25T	83T
Z_7342818_C	27C	81C
Z_7342819_T	28T	84T
Z_7342820_G	27G	86G
Z_7342821_C	30C	88C
Z_7342822_A	31A	88A
Z_7342823_G	30G	88G
Z_7342824_C	29C	90C
Z_7342825_A	31A	91A
Z_7342826_A	31A	91A
Z_7342827_G	31G	88G
Z_7342828_A	31A	88A
Z_7342829_C	30C	87C
Z_7342830_C	33C	85C
Z_7342831_T	33T	85T
Z_7342832_G	33G	83G
Z_7342833_T	33T	82T
Z_7342834_C	35C	81C
Z_7342835_C	35C	82C
Z_7342836_T	36T	82T
Z_7342837_T	36T	82T
Z_7342838_C	36C	82C
Z_7342839_C	36C	80C
Z_7342840_A	35A	79A
Z_7342841_C	36C	80C
Z_7342842_A	37A	81A
Z_7342843_T	35T	81T
Z_7342844_C	35C	81C
Z_7342845_C	36C	81C
Z_7342846_T	35T	82T
Z_7342847_T	35T	82T

Z_7342848_G	35G	82G
Z_7342849_A	35A	83A
Z_7342850_T	35T	83T
Z_7342851_T	35T	84T
Z_7342852_G	30G	82G
Z_7342853_C	31C	81C
Z_7342854_C	32C	80C
Z_7342855_T	30T	79T
Z_7342856_C	28C	79C
Z_7342857_G	30G	82G
Z_7342858_T	29T	82T
Z_7342859_C	28C	83C
Z_7342860_T	29T	84T
Z_7342861_T	30T	84T
Z_7342862_T	28T	84T
Z_7342863_T	27T	84T
Z_7342864_C	27C	84C
Z_7342865_A	28A	84A
Z_7342866_A	28A	85A
Z_7342867_G	25G	83G
Z_7342868_A	25A	81A
Z_7342869_T	25T	83T
Z_7342870_C	25T	40T_41C
Z_7342871_C	25C	82C
Z_7342872_C	25C	81C
Z_7342873_A	25A	78A
Z_7342874_C	26C	78C
Z_7342875_C	26C	79C
Z_7342876_A	26A	77A
Z_7342877_G	26G	78G
Z_7342878_T	28T	77T
Z_7342879_T	29T	77T
Z_7342880_T	30G	1A_39T_38G
Z_7342881_C	31C	75C
Z_7342882_C	32C	75C
Z_7342883_T	33T	73T
Z_7342884_T	34T	72T
Z_7342885_C	35C	72C
Z_7342886_A	33A	70A
Z_7342887_A	34A	70A
Z_7342888_A	32A	71A
Z_7342889_C	32C	72C
Z_7342890_G	32G	74G

Z_7342891_G	30G	78G
Z_7342892_A	28A	78A
Z_7342893_C	28C	79C
Z_7342894_A	28A	78A
Z_7342895_T	28T	80T
Z_7342896_T	28T	79T
Z_7342897_A	28A	79A
Z_7342898_A	29A	76A
Z_7342899_G	30G	75G
Z_7342900_C	29C	75C
Z_7342901_T	30T	72T
Z_7342902_G	29G	72G
Z_7342903_G	31C	71G
Z_7342904_C	29C	73C
Z_7342905_C	29C	72C
Z_7342906_G	29G	74G
Z_7342907_G	29G	74G
Z_7342908_C	30C	75C
Z_7342909_C	31C	74C
Z_7342910_T	30T	73T
Z_7342911_C	33C	72C
Z_7342912_T	32T	75T
Z_7342913_A	32A	75A
Z_7342914_G	32G	72G
Z_7342915_T	31T	70T
Z_7342916_T	31T	69T
Z_7342917_T	29T	70T
Z_7342918_C	31C	70C
Z_7342919_C	34C	67C
Z_7342920_A	37A	67A
Z_7342921_G	35G	68G
Z_7342922_G	35G	69G
Z_7342923_A	37A	69A
Z_7342924_C	34C	69C
Z_7342925_C	37C	70C
Z_7342926_C	37C	72C
Z_7342927_C	38C	72C
Z_7342928_A	40A	72A
Z_7342929_C	39C	71C
Z_7342930_C	39C	71C
Z_7342931_C	38C	70C
Z_7342932_C	39C	70C
Z_7342933_T	39T	70T

Z_7342934_G	39G	72G
Z_7342935_G	39G	72G
Z_7342936_A	38A	67A
Z_7342937_C	39C	69C
Z_7342938_C	38C	69C
Z_7342939_C	42C	69C
Z_7342940_T	43T	70T
Z_7342941_T	43T	67T
Z_7342942_T	44T	67T
Z_7342943_C	44C	70C
Z_7342944_T	43T	70T
Z_7342945_G	43G	70G
Z_7342946_A	41A	69A
Z_7342947_A	42A	68A
Z_7342948_A	40A	69A
Z_7342949_A	38A	71A
Z_7342950_G	39A	28A_43G
Z_7342951_G	37G	73G
Z_7342952_C	36C	71C
Z_7342953_T	37T	71T
Z_7342954_G	35G	70G
Z_7342955_G	36G	70G
Z_7342956_T	36T	70T
Z_7342957_G	34G	71G
Z_7342958_C	36C	72C
Z_7342959_T	35T	74T
Z_7342960_A	35A	72A
Z_7342961_C	35C	71C
Z_7342962_C	36C	71C
Z_7342963_C	36C	73C
Z_7342964_T	35T	72T
Z_7342965_G	37G	72G
Z_7342966_G	37G	72G
Z_7342967_C	35C	72C
Z_7342968_C	35C	72C
Z_7342969_G	33G	73G
Z_7342970_C	35C	72C
Z_7342971_T	35T	72T
Z_7342972_T	36T	73T
Z_7342973_T	34T	73T
Z_7342974_C	35C	76C
Z_7342975_C	34C	76C
Z_7342976_A	36A	77A

Z_7342977_G	36G	77G
Z_7342978_G	37G	78G
Z_7342979_A	36A	78A
Z_7342980_G	38G	79G
Z_7342981_A	35A	77A
Z_7342982_C	36C	78C
Z_7342983_A	36A	78A
Z_7342984_A	36A	77A
Z_7342985_G	37G	74G
Z_7342986_G	37G	71G
Z_7342987_A	37A	73A
Z_7342988_A	35A	72A
Z_7342989_G	34G	72G
Z_7342990_C	30C	69C
Z_7342991_A	30A	74A
Z_7342992_C	30C	74C
Z_7342993_A	30A	75A
Z_7342994_T	31T	75T
Z_7342995_G	34G	77G
Z_7342996_C	33C	76C
Z_7342997_A	35A	76A
Z_7342998_A	34A	77A
Z_7342999_A	34A	77A
Z_7343000_C	34C	79C
Z_7343001_A	34A	76A
Z_7343002_A	33A	77A
Z_7343003_A	34A	76A
Z_7343004_A	34A	76A
Z_7343005_C	33C	76C
Z_7343006_A	33A	77A
Z_7343007_C	32C	74C
Z_7343008_G	31G	75G
Z_7343009_A	32A	75A
Z_7343010_T	31T	75T
Z_7343011_T	31T	74T
Z_7343012_T	30T	73T
Z_7343013_G	30G	74G
Z_7343014_T	30T	74T
Z_7343015_A	29A	73A
Z_7343016_C	28C	73C
Z_7343017_A	30A	71A
Z_7343018_A	31A	72A
Z_7343019_C	31C	73C

Z_7343020_T	31T	71T
Z_7343021_A	31A	72A
Z_7343022_T	32T	72T
Z_7343023_C	33C	72C
Z_7343024_T	33T	74T
Z_7343025_T	32T	76T
Z_7343026_A	32A	77A
Z_7343027_T	32T	79T
Z_7343028_C	32C	78C
Z_7343029_C	34C	78C
Z_7343030_T	35T	78T
Z_7343031_G	34G	78G
Z_7343032_G	34G	78G
Z_7343033_G	35G	78G
Z_7343034_C	36C	78C
Z_7343035_A	36G	78A
Z_7343036_C	37C	79C
Z_7343037_A	36A	79A
Z_7343038_C	38C	78C
Z_7343039_T	38T	77T
Z_7343040_T	36T	77T
Z_7343041_T	35T	77T
Z_7343042_T	35T	78T
Z_7343043_C	32C	77C
Z_7343044_T	34T	75T
Z_7343045_G	34G	77G
Z_7343046_A	33A	47A_28T
Z_7343047_A	31A	74A
Z_7343048_C	29C	74C
Z_7343049_C	28C	74C
Z_7343050_T	11A_38T_21G_45C	15A_88T_30G_60C
Z_7343051_C	33C	79C
Z_7343052_T	17T	69T
Z_7343053_G	19G	71G
Z_7343054_T	19T	73T
Z_7343055_G	20G	75G
Z_7343056_C	21C	75C
Z_7343057_T	22A	27A_49T
Z_7343058_T	24T	75T
Z_7343059_T	24T	75T
Z_7343060_T	24T	75T
Z_7343061_C	25C	74C
Z_7343062_A	27A	75A

Z_7343063_T	28C	44T_31C
Z_7343064_A	29A	74A
Z_7343065_G	29G	76G
Z_7343066_C	29C	75C
Z_7343067_C	29C	77C
Z_7343068_T	29T	77T
Z_7343069_T	29T	76T
Z_7343070_C	30C	78C
Z_7343071_T	30T	77T
Z_7343072_G	29A	33A_46G
Z_7343073_T	29T	78T
Z_7343074_T	31T	78T
Z_7343075_G	31G	77G
Z_7343076_C	31C	78C
Z_7343077_A	33A	76A
Z_7343078_A	34A	75A
Z_7343079_C	34C	76C
Z_7343080_C	35C	76C
Z_7343081_A	36A	78A
Z_7343082_C	37C	75C
Z_7343083_C	38C	76C
Z_7343084_A	40A	76A
Z_7343085_A	38A	76A
Z_7343086_T	38T	73T
Z_7343087_G	39G	75G
Z_7343088_G	39G	74G
Z_7343089_A	38A	71A
Z_7343090_C	39C	72C
Z_7343091_C	36C	71C
Z_7343092_T	37T	72T
Z_7343093_G	38G	71G
Z_7343094_T	39T	70T
Z_7343095_T	39T	67T
Z_7343096_G	38G	71G
Z_7343097_G	36G	72G
Z_7343098_C	36C	73C
Z_7343099_T	37T	72T
Z_7343100_A	35A	73A
Z_7343101_C	35C	74C
Z_7343102_A	35A	75A
Z_7343103_G	34G	74G
Z_7343104_A	33A	73A
Z_7343105_C	31C	73C

Z_7343106_G	31G	74G
Z_7343107_A	31A	74A
Z_7343108_T	32T	75T
Z_7343109_G	32G	74G
Z_7343110_A	31A	75A
Z_7343111_C	32C	77C
Z_7343112_A	32A	49A_28C
Z_7343113_A	32A	77A
Z_7343114_C	33C	77C
Z_7343115_A	32A	77A
Z_7343116_T	33T	74T
Z_7343117_T	32T	74T
Z_7343118_C	34C	75C
Z_7343119_G	36G	29A_45G
Z_7343120_C	36C	70C
Z_7343121_A	36A	69A
Z_7343122_A	36A	68A
Z_7343123_C	34C	68C
Z_7343124_T	34T	67T
Z_7343125_C	33C	67C
Z_7343126_A	31A	67A
Z_7343127_A	31A	67A
Z_7343128_A	29A	69A
Z_7343129_G	32G	70G
Z_7343130_C	32C	70C
Z_7343131_C	32T	70C
Z_7343132_G	29G	71G
Z_7343133_T	32T	70T
Z_7343134_C	32C	69C
Z_7343135_C	32C	70C
Z_7343136_T	34T	68T
Z_7343137_T	32T	67T
Z_7343138_T	35T	68T
Z_7343139_A	35A	67A
Z_7343140_T	36T	66T
Z_7343141_T	38T	67T
Z_7343142_G	38G	66G
Z_7343143_G	39G	68G
Z_7343144_G	38G	68G
Z_7343145_C	39C	68C
Z_7343146_T	39T	68T
Z_7343147_G	40G	69G
Z_7343148_T	39T	66T

Z_7343149_A	39A	64A
Z_7343150_T	39T	62T
Z_7343151_T	39T	64T
Z_7343152_T	41T	63T
Z_7343153_T	40T	63T
Z_7343154_A	37A	62A
Z_7343155_T	38T	62T
Z_7343156_G	36T	17T_44G
Z_7343157_G	36A	16A_44G
Z_7343158_T	42T	62T
Z_7343159_T	42T	62T
Z_7343160_T	43T	64T
Z_7343161_T	43T	64T
Z_7343162_A	43A	62A
Z_7343163_G	42G	65G
Z_7343164_A	41A	63A
Z_7343165_C	43C	62C
Z_7343166_A	42A	62A
Z_7343167_G	41G	60G
Z_7343168_T	43T	60T
Z_7343169_G	43G	62G
Z_7343170_A	44A	63A
Z_7343171_A	44A	63A
Z_7343172_G	44G	65G
Z_7343173_C	45C	65C
Z_7343174_C	46C	65C
Z_7343175_T	49T	65T
Z_7343176_G	48G	64G
Z_7343177_A	47A	67A
Z_7343178_T	51T	66T
Z_7343179_C	51C	65C
Z_7343180_C	51C	66C
Z_7343181_A	54A	66A
Z_7343182_C	54C	64C
Z_7343183_A	56A	64A
Z_7343184_G	54G	64G
Z_7343185_A	54A	63A
Z_7343186_A	52A	65A
Z_7343187_G	52G	64G
Z_7343188_A	50A	65A
Z_7343189_C	50C	67C
Z_7343190_T	51T	67T
Z_7343191_G	53G	63G

Z_7343192_A	54A	63A
Z_7343193_T	53T	63T
Z_7343194_T	54T	63T
Z_7343195_T	54T	62T
Z_7343196_A	55A	62A
Z_7343197_A	57A	61A
Z_7343198_G	57G	61G
Z_7343199_A	57A	62A
Z_7343200_G	58G	63G
Z_7343201_C	58C	62C
Z_7343202_A	59A	61A
Z_7343203_A	57A	61A
Z_7343204_G	56G	61G
Z_7343205_G	56G	59G
Z_7343206_A	54A	60A
Z_7343207_A	55A	63A
Z_7343208_A	54A	64A
Z_7343209_A	54A	64A
Z_7343210_G	52G	63G
Z_7343211_G	48G	65G
Z_7343212_A	51A	65A
Z_7343213_A	50A	67A
Z_7343214_G	47G	66G
Z_7343215_G	48G	66G
Z_7343216_C	50C	66C
Z_7343217_T	47T	64T
Z_7343218_T	47T	66T
Z_7343219_T	46T	65T
Z_7343220_C	47C	65C
Z_7343221_T	48T	67T
Z_7343222_C	45C	66C
Z_7343223_C	45C	65C
Z_7343224_C	44C	64C
Z_7343225_C	44T	26T_38C
Z_7343226_G	44G	63G
Z_7343227_A	43A	62A
Z_7343228_T	43T	62T
Z_7343229_A	43A	62A
Z_7343230_A	42A	62A
Z_7343231_A	41A	62A
Z_7343232_G	41G	63G
Z_7343233_G	41C	35G_27C
Z_7343234_A	41A	62A

Z_7343235_C	40C	63C
Z_7343236_T	41T	62T
Z_7343237_A	42A	63A
Z_7343238_T	44T	61T
Z_7343239_A	43A	63A
Z_7343240_G	42G	63G
Z_7343241_G	42G	63G
Z_7343242_A	43A	63A
Z_7343243_A	43A	64A
Z_7343244_G	44G	65G
Z_7343245_A	40A	65A
Z_7343246_A	41A	63A
Z_7343247_A	43A	63A
Z_7343248_A	42A	62A
Z_7343249_G	42G	62G
Z_7343250_G	43G	61G
Z_7343251_C	39C	62C
Z_7343252_A	39A	29A_30C
Z_7343253_G	40G	27A_30G
Z_7343254_G	41G	59G
Z_7343255_T	41T	62T
Z_7343256_G	40G	62G
Z_7343257_A	41A	65A
Z_7343258_T	41T	60T
Z_7343259_C	41C	62C
Z_7343260_T	41T	61T
Z_7343261_T	39T	63T
Z_7343262_A	38A	64A
Z_7343263_G	40G	63G
Z_7343264_T	43T	62T
Z_7343265_G	43G	62G
Z_7343266_G	41G	61G
Z_7343267_G	38G	61G
Z_7343268_T	40T	61T
Z_7343269_T	40T	61T
Z_7343270_T	37T	61T
Z_7343271_T	37T	62T
Z_7343272_T	38T	58T
Z_7343273_C	38C	57C
Z_7343274_T	38T	59T
Z_7343275_A	37A	59A
Z_7343276_T	38T	58T
Z_7343277_G	38G	57G

Z_7343278_T	39T	57T
Z_7343279_T	40T	59T
Z_7343280_C	41C	60C
Z_7343281_A	40A	59A
Z_7343282_T	39T	59T
Z_7343283_C	41C	60C
Z_7343284_T	42T	57T
Z_7343285_T	42T	58T
Z_7343286_T	42T	58T
Z_7343287_A	42A	60A
Z_7343288_A	41A	61A
Z_7343289_T	44T	62T
Z_7343290_T	43T	62T
Z_7343291_T	42T	63T
Z_7343292_C	41C	63C
Z_7343293_T	40T	64T
Z_7343294_T	41T	63T
Z_7343295_T	42T	63T
Z_7343296_G	43G	63G
Z_7343297_G	41G	64G
Z_7343298_C	43C	63C
Z_7343299_G	44G	63G
Z_7343300_T	44T	63T
Z_7343301_A	46A	61A
Z_7343302_T	47T	60T
Z_7343303_T	47T	59T
Z_7343304_A	46A	60A
Z_7343305_A	46A	60A
Z_7343306_G	44G	62G
Z_7343307_C	43C	65C
Z_7343308_C	41C	62C
Z_7343309_T	43T	62T
Z_7343310_T	43T	62T
Z_7343311_G	42G	63G
Z_7343312_A	41A	65A
Z_7343313_T	42T	64T
Z_7343314_T	41T	64T
Z_7343315_A	41A	64A
Z_7343316_C	38C	63C
Z_7343317_T	38T	63T
Z_7343318_T	39T	63T
Z_7343319_A	39A	65A
Z_7343320_A	39A	66A

Z_7343321_A	39A	66A
Z_7343322_T	40T	66T
Z_7343323_T	38T	68T
Z_7343324_G	37G	68G
Z_7343325_T	39T	70T
Z_7343326_A	38A	71A
Z_7343327_A	39A	69A
Z_7343328_T	39T	69T
Z_7343329_T	40T	68T
Z_7343330_A	39A	67A
Z_7343331_T	37T	68T
Z_7343332_G	40G	68G
Z_7343333_G	37G	68G
Z_7343334_C	37C	67C
Z_7343335_A	38A	67A
Z_7343336_T	39T	67T
Z_7343337_A	38A	68A
Z_7343338_T	40T	67T
Z_7343339_C	41C	68C
Z_7343340_T	41T	69T
Z_7343341_T	40T	70T
Z_7343342_T	40T	70T
Z_7343343_T	40T	70T
Z_7343344_G	40G	70G
Z_7343345_G	41G	69G
Z_7343346_G	41G	69G
Z_7343347_T	42T	69T
Z_7343348_T	41T	69T
Z_7343349_G	41G	68G
Z_7343350_G	41G	66G
Z_7343351_T	39T	64T
Z_7343352_T	39T	64T
Z_7343353_T	40T	65T
Z_7343354_T	39T	64T
Z_7343355_G	38G	63G
Z_7343356_A	38A	63A
Z_7343357_A	38A	63A
Z_7343358_T	39T	63T
Z_7343359_T	38T	64T
Z_7343360_T	38T	63T
Z_7343361_G	37A	34A_29G
Z_7343362_C	37C	62C
Z_7343363_T	37T	62T

Z_7343364_G	36G	62G
Z_7343365_G	35G	61G
Z_7343366_T	34T	59T
Z_7343367_G	34G	59G
Z_7343368_G	33G	57G
Z_7343369_G	32G	58G
Z_7343370_T	32T	58T
Z_7343371_G	31G	59G
Z_7343372_G	30G	59G
Z_7343373_T	30T	60T
Z_7343374_G	30G	60G
Z_7343375_T	26G	58T
Z_7343376_G	28G	57G
Z_7343377_T	26T	36T_23G
Z_7343378_T	25G	53T
Z_7343379_G	26T	23T_33G
Z_7343380_T	32T	57T
Z_7343381_T	33T	56T
Z_7343382_T	33T	56T
Z_7343383_T	33T	56T
Z_7343384_T	32T	56T
Z_7343385_T	32T	55T
Z_7343386_T	33T	52T
Z_7343387_C	34C	52C
Z_7343388_T	35T	51T
Z_7343389_T	34T	51T
Z_7343390_T	34T	51T
Z_7343391_T	33T	51T
Z_7343392_C	33C	51C
Z_7343393_A	34A	51A
Z_7343394_A	34A	51A
Z_7343395_A	32A	53A
Z_7343396_A	32A	53A
Z_7343397_A	32A	53A
Z_7343398_T	32T	53T
Z_7343399_G	31G	52G
Z_7343400_C	31C	52C
Z_7343401_T	31T	48T
Z_7343402_T	31T	47T
Z_7343403_T	31T	48T
Z_7343404_T	30T	51T
Z_7343405_T	29T	52T
Z_7343406_G	28G	53G

Z_7343407_T	30T	53T
Z_7343408_T	30T	53T
Z_7343409_T	29T	53T
Z_7343410_C	29C	54C
Z_7343411_T	29T	54T
Z_7343412_C	28C	54C
Z_7343413_C	29C	54C
Z_7343414_C	29C	52C
Z_7343415_T	27T	52T
Z_7343416_C	27C	53C
Z_7343417_C	26C	54C
Z_7343418_T	26T	54T
Z_7343419_C	25C	54C
Z_7343420_T	24T	52T
Z_7343421_A	24A	50A
Z_7343422_T	25C	28T_1G_22C
Z_7343423_C	25C	50C
Z_7343424_T	29T	54T
Z_7343425_G	29G	55G
Z_7343426_T	28T	54T
Z_7343427_G	30G	53G
Z_7343428_A	30A	52A
Z_7343429_T	30T	52T
Z_7343430_G	32G	54G
Z_7343431_G	31G	55G
Z_7343432_A	33A	55A
Z_7343433_T	33T	57T
Z_7343434_T	34T	56T
Z_7343435_T	34T	55T
Z_7343436_G	33G	55G
Z_7343437_A	33A	57A
Z_7343438_A	34A	30A_27C
Z_7343439_A	33A	56A
Z_7343440_A	33A	56A
Z_7343441_G	34G	56G
Z_7343442_A	34A	56A
Z_7343443_G	34G	58G
Z_7343444_G	33G	60G
Z_7343445_T	33T	59T
Z_7343446_G	35G	58G
Z_7343447_T	36T	58T
Z_7343448_T	36T	59T
Z_7343449_T	36T	61T

Z_7343450_T	35T	61T
Z_7343451_C	34C	60C
Z_7343452_G	33G	30A_29G
Z_7343453_G	32G	60G
Z_7343454_C	34C	61C
Z_7343455_A	35A	61A
Z_7343456_T	33T	62T
Z_7343457_G	33G	62G
Z_7343458_G	28G	61G
Z_7343459_C	32C	60C
Z_7343460_G	32G	61G
Z_7343461_T	32T	62T
Z_7343462_G	32G	62G
Z_7343463_G	34G	62G
Z_7343464_T	35T	62T
Z_7343465_G	37G	65G
Z_7343466_A	37A	65A
Z_7343467_A	37A	66A
Z_7343468_T	36T	65T
Z_7343469_T	36T	65T
Z_7343470_A	37A	65A
Z_7343471_A	39A	65A
Z_7343472_A	37A	66A
Z_7343473_A	38A	66A
Z_7343474_C	37C	67C
Z_7343475_A	39A	68A
Z_7343476_G	39G	67G
Z_7343477_G	37G	66G
Z_7343478_A	34A	67A
Z_7343479_T	35T	65T
Z_7343480_T	35T	69T
Z_7343481_A	35A	70A
Z_7343482_A	35A	71A
Z_7343483_A	35A	71A
Z_7343484_A	35A	72A
Z_7343485_A	37A	73A
Z_7343486_T	37T	74T
Z_7343487_T	37T	73T
Z_7343488_A	38A	74A
Z_7343489_G	38G	73G
Z_7343490_C	37C	73C
Z_7343491_C	39C	75C
Z_7343492_A	38A	73A

Z_7343493_A	37A	75A
Z_7343494_C	34C	76C
Z_7343495_T	35T	75T
Z_7343496_C	36C	75C
Z_7343497_A	36A	75A
Z_7343498_C	34C	74C
Z_7343499_T	34T	71T
Z_7343500_A	32A	70A
Z_7343501_A	34A	68A
Z_7343502_A	32A	67A
Z_7343503_T	35T	70T
Z_7343504_G	32G	70G
Z_7343505_A	35A	69A
Z_7343506_G	35G	71G
Z_7343507_C	35C	71C
Z_7343508_A	36A	73A
Z_7343509_A	39A	71A
Z_7343510_C	38C	72C
Z_7343511_T	38T	72T
Z_7343512_G	39G	74G
Z_7343513_C	40C	73C
Z_7343514_T	40T	73T
Z_7343515_C	40C	72C
Z_7343516_A	39A	76A
Z_7343517_G	39G	76G
Z_7343518_A	40A	77A
Z_7343519_A	41A	77A
Z_7343520_A	38A	77A
Z_7343521_A	41A	80A
Z_7343522_G	41G	81G
Z_7343523_G	40G	81G
Z_7343524_A	42A	85A
Z_7343525_A	41A	83A
Z_7343526_G	40G	83G
Z_7343527_G	40G	83G
Z_7343528_C	42C	82C
Z_7343529_A	41A	84A
Z_7343530_G	42G	84G
Z_7343531_T	43T	83T
Z_7343532_C	43C	81C
Z_7343533_C	44C	81C
Z_7343534_A	43A	81A
Z_7343535_A	42A	81A

Z_7343536_T	43T	81T
Z_7343537_T	44T	82T
Z_7343538_C	42C	79C
Z_7343539_T	44T	78T
Z_7343540_G	44G	78G
Z_7343541_C	43C	78C
Z_7343542_A	44A	78A
Z_7343543_C	44C	76C
Z_7343544_T	44T	74T
Z_7343545_T	44T	75T
Z_7343546_T	42T	75T
Z_7343547_C	44C	76C
Z_7343548_C	45C	76C
Z_7343549_A	46A	75A
Z_7343550_G	45G	74G
Z_7343551_C	47C	74C
Z_7343552_A	47A	73A
Z_7343553_G	47G	73G
Z_7343554_A	46A	74A
Z_7343555_A	45A	75A
Z_7343556_A	45A	75A
Z_7343557_A	45A	75A
Z_7343558_T	44T	76T
Z_7343559_C	45C	78C
Z_7343560_C	49C	78C
Z_7343561_A	49A	78A
Z_7343562_A	49A	78A
Z_7343563_G	49G	77G
Z_7343564_T	49T	78T
Z_7343565_A	48A	77A
Z_7343566_G	47G	78G
Z_7343567_C	46C	78C
Z_7343568_T	48T	76T
Z_7343569_C	51C	76C
Z_7343570_T	52T	75T
Z_7343571_A	50A	76A
Z_7343572_A	49A	76A
Z_7343573_G	49G	76G
Z_7343574_A	50A	77A
Z_7343575_T	49T	75T
Z_7343576_T	49T	74T
Z_7343577_T	48T	74T
Z_7343578_C	46C	73C

Z_7343579_C	44C	73C
Z_7343580_A	44A	73A
Z_7343581_A	45A	75A
Z_7343582_C	43C	75C
Z_7343583_C	41C	74C
Z_7343584_C	39C	73C
Z_7343585_A	44A	73A
Z_7343586_G	40G	72G
Z_7343587_G	38G	72G
Z_7343588_A	40A	72A
Z_7343589_C	40C	71C
Z_7343590_T	40T	71T
Z_7343591_T	38T	41T_1G_28C
Z_7343592_T	40T	70T
Z_7343593_T	40T	72T
Z_7343594_T	38T	73T
Z_7343595_T	40T	41T_32C
Z_7343596_C	39C	74C
Z_7343597_T	41T	73T
Z_7343598_C	42C	71C
Z_7343599_G	42G	72G
Z_7343600_G	42G	72G
Z_7343601_G	43G	70G
Z_7343602_C	43C	71C
Z_7343603_A	41G	32A_39G
Z_7343604_C	41C	72C
Z_7343605_T	40T	69T
Z_7343606_T	41T	70T
Z_7343607_T	41T	70T
Z_7343608_T	41T	71T
Z_7343609_T	41T	73T
Z_7343610_T	40T	76T
Z_7343611_G	40G	73G
Z_7343612_T	39T	73T
Z_7343613_C	41C	73C
Z_7343614_T	40T	70T
Z_7343615_A	39A	71A
Z_7343616_G	39G	69G
Z_7343617_G	39G	69G
Z_7343618_A	37A	72A
Z_7343619_A	37A	68A
Z_7343620_C	38C	32T_36C
Z_7343621_C	39C	67C

Z_7343622_A	39A	67A
Z_7343623_G	39G	66G
Z_7343624_C	39C	63C
Z_7343625_C	40C	65C
Z_7343626_A	40A	65A
Z_7343627_G	40G	65G
Z_7343628_G	42G	65G
Z_7343629_A	40A	66A
Z_7343630_A	37A	66A
Z_7343631_G	37G	68G
Z_7343632_A	37A	69A
Z_7343633_G	37G	71G
Z_7343634_A	37A	71A
Z_7343635_G	36G	72G
Z_7343636_A	35A	76A
Z_7343637_T	36T	76T
Z_7343638_T	35T	78T
Z_7343639_C	34C	79C
Z_7343640_G	34G	79G
Z_7343641_G	33G	79G
Z_7343642_T	34T	78T
Z_7343643_T	32T	78T
Z_7343644_C	31C	77C
Z_7343645_T	32T	77T
Z_7343646_T	32T	79T
Z_7343647_T	34T	79T
Z_7343648_T	32T	81T
Z_7343649_C	33C	81C
Z_7343650_A	34A	80A
Z_7343651_C	36C	79C
Z_7343652_A	35A	79A
Z_7343653_A	38A	78A
Z_7343654_A	38A	76A
Z_7343655_A	38A	73A
Z_7343656_T	40T	71T
Z_7343657_C	42C	74C
Z_7343658_A	42A	74A
Z_7343659_G	42G	73G
Z_7343660_T	42T	74T
Z_7343661_A	41A	73A
Z_7343662_A	39A	74A
Z_7343663_T	42T	76T
Z_7343664_C	41C	76C

Z_7343665_T	40T	77T
Z_7343666_T	39T	76T
Z_7343667_T	39T	77T
Z_7343668_G	39G	78G
Z_7343669_G	37G	77G
Z_7343670_A	37A	76A
Z_7343671_C	36C	76C
Z_7343672_A	36A	76A
Z_7343673_T	36T	76T
Z_7343674_G	36G	76G
Z_7343675_A	37A	75A
Z_7343676_T	35T	71T
Z_7343677_G	35G	72G
Z_7343678_A	34A	72A
Z_7343679_T	33T	73T
Z_7343680_C	33C	73C
Z_7343681_G	35G	73G
Z_7343682_C	35C	75C
Z_7343683_A	33A	72A
Z_7343684_C	32C	72C
Z_7343685_A	33A	72A
Z_7343686_G	34G	72G
Z_7343687_A	34A	73A
Z_7343688_C	35C	74C
Z_7343689_T	34T	74T
Z_7343690_A	34A	73A
Z_7343691_G	33G	73G
Z_7343692_A	35A	72A
Z_7343693_G	35G	72G
Z_7343694_C	35C	71C
Z_7343695_C	34C	73C
Z_7343696_G	34G	74G
Z_7343697_C	34C	75C
Z_7343698_G	31G	73G
Z_7343699_A	31G	44A_28G
Z_7343700_T	36T	72T
Z_7343701_A	36A	73A
Z_7343702_T	37T	71T
Z_7343703_C	36C	71C
Z_7343704_T	37T	70T
Z_7343705_G	37G	69G
Z_7343706_A	40A	70A
Z_7343707_C	40C	69C

Z_7343708_G	40G	71G
Z_7343709_C	40C	71C
Z_7343710_A	40A	70A
Z_7343711_G	40G	69G
Z_7343712_A	40A	71A
Z_7343713_C	41C	69C
Z_7343714_A	41A	69A
Z_7343715_A	40A	72A
Z_7343716_T	40T	71T
Z_7343717_G	38T	30T_40G
Z_7343718_A	41A	71A
Z_7343719_A	40A	72A
Z_7343720_G	40G	71G
Z_7343721_T	38T	70T
Z_7343722_C	38C	73C
Z_7343723_C	35C	73C
Z_7343724_T	36T	72T
Z_7343725_T	38T	72T
Z_7343726_G	35G	71G
Z_7343727_G	33G	70G
Z_7343728_C	33C	68C
Z_7343729_A	33A	67A
Z_7343730_G	33G	67G
Z_7343731_C	33C	64C
Z_7343732_T	33T	64T
Z_7343733_A	30A	64A
Z_7343734_T	30T	65T
Z_7343735_C	31C	65C
Z_7343736_G	30A	66G
Z_7343737_C	31C	66C
Z_7343738_C	31C	67C
Z_7343739_A	31A	67A
Z_7343740_A	32A	68A
Z_7343741_G	32G	68G
Z_7343742_C	32C	68C
Z_7343743_G	31G	67G
Z_7343744_T	32T	66T
Z_7343745_C	32C	69C
Z_7343746_C	32C	71C
Z_7343747_T	31T	71T
Z_7343748_T	34T	77T
Z_7343749_C	35C	76C
Z_7343750_T	36T	76T

Z_7343751_G	35G	78G
Z_7343752_G	34G	75G
Z_7343753_C	34C	76C
Z_7343754_A	32G	38A_38G
Z_7343755_T	32T	76T
Z_7343756_T	33T	76T
Z_7343757_A	33A	76A
Z_7343758_A	33A	76A
Z_7343759_A	33A	76A
Z_7343760_G	34G	76G
Z_7343761_C	33C	77C
Z_7343762_T	32T	79T
Z_7343763_G	34G	78G
Z_7343764_A	35A	79A
Z_7343765_G	33G	80G
Z_7343766_G	33G	79G
Z_7343767_A	31A	82A
Z_7343768_G	30G	83G
Z_7343769_A	35A	83A
Z_7343770_A	33G	43A_40G
Z_7343771_G	32G	85G
Z_7343772_G	32G	86G
Z_7343773_A	35A	84A
Z_7343774_T	33T	83T
Z_7343775_C	37C	86C
Z_7343776_G	34G	86G
Z_7343777_G	33G	84G
Z_7343778_G	33G	84G
Z_7343779_A	33A	85A
Z_7343780_A	33A	85A
Z_7343781_C	33C	85C
Z_7343782_G	33G	85G
Z_7343783_T	32T	81T
Z_7343784_A	32A	82A
Z_7343785_A	32A	83A
Z_7343786_G	33G	83G
Z_7343787_A	32A	84A
Z_7343788_T	32T	83T
Z_7343789_C	33C	83C
Z_7343790_C	55T_32C	72T_81C
Z_7343791_C	33C	80C
Z_7343792_C	32C	79C
Z_7343793_A	33A	80A

Z_7343794_C	33C	80C
Z_7343795_T	33T	80T
Z_7343796_G	33G	80G
Z_7343797_C	34C	79C
Z_7343798_C	33C	78C
Z_7343799_A	34A	79A
Z_7343800_A	34A	78A
Z_7343801_C	34C	80C
Z_7343802_T	34T	80T
Z_7343803_T	34T	79T
Z_7343804_T	32T	81T
Z_7343805_G	32G	81G
Z_7343806_G	34G	81G
Z_7343807_A	34A	78A
Z_7343808_G	35G	78G
Z_7343809_A	35A	76A
Z_7343810_C	34C	77C
Z_7343811_A	35A	80A
Z_7343812_C	37C	80C
Z_7343813_A	36A	78A
Z_7343814_A	35A	79A
Z_7343815_A	35A	78A
Z_7343816_G	35G	78G
Z_7343817_C	31C	77C
Z_7343818_C	31C	78C
Z_7343819_A	30A	78A
Z_7343820_C	32C	77C
Z_7343821_A	33A	77A
Z_7343822_G	33G	77G
Z_7343823_G	34G	79G
Z_7343824_A	34A	79A
Z_7343825_A	35A	80A
Z_7343826_A	36A	80A
Z_7343827_A	36A	81A
Z_7343828_A	36A	79A
Z_7343829_G	36G	80G
Z_7343830_C	35C	80C
Z_7343831_A	34A	81A
Z_7343832_C	32C	82C
Z_7343833_A	32A	81A
Z_7343834_A	32A	79A
Z_7343835_A	33A	79A
Z_7343836_A	34A	79A

Z_7343837_G	34G	79G
Z_7343838_C	30C	78C
Z_7343839_T	32T	79T
Z_7343840_A	32A	83A
Z_7343841_T	31T	80T
Z_7343842_T	28T	81T
Z_7343843_C	31C	77C
Z_7343844_A	29A	77A
Z_7343845_G	28G	77G
Z_7343846_A	29A	77A
Z_7343847_T	28T	79T
Z_7343848_C	29C	78C
Z_7343849_T	28T	79T
Z_7343850_G	28G	79G
Z_7343851_C	28C	79C
Z_7343852_C	29C	80C
Z_7343853_A	30A	79A
Z_7343854_T	29T	78T
Z_7343855_G	28G	76G
Z_7343856_T	29T	78T
Z_7343857_A	29A	76A
Z_7343858_G	30G	77G
Z_7343859_A	26A	78A
Z_7343860_C	26C	78C
Z_7343861_G	26G	43A_38G
Z_7343862_C	27C	79C
Z_7343863_A	28A	78A
Z_7343864_C	27C	76C
Z_7343865_A	30A	76A
Z_7343866_G	32G	77G
Z_7343867_A	32A	76A
Z_7343868_C	31C	77C
Z_7343869_T	32T	77T
Z_7343870_G	33G	76G
Z_7343871_T	33T	78T
Z_7343872_T	33T	78T
Z_7343873_T	34T	78T
Z_7343874_G	34G	76G
Z_7343875_G	36G	77G
Z_7343876_G	35G	78G
Z_7343877_A	34A	78A
Z_7343878_C	32C	77C
Z_7343879_A	33A	78A

Z_7343880_C	33C	79C
Z_7343881_A	33A	81A
Z_7343882_C	32C	81C
Z_7343883_A	35A	81A
Z_7343884_C	35C	82C
Z_7343885_A	36A	84A
Z_7343886_C	36C	84C
Z_7343887_A	38A	85A
Z_7343888_C	38C	84C
Z_7343889_A	62A_24G_24C	87A
Z_7343890_C	7C	40G_47C
Z_7343891_A	9A	86A
Z_7343892_G	9C	86G
Z_7343893_A	35A	85A
Z_7343894_T	35T	46T_37C
Z_7343895_G	35G	84G
Z_7343896_T	37T	82T
Z_7343897_A	37A	84A
Z_7343898_C	38C	83C
Z_7343899_A	39A	81A
Z_7343900_C	39C	82C
Z_7343901_A	38A	83A
Z_7343902_C	38C	88C
Z_7343903_T	38T	87T
Z_7343904_G	37G	88G
Z_7343905_A	37A	86A
Z_7343906_A	39A	84A
Z_7343907_C	40C	83C
Z_7343908_C	40C	84C
Z_7343909_T	38T	84T
Z_7343910_C	38C	84C
Z_7343911_A	36A	84A
Z_7343912_G	37G	84G
Z_7343913_A	34G	83A
Z_7343914_G	37G	86G
Z_7343915_G	37G	87G
Z_7343916_G	36G	88G
Z_7343917_C	37C	89C
Z_7343918_C	37C	89C
Z_7343919_G	37G	88G
Z_7343920_C	38C	88C
Z_7343921_A	39A	88A
Z_7343922_C	39C	87C

Z_7343923_A	39A	89A
Z_7343924_C	38C	87C
Z_7343925_A	39A	86A
Z_7343926_A	41A	85A
Z_7343927_C	40C	85C
Z_7343928_C	41C	84C
Z_7343929_C	43C	85C
Z_7343930_C	43C	86C
Z_7343931_C	43C	86C
Z_7343932_A	43A	87A
Z_7343933_A	44A	86A
Z_7343934_G	45G	85G
Z_7343935_C	43C	81C
Z_7343936_C	43C	81C
Z_7343937_T	41T	81T
Z_7343938_C	42C	45T_1G_36C
Z_7343939_T	40T	82T
Z_7343940_G	39G	83G
Z_7343941_C	38C	83C
Z_7343942_A	39A	81A
Z_7343943_T	38T	82T
Z_7343944_G	38G	84G
Z_7343945_T	37T	83T
Z_7343946_T	37T	85T
Z_7343947_A	37A	84A
Z_7343948_T	37T	83T
Z_7343949_C	36C	83C
Z_7343950_C	38C	86C
Z_7343951_T	38T	84T
Z_7343952_C	37C	84C
Z_7343953_T	36T	83T
Z_7343954_G	36G	80G
Z_7343955_A	35A	81A
Z_7343956_A	33A	79A
Z_7343957_T	33T	79T
Z_7343958_G	33G	80G
Z_7343959_A	33A	80A
Z_7343960_T	33T	81T
Z_7343961_T	31T	83T
Z_7343962_T	32T	85T
Z_7343963_T	31T	84T
Z_7343964_A	32A	86A
Z_7343965_T	32T	86T

Z_7343966_T	31T	86T
Z_7343967_T	32T	87T
Z_7343968_A	32A	88A
Z_7343969_A	32A	88A
Z_7343970_A	33A	87A
Z_7343971_A	33A	86A
Z_7343972_G	33G	86G
Z_7343973_G	34G	86G
Z_7343974_C	35C	87C
Z_7343975_C	35C	85C
Z_7343976_C	34C	83C
Z_7343977_T	35T	83T
Z_7343978_T	35T	83T
Z_7343979_T	34T	83T
Z_7343980_T	35T	84T
Z_7343981_A	35A	84A
Z_7343982_A	37A	84A
Z_7343983_A	37A	85A
Z_7343984_G	33G	83G
Z_7343985_G	37G	84G
Z_7343986_G	37G	87G
Z_7343987_G	37G	87G
Z_7343988_G	36G	87G
Z_7343989_C	37C	87C
Z_7343990_A	38A	87A
Z_7343991_A	38A	87A
Z_7343992_G	37G	85G
Z_7343993_A	37A	85A
Z_7343994_A	38A	86A
Z_7343995_G	38G	85G
Z_7343996_G	36G	83G
Z_7343997_G	35G	80G
Z_7343998_A	35A	80A
Z_7343999_A	34A	79A
Z_7344000_T	33T	79T
Z_7344001_C	32C	79C
Z_7344002_G	32G	80G
Z_7344003_T	32T	79T
Z_7344004_C	31C	77C
Z_7344005_C	31C	77C
Z_7344006_T	33C	78T
Z_7344007_G	33G	78G
Z_7344008_C	32C	79C

Z_7344009_G	34G	76G
Z_7344010_T	34T	76T
Z_7344011_T	32T	75T
Z_7344012_T	35T	75T
Z_7344013_C	35C	75C
Z_7344014_A	35A	75A
Z_7344015_C	35C	75C
Z_7344016_A	35A	75A
Z_7344017_C	36C	75C
Z_7344018_C	34C	74C
Z_7344019_T	36T	74T
Z_7344020_G	36G	73G
Z_7344021_T	32T	71T
Z_7344022_G	33G	71G
Z_7344023_G	32G	71G
Z_7344024_C	33C	71C
Z_7344025_T	35T	70T
Z_7344026_G	37G	71G
Z_7344027_C	37C	71C
Z_7344028_T	36T	70T
Z_7344029_T	37T	72T
Z_7344030_T	37T	72T
Z_7344031_G	37G	71G
Z_7344032_T	38T	74T
Z_7344033_T	37T	73T
Z_7344034_C	36C	73C
Z_7344035_C	35C	73C
Z_7344036_A	34A	43A_28G
Z_7344037_C	36C	72C
Z_7344038_T	35T	70T
Z_7344039_C	34C	68C
Z_7344040_T	33T	67T
Z_7344041_A	35A	65A
Z_7344042_A	36A	67A
Z_7344043_A	36A	67A
Z_7344044_A	36A	68A
Z_7344045_A	36A	66A
Z_7344046_T	37T	66T
Z_7344047_A	36A	66A
Z_7344048_A	36A	66A
Z_7344049_A	36A	66A
Z_7344050_A	36A	65A
Z_7344051_T	35T	65T

Z_7344052_A	34A	66A
Z_7344053_A	35A	66A
Z_7344054_A	35A	67A
Z_7344055_A	36A	68A
Z_7344056_T	37T	66T
Z_7344057_T	37T	64T
Z_7344058_G	37G	65G
Z_7344059_G	36G	63G
Z_7344060_A	35A	63A
Z_7344061_A	35A	61A
Z_7344062_A	35A	59A
Z_7344063_C	37C	60C
Z_7344064_C	37C	60C
Z_7344065_G	38G	59G
Z_7344066_T	39T	60T
Z_7344067_T	37T	60T
Z_7344068_T	37T	60T
Z_7344069_C	39C	57C
Z_7344070_T	41T	59T
Z_7344071_G	42G	61G
Z_7344072_G	41G	62G
Z_7344073_G	43G	62G
Z_7344074_T	46T	62T
Z_7344075_C	44C	60C
Z_7344076_T	43T	61T
Z_7344077_G	42G	59G
Z_7344078_G	42G	58G
Z_7344079_G	41G	58G
Z_7344080_G	42G	56G
Z_7344081_G	42G	53G
Z_7344082_A	41A	52A
Z_7344083_A	40A	53A
Z_7344084_A	40A	53A
Z_7344085_C	41C	52C
Z_7344086_G	43G	52G
Z_7344087_C	41C	52C
Z_7344088_T	42T	51T
Z_7344089_G	41G	52G
Z_7344090_T	40T	52T
Z_7344091_C	41C	52C
Z_7344092_C	42C	51C
Z_7344093_A	42A	51A
Z_7344094_G	43G	51G

Z_7344095_G	41G	52G
Z_7344096_A	42A	52A
Z_7344097_C	42C	51C
Z_7344098_C	43C	51C
Z_7344099_T	45T	51T
Z_7344100_G	45G	51G
Z_7344101_A	46A	50A
Z_7344102_A	45A	50A
Z_7344103_A	46A	49A
Z_7344104_C	46C	49C
Z_7344105_A	47A	48A
Z_7344106_G	47G	47G
Z_7344107_C	43C	47C
Z_7344108_C	45C	47C
Z_7344109_C	44C	49C
Z_7344110_T	44T	50T
Z_7344111_C	42C	51C
Z_7344112_T	41T	48T
Z_7344113_C	41C	48C
Z_7344114_C	23A_1G_82C	48C
Z_7344115_C	17C	47C
Z_7344116_C	16C	50C
Z_7344117_A	16A	48A
Z_7344118_C	39C	50C
Z_7344119_C	40C	49C
Z_7344120_C	40C	52C
Z_7344121_A	25A	51A
Z_7344122_C	30C	53C
Z_7344123_C	31C	55C
Z_7344124_C	25C	54C
Z_7344125_C	25C	54C
Z_7344126_T	26C	33T_21C
Z_7344127_C	30C	50C
Z_7344128_A	29A	52A
Z_7344129_A	32A	54A
Z_7344130_A	32A	53A
Z_7344131_A	33A	53A
Z_7344132_C	32T	53C
Z_7344133_A	33A	56A
Z_7344134_G	32G	58G
Z_7344135_G	34G	58G
Z_7344136_G	36G	58G
Z_7344137_A	37A	57A

Z_7344138_A	37A	58A
Z_7344139_A	39A	56A
Z_7344140_T	40T	55T
Z_7344141_C	42C	56C
Z_7344142_A	42A	58A
Z_7344143_T	42T	58T
Z_7344144_G	40G	60G
Z_7344145_C	40C	60C
Z_7344146_C	41C	59C
Z_7344147_C	38C	57C
Z_7344148_T	38T	59T
Z_7344149_A	38G	58A
Z_7344150_T	39T	59T
Z_7344151_A	39A	60A
Z_7344152_A	39A	61A
Z_7344153_C	39C	61C
Z_7344154_A	39A	64A
Z_7344155_C	40C	65C
Z_7344156_G	39G	65G
Z_7344157_C	40C	64C
Z_7344158_A	42A	64A
Z_7344159_A	42A	64A
Z_7344160_T	40T	63T
Z_7344161_C	40C	67C
Z_7344162_C	41C	68C
Z_7344163_T	41T	67T
Z_7344164_C	42C	70C
Z_7344165_A	43A	69A
Z_7344166_G	41G	66G
Z_7344167_G	40G	66G
Z_7344168_G	39G	66G
Z_7344169_G	36G	65G
Z_7344170_C	37C	65C
Z_7344171_A	37A	65A
Z_7344172_T	36T	65T
Z_7344173_G	36G	66G
Z_7344174_A	37A	66A
Z_7344175_G	36G	66G
Z_7344176_A	37A	66A
Z_7344177_T	37T	66T
Z_7344178_G	37G	66G
Z_7344179_G	38G	68G
Z_7344180_T	38T	68T

Z_7344181_C	37C	72C
Z_7344182_T	39T	73T
Z_7344183_T	38T	76T
Z_7344184_T	37T	74T
Z_7344185_T	38T	75T
Z_7344186_G	38G	77G
Z_7344187_A	39A	78A
Z_7344188_A	40A	79A
Z_7344189_G	39G	79G
Z_7344190_T	39T	78T
Z_7344191_T	39T	79T
Z_7344192_G	39G	81G
Z_7344193_T	39T	82T
Z_7344194_A	41A	82A
Z_7344195_A	41A	82A
Z_7344196_A	39A	82A
Z_7344197_G	38G	82G
Z_7344198_A	39A	82A
Z_7344199_A	37A	81A
Z_7344200_A	37A	82A
Z_7344201_A	36A	82A
Z_7344202_G	39G	80G
Z_7344203_A	40A	80A
Z_7344204_A	39A	79A
Z_7344205_T	40T	76T
Z_7344206_A	39A	76A
Z_7344207_G	38G	75G
Z_7344208_G	39G	78G
Z_7344209_G	38G	35A_46G
Z_7344210_T	37T	83T
Z_7344211_T	36T	82T
Z_7344212_T	38T	82T
Z_7344213_A	38A	81A
Z_7344214_A	37A	83A
Z_7344215_G	37G	80G
Z_7344216_G	36G	80G
Z_7344217_G	37G	80G
Z_7344218_T	36T	78T
Z_7344219_G	37G	78G
Z_7344220_C	35C	33T_45C
Z_7344221_G	36G	80G
Z_7344222_A	37A	80A
Z_7344223_G	37G	78G

Z_7344224_G	37G	77G
Z_7344225_T	36T	77T
Z_7344226_G	37G	80G
Z_7344227_G	36G	80G
Z_7344228_G	35G	76G
Z_7344229_T	35T	75T
Z_7344230_G	35G	75G
Z_7344231_A	35A	73A
Z_7344232_A	35A	74A
Z_7344233_C	35C	75C
Z_7344234_C	33C	76C
Z_7344235_T	32T	76T
Z_7344236_G	32G	75G
Z_7344237_G	34G	74G
Z_7344238_G	34G	73G
Z_7344239_A	36A	72A
Z_7344240_C	36C	77C
Z_7344241_C	35C	77C
Z_7344242_T	36T	77T
Z_7344243_C	37C	76C
Z_7344244_C	36C	76C
Z_7344245_T	37T	73T
Z_7344246_G	37G	71G
Z_7344247_C	38C	69C
Z_7344248_A	38A	68A
Z_7344249_T	36T	30A_36T
Z_7344250_C	37C	65C
Z_7344251_C	38C	65C
Z_7344252_C	37T	66C
Z_7344253_G	37G	67G
Z_7344254_T	37T	69T
Z_7344255_G	37G	70G
Z_7344256_G	39G	66G
Z_7344257_G	38G	66G
Z_7344258_C	38C	67C
Z_7344259_T	38T	66T
Z_7344260_G	38G	67G
Z_7344261_C	39C	69C
Z_7344262_A	39A	71A
Z_7344263_C	40C	71C
Z_7344264_T	38T	72T
Z_7344265_T	38T	72T
Z_7344266_T	38T	72T

Z_7344267_T	37T	74T
Z_7344268_T	37T	72T
Z_7344269_A	37A	73A
Z_7344270_C	37C	73C
Z_7344271_C	37C	74C
Z_7344272_C	35C	74C
Z_7344273_A	35A	76A
Z_7344274_C	34C	75C
Z_7344275_C	33C	75C
Z_7344276_C	33C	72C
Z_7344277_A	33A	71A
Z_7344278_T	33T	68T
Z_7344279_T	34T	68T
Z_7344280_T	33T	68T
Z_7344281_C	32C	67C
Z_7344282_A	31A	65A
Z_7344283_A	31A	64A
Z_7344284_A	31A	64A
Z_7344285_G	31G	64G
Z_7344286_C	29G	30G_31C
Z_7344287_A	28G	31A_28G
Z_7344288_G	31G	59G
Z_7344289_G	32G	29A_32G
Z_7344290_A	33A	62A
Z_7344291_C	34C	63C
Z_7344292_A	34A	64A
Z_7344293_A	36A	64A
Z_7344294_A	37A	64A
Z_7344295_T	37T	65T
Z_7344296_A	36A	66A
Z_7344297_T	36T	67T
Z_7344298_G	36G	67G
Z_7344299_G	36G	65G
Z_7344300_G	37G	69G
Z_7344301_G	36G	71G
Z_7344302_C	36C	71C
Z_7344303_C	36C	70C
Z_7344304_C	35C	67C
Z_7344305_G	34G	65G
Z_7344306_C	36C	65C
Z_7344307_T	34T	64T
Z_7344308_G	34G	65G
Z_7344309_C	34C	64C

Z_7344310_T	34C	33T_31C
Z_7344311_T	34T	63T
Z_7344312_A	32A	62A
Z_7344313_G	31G	63G
Z_7344314_A	32A	63A
Z_7344315_C	31C	64C
Z_7344316_T	31T	62T
Z_7344317_G	28G	62G
Z_7344318_G	28G	62G
Z_7344319_C	28G	29G_32C
Z_7344320_C	28C	60C
Z_7344321_T	27T	58T
Z_7344322_T	27T	58T
Z_7344323_G	27G	58G
Z_7344324_C	26C	58C
Z_7344325_T	26T	58T
Z_7344326_C	24C	58C
Z_7344327_C	24C	57C
Z_7344328_T	23T	56T
Z_7344329_G	23G	57G
Z_7344330_C	15A_12T_10G_37C	80A_41T_20G_117C
Z_7344331_G	35G	64G
Z_7344332_C	34C	64C
Z_7344333_C	33C	65C
Z_7344334_G	18G	65G
Z_7344335_C	18C	64C
Z_7344336_C	21C	65C
Z_7344337_T	22T	65T
Z_7344338_G	22G	65G
Z_7344339_G	23G	66G
Z_7344340_C	24C	66C
Z_7344341_A	25A	67A
Z_7344342_G	25G	67G
Z_7344343_G	25A	67G
Z_7344344_C	25C	66C
Z_7344345_A	25A	65A
Z_7344346_G	26G	66G
Z_7344347_A	26A	65A
Z_7344348_A	26A	65A
Z_7344349_A	27A	63A
Z_7344350_T	27T	63T
Z_7344351_T	29T	63T
Z_7344352_G	29G	61G

Z_7344353_A	29A	62A
Z_7344354_A	30A	61A
Z_7344355_G	29G	63G
Z_7344356_G	31G	35G_24C
Z_7344357_G	31G	58G
Z_7344358_G	31G	57G
Z_7344359_C	31C	58C
Z_7344360_G	32G	59G
Z_7344361_A	32A	57A
Z_7344362_T	33T	55T
Z_7344363_G	35G	56G
Z_7344364_G	35G	55G
Z_7344365_A	35A	55A
Z_7344366_C	36C	56C
Z_7344367_T	36T	57T
Z_7344368_T	36T	57T
Z_7344369_C	33C	56C
Z_7344370_C	37C	56C
Z_7344371_T	38T	56T
Z_7344372_A	38A	55A
Z_7344373_C	39C	55C
Z_7344374_C	38C	54C
Z_7344375_C	39C	57C
Z_7344376_T	38T	57T
Z_7344377_C	39C	57C
Z_7344378_T	38T	57T
Z_7344379_G	38G	57G
Z_7344380_A	38A	56A
Z_7344381_C	40C	58C
Z_7344382_C	40C	58C
Z_7344383_C	40C	58C
Z_7344384_T	39T	61T
Z_7344385_A	39A	60A
Z_7344386_A	40A	59A
Z_7344387_G	39G	59G
Z_7344388_C	39C	58C
Z_7344389_T	39T	58T
Z_7344390_C	38C	58C
Z_7344391_C	39C	57C
Z_7344392_A	39A	56A
Z_7344393_A	39A	55A
Z_7344394_A	39A	54A
Z_7344395_G	38G	53G

Z_7344396_C	38C	52C
Z_7344397_A	35A	52A
Z_7344398_T	35T	52T
Z_7344399_T	35G	29T_22G
Z_7344400_G	34G	51G
Z_7344401_G	33G	51G
Z_7344402_T	33T	52T
Z_7344403_C	34C	53C
Z_7344404_C	34C	54C
Z_7344405_A	33A	54A
Z_7344406_T	30C	31T_21C
Z_7344407_C	29C	55C
Z_7344408_C	29C	54C
Z_7344409_A	29A	54A
Z_7344410_G	28G	54G
Z_7344411_C	27C	54C
Z_7344412_C	27C	54C
Z_7344413_A	27A	55A
Z_7344414_A	27A	55A
Z_7344415_G	27G	54G
Z_7344416_T	26T	53T
Z_7344417_G	60A_15T_56G_15C	56A_14T_80G_14C
Z_7344418_A	1C	32A
Z_7344419_C	null	33C
Z_7344420_T	null	33T
Z_7344421_A	null	32A
Z_7344422_G	null	32G
Z_7344423_A	null	33A
Z_7344424_A	null	32A
Z_7344425_G	null	31G
Z_7344426_G	26G	53G
Z_7344427_C	26C	54C
Z_7344428_T	27T	55T
Z_7344429_G	27G	54G
Z_7344430_G	26G	52G
Z_7344431_C	27C	53C
Z_7344432_A	27A	54A
Z_7344433_C	27C	56C
Z_7344434_A	27A	55A
Z_7344435_A	28A	55A
Z_7344436_A	29A	54A
Z_7344437_T	29C	30T_25C
Z_7344438_G	31G	55G

Z_7344439_T	32T	57T
Z_7344440_T	32T	57T
Z_7344441_G	32G	57G
Z_7344442_A	33A	58A
Z_7344443_G	33G	57G
Z_7344444_A	35A	56A
Z_7344445_C	36C	56C
Z_7344446_G	36G	27A_31G
Z_7344447_C	37C	58C
Z_7344448_C	39C	59C
Z_7344449_G	35G	59G
Z_7344450_A	37A	57A
Z_7344451_G	39G	63G
Z_7344452_G	40G	65G
Z_7344453_C	41C	65C
Z_7344454_T	41T	64T
Z_7344455_G	40G	66G
Z_7344456_C	40C	66C
Z_7344457_T	41T	65T
Z_7344458_G	41G	65G
Z_7344459_T	41T	64T
Z_7344460_A	40A	65A
Z_7344461_G	40G	67G
Z_7344462_T	39T	67T
Z_7344463_A	39A	68A
Z_7344464_C	39C	71C
Z_7344465_G	38G	28A_43G
Z_7344466_A	37A	71A
Z_7344467_G	36G	71G
Z_7344468_A	36A	70A
Z_7344469_T	111T_99C	99T_45C
Z_7344470_C	12C	51C
Z_7344471_T	12T	56T
Z_7344472_C	12C	57C
Z_7344473_T	12T	57T
Z_7344474_C	12C	58C
Z_7344475_T	11T	76T
Z_7344476_C	11C	74C
Z_7344477_T	38T	75T
Z_7344478_C	39C	75C
Z_7344479_T	39T	74T
Z_7344480_C	39C	73C
Z_7344481_T	41T	72T

Z_7344482_C	38C	73C
Z_7344483_T	40T	73T
Z_7344484_C	41C	74C
Z_7344485_T	41T	74T
Z_7344486_C	34C	65C
Z_7344487_T	38T	67T
Z_7344488_C	36C	67C
Z_7344489_T	36T	67T
Z_7344490_C	36C	68C
Z_7344491_T	36T	68T
Z_7344492_C	37C	25A_43C
Z_7344493_T	37T	68T
Z_7344494_G	37G	68G
Z_7344495_T	37T	68T
Z_7344496_T	37T	68T
Z_7344497_G	38G	67G
Z_7344498_C	38C	65C
Z_7344499_A	38A	65A
Z_7344500_T	38T	66T
Z_7344501_T	38T	65T
Z_7344502_T	39T	60T
Z_7344503_T	40T	61T
Z_7344504_C	38C	62C
Z_7344505_C	41C	61C
Z_7344506_A	41A	62A
Z_7344507_C	39C	63C
Z_7344508_A	41A	62A
Z_7344509_C	41C	63C
Z_7344510_A	42A	63A
Z_7344511_A	43A	64A
Z_7344512_T	45T	63T
Z_7344513_C	45C	65C
Z_7344514_C	43C	63C
Z_7344515_T	42T	62T
Z_7344516_C	41C	64C
Z_7344517_T	40T	66T
Z_7344518_T	39T	66T
Z_7344519_T	42T	66T
Z_7344520_A	41A	66A
Z_7344521_A	41A	68A
Z_7344522_G	38G	69G
Z_7344523_A	39A	66A
Z_7344524_G	39G	69G

Z_7344525_G	37G	68G
Z_7344526_C	39C	71C
Z_7344527_G	41G	71G
Z_7344528_A	40A	69A
Z_7344529_C	38C	70C
Z_7344530_A	38A	69A
Z_7344531_A	36A	70A
Z_7344532_C	35C	71C
Z_7344533_G	37G	70G
Z_7344534_A	36A	68A
Z_7344535_A	35A	69A
Z_7344536_T	38T	72T
Z_7344537_G	38G	70G
Z_7344538_C	37C	72C
Z_7344539_A	37A	72A
Z_7344540_A	38A	72A
Z_7344541_C	40C	71C
Z_7344542_A	39A	71A
Z_7344543_A	40A	72A
Z_7344544_T	40T	73T
Z_7344545_T	40T	75T
Z_7344546_G	39G	72G
Z_7344547_C	37C	70C
Z_7344548_T	37T	72T
Z_7344549_G	41G	72G
Z_7344550_T	41T	71T
Z_7344551_G	39G	71G
Z_7344552_T	41T	70T
Z_7344553_G	39G	69G
Z_7344554_T	37T	70T
Z_7344555_T	36T	71T
Z_7344556_T	37T	71T
Z_7344557_A	36A	71A
Z_7344558_G	37G	69G
Z_7344559_G	37G	66G
Z_7344560_G	38G	64G
Z_7344561_A	37A	64A
Z_7344562_G	37G	65G
Z_7344563_G	37G	66G
Z_7344564_G	37G	65G
Z_7344565_T	37T	65T
Z_7344566_G	37G	65G
Z_7344567_T	36T	65T

Z_7344568_G	36G	65G
Z_7344569_T	36T	65T
Z_7344570_G	36G	67G
Z_7344571_T	37T	67T
Z_7344572_G	36G	66G
Z_7344573_T	36T	67T
Z_7344574_G	37G	66G
Z_7344575_T	36T	65T
Z_7344576_C	36C	65C
Z_7344577_T	37T	65T
Z_7344578_A	37A	65A
Z_7344579_T	36T	66T
Z_7344580_G	35G	71G
Z_7344581_T	35T	70T
Z_7344582_G	35G	69G
Z_7344583_T	34T	72T
Z_7344584_G	34G	71G
Z_7344585_G	35G	72G
Z_7344586_A	35A	74A
Z_7344587_A	38A	74A
Z_7344588_C	38C	74C
Z_7344589_G	37G	75G
Z_7344590_G	36G	76G
Z_7344591_T	38T	75T
Z_7344592_G	38G	77G
Z_7344593_C	38C	78C
Z_7344594_A	38A	79A
Z_7344595_A	37A	78A
Z_7344596_T	36T	78T
Z_7344597_T	35T	79T
Z_7344598_A	34A	79A
Z_7344599_T	33T	80T
Z_7344600_T	31T	81T
Z_7344601_T	32T	81T
Z_7344602_G	31G	81G
Z_7344603_G	31G	81G
Z_7344604_T	31T	81T
Z_7344605_G	31G	81G
Z_7344606_A	28A	79A
Z_7344607_A	27A	79A
Z_7344608_G	27G	78G
Z_7344609_A	26A	79A
Z_7344610_T	26T	79T

Z_7344611_T	25T	78T
Z_7344612_G	27G	76G
Z_7344613_G	26G	76G
Z_7344614_A	27A	78A
Z_7344615_G	27G	78G
Z_7344616_G	27G	78G
Z_7344617_T	29T	77T
Z_7344618_G	29G	78G
Z_7344619_T	25T	78T
Z_7344620_C	25C	78C
Z_7344621_A	26A	76A
Z_7344622_C	25C	75C
Z_7344623_C	25C	77C
Z_7344624_C	26C	77C
Z_7344625_T	27T	79T
Z_7344626_G	28G	78G
Z_7344627_A	28A	79A
Z_7344628_C	29C	78C
Z_7344629_A	30A	78A
Z_7344630_A	30A	77A
Z_7344631_T	30T	77T
Z_7344632_T	31T	78T
Z_7344633_G	31G	80G
Z_7344634_A	31A	82A
Z_7344635_G	31G	81G
Z_7344636_A	31A	80A
Z_7344637_A	31A	81A
Z_7344638_T	32T	79T
Z_7344639_G	32G	78G
Z_7344640_A	32A	76A
Z_7344641_A	32A	75A
Z_7344642_G	32G	75G
Z_7344643_C	31C	73C
Z_7344644_A	32A	72A
Z_7344645_C	35C	72C
Z_7344646_G	34G	73G
Z_7344647_G	33G	72G
Z_7344648_G	31G	75G
Z_7344649_A	31A	78A
Z_7344650_G	32G	77G
Z_7344651_G	34G	78G
Z_7344652_A	33A	79A
Z_7344653_C	34C	80C

Z_7344654_C	36C	81C
Z_7344655_A	35A	81A
Z_7344656_G	33G	79G
Z_7344657_A	34A	77A
Z_7344658_A	33A	80A
Z_7344659_A	34A	79A
Z_7344660_T	35T	79T
Z_7344661_G	34G	80G
Z_7344662_A	34A	76A
Z_7344663_C	32C	77C
Z_7344664_A	34A	77A
Z_7344665_A	35A	75A
Z_7344666_T	33T	75T
Z_7344667_C	34C	75C
Z_7344668_A	33A	77A
Z_7344669_A	34A	77A
Z_7344670_A	34A	78A
Z_7344671_A	34A	78A
Z_7344672_G	34G	77G
Z_7344673_C	33C	77C
Z_7344674_T	33T	74T
Z_7344675_T	34T	72T
Z_7344676_C	33C	72C
Z_7344677_C	33C	73C
Z_7344678_A	35A	71A
Z_7344679_C	34C	72C
Z_7344680_C	35C	72C
Z_7344681_A	34A	70A
Z_7344682_G	33G	68G
Z_7344683_G	33G	70G
Z_7344684_G	33G	70G
Z_7344685_T	33T	69T
Z_7344686_A	33A	69A
Z_7344687_A	31A	68A
Z_7344688_A	32A	67A
Z_7344689_G	32G	67G
Z_7344690_T	32T	67T
Z_7344691_G	32G	67G
Z_7344692_T	32T	66T
Z_7344693_T	32T	66T
Z_7344694_A	31A	64A
Z_7344695_A	30A	62A
Z_7344696_T	29T	62T

Z_7344697_A	29A	61A
Z_7344698_A	27A	61A
Z_7344699_C	26C	60C
Z_7344700_A	25A	61A
Z_7344701_A	25A	60A
Z_7344702_C	24C	60C
Z_7344703_C	24C	59C
Z_7344704_T	24T	58T
Z_7344705_A	23A	57A
Z_7344706_A	24A	57A
Z_7344707_C	24C	57C
Z_7344708_T	23T	56T
Z_7344709_G	24G	54G
Z_7344710_C	25C	53C
Z_7344711_T	24T	52T
Z_7344712_T	23T	51T
Z_7344713_T	22T	50T
Z_7344714_C	20C	49C
Z_7344715_A	19A	50A
Z_7344716_C	19C	49C
Z_7344717_A	18A	49A
Z_7344718_T	19T	47T
Z_7344719_A	19A	47A
Z_7344720_C	18C	45C
Z_7344721_C	16C	45C
Z_7344722_A	16A	44A
Z_7344723_T	15T	44T
Z_7344724_G	15G	44G
Z_7344725_A	15A	44A
Z_7344726_T	14T	41T
Z_7344727_G	12G	41G
Z_7344728_A	12A	38A
Z_7344729_T	12T	36T
Z_7344730_G	11G	36G
Z_7344731_A	11A	37A
Z_7344732_T	11T	36T
Z_7344733_G	11G	37G
Z_7344734_A	11A	38A
Z_7344735_T	10T	39T
Z_7344736_G	10G	39G
Z_7344737_G	11G	39G
Z_7344738_C	11C	39C
Z_7344739_C	10C	39C

Z_7344740_T	10T	38T
Z_7344741_G	10G	33G
Z_7344742_C	9C	32C
Z_7344743_C	8C	29C
Z_7344744_C	8C	28C
Z_7344745_A	7A_1T_18C	26A
Z_7344746_C	9C	23C
Z_7344747_C	9C	23C
Z_7344748_C	9C	23C
Z_7344749_A	8A	21A
Z_7344750_C	9C	21C
Z_7344751_C	9C	21C
Z_7344752_C	8C	21C
Z_7344753_C	8C	18C
Z_7344754_C	8C	18C
Z_7344755_C	8C	17C
Z_7344756_A	8A	13A
Z_7344757_C	7C	14C
Z_7344758_C	7C	14C
Z_7344759_G	7G	13G
Z_7344760_G	7G	14G
Z_7344761_G	7G	17G
Z_7344762_C	7C	8A_11C
Z_7344763_C	7C	18C
Z_7344764_C	6C	18C
Z_7344765_C	7C	18C
Z_7344766_C	8C	18C
Z_7344767_T	9T	18T
Z_7344768_T	9T	18T
Z_7344769_G	9G	18G
Z_7344770_C	10C	18C
Z_7344771_T	10T	19T
Z_7344772_C	10C	19C
Z_7344773_C	10C	19C
Z_7344774_C	11C	21C
Z_7344775_C	11C	21C
Z_7344776_A	10A	20A
Z_7344777_G	9G	21G
Z_7344778_A	10A	20A
Z_7344779_A	11A	21A
Z_7344780_A	10A	21A
Z_7344781_G	9G	23G
Z_7344782_C	10C	24C

Z_7344783_T	10T	25T
Z_7344784_A	10A	25A
Z_7344785_A	11A	25A
Z_7344786_C	11C	26C
Z_7344787_A	11A	28A
Z_7344788_G	12G	29G
Z_7344789_A	12A	30A
Z_7344790_A	12A	30A
Z_7344791_G	14G	30G
Z_7344792_A	14A	28A
Z_7344793_C	14C	33C
Z_7344794_A	14A	33A
Z_7344795_A	14A	33A
Z_7344796_C	14C	34C
Z_7344797_C	14C	34C
Z_7344798_T	14T	35T
Z_7344799_C	14C	37C
Z_7344800_G	14G	38G
Z_7344801_C	14C	40C
Z_7344802_C	15C	40C
Z_7344803_T	15T	41T
Z_7344804_T	14T	41T
Z_7344805_C	15C	44C
Z_7344806_T	14T	45T
Z_7344807_C	15C	46C
Z_7344808_A	16A	48A
Z_7344809_G	16G	52G
Z_7344810_T	17T	53T
Z_7344811_T	16T	53T
Z_7344812_C	17C	53C
Z_7344813_A	17A	54A
Z_7344814_G	17G	54G
Z_7344815_C	16C	54C
Z_7344816_C	16C	54C
Z_7344817_A	17A	55A
Z_7344818_C	17C	56C
Z_7344819_T	18T	57T
Z_7344820_C	18C	56C
Z_7344821_A	18A	59A
Z_7344822_T	18T	58T
Z_7344823_A	19A	58A
Z_7344824_A	19A	59A
Z_7344825_T	19T	60T

Z_7344826_A	18A	61A
Z_7344827_T	19T	62T
Z_7344828_T	19T	61T
Z_7344829_C	19C	61C
Z_7344830_T	19T	63T
Z_7344831_G	20G	63G
Z_7344832_G	20G	64G
Z_7344833_C	20C	64C
Z_7344834_T	20T	65T
Z_7344835_C	19G	27G_40C
Z_7344836_T	18T	67T
Z_7344837_C	17C	68C
Z_7344838_T	17T	68T
Z_7344839_C	17C	69C
Z_7344840_T	16T	69T
Z_7344841_C	17C	72C
Z_7344842_T	17C	44T_30C
Z_7344843_G	17G	75G
Z_7344844_C	16C	75C
Z_7344845_A	16A	75A
Z_7344846_T	16T	76T
Z_7344847_G	16G	76G
Z_7344848_G	15G	76G
Z_7344849_C	14C	77C
Z_7344850_G	14G	79G
Z_7344851_A	15A	79A
Z_7344852_G	15G	78G
Z_7344853_C	16C	77C
Z_7344854_A	16A	80A
Z_7344855_T	15T	80T
Z_7344856_C	15C	78C
Z_7344857_C	16C	76C
Z_7344858_A	15A	77A
Z_7344859_G	14G	77G
Z_7344860_C	15C	77C
Z_7344861_T	14T	77T
Z_7344862_G	14G	77G
Z_7344863_C	14C	78C
Z_7344864_A	16A	78A
Z_7344865_G	16G	78G
Z_7344866_G	15G	79G
Z_7344867_G	16G	78G
Z_7344868_C	16C	81C

Z_7344869_C	16C	79C
Z_7344870_G	17G	84G
Z_7344871_G	18G	83G
Z_7344872_T	18T	85T
Z_7344873_T	18T	83T
Z_7344874_T	18T	85T
Z_7344875_T	17T	86T
Z_7344876_A	17A	84A
Z_7344877_A	18A	83A
Z_7344878_G	17G	82G
Z_7344879_G	17G	82G
Z_7344880_A	17A	84A
Z_7344881_A	18A	84A
Z_7344882_G	17G	84G
Z_7344883_A	17A	83A
Z_7344884_T	18T	81T
Z_7344885_C	20C	84C
Z_7344886_T	22T	85T
Z_7344887_A	21A	85A
Z_7344888_A	21A	83A
Z_7344889_A	20A	84A
Z_7344890_A	20A	84A
Z_7344891_T	21T	83T
Z_7344892_T	21T	84T
Z_7344893_A	22A	87A
Z_7344894_A	22A	84A
Z_7344895_A	22A	85A
Z_7344896_T	22T	82T
Z_7344897_T	23T	84T
Z_7344898_G	23G	84G
Z_7344899_G	23G	84G
Z_7344900_A	24A	83A
Z_7344901_A	23A	82A
Z_7344902_G	23G	82G
Z_7344903_C	23C	81C
Z_7344904_A	23A	78A
Z_7344905_G	23G	76G
Z_7344906_C	23C	77C
Z_7344907_T	23T	77T
Z_7344908_T	22A	35A_40T
Z_7344909_A	24A	40A_35C
Z_7344910_A	25A	74A
Z_7344911_A	23A	74A

Z_7344912_A	24A	74A
Z_7344913_A	26A	74A
Z_7344914_A	26A	73A
Z_7344915_A	26A	74A
Z_7344916_T	26T	73T
Z_7344917_G	27G	73G
Z_7344918_G	27G	73G
Z_7344919_A	27A	72A
Z_7344920_A	27A	73A
Z_7344921_A	26A	72A
Z_7344922_G	26G	71G
Z_7344923_T	27T	71T
Z_7344924_A	27A	71A
Z_7344925_T	27T	67T
Z_7344926_T	26T	69T
Z_7344927_C	27C	69C
Z_7344928_T	27T	69T
Z_7344929_A	28A	67A
Z_7344930_A	29A	66A
Z_7344931_T	28T	66T
Z_7344932_T	28T	65T
Z_7344933_A	28A	66A
Z_7344934_A	27A	67A
Z_7344935_C	28C	67C
Z_7344936_T	28T	65T
Z_7344937_G	28G	64G
Z_7344938_C	30C	64C
Z_7344939_C	31C	65C
Z_7344940_C	31C	65C
Z_7344941_C	29C	65C
Z_7344942_T	30T	65T
Z_7344943_G	30G	68G
Z_7344944_G	30G	68G
Z_7344945_G	30G	66G
Z_7344946_G	31G	66G
Z_7344947_C	29C	66C
Z_7344948_T	32T	67T
Z_7344949_C	31C	67C
Z_7344950_G	32G	65G
Z_7344951_T	31T	65T
Z_7344952_C	31C	66C
Z_7344953_A	31A	67A
Z_7344954_G	30G	67G

Z_7344955_T	28T	69T
Z_7344956_G	26G	69G
Z_7344957_T	26T	71T
Z_7344958_C	26C	71C
Z_7344959_G	26G	70G
Z_7344960_T	25T	70T
Z_7344961_C	24C	68C
Z_7344962_A	25A	68A
Z_7344963_T	23T	66T
Z_7344964_T	23T	65T
Z_7344965_T	25T	61T
Z_7344966_A	25A	60A
Z_7344967_T	24T	60T
Z_7344968_T	24T	60T
Z_7344969_C	24C	59C
Z_7344970_C	23C	61C
Z_7344971_A	23A	61A
Z_7344972_C	25C	61C
Z_7344973_C	24C	63C
Z_7344974_A	25A	65A
Z_7344975_G	26G	64G
Z_7344976_A	25A	63A
Z_7344977_G	25G	63G
Z_7344978_G	25G	63G
Z_7344979_A	24A	64A
Z_7344980_G	24G	63G
Z_7344981_C	22C	64C
Z_7344982_C	24C	64C
Z_7344983_A	22G	33A_30G
Z_7344984_G	24G	62G
Z_7344985_A	24G	33A_28G
Z_7344986_C	25C	64C
Z_7344987_T	25T	63T
Z_7344988_G	25G	59G
Z_7344989_G	25G	59G
Z_7344990_T	25T	59T
Z_7344991_T	26T	59T
Z_7344992_C	27C	61C
Z_7344993_A	26A	61A
Z_7344994_A	27A	64A
Z_7344995_A	27A	66A
Z_7344996_A	26A	66A
Z_7344997_C	27C	66C

Z_7344998_T	27T	65T
Z_7344999_A	27A	66A
Z_7345000_G	24G	67G
Z_7345001_G	27G	68G
Z_7345002_T	27T	68T
Z_7345003_A	28A	68A
Z_7345004_G	27G	70G
Z_7345005_G	25G	70G
Z_7345006_A	28A	69A
Z_7345007_T	28T	72T
Z_7345008_T	26T	71T
Z_7345009_C	25C	73C
Z_7345010_C	23C	73C
Z_7345011_C	25C	72C
Z_7345012_C	24C	72C
Z_7345013_A	26A	73A
Z_7345014_A	27A	73A
Z_7345015_A	28A	71A
Z_7345016_T	27T	73T
Z_7345017_G	27G	72G
Z_7345018_T	25T	73T
Z_7345019_C	25C	73C
Z_7345020_T	24T	73T
Z_7345021_C	24C	72C
Z_7345022_C	24C	72C
Z_7345023_T	26T	72T
Z_7345024_G	31G	72G
Z_7345025_C	32C	75C
Z_7345026_A	33A	76A
Z_7345027_G	34G	77G
Z_7345028_T	35T	77T
Z_7345029_T	36T	77T
Z_7345030_G	36G	77G
Z_7345031_C	36C	77C
Z_7345032_T	36T	75T
Z_7345033_T	38T	75T
Z_7345034_T	38T	73T
Z_7345035_G	36G	73G
Z_7345036_C	37C	72C
Z_7345037_T	39T	72T
Z_7345038_A	40A	71A
Z_7345039_A	44A	73A
Z_7345040_A	44A	73A

Z_7345041_A	45A	76A
Z_7345042_C	43C	77C
Z_7345043_T	46T	76T
Z_7345044_G	45G	75G
Z_7345045_A	44A	76A
Z_7345046_A	43A	76A
Z_7345047_A	44A	74A
Z_7345048_C	45C	72C
Z_7345049_T	44T	73T
Z_7345050_T	46T	71T
Z_7345051_G	48G	73G
Z_7345052_G	45G	71G
Z_7345053_G	46G	70G
Z_7345054_G	45G	71G
Z_7345055_A	45A	71A
Z_7345056_T	45T	71T
Z_7345057_A	45A	71A
Z_7345058_G	46G	70G
Z_7345059_A	45A	70A
Z_7345060_G	46G	70G
Z_7345061_T	45T	70T
Z_7345062_G	43G	69G
Z_7345063_C	46C	71C
Z_7345064_T	44T	71T
Z_7345065_C	44C	69C
Z_7345066_C	42C	70C
Z_7345067_T	43T	70T
Z_7345068_A	44A	69A
Z_7345069_C	46C	68C
Z_7345070_A	46A	69A
Z_7345071_T	44T	69T
Z_7345072_A	46A	68A
Z_7345073_T	45T	68T
Z_7345074_G	44G	67G
Z_7345075_G	43G	67G
Z_7345076_A	43A	66A
Z_7345077_G	42G	67G
Z_7345078_G	43G	66G
Z_7345079_T	42T	67T
Z_7345080_T	45T	66T
Z_7345081_C	45C	66C
Z_7345082_T	44T	67T
Z_7345083_G	45G	67G

Z_7345084_T	45T	68T
Z_7345085_C	44C	68C
Z_7345086_C	45C	68C
Z_7345087_A	46A	66A
Z_7345088_G	45G	67G
Z_7345089_C	46C	65C
Z_7345090_C	46C	63C
Z_7345091_C	47C	63C
Z_7345092_T	47T	62T
Z_7345093_T	45T	65T
Z_7345094_G	40G	63G
Z_7345095_T	39T	63T
Z_7345096_G	38G	61G
Z_7345097_G	37G	61G
Z_7345098_A	36A	61A
Z_7345099_C	35C	59C
Z_7345100_A	75A_20T	109A
Z_7345101_A	15A	34A
Z_7345102_A	15A	32A
Z_7345103_T	null	29T
Z_7345104_A	22A	52A
Z_7345105_A	22A	52A
Z_7345106_A	24A	53A
Z_7345107_T	24C	28T_26C
Z_7345108_G	29G	55G
Z_7345109_C	29C	55C
Z_7345110_A	29A	55A
Z_7345111_C	29C	54C
Z_7345112_A	30A	56A
Z_7345113_G	31G	56G
Z_7345114_G	31G	57G
Z_7345115_G	33G	57G
Z_7345116_G	33G	56G
Z_7345117_C	35C	55C
Z_7345118_T	36T	55T
Z_7345119_A	36A	56A
Z_7345120_T	36T	58T
Z_7345121_A	36A	59A
Z_7345122_A	36A	60A
Z_7345123_A	36A	59A
Z_7345124_A	35A	57A
Z_7345125_A	33A	56A
Z_7345126_C	33C	56C

Z_7345127_C	36C	55C
Z_7345128_T	37T	55T
Z_7345129_A	39A	56A
Z_7345130_T	39T	56T
Z_7345131_T	41T	56T
Z_7345132_C	41C	57C
Z_7345133_T	41T	58T
Z_7345134_T	41T	56T
Z_7345135_C	42C	56C
Z_7345136_T	42T	55T
Z_7345137_C	39C	54C
Z_7345138_T	39G	26A_27T
Z_7345139_G	42G	53G
Z_7345140_T	43T	51T
Z_7345141_T	43T	51T
Z_7345142_A	43A	51A
Z_7345143_G	41G	52G
Z_7345144_A	44A	53A
Z_7345145_A	45A	53A
Z_7345146_A	44A	53A
Z_7345147_G	45G	54G
Z_7345148_C	45C	54C
Z_7345149_A	47A	53A
Z_7345150_G	48G	51G
Z_7345151_G	48A	22A_29G
Z_7345152_C	47C	51C
Z_7345153_T	48T	51T
Z_7345154_G	47G	22A_30G
Z_7345155_A	47A	53A
Z_7345156_C	47C	53C
Z_7345157_C	47C	53C
Z_7345158_C	47C	52C
Z_7345159_T	49T	52T
Z_7345160_G	49G	56G
Z_7345161_T	48T	57T
Z_7345162_G	48C	34G_25C
Z_7345163_C	47C	57C
Z_7345164_A	50A	57A
Z_7345165_C	50C	56C
Z_7345166_A	51A	57A
Z_7345167_C	50C	56C
Z_7345168_A	51A	54A
Z_7345169_C	53C	52C

Z_7345170_A	54A	52A
Z_7345171_C	54C	53C
Z_7345172_C	54C	53C
Z_7345173_C	54C	52C
Z_7345174_G	53G	52G
Z_7345175_G	53G	52G
Z_7345176_C	49C	54C
Z_7345177_C	52C	53C
Z_7345178_G	52G	52G
Z_7345179_C	52C	52C
Z_7345180_C	52C	51C
Z_7345181_T	55T	50T
Z_7345182_T	55T	50T
Z_7345183_T	56T	49T
Z_7345184_G	56G	48G
Z_7345185_C	55C	20G_27C
Z_7345186_T	54T	47T
Z_7345187_A	53A	47A
Z_7345188_T	52T	46T
Z_7345189_G	51G	45G
Z_7345190_A	52A	44A
Z_7345191_A	54A	44A
Z_7345192_G	55G	17T_25G
Z_7345193_A	53A	42A
Z_7345194_A	54A	42A
Z_7345195_T	55T	25T_17C
Z_7345196_T	55T	42T
Z_7345197_T	53T	42T
Z_7345198_G	52G	42G
Z_7345199_G	51G	42G
Z_7345200_A	50A	42A
Z_7345201_A	49A	41A
Z_7345202_G	50G	41G
Z_7345203_A	51A	41A
Z_7345204_T	54T	41T
Z_7345205_G	53G	41G
Z_7345206_C	52C	40C
Z_7345207_T	54T	38T
Z_7345208_A	55A	38A
Z_7345209_T	53T	38T
Z_7345210_T	52T	38T
Z_7345211_C	53C	39C
Z_7345212_T	50T	39T

Z_7345213_T	50T	60A_49T_60G_60C
Z_7345214_G	49G	28G
Z_7345215_A	49A	17A
Z_7345216_A	49A	17A
Z_7345217_A	49A	17A
Z_7345218_C	48C	17C
Z_7345219_A	46A	18A
Z_7345220_A	46A	17A
Z_7345221_C	45C	18C
Z_7345222_C	45C	18C
Z_7345223_G	45G	17G
Z_7345224_C	43C	17C
Z_7345225_C	43C	17C
Z_7345226_A	43A	17A
Z_7345227_C	42C	17C
Z_7345228_G	41G	17G
Z_7345229_G	39G	17G
Z_7345230_T	38T	17T
Z_7345231_G	38G	17G
Z_7345232_G	38G	17G
Z_7345233_T	28A_38T_28G	28T
Z_7345234_G	8G	28G
Z_7345235_A	6G	17A_12G
Z_7345236_T	33T	37T
Z_7345237_G	32G	37G
Z_7345238_G	32G	39G
Z_7345239_T	29T	41T
Z_7345240_G	30G	41G
Z_7345241_G	29G	43G
Z_7345242_G	29G	42G
Z_7345243_G	28G	42G
Z_7345244_A	28A	42A
Z_7345245_T	27T	41T
Z_7345246_A	26A	41A
Z_7345247_A	27A	41A
Z_7345248_T	28T	41T
Z_7345249_G	27G	43G
Z_7345250_G	27G	43G
Z_7345251_G	28G	43G
Z_7345252_G	28G	44G
Z_7345253_A	27A	45A
Z_7345254_C	25C	45C
Z_7345255_T	25T	43T

Z_7345256_G	25G	46G
Z_7345257_A	24A	45A
Z_7345258_A	26A	45A
Z_7345259_G	25G	45G
Z_7345260_T	25C	27T_19C
Z_7345261_C	25C	46C
Z_7345262_C	24C	47C
Z_7345263_C	23C	47C
Z_7345264_T	23T	47T
Z_7345265_A	23A	47A
Z_7345266_A	23A	48A
Z_7345267_A	23A	51A
Z_7345268_G	24G	51G
Z_7345269_A	23A	50A
Z_7345270_T	24T	51T
Z_7345271_A	24A	49A
Z_7345272_T	23T	49T
Z_7345273_C	23T	49C
Z_7345274_G	23G	17A_30G
Z_7345275_T	22T	44T
Z_7345276_T	22T	43T
Z_7345277_T	19T	42T
Z_7345278_T	19T	25T_17G
Z_7345279_T	19T	42T
Z_7345280_T	20T	25T_17C
Z_7345281_C	20C	45C
Z_7345282_C	22C	45C
Z_7345283_A	23A	45A
Z_7345284_A	23A	45A
Z_7345285_G	23G	45G
Z_7345286_C	24C	44C
Z_7345287_T	23T	43T
Z_7345288_T	22T	43T
Z_7345289_G	21G	43G
Z_7345290_G	21G	43G
Z_7345291_C	21C	43C
Z_7345292_T	22T	42T
Z_7345293_G	22G	43G
Z_7345294_A	25A	43A
Z_7345295_G	25G	43G
Z_7345296_A	25A	44A
Z_7345297_A	27A	43A
Z_7345298_G	27G	44G

Z_7345299_T	27T	44T
Z_7345300_A	27A	45A
Z_7345301_T	26T	45T
Z_7345302_T	27T	44T
Z_7345303_C	27C	44C
Z_7345304_G	26G	44G
Z_7345305_C	27C	21T_23C
Z_7345306_T	28T	41T
Z_7345307_G	28G	41G
Z_7345308_T	28T	41T
Z_7345309_T	30T	41T
Z_7345310_A	30A	42A
Z_7345311_G	30G	41G
Z_7345312_C	30C	40C
Z_7345313_C	30C	41C
Z_7345314_A	30A	39A
Z_7345315_C	30C	38C
Z_7345316_C	30C	38C
Z_7345317_C	29C	37C
Z_7345318_A	28A	36A
Z_7345319_G	27G	36G
Z_7345320_A	29A	37A
Z_7345321_G	30G	37G
Z_7345322_T	30T	37T
Z_7345323_A	30A	37A
Z_7345324_G	30G	36G
Z_7345325_A	29A	36A
Z_7345326_C	29C	36C
Z_7345327_T	28T	34T
Z_7345328_T	26T	34T
Z_7345329_C	26C	36C
Z_7345330_G	26G	35G
Z_7345331_G	26G	35G
Z_7345332_T	25T	36T
Z_7345333_C	29C	34C
Z_7345334_T	32T	33T
Z_7345335_A	32A	33A
Z_7345336_A	31A	31A
Z_7345337_A	30A	31A
Z_7345338_T	31T	31T
Z_7345339_T	31G	16T_15G_1C
Z_7345340_G	30G	32G
Z_7345341_G	32G	33G

Z_7345342_C	32C	34C
Z_7345343_A	33A	34A
Z_7345344_G	34G	32G
Z_7345345_G	34G	32G
Z_7345346_A	35A	33A
Z_7345347_T	35T	32T
Z_7345348_A	35A	31A
Z_7345349_T	35T	31T
Z_7345350_A	35A	31A
Z_7345351_A	34A	30A
Z_7345352_G	32G	32G
Z_7345353_T	31T	32T
Z_7345354_T	32T	31T
Z_7345355_A	32A	31A
Z_7345356_G	31G	30G
Z_7345357_T	31T	29T
Z_7345358_T	31T	30T
Z_7345359_A	30A	30A
Z_7345360_G	29G	29G
Z_7345361_T	29T	29T
Z_7345362_T	29T	26T
Z_7345363_A	29A	26A
Z_7345364_G	26G	25G
Z_7345365_T	26T	25T
Z_7345366_T	26T	25T
Z_7345367_A	24A	25A
Z_7345368_G	25G	25G
Z_7345369_T	25T	25T
Z_7345370_T	25T	25T
Z_7345371_A	25A	25A
Z_7345372_G	24G	24G
Z_7345373_T	25T	23T
Z_7345374_T	25T	23T
Z_7345375_A	26A	23A
Z_7345376_G	25G	23G
Z_7345377_T	25T	24T
Z_7345378_T	26T	24T
Z_7345379_A	25A	24A
Z_7345380_G	25G	23G
Z_7345381_T	25T	24T
Z_7345382_T	25T	24T
Z_7345383_A	25A	24A
Z_7345384_G	26G	24G

Z_7345385_T	26T	24T
Z_7345386_T	26T	24T
Z_7345387_A	26A	24A
Z_7345388_G	26G	23G
Z_7345389_T	26T	25T
Z_7345390_T	25T	25T
Z_7345391_A	23A	24A
Z_7345392_G	23G	24G
Z_7345393_T	23T	22T
Z_7345394_T	23T	23T
Z_7345395_A	23A	22A
Z_7345396_G	21G	20G
Z_7345397_T	22T	22T
Z_7345398_T	22T	23T
Z_7345399_A	22A	22A
Z_7345400_G	22G	22G
Z_7345401_T	22T	22T
Z_7345402_T	22T	22T
Z_7345403_A	20A	22A
Z_7345404_G	17G	21G
Z_7345405_T	17T	21T
Z_7345406_T	18T	21T
Z_7345407_A	18A	19A
Z_7345408_G	17G	19G
Z_7345409_T	17T	19T
Z_7345410_T	16T	19T
Z_7345411_A	15A	19A
Z_7345412_G	15G	19G
Z_7345413_T	14T	21T
Z_7345414_T	14T	21T
Z_7345415_A	15A	19A
Z_7345416_G	13G	20G
Z_7345417_T	12T	21T
Z_7345418_T	13T	21T
Z_7345419_A	13A	21A
Z_7345420_G	11G	21G
Z_7345421_T	10T	21T
Z_7345422_T	11T	21T
Z_7345423_A	11A	19A
Z_7345424_G	11G	18G
Z_7345425_T	11T	18T
Z_7345426_T	11T	18T
Z_7345427_A	11A	17A

Z_7345428_G	11G	17G
Z_7345429_T	11T	16T
Z_7345430_T	11T	16T
Z_7345431_A	11A	15A
Z_7345432_G	11G	15G
Z_7345433_T	11T	15T
Z_7345434_T	11T	14T
Z_7345435_A	11A	15A
Z_7345436_G	9G	13G
Z_7345437_A	9A	12A
Z_7345438_T	9T	12T
Z_7345439_A	9A	12A
Z_7345440_G	9G	12G
Z_7345441_A	9A	10A
Z_7345442_T	9T	10T
Z_7345443_A	9A	10A
Z_7345444_G	9G	10G
Z_7345445_A	9A	10A
Z_7345446_T	8T	10T
Z_7345447_A	7A	9A
Z_7345448_G	7G	9G
Z_7345449_A	5A	8A
Z_7345450_T	5T	8T
Z_7345451_A	5A	8A
Z_7345452_G	5G	8G
Z_7345453_A	5A	8A
Z_7345454_T	4T	8T
Z_7345455_A	3A_1G	8A
Z_7345456_G	4G	8G
Z_7345457_A	4A	9A
Z_7345458_T	4T	9T
Z_7345459_A	4A	9A
Z_7345460_G	4G	9G
Z_7345461_A	4A	9A
Z_7345462_T	4T	9T
Z_7345463_A	4A	9A
Z_7345464_G	4G	9G
Z_7345465_A	4A	10A
Z_7345466_T	4T	9T
Z_7345467_A	4A	11A
Z_7345468_G	4G	11G
Z_7345469_A	4A	11A
Z_7345470_T	4T	10T

Z_7345471_A	4A	10A
Z_7345472_G	4G	10G
Z_7345473_A	4A	10A
Z_7345474_T	4T	10T
Z_7345475_A	4A	10A
Z_7345476_G	3G	10G
Z_7345477_A	3A	10A
Z_7345478_T	3T	10T
Z_7345479_A	3A	10A
Z_7345480_G	3G	10G
Z_7345481_A	3A	11A
Z_7345482_T	3T	11T
Z_7345483_A	3A	11A
Z_7345484_G	2G	10G
Z_7345485_A	2A	10A
Z_7345486_T	2T	10T
Z_7345487_A	2A	10A
Z_7345488_G	2G	10G
Z_7345489_A	2A	8A
Z_7345490_T	1T	9T
Z_7345491_A	1A	9A
Z_7345492_G	1G	9G
Z_7345493_A	1A	10A
Z_7345494_T	2T	10T
Z_7345495_A	2A	10A
Z_7345496_G	2G	10G
Z_7345497_A	2A	10A
Z_7345498_T	2T	10T
Z_7345499_A	2A	11A
Z_7345500_G	2G	11G
Z_7345501_A	2A	11A
Z_7345502_T	2T	11T
Z_7345503_A	2A	11A
Z_7345504_G	2G	11G
Z_7345505_A	2A	11A
Z_7345506_T	1T	11T
Z_7345507_A	1A	11A
Z_7345508_G	1G	9G
Z_7345509_A	1A	9A
Z_7345510_T	1T	9T
Z_7345511_A	1A	9A
Z_7345512_G	1G	8G
Z_7345513_A	1A	9A

Z_7345514_T	1T	9T
Z_7345515_A	1A	9A
Z_7345516_G	1G	9G
Z_7345517_A	1A	9A
Z_7345518_T	1T	9T
Z_7345519_A	1A	9A
Z_7345520_G	1G	9G
Z_7345521_A	1A	9A
Z_7345522_T	1T	9T
Z_7345523_A	1A	9A
Z_7345524_G	1G	9G
Z_7345525_A	1A	11A
Z_7345526_T	1T	11T
Z_7345527_A	1A	11A
Z_7345528_G	1G	11G
Z_7345529_A	1A	10A
Z_7345530_T	1T	10T
Z_7345531_A	1A	10A
Z_7345532_G	1G	11G
Z_7345533_A	1A	10A
Z_7345534_T	1T	11T
Z_7345535_A	1A	11A
Z_7345536_G	1G	11G
Z_7345537_A	1A	12A
Z_7345538_T	1T	12T
Z_7345539_A	1A	12A
Z_7345540_G	1G	11G
Z_7345541_A	1A	12A
Z_7345542_T	1T	12T
Z_7345543_A	1A	12A
Z_7345544_G	1G	11G
Z_7345545_A	1A	13A
Z_7345546_T	1T	13T
Z_7345547_A	1A	13A
Z_7345548_G	1A_1G	12G
Z_7345549_A	5A	19A
Z_7345550_T	6T	19T
Z_7345551_A	6A	19A
Z_7345552_A	6A	18A
Z_7345553_A	6A	18A
Z_7345554_T	6T	18T
Z_7345555_A	6A	18A
Z_7345556_A	6A	19A

Z_7345557_A	6A	19A
Z_7345558_T	6T	21T
Z_7345559_A	6A	21A
Z_7345560_A	8A	20A
Z_7345561_A	8A	21A
Z_7345562_T	9T	20T
Z_7345563_A	10A	20A
Z_7345564_A	9A	19A
Z_7345565_A	9A	20A
Z_7345566_T	9T	20T
Z_7345567_A	9A	22A
Z_7345568_A	9A	24A
Z_7345569_A	9A	24A
Z_7345570_T	10T	24T
Z_7345571_A	11A	24A
Z_7345572_A	11A	24A
Z_7345573_A	11A	25A
Z_7345574_T	11T	26T
Z_7345575_A	11A	26A
Z_7345576_A	11A	25A
Z_7345577_A	11A	26A
Z_7345578_T	12T	27T
Z_7345579_A	13A	28A
Z_7345580_A	14A	28A
Z_7345581_A	14A	28A
Z_7345582_T	14T	29T
Z_7345583_A	16A	29A
Z_7345584_A	16A	30A
Z_7345585_A	16A	31A
Z_7345586_T	16T	32T
Z_7345587_A	17A	32A
Z_7345588_G	17G	32G
Z_7345589_G	17G	33G
Z_7345590_G	18G	33G
Z_7345591_T	18T	34T
Z_7345592_A	18A	34A
Z_7345593_C	19T	16T_19C
Z_7345594_G	20G	34G
Z_7345595_A	21A	34A
Z_7345596_T	20T	34T
Z_7345597_C	21C	35C
Z_7345598_C	22C	35C
Z_7345599_A	22A	36A

Z_7345600_G	22G	36G
Z_7345601_A	24A	37A
Z_7345602_T	24T	37T
Z_7345603_C	24C	37C
Z_7345604_A	23A	37A
Z_7345605_A	23A	37A
Z_7345606_G	23G	37G
Z_7345607_C	22C	37C
Z_7345608_C	21C	37C
Z_7345609_A	21A	38A
Z_7345610_G	20G	38G
Z_7345611_G	22G	40G
Z_7345612_G	22G	40G
Z_7345613_A	23A	40A
Z_7345614_A	23A	40A
Z_7345615_C	23C	40C
Z_7345616_A	23A	40A
Z_7345617_G	24G	40G
Z_7345618_A	24A	40A
Z_7345619_T	24T	40T
Z_7345620_G	24G	37G
Z_7345621_T	23T	37T
Z_7345622_T	23T	36T
Z_7345623_G	24G	36G
Z_7345624_T	24T	36T
Z_7345625_C	24C	37C
Z_7345626_T	24T	37T
Z_7345627_T	25T	36T
Z_7345628_G	25G	37G
Z_7345629_G	24G	38G
Z_7345630_A	23A	40A
Z_7345631_A	24A	39A
Z_7345632_G	23G	39G
Z_7345633_A	22A	41A
Z_7345634_G	22G	41G
Z_7345635_A	22A	41A
Z_7345636_C	22C	41C
Z_7345637_G	23G	41G
Z_7345638_C	23C	42C
Z_7345639_A	23A	43A
Z_7345640_G	23G	44G
Z_7345641_A	22A	44A
Z_7345642_C	22C	46C

Z_7345643_A	22A	45A
Z_7345644_A	22A	43A
Z_7345645_G	21G	43G
Z_7345646_C	22C	43C
Z_7345647_A	22A	44A
Z_7345648_A	22A	45A
Z_7345649_C	22C	45C
Z_7345650_T	21T	45T
Z_7345651_G	20G	45G
Z_7345652_T	21T	46T
Z_7345653_G	20G	44G
Z_7345654_G	20G	43G
Z_7345655_G	20G	46G
Z_7345656_A	19A	45A
Z_7345657_G	19G	45G
Z_7345658_G	20G	45G
Z_7345659_G	20G	45G
Z_7345660_A	19A	44A
Z_7345661_G	21G	45G
Z_7345662_A	21A	42A
Z_7345663_G	21G	41G
Z_7345664_G	21G	41G
Z_7345665_A	20A	42A
Z_7345666_G	20G	43G
Z_7345667_G	21G	43G
Z_7345668_A	20A	23A_1T_19G
Z_7345669_T	20T	42T
Z_7345670_G	20G	42G
Z_7345671_T	18T	42T
Z_7345672_T	18T	41T
Z_7345673_C	18C	41C
Z_7345674_C	21C	40C
Z_7345675_T	21T	40T
Z_7345676_T	21T	41T
Z_7345677_G	21G	40G
Z_7345678_C	22C	40C
Z_7345679_A	22A	39A
Z_7345680_A	22A	37A
Z_7345681_A	22A	35A
Z_7345682_C	22C	37C
Z_7345683_C	19T	16T_21C
Z_7345684_A	20A	36A
Z_7345685_C	20T	36C

Z_7345686_G	22G	36G
Z_7345687_T	22T	36T
Z_7345688_C	22C	35C
Z_7345689_A	22A	36A
Z_7345690_T	21T	35T
Z_7345691_G	21G	37G
Z_7345692_C	21C	36C
Z_7345693_C	21C	36C
Z_7345694_T	22T	33T
Z_7345695_C	22C	37C
Z_7345696_G	22G	36G
Z_7345697_G	21G	36G
Z_7345698_G	21G	36G
Z_7345699_C	22C	36C
Z_7345700_A	22A	36A
Z_7345701_G	21G	37G
Z_7345702_A	21A	36A
Z_7345703_C	21C	37C
Z_7345704_C	21C	36C
Z_7345705_C	21C	37C
Z_7345706_A	22A	36A
Z_7345707_T	22T	38T
Z_7345708_C	22C	38C
Z_7345709_G	22G	38G
Z_7345710_C	22C	38C
Z_7345711_A	23A	38A
Z_7345712_G	24G	38G
Z_7345713_T	27T	39T
Z_7345714_G	27G	40G
Z_7345715_G	28G	41G
Z_7345716_G	28G	43G
Z_7345717_G	28G	42G
Z_7345718_C	27C	42C
Z_7345719_A	26A	42A
Z_7345720_G	26G	42G
Z_7345721_A	26A	43A
Z_7345722_G	26G	43G
Z_7345723_G	24G	14A_28G
Z_7345724_A	26A	41A
Z_7345725_G	26G	39G
Z_7345726_A	26A	40A
Z_7345727_A	26A	41A
Z_7345728_G	25G	40G

Z_7345729_A	25A	40A
Z_7345730_G	25G	40G
Z_7345731_G	25G	40G
Z_7345732_G	26G	40G
Z_7345733_G	25G	38G
Z_7345734_A	26A	38A
Z_7345735_A	26A	37A
Z_7345736_G	25G	37G
Z_7345737_C	27C	35C
Z_7345738_C	27C	35C
Z_7345739_A	27A	35A
Z_7345740_A	27A	36A
Z_7345741_A	27A	36A
Z_7345742_G	27G	38G
Z_7345743_A	26A	37A
Z_7345744_A	24A	38A
Z_7345745_C	25C	39C
Z_7345746_C	28C	38C
Z_7345747_A	29A	37A
Z_7345748_G	27G	36G
Z_7345749_A	28A	37A
Z_7345750_A	28A	36A
Z_7345751_G	26G	37G
Z_7345752_C	26C	38C
Z_7345753_C	26C	39C
Z_7345754_C	25C	41C
Z_7345755_C	28C	42C
Z_7345756_A	28A	40A
Z_7345757_C	28C	41C
Z_7345758_G	30G	42G
Z_7345759_T	29T	41T
Z_7345760_C	30C	41C
Z_7345761_C	31A	40C
Z_7345762_A	31A	40A
Z_7345763_G	31G	39G
Z_7345764_C	30C	40C
Z_7345765_A	31A	41A
Z_7345766_A	31A	43A
Z_7345767_C	31T	20T_22C
Z_7345768_G	32G	43G
Z_7345769_C	31C	44C
Z_7345770_A	32A	45A
Z_7345771_G	33G	44G

Z_7345772_C	33C	46C
Z_7345773_T	33T	46T
Z_7345774_G	33G	46G
Z_7345775_C	34C	48C
Z_7345776_A	33A	47A
Z_7345777_G	32G	48G
Z_7345778_C	33C	50C
Z_7345779_A	33A	50A
Z_7345780_G	32G	50G
Z_7345781_A	31A	49A
Z_7345782_G	29G	48G
Z_7345783_A	27A	50A
Z_7345784_G	27G	50G
Z_7345785_C	26C	52C
Z_7345786_T	26T	51T
Z_7345787_T	25T	53T
Z_7345788_T	26T	53T
Z_7345789_T	26T	54T
Z_7345790_C	26C	52C
Z_7345791_C	26C	53C
Z_7345792_C	27C	53C
Z_7345793_C	28C	53C
Z_7345794_C	28C	52C
Z_7345795_G	28G	56G
Z_7345796_G	29G	55G
Z_7345797_C	29C	55C
Z_7345798_T	30T	55T
Z_7345799_G	30G	55G
Z_7345800_C	30C	55C
Z_7345801_C	30C	54C
Z_7345802_A	29A	53A
Z_7345803_A	29A	52A
Z_7345804_A	29A	52A
Z_7345805_A	27A	52A
Z_7345806_A	27A	52A
Z_7345807_T	27T	52T
Z_7345808_A	25A	52A
Z_7345809_C	25C	50C
Z_7345810_T	26T	50T
Z_7345811_C	26C	49C
Z_7345812_T	27T	48T
Z_7345813_G	27G	50G
Z_7345814_G	27G	50G

Z_7345815_C	26C	49C
Z_7345816_A	27A	49A
Z_7345817_A	26A	48A
Z_7345818_G	24G	48G
Z_7345819_G	23G	48G
Z_7345820_A	25A	48A
Z_7345821_A	26A	49A
Z_7345822_T	24T	48T
Z_7345823_C	26C	48C
Z_7345824_A	27A	48A
Z_7345825_G	27G	48G
Z_7345826_G	24G	49G
Z_7345827_A	23A	51A
Z_7345828_A	23A	52A
Z_7345829_G	22G	52G
Z_7345830_A	22A	51A
Z_7345831_G	22G	52G
Z_7345832_A	22A	53A
Z_7345833_G	22G	53G
Z_7345834_G	22G	53G
Z_7345835_A	22A	52A
Z_7345836_G	22G	52G
Z_7345837_A	22A	51A
Z_7345838_G	22G	50G
Z_7345839_G	20G	50G
Z_7345840_T	23T	49T_31G_1C
Z_7345841_G	22G	51G
Z_7345842_G	21G	52G
Z_7345843_G	21G	52G
Z_7345844_G	21G	52G
Z_7345845_G	23G	52G
Z_7345846_G	22G	50G
Z_7345847_T	23T	48T
Z_7345848_C	22C	49C
Z_7345849_A	22A	49A
Z_7345850_T	23T	50T
Z_7345851_G	22G	50G
Z_7345852_C	24C	50C
Z_7345853_T	24T	49T
Z_7345854_G	24G	49G
Z_7345855_A	26A	48A
Z_7345856_C	26C	49C
Z_7345857_C	26C	49C

Z_7345858_C	27C	48C
Z_7345859_A	29A	48A
Z_7345860_G	29G	48G
Z_7345861_A	29A	47A
Z_7345862_T	28T	48T
Z_7345863_C	29C	46C
Z_7345864_A	28A	46A
Z_7345865_A	28A	46A
Z_7345866_A	27A	47A
Z_7345867_A	28A	45A
Z_7345868_C	26C	45C
Z_7345869_C	29C	45C
Z_7345870_C	29C	46C
Z_7345871_G	30G	46G
Z_7345872_A	29A	43A
Z_7345873_C	29C	43C
Z_7345874_G	29G	46G
Z_7345875_A	29A	46A
Z_7345876_A	29A	49A
Z_7345877_A	30A	49A
Z_7345878_G	30G	47G
Z_7345879_G	31G	46G
Z_7345880_C	32C	47C
Z_7345881_A	32A	47A
Z_7345882_G	32G	45G
Z_7345883_G	30G	46G
Z_7345884_A	30A	45A
Z_7345885_T	32T	48T
Z_7345886_G	31G	49G
Z_7345887_A	30A	50A
Z_7345888_G	31G	47G
Z_7345889_C	31C	48C
Z_7345890_G	31G	48G
Z_7345891_C	30C	48C
Z_7345892_T	29T	45T
Z_7345893_C	30C	49C
Z_7345894_A	29A	48A
Z_7345895_G	29G	49G
Z_7345896_C	29C	49C
Z_7345897_G	29G	49G
Z_7345898_G	29G	50G
Z_7345899_T	27T	50T
Z_7345900_C	29C	51C

Z_7345901_C	30C	51C
Z_7345902_A	30A	52A
Z_7345903_C	30C	54C
Z_7345904_G	30G	54G
Z_7345905_T	30T	52T
Z_7345906_G	30G	1A_27G_24C
Z_7345907_A	31A	54A
Z_7345908_A	31A	53A
Z_7345909_G	29G	54G
Z_7345910_A	27A	54A
Z_7345911_G	31G	55G
Z_7345912_T	31T	55T
Z_7345913_C	31C	57C
Z_7345914_C	32C	57C
Z_7345915_C	30C	57C
Z_7345916_A	30A	55A
Z_7345917_G	30G	55G
Z_7345918_A	29A	56A
Z_7345919_G	31G	56G
Z_7345920_G	30G	54G
Z_7345921_G	30G	53G
Z_7345922_A	29A	53A
Z_7345923_A	29A	53A
Z_7345924_G	29G	56G
Z_7345925_A	27A	58A
Z_7345926_A	28A	59A
Z_7345927_A	28A	59A
Z_7345928_G	27G	60G
Z_7345929_A	26A	63A
Z_7345930_G	25G	64G
Z_7345931_G	25G	64G
Z_7345932_C	25C	66C
Z_7345933_A	24A	68A
Z_7345934_A	24A	68A
Z_7345935_G	24G	66G
Z_7345936_G	24G	69G
Z_7345937_G	19A_24G	22A_68G
Z_7345938_A	4A_1G	46A
Z_7345939_G	23G	69G
Z_7345940_A	23A	71A
Z_7345941_G	22G	71G
Z_7345942_A	23A	69A
Z_7345943_G	22G	69G

Z_7345944_A	22A	69A
Z_7345945_A	23A	68A
Z_7345946_G	23G	70G
Z_7345947_C	25C	70C
Z_7345948_C	25C	70C
Z_7345949_A	27A	70A
Z_7345950_C	26C	70C
Z_7345951_G	26G	72G
Z_7345952_G	24G	72G
Z_7345953_A	24A	72A
Z_7345954_A	24A	70A
Z_7345955_A	24A	70A
Z_7345956_C	24C	71C
Z_7345957_C	20C	72C
Z_7345958_C	23C	72C
Z_7345959_A	24A	71A
Z_7345960_G	23G	70G
Z_7345961_T	23T	70T
Z_7345962_G	24G	70G
Z_7345963_G	23G	70G
Z_7345964_G	26G	71G
Z_7345965_G	27G	69G
Z_7345966_T	28T	68T
Z_7345967_G	29G	68G
Z_7345968_G	29G	68G
Z_7345969_C	30C	65C
Z_7345970_C	30C	67C
Z_7345971_C	29C	63C
Z_7345972_C	30C	64C
Z_7345973_A	30A	63A
Z_7345974_C	29C	63C
Z_7345975_G	29G	65G
Z_7345976_G	28G	66G
Z_7345977_C	29C	65C
Z_7345978_T	25G	46T_20G
Z_7345979_G	25A	66G
Z_7345980_A	27A	63A
Z_7345981_G	27G	62G
Z_7345982_G	27G	63G
Z_7345983_G	27G	64G
Z_7345984_A	27A	62A
Z_7345985_G	31G	63G
Z_7345986_G	30G	64G

Z_7345987_T	31T	63T
Z_7345988_G	34G	64G
Z_7345989_A	33A	64A
Z_7345990_G	33G	64G
Z_7345991_C	31C	64C
Z_7345992_T	32T	64T
Z_7345993_G	32G	63G
Z_7345994_G	31G	64G
Z_7345995_A	30G	43A_21G
Z_7345996_C	28T	66C
Z_7345997_C	33C	65C
Z_7345998_A	33A	66A
Z_7345999_T	34T	66T
Z_7346000_T	35T	67T
Z_7346001_C	35C	66C
Z_7346002_C	35C	67C
Z_7346003_C	35C	68C
Z_7346004_C	38C	69C
Z_7346005_A	39A	68A
Z_7346006_A	40A	68A
Z_7346007_A	38A	67A
Z_7346008_G	40G	66G
Z_7346009_A	37A	66A
Z_7346010_G	39G	66G
Z_7346011_G	40G	67G
Z_7346012_T	39T	66T
Z_7346013_G	39G	66G
Z_7346014_G	39G	68G
Z_7346015_A	36A	68A
Z_7346016_A	37G	40A_28G_1C
Z_7346017_T	36T	69T
Z_7346018_G	34G	69G
Z_7346019_G	35G	66G
Z_7346020_A	35A	68A
Z_7346021_T	35T	68T
Z_7346022_G	34G	67G
Z_7346023_T	33T	67T
Z_7346024_C	34C	69C
Z_7346025_G	34G	68G
Z_7346026_C	33C	65C
Z_7346027_A	34A	64A
Z_7346028_G	34G	63G
Z_7346029_C	31C	62C

Z_7346030_C	33C	62C
Z_7346031_C	32C	61C
Z_7346032_C	33C	64C
Z_7346033_A	32A	64A
Z_7346034_T	31T	64T
Z_7346035_A	30A	62A
Z_7346036_G	29G	62G
Z_7346037_A	32A	63A
Z_7346038_A	31A	62A
Z_7346039_G	30G	62G
Z_7346040_G	30G	63G
Z_7346041_G	30G	62G
Z_7346042_A	30A	65A
Z_7346043_G	29G	64G
Z_7346044_A	29A	66A
Z_7346045_T	33T	67T
Z_7346046_G	32G	66G
Z_7346047_C	32C	66C
Z_7346048_C	33C	67C
Z_7346049_C	33C	68C
Z_7346050_T	35T	67T
Z_7346051_C	36C	67C
Z_7346052_A	36A	66A
Z_7346053_C	36C	67C
Z_7346054_T	36T	67T
Z_7346055_C	31C	70C
Z_7346056_T	32T	70T
Z_7346057_G	31G	70G
Z_7346058_G	29G	71G
Z_7346059_A	28A	71A
Z_7346060_C	29C	71C
Z_7346061_A	31A	71A
Z_7346062_G	30G	71G
Z_7346063_A	28A	71A
Z_7346064_G	28G	70G
Z_7346065_C	30C	71C
Z_7346066_A	30A	71A
Z_7346067_A	30A	70A
Z_7346068_G	32G	70G
Z_7346069_G	32G	71G
Z_7346070_C	33C	70C
Z_7346071_C	34C	69C
Z_7346072_G	34G	70G

Z_7346073_T	34T	69T
Z_7346074_G	32G	68G
Z_7346075_G	30G	68G
Z_7346076_G	30G	68G
Z_7346077_G	30G	69G
Z_7346078_C	30C	73C
Z_7346079_T	30T	75T
Z_7346080_C	32C	74C
Z_7346081_T	32T	74T
Z_7346082_T	33T	75T
Z_7346083_C	33C	75C
Z_7346084_T	33T	76T
Z_7346085_G	32G	76G
Z_7346086_C	34C	77C
Z_7346087_C	34C	77C
Z_7346088_A	37A	76A
Z_7346089_C	37C	75C
Z_7346090_C	37C	74C
Z_7346091_C	36C	72C
Z_7346092_C	39C	73C
Z_7346093_T	41T	74T
Z_7346094_G	40G	74G
Z_7346095_G	41G	72G
Z_7346096_G	40A	72G
Z_7346097_C	40C	71C
Z_7346098_A	41A	71A
Z_7346099_A	41A	69A
Z_7346100_G	41G	69G
Z_7346101_C	41C	69C
Z_7346102_A	42A	69A
Z_7346103_G	41G	33A_36G
Z_7346104_G	43G	69G
Z_7346105_G	40G	71G
Z_7346106_G	42G	70G
Z_7346107_A	38A	71A
Z_7346108_T	38T	70T
Z_7346109_C	39C	68C
Z_7346110_C	39C	68C
Z_7346111_T	38T	68T
Z_7346112_C	38C	68C
Z_7346113_A	39A	67A
Z_7346114_G	38G	67G
Z_7346115_T	35T	33T_34C

Z_7346116_G	36G	67G
Z_7346117_G	34G	68G
Z_7346118_G	34G	67G
Z_7346119_G	35G	64G
Z_7346120_A	34A	64A
Z_7346121_A	32A	63A
Z_7346122_C	33C	64C
Z_7346123_C	34C	64C
Z_7346124_C	34C	65C
Z_7346125_G	36G	64G
Z_7346126_T	35T	62T
Z_7346127_C	36C	61C
Z_7346128_A	34G	32A_29G
Z_7346129_A	35A	62A
Z_7346130_G	36G	64G
Z_7346131_A	35A	64A
Z_7346132_C	35C	64C
Z_7346133_A	36G	63A
Z_7346134_G	36G	62G
Z_7346135_A	35A	61A_25C
Z_7346136_C	36C	65C
Z_7346137_A	36A	61A
Z_7346138_C	34C	62C
Z_7346139_A	33A	62A
Z_7346140_C	34C	62C
Z_7346141_A	34A	61A
Z_7346142_A	33A	60A
Z_7346143_G	33G	61G
Z_7346144_G	33G	60G
Z_7346145_G	35G	60G
Z_7346146_G	34G	60G
Z_7346147_C	35C	60C
Z_7346148_G	33G	60G
Z_7346149_A	33A	59A
Z_7346150_T	31T	57T
Z_7346151_G	31G	56G
Z_7346152_G	29G	55G
Z_7346153_A	31G	29A_27G
Z_7346154_A	31A	55A
Z_7346155_A	31A	54A
Z_7346156_A	31A	54A
Z_7346157_A	31A	54A
Z_7346158_C	30C	55C

Z_7346159_G	28G	57G
Z_7346160_C	30C	57C
Z_7346161_T	29T	56T
Z_7346162_C	28C	57C
Z_7346163_T	26T	56T
Z_7346164_C	27C	55C
Z_7346165_C	28C	56C
Z_7346166_A	28A	54A
Z_7346167_C	28C	57C
Z_7346168_A	27A	58A
Z_7346169_T	26T	57T
Z_7346170_G	26G	57G
Z_7346171_C	26C	58C
Z_7346172_T	25T	54T
Z_7346173_C	25C	52C
Z_7346174_A	24A	53A
Z_7346175_G	24G	52G
Z_7346176_A	24A	51A
Z_7346177_A	24A	53A
Z_7346178_A	24A	53A
Z_7346179_T	23T	55T
Z_7346180_A	24A	56A
Z_7346181_G	25G	57G
Z_7346182_T	24T	58T
Z_7346183_G	25G	58G
Z_7346184_T	22T	57T
Z_7346185_C	25C	57C
Z_7346186_C	25C	56C
Z_7346187_C	25C	55C
Z_7346188_A	25C	30A_24C
Z_7346189_T	23T	55T
Z_7346190_T	22T	56T
Z_7346191_C	22C	56C
Z_7346192_G	21G	58G
Z_7346193_T	21T	57T
Z_7346194_T	20T	58T
Z_7346195_A	19A	58A
Z_7346196_T	19T	58T
Z_7346197_T	19T	58T
Z_7346198_G	20G	60G
Z_7346199_C	20C	58C
Z_7346200_T	22T	60T
Z_7346201_G	21G	61G

Z_7346202_A	23A	59A
Z_7346203_A	22A	63A
Z_7346204_T	22T	64T
Z_7346205_G	23G	64G
Z_7346206_T	21C	1A_36T_26C
Z_7346207_C	21C	64C
Z_7346208_A	21A	64A
Z_7346209_C	20C	63C
Z_7346210_T	20G	37T_28G
Z_7346211_G	20G	63G
Z_7346212_A	22A	62A
Z_7346213_G	24G	62G
Z_7346214_C	24C	63C
Z_7346215_A	23C	36A_26C
Z_7346216_A	27A	65A
Z_7346217_A	27A	64A
Z_7346218_A	28A	64A
Z_7346219_A	28A	64A
Z_7346220_A	28A	67A
Z_7346221_G	27G	67G
Z_7346222_A	26A	68A
Z_7346223_G	28G	68G
Z_7346224_G	28G	66G
Z_7346225_T	27T	66T
Z_7346226_G	28G	65G
Z_7346227_T	30C	39T_27C
Z_7346228_T	31T	64T
Z_7346229_A	32A	64A
Z_7346230_C	30C	64C
Z_7346231_G	31G	63G
Z_7346232_A	31A	66A
Z_7346233_A	30A	65A
Z_7346234_A	31A	65A
Z_7346235_A	30A	65A
Z_7346236_T	31T	65T
Z_7346237_G	31G	65G
Z_7346238_T	30T	66T
Z_7346239_T	33T	67T
Z_7346240_C	33C	67C
Z_7346241_C	35C	67C
Z_7346242_T	35T	68T
Z_7346243_G	35G	69G
Z_7346244_G	36G	68G

Z_7346245_A	35A	70A
Z_7346246_T	36T	71T
Z_7346247_T	38T	72T
Z_7346248_C	38C	73C
Z_7346249_A	38A	73A
Z_7346250_A	37A	73A
Z_7346251_C	36C	73C
Z_7346252_C	36C	73C
Z_7346253_A	36A	72A
Z_7346254_A	37A	69A
Z_7346255_C	39C	71C
Z_7346256_C	38C	71C
Z_7346257_T	39T	72T
Z_7346258_T	39T	72T
Z_7346259_G	39G	72G
Z_7346260_C	40C	71C
Z_7346261_A	40A	71A
Z_7346262_A	39A	68A
Z_7346263_A	40A	67A
Z_7346264_C	40C	66C
Z_7346265_A	39A	66A
Z_7346266_G	40G	65G
Z_7346267_A	40A	66A
Z_7346268_C	38C	66C
Z_7346269_C	37C	63C
Z_7346270_T	35T	63T
Z_7346271_C	33C	62C
Z_7346272_A	32A	60A
Z_7346273_T	33T	59T
Z_7346274_G	33G	58G
Z_7346275_T	31T	58T
Z_7346276_G	31G	57G
Z_7346277_T	30T	56T
Z_7346278_C	30C	57C
Z_7346279_T	31T	57T
Z_7346280_G	30G	59G
Z_7346281_T	31T	93T_11G_33C
Z_7346282_C	30C	51C
Z_7346283_T	28T	51T
Z_7346284_C	1G_4C	36C
Z_7346285_T	5T	37T
Z_7346286_G	4G	37G
Z_7346287_T	4T	35T

Z_7346288_C	4C	34C
Z_7346289_T	4T	44T
Z_7346290_C	4C	44C
Z_7346291_T	4T	44T
Z_7346292_G	7G	45G
Z_7346293_T	7T	44T
Z_7346294_G	7G	44G
Z_7346295_T	7T	42T
Z_7346296_G	7G	42G
Z_7346297_T	7T	42T
Z_7346298_G	7G	41G
Z_7346299_T	7T	43T
Z_7346300_G	7G	11A_31G_1C
Z_7346301_T	7T	32T_11C
Z_7346302_G	7G	42G
Z_7346303_T	7T	42T
Z_7346304_G	7G	42G
Z_7346305_T	7T	39T
Z_7346306_G	7G	40G
Z_7346307_T	7T	40T
Z_7346308_G	6G	40G
Z_7346309_T	6T	40T
Z_7346310_G	6G	40G
Z_7346311_T	6T	29T_11C
Z_7346312_G	6G	40G
Z_7346313_T	6T	40T
Z_7346314_G	6G	39G
Z_7346315_T	6T	37T
Z_7346316_G	6G	37G
Z_7346317_T	6T	25T_10C
Z_7346318_G	6G	35G
Z_7346319_T	5T	36T
Z_7346320_G	5G	36G
Z_7346321_T	5T	36T
Z_7346322_G	5G	36G
Z_7346323_T	5T	26T_9C
Z_7346324_G	5G	37G
Z_7346325_T	5T	37T
Z_7346326_G	5G	36G
Z_7346327_T	5T	35T
Z_7346328_G	5G	35G
Z_7346329_T	5T	29T
Z_7346330_G	5G	36G

Z_7346331_T	5T	37T
Z_7346332_G	5G	35G
Z_7346333_T	4T	35T
Z_7346334_G	4G	35G
Z_7346335_T	4T	35T
Z_7346336_G	4G	37G
Z_7346337_T	4T	36T
Z_7346338_G	4G	36G
Z_7346339_T	3T	34T
Z_7346340_G	3G	34G
Z_7346341_T	2T	31T
Z_7346342_G	2G	30G
Z_7346343_T	4T	26T
Z_7346344_G	4G	28G
Z_7346345_T	4T	27T
Z_7346346_G	4G	28G
Z_7346347_T	4T	26T
Z_7346348_G	3G	28G
Z_7346349_T	3T	25T
Z_7346350_G	3G	27G
Z_7346351_T	3T	26T
Z_7346352_G	3G	26G
Z_7346353_T	3T	25T
Z_7346354_G	4G	28G
Z_7346355_T	4T	25T
Z_7346356_G	9G	29G
Z_7346357_T	10T	27T
Z_7346358_G	9G	28G
Z_7346359_T	10T	26T
Z_7346360_G	10G	28G
Z_7346361_T	11T	25T
Z_7346362_G	11G	29G
Z_7346363_T	12T	28T
Z_7346364_G	14G	31G
Z_7346365_T	14T	31T
Z_7346366_G	14G	32G
Z_7346367_T	14T	32T
Z_7346368_G	16G	31G
Z_7346369_T	18T	28T
Z_7346370_G	18G	30G
Z_7346371_T	19T	30T
Z_7346372_G	19G	29G
Z_7346373_T	19T	29T

Z_7346374_G	21G	30G
Z_7346375_T	21T	27T
Z_7346376_G	21G	27G
Z_7346377_T	21T	27T
Z_7346378_G	22G	26G
Z_7346379_T	22T	27T
Z_7346380_G	22G	28G
Z_7346381_T	23T	27T
Z_7346382_G	23G	28G
Z_7346383_T	23T	28T
Z_7346384_G	23G	28G
Z_7346385_T	23T	28T
Z_7346386_G	23G	29G
Z_7346387_T	24T	28T
Z_7346388_G	25G	28G
Z_7346389_T	25T	29T
Z_7346390_G	25G	29G
Z_7346391_T	25T	29T
Z_7346392_G	25G	29G
Z_7346393_T	25T	29T
Z_7346394_G	26G	28G
Z_7346395_T	27T	28T
Z_7346396_G	27G	28G
Z_7346397_T	27T	28T
Z_7346398_G	27G	28G
Z_7346399_T	27T	28T
Z_7346400_G	27G	28G
Z_7346401_T	28T	28T
Z_7346402_G	28G	29G
Z_7346403_T	28T	29T
Z_7346404_G	28G	29G
Z_7346405_T	28T	29T
Z_7346406_G	28G	29G
Z_7346407_T	29T	29T
Z_7346408_G	29G	29G
Z_7346409_T	29T	30T
Z_7346410_G	29G	30G
Z_7346411_T	30T	30T
Z_7346412_G	30G	32G
Z_7346413_T	24T	31T
Z_7346414_C	22C	33C
Z_7346415_C	22C	33C
Z_7346416_A	4A_12C	32A

Z_7346417_C	12T_3C	8T_24C
Z_7346418_G	17G	33G
Z_7346419_C	16C	29C
Z_7346420_A	15A	29A
Z_7346421_T	14T	30T
Z_7346422_C	14C	30C
Z_7346423_T	15T	29T
Z_7346424_C	16C	30C
Z_7346425_C	16C	30C
Z_7346426_T	16T	29T
Z_7346427_T	18T	28T
Z_7346428_C	17C	29C
Z_7346429_G	18G	30G
Z_7346430_G	19G	30G
Z_7346431_A	18A	30A
Z_7346432_C	18C	32C
Z_7346433_A	18A	32A
Z_7346434_A	19A	33A
Z_7346435_G	19G	33G
Z_7346436_C	19C	33C
Z_7346437_A	19A	33A
Z_7346438_A	18A	34A
Z_7346439_T	19T	34T
Z_7346440_T	19T	34T
Z_7346441_T	19T	33T
Z_7346442_A	20A	33A
Z_7346443_C	20C	34C
Z_7346444_A	21A	35A
Z_7346445_G	22G	35G
Z_7346446_C	22C	34C
Z_7346447_C	22C	35C
Z_7346448_G	23G	34G
Z_7346449_C	23C	33C
Z_7346450_C	22C	32C
Z_7346451_A	23A	32A
Z_7346452_G	25G	34G
Z_7346453_G	25G	34G
Z_7346454_A	25A	33A
Z_7346455_G	26G	34G
Z_7346456_C	26C	33C
Z_7346457_T	26T	34T
Z_7346458_T	25T	34T
Z_7346459_C	28C	33C

Z_7346460_C	30C	33C
Z_7346461_A	32A	33A
Z_7346462_T	32T	33T
Z_7346463_T	32T	33T
Z_7346464_T	30T	33T
Z_7346465_G	30G	31G
Z_7346466_C	29C	31C
Z_7346467_T	30T	31T
Z_7346468_G	31G	31G
Z_7346469_G	31G	30G
Z_7346470_G	31G	30G
Z_7346471_G	31G	30G
Z_7346472_G	31G	31G
Z_7346473_C	31C	31C
Z_7346474_G	31G	32G
Z_7346475_A	31A	31A
Z_7346476_A	34A	31A
Z_7346477_A	33A	31A
Z_7346478_G	32G	31G
Z_7346479_T	33T	31T
Z_7346480_C	33C	31C
Z_7346481_A	33A	30A
Z_7346482_C	33C	30C
Z_7346483_T	31T	30T
Z_7346484_C	33C	30C
Z_7346485_G	33G	30G
Z_7346486_T	33T	31T
Z_7346487_A	33A	31A
Z_7346488_A	30A	31A
Z_7346489_T	31T	30T
Z_7346490_A	31A	30A
Z_7346491_G	30G	32G
Z_7346492_A	50A_1G_40C	31A
Z_7346493_C	33C	32C
Z_7346494_C	27C	32C
Z_7346495_C	34C	33C
Z_7346496_C	34C	34C
Z_7346497_C	33C	34C
Z_7346498_C	34C	34C
Z_7346499_C	34C	34C
Z_7346500_C	34C	34C
Z_7346501_C	34C	33C
Z_7346502_A	28A	31A

Z_7346503_C	30C	31C
Z_7346504_C	29C	30C
Z_7346505_T	30T	30T
Z_7346506_G	26G	30G
Z_7346507_C	28C	29C
Z_7346508_C	27C	28C
Z_7346509_A	26A	26A
Z_7346510_G	28G	28G
Z_7346511_A	28A	27A
Z_7346512_C	27C	27C
Z_7346513_C	28C	30C
Z_7346514_G	24G	31G
Z_7346515_C	27C	31C
Z_7346516_A	27A	31A
Z_7346517_G	28G	30G
Z_7346518_C	27C	29C
Z_7346519_C	26C	30C
Z_7346520_C	26C	31C
Z_7346521_C	26C	31C
Z_7346522_A	24A	32A
Z_7346523_C	25C	34C
Z_7346524_A	25A	34A
Z_7346525_C	25C	33C
Z_7346526_G	22G	21G_11C
Z_7346527_C	23C	33C
Z_7346528_A	20A	33A
Z_7346529_C	21C	32C
Z_7346530_C	21C	32C
Z_7346531_T	21T	32T
Z_7346532_G	22G	33G
Z_7346533_C	22C	32C
Z_7346534_T	21T	32T
Z_7346535_T	22T	32T
Z_7346536_G	23G	33G
Z_7346537_G	25G	33G
Z_7346538_A	23A	34A
Z_7346539_T	23T	33T
Z_7346540_A	24A	33A
Z_7346541_T	24T	33T
Z_7346542_A	23A	34A
Z_7346543_C	23C	34C
Z_7346544_G	24G	34G
Z_7346545_G	24G	35G

Z_7346546_A	24A	35A
Z_7346547_G	24G	34G
Z_7346548_T	23T	34T
Z_7346549_T	23T	34T
Z_7346550_T	23T	34T
Z_7346551_A	22A	35A
Z_7346552_T	23T	36T
Z_7346553_T	24T	36T
Z_7346554_A	25A	35A
Z_7346555_A	26A	35A
Z_7346556_A	26A	35A
Z_7346557_T	27T	36T
Z_7346558_T	27T	37T
Z_7346559_A	28A	37A
Z_7346560_T	28T	37T
Z_7346561_G	28G	37G
Z_7346562_T	28T	37T
Z_7346563_T	29T	38T
Z_7346564_T	28T	38T
Z_7346565_T	29T	40T
Z_7346566_T	29T	40T
Z_7346567_T	30T	39T
Z_7346568_A	30A	41A
Z_7346569_A	31A	40A
Z_7346570_A	30A	39A
Z_7346571_G	30G	40G
Z_7346572_G	31G	40G
Z_7346573_C	31C	40C
Z_7346574_T	31T	40T
Z_7346575_G	30G	40G
Z_7346576_C	29C	41C
Z_7346577_A	31A	41A
Z_7346578_C	29C	41C
Z_7346579_T	31T	41T
Z_7346580_T	30T	40T
Z_7346581_G	29G	41G
Z_7346582_G	28G	40G
Z_7346583_A	30A	42A
Z_7346584_A	31A	44A
Z_7346585_A	31A	45A
Z_7346586_G	31G	44G
Z_7346587_G	31G	45G
Z_7346588_G	32G	44G

Z_7346589_C	31C	45C
Z_7346590_A	31A	44A
Z_7346591_G	32G	42G
Z_7346592_A	32A	42A
Z_7346593_A	32A	45A
Z_7346594_A	32A	46A
Z_7346595_T	33T	46T
Z_7346596_A	34A	47A
Z_7346597_G	35G	47G
Z_7346598_G	35G	47G
Z_7346599_C	35C	47C
Z_7346600_C	36C	48C
Z_7346601_A	35A	48A
Z_7346602_G	35G	48G
Z_7346603_A	35A	50A
Z_7346604_G	36G	51G
Z_7346605_T	35T	49T
Z_7346606_T	36T	51T
Z_7346607_T	34T	50T
Z_7346608_A	34A	48A
Z_7346609_A	34A	47A
Z_7346610_G	33G	47G
Z_7346611_G	33G	47G
Z_7346612_G	33G	47G
Z_7346613_C	31C	49C
Z_7346614_G	32G	49G
Z_7346615_A	33A	48A
Z_7346616_A	33A	48A
Z_7346617_A	33A	47A
Z_7346618_T	33T	45T
Z_7346619_C	32C	45C
Z_7346620_C	33C	46C
Z_7346621_C	33C	45C
Z_7346622_A	32A	44A
Z_7346623_G	31G	44G
Z_7346624_G	31G	45G
Z_7346625_G	30G	45G
Z_7346626_C	30C	45C
Z_7346627_A	29A	45A
Z_7346628_A	29A	45A
Z_7346629_T	27T	45T
Z_7346630_C	27C	46C
Z_7346631_T	27T	46T

Z_7346632_C	25C	46C
Z_7346633_T	25T	46T
Z_7346634_T	25T	45T
Z_7346635_C	25C	46C
Z_7346636_C	25C	46C
Z_7346637_A	25A	44A
Z_7346638_C	24C	45C
Z_7346639_G	23G	45G
Z_7346640_T	23T	44T
Z_7346641_T	22T	46T
Z_7346642_G	22G	46G
Z_7346643_G	22G	46G
Z_7346644_C	22C	46C
Z_7346645_T	22T	46T
Z_7346646_G	22G	45G
Z_7346647_C	23C	44C
Z_7346648_C	24C	43C
Z_7346649_A	23A	44A
Z_7346650_G	23G	44G
Z_7346651_A	22A	44A
Z_7346652_C	23C	43C
Z_7346653_T	22T	42T
Z_7346654_C	21C	43C
Z_7346655_T	20T	43T
Z_7346656_T	20C	24T_20C
Z_7346657_C	20C	43C
Z_7346658_A	19A	42A
Z_7346659_G	19G_15C	43G
Z_7346660_C	24C	19T_22C
Z_7346661_C	23C	41C
Z_7346662_C	23C	41C
Z_7346663_C	23C	40C
Z_7346664_T	19T	40T
Z_7346665_G	19G	40G
Z_7346666_C	19C	40C
Z_7346667_A	21A	40A
Z_7346668_A	20A	40A
Z_7346669_A	21A	39A
Z_7346670_T	21T	39T
Z_7346671_C	20C	40C
Z_7346672_T	21T	41T
Z_7346673_T	20T	41T
Z_7346674_C	19C	41C

Z_7346675_T	18T	41T
Z_7346676_C	18C	40C
Z_7346677_C	17C	41C
Z_7346678_C	17C	40C
Z_7346679_A	19A	37A
Z_7346680_T	18T	37T
Z_7346681_C	19C	37C
Z_7346682_A	19A	36A
Z_7346683_G	17G	37G
Z_7346684_C	20C	36C
Z_7346685_C	20C	36C
Z_7346686_T	20T	36T
Z_7346687_T	20T	36T
Z_7346688_G	21G	33G
Z_7346689_A	22A	33A
Z_7346690_C	21C	34C
Z_7346691_A	22A	32A
Z_7346692_C	22C	34C
Z_7346693_T	23T	34T
Z_7346694_T	24T	35T
Z_7346695_C	24C	34C
Z_7346696_T	26T	34T
Z_7346697_T	25T	34T
Z_7346698_A	25A	32A
Z_7346699_T	25T	30T
Z_7346700_T	25T	33T
Z_7346701_T	25T	31T
Z_7346702_C	25C	31C
Z_7346703_A	25A	31A
Z_7346704_G	24G	31G
Z_7346705_C	23C	31C
Z_7346706_T	24T	31T
Z_7346707_C	24C	31C
Z_7346708_A	25A	29A
Z_7346709_C	25C	28C
Z_7346710_T	25T	28T
Z_7346711_T	25T	28T
Z_7346712_A	25A	27A
Z_7346713_G	26G	28G
Z_7346714_A	26A	28A
Z_7346715_G	26G	28G
Z_7346716_A	24A	28A
Z_7346717_G	23G	27G

Z_7346718_G	23G	27G
Z_7346719_G	24G	26G
Z_7346720_T	24T	26T
Z_7346721_A	25A	26A
Z_7346722_T	24T	25T
Z_7346723_T	24T	25T
Z_7346724_T	23T	25T
Z_7346725_A	23A	25A
Z_7346726_T	23T	24T
Z_7346727_G	22G	24G
Z_7346728_G	21G	23G
Z_7346729_G	20G	23G
Z_7346730_T	20T	22T
Z_7346731_G	19G	22G
Z_7346732_T	20T	22T
Z_7346733_C	19C	21C
Z_7346734_A	21A	20A
Z_7346735_T	21T	22T
Z_7346736_C	21C	20C
Z_7346737_T	21T	19T
Z_7346738_C	21C	19C
Z_7346739_T	19T	19T
Z_7346740_G	21G	19G
Z_7346741_A	20A	19A
Z_7346742_C	21C	18C
Z_7346743_C	21C	20C
Z_7346744_C	21C	19C
Z_7346745_T	21T	19T
Z_7346746_T	21T	19T
Z_7346747_C	19C	19C
Z_7346748_C	22C	19C
Z_7346749_T	21T	18T
Z_7346750_C	21C	18C
Z_7346751_T	21T	17T
Z_7346752_C	21C	17C
Z_7346753_C	20C	16C
Z_7346754_A	18A	16A
Z_7346755_C	20C	16C
Z_7346756_C	20C	16C
Z_7346757_T	20T	16T
Z_7346758_C	18C	15C
Z_7346759_G	18G	12G_3C
Z_7346760_G	18G	15G

Z_7346761_A	18A	14A
Z_7346762_A	17A	14A
Z_7346763_C	16C	14C
Z_7346764_G	14G	3A_11G
Z_7346765_C	14C	15C
Z_7346766_T	12T	14T
Z_7346767_T	12T	13T
Z_7346768_G	13G	13G
Z_7346769_G	13G	13G
Z_7346770_C	13C	13C
Z_7346771_C	13C	14C
Z_7346772_T	13T	13T
Z_7346773_G	13G	13G
Z_7346774_C	13C	13C
Z_7346775_T	13T	13T
Z_7346776_T	12T	14T
Z_7346777_C	12C	6A_3T_11C
Z_7346778_A	11A	6A_2C
Z_7346779_C	19C	6T_8C
Z_7346780_C	3C	15C
Z_7346781_C	11C	15C
Z_7346782_C	11C	15C
Z_7346783_C	10C	17C
Z_7346784_C	10C	16C
Z_7346785_C	10C	15C
Z_7346786_C	8C	15C
Z_7346787_C	8C	15C
Z_7346788_T	8T	15T
Z_7346789_T	9T	14T
Z_7346790_T	9T	14T
Z_7346791_T	9T	13T
Z_7346792_T	9T	14T
Z_7346793_T	9T	14T
Z_7346794_T	9T	16T
Z_7346795_T	9T	16T
Z_7346796_G	9G	16G
Z_7346797_C	9C	14C
Z_7346798_C	9C	14C
Z_7346799_A	9A	14A
Z_7346800_C	9C	14C
Z_7346801_A	9A	14A
Z_7346802_C	9C	14C
Z_7346803_G	9G	14G

Z_7346804_A	9A	14A
Z_7346805_C	9C	14C
Z_7346806_T	8T	14T
Z_7346807_C	8C	14C
Z_7346808_C	7C	14C
Z_7346809_C	6C	15C
Z_7346810_T	6T	13T
Z_7346811_C	6C	14C
Z_7346812_C	5C	14C
Z_7346813_A	5A	13A
Z_7346814_T	6T	13T
Z_7346815_A	6A	13A
Z_7346816_A	6A	13A
Z_7346817_A	6A	13A
Z_7346818_C	6C	13C
Z_7346819_A	6A	13A
Z_7346820_A	5A	13A
Z_7346821_G	5G	13G
Z_7346822_C	5C	12C
Z_7346823_A	5A	12A
Z_7346824_C	5C	12C
Z_7346825_G	5G	12G
Z_7346826_G	5G	12G
Z_7346827_C	3C	12C
Z_7346828_T	3T	12T
Z_7346829_G	3G	12G
Z_7346830_C	3C	11C
Z_7346831_C	3C	11C
Z_7346832_C	3C	11C
Z_7346833_A	3A	11A
Z_7346834_G	3G	11G
Z_7346835_A	3A	11A
Z_7346836_C	3C	12C
Z_7346837_C	3C	12C
Z_7346838_C	3C	12C
Z_7346839_C	3C	12C
Z_7346840_A	2A	11A
Z_7346841_G	2G	11G
Z_7346842_G	2G	11G
Z_7346843_C	2C	11C
Z_7346844_C	2C	11C
Z_7346845_T	1T_1C	11T
Z_7346846_C	2C	11C

Z_7346847_C	2C	11C
Z_7346848_C	2C	11C
Z_7346849_C	2C	11C
Z_7346850_C	2C	11C
Z_7346851_C	2C	11C
Z_7346852_C	2C	11C
Z_7346853_C	2C	10C
Z_7346854_C	2C	10C
Z_7346855_A	2A	9A
Z_7346856_C	2C	4T_6C
Z_7346857_G	1G_1C	10G
Z_7346858_C	2C	10C
Z_7346859_C	1C	10C
Z_7346860_A	1A	8A
Z_7346861_T	1T	9T
Z_7346862_A	2A	9A
Z_7346863_C	2C	9C
Z_7346864_A	2A	9A
Z_7346865_C	2C	9C
Z_7346866_A	2A	10A
Z_7346867_C	2C	11C
Z_7346868_C	2C	11C
Z_7346869_T	2T	12T
Z_7346870_C	2C	12C
Z_7346871_C	2C	11C
Z_7346872_G	2G	10G
Z_7346873_T	2T	9T
Z_7346874_T	2T	10T
Z_7346875_C	2C	10C
Z_7346876_T	2T	10T
Z_7346877_A	3A	10A
Z_7346878_T	3T	10T
Z_7346879_T	3T	10T
Z_7346880_A	3A	10A
Z_7346881_G	3G	10G
Z_7346882_T	4T	10T
Z_7346883_C	4C	11C
Z_7346884_T	3T	11T
Z_7346885_C	3C	11C
Z_7346886_G	3G	11G
Z_7346887_C	3C	10C
Z_7346888_C	3C	10C
Z_7346889_T	3T	9T

Z_7346890_T	3T_2C	9T
Z_7346891_C	3C	10C
Z_7346892_C	3C	10C
Z_7346893_C	1T_3C	10C
Z_7346894_C	3C	11C
Z_7346895_C	4C	11C
Z_7346896_C	4C	11C
Z_7346897_C	4C	11C
Z_7346898_C	4C	11C
Z_7346899_C	5C	12C
Z_7346900_C	5C	12C
Z_7346901_A	5A	12A
Z_7346902_A	5A	14A
Z_7346903_G	5G	13G
Z_7346904_G	5G	13G
Z_7346905_C	1A_4C	13C
Z_7346906_T	5T	13T
Z_7346907_G	6G	13G
Z_7346908_G	6G	14G
Z_7346909_C	6C	14C
Z_7346910_G	6G	14G
Z_7346911_G	6G	14G
Z_7346912_A	6A	15A
Z_7346913_G	6G	15G
Z_7346914_C	6C	15C
Z_7346915_C	7C	14C
Z_7346916_T	7T	15T
Z_7346917_G	9G	15G
Z_7346918_T	9T	15T
Z_7346919_A	8A	15A
Z_7346920_A	9A	16A
Z_7346921_T	9T	16T
Z_7346922_T	10T	16T
Z_7346923_C	11C	16C
Z_7346924_T	10T	16T
Z_7346925_C	11C	16C
Z_7346926_C	11C	16C
Z_7346927_C	11C	16C
Z_7346928_A	11A	16A
Z_7346929_G	11G	16G
Z_7346930_C	11T	16C
Z_7346931_G	10G	16G
Z_7346932_G	10G	16G

Z_7346933_C	10C	17C
Z_7346934_T	10T	18T
Z_7346935_G	10G	18G
Z_7346936_A	9A	18A
Z_7346937_C	10C	18C
Z_7346938_A	10A	20A
Z_7346939_C	10C	20C
Z_7346940_C	9C	20C
Z_7346941_A	11A	20A
Z_7346942_C	10C	20C
Z_7346943_A	11A	20A
Z_7346944_G	11G	20G
Z_7346945_G	12G	20G
Z_7346946_G	12G	21G
Z_7346947_A	11A	21A
Z_7346948_T	12T	22T
Z_7346949_G	12G	22G
Z_7346950_G	12G	23G
Z_7346951_C	11C	23C
Z_7346952_G	11G	23G
Z_7346953_G	11G	24G
Z_7346954_C	12C	24C
Z_7346955_A	12A	25A
Z_7346956_G	12G	25G
Z_7346957_G	12G	24G
Z_7346958_A	13A	24A
Z_7346959_C	14C	24C
Z_7346960_A	14A	24A
Z_7346961_T	14T	22T
Z_7346962_C	14C	21C
Z_7346963_A	14A	21A
Z_7346964_C	15C	10G_10C
Z_7346965_T	14T	20T
Z_7346966_C	14C	20C
Z_7346967_T	14T	21T
Z_7346968_T	14T	21T
Z_7346969_C	13C	20C
Z_7346970_A	12A	20A
Z_7346971_G	11G	20G
Z_7346972_G	13G	20G
Z_7346973_G	14G	20G
Z_7346974_G	13G	21G
Z_7346975_G	14G	21G

Z_7346976_C	13G	11G_10C
Z_7346977_C	13C	21C
Z_7346978_C	13C	21C
Z_7346979_C	14C	21C
Z_7346980_C	14C	21C
Z_7346981_C	14C	21C
Z_7346982_C	14C	21C
Z_7346983_T	14T	21T
Z_7346984_T	14T	20T
Z_7346985_T	13T	20T
Z_7346986_C	13C	19C
Z_7346987_C	9A_11C	16A_27C
Z_7346988_A	2A	14A
Z_7346989_C	11C	13C
Z_7346990_A	11A	13A
Z_7346991_C	11C	21C
Z_7346992_A	11A	23A
Z_7346993_C	10C	23C
Z_7346994_A	10A	22A
Z_7346995_C	11C	22C
Z_7346996_A	11A	22A
Z_7346997_C	10C	20C
Z_7346998_A	11A	21A
Z_7346999_C	11C	21C
Z_7347000_A	12A	21A
Z_7347001_C	12C	21C
Z_7347002_A	12A	21A
Z_7347003_C	12C	22C
Z_7347004_A	12C	24A
Z_7347005_C	12C	25C
Z_7347006_A	12A	26A
Z_7347007_T	12T	25T
Z_7347008_G	12G	25G
Z_7347009_G	12G	26G
Z_7347010_A	12A	26A
Z_7347011_T	12T	27T
Z_7347012_T	13T	27T
Z_7347013_G	12G	27G
Z_7347014_G	12G	27G
Z_7347015_C	12C	27C
Z_7347016_A	11A	27A
Z_7347017_G	13G	29G
Z_7347018_G	12G	30G

Z_7347019_C	12C	30C
Z_7347020_G	11G	30G
Z_7347021_A	12A	31A
Z_7347022_A	12A	31A
Z_7347023_G	12G	33G
Z_7347024_G	12G	33G
Z_7347025_C	12C	33C
Z_7347026_T	12T	34T
Z_7347027_C	12C	35C
Z_7347028_T	12T	33T
Z_7347029_C	12C	33C
Z_7347030_T	14T	33T
Z_7347031_C	13C	33C
Z_7347032_G	14G	33G
Z_7347033_T	14T	32T
Z_7347034_T	14T	33T
Z_7347035_T	16T	34T
Z_7347036_G	14G	33G
Z_7347037_T	15T	30T
Z_7347038_C	15C	31C
Z_7347039_A	15A	30A
Z_7347040_A	15A	31A
Z_7347041_C	15C	32C
Z_7347042_G	15G	32G
Z_7347043_C	16C	31C
Z_7347044_C	16C	31C
Z_7347045_A	15G	17A_13G
Z_7347046_G	16G	29G
Z_7347047_A	17A	31A
Z_7347048_C	17C	32C
Z_7347049_T	17T	31T
Z_7347050_G	18G	32G
Z_7347051_A	17A	32A
Z_7347052_C	18C	32C
Z_7347053_A	18A	32A
Z_7347054_G	18G	32G
Z_7347055_A	19A	33A
Z_7347056_A	20A	33A
Z_7347057_T	21T	33T
Z_7347058_T	22T	34T
Z_7347059_A	23A	35A
Z_7347060_C	24C	35C
Z_7347061_T	24T	40T

Z_7347062_C	23C	40C
Z_7347063_G	23G	40G
Z_7347064_C	23C	40C
Z_7347065_C	22C	41C
Z_7347066_C	22C	41C
Z_7347067_T	22T	43T
Z_7347068_C	22C	45C
Z_7347069_A	22A	45A
Z_7347070_A	22A	46A
Z_7347071_A	23A	45A
Z_7347072_T	23T	46T
Z_7347073_G	24G	45G
Z_7347074_A	25A	46A
Z_7347075_A	25A	47A
Z_7347076_A	26A	48A
Z_7347077_C	27C	47C
Z_7347078_G	27G	47G
Z_7347079_A	27A	47A
Z_7347080_T	27T	47T
Z_7347081_C	28C	47C
Z_7347082_T	27T	47T
Z_7347083_A	26A	46A
Z_7347084_T	27T	47T
Z_7347085_C	28C	48C
Z_7347086_T	28T	48T
Z_7347087_G	26G	50G
Z_7347088_G	26G	50G
Z_7347089_G	26G	50G
Z_7347090_G	26G	52G
Z_7347091_G	28G	52G
Z_7347092_A	28A	53A
Z_7347093_G	28G	52G
Z_7347094_T	28T	52T
Z_7347095_T	28T	52T
Z_7347096_A	30A	53A
Z_7347097_A	30A	53A
Z_7347098_A	30A	51A
Z_7347099_C	30C	50C
Z_7347100_C	27C	49C
Z_7347101_T	27T	49T
Z_7347102_A	26A	49A
Z_7347103_T	27T	51T
Z_7347104_T	27T	51T

Z_7347105_T	26T	51T
Z_7347106_C	25C	50C
Z_7347107_C	26C	50C
Z_7347108_A	26A	50A
Z_7347109_G	26G	50G
Z_7347110_T	27T	49T
Z_7347111_G	26G	50G
Z_7347112_G	26G	49G
Z_7347113_A	24A	46A
Z_7347114_G	25G	49G
Z_7347115_C	25C	49C
Z_7347116_T	24T	48T
Z_7347117_G	24G	48G
Z_7347118_A	24A	46A
Z_7347119_G	25G	46G
Z_7347120_A	24A	47A
Z_7347121_A	24A	46A
Z_7347122_G	23G	45G
Z_7347123_A	24A	46A
Z_7347124_G	24G	46G
Z_7347125_A	23A	47A
Z_7347126_G	23G	47G
Z_7347127_A	22A	47A
Z_7347128_G	21G	47G
Z_7347129_A	20A	45A
Z_7347130_G	19G	44G
Z_7347131_G	19G	44G
Z_7347132_G	19G	44G
Z_7347133_T	20T	44T
Z_7347134_T	21T	44T
Z_7347135_T	22T	43T
Z_7347136_T	22T	44T
Z_7347137_A	22A	44A
Z_7347138_G	23G	44G
Z_7347139_G	22G	44G
Z_7347140_G	21G	46G
Z_7347141_G	21G	46G
Z_7347142_G	23G	44G
Z_7347143_G	23G	44G
Z_7347144_G	23G	45G
Z_7347145_A	21A	43A
Z_7347146_C	23C	43C
Z_7347147_C	21C	43C

Z_7347148_C	22C	43C
Z_7347149_A	22A	42A
Z_7347150_G	22G	41G
Z_7347151_C	21C	42C
Z_7347152_A	21A	43A
Z_7347153_A	22A	42A
Z_7347154_T	23T	41T
Z_7347155_G	21G	41G
Z_7347156_T	22T	36T
Z_7347157_C	23C	35C
Z_7347158_A	22A	35A
Z_7347159_A	22A	34A
Z_7347160_G	22G	34G
Z_7347161_A	21A	34A
Z_7347162_G	22G	32G
Z_7347163_C	22C	31C
Z_7347164_C	22C	32C
Z_7347165_C	23C	31C
Z_7347166_A	21A	31A
Z_7347167_A	21A	32A
Z_7347168_T	21T	33T
Z_7347169_T	21T	32T
Z_7347170_A	21A	30A
Z_7347171_A	21A	28A
Z_7347172_T	20T	32T
Z_7347173_T	20T	32T
Z_7347174_T	19T	29T
Z_7347175_G	20G	32G
Z_7347176_G	20G	33G
Z_7347177_G	20G	33G
Z_7347178_G	20G	34G
Z_7347179_C	19C	33C
Z_7347180_A	18A	29A
Z_7347181_G	19G	33G
Z_7347182_G	19G	31G
Z_7347183_G	19G	31G
Z_7347184_T	19T	31T
Z_7347185_G	20G	34G
Z_7347186_G	20G	34G
Z_7347187_G	19G	34G
Z_7347188_G	19G	34G
Z_7347189_C	18C	34C
Z_7347190_A	20A	34A

Z_7347191_G	20G	34G
Z_7347192_G	18G	34G
Z_7347193_C	21C	1T_6G_26C
Z_7347194_C	23C	31C
Z_7347195_C	23C	33C
Z_7347196_C	21C	32C
Z_7347197_C	23C	30C
Z_7347198_C	23C	29C
Z_7347199_T	18T_6C	25T
Z_7347200_T	26T	27T
Z_7347201_C	27C	27C
Z_7347202_C	26C	27C
Z_7347203_C	25C	29C
Z_7347204_C	25C	29C
Z_7347205_C	24C	30C
Z_7347206_A	15A_8C	25A
Z_7347207_C	25C	27C
Z_7347208_C	26C	27C
Z_7347209_C	26C	26C
Z_7347210_C	26C	27C
Z_7347211_A	23A	27A
Z_7347212_C	24C	27C
Z_7347213_C	23C	28C
Z_7347214_C	23C	28C
Z_7347215_C	22C	28C
Z_7347216_G	21G	28G
Z_7347217_T	21T	28T
Z_7347218_C	20C	29C
Z_7347219_A	21A	30A
Z_7347220_C	20C	31C
Z_7347221_A	21A	32A
Z_7347222_A	22A	33A
Z_7347223_G	20G	36G
Z_7347224_G	22G	36G
Z_7347225_G	22G	36G
Z_7347226_C	24C	38C
Z_7347227_G	26G	38G
Z_7347228_A	26A	38A
Z_7347229_T	27T	38T
Z_7347230_G	29G	40G
Z_7347231_A	27A	40A
Z_7347232_G	28G	42G
Z_7347233_T	28T	43T

Z_7347234_C	28C	44C
Z_7347235_G	27G	43G
Z_7347236_G	26G	44G
Z_7347237_A	27A	46A
Z_7347238_T	27T	45T
Z_7347239_G	26G	45G
Z_7347240_T	29T	46T
Z_7347241_A	30A	47A
Z_7347242_T	28T	48T
Z_7347243_C	30C	48C
Z_7347244_C	31C	48C
Z_7347245_G	32G	49G
Z_7347246_A	33A	48A
Z_7347247_G	34G	47G
Z_7347248_A	34A	48A
Z_7347249_T	35T	48T
Z_7347250_C	35C	49C
Z_7347251_C	35C	49C
Z_7347252_A	35A	50A
Z_7347253_C	35C	53C
Z_7347254_C	35C	54C
Z_7347255_A	35A	56A
Z_7347256_A	35A	57A
Z_7347257_C	36C	57C
Z_7347258_T	36T	56T
Z_7347259_T	36T	56T
Z_7347260_T	34T	56T
Z_7347261_A	35A	57A
Z_7347262_T	36T	56T
Z_7347263_G	34G	55G
Z_7347264_G	33G	55G
Z_7347265_A	33A	54A
Z_7347266_C	32C	53C
Z_7347267_G	32G	53G
Z_7347268_G	31G	53G
Z_7347269_A	31A	52A
Z_7347270_G	30G	53G
Z_7347271_G	29G	52G
Z_7347272_A	29A	52A
Z_7347273_T	29T	52T
Z_7347274_A	29A	54A
Z_7347275_C	29C	54C
Z_7347276_C	29C	53C

Z_7347277_T	27T	54T
Z_7347278_T	26T	54T
Z_7347279_T	29T	54T
Z_7347280_A	31A	52A
Z_7347281_T	31T	52T
Z_7347282_A	31A	51A
Z_7347283_A	31A	51A
Z_7347284_G	30G	52G
Z_7347285_C	29C	53C
Z_7347286_C	30C	53C
Z_7347287_A	31A	53A
Z_7347288_A	31A	53A
Z_7347289_A	31A	53A
Z_7347290_A	32A	54A
Z_7347291_A	32A	53A
Z_7347292_C	31C	53C
Z_7347293_C	32C	53C
Z_7347294_T	31T	54T
Z_7347295_A	30A	54A
Z_7347296_T	29T	54T
Z_7347297_A	28A	55A
Z_7347298_A	28A	53A
Z_7347299_A	28A	53A
Z_7347300_G	26G	54G
Z_7347301_A	26A	54A
Z_7347302_G	26G	57G
Z_7347303_G	26G	57G
Z_7347304_G	25G	58G
Z_7347305_C	26C	58C
Z_7347306_C	26C	58C
Z_7347307_G	25G	58G
Z_7347308_A	25A	57A
Z_7347309_G	25G	58G
Z_7347310_A	23A	58A
Z_7347311_A	22A	61A
Z_7347312_A	22A	62A
Z_7347313_A	23A	61A
Z_7347314_A	22A	60A
Z_7347315_A	20A	58A
Z_7347316_A	20A	58A
Z_7347317_A	19A	57A
Z_7347318_A	20A	55A
Z_7347319_A	19A	53A

Z_7347320_C	20C	54C
Z_7347321_A	20A	53A
Z_7347322_C	20C	54C
Z_7347323_A	20A	55A
Z_7347324_G	21G	55G
Z_7347325_T	21T	53T
Z_7347326_G	20G	51G
Z_7347327_C	20C	51C
Z_7347328_A	20A	51A
Z_7347329_G	20G	51G
Z_7347330_G	20G	52G
Z_7347331_C	21C	51C
Z_7347332_T	20T	50T
Z_7347333_G	19G	51G
Z_7347334_A	19A	51A
Z_7347335_A	18A	52A
Z_7347336_A	18A	51A
Z_7347337_A	18A	50A
Z_7347338_G	18G	49G
Z_7347339_C	18C	50C
Z_7347340_A	18A	50A
Z_7347341_A	17A	51A
Z_7347342_A	17A	52A
Z_7347343_G	16G	52G
Z_7347344_T	18T	51T
Z_7347345_T	19T	52T
Z_7347346_C	19C	51C
Z_7347347_C	21C	52C
Z_7347348_T	20T	50T
Z_7347349_T	19T	49T
Z_7347350_G	18G	48G
Z_7347351_G	16G	47G
Z_7347352_C	17C	47C
Z_7347353_A	17A	49A
Z_7347354_A	18A	49A
Z_7347355_A	19A	50A
Z_7347356_G	19G	49G
Z_7347357_T	19T	50T
Z_7347358_G	18G	49G
Z_7347359_C	20C	49C
Z_7347360_C	20C	49C
Z_7347361_T	20T	48T
Z_7347362_C	6T_20C	50C

Z_7347363_T	22T	50T
Z_7347364_T	23T	53T
Z_7347365_T	23T	54T
Z_7347366_T	61T_19G	55T
Z_7347367_G	22G	52G
Z_7347368_G	22G	55G
Z_7347369_G	25G	53G
Z_7347370_G	25G	54G
Z_7347371_G	25G	54G
Z_7347372_G	24G	54G
Z_7347373_G	25G	53G
Z_7347374_T	22G	52T
Z_7347375_G	25G	52G
Z_7347376_A	22A	53A
Z_7347377_C	25C	53C
Z_7347378_T	25T	53T
Z_7347379_A	25A	53A
Z_7347380_C	25C	51C
Z_7347381_A	25A	51A
Z_7347382_G	22G	51G
Z_7347383_A	24A	52A
Z_7347384_T	23T	52T
Z_7347385_C	22C	51C
Z_7347386_C	22C	51C
Z_7347387_C	23C	54C
Z_7347388_A	24A	54A
Z_7347389_G	25G	54G
Z_7347390_A	23A	53A
Z_7347391_A	26A	55A
Z_7347392_T	26T	55T
Z_7347393_C	26C	57C
Z_7347394_C	26C	57C
Z_7347395_C	26C	58C
Z_7347396_C	27C	58C
Z_7347397_C	27C	56C
Z_7347398_A	27A	56A
Z_7347399_G	27G	58G
Z_7347400_A	26A	57A
Z_7347401_G	28G	59G
Z_7347402_T	28T	59T
Z_7347403_C	28C	59C
Z_7347404_C	29C	59C
Z_7347405_A	29A	59A

Z_7347406_C	29C	57C
Z_7347407_A	30A	56A
Z_7347408_C	30C	56C
Z_7347409_A	31A	57A
Z_7347410_T	31T	57T
Z_7347411_A	31A	57A
Z_7347412_A	29A	56A
Z_7347413_G	30G	57G
Z_7347414_C	30C	56C
Z_7347415_G	30G	56G
Z_7347416_C	30C	57C
Z_7347417_T	30T	53T
Z_7347418_T	31T	55T
Z_7347419_G	31G	55G
Z_7347420_C	28C	56C
Z_7347421_C	27C	56C
Z_7347422_C	28C	57C
Z_7347423_G	29G	60G
Z_7347424_T	30T	59T
Z_7347425_A	28A	60A
Z_7347426_T	29T	59T
Z_7347427_T	28T	59T
Z_7347428_T	29T	59T
Z_7347429_T	30T	58T
Z_7347430_G	29G	59G
Z_7347431_G	30G	60G
Z_7347432_A	27A	62A
Z_7347433_T	29T	64T
Z_7347434_T	27T	64T
Z_7347435_A	26A	64A
Z_7347436_G	27G	62G
Z_7347437_G	25G	61G
Z_7347438_C	28C	60C
Z_7347439_A	26A	62A
Z_7347440_A	27A	63A
Z_7347441_A	27A	63A
Z_7347442_G	27G	62G
Z_7347443_T	27T	61T
Z_7347444_G	28G	62G
Z_7347445_G	27G	62G
Z_7347446_T	28T	62T
Z_7347447_C	30C	63C
Z_7347448_T	31T	62T

Z_7347449_G	32G	63G
Z_7347450_T	33T	62T
Z_7347451_G	32G	63G
Z_7347452_G	34G	63G
Z_7347453_A	35A	61A
Z_7347454_C	35C	62C
Z_7347455_C	36C	63C
Z_7347456_A	36A	64A
Z_7347457_A	37A	63A
Z_7347458_C	37C	62C
Z_7347459_C	37C	61C
Z_7347460_T	37T	61T
Z_7347461_T	36T	61T
Z_7347462_G	36G	61G
Z_7347463_A	35A	61A
Z_7347464_C	34C	64C
Z_7347465_T	36T	63T
Z_7347466_G	35G	62G
Z_7347467_G	35G	64G
Z_7347468_G	35G	65G
Z_7347469_G	35G	66G
Z_7347470_C	38C	67C
Z_7347471_G	38G	68G
Z_7347472_T	37T	68T
Z_7347473_C	37C	67C
Z_7347474_G	38G	70G
Z_7347475_C	36C	70C
Z_7347476_C	37C	70C
Z_7347477_C	36C	70C
Z_7347478_C	35C	71C
Z_7347479_T	35T	71T
Z_7347480_G	34G	72G
Z_7347481_G	36G	72G
Z_7347482_G	36G	69G
Z_7347483_T	37T	70T
Z_7347484_G	37G	70G
Z_7347485_C	38C	70C
Z_7347486_T	38T	68T
Z_7347487_G	38G	68G
Z_7347488_A	38A	67A
Z_7347489_A	38A	66A
Z_7347490_A	39A	65A
Z_7347491_T	39T	63T

Z_7347492_G	38G	62G
Z_7347493_T	40T	35T_27C
Z_7347494_G	40G	60G
Z_7347495_G	41G	60G
Z_7347496_A	40A	58A
Z_7347497_A	40G	33A_26G
Z_7347498_G	40G	61G
Z_7347499_G	39G	63G
Z_7347500_T	39T	63T
Z_7347501_G	38G	63G
Z_7347502_C	40C	63C
Z_7347503_C	38C	62C
Z_7347504_A	38A	61A
Z_7347505_A	39A	61A
Z_7347506_A	39A	61A
Z_7347507_A	39A	61A
Z_7347508_T	38T	62T
Z_7347509_A	38A	62A
Z_7347510_C	37C	61C
Z_7347511_A	37A	61A
Z_7347512_G	37G	61G
Z_7347513_A	37A	62A
Z_7347514_G	36G	63G
Z_7347515_G	37G	61G
Z_7347516_G	36T	29T_33G
Z_7347517_A	35A	61A
Z_7347518_A	34A	58A
Z_7347519_G	32G	56G
Z_7347520_T	31T	58T
Z_7347521_C	32C	58C
Z_7347522_T	31T	59T
Z_7347523_A	30A	57A
Z_7347524_C	29C	57C
Z_7347525_T	29T	56T
Z_7347526_C	29C	55C
Z_7347527_G	28G	53G
Z_7347528_T	29T	28T_24C
Z_7347529_G	28G	52G
Z_7347530_A	28A	52A
Z_7347531_G	29G	53G
Z_7347532_C	29T	53C
Z_7347533_A	29A	28A_26G
Z_7347534_T	29T	53T

Z_7347535_T	29T	53T
Z_7347536_A	30A	53A
Z_7347537_A	30A	54A
Z_7347538_G	30G	55G
Z_7347539_A	30A	56A
Z_7347540_A	27A	55A
Z_7347541_A	26A	54A
Z_7347542_A	25A	53A
Z_7347543_T	26T	56T
Z_7347544_A	25A	54A
Z_7347545_C	25C	55C
Z_7347546_G	25G	57G
Z_7347547_T	25T	56T
Z_7347548_G	25G	58G
Z_7347549_G	26G	58G
Z_7347550_G	27G	29G_28C
Z_7347551_A	26A	57A
Z_7347552_A	29A	58A
Z_7347553_C	28C	59C
Z_7347554_A	28A	59A
Z_7347555_G	27G	57G
Z_7347556_C	28C	57C
Z_7347557_T	29T	56T
Z_7347558_C	28C	56C
Z_7347559_T	26T	52T
Z_7347560_C	27C	53C
Z_7347561_A	26G	28A_26G
Z_7347562_G	26G	52G
Z_7347563_T	25T	50T
Z_7347564_C	25C	50C
Z_7347565_C	25C	49C
Z_7347566_T	25T	48T
Z_7347567_G	25G	50G
Z_7347568_T	24T	52T
Z_7347569_T	24T	51T
Z_7347570_C	24C	51C
Z_7347571_T	24T	51T
Z_7347572_G	22G	51G
Z_7347573_T	23T	51T
Z_7347574_T	22T	49T
Z_7347575_C	20C	46C
Z_7347576_T	23T	50T
Z_7347577_T	23T	50T_17C

Z_7347578_C	17T_40C	49C
Z_7347579_T	21T	49T
Z_7347580_T	23T	48T
Z_7347581_C	21C	49C
Z_7347582_C	22C	32T_48C
Z_7347583_T	32T	33T
Z_7347584_T	8T_1G_8C	29T
Z_7347585_C	26C	50C
Z_7347586_C	27C	49C
Z_7347587_C	27C	48C
Z_7347588_C	27C	49C
Z_7347589_C	27C	50C
Z_7347590_C	28C	51C
Z_7347591_C	27C	51C
Z_7347592_C	28C	51C
Z_7347593_C	28C	53C
Z_7347594_A	29A	52A
Z_7347595_A	28A	54A
Z_7347596_A	28A	54A
Z_7347597_C	28C	53C
Z_7347598_C	28C	53C
Z_7347599_T	28T	53T
Z_7347600_A	27A	56A
Z_7347601_A	27A	57A
Z_7347602_A	25A	56A
Z_7347603_C	26C	58C
Z_7347604_T	27T	60T
Z_7347605_T	25T	60T
Z_7347606_C	23C	60C
Z_7347607_C	26C	61C
Z_7347608_T	27T	62T
Z_7347609_T	27T	61T
Z_7347610_T	27T	60T
Z_7347611_T	27T	59T
Z_7347612_T	27T	60T
Z_7347613_T	28T	60T
Z_7347614_C	28C	62C
Z_7347615_T	29T	63T
Z_7347616_T	29T	62T
Z_7347617_C	29C	62C
Z_7347618_A	29A	62A
Z_7347619_C	20G	29G_32C
Z_7347620_T	21G	32T_29G

Z_7347621_G	25G	67G
Z_7347622_G	27G	68G
Z_7347623_T	28T	68T
Z_7347624_G	30G	70G
Z_7347625_G	31G	69G
Z_7347626_T	31T	67T
Z_7347627_A	32A	67A
Z_7347628_C	33C	67C
Z_7347629_C	34C	68C
Z_7347630_A	34A	66A
Z_7347631_G	34G	68G
Z_7347632_C	35C	69C
Z_7347633_T	35T	66T
Z_7347634_C	36C	65C
Z_7347635_C	37C	66C
Z_7347636_T	37T	65T
Z_7347637_G	38G	66G
Z_7347638_G	39G	65G
Z_7347639_G	39C	38G_28C
Z_7347640_A	39A	66A
Z_7347641_A	38A	67A
Z_7347642_A	38A	68A
Z_7347643_C	38C	67C
Z_7347644_A	39A	67A
Z_7347645_T	38T	68T
Z_7347646_T	39T	67T
Z_7347647_G	37G	68G
Z_7347648_G	39G	67G
Z_7347649_G	38G	66G
Z_7347650_G	38G	66G
Z_7347651_G	38G	67G
Z_7347652_G	37G	67G
Z_7347653_G	35G	67G
Z_7347654_G	36G	66G
Z_7347655_C	37C	66C
Z_7347656_G	37G	66G
Z_7347657_C	37C	67C
Z_7347658_T	37T	62T
Z_7347659_C	38C	67C
Z_7347660_T	39T	66T
Z_7347661_T	39T	68T
Z_7347662_C	38C	69C
Z_7347663_C	38T	66C

Z_7347664_A	38A	66A
Z_7347665_C	39C	65C
Z_7347666_T	41T	65T
Z_7347667_C	41C	68C
Z_7347668_A	40A	68A
Z_7347669_C	41C	69C
Z_7347670_C	43C	69C
Z_7347671_T	44T	70T
Z_7347672_C	45C	72C
Z_7347673_T	45T	72T
Z_7347674_T	44T	74T
Z_7347675_C	44C	73C
Z_7347676_C	44C	74C
Z_7347677_C	42C	75C
Z_7347678_C	42C	76C
Z_7347679_A	43A	76A
Z_7347680_T	45T	74T
Z_7347681_A	46A	75A
Z_7347682_G	44G	76G
Z_7347683_G	37T_43G	33T_77G
Z_7347684_A	37A	74A
Z_7347685_A	41A	75A
Z_7347686_A	39A	75A
Z_7347687_A	39A	77A
Z_7347688_G	38G	76G
Z_7347689_G	38G	76G
Z_7347690_A	37A	74A
Z_7347691_T	37T	73T
Z_7347692_G	36G	73G
Z_7347693_C	36C	71C
Z_7347694_T	33C	71T
Z_7347695_G	33G	70G
Z_7347696_G	32G	70G
Z_7347697_G	32G	72G
Z_7347698_A	31A	70A
Z_7347699_C	30C	70C
Z_7347700_T	31T	69T
Z_7347701_A	31A	68A
Z_7347702_G	30G	69G
Z_7347703_A	32A	68A
Z_7347704_G	32G	68G
Z_7347705_G	33G	68G
Z_7347706_G	35G	68G

Z_7347707_G	34G	67G
Z_7347708_G	36G	65G
Z_7347709_C	36C	65C
Z_7347710_C	36C	66C
Z_7347711_T	36T	66T
Z_7347712_A	36A	66A
Z_7347713_A	35A	64A
Z_7347714_T	35T	64T
Z_7347715_C	34C	63C
Z_7347716_C	33C	63C
Z_7347717_T	33T	63T
Z_7347718_G	33G	62G
Z_7347719_C	33C	63C
Z_7347720_A	33A	65A
Z_7347721_C	33C	66C
Z_7347722_T	32C	40T_26C
Z_7347723_A	31A	66A
Z_7347724_T	30T	67T
Z_7347725_A	32A	67A
Z_7347726_T	33T	66T
Z_7347727_T	32T	66T
Z_7347728_T	34T	66T
Z_7347729_C	33C	64C
Z_7347730_A	33A	64A
Z_7347731_T	32T	67T
Z_7347732_G	34G	69G
Z_7347733_C	34C	69C
Z_7347734_A	34A	70A
Z_7347735_T	33T	68T
Z_7347736_T	32T	62T
Z_7347737_C	33C	65C
Z_7347738_T	33T	64T
Z_7347739_T	31T	65T
Z_7347740_C	34C	65C
Z_7347741_C	34C	68C
Z_7347742_T	35T	68T
Z_7347743_G	35G	68G
Z_7347744_A	34A	68A
Z_7347745_C	34C	67C
Z_7347746_T	35T	66T
Z_7347747_G	35G	67G
Z_7347748_C	36C	71C
Z_7347749_C	34C	71C

Z_7347750_A	33T	43A_29T
Z_7347751_A	34A	72A
Z_7347752_C	34C	72C
Z_7347753_T	34T	72T
Z_7347754_T	33T	74T
Z_7347755_C	35C	74C
Z_7347756_T	34T	74T
Z_7347757_C	34C	73C
Z_7347758_T	35T	72T
Z_7347759_C	38C	73C
Z_7347760_T	37T	73T
Z_7347761_A	37A	72A
Z_7347762_A	36A	70A
Z_7347763_G	37G	69G
Z_7347764_C	36C	70C
Z_7347765_C	38C	69C
Z_7347766_A	38G	40A_28G
Z_7347767_G	38G	67G
Z_7347768_G	40G	67G
Z_7347769_T	40T	67T
Z_7347770_G	40G	70G
Z_7347771_T	41T	68T
Z_7347772_G	41G	70G
Z_7347773_T	39T	67T
Z_7347774_G	40G	67G
Z_7347775_T	38T	67T
Z_7347776_G	36G	67G
Z_7347777_T	37T	66T
Z_7347778_G	32G	65G
Z_7347779_T	33T	64T
Z_7347780_G	34G	63G
Z_7347781_C	35C	63C
Z_7347782_T	34T	61T
Z_7347783_T	35T	35T_27C
Z_7347784_T	34T	62T
Z_7347785_G	33G	65G
Z_7347786_T	34T	64T
Z_7347787_G	34G	65G
Z_7347788_G	34G	65G
Z_7347789_C	35C	64C
Z_7347790_T	36T	64T
Z_7347791_G	35G	64G
Z_7347792_T	36T	62T

Z_7347793_T	36T	64T
Z_7347794_T	36T	63T
Z_7347795_T	36T	63T
Z_7347796_A	36A	66A
Z_7347797_T	36T	68T
Z_7347798_G	34G	70G
Z_7347799_T	35T	69T
Z_7347800_C	34C	71C
Z_7347801_T	34T	71T
Z_7347802_C	33C	71C
Z_7347803_C	32C	72C
Z_7347804_A	32A	70A
Z_7347805_G	32G	70G
Z_7347806_T	31T	70T
Z_7347807_T	31T	68T
Z_7347808_T	31T	67T
Z_7347809_C	31C	68C
Z_7347810_C	29C	66C
Z_7347811_A	28A	68A
Z_7347812_G	28G	67G
Z_7347813_C	28C	66C
Z_7347814_C	30C	65C
Z_7347815_T	32T	63T
Z_7347816_T	31T	61T
Z_7347817_C	32C	61C
Z_7347818_T	30T	60T
Z_7347819_T	33T	60T
Z_7347820_T	33T	59T
Z_7347821_G	32G	60G
Z_7347822_T	33T	59T
Z_7347823_C	33C	60C
Z_7347824_A	33A	61A
Z_7347825_C	32C	60C
Z_7347826_T	34T	58T
Z_7347827_G	35G	56G
Z_7347828_G	36G	56G
Z_7347829_T	33T	57T
Z_7347830_T	33T	57T
Z_7347831_A	33A	57A
Z_7347832_T	33T	57T
Z_7347833_G	34G	58G
Z_7347834_C	33C	57C
Z_7347835_T	34T	56T

Z_7347836_G	35G	54G
Z_7347837_G	34G	54G
Z_7347838_G	33G	54G
Z_7347839_T	34T	54T
Z_7347840_A	34A	55A
Z_7347841_A	35A	55A
Z_7347842_A	35A	55A
Z_7347843_G	36G	51G
Z_7347844_G	34G	50G
Z_7347845_C	34C	51C
Z_7347846_A	33A	51A
Z_7347847_G	33G	50G
Z_7347848_A	34A	50A
Z_7347849_T	35T	48T
Z_7347850_G	35G	47G
Z_7347851_G	36G	47G
Z_7347852_G	38G	46G
Z_7347853_A	38A	46A
Z_7347854_G	38G	46G
Z_7347855_C	38C	47C
Z_7347856_T	37T	48T
Z_7347857_G	37G	47G
Z_7347858_T	37T	46T
Z_7347859_G	36G	45G
Z_7347860_C	35C	46C
Z_7347861_T	34T	46T
Z_7347862_C	35C	45C
Z_7347863_A	35A	45A
Z_7347864_A	36A	43A
Z_7347865_A	34A	41A
Z_7347866_C	34C	45C
Z_7347867_C	34C	45C
Z_7347868_A	34A	45A
Z_7347869_C	33C	45C
Z_7347870_A	33A	48A
Z_7347871_T	33T	48T
Z_7347872_C	33C	50C
Z_7347873_T	33T	50T
Z_7347874_G	33G	50G
Z_7347875_G	35G	50G
Z_7347876_A	36A	50A
Z_7347877_C	37C	50C
Z_7347878_A	37A	50A

Z_7347879_G	36G	50G
Z_7347880_C	36C	47C
Z_7347881_C	37C	47C
Z_7347882_T	36T	47T
Z_7347883_C	34C	48C
Z_7347884_A	33A	49A
Z_7347885_C	33C	51C
Z_7347886_T	34T	51T
Z_7347887_T	33T	54T
Z_7347888_C	34C	52C
Z_7347889_C	31C	52C
Z_7347890_C	31C	54C
Z_7347891_C	31C	51C
Z_7347892_T	29T	48T
Z_7347893_C	28C	46C
Z_7347894_C	28C	47C
Z_7347895_C	28C	45C
Z_7347896_A	26A	44A
Z_7347897_G	25G	45G
Z_7347898_T	23T	45T
Z_7347899_C	23C	45C
Z_7347900_A	23A	44A
Z_7347901_A	23A	43A
Z_7347902_A	23A	45A
Z_7347903_G	18A_18T_40G_30C	46G
Z_7347904_C	22C	47C
Z_7347905_C	21C	47C
Z_7347906_T	6T	45T
Z_7347907_C	6C	46C
Z_7347908_T	6C	26T_22C
Z_7347909_C	6C	49C
Z_7347910_T	6T	50T
Z_7347911_A	7A	50A
Z_7347912_A	7A	50A
Z_7347913_A	8G	48A
Z_7347914_C	21C	50C
Z_7347915_C	21C	51C
Z_7347916_C	21C	49C
Z_7347917_A	21A	49A
Z_7347918_C	21C	48C
Z_7347919_C	21C	48C
Z_7347920_A	21A	48A
Z_7347921_T	21T	49T

Z_7347922_G	23G	49G
Z_7347923_C	23C	50C
Z_7347924_T	23T	49T
Z_7347925_T	23T	50T
Z_7347926_C	23C	50C
Z_7347927_C	24C	50C
Z_7347928_T	26T	49T
Z_7347929_T	26T	48T
Z_7347930_T	26T	50T
Z_7347931_T	26T	50T
Z_7347932_C	25C	51C
Z_7347933_T	25T	51T
Z_7347934_A	25A	51A
Z_7347935_T	26T	49T
Z_7347936_C	28C	52C
Z_7347937_T	28T	52T
Z_7347938_C	28C	52C
Z_7347939_A	31A	54A
Z_7347940_T	30T	53T
Z_7347941_T	31T	54T
Z_7347942_T	30T	54T
Z_7347943_C	30C	55C
Z_7347944_C	32C	59C
Z_7347945_T	31T	58T
Z_7347946_C	30C	60C
Z_7347947_A	29A	61A
Z_7347948_C	28C	61C
Z_7347949_C	32C	62C
Z_7347950_T	33T	61T
Z_7347951_T	31T	61T
Z_7347952_T	32T	63T
Z_7347953_C	33C	62C
Z_7347954_C	33C	64C
Z_7347955_A	33A	63A
Z_7347956_C	33C	63C
Z_7347957_C	34C	65C
Z_7347958_T	35T	67T
Z_7347959_T	37T	67T
Z_7347960_A	34A	67A
Z_7347961_T	33T	64T
Z_7347962_T	33T	63T
Z_7347963_C	34C	63C
Z_7347964_C	36C	64C

Z_7347965_A	36A	63A
Z_7347966_T	36T	64T
Z_7347967_A	37A	62A
Z_7347968_C	37C	62C
Z_7347969_C	37C	61C
Z_7347970_T	38T	62T
Z_7347971_A	39A	62A
Z_7347972_A	39A	63A
Z_7347973_G	39G	64G
Z_7347974_C	40C	65C
Z_7347975_C	41C	66C
Z_7347976_A	42A	65A
Z_7347977_A	43A	65A
Z_7347978_C	44C	64C
Z_7347979_A	44A	64A
Z_7347980_C	44C	62C
Z_7347981_A	43A	62A
Z_7347982_G	43G	59G
Z_7347983_A	42A	59A
Z_7347984_G	43G	59G
Z_7347985_T	43T	57T
Z_7347986_G	43G	58G
Z_7347987_C	43C	58C
Z_7347988_T	43T	58T
Z_7347989_C	42C	57C
Z_7347990_C	44C	58C
Z_7347991_A	45A	58A
Z_7347992_C	43C	58C
Z_7347993_A	45A	59A
Z_7347994_A	45A	59A
Z_7347995_T	45T	59T
Z_7347996_G	45T	26T_33G
Z_7347997_A	45A	57A
Z_7347998_A	43A	59A
Z_7347999_G	43G	58G
Z_7348000_C	44G	61C
Z_7348001_C	45C	66C
Z_7348002_A	46A	66A
Z_7348003_A	45A	65A
Z_7348004_T	44T	64T
Z_7348005_G	44G	63G
Z_7348006_C	42C	63C
Z_7348007_C	42C	26T_36C

Z_7348008_A	42G	36A_27G
Z_7348009_A	41A	62A
Z_7348010_G	41G	64G
Z_7348011_T	41T	65T
Z_7348012_G	42G	65G
Z_7348013_A	41A	65A
Z_7348014_G	38G	66G
Z_7348015_G	38G	66G
Z_7348016_G	38G	65G
Z_7348017_C	39C	69C
Z_7348018_A	38A	68A
Z_7348019_T	37T	68T
Z_7348020_T	36T	67T
Z_7348021_G	36G	68G
Z_7348022_C	36C	66C
Z_7348023_C	35C	67C
Z_7348024_T	37T	67T
Z_7348025_T	36T	65T
Z_7348026_G	35G	64G
Z_7348027_A	37A	63A
Z_7348028_T	36T	63T
Z_7348029_A	35A	64A
Z_7348030_C	34C	64C
Z_7348031_A	34A	63A
Z_7348032_G	34G	62G
Z_7348033_A	33A	61A
Z_7348034_C	31C	60C
Z_7348035_C	32C	64C
Z_7348036_T	31T	63T
Z_7348037_T	29T	64T
Z_7348038_C	29C	63C
Z_7348039_C	29C	61C
Z_7348040_T	28T	61T
Z_7348041_C	27C	59C
Z_7348042_G	26G	59G
Z_7348043_G	31G	59G
Z_7348044_G	32G	58G
Z_7348045_G	31G	58G
Z_7348046_C	31C	56C
Z_7348047_G	31G	56G
Z_7348048_C	31C	56C
Z_7348049_T	30T	54T
Z_7348050_T	31T	57T

Z_7348051_A	31A	56A
Z_7348052_A	33A	56A
Z_7348053_C	34C	56C
Z_7348054_G	34G	56G
Z_7348055_A	34A	57A
Z_7348056_C	34C	56C
Z_7348057_T	34T	58T
Z_7348058_G	34G	58G
Z_7348059_T	35T	61T
Z_7348060_C	35T	60C
Z_7348061_T	34T	60T
Z_7348062_T	34T	60T
Z_7348063_G	34G	62G
Z_7348064_C	32C	63C
Z_7348065_A	32A	63A
Z_7348066_G	32G	63G
Z_7348067_A	31A	62A
Z_7348068_A	31A	61A
Z_7348069_T	31T	62T
Z_7348070_G	31G	61G
Z_7348071_T	30T	62T
Z_7348072_C	29C	63C
Z_7348073_T	14C	64T
Z_7348074_G	14A	64G
Z_7348075_C	14C	63C
Z_7348076_C	14C	65C
Z_7348077_C	14T	65C
Z_7348078_T	16C	65T
Z_7348079_A	23A	66A
Z_7348080_C	24C	31G_36C
Z_7348081_A	24A	67A
Z_7348082_G	24G	67G
Z_7348083_A	24A	66A
Z_7348084_T	24T	67T
Z_7348085_T	24C	35T_32C
Z_7348086_G	24G	66G
Z_7348087_G	24G	67G
Z_7348088_A	23A	69A
Z_7348089_G	25G	68G
Z_7348090_G	26G	69G
Z_7348091_T	26T	68T
Z_7348092_G	26T	69G
Z_7348093_C	26C	69C

Z_7348094_C	26C	70C
Z_7348095_T	27T	68T
Z_7348096_T	28T	62T
Z_7348097_C	28C	63C
Z_7348098_A	28A	62A
Z_7348099_C	28C	62C
Z_7348100_T	30T	62T
Z_7348101_G	31G	61G
Z_7348102_G	31G	61G
Z_7348103_C	30C	62C
Z_7348104_C	32C	63C
Z_7348105_T	32T	64T
Z_7348106_C	31C	63C
Z_7348107_A	33A	63A
Z_7348108_A	34A	64A
Z_7348109_A	36A	63A
Z_7348110_G	35G	63G
Z_7348111_G	36G	65G
Z_7348112_C	34C	63C
Z_7348113_T	34T	63T
Z_7348114_T	33T	63T
Z_7348115_T	32T	63T
Z_7348116_T	32T	62T
Z_7348117_C	31C	63C
Z_7348118_A	32A	62A
Z_7348119_G	32G	64G
Z_7348120_G	31G	64G
Z_7348121_G	31G	64G
Z_7348122_A	31A	65A
Z_7348123_G	30G	65G
Z_7348124_T	30T_27C	65T_28C
Z_7348125_C	31C	68C
Z_7348126_C	32C	67C
Z_7348127_A	31A	66A
Z_7348128_C	31C	66C
Z_7348129_C	31C	67C
Z_7348130_T	33T	66T
Z_7348131_G	33G	66G
Z_7348132_G	33G	66G
Z_7348133_C	32C	66C
Z_7348134_C	31C	65C
Z_7348135_T	32T	65T
Z_7348136_T	31T	64T

Z_7348137_C	34C	64C
Z_7348138_A	34A	64A
Z_7348139_G	35G	65G
Z_7348140_G	38G	67G
Z_7348141_T	38T	66T
Z_7348142_C	36C	66C
Z_7348143_T	36T	67T
Z_7348144_C	36C	69C
Z_7348145_T	36T	69T
Z_7348146_G	37G	67G
Z_7348147_T	37T	68T
Z_7348148_A	36A	66A
Z_7348149_A	38A	69A
Z_7348150_C	38C	70C
Z_7348151_T	38T	71T
Z_7348152_G	38G	69G
Z_7348153_C	37C	67C
Z_7348154_C	36C	65C
Z_7348155_C	38C	65C
Z_7348156_C	39C	64C
Z_7348157_T	40T	64T
Z_7348158_T	40T	61T
Z_7348159_C	40C	62C
Z_7348160_C	40C	61C
Z_7348161_T	41T	61T
Z_7348162_C	43C	59C
Z_7348163_C	44C	60C
Z_7348164_A	43A	60A
Z_7348165_A	42A	59A
Z_7348166_A	42A	58A
Z_7348167_T	45T	57T
Z_7348168_A	46A	56A
Z_7348169_C	44C	60C
Z_7348170_A	43A	61A
Z_7348171_C	43C	60C
Z_7348172_C	42C	59C
Z_7348173_C	41C	59C
Z_7348174_C	39C	60C
Z_7348175_A	41A	58A
Z_7348176_A	39A	59A
Z_7348177_A	38A	59A
Z_7348178_C	37C	60C
Z_7348179_A	37A	59A

Z_7348180_A	36A	59A
Z_7348181_C	36C	59C
Z_7348182_A	37A	57A
Z_7348183_C	37C	58C
Z_7348184_A	38A	58A
Z_7348185_A	38A	61A
Z_7348186_A	38A	62A
Z_7348187_C	39C	61C
Z_7348188_G	39G	62G
Z_7348189_C	40C	61C
Z_7348190_A	41A	60A
Z_7348191_A	41A	60A
Z_7348192_A	41A	60A
Z_7348193_G	42G	61G
Z_7348194_T	42T	62T
Z_7348195_G	40G	63G
Z_7348196_T	39T	62T
Z_7348197_T	39T	61T
Z_7348198_C	40C	61C
Z_7348199_T	40T	58T
Z_7348200_T	42T	56T
Z_7348201_C	42C	56C
Z_7348202_A	43A	58A
Z_7348203_T	43T	57T
Z_7348204_T	43T	59T
Z_7348205_C	42C	59C
Z_7348206_A	42A	58A
Z_7348207_T	39T	59T
Z_7348208_C	39C	61C
Z_7348209_C	38C	64C
Z_7348210_A	38A	67A
Z_7348211_C	38C	66C
Z_7348212_A	37A	68A
Z_7348213_T	39T	72T
Z_7348214_C	40C	72C
Z_7348215_C	39C	72C
Z_7348216_T	39T	71T
Z_7348217_C	39C	72C
Z_7348218_A	39A	70A
Z_7348219_G	38G	68G
Z_7348220_C	38C	69C
Z_7348221_T	40T	69T
Z_7348222_T	40T	68T

Z_7348223_T	40T	71T
Z_7348224_C	41C	70C
Z_7348225_A	39A	72A
Z_7348226_A	41A	71A
Z_7348227_A	40A	71A
Z_7348228_A	42A	75A
Z_7348229_A	41A	75A
Z_7348230_C	41C	77C
Z_7348231_G	42G	77G
Z_7348232_A	40A	78A
Z_7348233_A	39A	78A
Z_7348234_T	41T	77T
Z_7348235_T	44T	75T
Z_7348236_T	45T	77T
Z_7348237_T	46T	78T
Z_7348238_G	47G	79G
Z_7348239_T	48T	77T
Z_7348240_G	48G	80G
Z_7348241_C	50C	81C
Z_7348242_T	51T	83T
Z_7348243_C	52C	83C
Z_7348244_G	53G	84G
Z_7348245_C	53C	82C
Z_7348246_C	53C	81C
Z_7348247_A	54A	81A
Z_7348248_T	53T	81T
Z_7348249_G	52G	82G
Z_7348250_C	55C	84C
Z_7348251_T	55T	85T
Z_7348252_C	54C	85C
Z_7348253_T	56T	85T
Z_7348254_T	56T	85T
Z_7348255_G	56G	85G
Z_7348256_T	56T	84T
Z_7348257_G	54G	87G
Z_7348258_C	54C	86C
Z_7348259_A	56A	85A
Z_7348260_A	55A	85A
Z_7348261_T	52T	86T
Z_7348262_A	53A	86A
Z_7348263_T	53T	84T
Z_7348264_A	53A	82A
Z_7348265_G	54G	81G

Z_7348266_C	55C	82C
Z_7348267_T	58T	83T
Z_7348268_T	58T	83T
Z_7348269_C	57C	83C
Z_7348270_C	55C	83C
Z_7348271_A	55A	82A
Z_7348272_G	55G	83G
Z_7348273_C	55C	83C
Z_7348274_A	56A	82A
Z_7348275_T	54T	84T
Z_7348276_C	54C	83C
Z_7348277_T	55T	81T
Z_7348278_G	54G	82G
Z_7348279_C	55C	83C
Z_7348280_C	52C	80C
Z_7348281_A	53A	79A
Z_7348282_G	52G	79G
Z_7348283_C	52C	79C
Z_7348284_T	51T	80T
Z_7348285_G	49G	42A_37G_1C
Z_7348286_G	48G	82G
Z_7348287_T	49T	82T
Z_7348288_G	49G	81G
Z_7348289_C	48C	80C
Z_7348290_A	49A	81A
Z_7348291_G	48G	81G
Z_7348292_C	47C	81C
Z_7348293_C	48C	83C
Z_7348294_A	48A	84A
Z_7348295_C	48C	86C
Z_7348296_C	47C	87C
Z_7348297_G	47A	51A_37G
Z_7348298_G	46G	90G
Z_7348299_A	46A	87A
Z_7348300_T	46T	87T
Z_7348301_C	45C	89C
Z_7348302_A	47A	87A
Z_7348303_G	47G	86G
Z_7348304_T	45T	85T
Z_7348305_T	43T	83T
Z_7348306_A	42A	84A
Z_7348307_T	38T	82T
Z_7348308_G	36G	81G

Z_7348309_T	36T	79T
Z_7348310_G	36G	81G
Z_7348311_A	34A	79A
Z_7348312_T	33T	80T
Z_7348313_T	33T	81T
Z_7348314_C	32C	81C
Z_7348315_T	33T	80T
Z_7348316_G	34G	81G
Z_7348317_G	34G	81G
Z_7348318_G	33G	78G
Z_7348319_A	33A	77A
Z_7348320_G	33G	75G
Z_7348321_C	33C	29T_47C
Z_7348322_C	32C	77C
Z_7348323_C	30C	73C
Z_7348324_A	30A	73A
Z_7348325_G	30G	73G
Z_7348326_A	30A	73A
Z_7348327_A	30A	73A
Z_7348328_A	31A	73A
Z_7348329_A	30A	73A
Z_7348330_A	30A	73A
Z_7348331_A	30A	72A
Z_7348332_T	29T	71T
Z_7348333_A	28A	68A
Z_7348334_A	28A	44A_23T
Z_7348335_A	28A	62A
Z_7348336_A	26A	65A
Z_7348337_C	25C	62C
Z_7348338_T	25T	62T
Z_7348339_T	25T	61T
Z_7348340_C	24C	62C
Z_7348341_T	24T	66T
Z_7348342_C	24C	66C
Z_7348343_C	24C	66C
Z_7348344_A	25G	65A
Z_7348345_A	26A	64A
Z_7348346_A	27A	63A
Z_7348347_C	28C	66C
Z_7348348_A	29A	67A
Z_7348349_C	28C	66C
Z_7348350_T	29T	66T
Z_7348351_G	27G	66G

Z_7348352_A	29A	67A
Z_7348353_A	29A	67A
Z_7348354_A	28A	67A
Z_7348355_A	28A	69A
Z_7348356_A	33A	69A
Z_7348357_G	33G	26A_44G
Z_7348358_A	33A	72A
Z_7348359_G	33G	71G
Z_7348360_A	32A	74A
Z_7348361_G	31G	75G
Z_7348362_G	30G	73G
Z_7348363_G	30G	73G
Z_7348364_T	30T	73T
Z_7348365_C	31C	73C
Z_7348366_T	32T	73T
Z_7348367_G	31G	72G
Z_7348368_A	28A	71A
Z_7348369_C	28C	71C
Z_7348370_C	31C	70C
Z_7348371_T	31T	72T
Z_7348372_G	31G	73G
Z_7348373_A	30A	72A
Z_7348374_C	31C	71C
Z_7348375_A	32A	72A
Z_7348376_A	32A	73A
Z_7348377_A	33A	73A
Z_7348378_A	32A	72A
Z_7348379_C	32C	72C
Z_7348380_A	32A	72A
Z_7348381_A	33A	70A
Z_7348382_A	35A	70A
Z_7348383_G	34G	70G
Z_7348384_C	34C	70C
Z_7348385_A	33A	69A
Z_7348386_A	34A	69A
Z_7348387_T	34T	69T
Z_7348388_A	34A	68A
Z_7348389_A	34A	67A
Z_7348390_C	34C	66C
Z_7348391_C	35C	65C
Z_7348392_T	37T	62T
Z_7348393_A	37A	60A
Z_7348394_C	37C	61C

Z_7348395_C	36C	27G_37C
Z_7348396_C	38C	62C
Z_7348397_A	38A	62A
Z_7348398_G	38G	62G
Z_7348399_G	38G	60G
Z_7348400_C	38C	59C
Z_7348401_A	39A	58A
Z_7348402_C	39C	58C
Z_7348403_C	39C	56C
Z_7348404_C	37C	56C
Z_7348405_T	38T	55T
Z_7348406_C	39C	58C
Z_7348407_C	37C	58C
Z_7348408_G	37G	59G
Z_7348409_G	38G	60G
Z_7348410_C	37C	62C
Z_7348411_T	39T	61T
Z_7348412_C	38C	62C
Z_7348413_C	38C	62C
Z_7348414_T	40T	65T
Z_7348415_A	39A	66A
Z_7348416_T	40T	65T
Z_7348417_C	40C	65C
Z_7348418_A	41A	65A
Z_7348419_T	41T	64T
Z_7348420_T	40T	64T
Z_7348421_C	39C	65C
Z_7348422_C	39C	65C
Z_7348423_C	39T	66C
Z_7348424_A	39A	66A
Z_7348425_A	39A	67A
Z_7348426_T	36T	66T
Z_7348427_T	37T	66T
Z_7348428_G	38G	68G
Z_7348429_G	38G	69G
Z_7348430_C	35C	30T_39C
Z_7348431_G	38G	68G
Z_7348432_A	38A	68A
Z_7348433_C	39C	70C
Z_7348434_A	40A	66A
Z_7348435_C	39C	67C
Z_7348436_C	39G	64C
Z_7348437_T	38T	64T

Z_7348438_C	38C	63C
Z_7348439_C	40C	64C
Z_7348440_T	39T	63T
Z_7348441_G	40G	63G
Z_7348442_G	40G	61G
Z_7348443_G	38G	60G
Z_7348444_G	39G	60G
Z_7348445_G	39G	61G
Z_7348446_T	40T	62T
Z_7348447_G	38G	59G
Z_7348448_A	39A	59A
Z_7348449_T	39T	61T
Z_7348450_G	41G	60G
Z_7348451_G	39G	60G
Z_7348452_G	38G	59G
Z_7348453_A	39A	59A
Z_7348454_G	38G	59G
Z_7348455_T	38T	58T
Z_7348456_C	37T	57C
Z_7348457_G	37G	59G
Z_7348458_T	40T	60T
Z_7348459_A	41A	61A
Z_7348460_G	40G	62G
Z_7348461_T	39T	62T
Z_7348462_T	37T	63T
Z_7348463_G	38G	63G
Z_7348464_G	37G	63G
Z_7348465_G	39G	64G
Z_7348466_C	39C	63C
Z_7348467_C	38C	62C
Z_7348468_C	37C	62C
Z_7348469_A	37A	62A
Z_7348470_T	37T	60T
Z_7348471_C	36C	62C
Z_7348472_T	36T	60T
Z_7348473_G	37G	60G
Z_7348474_G	39G	61G
Z_7348475_A	40A	60A
Z_7348476_A	39A	61A
Z_7348477_A	39A	61A
Z_7348478_G	37G	61G
Z_7348479_C	36C	62C
Z_7348480_A	36A	60A

Z_7348481_C	36C	61C
Z_7348482_C	37C	62C
Z_7348483_T	37T	61T
Z_7348484_G	36G	60G
Z_7348485_G	35G	60G
Z_7348486_T	34T	37T_25C
Z_7348487_T	33T	63T
Z_7348488_A	32A	64A
Z_7348489_G	33G	63G
Z_7348490_G	32G	60G
Z_7348491_A	33A	61A
Z_7348492_G	33G	60G
Z_7348493_A	33A	61A
Z_7348494_G	33G	61G
Z_7348495_T	32T	61T
Z_7348496_C	32C	61C
Z_7348497_T	30T	61T
Z_7348498_T	29T	60T
Z_7348499_A	32A	61A
Z_7348500_G	32G	61G
Z_7348501_G	32G	58G
Z_7348502_A	31A	57A
Z_7348503_A	31A	57A
Z_7348504_G	29G	55G
Z_7348505_G	29G	55G
Z_7348506_C	50A_28C	40A_55C
Z_7348507_G	30G	53G
Z_7348508_A	33A	54A
Z_7348509_G	31G	51G
Z_7348510_A	31A	50A
Z_7348511_G	32G	50G
Z_7348512_C	31C	49C
Z_7348513_G	31G	49G
Z_7348514_G	30G	26G_22C
Z_7348515_T	31T	47T
Z_7348516_C	32C	47C
Z_7348517_T	32T	46T
Z_7348518_C	30C	47C
Z_7348519_T	30T	48T
Z_7348520_T	29C	26T_23C
Z_7348521_G	31G	49G
Z_7348522_T	32T	48T
Z_7348523_C	34C	52C

Z_7348524_C	35C	52C
Z_7348525_T	36T	51T
Z_7348526_A	35A	51A
Z_7348527_T	34T	51T
Z_7348528_C	34C	50C
Z_7348529_A	34A	49A
Z_7348530_C	35C	49C
Z_7348531_A	34A	49A
Z_7348532_A	33A	49A
Z_7348533_A	33A	49A
Z_7348534_A	33A	48A
Z_7348535_A	34A	47A
Z_7348536_C	34G	47C
Z_7348537_T	35T	47T
Z_7348538_T	34T	49T
Z_7348539_C	36C	48C
Z_7348540_A	37A	47A
Z_7348541_A	34A	48A
Z_7348542_C	34C	49C
Z_7348543_A	34A	49A
Z_7348544_T	34T	48T
Z_7348545_C	34C	47C
Z_7348546_T	35T	45T
Z_7348547_C	36C	46C
Z_7348548_C	37C	45C
Z_7348549_A	36A	45A
Z_7348550_C	36C	42C
Z_7348551_G	35G	41G
Z_7348552_T	35T	41T
Z_7348553_C	35C	41C
Z_7348554_C	37C	41C
Z_7348555_G	37G	41G
Z_7348556_G	37G	42G
Z_7348557_A	37A	40A
Z_7348558_G	37G	42G
Z_7348559_T	40T	41T
Z_7348560_T	40T	43T
Z_7348561_C	41C	42C
Z_7348562_A	41A	41A
Z_7348563_C	41C	41C
Z_7348564_A	42A	41A
Z_7348565_A	42A	43A
Z_7348566_G	42G	42G

Z_7348567_G	39G	41G
Z_7348568_A	40A	41A
Z_7348569_C	40C	41C
Z_7348570_C	39C	42C
Z_7348571_A	40A	42A
Z_7348572_G	36G	43G
Z_7348573_C	35C	43C
Z_7348574_T	38T	42T
Z_7348575_C	37C	43C
Z_7348576_T	38C	26T_18C
Z_7348577_G	36G	45G
Z_7348578_A	37A	46A
Z_7348579_G	39G	47G
Z_7348580_C	39C	48C
Z_7348581_C	39C	46C
Z_7348582_G	38G	46G
Z_7348583_T	38T	46T
Z_7348584_G	38G	46G
Z_7348585_A	36A	46A
Z_7348586_C	36C	43C
Z_7348587_C	36C	43C
Z_7348588_T	36T	43T
Z_7348589_T	36T	44T
Z_7348590_G	35G	46G
Z_7348591_A	33A	47A
Z_7348592_A	33A	48A
Z_7348593_A	31A	48A
Z_7348594_A	30A	47A
Z_7348595_C	29C	48C
Z_7348596_A	29A	48A
Z_7348597_G	30G	48G
Z_7348598_G	30G	47G
Z_7348599_T	31T	47T
Z_7348600_G	31G	46G
Z_7348601_C	31C	48C
Z_7348602_G	31G	47G
Z_7348603_G	30G	49G
Z_7348604_A	31A	51A
Z_7348605_A	31A	49A
Z_7348606_C	31T	50C
Z_7348607_C	31C	52C
Z_7348608_C	31C	52C
Z_7348609_G	30G	52G

Z_7348610_C	32C	52C
Z_7348611_A	32A	51A
Z_7348612_C	32C	53C
Z_7348613_C	32C	54C
Z_7348614_C	32C	53C
Z_7348615_G	32G	52G
Z_7348616_C	32C	54C
Z_7348617_T	30T	53T
Z_7348618_C	30C	50C
Z_7348619_C	31C	50C
Z_7348620_C	31C	51C
Z_7348621_A	31A	51A
Z_7348622_C	33C	51C
Z_7348623_G	33G	50G
Z_7348624_C	32C	52C
Z_7348625_C	32C	52C
Z_7348626_C	31C	52C
Z_7348627_T	31T	53T
Z_7348628_G	31G	56G
Z_7348629_C	29C	56C
Z_7348630_T	28T	58T
Z_7348631_T	27T	59T
Z_7348632_T	28T	60T
Z_7348633_G	29G	58G
Z_7348634_C	29C	59C
Z_7348635_A	29A	59A
Z_7348636_A	31A	58A
Z_7348637_G	30G	56G
Z_7348638_C	30C	57C
Z_7348639_C	30C	57C
Z_7348640_G	30G	57G
Z_7348641_C	29C	58C
Z_7348642_T	30T	57T
Z_7348643_C	30C	59C
Z_7348644_T	29T	58T
Z_7348645_A	31A	58A
Z_7348646_A	31A	59A
Z_7348647_G	31G	60G
Z_7348648_C	32C	62C
Z_7348649_A	32A	61A
Z_7348650_G	33G	60G
Z_7348651_A	31A	60A
Z_7348652_C	34C	60C

Z_7348653_T	34T	59T
Z_7348654_G	35G	59G
Z_7348655_C	35C	57C
Z_7348656_T	36T	57T
Z_7348657_T	37T	57T
Z_7348658_T	37T	58T
Z_7348659_C	35C	58C
Z_7348660_T	35T	57T
Z_7348661_C	34C	58C
Z_7348662_A	35A	60A
Z_7348663_G	35G	60G
Z_7348664_T	37T	60T
Z_7348665_G	37G	59G
Z_7348666_G	37G	61G
Z_7348667_C	37C	60C
Z_7348668_T	36T	60T
Z_7348669_T	36T	60T
Z_7348670_T	35T	59T
Z_7348671_G	36G	59G
Z_7348672_C	35C	59C
Z_7348673_C	36C	58C
Z_7348674_A	36A	56A
Z_7348675_C	36C	56C
Z_7348676_G	38G	58G
Z_7348677_G	39G	57G
Z_7348678_G	40G	57G
Z_7348679_A	43A	57A
Z_7348680_A	42A	57A
Z_7348681_A	42A	57A
Z_7348682_G	42G	57G
Z_7348683_G	39G	58G
Z_7348684_A	41A	57A
Z_7348685_G	40G	56G
Z_7348686_G	42G	56G
Z_7348687_A	43A	55A
Z_7348688_C	45C	55C
Z_7348689_A	45A	55A
Z_7348690_A	43A	53A
Z_7348691_A	44A	52A
Z_7348692_A	42A	53A
Z_7348693_A	44A	54A
Z_7348694_C	43C	54C
Z_7348695_A	43A	55A

Z_7348696_G	42G	53G
Z_7348697_C	42C	53C
Z_7348698_A	44A	53A
Z_7348699_G	42G	52G
Z_7348700_A	46A	54A
Z_7348701_T	47T	55T
Z_7348702_T	49T	53T
Z_7348703_C	48C	53C
Z_7348704_C	47C	55C
Z_7348705_G	46G	56G
Z_7348706_T	47T	54T
Z_7348707_C	47C	52C
Z_7348708_T	48T	51T
Z_7348709_G	49G	51G
Z_7348710_G	49G	52G
Z_7348711_A	48A	50A
Z_7348712_G	48G	52G
Z_7348713_G	48G	52G
Z_7348714_T	49T	53T
Z_7348715_A	49A	52A
Z_7348716_T	49T	52T
Z_7348717_G	49G	53G
Z_7348718_A	48A	54A
Z_7348719_A	48A	53A
Z_7348720_A	47A	53A
Z_7348721_T	47T	54T
Z_7348722_G	46G	54G
Z_7348723_G	48G	51G
Z_7348724_T	45T	51T
Z_7348725_G	46G	24A_25G
Z_7348726_G	43G	49G
Z_7348727_T	46T	48T
Z_7348728_T	47T	48T
Z_7348729_A	48A	47A
Z_7348730_T	50T	48T
Z_7348731_T	49T	48T
Z_7348732_G	51G	48G
Z_7348733_A	51A	46A
Z_7348734_T	49T	47T
Z_7348735_A	49A	47A
Z_7348736_A	48A	47A
Z_7348737_G	48G	46G
Z_7348738_A	46A	45A

Z_7348739_A	48A	45A
Z_7348740_A	48A	45A
Z_7348741_T	47T	45T
Z_7348742_A	48A	45A
Z_7348743_T	48T	43T
Z_7348744_T	48T	45T
Z_7348745_G	45G	45G
Z_7348746_T	45T	44T
Z_7348747_G	42G	43G
Z_7348748_C	41C	43C
Z_7348749_T	40T	43T
Z_7348750_G	39G	45G
Z_7348751_G	41G	45G
Z_7348752_G	41G	45G
Z_7348753_T	42T	45T
Z_7348754_G	42G	49G
Z_7348755_G	42G	49G
Z_7348756_C	40C	48C
Z_7348757_A	39A	46A
Z_7348758_G	38G	47G
Z_7348759_A	38A	48A
Z_7348760_G	37G	49G
Z_7348761_G	36G	49G
Z_7348762_C	38C	48C
Z_7348763_T	38T	50T
Z_7348764_G	38G	50G
Z_7348765_G	38G	50G
Z_7348766_A	39A	49A
Z_7348767_A	38A	49A
Z_7348768_A	38A	51A
Z_7348769_T	37T	51T
Z_7348770_A	35A	51A
Z_7348771_A	34A	49A
Z_7348772_A	32A	49A
Z_7348773_G	31G	51G
Z_7348774_A	33A	52A
Z_7348775_T	31T	51T
Z_7348776_C	33C	53C
Z_7348777_G	32G	54G
Z_7348778_G	34G	54G
Z_7348779_G	34G	54G
Z_7348780_A	34A	53A
Z_7348781_A	35A	54A

Z_7348782_A	35A	55A
Z_7348783_A	36A	55A
Z_7348784_G	34G	55G
Z_7348785_C	32C	55C
Z_7348786_A	31A	57A
Z_7348787_T	31T	54T
Z_7348788_C	31C	54C
Z_7348789_A	30A	55A
Z_7348790_G	30G	54G
Z_7348791_C	30C	55C
Z_7348792_C	28C	55C
Z_7348793_A	27A	54A
Z_7348794_C	29C	53C
Z_7348795_T	29C	50T
Z_7348796_G	30G	50G
Z_7348797_C	30C	50C
Z_7348798_G	30G	49G
Z_7348799_T	29T	48T
Z_7348800_T	27T	49T
Z_7348801_T	26C	49T
Z_7348802_G	25G	49G
Z_7348803_G	27G	49G
Z_7348804_G	27G	49G
Z_7348805_G	27G	46G
Z_7348806_C	27T	48C
Z_7348807_C	27C	47C
Z_7348808_C	27C	47C
Z_7348809_G	27A	46G
Z_7348810_A	33A	46A
Z_7348811_A	33A	46A
Z_7348812_G	34G	45G
Z_7348813_A	33A	46A
Z_7348814_G	33G	45G
Z_7348815_G	33G	45G
Z_7348816_T	33T	45T
Z_7348817_C	33C	45C
Z_7348818_A	32A	49A
Z_7348819_C	32C	49C
Z_7348820_C	31C	49C
Z_7348821_A	30C	48A
Z_7348822_C	28C	49C
Z_7348823_G	30G	50G
Z_7348824_T	29T	50T

Z_7348825_C	31C	51C
Z_7348826_T	30T	51T
Z_7348827_G	31G	52G
Z_7348828_A	29A	52A
Z_7348829_C	30C	54C
Z_7348830_C	29C	54C
Z_7348831_C	29C	53C
Z_7348832_C	28C	54C
Z_7348833_A	30A	55A
Z_7348834_G	30G	56G
Z_7348835_G	30G	56G
Z_7348836_C	29C	55C
Z_7348837_T	29T	55T
Z_7348838_G	29G	55G
Z_7348839_A	29A	53A
Z_7348840_A	24G	53A
Z_7348841_G	23G	54G
Z_7348842_C	23C	55C
Z_7348843_T	22T	55T
Z_7348844_C	21T	57C
Z_7348845_G	22G	57G
Z_7348846_C	22C	57C
Z_7348847_T	23T	57T
Z_7348848_A	23A	55A
Z_7348849_G	23G	53G
Z_7348850_A	23A	52A
Z_7348851_G	24G	52G
Z_7348852_G	23G	52G
Z_7348853_A	22A	51A
Z_7348854_A	22A	50A
Z_7348855_T	20T	49T
Z_7348856_C	22C	48C
Z_7348857_T	22T	47T
Z_7348858_G	23G	45G
Z_7348859_C	22C	43C
Z_7348860_C	21C	45C
Z_7348861_C	23C	46C
Z_7348862_C	23C	45C
Z_7348863_C	22C	43C
Z_7348864_C	21C	45C
Z_7348865_A	21A	41A
Z_7348866_C	21C	44C
Z_7348867_C	20C	44C

Z_7348868_C	19C	43C
Z_7348869_C	20C	43C
Z_7348870_T	21T	43T
Z_7348871_C	23C	44C
Z_7348872_T	24T	45T
Z_7348873_G	22G	44G
Z_7348874_G	22G	45G
Z_7348875_C	22C	46C
Z_7348876_C	22C	46C
Z_7348877_G	21G	46G
Z_7348878_A	20A	45A
Z_7348879_T	20T	47T
Z_7348880_A	21A	47A
Z_7348881_G	21G	45G
Z_7348882_C	19C	46C
Z_7348883_C	20C	47C
Z_7348884_C	21C	48C
Z_7348885_A	22A	49A
Z_7348886_G	22G	50G
Z_7348887_C	21C	51C
Z_7348888_T	22T	50T
Z_7348889_G	23G	52G
Z_7348890_A	23A	51A
Z_7348891_G	23G	53G
Z_7348892_C	22C	52C
Z_7348893_C	22C	52C
Z_7348894_C	24C	54C
Z_7348895_T	25T	52T
Z_7348896_T	26T	52T
Z_7348897_T	27T	52T
Z_7348898_C	27C	53C
Z_7348899_T	30T	54T
Z_7348900_G	30G	56G
Z_7348901_T	29T	56T
Z_7348902_T	28T	55T
Z_7348903_C	27C	57C
Z_7348904_A	28A	57A
Z_7348905_G	28G	58G
Z_7348906_C	28C	58C
Z_7348907_C	27C	58C
Z_7348908_A	27A	56A
Z_7348909_G	28G	57G
Z_7348910_G	28G	57G

Z_7348911_C	28C	59C
Z_7348912_C	28C	58C
Z_7348913_A	28A	54A
Z_7348914_A	29A	54A
Z_7348915_T	29T	55T
Z_7348916_A	29A	55A
Z_7348917_C	29C	55C
Z_7348918_G	30G	54G
Z_7348919_T	30T	52T
Z_7348920_T	30T	51T
Z_7348921_A	29A	51A
Z_7348922_C	29C	50C
Z_7348923_T	33T	50T
Z_7348924_C	33C	49C
Z_7348925_C	32C	51C
Z_7348926_C	34C	51C
Z_7348927_A	35A	50A
Z_7348928_T	34T	51T
Z_7348929_T	33T	51T
Z_7348930_G	33G	51G
Z_7348931_A	33A	52A
Z_7348932_T	33T	51T
Z_7348933_T	33T	51T
Z_7348934_G	33G	52G
Z_7348935_C	33C	53C
Z_7348936_C	31C	52C
Z_7348937_A	33A	51A
Z_7348938_G	33G	51G
Z_7348939_T	31T	49T
Z_7348940_T	32T	47T
Z_7348941_G	31G	47G
Z_7348942_C	31C	47C
Z_7348943_C	33C	47C
Z_7348944_A	33A	48A
Z_7348945_C	34C	49C
Z_7348946_C	35C	49C
Z_7348947_C	34C	49C
Z_7348948_C	34C	49C
Z_7348949_C	35C	49C
Z_7348950_A	34A	49A
Z_7348951_C	35C	49C
Z_7348952_T	35T	49T
Z_7348953_G	36G	48G

Z_7348954_G	35G	48G
Z_7348955_T	34T	48T
Z_7348956_T	34T	47T
Z_7348957_T	33T	48T
Z_7348958_A	33A	48A
Z_7348959_T	32T	48T
Z_7348960_A	32A	48A
Z_7348961_T	32T	47T
Z_7348962_T	32T	47T
Z_7348963_G	30G	48G
Z_7348964_T	30T	49T
Z_7348965_C	29C	48C
Z_7348966_G	28G	48G
Z_7348967_C	28C	47C
Z_7348968_T	28T	48T
Z_7348969_C	26C	46C
Z_7348970_T	26T	48T
Z_7348971_C	26C	53C
Z_7348972_A	26G	53A
Z_7348973_A	27A	52A
Z_7348974_T	27T	52T
Z_7348975_G	27G	52G
Z_7348976_T	27T	52T
Z_7348977_T	27T	52T
Z_7348978_T	27T	51T
Z_7348979_T	25T	50T
Z_7348980_C	26A	24A_24C
Z_7348981_G	26G	49G
Z_7348982_T	25T	48T
Z_7348983_G	25G	48G
Z_7348984_G	24G	48G
Z_7348985_G	24G	48G
Z_7348986_A	24A	47A
Z_7348987_C	23C	48C
Z_7348988_A	22A	48A
Z_7348989_A	22A	47A
Z_7348990_T	22T	47T
Z_7348991_A	22A	48A
Z_7348992_T	22T	48T
Z_7348993_A	18A	49A
Z_7348994_A	18A	48A
Z_7348995_A	18A	47A
Z_7348996_A	42A_30C	49A

Z_7348997_C	11A	65A_111C
Z_7348998_A	11A	33A
Z_7348999_C	11C	30C
Z_7349000_A	11A	35A
Z_7349001_C	11C	36C
Z_7349002_A	11A	36A
Z_7349003_C	11C	36C
Z_7349004_A	11A	35A
Z_7349005_C	11C	34C
Z_7349006_A	11A	36A
Z_7349007_C	10C	35C
Z_7349008_A	15A	48A
Z_7349009_C	5A_10C	13A_36C
Z_7349010_A	23A	51A
Z_7349011_C	22C	50C
Z_7349012_A	21A	50A
Z_7349013_C	20C	50C
Z_7349014_A	20A	52A
Z_7349015_C	23C	53C
Z_7349016_A	19A	54A
Z_7349017_C	20C	54C
Z_7349018_A	20A	53A
Z_7349019_C	20C	54C
Z_7349020_A	21A	53A
Z_7349021_C	21C	55C
Z_7349022_A	22A	55A
Z_7349023_C	22C	55C
Z_7349024_A	23A	34A_21C
Z_7349025_C	23C	56C
Z_7349026_A	23A	56A
Z_7349027_C	23C	56C
Z_7349028_G	24G	57G
Z_7349029_G	27G	58G
Z_7349030_C	27C	58C
Z_7349031_A	28A	57A
Z_7349032_G	29G	59G
Z_7349033_G	28G	60G
Z_7349034_A	29A	60A
Z_7349035_G	29G	59G
Z_7349036_T	29T	59T
Z_7349037_T	28T	59T
Z_7349038_C	29C	60C
Z_7349039_A	30A	33A_27C

Z_7349040_T	31T	60T
Z_7349041_T	32T	59T
Z_7349042_T	32T	60T
Z_7349043_A	31A	60A
Z_7349044_A	32A	60A
Z_7349045_C	31T	60C
Z_7349046_T	32T	60T
Z_7349047_C	32C	62C
Z_7349048_A	32A	63A
Z_7349049_T	34T	65T
Z_7349050_T	34T	66T
Z_7349051_C	34C	66C
Z_7349052_T	33T	66T
Z_7349053_C	32C	65C
Z_7349054_G	33G	65G
Z_7349055_G	33G	65G
Z_7349056_C	33C	65C
Z_7349057_A	33A	64A
Z_7349058_G	32G	67G
Z_7349059_G	32G	66G
Z_7349060_G	31G	68G
Z_7349061_C	33C	67C
Z_7349062_C	33C	67C
Z_7349063_C	33C	65C
Z_7349064_T	30T	65T
Z_7349065_T	31T	62T
Z_7349066_T	31T	58T
Z_7349067_T	32T	57T
Z_7349068_A	32A	57A
Z_7349069_C	32C	57C
Z_7349070_C	30T	29T_28C
Z_7349071_G	30G	58G
Z_7349072_T	29C	28T_30C
Z_7349073_G	34G	59G
Z_7349074_C	33C	61C
Z_7349075_A	35A	62A
Z_7349076_A	34A	61A
Z_7349077_A	35A	62A
Z_7349078_G	35G	62G
Z_7349079_C	35C	61C
Z_7349080_A	37A	62A
Z_7349081_G	39G	63G
Z_7349082_G	38G	64G

Z_7349083_A	39A	65A
Z_7349084_G	38G	64G
Z_7349085_G	35G	65G
Z_7349086_A	36A	64A
Z_7349087_T	35T	62T
Z_7349088_G	35G	63G
Z_7349089_G	35G	65G
Z_7349090_G	35G	64G
Z_7349091_T	34T	62T
Z_7349092_T	34T	61T
Z_7349093_G	33G	60G
Z_7349094_G	34G	60G
Z_7349095_A	35A	59A
Z_7349096_A	35A	59A
Z_7349097_A	35A	57A
Z_7349098_G	34G	57G
Z_7349099_G	33G	59G
Z_7349100_A	33A	59A
Z_7349101_A	32A	59A
Z_7349102_G	32G	60G
Z_7349103_A	34A	61A
Z_7349104_G	34G	60G
Z_7349105_A	33A	60A
Z_7349106_A	34A	60A
Z_7349107_A	34A	62A
Z_7349108_A	34A	60A
Z_7349109_A	33A	59A
Z_7349110_G	31G	57G
Z_7349111_G	30G	56G
Z_7349112_A	30A	61A
Z_7349113_C	30C	61C
Z_7349114_G	29G	61G
Z_7349115_A	31A	61A
Z_7349116_A	31A	63A
Z_7349117_A	31A	63A
Z_7349118_C	34C	63C
Z_7349119_A	33A	64A
Z_7349120_A	33A	63A
Z_7349121_G	33G	64G
Z_7349122_T	33T	64T
Z_7349123_C	33C	64C
Z_7349124_T	33T	62T
Z_7349125_T	32T	62T

Z_7349126_T	33T	62T
Z_7349127_G	34G	60G
Z_7349128_C	33C	64C
Z_7349129_C	32C	64C
Z_7349130_A	35A	66A
Z_7349131_A	34A	65A
Z_7349132_G	34G	64G
Z_7349133_G	34G	63G
Z_7349134_A	34A	65A
Z_7349135_A	35A	65A
Z_7349136_A	33A	64A_54T
Z_7349137_T	35T	63T
Z_7349138_T	34T	65T
Z_7349139_T	30T	65T
Z_7349140_T	33T	65T
Z_7349141_A	33A	35A_30T
Z_7349142_A	33A	65A
Z_7349143_A	33A	66A
Z_7349144_A	34A	65A
Z_7349145_A	33A	65A
Z_7349146_A	32A	68A
Z_7349147_C	33C	71C
Z_7349148_A	33A	71A
Z_7349149_C	33C	72C
Z_7349150_T	33T	73T
Z_7349151_C	32C	72C
Z_7349152_C	33C	72C
Z_7349153_T	33T	67T
Z_7349154_C	33C	69C
Z_7349155_T	35T	69T
Z_7349156_T	34T	70T
Z_7349157_T	34T	70T
Z_7349158_A	34A	71A
Z_7349159_T	35T	72T
Z_7349160_A	35A	72A
Z_7349161_A	35A	71A
Z_7349162_G	36G	72G
Z_7349163_G	38G	73G
Z_7349164_T	37T	72T
Z_7349165_C	38C	73C
Z_7349166_A	39A	76A
Z_7349167_A	39A	78A
Z_7349168_G	41G	78G

Z_7349169_A	41A	75A
Z_7349170_G	41G	76G
Z_7349171_A	41A	75A
Z_7349172_T	41T	76T
Z_7349173_G	39G	77G
Z_7349174_G	40G	76G
Z_7349175_C	40C	74C
Z_7349176_C	40C	73C
Z_7349177_A	41A	73A
Z_7349178_C	40C	73C
Z_7349179_A	40A	73A
Z_7349180_C	41C	72C
Z_7349181_C	43C	72C
Z_7349182_T	44T	71T
Z_7349183_T	44T	73T
Z_7349184_T	45T	71T
Z_7349185_G	43G	74G
Z_7349186_T	43T	73T
Z_7349187_G	43G	74G
Z_7349188_G	39G	78G
Z_7349189_C	41C	78C
Z_7349190_T	41T	79T
Z_7349191_G	41G	80G
Z_7349192_A	42A	81A
Z_7349193_T	42T	83T
Z_7349194_G	42G	80G
Z_7349195_G	40G	47G_33C
Z_7349196_G	41G	79G
Z_7349197_A	42A	78A
Z_7349198_T	42T	82T
Z_7349199_T	42T	82T
Z_7349200_T	41T	80T
Z_7349201_G	41G	81G
Z_7349202_C	41C	82C
Z_7349203_T	43T	82T
Z_7349204_C	42C	83C
Z_7349205_C	43C	82C
Z_7349206_C	43C	83C
Z_7349207_A	42A	81A
Z_7349208_C	43C	82C
Z_7349209_C	43C	80C
Z_7349210_G	42G	81G
Z_7349211_G	41G	80G

Z_7349212_A	41A	79A
Z_7349213_T	40T	79T
Z_7349214_C	40C	78C
Z_7349215_G	41G	80G
Z_7349216_A	42A	80A
Z_7349217_G	41G	81G
Z_7349218_G	42G	80G
Z_7349219_G	42G	80G
Z_7349220_A	39A	82A
Z_7349221_G	40G	77G
Z_7349222_G	40G	81G
Z_7349223_G	40G	80G
Z_7349224_C	40C	80C
Z_7349225_T	38T	82T
Z_7349226_T	38T	82T
Z_7349227_T	38T	84T
Z_7349228_C	37C	83C
Z_7349229_T	37T	81T
Z_7349230_A	37A	80A
Z_7349231_C	37C	83C
Z_7349232_G	36G	83G
Z_7349233_G	34G	81G
Z_7349234_C	34C	82C
Z_7349235_T	33T	81T
Z_7349236_T	32T	81T
Z_7349237_C	31C	81C
Z_7349238_C	31C	79C
Z_7349239_A	31A	81A
Z_7349240_A	31A	81A
Z_7349241_A	31A	76A
Z_7349242_G	29G	75G
Z_7349243_G	30G	76G
Z_7349244_G	29G	76G
Z_7349245_G	29G	75G
Z_7349246_A	28A	75A
Z_7349247_A	28A	77A
Z_7349248_C	31C	77C
Z_7349249_A	31A	78A
Z_7349250_G	30A	78G
Z_7349251_G	29G	77G
Z_7349252_C	28C	78C
Z_7349253_C	30C	80C
Z_7349254_C	29C	81C

Z_7349255_C	29C	80C
Z_7349256_A	29A	81A
Z_7349257_T	28T	80T
Z_7349258_G	28G	80G
Z_7349259_A	26A	80A
Z_7349260_T	27T	81T
Z_7349261_G	27G	78G
Z_7349262_G	26G	75G
Z_7349263_C	27C	75C
Z_7349264_A	27A	74A
Z_7349265_G	28G	76G
Z_7349266_G	27G	76G
Z_7349267_T	27T	76T
Z_7349268_G	27G	75G
Z_7349269_A	26A	76A
Z_7349270_A	26A	76A
Z_7349271_G	27G	77G
Z_7349272_C	27C	77C
Z_7349273_G	26G	77G
Z_7349274_T	26T	75T
Z_7349275_T	25T	77T
Z_7349276_G	25G	76G
Z_7349277_A	25A	74A
Z_7349278_T	24T	74T
Z_7349279_G	25G	74G
Z_7349280_G	26G	73G
Z_7349281_A	26A	76A
Z_7349282_T	26C	40T_36C
Z_7349283_G	27G	75G
Z_7349284_C	26C	75C
Z_7349285_T	26T	74T
Z_7349286_C	25C	75C
Z_7349287_C	26C	74C
Z_7349288_A	25A	72A
Z_7349289_T	24T	72T
Z_7349290_G	24G	73G
Z_7349291_A	23C	37A_32C
Z_7349292_G	24G	32A_38G
Z_7349293_C	28C	69C
Z_7349294_A	29A	69A
Z_7349295_G	29G	69G
Z_7349296_A	30A	34A_35G
Z_7349297_C	32C	74C

Z_7349298_T	32T	75T
Z_7349299_G	32G	76G
Z_7349300_A	32A	76A
Z_7349301_G	32G	77G
Z_7349302_T	32T	76T
Z_7349303_T	31T	78T
Z_7349304_G	32G	77G
Z_7349305_G	32A	77G
Z_7349306_T	34T	77T
Z_7349307_T	35T	79T
Z_7349308_G	33G	79G
Z_7349309_C	33C	81C
Z_7349310_T	32T	79T
Z_7349311_G	34G	78G
Z_7349312_A	33A	79A
Z_7349313_C	35C	80C
Z_7349314_T	36T	82T
Z_7349315_G	37G	80G
Z_7349316_C	35C	81C
Z_7349317_T	36T	80T
Z_7349318_A	34A	80A
Z_7349319_G	34G	79G
Z_7349320_G	34G	77G
Z_7349321_A	33A	77A
Z_7349322_A	33A	78A_28C
Z_7349323_C	31C	50C
Z_7349324_A	31A	74A
Z_7349325_C	31C	74C
Z_7349326_A	31A	75A
Z_7349327_C	31C	75C
Z_7349328_A	31A	74A
Z_7349329_C	30C	74C
Z_7349330_A	31A	73A
Z_7349331_C	31C	74C
Z_7349332_A	32A	73A
Z_7349333_C	32C	73C
Z_7349334_A	33A	72A
Z_7349335_C	31C	73C
Z_7349336_A	32A	75A
Z_7349337_C	33C	77C
Z_7349338_A	34A	76A
Z_7349339_C	35C	75C
Z_7349340_A	35A	75A

Z_7349341_C	35C	76C
Z_7349342_A	35A	77A
Z_7349343_C	10A_1T_42C	78C
Z_7349344_A	33A	76A
Z_7349345_C	33C	77C
Z_7349346_A	33A	82A
Z_7349347_C	32C	74C
Z_7349348_A	33A	171A_15T_77C
Z_7349349_C	33C	52C
Z_7349350_A	26A	58A
Z_7349351_C	42A_7T_47C	50C
Z_7349352_A	11A	52A
Z_7349353_C	11C	52C
Z_7349354_A	11A	53A
Z_7349355_C	11C	51C
Z_7349356_A	11A	51A
Z_7349357_C	11C	49C
Z_7349358_A	11A	69A
Z_7349359_A	20A_10T_11C	36A
Z_7349360_A	1A	39A
Z_7349361_T	1T	36T
Z_7349362_C	1C	70C
Z_7349363_T	22T	72T
Z_7349364_C	23C	72C
Z_7349365_T	23T	73T
Z_7349366_C	26C	75C
Z_7349367_T	27T	75T
Z_7349368_T	25T	74T
Z_7349369_T	26T	74T
Z_7349370_T	26T	76T
Z_7349371_C	25C	77C
Z_7349372_T	26T	77T
Z_7349373_A	26A	77A
Z_7349374_G	27G	78G
Z_7349375_A	27A	80A
Z_7349376_C	28C	80C
Z_7349377_T	27T	80T
Z_7349378_T	29T	82T
Z_7349379_A	31A	80A
Z_7349380_A	31A	78A
Z_7349381_A	32A	79A
Z_7349382_A	32A	79A
Z_7349383_C	32C	81C

Z_7349384_C	34T	80C
Z_7349385_G	36G	79G
Z_7349386_T	35T	79T
Z_7349387_A	37A	78A
Z_7349388_G	36G	81G
Z_7349389_A	37A	78A
Z_7349390_A	38A	75A
Z_7349391_A	39A	76A
Z_7349392_A	39A	77A
Z_7349393_A	38A	77A
Z_7349394_A	37A	77A
Z_7349395_T	36T	78T
Z_7349396_G	35G	77G
Z_7349397_T	36T	79T
Z_7349398_A	36A	80A
Z_7349399_A	37A	78A
Z_7349400_T	37T	78T
Z_7349401_C	40C	77C
Z_7349402_T	41C	77T
Z_7349403_A	43A	77A
Z_7349404_G	41G	76G
Z_7349405_G	41G	77G
Z_7349406_A	42A	78A
Z_7349407_G	41G	83G
Z_7349408_C	41C	83C
Z_7349409_C	42C	85C
Z_7349410_C	40C	84C
Z_7349411_C	40C	83C
Z_7349412_A	40A	80A
Z_7349413_T	39T	78T
Z_7349414_T	40T	79T
Z_7349415_C	38C	77C
Z_7349416_C	40C	78C
Z_7349417_G	40G	76G
Z_7349418_A	40A	77A
Z_7349419_A	40A	79A
Z_7349420_C	39C	78C
Z_7349421_C	43C	79C
Z_7349422_C	42C	81C
Z_7349423_G	45G	81G
Z_7349424_T	43T	82T
Z_7349425_G	43G	84G
Z_7349426_G	43G	85G

Z_7349427_C	45C	84C
Z_7349428_A	46A	83A
Z_7349429_A	47A	84A
Z_7349430_T	47T	86T
Z_7349431_T	49T	86T
Z_7349432_T	50T	87T
Z_7349433_C	49C	87C
Z_7349434_C	48C	87C
Z_7349435_G	47G	86G
Z_7349436_A	47A	85A
Z_7349437_A	48A	86A
Z_7349438_C	48C	86C
Z_7349439_C	50C	85C
Z_7349440_A	50A	86A
Z_7349441_C	51C	87C
Z_7349442_C	50C	86C
Z_7349443_T	51T	87T
Z_7349444_T	50T	88T
Z_7349445_C	52C	88C
Z_7349446_A	51A	87A
Z_7349447_A	51A	88A
Z_7349448_G	49G	89G
Z_7349449_G	48G	88G
Z_7349450_G	47G	88G
Z_7349451_C	45C	89C
Z_7349452_A	46A	89A
Z_7349453_G	44G	87G
Z_7349454_C	41C	88C
Z_7349455_C	41C	89C
Z_7349456_C	41C	89C
Z_7349457_C	42C	88C
Z_7349458_A	41A	86A
Z_7349459_G	41G	86G
Z_7349460_A	38G	147A_66G_32C
Z_7349461_G	39G	51G
Z_7349462_G	39G	51G
Z_7349463_A	37A	52A
Z_7349464_C	39C	53C
Z_7349465_A	40A	50A
Z_7349466_G	40G	82G
Z_7349467_G	39G	84G
Z_7349468_G	38G	85G
Z_7349469_A	37A	84A

Z_7349470_C	36C	83C
Z_7349471_G	33G	85G
Z_7349472_G	32G	85G
Z_7349473_C	32C	83C
Z_7349474_T	33T	82T
Z_7349475_C	34C	83C
Z_7349476_C	33C	82C
Z_7349477_A	35G	47A_36G_1C
Z_7349478_T	34T	83T
Z_7349479_T	34T	83T
Z_7349480_A	35A	83A
Z_7349481_C	34C	83C
Z_7349482_A	35A	83A
Z_7349483_G	35G	82G
Z_7349484_T	36T	82T
Z_7349485_A	36A	82A
Z_7349486_G	35G	83G
Z_7349487_T	36T	81T
Z_7349488_C	37C	81C
Z_7349489_T	37T	81T
Z_7349490_A	36A	79A
Z_7349491_T	35T	79T
Z_7349492_G	36G	77G
Z_7349493_C	34C	76C
Z_7349494_T	34T	76T
Z_7349495_C	32C	77C
Z_7349496_G	35G	76G
Z_7349497_G	36G	76G
Z_7349498_G	34G	77G
Z_7349499_G	34G	77G
Z_7349500_C	34C	78C
Z_7349501_T	34T	83T
Z_7349502_T	33T	82T
Z_7349503_G	33G	83G
Z_7349504_A	34A	82A
Z_7349505_A	34A	82A
Z_7349506_T	33T	83T
Z_7349507_C	31C	83C
Z_7349508_A	31A	79A
Z_7349509_G	30G	79G
Z_7349510_G	30G	77G
Z_7349511_G	29G	76G
Z_7349512_A	28A	76A

Z_7349513_G	29G	74G
Z_7349514_G	29G	72G
Z_7349515_T	29T	71T
Z_7349516_G	29G	70G
Z_7349517_G	28G	68G
Z_7349518_A	29A	67A
Z_7349519_A	29A	67A
Z_7349520_C	29C	68C
Z_7349521_T	29T	68T
Z_7349522_C	29C	68C
Z_7349523_T	28T	66T
Z_7349524_C	28C	65C
Z_7349525_T	29T	63T
Z_7349526_A	29G	37A_24G
Z_7349527_T	28T	59T
Z_7349528_T	28T	59T
Z_7349529_C	29C	60C
Z_7349530_A	31A	61A
Z_7349531_A	30A	60A
Z_7349532_A	30A	61A
Z_7349533_G	29C	36G_25C
Z_7349534_G	28G	61G
Z_7349535_A	28A	60A
Z_7349536_C	27C	59C
Z_7349537_C	27C	58C
Z_7349538_A	28A	56A
Z_7349539_G	28G	55G
Z_7349540_C	27C	56C
Z_7349541_T	28T	55T
Z_7349542_T	27T	52T
Z_7349543_C	26C	53C
Z_7349544_A	26A	53A
Z_7349545_C	26C	53C
Z_7349546_A	26A	53A
Z_7349547_A	25A	52A
Z_7349548_G	25G	52G
Z_7349549_A	25A	51A
Z_7349550_G	23G	50G
Z_7349551_G	24G	50G
Z_7349552_C	23C	50C
Z_7349553_G	22G	50G
Z_7349554_G	21G	50G
Z_7349555_C	15G	17G_29C

Z_7349556_T	15T	47T
Z_7349557_T	null	16A_46T_64C
Z_7349558_C	null	30C
Z_7349559_A	null	28A
Z_7349560_C	null	30C
Z_7349561_C	null	31C
Z_7349562_C	null	31C
Z_7349563_C	20C	49C
Z_7349564_A	21A	52A
Z_7349565_G	21G	52G
Z_7349566_A	22A	51A
Z_7349567_T	23T	51T
Z_7349568_T	24T	33T_19C
Z_7349569_G	25G	54G
Z_7349570_A	25A	52A
Z_7349571_A	26A	53A
Z_7349572_C	26C	53C
Z_7349573_G	27G	53G
Z_7349574_G	26G	53G
Z_7349575_G	26G	53G
Z_7349576_T	23T	53T
Z_7349577_C	23C	53C
Z_7349578_A	23A	52A
Z_7349579_G	23G	51G
Z_7349580_T	21T	50T
Z_7349581_C	21C	48C
Z_7349582_C	22C	48C
Z_7349583_C	22C	48C
Z_7349584_T	21T	47T
Z_7349585_C	23C	47C
Z_7349586_T	22T	48T
Z_7349587_C	23C	49C
Z_7349588_A	23A	49A
Z_7349589_C	23C	50C
Z_7349590_C	22C	50C
Z_7349591_C	21C	51C
Z_7349592_A	21A	50A
Z_7349593_G	21G	49G
Z_7349594_A	20A	49A
Z_7349595_C	20C	50C
Z_7349596_C	20C	47C
Z_7349597_C	19C	47C
Z_7349598_C	20C	47C

Z_7349599_A	20A	47A
Z_7349600_C	19C	46C
Z_7349601_G	20G	44G
Z_7349602_C	20C	41C
Z_7349603_C	20C	41C
Z_7349604_A	19A	40A
Z_7349605_A	16A	40A
Z_7349606_C	null	40C
Z_7349607_G	null	40G
Z_7349608_G	null	40G
Z_7349609_C	null	40C
Z_7349610_T	null	41T
Z_7349611_G	null	30G
Z_7349612_C	null	31C
Z_7349613_C	null	31C
Z_7349614_C	null	31C
Z_7349615_C	null	30C
Z_7349616_C	null	30C
Z_7349617_C	null	30C
Z_7349618_A	null	28A
Z_7349619_G	null	30G
Z_7349620_C	null	30C
Z_7349621_G	null	30G
Z_7349622_G	null	31G
Z_7349623_T	null	29T
Z_7349624_T	null	32T
Z_7349625_C	null	32C
Z_7349626_T	null	32T
Z_7349627_G	null	33G
Z_7349628_C	null	33C
Z_7349629_A	null	33A
Z_7349630_T	null	34T
Z_7349631_T	null	34T
Z_7349632_C	null	35C
Z_7349633_T	null	36T
Z_7349634_A	null	35A
Z_7349635_G	null	35G
Z_7349636_C	null	36C
Z_7349637_T	null	37T
Z_7349638_C	null	37C
Z_7349639_T	null	38T
Z_7349640_G	null	37G
Z_7349641_C	null	36C

Z_7349642_C	null	35C
Z_7349643_C	null	34C
Z_7349644_G	null	35G
Z_7349645_C	null	35C
Z_7349646_C	null	36C
Z_7349647_T	null	34T
Z_7349648_C	null	36C
Z_7349649_C	null	37C
Z_7349650_C	null	37C
Z_7349651_C	null	37C
Z_7349652_C	null	37C
Z_7349653_T	null	34T
Z_7349654_C	null	36C
Z_7349655_C	null	35C
Z_7349656_T	null	32T
Z_7349657_C	null	34C
Z_7349658_A	null	34A
Z_7349659_A	null	31A
Z_7349660_G	null	31G
Z_7349661_G	null	30G
Z_7349662_A	null	31A
Z_7349663_G	null	30G
Z_7349664_A	null	29A
Z_7349665_G	17G	40G
Z_7349666_C	17C	42C
Z_7349667_T	17T	43T
Z_7349668_A	17A	45A
Z_7349669_G	17G	45G
Z_7349670_A	17A	45A
Z_7349671_A	17A	48A
Z_7349672_G	17G	49G
Z_7349673_G	17G	49G
Z_7349674_A	17A	49A
Z_7349675_G	17G	50G
Z_7349676_A	17A	51A
Z_7349677_G	17G	52G
Z_7349678_G	17G	52G
Z_7349679_C	17T	16T_39C
Z_7349680_C	17C	56C
Z_7349681_T	17T	55T
Z_7349682_T	18T	55T
Z_7349683_T	18T	55T
Z_7349684_G	18G	55G

Z_7349685_T	18T	54T
Z_7349686_G	17G	55G
Z_7349687_G	18G	55G
Z_7349688_C	18C	55C
Z_7349689_A	18A	56A
Z_7349690_T	19T	57T
Z_7349691_A	20A	57A
Z_7349692_G	20G	58G
Z_7349693_C	20C	59C
Z_7349694_C	21C	59C
Z_7349695_G	21G	60G
Z_7349696_T	23T	60T
Z_7349697_T	24T	60T
Z_7349698_G	25G	60G
Z_7349699_G	25G	63G
Z_7349700_A	25A	63A
Z_7349701_G	26G	63G
Z_7349702_A	25A	62A
Z_7349703_G	25G	63G
Z_7349704_C	25C	64C
Z_7349705_T	24T	63T
Z_7349706_G	24G	64G
Z_7349707_G	22G	25A_41G
Z_7349708_G	22G	65G
Z_7349709_C	22C	63C
Z_7349710_T	22T	63T
Z_7349711_G	22G	65G
Z_7349712_A	21A	66A
Z_7349713_G	22G	66G
Z_7349714_A	24A	67A
Z_7349715_C	24C	66C
Z_7349716_T	26T	67T
Z_7349717_G	27G	66G
Z_7349718_G	28G	68G
Z_7349719_A	29A	68A
Z_7349720_G	29G	68G
Z_7349721_A	26A	65A
Z_7349722_G	25G	67G
Z_7349723_G	24G	67G
Z_7349724_G	23G	66G
Z_7349725_T	20T	65T
Z_7349726_C	20C	66C
Z_7349727_C	20C	65C

Z_7349728_A	19A	64A
Z_7349729_G	20G	63G
Z_7349730_G	20G	63G
Z_7349731_T	20T	63T
Z_7349732_T	19T	62T
Z_7349733_T	20T	62T
Z_7349734_C	20C	59C
Z_7349735_T	18T	62T
Z_7349736_A	19A	61A
Z_7349737_G	21G	61G
Z_7349738_T	20T	61T
Z_7349739_C	21C	61C
Z_7349740_C	21C	62C
Z_7349741_T	20T	61T
Z_7349742_C	21C	61C
Z_7349743_A	21A	62A
Z_7349744_T	21T	60T
Z_7349745_G	21G	61G
Z_7349746_G	21G	61G
Z_7349747_A	22A	60A
Z_7349748_G	22G	61G
Z_7349749_G	22G	61G
Z_7349750_C	22C	62C
Z_7349751_C	22C	61C
Z_7349752_A	20A	61A
Z_7349753_A	21A	61A
Z_7349754_C	21C	61C
Z_7349755_T	21T	61T
Z_7349756_A	21A	39A_22G
Z_7349757_C	21C	61C
Z_7349758_A	22A	61A
Z_7349759_C	22C	61C
Z_7349760_T	21T	60T
Z_7349761_T	21T	59T
Z_7349762_T	21T	57T
Z_7349763_G	21G	56G
Z_7349764_G	21G	55G
Z_7349765_G	21G	55G
Z_7349766_G	19G	52G
Z_7349767_T	18T	51T
Z_7349768_G	18G	52G
Z_7349769_G	19G	52G
Z_7349770_T	19T	51T

Z_7349771_G	18G	50G
Z_7349772_G	18G	49G
Z_7349773_A	18A	49A
Z_7349774_A	19A	50A
Z_7349775_A	19A	47A
Z_7349776_G	19G	46G
Z_7349777_G	19G	46G
Z_7349778_A	19A	29A_18G
Z_7349779_G	19G	47G
Z_7349780_G	40G_11C	47G
Z_7349781_G	null	47G
Z_7349782_G	null	44G
Z_7349783_C	null	45C
Z_7349784_G	11G	43G
Z_7349785_T	11T	41T
Z_7349786_C	11C	40C
Z_7349787_C	11C	41C
Z_7349788_C	11C	39C
Z_7349789_T	11T	38T
Z_7349790_C	11C	38C
Z_7349791_A	12A	36A
Z_7349792_C	11C	37C
Z_7349793_C	10C	37C
Z_7349794_C	10C	36C
Z_7349795_T	10T	34T
Z_7349796_T	10T	34T
Z_7349797_T	11T	35T
Z_7349798_C	11C	34C
Z_7349799_C	11C	35C
Z_7349800_C	11C	35C
Z_7349801_T	10T	31T
Z_7349802_C	10C	28C
Z_7349803_T	9T	27T
Z_7349804_A	8A	27A
Z_7349805_C	9C	27C
Z_7349806_C	9C	26C
Z_7349807_C	9C	26C
Z_7349808_A	7A_1T_1C	23A
Z_7349809_C	9C	24C
Z_7349810_C	9C	24C
Z_7349811_C	7C	7G_18C
Z_7349812_C	7C	25C
Z_7349813_T	6T	22T

Z_7349814_C	6C	22C
Z_7349815_C	6C	21C
Z_7349816_T	3A_5T_9C	21T
Z_7349817_C	5C	21C
Z_7349818_C	5C	20C
Z_7349819_A	5A	19A
Z_7349820_A	5C	13A_6C
Z_7349821_C	5C	19C
Z_7349822_C	5C	19C
Z_7349823_A	5A	19A
Z_7349824_C	5C	19C
Z_7349825_C	5C	19C
Z_7349826_C	5C	19C
Z_7349827_A	5A	19A
Z_7349828_C	5C	19C
Z_7349829_C	5C	18C
Z_7349830_C	6C	19C
Z_7349831_A	6A	19A
Z_7349832_C	6C	19C
Z_7349833_C	6C	19C
Z_7349834_C	6C	18C
Z_7349835_G	5G	17G
Z_7349836_G	5G	17G
Z_7349837_C	5C	17C
Z_7349838_C	5C	16C
Z_7349839_A	5A	16A
Z_7349840_G	5G	16G
Z_7349841_C	5C	15C
Z_7349842_A	5A	15A
Z_7349843_C	5C	18C
Z_7349844_C	5C	18C
Z_7349845_C	5C	18C
Z_7349846_C	5C	18C
Z_7349847_T	5T	18T
Z_7349848_C	5C	19C
Z_7349849_C	5C	19C
Z_7349850_C	5C	21C
Z_7349851_C	5C	21C
Z_7349852_C	4C	22C
Z_7349853_T	4T	22T
Z_7349854_T	4C	22T
Z_7349855_C	5C	22C
Z_7349856_C	6C	23C

Z_7349857_T	6T	25T
Z_7349858_C	6C	27C
Z_7349859_T	6T	27T
Z_7349860_C	6C	28C
Z_7349861_A	9A	28A
Z_7349862_C	9C	28C
Z_7349863_C	8C	28C
Z_7349864_T	8T	28T
Z_7349865_C	8C	28C
Z_7349866_C	8C	28C
Z_7349867_A	8A	29A
Z_7349868_G	8G	29G
Z_7349869_C	8C	28C
Z_7349870_T	8T	28T
Z_7349871_C	8C	29C
Z_7349872_C	9C	28C
Z_7349873_T	9T	27T
Z_7349874_T	10T	25T
Z_7349875_G	10G	25G
Z_7349876_A	12A	25A
Z_7349877_A	12A	24A
Z_7349878_A	13A	26A
Z_7349879_T	13T	26T
Z_7349880_A	13A	26A
Z_7349881_A	13A	28A
Z_7349882_A	13A	27A
Z_7349883_T	14T	28T
Z_7349884_G	14G	28G
Z_7349885_C	14C	29C
Z_7349886_A	15A	29A
Z_7349887_G	16G	29G
Z_7349888_C	16C	30C
Z_7349889_T	17T	31T
Z_7349890_T	17T	31T
Z_7349891_C	18C	32C
Z_7349892_G	18G	31G
Z_7349893_G	18G	31G
Z_7349894_G	18G	31G
Z_7349895_C	17C	31C
Z_7349896_C	19C	32C
Z_7349897_C	19C	32C
Z_7349898_A	19A	32A
Z_7349899_C	19C	32C

Z_7349900_T	18T	32T
Z_7349901_C	18C	32C
Z_7349902_C	20C	32C
Z_7349903_A	20A	32A
Z_7349904_C	20C	32C
Z_7349905_A	19A	33A
Z_7349906_A	19A	32A
Z_7349907_T	20T	33T
Z_7349908_G	21G	32G
Z_7349909_G	21G	34G
Z_7349910_C	22C	35C
Z_7349911_G	22G	34G
Z_7349912_A	22A	35A
Z_7349913_A	22A	35A
Z_7349914_G	21G	35G
Z_7349915_A	22A	35A
Z_7349916_G	23G	35G
Z_7349917_G	23G	35G
Z_7349918_G	23G	35G
Z_7349919_T	23T	35T
Z_7349920_C	21C	37C
Z_7349921_T	24T	37T
Z_7349922_G	25G	37G
Z_7349923_G	25G	37G
Z_7349924_T	25T	37T
Z_7349925_C	23C	36C
Z_7349926_G	23G	36G
Z_7349927_G	22G	37G
Z_7349928_C	23C	38C
Z_7349929_C	23C	38C
Z_7349930_A	23A	38A
Z_7349931_T	20T	38T
Z_7349932_C	20C	38C
Z_7349933_C	20C	38C
Z_7349934_G	20G	39G
Z_7349935_G	20G	39G
Z_7349936_C	20C	39C
Z_7349937_A	20A	39A
Z_7349938_C	20C	36C
Z_7349939_A	20A	35A
Z_7349940_T	20T	35T
Z_7349941_G	19G	35G
Z_7349942_A	18A	36A

Z_7349943_G	18G	35G
Z_7349944_C	17C	35C
Z_7349945_C	17C	33C
Z_7349946_C	15C	33C
Z_7349947_A	14G	24A_9G
Z_7349948_A	14A	33A
Z_7349949_A	14A	33A
Z_7349950_G	14G	32G
Z_7349951_G	13G	33G
Z_7349952_G	15G	31G
Z_7349953_G	15G	29G
Z_7349954_C	15C	29C
Z_7349955_T	15T	28T
Z_7349956_G	14G	28G
Z_7349957_A	13A	28A
Z_7349958_G	13G	28G
Z_7349959_C	12C	28C
Z_7349960_T	12T	28T
Z_7349961_T	12T	28T
Z_7349962_C	12C	27C
Z_7349963_T	12T	27T
Z_7349964_C	12C	27C
Z_7349965_G	11G	28G
Z_7349966_T	11T	27T
Z_7349967_G	12G	26G
Z_7349968_G	11G	27G
Z_7349969_C	12C	28C
Z_7349970_G	12G	28G
Z_7349971_G	12G	29G
Z_7349972_C	10C	29C
Z_7349973_C	10C	29C
Z_7349974_C	10C	30C
Z_7349975_T	10T	31T
Z_7349976_T	10T	29T
Z_7349977_G	10G	29G
Z_7349978_C	9C	29C
Z_7349979_T	11T	29T
Z_7349980_T	10T	29T
Z_7349981_T	10T	29T
Z_7349982_G	9G	29G
Z_7349983_C	11C	28C
Z_7349984_T	11T	28T
Z_7349985_C	11C	29C

Z_7349986_C	10C	28C
Z_7349987_T	10T	29T
Z_7349988_G	10G	29G
Z_7349989_T	10T	19T_10C
Z_7349990_T	10T	28T
Z_7349991_G	9G	26G
Z_7349992_C	8C	26C
Z_7349993_A	8A	26A
Z_7349994_G	8G	27G
Z_7349995_G	8G	27G
Z_7349996_C	8C	27C
Z_7349997_A	8A	27A
Z_7349998_G	8G	27G
Z_7349999_G	8G	27G
Z_7350000_C	8C	26C
Z_7350001_C	8C	27C
Z_7350002_A	8A	26A
Z_7350003_G	8G	26G
Z_7350004_G	8G	25G
Z_7350005_A	8A	23A
Z_7350006_T	9T	24T
Z_7350007_G	9G	22G
Z_7350008_C	9C	22C
Z_7350009_G	9G	24G
Z_7350010_G	9G	24G
Z_7350011_C	9C	23C
Z_7350012_C	9C	23C
Z_7350013_C	9C	23C
Z_7350014_T	9T	22T
Z_7350015_T	9T	21T
Z_7350016_G	9G	21G
Z_7350017_A	9A	21A
Z_7350018_C	9C	21C
Z_7350019_C	9C	21C
Z_7350020_T	10T	21T
Z_7350021_G	9G	21G
Z_7350022_C	9C	20C
Z_7350023_G	9G	19G
Z_7350024_C	9A	19C
Z_7350025_C	9C	19C
Z_7350026_T	9T	19T
Z_7350027_C	9C	19C
Z_7350028_G	9G	20G

Z_7350029_T	10T	19T
Z_7350030_C	10C	19C
Z_7350031_G	9C	14G_5C
Z_7350032_G	9G	19G
Z_7350033_G	9G	20G
Z_7350034_C	9C	21C
Z_7350035_T	10T	21T
Z_7350036_C	9C	21C
Z_7350037_C	9C	20C
Z_7350038_A	10A	19A
Z_7350039_G	10G	20G
Z_7350040_C	10C	21C
Z_7350041_T	10T	21T
Z_7350042_G	10G	19G
Z_7350043_C	10C	20C
Z_7350044_A	10A	20A
Z_7350045_G	10G	20G
Z_7350046_C	10C	19C
Z_7350047_A	10A	19A
Z_7350048_G	10G	19G
Z_7350049_C	8C	19C
Z_7350050_T	8T	19T
Z_7350051_T	8T	19T
Z_7350052_G	8G	19G
Z_7350053_A	8A	19A
Z_7350054_G	7G	19G
Z_7350055_A	6A	19A
Z_7350056_A	7A	19A
Z_7350057_T	6T	19T
Z_7350058_C	7C	19C
Z_7350059_A	7A	19A
Z_7350060_C	7C	18C
Z_7350061_G	7G	18G
Z_7350062_T	8T	18T
Z_7350063_C	7C	18C
Z_7350064_G	8G	17G
Z_7350065_G	8G	17G
Z_7350066_G	8G	16G
Z_7350067_G	8G	16G
Z_7350068_A	8A	15A
Z_7350069_T	8T	13T
Z_7350070_G	8G	13G
Z_7350071_T	8T	12T

Z_7350072_C	8C	14C
Z_7350073_C	8C	14C
Z_7350074_G	8G	14G
Z_7350075_G	8G	14G
Z_7350076_C	7C	14C
Z_7350077_G	8G	14G
Z_7350078_C	8C	14C
Z_7350079_T	8T	11T
Z_7350080_G	8G	12G
Z_7350081_T	7T	12T
Z_7350082_C	8T	11C
Z_7350083_G	8G	11G
Z_7350084_T	8T	10T
Z_7350085_G	8G	11G
Z_7350086_G	8G	11G
Z_7350087_T	8T	11T
Z_7350088_T	8T	11T
Z_7350089_G	8G	10G
Z_7350090_G	7G	10G
Z_7350091_G	7G	10G
Z_7350092_G	7G	11G
Z_7350093_A	7A	11A
Z_7350094_C	7C	12C
Z_7350095_C	7C	13C
Z_7350096_C	6C	12C
Z_7350097_T	7T	12T
Z_7350098_C	6C	12C
Z_7350099_C	6C	13C
Z_7350100_T	6T	13T
Z_7350101_C	6C	13C
Z_7350102_C	6C	13C
Z_7350103_T	6T	13T
Z_7350104_C	6C	11C
Z_7350105_C	5C	11C
Z_7350106_T	5T	11T
Z_7350107_C	1G_4C	11C
Z_7350108_C	4C	11C
Z_7350109_A	4T	10A
Z_7350110_C	4C	11C
Z_7350111_C	4C	11C
Z_7350112_A	4A	11A
Z_7350113_C	4C	10C
Z_7350114_C	4C	11C

Z_7350115_A	4A	9A
Z_7350116_C	4C	9C
Z_7350117_C	3C	9C
Z_7350118_A	3A	9A
Z_7350119_C	3C	9C
Z_7350120_C	2C	9C
Z_7350121_C	2C	8C
Z_7350122_C	2C	9C
Z_7350123_C	2C	8C
Z_7350124_T	2T	8T
Z_7350125_C	2C	8C
Z_7350126_C	2C	8C
Z_7350127_A	2A	8A
Z_7350128_C	2C	8C
Z_7350129_C	2C	7C
Z_7350130_C	2C	8C
Z_7350131_C	3C	8C
Z_7350132_C	2C	8C
Z_7350133_T	2T	7T
Z_7350134_C	2C	9C
Z_7350135_C	2C	8C
Z_7350136_G	2A	7G
Z_7350137_C	2C	8C
Z_7350138_C	2C	8C
Z_7350139_C	2C	8C
Z_7350140_C	2C	8C
Z_7350141_C	2C	8C
Z_7350142_T	2T	8T
Z_7350143_C	3C	10C
Z_7350144_C	3C	10C
Z_7350145_A	4A	10A
Z_7350146_C	4C	10C
Z_7350147_C	3C	10C
Z_7350148_C	3C	11C
Z_7350149_C	3C	11C
Z_7350150_C	3C	11C
Z_7350151_A	3A	12A
Z_7350152_C	3C	12C
Z_7350153_T	3T	12T
Z_7350154_A	5A	12A
Z_7350155_C	5C	13C
Z_7350156_C	5C	13C
Z_7350157_G	7G	12G

Z_7350158_C	8C	14C
Z_7350159_C	8C	14C
Z_7350160_C	8C	14C
Z_7350161_C	7C	14C
Z_7350162_C	8C	14C
Z_7350163_A	8A	13A
Z_7350164_C	8C	14C
Z_7350165_T	8T	14T
Z_7350166_G	8G	14G
Z_7350167_C	9C	14C
Z_7350168_C	9C	14C
Z_7350169_C	9C	14C
Z_7350170_C	9C	13C
Z_7350171_C	9C	13C
Z_7350172_T	9T	13T
Z_7350173_C	9C	13C
Z_7350174_C	9C	15C
Z_7350175_G	8G	15G
Z_7350176_C	9C	15C
Z_7350177_C	9C	15C
Z_7350178_T	9T	14T
Z_7350179_G	8G	14G
Z_7350180_G	9G	14G
Z_7350181_G	9G	14G
Z_7350182_T	8T	14T
Z_7350183_A	9A	14A
Z_7350184_A	8A	15A
Z_7350185_G	9G	15G
Z_7350186_C	9C	15C
Z_7350187_C	10C	14C
Z_7350188_T	10T	14T
Z_7350189_C	10C	13C
Z_7350190_G	10G	12G
Z_7350191_G	10G	14G
Z_7350192_G	10G	14G
Z_7350193_A	10A	15A
Z_7350194_T	10T	13T
Z_7350195_A	10A	14A
Z_7350196_C	9T	14C
Z_7350197_G	9G	14G
Z_7350198_G	9G	15G
Z_7350199_A	9G	16A
Z_7350200_T	10T	17T

Z_7350201_A	10A	17A
Z_7350202_A	10A	17A
Z_7350203_G	10G	17G
Z_7350204_C	10C	18C
Z_7350205_A	10A	18A
Z_7350206_G	10G	18G
Z_7350207_G	10G	18G
Z_7350208_T	10T	18T
Z_7350209_G	10G	18G
Z_7350210_C	10C	17C
Z_7350211_C	11C	18C
Z_7350212_G	11G	18G
Z_7350213_C	10C	18C
Z_7350214_C	10C	19C
Z_7350215_T	9T	19T
Z_7350216_C	9C	21C
Z_7350217_G	10G	21G
Z_7350218_T	10T	22T
Z_7350219_G	10G	22G
Z_7350220_C	9G	22C
Z_7350221_G	9G	22G
Z_7350222_G	11G	24G
Z_7350223_A	12A	23A
Z_7350224_T	11T	26T
Z_7350225_G	11G	24G
Z_7350226_C	11C	26C
Z_7350227_G	11G	26G
Z_7350228_G	10G	25G
Z_7350229_C	10C	25C
Z_7350230_C	10C	25C
Z_7350231_C	10C	25C
Z_7350232_A	10A	24A
Z_7350233_T	10T	25T
Z_7350234_A	10A	25A
Z_7350235_T	10C	25T
Z_7350236_G	10G	25G
Z_7350237_G	9G	24G
Z_7350238_G	9G	23G
Z_7350239_T	10T	24T
Z_7350240_C	10C	24C
Z_7350241_T	10T	24T
Z_7350242_G	10G	24G
Z_7350243_G	9G	23G

Z_7350244_A	11A	23A
Z_7350245_A	11A	23A
Z_7350246_A	11A	22A
Z_7350247_C	10C	22C
Z_7350248_T	11T	23T
Z_7350249_C	10C	23C
Z_7350250_A	11A	22A
Z_7350251_G	10G	22G
Z_7350252_A	12A	22A
Z_7350253_C	11C	21C
Z_7350254_T	12T	21T
Z_7350255_T	12T	22T
Z_7350256_G	11G	22G
Z_7350257_A	11A	22A
Z_7350258_T	11T	22T
Z_7350259_G	11G	22G
Z_7350260_G	11G	23G
Z_7350261_C	11C	23C
Z_7350262_C	13C	23C
Z_7350263_C	13C	23C
Z_7350264_G	13G	23G
Z_7350265_G	13G	24G
Z_7350266_C	13C	24C
Z_7350267_T	13T	23T
Z_7350268_A	14A	25A
Z_7350269_G	14G	23G
Z_7350270_T	14T	24T
Z_7350271_G	15G	24G
Z_7350272_A	16A	24A
Z_7350273_A	16A	24A
Z_7350274_G	16G	24G
Z_7350275_G	16G	24G
Z_7350276_G	16G	25G
Z_7350277_G	15G	25G
Z_7350278_G	15G	25G
Z_7350279_G	17G	24G
Z_7350280_G	17G	24G
Z_7350281_T	16T	24T
Z_7350282_A	17A	23A
Z_7350283_G	17G	23G
Z_7350284_T	16T	24T
Z_7350285_G	16G	24G
Z_7350286_G	16G	21G

Z_7350287_T	16T	21T
Z_7350288_A	17A	21A
Z_7350289_G	17G	21G
Z_7350290_C	19C	21C
Z_7350291_C	18C	21C
Z_7350292_C	18C	22C
Z_7350293_G	17G	21G
Z_7350294_A	16A	20A
Z_7350295_G	17G	19G
Z_7350296_A	15A	20A
Z_7350297_G	14G	21G
Z_7350298_G	14G	21G
Z_7350299_G	14G	20G
Z_7350300_C	14C	21C
Z_7350301_C	13C	22C
Z_7350302_C	13C	22C
Z_7350303_C	13C	22C
Z_7350304_A	14A	21A
Z_7350305_T	14T	21T
Z_7350306_G	12G	22G
Z_7350307_G	14G	22G
Z_7350308_C	15C	21C
Z_7350309_C	14C	22C
Z_7350310_C	13C	22C
Z_7350311_G	14G	20G
Z_7350312_C	14C	20C
Z_7350313_G	14G	19G
Z_7350314_G	14G	19G
Z_7350315_C	14C	19C
Z_7350316_G	14G	20G
Z_7350317_G	14G	18G
Z_7350318_C	14C	18C
Z_7350319_G	14G	16G
Z_7350320_G	14G	16G
Z_7350321_G	14G	16G
Z_7350322_G	13G	16G
Z_7350323_A	13A	15A
Z_7350324_C	13C	16C
Z_7350325_G	14A	5A_12G
Z_7350326_G	14G	17G
Z_7350327_G	14G	17G
Z_7350328_C	14C	17C
Z_7350329_A	14A	15A

Z_7350330_C	14C	17C
Z_7350331_G	14G	17G
Z_7350332_G	13G	17G
Z_7350333_C	13C	16C
Z_7350334_C	13C	16C
Z_7350335_A	13A	15A
Z_7350336_G	13G	16G
Z_7350337_G	14G	16G
Z_7350338_G	14G	16G
Z_7350339_C	15C	16C
Z_7350340_G	14G	16G
Z_7350341_G	13G	16G
Z_7350342_G	12G	16G
Z_7350343_G	12G	15G
Z_7350344_G	11G	16G
Z_7350345_T	11T	16T
Z_7350346_C	12C	17C
Z_7350347_C	12C	17C
Z_7350348_C	12C	17C
Z_7350349_G	11G	17G
Z_7350350_T	11T	17T
Z_7350351_A	10A	16A
Z_7350352_G	10G	17G
Z_7350353_G	10G	19G
Z_7350354_G	11G	20G
Z_7350355_G	11G	19G
Z_7350356_C	11C	19C
Z_7350357_T	11T	17T
Z_7350358_G	10G	19G
Z_7350359_C	11C	18C
Z_7350360_G	9G	19G
Z_7350361_C	9C	19C
Z_7350362_T	9T	17T
Z_7350363_C	9C	18C
Z_7350364_A	9G	10A_9G
Z_7350365_T	8T	17T
Z_7350366_A	9A	18A
Z_7350367_G	9G	18G
Z_7350368_T	9T	17T
Z_7350369_C	9C	18C
Z_7350370_C	9C	18C
Z_7350371_A	9A	17A
Z_7350372_C	9C	17C

Z_7350373_A	9A	18A
Z_7350374_G	9G	18G
Z_7350375_G	9G	19G
Z_7350376_G	10G	19G
Z_7350377_G	11G	19G
Z_7350378_T	11T	18T
Z_7350379_C	11C	19C
Z_7350380_A	11A	19A
Z_7350381_T	11T	18T
Z_7350382_G	12G	19G
Z_7350383_G	12G	19G
Z_7350384_C	12C	19C
Z_7350385_A	12G	10A_9G
Z_7350386_C	12C	18C
Z_7350387_C	12C	18C
Z_7350388_G	12G	18G
Z_7350389_C	13C	18C
Z_7350390_T	13T	18T
Z_7350391_G	13G	17G
Z_7350392_C	13C	16C
Z_7350393_T	14T	16T
Z_7350394_C	15C	17C
Z_7350395_C	13C	17C
Z_7350396_C	14C	16C
Z_7350397_G	14G	16G
Z_7350398_C	14C	16C
Z_7350399_T	14T	16T
Z_7350400_G	13G	16G
Z_7350401_C	14C	16C
Z_7350402_C	14C	16C
Z_7350403_C	14C	16C
Z_7350404_C	14C	15C
Z_7350405_C	13C	15C
Z_7350406_T	14T	15T
Z_7350407_G	13G	15G
Z_7350408_C	12C	15C
Z_7350409_C	10C	6T_9C
Z_7350410_G	11G	15G
Z_7350411_G	11G	13G
Z_7350412_A	12A	13A
Z_7350413_G	12G	13G
Z_7350414_G	12G	13G
Z_7350415_G	13G	14G

Z_7350416_A	14A	13A
Z_7350417_G	14G	13G
Z_7350418_G	14G	13G
Z_7350419_C	14C	13C
Z_7350420_C	13C	12C
Z_7350421_C	14C	11C
Z_7350422_T	14T	11T
Z_7350423_T	14T	12T
Z_7350424_G	14G	12G
Z_7350425_G	14G	12G
Z_7350426_C	14C	12C
Z_7350427_T	12T	11T
Z_7350428_C	13C	12C
Z_7350429_C	12C	12C
Z_7350430_C	12C	12C
Z_7350431_A	12A	12A
Z_7350432_C	12C	12C
Z_7350433_C	11C	12C
Z_7350434_C	12C	12C
Z_7350435_C	10C	11C
Z_7350436_G	11G	12G
Z_7350437_G	11G	12G
Z_7350438_G	11G	12G
Z_7350439_G	11G	11G
Z_7350440_T	10T	11T
Z_7350441_G	11G	11G
Z_7350442_G	11G	11G
Z_7350443_T	11T	11T
Z_7350444_G	11G	11G
Z_7350445_G	11G	10G
Z_7350446_T	9T	10T
Z_7350447_G	9G	10G
Z_7350448_G	8G	8G
Z_7350449_G	8G	7G
Z_7350450_G	8G	7G
Z_7350451_A	8A	6A
Z_7350452_T	6T	7T
Z_7350453_G	7G	7G
Z_7350454_G	6G	7G
Z_7350455_G	6G	6G
Z_7350456_G	6G	6G
Z_7350457_A	6A	6A
Z_7350458_T	5T	6T

Z_7350459_G	5G	5G
Z_7350460_A	5A	5A
Z_7350461_T	5T	6T
Z_7350462_G	5G	6G
Z_7350463_G	4G	6G
Z_7350464_T	4T	6T
Z_7350465_G	4G	6G
Z_7350466_G	4G	6G
Z_7350467_T	4T	5T
Z_7350468_G	4G	5G
Z_7350469_G	5G	5G
Z_7350470_T	5T	4T
Z_7350471_G	4G	5G
Z_7350472_G	4G	6G
Z_7350473_T	4T	7T
Z_7350474_G	4G	8G
Z_7350475_G	4G	8G
Z_7350476_T	4T	8T
Z_7350477_G	4G	9G
Z_7350478_G	4G	8G
Z_7350479_T	4T	7T_2G
Z_7350480_G	4G	13G
Z_7350481_G	4G	13G
Z_7350482_G	3G	14G
Z_7350483_G	3G	13G
Z_7350484_A	3A	14A
Z_7350485_T	2T	15T
Z_7350486_G	1G	22G
Z_7350487_A	1A	22A
Z_7350488_T	1T	22T
Z_7350489_G	1G	23G
Z_7350490_G	1G	22G
Z_7350491_T	1T	22T
Z_7350492_G	1G	22G
Z_7350493_G	2G	22G
Z_7350494_T	3T	22T
Z_7350495_G	3G	22G
Z_7350496_G	3G	22G
Z_7350497_T	3T	22T
Z_7350498_G	3G	23G
Z_7350499_G	3G	23G
Z_7350500_T	3T	23T
Z_7350501_G	3G	23G

Z_7350502_A	2G_1C	13A_10G
Z_7350503_T	3T	23T
Z_7350504_G	3G	23G
Z_7350505_A	3A	23A
Z_7350506_G	4G	23G
Z_7350507_C	4C	23C
Z_7350508_C	4C	23C
Z_7350509_A	4A	23A
Z_7350510_G	4G	22G
Z_7350511_G	4G	22G
Z_7350512_C	4C	22C
Z_7350513_C	4C	22C
Z_7350514_G	4G	22G
Z_7350515_T	4T	22T
Z_7350516_G	5G	22G
Z_7350517_G	5G	22G
Z_7350518_A	4A_1T	22A
Z_7350519_G	5G	22G
Z_7350520_C	5C	22C
Z_7350521_G	5G	22G
Z_7350522_A	5A	22A
Z_7350523_G	5G	22G
Z_7350524_G	5G	22G
Z_7350525_C	5C	22C
Z_7350526_C	5C	22C
Z_7350527_G	5G	22G
Z_7350528_G	5G	23G
Z_7350529_C	5C	23C
Z_7350530_A	5A	23A
Z_7350531_G	5G	22G
Z_7350532_G	5G	22G
Z_7350533_C	6C	22C
Z_7350534_T	6T	22T
Z_7350535_C	6C	22C
Z_7350536_C	6C	22C
Z_7350537_C	6C	22C
Z_7350538_A	6A	20A
Z_7350539_T	6T	21T
Z_7350540_A	6A	21A
Z_7350541_G	6G	21G
Z_7350542_T	6T	20T
Z_7350543_C	6C	19C
Z_7350544_C	7C	19C

Z_7350545_C	7C	19C
Z_7350546_C	7C	17C
Z_7350547_T	7T	17T
Z_7350548_G	7G	17G
Z_7350549_C	7C	17C
Z_7350550_C	7C	17C
Z_7350551_T	7T	17T
Z_7350552_G	7G	17G
Z_7350553_G	7G	17G
Z_7350554_A	7A	16A
Z_7350555_C	7C	14C
Z_7350556_G	8G	14G
Z_7350557_G	8G	13G
Z_7350558_G	8G	13G
Z_7350559_G	9G	13G
Z_7350560_G	9G	11G
Z_7350561_A	9A	11A
Z_7350562_C	9C	11C
Z_7350563_A	8A	11A
Z_7350564_C	7C	10C
Z_7350565_G	7G	3T_6G
Z_7350566_A	7A	7A
Z_7350567_T	7T	6T
Z_7350568_G	7G	5G
Z_7350569_G	7G	5G
Z_7350570_G	8G	4G_1C
Z_7350571_G	7G	5G
Z_7350572_G	8G	5G
Z_7350573_G	8G	5G
Z_7350574_C	8C	2T_3C
Z_7350575_G	8G	4G
Z_7350576_G	6G	5G
Z_7350577_A	7A	5A
Z_7350578_G	7G	5G
Z_7350579_G	7G	5G
Z_7350580_A	7A	4A
Z_7350581_G	7G	4G
Z_7350582_G	7G	4G
Z_7350583_A	7A	4A
Z_7350584_G	7G	4G
Z_7350585_G	6G	4G
Z_7350586_G	6G	4G
Z_7350587_G	6G	4G

Z_7350588_G	6G	4G
Z_7350589_C	6T	4C
Z_7350590_G	6G	4G
Z_7350591_G	6G	4G
Z_7350592_T	6T	3T_1G
Z_7350593_G	7G	4G
Z_7350594_G	7G	4G
Z_7350595_G	7G	4G
Z_7350596_G	7G	4G
Z_7350597_G	7G	5G
Z_7350598_C	7C	5C
Z_7350599_G	8G	5G
Z_7350600_G	8G	5G
Z_7350601_G	8G	5G
Z_7350602_G	9G	5G
Z_7350603_G	8G	5G
Z_7350604_C	8C	5C
Z_7350605_G	9G	5G
Z_7350606_G	9G	4G_1C
Z_7350607_G	9G	5G
Z_7350608_G	8G	5G
Z_7350609_G	8G	4G_1C
Z_7350610_G	8G	6G
Z_7350611_C	8C	7C
Z_7350612_C	8C	7C
Z_7350613_C	7C	7C
Z_7350614_G	7G	7G
Z_7350615_T	6T	6T
Z_7350616_C	7C	8C
Z_7350617_G	8G	8G
Z_7350618_C	8C	8C
Z_7350619_G	9G	8G
Z_7350620_G	9G	8G
Z_7350621_G	9G	9G
Z_7350622_G	9G	9G
Z_7350623_G	9G	8G
Z_7350624_C	8C	8C
Z_7350625_C	9C	2T_2G_8C
Z_7350626_G	8G	7G
Z_7350627_T	6C	6T
Z_7350628_C	6C	9C
Z_7350629_G	6G	9G
Z_7350630_T	6T	8T

Z_7350631_C	6C	7C
Z_7350632_T	6T	7T
Z_7350633_C	6C	7C
Z_7350634_C	6C	8C
Z_7350635_A	6A	8A
Z_7350636_G	8G	8G
Z_7350637_C	8C	8C
Z_7350638_T	9T	8T
Z_7350639_T	10T	8T
Z_7350640_G	10G	9G
Z_7350641_A	9A	8A
Z_7350642_A	9A	9A
Z_7350643_C	9C	9C
Z_7350644_C	9C	9C
Z_7350645_C	10C	10C
Z_7350646_G	10G	10G
Z_7350647_T	10T	10T
Z_7350648_T	11T	11T
Z_7350649_G	11G	13G
Z_7350650_G	11G	13G
Z_7350651_C	11C	13C
Z_7350652_G	11G	13G
Z_7350653_C	11C	13C
Z_7350654_G	12G	13G
Z_7350655_G	13G	13G
Z_7350656_A	14A	14A
Z_7350657_T	14T	14T
Z_7350658_C	13C	14C
Z_7350659_A	16A	14A
Z_7350660_G	18G	15G
Z_7350661_G	18G	15G
Z_7350662_G	17G	15G
Z_7350663_C	18C	15C
Z_7350664_C	18C	15C
Z_7350665_T	18T	15T
Z_7350666_T	19T	15T
Z_7350667_C	20C	15C
Z_7350668_T	19T	15T
Z_7350669_T	20T	16T
Z_7350670_C	20C	17C
Z_7350671_T	20T	19T
Z_7350672_G	20G	20G
Z_7350673_C	20C	20C

Z_7350674_T	20T	21T
Z_7350675_G	21G	21G
Z_7350676_C	21C	21C
Z_7350677_T	21T	21T
Z_7350678_T	21T	23T
Z_7350679_C	21C	23C
Z_7350680_A	21A	23A
Z_7350681_G	20G	24G
Z_7350682_G	21G	24G
Z_7350683_G	22G	24G
Z_7350684_C	22C	24C
Z_7350685_G	22G	24G
Z_7350686_C	23C	24C
Z_7350687_G	22G	25G
Z_7350688_G	22G	25G
Z_7350689_T	22T	28T
Z_7350690_C	22C	28C
Z_7350691_C	22C	28C
Z_7350692_C	23C	29C
Z_7350693_G	23G	29G
Z_7350694_C	23C	29C
Z_7350695_T	24T	29T
Z_7350696_T	24T	29T
Z_7350697_G	25G	29G
Z_7350698_T	25T	30T
Z_7350699_A	26A	30A
Z_7350700_C	25C	31C
Z_7350701_A	26A	33A
Z_7350702_T	26T	33T
Z_7350703_C	25C	34C
Z_7350704_G	26G	35G
Z_7350705_G	28G	35G
Z_7350706_T	25T	35T
Z_7350707_C	28C	35C
Z_7350708_C	27C	34C
Z_7350709_G	26G	33G
Z_7350710_A	25A	34A
Z_7350711_A	25A	34A
Z_7350712_C	26C	34C
Z_7350713_T	26T	34T
Z_7350714_T	25T	36T
Z_7350715_G	25G	37G
Z_7350716_T	24T	35T

Z_7350717_T	25T	36T
Z_7350718_G	25G	36G
Z_7350719_C	26C	36C
Z_7350720_G	26G	36G
Z_7350721_T	25T	36T
Z_7350722_C	26C	36C
Z_7350723_C	26C	36C
Z_7350724_G	24G	36G
Z_7350725_C	24C	36C
Z_7350726_C	22C	36C
Z_7350727_T	22T	37T
Z_7350728_C	23C	37C
Z_7350729_T	21T	36T
Z_7350730_C	19C	36C
Z_7350731_A	22A	36A
Z_7350732_T	23T	36T
Z_7350733_C	25C	36C
Z_7350734_C	25C	36C
Z_7350735_G	25G	35G
Z_7350736_G	24G	36G
Z_7350737_T	23T	37T
Z_7350738_C	23C	37C
Z_7350739_T	24T	37T
Z_7350740_G	24G	35G
Z_7350741_C	22C	37C
Z_7350742_G	23G	37G
Z_7350743_C	23C	37C
Z_7350744_G	22G	35G
Z_7350745_C	20C	35C
Z_7350746_A	21A	35A
Z_7350747_C	20C	35C
Z_7350748_G	20G	35G
Z_7350749_G	21G	35G
Z_7350750_C	20C	36C
Z_7350751_T	21T	35T
Z_7350752_G	21G	36G
Z_7350753_G	20G	36G
Z_7350754_A	20A	36A
Z_7350755_G	21G	35G
Z_7350756_G	19G	36G
Z_7350757_A	21A	37A
Z_7350758_G	21G	37G
Z_7350759_A	21A	36A

Z_7350760_G	21G	37G
Z_7350761_G	21G	37G
Z_7350762_A	20A	37A
Z_7350763_G	20G	37G
Z_7350764_A	21A	36A
Z_7350765_G	20G	37G
Z_7350766_A	20A	34A
Z_7350767_A	19A	33A
Z_7350768_G	18G	33G
Z_7350769_G	18G	32G
Z_7350770_G	18G	32G
Z_7350771_A	18A	32A
Z_7350772_A	18A	33A
Z_7350773_G	19G	31G
Z_7350774_A	21A	32A
Z_7350775_A	19A	32A
Z_7350776_G	20G	10A_21G
Z_7350777_T	18T	31T
Z_7350778_C	19C	32C
Z_7350779_G	19G	33G
Z_7350780_G	20G	33G
Z_7350781_G	20G	33G
Z_7350782_G	19G	32G
Z_7350783_A	20A	31A
Z_7350784_G	21G	29G
Z_7350785_A	22A	29A
Z_7350786_A	23A	29A
Z_7350787_A	22A	27A
Z_7350788_G	24G	28G
Z_7350789_C	22C	26C
Z_7350790_A	23A	28A
Z_7350791_G	23G	28G
Z_7350792_A	23A	28A
Z_7350793_C	22C	27C
Z_7350794_C	23C	27C
Z_7350795_C	24C	26C
Z_7350796_T	23C	16T_7C
Z_7350797_T	25T	24T
Z_7350798_G	22G	23G
Z_7350799_G	26G	24G
Z_7350800_C	26C	24C
Z_7350801_G	24G	24G
Z_7350802_T	22T	24T

Z_7350803_C	21C	24C
Z_7350804_A	21A	26A
Z_7350805_G	21G	25G
Z_7350806_G	22G	25G
Z_7350807_C	22C	25C
Z_7350808_C	23C	25C
Z_7350809_C	23T	8T_18C
Z_7350810_A	23A	26A
Z_7350811_G	24G	26G
Z_7350812_G	24G	26G
Z_7350813_G	24G	26G
Z_7350814_G	25G	26G
Z_7350815_T	26T	26T
Z_7350816_C	24C	25C
Z_7350817_T	26T	25T
Z_7350818_G	29G	26G
Z_7350819_T	28T	26T
Z_7350820_T	29T	27T
Z_7350821_C	29C	29C
Z_7350822_A	30A	28A
Z_7350823_C	30C	28C
Z_7350824_A	30A	28A
Z_7350825_C	29C	29C
Z_7350826_A	29A	29A
Z_7350827_A	28A	29A
Z_7350828_G	29G	29G
Z_7350829_C	29C	29C
Z_7350830_T	29T	29T
Z_7350831_G	31G	29G
Z_7350832_G	31G	28G
Z_7350833_G	32G	28G
Z_7350834_G	32G	28G
Z_7350835_A	33A	48A_60G_30C
Z_7350836_G	33G	18G
Z_7350837_G	34G	18G
Z_7350838_C	33C	17C
Z_7350839_A	34A	18A
Z_7350840_G	35G	18G
Z_7350841_G	36G	19G
Z_7350842_C	36C	19C
Z_7350843_A	34A	19A
Z_7350844_G	33G	19G
Z_7350845_G	35G	18G

Z_7350846_C	33C	18C
Z_7350847_A	34A	28A
Z_7350848_G	33G	28G
Z_7350849_G	34G	26G
Z_7350850_C	34C	27C
Z_7350851_A	34A	27A
Z_7350852_G	35A	26G
Z_7350853_G	35G	27G
Z_7350854_C	32C	27C
Z_7350855_A	36A	27A
Z_7350856_G	34G	29G
Z_7350857_G	35G	29G
Z_7350858_C	33C	29C
Z_7350859_A	33A	30A
Z_7350860_G	34G	30G
Z_7350861_C	36C	31C
Z_7350862_C	36C	32C
Z_7350863_T	37T	33T
Z_7350864_C	36C	34C
Z_7350865_G	34G	34G
Z_7350866_G	36G	34G
Z_7350867_T	37T	35T
Z_7350868_G	37G	35G
Z_7350869_G	33G	36G
Z_7350870_G	35G	36G
Z_7350871_T	35T	35T
Z_7350872_T	37T	37T
Z_7350873_G	37G	36G
Z_7350874_G	37G	35G
Z_7350875_A	38A	35A
Z_7350876_G	38G	35G
Z_7350877_G	39G	36G
Z_7350878_C	38C	36C
Z_7350879_A	37A	35A
Z_7350880_G	37G	35G
Z_7350881_G	36G	34G
Z_7350882_A	37A	34A
Z_7350883_G	37G	34G
Z_7350884_G	38G	35G
Z_7350885_A	36A	35A
Z_7350886_G	36G	35G
Z_7350887_A	37A	35A
Z_7350888_C	35C	36C

Z_7350889_T	35T	36T
Z_7350890_T	35T	36T
Z_7350891_G	37G	37G
Z_7350892_G	36G	36G
Z_7350893_A	36A	36A
Z_7350894_A	36A	36A
Z_7350895_A	35A	37A
Z_7350896_A	37A	37A
Z_7350897_G	38G	37G
Z_7350898_G	38G	37G
Z_7350899_A	39A	37A
Z_7350900_T	39T	37T
Z_7350901_G	37G	36G
Z_7350902_C	36C	36C
Z_7350903_T	35T	35T
Z_7350904_C	35C	35C
Z_7350905_C	35C	34C
Z_7350906_G	34G	33G
Z_7350907_T	35T	32T
Z_7350908_C	35C	33C
Z_7350909_T	36T	33T
Z_7350910_C	36C	34C
Z_7350911_C	37C	33C
Z_7350912_G	37G	34G
Z_7350913_G	38G	34G
Z_7350914_T	39T	36T
Z_7350915_T	37T	35T
Z_7350916_G	39G	33G
Z_7350917_C	39C	33C
Z_7350918_T	40T	33T
Z_7350919_A	38A	33A
Z_7350920_G	38G	36G
Z_7350921_G	37G	36G
Z_7350922_C	37C	36C
Z_7350923_A	39A	36A
Z_7350924_A	38A	37A
Z_7350925_C	36C	36C
Z_7350926_C	36C	35C
Z_7350927_C	37C	36C
Z_7350928_C	37C	35C
Z_7350929_T	37T	35T
Z_7350930_C	39C	35C
Z_7350931_C	37C	34C

Z_7350932_C	38C	34C
Z_7350933_A	38A	34A
Z_7350934_A	38A	35A
Z_7350935_C	38C	35C
Z_7350936_C	39C	35C
Z_7350937_C	38C	37C
Z_7350938_C	39C	37C
Z_7350939_A	38A	37A
Z_7350940_T	38T	38T
Z_7350941_G	39G	38G
Z_7350942_C	39C	40C
Z_7350943_A	40A	41A
Z_7350944_G	41G	41G
Z_7350945_C	39C	43C
Z_7350946_C	40C	42C
Z_7350947_C	40C	41C
Z_7350948_T	40T	41T
Z_7350949_G	40G	42G
Z_7350950_G	40G	41G
Z_7350951_G	40G	40G
Z_7350952_G	40G	41G
Z_7350953_G	41G	41G
Z_7350954_A	40A	43A
Z_7350955_G	41G	43G
Z_7350956_G	41G	44G
Z_7350957_T	39T	43T
Z_7350958_C	38C	41C
Z_7350959_T	38T	42T
Z_7350960_C	37C	43C
Z_7350961_T	37T	45T
Z_7350962_T	37T	45T
Z_7350963_A	38A	44A
Z_7350964_G	38G	44G
Z_7350965_C	38C	44C
Z_7350966_A	38A	44A
Z_7350967_A	37A	41A
Z_7350968_C	37C	43C
Z_7350969_T	37T	44T
Z_7350970_T	37T	44T
Z_7350971_G	36A	47G
Z_7350972_G	36G	46G
Z_7350973_G	36G	46G
Z_7350974_G	39G	46G

Z_7350975_G	37G	46G
Z_7350976_A	39A	47A
Z_7350977_C	40C	47C
Z_7350978_T	40T	46T
Z_7350979_G	39G	46G
Z_7350980_C	38C	46C
Z_7350981_T	36T	46T
Z_7350982_T	36T	46T
Z_7350983_C	36C	46C
Z_7350984_C	35C	47C
Z_7350985_T	35T	47T
Z_7350986_T	36T	46T
Z_7350987_T	36T	46T
Z_7350988_T	36T	28T_19C
Z_7350989_G	36G	47G
Z_7350990_G	36G	46G
Z_7350991_C	37C	47C
Z_7350992_C	36C	47C
Z_7350993_C	36C	47C
Z_7350994_C	37C	48C
Z_7350995_T	37T	48T
Z_7350996_C	36C	49C
Z_7350997_T	36T	50T
Z_7350998_A	37A	50A
Z_7350999_A	37A	50A
Z_7351000_G	33G	50G
Z_7351001_A	32A	50A
Z_7351002_T	32T	50T
Z_7351003_G	32G	50G
Z_7351004_A	33A	50A
Z_7351005_T	33T	48T
Z_7351006_C	33C	48C
Z_7351007_C	33C	47C
Z_7351008_T	32T	47T
Z_7351009_T	33T	44T
Z_7351010_G	33G	45G
Z_7351011_C	32C	45C
Z_7351012_A	32A	45A
Z_7351013_C	36C	46C
Z_7351014_T	35T	46T
Z_7351015_T	34T	42T
Z_7351016_T	33C	25T_18C
Z_7351017_G	34G	44G

Z_7351018_G	36G	43G
Z_7351019_T	37T	44T
Z_7351020_G	35G	45G
Z_7351021_A	37A	45A
Z_7351022_G	37G	44G
Z_7351023_T	36T	45T
Z_7351024_T	35T	45T
Z_7351025_T	33T	45T
Z_7351026_C	34C	45C
Z_7351027_G	35G	44G
Z_7351028_G	35G	45G
Z_7351029_T	35T	42T
Z_7351030_G	35G	44G
Z_7351031_A	34A	43A
Z_7351032_A	34A	41A
Z_7351033_A	33A	41A
Z_7351034_A	34A	45A
Z_7351035_G	34G	44G
Z_7351036_T	34T	45T
Z_7351037_T	34T	42T
Z_7351038_C	32C	42C
Z_7351039_C	33C	44C
Z_7351040_T	34T	42T
Z_7351041_T	33T	43T
Z_7351042_C	34C	43C
Z_7351043_C	33T	44C
Z_7351044_G	33G	44G
Z_7351045_G	33G	44G
Z_7351046_G	34G	44G
Z_7351047_A	33A	44A
Z_7351048_C	33C	46C
Z_7351049_C	34T	43C
Z_7351050_T	35T	44T
Z_7351051_T	35T	43T
Z_7351052_C	35C	43C
Z_7351053_T	35T	43T
Z_7351054_G	34G	42G
Z_7351055_C	34C	40C
Z_7351056_T	30T	40T
Z_7351057_C	32C	40C
Z_7351058_C	31C	41C
Z_7351059_C	31C	42C
Z_7351060_A	32A	41A

Z_7351061_G	31G	42G
Z_7351062_A	29A	42A
Z_7351063_A	30A	42A
Z_7351064_T	30T	41T
Z_7351065_T	31T	41T
Z_7351066_C	30C	39C
Z_7351067_C	32C	39C
Z_7351068_C	31C	40C
Z_7351069_C	30C	40C
Z_7351070_A	31A	40A
Z_7351071_G	32G	41G
Z_7351072_C	32C	41C
Z_7351073_C	33C	41C
Z_7351074_A	32A	41A
Z_7351075_G	31G	42G
Z_7351076_G	31G	42G
Z_7351077_G	31G	42G
Z_7351078_T	31T	42T
Z_7351079_C	31C	42C
Z_7351080_A	32A	43A
Z_7351081_C	32C	43C
Z_7351082_C	31C	44C
Z_7351083_T	26T	44T
Z_7351084_G	26G	44G
Z_7351085_T	26T	43T
Z_7351086_G	25G	44G
Z_7351087_G	24G	44G
Z_7351088_G	23G	43G
Z_7351089_G	23G	45G
Z_7351090_C	25C	46C
Z_7351091_A	24A	46A
Z_7351092_T	27T	45T
Z_7351093_T	28T	46T
Z_7351094_C	30C	46C
Z_7351095_T	31T	46T
Z_7351096_C	31C	48C
Z_7351097_G	32G	48G
Z_7351098_G	32G	49G
Z_7351099_G	32G	49G
Z_7351100_G	32G	49G
Z_7351101_T	31T	50T
Z_7351102_C	33C	52C
Z_7351103_A	33A	54A

Z_7351104_T	34T	54T
Z_7351105_A	35A	54A
Z_7351106_T	34T	54T
Z_7351107_G	34G	56G
Z_7351108_C	33C	55C
Z_7351109_C	32C	54C
Z_7351110_C	34C	57C
Z_7351111_A	34A	56A
Z_7351112_A	34A	56A
Z_7351113_A	32A	57A
Z_7351114_A	31A	57A
Z_7351115_A	31A	56A
Z_7351116_A	30A	57A
Z_7351117_A	31A	58A
Z_7351118_G	32G	56G
Z_7351119_G	31G	27T_29G
Z_7351120_A	30A	57A
Z_7351121_A	30A	57A
Z_7351122_C	29C	57C
Z_7351123_T	32T	58T
Z_7351124_C	32C	58C
Z_7351125_G	33G	57G
Z_7351126_G	34G	58G
Z_7351127_C	35C	57C
Z_7351128_C	35C	57C
Z_7351129_A	36A	54A
Z_7351130_A	35A	54A
Z_7351131_G	34G	52G
Z_7351132_C	34C	53C
Z_7351133_C	35C	53C
Z_7351134_C	34C	53C
Z_7351135_T	36T	54T
Z_7351136_G	35G	54G
Z_7351137_C	34C	28T_26C
Z_7351138_G	34G	54G
Z_7351139_A	35A	53A
Z_7351140_G	34G	54G
Z_7351141_G	33G	54G
Z_7351142_T	33T	54T
Z_7351143_T	34T	54T
Z_7351144_G	34G	55G
Z_7351145_G	34G	55G
Z_7351146_C	35C	54C

Z_7351147_C	34C	54C
Z_7351148_A	35A	56A
Z_7351149_G	34G	56G
Z_7351150_A	35A	56A
Z_7351151_A	35A	57A
Z_7351152_G	35G	57G
Z_7351153_G	36G	56G
Z_7351154_T	35T	54T
Z_7351155_T	36T	55T
Z_7351156_T	34T	55T
Z_7351157_T	35T	55T
Z_7351158_T	34T	56T
Z_7351159_C	32C	57C
Z_7351160_T	31T	56T
Z_7351161_C	32C	56C
Z_7351162_T	28T	56T
Z_7351163_G	28G	55G
Z_7351164_C	26C	56C
Z_7351165_A	26A	55A
Z_7351166_T	25T	55T
Z_7351167_C	25C	55C
Z_7351168_A	25A	55A
Z_7351169_G	26G	54G
Z_7351170_G	26G	53G
Z_7351171_G	26G	23A_31G
Z_7351172_T	25T	56T
Z_7351173_C	24C	57C
Z_7351174_A	26A	56A
Z_7351175_G	24G	55G
Z_7351176_A	25A	56A
Z_7351177_G	26G	55G
Z_7351178_G	26G	55G
Z_7351179_G	25G	56G
Z_7351180_A	25A	56A
Z_7351181_C	27C	56C
Z_7351182_G	27G	56G
Z_7351183_G	28G	55G
Z_7351184_A	27A	56A
Z_7351185_C	29C	54C
Z_7351186_T	27T	54T
Z_7351187_C	28C	55C
Z_7351188_A	28A	54A
Z_7351189_T	28T	54T

Z_7351190_C	28C	55C
Z_7351191_A	28A	54A
Z_7351192_G	28G	56G
Z_7351193_C	26C	56C
Z_7351194_C	26C	56C
Z_7351195_C	24C	57C
Z_7351196_T	24T	56T
Z_7351197_T	24T	55T
Z_7351198_A	24A	54A
Z_7351199_A	24A	55A
Z_7351200_A	25A	55A
Z_7351201_C	26C	57C
Z_7351202_C	26C	55C
Z_7351203_C	27C	55C
Z_7351204_T	27T	56T
Z_7351205_G	25G	53G
Z_7351206_T	25T	55T
Z_7351207_A	27A	56A
Z_7351208_C	26C	56C
Z_7351209_A	29A	55A
Z_7351210_A	30A	55A
Z_7351211_A	30A	54A
Z_7351212_T	29T	54T
Z_7351213_T	28T	54T
Z_7351214_T	28T	56T
Z_7351215_G	28G	55G
Z_7351216_G	28G	55G
Z_7351217_A	29A	55A
Z_7351218_C	28C	54C
Z_7351219_T	28T	54T
Z_7351220_T	27T	53T
Z_7351221_C	28C	51C
Z_7351222_T	28T	51T
Z_7351223_C	27C	50C
Z_7351224_C	27C	53C
Z_7351225_C	26C	53C
Z_7351226_C	26C	53C
Z_7351227_C	26C	53C
Z_7351228_C	21T_26C	52C
Z_7351229_T	3A_3T_5C	21A_50T_23C
Z_7351230_C	23C	30C
Z_7351231_A	21C	29A
Z_7351232_C	29C	50C

Z_7351233_C	29C	50C
Z_7351234_A	28A	52A
Z_7351235_C	29C	51C
Z_7351236_C	29C	51C
Z_7351237_A	31A	51A
Z_7351238_T	31T	50T
Z_7351239_T	33T	53T
Z_7351240_C	33C	53C
Z_7351241_T	33T	53T
Z_7351242_G	32G	53G
Z_7351243_C	34C	51C
Z_7351244_C	34C	52C
Z_7351245_C	35C	52C
Z_7351246_A	34A	52A
Z_7351247_A	35A	54A
Z_7351248_A	35A	54A
Z_7351249_C	35C	55C
Z_7351250_C	35C	55C
Z_7351251_C	35C	55C
Z_7351252_T	36T	55T
Z_7351253_C	35C	56C
Z_7351254_T	34T	56T
Z_7351255_A	34A	56A
Z_7351256_T	37T	56T
Z_7351257_G	38G	57G
Z_7351258_T	38T	57T
Z_7351259_G	39G	57G
Z_7351260_G	37G	58G
Z_7351261_G	40G	57G
Z_7351262_T	40T	54T
Z_7351263_C	41C	56C
Z_7351264_T	40T	58T
Z_7351265_C	44C	58C
Z_7351266_C	45C	58C
Z_7351267_C	46C	58C
Z_7351268_T	46T	57T
Z_7351269_A	45A	57A
Z_7351270_T	47T	54T
Z_7351271_A	47A	55A
Z_7351272_T	46T	55T
Z_7351273_G	45G	54G
Z_7351274_G	45G	54G
Z_7351275_G	44G	55G

Z_7351276_G	43G	55G
Z_7351277_G	43G	55G
Z_7351278_G	42G	54G
Z_7351279_C	41C	53C
Z_7351280_A	41A	54A
Z_7351281_T	37T	54T
Z_7351282_C	36C	53C
Z_7351283_A	36A	29A_24G
Z_7351284_G	35G	52G
Z_7351285_A	35A	51A
Z_7351286_A	34A	50A
Z_7351287_G	32G	49G
Z_7351288_G	33G	49G
Z_7351289_A	32A	46A
Z_7351290_A	32A	47A
Z_7351291_C	17T	46C
Z_7351292_G	17G	45G
Z_7351293_C	58C	84C
Z_7351294_C	2C	32C
Z_7351295_G	2C	22G
Z_7351296_C	2C	21C
Z_7351297_C	2C	21C
Z_7351298_C	15C	35C
Z_7351299_C	15C	35C
Z_7351300_C	15C	36C
Z_7351301_C	15C	36C
Z_7351302_C	15C	36C
Z_7351303_C	15T	13T_23C
Z_7351304_A	15C	23A_14C
Z_7351305_G	17G	36G
Z_7351306_T	19T	37T
Z_7351307_T	19T	37T
Z_7351308_G	20C	24G_13C
Z_7351309_G	35G	44G
Z_7351310_A	35A	44A
Z_7351311_C	35C	45C
Z_7351312_A	35A	45A
Z_7351313_T	35T	45T
Z_7351314_C	35C	45C
Z_7351315_A	35A	46A
Z_7351316_G	34G	46G
Z_7351317_G	35G	46G
Z_7351318_C	38C	46C

Z_7351319_C	39C	47C
Z_7351320_T	40T	26T_22C
Z_7351321_C	40C	48C
Z_7351322_C	41C	47C
Z_7351323_T	40T	48T
Z_7351324_C	40C	48C
Z_7351325_A	42A	49A
Z_7351326_A	43G	51A
Z_7351327_G	42G	52G
Z_7351328_C	45C	52C
Z_7351329_C	46C	52C
Z_7351330_C	46C	55C
Z_7351331_T	44T	54T
Z_7351332_T	42T	56T
Z_7351333_A	42A	57A
Z_7351334_A	41A	55A
Z_7351335_C	42A	25A_32C
Z_7351336_C	40C	57C
Z_7351337_A	41A	57A
Z_7351338_A	40A	58A
Z_7351339_A	41A	57A
Z_7351340_G	41G	56G
Z_7351341_G	40G	54G
Z_7351342_C	40C	53C
Z_7351343_A	40A	53A
Z_7351344_G	40G	51G
Z_7351345_T	39T	52T
Z_7351346_T	40T	52T
Z_7351347_T	42T	52T
Z_7351348_C	40C	53C
Z_7351349_C	41C	54C
Z_7351350_A	40A	54A
Z_7351351_A	40A	54A
Z_7351352_A	40A	54A
Z_7351353_C	38C	55C
Z_7351354_C	37C	54C
Z_7351355_A	37A	53A
Z_7351356_C	36C	53C
Z_7351357_A	36A	53A
Z_7351358_T	36T	56T
Z_7351359_T	37T	57T
Z_7351360_C	36C	57C
Z_7351361_A	36A	57A

Z_7351362_A	37A	61A
Z_7351363_G	37G	60G
Z_7351364_G	36G	58G
Z_7351365_G	37G	60G
Z_7351366_C	36C	60C
Z_7351367_A	35A	59A
Z_7351368_G	34G	62G
Z_7351369_A	33A	62A
Z_7351370_C	34C	62C
Z_7351371_C	34C	63C
Z_7351372_C	35C	63C
Z_7351373_C	35C	64C
Z_7351374_A	35A	64A
Z_7351375_G	32G	63G
Z_7351376_G	30G	63G
Z_7351377_A	31A	63A
Z_7351378_G	31G	64G
Z_7351379_G	29G	67G
Z_7351380_A	30A	65A
Z_7351381_C	29C	66C
Z_7351382_T	30T	67T
Z_7351383_C	30C	65C
Z_7351384_T	32T	67T
Z_7351385_G	32G	68G
Z_7351386_C	32C	68C
Z_7351387_T	32T	68T
Z_7351388_G	29G	68G
Z_7351389_C	28C	69C
Z_7351390_A	28A	68A
Z_7351391_G	30G	68G
Z_7351392_T	30T	71T
Z_7351393_A	30A	71A
Z_7351394_G	31G	71G
Z_7351395_T	30T	71T
Z_7351396_C	30C	71C
Z_7351397_T	30T	71T
Z_7351398_A	29A	71A
Z_7351399_T	30T	69T
Z_7351400_G	31G	69G
Z_7351401_C	31C	69C
Z_7351402_T	31T	69T
Z_7351403_C	32C	71C
Z_7351404_G	32G	69G

Z_7351405_G	33G	68G
Z_7351406_A	33A	69A
Z_7351407_C	33C	68C
Z_7351408_C	34C	70C
Z_7351409_G	32G	70G
Z_7351410_G	32G	69G
Z_7351411_A	33A	68A
Z_7351412_C	34C	69C
Z_7351413_C	35C	69C
Z_7351414_A	36A	69A
Z_7351415_G	35G	68G
Z_7351416_G	34G	70G
Z_7351417_G	33G	70G
Z_7351418_G	34G	69G
Z_7351419_G	35G	70G
Z_7351420_C	33C	68C
Z_7351421_T	36T	66T
Z_7351422_G	36G	67G
Z_7351423_A	36A	68A
Z_7351424_A	37A	66A
Z_7351425_T	37T	64T
Z_7351426_C	37C	64C
Z_7351427_A	38A	63A
Z_7351428_G	38G	62G
Z_7351429_G	36G	62G
Z_7351430_G	37G	32G_30C
Z_7351431_A	38A	62A
Z_7351432_G	38G	63G
Z_7351433_G	40G	63G
Z_7351434_T	40T	63T
Z_7351435_T	40T	62T
Z_7351436_A	39A	63A
Z_7351437_A	39A	62A
Z_7351438_A	40A	61A
Z_7351439_C	40C	62C
Z_7351440_T	39T	61T
Z_7351441_G	41G	62G
Z_7351442_A	39A	61A
Z_7351443_G	40G	61G
Z_7351444_C	38C	61C
Z_7351445_T	40T	61T
Z_7351446_G	43G	61G
Z_7351447_A	42A	60A

Z_7351448_A	44A	60A
Z_7351449_G	44G	61G
Z_7351450_C	45C	62C
Z_7351451_T	46T	62T
Z_7351452_A	46A	63A
Z_7351453_A	46A	61A
Z_7351454_G	46G	60G
Z_7351455_A	45A	60A
Z_7351456_A	49A	63A
Z_7351457_T	48T	60T
Z_7351458_A	49A	64A
Z_7351459_C	47C	64C
Z_7351460_A	49A	64A
Z_7351461_T	48T	63T
Z_7351462_C	50C	63C
Z_7351463_A	51A	62A
Z_7351464_G	50G	61G
Z_7351465_G	50G	61G
Z_7351466_A	49A	60A
Z_7351467_T	49T	61T
Z_7351468_G	50G	61G
Z_7351469_T	49T	60T
Z_7351470_G	48G	60G
Z_7351471_T	47T	61T
Z_7351472_G	49G	63G
Z_7351473_T	48T	64T
Z_7351474_G	49G	62G
Z_7351475_G	47G	63G
Z_7351476_A	47A	62A
Z_7351477_A	47A	62A
Z_7351478_G	47G	62G
Z_7351479_G	47G	61G
Z_7351480_G	45G	60G
Z_7351481_G	45G	60G
Z_7351482_A	46A	60A
Z_7351483_T	44T	61T
Z_7351484_A	44A	60A
Z_7351485_C	43A	61C
Z_7351486_G	42G	62G
Z_7351487_T	42T	61T
Z_7351488_T	41T	60T
Z_7351489_C	39C	61C
Z_7351490_C	40C	61C

Z_7351491_T	40T	59T
Z_7351492_C	40C	60C
Z_7351493_T	41T	61T
Z_7351494_T	40T	61T
Z_7351495_T	41T	61T
Z_7351496_G	41G	61G
Z_7351497_C	41C	61C
Z_7351498_A	42A	59A
Z_7351499_G	43G	59G
Z_7351500_C	43C	60C
Z_7351501_C	44C	59C
Z_7351502_C	41C	59C
Z_7351503_C	41C	61C
Z_7351504_T	40T	60T
Z_7351505_C	41C	61C
Z_7351506_C	41C	62C
Z_7351507_G	42G	62G
Z_7351508_G	41G	62G
Z_7351509_A	42A	61A
Z_7351510_T	42T	62T
Z_7351511_T	40T	61T
Z_7351512_G	40G	62G
Z_7351513_G	40G	63G
Z_7351514_G	40G	62G
Z_7351515_C	39C	62C
Z_7351516_C	38C	62C
Z_7351517_C	37C	61C
Z_7351518_C	37C	60C
Z_7351519_T	36T	60T
Z_7351520_C	35C	60C
Z_7351521_T	37T	60T
Z_7351522_T	38T	59T
Z_7351523_C	39C	60C
Z_7351524_C	38C	60C
Z_7351525_C	38C	59C
Z_7351526_A	35A	58A
Z_7351527_G	36G	57G
Z_7351528_C	36C	56C
Z_7351529_T	36T	56T
Z_7351530_G	35G	56G
Z_7351531_C	33C	56C
Z_7351532_T	33T	60T
Z_7351533_C	32G	25G_35C

Z_7351534_T	32T	60T
Z_7351535_C	31C	60C
Z_7351536_A	31A	61A
Z_7351537_A	31A	61A
Z_7351538_G	29G	60G
Z_7351539_T	29C	37T_23C
Z_7351540_A	29A	59A
Z_7351541_T	29T	59T
Z_7351542_C	28C	58C
Z_7351543_C	28C	58C
Z_7351544_A	26A	57A
Z_7351545_G	26G	56G
Z_7351546_T	25T	56T
Z_7351547_T	25T	55T
Z_7351548_G	25G	54G
Z_7351549_C	24C	55C
Z_7351550_T	23T	54T
Z_7351551_T	23T	53T
Z_7351552_A	23A	51A
Z_7351553_G	23G	48G
Z_7351554_A	23A	49A
Z_7351555_A	25A	49A
Z_7351556_A	26A	48A
Z_7351557_G	26G	50G
Z_7351558_C	26C	48C
Z_7351559_G	27A	48G
Z_7351560_C	26C	48C
Z_7351561_C	25C	48C
Z_7351562_T	25T	47T
Z_7351563_C	24C	49C
Z_7351564_C	23C	51C
Z_7351565_A	24A	54A
Z_7351566_G	26G	53G
Z_7351567_C	25C	51C
Z_7351568_A	24A	50A
Z_7351569_G	25G	50G
Z_7351570_A	25A	49A
Z_7351571_T	25T	49T
Z_7351572_G	26G	50G
Z_7351573_G	25G	52G
Z_7351574_G	27G	52G
Z_7351575_G	27G	53G
Z_7351576_G	28G	54G

Z_7351577_T	27T	55T
Z_7351578_G	28G	56G
Z_7351579_G	28G	55G
Z_7351580_C	28C	55C
Z_7351581_C	28C	54C
Z_7351582_C	29C	53C
Z_7351583_T	29T	53T
Z_7351584_T	27T	51T
Z_7351585_C	27C	51C
Z_7351586_C	25C	51C
Z_7351587_A	25A	50A
Z_7351588_G	26G	49G
Z_7351589_A	27A	50A
Z_7351590_C	27C	51C
Z_7351591_A	26A	51A
Z_7351592_T	24T	52T
Z_7351593_A	24A	53A
Z_7351594_G	24G	53G
Z_7351595_G	23G	52G
Z_7351596_A	23A	54A
Z_7351597_G	23G	56G
Z_7351598_A	24A	52A
Z_7351599_A	24A	52A
Z_7351600_A	24A	53A
Z_7351601_A	24A	53A
Z_7351602_G	21G	53G
Z_7351603_C	22G	18G_35C
Z_7351604_C	22C	53C
Z_7351605_A	22A	52A
Z_7351606_G	22G	51G
Z_7351607_T	21T	50T
Z_7351608_C	22C	49C
Z_7351609_T	22T	49T
Z_7351610_C	21C	50C
Z_7351611_C	22C	51C
Z_7351612_C	21C	48C
Z_7351613_C	21C	49C
Z_7351614_A	18C	48A
Z_7351615_C	18C	48C
Z_7351616_C	17T	49C
Z_7351617_A	18C	36A_13C
Z_7351618_C	19C	49C
Z_7351619_C	19C	50C

Z_7351620_A	19A	50A
Z_7351621_C	18T	50C
Z_7351622_C	19C	49C
Z_7351623_A	19G	37A_13G
Z_7351624_C	28C	51C
Z_7351625_C	27C	51C
Z_7351626_T	28T	51T
Z_7351627_C	29C	47C
Z_7351628_C	29C	47C
Z_7351629_A	30A	48A
Z_7351630_A	30A	48A
Z_7351631_C	30C	47C
Z_7351632_A	31A	47A
Z_7351633_A	31A	48A
Z_7351634_C	33C	46C
Z_7351635_A	33A	46A
Z_7351636_A	31A	46A
Z_7351637_G	30G	46G
Z_7351638_G	29G	48G
Z_7351639_G	30G	49G
Z_7351640_G	31G	49G
Z_7351641_G	29G	49G
Z_7351642_A	28A	35A_15G
Z_7351643_A	28A	50A
Z_7351644_A	26A	49A
Z_7351645_A	26A	50A
Z_7351646_A	25A	50A
Z_7351647_A	24A	49A
Z_7351648_C	24C	49C
Z_7351649_C	22C	50C
Z_7351650_C	22C	13T_34C
Z_7351651_T	23T	46T
Z_7351652_C	22C	47C
Z_7351653_C	20C	47C
Z_7351654_C	23C	47C
Z_7351655_T	24T	48T
Z_7351656_G	25G	48G
Z_7351657_G	24G	49G
Z_7351658_A	23A	47A
Z_7351659_C	23C	45C
Z_7351660_A	24A	43A
Z_7351661_T	25T	44T
Z_7351662_C	25C	44C

Z_7351663_A	26A	45A
Z_7351664_G	26G	45G
Z_7351665_G	26G	45G
Z_7351666_A	26A	45A
Z_7351667_C	26C	44C
Z_7351668_T	25T	42T
Z_7351669_G	25G	42G
Z_7351670_T	25T	41T
Z_7351671_T	24T	40T
Z_7351672_C	25C	39C
Z_7351673_G	25G	37G
Z_7351674_G	25C	26G_11C
Z_7351675_C	25C	37C
Z_7351676_T	24T	36T
Z_7351677_T	24T	35T
Z_7351678_T	23T	36T
Z_7351679_T	22T	36T
Z_7351680_T	22T	36T
Z_7351681_T	22T	36T
Z_7351682_A	22A	36A
Z_7351683_G	22G	36G
Z_7351684_A	22A	35A
Z_7351685_G	21G	34G
Z_7351686_G	21G	34G
Z_7351687_G	18G	34G
Z_7351688_T	17T	32T
Z_7351689_G	16G	32G
Z_7351690_C	15C	32C
Z_7351691_C	15C	31C
Z_7351692_A	15A	24A
Z_7351693_G	14G	26G
Z_7351694_G	14G	26G
Z_7351695_T	2C	25T
Z_7351696_A	2A	25A
Z_7351697_G	1A	24G
Z_7351698_G	1G_4C	25G
Z_7351699_T	2C	24T
Z_7351700_C	3C	25C
Z_7351701_C	3C	25C
Z_7351702_C	3C	25C
Z_7351703_C	3C	25C
Z_7351704_T	3C	24T
Z_7351705_G	4G	33G

Z_7351706_C	4C	32C
Z_7351707_A	4A	32A
Z_7351708_G	4G	33G
Z_7351709_G	5G	33G
Z_7351710_A	5A	33A
Z_7351711_G	6G	34G
Z_7351712_G	6G	34G
Z_7351713_A	6A	33A
Z_7351714_G	6G	33G
Z_7351715_A	7A	32A
Z_7351716_A	7A	32A
Z_7351717_A	7A	32A
Z_7351718_G	7G	32G
Z_7351719_G	7G	32G
Z_7351720_A	7A	32A
Z_7351721_G	8G	32G
Z_7351722_G	8G	33G
Z_7351723_A	9A	34A
Z_7351724_A	9G	21A_12G
Z_7351725_G	8G	33G
Z_7351726_G	8G	33G
Z_7351727_G	7G	33G
Z_7351728_G	8G	33G
Z_7351729_G	8G	33G
Z_7351730_T	8T	32T
Z_7351731_G	8G	34G
Z_7351732_A	8A	33A
Z_7351733_C	8C	33C
Z_7351734_T	8T	32T
Z_7351735_T	9T	32T
Z_7351736_T	10T	32T
Z_7351737_G	10G	32G
Z_7351738_C	10C	32C
Z_7351739_C	11C	32C
Z_7351740_A	11A	33A
Z_7351741_G	11G	33G
Z_7351742_C	11C	34C
Z_7351743_C	11C	34C
Z_7351744_A	11A	33A
Z_7351745_G	10G	34G
Z_7351746_C	10C	34C
Z_7351747_C	10C	33C
Z_7351748_T	10T	33T

Z_7351749_A	11A	32A
Z_7351750_G	11G	31G
Z_7351751_C	11C	32C
Z_7351752_G	11G	31G
Z_7351753_A	11A	30A
Z_7351754_C	11C	30C
Z_7351755_T	11T	28T
Z_7351756_C	11C	27C
Z_7351757_C	11C	27C
Z_7351758_C	11C	26C
Z_7351759_A	10A	25A
Z_7351760_C	11C	24C
Z_7351761_A	11A	25A
Z_7351762_C	11C	25C
Z_7351763_G	11A	25G
Z_7351764_T	12T	24T
Z_7351765_C	12C	25C
Z_7351766_A	12A	25A
Z_7351767_G	12G	25G
Z_7351768_G	12G	25G
Z_7351769_T	11T	24T
Z_7351770_T	10T	25T
Z_7351771_G	10G	25G
Z_7351772_G	10G	25G
Z_7351773_G	10G	24G
Z_7351774_C	10C	24C
Z_7351775_G	10G	24G
Z_7351776_G	10G	22G
Z_7351777_A	10A	23A
Z_7351778_G	10G	23G
Z_7351779_G	9G	23G
Z_7351780_G	9G	23G
Z_7351781_A	8A	23A
Z_7351782_C	8C	24C
Z_7351783_C	8C	24C
Z_7351784_C	8C	24C
Z_7351785_A	7A	23A
Z_7351786_A	7A	22A
Z_7351787_A	7A	21A
Z_7351788_T	7T	19T
Z_7351789_C	7C	18C
Z_7351790_A	6A	16A
Z_7351791_T	6T	16T

Z_7351792_G	6G	16G
Z_7351793_G	6G	16G
Z_7351794_G	5G	16G
Z_7351795_C	1G_4C	16C
Z_7351796_T	1A_4T	16T
Z_7351797_C	5C	16C
Z_7351798_C	5A	6G_10C
Z_7351799_C	5C	15C
Z_7351800_C	6C	15C
Z_7351801_T	5T	15T
Z_7351802_C	6C	15C
Z_7351803_C	6C	15C
Z_7351804_C	6C	15C
Z_7351805_A	6A	15A
Z_7351806_G	5G	15G
Z_7351807_G	4G	14G
Z_7351808_C	4C	14C
Z_7351809_A	4A	15A
Z_7351810_G	3G	15G
Z_7351811_G	3G	15G
Z_7351812_C	3C	15C
Z_7351813_A	3A	14A
Z_7351814_G	3G	14G
Z_7351815_C	3C	14C
Z_7351816_T	3T	13T
Z_7351817_G	4G	13G
Z_7351818_T	4T	12T
Z_7351819_C	4C	12C
Z_7351820_T	3T	12T
Z_7351821_T	3T	11T
Z_7351822_G	3G	11G
Z_7351823_G	3G	11G
Z_7351824_C	3C	11C
Z_7351825_C	3C	11C
Z_7351826_T	3T	9T
Z_7351827_G	3G	9G
Z_7351828_C	3C	9C
Z_7351829_A	3A	8A
Z_7351830_A	3A	8A
Z_7351831_A	3A	8A
Z_7351832_C	3C	8C
Z_7351833_A	3A	8A
Z_7351834_A	3A	8A

Z_7351835_G	1G_1C	8G
Z_7351836_C	2C	7C
Z_7351837_T	2T	6T
Z_7351838_T	2T	6T
Z_7351839_C	2C	6C
Z_7351840_T	2T_2C	6T
Z_7351841_C	2C	5C
Z_7351842_C	2C	5C
Z_7351843_C	2C	1T_4C
Z_7351844_C	2C	5C
Z_7351845_C	2C	5C
Z_7351846_C	2C	5C
Z_7351847_C	2C	5C
Z_7351848_C	2C	5C
Z_7351849_C	2C	5C
Z_7351850_C	2C	5C
Z_7351851_A	2A	5A
Z_7351852_C	2C	5C
Z_7351853_A	2A	5A
Z_7351854_T	2T	5T
Z_7351855_G	2G	5G
Z_7351856_C	2C	5C
Z_7351857_G	2G	5G
Z_7351858_T	2C	4T_1C
Z_7351859_C	2C	5C
Z_7351860_C	2C	5C
Z_7351861_T	2T	5T
Z_7351862_G	2G	5G
Z_7351863_A	2A	5A
Z_7351864_C	2C	6C
Z_7351865_T	2T	6T
Z_7351866_C	2C	6C
Z_7351867_A	3A	6A
Z_7351868_G	3G	6G
Z_7351869_G	2G	6G
Z_7351870_T	2T	5T
Z_7351871_G	2G	6G
Z_7351872_A	2A	6A
Z_7351873_A	2A	5A
Z_7351874_G	3G	5G
Z_7351875_G	3G	5G
Z_7351876_A	3A	5A
Z_7351877_G	2G	4G

Z_7351878_A	2A	4A
Z_7351879_G	2G	4G
Z_7351880_G	2G	4G
Z_7351881_G	2G	3G
Z_7351882_G	2G	3G
Z_7351883_T	2T	3T
Z_7351884_A	2A	3A
Z_7351885_C	2C	2C
Z_7351886_A	2A	2A
Z_7351887_A	2A	2A
Z_7351888_A	2A	2A
Z_7351889_C	2C	2C
Z_7351890_C	2C	3C
Z_7351891_G	2G	3G
Z_7351892_G	2G	3G
Z_7351893_C	2C	3C
Z_7351894_T	2T_2C	3T
Z_7351895_C	2C	3C
Z_7351896_C	2C	1G_2C
Z_7351897_C	2C	3C
Z_7351898_C	2C	1A_2C
Z_7351899_C	2C	3C
Z_7351900_C	2C	3C
Z_7351901_C	2C	3C
Z_7351902_C	2C	3C
Z_7351903_C	2C	3C
Z_7351904_C	2C	2C
Z_7351905_C	2C	3C
Z_7351906_G	2C	3A_1G
Z_7351907_G	1G_1C	4G
Z_7351908_T	2T	4T
Z_7351909_T	2T	4T
Z_7351910_T	2T	4T
Z_7351911_G	2G	5G
Z_7351912_T	2T	5T
Z_7351913_T	2T	6T
Z_7351914_C	2C	6C
Z_7351915_T	3T	6T
Z_7351916_G	3G	7G
Z_7351917_T	5T	7T
Z_7351918_G	5G	7G
Z_7351919_T	5T	7T
Z_7351920_T	5T	7T

Z_7351921_T	5T	8T
Z_7351922_T	5T	8T
Z_7351923_G	5G	8G
Z_7351924_G	6G	9G
Z_7351925_C	6C	9C
Z_7351926_T	7T	11T
Z_7351927_T	7T	12T
Z_7351928_C	8C	14C
Z_7351929_G	8G	14G
Z_7351930_A	6A_1G_1C	14A
Z_7351931_T	8T	15T
Z_7351932_T	8T	15T
Z_7351933_T	8T	17T
Z_7351934_T	8T	18T
Z_7351935_C	8C	18C
Z_7351936_C	7C	19C
Z_7351937_C	7C	20C
Z_7351938_T	7T	21T
Z_7351939_A	8A	21A
Z_7351940_T	9T	21T
Z_7351941_C	9C	22C
Z_7351942_A	9A	22A
Z_7351943_G	9G	23G
Z_7351944_C	9C	25C
Z_7351945_T	9T	28T
Z_7351946_T	10T	29T
Z_7351947_G	10G	29G
Z_7351948_G	10G	29G
Z_7351949_A	11A	29A
Z_7351950_A	11A	29A
Z_7351951_G	11G	30G
Z_7351952_C	11C	30C
Z_7351953_C	11C	33C
Z_7351954_A	12A	33A
Z_7351955_A	12A	33A
Z_7351956_G	12G	33G
Z_7351957_C	12C	33C
Z_7351958_C	14C	33C
Z_7351959_A	15G	19A_13G
Z_7351960_G	15G	32G
Z_7351961_A	15A	33A
Z_7351962_A	15A	34A
Z_7351963_A	16A	36A

Z_7351964_G	16G	36G
Z_7351965_A	15A	37A
Z_7351966_T	16T	37T
Z_7351967_G	15G	37G
Z_7351968_A	16A	38A
Z_7351969_T	16T	39T
Z_7351970_T	18T	40T
Z_7351971_C	18C	40C
Z_7351972_A	18G	22A_19G
Z_7351973_G	19G	43G
Z_7351974_A	20A	43A
Z_7351975_T	20T	43T
Z_7351976_T	21T	43T
Z_7351977_T	21T	44T
Z_7351978_T	21T	45T
Z_7351979_A	22A	45A
Z_7351980_T	22T	46T
Z_7351981_G	22G	46G
Z_7351982_A	23A	46A
Z_7351983_A	22A	46A
Z_7351984_A	24A	46A
Z_7351985_C	23C	45C
Z_7351986_A	24A	45A
Z_7351987_C	22C	45C
Z_7351988_A	23A	46A
Z_7351989_C	24C	46C
Z_7351990_A	24A	46A
Z_7351991_C	24C	46C
Z_7351992_A	24A	46A
Z_7351993_C	24C	47C
Z_7351994_A	24A	47A
Z_7351995_C	24C	47C
Z_7351996_A	23A	47A
Z_7351997_C	23C	47C
Z_7351998_C	22T	21T_27C
Z_7351999_G	22G	48G
Z_7352000_G	24G	47G
Z_7352001_A	22A	64A
Z_7352002_A	23A	28A
Z_7352003_A	25A	49A
Z_7352004_A	26A	49A
Z_7352005_A	26A	49A
Z_7352006_A	26A	49A

Z_7352007_A	26A	49A
Z_7352008_A	27A	49A
Z_7352009_A	26A	49A
Z_7352010_A	25A	51A
Z_7352011_C	26C	51C
Z_7352012_C	26C	51C
Z_7352013_T	26T	51T
Z_7352014_T	25T	51T
Z_7352015_C	27C	51C
Z_7352016_A	27G	51A
Z_7352017_C	26C	51C
Z_7352018_C	27C	51C
Z_7352019_A	27A	50A
Z_7352020_A	26A	50A
Z_7352021_G	26G	48G
Z_7352022_G	27G	47G
Z_7352023_A	27A	45A
Z_7352024_A	27A	45A
Z_7352025_G	26G	46G
Z_7352026_C	25C	46C
Z_7352027_T	26T	46T
Z_7352028_T	26T	44T
Z_7352029_A	25A	44A
Z_7352030_G	23G	43G
Z_7352031_G	23G	42G
Z_7352032_A	23A	42A
Z_7352033_G	25G	43G
Z_7352034_C	25C	43C
Z_7352035_A	24A	44A
Z_7352036_A	25A	45A
Z_7352037_A	25A	45A
Z_7352038_G	26G	43G
Z_7352039_C	26C	19T_22C
Z_7352040_C	27C	41C
Z_7352041_C	25C	41C
Z_7352042_A	25A	39A
Z_7352043_G	25G	39G
Z_7352044_C	25C	39C
Z_7352045_T	26T	39T
Z_7352046_G	27G	39G
Z_7352047_C	27C	39C
Z_7352048_G	27G	38G
Z_7352049_A	28A	38A

Z_7352050_T	27T	36T
Z_7352051_T	26T	35T
Z_7352052_A	27A	35A
Z_7352053_T	26T	35T
Z_7352054_T	26T	35T
Z_7352055_T	25T	35T
Z_7352056_C	26C	35C
Z_7352057_C	26C	34C
Z_7352058_G	26G	33G
Z_7352059_G	26G	35G
Z_7352060_C	26C	33C
Z_7352061_C	25C	33C
Z_7352062_C	26C	33C
Z_7352063_C	26C	33C
Z_7352064_C	26C	34C
Z_7352065_C	27C	33C
Z_7352066_G	27A	15A_17G_1C
Z_7352067_C	27C	32C
Z_7352068_C	27C	34C
Z_7352069_C	29C	34C
Z_7352070_G	27G	32G
Z_7352071_G	27G	32G
Z_7352072_C	27C	31C
Z_7352073_T	28T	31T
Z_7352074_C	28C	31C
Z_7352075_A	28A	30A
Z_7352076_A	28A	32A
Z_7352077_T	28T	32T
Z_7352078_C	27C	33C
Z_7352079_T	28T	33T
Z_7352080_A	28A	33A
Z_7352081_G	29G	35G
Z_7352082_A	29A	18A_17G
Z_7352083_G	31G	35G
Z_7352084_C	31C	35C
Z_7352085_C	29C	37C
Z_7352086_C	29C	36C
Z_7352087_A	28A	38A
Z_7352088_A	28A	36A
Z_7352089_G	26G	37G
Z_7352090_G	27G	37G
Z_7352091_G	28G	38G
Z_7352092_A	24A	38A

Z_7352093_T	27T	35T
Z_7352094_G	28G	37G
Z_7352095_C	29C	36C
Z_7352096_C	28C	37C
Z_7352097_C	30C	38C
Z_7352098_C	31C	38C
Z_7352099_A	30A	37A
Z_7352100_G	31G	39G
Z_7352101_G	31G	38G
Z_7352102_C	32C	38C
Z_7352103_T	30T	38T
Z_7352104_G	29G	40G
Z_7352105_A	29A	37A
Z_7352106_G	29G	38G
Z_7352107_T	28T	38T
Z_7352108_C	30C	38C
Z_7352109_C	30T	38C
Z_7352110_C	28C	39C
Z_7352111_T	29T	39T
Z_7352112_C	30C	39C
Z_7352113_G	30G	38G
Z_7352114_C	29C	39C
Z_7352115_G	28G	39G
Z_7352116_G	27G	39G
Z_7352117_C	26C	39C
Z_7352118_G	26G	39G
Z_7352119_C	27C	40C
Z_7352120_G	27G	41G
Z_7352121_A	29A	42A
Z_7352122_A	29A	42A
Z_7352123_A	30A	44A
Z_7352124_A	30A	44A
Z_7352125_G	32G	44G
Z_7352126_C	31C	44C
Z_7352127_A	30A	43A
Z_7352128_C	29C	41C
Z_7352129_T	29T	43T
Z_7352130_G	28G	42G
Z_7352131_A	28A	42A
Z_7352132_G	30G	22A_20G
Z_7352133_T	31T	42T
Z_7352134_T	30T	42T
Z_7352135_A	30A	41A

Z_7352136_T	30T	41T
Z_7352137_T	30T	43T
Z_7352138_T	31T	43T
Z_7352139_T	29T	43T
Z_7352140_T	30T	43T
Z_7352141_A	28A	43A
Z_7352142_T	31T	44T
Z_7352143_T	29T	43T
Z_7352144_T	28T	43T
Z_7352145_A	28A	43A
Z_7352146_T	30T	43T
Z_7352147_T	31T	44T
Z_7352148_C	31C	44C
Z_7352149_A	31A	44A
Z_7352150_T	29T	44T
Z_7352151_T	28T	45T
Z_7352152_G	29G	45G
Z_7352153_G	30G	45G
Z_7352154_A	30A	45A
Z_7352155_A	32A	45A
Z_7352156_C	32C	45C
Z_7352157_A	33A	44A
Z_7352158_A	33A	31A
Z_7352159_A	34A	31A
Z_7352160_G	34G	18A_24T_37G_6C
Z_7352161_A	34A	26A
Z_7352162_G	34G	25G
Z_7352163_T	35T	24T
Z_7352164_T	37T	25T
Z_7352165_C	34C	25C
Z_7352166_A	38A	26A
Z_7352167_A	39A	27A
Z_7352168_T	38T	27T
Z_7352169_T	39T	27T
Z_7352170_T	38T	33T
Z_7352171_A	41A	32A
Z_7352172_T	41T	32T
Z_7352173_T	42T	32T
Z_7352174_T	43T	24A_32T_18G_30C
Z_7352175_A	43A	26A
Z_7352176_C	42C	24C
Z_7352177_C	44C	25C
Z_7352178_G	42G	24G

Z_7352179_G	45G	24G
Z_7352180_A	46A	24A
Z_7352181_G	47G	24G
Z_7352182_C	47C	25C
Z_7352183_A	47A	25A
Z_7352184_C	47C	25C
Z_7352185_A	48A	24A
Z_7352186_C	49T	26C
Z_7352187_T	47T	54T
Z_7352188_C	49C	54C
Z_7352189_C	48C	54C
Z_7352190_A	48A	54A
Z_7352191_T	47T	54T
Z_7352192_A	48A	52A
Z_7352193_C	46C	52C
Z_7352194_T	47T	53T
Z_7352195_T	46T	53T
Z_7352196_T	46T	53T
Z_7352197_A	48A	55A
Z_7352198_C	48C	55C
Z_7352199_T	49T	55T
Z_7352200_G	51G	56G
Z_7352201_T	50C	56T
Z_7352202_A	50A	57A
Z_7352203_A	49A	59A
Z_7352204_T	53T	60T
Z_7352205_G	52G	60G
Z_7352206_T	53T	64T
Z_7352207_A	53A	65A
Z_7352208_G	53G	65G
Z_7352209_G	53G	65G
Z_7352210_A	52A	67A
Z_7352211_A	52A	67A
Z_7352212_A	52A	67A
Z_7352213_G	54G	68G
Z_7352214_G	54G	68G
Z_7352215_A	54A	68A
Z_7352216_C	51C	69C
Z_7352217_T	52T	70T
Z_7352218_G	53G	70G
Z_7352219_T	53T	70T
Z_7352220_T	54T	70T
Z_7352221_C	53C	70C

Z_7352222_C	50C	70C
Z_7352223_T	51T	72T
Z_7352224_T	50T	70T
Z_7352225_C	49C	70C
Z_7352226_A	49A	70A
Z_7352227_T	51T	70T
Z_7352228_T	51T	70T
Z_7352229_T	50T	70T
Z_7352230_C	50C	72C
Z_7352231_T	51T	70T
Z_7352232_G	51G	40A_30G
Z_7352233_C	51C	70C
Z_7352234_G	48G	70G
Z_7352235_T	48T	70T
Z_7352236_A	48A	72A
Z_7352237_A	46A	72A
Z_7352238_T	46T	72T
Z_7352239_G	45G	73G
Z_7352240_G	44G	71G
Z_7352241_G	41G	71G
Z_7352242_G	42G	71G
Z_7352243_A	40A	67A
Z_7352244_G	43G	31G_32C
Z_7352245_A	41A	33A_31G
Z_7352246_C	43C	63C
Z_7352247_A	43A	64A
Z_7352248_C	45C	64C
Z_7352249_A	44A	63A
Z_7352250_G	43G	64G
Z_7352251_A	42A	63A
Z_7352252_A	42A	62A
Z_7352253_T	42T	66T
Z_7352254_G	41G	66G
Z_7352255_G	39G	65G
Z_7352256_T	43T	63T
Z_7352257_G	43G	63G
Z_7352258_C	42C	64C
Z_7352259_C	42C	62C
Z_7352260_T	43T	63T
Z_7352261_A	43A	62A
Z_7352262_T	42T	61T
Z_7352263_G	43G	62G
Z_7352264_A	42A	63A

Z_7352265_A	42A	66A
Z_7352266_T	42T	66T
Z_7352267_A	40A	67A
Z_7352268_T	41T	67T
Z_7352269_A	39A	68A
Z_7352270_C	37C	69C
Z_7352271_T	40T	69T
Z_7352272_T	40T	68T
Z_7352273_G	41G	69G
Z_7352274_G	38G	70G
Z_7352275_T	38T	71T
Z_7352276_T	38T	72T
Z_7352277_T	39T	69T
Z_7352278_A	39A	70A
Z_7352279_T	41T	70T
Z_7352280_T	41T	70T
Z_7352281_T	41T	68T
Z_7352282_T	40T	67T
Z_7352283_T	38T	68T
Z_7352284_C	38C	70C
Z_7352285_C	38C	70C
Z_7352286_G	38G	72G
Z_7352287_G	37G	72G
Z_7352288_A	35A	72A
Z_7352289_C	38C	71C
Z_7352290_A	38A	71A
Z_7352291_G	37G	71G
Z_7352292_C	38C	68C
Z_7352293_A	37A	68A
Z_7352294_T	38A	67T
Z_7352295_A	38A	66A
Z_7352296_A	39A	67A
Z_7352297_T	38T	67T
Z_7352298_T	39T	66T
Z_7352299_T	39T	65T
Z_7352300_C	39C	66C
Z_7352301_A	38A	62A
Z_7352302_A	38A	61A
Z_7352303_A	39A	62A
Z_7352304_A	41A	62A
Z_7352305_T	41T	60T
Z_7352306_G	42G	60G
Z_7352307_A	41A	60A

Z_7352308_A	41A	59A
Z_7352309_A	42A	59A
Z_7352310_C	42C	60C
Z_7352311_T	40T	60T
Z_7352312_T	40T	60T
Z_7352313_A	41A	62A
Z_7352314_A	39A	62A
Z_7352315_T	39T	61T
Z_7352316_T	40T	59T
Z_7352317_A	39A	60A
Z_7352318_T	37T	58T
Z_7352319_T	37T	59T
Z_7352320_A	38A	59A
Z_7352321_G	39G	58G
Z_7352322_T	40T	57T
Z_7352323_C	41C	57C
Z_7352324_C	41C	57C
Z_7352325_A	41A	56A
Z_7352326_T	38T	57T
Z_7352327_T	38T	55T
Z_7352328_T	38T	55T
Z_7352329_T	39T	55T
Z_7352330_G	37G	54G
Z_7352331_G	37G	52G
Z_7352332_C	38C	50C
Z_7352333_A	38A	51A
Z_7352334_G	38G	28G_22C
Z_7352335_G	38G	51G
Z_7352336_G	38G	54G
Z_7352337_C	38C	54C
Z_7352338_C	39C	56C
Z_7352339_A	39A	56A
Z_7352340_G	39G	54G
Z_7352341_C	36C	54C
Z_7352342_T	36T	53T
Z_7352343_A	36A	54A
Z_7352344_A	37A	54A
Z_7352345_G	38G	53G
Z_7352346_T	39T	54T
Z_7352347_G	38G	54G
Z_7352348_T	39T	51T
Z_7352349_T	37T	50T
Z_7352350_C	39C	51C

Z_7352351_T	39T	51T
Z_7352352_G	40G	51G
Z_7352353_G	40G	50G
Z_7352354_G	41G	50G
Z_7352355_A	42A	50A
Z_7352356_C	42C	50C
Z_7352357_T	43T	50T
Z_7352358_A	43A	49A
Z_7352359_C	40T	48C
Z_7352360_A	40A	45A
Z_7352361_G	41G	45G
Z_7352362_C	43C	23T_23C
Z_7352363_T	42T	44T
Z_7352364_C	42C	44C
Z_7352365_G	41A	43G
Z_7352366_C	40C	45C
Z_7352367_A	38A	47A
Z_7352368_T	38T	46T
Z_7352369_G	38G	46G
Z_7352370_C	38C	45C
Z_7352371_T	39T	44T
Z_7352372_C	37C	44C
Z_7352373_C	38C	43C
Z_7352374_T	38T	45T
Z_7352375_T	39T	45T
Z_7352376_G	36G	44G
Z_7352377_A	36A	43A
Z_7352378_C	36C	43C
Z_7352379_C	35C	42C
Z_7352380_T	36T	43T
Z_7352381_C	36C	41C
Z_7352382_T	36T	41T
Z_7352383_G	34G	41G
Z_7352384_G	34G	41G
Z_7352385_T	29C	23T_17C
Z_7352386_G	28A	17A_23G
Z_7352387_G	28A	40G
Z_7352388_G	35G	40G
Z_7352389_G	38G	40G
Z_7352390_T	38T	39T
Z_7352391_T	38T	39T
Z_7352392_G	38G	38G
Z_7352393_A	39G	21A_16G

Z_7352394_G	40G	37G
Z_7352395_T	41T	37T
Z_7352396_G	43G	38G
Z_7352397_G	44G	38G
Z_7352398_G	45G	37G
Z_7352399_A	47A	39A
Z_7352400_A	47A	39A
Z_7352401_T	49T	39T
Z_7352402_G	47G	41G
Z_7352403_A	47A	41A
Z_7352404_T	49T	41T
Z_7352405_G	48G	44G
Z_7352406_G	48G	42G
Z_7352407_G	49G	43G
Z_7352408_A	47A	42A
Z_7352409_G	47G	42G
Z_7352410_T	47T	41T
Z_7352411_T	47T	43T
Z_7352412_T	48C	28T_16C
Z_7352413_T	48T	44T
Z_7352414_A	49A	43A
Z_7352415_G	49G	43G
Z_7352416_T	48T	45T
Z_7352417_C	49C	45C
Z_7352418_C	47C	45C
Z_7352419_C	48C	46C
Z_7352420_A	46A	45A
Z_7352421_A	46A	43A
Z_7352422_A	44A	44A
Z_7352423_C	46C	45C
Z_7352424_A	45A	46A
Z_7352425_A	47A	47A
Z_7352426_C	46C	47C
Z_7352427_T	46T	47T
Z_7352428_A	48A	46A
Z_7352429_G	47G	46G
Z_7352430_A	47A	46A
Z_7352431_A	46A	44A
Z_7352432_G	43G	48G
Z_7352433_G	43G	46G
Z_7352434_G	43G	49G
Z_7352435_T	43T	48T
Z_7352436_C	46C	49C

Z_7352437_C	47C	49C
Z_7352438_T	49T	49T
Z_7352439_C	49G	22G_28C
Z_7352440_G	49G	52G
Z_7352441_G	49G	53G
Z_7352442_T	49T	54T
Z_7352443_C	49C	53C
Z_7352444_A	48C	54A
Z_7352445_C	48C	59C
Z_7352446_T	47T	59T
Z_7352447_T	47T	59T
Z_7352448_G	46G	58G
Z_7352449_C	45C	57C
Z_7352450_A	43A	58A
Z_7352451_G	41G	59G
Z_7352452_G	40G	59G
Z_7352453_T	42T	59T
Z_7352454_C	41C	60C
Z_7352455_T	41T	59T
Z_7352456_C	41C	58C
Z_7352457_T	41T	57T
Z_7352458_G	41G	57G
Z_7352459_G	38G	57G
Z_7352460_C	38T	58C
Z_7352461_G	39G	56G
Z_7352462_G	39G	55G
Z_7352463_G	38G	57G
Z_7352464_G	32A_38G	22A_53G_1C
Z_7352465_A	41A	57A
Z_7352466_A	39A	57A
Z_7352467_A	38A	59A
Z_7352468_A	38A	59A
Z_7352469_T	36T	57T
Z_7352470_G	36G	58G
Z_7352471_G	37G	60G
Z_7352472_A	37A	60A
Z_7352473_T	35T	60T
Z_7352474_C	35C	60C
Z_7352475_C	34C	60C
Z_7352476_T	36T	61T
Z_7352477_A	39G	33A_28G
Z_7352478_T	41T	61T
Z_7352479_C	40C	60C

Z_7352480_C	40C	60C
Z_7352481_T	39T	60T
Z_7352482_C	38C	60C
Z_7352483_A	37A	60A
Z_7352484_C	34C	60C
Z_7352485_C	35C	59C
Z_7352486_A	33A	60A
Z_7352487_G	33G	60G
Z_7352488_A	33A	60A
Z_7352489_T	34T	60T
Z_7352490_C	35C	60C
Z_7352491_C	34C	60C
Z_7352492_T	34T	60T
Z_7352493_T	35T	62T
Z_7352494_C	32C	60C
Z_7352495_T	34T	60T
Z_7352496_A	33A	58A
Z_7352497_G	34G	58G
Z_7352498_A	34A	57A
Z_7352499_A	35A	55A
Z_7352500_G	37G	57G
Z_7352501_G	37G	55G
Z_7352502_A	37A	55A
Z_7352503_G	37G	54G
Z_7352504_A	37A	55A
Z_7352505_T	37T	55T
Z_7352506_C	35C	53C
Z_7352507_A	34A	52A
Z_7352508_C	34C	52C
Z_7352509_G	34G	53G
Z_7352510_G	32G	52G
Z_7352511_G	32G	52G
Z_7352512_G	33G	53G
Z_7352513_A	31A	56A
Z_7352514_T	32T	60T
Z_7352515_T	32T	61T
Z_7352516_G	32G	61G
Z_7352517_A	32A	64A
Z_7352518_A	32A	64A
Z_7352519_C	33C	63C
Z_7352520_T	35T	62T
Z_7352521_G	32G	62G
Z_7352522_G	34G	62G

Z_7352523_C	34C	63C
Z_7352524_A	34A	63A
Z_7352525_A	35A	63A
Z_7352526_C	37C	60C
Z_7352527_C	36C	59C
Z_7352528_T	35T	59T
Z_7352529_T	35T	58T
Z_7352530_C	34C	61C
Z_7352531_C	34C	61C
Z_7352532_A	34A	60A
Z_7352533_C	36C	61C
Z_7352534_C	35C	60C
Z_7352535_C	34C	58C
Z_7352536_T	34T	55T
Z_7352537_C	36C	56C
Z_7352538_T	35T	56T
Z_7352539_C	38C	54C
Z_7352540_T	39T	50T
Z_7352541_A	40A	50A
Z_7352542_G	40G	49G
Z_7352543_C	40C	49C
Z_7352544_T	41T	50T
Z_7352545_T	40T	50T
Z_7352546_T	40T	49T
Z_7352547_C	38C	49C
Z_7352548_A	37A	50A
Z_7352549_T	38T	49T
Z_7352550_A	37A	50A
Z_7352551_T	37T	50T
Z_7352552_T	37T	50T
Z_7352553_G	37G	50G
Z_7352554_A	37A	51A
Z_7352555_T	37T	49T
Z_7352556_C	39C	50C
Z_7352557_C	37C	50C
Z_7352558_C	38C	48C
Z_7352559_T	37T	50T
Z_7352560_C	37C	50C
Z_7352561_T	37T	50T
Z_7352562_G	37G	51G
Z_7352563_A	36A	52A
Z_7352564_G	36G	52G
Z_7352565_G	34G	51G

Z_7352566_A	34A	49A
Z_7352567_G	32G	50G
Z_7352568_G	32G	51G
Z_7352569_A	32A	50A
Z_7352570_A	31A	50A
Z_7352571_G	31G	52G
Z_7352572_C	30C	52C
Z_7352573_T	31T	52T
Z_7352574_G	30G	52G
Z_7352575_T	29T	51T
Z_7352576_T	30T	52T
Z_7352577_C	30C	53C
Z_7352578_T	30T	53T
Z_7352579_G	32A	24A_29G
Z_7352580_G	32G	53G
Z_7352581_G	32G	54G
Z_7352582_T	33T	56T
Z_7352583_G	35G	57G
Z_7352584_A	35A	57A
Z_7352585_A	35A	57A
Z_7352586_A	35A	56A
Z_7352587_A	34A	56A
Z_7352588_C	34C	55C
Z_7352589_T	34T	57T
Z_7352590_C	32C	56C
Z_7352591_C	33C	59C
Z_7352592_A	32A	60A
Z_7352593_G	33G	62G
Z_7352594_T	34T	62T
Z_7352595_T	34T	61T
Z_7352596_G	32G	61G
Z_7352597_A	32A	61A
Z_7352598_T	32T	62T
Z_7352599_A	31A	59A
Z_7352600_C	30C	61C
Z_7352601_A	33A	61A
Z_7352602_A	35A	62A
Z_7352603_T	35T	62T
Z_7352604_G	36G	61G
Z_7352605_G	37G	61G
Z_7352606_G	40G	61G
Z_7352607_C	38C	60C
Z_7352608_T	38T	58T

Z_7352609_T	35T	52T
Z_7352610_C	33C	52C
Z_7352611_A	32A	52A
Z_7352612_A	32A	48A
Z_7352613_T	32T	48T
Z_7352614_G	31G	49G
Z_7352615_A	31A	49A
Z_7352616_T	30T	49T
Z_7352617_T	29T	49T
Z_7352618_T	30T	48T
Z_7352619_T	29T	48T
Z_7352620_T	29T	47T
Z_7352621_C	28C	45C
Z_7352622_C	29C	46C
Z_7352623_T	30T	45T
Z_7352624_C	30C	45C
Z_7352625_T	30T	42T
Z_7352626_T	28T	42T
Z_7352627_T	28C	42T
Z_7352628_G	28A	41G
Z_7352629_G	29G	40G
Z_7352630_C	29C	41C
Z_7352631_A	29A	42A
Z_7352632_T	29T	43T
Z_7352633_G	29G	43G
Z_7352634_A	30A	43A
Z_7352635_T	30T	43T
Z_7352636_T	30T	44T
Z_7352637_A	31A	45A
Z_7352638_A	31A	45A
Z_7352639_A	29A	44A
Z_7352640_A	28A	44A
Z_7352641_A	29A	44A
Z_7352642_G	29G	44G
Z_7352643_A	28A	43A
Z_7352644_G	28G	43G
Z_7352645_A	27A	42A
Z_7352646_G	27G	42G
Z_7352647_G	28G	42G
Z_7352648_A	29A	42A
Z_7352649_A	27A	41A
Z_7352650_A	28A	40A
Z_7352651_C	26C	40C

Z_7352652_A	27A	40A
Z_7352653_A	25A	40A
Z_7352654_A	25A	38A
Z_7352655_G	26G	38G
Z_7352656_C	26C	38C
Z_7352657_A	26A	35A
Z_7352658_T	26T	34T
Z_7352659_C	26C	34C
Z_7352660_C	26C	34C
Z_7352661_A	25A	35A
Z_7352662_C	25G	35C
Z_7352663_C	24C	34C
Z_7352664_T	23T	34T
Z_7352665_A	22A	33A
Z_7352666_C	22C	30C
Z_7352667_A	22A	30A
Z_7352668_A	22A	30A
Z_7352669_A	22A	30A
Z_7352670_C	22C	31C
Z_7352671_C	20C	30C
Z_7352672_C	18C	30C
Z_7352673_A	17A	31A
Z_7352674_C	16C	31C
Z_7352675_A	16A	31A
Z_7352676_C	13C	30C
Z_7352677_A	13A	30A
Z_7352678_G	13G	29G
Z_7352679_G	12G	29G
Z_7352680_A	12A	29A
Z_7352681_A	12A	29A
Z_7352682_C	12T	29C
Z_7352683_A	11A	28A
Z_7352684_T	13T	26T
Z_7352685_G	13G	27G
Z_7352686_C	13C	24C
Z_7352687_G	13G	24G
Z_7352688_G	12G	22G
Z_7352689_C	7A_12C	8G_22C
Z_7352690_G	4A_7G	21G
Z_7352691_G	11G	21G
Z_7352692_G	11G	21G
Z_7352693_G	10G	19G
Z_7352694_G	9G	20G

Z_7352695_G	10G	20G
Z_7352696_G	9G	20G
Z_7352697_G	11G	19G
Z_7352698_G	11G	19G
Z_7352699_G	11G	19G
Z_7352700_G	10A	20G
Z_7352701_C	10C	19C
Z_7352702_T	11T	19T
Z_7352703_G	11G	24G
Z_7352704_G	11G	25G
Z_7352705_T	11T	25T
Z_7352706_T	12T	26T
Z_7352707_T	12T	26T
Z_7352708_T	12T	28T
Z_7352709_G	13G	27G
Z_7352710_G	13G	28G
Z_7352711_C	13C	28C
Z_7352712_T	13T	28T
Z_7352713_T	13T	28T
Z_7352714_C	13C	28C
Z_7352715_T	15T	28T
Z_7352716_T	14C	30T
Z_7352717_C	14C	29C
Z_7352718_T	14T	30T
Z_7352719_G	13G	31G
Z_7352720_A	13A	31A
Z_7352721_C	12C	34C
Z_7352722_T	12T	34T
Z_7352723_C	12C	35C
Z_7352724_C	10C	35C
Z_7352725_A	13A	38A
Z_7352726_A	14A	37A
Z_7352727_C	14C	37C
Z_7352728_A	15A	37A
Z_7352729_T	17T	38T
Z_7352730_G	18G	37G
Z_7352731_T	17C	20T_1G_16C
Z_7352732_C	18C	36C
Z_7352733_T	17T	39T
Z_7352734_C	18C	39C
Z_7352735_C	18C	39C
Z_7352736_T	18T	38T
Z_7352737_T	18T	40T

Z_7352738_C	18C	41C
Z_7352739_C	18C	41C
Z_7352740_T	20T	42T
Z_7352741_T	20T	43T
Z_7352742_T	20T	43T
Z_7352743_C	19C	43C
Z_7352744_C	20C	44C
Z_7352745_T	20T	43T
Z_7352746_A	20A	46A
Z_7352747_T	21T	47T
Z_7352748_G	21G	46G
Z_7352749_G	21G	47G
Z_7352750_C	21C	49C
Z_7352751_C	21C	50C
Z_7352752_T	20T	49T
Z_7352753_C	20C	50C
Z_7352754_T	19T	52T
Z_7352755_G	20G	52G
Z_7352756_G	22G	51G
Z_7352757_G	23G	50G
Z_7352758_C	24C	51C
Z_7352759_A	26A	52A
Z_7352760_G	26G	52G
Z_7352761_A	26A	52A
Z_7352762_G	26G	54G
Z_7352763_T	26T	54T
Z_7352764_G	25G	53G
Z_7352765_C	25C	52C
Z_7352766_T	26T	53T
Z_7352767_T	25T	53T
Z_7352768_C	25C	54C
Z_7352769_T	27T	54T
Z_7352770_G	27G	55G
Z_7352771_A	28A	53A
Z_7352772_A	28A	53A
Z_7352773_C	30C	54C
Z_7352774_A	30A	56A
Z_7352775_T	30T	56T
Z_7352776_G	30G	54G
Z_7352777_C	31C	54C
Z_7352778_A	31A	54A
Z_7352779_C	29C	55C
Z_7352780_A	30A	54A

Z_7352781_G	32G	53G
Z_7352782_A	32A	53A
Z_7352783_A	31A	54A
Z_7352784_T	31T	54T
Z_7352785_A	31A	54A
Z_7352786_C	28C	53C
Z_7352787_C	28C	56C
Z_7352788_C	30C	59C
Z_7352789_T	30T	60T
Z_7352790_T	30T	58T
Z_7352791_C	30C	60C
Z_7352792_C	31C	61C
Z_7352793_T	31T	59T
Z_7352794_A	30A	60A
Z_7352795_G	29G	60G
Z_7352796_A	28A	58A
Z_7352797_A	27A	60A
Z_7352798_T	28T	61T
Z_7352799_G	26G	61G
Z_7352800_A	26A	61A
Z_7352801_G	26G	61G
Z_7352802_C	27C	61C
Z_7352803_T	28T	59T
Z_7352804_T	29T	60T
Z_7352805_T	29T	59T
Z_7352806_C	29C	59C
Z_7352807_T	29T	59T
Z_7352808_G	29G	60G
Z_7352809_G	27G	59G
Z_7352810_C	26C	60C
Z_7352811_T	27T	57T
Z_7352812_T	27T	60T
Z_7352813_C	27C	59C
Z_7352814_T	26T	58T
Z_7352815_A	26G	57A
Z_7352816_G	26G	55G
Z_7352817_T	26T	52T
Z_7352818_C	25C	54C
Z_7352819_C	26C	54C
Z_7352820_A	27A	51A
Z_7352821_T	27T	51T
Z_7352822_A	27A	51A
Z_7352823_G	27G	50G

Z_7352824_G	26G	52G
Z_7352825_A	26G	31A_21G
Z_7352826_T	24T	52T
Z_7352827_G	23G	52G
Z_7352828_C	23C	49C
Z_7352829_A	21A	48A
Z_7352830_T	22T	49T
Z_7352831_C	22C	49C
Z_7352832_T	22T	48T
Z_7352833_A	22A	47A
Z_7352834_C	23C	47C
Z_7352835_T	22T	45T
Z_7352836_T	23T	46T
Z_7352837_A	23A	46A
Z_7352838_G	22G	46G
Z_7352839_G	21G	44G
Z_7352840_C	23C	44C
Z_7352841_C	21C	43C
Z_7352842_A	21A	43A
Z_7352843_A	19A	42A
Z_7352844_T	19T	40T
Z_7352845_T	22T	44T
Z_7352846_G	21G	42G
Z_7352847_T	20T	42T
Z_7352848_G	20G	43G
Z_7352849_T	20T	41T
Z_7352850_G	20G	43G
Z_7352851_T	18T	41T
Z_7352852_G	29G	43G
Z_7352853_T	29T	42T
Z_7352854_G	27G	43G
Z_7352855_T	27T	43T
Z_7352856_G	27G	43G
Z_7352857_T	26T	39T
Z_7352858_G	26G	42G
Z_7352859_T	26T	44T
Z_7352860_G	26G	45G
Z_7352861_T	26T	44T
Z_7352862_G	25G	46G
Z_7352863_T	27T	45T
Z_7352864_G	27G	45G
Z_7352865_T	28T	47T
Z_7352866_G	29G	48G

Z_7352867_T	29T	47T
Z_7352868_G	28G	46G
Z_7352869_T	27T	45T
Z_7352870_G	28G	45G
Z_7352871_T	28T	45T
Z_7352872_G	28G	44G
Z_7352873_T	26T	44T
Z_7352874_G	26G	42G
Z_7352875_T	26T	42T
Z_7352876_G	26G	44G
Z_7352877_T	26T	44T
Z_7352878_G	26G	44G
Z_7352879_T	27T	44T
Z_7352880_G	27G	44G
Z_7352881_T	26T	45T
Z_7352882_G	27G	43G
Z_7352883_T	27T	41T
Z_7352884_G	27G	41G
Z_7352885_T	29T	41T
Z_7352886_G	28G	41G
Z_7352887_T	28T	41T
Z_7352888_G	28G	42G
Z_7352889_T	28T	41T
Z_7352890_G	27G	41G
Z_7352891_T	27T	41T
Z_7352892_G	27G	40G
Z_7352893_T	27T	39T
Z_7352894_G	28G	38G
Z_7352895_T	28T	39T
Z_7352896_G	28G	40G
Z_7352897_T	30T	40T
Z_7352898_G	29G	40G
Z_7352899_T	30T	40T
Z_7352900_G	30G	40G
Z_7352901_T	31T	41T
Z_7352902_G	31G	41G
Z_7352903_T	31T	41T
Z_7352904_G	31G	41G
Z_7352905_T	31T	40T
Z_7352906_G	30G	41G
Z_7352907_T	30T	40T
Z_7352908_G	30G	40G
Z_7352909_T	31T	40T

Z_7352910_G	30G	41G
Z_7352911_T	30T	41T
Z_7352912_G	31G	41G
Z_7352913_T	32T	41T
Z_7352914_G	32G	41G
Z_7352915_T	29T	40T
Z_7352916_G	29G	39G
Z_7352917_T	29T	40T
Z_7352918_G	30G	42G
Z_7352919_T	30T	42T
Z_7352920_G	30G	43G
Z_7352921_T	31T	43T
Z_7352922_G	20G	43G
Z_7352923_T	20T	43T
Z_7352924_G	20G	43G
Z_7352925_A	18A_1T_13C	39A_13C
Z_7352926_C	3G_5C	26C
Z_7352927_A	22A	42A
Z_7352928_C	22C	43C
Z_7352929_A	22A	43A
Z_7352930_C	18C	44C
Z_7352931_A	18A	40A
Z_7352932_A	18A	39A
Z_7352933_A	17A	39A
Z_7352934_T	18T	40T
Z_7352935_C	17C	40C
Z_7352936_T	18T	41T
Z_7352937_G	17G	41G
Z_7352938_C	18C	41C
Z_7352939_A	18A	41A
Z_7352940_G	18G	41G
Z_7352941_G	18G	44G
Z_7352942_C	17C	43C
Z_7352943_T	17T	43T
Z_7352944_T	17T	43T
Z_7352945_C	17C	42C
Z_7352946_T	18T	44T
Z_7352947_G	18G	43G
Z_7352948_T	18T	43T
Z_7352949_T	18T	41T
Z_7352950_G	18G	41G
Z_7352951_A	16A	43A
Z_7352952_T	19T	45T

Z_7352953_C	19C	44C
Z_7352954_A	21A	44A
Z_7352955_C	21C	43C
Z_7352956_A	21A	47A
Z_7352957_G	19G	47G
Z_7352958_A	19A	47A
Z_7352959_G	19C	23G_24C
Z_7352960_C	21C	46C
Z_7352961_T	22T	46T
Z_7352962_T	22T	45T
Z_7352963_T	23T	45T
Z_7352964_T	22T	45T
Z_7352965_C	24C	46C
Z_7352966_T	24T	47T
Z_7352967_T	24T	48T
Z_7352968_T	24T	48T
Z_7352969_A	22A	48A
Z_7352970_A	23A	48A
Z_7352971_A	24A	46A
Z_7352972_A	24A	46A
Z_7352973_C	24C	46C
Z_7352974_A	24A	47A
Z_7352975_T	25T	46T
Z_7352976_T	25T	46T
Z_7352977_C	26A	47C
Z_7352978_G	26G	46G
Z_7352979_T	28T	45T
Z_7352980_C	28C	45C
Z_7352981_T	26T	44T
Z_7352982_C	26C	45C
Z_7352983_C	27C	46C
Z_7352984_T	27T	47T
Z_7352985_T	25T	46T
Z_7352986_C	26C	48C
Z_7352987_C	26C	49C
Z_7352988_A	27A	52A
Z_7352989_C	28C	52C
Z_7352990_A	27A	53A
Z_7352991_G	26G	53G
Z_7352992_C	28C	51C
Z_7352993_A	28A	51A
Z_7352994_A	28A	53A
Z_7352995_T	28T	54T

Z_7352996_A	27A	55A
Z_7352997_A	26A	55A
Z_7352998_A	27A	54A
Z_7352999_C	27C	55C
Z_7353000_T	27T	58T
Z_7353001_T	26T	59T
Z_7353002_T	27T	59T
Z_7353003_G	26G	60G
Z_7353004_A	27A	58A
Z_7353005_T	28T	59T
Z_7353006_A	30A	59A
Z_7353007_T	30T	59T
Z_7353008_A	31A	61A
Z_7353009_T	31T	62T
Z_7353010_T	31T	64T
Z_7353011_T	31T	64T
Z_7353012_A	30A	63A
Z_7353013_T	29T	62T
Z_7353014_A	29A	64A
Z_7353015_A	29A	64A
Z_7353016_T	24T	64T
Z_7353017_T	26A	63T
Z_7353018_T	35T	63T
Z_7353019_T	35T	63T
Z_7353020_T	35T	64T
Z_7353021_T	35T	63T
Z_7353022_T	33T	61T
Z_7353023_T	31T	60T
Z_7353024_C	33C	61C
Z_7353025_C	33C	60C
Z_7353026_C	35C	60C
Z_7353027_C	34C	60C
Z_7353028_A	35A	62A
Z_7353029_T	35T	62T
Z_7353030_T	35T	63T
Z_7353031_T	34T	62T
Z_7353032_T	33T	63T
Z_7353033_G	32G	64G
Z_7353034_C	31C	64C
Z_7353035_T	32T	64T
Z_7353036_T	32T	63T
Z_7353037_T	32T	64T
Z_7353038_G	33G	64G

Z_7353039_G	33G	64G
Z_7353040_A	33A	65A
Z_7353041_T	33T	65T
Z_7353042_C	30C	64C
Z_7353043_T	33T	65T
Z_7353044_G	33G	66G
Z_7353045_T	33T	66T
Z_7353046_G	32G	64G
Z_7353047_A	34A	66A
Z_7353048_T	32T	67T
Z_7353049_C	33C	32T_34C
Z_7353050_G	30G	68G
Z_7353051_A	30A	66A
Z_7353052_A	31A	66A
Z_7353053_G	31G	66G
Z_7353054_T	30T	66T
Z_7353055_T	29T	67T
Z_7353056_A	29A	66A
Z_7353057_G	28G	66G
Z_7353058_A	29A	66A
Z_7353059_T	29T	66T
Z_7353060_T	29T	67T
Z_7353061_C	29C	68C
Z_7353062_T	29T	67T
Z_7353063_G	27G	68G
Z_7353064_T	28T	68T
Z_7353065_T	28T	68T
Z_7353066_C	28C	68C
Z_7353067_T	27T	69T
Z_7353068_T	27T	70T
Z_7353069_T	26C	70T
Z_7353070_T	27T	70T
Z_7353071_G	27G	70G
Z_7353072_A	27A	69A
Z_7353073_A	27A	72A
Z_7353074_A	27A	72A
Z_7353075_C	25C	24A_71C
Z_7353076_T	21C	25A_41T
Z_7353077_A	19A	66A
Z_7353078_A	19A	66A
Z_7353079_G	16A	24A_39G
Z_7353080_A	18A	65A
Z_7353081_A	16C	66A

Z_7353082_C	22C	67C
Z_7353083_C	23C	69C
Z_7353084_C	25C	67C
Z_7353085_G	24G	67G
Z_7353086_A	23A	68A
Z_7353087_C	22C	67C
Z_7353088_A	23A	67A
Z_7353089_A	24A	67A
Z_7353090_T	26T	67T
Z_7353091_T	26T	68T
Z_7353092_C	26C	68C
Z_7353093_C	26C	68C
Z_7353094_T	25T	67T
Z_7353095_G	26G	67G
Z_7353096_T	24T	65T
Z_7353097_T	24T	66T
Z_7353098_G	24G	67G
Z_7353099_T	24T	66T
Z_7353100_C	24C	67C
Z_7353101_C	24C	66C
Z_7353102_A	24A	65A
Z_7353103_C	25C	65C
Z_7353104_T	27T	62T
Z_7353105_C	29C	64C
Z_7353106_A	29A	63A
Z_7353107_A	30A	63A
Z_7353108_A	29A	64A
Z_7353109_A	28A	64A
Z_7353110_A	28A	62A
Z_7353111_G	27G	62G
Z_7353112_G	29G	61G
Z_7353113_G	28G	62G
Z_7353114_G	29G	62G
Z_7353115_T	29T	64T
Z_7353116_G	30G	65G
Z_7353117_G	29G	66G
Z_7353118_A	28G	65A
Z_7353119_A	30A	65A
Z_7353120_A	31A	66A
Z_7353121_A	34A	69A
Z_7353122_A	34A	70A
Z_7353123_C	37C	67C
Z_7353124_A	37A	66A

Z_7353125_A	37A	69A
Z_7353126_C	39C	72C
Z_7353127_C	39C	71C
Z_7353128_C	39C	72C
Z_7353129_G	39G	75G
Z_7353130_G	38G	76G
Z_7353131_A	40A	76A
Z_7353132_A	41A	80A
Z_7353133_A	42A	80A
Z_7353134_T	43T	82T
Z_7353135_A	43A	80A
Z_7353136_T	42T	80T
Z_7353137_C	43C	79C
Z_7353138_C	41C	79C
Z_7353139_A	42A	78A
Z_7353140_G	41G	81G
Z_7353141_A	42A	79A
Z_7353142_C	42C	77C
Z_7353143_C	41C	78C
Z_7353144_T	40T	78T
Z_7353145_C	41G	76C
Z_7353146_G	41G	76G
Z_7353147_A	41A	76A
Z_7353148_C	41C	77C
Z_7353149_A	42A	76A
Z_7353150_G	44G	75G
Z_7353151_T	44T	77T
Z_7353152_G	45G	77G
Z_7353153_C	44C	77C
Z_7353154_C	45C	77C
Z_7353155_G	45G	77G
Z_7353156_G	47G	76G
Z_7353157_A	47A	78A
Z_7353158_A	45A	76A
Z_7353159_A	46A	78A
Z_7353160_A	45A	78A
Z_7353161_A	45A	76A
Z_7353162_G	46G	76G
Z_7353163_C	46C	76C
Z_7353164_A	44A	77A
Z_7353165_T	46T	76T
Z_7353166_C	46C	77C
Z_7353167_C	47C	79C

Z_7353168_G	49G	76G
Z_7353169_G	48G	75G
Z_7353170_A	50A	75A
Z_7353171_T	50T	74T
Z_7353172_T	51T	75T
Z_7353173_T	49T	75T
Z_7353174_C	47C	76C
Z_7353175_A	46A	76A
Z_7353176_G	46G	75G
Z_7353177_C	46C	74C
Z_7353178_C	47C	71C
Z_7353179_A	47A	71A
Z_7353180_T	47T	71T
Z_7353181_G	47G	70G
Z_7353182_C	45C	72C
Z_7353183_A	46A	73A
Z_7353184_G	45G	73G
Z_7353185_C	43C	74C
Z_7353186_C	44C	73C
Z_7353187_T	42T	72T
Z_7353188_C	45C	71C
Z_7353189_A	44A	72A
Z_7353190_G	42G	71G
Z_7353191_T	40T	72T
Z_7353192_G	39G	71G
Z_7353193_T	37T	71T
Z_7353194_A	37A	71A
Z_7353195_A	38A	69A
Z_7353196_C	35C	70C
Z_7353197_C	34C	70C
Z_7353198_C	34C	69C
Z_7353199_C	35C	70C
Z_7353200_G	32G	67G
Z_7353201_T	32T	68T
Z_7353202_T	33T	68T
Z_7353203_C	30C	67C
Z_7353204_A	33A	67A
Z_7353205_T	31T	69T
Z_7353206_C	32C	70C
Z_7353207_T	33T	71T
Z_7353208_T	33T	71T
Z_7353209_T	33T	71T
Z_7353210_T	33T	70T

Z_7353211_T	32T	71T
Z_7353212_T	32T	71T
Z_7353213_T	33T	73T
Z_7353214_T	33T_28C	73T
Z_7353215_C	4T	68C
Z_7353216_T	33T	69T
Z_7353217_T	33T	67T
Z_7353218_C	35C	68C
Z_7353219_C	35C	68C
Z_7353220_A	35A	68A
Z_7353221_C	34C	64C
Z_7353222_C	34C	64C
Z_7353223_A	35A	66A
Z_7353224_A	35A	64A
Z_7353225_A	37A	62A
Z_7353226_C	36C	60C
Z_7353227_A	36A	59A
Z_7353228_A	34A	57A
Z_7353229_T	34T	58T
Z_7353230_T	34T	58T
Z_7353231_C	33C	60C
Z_7353232_G	36G	61G
Z_7353233_C	36C	61C
Z_7353234_T	35T	62T
Z_7353235_T	35T	61T
Z_7353236_T	36T	60T
Z_7353237_C	34C	63C
Z_7353238_A	33A	64A
Z_7353239_C	33C	64C
Z_7353240_T	31T	64T
Z_7353241_T	31T	64T
Z_7353242_T	29T	62T
Z_7353243_C	29C	61C
Z_7353244_T	28T	63T
Z_7353245_C	28C	64C
Z_7353246_T	28T	65T
Z_7353247_T	28T	65T
Z_7353248_A	28A	66A
Z_7353249_T	28T	66T
Z_7353250_T	27T	65T
Z_7353251_C	27C	65C
Z_7353252_A	27A	64A
Z_7353253_A	28A	64A

Z_7353254_G	29G	61G
Z_7353255_A	28A	61A
Z_7353256_C	30C	63C
Z_7353257_C	30C	65C
Z_7353258_C	30C	65C
Z_7353259_C	31C	65C
Z_7353260_C	30C	67C
Z_7353261_G	31G	69G
Z_7353262_A	32A	68A
Z_7353263_A	33A	66A
Z_7353264_C	35C	67C
Z_7353265_G	36G	69G
Z_7353266_T	36T	73T
Z_7353267_T	35T	73T
Z_7353268_C	34C	75C
Z_7353269_A	36A	75A
Z_7353270_A	36A	76A
Z_7353271_G	38G	77G
Z_7353272_G	39A	77G
Z_7353273_A	40A	76A
Z_7353274_T	39T	76T
Z_7353275_T	41T	77T
Z_7353276_C	39C	77C
Z_7353277_C	39C	75C
Z_7353278_T	41T	75T
Z_7353279_G	41G	74G
Z_7353280_C	41C	73C
Z_7353281_C	42C	73C
Z_7353282_T	43T	76T
Z_7353283_T	43T	76T
Z_7353284_T	43T	76T
Z_7353285_C	42C	74C
Z_7353286_T	42T	74T
Z_7353287_T	42T	75T
Z_7353288_C	40C	75C
Z_7353289_T	40T	74T
Z_7353290_C	40C	73C
Z_7353291_C	41C	75C
Z_7353292_T	41T	77T
Z_7353293_C	40C	76C
Z_7353294_C	39C	76C
Z_7353295_G	36G	76G
Z_7353296_C	36C	74C

Z_7353297_C	36C	76C
Z_7353298_T	36T	75T
Z_7353299_G	36G	75G
Z_7353300_G	37G	73G
Z_7353301_A	38A	71A
Z_7353302_T	35T	69T
Z_7353303_T	36T	72T
Z_7353304_C	36C	71C
Z_7353305_A	37A	71A
Z_7353306_G	39G	69G
Z_7353307_C	40C	68C
Z_7353308_C	40C	68C
Z_7353309_A	40A	68A
Z_7353310_G	40G	70G
Z_7353311_C	41C	71C
Z_7353312_G	41G	72G
Z_7353313_A	41A	70A
Z_7353314_T	42C	41T_32C
Z_7353315_C	42C	73C
Z_7353316_T	42T	72T
Z_7353317_C	41C	71C
Z_7353318_T	41T	70T
Z_7353319_T	41T	69T
Z_7353320_T	41T	68T
Z_7353321_A	42A	69A
Z_7353322_T	45T	69T
Z_7353323_T	46T	72T
Z_7353324_T	45T	70T
Z_7353325_G	45G	69G
Z_7353326_C	41C	68C
Z_7353327_C	42C	67C
Z_7353328_C	42C	69C
Z_7353329_G	42G	69G
Z_7353330_A	43G	69A
Z_7353331_T	43T	69T
Z_7353332_T	44T	66T
Z_7353333_T	45T	66T
Z_7353334_G	44G	64G
Z_7353335_G	42G	65G
Z_7353336_G	44G	67G
Z_7353337_T	44T	67T
Z_7353338_T	45T	68T
Z_7353339_T	44T	65T

Z_7353340_A	45A	64A
Z_7353341_A	43A	64A
Z_7353342_G	43G	65G
Z_7353343_A	43A	66A
Z_7353344_A	44A	66A
Z_7353345_A	44A	67A
Z_7353346_A	46A	68A
Z_7353347_T	47T	67T
Z_7353348_A	46A	67A
Z_7353349_T	46T	68T
Z_7353350_A	45A	71A
Z_7353351_T	44T	70T
Z_7353352_T	45T	68T
Z_7353353_A	44A	68A
Z_7353354_G	44G	68G
Z_7353355_A	44A	65A
Z_7353356_T	45T	66T
Z_7353357_A	47A	66A
Z_7353358_T	46T	67T
Z_7353359_T	47T	69T
Z_7353360_C	47C	70C
Z_7353361_A	46T	35A_34T
Z_7353362_C	46C	68C
Z_7353363_T	45T	67T
Z_7353364_C	46C	68C
Z_7353365_T	46T	67T
Z_7353366_T	46T	67T
Z_7353367_T	46T	67T
Z_7353368_T	47T	67T
Z_7353369_T	48T	68T
Z_7353370_T	46T	68T
Z_7353371_T	46T	68T
Z_7353372_A	48A	68A
Z_7353373_A	48A	69A
Z_7353374_A	47A	70A
Z_7353375_A	46A	71A
Z_7353376_C	45C	71C
Z_7353377_G	44G	68G
Z_7353378_C	44C	68C
Z_7353379_C	45C	30T_38C
Z_7353380_A	47A	70A
Z_7353381_A	47A	69A
Z_7353382_A	47A	69A

Z_7353383_C	49C	69C
Z_7353384_A	49A	69A
Z_7353385_A	49A	70A
Z_7353386_A	49A	73A
Z_7353387_G	50G	70G
Z_7353388_A	49A	71A
Z_7353389_G	49G	70G
Z_7353390_T	49T	70T
Z_7353391_C	48C	70C
Z_7353392_A	45A	70A
Z_7353393_T	45T	72T
Z_7353394_T	46T	72T
Z_7353395_T	46T	73T
Z_7353396_A	47A	73A
Z_7353397_A	47A	74A
Z_7353398_A	47A	71A
Z_7353399_A	45A	70A
Z_7353400_C	48C	72C
Z_7353401_G	47G	71G
Z_7353402_A	46A	72A
Z_7353403_G	43G	72G
Z_7353404_G	42G	74G
Z_7353405_C	43C	73C
Z_7353406_A	44A	73A
Z_7353407_T	44T	72T
Z_7353408_T	44T	73T
Z_7353409_T	46T	70T
Z_7353410_A	44A	70A
Z_7353411_C	45C	26T_40C
Z_7353412_G	48G	67G
Z_7353413_C	47C	66C
Z_7353414_A	46A	65A
Z_7353415_T	44T	39T_26C
Z_7353416_T	42T	65T
Z_7353417_G	44G	65G
Z_7353418_A	43G	38A_24G
Z_7353419_A	44A	64A
Z_7353420_G	44G	66G
Z_7353421_T	44T	66T
Z_7353422_T	42T	66T
Z_7353423_G	44G	24T_64G
Z_7353424_T	46T	66T
Z_7353425_A	47A	66A

Z_7353426_A	46A	67A
Z_7353427_C	44C	69C
Z_7353428_T	46T	70T
Z_7353429_T	47T	43T_27C
Z_7353430_T	46T	70T
Z_7353431_T	48T	71T
Z_7353432_G	47G	72G
Z_7353433_G	47G	70G
Z_7353434_T	51T	71T
Z_7353435_G	51G	74G
Z_7353436_C	49C	72C
Z_7353437_T	50T	71T
Z_7353438_G	49G	71G
Z_7353439_A	49A	71A
Z_7353440_T	51T	68T
Z_7353441_C	49C	26T_42C
Z_7353442_T	49T	69T
Z_7353443_C	47C	68C
Z_7353444_C	48C	66C
Z_7353445_T	48T	66T
Z_7353446_G	47G	65G
Z_7353447_C	48C	65C
Z_7353448_A	48A	65A
Z_7353449_A	47A	63A
Z_7353450_A	46A	62A
Z_7353451_G	45G	61G
Z_7353452_C	44C	60C
Z_7353453_A	46A	62A
Z_7353454_T	45T	59T
Z_7353455_G	42G	54G
Z_7353456_A	44A	55A
Z_7353457_T	44T	55T
Z_7353458_A	70A_29T_32G	69A_15T_16G
Z_7353459_A	46A	56A
Z_7353460_T	46T	59T
Z_7353461_G	46G	59G
Z_7353462_A	46A	59A
Z_7353463_T	45T	59T
Z_7353464_G	47G	58G
Z_7353465_A	49A	59A
Z_7353466_T	48T	59T
Z_7353467_G	47G	59G
Z_7353468_A	47A	59A

Z_7353469_T	47T	58T
Z_7353470_G	45G	57G
Z_7353471_A	45A	57A
Z_7353472_T	45T	57T
Z_7353473_A	40A	56A
Z_7353474_A	40A	56A
Z_7353475_C	41C	56C
Z_7353476_C	42C	57C
Z_7353477_A	42A	58A
Z_7353478_T	41T	60T
Z_7353479_C	40C	61C
Z_7353480_A	42A	60A
Z_7353481_T	42T	59T
Z_7353482_G	42G	59G
Z_7353483_G	43G	59G
Z_7353484_G	39G	59G
Z_7353485_A	40A	60A
Z_7353486_G	43G	59G
Z_7353487_A	43A	59A
Z_7353488_A	41A	57A
Z_7353489_A	42A	57A
Z_7353490_G	41G	59G
Z_7353491_G	41G	59G
Z_7353492_A	41A	60A
Z_7353493_A	41A	62A
Z_7353494_G	42G	63G
Z_7353495_T	39T	61T
Z_7353496_G	40G	61G
Z_7353497_G	39T	61G
Z_7353498_A	39A	60A
Z_7353499_G	39G	60G
Z_7353500_A	37A	61A
Z_7353501_G	37G	59G
Z_7353502_A	38A	57A
Z_7353503_A	39A	60A
Z_7353504_A	40A	60A
Z_7353505_G	38G	60G
Z_7353506_C	37C	61C
Z_7353507_C	36T	60C
Z_7353508_G	37G	60G
Z_7353509_C	37C	61C
Z_7353510_A	39A	60A
Z_7353511_A	39A	60A

Z_7353512_A	38A	60A
Z_7353513_C	38C	60C
Z_7353514_G	37G	59G
Z_7353515_G	37G	58G
Z_7353516_G	38G	58G
Z_7353517_C	39C	58C
Z_7353518_T	39T	58T
Z_7353519_T	39T	60T
Z_7353520_G	39G	60G
Z_7353521_A	41A	58A
Z_7353522_A	41A	55A
Z_7353523_A	38A	54A
Z_7353524_T	37T	54T
Z_7353525_T	36T	54T
Z_7353526_C	36C	53C
Z_7353527_G	36G	51G
Z_7353528_T	35T	51T
Z_7353529_A	36A	50A
Z_7353530_C	37C	51C
Z_7353531_T	38T	51T
Z_7353532_T	40T	53T
Z_7353533_T	40T	52T
Z_7353534_C	38C	52C
Z_7353535_A	36A	52A
Z_7353536_A	37A	53A
Z_7353537_G	38G	52G
Z_7353538_T	38T	52T
Z_7353539_G	38G	52G
Z_7353540_C	38C	53C
Z_7353541_T	39T	53T
Z_7353542_C	39C	55C
Z_7353543_T	40T	55T
Z_7353544_A	40A	55A
Z_7353545_A	41A	55A
Z_7353546_A	40A	55A
Z_7353547_A	40A	54A
Z_7353548_A	40A	53A
Z_7353549_C	38C	53C
Z_7353550_C	37C	53C
Z_7353551_C	39C	53C
Z_7353552_T	39T	53T
Z_7353553_G	38G	52G
Z_7353554_A	39A	51A

Z_7353555_T	40T	48T
Z_7353556_C	39C	51C
Z_7353557_C	38C	52C
Z_7353558_C	40C	53C
Z_7353559_G	39G	53G
Z_7353560_C	41C	52C
Z_7353561_T	42T	51T
Z_7353562_T	42T	52T
Z_7353563_T	41T	51T
Z_7353564_T	40T	51T
Z_7353565_C	40C	51C
Z_7353566_C	41C	51C
Z_7353567_A	41A	51A
Z_7353568_C	42C	50C
Z_7353569_G	42G	51G
Z_7353570_A	42A	51A
Z_7353571_A	41A	51A
Z_7353572_A	39A	49A
Z_7353573_T	37T	47T
Z_7353574_A	37A	47A
Z_7353575_A	39A	46A
Z_7353576_T	38T	45T
Z_7353577_A	39A	45A
Z_7353578_A	40A	46A
Z_7353579_T	40T	46T
Z_7353580_A	38A	46A
Z_7353581_A	37A	46A
Z_7353582_T	39T	47T
Z_7353583_C	39C	48C
Z_7353584_A	39A	48A
Z_7353585_G	38G	47G
Z_7353586_G	36G	48G
Z_7353587_G	36G	47G
Z_7353588_C	39C	46C
Z_7353589_T	39T	46T
Z_7353590_G	37G	47G
Z_7353591_T	37T	46T
Z_7353592_G	37G	46G
Z_7353593_A	38A	48A
Z_7353594_T	38T	50T
Z_7353595_T	37T	49T
Z_7353596_T	39T	50T
Z_7353597_G	39G	50G

Z_7353598_A	39A	47A
Z_7353599_G	40G	47G
Z_7353600_C	41C	47C
Z_7353601_C	42C	47C
Z_7353602_A	42A	48A
Z_7353603_G	41G	48G
Z_7353604_G	41G	49G
Z_7353605_C	41C	49C
Z_7353606_G	40G	49G
Z_7353607_C	39C	49C
Z_7353608_T	41T	48T
Z_7353609_T	41T	50T
Z_7353610_T	41T	50T
Z_7353611_G	40G	51G
Z_7353612_C	40C	51C
Z_7353613_A	39A	52A
Z_7353614_C	40C	51C
Z_7353615_A	38A	51A
Z_7353616_T	39T	51T
Z_7353617_G	41G	51G
Z_7353618_T	43T	52T
Z_7353619_T	42T	53T
Z_7353620_C	41C	54C
Z_7353621_A	41A	55A
Z_7353622_G	41G	55G
Z_7353623_G	41G	55G
Z_7353624_A	40A	56A
Z_7353625_A	40A	54A
Z_7353626_T	38T	55T
Z_7353627_A	39A	53A
Z_7353628_C	39C	54C
Z_7353629_T	39T	53T
Z_7353630_G	37G	54G
Z_7353631_T	36T	53T
Z_7353632_C	35C	54C
Z_7353633_T	36T	54T
Z_7353634_T	36T	58T
Z_7353635_A	36A	58A
Z_7353636_G	34G	58G
Z_7353637_T	35T	56T
Z_7353638_G	34G	56G
Z_7353639_A	34A	56A
Z_7353640_T	34T	54T

Z_7353641_C	34C	55C
Z_7353642_G	34G	56G
Z_7353643_G	33G	57G
Z_7353644_G	32C	28G_28C
Z_7353645_C	32T	29T_28C
Z_7353646_T	34T	57T
Z_7353647_A	34A	58A
Z_7353648_C	34C	60C
Z_7353649_T	35T	60T
Z_7353650_C	35C	64C
Z_7353651_T	36T	62T
Z_7353652_A	37A	33A_29G
Z_7353653_A	37A	61A
Z_7353654_G	37G	61G
Z_7353655_G	37G	62G
Z_7353656_T	36T	62T
Z_7353657_T	36T	62T
Z_7353658_G	34G	62G
Z_7353659_A	34A	62A
Z_7353660_A	35A	62A
Z_7353661_G	34G	63G
Z_7353662_G	34G	64G
Z_7353663_C	34C	65C
Z_7353664_T	34T	65T
Z_7353665_G	34G	64G
Z_7353666_G	35G	64G
Z_7353667_C	35C	65C
Z_7353668_T	37T	64T
Z_7353669_T	37T	64T
Z_7353670_T	35T	64T
Z_7353671_A	34A	63A
Z_7353672_T	33T	64T
Z_7353673_A	34A	68A
Z_7353674_C	35A	68C
Z_7353675_C	37C	67C
Z_7353676_G	38G	67G
Z_7353677_A	37A	65A
Z_7353678_A	37A	64A
Z_7353679_A	37A	64A
Z_7353680_G	37G	65G
Z_7353681_C	36C	65C
Z_7353682_C	38C	64C
Z_7353683_T	38T	65T

Z_7353684_G	39G	64G
Z_7353685_A	38A	62A
Z_7353686_G	38G	64G
Z_7353687_T	36T	64T
Z_7353688_A	35A	62A
Z_7353689_A	36A	61A
Z_7353690_C	36C	64C
Z_7353691_G	33G	65G
Z_7353692_G	34G	65G
Z_7353693_T	34T	65T
Z_7353694_A	35A	67A
Z_7353695_A	36A	65A
Z_7353696_T	37T	66T
Z_7353697_A	39A	65A
Z_7353698_A	39A	66A
Z_7353699_A	40A	66A
Z_7353700_T	39T	69T
Z_7353701_A	41A	69A
Z_7353702_A	41A	71A
Z_7353703_T	41T	71T
Z_7353704_A	41A	70A
Z_7353705_A	43A	70A
Z_7353706_T	44T	72T
Z_7353707_T	44T	74T
Z_7353708_A	43T	46A_27T
Z_7353709_A	44A	72A
Z_7353710_A	46A	73A
Z_7353711_A	45A	73A
Z_7353712_A	45A	75A
Z_7353713_A	46A	75A
Z_7353714_A	46A	74A
Z_7353715_G	46G	74G
Z_7353716_A	46A	76A
Z_7353717_A	48A	76A
Z_7353718_T	48T	77T
Z_7353719_A	49A	77A
Z_7353720_T	49T	79T
Z_7353721_A	49A	80A
Z_7353722_C	47T	82C
Z_7353723_T	47T	84T
Z_7353724_G	47G	86G
Z_7353725_T	50T	85T
Z_7353726_A	50A	86A

Z_7353727_G	51G	86G
Z_7353728_A	52A	87A
Z_7353729_T	53T	83T
Z_7353730_C	54C	82C
Z_7353731_A	56A	82A
Z_7353732_G	56G	83G
Z_7353733_C	56C	84C
Z_7353734_T	57T	84T
Z_7353735_T	56T	84T
Z_7353736_T	57T	85T
Z_7353737_A	57A	85A
Z_7353738_G	55G	85G
Z_7353739_T	54T	87T
Z_7353740_T	54T	85T
Z_7353741_T	56T	85T
Z_7353742_C	54C	84C
Z_7353743_T	53T	83T
Z_7353744_C	49C	84C
Z_7353745_T	51T	81T
Z_7353746_T	50T	81T
Z_7353747_C	52C	80C
Z_7353748_G	52G	80G
Z_7353749_A	52A	80A
Z_7353750_A	54A	79A
Z_7353751_G	53G	81G
Z_7353752_T	52T	82T
Z_7353753_G	52G	82G
Z_7353754_G	50G	82G
Z_7353755_T	50T	82T
Z_7353756_G	51G	82G
Z_7353757_G	51G	79G
Z_7353758_T	51T	79T
Z_7353759_T	50T	79T
Z_7353760_T	50T	79T
Z_7353761_G	50G	79G
Z_7353762_C	50C	78C
Z_7353763_A	50A	46A_32T
Z_7353764_C	51C	78C
Z_7353765_T	49T	77T
Z_7353766_C	49C	80C
Z_7353767_A	45A	81A
Z_7353768_G	45G	76G
Z_7353769_A	45A	77A

Z_7353770_A	46A	78A
Z_7353771_A	44A	78A
Z_7353772_G	44G	79G
Z_7353773_A	45A	79A
Z_7353774_A	46A	80A
Z_7353775_G	46G	79G
Z_7353776_G	46G	76G
Z_7353777_T	45T	76T
Z_7353778_C	46C	76C
Z_7353779_C	47C	78C
Z_7353780_C	45C	77C
Z_7353781_A	45A	77A
Z_7353782_G	47G	77G
Z_7353783_G	45G	77G
Z_7353784_G	45G	76G
Z_7353785_A	45A	73A
Z_7353786_A	47A	72A
Z_7353787_A	46A	72A
Z_7353788_T	44T	72T
Z_7353789_A	44A	70A
Z_7353790_T	42T	73T
Z_7353791_T	43T	72T
Z_7353792_T	43T	73T
Z_7353793_T	44T	72T
Z_7353794_G	44G	69G
Z_7353795_T	42T	68T
Z_7353796_T	41T	67T
Z_7353797_T	41T	65T
Z_7353798_T	38T	66T
Z_7353799_T	38T	66T
Z_7353800_A	38A	68A
Z_7353801_C	38C	66C
Z_7353802_A	38A	65A
Z_7353803_A	38A	65A
Z_7353804_A	38A	64A
Z_7353805_G	37G	64G
Z_7353806_G	36G	63G
Z_7353807_T	36T	63T
Z_7353808_C	35C	60C
Z_7353809_T	36T	62T
Z_7353810_T	38T	61T
Z_7353811_C	34C	58C
Z_7353812_T	35T	58T

Z_7353813_T	35T	58T
Z_7353814_C	34C	58C
Z_7353815_C	34C	56C
Z_7353816_A	35A	54A
Z_7353817_T	34T	52T
Z_7353818_T	33T	51T
Z_7353819_A	34A	50A
Z_7353820_C	32C	50C
Z_7353821_T	33T	49T
Z_7353822_T	34T	49T
Z_7353823_T	34T	49T
Z_7353824_T	34T	50T
Z_7353825_C	33C	49C
Z_7353826_G	33G	49G
Z_7353827_G	33G	48G
Z_7353828_A	33A	47A
Z_7353829_G	33G	47G
Z_7353830_A	34A	47A
Z_7353831_A	34A	46A
Z_7353832_A	34A	45A
Z_7353833_G	34G	45G
Z_7353834_C	30C	42C
Z_7353835_T	31T	43T
Z_7353836_C	32C	43C
Z_7353837_T	32T	44T
Z_7353838_G	32G	43G
Z_7353839_A	30A	42A
Z_7353840_C	31T	18T_23C
Z_7353841_C	29C	40C
Z_7353842_A	31A	40A
Z_7353843_G	32G	40G
Z_7353844_C	30C	40C
Z_7353845_C	29C	40C
Z_7353846_A	29A	37A
Z_7353847_T	29T	38T
Z_7353848_C	28C	38C
Z_7353849_T	27T	38T
Z_7353850_G	27G	37G
Z_7353851_G	27G	37G
Z_7353852_G	25G	37G
Z_7353853_G	24G	37G
Z_7353854_A	23A	39A
Z_7353855_A	23A	40A

Z_7353856_A	22A	41A
Z_7353857_A	21A	43A
Z_7353858_T	21T	43T
Z_7353859_A	21A	44A
Z_7353860_A	22A	45A
Z_7353861_A	22A	43A
Z_7353862_A	23A	42A
Z_7353863_T	21T	43T
Z_7353864_C	23C	42C
Z_7353865_T	23T	43T
Z_7353866_G	23G	43G
Z_7353867_G	22G	42G
Z_7353868_T	21T	43T
Z_7353869_A	23A	45A
Z_7353870_G	23G	44G
Z_7353871_G	23G	44G
Z_7353872_A	22A	45A
Z_7353873_A	23A	45A
Z_7353874_G	22G	44G
Z_7353875_A	23A	44A
Z_7353876_C	23C	43C
Z_7353877_C	21C	43C
Z_7353878_C	22C	43C
Z_7353879_A	24A	43A
Z_7353880_G	21G	44G
Z_7353881_G	22G	44G
Z_7353882_A	22A	44A
Z_7353883_C	21C	46C
Z_7353884_G	21G	46G
Z_7353885_C	21C	45C
Z_7353886_T	20T	46T
Z_7353887_A	19A	45A
Z_7353888_A	21A	45A
Z_7353889_A	21A	46A
Z_7353890_A	22A	41A
Z_7353891_A	22A	45A
Z_7353892_C	21C	46C
Z_7353893_G	21G	47G
Z_7353894_C	21C	47C
Z_7353895_C	21C	45C
Z_7353896_G	21G	45G
Z_7353897_C	19C	44C
Z_7353898_T	20T	43T

Z_7353899_T	18T	44T
Z_7353900_G	19G	44G
Z_7353901_G	19G	46G
Z_7353902_A	21A	46A
Z_7353903_G	20G	46G
Z_7353904_A	20A	47A
Z_7353905_G	19G	48G
Z_7353906_G	21G	47G
Z_7353907_C	21C	47C
Z_7353908_A	22A	48A
Z_7353909_G	23G	47G
Z_7353910_A	21A	47A
Z_7353911_T	21T	47T
Z_7353912_C	23C	48C
Z_7353913_T	21T	47T
Z_7353914_A	20A	46A
Z_7353915_A	21A	47A
Z_7353916_C	22C	46C
Z_7353917_A	24A	48A
Z_7353918_C	23C	19G_30C
Z_7353919_G	24G	49G
Z_7353920_T	23T	49T
Z_7353921_C	24C	50C
Z_7353922_T	24T	50T
Z_7353923_G	23G	50G
Z_7353924_C	24C	50C
Z_7353925_A	25A	50A
Z_7353926_G	25G	49G
Z_7353927_G	24G	50G
Z_7353928_C	25C	50C
Z_7353929_T	27T	50T
Z_7353930_A	26A	51A
Z_7353931_T	27T	51T
Z_7353932_C	27C	51C
Z_7353933_G	28G	51G
Z_7353934_T	27T	52T
Z_7353935_C	29C	52C
Z_7353936_G	29G	52G
Z_7353937_G	29G	52G
Z_7353938_A	29A	53A
Z_7353939_A	29A	53A
Z_7353940_T	28T	53T
Z_7353941_A	28A	54A

Z_7353942_A	27A	56A
Z_7353943_C	27C	57C
Z_7353944_A	27A	57A
Z_7353945_G	27G	58G
Z_7353946_A	27C	57A
Z_7353947_G	27G	57G
Z_7353948_C	27C	57C
Z_7353949_A	26A	55A
Z_7353950_A	26A	54A
Z_7353951_T	26T	55T
Z_7353952_C	25C	53C
Z_7353953_T	26T	53T
Z_7353954_G	26G	52G
Z_7353955_A	26A	51A
Z_7353956_A	26A	51A
Z_7353957_G	26G	50G
Z_7353958_C	25C	49C
Z_7353959_G	23G	48G
Z_7353960_T	23T	49T
Z_7353961_G	23G	49G
Z_7353962_T	23T	49T
Z_7353963_T	23T	47T
Z_7353964_T	24T	46T
Z_7353965_A	24A	46A
Z_7353966_C	24C	47C
Z_7353967_T	24T	46T
Z_7353968_C	24C	46C
Z_7353969_G	26G	46G
Z_7353970_G	26G	47G
Z_7353971_A	25A	47A
Z_7353972_A	24A	47A
Z_7353973_G	24G	48G
Z_7353974_A	24A	49A
Z_7353975_A	24A	48A
Z_7353976_A	23A	47A
Z_7353977_A	23A	48A
Z_7353978_A	22A	46A
Z_7353979_G	21G	45G
Z_7353980_C	20C	43C
Z_7353981_G	21G	42G
Z_7353982_A	19A	42A
Z_7353983_A	18A	25A_17G
Z_7353984_T	18T	41T

Z_7353985_A	18A	41A
Z_7353986_A	17A	43A
Z_7353987_C	16C	42C
Z_7353988_A	16A	42A
Z_7353989_A	16A	42A
Z_7353990_G	16G	41G
Z_7353991_C	16C	41C
Z_7353992_T	16T	43T
Z_7353993_C	16C	44C
Z_7353994_A	16A	44A
Z_7353995_A	15A	44A
Z_7353996_C	15C	42C
Z_7353997_A	15A	41A
Z_7353998_A	14A	41A
Z_7353999_G	14G	40G
Z_7354000_G	14G	39G
Z_7354001_A	13A	40A
Z_7354002_C	12C	41C
Z_7354003_T	11T	40T
Z_7354004_T	11T	41T
Z_7354005_T	10T	41T
Z_7354006_G	10G	41G
Z_7354007_A	11A	40A
Z_7354008_G	11G	39G
Z_7354009_G	10G	39G
Z_7354010_T	10T	38T
Z_7354011_C	9C	39C
Z_7354012_C	9C	37C
Z_7354013_C	9G	13G_23C
Z_7354014_C	9C	35C
Z_7354015_T	9T	36T
Z_7354016_C	9C	35C
Z_7354017_T	10T	35T
Z_7354018_T	10T	35T
Z_7354019_C	10C	35C
Z_7354020_C	10C	35C
Z_7354021_T	10T	35T
Z_7354022_A	10A	34A
Z_7354023_A	10A	34A
Z_7354024_T	10T	34T
Z_7354025_T	10T	33T
Z_7354026_T	10T	33T
Z_7354027_C	10C	32C

Z_7354028_C	10C	32C
Z_7354029_A	10A	31A
Z_7354030_C	10C	31C
Z_7354031_T	9T	31T
Z_7354032_C	9C	31C
Z_7354033_T	9T	30T
Z_7354034_T	8T	30T
Z_7354035_G	8G	30G
Z_7354036_A	8A	28A
Z_7354037_G	9G	26G
Z_7354038_A	9A	25A
Z_7354039_C	7C	25C
Z_7354040_A	7A	25A
Z_7354041_C	7C	25C
Z_7354042_A	7A	25A
Z_7354043_A	7A	25A
Z_7354044_A	7A	25A
Z_7354045_G	7G	25G
Z_7354046_C	6C	23C
Z_7354047_G	6G	23G
Z_7354048_G	6G	23G
Z_7354049_G	6G	24G
Z_7354050_C	16G_6C	18G_22C
Z_7354051_G	6G	25G
Z_7354052_G	6G	26G
Z_7354053_G	6G	26G
Z_7354054_G	6G	27G
Z_7354055_G	6G	26G
Z_7354056_G	6G	27G
Z_7354057_G	6G	27G
Z_7354058_G	6G	27G
Z_7354059_C	5C	22C
Z_7354060_G	5G	25G
Z_7354061_A	5A	23A
Z_7354062_A	5A	24A
Z_7354063_C	5C	24C
Z_7354064_G	5G	24G
Z_7354065_A	5A	22A
Z_7354066_G	5G	23G
Z_7354067_A	4A_1T	23A
Z_7354068_G	5G	25G
Z_7354069_T	5T	25T
Z_7354070_T	4T	24T

Z_7354071_C	4C	25C
Z_7354072_T	4T	24T
Z_7354073_A	3A	23A
Z_7354074_G	3G	25G
Z_7354075_C	3C	25C
Z_7354076_C	1A_3C	25C
Z_7354077_A	3A	25A
Z_7354078_C	1C	25C
Z_7354079_G	null	26G
Z_7354080_G	null	24G
Z_7354081_G	null	23G
Z_7354082_A	null	23A
Z_7354083_G	null	22G
Z_7354084_G	null	23G
Z_7354085_T	1T	21T
Z_7354086_T	1T	20T
Z_7354087_T	1T	19T
Z_7354088_T	1T	20T
Z_7354089_G	1G	20G
Z_7354090_C	1C	20C
Z_7354091_A	1A	20A
Z_7354092_T	1T	20T
Z_7354093_T	1T	20T
Z_7354094_C	1C	21C
Z_7354095_A	1A	21A
Z_7354096_C	1C	20C
Z_7354097_A	1A	20A
Z_7354098_C	null	21C
Z_7354099_G	null	21G
Z_7354100_T	null	21T
Z_7354101_G	null	21G
Z_7354102_C	null	21C
Z_7354103_A	null	21A
Z_7354104_G	null	22G
Z_7354105_G	null	21G
Z_7354106_G	null	21G
Z_7354107_A	null	20A
Z_7354108_G	null	19G
Z_7354109_G	null	19G
Z_7354110_C	11C	21C
Z_7354111_G	11G	22G
Z_7354112_T	11T	22T
Z_7354113_G	11G	22G

Z_7354114_T	11T	23T
Z_7354115_A	11A	24A
Z_7354116_T	11T	24T
Z_7354117_T	11T	24T
Z_7354118_T	11T	24T
Z_7354119_G	11G	25G
Z_7354120_G	11G	25G
Z_7354121_C	11C	26C
Z_7354122_T	11T	27T
Z_7354123_T	11T	27T
Z_7354124_T	11T	27T
Z_7354125_T	11T	28T
Z_7354126_C	11C	28C
Z_7354127_T	11C	28T
Z_7354128_T	11T	28T
Z_7354129_T	11T	28T
Z_7354130_T	11T	28T
Z_7354131_T	11T	28T
Z_7354132_C	12C	26C
Z_7354133_T	12T	26T
Z_7354134_A	13A	27A
Z_7354135_C	12C	27C
Z_7354136_C	12C	28C
Z_7354137_A	12A	29A
Z_7354138_A	13A	29A
Z_7354139_G	13G	28G
Z_7354140_A	13A	28A
Z_7354141_C	13C	28C
Z_7354142_T	14T	29T
Z_7354143_G	13G	30G
Z_7354144_G	14G	29G
Z_7354145_G	14G	29G
Z_7354146_G	15G	30G
Z_7354147_G	17G	31G
Z_7354148_T	17T	31T
Z_7354149_G	17G	30G
Z_7354150_G	17G	30G
Z_7354151_G	17G	29G
Z_7354152_G	17G	29G
Z_7354153_T	17T	29T
Z_7354154_G	17G	29G
Z_7354155_G	17G	29G
Z_7354156_G	17G	31G

Z_7354157_G	15G	33G
Z_7354158_G	14G	34G
Z_7354159_A	14A	34A
Z_7354160_G	13G	36G
Z_7354161_G	14G	37G
Z_7354162_G	14G	36G
Z_7354163_A	14A	35A
Z_7354164_A	14A	33A
Z_7354165_G	14G	35G
Z_7354166_G	13G	35G
Z_7354167_A	14A	35A
Z_7354168_T	14T	35T
Z_7354169_T	13T	35T
Z_7354170_G	13G	36G
Z_7354171_A	15A	36A
Z_7354172_T	15T	36T
Z_7354173_G	15G	36G
Z_7354174_G	14G	35G
Z_7354175_A	14A	36A
Z_7354176_T	13T	36T
Z_7354177_C	13C	37C
Z_7354178_G	13G	37G
Z_7354179_G	13G	37G
Z_7354180_A	13A	38A
Z_7354181_G	13G	38G
Z_7354182_G	14G	38G
Z_7354183_T	14T	37T
Z_7354184_G	14G	37G
Z_7354185_C	14C	37C
Z_7354186_A	14A	37A
Z_7354187_A	14A	37A
Z_7354188_G	14G	37G
Z_7354189_A	15A	37A
Z_7354190_G	15G	37G
Z_7354191_T	16T	37T
Z_7354192_G	16G	36G
Z_7354193_A	18A	35A
Z_7354194_A	18A	34A
Z_7354195_G	18G	34G
Z_7354196_C	17C	34C
Z_7354197_A	18A	34A
Z_7354198_G	18G	34G
Z_7354199_C	18C	33C

Z_7354200_A	18A	34A
Z_7354201_A	19A	35A
Z_7354202_G	17G	35G
Z_7354203_C	19C	36C
Z_7354204_A	18A	37A
Z_7354205_A	18A	35A
Z_7354206_G	19G	34G
Z_7354207_G	20G	34G
Z_7354208_A	19A	34A
Z_7354209_A	19A	33A
Z_7354210_G	21G	32G
Z_7354211_C	21C	32C
Z_7354212_A	20A	33A
Z_7354213_G	19G	33G
Z_7354214_C	21C	30C
Z_7354215_C	19C	32C
Z_7354216_C	19C	32C
Z_7354217_T	17T	30T
Z_7354218_C	17C	31C
Z_7354219_C	17C	31C
Z_7354220_C	16C	30C
Z_7354221_C	17C	30C
Z_7354222_T	17T	27T
Z_7354223_T	18T	29T
Z_7354224_C	18C	29C
Z_7354225_C	18C	30C
Z_7354226_C	18C	30C
Z_7354227_G	18G	27G
Z_7354228_T	18T	26T
Z_7354229_C	20C	30C
Z_7354230_C	20C	30C
Z_7354231_C	19C	30C
Z_7354232_C	19C	31C
Z_7354233_C	18C	32C
Z_7354234_C	18C	32C
Z_7354235_A	16A	25A_1G_6C
Z_7354236_C	18C	32C
Z_7354237_C	18C	32C
Z_7354238_C	18C	32C
Z_7354239_C	17C	32C
Z_7354240_C	19C	31C
Z_7354241_G	16G	27G
Z_7354242_A	17A	29A

Z_7354243_A	17A	30A
Z_7354244_A	18A	30A
Z_7354245_G	18G	30G
Z_7354246_G	17G	31G
Z_7354247_C	18C	32C
Z_7354248_G	18G	11A_22G
Z_7354249_C	18C	35C
Z_7354250_G	19G	35G
Z_7354251_G	19G	35G
Z_7354252_A	18C	20A_1T_1G_13C
Z_7354253_C	18C	36C
Z_7354254_C	18C	36C
Z_7354255_T	18T	36T
Z_7354256_C	19C	36C
Z_7354257_C	19C	37C
Z_7354258_T	18T	38T
Z_7354259_G	18G	37G
Z_7354260_C	17C	39C
Z_7354261_G	17G	39G
Z_7354262_C	16C	40C
Z_7354263_C	15C	41C
Z_7354264_C	15C	42C
Z_7354265_C	15C	42C
Z_7354266_A	14A	41A
Z_7354267_C	15C	44C
Z_7354268_A	16A	44A
Z_7354269_C	17C	44C
Z_7354270_G	18G	44G
Z_7354271_G	18G	46G
Z_7354272_A	17A	46A
Z_7354273_G	17G	46G
Z_7354274_C	18C	46C
Z_7354275_T	18T	44T
Z_7354276_T	18T	44T
Z_7354277_G	17G	45G
Z_7354278_C	17C	46C
Z_7354279_T	17T	47T
Z_7354280_T	15T	47T
Z_7354281_A	15A	47A
Z_7354282_C	14C	47C
Z_7354283_G	14G	49G
Z_7354284_A	14A	47A
Z_7354285_T	15T	49T

Z_7354286_G	15G	50G
Z_7354287_G	15G	50G
Z_7354288_G	15G	50G
Z_7354289_A	15A	50A
Z_7354290_A	16A	50A
Z_7354291_A	16A	51A
Z_7354292_A	16A	50A
Z_7354293_C	16C	52C
Z_7354294_G	17G	52G
Z_7354295_G	17G	52G
Z_7354296_A	18A	51A
Z_7354297_A	18A	51A
Z_7354298_C	18C	50C
Z_7354299_C	17C	49C
Z_7354300_A	17A	50A
Z_7354301_C	17C	52C
Z_7354302_A	17A	50A
Z_7354303_T	17T	52T
Z_7354304_T	19T	51T
Z_7354305_C	20C	53C
Z_7354306_G	20G	53G
Z_7354307_G	20G	53G
Z_7354308_G	20G	55G
Z_7354309_C	20C	56C
Z_7354310_A	20A	56A
Z_7354311_A	21A	56A
Z_7354312_A	22A	55A
Z_7354313_G	22G	54G
Z_7354314_A	22A	57A
Z_7354315_G	22G	57G
Z_7354316_G	23C	57G
Z_7354317_G	23G	57G
Z_7354318_G	24G	59G
Z_7354319_T	24T	60T
Z_7354320_G	25G	61G
Z_7354321_C	25C	62C
Z_7354322_A	25A	64A
Z_7354323_A	25A	62A
Z_7354324_G	23G	61G
Z_7354325_C	25C	19A_62C
Z_7354326_A	24A	63A
Z_7354327_A	25A	62A
Z_7354328_A	25A	62A

Z_7354329_A	25A	62A
Z_7354330_A	26A	63A
Z_7354331_A	26T	63A
Z_7354332_A	29A	61A
Z_7354333_A	31A	61A
Z_7354334_A	31A	61A
Z_7354335_A	31A	62A
Z_7354336_T	31T	62T
Z_7354337_C	30C	59C
Z_7354338_T	32T	61T
Z_7354339_T	31T	61T
Z_7354340_T	30T	63T
Z_7354341_G	29G	62G
Z_7354342_C	29C	61C
Z_7354343_T	29T	59T
Z_7354344_T	28T	60T
Z_7354345_G	28G	60G
Z_7354346_A	28A	60A
Z_7354347_A	26A	60A
Z_7354348_C	28C	60C
Z_7354349_G	28G	61G
Z_7354350_T	29T	61T
Z_7354351_A	29A	61A
Z_7354352_A	29A	60A
Z_7354353_A	30A	60A
Z_7354354_A	29A	61A
Z_7354355_C	27C	60C
Z_7354356_A	28A	60A
Z_7354357_G	28G	61G
Z_7354358_C	28C	61C
Z_7354359_A	29A	60A
Z_7354360_A	28A	59A
Z_7354361_T	28T	59T
Z_7354362_A	27A	58A
Z_7354363_C	27C	60C
Z_7354364_C	27C	61C
Z_7354365_C	26C	59C
Z_7354366_C	26C	58C
Z_7354367_T	26T	58T
Z_7354368_A	26A	58A
Z_7354369_A	25A	58A
Z_7354370_C	25C	58C
Z_7354371_G	25G	58G

Z_7354372_C	25C	58C
Z_7354373_T	26T	61T
Z_7354374_T	26T	61T
Z_7354375_A	24A	61A
Z_7354376_C	26C	59C
Z_7354377_A	26A	59A
Z_7354378_C	25C	60C
Z_7354379_A	25A	60A
Z_7354380_C	25C	61C
Z_7354381_G	27G	60G
Z_7354382_G	26G	59G
Z_7354383_A	26A	61A
Z_7354384_C	25C	61C
Z_7354385_C	25C	60C
Z_7354386_A	25A	61A
Z_7354387_G	24G	61G
Z_7354388_A	25A	60A
Z_7354389_A	24A	61A
Z_7354390_A	24A	60A
Z_7354391_C	23C	61C
Z_7354392_T	23T	61T
Z_7354393_G	23G	62G
Z_7354394_G	24G	62G
Z_7354395_A	22A	60A
Z_7354396_T	22T	60T
Z_7354397_G	22G	60G
Z_7354398_G	22G	59G
Z_7354399_G	22G	59G
Z_7354400_A	21A	59A
Z_7354401_G	21G	59G
Z_7354402_C	20C	61C
Z_7354403_C	18C	59C
Z_7354404_C	18C	59C
Z_7354405_C	18C	60C
Z_7354406_T	18T	61T
Z_7354407_A	18A	62A
Z_7354408_G	17G	63G
Z_7354409_A	17A	63A
Z_7354410_A	17A	63A
Z_7354411_G	17G	64G
Z_7354412_G	16G	63G
Z_7354413_C	16C	31G_32C
Z_7354414_C	18C	61C

Z_7354415_T	17T	61T
Z_7354416_C	18C	59C
Z_7354417_C	20C	59C
Z_7354418_C	19C	59C
Z_7354419_A	19A	59A
Z_7354420_A	19A	60A
Z_7354421_G	19G	59G
Z_7354422_G	20G	59G
Z_7354423_C	19C	58C
Z_7354424_T	20T	58T
Z_7354425_G	20G	57G
Z_7354426_T	19T	57T
Z_7354427_T	19T	57T
Z_7354428_G	19G	58G
Z_7354429_C	18C	59C
Z_7354430_C	19C	57C
Z_7354431_G	20G	57G
Z_7354432_G	20G	58G
Z_7354433_A	20A	57A
Z_7354434_G	20G	56G
Z_7354435_A	21A	53A
Z_7354436_T	21T	54T
Z_7354437_C	19C	54C
Z_7354438_G	19G	53G
Z_7354439_T	19T	52T
Z_7354440_C	20C	52C
Z_7354441_A	21A	52A
Z_7354442_C	22C	51C
Z_7354443_C	21C	48C
Z_7354444_C	20C	47C
Z_7354445_G	20G	47G
Z_7354446_T	18T	47T
Z_7354447_T	18T	48T
Z_7354448_C	19C	50C
Z_7354449_A	20A	49A
Z_7354450_C	20C	51C
Z_7354451_A	18A	52A
Z_7354452_C	18C	51C
Z_7354453_G	17G	52G
Z_7354454_T	17T	52T
Z_7354455_G	17G	53G
Z_7354456_C	17C	53C
Z_7354457_A	17A	54A

Z_7354458_A	16A	53A
Z_7354459_C	16C	52C
Z_7354460_C	16C	54C
Z_7354461_C	15C	56C
Z_7354462_C	17C	57C
Z_7354463_C	17C	57C
Z_7354464_G	15G	59G
Z_7354465_C	16C	60C
Z_7354466_G	15G	60G
Z_7354467_C	16C	60C
Z_7354468_G	16G	57G
Z_7354469_G	15G	55G
Z_7354470_C	18C	57C
Z_7354471_C	18C	58C
Z_7354472_C	20C	61C
Z_7354473_T	22T	64T
Z_7354474_C	22C	64C
Z_7354475_C	22C	63C
Z_7354476_A	23A	63A
Z_7354477_C	23C	64C
Z_7354478_C	24C	62C
Z_7354479_C	25C	65C
Z_7354480_A	26A	65A
Z_7354481_A	27A	64A
Z_7354482_C	27C	64C
Z_7354483_C	28C	64C
Z_7354484_C	28C	63C
Z_7354485_G	28G	64G
Z_7354486_G	28G	64G
Z_7354487_G	27G	64G
Z_7354488_A	27A	63A
Z_7354489_G	27G	62G
Z_7354490_T	26T	63T
Z_7354491_C	25C	63C
Z_7354492_C	25C	63C
Z_7354493_C	28C	65C
Z_7354494_T	26T	65T
Z_7354495_C	28C	65C
Z_7354496_C	29C	65C
Z_7354497_G	31G	63G
Z_7354498_C	31C	63C
Z_7354499_T	30T	65T
Z_7354500_C	30C	64C

Z_7354501_A	30A	64A
Z_7354502_C	32C	64C
Z_7354503_C	32C	63C
Z_7354504_T	31T	62T
Z_7354505_T	30T	64T
Z_7354506_C	31C	67C
Z_7354507_C	32C	67C
Z_7354508_A	32A	66A
Z_7354509_G	32G	66G
Z_7354510_C	32C	67C
Z_7354511_C	31C	65C
Z_7354512_T	30T	64T
Z_7354513_C	31C	64C
Z_7354514_A	31A	63A
Z_7354515_T	32T	63T
Z_7354516_G	33G	62G
Z_7354517_C	34C	64C
Z_7354518_C	34C	63C
Z_7354519_C	32C	68C
Z_7354520_A	34A	69A
Z_7354521_C	33C	70C
Z_7354522_G	33G	71G
Z_7354523_G	35G	70G
Z_7354524_C	35C	70C
Z_7354525_C	35C	70C
Z_7354526_A	36A	69A
Z_7354527_G	37G	70G
Z_7354528_G	38G	70G
Z_7354529_C	38C	69C
Z_7354530_A	38A	70A
Z_7354531_C	38C	72C
Z_7354532_T	37T	74T
Z_7354533_T	36T	75T
Z_7354534_C	37C	76C
Z_7354535_T	38T	76T
Z_7354536_G	39G	77G
Z_7354537_G	38G	78G
Z_7354538_A	38A	77A
Z_7354539_A	40A	78A
Z_7354540_G	38G	77G
Z_7354541_C	38C	76C
Z_7354542_G	36G	77G
Z_7354543_G	35G	75G

Z_7354544_C	35C	73C
Z_7354545_A	34A	73A
Z_7354546_G	35G	72G
Z_7354547_T	34T	75T
Z_7354548_A	34A	76A
Z_7354549_G	34G	75G
Z_7354550_G	32G	76G
Z_7354551_G	32G	76G
Z_7354552_G	35G	76G
Z_7354553_C	33C	76C
Z_7354554_A	32A	77A
Z_7354555_C	35C	78C
Z_7354556_C	34C	75C
Z_7354557_T	34T	75T
Z_7354558_C	34C	79C
Z_7354559_T	34T	79T
Z_7354560_T	34T	81T
Z_7354561_G	34G	81G
Z_7354562_C	34C	82C
Z_7354563_G	31G	82G
Z_7354564_C	32C	82C
Z_7354565_T	31T	82T
Z_7354566_G	32G	82G
Z_7354567_C	31C	79C
Z_7354568_G	31G	76G
Z_7354569_T	31T	77T
Z_7354570_C	30C	76C
Z_7354571_T	31T	76T
Z_7354572_T	30T	76T
Z_7354573_G	32G	78G
Z_7354574_T	32T	75T
Z_7354575_C	32C	75C
Z_7354576_G	32G	74G
Z_7354577_A	31A	76A
Z_7354578_T	31T	77T
Z_7354579_C	31C	77C
Z_7354580_T	31T	76T
Z_7354581_T	31T	76T
Z_7354582_G	35G	78G
Z_7354583_C	34C	78C
Z_7354584_A	35A	81A
Z_7354585_G	35G	81G
Z_7354586_T	32T	81T

Z_7354587_T	33T	82T
Z_7354588_C	31C	83C
Z_7354589_T	32T	84T
Z_7354590_G	30G	85G
Z_7354591_G	31G	86G
Z_7354592_C	29C	85C
Z_7354593_T	30T	84T
Z_7354594_C	31C	84C
Z_7354595_T	31T	82T
Z_7354596_C	29C	82C
Z_7354597_G	29G	81G
Z_7354598_G	27G	83G
Z_7354599_T	28T	82T
Z_7354600_A	28A	81A
Z_7354601_C	27C	79C
Z_7354602_A	27A	80A
Z_7354603_C	27C	80C
Z_7354604_G	27G	81G
Z_7354605_T	26T	81T
Z_7354606_G	25G	82G
Z_7354607_T	26T	83T
Z_7354608_A	26A	83A
Z_7354609_A	26A	86A
Z_7354610_T	26T	86T
Z_7354611_G	27G	87G
Z_7354612_C	30C	87C
Z_7354613_T	29T	90T
Z_7354614_T	29T	86T
Z_7354615_G	29G	85G
Z_7354616_T	28T	85T
Z_7354617_T	28T	85T
Z_7354618_G	27G	84G
Z_7354619_T	27T	86T
Z_7354620_T	28T	88T
Z_7354621_C	28C	90C
Z_7354622_T	25T	89T
Z_7354623_G	24G	90G
Z_7354624_C	26C	90C
Z_7354625_A	23A	89A
Z_7354626_C	23C	88C
Z_7354627_G	24G	87G
Z_7354628_G	24G	88G
Z_7354629_T	24T	86T

Z_7354630_C	24C	87C
Z_7354631_C	24C	86C
Z_7354632_G	25G	85G
Z_7354633_C	23C	84C
Z_7354634_T	24T	84T
Z_7354635_T	26T	85T
Z_7354636_G	24G	85G
Z_7354637_A	25A	84A
Z_7354638_A	25A	85A
Z_7354639_G	25G	85G
Z_7354640_A	26A	85A
Z_7354641_A	25A	87A
Z_7354642_G	26G	87G
Z_7354643_C	25C	85C
Z_7354644_C	24C	86C
Z_7354645_C	25C	85C
Z_7354646_T	25T	84T
Z_7354647_G	25G	84G
Z_7354648_G	25G	84G
Z_7354649_A	27A	82A
Z_7354650_C	27C	81C
Z_7354651_G	27G	81G
Z_7354652_G	24G	81G
Z_7354653_G	24G	79G
Z_7354654_G	25G	78G
Z_7354655_A	24A	77A
Z_7354656_G	24G	80G
Z_7354657_A	25A	78A
Z_7354658_A	27A	78A
Z_7354659_A	27A	81A
Z_7354660_G	29G	81G
Z_7354661_C	29C	80C
Z_7354662_G	30G	80G
Z_7354663_A	30A	80A
Z_7354664_G	30G	82G
Z_7354665_C	30C	82C
Z_7354666_G	30G	82G
Z_7354667_A	30A	81A
Z_7354668_G	29A_30G	79G
Z_7354669_A	33A	79A
Z_7354670_A	33A	78A
Z_7354671_A	35A	79A
Z_7354672_A	35A	79A

Z_7354673_A	35A	78A
Z_7354674_A	36A	81A
Z_7354675_G	37G	84G
Z_7354676_A	39A	88A
Z_7354677_G	39G	87G
Z_7354678_A	38A	90A
Z_7354679_T	38T	88T
Z_7354680_G	38G	89G
Z_7354681_G	35G	89G
Z_7354682_G	35G	87G
Z_7354683_G	35G	87G
Z_7354684_G	36G	89G
Z_7354685_A	36A	88A
Z_7354686_C	35C	89C
Z_7354687_T	38T	89T
Z_7354688_G	38G	88G
Z_7354689_A	38A	87A
Z_7354690_G	41G	86G
Z_7354691_A	41A	88A
Z_7354692_C	41C	88C
Z_7354693_A	41A	86A
Z_7354694_C	41C	87C
Z_7354695_G	42G	89G
Z_7354696_G	43G	92G
Z_7354697_G	44G	92G
Z_7354698_G	44G	93G
Z_7354699_A	45A	93A
Z_7354700_C	45C	93C
Z_7354701_G	46G	94G
Z_7354702_G	47G	93G
Z_7354703_A	47A	93A
Z_7354704_A	47A	90A
Z_7354705_G	46G	90G
Z_7354706_A	47A	90A
Z_7354707_A	48A	89A
Z_7354708_G	49G	86G
Z_7354709_G	48G	86G
Z_7354710_C	47C	86C
Z_7354711_C	48C	85C
Z_7354712_A	47A	82A
Z_7354713_C	48C	82C
Z_7354714_G	48G	82G
Z_7354715_G	45G	80G

Z_7354716_A	47A	78A
Z_7354717_T	49T	79T
Z_7354718_A	46A	79A
Z_7354719_T	46T	80T
Z_7354720_T	45T	103T_27C
Z_7354721_T	45T	81T
Z_7354722_C	46C	82C
Z_7354723_T	42T	80T
Z_7354724_C	45C	80C
Z_7354725_T	45T	79T
Z_7354726_C	45C	80C
Z_7354727_C	43C	36T_45C
Z_7354728_C	42C	81C
Z_7354729_T	41T	81T
Z_7354730_C	44C	82C
Z_7354731_T	42T	83T
Z_7354732_C	43C	83C
Z_7354733_T	43T	84T
Z_7354734_T	43T	82T
Z_7354735_T	43T	86T
Z_7354736_T	45T	84T
Z_7354737_C	45C	80C
Z_7354738_T	44T	81T
Z_7354739_T	41T	82T
Z_7354740_T	41T	84T
Z_7354741_T	38T	84T
Z_7354742_T	40T	85T
Z_7354743_T	40T	86T
Z_7354744_T	42T	85T
Z_7354745_C	43C	85C
Z_7354746_A	42A	85A
Z_7354747_G	42G	87G
Z_7354748_T	42T	85T
Z_7354749_C	41C	83C
Z_7354750_G	42G	81G
Z_7354751_C	43C	81C
Z_7354752_T	43T	83T
Z_7354753_A	44A	84A
Z_7354754_T	43T	85T
Z_7354755_T	42T	86T
Z_7354756_T	42T	87T
Z_7354757_G	40G	87G
Z_7354758_G	40G	86G

Z_7354759_A	39A	85A
Z_7354760_G	38G	87G
Z_7354761_A	38A	86A
Z_7354762_A	39A	87A
Z_7354763_A	40A	87A
Z_7354764_T	40T	88T
Z_7354765_G	39G	87G
Z_7354766_C	37C	86C
Z_7354767_A	36A	87A
Z_7354768_C	36C	88C
Z_7354769_T	36T	85T
Z_7354770_T	33T	82T
Z_7354771_T	33T	78T
Z_7354772_A	33A	76A
Z_7354773_T	30T	75T
Z_7354774_C	32C	75C
Z_7354775_T	33T	76T
Z_7354776_A	31A	77A
Z_7354777_T	31T	76T
Z_7354778_G	30G	76G
Z_7354779_C	31C	72C
Z_7354780_T	32T	73T
Z_7354781_C	31C	76C
Z_7354782_A	32A	76A
Z_7354783_G	30G	77G
Z_7354784_G	31G	75G
Z_7354785_A	31A	75A
Z_7354786_A	31A	76A
Z_7354787_C	29C	78C
Z_7354788_A	29A	78A
Z_7354789_C	30C	77C
Z_7354790_G	31G	42T_31G
Z_7354791_C	31C	72C
Z_7354792_T	31T	70T
Z_7354793_C	33C	71C
Z_7354794_G	32G	73G
Z_7354795_C	32C	75C
Z_7354796_C	32C	74C
Z_7354797_T	34T	75T
Z_7354798_T	34T	77T
Z_7354799_T	33T	77T
Z_7354800_T	31T	76T
Z_7354801_C	32C	78C

Z_7354802_T	31T	77T
Z_7354803_C	31C	78C
Z_7354804_A	32A	80A
Z_7354805_G	30G	81G
Z_7354806_G	30G	81G
Z_7354807_C	32C	81C
Z_7354808_G	32G	81G
Z_7354809_T	33T	82T
Z_7354810_G	33G	83G
Z_7354811_C	30C	83C
Z_7354812_T	32T	82T
Z_7354813_A	33A	80A
Z_7354814_T	31T	80T
Z_7354815_A	30A	81A
Z_7354816_C	30T	37T_46C
Z_7354817_C	28C	83C
Z_7354818_C	30C	82C
Z_7354819_C	30C	82C
Z_7354820_A	30A	82A
Z_7354821_G	30G	82G
Z_7354822_A	30A	80A
Z_7354823_G	29G	82G
Z_7354824_T	31T	82T
Z_7354825_G	31G	80G
Z_7354826_A	31A	78A
Z_7354827_A	32A	78A
Z_7354828_G	31G	79G
Z_7354829_G	31G	79G
Z_7354830_G	31G	81G
Z_7354831_G	31G	80G
Z_7354832_T	29T	80T
Z_7354833_T	28C	80T
Z_7354834_G	30G	78G
Z_7354835_G	33G	79G
Z_7354836_T	34T	78T
Z_7354837_G	36G	77G
Z_7354838_C	38C	77C
Z_7354839_G	39G	77G
Z_7354840_G	40G	76G
Z_7354841_T	40T	75T
Z_7354842_C	40C	74C
Z_7354843_A	41A	74A
Z_7354844_G	41G	72G

Z_7354845_A	40A	72A
Z_7354846_T	40T	72T
Z_7354847_C	41C	71C
Z_7354848_A	41A	69A
Z_7354849_T	41T	66T
Z_7354850_C	40C	67C
Z_7354851_T	44T	65T
Z_7354852_T	45T	66T
Z_7354853_A	45A	67A
Z_7354854_G	45G	67G
Z_7354855_T	45T	65T
Z_7354856_C	45C	66C
Z_7354857_T	47T	67T
Z_7354858_C	48C	67C
Z_7354859_T	49T	66T
Z_7354860_C	49C	66C
Z_7354861_T	48T	66T
Z_7354862_C	48C	67C
Z_7354863_G	46G	67G
Z_7354864_G	47G	68G
Z_7354865_T	46T	68T
Z_7354866_T	44T	69T
Z_7354867_T	45T	68T
Z_7354868_A	45A	69A
Z_7354869_A	45A	71A
Z_7354870_A	45A	69A
Z_7354871_C	45C	68C
Z_7354872_C	46C	68C
Z_7354873_C	48C	67C
Z_7354874_C	48C	67C
Z_7354875_T	50T	66T
Z_7354876_T	51T	65T
Z_7354877_A	51A	67A
Z_7354878_A	54A	68A
Z_7354879_T	54T	70T
Z_7354880_T	55T	70T
Z_7354881_G	55G	70G
Z_7354882_C	54C	69C
Z_7354883_T	52T	68T
Z_7354884_C	53C	67C
Z_7354885_T	53T	68T
Z_7354886_T	52T	68T
Z_7354887_T	52T	69T

Z_7354888_A	51A	70A
Z_7354889_A	51A	67A
Z_7354890_A	53A	64A
Z_7354891_A	53A	64A
Z_7354892_T	56T	65T
Z_7354893_A	56A	63A
Z_7354894_C	54C	64C
Z_7354895_C	54C	65C
Z_7354896_C	55C	64C
Z_7354897_T	55T	64T
Z_7354898_T	54T	63T
Z_7354899_A	54A	61A
Z_7354900_C	55C	60C
Z_7354901_A	57A	62A
Z_7354902_A	57A	63A
Z_7354903_T	56T	63T
Z_7354904_T	56T	62T
Z_7354905_A	52A	62A
Z_7354906_T	52T	64T
Z_7354907_T	51T	65T
Z_7354908_A	50A	65A
Z_7354909_A	49A	67A
Z_7354910_T	49T	65T
Z_7354911_T	49T	63T
Z_7354912_C	49C	64C
Z_7354913_A	48A	65A
Z_7354914_C	50C	65C
Z_7354915_C	49C	65C
Z_7354916_T	50T	34T_31G
Z_7354917_A	48A	65A
Z_7354918_T	47T	64T
Z_7354919_T	48T	64T
Z_7354920_G	47G	64G
Z_7354921_A	43A	65A
Z_7354922_G	43G	65G
Z_7354923_T	42T	65T
Z_7354924_A	42A	64A
Z_7354925_G	41G	60G
Z_7354926_G	42G	60G
Z_7354927_T	41T	60T
Z_7354928_A	40A	59A
Z_7354929_G	37G	59G
Z_7354930_G	37G	60G

Z_7354931_T	38T	60T
Z_7354932_C	38C	61C
Z_7354933_A	39A	60A
Z_7354934_C	40C	59C
Z_7354935_T	39T	59T
Z_7354936_A	39A	58A
Z_7354937_A	39A	61A
Z_7354938_C	40C	63C
Z_7354939_G	40G	64G
Z_7354940_C	38C	64C
Z_7354941_G	38G	65G
Z_7354942_A	38A	64A
Z_7354943_G	36G	64G
Z_7354944_T	35T	65T
Z_7354945_A	33A	65A
Z_7354946_A	32A	65A
Z_7354947_T	30T	65T
Z_7354948_T	28T	63T
Z_7354949_T	27T	64T
Z_7354950_A	27A	63A
Z_7354951_A	28A	63A
Z_7354952_T	30T	62T
Z_7354953_T	30T	62T
Z_7354954_A	31A	63A
Z_7354955_T	30T	64T
Z_7354956_G	29G	65G
Z_7354957_C	29C	64C
Z_7354958_T	29T	62T
Z_7354959_A	29A	60A
Z_7354960_A	27A	61A
Z_7354961_T	26T	62T
Z_7354962_C	25C	65C
Z_7354963_G	25G	64G
Z_7354964_C	25C	61C
Z_7354965_C	25C	63C
Z_7354966_G	24G	64G
Z_7354967_C	23C	65C
Z_7354968_T	26T	66T
Z_7354969_T	26T	67T
Z_7354970_T	26C	67T
Z_7354971_A	25A	65A
Z_7354972_G	25G	63G
Z_7354973_C	26C	62C

Z_7354974_T	27T	60T
Z_7354975_A	28A	61A
Z_7354976_A	29A	62A
Z_7354977_C	28C	61C
Z_7354978_G	28G	61G
Z_7354979_A	28A	62A
Z_7354980_A	27A	61A
Z_7354981_G	27G	63G
Z_7354982_G	26G	60G
Z_7354983_G	28G	65G
Z_7354984_A	27A	68A
Z_7354985_A	28A	69A
Z_7354986_A	29A	69A
Z_7354987_T	30T	67T
Z_7354988_G	29G	67G
Z_7354989_G	30G	66G
Z_7354990_G	31G	64G
Z_7354991_C	32C	65C
Z_7354992_A	34A	65A
Z_7354993_T	35T	65T
Z_7354994_A	35A	65A
Z_7354995_A	35A	65A
Z_7354996_G	34G	63G
Z_7354997_A	35A	64A
Z_7354998_G	35G	64G
Z_7354999_G	34G	64G
Z_7355000_G	36G	63G
Z_7355001_G	37A	61G
Z_7355002_T	37T	60T
Z_7355003_C	36C	60C
Z_7355004_T	37T	58T
Z_7355005_C	37C	57C
Z_7355006_C	38C	56C
Z_7355007_C	38C	58C
Z_7355008_T	37T	57T
Z_7355009_T	38T	58T
Z_7355010_T	39T	58T
Z_7355011_C	42C	58C
Z_7355012_A	43A	57A
Z_7355013_G	42G	58G
Z_7355014_G	42G	59G
Z_7355015_A	42A	59A
Z_7355016_A	42A	58A

Z_7355017_C	42C	58C
Z_7355018_T	40T	59T
Z_7355019_C	40C	60C
Z_7355020_G	40G	61G
Z_7355021_G	39G	62G
Z_7355022_G	36G	63G
Z_7355023_G	36G	62G
Z_7355024_A	35A	63A
Z_7355025_A	35A	64A
Z_7355026_G	35G	63G
Z_7355027_G	37G	63G
Z_7355028_A	34A	63A
Z_7355029_G	50T_84G_150C	63G
Z_7355030_G	null	64G
Z_7355031_C	null	64C
Z_7355032_C	null	62C
Z_7355033_C	null	61C
Z_7355034_C	null	58C
Z_7355035_T	null	61T
Z_7355036_C	null	61C
Z_7355037_C	null	64C
Z_7355038_G	1G	64G
Z_7355039_T	1A_1G	64T
Z_7355040_G	35G	63G
Z_7355041_T	35T	66T
Z_7355042_T	35T	66T
Z_7355043_C	37C	66C
Z_7355044_A	37A	65A
Z_7355045_C	37C	65C
Z_7355046_T	36T	65T
Z_7355047_T	37T	65T
Z_7355048_C	38C	65C
Z_7355049_A	38A	66A
Z_7355050_G	38G	65G
Z_7355051_A	37A	67A
Z_7355052_T	38T	66T
Z_7355053_G	38G	66G
Z_7355054_C	38C	67C
Z_7355055_G	37G	67G
Z_7355056_A	40A	66A
Z_7355057_A	41A	63A
Z_7355058_A	41A	63A
Z_7355059_G	41G	64G

Z_7355060_C	41C	63C
Z_7355061_A	44A	64A
Z_7355062_A	43A	66A
Z_7355063_C	43C	69C
Z_7355064_G	43G	68G
Z_7355065_A	44A	68A
Z_7355066_G	44G	70G
Z_7355067_A	43A	71A
Z_7355068_C	43C	71C
Z_7355069_G	42G	72G
Z_7355070_G	41G	72G
Z_7355071_T	45T	69T
Z_7355072_T	44T	70T
Z_7355073_C	42C	70C
Z_7355074_C	43C	69C
Z_7355075_T	43T	69T
Z_7355076_G	44G	67G
Z_7355077_G	43G	67G
Z_7355078_G	43G	26A_37G
Z_7355079_G	43G	60G
Z_7355080_C	44C	59C
Z_7355081_A	41A	59A
Z_7355082_T	40T	59T
Z_7355083_C	40C	63C
Z_7355084_G	40G	63G
Z_7355085_A	40A	63A
Z_7355086_T	40T	63T
Z_7355087_T	39T	63T
Z_7355088_A	39A	64A
Z_7355089_C	40C	65C
Z_7355090_G	39G	65G
Z_7355091_T	38T	65T
Z_7355092_G	35G	65G
Z_7355093_T	34T	65T
Z_7355094_T	34C	65T
Z_7355095_T	33T	66T
Z_7355096_T	32T	66T
Z_7355097_T	32T	65T
Z_7355098_T	32T	65T
Z_7355099_T	32T	67T
Z_7355100_T	31T	67T
Z_7355101_T	31T	67T
Z_7355102_T	25A	21A_41T

Z_7355103_A	26A	62A
Z_7355104_A	99A_25C	62A
Z_7355105_A	40A	124A
Z_7355106_C	38C	62C
Z_7355107_G	38G	63G
Z_7355108_T	39T	62T
Z_7355109_C	39C	62C
Z_7355110_G	39G	63G
Z_7355111_G	41G	64G
Z_7355112_A	41A	65A
Z_7355113_A	40A	64A
Z_7355114_A	43A	64A
Z_7355115_G	43G	65G
Z_7355116_A	43A	63A
Z_7355117_T	44T	62T
Z_7355118_T	44T	68T
Z_7355119_T	43T	68T
Z_7355120_T	43T	67T
Z_7355121_C	42C	67C
Z_7355122_C	40C	67C
Z_7355123_G	38G	67G
Z_7355124_C	38C	66C
Z_7355125_T	38T	65T
Z_7355126_T	38T	65T
Z_7355127_A	35A	64A
Z_7355128_A	35A	63A
Z_7355129_G	35G	63G
Z_7355130_C	34C	61C
Z_7355131_C	32C	61C
Z_7355132_G	32G	59G
Z_7355133_G	32G	59G
Z_7355134_T	31T	59T
Z_7355135_C	31C	18T_41C
Z_7355136_C	31C	56C
Z_7355137_G	27G	56G
Z_7355138_A	28A	56A
Z_7355139_A	27A	56A
Z_7355140_A	26G	36A_1T_19G
Z_7355141_C	26C	55C
Z_7355142_C	25C	55C
Z_7355143_C	25C	55C
Z_7355144_A	25A	53A
Z_7355145_A	24A	53A

Z_7355146_G	23G	48G
Z_7355147_T	24C	31T_2G_17C
Z_7355148_C	24C	51C
Z_7355149_G	24G	50G
Z_7355150_C	25C	50C
Z_7355151_C	25C	49C
Z_7355152_T	25T	47T
Z_7355153_C	25C	47C
Z_7355154_C	25C	45C
Z_7355155_A	25A	44A
Z_7355156_A	24A	44A
Z_7355157_T	24T	41T
Z_7355158_A	24A	37A
Z_7355159_T	24T	36T
Z_7355160_C	23C	35C
Z_7355161_T	23T	33T
Z_7355162_C	23C	33C
Z_7355163_A	22A	32A
Z_7355164_T	22T	32T
Z_7355165_C	22C	32C
Z_7355166_T	20T	32T
Z_7355167_T	19T	33T
Z_7355168_G	17G	34G
Z_7355169_A	13G	26A_8G
Z_7355170_A	10A	34A
Z_7355171_G	9G	34G
Z_7355172_G	8G	34G
Z_7355173_C	8C	34C
Z_7355174_G	7G	33G
Z_7355175_G	7G	33G
Z_7355176_C	12C	34C
Z_7355177_T	14T	34T
Z_7355178_C	15C	32C
Z_7355179_C	15C	33C
Z_7355180_A	16A	33A
Z_7355181_A	16A	33A
Z_7355182_T	16T	33T
Z_7355183_A	16A	32A
Z_7355184_T	19T	31T
Z_7355185_C	20C	31C
Z_7355186_T	20T	31T
Z_7355187_C	22C	31C
Z_7355188_A	23A	31A

Z_7355189_T	22T	29T
Z_7355190_C	22C	28C
Z_7355191_T	24T	29T
Z_7355192_T	25T	29T
Z_7355193_G	25G	29G
Z_7355194_G	25G	27G
Z_7355195_A	27A	27A
Z_7355196_G	25G	26G
Z_7355197_G	27G	25G
Z_7355198_C	28C	29C
Z_7355199_G	28G	29G
Z_7355200_G	28G	30G
Z_7355201_A	29A	30A
Z_7355202_T	30T	31T
Z_7355203_G	30G	32G
Z_7355204_A	31A	31A
Z_7355205_T	32T	32T
Z_7355206_G	33G	31G
Z_7355207_A	32A	29A
Z_7355208_G	34G	29G
Z_7355209_A	34A	29A
Z_7355210_G	34G	28G
Z_7355211_A	34A	28A
Z_7355212_T	34T	28T
Z_7355213_G	34G	26G
Z_7355214_T	34T	27T
Z_7355215_A	35A	26A
Z_7355216_G	35G	9T_26G
Z_7355217_T	35T	29T
Z_7355218_C	31T	27C
Z_7355219_C	30C	29C
Z_7355220_C	29C	30C
Z_7355221_C	29C	30C
Z_7355222_C	28C	33C
Z_7355223_C	26C	33C
Z_7355224_C	28C	33C
Z_7355225_C	26C	32C
Z_7355226_C	27C	33C
Z_7355227_C	27C	33C
Z_7355228_T	26T	29T
Z_7355229_T	28T	30T
Z_7355230_T	28T	31T
Z_7355231_C	27C	32C

Z_7355232_T	28T	32T
Z_7355233_G	27A	11A_1T_20G_1C
Z_7355234_G	27G	34G
Z_7355235_C	30C	36C
Z_7355236_C	31C	35C
Z_7355237_A	31A	34A
Z_7355238_G	32G	35G
Z_7355239_C	31C	36C
Z_7355240_A	33A	35A
Z_7355241_C	32C	36C
Z_7355242_C	33C	36C
Z_7355243_C	32C	37C
Z_7355244_C	33C	37C
Z_7355245_T	32T	38T
Z_7355246_T	30T	38T
Z_7355247_A	31A	38A
Z_7355248_G	30G	38G
Z_7355249_G	31G	40G
Z_7355250_A	32A	40A
Z_7355251_G	31G	40G
Z_7355252_A	32A	39A
Z_7355253_C	34C	39C
Z_7355254_A	30A	40A
Z_7355255_G	31G	41G
Z_7355256_C	30C	42C
Z_7355257_T	29T	41T
Z_7355258_G	29G	41G
Z_7355259_C	29C	43C
Z_7355260_G	29G	43G
Z_7355261_T	28T	44T
Z_7355262_G	27G	43G
Z_7355263_G	27G	44G
Z_7355264_A	27A	43A
Z_7355265_G	26G	43G
Z_7355266_A	26A	43A
Z_7355267_C	27C	43C
Z_7355268_T	26T	43T
Z_7355269_T	27T	43T
Z_7355270_T	26T	44T
Z_7355271_G	27G	44G
Z_7355272_C	27C	44C
Z_7355273_G	27G	44G
Z_7355274_C	26C	44C

Z_7355275_T	25T	44T
Z_7355276_C	24C	44C
Z_7355277_T	24T	45T
Z_7355278_G	24G	45G
Z_7355279_A	24A	46A
Z_7355280_T	24T	47T
Z_7355281_A	24A	48A
Z_7355282_C	24C	48C
Z_7355283_C	24C	49C
Z_7355284_C	25C	49C
Z_7355285_A	23A	51A
Z_7355286_C	24C	53C
Z_7355287_A	25A	53A
Z_7355288_G	25G	53G
Z_7355289_G	24G	54G
Z_7355290_A	25A	54A
Z_7355291_T	25T	54T
Z_7355292_C	25C	55C
Z_7355293_G	25G	52G
Z_7355294_G	26G	51G
Z_7355295_G	26G	50G
Z_7355296_A	25A	49A
Z_7355297_G	28G	48G
Z_7355298_T	28T	48T
Z_7355299_A	28A	32A_18G
Z_7355300_G	29G	50G
Z_7355301_C	30C	50C
Z_7355302_A	32A	50A
Z_7355303_T	32T	50T
Z_7355304_C	30C	51C
Z_7355305_C	29C	51C
Z_7355306_T	31C	31T_19C
Z_7355307_C	33C	51C
Z_7355308_G	33G	52G
Z_7355309_G	33G	52G
Z_7355310_C	31C	52C
Z_7355311_G	31G	53G
Z_7355312_C	33C	52C
Z_7355313_T	34T	43T
Z_7355314_T	34T	52T
Z_7355315_C	34C	51C
Z_7355316_T	34T	55T
Z_7355317_T	32T	53T

Z_7355318_T	33T	55T
Z_7355319_T	31T	55T
Z_7355320_G	33G	55G
Z_7355321_C	33C	55C
Z_7355322_G	32G	56G
Z_7355323_C	31C	56C
Z_7355324_C	33C	56C
Z_7355325_G	33G	55G
Z_7355326_T	33T	56T
Z_7355327_A	34A	55A
Z_7355328_T	32T	54T
Z_7355329_C	33C	51C
Z_7355330_C	32C	52C
Z_7355331_T	33T	52T
Z_7355332_A	33A	52A
Z_7355333_T	33T	53T
Z_7355334_C	32C	53C
Z_7355335_G	32G	54G
Z_7355336_G	32G	53G
Z_7355337_C	32C	54C
Z_7355338_C	32C	53C
Z_7355339_A	32A	53A
Z_7355340_C	32C	55C
Z_7355341_G	31G	57G
Z_7355342_T	30T	59T
Z_7355343_T	30T	58T
Z_7355344_T	30T	59T
Z_7355345_A	31A	59A
Z_7355346_A	30A	59A
Z_7355347_A	31A	59A
Z_7355348_A	31A	59A
Z_7355349_C	33C	59C
Z_7355350_A	33A	60A
Z_7355351_C	34C	59C
Z_7355352_A	34A	58A
Z_7355353_C	34C	59C
Z_7355354_C	33C	56C
Z_7355355_C	33C	57C
Z_7355356_G	33G	56G
Z_7355357_C	33C	57C
Z_7355358_A	33A	57A
Z_7355359_A	33A	57A
Z_7355360_T	33T	56T

Z_7355361_C	34C	59C
Z_7355362_T	34T	60T
Z_7355363_G	33G	61G
Z_7355364_G	34G	62G
Z_7355365_A	36A	61A
Z_7355366_G	38G	61G
Z_7355367_G	36G	61G
Z_7355368_A	36A	60A
Z_7355369_G	37G	60G
Z_7355370_A	36A	60A
Z_7355371_G	36G	60G
Z_7355372_A	35A	59A
Z_7355373_A	35A	60A
Z_7355374_A	36A	61A
Z_7355375_A	36A	60A
Z_7355376_A	34A	59A
Z_7355377_A	33A	58A
Z_7355378_C	33C	58C
Z_7355379_T	33T	58T
Z_7355380_C	33C	57C
Z_7355381_C	32C	54C
Z_7355382_G	30G	57G
Z_7355383_C	29C	56C
Z_7355384_G	28G	57G
Z_7355385_T	28T	56T
Z_7355386_T	28T	55T
Z_7355387_T	28T	56T
Z_7355388_T	27T	57T
Z_7355389_T	27T	58T
Z_7355390_T	25T	59T
Z_7355391_T	25T	59T
Z_7355392_T	25T	59T
Z_7355393_T	16A	59T
Z_7355394_T	14A	55T
Z_7355395_T	14A	56T
Z_7355396_A	70A	57A
Z_7355397_A	23A	57A
Z_7355398_A	25A	57A
Z_7355399_T	27T	58T
Z_7355400_T	29T	60T
Z_7355401_G	29G	61G
Z_7355402_A	27A	60A
Z_7355403_T	29T	60T

Z_7355404_T	30T	59T
Z_7355405_T	30T	59T
Z_7355406_G	30G	58G
Z_7355407_A	31A	58A
Z_7355408_A	31A	58A
Z_7355409_T	31T	58T
Z_7355410_T	33T	59T
Z_7355411_A	33A	59A
Z_7355412_G	33G	56G
Z_7355413_G	32G	55G
Z_7355414_G	34G	55G
Z_7355415_C	34C	56C
Z_7355416_A	34A	57A
Z_7355417_A	35A	59A
Z_7355418_T	35T	59T
Z_7355419_T	35T	59T
Z_7355420_C	36C	59C
Z_7355421_G	36G	57G
Z_7355422_T	35T	57T
Z_7355423_T	35T	57T
Z_7355424_T	35T	58T
Z_7355425_T	35T	56T
Z_7355426_A	34A	28A_28T
Z_7355427_A	35A	59A
Z_7355428_A	36A	58A
Z_7355429_A	34A	57A
Z_7355430_A	33A	56A
Z_7355431_A	32A	56A
Z_7355432_A	31A	56A
Z_7355433_A	32A	55A
Z_7355434_G	31C	55G
Z_7355435_T	31T	56T
Z_7355436_T	31T	52T
Z_7355437_T	30T	53T
Z_7355438_A	30A	53A
Z_7355439_G	29G	52G
Z_7355440_C	29C	55C
Z_7355441_A	30A	55A
Z_7355442_A	30A	57A
Z_7355443_A	31G	57A
Z_7355444_C	32C	57C
Z_7355445_C	32C	59C
Z_7355446_T	32T	57T

Z_7355447_C	34C	57C
Z_7355448_C	33C	57C
Z_7355449_C	33C	57C
Z_7355450_C	33C	57C
Z_7355451_A	34A	57A
Z_7355452_A	34A	57A
Z_7355453_A	33A	56A
Z_7355454_A	33A	56A
Z_7355455_G	32G	59G
Z_7355456_T	33T	59T
Z_7355457_A	33A	57A
Z_7355458_T	33T	57T
Z_7355459_G	33G	59G
Z_7355460_C	33C	59C
Z_7355461_C	33C	59C
Z_7355462_T	32T	60T
Z_7355463_A	31A	60A
Z_7355464_A	31A	60A
Z_7355465_A	31A	60A
Z_7355466_G	30G	61G
Z_7355467_G	28G	62G
Z_7355468_G	26G	64G
Z_7355469_T	25T	61T
Z_7355470_G	22G	61G
Z_7355471_T	24T	63T
Z_7355472_A	25A	63A
Z_7355473_A	25A	62A
Z_7355474_G	25G	63G
Z_7355475_C	26C	62C
Z_7355476_T	27T	62T
Z_7355477_C	28C	60C
Z_7355478_C	28C	61C
Z_7355479_T	28T	60T
Z_7355480_C	26C	60C
Z_7355481_G	26G	60G
Z_7355482_G	28G	59G
Z_7355483_G	28G	59G
Z_7355484_G	27G	59G
Z_7355485_C	27C	58C
Z_7355486_A	26A	59A
Z_7355487_A	25A	59A
Z_7355488_C	25C	59C
Z_7355489_A	26A	59A

Z_7355490_C	26C	57C
Z_7355491_C	25C	57C
Z_7355492_T	25T	58T
Z_7355493_T	24T	58T
Z_7355494_T	25T	58T
Z_7355495_T	27T	57T
Z_7355496_C	26C	58C
Z_7355497_C	27C	58C
Z_7355498_T	26T	58T
Z_7355499_A	26A	58A
Z_7355500_T	26T	58T
Z_7355501_T	26T	58T
Z_7355502_T	26T	58T
Z_7355503_G	24G	58G
Z_7355504_T	24T	59T
Z_7355505_G	24G	59G
Z_7355506_C	23C	59C
Z_7355507_A	23A	59A
Z_7355508_T	23T	59T
Z_7355509_A	23A	58A
Z_7355510_G	23G	57G
Z_7355511_C	22C	57C
Z_7355512_T	22T	55T
Z_7355513_G	20G	56G
Z_7355514_C	20C	56C
Z_7355515_G	20C	29G_27C
Z_7355516_G	19G	56G
Z_7355517_C	19C	56C
Z_7355518_G	18G	57G
Z_7355519_T	20T	56T
Z_7355520_G	18G	56G
Z_7355521_G	19G	56G
Z_7355522_C	20C	55C
Z_7355523_A	20A	56A
Z_7355524_A	21A	56A
Z_7355525_T	20T	57T
Z_7355526_C	21C	58C
Z_7355527_A	22A	56A
Z_7355528_C	22C	57C
Z_7355529_A	23A	56A
Z_7355530_C	23C	57C
Z_7355531_A	23A	55A
Z_7355532_C	23C	54C

Z_7355533_C	23C	52C
Z_7355534_A	22A	52A
Z_7355535_C	23C	50C
Z_7355536_A	23A	49A
Z_7355537_C	23C	48C
Z_7355538_G	23G	48G
Z_7355539_A	23A	48A
Z_7355540_C	23C	45C
Z_7355541_T	22T	43T
Z_7355542_C	20C	47C
Z_7355543_A	21G	47A
Z_7355544_T	20T	49T
Z_7355545_T	19T	49T
Z_7355546_T	17T	49T
Z_7355547_A	16A	50A
Z_7355548_A	16A	48A
Z_7355549_A	17A	48A
Z_7355550_T	17T	46T
Z_7355551_A	17A	45A
Z_7355552_A	15A	45A
Z_7355553_T	14T	44T
Z_7355554_A	15A	41A
Z_7355555_G	15G	41G
Z_7355556_A	15T	40A
Z_7355557_C	14C	41C
Z_7355558_G	15G	41G
Z_7355559_C	14C	41C
Z_7355560_A	14A	40A
Z_7355561_A	14A	36A
Z_7355562_C	10G_14C	38C
Z_7355563_G	16G	37G
Z_7355564_G	16G	38G
Z_7355565_G	16G	38G
Z_7355566_G	16G	36G
Z_7355567_G	16G	37G
Z_7355568_G	15G	37G
Z_7355569_G	17G	36G
Z_7355570_G	16G	37G
Z_7355571_G	18G	37G
Z_7355572_G	18G	37G
Z_7355573_C	18C	37C
Z_7355574_A	19A	37A
Z_7355575_G	18G	37G

Z_7355576_C	18C	37C
Z_7355577_T	18T	37T
Z_7355578_G	19G	40G
Z_7355579_C	19C	42C
Z_7355580_T	20T	41T
Z_7355581_T	20T	43T
Z_7355582_T	20T	46T
Z_7355583_A	19A	47A
Z_7355584_T	21T	47T
Z_7355585_T	21T	47T
Z_7355586_C	22C	48C
Z_7355587_C	22C	47C
Z_7355588_A	23A	45A
Z_7355589_C	24C	48C
Z_7355590_A	24A	49A
Z_7355591_C	24C	46C
Z_7355592_C	25C	45C
Z_7355593_A	24A	46A
Z_7355594_G	24G	47G
Z_7355595_T	24T	48T
Z_7355596_G	24G	48G
Z_7355597_G	24G	48G
Z_7355598_C	23C	50C
Z_7355599_G	25G	18A_31G
Z_7355600_G	23G	47G
Z_7355601_G	24G	46G
Z_7355602_A	25A	43A
Z_7355603_C	25C	48C
Z_7355604_C	23C	48C
Z_7355605_T	23T	48T
Z_7355606_A	23A	48A
Z_7355607_A	24A	48A
Z_7355608_A	25A	46A
Z_7355609_G	25G	46G
Z_7355610_A	25A	46A
Z_7355611_A	26A	46A
Z_7355612_G	26G	47G
Z_7355613_T	26T	45T
Z_7355614_C	27C	46C
Z_7355615_C	26C	46C
Z_7355616_T	27T	47T
Z_7355617_C	29C	47C
Z_7355618_G	29G	45G

Z_7355619_G	30G	46G
Z_7355620_A	31A	47A
Z_7355621_A	31A	49A
Z_7355622_G	32G	49G
Z_7355623_T	33T	48T
Z_7355624_C	33C	49C
Z_7355625_C	32C	49C
Z_7355626_C	33C	49C
Z_7355627_A	33A	49A
Z_7355628_G	34G	49G
Z_7355629_A	33A	49A
Z_7355630_G	33G	49G
Z_7355631_A	33A	50A
Z_7355632_T	33T	52T
Z_7355633_T	34T	52T
Z_7355634_C	34C	54C
Z_7355635_C	33C	54C
Z_7355636_T	31T	52T
Z_7355637_C	33C	50C
Z_7355638_T	34T	51T
Z_7355639_G	34G	50G
Z_7355640_A	32A	49A
Z_7355641_G	34G	50G
Z_7355642_C	33C	51C
Z_7355643_A	33A	51A
Z_7355644_T	34T	51T
Z_7355645_G	34G	52G
Z_7355646_T	34T	52T
Z_7355647_A	34A	52A
Z_7355648_C	35C	53C
Z_7355649_A	35A	53A
Z_7355650_A	35A	54A
Z_7355651_A	36A	54A
Z_7355652_G	37G	55G
Z_7355653_C	37C	55C
Z_7355654_G	35G	56G
Z_7355655_C	36C	56C
Z_7355656_G	34G	56G
Z_7355657_C	34C	55C
Z_7355658_A	33A	54A
Z_7355659_G	32G	54G
Z_7355660_C	34C	54C
Z_7355661_A	33A	52A

Z_7355662_C	32G	53C
Z_7355663_G	33G	52G
Z_7355664_T	32T	53T
Z_7355665_C	33C	52C
Z_7355666_T	32T	52T
Z_7355667_T	31T	53T
Z_7355668_G	31G	54G
Z_7355669_G	28G	54G
Z_7355670_C	29C	56C
Z_7355671_C	30C	55C
Z_7355672_C	28C	56C
Z_7355673_C	28C	55C
Z_7355674_A	29A	53A
Z_7355675_C	29C	52C
Z_7355676_C	28T	52C
Z_7355677_C	28C	52C
Z_7355678_C	27C	52C
Z_7355679_G	27G	51G
Z_7355680_A	25A	48A
Z_7355681_G	26G	49G
Z_7355682_C	26C	50C
Z_7355683_C	25C	49C
Z_7355684_G	25G	49G
Z_7355685_G	25G	48G
Z_7355686_C	24C	50C
Z_7355687_C	23C	50C
Z_7355688_A	31A_45G_18C	49A
Z_7355689_G	12G	51G
Z_7355690_G	1G	50G
Z_7355691_A	null	49A
Z_7355692_C	null	48C
Z_7355693_C	null	48C
Z_7355694_G	null	47G
Z_7355695_G	null	50G
Z_7355696_G	null	51G
Z_7355697_A	12A	50A
Z_7355698_G	12G	49G
Z_7355699_A	12A	50A
Z_7355700_G	12G	49G
Z_7355701_G	12G	49G
Z_7355702_A	12A	49A
Z_7355703_G	12G	49G
Z_7355704_A	12C	49A

Z_7355705_T	12T	49T
Z_7355706_G	14G	49G
Z_7355707_A	15T	47A
Z_7355708_G	16G	48G
Z_7355709_A	16A	48A
Z_7355710_C	16C	49C
Z_7355711_C	17C	48C
Z_7355712_C	17C	46C
Z_7355713_C	17C	50C
Z_7355714_C	16C	49C
Z_7355715_T	17T	48T
Z_7355716_A	18A	46A
Z_7355717_A	18A	46A
Z_7355718_C	19C	47C
Z_7355719_T	19T	46T
Z_7355720_G	19G	49G
Z_7355721_G	18G	48G
Z_7355722_G	18G	48G
Z_7355723_G	19G	49G
Z_7355724_T	19T	51T
Z_7355725_G	19G	52G
Z_7355726_C	20C	51C
Z_7355727_T	19T	50T
Z_7355728_G	19G	50G
Z_7355729_A	17A	49A
Z_7355730_G	17G	51G
Z_7355731_C	16C	52C
Z_7355732_T	15T	48T
Z_7355733_C	16C	51C
Z_7355734_T	15T	53T
Z_7355735_A	17A	53A
Z_7355736_G	16G	54G
Z_7355737_A	17A	52A
Z_7355738_T	17T	53T
Z_7355739_C	17C	52C
Z_7355740_C	18C	54C
Z_7355741_C	18C	54C
Z_7355742_A	18A	54A
Z_7355743_T	18T	54T
Z_7355744_C	19C	55C
Z_7355745_C	19C	29A_26C
Z_7355746_T	20T	55T
Z_7355747_C	20C	55C

Z_7355748_C	19C	55C
Z_7355749_T	18T	53T
Z_7355750_C	19C	54C
Z_7355751_C	18C	54C
Z_7355752_G	18G	54G
Z_7355753_G	18A	24A_30G
Z_7355754_G	16G	54G
Z_7355755_C	16C	54C
Z_7355756_C	16C	54C
Z_7355757_A	15A	54A
Z_7355758_G	15G	54G
Z_7355759_C	15C	55C
Z_7355760_A	15A	55A
Z_7355761_G	15G	55G
Z_7355762_G	42A_15G_28C	51A_54G_34C
Z_7355763_A	null	29A
Z_7355764_C	null	31C
Z_7355765_C	null	29C
Z_7355766_A	null	29A
Z_7355767_A	null	29A
Z_7355768_G	14G	47G
Z_7355769_A	15G	30A_17G
Z_7355770_C	24C	50C
Z_7355771_T	24T	50T
Z_7355772_C	24C	50C
Z_7355773_A	24A	50A
Z_7355774_C	24C	50C
Z_7355775_G	25G	50G
Z_7355776_G	24G	50G
Z_7355777_C	25C	50C
Z_7355778_T	24T	51T
Z_7355779_C	25C	51C
Z_7355780_A	25A	51A
Z_7355781_T	25T	49T
Z_7355782_G	25G	49G
Z_7355783_G	24G	48G
Z_7355784_A	24A	48A
Z_7355785_A	24A	48A
Z_7355786_T	25T	48T
Z_7355787_C	24C	49C
Z_7355788_T	24T	49T
Z_7355789_G	24G	49G
Z_7355790_G	23G	47G

Z_7355791_A	24A	48A
Z_7355792_G	25G	19A_31G
Z_7355793_T	26T	52T
Z_7355794_C	25C	52C
Z_7355795_C	26C	53C
Z_7355796_A	26A	53A
Z_7355797_T	27T	54T
Z_7355798_C	26C	54C
Z_7355799_A	27A	55A
Z_7355800_A	27A	56A
Z_7355801_C	28C	56C
Z_7355802_A	27A	58A
Z_7355803_T	28T	58T
Z_7355804_C	28C	58C
Z_7355805_T	30T	58T
Z_7355806_G	31G	58G
Z_7355807_G	31G	58G
Z_7355808_A	32A	57A
Z_7355809_G	34G	57G
Z_7355810_G	34G	57G
Z_7355811_G	32G	56G
Z_7355812_C	31C	56C
Z_7355813_A	31A	55A
Z_7355814_G	32G	53G
Z_7355815_C	32C	51C
Z_7355816_A	31A	51A
Z_7355817_A	31A	52A
Z_7355818_T	31T	52T
Z_7355819_C	31C	52C
Z_7355820_T	30T	52T
Z_7355821_C	31C	53C
Z_7355822_A	30A	54A
Z_7355823_A	31A	54A
Z_7355824_A	30A	52A
Z_7355825_T	29T	49T
Z_7355826_T	27T	48T
Z_7355827_C	29C	47C
Z_7355828_T	28T	47T
Z_7355829_T	27T	45T
Z_7355830_T	26T	45T
Z_7355831_A	25A	45A
Z_7355832_A	25A	44A
Z_7355833_T	24T	43T

Z_7355834_T	26T	46T
Z_7355835_A	26A	45A
Z_7355836_A	26A	45A
Z_7355837_G	26G	46G
Z_7355838_G	28G	45G
Z_7355839_C	27C	46C
Z_7355840_T	28T	44T
Z_7355841_A	29A	42A
Z_7355842_C	30C	42C
Z_7355843_C	31C	42C
Z_7355844_T	33T	51T_27C
Z_7355845_C	33C	44C
Z_7355846_T	31T	41T
Z_7355847_C	33C	43C
Z_7355848_C	34C	43C
Z_7355849_C	36C	48C
Z_7355850_C	37C	42C
Z_7355851_C	37C	42C
Z_7355852_C	37C	42C
Z_7355853_C	1A_33T_1G_56C	42C
Z_7355854_T	35T	30T_12C
Z_7355855_T	38T	29T_13C
Z_7355856_T	37T	44T
Z_7355857_T	37T	44T
Z_7355858_T	37T	44T
Z_7355859_T	38T	44T
Z_7355860_T	39T	44T
Z_7355861_T	40T	44T
Z_7355862_T	38T	30T_14C
Z_7355863_C	37C	44C
Z_7355864_C	37C	44C
Z_7355865_T	36T	44T
Z_7355866_T	36T	44T
Z_7355867_T	37T	42T
Z_7355868_T	38T	42T
Z_7355869_G	35G	42G
Z_7355870_G	36G	40G
Z_7355871_T	35T	44T
Z_7355872_T	35T	46T
Z_7355873_T	34T	46T
Z_7355874_C	34C	47C
Z_7355875_A	35A	47A
Z_7355876_A	33A	47A

Z_7355877_A	32A	47A
Z_7355878_C	33C	46C
Z_7355879_T	33T	45T
Z_7355880_G	34G	44G
Z_7355881_A	33A	44A
Z_7355882_T	33T	45T
Z_7355883_G	32G	45G
Z_7355884_T	32T	45T
Z_7355885_A	33A	45A
Z_7355886_T	33T	42T
Z_7355887_G	32G	38G
Z_7355888_C	33C	36C
Z_7355889_C	31C	38C
Z_7355890_T	32T	38T
Z_7355891_C	31C	38C
Z_7355892_G	31G	38G
Z_7355893_A	30A	37A
Z_7355894_A	31A	38A
Z_7355895_A	29A	37A
Z_7355896_T	29T	37T
Z_7355897_A	29A	35A
Z_7355898_C	29C	35C
Z_7355899_C	28C	36C
Z_7355900_A	29A	36A
Z_7355901_A	29A	36A
Z_7355902_A	27A	36A
Z_7355903_A	26A	35A
Z_7355904_A	26A	36A
Z_7355905_A	26A	36A
Z_7355906_G	null	22G
Z_7355907_A	null	22A
Z_7355908_T	null	22T
Z_7355909_G	null	24G
Z_7355910_C	null	24C
Z_7355911_G	2G	25G
Z_7355912_G	2G	26G
Z_7355913_G	2G	25G
Z_7355914_T	2T	25T
Z_7355915_T	2T	25T
Z_7355916_T	2T	25T
Z_7355917_A	2A	24A
Z_7355918_T	2T	25T
Z_7355919_G	2G	25G

Z_7355920_C	2C	26C
Z_7355921_G	2G	25G
Z_7355922_C	null	25C
Z_7355923_G	1G	25G
Z_7355924_C	2G	24C
Z_7355925_G	7G	24G
Z_7355926_G	7G	24G
Z_7355927_G	7G	24G
Z_7355928_G	7G	24G
Z_7355929_G	7G	24G
Z_7355930_G	7G	22G
Z_7355931_G	7G	22G
Z_7355932_T	7G	22T
Z_7355933_T	7T	22T
Z_7355934_G	7G	24G
Z_7355935_A	7A	22A
Z_7355936_G	8G	21G
Z_7355937_C	9C	21C
Z_7355938_T	9T	21T
Z_7355939_T	9G	20T
Z_7355940_C	10C	23C
Z_7355941_G	12G	23G
Z_7355942_T	13T	23T
Z_7355943_A	14G	20A
Z_7355944_T	19T	23T
Z_7355945_T	19T	23T
Z_7355946_G	19G	23G
Z_7355947_G	21G	24G
Z_7355948_C	21C	23C
Z_7355949_C	21C	23C
Z_7355950_A	23A	22A
Z_7355951_T	24T	22T
Z_7355952_C	24C	22C
Z_7355953_C	23C	22C
Z_7355954_G	26G	22G
Z_7355955_T	27T	22T
Z_7355956_G	27G	22G
Z_7355957_G	26G	25G
Z_7355958_T	28T	24T
Z_7355959_T	28T	24T
Z_7355960_T	29T	25T
Z_7355961_A	28A	25A
Z_7355962_A	30A	25A

Z_7355963_C	28C	25C
Z_7355964_A	28A	25A
Z_7355965_G	28G	25G
Z_7355966_C	29C	23C
Z_7355967_T	29T	24T
Z_7355968_G	28G	24G
Z_7355969_G	28G	24G
Z_7355970_G	26G	25G
Z_7355971_A	28A	25A
Z_7355972_G	28G	26G
Z_7355973_G	28G	26G
Z_7355974_C	30C	27C
Z_7355975_C	29C	27C
Z_7355976_G	27G	27G
Z_7355977_A	26A	28A
Z_7355978_A	28A	28A
Z_7355979_C	29C	28C
Z_7355980_G	29G	28G
Z_7355981_A	30A	29A
Z_7355982_G	30G	29G
Z_7355983_G	29G	31G
Z_7355984_C	30C	29C
Z_7355985_C	31C	29C
Z_7355986_G	32G	31G
Z_7355987_A	32A	32A
Z_7355988_A	33A	32A
Z_7355989_C	34C	31C
Z_7355990_T	34T	30T
Z_7355991_G	34G	30G
Z_7355992_A	35A	30A
Z_7355993_A	37A	29A
Z_7355994_A	36A	29A
Z_7355995_A	38A	28A
Z_7355996_T	39T	28T
Z_7355997_C	40C	28C
Z_7355998_G	40G	29G
Z_7355999_C	37C	29C
Z_7356000_C	40C	29C
Z_7356001_C	41C	29C
Z_7356002_C	42C	29C
Z_7356003_A	43A	29A
Z_7356004_A	44A	27A
Z_7356005_T	45T	27T

Z_7356006_A	45A	28A
Z_7356007_A	44A	29A
Z_7356008_A	44A	30A
Z_7356009_T	44T	30T
Z_7356010_T	44T	30T
Z_7356011_G	43G	30G
Z_7356012_T	41T	30T
Z_7356013_C	40C	31C
Z_7356014_A	42A	32A
Z_7356015_A	42A	33A
Z_7356016_C	43C	33C
Z_7356017_G	41G	33G
Z_7356018_A	41A	32A
Z_7356019_C	42C	33C
Z_7356020_G	39G	33G
Z_7356021_T	39T	34T
Z_7356022_T	39T	35T
Z_7356023_T	40T	35T
Z_7356024_A	38A	36A
Z_7356025_A	38A	36A
Z_7356026_T	38T	36T
Z_7356027_A	38A	38A
Z_7356028_A	38A	38A
Z_7356029_A	38A	36A
Z_7356030_A	37A	37A
Z_7356031_G	37G	37G
Z_7356032_T	36T	37T
Z_7356033_T	37T	36T
Z_7356034_C	37C	37C
Z_7356035_C	37C	37C
Z_7356036_C	35C	38C
Z_7356037_A	36A	38A
Z_7356038_C	36C	38C
Z_7356039_A	37A	38A
Z_7356040_A	37A	38A
Z_7356041_G	36G	39G
Z_7356042_G	36G	38G
Z_7356043_G	36G	39G
Z_7356044_A	36A	40A
Z_7356045_A	36A	40A
Z_7356046_A	36A	40A
Z_7356047_G	36G	41G
Z_7356048_T	22A_56T_21G	14A_55T_14G

Z_7356049_G	36G	43G
Z_7356050_T	36T	43T
Z_7356051_A	35A	43A
Z_7356052_T	23T_12G	36T
Z_7356053_T	35T	41T
Z_7356054_A	35A	42A
Z_7356055_T	34T	41T
Z_7356056_T	9T_23G	24T_20G
Z_7356057_A	32A	41A
Z_7356058_C	23C	40C
Z_7356059_T	24T	39T
Z_7356060_G	25G	41G
Z_7356061_G	26G	41G
Z_7356062_G	25G	39G
Z_7356063_A	25A	39A
Z_7356064_A	24A	37A
Z_7356065_T	25T	37T
Z_7356066_G	25G	35G
Z_7356067_A	25A	36A
Z_7356068_C	24C	37C
Z_7356069_G	23G	36G
Z_7356070_T	23T	35T
Z_7356071_T	24T	37T
Z_7356072_T	22T	37T
Z_7356073_C	22C	39C
Z_7356074_C	22C	39C
Z_7356075_C	22C	38C
Z_7356076_T	22T	37T
Z_7356077_A	21A	37A
Z_7356078_G	20G	36G
Z_7356079_G	20G	35G
Z_7356080_G	47A_14G	42A_33G
Z_7356081_A	3A	19A
Z_7356082_A	3A	20A
Z_7356083_A	2T	20A
Z_7356084_A	16A	34A
Z_7356085_A	18A	34A
Z_7356086_A	17A	34A
Z_7356087_A	17T	20A_14T
Z_7356088_A	20A	36A
Z_7356089_A	20A	39A
Z_7356090_A	20A	39A
Z_7356091_A	20A	40A

Z_7356092_G	19G	40G
Z_7356093_A	21A	40A
Z_7356094_A	22A	40A
Z_7356095_A	22A	40A
Z_7356096_G	20G	40G
Z_7356097_A	20G	23A_17G
Z_7356098_G	21G	40G
Z_7356099_T	20T	40T
Z_7356100_A	21A	40A
Z_7356101_T	23T	38T
Z_7356102_T	23T	37T
Z_7356103_T	23T	37T
Z_7356104_T	22T	36T
Z_7356105_T	23T	36T
Z_7356106_A	23A	37A
Z_7356107_G	23G	39G
Z_7356108_T	23T	38T
Z_7356109_G	22G	40G
Z_7356110_G	21G	37G
Z_7356111_G	23G	38G
Z_7356112_G	23G	38G
Z_7356113_G	23G	38G
Z_7356114_A	23A	38A
Z_7356115_A	21A	39A
Z_7356116_G	21G	41G
Z_7356117_G	24G	38G
Z_7356118_T	24T	39T
Z_7356119_T	24T	38T
Z_7356120_T	24T	39T
Z_7356121_T	25T	41T
Z_7356122_G	25G	39G
Z_7356123_A	24A	40A
Z_7356124_G	27G	40G
Z_7356125_C	27C	39C
Z_7356126_A	27A	39A
Z_7356127_A	28A	39A
Z_7356128_T	27T	39T
Z_7356129_T	29T	39T
Z_7356130_G	28G	37G
Z_7356131_C	28C	37C
Z_7356132_T	28T	36T
Z_7356133_A	29A	37A
Z_7356134_A	28A	37A

Z_7356135_A	28A	37A
Z_7356136_T	29T	38T
Z_7356137_G	30A	14A_23G
Z_7356138_A	30A	36A
Z_7356139_G	30G	36G
Z_7356140_G	31G	35G
Z_7356141_A	31A	36A
Z_7356142_T	32T	35T
Z_7356143_A	32A	36A
Z_7356144_A	31A	35A
Z_7356145_T	30T	35T
Z_7356146_T	32T	34T
Z_7356147_A	32A	34A
Z_7356148_T	33T	34T
Z_7356149_T	32T	35T
Z_7356150_A	34A	36A
Z_7356151_G	34G	38G
Z_7356152_T	36T	37T
Z_7356153_G	36G	37G
Z_7356154_G	38G	37G
Z_7356155_G	36G	38G
Z_7356156_G	36G	38G
Z_7356157_G	34G	38G
Z_7356158_A	34A	38A
Z_7356159_G	33G	37G
Z_7356160_G	34G	38G
Z_7356161_T	33T	39T
Z_7356162_T	32T	41T
Z_7356163_T	34T	42T
Z_7356164_T	33T	41T
Z_7356165_G	34G	42G
Z_7356166_T	35T	40T
Z_7356167_T	35T	40T
Z_7356168_T	36T	39T
Z_7356169_A	37A	39A
Z_7356170_A	37A	40A
Z_7356171_T	36T	40T
Z_7356172_T	37T	39T
Z_7356173_G	37G	38G
Z_7356174_T	38T	40T
Z_7356175_T	38T	40T
Z_7356176_G	39G	39G
Z_7356177_C	39C	38C

Z_7356178_A	40A	37A
Z_7356179_T	42T	38T
Z_7356180_A	41A	38A
Z_7356181_G	40G	39G
Z_7356182_G	40G	39G
Z_7356183_A	39A	40A
Z_7356184_T	38T	37T
Z_7356185_A	38A	38A
Z_7356186_A	37A	37A
Z_7356187_T	35T	37T
Z_7356188_T	37T	38T
Z_7356189_A	37A	38A
Z_7356190_C	37C	38C
Z_7356191_T	38T	38T
Z_7356192_G	39G	38G
Z_7356193_G	39G	38G
Z_7356194_G	37G	11T_38G
Z_7356195_T	35G	40T
Z_7356196_T	37T	42T
Z_7356197_T	37T	43T
Z_7356198_T	37T	42T
Z_7356199_T	36T	44T
Z_7356200_T	36T	44T
Z_7356201_T	37T	44T
Z_7356202_A	34A	42A
Z_7356203_A	35A	44A
Z_7356204_T	34T	42T
Z_7356205_G	35G	44G
Z_7356206_T	37T	44T
Z_7356207_T	37T	44T
Z_7356208_T	37T	44T
Z_7356209_A	38A	44A
Z_7356210_A	38A	43A
Z_7356211_G	39G	40G
Z_7356212_A	39A	40A
Z_7356213_C	39C	41C
Z_7356214_A	41A	40A
Z_7356215_C	43C	39C
Z_7356216_T	43T	37T
Z_7356217_T	44T	38T
Z_7356218_C	44C	38C
Z_7356219_C	43C	39C
Z_7356220_G	45G	39G

Z_7356221_C	44C	39C
Z_7356222_T	45T	39T
Z_7356223_T	43T	39T
Z_7356224_T	42T	39T
Z_7356225_C	41C	39C
Z_7356226_T	43T	40T
Z_7356227_C	43C	39C
Z_7356228_C	43C	40C
Z_7356229_T	42T	40T
Z_7356230_C	42C	40C
Z_7356231_T	42T	40T
Z_7356232_G	42G	40G
Z_7356233_G	42G	40G
Z_7356234_C	43C	39C
Z_7356235_C	43C	39C
Z_7356236_T	42T	39T
Z_7356237_C	42C	40C
Z_7356238_A	42A	40A
Z_7356239_G	40G	40G
Z_7356240_T	41T	41T
Z_7356241_A	42A	41A
Z_7356242_G	40G	41G
Z_7356243_G	39G	40G
Z_7356244_G	40G	41G
Z_7356245_G	39G	40G
Z_7356246_G	38G	38G
Z_7356247_A	38G	19A_17G
Z_7356248_C	38C	35C
Z_7356249_A	37A	34A
Z_7356250_T	36T	34T
Z_7356251_C	36C	34C
Z_7356252_C	36C	34C
Z_7356253_A	36A	35A
Z_7356254_G	37G	35G
Z_7356255_C	38C	35C
Z_7356256_C	39C	33C
Z_7356257_C	39C	32C
Z_7356258_T	40T	31T
Z_7356259_C	40C	31C
Z_7356260_T	41T	31T
Z_7356261_G	42G	32G
Z_7356262_C	42C	33C
Z_7356263_C	42C	33C

Z_7356264_C	44C	32C
Z_7356265_C	45C	32C
Z_7356266_A	45A	32A
Z_7356267_C	45C	31C
Z_7356268_A	46A	31A
Z_7356269_G	45G	31G
Z_7356270_T	47T	31T
Z_7356271_G	46G	30G
Z_7356272_G	46G	33G
Z_7356273_G	46G	33G
Z_7356274_C	45C	32C
Z_7356275_T	46T	32T
Z_7356276_C	44C	30C
Z_7356277_C	43C	31C
Z_7356278_T	43T	32T
Z_7356279_T	42T	31T
Z_7356280_T	42T	31T
Z_7356281_C	41C	31C
Z_7356282_T	42T	31T
Z_7356283_G	41G	32G
Z_7356284_C	41C	32C
Z_7356285_C	39C	33C
Z_7356286_C	39C	32C
Z_7356287_C	37C	33C
Z_7356288_A	37A	32A
Z_7356289_C	37C	33C
Z_7356290_G	35G	32G
Z_7356291_G	34G	30G
Z_7356292_G	35G	29G
Z_7356293_T	35T	28T
Z_7356294_G	35G	29G
Z_7356295_C	36C	30C
Z_7356296_A	34A	30A
Z_7356297_A	35A	28A
Z_7356298_A	35A	29A
Z_7356299_G	36G	29G
Z_7356300_T	35T	27T
Z_7356301_C	36C	26C
Z_7356302_C	35C	26C
Z_7356303_G	35G	26G
Z_7356304_C	36C	26C
Z_7356305_G	36G	26G
Z_7356306_C	35C	26C

Z_7356307_C	37C	26C
Z_7356308_C	36C	26C
Z_7356309_C	37C	26C
Z_7356310_T	35T	25T
Z_7356311_T	34T	26T
Z_7356312_C	34C	25C
Z_7356313_C	36C	26C
Z_7356314_A	35A	25A
Z_7356315_C	37C	26C
Z_7356316_G	37G	26G
Z_7356317_C	37C	26C
Z_7356318_C	38C	26C
Z_7356319_T	37T	26T
Z_7356320_G	37G	26G
Z_7356321_C	37C	25C
Z_7356322_C	37C	25C
Z_7356323_A	36A	24A
Z_7356324_A	37A	24A
Z_7356325_G	36G	25G
Z_7356326_G	35G	25G
Z_7356327_G	33G	26G
Z_7356328_A	33A	26A
Z_7356329_A	31A	27A
Z_7356330_G	14A	6A_20G
Z_7356331_A	13G	20A_5G
Z_7356332_G	13A	6A_18G
Z_7356333_G	15G	24G
Z_7356334_A	15A	24A
Z_7356335_C	15C	24C
Z_7356336_A	15A	24A
Z_7356337_C	15T	5T_20C
Z_7356338_C	16C	25C
Z_7356339_G	16A	5A_19G
Z_7356340_A	16A	24A
Z_7356341_C	16C	25C
Z_7356342_C	16C	26C
Z_7356343_T	17G	20T_6G
Z_7356344_G	26G	33G
Z_7356345_C	26C	33C
Z_7356346_A	26A	33A
Z_7356347_G	26G	33G
Z_7356348_G	26G	33G
Z_7356349_C	27C	35C

Z_7356350_T	28T	35T
Z_7356351_C	28C	35C
Z_7356352_C	28T	14T_21C
Z_7356353_G	31G	35G
Z_7356354_A	31A	36A
Z_7356355_G	32G	38G
Z_7356356_C	34C	39C
Z_7356357_G	35G	39G
Z_7356358_C	35C	39C
Z_7356359_C	36C	39C
Z_7356360_C	37C	39C
Z_7356361_A	37A	39A
Z_7356362_T	36T	40T
Z_7356363_C	36C	40C
Z_7356364_C	36C	38C
Z_7356365_A	36A	38A
Z_7356366_G	36G	38G
Z_7356367_A	36A	36A
Z_7356368_G	34G	36G
Z_7356369_A	35A	36A
Z_7356370_T	35T	36T
Z_7356371_G	35G	36G
Z_7356372_T	34T	34T
Z_7356373_T	36T	34T
Z_7356374_G	36G	35G
Z_7356375_C	35C	35C
Z_7356376_G	35G	35G
Z_7356377_G	33G	34G
Z_7356378_G	34G	33G
Z_7356379_G	39G	33G
Z_7356380_C	39C	32C
Z_7356381_G	39G	33G
Z_7356382_C	39C	32C
Z_7356383_A	36A	32A
Z_7356384_A	35A	32A
Z_7356385_A	33A	32A
Z_7356386_C	35C	33C
Z_7356387_C	33C	32C
Z_7356388_T	35T	33T
Z_7356389_G	36G	32G
Z_7356390_T	36T	30T
Z_7356391_T	35T	32T
Z_7356392_T	35T	32T

Z_7356393_G	35G	31G
Z_7356394_G	36G	31G
Z_7356395_A	35A	32A
Z_7356396_T	36T	32T
Z_7356397_G	35G	32G
Z_7356398_G	35G	32G
Z_7356399_C	36C	32C
Z_7356400_G	35G	32G
Z_7356401_C	35C	32C
Z_7356402_G	35G	32G
Z_7356403_C	34C	32C
Z_7356404_A	34A	32A
Z_7356405_A	35A	32A
Z_7356406_G	35G	32G
Z_7356407_G	36G	33G
Z_7356408_A	35A	32A
Z_7356409_G	36G	32G
Z_7356410_A	36A	32A
Z_7356411_A	36A	32A
Z_7356412_T	35T	32T
Z_7356413_C	37C	32C
Z_7356414_G	36G	13T_19G
Z_7356415_T	37T	32T
Z_7356416_C	37C	32C
Z_7356417_C	37C	31C
Z_7356418_C	37C	31C
Z_7356419_A	37A	31A
Z_7356420_C	35C	31C
Z_7356421_C	36C	29C
Z_7356422_C	36C	29C
Z_7356423_A	35A	27A
Z_7356424_C	34C	25C
Z_7356425_T	35T	25T
Z_7356426_C	33C	25C
Z_7356427_T	34T	25T
Z_7356428_C	32C	25C
Z_7356429_G	33G	25G
Z_7356430_A	32A	25A
Z_7356431_A	33A	25A
Z_7356432_G	33G	24G
Z_7356433_G	31G	24G
Z_7356434_T	32T	24T
Z_7356435_G	32G	23G

Z_7356436_G	30G	22G
Z_7356437_A	33A	22A
Z_7356438_A	32A	22A
Z_7356439_C	33C	21C
Z_7356440_A	34A	21A
Z_7356441_A	34A	21A
Z_7356442_G	34G	21G
Z_7356443_C	32C	19C
Z_7356444_C	31C	18C
Z_7356445_G	31G	18G
Z_7356446_G	31G	18G
Z_7356447_T	30T	10T_8C
Z_7356448_C	31C	18C
Z_7356449_A	26A	17A
Z_7356450_G	26G	16G
Z_7356451_A	26A	14A
Z_7356452_T	27T	13T
Z_7356453_T	27T	13T
Z_7356454_A	27A	13A
Z_7356455_T	27T	13T
Z_7356456_T	25T	12T
Z_7356457_G	40G	12G
Z_7356458_G	8G	13G
Z_7356459_G	22G	15G
Z_7356460_G	23G	15G
Z_7356461_G	23G	15G
Z_7356462_G	23G	15G
Z_7356463_G	23G	15G
Z_7356464_G	21G	15G
Z_7356465_G	17G	15G
Z_7356466_G	18G	16G
Z_7356467_C	17C	16C
Z_7356468_G	18A	17G
Z_7356469_C	19C	17C
Z_7356470_C	19C	15C
Z_7356471_C	20C	17C
Z_7356472_A	20A	17A
Z_7356473_G	20G	17G
Z_7356474_G	20G	17G
Z_7356475_T	21T	17T
Z_7356476_T	19T	17T
Z_7356477_T	19T	17T
Z_7356478_T	19T	16T

Z_7356479_T	18T	17T
Z_7356480_C	18C	18C
Z_7356481_T	18T	13T_6C
Z_7356482_C	18C	19C
Z_7356483_T	18T	19T
Z_7356484_C	17C	21C
Z_7356485_T	16T	22T
Z_7356486_T	16T	20T
Z_7356487_C	16C	21C
Z_7356488_C	19C	22C
Z_7356489_T	21T	22T
Z_7356490_T	21T	25T
Z_7356491_G	21G	26G
Z_7356492_G	21G	28G
Z_7356493_G	20G	28G
Z_7356494_T	23G	20T_8G
Z_7356495_T	24T	28T
Z_7356496_G	26G	30G
Z_7356497_G	25G	31G
Z_7356498_G	28G	31G
Z_7356499_G	28G	31G
Z_7356500_T	27T	30T
Z_7356501_G	28G	34G
Z_7356502_G	28G	33G
Z_7356503_G	27G	34G
Z_7356504_A	29A	33A
Z_7356505_G	31G	33G
Z_7356506_T	32T	34T
Z_7356507_T	32T	35T
Z_7356508_A	32A	35A
Z_7356509_G	31G	35G
Z_7356510_G	32G	35G
Z_7356511_A	32A	37A
Z_7356512_A	31A	37A
Z_7356513_A	31A	37A
Z_7356514_A	32A	37A
Z_7356515_C	32C	39C
Z_7356516_A	32A	38A
Z_7356517_G	33G	38G
Z_7356518_G	35G	38G
Z_7356519_G	35G	38G
Z_7356520_T	37T	38T
Z_7356521_G	38G	39G

Z_7356522_A	38A	39A
Z_7356523_A	37A	40A
Z_7356524_G	36G	41G
Z_7356525_G	34G	41G
Z_7356526_C	37C	41C
Z_7356527_C	36C	42C
Z_7356528_A	37A	42A
Z_7356529_A	38A	42A
Z_7356530_C	39C	42C
Z_7356531_A	39A	43A
Z_7356532_C	41C	43C
Z_7356533_C	41C	42C
Z_7356534_C	41C	43C
Z_7356535_A	43A	44A
Z_7356536_T	43T	45T
Z_7356537_T	44T	47T
Z_7356538_T	44T	48T
Z_7356539_T	43T	50T
Z_7356540_A	42A	53A
Z_7356541_G	42G	53G
Z_7356542_G	42G	54G
Z_7356543_T	43T	55T
Z_7356544_T	42T	55T
Z_7356545_A	43A	55A
Z_7356546_A	43A	56A
Z_7356547_T	44T	56T
Z_7356548_T	44T	56T
Z_7356549_C	44C	56C
Z_7356550_C	47C	57C
Z_7356551_A	47A	57A
Z_7356552_T	48T	58T
Z_7356553_G	48G	59G
Z_7356554_T	48T	57T
Z_7356555_G	46G	59G
Z_7356556_G	47G	60G
Z_7356557_A	44A	60A
Z_7356558_C	45C	60C
Z_7356559_T	43T	60T
Z_7356560_G	43G	59G
Z_7356561_G	46G	61G
Z_7356562_G	46G	60G
Z_7356563_A	45A	59A
Z_7356564_T	46T	59T

Z_7356565_A	45A	59A
Z_7356566_G	44G	59G
Z_7356567_C	42C	59C
Z_7356568_A	43A	59A
Z_7356569_T	42T	60T
Z_7356570_T	45T	61T
Z_7356571_A	45A	62A
Z_7356572_A	45A	63A
Z_7356573_A	45A	64A
Z_7356574_A	44A	64A
Z_7356575_C	43C	63C
Z_7356576_A	43A	63A
Z_7356577_A	44A	62A
Z_7356578_A	47A	63A
Z_7356579_A	47A	62A
Z_7356580_T	46T	61T
Z_7356581_T	45T	61T
Z_7356582_C	49C	60C
Z_7356583_A	49A	59A
Z_7356584_A	48A	58A
Z_7356585_A	49A	56A
Z_7356586_A	49A	55A
Z_7356587_A	47A	56A
Z_7356588_T	47T	57T
Z_7356589_A	48A	58A
Z_7356590_T	46T	57T
Z_7356591_A	44A	58A
Z_7356592_T	45T	58T
Z_7356593_T	46T	59T
Z_7356594_T	47T	60T
Z_7356595_T	47T	58T
Z_7356596_T	46T	58T
Z_7356597_C	46C	58C
Z_7356598_C	47C	58C
Z_7356599_A	46A	57A
Z_7356600_G	45G	59G
Z_7356601_T	44T	58T
Z_7356602_T	42T	58T
Z_7356603_A	43A	59A
Z_7356604_A	44A	60A
Z_7356605_A	42A	60A
Z_7356606_A	42A	60A
Z_7356607_T	41T	63T

Z_7356608_A	42A	62A
Z_7356609_A	43A	64A
Z_7356610_G	43G	62G
Z_7356611_G	45G	63G
Z_7356612_G	47A	63G
Z_7356613_A	47A	65A
Z_7356614_G	47G	67G
Z_7356615_C	48C	67C
Z_7356616_A	47A	67A
Z_7356617_T	47T	67T
Z_7356618_A	47A	66A
Z_7356619_G	48G	67G
Z_7356620_A	46A	67A
Z_7356621_C	47C	68C
Z_7356622_G	47G	67G
Z_7356623_G	47G	67G
Z_7356624_A	48A	67A
Z_7356625_C	48C	69C
Z_7356626_T	51T	68T
Z_7356627_C	56C	68C
Z_7356628_A	54A	70A
Z_7356629_A	54A	69A
Z_7356630_C	53C	68C
Z_7356631_A	51A	67A
Z_7356632_G	53G	29A_36G
Z_7356633_C	54C	65C
Z_7356634_A	54A	63A
Z_7356635_A	53A	60A
Z_7356636_T	53T	60T
Z_7356637_A	53A	59A
Z_7356638_C	51C	59C
Z_7356639_A	52A	60A
Z_7356640_G	52G	59G
Z_7356641_A	53A	61A
Z_7356642_A	53A	61A
Z_7356643_T	54T	62T
Z_7356644_T	54T	62T
Z_7356645_T	53T	63T
Z_7356646_T	52T	63T
Z_7356647_T	51T	63T
Z_7356648_A	49A	34A_26T_1G
Z_7356649_A	48A	61A
Z_7356650_A	52A	59A

Z_7356651_A	53A	58A
Z_7356652_G	50G	58G
Z_7356653_C	52C	59C
Z_7356654_C	52C	26T_33C
Z_7356655_T	51T	61T
Z_7356656_T	52T	59T
Z_7356657_T	53T	59T
Z_7356658_A	51A	60A
Z_7356659_T	51T	61T
Z_7356660_A	52A	61A
Z_7356661_A	52A	62A
Z_7356662_A	52A	63A
Z_7356663_G	51G	63G
Z_7356664_T	50T	63T
Z_7356665_T	49T	64T
Z_7356666_A	51A	62A
Z_7356667_A	51A	61A
Z_7356668_A	52A	61A
Z_7356669_C	52C	61C
Z_7356670_T	53T	63T
Z_7356671_A	54A	62A
Z_7356672_A	54A	62A
Z_7356673_G	55G	61G
Z_7356674_A	54A	63A
Z_7356675_T	54T	63T
Z_7356676_T	55T	62T
Z_7356677_T	55T	62T
Z_7356678_T	55C	28T_34C
Z_7356679_C	54C	64C
Z_7356680_C	51C	64C
Z_7356681_C	49C	65C
Z_7356682_T	51T	63T
Z_7356683_G	50G	62G
Z_7356684_G	51G	62G
Z_7356685_G	49G	62G
Z_7356686_C	49C	61C
Z_7356687_G	47G	62G
Z_7356688_G	47G	61G
Z_7356689_G	47G	61G
Z_7356690_G	45G	61G
Z_7356691_G	47G	60G
Z_7356692_A	46A	60A
Z_7356693_A	47A	60A

Z_7356694_G	47G	60G
Z_7356695_A	47A	58A
Z_7356696_G	42G	59G
Z_7356697_A	40A	57A
Z_7356698_A	40A	58A
Z_7356699_C	41C	59C
Z_7356700_C	41C	59C
Z_7356701_C	41C	57C
Z_7356702_G	39G	56G
Z_7356703_C	40C	58C
Z_7356704_C	37C	25G_32C
Z_7356705_T	39T	57T
Z_7356706_G	39G	57G
Z_7356707_A	42A	56A
Z_7356708_A	41A	56A
Z_7356709_A	40A	55A
Z_7356710_A	39A	56A
Z_7356711_T	38T	55T
Z_7356712_A	40A	55A
Z_7356713_T	39T	56T
Z_7356714_T	40T	55T
Z_7356715_A	40A	56A
Z_7356716_A	41A	55A
Z_7356717_T	41T	55T
Z_7356718_T	41T	56T
Z_7356719_C	43C	55C
Z_7356720_G	40G	55G
Z_7356721_T	39T	30T_26C
Z_7356722_A	42A	56A
Z_7356723_A	42A	54A
Z_7356724_A	41A	55A
Z_7356725_A	44A	56A
Z_7356726_A	45A	56A
Z_7356727_A	46A	58A
Z_7356728_A	46A	57A
Z_7356729_G	47G	28T_29G
Z_7356730_T	46T	58T
Z_7356731_A	46A	59A
Z_7356732_G	46G	59G
Z_7356733_T	50T	58T
Z_7356734_T	50T	58T
Z_7356735_C	51C	58C
Z_7356736_C	51C	56C

Z_7356737_C	53C	57C
Z_7356738_A	52A	58A
Z_7356739_T	52T	60T
Z_7356740_T	51T	59T
Z_7356741_A	50A	62A
Z_7356742_A	49A	61A
Z_7356743_A	49A	63A
Z_7356744_A	49A	62A
Z_7356745_G	47G	62G
Z_7356746_G	46G	63G
Z_7356747_G	83T_46G	51T_63G
Z_7356748_T	53T	64T
Z_7356749_T	54T	64T
Z_7356750_T	54T	62T
Z_7356751_T	57T	62T
Z_7356752_T	56T	62T
Z_7356753_T	57T	62T
Z_7356754_T	56T	61T
Z_7356755_C	55C	61C
Z_7356756_C	55C	61C
Z_7356757_C	56C	60C
Z_7356758_G	56G	61G
Z_7356759_A	53A	61A
Z_7356760_C	51C	62C
Z_7356761_G	52G	62G
Z_7356762_G	51G	63G
Z_7356763_G	53G	65G
Z_7356764_T	54T	65T
Z_7356765_C	55C	65C
Z_7356766_G	55G	64G
Z_7356767_C	55C	64C
Z_7356768_T	57T	65T
Z_7356769_A	58A	64A
Z_7356770_A	58A	65A
Z_7356771_A	58A	69A
Z_7356772_C	57C	66C
Z_7356773_C	55C	66C
Z_7356774_C	55C	68C
Z_7356775_A	54A	67A
Z_7356776_C	56C	66C
Z_7356777_C	55C	65C
Z_7356778_C	55C	66C
Z_7356779_A	56A	66A

Z_7356780_A	55A	66A
Z_7356781_A	56A	66A
Z_7356782_T	55T	65T
Z_7356783_T	57T	65T
Z_7356784_G	56G	66G
Z_7356785_G	55G	67G
Z_7356786_C	53C	67C
Z_7356787_G	53G	69G
Z_7356788_T	54T	69T
Z_7356789_T	54T	66T
Z_7356790_G	51G	68G
Z_7356791_T	53T	68T
Z_7356792_T	53T	69T
Z_7356793_T	50T	69T
Z_7356794_T	49T	69T
Z_7356795_A	50A	68A
Z_7356796_T	51T	68T
Z_7356797_T	51T	66T
Z_7356798_T	51T	67T
Z_7356799_C	50C	65C
Z_7356800_T	52T	66T
Z_7356801_A	48A	66A
Z_7356802_T	48T	66T
Z_7356803_A	47A	66A
Z_7356804_C	44C	65C
Z_7356805_T	45T	64T
Z_7356806_G	46G	64G
Z_7356807_A	46A	64A
Z_7356808_T	46T	64T
Z_7356809_T	46T	63T
Z_7356810_T	45T	63T
Z_7356811_T	46T	64T
Z_7356812_C	45C	64C
Z_7356813_T	45T	63T
Z_7356814_T	44T	63T
Z_7356815_A	43A	62A
Z_7356816_T	45T	62T
Z_7356817_T	45T	63T
Z_7356818_T	43T	63T
Z_7356819_G	41G	63G
Z_7356820_A	41A	61A
Z_7356821_G	60T_38G	62G
Z_7356822_T	38T	62T

Z_7356823_T	38T	61T
Z_7356824_T	38T	63T
Z_7356825_C	38T	66C
Z_7356826_T	42T	66T
Z_7356827_T	43T	68T
Z_7356828_T	44T	68T
Z_7356829_T	44T	68T
Z_7356830_T	44T	67T
Z_7356831_C	39C	68C
Z_7356832_T	40T	68T
Z_7356833_T	41T	67T
Z_7356834_A	44A	66A
Z_7356835_A	44A	66A
Z_7356836_T	45T	63T
Z_7356837_C	42C	65C
Z_7356838_A	43A	65A
Z_7356839_T	45T	66T
Z_7356840_T	45T	65T
Z_7356841_C	44C	67C
Z_7356842_G	44G	68G
Z_7356843_G	43G	70G
Z_7356844_T	42T	70T
Z_7356845_C	43C	72C
Z_7356846_T	42T	73T
Z_7356847_C	45C	74C
Z_7356848_A	45A	74A
Z_7356849_T	45T	75T
Z_7356850_T	45T	72T
Z_7356851_T	44T	72T
Z_7356852_A	43A	72A
Z_7356853_T	44T	74T
Z_7356854_T	43T	74T
Z_7356855_T	44T	73T
Z_7356856_C	45C	73C
Z_7356857_T	46T	72T
Z_7356858_A	46A	70A
Z_7356859_A	46A	69A
Z_7356860_A	46A	69A
Z_7356861_C	46C	69C
Z_7356862_G	45G	67G
Z_7356863_G	44G	65G
Z_7356864_A	45A	67A
Z_7356865_C	45C	67C

Z_7356866_T	45T	63T
Z_7356867_G	43A	63G
Z_7356868_A	42A	63A
Z_7356869_G	44G	62G
Z_7356870_C	45C	62C
Z_7356871_G	45G	63G
Z_7356872_A	44A	64A
Z_7356873_T	39G	65T
Z_7356874_C	39C	66C
Z_7356875_T	39T	67T
Z_7356876_C	39C	68C
Z_7356877_A	39T	39A_30T
Z_7356878_A	40A	70A
Z_7356879_A	39A	70A
Z_7356880_A	40A	72A
Z_7356881_T	40T	72T
Z_7356882_A	40T	71A
Z_7356883_A	40A	72A
Z_7356884_T	39T	73T
Z_7356885_C	40A	73C
Z_7356886_T	49T	72T
Z_7356887_G	50G	71G
Z_7356888_A	51A	71A
Z_7356889_C	50C	72C
Z_7356890_C	50C	72C
Z_7356891_G	50G	30A_41G
Z_7356892_G	52G	71G
Z_7356893_G	51G	70G
Z_7356894_A	52A	71A
Z_7356895_C	51C	71C
Z_7356896_A	52A	70A
Z_7356897_G	51G	72G
Z_7356898_T	49T	73T
Z_7356899_G	51G	75G
Z_7356900_G	50G	75G
Z_7356901_C	51C	75C
Z_7356902_T	51T	76T
Z_7356903_T	50T	78T
Z_7356904_A	47A	81A
Z_7356905_A	46A	80A
Z_7356906_T	45T	79T
Z_7356907_T	44T	79T
Z_7356908_T	45T	79T

Z_7356909_A	42A	77A
Z_7356910_C	42C	80C
Z_7356911_G	43G	37A_43G
Z_7356912_C	43C	79C
Z_7356913_C	42C	79C
Z_7356914_G	45G	79G
Z_7356915_G	46G	79G
Z_7356916_A	46A	79A
Z_7356917_A	44A	77A
Z_7356918_G	43G	78G
Z_7356919_G	42G	76G
Z_7356920_A	42A	74A
Z_7356921_A	42A	73A
Z_7356922_T	128T	72T
Z_7356923_T	12T	75T
Z_7356924_T	11T	76T
Z_7356925_T	7A_4T	78T
Z_7356926_T	31T	77T
Z_7356927_T	33T	76T
Z_7356928_T	34T	37A_39T
Z_7356929_A	34A	75A
Z_7356930_A	34A	74A
Z_7356931_A	34A	74A
Z_7356932_A	34A	72A
Z_7356933_A	34A	70A
Z_7356934_A	35A	69A
Z_7356935_G	35G	71G
Z_7356936_A	38A	73A
Z_7356937_A	92A_27G	74A
Z_7356938_A	11A	72A
Z_7356939_A	9A	73A
Z_7356940_G	6A_4G	72G
Z_7356941_A	38A	74A
Z_7356942_A	38A	73A
Z_7356943_A	36A	74A
Z_7356944_A	37A	74A
Z_7356945_A	37A	74A
Z_7356946_A	38A	74A
Z_7356947_G	39G	74G
Z_7356948_A	39A	76A
Z_7356949_A	39A	78A
Z_7356950_C	37C	78C
Z_7356951_A	38A	77A

Z_7356952_C	38C	77C
Z_7356953_G	38G	78G
Z_7356954_C	38C	78C
Z_7356955_A	38A	79A
Z_7356956_A	38A	79A
Z_7356957_T	39T	81T
Z_7356958_C	40C	81C
Z_7356959_C	42C	80C
Z_7356960_A	42A	80A
Z_7356961_G	42G	80G
Z_7356962_A	42A	80A
Z_7356963_T	41T	80T
Z_7356964_T	39T	79T
Z_7356965_C	39C	79C
Z_7356966_G	38G	79G
Z_7356967_G	38G	79G
Z_7356968_G	36A	77G
Z_7356969_A	38A	75A
Z_7356970_C	39C	74C
Z_7356971_T	40T	72T
Z_7356972_C	40T	34T_38C
Z_7356973_T	40T	71T
Z_7356974_G	40G	70G
Z_7356975_C	39C	68C
Z_7356976_A	40A	70A
Z_7356977_T	40A	33A_37T
Z_7356978_A	42A	70A
Z_7356979_C	42C	69C
Z_7356980_T	43T	70T
Z_7356981_G	43G	70G
Z_7356982_A	44A	70A
Z_7356983_A	44A	68A
Z_7356984_G	43G	69G
Z_7356985_G	44G	69G
Z_7356986_A	44A	69A
Z_7356987_T	43T	69T
Z_7356988_A	44A	70A
Z_7356989_A	43A	71A
Z_7356990_C	43C	69C
Z_7356991_G	43T	37T_32G
Z_7356992_G	42G	68G
Z_7356993_C	40C	68C
Z_7356994_C	40C	67C

Z_7356995_A	43A	68A
Z_7356996_A	43A	67A
Z_7356997_T	43T	66T
Z_7356998_C	43C	65C
Z_7356999_G	43G	63G
Z_7357000_A	43A	63A
Z_7357001_A	41A	63A
Z_7357002_G	40G	64G
Z_7357003_T	39T	65T
Z_7357004_G	41G	66G
Z_7357005_A	38A	63A
Z_7357006_C	38C	64C
Z_7357007_T	38T	64T
Z_7357008_C	35C	63C
Z_7357009_C	37C	63C
Z_7357010_G	37G	63G
Z_7357011_A	38A	62A
Z_7357012_A	38A	62A
Z_7357013_T	38T	62T
Z_7357014_T	37T	63T
Z_7357015_G	37G	64G
Z_7357016_T	36C	32T_32C
Z_7357017_G	35G	63G
Z_7357018_G	35G	60G
Z_7357019_T	36T	57T
Z_7357020_T	38T	57T
Z_7357021_A	37A	58A
Z_7357022_G	38G	59G
Z_7357023_C	38C	59C
Z_7357024_T	37T	60T
Z_7357025_G	37G	60G
Z_7357026_C	36C	61C
Z_7357027_A	38A	62A
Z_7357028_C	35C	61C
Z_7357029_T	35T	61T
Z_7357030_T	35T	59T
Z_7357031_T	34T	57T
Z_7357032_G	33G	57G
Z_7357033_G	36G	56G
Z_7357034_A	36A	55A
Z_7357035_T	36T	55T
Z_7357036_C	35C	52C
Z_7357037_C	38C	52C

Z_7357038_T	38T	53T
Z_7357039_T	37T	53T
Z_7357040_T	36T	54T
Z_7357041_G	35G	55G
Z_7357042_A	34A	54A
Z_7357043_A	34A	50A
Z_7357044_A	34A	48A
Z_7357045_G	34G	48G
Z_7357046_G	32G	49G
Z_7357047_A	34A	49A
Z_7357048_A	32A	47A
Z_7357049_C	34C	48C
Z_7357050_C	32C	47C
Z_7357051_T	32T	48T
Z_7357052_C	31C	47C
Z_7357053_G	32G	28G_19C
Z_7357054_G	33G	48G
Z_7357055_G	34G	47G
Z_7357056_A	34A	47A
Z_7357057_A	34A	48A
Z_7357058_A	35A	48A
Z_7357059_G	36G	49G
Z_7357060_C	34C	51C
Z_7357061_G	36G	50G
Z_7357062_A	35A	49A
Z_7357063_C	37C	49C
Z_7357064_C	37C	51C
Z_7357065_T	35T	50T
Z_7357066_A	36A	51A
Z_7357067_T	37T	51T
Z_7357068_A	38A	53A
Z_7357069_T	38T	54T
Z_7357070_T	37T	52T
Z_7357071_A	36A	51A
Z_7357072_C	37C	50C
Z_7357073_C	38C	51C
Z_7357074_A	36A	51A
Z_7357075_T	37G	28T_22G
Z_7357076_C	37C	53C
Z_7357077_G	37G	52G
Z_7357078_G	37G	52G
Z_7357079_C	37C	51C
Z_7357080_A	37A	53A

Z_7357081_A	37A	53A
Z_7357082_G	38G	53G
Z_7357083_G	37G	54G
Z_7357084_T	38T	52T
Z_7357085_T	38T	52T
Z_7357086_T	37T	54T
Z_7357087_A	37A	53A
Z_7357088_T	38T	53T
Z_7357089_A	37A	53A
Z_7357090_T	34T	52T
Z_7357091_T	34T	52T
Z_7357092_C	35C	53C
Z_7357093_T	36T	54T
Z_7357094_C	36C	53C
Z_7357095_A	37A	54A
Z_7357096_C	38C	54C
Z_7357097_G	35G	53G
Z_7357098_G	34G	52G
Z_7357099_C	34T	25T_27C
Z_7357100_C	36C	54C
Z_7357101_T	39T	53T
Z_7357102_T	38T	56T
Z_7357103_T	37T	57T
Z_7357104_C	37C	58C
Z_7357105_T	38T	58T
Z_7357106_C	40C	60C
Z_7357107_T	42T	63T
Z_7357108_A	43A	63A
Z_7357109_G	43G	62G
Z_7357110_A	42A	61A
Z_7357111_A	42A	62A
Z_7357112_C	42C	64C
Z_7357113_T	44T	67T
Z_7357114_A	44A	68A
Z_7357115_C	46C	67C
Z_7357116_G	47G	66G
Z_7357117_G	45G	66G
Z_7357118_A	46A	66A
Z_7357119_T	44T	65T
Z_7357120_C	44C	66C
Z_7357121_T	43T	67T
Z_7357122_C	42C	66C
Z_7357123_C	42C	67C

Z_7357124_A	40A	66A
Z_7357125_C	40C	67C
Z_7357126_G	41G	67G
Z_7357127_A	42A	65A
Z_7357128_A	41A	65A
Z_7357129_A	40A	65A
Z_7357130_A	40A	66A
Z_7357131_A	39A	66A
Z_7357132_C	40C	67C
Z_7357133_C	38C	66C
Z_7357134_G	38G	65G
Z_7357135_G	37G	31A_33G
Z_7357136_A	37A	63A
Z_7357137_T	41T	63T
Z_7357138_C	39C	31T_32C
Z_7357139_G	41G	66G
Z_7357140_A	41A	65A
Z_7357141_C	42C	64C
Z_7357142_G	42G	65G
Z_7357143_C	40C	66C
Z_7357144_C	38C	66C
Z_7357145_G	41G	66G
Z_7357146_G	42G	64G
Z_7357147_A	42A	63A
Z_7357148_T	43T	64T
Z_7357149_T	46T	64T
Z_7357150_G	45G	64G
Z_7357151_C	45C	65C
Z_7357152_A	46A	67A
Z_7357153_G	46G	68G
Z_7357154_A	47A	68A
Z_7357155_T	48T	66T
Z_7357156_G	48G	66G
Z_7357157_T	48T	66T
Z_7357158_A	46A	68A
Z_7357159_A	47A	66A
Z_7357160_C	48C	66C
Z_7357161_C	47C	65C
Z_7357162_C	49C	64C
Z_7357163_T	49T	63T
Z_7357164_G	49G	64G
Z_7357165_G	48G	66G
Z_7357166_C	47C	68C

Z_7357167_G	48G	69G
Z_7357168_C	47C	68C
Z_7357169_C	48C	68C
Z_7357170_C	46C	70C
Z_7357171_A	45A	68A
Z_7357172_G	45G	68G
Z_7357173_A	45A	68A
Z_7357174_T	46T	67T
Z_7357175_T	46T	66T
Z_7357176_C	44C	67C
Z_7357177_G	43G	66G
Z_7357178_C	43C	68C
Z_7357179_T	46T	68T
Z_7357180_G	47G	69G
Z_7357181_G	49G	67G
Z_7357182_G	48G	66G
Z_7357183_A	48A	67A
Z_7357184_A	48A	65A
Z_7357185_C	46C	66C
Z_7357186_C	46C	70C
Z_7357187_G	46A	34A_35G
Z_7357188_A	45A	68A
Z_7357189_A	46A	68A
Z_7357190_G	45G	68G
Z_7357191_C	46C	69C
Z_7357192_G	46G	69G
Z_7357193_C	47C	69C
Z_7357194_A	46A	68A
Z_7357195_G	47G	66G
Z_7357196_A	46A	68A
Z_7357197_A	45A	65A
Z_7357198_A	45A	65A
Z_7357199_T	46T	65T
Z_7357200_T	46T	65T
Z_7357201_T	46T	63T
Z_7357202_A	47A	60A
Z_7357203_G	46G	60G
Z_7357204_A	47A	61A
Z_7357205_G	47G	60G
Z_7357206_A	48A	59A
Z_7357207_A	42A	58A
Z_7357208_A	42A	55A
Z_7357209_T	41T	53T

Z_7357210_T	39T	53T
Z_7357211_T	42T	54T
Z_7357212_A	44A	53A
Z_7357213_A	45A	52A
Z_7357214_A	45A	52A
Z_7357215_A	42A	52A
Z_7357216_G	42G	52G
Z_7357217_A	42A	52A
Z_7357218_A	41A	52A
Z_7357219_G	39G	53G
Z_7357220_G	39G	53G
Z_7357221_C	38C	53C
Z_7357222_G	39G	54G
Z_7357223_A	38A	54A
Z_7357224_A	37A	55A
Z_7357225_G	37G	54G
Z_7357226_T	39T	56T
Z_7357227_C	40C	57C
Z_7357228_A	40A	56A
Z_7357229_A	38A	56A
Z_7357230_G	36G	56G
Z_7357231_A	36A	57A
Z_7357232_G	36G	57G
Z_7357233_T	35T	57T
Z_7357234_T	35T	56T
Z_7357235_A	35A	56A
Z_7357236_C	35C	55C
Z_7357237_T	34T	56T
Z_7357238_C	34C	55C
Z_7357239_C	35C	55C
Z_7357240_G	36G	55G
Z_7357241_G	35G	56G
Z_7357242_G	35G	55G
Z_7357243_A	35A	56A
Z_7357244_C	36C	56C
Z_7357245_T	38T	57T
Z_7357246_A	40A	57A
Z_7357247_A	40A	54A
Z_7357248_A	42A	53A
Z_7357249_T	41T	52T
Z_7357250_A	42A	52A
Z_7357251_A	40A	53A
Z_7357252_T	40T	52T

Z_7357253_A	41A	51A
Z_7357254_A	42A	51A
Z_7357255_C	43C	54C
Z_7357256_C	46C	54C
Z_7357257_T	47T	53T
Z_7357258_T	47T	54T
Z_7357259_C	47C	52C
Z_7357260_T	47T	51T
Z_7357261_G	46G	50G
Z_7357262_T	46T	48T
Z_7357263_C	45C	49C
Z_7357264_T	46T	51T
Z_7357265_C	45C	50C
Z_7357266_A	46A	51A
Z_7357267_A	48A	52A
Z_7357268_T	48T	54T
Z_7357269_T	47T	54T
Z_7357270_C	47C	55C
Z_7357271_G	47G	54G
Z_7357272_A	47A	54A
Z_7357273_G	48G	53G
Z_7357274_A	48A	53A
Z_7357275_G	47G	53G
Z_7357276_G	46G	53G
Z_7357277_G	46G	52G
Z_7357278_C	46C	52C
Z_7357279_T	47T	53T
Z_7357280_C	47C	53C
Z_7357281_A	46A	50A
Z_7357282_A	45A	50A
Z_7357283_T	44T	49T
Z_7357284_T	45T	50T
Z_7357285_C	47C	50C
Z_7357286_G	45G	49G
Z_7357287_A	47A	49A
Z_7357288_G	46G	49G
Z_7357289_A	50A	49A
Z_7357290_G	49G	50G
Z_7357291_T	51T	48T
Z_7357292_C	50C	48C
Z_7357293_G	49G	48G
Z_7357294_A	49A	47A
Z_7357295_A	50A	47A

Z_7357296_A	47A	49A
Z_7357297_A	48A	50A
Z_7357298_A	49A	50A
Z_7357299_C	50C	51C
Z_7357300_C	49C	51C
Z_7357301_G	49G	51G
Z_7357302_G	47G	52G
Z_7357303_C	48C	52C
Z_7357304_T	48T	52T
Z_7357305_T	48T	52T
Z_7357306_C	49C	51C
Z_7357307_G	49G	51G
Z_7357308_A	48A	51A
Z_7357309_A	49A	51A
Z_7357310_A	50A	51A
Z_7357311_C	52C	50C
Z_7357312_G	54G	53G
Z_7357313_C	55C	52C
Z_7357314_A	54A	53A
Z_7357315_C	52C	54C
Z_7357316_G	51G	54G
Z_7357317_G	48G	54G
Z_7357318_A	48A	55A
Z_7357319_A	47A	55A
Z_7357320_A	45A	55A
Z_7357321_G	46G	52G
Z_7357322_T	46T	50T
Z_7357323_C	45C	50C
Z_7357324_C	45C	49C
Z_7357325_C	43C	49C
Z_7357326_C	41C	48C
Z_7357327_G	40G	48G
Z_7357328_C	41C	49C
Z_7357329_G	41G	50G
Z_7357330_G	41G	51G
Z_7357331_G	41G	52G
Z_7357332_C	41C	51C
Z_7357333_T	40T	53T
Z_7357334_C	41C	53C
Z_7357335_T	41T	54T
Z_7357336_A	41A	53A
Z_7357337_T	39T	54T
Z_7357338_T	39C	55T

Z_7357339_A	39A	54A
Z_7357340_G	39G	54G
Z_7357341_T	39T	55T
Z_7357342_A	38A	55A
Z_7357343_T	38T	55T
Z_7357344_T	38T	55T
Z_7357345_A	38A	56A
Z_7357346_C	38C	56C
Z_7357347_C	38C	57C
Z_7357348_G	39G	57G
Z_7357349_C	39C	57C
Z_7357350_T	40T	54T
Z_7357351_G	40G	54G
Z_7357352_C	40C	56C
Z_7357353_T	41T	57T
Z_7357354_G	40G	57G
Z_7357355_C	38C	60C
Z_7357356_G	37G	60G
Z_7357357_G	37G	61G
Z_7357358_T	37T	60T
Z_7357359_T	34T	58T
Z_7357360_A	33A	58A
Z_7357361_A	33A	58A
Z_7357362_T	33T	57T
Z_7357363_A	33A	56A
Z_7357364_T	33T	58T
Z_7357365_T	33T	58T
Z_7357366_A	33A	58A
Z_7357367_A	33A	57A
Z_7357368_T	35T	58T
Z_7357369_A	35A	58A
Z_7357370_G	36G	58G
Z_7357371_C	37C	58C
Z_7357372_A	39A	58A
Z_7357373_T	39T	58T
Z_7357374_T	39T	57T
Z_7357375_G	40G	57G
Z_7357376_T	41T	57T
Z_7357377_A	41A	57A
Z_7357378_T	41T	60T
Z_7357379_T	42T	59T
Z_7357380_A	41A	59A
Z_7357381_A	40A	62A

Z_7357382_T	39T	62T
Z_7357383_A	37A	64A
Z_7357384_A	37A	64A
Z_7357385_T	37T	63T
Z_7357386_A	37A	63A
Z_7357387_A	37A	64A
Z_7357388_T	36T	65T
Z_7357389_G	34G	65G
Z_7357390_A	37A	66A
Z_7357391_C	35C	64C
Z_7357392_G	35G	63G
Z_7357393_A	35A	64A
Z_7357394_T	33T	62T
Z_7357395_G	32G	64G
Z_7357396_A	32A	64A
Z_7357397_T	34T	65T
Z_7357398_A	33A	67A
Z_7357399_A	34A	67A
Z_7357400_T	34T	67T
Z_7357401_A	35A	67A
Z_7357402_G	36G	69G
Z_7357403_C	36C	69C
Z_7357404_A	37A	69A
Z_7357405_G	37G	70G
Z_7357406_T	38T	70T
Z_7357407_A	39A	68A
Z_7357408_G	38G	69G
Z_7357409_C	39C	67C
Z_7357410_A	41A	66A
Z_7357411_G	40G	66G
Z_7357412_C	38C	65C
Z_7357413_G	39G	64G
Z_7357414_G	39G	64G
Z_7357415_C	39C	65C
Z_7357416_A	40A	65A
Z_7357417_A	41A	66A
Z_7357418_C	38C	69C
Z_7357419_A	40A	69A
Z_7357420_G	38G	70G
Z_7357421_T	38T	70T
Z_7357422_A	37A	70A
Z_7357423_A	38A	69A
Z_7357424_C	39C	68C

Z_7357425_A	39A	69A
Z_7357426_G	39G	67G
Z_7357427_T	39T	68T
Z_7357428_A	39A	65A
Z_7357429_A	40A	67A
Z_7357430_T	40T	66T
Z_7357431_C	39C	66C
Z_7357432_G	39G	65G
Z_7357433_C	39C	64C
Z_7357434_A	40A	64A
Z_7357435_A	40A	63A
Z_7357436_C	40C	61C
Z_7357437_A	39A	61A
Z_7357438_G	36G	61G
Z_7357439_C	35C	63C
Z_7357440_A	34A	62A
Z_7357441_A	33A	61A
Z_7357442_T	31T	60T
Z_7357443_A	33A	61A
Z_7357444_G	34G	62G
Z_7357445_C	32A	62C
Z_7357446_G	30G	62G
Z_7357447_G	32G	60G
Z_7357448_C	32C	59C
Z_7357449_A	31A	59A
Z_7357450_A	30A	54A
Z_7357451_G	32G	54G
Z_7357452_A	31A	53A
Z_7357453_A	31A	53A
Z_7357454_C	33C	54C
Z_7357455_G	34G	57G
Z_7357456_A	33A	57A
Z_7357457_A	35A	58A
Z_7357458_A	35A	57A
Z_7357459_A	36A	56A
Z_7357460_C	34C	58C
Z_7357461_G	33G	58G
Z_7357462_A	34A	60A
Z_7357463_T	35T	58T
Z_7357464_T	36T	57T
Z_7357465_T	36T	57T
Z_7357466_A	36A	57A
Z_7357467_A	35A	58A

Z_7357468_G	34G	59G
Z_7357469_C	33C	60C
Z_7357470_G	33G	59G
Z_7357471_T	36T	59T
Z_7357472_T	35T	60T
Z_7357473_A	34A	60A
Z_7357474_A	34A	59A
Z_7357475_G	33G	61G
Z_7357476_T	33T	59T
Z_7357477_G	33G	59G
Z_7357478_G	33G	58G
Z_7357479_C	34C	58C
Z_7357480_C	31C	59C
Z_7357481_A	32A	59A
Z_7357482_C	32C	61C
Z_7357483_A	32A	60A
Z_7357484_A	33A	61A
Z_7357485_T	33T	59T
Z_7357486_T	32T	60T
Z_7357487_G	33G	61G
Z_7357488_C	33T	59C
Z_7357489_C	32C	60C
Z_7357490_A	32A	59A
Z_7357491_T	33T	61T
Z_7357492_C	33C	59C
Z_7357493_A	31A	57A
Z_7357494_T	31T	56T
Z_7357495_T	31T	57T
Z_7357496_T	32T	57T
Z_7357497_C	33C	56C
Z_7357498_T	32T	56T
Z_7357499_G	30G	56G
Z_7357500_T	31T	56T
Z_7357501_T	31T	56T
Z_7357502_T	32T	56T
Z_7357503_A	32A	55A
Z_7357504_A	33A	55A
Z_7357505_T	34T	57T
Z_7357506_T	35T	58T
Z_7357507_A	36A	58A
Z_7357508_G	36G	60G
Z_7357509_A	37A	59A
Z_7357510_A	38A	59A

Z_7357511_C	39C	60C
Z_7357512_T	40T	32T_28C
Z_7357513_C	38C	58C
Z_7357514_T	37T	59T
Z_7357515_T	37T	58T
Z_7357516_C	37C	59C
Z_7357517_C	35C	60C
Z_7357518_C	36C	60C
Z_7357519_T	37T	60T
Z_7357520_C	37C	60C
Z_7357521_C	39C	60C
Z_7357522_T	38T	60T
Z_7357523_G	38G	60G
Z_7357524_T	38T	57T
Z_7357525_A	36A	58A
Z_7357526_T	37T	58T
Z_7357527_C	34C	59C
Z_7357528_G	37G	59G
Z_7357529_T	36T	59T
Z_7357530_C	36C	59C
Z_7357531_T	37T	62T
Z_7357532_T	40T	63T
Z_7357533_A	40A	67A
Z_7357534_A	39A	67A
Z_7357535_A	41A	68A
Z_7357536_C	40C	69C
Z_7357537_C	42C	69C
Z_7357538_A	43A	72A
Z_7357539_A	43A	71A
Z_7357540_A	44A	74A
Z_7357541_G	41G	74G
Z_7357542_A	42A	75A
Z_7357543_G	41G	74G
Z_7357544_A	43A	76A
Z_7357545_G	43G	77G
Z_7357546_A	45A	77A
Z_7357547_G	44G	79G
Z_7357548_G	43G	80G
Z_7357549_G	42G	79G
Z_7357550_G	45G	76G
Z_7357551_A	45A	76A
Z_7357552_G	46G	76G
Z_7357553_G	45G	78G

Z_7357554_A	44A	77A
Z_7357555_A	45A	77A
Z_7357556_T	45T	77T
Z_7357557_C	43C	75C
Z_7357558_G	43G	75G
Z_7357559_C	44C	75C
Z_7357560_A	44A	75A
Z_7357561_T	46T	75T
Z_7357562_T	48T	73T
Z_7357563_T	47T	75T
Z_7357564_A	47A	74A
Z_7357565_T	47T	76T
Z_7357566_T	46T	78T
Z_7357567_T	47T	77T
Z_7357568_T	48T	76T
Z_7357569_A	49A	75A
Z_7357570_A	50A	74A
Z_7357571_A	50A	75A
Z_7357572_C	50C	75C
Z_7357573_G	52G	75G
Z_7357574_C	51C	77C
Z_7357575_G	49G	77G
Z_7357576_C	49C	78C
Z_7357577_G	47G	77G
Z_7357578_C	48C	78C
Z_7357579_C	46C	78C
Z_7357580_C	47C	79C
Z_7357581_G	46G	79G
Z_7357582_G	46G	78G
Z_7357583_C	47C	78C
Z_7357584_G	48G	78G
Z_7357585_T	48T	78T
Z_7357586_C	48C	77C
Z_7357587_A	48A	76A
Z_7357588_C	48C	76C
Z_7357589_C	45C	77C
Z_7357590_C	45C	79C
Z_7357591_A	46A	80A
Z_7357592_C	44C	80C
Z_7357593_C	47C	80C
Z_7357594_A	46A	80A
Z_7357595_T	44T	79T
Z_7357596_C	45C	82C

Z_7357597_A	44A	81A
Z_7357598_T	43T	83T
Z_7357599_A	43A	85A
Z_7357600_A	44A	86A
Z_7357601_A	43A	85A
Z_7357602_T	42T	88T
Z_7357603_A	43A	86A
Z_7357604_A	42A	86A
Z_7357605_T	42T	85T
Z_7357606_A	44A	85A
Z_7357607_A	44A	85A
Z_7357608_T	46T	86T
Z_7357609_T	46T	86T
Z_7357610_C	46C	85C
Z_7357611_G	46G	86G
Z_7357612_A	45A	86A
Z_7357613_T	47T	88T
Z_7357614_T	46T	88T
Z_7357615_T	46T	88T
Z_7357616_T	44T	90T
Z_7357617_A	44A	90A
Z_7357618_T	44T	90T
Z_7357619_T	44T	90T
Z_7357620_T	41T	91T
Z_7357621_G	42G	94G
Z_7357622_A	40A	94A
Z_7357623_A	41A	95A
Z_7357624_T	42T	96T
Z_7357625_T	42T	95T
Z_7357626_T	44T	93T
Z_7357627_C	45C	93C
Z_7357628_T	45T	90T
Z_7357629_C	44C	91C
Z_7357630_C	45C	89C
Z_7357631_C	44C	88C
Z_7357632_C	44C	89C
Z_7357633_G	45G	85G
Z_7357634_G	46G	87G
Z_7357635_G	45G	84G
Z_7357636_G	45G	84G
Z_7357637_G	43G	83G
Z_7357638_A	42A	83A
Z_7357639_G	42G	82G

Z_7357640_G	41G	81G
Z_7357641_G	40G	81G
Z_7357642_T	40T	80T
Z_7357643_T	40T	80T
Z_7357644_T	39T	81T
Z_7357645_G	38G	83G
Z_7357646_C	40C	82C
Z_7357647_T	40T	81T
Z_7357648_A	41A	79A
Z_7357649_C	41C	79C
Z_7357650_A	41A	77A
Z_7357651_T	42T	80T
Z_7357652_G	41G	81G
Z_7357653_G	42G	81G
Z_7357654_A	40A	81A
Z_7357655_T	43T	82T
Z_7357656_T	43T	82T
Z_7357657_A	43A	82A
Z_7357658_T	44T	81T
Z_7357659_G	45G	82G
Z_7357660_A	44A	82A
Z_7357661_T	44T	81T
Z_7357662_A	44A	83A
Z_7357663_A	43A	82A
Z_7357664_T	43T	82T
Z_7357665_G	43G	82G
Z_7357666_C	41C	81C
Z_7357667_C	41C	82C
Z_7357668_G	42G	81G
Z_7357669_C	44C	79C
Z_7357670_C	43C	80C
Z_7357671_A	44A	79A
Z_7357672_G	43G	81G
Z_7357673_C	42C	81C
Z_7357674_A	42A	79A
Z_7357675_G	41G	80G
Z_7357676_C	40A	79C
Z_7357677_G	39G	80G
Z_7357678_G	36G	82G
Z_7357679_C	35C	82C
Z_7357680_G	35G	82G
Z_7357681_A	36A	83A
Z_7357682_T	36T	82T

Z_7357683_T	34T	84T
Z_7357684_C	34C	83C
Z_7357685_T	34T	82T
Z_7357686_T	34T	82T
Z_7357687_T	34T	85T
Z_7357688_C	33C	86C
Z_7357689_T	35T	86T
Z_7357690_C	35C	86C
Z_7357691_C	34C	82C
Z_7357692_G	34G	83G
Z_7357693_G	34G	82G
Z_7357694_C	33C	79C
Z_7357695_A	33A	77A
Z_7357696_G	31G	75G
Z_7357697_C	32C	75C
Z_7357698_G	32G	75G
Z_7357699_G	33G	75G
Z_7357700_C	32C	75C
Z_7357701_G	29G	75G
Z_7357702_A	29A	75A
Z_7357703_A	33A	75A
Z_7357704_G	32G	74G
Z_7357705_G	31G	74G
Z_7357706_A	33A	77A
Z_7357707_G	34G	76G
Z_7357708_G	34G	74G
Z_7357709_C	34C	73C
Z_7357710_T	34T	71T
Z_7357711_G	33G	72G
Z_7357712_A	34A	72A
Z_7357713_G	34G	73G
Z_7357714_T	35T	74T
Z_7357715_T	35T	73T
Z_7357716_T	32T	71T
Z_7357717_T	35T	70T
Z_7357718_C	34C	69C
Z_7357719_C	33C	70C
Z_7357720_C	33C	70C
Z_7357721_G	33G	71G
Z_7357722_T	32T	70T
Z_7357723_C	32C	70C
Z_7357724_G	31G	67G
Z_7357725_G	29G	68G

Z_7357726_G	31G	67G
Z_7357727_T	30T	67T
Z_7357728_T	31T	67T
Z_7357729_C	31C	66C
Z_7357730_G	31G	67G
Z_7357731_G	32G	67G
Z_7357732_T	32T	67T
Z_7357733_G	32G	66G
Z_7357734_C	31C	67C
Z_7357735_A	32A	67A
Z_7357736_G	32G	66G
Z_7357737_G	30G	66G
Z_7357738_A	30A	66A
Z_7357739_T	29T	64T
Z_7357740_G	31G	64G
Z_7357741_G	31G	65G
Z_7357742_G	31G	66G
Z_7357743_T	29T	67T
Z_7357744_G	30G	67G
Z_7357745_C	31C	67C
Z_7357746_C	30C	65C
Z_7357747_T	30T	64T
Z_7357748_T	30T	65T
Z_7357749_T	29T	65T
Z_7357750_A	30A	64A
Z_7357751_A	29A	65A
Z_7357752_G	30G	66G
Z_7357753_C	30C	66C
Z_7357754_C	30C	67C
Z_7357755_T	30T	66T
Z_7357756_T	30T	65T
Z_7357757_T	30T	65T
Z_7357758_T	27C	63T
Z_7357759_C	30C	64C
Z_7357760_C	29C	65C
Z_7357761_C	30C	65C
Z_7357762_G	29G	65G
Z_7357763_G	29G	66G
Z_7357764_G	29G	67G
Z_7357765_G	21A_27G	64G
Z_7357766_G	26A	67G
Z_7357767_A	31A	65A
Z_7357768_G	31G	65G

Z_7357769_A	31A	67A
Z_7357770_G	33G	67G
Z_7357771_G	33G	67G
Z_7357772_A	33A	66A
Z_7357773_A	34A	66A
Z_7357774_T	33T	66T
Z_7357775_T	34T	65T
Z_7357776_C	33C	64C
Z_7357777_C	34C	64C
Z_7357778_C	32C	64C
Z_7357779_C	34C	65C
Z_7357780_C	35C	65C
Z_7357781_A	34A	64A
Z_7357782_G	32G	60G
Z_7357783_C	31C	61C
Z_7357784_C	32T	62C
Z_7357785_T	31T	62T
Z_7357786_G	31G	62G
Z_7357787_C	30C	62C
Z_7357788_G	29G	62G
Z_7357789_G	30G	62G
Z_7357790_A	29A	62A
Z_7357791_C	29C	64C
Z_7357792_C	29C	65C
Z_7357793_T	29T	66T
Z_7357794_A	29A	67A
Z_7357795_T	28T	68T
Z_7357796_T	27T	71T
Z_7357797_T	28T	71T
Z_7357798_C	26C	71C
Z_7357799_T	26T	71T
Z_7357800_T	23T	71T
Z_7357801_G	23G	68G
Z_7357802_G	23G	69G
Z_7357803_C	22C	73C
Z_7357804_G	23G	74G
Z_7357805_A	23A	75A
Z_7357806_T	23T	72T
Z_7357807_G	23G	72G
Z_7357808_G	23G	72G
Z_7357809_C	21C	71C
Z_7357810_C	null	71C
Z_7357811_T	null	71T

Z_7357812_G	null	71G
Z_7357813_G	null	71G
Z_7357814_C	null	69C
Z_7357815_G	null	71G
Z_7357816_G	null	71G
Z_7357817_C	null	70C
Z_7357818_T	null	70T
Z_7357819_T	null	69T
Z_7357820_A	null	70A
Z_7357821_G	null	71G
Z_7357822_G	null	73G
Z_7357823_T	null	75T
Z_7357824_G	null	75G
Z_7357825_T	null	74T
Z_7357826_T	null	76T
Z_7357827_T	null	75T
Z_7357828_C	null	76C
Z_7357829_C	6C	75C
Z_7357830_A	7A	75A
Z_7357831_T	8T	76T
Z_7357832_C	8C	76C
Z_7357833_T	8T	75T
Z_7357834_G	8G	75G
Z_7357835_T	8T	75T
Z_7357836_G	8G	75G
Z_7357837_T	8T	71T
Z_7357838_C	8C	73C
Z_7357839_T	16T_8C	73T
Z_7357840_C	9C	75C
Z_7357841_C	9C	74C
Z_7357842_C	9C	72C
Z_7357843_C	9C	75C
Z_7357844_C	11C	76C
Z_7357845_C	12C	76C
Z_7357846_G	11C	74G
Z_7357847_C	13C	75C
Z_7357848_A	15A	74A
Z_7357849_A	16A	73A
Z_7357850_C	16C	71C
Z_7357851_A	16A	72A
Z_7357852_A	16A	71A
Z_7357853_T	16T	70T
Z_7357854_A	16A	72A

Z_7357855_T	16T	71T
Z_7357856_T	16T	70T
Z_7357857_A	16A	70A
Z_7357858_A	16A	69A
Z_7357859_C	16C	70C
Z_7357860_A	16A	70A
Z_7357861_G	16G	67G
Z_7357862_G	17G	66G
Z_7357863_G	17G	66G
Z_7357864_G	17A	65G
Z_7357865_T	18T	63T
Z_7357866_C	18C	66C
Z_7357867_T	20T	66T
Z_7357868_G	21G	65G
Z_7357869_G	21G	65G
Z_7357870_G	23G	65G
Z_7357871_A	22A	62A
Z_7357872_T	23T	64T
Z_7357873_C	25C	67C
Z_7357874_A	25A	66A
Z_7357875_G	25G	65G
Z_7357876_C	25C	65C
Z_7357877_T	25T	65T
Z_7357878_G	27G	33T_33G
Z_7357879_G	26G	65G
Z_7357880_G	27G	64G
Z_7357881_A	27A	65A
Z_7357882_T	26T	66T
Z_7357883_C	28C	65C
Z_7357884_C	28C	65C
Z_7357885_T	27T	66T
Z_7357886_G	27G	65G
Z_7357887_T	27T	65T
Z_7357888_G	28G	65G
Z_7357889_A	26A	65A
Z_7357890_T	26T	63T
Z_7357891_C	26C	60C
Z_7357892_C	25C	59C
Z_7357893_G	24G	57G
Z_7357894_C	25C	60C
Z_7357895_T	25T	60T
Z_7357896_G	25G	59G
Z_7357897_C	25C	59C

Z_7357898_C	23T	55C
Z_7357899_T	25T	54T
Z_7357900_G	25G	54G
Z_7357901_G	25G	54G
Z_7357902_T	25T	54T
Z_7357903_G	31G	53G
Z_7357904_G	32G	54G
Z_7357905_G	32G	56G
Z_7357906_G	31G	56G
Z_7357907_G	15T_61G_15C	55G
Z_7357908_T	26T	54T
Z_7357909_C	26C	57C
Z_7357910_T	28T	56T
Z_7357911_C	28C	54C
Z_7357912_T	28T	55T
Z_7357913_C	27C	55C
Z_7357914_T	53T_22G_7C	55T
Z_7357915_C	27C	55C
Z_7357916_C	25C	55C
Z_7357917_C	25C	54C
Z_7357918_C	25C	52C
Z_7357919_C	25C	53C
Z_7357920_C	25C	52C
Z_7357921_C	25C	51C
Z_7357922_C	25C	51C
Z_7357923_C	24C	51C
Z_7357924_G	25G	51G
Z_7357925_T	23T	50T
Z_7357926_G	24G	49G
Z_7357927_C	23C	49C
Z_7357928_G	25G	49G
Z_7357929_C	25C	48C
Z_7357930_G	25G	47G
Z_7357931_C	25C	47C
Z_7357932_T	23T	48T
Z_7357933_C	23C	50C
Z_7357934_G	21G	48G
Z_7357935_G	23G	48G
Z_7357936_A	23A	47A
Z_7357937_C	21C	49C
Z_7357938_T	22T	46T
Z_7357939_C	21C	47C
Z_7357940_C	22C	48C

Z_7357941_T	22T	48T
Z_7357942_G	21G	46G
Z_7357943_C	20C	46C
Z_7357944_G	22G	47G
Z_7357945_T	22T	46T
Z_7357946_A	24A	49A
Z_7357947_T	24T	48T
Z_7357948_C	23C	48C
Z_7357949_T	23T	48T
Z_7357950_T	22T	48T
Z_7357951_T	23T	49T
Z_7357952_T	22T	49T
Z_7357953_G	23G	49G
Z_7357954_C	23C	47C
Z_7357955_G	22G	50G
Z_7357956_C	22C	50C
Z_7357957_C	21C	49C
Z_7357958_A	21G	31A_19G
Z_7357959_G	22G	49G
Z_7357960_G	21G	49G
Z_7357961_C	22C	47C
Z_7357962_T	22T	47T
Z_7357963_G	22G	48G
Z_7357964_C	23C	46C
Z_7357965_G	20G	47G
Z_7357966_A	19A	47A
Z_7357967_A	19A	48A
Z_7357968_A	18A	45A
Z_7357969_C	18C	47C
Z_7357970_G	18G	45G
Z_7357971_T	18T	46T
Z_7357972_T	17T	41T
Z_7357973_C	19C	46C
Z_7357974_C	20C	46C
Z_7357975_T	18T	46T
Z_7357976_C	19C	45C
Z_7357977_T	20T	44T
Z_7357978_G	20G	45G
Z_7357979_T	20T	46T
Z_7357980_T	21T	46T
Z_7357981_T	21T	46T
Z_7357982_T	21T	46T
Z_7357983_G	21G	44G

Z_7357984_G	20G	45G
Z_7357985_A	21A	44A
Z_7357986_T	20T	44T
Z_7357987_C	22C	45C
Z_7357988_C	22C	49C
Z_7357989_A	22A	47A
Z_7357990_A	22A	47A
Z_7357991_A	22A	47A
Z_7357992_A	22A	48A
Z_7357993_C	22C	49C
Z_7357994_A	22A	50A
Z_7357995_G	23G	50G
Z_7357996_A	23A	44A
Z_7357997_G	22G	50G
Z_7357998_G	21G	50G
Z_7357999_A	21A	41A
Z_7358000_A	21A	40A
Z_7358001_C	20C	47C
Z_7358002_G	19G	45G
Z_7358003_T	20T	47T
Z_7358004_T	19T	50T
Z_7358005_T	19T	50T
Z_7358006_T	19T	52T
Z_7358007_C	18C	52C
Z_7358008_T	19T	52T
Z_7358009_A	18A	52A
Z_7358010_G	18G	51G
Z_7358011_C	18C	51C
Z_7358012_G	18G	50G
Z_7358013_T	18T	50T
Z_7358014_C	16C	21T_29C
Z_7358015_G	17G	50G
Z_7358016_A	15A	48A
Z_7358017_T	69T	50T
Z_7358018_C	6T	50C
Z_7358019_T	6T	116T
Z_7358020_T	6T	36T
Z_7358021_T	5T	37T
Z_7358022_T	5T	38T
Z_7358023_T	4T	38T
Z_7358024_T	4T	62T
Z_7358025_T	5T	62T
Z_7358026_T	13T	63T

Z_7358027_T	13T	58T
Z_7358028_T	14T	58T
Z_7358029_T	14T	58T
Z_7358030_T	14T	57T
Z_7358031_T	14T	56T
Z_7358032_C	14T	57C
Z_7358033_T	14T	58T
Z_7358034_T	14T	58T
Z_7358035_C	14C	59C
Z_7358036_C	14C	59C
Z_7358037_T	14T	59T
Z_7358038_G	15G	59G
Z_7358039_G	15G	60G
Z_7358040_C	14C	60C
Z_7358041_G	15G	61G
Z_7358042_T	15T	60T
Z_7358043_G	17G	60G
Z_7358044_G	17G	59G
Z_7358045_G	17G	59G
Z_7358046_C	18C	57C
Z_7358047_G	19G	59G
Z_7358048_A	19A	59A
Z_7358049_G	19G	59G
Z_7358050_G	19G	59G
Z_7358051_G	21G	59G
Z_7358052_A	19A	63A
Z_7358053_T	19T	63T
Z_7358054_C	20C	65C
Z_7358055_T	20T	65T
Z_7358056_C	20C	65C
Z_7358057_T	21T	65T
Z_7358058_T	20T	65T
Z_7358059_T	19T	65T
Z_7358060_A	19A	65A
Z_7358061_A	19A	70A
Z_7358062_G	19G	70G
Z_7358063_G	18G	70G
Z_7358064_G	19G	70G
Z_7358065_T	19T	71T
Z_7358066_C	19C	72C
Z_7358067_C	19C	72C
Z_7358068_G	19G	71G
Z_7358069_A	19A	69A

Z_7358070_T	20T	69T
Z_7358071_A	20A	69A
Z_7358072_G	20G	68G
Z_7358073_A	20A	67A
Z_7358074_T	20G	68T
Z_7358075_A	21A	68A
Z_7358076_A	21A	70A
Z_7358077_G	21G	70G
Z_7358078_A	22A	72A
Z_7358079_T	22T	71T
Z_7358080_T	22T	74T
Z_7358081_T	23T	74T
Z_7358082_T	20C	72T
Z_7358083_C	23C	69C
Z_7358084_C	24C	71C
Z_7358085_C	23C	71C
Z_7358086_C	23C	71C
Z_7358087_T	23T	70T
Z_7358088_T	23T	68T
Z_7358089_T	20T	66T
Z_7358090_T	22T	66T
Z_7358091_C	22C	66C
Z_7358092_A	21A	65A
Z_7358093_A	22A	65A
Z_7358094_A	21A	67A
Z_7358095_A	21A	66A
Z_7358096_G	20G	67G
Z_7358097_A	20A	67A
Z_7358098_G	21G	66G
Z_7358099_A	21A	65A
Z_7358100_G	21G	65G
Z_7358101_G	21G	64G
Z_7358102_C	21C	65C
Z_7358103_G	22G	67G
Z_7358104_C	22C	66C
Z_7358105_G	22G	67G
Z_7358106_C	22C	67C
Z_7358107_T	22T	68T
Z_7358108_G	21G	70G
Z_7358109_G	21G	70G
Z_7358110_T	22C	40T_29C
Z_7358111_G	22G	69G
Z_7358112_G	22G	69G

Z_7358113_T	19T	66T
Z_7358114_C	20C	65C
Z_7358115_T	19T	65T
Z_7358116_C	18C	66C
Z_7358117_T	17T	65T
Z_7358118_T	17T	66T
Z_7358119_G	17G	66G
Z_7358120_G	17G	67G
Z_7358121_C	16C	66C
Z_7358122_A	16A	66A
Z_7358123_G	17G	67G
Z_7358124_A	16A	66A
Z_7358125_G	16G	68G
Z_7358126_G	15G	68G
Z_7358127_T	16T	69T
Z_7358128_T	16T	67T
Z_7358129_G	16G	69G
Z_7358130_G	16G	67G
Z_7358131_G	16G	67G
Z_7358132_C	16C	68C
Z_7358133_A	15A	68A
Z_7358134_T	15T	67T
Z_7358135_T	14T	66T
Z_7358136_T	14T	66T
Z_7358137_T	15T	66T
Z_7358138_A	15A	66A
Z_7358139_G	17G	66G
Z_7358140_G	18G	63G
Z_7358141_G	18G	64G
Z_7358142_G	18G	65G
Z_7358143_C	18C	64C
Z_7358144_G	17G	64G
Z_7358145_T	16T	63T
Z_7358146_C	16C	61C
Z_7358147_C	16C	58C
Z_7358148_C	15C	57C
Z_7358149_T	15T	57T
Z_7358150_C	15C	56C
Z_7358151_G	14G	56G
Z_7358152_A	14A	55A
Z_7358153_G	13G	56G
Z_7358154_C	13C	57C
Z_7358155_A	13A	56A

Z_7358156_A	13A	52A
Z_7358157_C	13C	53C
Z_7358158_G	13G	53G
Z_7358159_C	13C	52C
Z_7358160_C	14C	51C
Z_7358161_G	14A	49G
Z_7358162_G	15G	48G
Z_7358163_G	14G	48G
Z_7358164_T	14T	48T
Z_7358165_T	15T	48T
Z_7358166_G	15G	49G
Z_7358167_T	15T	47T
Z_7358168_G	13G	49G
Z_7358169_G	13G	48G
Z_7358170_G	13G	46G
Z_7358171_G	13G	45G
Z_7358172_G	12G	45G
Z_7358173_A	12A	42A
Z_7358174_T	11T	42T
Z_7358175_C	12C	40C
Z_7358176_C	12C	40C
Z_7358177_C	12C	40C
Z_7358178_T	12T	40T
Z_7358179_T	12T	39T
Z_7358180_C	11C	38C
Z_7358181_C	11C	39C
Z_7358182_C	11C	39C
Z_7358183_T	11T	37T
Z_7358184_C	11C	39C
Z_7358185_C	11T	41C
Z_7358186_T	11T	41T
Z_7358187_C	10C	41C
Z_7358188_G	10G	41G
Z_7358189_A	11A	38A
Z_7358190_T	12T	37T
Z_7358191_C	12C	37C
Z_7358192_G	12G	37G
Z_7358193_G	12G	37G
Z_7358194_C	12C	36C
Z_7358195_C	13C	36C
Z_7358196_T	12T	34T
Z_7358197_C	11C	34C
Z_7358198_C	12C	32C

Z_7358199_C	12C	33C
Z_7358200_C	11C	33C
Z_7358201_C	12C	31C
Z_7358202_T	12T	26T
Z_7358203_G	12G	29G
Z_7358204_G	12G	27G
Z_7358205_C	12C	26C
Z_7358206_G	12G	27G
Z_7358207_C	12C	29C
Z_7358208_C	12C	29C
Z_7358209_G	10C	18G_9C
Z_7358210_C	8C	27C
Z_7358211_C	7C	25C
Z_7358212_A	8A	24A
Z_7358213_A	8A	24A
Z_7358214_A	8A	25A
Z_7358215_G	7G	25G
Z_7358216_C	8C	26C
Z_7358217_A	9A	25A
Z_7358218_C	9C	25C
Z_7358219_G	9G	27G
Z_7358220_A	9A	25A
Z_7358221_C	9C	26C
Z_7358222_C	9C	25C
Z_7358223_C	9C	26C
Z_7358224_T	9T	24T
Z_7358225_C	9C	25C
Z_7358226_T	8T	24T
Z_7358227_C	9C	26C
Z_7358228_C	9C	26C
Z_7358229_T	9T	26T
Z_7358230_C	8C	26C
Z_7358231_T	8T	25T
Z_7358232_C	7C	26C
Z_7358233_A	7A	24A
Z_7358234_C	3A_6T_16C	26C
Z_7358235_C	6C	26C
Z_7358236_C	3T_3C	27C
Z_7358237_C	6C	26C
Z_7358238_C	3T_3C	26C
Z_7358239_C	6C	27C
Z_7358240_T	2A_4T	27T
Z_7358241_C	6C	28C

Z_7358242_C	6C	29C
Z_7358243_T	6T	30T
Z_7358244_C	8C	29C
Z_7358245_C	8C	29C
Z_7358246_C	9C	29C
Z_7358247_T	6T_3C	29T
Z_7358248_C	9C	32C
Z_7358249_C	9C	31C
Z_7358250_T	9T	30T
Z_7358251_C	9C	30C
Z_7358252_C	9C	31C
Z_7358253_T	2T_6C	31T
Z_7358254_C	7C	32C
Z_7358255_C	7C	33C
Z_7358256_C	7C	33C
Z_7358257_T	6T	33T
Z_7358258_C	6C	33C
Z_7358259_C	6C	34C
Z_7358260_T	7T	35T
Z_7358261_C	7C	36C
Z_7358262_C	6C	36C
Z_7358263_C	6C	34C
Z_7358264_G	5G	34G
Z_7358265_G	6G	34G
Z_7358266_G	6G	35G
Z_7358267_G	6G	36G
Z_7358268_A	6A	35A
Z_7358269_T	6T	36T
Z_7358270_C	6C	36C
Z_7358271_C	7C	36C
Z_7358272_C	8C	37C
Z_7358273_A	8A	37A
Z_7358274_G	8G	37G
Z_7358275_G	8G	37G
Z_7358276_C	9C	37C
Z_7358277_C	9C	37C
Z_7358278_G	9G	36G
Z_7358279_G	9G	37G
Z_7358280_C	9C	35C
Z_7358281_T	9T	34T
Z_7358282_T	10T	35T
Z_7358283_C	10C	36C
Z_7358284_T	10T	36T

Z_7358285_G	10G	36G
Z_7358286_C	11C	37C
Z_7358287_C	11C	37C
Z_7358288_T	10T	39T
Z_7358289_C	10C	41C
Z_7358290_T	10T	40T
Z_7358291_C	10C	42C
Z_7358292_C	10C	41C
Z_7358293_T	10T	41T
Z_7358294_G	10G	40G
Z_7358295_C	10C	40C
Z_7358296_C	10C	40C
Z_7358297_C	11C	40C
Z_7358298_G	11G	41G
Z_7358299_A	11A	39A
Z_7358300_C	11C	42C
Z_7358301_C	11C	42C
Z_7358302_C	10C	42C
Z_7358303_G	10G	42G
Z_7358304_T	10T	40T
Z_7358305_C	11C	41C
Z_7358306_G	10G	43G
Z_7358307_C	10C	44C
Z_7358308_G	12G	43G
Z_7358309_G	13G	42G
Z_7358310_G	13G	41G
Z_7358311_C	13C	40C
Z_7358312_T	13T	39T
Z_7358313_T	13T	41T
Z_7358314_A	14A	39A
Z_7358315_C	14C	39C
Z_7358316_C	14C	40C
Z_7358317_T	14T	40T
Z_7358318_T	14T	39T
Z_7358319_G	14G	42G
Z_7358320_C	14C	42C
Z_7358321_A	14A	42A
Z_7358322_G	14G	42G
Z_7358323_C	14C	42C
Z_7358324_T	14T	42T
Z_7358325_T	14T	43T
Z_7358326_T	15T	45T
Z_7358327_C	15C	44C

Z_7358328_G	15G	46G
Z_7358329_C	15C	46C
Z_7358330_A	14A	45A
Z_7358331_G	14G	46G
Z_7358332_G	15G	46G
Z_7358333_T	15T	46T
Z_7358334_G	15G	46G
Z_7358335_A	15A	47A
Z_7358336_G	16G	46G
Z_7358337_G	16G	44G
Z_7358338_A	16A	43A
Z_7358339_G	16G	43G
Z_7358340_A	16A	42A
Z_7358341_C	15C	42C
Z_7358342_C	14C	43C
Z_7358343_G	15G	40G
Z_7358344_T	15T	40T
Z_7358345_A	14A	40A
Z_7358346_A	14A	40A
Z_7358347_T	13T	38T
Z_7358348_G	12G	39G
Z_7358349_G	12G	38G
Z_7358350_T	13T	38T
Z_7358351_A	13A	38A
Z_7358352_G	12G	38G
Z_7358353_C	12C	38C
Z_7358354_C	12C	37C
Z_7358355_G	12G	37G
Z_7358356_G	10G	37G
Z_7358357_A	12A	37A
Z_7358358_G	12G	38G
Z_7358359_A	12A	37A
Z_7358360_C	12C	39C
Z_7358361_C	12C	38C
Z_7358362_T	12T	39T
Z_7358363_T	12T	40T
Z_7358364_G	12G	41G
Z_7358365_T	11T	40T
Z_7358366_C	12C	40C
Z_7358367_C	11C	39C
Z_7358368_C	11C	40C
Z_7358369_C	11C	40C
Z_7358370_G	12G	40G

Z_7358371_C	11C	40C
Z_7358372_A	11A	40A
Z_7358373_G	11G	41G
Z_7358374_A	11A	40A
Z_7358375_C	10C	41C
Z_7358376_G	11G	41G
Z_7358377_G	11G	40G
Z_7358378_G	9G	40G
Z_7358379_A	8A	41A
Z_7358380_C	8C	41C
Z_7358381_A	8A	41A
Z_7358382_G	9G	42G
Z_7358383_A	9A	40A
Z_7358384_G	9G	38G
Z_7358385_C	9C	38C
Z_7358386_T	9T	37T
Z_7358387_C	9C	38C
Z_7358388_G	10G	38G
Z_7358389_T	10T	38T
Z_7358390_C	10C	38C
Z_7358391_C	10C	39C
Z_7358392_A	10A	39A
Z_7358393_G	10G	40G
Z_7358394_A	10A	40A
Z_7358395_T	10T	39T
Z_7358396_C	9C	40C
Z_7358397_C	9C	38C
Z_7358398_T	9T	38T
Z_7358399_C	10C	38C
Z_7358400_G	11G	38G
Z_7358401_T	11T	38T
Z_7358402_C	11C	37C
Z_7358403_G	11G	37G
Z_7358404_T	11T	37T
Z_7358405_A	10A	37A
Z_7358406_G	10G	37G
Z_7358407_G	10G	37G
Z_7358408_A	11A	36A
Z_7358409_A	11A	37A
Z_7358410_T	12T	35T
Z_7358411_A	12A	37A
Z_7358412_A	12A	37A
Z_7358413_T	11T	37T

Z_7358414_C	12C	34C
Z_7358415_C	13C	34C
Z_7358416_A	13A	36A
Z_7358417_T	13T	38T
Z_7358418_C	14C	38C
Z_7358419_C	14C	38C
Z_7358420_C	15C	36C
Z_7358421_C	15C	36C
Z_7358422_G	15G	36G
Z_7358423_C	15C	34C
Z_7358424_C	15C	35C
Z_7358425_G	15G	35G
Z_7358426_C	15C	34C
Z_7358427_C	15C	34C
Z_7358428_G	14G	33G
Z_7358429_G	15G	33G
Z_7358430_A	15A	32A
Z_7358431_C	15C	32C
Z_7358432_C	15C	34C

the *nr5a1* gene locus (26,763 bp in length) across 20 samples.

ZZ_3	ZZ_4	ZZ_5
86G	76G	106G
87T	78T	105T
88C	80C	101C
88C	81C	103C
87C	81C	101C
84A	79A	101A
89G	79G	102G
72C	68C	102C
86T	80T	103T
85C	79C	103C
86C	80C	101C
86T	79T	101T
88G	78G	101G
87C	77C	96C
87C	77C	100C
87A	75A	101A
86A	73A	99A
81C	73C	96C
86C	71C	99C
86T	71T	98T
85A	72A	99A
84G	71G	98G
83C	74C	102C
67A	66A	98A
82G	76G	97G
82T	75T	96T
82T	78T	93T
82C	78C	90C
69A	71A	88A
82A	77A	91A
82A	77A	91A
80A	77A	90A
76G	74G	88G
80C	77C	87C
80A	75A	85A
70T	73T	85T
80G	74G	83G
5A_48T_27C	76T	77T
81A	74A	83A
81A	75A	77A
81A	76A	80A

80T	75T	79T
81G	73G	75G
77C	73C	71C
76G	73G	70G
79A	73A	70A
77G	73G	70G
70T	74T	70T
70A	69A	67A
70G	70G	67G
72A	73A	68A
72T	74T	68T
72A	72A	68A
71A	72A	66A
44A_26G	41A_32G	53G
70T	75T	59T
70A	75A	59A
70G	76G	57G
66G	76G	56G
26T_40G	34T_42G	51T
66A	74A	52A
24G_41C	75C	48G
62C	75C	57C
70A	74A	58A
70C	76C	57C
66C	75C	59C
63A	73A	56A
62T	72T	57T
57A	71A	58A
62G	72G	60G
61T	74T	59T
26A_35G	74A	59G
62G	74G	59G
62G	75G	61G
62A	76A	62A
61A	73A	62A
61G	71G	61G
59G	71G	64G
59T	71T	66T
58A	73A	70A
57A	73A	70A
58T	73T	69T
58G	73G	69G
54G	73G	68G

59C	74C	68C
59G	73G	66G
60T	75T	66T
60T	78T	66T
60C	78C	67C
58T	79T	68T
59A	81A	72A
58T	82T	71T
57G	82G	71G
57T	82T	70T
57C	84C	72C
57T	86T	74T
56A	86A	74A
57G	84G	73G
56T	84T	72T
56A	91A	72A
56G	93G	71G
56A	94A	73A
56A	96A	72A
55A	97A	71A
58C	98C	71C
57T	101T	71T
57G	102G	73G
56T	102T	70T
57C	100C	70C
57C	91C	72C
59A	99A	71A
59C	100C	73C
61G	101G	75G
61G	101G	76G
61A	102A	76A
61C	104C	77C
61A	104A	78A
59A	104A	78A
59A	104A	77A
59T	64T_39C	76T
60G	104G	76G
60C	90C	77C
59C	38T_1G_64C	76C
60G	102G	78G
62G	103G	75G
62C	48A_1G_55C	74C
62T	129T	74T

62C	130C	73C
62T	48A_82T	77T
63A	130A	76A
62C	133C	75C
62A	132A	77A
62G	132G	77G
64C	133C	78C
64T	134T	78T
65T	135T	81T
65G	138G	81G
66G	137G	81G
66A	138A	81A
64G	137G	80G
65A	135A	79A
65T	134T	78T
67G	130G	81G
68G	130G	78G
67G	128G	77G
68G	128G	77G
68A	125A	80A
66T	125T	77T
68G	124G	78G
64A	125A	76A
68G	126G	77G
68C	126C	29G_49C
68A	126A	78A
67C	123C	83C
67C	124C	81C
68G	125G	39A_43G_1C
69C	125C	84C
66G	124G	83G
67C	125C	85C
68C	75A_50T_100G_175C	85C
69C	99C	82C
70T	99T	83T
69A	99A	82A
71G	99G	81G
70A	100A	80A
69G	100G	80G
70T	99T	78T
72C	101C	82C
71G	101G	82G
72G	101G	83G

73A	101A	87A
76C	125C	84C
74A	125A	86A
73C	125C	88C
72A	122A	89A
74A	122A	90A
74C	123C	89C
73T	121T	89T
71G	120G	90G
72G	118G	91G
71A	117A	90A
71C	117C	89C
72T	117T	89T
69A	119A	88A
69A	118A	89A
69A	117A	89A
69T	117T	90T
69G	119G	89G
68T	122T	89T
70C	122C	90C
70A	123A	91A
70A	123A	91A
70G	126G	90G
68G	126G	89G
68G	125G	89G
67G	123G	90G
67A	123A	89A
68A	123A	92A
68C	123C	89C
68C	99C	88C
67T	117T	88T
65T	116T	89T
66T	99T	89T
68A	100A	86A
65C	117C	86C
63C	118C	85C
64T	117T	85T
64T	112T	86T
64T	112T	87T
63A	113A	87A
62C	112C	89C
61C	96C	85C
63T	95T	84T

64A	80A	87A
62T	80T	86T
59A	78A	83A
59T	79T	82T
57C	78C	82C
58T	80T	82T
58G	80G	79G
58G	75G	79G
58C	78C	78C
59T	75T	78T
59A	77A	74A
58C	76C	76C
57T	73T	74T
59C	72C	73C
58T	69T	71T
58G	70G	72G
57T	72T	71C
56C	73C	71C
54A	73A	69A
55C	73C	70C
56C	71C	70C
57C	71C	38T_32C
56T	70T	69T
57C	71C	70C
56T	73T	71T
60C	74C	70C
60T	73T	70T
60C	73C	65C
61T	73T	65T
59A	70A	64A
63C	72C	61C
63C	72C	61C
63C	72C	61C
61T	72T	61T
63C	72C	62C
65C	72C	60C
65C	71C	61C
63G	71G	60G
64G	71G	25A_34G
63T	70T	57T
61G	69G	57G
60G	70G	60G
60G	70G	59G

60T	66T	57T
58G	69G	57G
58G	69G	55G
58A	67A	55A
58T	70T	55T
58T	70T	56T
57C	70C	57C
57T	71T	57T
57C	72C	59C
56G	72G	57G
56T	72T	57T
57A	72A	57A
55G	73G	57G
55T	74T	57T
54T	73T	57T
54C	72C	55C
54C	72C	57C
54T	74T	60T
53A	75A	58A
53C	75C	58C
52T	75T	58T
53C	74C	57C
54A	74A	56A
53C	73C	54C
51A	73A	54A
51C	73C	55C
50T	74T	57T
49G	72G	53G
50T	71T	54T
49C	72C	55C
49A	73A	52A
48A	71A	50A
47G	71G	51G
46T	70T	52T
44G	71G	52G
42A	70A	55A
42T	72T	54T
43A	72A	54A
42A	72A	53A
42T	71T	53T
44T	70T	53T
44C	72C	56C
46A	74A	55A

44T	74T	55T
44C	72C	56C
45C	76C	56C
46T	75T	56T
46T	75T	54T
45C	75C	56C
45A	75A	56A
45C	75C	57C
44A	73A	54A
43G	70G	56G
42T	69T	56T
42C	70C	57C
42T	70T	59T
42G	66G	59G
42A	64A	59A
42A	63A	58A
42T	63T	57T
42G	65G	58G
43C	66C	57C
45A	66A	57A
45A	67A	57A
45A	65A	56A
44G	66G	79G
45G	67G	10A_34G
42A_13G	28A_1T_83G	19A_72G
43A	57A	47A
43A	63A	52A
42A	64A	52A
42A	64A	53A
42A	65A	53A
40A	57A	55A
39A_24T	58T	35A_49T
44A	63A	57A
44A	63A	54A
43A	64A	60A
43A	63A	60A
43A	63A	62A
42T	65T	60T
42A	66A	61A
42A	66A	54A
42G	63G	57G
43G	64G	57G
42G	63G	57G

41C	63C	60C
41C	63C	59C
42A	63A	59A
41G	64G	57G
41C	65C	56C
41T	65T	56T
41C	64C	55C
41A	65A	56A
41C	66C	56C
41T	65T	58T
41G	67G	57G
41G	68G	56G
39T	65T	56T
40C	66C	57C
39C	66C	56C
37C	66C	57C
37C	66C	59C
36A	66A	58A
35A	67A	57A
35G	67G	59G
34C	68C	57C
34A	67A	57A
33G	67G	57G
34C	66C	59C
35A	67A	59A
34A	69A	59A
33A	69A	58A
33G	70G	58G
33A	70A	61A
33T	70T	61T
33A	70A	61A
34A	70A	61A
34G	71G	62G
34G	71G	62G
35A	71A	60A
35C	71C	60C
35T	69T	59T
35C	68C	57C
17A_17G	33A_35G	57G
34G	68G	59G
35C	69C	59C
35A	69A	59A
37A	67A	59A

38A	67A	59A
37G	67G	60G
37C	66C	60C
38G	66G	60G
38G	66G	60G
39G	68G	62G
39G	68G	60G
40A	68A	59A
41G	68G	60G
41A	68A	61A
41A	68A	61A
40A	67A	62A
39A	67A	62A
40C	68C	64C
40C	69C	63C
39C	69C	62C
40A	68A	63A
39C	69C	63C
40A	71A	60A
41C	70C	59C
40T	71T	57T
40T	73T	57T
40G	72G	59G
41A	73A	57A
41C	74C	58C
42T	74T	60T
42A	73A	59A
40T	73T	56T
40A	72A	54A
40A	72A	53A
38G	70G	53G
37T	67T	53T
35G	70G	52G
34A	69A	53A
36A	67A	52A
38T	67T	53T
35C	68C	52C
35G	69G	52G
36T	70T	51T
36A	69A	51A
37A	69A	51A
39A	69A	50A
40A	68A	52A

40T	68T	51T
41T	68T	54T
41T	70T	54T
40A	71A	53A
41T	70T	54T
41T	71T	54T
42G	70G	54G
42G	70G	51G
42A	70A	50A
42A	68A	52A
42T	67T	51T
43G	66G	55G
44T	65T	52T
45C	67C	53C
46T	66T	53T
46T	67T	53T
46C	67C	51C
46C	66C	53C
46A	64A	55A
45T	64T	53T
45T	64T	52T
45T	66T	53T
46G	63G	54G
45G	64G	53G
45T	63T	53T
45A	63A	54A
44A	64A	54A
45G	62G	55G
45A	61A	56A
46G	62G	56G
46A	61A	58A
46G	62G	58G
46C	61C	56C
46T	63T	56T
47C	62C	56C
47A	62A	56A
46T	62T	56T
47C	61C	56C
47T	61T	56T
47T	59T	55T
47A	60A	55A
45A	59A	56A
47C	59C	56C

47C	60C	56C
47C	58C	56C
47C	58C	59C
47C	58C	59C
46T	58T	59T
47C	58C	63C
47T	57T	60T
46G	59G	61G
45G	59G	61G
46G	58G	61G
46G	57G	61G
46A	57A	62A
46G	59G	63G
46T	60T	63T
46A	61A	63A
47A	61A	65A
46A	60A	68A
46A	60A	68A
46G	59G	68G
46G	60G	67G
46G	62G	66G
46A	63A	67A
45A	62A	67A
46A	62A	67A
46C	60C	66C
45A	61A	67A
46C	61C	63C
48T	61T	65T
50T	61T	62T
49A	61A	62A
49A	61A	63A
48G	60G	64G
49G	60G	64G
46A	60A	62A
46C	59C	64C
45A	60A	64A
47C	62C	64C
47T	63T	64T
48T	62T	61T
48T	60T	60T
48T	61T	60T
47T	62T	60T
46C	63C	60C

46G	62G	61G
46T	61T	59T
47T	61T	59T
47T	61T	62T
46C	62C	62C
46G	60G	61G
46A	60A	61A
46G	60G	62G
46A	61A	30A_32G
45A	61A	61A
44A	61A	61A
45G	60G	61G
45A	58A	59A
45A	58A	59A
44A	58A	57A
44G	57G	58G
43G	57G	60G
43A	57A	60A
43A	57A	59A
44A	58A	58A
44A	58A	57A
44A	58A	57A
43C	59C	57C
44C	60C	60C
44A	61A	59A
42A	61A	60A
87A_46G_1C	109A_50G	92A_40G
43A	59A	63A
43G	59G	62G
43A	60A	60A
43G	59G	62G
42A	61A	62A
40G	61G	59G
39A	62A	63A
40G	63G	62G
39A	62A	61A
39G	60G	61G
39A	63A	62A
39G	66G	62G
39A	67A	61A
38G	69G	63G
42A	69A	64A
41T	68T	61T

41C	70C	61C
41A	71A	63A
41C	69C	64C
45A	73A	65A
46G	72G	65G
48A	73A	65A
48G	73G	66G
47A	73A	65A
48G	73G	66G
49A	73A	65A
49G	71G	65G
50A	73A	70A
49G	71G	63G
49A	70A	61A
48G	69G	63G
48A	69A	64A
34A_14G	85A_26G	27A_34G
48A	69A	63A
48G	67G	65G
47A	67A	65A
47G	65G	64G
47A	67A	66A
47G	67G	66G
47A	70A	66A
48G	71G	64G
50A	72A	65A
49G	74G	65G
49A	72A	63A
50G	75G	64G
50A	75A	60A
23A_23T_74G	37A_37T_111G	20A_40T_99G
26A	33A	32A
26G	41G	14T_38G
19A_21T_21G_13C	22A	21A
29G	59G	24G
30T	48T	24A
31G	65G	59G
32T	64T	61T
33G	65G	62G
33A	65A	66A
33C	65C	67C
33A	67A	69A
32C	68C	70C

43A	68A	71A
44T	69T	72T
44T	69T	73T
44C	70C	74C
44A	71A	73A
44A	70A	71A
44A	68A	72A
45C	69C	72C
43T	69T	73T
43A	68A	74A
43T	69T	73T
43G	69G	71G
43T	70T	70T
44T	68T	68T
45C	67C	71C
45T	68T	69T
44C	67C	67C
44C	67C	68C
45A	69A	69A
46G	68G	68G
45C	69C	65C
15A_29T	25A_45T	65A
44A	70A	66A
43T	69T	63T
44T	69T	65T
43T	69T	66T
43C	70C	65C
43A	70A	63A
43A	69A	63A
42A	68A	62A
42C	66C	60C
43C	66C	63C
44A	67A	65A
44C	66C	67C
44T	66T	66T
44G	65G	68G
45C	66C	70C
45A	66A	70A
47C	68C	72C
47T	68T	72T
48C	67C	74C
49A	67A	74A
49T	67T	75T

51G	68G	76G
51C	69C	77C
51A	66A	76A
51C	66C	75C
51G	69G	74G
51T	70T	74T
51C	70C	77C
52T	70T	78T
52A	70A	81A
52T	71T	80T
53G	71G	81G
53C	70C	81C
52C	71C	81C
52G	73G	81G
55A	72A	81A
56A	75A	83A
58C	73C	80C
58A	72A	78A
58G	73G	77G
58A	73A	77A
57G	74G	77G
56T	74T	74T
57C	75C	75C
57C	77C	71C
57A	74A	72A
58G	76G	70G
57A	75A	69A
58C	75C	69C
58G	75G	70G
58T	73T	69T
58C	75C	68C
59A	77A	66A
59C	74C	69C
57A	71A	69A
59G	71G	69G
60G	70G	68G
58A	70A	69A
58A	69A	72A
59C	69C	73C
21A_38G	69G	74G
60G	69G	76G
59A	69A	76A
59A	69A	77A

59A	68A	77A
58A	69A	77A
59A	70A	77A
58A	71A	79A
58G	73G	80G
59G	74G	83G
56T	72T	84T
56A	73A	83A
56G	73G	81G
56C	73C	81C
56A	74A	81A
57A	74A	81A
58C	74C	28T_48C
57A	75A	49A_1T_27G
58T	75T	82T
57G	75G	82G
55T	74T	83T
55T	73T	82T
53T	72T	82T
54G	70G	79G
55T	68T	80T
53G	70G	79G
53G	68G	77G
53A	67A	78A
53G	67G	77G
53G	69G	77G
52T	66T	77T
52G	68G	77G
51C	70C	76C
49A	71A	78A
51G	71G	80G
51A	71A	80A
51A	69A	80A
53A	69A	77A
53G	68G	78G
52C	66C	75C
52C	66C	76C
52A	65A	75A
54T	64T	76T
54T	66T	77T
54G	66G	76G
53C	66C	75C
54T	66T	75T

55G	66G	74G
56C	65C	74C
56G	35A_31G	73G
56G	65G	76G
55T	66T	75T
54G	65G	75G
53A	63A	73A
53C	65C	75C
54C	63C	74C
52C	61C	75C
51T	62T	75T
52T	62T	75T
52G	61G	73G
53A	63A	76A
53T	63T	76T
54G	63G	80G
55G	64G	79G
55C	63C	80C
55A	62A	80A
55C	63C	80C
56A	63A	78A
57A	68A	78A
58A	68A	78A
59A	68A	78A
59A	67A	72A
59A	69A	41A_31T
54A	68A	60A
58G	70G	71G
57G	72G	70G
59G	71G	67G
57G	70G	60G
57A	70A	62A
60T	71T	62T
61G	71G	62G
61G	72G	62G
60A	71A	62A
60A	69A	63A
60A	69A	65A
60T	69T	66T
61G	67G	64G
62C	66C	62C
63T	66T	61T
63C	66C	60C

65C	64C	60C
64T	65T	59T
66G	65G	60G
66A	65A	61A
66C	64C	62C
66A	63A	63A
63A	63A	63A
62A	59A	63A
62C	63C	64C
62C	63C	65C
62C	63C	65C
62A	63A	63A
62A	62A	64A
62C	61C	63C
62A	61A	64A
64C	58C	62C
63A	58A	61A
62A	57A	61A
62G	56G	63G
61G	57G	61G
61C	57C	62C
60A	57A	61A
61A	56A	62A
60G	55G	63G
60G	56G	63G
59A	57A	65A
59G	58G	64G
58T	59T	63T
59A	59A	65A
59G	61G	65G
59A	61A	64A
60T	60T	68T
59T	62T	43T_26C
59C	61C	70C
60G	62G	69G
62C	62C	73C
63T	62T	72T
62G	62G	73G
61C	60C	75C
59A	60A	74A
58G	59G	75G
58G	59G	71G
58A	60A	73A

58A	61A	69A
58C	62C	70C
60A	61A	69A
59A	60A	68A
60C	59C	65C
60A	58A	66A
60C	57C	65C
31T_30C	22T_36C	65T
61G	58G	65G
62T	59T	65T
63C	59C	64C
61C	57C	64C
61A	57A	64A
62G	58G	64G
64C	58C	33G_28C
62C	60C	62C
62C	61C	67C
64A	59A	68A
64G	60G	68G
63T	61T	69T
65T	61T	68T
65C	61C	67C
63A	61A	66A
65G	63G	71G
61G	61G	21A_63G
31A_30G	36A_25G	21A_43G
59A_30G	59A_25G	61A
65A	63A	76A
65A	63A	76A
65A	65A	74A
67A	64A	74A
67A	66A	75A
65A	66A	74A
65A	65A	74A
65T	63T	71T
65C	64C	71C
66A	64A	73A
66C	64C	70C
37T_1G_29C	41T_23C	71C
69T	64T	71T
69G	64G	74G
70T	64T	72T
69C	64C	74C

70T	64T	74T
70G	63G	73G
71A	62A	73A
70T	63T	74T
70C	65C	75C
69T	65T	78T
68A	66A	77A
68C	66C	75C
67A	65A	77A
67T	65T	80T
67T	65T	79T
65G	64G	79G
65C	63C	78C
61A	63A	78A
61C	63C	77C
61G	63G	76G
61T	60T	77T
62T	59T	76T
62T	59T	76T
61A	57A	77A
61T	57T	76T
27A_34T	22A_35T	74A
59G	57G	74G
57C	57C	73C
57C	57C	76C
57A	58A	76A
58T	57T	77T
58T	57T	75T
58T	57T	73T
58G	56G	75G
25A_30G	25A_33G	74A
55G	60G	75G
56A	61A	74A
54C	61C	74C
54C	60C	73C
52A	59A	73A
52C	59C	72C
51T	55T	59T
21T_31C	21T_33C	59T
52G	54G	62G
53A	55A	64A
53T	55T	65T
55C	58C	75C

24A_31T	25A_33T	75A
55T	64T	76T
56A	64A	81A
56G	65G	81G
56C	65C	83C
56T	65T	83T
57C	66C	85C
58C	66C	83C
60A	65A	84A
61T	65T	85T
61T	68T	82T
60C	68C	81C
62C	68C	79C
62C	69C	80C
63C	69C	80C
63A	70A	79A
64A	68A	83A
64A	67A	82A
63A	66A	81A
63T	67T	82T
63C	65C	83C
63C	64C	82C
64T	63T	84T
63G	62G	83G
63G	62G	84G
63G	63G	82G
60A	64A	81A
62T	63T	81T
63T	63T	84T
63G	63G	84G
63G	62G	85G
64T	62T	85T
64A	62A	83A
67G	61G	83G
68A	62A	81A
68T	61T	80T
66A	61A	77A
65G	62G	79G
64G	62G	79G
63G	61G	81G
64G	60G	82G
63G	62G	81G
61T	61T	79T

62A	62A	76A
62T	63T	76T
62G	65G	75G
62T	65T	74T
62A	65A	73A
59G	67G	73G
59A	66A	75A
60A	65A	76A
61A	67A	73A
64T	68T	73T
64G	68G	74G
63T	66T	76T
66C	66C	76C
65T	64T	76T
65G	65G	77G
65C	66C	77C
65T	66T	78T
64A	66A	81A
64G	66G	83G
64C	66C	82C
64A	64A	79A
62A	64A	81A
62A	62A	79A
63G	63G	78G
64C	63C	76C
61C	63C	74C
62T	62T	75T
61C	63C	77C
61C	62C	77C
61C	63C	73C
59G	64G	74G
58A	65A	74A
58A	66A	75A
57C	68C	73C
57T	69T	75T
56C	69C	78C
56T	70T	78T
56G	70G	80G
57G	68G	81G
57C	67C	81C
57A	67A	82A
59G	67G	82G
61A	66A	82A

63G	66G	78G
62A	71A	78A
64A	69A	79A
64C	70C	79C
64T	70T	78T
64C	68C	78C
63T	69T	78T
63T	68T	77T
62A	69A	75A
61A	70A	75A
61G	70G	74G
60C	70C	75C
60C	70C	73C
60C	71C	73C
58A	29A_43G	29A_42G
58T	71T	71T
58C	70C	70C
59C	72C	85C
59C	74C	84C
58C	70C	82C
59A	74A	84A
60A	72A	84A
59A	70A	86A
59C	70C	85C
58T	69T	85T
59G	70G	87G
59C	70C	85C
58A	69A	86A
58A	69A	88A
58A	68A	90A
58C	69T	46T_43C
59T	69T	90T
58C	68C	92C
59C	68C	90C
57A	68A	91A
59A	66A	92A
58T	67T	94T
59A	68A	93A
60C	68C	90C
61T	68T	89T
60A	67A	88A
60G	67G	86G
59A	67A	89A

59A	67A	87A
59C	64C	86C
59A	65A	84A
59T	65T	87T
58G	64G	87G
59C	65C	85C
58C	66C	85C
59A	66A	83A
57C	36T_30C	77T
57C	68C	80C
58A	32A_36G	79G
59A	72A	89A
61G	74G	89G
60A	74A	89A
60T	73T	94T
60T	74T	91T
60T	75T	90T
59T	74T	88T
58G	72G	89G
57C	72C	89C
57T	73T	43T_45C
57A	73A	86A
57T	75T	85T
59A	73A	85A
59C	76C	85C
56A	76A	86A
56T	78T	88T
56A	78A	87A
57C	79C	88C
58A	79A	87A
60A	79A	90A
61A	79A	90A
62T	77T	93T
64C	79C	94C
63C	79C	94C
64C	79C	94C
64A	77A	96A
63A	75A	98A
64G	76G	95G
64A	76A	92A
64T	76T	89T
64T	75T	92T
61T	72T	87T

60T	73T	87T
57G	74G	87G
56T	74T	88T
57T	75T	89T
55G	75G	92G
56A	75A	90A
57G	75G	91G
59A	75A	91A
59T	76T	90T
59G	75G	88G
60G	74G	91G
61A	75A	91A
61G	76G	91G
61C	78C	89C
60T	79T	89T
60G	80G	89G
59T	80T	87T
60G	38A_42G	88G
60A	80A	87A
60T	82T	85T
60A	84A	83A
59A	82A	84A
59A	83A	85A
61T	82T	86T
62G	82G	88G
65G	81G	89G
65T	81T	86T
65A	81A	86A
64T	80T	84T
65C	80C	85C
64A	80A	82A
64A	79A	83A
63A	79A	82A
62C	78C	83C
59T	78T	83T
58G	77G	81G
59A	74A	78A
56C	75C	80C
56A	76A	76A
56C	75C	76C
57A	76A	78A
58A	76A	72A
58C	77C	72C

58C	75C	72C
58T	75T	74T
59G	76G	74G
60T	75T	75T
60G	75G	73G
61T	36T_40C	76T
60C	78C	75C
62A	78A	78A
62T	78T	79T
62G	81G	76G
64T	80T	78T
64A	81A	78A
63G	80G	79G
63A	84A	80A
63G	83G	79G
63G	84G	79G
62G	83G	76G
61T	86T	75T
62C	86C	75C
62T	85T	75T
62C	85C	75C
62C	85C	75C
61A	82A	77A
62T	84T	79T
61C	85C	78C
63T	86T	75T
62C	85C	78C
63T	86T	78T
61C	84C	77C
61T	84T	76T
61T	84T	75T
62T	85T	74T
61C	86C	73C
61T	86T	73T
59T	86T	73T
58G	85G	72G
58A	87A	72A
58A	86A	72A
58T	86T	71T
57A	84A	69A
56T	80T	70T
56C	79C	71C
55C	80C	71C

56C	78C	73C
54A	78A	72A
54C	78C	73C
54C	77C	72C
55C	77C	73C
54A	78A	72A
54G	78G	73G
54T	77T	74T
54G	77G	72G
55G	77G	71G
55T	77T	76T
54T	77T	75T
54T	77T	77T
54C	81C	77C
52T	81T	77T
53C	80C	78C
53T	80T	77T
52C	80C	77C
52T	79T	76T
51T	78T	74T
49C	78C	76C
24T_24C	43T_36C	33T_42C
47T	79T	78T
47T	81T	79T
45A	79A	77A
45C	80C	77C
45A	82A	79A
22T_25C	82C	79C
47A	84A	78A
46C	83C	77C
46C	82C	77C
48C	82C	77C
49A	81A	77A
49C	81C	77C
49A	79A	75A
49C	79C	76C
50A	79A	77A
51A	78A	75A
50A	78A	76A
52C	77C	74C
50C	78C	40T_34C
47T	77T	72T
49G	76G	70G

49G	75G	71G
48T	76T	70T
47C	76C	71C
49T	75T	70T
49G	74G	73G
49G	73G	70G
49A	72A	72A
49G	72G	70G
50A	72A	70A
53G	69G	72G
53A	69A	69A
52A	67A	67A
53C	67C	67C
53G	67G	70G
52C	68C	70C
50A	68A	68A
48C	69C	69C
49T	69T	69T
50C	71C	69C
50T	70T	70T
51T	70T	71T
51G	70G	72G
20T_31C	70T	73T
51T	70T	74T
52T	70T	73T
53T	71T	74T
54T	71T	73T
53A	71A	74A
53A	72A	75A
53A	72A	74A
51C	75C	74C
52G	73G	75G
51G	73G	75G
51T	71T	73T
52T	73T	71T
52G	72G	71G
51T	73T	71T
49G	73G	66G
49G	73G	28T_93G_1C
48G	74G	64G
48T	73T	64T
23A_23G	73A	56G
46T	73T	60T

23T_23C	68C	26T_35C
46G	70G	62G
46G	69G	62G
23A_23T	69A	38A_27T
48G	67G	76G
49G	68G	80G
51T	65T	81T
51T	68T	82T
52T	65T	81T
52T	66T	81T
53T	67T	81T
53A	66A	80A
52T	67T	79T
51G	67G	78G
51G	65G	76G
51A	65A	77A
51C	65C	77C
51C	66C	77C
50A	67A	78A
50G	67G	78G
50G	67G	78G
49A	68A	77A
48T	67T	77T
49T	32T_34G	77T
49T	66T	77T
49G	67G	77G
49T	68T	78T
50A	66A	77A
51A_34T_34G_83C	65C	75C
33A	66A	72A
31G	66G	72G
31T	69C	72C
31C	69C	73C
31T	68T	72T
31A	68A	72A
31T	67T	74T
31A	66A	74A
30G	66G	75G
51C	66C	74C
52A	65A	75A
52A	67A	73A
52A	67A	76A
53G	69G	76G

53A	69A	76A
53T	68T	77T
54G	68G	77G
57C	65C	76C
57T	62T	74T
57G	61G	73G
56C	60C	73C
56C	60C	72C
56T	58T	71T
56T	59T	71T
56C	59C	71C
57A	59A	70A
57C	58C	29T_41C
35A_23G	27A_30G	70G
58T	56T	71T
57C	55C	69C
58A	53A	69A
58A	52A	69A
57T	52T	66T
58G	50G	66G
58A	49A	68A
58A	50A	67A
22T_36C	51C	66C
58A	52A	72A
58G	50G	72G
59A	51A	71A
60A	51A	69A
60A	50A	68A
57A	51A	67A
56G	51G	67G
54C	52C	65C
54A	52A	63A
56A	52A	60A
55C	52C	61C
55C	54C	61C
54T	55T	61T
53C	55C	62C
54T	55T	61T
51T	55T	63T
52C	55C	64C
51T	55T	64T
52A	55A	63A
49A	55A	63A

46A	55A	64A
45G	56G	67G
47C	57C	67C
47A	57A	67A
46C	57C	65C
47A	56A	65A
47T	52T	66T
29T_18C	53C	38T_29C
46T	54T	68T
46G	53G	69G
45T	53T	68T
45G	53G	69G
45C	54C	70C
45A	54A	70A
45C	54C	68C
47A	55A	70A
46T	54T	71T
45C	52C	71C
46T	52T	70T
46G	49G	70G
46T	51T	70T
46G	49G	68G
45C	49C	69C
45A	50A	69A
46G	50G	70G
47G	50G	67G
47T	50T	68T
47G	50G	67G
47T	50T	65T
47T	49T	65T
47T	46T	70T
48G	45G	73G
48C	46C	71C
48A	45A	72A
48T	44T	73T
46T	44T	73T
46C	44C	74C
46A	24A_20T	72A
46G	44G	73G
38A_45T_38C	57A_43T_57C	25A_74T_25C
21A_46C	46C	77C
26A	46A	76A
45C	46C	77C

46A	45A	78A
45C	44C	80C
45A	44A	101A_29G_50C
46C	43C	81C
46A	43A	79A
46C	44C	79C
47A	43A	72A
48C	43C	78C
48A	42A	76A
47C	40C	75C
48A	40A	73A
48C	40C	75C
48A	40A	75A
50C	39C	78C
47G	33G	70G
46C	36C	72C
47A	35A	71A
48G	36G	71G
49A	36A	67A
50A	36A	70A
50G	34G	70G
50C	34C	67C
49C	34C	68C
49T	34T	69T
49A	34A	68A
48G	34G	68G
49A	32A	68A
50A	34A	68A
51A	34A	71A
51G	32G	70G
51G	32G	69G
48G	33G	69G
49T	34T	67T
49G	34G	66G
49G	33G	66G
49G	35G	68G
50C	35C	68C
49T	36T	34T_36G
51T	36T	69T
50G	36G	68G
51T	36T	68T
51A	36A	69A
51G	36G	67G

51A	36A	67A
52A	36A	67A
25T_28C	38C	69C
53T	39T	69T
52G	39G	70G
52A	39A	68A
51T	38T	70T
53T	37T	69T
53C	39C	70C
54C	38C	69C
53A	40A	68A
52A	39A	66A
53A	38A	63A
52G	38G	60G
52G	39G	60G
52G	39G	60G
51T	40T	61T
52G	39G	62G
51G	40G	63G
52G	39G	61G
53A	40A	63A
53G	40G	64G
54C	39C	67C
54A	36A	68A
54A	36A	68A
52A	37A	69A
52A	38A	69A
53C	38C	68G
54A	37A	68A
53G	37G	68G
52T	38T	67T
53C	37C	69C
53A	37A	69A
53G	39G	69G
53G	39G	67G
53G	39G	67G
52C	39C	70C
52T	40T	71T
53T	42T	69T
54T	43T	68T
54T	42T	69T
52A	43A	70A
52A	44A	71A

53A	44A	69A
53G	44G	73G
53C	44C	74C
53T	46T	73T
53G	45G	73G
52G	45G	74G
53T	44T	75T
53C	45C	78C
53C	45C	77C
54T	44T	75T
53A	44A	75A
52A	45A	76A
52A	45A	73A
25A_27G	45G	72G
51A	45A	73A
53C	46C	76C
53C	47C	79C
50G	46G	79G
50G	46G	77G
51G	46G	77G
50A	46A	78A
52G	44G	75G
52G	45G	76G
52A	47A	76A
53A	47A	76A
53T	48T	75T
52C	46C	77C
52G	45G	77G
51T	47T	76T
50C	46C	73C
51C	46C	75C
51A	46A	74A
49A	44A	72A
49C	46C	71C
47T	46T	74T
49C	46C	74C
49C	46C	75C
48C	46C	77C
48A	48A	78A
48T	47T	80T
47C	47C	80C
47A	44A	80A
50C	48C	80C

50C	49C	77C
49C	50C	77C
49T	50T	77T
48C	50C	77C
46A	50A	75A
46C	50C	75C
46C	50C	73C
46A	50A	72A
45G	48G	73G
44C	48C	73C
43C	50C	74C
42A	50A	74A
42T	54T	72T
42G	55G	72G
42T	55T	72T
42T	56T	70T
42G	56G	72G
42G	58G	71G
41C	58C	71C
40T	58T	72T
40G	58G	69G
42A	58A	68A
42G	57G	69G
40G	58G	71G
40A	58A	69A
39C	33T_26C	34T_34C
39G	58G	69G
39A	58A	71A
39T	57T	69T
36G	57G	69G
37G	57G	69G
36G	57G	71G
38A	57A	68A
38G	57G	69G
40C	57C	65C
40T	57T	66T
40G	55G	68G
41T	54T	66T
42A	54A	66A
42G	54G	65G
42T	56T	66T
40G	55G	65G
40A	56A	63A

39G	56G	61G
39A	55A	64A
39A	58A	65A
41C	58C	67C
41T	59T	65T
42T	58T	65T
42C	60C	64C
44G	60G	36A_28G
44G	60G	65G
44C	61C	66C
44A	60A	67A
44G	60G	67G
44G	61G	68G
44G	61G	70G
42T	60T	68T
44G	61G	69G
43C	61C	70C
43G	61G	73G
42A	61A	71A
43G	62G	71G
43G	62G	70G
42T	61T	69T
42T	62T	70T
43C	62C	69C
43A	61A	70A
43C	61C	75C
44C	62C	74C
44C	62C	75C
44A	61A	77A
43A	59A	77A
42G	58G	77G
41C	57C	77C
42G	55G	76G
42G	56G	76G
40C	56C	77C
40T	56T	79T
41T	56T	77T
41C	54C	79C
41A	54A	79A
41A	54A	80A
41A	54A	80A
41A	53A	80A
41C	54C	83C

42G	54G	81G
42A	55A	81A
42A	54A	82A
42A	54A	81A
43A	54A	82A
44C	52C	81C
22T_21C	54C	80C
43C	54C	78C
43A	54A	80A
43A	56A	80A
42T	58T	37T_43C
43G	59G	80G
22T_20C	58C	80C
42A	59A	79A
43A	57A	81A
41C	57C	80C
42A	56A	80A
42C	56C	80C
42A	56A	81A
42G	56G	79G
43A	56A	78A
44G	57G	80G
45A	59A	79A
45G	60G	80G
44G	60G	80G
45A	26A_34G	79G
46A	62A	81A
46A	63A	80A
45C	64C	80C
46A	62A	78A
46T	62T	76T
46T	34T_28C	76T
44T	62T	78T
43G	61G	76G
43T	60T	75T
43A	61A	73A
43C	61C	74C
43A	60A	72A
43A	59A	72A
43A	59A	73A
43G	58G	71G
43A	60A	71A
43A	61A	70A

20A_23G	63G	71G
43T	62T	69T
43C	61C	69C
43T	57T	69T
44G	59G	69G
45G	59G	68G
45A	59A	66A
45G	60G	66G
45G	58G	66G
45G	57G	62G
46A	58A	62A
46T	58T	61T
44C	63C	60C
44C	62C	62C
45C	61C	61C
45A	61A	62A
46A	60A	63A
47A	61A	62A
47G	61G	65G
46G	60G	61G
47C	60C	61C
48A	61A	59A
48G	62G	60G
47G	63G	58G
47G	64G	57G
45C	64C	58C
44C	64C	57C
42C	66C	57C
42T	66T	57T
42A	66A	33A_26G
41T	66T	59T
41T	65T	59T
41T	65T	59T
42G	64G	59G
43C	31G_33C	60C
43T	65T	58T
51A	62A	57A
48A	60A	57A
48G	61G	56G
47G	61G	55G
33C	59C	55C
32T	59T	53T
32C	59C	54C

30A	59A	53A
31A	59A	54A
23A_1G_8C	58A	53A
32A	61A	56A
32A	64A	55A
32G	64G	53G
32A	63A	54A
32G	64G	53G
33G	64G	53G
34A	64A	53A
33G	63G	53G
33C	64C	54C
34T	64T	53T
34G	63G	54G
34C	63C	55C
35A	63A	55A
35G	62G	55G
35G	62G	55G
35A	61A	57A
36G	61G	59G
36A	61A	57A
36C	62C	59C
36A	62A	59A
36A	62A	57A
38A	60A	58A
39A	62A	60A
39C	61C	60C
39A	61A	60A
38G	62G	59G
36A	63A	58A
38G	63G	58G
38A	64A	58A
38G	65G	60G
38A	64A	61A
38A	64A	61A
38T	63T	58T
38C	64C	58C
38C	64C	57C
38A	66A	57A
40A	66A	57A
40A	66A	54A
41G	65G	56G
41G	66G	56G

40T	64T	55T
41A	65A	55A
41C	64C	50C
41A	64A	52A
41C	65C	51C
40A	65A	52A
43A	65A	52A
42T	66T	54T
43A	66A	55A
42A	66A	56A
43T	65T	55T
43A	64A	58A
42G	64G	59G
41C	63C	56C
41A	62A	55A
41T	62T	55T
41C	62C	55C
40C	60C	56C
39C	59C	56C
40A	57A	56A
40A	59A	57A
39C	61C	56C
39T	60T	56T
39C	60C	56C
41T	59T	57T
41C	60C	56C
41A	59A	59A
41G	59G	59G
41C	59C	59C
41G	59G	58G
42G	58G	57G
42A	58A	59A
42G	56G	59G
42A	56A	59A
42C	54C	62C
42T	54T	61T
42C	57C	61C
42T	56T	60T
42G	57G	59G
42G	58G	59G
42G	57G	57G
42A	57A	61A
42A	58A	61A

42G	58G	59G
41A	59A	59A
41G	24A_34G	34A_24G
40C	53C	58C
39G	54G	59G
38T	55T	56T
38G	55G	57G
38T	54T	58T
37G	53G	57G
36C	54C	58C
37T	53T	58T
37T	53T	57T
37C	53C	56C
37G	53G	58G
37A	51A	57A
40A	52A	58A
39G	53G	57G
39C	54C	55C
40A	52A	54A
40G	51G	54G
40A	52A	54A
41G	52G	55G
42T	50T	54T
42T	51T	56T
41T	53T	59T
40A	54A	57A
40A	54A	58A
38A	57A	59A
39A	61A	60A
39A	61A	60A
39G	62G	61G
39A	62A	63A
39A	61A	62A
38C	61C	61C
38A	60G	59G
39G	60G	59G
38A	59A	59A
38G	55G	58G
37A	54A	55A
38G	54G	53G
38G	55G	53G
38A	55A	54A
37G	56G	55G

36G	55G	55G
36A	55A	57A
36C	27G_29C	25G_33C
36A	56A	60A
36G	57G	59G
37A	57A	59A
37A	57A	59A
39A	57A	60A
38G	57G	60G
39A	58A	62A
38C	58C	58C
38T	59T	57T
38A	58A	57A
38A	58A	57A
38A	58A	57A
36A	57A	55A
35G	57G	54G
36G	57G	50G
37G	57G	49G
38A	56A	51A
38G	56G	51G
36A	55A	53A
36G	55G	54G
36C	55C	54C
36T	55T	55T
36C	54C	51C
35G	54G	51G
37C	53C	51C
38T	51T	50T
38G	51G	50G
36G	51G	48G
37A	51A	47A
37A	51A	47A
37T	50T	48T
37T	50T	47T
37A	46A	48A
36G	47G	46G
37A	48A	46A
20G_17C	47G	22G_23C
38T	48T	45T
35G	48G	45G
35T	46T	45T
35A	48A	44A

42A	49A	54A
41G	50G	54G
42C	51C	54C
42C	51C	53C
42A	51A	66A
71A	53A	76A
31A	53A	47A
31A	53A	46A
31A	53A	44A
31C	53C	43C
42A	55A	51A
42A	53A	50A
41A	54A	49A
43A	56A	51A
43C	55C	50C
42A	57A	48A
43A	57A	49A
42A	57A	48A
43A	58A	49A
44C	59C	50C
44A	139A	50A
43A	42A	51A
45A	42A	53A
45A	38A	53A
46C	38C	52C
46A	38A	51A
46A	57A	52A
46A	56A	52A
45A	55A	45A
45C	55C	44C
39A	55A	34A
39A	56A	34A
39A	57A	34A
38G	57G	28G
38G	57G	28G
39A	57A	28A
42A	57A	29A
42A	59A	29A
44A	56A	32A
46A	55A	33A
46A	57A	34A
46C	57C	35C
46A	58A	35A

45T	58T	35T
45A	59A	35A
47T	59T	35T
46A	60A	36A
47G	62G	38G
48G	63G	39G
49C	63C	41C
49T	64T	42T
49C	66C	41C
48A	67A	42A
48G	68G	41G
48C	68C	40C
49T	69T	38T
50C	68C	40C
50T	67T	43T
50G	67G	43G
52A	68A	45A
51A	69A	45A
51G	70G	45G
26G_25C	69C	44C
25T_27C	69T	43T
52C	66C	46C
54A	68A	46A
55A	69A	46A
55A	67A	46A
55G	65G	46G
55G	65G	48G
55G	65G	47G
56C	65C	48C
56A	65A	48A
55A	65A	48A
55A	66A	48A
55G	65G	49G
55A	65A	48A
55G	67G	47G
55A	66A	47A
55A	66A	48A
55A	64A	48A
56G	65G	49G
56A	65A	49A
56T	65T	47T
56C	65C	48C
56A	64A	49A

57T	63T	47T
56C	62C	48C
56C	63C	46C
56T	63T	45T
56A	65A	46A
30A_25G	65G	19A_28G
57G	64G	46G
57A	65A	47A
56T	65T	48T
56G	65G	48G
56T	65T	47T
57C	66C	47C
57C	65C	47C
57C	66C	47C
57C	65C	45C
56A	65A	47A
59A	64A	47A
60A	65A	48A
60A	66A	50A
60A	65A	50A
60C	64C	50C
60T	64T	50T
59G	63G	47G
58A	63A	49A
56G	64G	49G
56C	64C	46C
56A	63A	48A
58C	65C	48C
58T	65T	48T
58C	65C	51C
57A	66A	50A
58A	66A	49A
58G	66G	49G
59C	65C	47C
59A	65A	45A
59A	64A	48A
58A	63A	47A
56G	63G	47G
57G	62G	46G
57G	62G	49G
56G	63G	47G
27T_29G	31T_33G	1A_22T_23G
56T	63T	46T

56T	65T	44T
57T	67T	44T
58T	67T	43T
58T	69T	45T
58T	65T	43T
58G	67G	43G
57A	66A	42A
57G	67G	42G
57A	65A	41A
56C	67C	42C
55C	67C	41C
55C	66C	41C
55C	66C	40C
53T	65T	40T
53G	65G	41G
52G	65G	41G
54C	33A_30C	40C
54G	63G	41G
55G	66G	41G
55G	66G	41G
54A	65A	40A
54G	66G	42G
54A	65A	42A
54T	64T	43T
54A	63A	44A
54G	65G	42G
54A	65A	41A
51C	65C	41C
51A	61A	42A
49A	63A	42A
47A	63A	42A
47A	61A	41A
46G	61G	40G
46G	61G	39G
46G	62G	38G
46A	61A	37A
45T	60T	37T
46G	60G	38G
45G	59G	38G
43G	59G	38G
42A	59A	37A
43G	55G	39G
43C	57C	37C

44A	56A	37A
44T	54T	37T
43C	56C	35C
43C	55C	35C
42T	55T	34T
42T	56T	34T
42C	58C	34C
40C	57C	30C
40C	55C	30C
40A	55A	31A
16A_24G	55G	18A_13G
38C	56C	31C
40C	57C	30C
39A	55A	28A
38C	55C	28C
37A	54A	27A
38G	54G	26G
38A	54A	26A
38G	53G	26G
37G	56G	25G
38A	56A	25A
38A	55A	25A
37A	55A	25A
38T	27T_28C	23T
38T	54T	21T
38A	53A	20A
38A	54A	19A
38G	54G	19G
38G	53G	18G
37A_31G	53A	18A_29G
38G	51G	20G
37G	53G	20G
36G	55G	21G
36G	55G	22G
35G	54G	22G
35G	54G	22G
35G	52G	22G
33C	53C	21C
33A	52A	23A
33T	54T	24T
33A	54A	25A
34A	54A	25A
34T	54T	24T

34T	53T	25T
33T	54T	26T
33T	54T	26T
35C	54C	25C
35C	53C	24C
35C	55C	10T_13C
33T	54T	22T
32T	55T	23T
32A	55A	23A
32A	55A	23A
32G	56G	22G
32T	55T	22T
33G	56G	24G
33G	57G	23G
33T	57T	23T
33T	58T	23T
32G	59G	23G
31T	62T	25T
31G	63G	25G
31C	63C	24C
31C	64C	24C
31C	64C	24C
30A	65A	24A
30A	65A	24A
30G	65G	25G
31C	65C	26C
31T	64T	25T
32T	64T	25T
32T	64T	26T
32C	66C	27C
32C	66C	27C
33C	66C	27C
33C	65C	29C
33C	62C	29C
31T	64T	28T
33A	62A	28A
33T	61T	29T
33T	60T	29T
33T	58T	29T
33T	57T	30T
32T	57T	30T
32A	56A	29A
32T	56T	30T

32A	55A	30A
32G	54G	28G
32G	55G	27G
32C	55C	28C
32C	53C	28C
31A	54A	27A
30G	54G	28G
29A	55A	28A
29A	57A	28A
29G	58G	28G
29C	58C	28C
29G	58G	28G
29G	59G	28G
29A	27A_32T	26A
29C	59C	26C
29T	60T	26T
28G	60G	26G
29T	61T	25T
29T	60T	24T
30C	61C	24C
31A	60A	23A
31G	61G	23G
31G	61G	21G
31T	61T	23T
31A	60A	22A
31G	60G	23G
30T	61T	23T
29G	61G	22G
30A	61A	21A
30T	61T	21T
30G	61G	21G
30A	58A	23A
30T	58T	24T
29G	60G	24G
29A	60A	23A
28T	61T	23T
27G	60G	24G
26G	60G	25G
26C	60C	25C
26A	60A	25A
26G	1T_33G_25C	25G
26G	59G	24G
27A	57A	24A

27T	57T	24T
27G	54G	22G
26C	54C	22C
9A_25T	53T	8A_21T_2G_1C
22G	54G	22G
23C	27G_81C	22C
15G_10C	24G	10G_25C
25C	26C	24C
25C	56C	24C
26C	56C	24C
25C	56C	24C
25C	55C	24C
26C	54C	23C
26C	55C	22C
25C	55C	23C
25T	55T	19T
25T	55T	21T
25T	55T	22T
25T	55T	22T
25C	55C	22C
25A	54A	25A
24G	55G	27G
24G	53G	26G
23A	54A	27A
23G	54G	29G
23G	55G	29G
22C	54C	29C
23A	55A	30A
23A	54A	31A
23C	54C	31C
25C	57C	31C
25C	57C	35C
24A	57A	35A
23C	31T_25C	35C
24C	57C	35C
24C	56C	35C
23C	32T_25C	35C
24T	58T	37T
24T	58T	38T
24T	57T	40T
23G	58G	40G
23C	57C	39C
23C	58C	39C

23C	58C	40C
22T	55T	39T
22G	58G	39G
22A	58A	41A
22A	58A	43A
23C	58C	41C
23T	59T	43T
22G	59G	44G
21C	57C	45C
22T	56T	45T
21T	54T	46T
22C	52C	47C
22C	53C	48C
24T	53T	47T
25G	53G	47G
25A	51A	46A
25G	51G	47G
26A	53A	48A
25A	55A	48A
25T	54T	47T
25C	54C	47C
26G	30A_26G	48G
25G	55G	46G
25C	53C	46C
26C	55C	46C
23T	55T	46T
26C	55C	49C
26C	54C	48C
27A	55A	48A
27G	54G	48G
28A	55A	48A
31C	56C	49C
32A	56A	48A
32A	56A	48A
31T	56T	47T
32C	58C	47C
32T	58T	47T
29T	56T	47T
32C	58C	47C
32C	56C	48C
32A	55A	48A
32A	53A	48A
32G	54G	48G

31G	53G	46G
32C	52C	46C
32A	51A	46A
33G	50G	45G
33A	27A_22C	18A_26C
33C	50C	42C
33G	50G	42G
32C	50C	41C
31A	48A	39A
32T	27T_1G_21C	20T_18C
32G	49G	39G
31G	47G	41G
31A	47A	40A
32G	47G	43G
32C	48C	44C
32C	47C	40C
32A	48A	41A
32A	47A	17A_24T
32G	48G	41G
32C	48C	42C
32C	46C	44C
32T	47T	42T
31C	49C	42C
31A	48A	40A
32C	49C	40C
34T	50T	41T
35C	51C	42C
35T	50T	42T
35T	50T	43T
36T	49T	44T
36C	49C	42C
36C	50C	41C
36A	47A	41A
35A	51A	39A
35A	51A	39A
36T	51T	38T
26A_61G	51G	39G
35G	44A_73G	38G
35G	29G	38G
35A	26A	37A
34A	26A	37A
34G	49G	38G
34G	49G	40G

34A	50A	39A
35A	51A	38A
35G	53G	40G
34G	54G	41G
35A	54A	41A
36A	54A	40A
36G	53G	40G
36G	54G	41G
36A	56A	41A
35A	56A	41A
38G	56G	39G
38G	56G	39G
38C	57C	39C
38C	58C	39C
38A	58A	40A
38A	58A	38A
38C	59C	39C
38C	61C	38C
38T	61T	40T
40A	62A	41A
42C	61C	42C
40A	62A	42A
39A	61A	42A
39A	61A	41A
39G	61G	18A_23G_1C
38C	63C	42C
38C	64C	41C
38C	64C	42C
37T	64T	42T
38T	62T	42T
38C	63C	43C
38C	65C	42C
39A	64A	42A
39G	63G	42G
40T	64T	41T
40C	63C	42C
40C	64C	42C
40C	64C	24G_18C
39T	63T	40T
38C	62C	39C
37T	62T	36T
38G	62G	36G
39A	61A	35A

39G	62G	36G
38A	61A	36A
39A	62A	36A
39C	61C	35C
36A	63A	33A
36G	63G	36G
37G	63G	36G
36G	62G	36G
37A	61A	35A
37C	61C	39C
36C	61C	38C
37A	60A	37A
37G	57G	38G
37A	56A	37A
36A	56A	36A
35A	56A	36A
35G	57G	36G
33A	57A	36A
32G	56G	35G
30A	56A	34A
30A	55A	33A
31G	54G	33G
30G	54G	31G
30C	53C	30C
30G	54G	30G
30C	55C	29C
30T	55T	26T
30A	56A	28A
30A	56A	27A
30G	57G	26G
30C	56C	25C
30C	56C	25C
30A	57A	26A
28A	58A	26A
29G	59G	26G
29G	59G	25G
29A	59A	25A
29A	58A	25A
30C	58C	25C
30A	57A	26A
30G	57G	26G
29C	57C	26C
30C	57C	25C

28A	57A	25A
28G_7C	53G_56C	25G_10C
30C	3C	23C
30C	58C	23C
30C	59C	22C
30C	58C	22C
23A_53C	59C	14A_36C
31C	58C	22C
30C	58C	21C
31C	58C	20C
27A	58A	20A
27A	59A	20A
28T	60T	20T
28G	59G	21G
26C	59C	20C
27A	58A	22A
29G	58G	24G
29A	59A	24A
28A	60A	24A
27G	60G	23G
28A	60A	23A
28G	61G	24G
28C	60C	25C
28A	58A	26A
28G	59G	26G
28G	58G	25G
28G	58G	27G
28T	57T	27T
29T	57T	28T
30G	59G	29G
30T	59T	26T
29T	61T	24T
29G	61G	27G
29G	63G	27G
29C	62C	26C
30A	62A	11A_16T
29C	61C	27C
28C	59C	26C
29C	57C	26C
29A	57A	26A
30C	58C	12T_16C
31G	32A_26G	28G
29A	24A_32G	28A

28T	58T	29T
29A	59A	30A
29T	59T	31T
29C	59C	33C
29G	60G	33G
29G	59G	34G
29C	58C	35C
28C	60C	37C
28T	60T	37T
26G	60G	37G
26C	59C	39C
7A_19G	35A_22G_1C	19A_19G
26G	57G	38G
25C	57C	39C
26T	55T	37T
27T	55T	40T
28C	56C	43C
28T	55T	21T_1G_22C
29T	54T	44T
29C	19T_33C	45C
28T	53T	45T
30G	52G	46G
31A	52A	46A
29G	52G	46G
30G	51G	47G
30G	52G	48G
30C	51C	49C
30G	51G	50G
30G	51G	50G
30A	51A	51A
30C	50C	27T_26C
29G	51G	53G
29C	51C	53C
29C	50C	53C
28A	51A	53A
28A	51A	54A
28G	50G	55G
27G	51G	55G
27A	51A	56A
26T	53T	55T
26C	52C	54C
27C	53C	55C
27T	53T	54T

27G	53G	53G
26G	54G	55G
26G	55G	57G
26G	57G	57G
26C	57C	59C
27C	57C	60C
27A	58A	59A
27A	57A	59A
27C	58C	62C
27C	58C	63C
26G	58G	61G
26C	58C	61C
16T_10C	21T_37C	28T_33C
26T	58T	60T
26T	59T	60T
26G	58G	61G
26G	58G	59G
27A	58A	55A
27G	57G	56G
27G	56G	54G
28C	56C	54C
28A	56A	54A
27G	55G	55G
27G	55G	56G
26G	56G	55G
28C	55C	55C
28A	55G	55G
27G	54G	55G
28C	55C	55C
28C	55C	54C
28G	55G	55G
29T	55T	54T
29G	55G	55G
29A	55A	56A
29G	53G	54G
29G	53G	55G
29C	54C	54C
29A	54A	51A
29C	53C	52C
29T	53T	53T
28G	52G	53G
28C	52C	54C
27A	52A	52A

28C	52C	51C
28C	52C	50C
28C	52C	49C
27C	51C	49C
27C	53C	49C
26T	53T	48T
26T	52T	46T
24A	50A	48A
24C	51C	48C
25C	51C	48C
24C	50C	47C
25T	50T	46T
25G	51G	48G
25T	52T	46T
25G	52G	45G
24C	53C	45C
24C	53C	45C
25G	53G	45G
25T	54T	47T
24C	54C	47C
24T	52T	46T
25T	52T	46T
25G	50G	45G
25C	51C	45C
25C	51C	45C
26C	51C	44C
25C	51C	44C
25C	51C	43C
12T_13C	51T	41T
24C	51C	40C
24C	50C	41C
22A	49A	40A
22G	47G	39G
22G	48G	39G
22G	47G	39G
22C	46C	36C
22C	46C	35C
25C	46C	36C
25A	45A	36A
13A_12G	45A	35A
25T	46T	35T
27C	45C	36C
26C	46C	35C

26T	46T	36T
13T_13C	47C	36C
26A	46A	37A
26A	46A	39A
26T	45T	39T
25C	45C	39C
25C	45C	39C
24C	44C	38C
23A	40A	39A
24C	44C	40C
24C	44C	44C
20A	41A	45A
22C	44C	44C
21C	45C	45C
21C	46C	42C
22A	46A	43A
22C	45C	43C
22G	45G	41G
22C	45C	42C
22C	45C	42C
22T	47T	41T
22G	47G	42G
22C	48C	42C
22C	49C	42C
22C	49C	41C
23C	48C	42C
22A	48A	40A
24G	48G	42G
24T	48T	42T
24C	46C	42C
25T	46T	42T
25C	45C	43C
24T	44T	42T
23C	44C	43C
23T	44T	42T
23A	44A	41A
24C	44C	40C
24T	42T	40T
24A	41A	40A
23C	41C	38C
23C	40C	38C
23C	40C	38C
23A	40A	41A

24G	40G	42G
15A_10G	21A_19G	16A_26G
25A	40A	42A
26G	41G	41G
26G	42G	44G
26G	42G	45G
26A	42A	45A
25C	42C	47C
26G	42G	47G
26G	42G	47G
26G	42G	47G
25A	41A_20G	47A_15G
27G	41G	54G
27G	41G	53G
27G	41G	55G
28G	41G	54G
28G	41G	53G
30G	41G	54G
31G	39G	56G
29A	41A	54A
29G	42G	56G
30A	42A	56A
31G	43G	57G
31G	43G	62G
31G	43G	62G
31G	45G	61G
31C	45C	62C
32C	45C	61C
32C	46C	62C
33T	47T	61T
32T	47T	61T
34G	47G	62G
34T	50T	62T
34G	50G	63G
36C	52C	64C
37G	53G	64G
37C	53C	64C
38A	51A	61A
38T	52T	29A_31T
39G	52G	60G
39C	52C	59C
41G	52G	60G
42C	51C	61C

41A	51A	60A
41A	48A	61A
41C	47C	62C
41T	48T	61T
43C	47C	63C
43A	47A	63A
43A	48A	63A
43G	48G	63G
42T	48T	63T
42C	47C	63C
43C	46C	62C
42G	47G	63G
41C	49C	63C
41T	47T	65T
41T	46T	67T
42G	46G	69G
41G	46G	68G
42C	46C	68C
42A	45A	68A
42T	46T	66T
43G	46G	67G
43G	47G	68G
44A	47A	70A
44G	48G	68G
44C	48C	70C
44A	49A	70A
45T	48T	67T
46T	48T	67T
43T	49T	69T
43C	49C	68C
43G	49G	68G
44A	49A	68A
42T	47T	66T
42G	45G	66G
43A	46A	65A
43G	46G	66G
44C	46C	66C
44A	47A	66A
44A	47A	67A
45A	47A	68A
45T	47T	69T
46T	47T	66T
46G	48G	66G

47T	45T	64T
46T	47T	64T
47G	47G	63G
47C	45C	62C
47A	45A	63A
47A	45A	63A
46G	45G	64G
46G	45G	59G
46A	46A	59A
47A	46A	60A
46C	46C	59C
46C	45C	59C
44T	45T	60T
46C	43C	62C
48T	44T	63T
49C	44C	62C
48C	45C	61C
47G	45G	61G
47C	46C	60C
49T	47T	59T
49C	46C	59C
49A	46A	59A
50G	47G	59G
50G	46G	60G
50T	46T	60T
50G	45G	57G
50C	45C	59C
49T	45T	59T
49T	45T	58T
50G	44G	57G
51T	45T	57T
50G	48G	55G
52G	49G	55G
52T	49T	55T
50A	49A	57A
50C	49C	57C
49A	51A	59A
48G	49G	58G
48G	49G	56G
49T	49T	57T
48A	45A	56A
49C	49C	54C
49T	49T	51T

49C	49C	51C
49C	49C	51C
49T	50T	54T
49C	50C	54C
48G	50G	55G
48G	50G	54G
47C	51C	52C
47C	50C	52C
45T	50T	50T
43G	50G	51G
44C	48C	51C
44A	48A	49A
43T	48T	48T
42G	47G	48G
42C	47C	46C
43T	46T	47T
43G	45G	48G
42A	46A	47A
41G	47G	47G
41G	47G	47G
42G	46G	48G
41A	46A	48A
40C	46C	47C
40C	44C	47C
40G	44G	48G
38G	42G	48G
37A	41A	49A
37T	42T	50T
36G	41G	51G
37T	41T	50T
36C	41C	51C
37G	41G	51G
36G	42G	53G
35C	42C	60C
35C	42C	60C
35A	42A	60A
35G	42G	60G
35C	42C	61C
33C	41C	61C
33G	41G	58G
33G	40G	56G
33A	40A	55A
33G	40G	58G

34G	40G	56G
33A	39A	58A
33G	38G	57G
33C	37C	57C
34A	37A	59A
34G	38G	59G
34C	36C	61C
37T	37T	62T
36G	38G	63G
36C	35C	63C
36C	37C	63C
36G	36G	63G
36G	36G	64G
36A	37A	66A
37A	36A	69A
37T	38T	69T
38T	37T	72T
37T	37T	71T
36G	37G	69G
36T	36T	68T
36C	37C	67C
36C	37C	68C
35G	37G	71G
36T	37T	70T
36G	37G	71G
35G	36G	71G
35T	36T	70T
34G	37G	70G
35T	35T	70T
35G	37G	67G
34A	38A	67A
34G	38G	67G
33G	39G	68G
34A	39A	70A
35T	41T	71T
35A	41A	73A
34G	41G	72G
33T	42T	74T
34G	41G	74G
34G	41G	73G
34C	40C	74C
34A	41A	74A
35G	40G	74G

34A	41A	72A
34T	41T	74T
34C	20T_21C	76C
34G	41G	78G
34T	41T	76T
32G	41G	76G
32T	41T	76T
33A	40A	76A
34C	40C	74C
34T	39T	73T
32C	38C	73C
32C	38C	73C
33A	38A	72A
33G	37G	73G
33C	37C	73C
33A	37A	76A
33G	37G	74G
33G	36G	67G
33G	35G	68G
33C	35C	68C
32G	36G	69G
31G	36G	69G
31C	34C	71C
27A	34G	72G
32T	34T	71T
33T	34T	72T
33G	33G	70G
33G	31G	74G
33C	31C	73C
33T	31T	74T
32T	30T	75T
32T	30T	75T
32C	31C	75C
33T	31T	76T
33C	32C	74C
33C	33C	72C
33T	33T	77T
33G	34G	76G
33G	34G	77G
33G	34G	77G
33C	33C	76C
33G	33G	73G
33T	33T	73T

34C	33C	72C
34T	33T	72T
34T	33T	72T
34T	34T	73T
34C	34C	74C
34A	34A	74A
33C	34C	72C
26A_7G	34G	73G
33A	34A	71A
33G	33G	71G
33G	31G	71G
32C	31C	71C
32T	31T	72T
32C	31C	72C
32G	30G	72G
33A	30A	71A
33G	30G	70G
33T	29T	68T
33T	29T	69T
33C	29C	67C
33T	28T	66T
34C	28C	66C
33C	28C	67C
32A	29A	67A
32C	29C	67C
31G	29G	68G
32T	29T	68T
31A	29A	69A
31T	28T	68T
31T	28T	67T
34T	29T	65T
34G	29G	66G
34A	29A	67A
34C	30C	68C
33A	29A	68A
33T	30T	70T
33C	31C	71C
33T	31T	72T
32G	31G	71G
32C	30C	72C
31A	30A	71A
28A	29A	69A
28C	26C	30T_36C

28G	26G	65G
28A	26A	66A
27A	26A	66A
27A	26A	64A
48A_24T_48G_2C	40A_20T_40G_2C	54A_27T_85G_2C
1A	3A	32A
1A	1A	30A
null	1G	30G
null	1G	30G
24C	22C	28A_1G_28C
29A	24A	65A
30A	23A	62A
30G	23G	63G
30G	23G	63G
31G	23G	63G
31A	23A	64A
31G	23G	65G
31A	24A	64A
31G	24G	64G
31G	24G	65G
31C	25C	62C
32G	25G	62G
32G	25G	62G
31A	23A	62A
31G	23G	61G
29T	24T	60T
31G	23G	60G
30A	23A	60A
29G	22G	61G
27G	22G	61G
24C	22C	61C
25C	23C	62C
25T	23T	62T
25G	23G	63G
26A	23A	61A
26C	23C	62C
27A	23A	61A
27G	23G	63G
26G	23G	63G
27C	22C	25T_37C
27G	22G	62G
26A	22A	65A
27G	22G	66G

27A	22A	66A
27G	23G	66G
27G	23G	65G
27C	23C	66C
26G	23G	66G
26G	23G	66G
26G	23G	68G
25C	12A_11C	67C
25G	24G	68G
25G	24G	68G
20T_5G	22T	65T
26T	25T	63T
26T	25T	62T
26C	25C	61C
27C	26C	61C
28T	26T	60T
27G	25G	61G
27T	25T	60T
27A	24A	61A
8T_17C	11T_12C	61C
25G	23G	60G
25C	23C	23T_36C
25C	23C	57C
26A	23A	58A
26T	23T	58T
26C	23C	58C
26C	23C	56C
26A	23A	56A
27G	22G	54G
26G	22G	53G
27T	21T	53T
28G	10A_10G	50G
27G	20G	47G
26T	19T	47T
28T	19T	46T
28T	19T	46T
28T	19T	45T
27T	19T	45T
27A	19A	44A
26A	19A	43A
25T	18T	42T
25T	19T	42T
25C	18C	38C

25T	18T	37T
26A	17A	37A
26A	17A	37A
26G	17G	36G
27A	16A	36A
27A	16A	35A
14C	null	5A_8G_13C
14A_14G	null	4G
15A	null	4A
15A	null	4A
15A	null	4A
14A	null	4A
14A	null	3A
14A	null	1A
14A	null	null
14G	null	null
14A	null	null
14G	null	null
14C	null	null
14A	null	null
14T	null	null
14C	null	null
14T	null	null
14T	null	null
14T	null	null
14T	null	null
14C	null	null
15C	null	null
15T	null	null
15G	null	null
15A	null	null
15G	null	null
15G	null	null
15A	2A	null
15G	2G	null
14G	2G	4G
14G	2G	4G
15G	2G	4G
15A	2A	6A
16A	2A	6A
16A	2A	6A
15A	2A	6A
15A	2A	6A

15A	2A	6A
15G	2G	6G
15G	2G	6G
15G	2G	6G
15G	2G	6G
15A	2A	6A
15G	2G	6G
15T	2T	6T
15G	2G	6G
15T	2T	6T
15G	2G	6G
15G	2G	6G
15A	2A	6A
15A	2A	6A
15C	2C	6C
15T	null	null
15A	null	null
15G	1G	2G
15T	1T	2T
15A	1A	2A
15T	1T	2T
17A	2A	2A
17G	2G	3G
17A	2A	3A
17C	2C	4C
17C	2C	4C
17A	2A	4A
17G	1T_1G	4G
17A	2A	4A
16C	2C	4C
16G	2G	4G
16G	2G	4G
16G	2G	4G
17C	2C	4C
16A	2G	4G
17G	1T_1G	4G
17G	2G	4G
17A	2A	5A
17T	2T	5T
17A	2A	5A
17C	2C	5C
17A	2A	5A
17A	2A	5A

16A	2A	5A
16T	2T	5T
16C	2C	5C
16A	2A	5A
15A	2A	5A
14A	2A	5A
14T	2T	5T
13A	2A	5A
14A	2A	5A
14A	2A	5A
14T	2T	5T
14A	2A	5A
14A	2A	5A
13A	2A	5A
13T	2T	5T
13A	2A	5A
13A	2A	5A
12A	2A	5A
12T	2T	5T
12A	2A	5A
11A	2A	5A
10A	2A	1A_4G
10T	2T	5T
9A	2A	5A
9A	2A	5A
10A	2A	5A
10T	2T	5T
9A	2A	5A
11A	2A	5A
26A_12T	2G	5A
5T	2T	1T
8A	2A	1A
8A	2A	1A
8A	2A	1A
8T	2T	1T
13A	2A	1A
12A	2A	1A
12A	2A	1A
5A_7T	null	1T
16A	null	1A
6T	null	1A
12A	null	1A
13A	null	1A

13A	null	1A
13T	null	null
13A	null	null
13A	null	null
13A	null	null
14T	null	null
14A	1A	1A
16A	1C	1C
16A	1A	1A
16A	1A	4A
17C	1C	9C
16C	1T	9T
31G	28G	46G
32C	29C	46C
32A	29A	47A
32A	30A	50A
32A	30A	50A
16T_16C	30T	52T
32A	31A	53A
32G	31G	53G
33T	31T	53T
33G	33G	55G
35C	34C	55C
35T	34T	56T
37G	35G	56G
36T	36T	57T
36T	36T	58T
38C	36C	58C
38T	37T	59T
38C	37C	60C
38C	37C	60C
40G	37G	60G
40G	38G	62G
40A	39A	62A
40T	39T	65T
40A	39A	65A
38A	39A	67A
39A	39A	69A
38T	39T	70T
38G	39G	70G
38T	39T	70T
38A	39A	69A
38A	39A	68A

38A	40A	69A
39A	41A	66A
17A_22G	41G	66G
42T	41T	28A_37T
42T	42T	68T
42C	42C	68C
42A	43A	69A
43A	43A	69A
43G	44G	71G
45A	44A	71A
45C	44C	73C
45A	43A	76A
45C	42C	75C
44A	42A	75A
44G	44G	76G
44G	45G	77G
44A	44A	78A
44A	45A	80A
45G	46G	77G
47C	45C	76C
47C	46C	74C
49T	47T	74T
49C	48C	74C
49C	48C	75C
50T	48T	72T
50A	48A	72A
50T	47T	74T
50T	47T	76T
50G	46G	76G
50A	46A	78A
50C	47C	77C
50T	47T	76T
52G	47G	75G
53T	46T	75T
53G	46G	74G
53G	20A_25G	75G
53A	46A	73A
53G	46G	69G
53A	47A	69A
53T	47T	69T
55G	46G	71G
56C	46C	70C
54A	47A	69A

54G	47G	69G
53A	46A	69A
54A	45A	69A
54G	45G	69G
55G	45G	70G
56A	44A	68A
55C	46C	68C
57C	45C	68C
56C	45C	70C
54T	45T	71T
54T	46T	71T
56T	46T	74T
57G	46G	77G
59C	45C	77C
60C	46C	78C
63A	48A	78A
62C	48C	78C
62T	47T	79T
62G	48G	76G
61G	47G	76G
60C	45C	75C
59A	46A	74A
58G	46G	74G
58A	45A	73A
58T	47T	71T
58T	48T	72T
60A	49A	71A
57G	48G	69G
57C	49C	69C
57T	49T	67T
56G	48G	67G
55G	47G	66G
55T	47T	68T
53G	46G	68G
54A	46A	67A
54T	46T	65T
54G	47G	66G
53C	46C	65C
53C	46C	62C
52A	45A	62A
53C	45C	65C
51G	44G	66G
52G	44G	65G

53A	45A	63A
53A	46A	60A
53T	46T	62T
53G	47G	62G
52T	46T	61T
52T	46T	60T
52C	45C	59C
52A	44A	58A
52C	46C	60C
54G	47G	61G
54A	49A	30A_30G
53A	50A	59A
54C	51C	58C
53A	50A	58A
53T	50T	56T
52T	50T	56T
52T	49T	55T
51T	53T	55T
51A	54A	55A
51A	53A	56A
52T	53T	57T
52T	53T	56T
51T	53T	57T
51C	54C	57C
51C	53C	56C
51A	53A	57A
51A	53A	32A_26G
51T	52T	61T
51G	53G	61G
51A	53A	60A
52G	54G	60G
52G	55G	60G
52T	55T	60T
52G	55G	59G
52G	53G	59G
54G	52G	58G
54G	52G	56G
51G	53G	55G
53A	53A	56A
53G	53G	54G
53A	52A	53A
52A	52A	52A
53A	51A	52A

51T	52T	52T
51C	51C	51C
51C	52C	52C
51C	52C	53C
53T	52T	52T
53C	50C	53C
53C	50C	55C
53T	50T	55T
54C	48C	55C
54C	50C	57C
53T	49T	57T
52A	50A	57A
50A	50A	57A
51G	51G	59G
51C	51C	59C
51A	51A	59A
51G	52G	59G
51G	52G	60G
51A	52A	60A
51A	52A	60A
51A	53A	58A
54A	52A	60A
54A	53A	61A
51G	53G	63G
50G	53G	62G
50G	53G	62G
50G	54G	65G
50T	53T	65T
51T	53T	65T
53A	52A	64A
54G	54G	63G
54C	54C	65C
54A	54A	66A
54C	54C	66C
54G	54G	68G
54G	54G	67G
54A	55A	69A
54A	56A	70A
52T	56T	69T
55A	57A	71A
54T	57T	70T
54T	58T	71T
53C	58C	71C

54A	57A	73A
54C	57C	76C
52A	59A	77A
51A	57A	76A
49A	60A	75A
48C	61C	76C
45T	63T	75T
47G	34G_30C	74G
46T	64T	76T
46C	63C	77C
47C	64C	76C
47T	66T	73T
47G	65G	73G
47T	33T_36C	73T
45T	68T	75T
45T	68T	78T
44T	68T	78T
42C	67C	78C
43T	67T	78T
44A	66A	77A
44A	66A	79A
45A	67A	79A
46G	67G	77G
45A	67A	74A
47A	69A	76A
48G	69G	76G
23G_24C	69G	78G
46C	68C	75C
46A	68A	76A
46A	70A	76A
47G	72G	76G
46T	73T	77T
46T	73T	77T
45A	73A	74A
32A_44G	30A_72G	92A_74G
43A	72A	78A
44A	72A	77A
47A	72A	77A
48A	72A	78A
51A	72A	79A
51A	72A	79A
51A	73A	78A
51A	71A	78A

47T	67T	74T
48C	69C	79C
47C	67C	79C
48T	66T	79T
48C	65C	77C
48A	66A	76A
48A	65A	74A
48G	64G	71G
48T	61T	70T
47G	60G	68G
46A	60A	68A
45C	60C	67C
42A	55A	44A
42G	56G	46G
42G	55G	44G
22A_21C	55C	44C
21A_22G	55A	44A
45A	55A	45A
45G	55G	45G
45A	56A	45A
51G	71G	61G
52A	70A	61A
53G	70G	63G
54A	72A	65A
57A	72A	65A
57C	72C	65C
58A	72A	65A
59A	75A	67A
59G	75G	67G
57A	77A	66A
57C	77C	67C
57C	76C	67C
57C	77C	68C
57A	77A	69A
56T	78T	68T
57T	78T	67T
58A	78A	64A
59G	79G	63G
58C	79C	63C
58T	80T	65T
61G	80G	65G
61C	80C	65C
60A	80A	64A

62A	80A	64A
61A	80A	65A
61A	80A	66A
61C	80C	68C
61T	80T	69T
61C	80C	71C
60A	79A	72A
61T	81T	70T
61G	83G	71G
61T	84T	70T
61A	85A	70A
59G	84G	71G
59C	84C	69C
59C	83C	72C
59A	82A	72A
60T	82T	71T
64T	82T	72T
65G	85G	76G
64G	84G	75G
64T	84T	75T
66T	84T	74T
66G	84G	73G
66A	83A	73A
67T	87T	73T
67G	86G	72G
68G	86G	72G
68G	88G	73G
68C	88C	72C
68T	88T	72T
69G	88G	67G
66T	88T	68T
66T	88T	69T
66A	86A	70A
66A	87A	70A
67T	89T	71T
68T	90T	70T
68C	89C	71C
67C	88C	72C
67A	87A	72A
68C	87C	69C
66T	85T	67T
66G	83G	70G
67A	83A	70A

67A	84A	68A
67A	82A	68A
67C	81C	71C
69A	81A	71A
69T	80T	71T
69C	79C	72C
71T	78T	71T
71C	77C	69C
72G	79G	72G
71T	79T	71T
74T	79T	72T
74T	80T	70T
73C	80C	71C
72T	80T	74T
73C	81C	75C
71A	82A	73A
71A	81A	73A
71G	82G	73G
72A	82A	76A
73G	82G	79G
75A	80A	79A
74A	80A	79A
74A	77A	77A
74G	78G	76G
74T	78T	77T
74G	80G	76G
74G	81G	75G
77G	80G	76G
76A	78A	77A
73A	78A	76A
37A_34C	78A	73A
71G	78G	73G
36T_36C	78T	71T
73G	77G	70G
74T	77T	71T
75C	76C	71C
74A	74A	70A
74G	77G	70G
74A	76A	68A
73A	75A	70A
74C	75C	68C
75A	74A	68A
73T	74T	68T

75C	72C	67C
75A	73A	63A
75A	74A	63A
74C	71C	63C
41T_35C	69C	62C
76G	30A_40G	34A_28G
76A	71A	67A
74T	68T	68T
75A	70A	68A
75G	71G	69G
75C	69C	68C
75T	69T	69T
75C	69C	67C
76T	71T	69T
75T	69T	70T
74T	71T	70T
74C	72C	68C
72T	72T	70T
71C	70C	73C
71T	70T	75T
73T	68T	74T
74G	68G	72G
74G	68G	72G
74A	69A	77A
74T	69T	77T
74G	68G	75G
73A	68A	76A
71A	66A	76A
70T	65T	77T
71T	67T	74T
71A	67A	75A
68A	67A	74A
68A	67A	73A
68T	67T	72A
66A	66A	72A
66G	65G	70G
66A	64A	73A
66T	65T	73T
66C	67C	73C
68A	68A	73A
68A	69A	71A
68G	69G	70G
69A	67A	73A

72C	66C	73C
74A	66A	73A
75A	66A	71A
73A	66A	69A
74G	65G	71G
74C	67C	71C
73A	69A	72A
70C	69C	71C
72A	66A	69A
74C	67C	68C
72A	67A	68A
72C	67C	68C
71A	67A	66A
68A	68A	66A
67A	64A	65A
67C	64C	63C
67C	63C	65C
67A	62A	65A
67C	62C	64C
68T	62T	65T
68G	62G	69G
68A	62A	70A
69T	62T	71T
69T	62T	73T
69T	62T	74T
69T	26T_33G	73T
69G	59G	75G
69A	59A	74A
71G	59G	73G
71G	58G	72G
70G	58G	74G
70G	58G	74G
70G	58G	75G
69T	57T	74T
68G	58G	73G
68C	58C	76C
68A	58A	76A
67C	57C	75C
67A	56A	74A
67A	57A	74A
65A	56A	74A
66T	55T	72T
65A	54A	73A

65C	55C	73C
62A	54A	71A
61A	54A	67A
62C	54C	68C
62T	54T	73T
61G	53G	73G
61T	51T	75T
61T	52T	70T
63C	51C	70C
62C	51C	70C
62T	50T	69T
61G	51G	70G
63C	50C	70C
64T	50T	70T
63G	49G	70G
65C	48C	72C
67T	48T	73T
66T	48T	73T
64T	47T	71T
64C	48C	75C
65A	48A	72A
65T	48T	74T
63A	48A	76A
62T	48T	75T
61C	49C	74C
62C	53C	74C
62A	54A	72A
63T	53T	72T
61T	50T	72T
63G	49G	73G
64G	49G	73G
63C	49C	72C
62T	49T	73T
62G	49G	73G
59C	49C	74C
59T	51T	77T
59C	50C	80C
59A	49A	79A
57G	50G	78G
56C	50C	79C
55T	50T	80T
56G	50G	81G
54C	49C	80C

53T	49T	80T
55G	49G	80G
55A	50A	80A
54T	51T	82T
52G	49G	79G
52C	49C	80C
50C	48C	81C
50A	48A	78A
54C	49C	76C
55G	48G	76G
56G	48G	76G
54C	47C	78C
53A	49A	79A
53T	49T	80T
52G	47G	77G
53T	49T	79T
53T	49T	77T
53C	50C	78C
53A	52A	79A
53T	52T	76T
54A	52A	76A
55A	52A	76A
58A	55A	77A
58C	55C	77C
58A	55A	76A
59T	55T	76T
59T	55T	74T
59C	55C	73C
63A	56A	74A
63C	52C	75C
61A	52A	73A
61T	51T	69T
61T	49T	68T
61C	47C	64C
59C	49C	64C
56C	50C	65C
56A	50A	65A
55A	50A	65A
56C	50C	64C
39A_17G	31A_18G	63A
57G	47G	65G
56C	48C	65C
54T	48T	63T

52T	47T	62T
52G	49G	64G
51G	49G	65G
51C	50C	63C
50A	50A	63A
50G	50G	62G
51A	51A	64A
50A	51A	65A
50T	50T	67T
50G	51G	69G
50G	52G	70G
49G	51G	71G
49A	52A	70A
48G	51G	67G
48A	52A	67A
47A	54A	66A
47A	54A	66A
46G	54G	66G
49A	55A	66A
47A	55A	62A
46G	56G	62G
46G	56G	62G
46A	57A	63A
46G	57G	63G
46C	58C	63C
46A	56A	63A
46C	32A_58C	24A_63C
46A	35A_27C	63A
47A	62A	62A
47A	62A	63A
47A	60A	59A
47A	61A	59A
47A	61A	59A
47A	62A	61A
48A	63A	62A
50G	62G	61G
51C	63C	62C
50C	64C	65C
51A	64A	66A
52C	64C	67C
53C	65C	68C
55T	66T	66T
53A	65A	68A

19T_37C	65C	68C
37A_19G	65G	69G
55T	65T	68T
55G	67G	70G
54A	67A	74A
54C	69C	76C
55C	68C	76C
56A	68A	77A
56T	68T	77T
60A	68A	76A
60C	68C	78C
60C	68C	79C
60A	68A	80A
60A	68A	80A
59A	68A	79A
60A	66A	78A
59A	62A	79A
58T	62T	80T
59C	63C	80C
61C	63C	80C
63C	65C	81C
64T	65T	83T
64C	65C	83C
64C	67C	84C
65A	67A	85A
66T	68T	85T
67T	69T	87T
68T	69T	86T
69T	70T	86T
69T	70T	85T
69G	72G	84G
68T	73T	84T
67T	72T	83T
68T	71T	83T
69G	71G	80G
69G	69G	79G
70A	70A	80A
70T	71T	81T
72C	71C	80C
72T	71T	80T
72G	70G	81G
71T	69T	82T
67G	68G	82G

66T	67T	83T
66G	68G	82G
64C	69C	81C
68A	69A	80A
68A	70A	80A
68A	70A	81A
69T	70T	80T
68A	69A	80A
68T	70T	79T
67G	69G	81G
68G	71G	83G
68G	70G	84G
67T	68T	84T
66G	68G	83G
67A	68A	84A
66A	69A	84A
66C	70C	85C
67T	70T	83T
68T	69T	81T
66C	68C	81C
65C	69C	82C
65T	69T	80T
65T	69T	77T
65C	68C	75C
65T	70T	72T
65C	71C	73C
65A	73A	71A
65A	74A	69A
66G	74G	70G
65A	75A	70A
65G	75G	70G
64A	73A	67A
66A	74A	65A
66G	74G	65G
65A	75A	64A
68G	74G	65G
68G	73G	70G
68G	73G	70G
68A	73A	70A
68G	72G	70G
70T	71T	70T
70G	73G	72G
70A	72A	73A

42A_29G	40A_32G	76A
70C	72C	76C
71T	70T	73T
71C	70C	79C
69A	69A	77A
71A	70A	76A
70T	68T	79T
71T	69T	78T
70G	68G	75G
67G	68G	75G
67G	68G	75G
69G	68G	75G
69C	66C	78C
70A	67A	78A
70T	66T	78T
70A	66A	78A
70C	66C	75C
70A	39A_27C	74A
71G	63G	76G
72G	63G	75G
73G	63G	73G
73A	63A	75A
76G	63G	77G
75G	63G	77G
78A	63A	78A
77A	62A	78A
76G	61G	78G
75G	62G	79G
75A	61A	78A
76G	61G	79G
75G	61G	77G
74C	61C	79C
74A	61A	78A
77A	62A	80A
76A	62A	81A
76G	61G	81G
78G	60G	79G
77A	60A	79A
77A	61A	80A
76C	59C	81C
74A	60A	83A
73T	60T	83T
75A	61A	83A

71G	62G	79G
71T	62T	79T
70A	63A	81A
70T	63T	80T
70G	64G	79G
70T	66T	80T
69A	66A	80A
70T	66T	79T
70T	66T	82T
68T	66T	81T
67G	68G	84G
65G	69G	83G
63G	69G	84G
63C	72C	83C
64A	70A	81A
63G	73G	81G
63G	69G	84G
64A	67A	84A
63T	66T	84T
62C	67C	82C
63T	67T	77T
63T	67T	76T
63G	68G	74G
65C	67C	75C
66C	67C	78C
65C	69C	78C
66C	71C	77C
65T	71T	74T
65T	70T	74T
62T	71T	73T
63A	72A	75A
63G	72G	75G
63A	72A	74A
62G	72G	73G
63C	73C	74C
63C	73C	75C
64A	72A	75A
63T	71T	74T
63G	70G	73G
63C	73C	72C
63A	71A	72A
63A	71A	72A
62A	69A	75A

63C	68C	77C
63T	66T	78T
63C	65C	77C
64C	66C	75C
66A	65A	77A
65G	66G	75G
65G	65G	73G
65G	64G	72G
64C	64C	73C
64C	64C	74C
64C	64C	74C
64A	64A	72A
64A	64A	74A
64A	64A	74A
64C	64C	75C
64A	63A	75A
65C	62C	74C
65G	60G	72G
65G	60G	75G
64C	60C	76C
65T	59T	76T
68G	59G	75G
68C	59C	74C
67A	60A	72A
67G	60G	71G
69T	59T	73T
66T	59T	72T
67C	59C	71C
66G	58G	73G
66T	58T	71T
67T	58T	73T
66G	58G	72G
65T	58T	71T
65T	59T	72T
64C	59C	72C
64C	58C	70C
63T	59T	72T
63C	59C	71C
63T	61T	72T
62G	61G	71G
63T	60T	73T
63A	60A	73A
63G	60G	73G

63A	60A	72A
63T	59T	74T
61G	59G	75G
61A	58A	77A
62T	58T	75T
62G	57G	78G
63G	58G	78G
63A	62A	77A
63T	61T	76T
62G	60G	74G
61G	61G	74G
60A	61A	74A
61C	61C	75C
59A	61A	75A
58C	64C	74C
56A	63A	75A
56G	64G	74G
56A	63A	72A
56T	40A_22T	71T
51A	63A	73A
53C	69C	73C
51C	69C	73C
53T	67T	73T
53T	69T	74T
50T	68T	75T
49A	68A	74A
49A	71A	71A
47T	67T	71T
46C	72C	71C
47T	67T	71T
47C	73C	72C
46C	72C	72C
45C	71C	71C
46T	72T	72T
47G	71G	71G
47C	72C	71C
48C	74C	70C
19A_29G	74G	69G
48T	74T	72T
48T	72T	69T
19T_29C	72C	70C
46T	72T	69T
47C	72C	69C

47A	71A	74A
48A	68A	77A
48A	66A	75A
49G	63G	73G
50C	63C	74C
50A	63A	73A
50G	60G	72G
49C	61C	72C
48A	61A	72A
48A	62A	72A
50A	62A	74A
50C	62C	78C
51A	58A	75A
50G	58G	76G
49G	61G	75G
49C	60C	75C
50T	59T	76T
49C	57C	76C
50A	58A	76A
51G	57G	75G
51C	55C	75C
51T	56T	73T
51G	56G	72G
51A	57A	72A
52C	57C	72C
52A	56A	73A
51T	56T	71T
52G	56G	70G
51G	56G	67G
51A	56A	65A
50A	54A	66A
50A	55A	67A
52C	55C	66C
53C	56C	68C
54A	56A	68A
55T	57T	69T
54T	55T	70T
54A	56A	71A
53T	56T	70T
53C	55C	70C
54C	56C	68C
54C	57C	69C
56C	56C	70C

57A	57A	74A
58G	57G	75G
58C	58C	75C
32A_26G	37A_24G	74G
60C	63C	75C
61C	63C	75C
61C	63C	75C
61A	64A	74A
62G	63G	74G
63A	63A	75A
62G	63G	76G
62C	63C	74C
58C	63C	74C
59C	65C	74C
59T	63T	74T
61C	64C	74C
59T	63T	74T
61C	63C	74C
61C	67C	74C
61C	67C	73C
61T	67T	73T
60G	67G	72G
58G	67G	70G
58C	65C	62C
58G	64G	61G
58C	66C	62C
58G	68G	63G
59G	68G	64G
60G	69G	66G
60C	67C	66C
60A	67A	67A
59T	68T	68T
59G	68G	69G
58T	70T	70T
59T	70T	71T
60G	72G	72G
61T	72T	72T
62G	73G	71G
60G	74G	67G
61A	75A	68A
60A	75A	67A
61A	72A	68A
62A	72A	67A

62G	73G	66G
60A	72A	66A
61G	72G	66G
60G	71G	68G
59T	72T	69T
62C	70C	69C
62T	70T	69T
61T	69T	68T
64A	69A	68A
66G	68G	67G
67G	67G	71G
66A	65A	68A
68A	66A	70A
67C	66C	71C
67A	63A	71A
67C	65C	70C
68A	66A	70A
68A	65A	68A
68G	65G	68G
67G	64G	64G
69C	65C	65C
70G	62G	65G
69C	64C	66C
70A	64A	62A
70A	62A	66A
70A	63A	64A
71G	64G	64G
71G	61G	59G
70T	61T	59T
71T	61T	61T
70C	62C	61C
70T	62T	62T
70T	62T	64T
70T	62T	65T
71T	63T	66T
69G	64G	65G
69G	64G	65G
69T	64T	66T
68G	65G	65G
67C	34T_31C	69C
66G	65G	71G
67A	64A	70A
67G	64G	70G

67A	62A	70A
65G	62G	69G
64G	62G	70G
64C	62C	71C
64C	63C	71C
64A	60A	73A
63T	61T	74T
63T	62T	74T
65A	63A	76A
64G	62G	75G
62C	62C	76C
63A	62A	75A
63G	62G	76G
63G	62G	76G
63T	61T	75T
64G	61G	74G
64T	62T	76T
65G	61G	77G
64A	62A	76A
64A	63A	75A
64G	63G	73G
66C	62C	73C
67C	62C	75C
65G	32A_30G	75G
64G	61G	75G
63G	61G	75G
63G	61G	74G
63A	61A	76A
63G	62G	77G
62A	63A	77A
62T	64T	75T
60A	64A	73A
61A	64A	74A
63C	66C	75C
60C	67C	75C
60A	67A	77A
63G	66G	77G
64A	65A	75A
63T	63T	74T
63C	63C	78C
64C	62C	78C
34T_31C	60C	77C
65C	60C	77C

64T	62T	77T
64T	62T	78T
63T	61T	78T
63A	62A	78A
65A	60A	78A
65G	60G	79G
63C	62C	78C
63T	62T	78T
63G	63G	75G
63G	59G	76G
63C	59C	75C
63A	59A	75A
63G	60G	74G
62G	60G	73G
64C	59C	72C
66T	58T	70T
66G	58G	67G
67T	57T	66T
66T	56T	64T
66G	56G	69G
66T	57T	67T
67T	57T	68T
66G	58G	69G
66G	59G	66G
68G	57G	66G
67A	57A	67G
68A	56A	66A
68A	57A	65A
67A	56A	65A
66A	55A	67A
66A	57A	71A
68G	57G	71G
67T	56T	69T
65G	57G	68G
66G	58G	68G
66G	57G	66G
66T	56T	68T
66G	57G	68G
66A	56A	69A
64T	56T	67T
65T	56T	65T
64T	55T	66T
62A	56A	64A

61T	56T	61T
59C	55C	60C
61C	56C	62C
60T	55T	62T
62C	56C	63C
61T	56T	65T
63A	55A	65A
61A	54A	64A
61T	53T	64T
61A	53A	63A
61G	54G	64G
58G	55G	63G
58C	57C	65C
61T	56T	64T
62T	57T	65T
59G	56G	67G
60A	57A	66A
59C	55C	68C
59A	56A	66A
59A	58A	66A
60C	56C	66C
61C	56C	68C
61C	55C	70C
61C	55C	67C
60C	55C	66C
60A	56A	65A
60G	55G	64G
60G	55G	37A_27G
60A	56A	66A
60G	56G	67G
59A	56A	70A
59G	56G	71G
61G	57G	70G
61A	56A	70A
61G	58G	70G
61C	58C	70C
61A	59A	69A
60A	60A	69A
61G	59G	70G
60C	60C	71C
60A	60A	72A
59G	59G	74G
57G	58G	74G

58C	58C	74C
59A	57A	75A
59A	58A	75A
60G	58G	71G
61T	58T	71T
61C	57C	71C
59A	58A	70A
59A	59A	71A
58G	59G	69G
58A	59A	69A
58G	59G	69G
58C	59C	70C
58C	61C	69C
59C	60C	67C
59C	61C	64C
58G	61G	63G
58G	61G	62G
59A	60A	61A
59C	59C	62C
58T	60T	63T
59C	59C	61C
59T	58T	60T
59G	58G	60G
59G	31T_27G	60G
57T	56T	61T
57T	56T	61T
58G	56G	61G
55A	56A	62A
53G	54G	62G
53A	55A	62A
53G	55G	62G
52G	57G	62G
51T	57T	61T
49C	57C	61C
50A	55A	62A
28T_1G_21C	55C	62T
49G	55G	62G
49C	55C	62C
48G	55G	62G
48G	56G	59G
49A	54A	63A
48G	54G	64G
28T_18C	52C	64C

49G	55G	63G
48G	55G	62G
49C	55C	63C
49A	55A	64A
49A	54A	63A
48C	55C	62C
48A	55A	63A
49T	56T	64T
48T	58T	66T
49A	59A	68A
46A	59A	69A
50C	59C	72C
49C	59C	73C
49C	57C	76C
31T_19C	57T	74T
48G	57G	72G
48G	57G	71G
47A	54A	70A
47A	55A	70A
47T	54T	70T
48G	55G	71G
48T	54T	71T
47T	53T	70T
48G	52G	70G
48A	53A	75A
47G	52G	73G
46G	55G	72G
46T	59T	72T
47T	59T	72T
47C	59C	72C
46C	58C	71C
47C	59C	71C
48C	61C	71C
48T	61T	70T
47T	61T	69T
47G	61G	72G
45A	61A	72A
46C	61C	72C
44A	61A	72A
44T	61T	73T
43T	60T	73T
43G	60G	71G
43A	60A	73A

28A_15G	59A	74A
43T	57T	74T
46C	56C	74C
46C	54C	72C
45A	55A	74A
45G	54G	74G
45T	55T	73T
13T_31C	54C	74C
44G	54G	73G
46T	53T	71T
45G	51G	72G
46T	50T	73T
44C	51C	74C
44C	24T_29C	35T_37C
46G	51G	73G
46A	52A	72A
47C	51C	71C
47T	53T	67T
47C	55C	68C
47T	53T	66T
47A	53A	66A
47G	53G	65G
46G	52G	65G
46G	52G	64G
45C	30T_21C	57C
30A_14G_1C	51G	25A_29G_1C
46C	30T_21C	55C
50G	50G	59G
50G	50G	59G
50T	53T	59T
50G	52G	58G
50C	51C	58C
51T	53T	56T
52C	53C	55C
51A	53A	55A
49T_17C	52T_50C	30T_21C
53C	55C	49C
54C	54C	49C
53C	54C	17A_43C
55G	52G	46G
55T	52T	46T
56C	52C	47C
57T	53T	48T

58C	53C	50C
59C	52C	51C
59A	51A	52A
55A	51A	51A
56G	52G	51G
57C	53C	50C
57C	55C	51C
18A_38G	55G	50G
55T	55T	50T
55G	54G	52G
56G	55G	52G
55A	54A	51A
55G	54G	51G
56C	52C	51C
56C	54C	51C
56A	53A	52A
60G	55G	53G
59C	54C	55C
20A_40G	26A_29G	26A_28G
60T	54T	53T
60T	54T	54T
60T	54T	55T
60T	54T	54T
61C	55C	53C
59C	55C	55C
59A	55A	28A_27G
58T	55T	54T
59A	56A	54A
60G	56G	54G
60A	55A	54A
60C	55C	54C
59A	56A	52A
59G	56G	52G
60T	54T	53T
60T	55T	54T
60T	56T	55T
59T	54T	53T
59C	56C	55C
23A_37G	57G	28A_22G
60T	57T	50T
61G	57G	49G
60G	55G	19A_28G
59T	54T	45T

60C	54C	45C
59A	52A	42A
37T_22C	54T	41C
59G	55G	41G
60T	56T	44T
59G	57G	44G
37A_23G	57A	46G
60C	57C	51C
60C	58C	52C
60A	59A	53A
60G	61G	53G
59C	58C	23T_31C
58G	62G	56G
60C	62C	56C
61G	61G	57G
61A	61A	57A
63C	59C	58C
63T	56T	57T
63A	58A	57A
64G	58G	57G
64A	60A	58A
64C	58C	57C
63A	58A	57A
63C	58C	59C
65G	59G	60G
66G	59G	59G
67A	58A	56A
68A	59A	58A
68C	60C	60C
69G	60G	60G
69T	58T	60T
68C	58C	64C
70G	61G	64G
71T	61T	64T
71T	61T	63T
71A	61A	61A
71C	62C	60C
71C	61C	64C
69T	61T	61T
69T	60T	64T
69T	61T	66T
70C	60C	65C
71C	63C	63C

70C	63C	65C
70A	63A	65A
70C	64C	65C
70C	63C	63C
69G	60G	64G
68A	60A	62A
66G	63G	65G
66G	61G	66G
65T	59T	65T
64G	58G	65G
64G	57G	64G
65T	57T	66T
65C	58C	67C
65C	58C	67C
64C	61C	67C
65T	65T	72T
65A	65A	74A
65T	66T	74T
67T	64T	76T
71T	64T	75T
70A	65A	77A
70C	65C	78C
70C	28T_35C	79C
69G	64G	79G
68T	64T	80T
69A	66A	80A
70T	68T	83T
69T	67T	87T
67T	68T	87T
67T	67T	86T
67T	68T	87T
66C	67C	88C
68G	69G	90G
68C	70C	88C
67T	69T	84T
67C	71C	87C
66C	70C	86C
67A	71A	86A
67T	69T	86T
67A	70A	86A
67A	68A	86A
67G	69G	85G
68A	69A	84A

43T_25C	69T	77C
66G	72G	85G
68C	74C	88C
69A	77A	88A
68C	79C	89C
68C	79C	89C
68T	78T	87T
64C	74C	83C
64T	77T	87T
63C	76C	88C
63C	77C	88C
63A	77A	88A
62T	76T	87T
61A	76A	87A
61A	75A	86A
61G	75G	83G
62A	75A	83A
61C	74C	82C
22A_39G	74G	29A_53G
60C	73C	84C
61A	73A	84A
61C	74C	80C
62C	73C	82C
62A	73A	83A
61A	73A	80A
61A	68A	80A
59T	70T	79T
18A_41T	70T	30A_47T
59T	68T	75T
58T	65T	74T
57T	65T	74T
56A	66A	72A
35A_19G_2C	38A_26G	68G
57G	67G	82G
56A	68A	80A
56G	67G	80G
57A	67A	80A
57A	67A	82A
57G	65G	40A_34G
57A	64A	76A
56A	64A	76A
57A	64A	78A
56A	65A	78A

57C	64C	79C
57A	64A	78A
58G	65G	80G
58G	65G	83G
54A	66A	83A
53A	66A	83A
56A	65A	83A
56A	65A	84A
56A	65A	89A
56A	65A	89A
50T	64T	88T
52A	64A	85A
58A	66A	85A
54T	66T	85T
54C	66C	83C
54T	65T	81T
53G	65G	79G
52T	65T	79T
53T	64T	79T
53T	64T	76T
54T	64T	76T
59C	64C	76C
60T	65T	75T
59T	66T	74T
58C	67C	26T_43C
53G	43A_24G	60G
57C	67C	71C
55T	66T	73T
55C	66C	78C
55C	66C	78C
56A	66A	73A
56T	66T	80T
55A	65A	79A
54A	64A	82A
53G	64G	82G
54A	64A	81A
54C	64C	81C
54G	64G	82G
54C	67C	80C
54A	68A	81A
54C	68C	84C
54A	67A	88A
54G	68G	90G

55A	69A	90A
55C	69C	90C
55T	70T	90T
54T	71T	88T
56T	71T	86T
55C	43G_25C	88C
56C	67C	88C
55A	66A	86A
53C	66C	85C
54C	66C	85C
55T	66T	85T
55C	66C	85C
56C	66C	89C
57C	66C	93C
57T	65T	89T
57G	41G_23C	86G
58T	63T	87T
57T	62T	85T
58T	62T	87T
58T	61T	88T
57G	62G	89G
56T	62T	86T
56G	62G	84G
54G	63G	84G
53G	61G	84G
51G	39A_14G	62G
51G	39A_13G	62G
32A_20G	39A_13G	67A
54A	56A	67A
55A	55A	68A
55A	58A	70A
57G	59G	70G
59T	58T	72T
62G	58G	72G
62C	57C	32A_1T_40C
64G	56G	74G
64T	57T	74T
63C	54C	74C
63T	54T	73T
63T	53T	76T
63A	54A	75A
63T	54T	76T
64A	55A	77A

67G	55G	77G
67T	55T	77T
71G	56G	82G
71C	56C	82C
71G	56G	82G
73A	56A	82A
74A	55A	85A
74A	57A	88A
75A	58A	86A
76A	60A	87A
75T	61T	87T
74C	61C	84C
74T	62T	81T
73A	62A	82A
76C	62C	83C
76T	62T	83T
42T_34C	62C	44T_42C
78G	62G	83G
79C	61C	84C
79A	62A	85A
81T	62T	87T
81T	61T	86T
81T	58T	86T
81T	58T	85T
83A	59A	82A
85A	57A	79A
85C	58C	78C
83A	58A	79A
84T	58T	79T
87G	58G	79G
87C	58C	81C
87T	58T	82T
85T	57T	84T
86T	57T	82T
86C	57C	83C
87T	55T	82T
88A	52A	82A
89A	56A	85A
89C	55C	86C
88T	55T	83T
88G	56G	80G
87C	58C	84C
87T	56T	86T

87A	56A	85A
86G	56G	84G
88G	55G	83G
88T	53T	82T
89T	54T	82T
89G	53G	84G
88G	53G	85G
88C	53C	87C
88A	53A	87A
86G	54G	87G
87G	53G	88G
85A	53C	87A
81G	51G	88G
76G	50G	85G
78T	50T	86T
79G	49G	86G
80G	49G	84G
81G	49G	85G
82A	50A	87A
83C	50C	87C
84A	50A	92A
83A	50A	93A
85G	49G	93G
83C	50C	92C
85G	49G	94G
90A	51A	92A
90T	50T	91T
89G	50G	93G
89G	51G	94G
89G	48G	90G
89A	48A	92A
90G	47G	96G
92C	48C	95C
91T	48T	92T
91G	48G	52A_41G
91A	46A	93A
90A	46A	92A
90T	45T	92T
88C	45C	93C
87T	47T	94T
86G	47G	94G
86T	47T	92T
88C	46C	90C

87A	48A	89A
85C	47C	90C
85G	47G	51A_38G
87T	47T	98T
85G	47G	95G
86G	49G	96G
86A	49A	96A
86T	49T	31A_158T_31G_62C
88T	49T	66T
88C	49C	64C
90G	49G	63G
89A	50A	63A
89T	51T	62T
89C	50C	62C
89T	49T	100T
90T	50T	101T
91A	50A	102A
88C	52C	102C
91A	53A	103A
91A	53A	103A
91T	54T	92T
94G	52G	100G
93G	51G	99G
91C	52C	99C
88T	52T	100T
88G	53G	98G
85G	53G	98G
84T	52T	99T
85C	53C	99C
83T	52T	98T
84T	52T	99T
84C	51C	100C
42T_42C	26T_27C	100C
83G	53G	100G
81A	53A	99A
79T	54T	98T
77C	54C	98C
77T	52T	96T
77T	53T	95T
75G	52G	94G
75C	52C	95C
75A	51A	93A
74G	51G	94G

75C	51C	93C
70C	51C	94C
77C	51C	92C
77A	51A	95A
77G	51G	92G
75A	50A	91A
74G	28A_23G	91G
73G	51G	90G
74C	50C	80C
75T	52T	88T
76T	52T	85T
74C	53C	83C
74T	54T	83T
74G	54G	85G
71T	55C	40T_42C
72G	55G	78G
66A	57A	79A
73T	58T	84T
73T	58T	79T
72T	57T	78T
69A	57A	78A
70A	56A	81A
70C	55C	79C
71C	56C	81C
41T_29C	56C	81C
69G	57G	84G
70C	57C	84C
68A	58A	82A
66G	56G	83G
66C	56C	83C
65G	56G	85G
66C	57C	86C
65C	55C	85C
65A	55A	85A
65C	57C	83C
65C	56C	81C
61T	56T	78T
30T_30G_89C	28T_28G_84C	31T_31G_110C
27G	27G	48G
28T	27T	48T
27C	27C	49C
62C	25T_31C	84C
60G	54G	78G

62T	54T	77T
62C	58C	77C
62C	58C	79C
62C	58C	81C
62C	59C	81C
63C	58C	83C
63T	59T	85T
61G	59G	87G
61G	61G	87G
59A	59A	87A
60A	60A	87A
60A	59A	85A
60G	60G	84G
60T	59T	83T
59T	60T	85T
60G	60G	85G
58A	60A	87A
58A	60A	87A
60C	59C	86C
62T	59T	83T
60A	59A	82A
60C	59C	82C
59C	58C	83C
59T	58T	83T
59C	58C	82C
59T	57T	82T
60C	56C	83C
58T	57T	82T
57T	59T	82T
58A	58A	81A
59A	59A	80A
59G	60G	81G
59G	61G	81G
60C	62C	82C
61A	60A	84A
61C	63C	84C
61T	63T	82T
60G	62G	82G
62G	64G	83G
62C	63C	83C
62A	63A	82A
63G	64G	80G
62G	64G	81G

62G	63G	79G
61A	60A	80A
63T	62T	80T
65A	63A	79A
63G	62G	78G
61T	63T	74T
62A	62A	74A
63A	64A	75A
63G	63G	72G
62A	63A	69A
61G	66G	70G
61C	65C	68C
60T	67T	67T
61A	66A	66A
61A	66A	66A
60A	67A	66A
58T	30T_36C	67T
60T	66T	65T
59C	64C	61C
59A	64A	61A
59T	64T	60T
59C	65C	59C
59C	66C	57C
59T	66T	57T
59T	66T	57T
60G	65G	60G
59G	65G	59G
60G	64G	60G
62G	64G	61G
61A	64A	60A
62A	62A	59A
61T	61T	60T
62G	63G	58G
62T	62T	59T
62T	63T	57T
62G	63G	58G
61A	65A	57A
62A	65A	59A
62C	65C	62C
62C	64C	63C
64T	66T	62T
64C	66C	60C
63C	67C	62C

60A	67A	62A
60G	67G	61G
60C	68C	63C
61A	67A	64A
61T	67T	65T
59G	66G	68G
60G	65G	67G
61G	63G	66G
61G	63G	67G
59T	65T	67T
61G	64G	65G
60C	66C	66C
59A	66A	65A
60G	34A_34G	66G
59C	68C	65C
58T	68T	67T
58A	68A	68A
57A	68A	68A
31T_25C	34T_35C	67C
56G	70G	68G
59G	70G	67G
60T	69T	66T
60C	68C	63C
60C	65C	62C
59C	30T_35C	63C
61T	63T	64T
61C	63C	64C
61T	63T	63T
60G	65G	63G
62G	64G	62G
62T	65T	63T
62G	65G	62G
61C	65C	63C
61C	65C	66C
59A	64A	65A
61C	64C	66C
61T	64T	67T
61T	64T	66T
62C	64C	71C
61A	64A	71A
61C	64C	72C
62G	64G	43A_30G
62C	64C	73C

62T	65T	73T
62G	66G	74G
60C	66C	75C
62A	66A	74A
62G	66G	74G
62T	66T	73T
63T	68T	72T
63T	66T	70T
62A	68A	70A
61C	68C	68C
60A	69A	69A
57G	69G	69G
57C	68C	69C
61A	67A	70A
61T	67T	69T
60C	66C	67C
61T	65T	69T
60G	66G	67G
60C	66C	70C
61C	65C	69C
63T	66T	70T
62T	65T	68T
64T	63T	65T
66T	63T	65T
65T	32A_30T	68T
64A	62A	68A
64A	60A	67A
63A	59A	64A
64C	57C	65C
61T	57T	64T
62G	56G	63G
27T_1G_34C	56T	61T
62A	56A	60A
62T	56T	59T
62G	57G	62G
61A	56A	63A
61G	57G	62G
60C	57C	61C
60T	58T	61T
60T	59T	63T
59T	57T	63T
57G	58G	63G
58C	59C	62C

58A	60A	61A
59G	59G	60G
58C	57C	59C
58A	58A	59A
58G	58G	60G
60A	59A	62A
33G_27C	59G	61G
59G	59G	36A_1T_24G_1C
57C	59C	62C
58C	59C	61C
58T	59T	61T
27T_31C	58C	63C
59G	59G	62G
59C	58C	68C
59C	57C	71C
60T	57T	71T
59G	57G	72G
60T	60T	73T
60T	61T	75T
61A	61A	74A
60T	62T	72T
62C	60C	73C
64C	61C	71C
66C	62C	72C
66C	29T_33C	74T
65G	60G	71G
66G	62G	70G
66A	59A	71A
64T	59T	69T
65C	59C	71C
65A	59A	72A
62A	55A	72A
61G	54G	74G
62C	53C	75C
64C	52C	77C
64C	26T_23C	78C
30T_33C	49C	78C
34A_29G	22A_26G	79A
63G	26A_23G	80G
63G	51G	82G
63T	54T	81T
63G	54G	82G
63C	54C	83C

63A	54A	81A
62C	54C	82C
28A_34G	53G	79G
62G	54G	80G
62A	54A	82A
62T	54T	84T
64A	54A	86A
64T	55T	86T
65T	56T	86T
64G	56G	86G
30A_36G	56G	88G
65G	56G	89G
65A	56A	88A
66T	56T	88T
67G	57G	88G
67G	58G	89G
66G	61G	89G
66A	61A	89A
68T	61T	88T
69A	59A	89A
69A	61A	90A
70A	59A	91A
69A	60A	92A
70A	61A	90A
70C	60C	90C
73A	60A	90A
70G	59G	90G
72C	59C	92C
72A	59A	94A
71C	58C	94C
41T_31C	59C	94C
71C	58C	94C
71A	59A	93A
72G	57G	93G
73T	58T	92T
74C	57C	92C
74C	57C	92C
73T	57T	91T
74T	58T	88T
74C	58C	91C
72A	57A	86A
71A	57A	83A
72A	54A	82A

71A	56A	81A
70G	55G	79G
70A	54A	77A
69C	54C	79C
67C	54C	79C
67A	54A	78A
67T	55T	76T
67C	56C	77C
67C	56C	76C
67A	56A	76A
66G	56G	77G
64C	55C	74C
64T	55T	73T
62T	56T	74T
60T	55T	73T
59G	56G	74G
60G	55G	71G
60G	55G	73G
60T	55T	72T
61T	57T	71T
61C	58C	71C
62A	59A	68A
61A	59A	67A
62T	57T	64T
62G	60G	63G
62G	60G	66G
62G	60G	67G
61A	59A	68A
61C	56C	68C
62A	57A	67A
62G	57G	69G
62A	57A	65A
62A	54A	65A
61G	54G	63G
62G	52G	62G
60C	51C	60C
61A	51A	59A
59G	50G	56G
59G	50G	57G
57C	49C	60C
57C	48C	61C
58A	48A	61A
58A	49A	60A

1A_31T_26C	23T_25C	59C
58G	48G	59G
58C	47C	57C
57C	45C	55C
57A	25A_21G	54A
58T	46T	53T
58C	46C	53C
60A	46A	55A
60A	45A	57A
60A	44A	57A
58C	43C	55C
59A	44A	52A
57G	44G	50G
55G	45G	49G
54A	23A_19G	49A
54T	42T	49T
55C	42C	48C
54T	42T	49T
54G	42G	50G
54T	42T	50T
53G	42G	36G_14C
54G	43G	50G
54C	43C	50C
54T	44T	49T
52G	43G	48G
51G	43G	47G
50G	41G	48G
50C	42C	47C
49A	42A	45A
49T	41T	44T
49T	41T	42T
61A_1G_26C	58A_41C	77A_64C
36A	17A	38A
36C	13A_17C	38C
36A	30A	38A
12A_37C	32C	38C
54A	33A	39A
54C	8A_31C	16A_40C
54A	45A	65A
56C	43C	65C
56A	46A	67A
55C	45C	66C
53A	44A	66A

54C	45C	67C
52A	43A	68A
32A_81C	42C	70C
31A	43A	68A
30C	43C	68C
24A	43A	69A
26C	37C	66C
42A	36A	66A
43C	37C	67C
18A_27C	37A	68A
44C	37C	68C
44A	37A	68A
44T	38T	63T
44C	38C	67C
44G	38G	67G
44C	40C	70C
44C	40C	71C
44A	41A	71A
44T	41T	73T
44A	40A	74A
44A	41A	71A
43C	41C	73C
41A	43A	73A
41T	43T	74T
42T	43T	76T
42T	44T	76T
42G	45G	78G
40G	45G	81G
39T	46T	81T
38G	46G	81G
39T	47T	82T
38T	46T	84T
38G	46G	85G
37A	45A	83A
37T_22C	44T_22C	83T_1G_36C
37C	44C	86C
39C	45C	86C
39C	45C	85C
39C	45C	84C
39C	44C	85C
38C	45C	85C
38C	21A_45C	83C
38C	22A_23C	81C

39A	43A	81A
39A	44A	85A
39A	46A	85A
40C	45C	85C
40T	45T	85T
40T	45T	87T
40G	45G	87G
41C	45C	87C
41C	45C	88C
41A	45A	89A
41G	45G	88G
41C	45C	86C
43T	44T	87T
43T	40T	86T
43G	17A_23G	86G
43G	40G	88G
43G	17A_23G	89G
43A	42A	87A
42A	42A	88A
41G	41G	87G
41G	42G	88G
40G	41G	85G
40A	43A	85A
39G	43G	86G
39T	44T	89T
39T	45T	91T
40C	44C	94C
40T	44T	92T
41C	44C	92C
41A	44A	96A
41T	43T	94T
40T	44T	94T
40T	44T	92T
41C	45C	95C
41A	46A	94A
42G	47G	94G
43G	47G	93G
43C	48C	93C
42A	47A	92A
42A	46A	92A
42G	45G	91G
42T	46T	88T
43T	47T	88T

41G	47G	87G
41T	47T	86T
41G	47G	87G
41A	47A	87A
41C	47C	88C
41A	47A	90A
41G	47G	89G
40A	44A	91A
38A	46A	91A
38T	45T	91T
38C	45C	88C
37C	44C	86C
37C	45C	86C
37C	45C	86C
38C	44C	86C
38T	45T	86T
37T	43T	83T
40T	43T	85T
39C	43C	82C
39T	41T	82T
40C	41C	82C
40A	41A	83A
40G	41G	84G
39A	42A	82A
38G	44G	82G
37T	44T	80T
36A	43A	81A
36A	43A	83A
36C	44C	81C
34A	44A	82A
35T	44T	83T
35T	44T	82T
36T	44T	79T
37C	43C	81C
38T	43T	79T
38G	43G	78G
38C	44C	77C
38A	42A	77A
38A	42A	78A
37A	42A	76A
36T	42T	76T
36C	41C	76C
36T	41T	75T

38T	42T	72T
39A	41A	71A
41T	42T	71T
42A	42A	71A
42T	42T	69T
42T	41T	70T
44T	41T	70T
43A	42A	69A
44T	41T	67T
45A	41A	65A
22G_23C	41G	62G
44A	42A	64A
44A	40A	60A
44T	40T	57T
43A	39A	61A
21A_21G	19A_19G	61G
42G	40G	62G
42G	39G	59G
42G	40G	59G
42A	40A	61A
43G	40G	60G
44G	40G	60G
45A	39A	60A
46A	40A	60A
49A	40A	61A
49T	38T	60T
50G	38G	61G
47T	38T	60T
48T	39T	59T
48A	39A	60A
48C	39C	59C
48C	38C	59C
48C	39C	57C
49T	39T	56T
50C	39C	55C
49C	39C	55C
49C	39C	55C
48T	38T	56T
48C	38C	55C
49C	39C	55C
49C	37C	55C
48A	36A	53A
47T	36T	53T

48T	36T	53T
49C	36C	50C
49G	36G	50G
47G	36G	17A_34G
24A_23G	52A	26A_34G
47A	16G	59A
47A	18A_18G	58A
47A	37A	60A
48A	37A	59A
47A	38A	60A
48A	38A	60A
49A	38A	58A
52A	38A	56A
53A	39A	57A
56C	39C	54C
57A	39A	53A
57A	39A	54A
56A	40A	53A
56A	38A	53A
56A	39A	55A
56A	39A	56A
57C	39C	54C
58A	41A	55A
58A	41A	56A
57A	41A	55A
56A	40A	55A
56C	40C	55C
55A	38A	56A
55C	39C	58C
55A	39A	58A
56A	38A	58A
55G	38G	59G
56C	39C	58C
56T	40T	58T
56A	40A	58A
54G	40G	58G
56G	42G	60G
56A	42A	59A
57G	43G	60G
56T	43T	58T
56T	43T	58T
57A	44A	60A
56C	44C	61C

57A	45A	60A
57T	44T	60T
57C	46C	61C
57G	47G	64G
59C	46C	65C
58T	45T	66T
61A	44A	66A
60A	45A	68A
58A	44A	68A
58T	43T	70T
58G	44G	70G
57T	45T	70T
58C	45C	72C
58A	44A	70A
58T	45T	72T
60A	45A	27A_45C
59T	47T	71T
61C	45C	72C
61T	45T	72T
62C	45C	73C
62T	43T	71T
60C	43C	72C
60T	43T	71T
60C	43C	73C
60T	43T	75T
58T	45T	75T
59C	45C	76C
62C	45C	77C
64A	44A	77A
64C	45C	76C
65G	45G	74G
66C	45C	73C
65G	45G	73G
67G	45G	73G
66G	45G	74G
66C	45C	75C
64T	44T	75T
63T	43T	76T
64G	44G	77G
63A	44A	79A
62C	43C	78C
62A	42A	76A
64A	44A	78A

63T	44T	78T
64G	46G	81G
64A	46A	82A
64G	46G	81G
64G	45G	79G
67G	46G	80G
67C	46C	79C
67A	46A	77A
67G	44G	81G
68T	44T	81T
69T	43T	80T
68C	42C	80C
67A	42A	79A
65G	42G	80G
65C	41C	83C
62G	42G	84G
63C	42C	83C
62G	42G	82G
62A	42A	84A
62T	42T	84T
61G	41G	84G
61A	39A	85A
60A	40A	84A
62C	40C	84C
62T	42T	83T
61C	43C	84C
62A	42A	85A
62C	43C	87C
62C	43C	87C
63T	43T	85T
63G	42G	83G
63G	41G	81G
64T	41T	81T
64T	41T	83T
64T	42T	80T
63G	44G	81G
63G	44G	77G
62C	45C	78C
62A	45A	78A
61G	45G	75G
63A	47A	76A
63A	48A	75A
62G	48G	75G

62A	47A	72A
62A	47A	75A
60A	48A	77A
58C	47C	77C
57C	46C	78C
56C	46C	78C
55T	45T	78T
55A	46A	77A
55T	45T	76T
55T	45T	78T
54C	46C	77C
54A	47A	76A
53T	47T	76T
51G	45G	77G
53C	45C	77C
53T	46T	77T
53T	47T	79T
53T	47T	80T
53A	47A	78A
53A	48A	78A
54T	48T	78T
54T	50T	78T
54G	50G	80G
53A	48A	81A
54A	47A	81A
54T	48T	81T
54A	47A	80A
51T	45T	80T
51T	44T	76T
51T	47T	76T
51G	47G	76G
51A	46A	76A
50A	47A	75A
50T	47T	75T
52A	47A	75A
52A	46A	71A
51T	44T	70T
52C	44C	70C
50T	44T	71T
49T	45T	71T
48T	45T	71T
50T	44T	72T
51C	44C	72C

51A	44A	72A
51T	44T	74T
50T	43T	73T
49C	44C	73C
50C	44C	72C
50T	44T	73T
48G	44G	74G
48C	42C	74C
48G	42G	73G
48A	41A	68A
47A	41A	68A
48T	41T	67T
49G	41G	68G
48A	41A	67A
48A	43A	66A
48T	42T	66T
48G	41G	67G
47T	41T	66T
44T	41T	66T
42G	42G	65G
41T	41T	68T
42C	41C	69C
42C	41C	70C
42A	41A	71A
41A	42A	72A
40A	42A	72A
39T	40T	73T
39A	41A	72A
39A	41A	72A
39C	41C	69C
39A	41A	68A
39A	40A	66A
39C	41C	25A_25T_65C
37A	40A	38A
37T	40T	39T
36C	37C	63C
36G	18A_20G	61G
36A	38A	60A
37C	38C	60C
34T	38T	61T
34C	40C	62C
34G	40G	63G
34C	41C	64C

33T	38T	66T
32C	41C	68C
32G	42G	68G
33C	44C	65C
33C	43C	66C
33G	43G	67G
33C	43C	66C
32A	43A	63A
32T	43T	62T
10A_32T_10G	15A_43T_15G	29A_63T_28G
33G	44G	71G
33A	44A	66A
32G	44G	67G
32A	44A	71A
31G	44G	72G
30A	42A	71A
29G	41G	73G
28A	40A	74A
28G	40G	74G
28A	41A	74A
28G	41G	76G
27A	41A	74A
27G	42G	74G
26A	42A	75A
26G	43G	77G
26A	42A	72A
27G	40G	73G
28A	40A	74A
28G	40G	72G
28A	40A	71A
27C	40C	67C
29C	39C	68C
29C	38C	68C
29A	38A	68A
29A	39A	69A
30G	40G	70G
30C	39C	72C
27A	39A	71A
28C	39C	69C
28G	39G	70G
28G	39G	70G
29C	40C	72C
31T	39T	73T

31G	41G	72G
31C	42C	70C
31G	41G	71G
31A	42A	70A
32A	42A	67A
31G	42G	65G
31A	40A	64A
31C	40C	63C
31T	39T	61T
30C	38C	62C
30C	38C	61C
30C	37C	63C
29T	35T	62T
29C	35C	62C
29C	36C	63C
28G	37G	62G
28A	36A	61A
28T	36T	61T
28G	36G	61G
68A_20G_31C	85A_24G_36C	63A
30C	38C	63C
29A	37A	61A
30A	38A	61A
29A	37A	62A
30A	37A	58A
29G	37G	62G
29C	37C	61C
29G	36G	60G
29C	36C	61C
29C	36C	60C
14A_11G	17A_15G	59G
24A	32G	30A_30G
23G	32G	58G
23A	32A	55A
23G	32G	58G
23A	33A	58A
23A	33A	55A
23G	33G	57G
22G	33G	59G
22A	35A	60A
23G	36G	57G
23A	36A	57A
22G	36G	61G

22G	36G	60G
22A	38A	60A
22C	39C	61C
21A	39A	62A
21G	37G	65G
22C	37C	67C
22C	37C	66C
22A	38A	63A
22G	38G	62G
22A	39A	63A
22G	38G	62G
22G	38G	61G
20T	37T	61T
22G	38G	59G
22G	38G	58G
22G	38G	58G
22A	39A	56A
22G	38G	57G
23G	37G	57G
24G	38G	56G
23G	38G	54G
23G	38G	54G
23A	36A	54A
24C	36C	53C
24C	37C	55C
25G	38G	52G
24T	37T	51T
24T	37T	51T
26T	38T	52T
26T	38T	52T
26T	37T	53T
26C	35C	55C
26T	35T	56T
26A	35A	57A
28A	33A	59A
26G	34G	59G
27C	33C	59C
27C	32C	59C
27C	32C	59C
27C	33C	61C
28T	33T	62T
28C	34C	61C
28A	34A	63A

28A	36A	63A
28G	36G	63G
27T	36T	62T
27G	37G	60G
27T	36T	59T
27T	36T	61T
27C	36C	60C
26T	37T	59T
26T	37T	57T
26T	36T	57T
26G	34G	56G
26C	35C	57C
26T	35T	57T
25T	34T	57T
25T	36T	59T
25C	34C	59C
26A	33A	59A
26G	32G	61G
26C	32C	59C
27G	32G	59G
27C	31C	59C
27C	31C	55C
26G	31G	55G
25C	31C	54C
23T	29T	55T
23T	29T	55T
23T	29T	52T
23T	30T	52T
23G	30G	53G
23T	30T	52T
24T	30T	51T
24T	30T	48T
26G	29G	50G
26C	29C	50C
26C	28C	51C
26G	28G	49G
26G	28G	49G
26A	26A	51A
25G	25G	53G
24G	27G	54G
26C	27C	55C
27T	27T	56T
26T	27T	56T

26C	27C	57C
27T	28T	56T
28G	26G	56G
28A	27A	55A
28T	28T	57T
28A	28A	59A
28A	26A	61A
28A	26A	61A
29T	26T	60T
29T	27T	61T
28G	27G	61G
28C	27C	61C
28A	27A	61A
28A	26A	61A
28C	26C	61C
27C	26C	60C
27G	27G	60G
27C	27C	60C
28T	27T	58T
27T	27T	59T
27A	27A	58A
27A	27A	57A
28G	28G	56G
28G	28G	54G
28G	28G	54G
28A	28A	54A
28A	27A	54A
28C	27C	54C
28G	27G	54G
28C	26C	56C
27G	26G	56G
27A	25A	57A
27G	24G	56G
28G	25G	57G
28C	26C	57C
28G	27G	58G
28C	26C	57C
28A	26A	58A
28T	26T	59T
27A	27A	58A
28A	26A	58A
29A	26A	57A
29A	27A	57A

29G	27G	57G
29C	27C	55C
30C	27C	54C
30G	27G	55G
29C	27C	56C
29T	27T	57T
29C	26C	57C
29T	27T	57T
29T	28T	55T
28G	28G	55G
27C	28C	54C
26T_20C	28T_10C	56T
28C	29C	54C
28C	29C	56C
28C	29C	56C
28C	28C	56C
29C	27C	52C
28C	28C	52C
25T	27T	52T
25C	27C	56C
25C	27C	55C
25C	27C	55C
25C	27C	53C
26T	28T	51T
24C	28C	51C
24G	28G	51G
23G	29G	51G
23A	28A	51A
23G	29G	52G
22T	27T	51T
22G	27G	52G
22G	27G	51G
21T	29T	49T
22T	29T	46T
22G	27G	48G
23T	28T	51T
23G	28G	49G
23T	27T	49T
23G	28G	47G
23A	28A	47A
23A	28A	47A
22T	27T	46T
23G	27G	45G

23G	27G	44G
23A	27A	43A
24A	27A	42A
24G	27G	43G
24T	27T	44T
24C	26C	45C
23A	27A	45A
23G	27G	45G
23G	27G	45G
23C	27C	46C
23G	27G	45G
23G	27G	45G
24G	28G	45G
24C	27C	44C
24G	29G	44G
24C	30C	43C
24A	29A	44A
24C	29C	43C
24G	29G	42G
24G	30G	41G
24G	30G	41G
24A	30A	40A
23G	30G	38G
23G	30G	39G
23C	30C	40C
23A	30A	40A
23G	29G	39G
23G	30G	40G
23A	30A	39A
23G	31G	40G
22G	30G	40G
21G	30G	40G
21G	30G	40G
21G	30G	38G
18T	30T	39T
20C	29C	41C
20A	29A	42A
21A	27A	42A
21T	26T	40T
20C	26C	40C
20C	26C	39C
19A	26A	40A
20T	26T	40T

20T	25T	40T
20A	24A	39A
20A	24A	40A
20G	25G	37G
20G	25G	37G
20T	24T	37T
20T	25T	38T
20C	25C	38C
20T	25T	41T
20A	25A	40A
20T	25T	40T
20T	24T	39T
19A	25A	38A
19A	25A	38A
19G	26G	37G
19T	27T	37T
20C	27C	37C
20C	27C	38C
21T	27T	37T
21C	27C	34C
21C	26C	34C
21T	26T	35T
21A	26A	35A
20G	26G	36G
20A	25A	36A
20G	25G	37G
20A	25A	37A
20A	25A	38A
20C	25C	38C
21A	25A	39A
20A	24A	39A
19A	24A	38A
20G	24G	37G
20C	23C	37C
19T	23T	38T
19A	23A	37A
19A	23A	36A
19C	23C	37C
20C	23C	37C
20T	22T	37T
20G	22G	36G
20T	21T	36T
20G	20G	36G

18C	21C	35C
18C	21C	35C
18A	21A	35A
18T	21T	35T
17C	21C	37C
17C	21C	37C
17T	20T	37T
15C	20C	36C
15A	20A	35A
15T	19T	34T
15A	20A	35A
15A	20A	36A
16A	21A	37A
16T	21T	37T
15A	20A	36A
15T	20T	34T
15T	20T	35T
15G	19G	36G
15C	17C	34C
15T	18T	32T
15G	19G	31G
15C	19C	32C
15A	19A	32A
15T	18T	32T
15G	18G	31G
15G	18G	30G
14A	16A	29A
14G	16G	30G
15G	16G	31G
15A	16A	29A
14G	16G	28G
14C	16C	27C
13C	16C	28C
13C	16C	27C
13C	16C	27C
13G	16G	24G
12A	16A	24A
12A	16A	24A
12T	16T	24T
12T	16T	24T
12T	15T	24T
12T	15T	24T
12A	15A	24A

12A	15A	24A
12T	15T	23T
12T	15T	23T
11A	15A	23A
11A	15A	23A
10A	13A	24A
11C	13C	24C
11C	12C	23C
13T	12T	22T
13C	12C	21C
12T	11T	21T
12T	11T	20T
12T	11T	20T
12A	11A	19A
13C	11C	17C
13C	11C	18C
13T	11T	18T
13G	10G	18G
13C	9C	18C
13T	9T	18T
13C	8C	18C
13A	8A	17A
13G	8G	17G
13G	7G	17G
13A	8A	17A
12G	8G	18G
12A	8A	18A
12T	8T	18T
11A	8A	18A
12G	8G	17G
12A	8A	18A
13G	9G	16G
14G	9G	15G
13G	9G	15G
13G	9G	15G
13G	9G	15G
13G	9G	15G
13A	8A	14A
13G	8G	12G
13G	9G	11G
13A	8A	11A
13G	8G	11G
12A	8A	11A

12G	8G	10G
12A	8A	9A
12A	8A	9A
12G	8G	11G
13A	8A	11A
20A_26G	15A_24G	16A_26G
13G	6G	10G
15G	6G	10G
15A	6A	10A
14G	7G	10G
14G	7G	10G
15G	8G	10G
15A	8A	9A
15G	8G	9G
15G	8G	9G
15G	9G	9G
15A	8A	9A
15G	8G	9G
15G	9G	10G
15G	9G	10G
14A	9A	10A
14G	9G	11G
14G	10G	11G
13G	10G	11G
13A	10A	13A
13G	11G	14G
14A	11A	11A
15G	13G	13G
15A	13A	15A
15A	14A	18A
15A	14A	19A
15T	15T	19T
15C	17C	18C
15T	17T	20T
15A	18A	21A
15T	19T	21T
14T	19T	22T
15T	19T	22T
14A	18A	22A
15G	18G	23G
15A	18A	22A
15C	18C	23C
14T	18T	23T

13T	20T	24T
14G	21G	25G
14A	22A	27A
17A	22A	29A
17C	22C	29C
17G	22G	29G
17T	22T	28T
17C	22C	29C
19G	22G	30G
20G	23G	31G
20T	23T	32T
20C	23C	32C
21A	24A	32A
21G	25G	32G
21C	25C	34C
20T	25T	35T
21T	26T	36T
22C	26C	36C
23T	25T	36T
24T	26T	36T
24G	26G	36G
25T	27T	38T
25G	27G	39G
27C	27C	39C
27T	28T	41T
27C	28C	41C
28A	28A	42A
28C	29C	43C
26T	30T	43T
28C	29C	45C
28A	30A	44A
28C	30C	44C
28A	30A	43A
28C	30C	44C
29A	30A	46A
29C	31C	46C
29A	31A	48A
29C	31C	46C
27A	31A	47A
26C	36C	47C
27A	34A	47A
20A_31C	35C	47C
27A	35A	47A

27C	35C	45C
27A	37A	48A
25C	33A	15A_32C
29A	37A	49A
29C	38C	48C
29A	36A	48A
29C	38C	45C
29A	39A	49A
29C	40C	48C
29A	39A	49A
29C	40C	49C
28A	27A_13G	47A
28C	40C	45C
28A	36A	41A
30C	40C	41C
27A	38A	41A
8A_8G_33C	38C	43C
17A	38A	38A
17C	40C	36C
16A	45A_34G_39C	23A_8G
27C	36A_57C	16A_43C
29A	23A	26A
27C	23C	26C
27A	22A	22A
27C	20T_1G_23C	7A_35T_24G_29C
26G	42G	38G
26T	42T	39T
30G	42G	40G
30T	44T	41T
31G	46G	42G
11A_32T_11G_11C	46T	42T
23G_11C	48G	42G
4A_6T_4C	48C	44C
17A	48A	44A
31T	48T	45T
32G	49G	44G
32T	50T	43T
34G	50G	43G
34T	51T	42T
34G	50G	40G
34T	50T	40T
34G	51G	39G
34T	30T_1G_23C	36T

35G	53G	36G
33T	53C	36C
36G	54G	35G
36C	54C	37C
35G	54G	37G
35C	55C	39C
35A	55A	38A
35C	56C	38C
34G	56G	38G
34C	56C	38C
34A	55A	38A
34A	55A	38A
34C	54C	39C
34A	53A	40A
35G	52G	42G
35C	51C	42C
35A	53A	40A
35A	53A	42A
36T	53T	43T
35G	55G	44G
34T	54T	45T
36A	54A	45A
36C	54C	47C
35T	54T	50T
35G	54G	49G
37G	55G	50G
37G	55G	51G
35A	53A	51A
37T	53T	50T
37T	53T	53T
36G	50G	53G
37T	52T	55T
37G	52G	55G
36C	52C	56C
37C	28A_24C	56A
38A	52A	57A
39C	53C	57C
39A	53A	58A
39G	53G	59G
40C	53C	58C
40C	52C	57C
40A	52A	58A
40G	53G	60G

39G	53G	62G
41A	53A	64A
39A	53A	62A
38A	53A	62A
38G	55G	60G
36A	56A	61A
36G	56G	59G
35G	56G	58G
32T	53T	58T
33G	56G	58G
32G	57G	57G
31T	56T	57T
34A	55A	57A
34T	55T	55T
34G	56G	58G
34G	57G	59G
34G	58G	56G
34G	59G	55G
33A	59A	55A
34G	58G	56G
36A	58A	56A
35G	58G	57G
36G	58G	58G
37T	56T	61T
38G	60G	61G
38C	31A_28C	30A_31C
38A	59A	62A
37A	60A	62A
38A	60A	62A
38T	59T	61T
38T	59T	61T
39C	60C	63C
40T	63T	63T
40C	64C	64C
42T	61T	64T
42A	61A	64A
42G	61G	63G
44T	61T	62T
45T	61T	63T
44A	59A	62A
44T	60T	62T
44T	60T	63T
45G	58G	65G

45G	58G	65G
45C	57C	63C
46A	60A	63A
48A	60A	62A
48T	61T	63T
48T	61T	62T
50A	61A	62A
50C	61C	60C
50A	59A	59A
50G	58G	60G
50C	58C	58C
51C	58C	59C
52T	29A_28T	59A
50A	57A	58A
49C	59C	58C
49A	58A	56A
49C	59C	59C
49A	59A	59A
48C	58C	58C
48A	57A	59A
47A	55A	59A
47T	56T	61T
45A	55A	60A
45A	55A	63A
45A	54A	64A
45G	54G	64G
45G	54G	62G
43C	53C	59C
43G	54A	62A
45A	53A	62A
47C	54C	62C
47A	54A	62A
46G	53G	63G
47T	52T	64T
45T	52T	66T
46C	53C	68C
46T	53T	69T
46C	54C	70C
47A	54A	70A
47G	53G	71G
47C	53C	72C
47A	52A	69A
47C	52C	67C

47C	53C	68C
48G	25A_1T_27G	31A_37G
48G	53G	69G
47A	52A	68A
49G	53G	68G
48G	53G	69G
48C	52C	70C
48T	51T	66T
49G	50G	69G
48T	49T	70T
48G	48G	69G
47A	49A	69A
46A	47A	71A
45G	47G	72G
46A	47A	70A
45T	47T	69T
43C	46C	69C
44A	47A	69A
44A	49A	68A
45A	47A	69A
46T	49T	72T
47C	49C	73C
47A	48A	73A
47C	47C	74C
46T	49T	74T
47T	49T	73T
47C	49C	72C
47C	48C	72C
47T	48T	72T
47G	48G	72G
46G	47G	73G
47T	48T	71T
48G	47G	72G
48C	46C	74C
48A	46A	73A
46C	46C	74C
46C	47C	73C
47A	47A	73A
48A	46A	72A
49G	45G	72G
49T	45T	71T
49C	46C	72C
49A	48A	71A

50G	49G	68G
50G	48G	68G
52T	48T	68T
50G	48G	69G
52G	48G	69G
52A	47A	68A
52G	64G	67G
52G	27G	66G
51G	46G	65G
51G	46G	65G
49G	46G	64G
48C	45C	66C
48C	47C	62C
47A	47A	62A
47T	47T	63T
45G	46G	63G
46G	45G	64G
48G	45G	63G
48C	45C	66C
48C	46C	64C
48A	44A	66A
48T	45T	66T
48G	45G	65G
47A	43A	61A
47G	42G	65G
46C	41C	66C
45A	40A	66A
45C	39C	66C
45C	39C	67C
43A	40A	68A
44G	39G	69G
44T	39T	69T
42T	39T	69T
43G	38G	67G
42C	38C	69C
42A	38A	67A
41G	37G	68G
39C	36C	67C
39C	37C	67C
39T	36T	65T
38C	36C	64C
37T	36C	64C
37T	36T	63T

37T	36T	62T
38T	36T	61T
40G	36G	63G
40G	36G	64G
40T	37T	63T
41T	35T	62T
41T	35T	65T
41T	34T	66T
43C	34C	65C
44T	34T	64T
44C	33C	64C
44T	34T	64T
43G	34G	65G
45G	34G	65G
45G	33G	65G
45A	34A	65A
45A	34A	65A
45G	34G	62G
46G	34G	63G
46T	34T	62T
45C	34C	62C
43T	32T	62T
44G	32G	67G
45G	31G	66G
44T	32T	66T
46G	31G	65G
47A	31A	65A
47A	32A	65A
47A	32A	65A
46G	31G	65G
46A	33A	64A
47A	33A	63A
47G	33G	61G
47A	34A	62A
48A	35A	62A
49G	37G	66G
49A	37A	66A
49A	38A	67A
49C	38C	65C
50C	38C	66C
51A	38A	67A
51C	38C	67C
50C	38C	66C

51A	39A	63A
52T	38T	64T
52C	39C	61C
52A	39A	61A
52C	39C	60C
53C	41C	61C
53A	42A	61A
54G	42G	61G
53G	41G	58G
56T	41T	57T
58G	42G	58G
56G	39G	58G
56A	39A	57A
55T	38T	57T
54G	37G	57G
55G	39G	57G
54A	40A	57A
54A	40A	57A
53G	42G	55G
53C	42C	58C
54T	41T	56T
56T	42T	56T
56T	42T	56T
55C	42C	57C
56T	41T	58T
56A	40A	57A
56A	42A	57A
56C	40C	57C
55A	41A	60A
55C	42C	59C
54A	42A	60A
54T	42T	60T
54G	42G	59G
54C	40C	57C
55A	39A	55A
54C	38C	57C
53A	37A	57A
53T	37T	59T
53G	37G	59G
52C	37C	61C
51A	37A	60A
51T	35T	60T
51C	36C	60C

51C	36C	60C
52A	35A	62A
52T	37T	62T
51G	37G	63G
52A	37A	63A
52A	38A	68A
52G	39G	64G
52G	20A_21G	31A_33G
51G	41G	65G
52A	41A	66A
54A	41A	66A
54A	42A	68A
54A	43A	68A
55G	43G	68G
54A	43A	66A
54A	43A	66A
53C	42C	69C
54T	42T	70T
56G	21T_21G	68G
55T	42T	65T
57C	41C	64C
57A	45A	35A_28C
30T_27C	46C	64C
57T	46T	62T
58A	47A	63A
58A	48A	62A
58A	48A	62A
59T	49T	62T
59T	49T	65T
58T	48T	65T
59C	48C	66C
59T	48T	64T
59T	48T	62T
59G	48G	62G
59A	48A	61A
58T	48T	62T
58C	49C	63C
55T	49T	63T
55T	50T	62T
55G	50G	62G
55A	49A	60A
56G	48G	59G
54T	49T	61T

53G	50G	60G
54T	50T	60T
57G	49G	59G
56A	49A	58A
55G	49G	61G
54A	72A	61A
57A	26A	63A
57A	51A	62A
58A	51A	62A
57A	50A	63A
31A_27T	50A	24A_39T
58A	50A	64A
59A	51A	61A_21C
58A	50A	61A
56A	52A	60A
57C	52C	58C
55A	52A	58A
54C	53C	59C
54A	53A	57A
55T	52T	58T
56G	52G	56G
57G	51G	58G
57A	49A	58A
58T	49T	55T
59T	50T	55T
60T	49T	57T
58T	48T	57T
57C	47C	55C
58T	45T	58T
58T	45T	56T
58T	46T	60T
59C	47C	57C
59C	47C	55C
59T	47T	55T
60T	46T	54T
59C	46C	53C
59A	47A	53A
59G	49G	52G
59C	48C	51C
58G	48G	51G
58C	48C	51C
58A	47A	52A
57A	46A	51A

58G	47G	49G
57A	46A	49A
55G	47G	49G
55T	46T	48T
56G	47G	48G
56A	47A	50A
56G	48G	49G
56A	48A	47A
58C	47C	47C
59A	44A	48A
59A	44A	48A
58G	44G	48G
57G	45G	46G
55A	46A	47A
55G	46G	48G
56C	46C	48C
55A	47A	48A
55A	47A	50A
55C	47C	50C
56T	50T	53T
56C	49C	52C
55T	49T	51T
56T	49T	52T
54G	47G	52G
56C	48C	52C
55T	49T	51T
25G_30C	50C	51C
55C	50C	51C
55A	50A	51A
56G	51G	53G
55T	53T	51T
56C	53C	49C
59T	53T	49T
59C	53C	49C
61T	52T	51T
61G	53G	50G
61C	53C	49C
62A	53A	50A
62A	52A	50A
63T	52T	51T
62G	51G	49G
62T	25T_24C	49C
62C	50C	50C

63T	50T	52T
61T	52T	51T
59T	55T	55T
59G	56G	55G
59C	56C	54C
59A	55A	50A
59C	56C	51C
60A	56A	51A
60A	57A	50A
59A	57A	50A
58A	57A	50A
57C	57C	51C
57A	57A	48A
31T_26C	55T	48T
58G	55G	44G
56A	56A	45A
57A	57A	45A
58C	56C	46C
57C	55C	46C
58C	56C	47C
58T	56T	48T
58T	54T	50T
58T	55T	51T
58T	56T	51T
59C	56C	55C
59T	56T	54T
59C	56C	56C
58A	56A	58A
59G	56G	59G
59A	57A	60A
59G	56G	60G
58G	55G	61G
58A	55A	59A
58G	56G	60G
58C	57C	62C
57A	57A	62A
55A	56A	61A
55A	56A	61A
54G	56G	60G
55C	55C	61C
55A	57A	59A
57C	57C	60C
57A	57A	61A

57C	57C	64C
56A	57A	64A
57A	58A	65A
56G	58G	63G
55G	59G	64G
56G	58G	67G
54C	59C	66C
55A	59A	67A
55C	57C	67C
57C	58C	66C
58C	60C	65C
57T	61T	66T
56C	61C	67C
57A	61A	67A
56A	61A	69A
54G	60G	69G
54C	60C	68C
55A	60A	67A
57G	58G	65G
56A	59A	66A
56G	59G	66G
55A	58A	67A
53A	58A	66A
52A	58A	65A
54G	60G	65G
54G	60G	65G
53C	60C	66C
54C	59C	64C
54G	32A_25G	65G
56G	57G	63G
56G	57G	63G
55T	58T	65T
56G	58G	65G
56A	59A	66A
54T	59T	66T
54T	60T	66T
54T	59T	64T
54T	59T	63T
54T	60T	62T
54C	60C	63C
55A	59A	65A
55A	60A	66A
53A	60A	65A

53A	60A	67A
53A	60A	67A
53G	60G	68G
53G	59G	33T_35G_1C
53T	57T	70T
53G	58G	68G
52C	59C	69C
53A	59A	69A
54A	60A	66A
54G	60G	66G
53C	57C	63C
53C	58C	62C
53A	58A	63A
52C	58C	63C
51C	57C	64C
52T	57T	64T
52G	58G	66G
52T	57T	68T
53C	57C	66C
53A	57A	66A
54A	58A	66A
55A	56A	67A
55C	56C	67C
52A	57A	65A
52C	59C	64C
50A	58A	63A
51C	60C	65C
50T	60T	61T
49T	60T	61T
49A	60A	63A
50A	60A	62A
50T	62T	62T
50C	62C	61C
52C	61C	61C
50T	61T	60T
51T	61T	60T
51A	59A	61A
52A	55A	62A
51A	56A	59A
53A	54A	63A
53G	54G	62G
52T	53T	63T
52C	53C	66C

52C	52C	67C
53A	54A	67A
52T	55T	67T
52G	55G	66G
52C	55C	66C
51A	54A	64A
53A	53A	63A
26T_27C	54C	67C
52G	53G	69G
52A	53A	69A
51A	54A	68A
52A	53A	38A_30G
52C	55C	67C
53A	54A	66A
53G	54G	66G
52C	54C	67C
54A	54A	68A
54G	56G	69G
55C	58C	71C
54C	59C	72C
54A	58A	72A
54C	58C	72C
55T	59T	72T
54G	60G	74G
53T	59T	73T
53A	58A	72A
53A	55A	37A_34T
53T	57T	71T
52T	57T	70T
52T	56T	70T
52T	57T	68T
49T	56T	68T
50C	56C	70C
49A	56A	70A
50C	56C	70C
26A_25G	56G	70G
51A	55A	71A
51G	55G	72G
50T	54T	71T
52A	55A	69A
51C	53C	68C
51C	53C	68C
51T	54T	68T

50T	55T	67T
50G	56G	64G
51G	55G	65G
50A	55A	66A
50T	55T	66T
49T	56T	66T
50T	57T	68T
51A	57A	67A
50G	58G	67G
47C	59C	67C
48A	61A	66A
48C	61C	68C
48C	61C	67C
48A	60A	66A
47C	61C	38T_28C
46G	58G	66G
45C	59C	65G
45T	59T	65T
45C	60C	65C
46A	60A	65A
45A	61A	65A
47A	61A	65A
46A	60A	66A
48T	61T	64T
49T	60T	64T
48A	60A	60A
48G	59G	58A
47T	58T	58T
48A	60A	59A
47G	60G	59G
47G	59G	59G
46G	60G	62G
47A	61A	63A
48G	61G	64G
47C	61C	63C
47T	62T	63T
47T	65T	63T
48G	65G	65G
48C	66C	65C
48G	67G	65G
48A	66A	63A
49G	65G	63G
48A	66A	60A

48G	66G	57G
49A	67A	58A
49G	67G	61G
50T	68T	60T
50G	69G	61G
50G	69G	60G
51G	70G	61G
51A	70A	61A
50A	70A	61A
50G	71G	63G
50A	70A	65A
48A	71A	62A
47G	72G	63G
47A	74A	62A
47T	74T	61T
46A	72A	63A
48A	71A	61A
48C	71C	61C
50C	72C	60C
50A	72A	63A
50T	72T	63T
47G	74G	63G
48C	74C	64C
48C	73C	68C
48T	74T	69T
48A	72A	69A
48G	69G	69G
48C	69C	68C
48T	69T	71T
47T	70T	71T
46T	70T	71T
47T	70T	72T
48A	70A	73A
49T	72T	73T
51T	73T	36T_37C
50G	73G	73G
50G	72G	73G
49T	72T	75T
50G	70G	73G
50G	70G	73G
48C	71C	74C
48A	69A	77A
48G	69G	76G

48G	70G	76G
51A	72A	76A
50A	72A	76A
50T	70T	76T
50A	70A	76A
50C	71C	78C
48C	71C	78C
48T	71T	77T
50T	71T	78T
50T	70T	80T
52A	69A	80A
52T	68T	81T
52G	70G	83G
52T	69T	83T
53T	69T	86T
53C	69C	84C
53T	69T	86T
53G	69G	87G
53A	69A	82A
53G	70G	83G
53G	72G	85G
52A	71A	82A
52C	70C	85C
50C	71C	85C
50A	70A	86A
49A	71A	86A
48A	71A	86G
47A	71A	89A
47C	69C	90C
47A	70A	86A
46C	69C	86C
46A	70A	86A
46A	72A	85A
46A	74A	87A
47A	73A	85A
47T	72T	83T
46C	75C	84C
46A	72A	82A
46G	75G	82G
46A	74A	81A
45G	73G	82G
44A	74A	81A
46A	74A	81A

46A	73A	84A
45T	71T	83T
46C	71C	82C
46C	72C	81C
48A	72A	83A
48G	71G	83G
48T	72T	82T
48T	73T	80T
49C	72C	80C
48T	73T	79T
47A	73A	80A
46G	71G	80G
45A	72A	79A
45A	74A	78A
44G	74G	79G
43A	74A	82A
44A	74A	80A
45G	75G	79G
45T	74T	79T
46G	73G	82G
48C	74C	82C
51T	76T	79T
52C	75C	79C
53T	76T	77T
52T	76T	79T
53T	77T	77T
54C	76C	78C
55A	74A	77A
54A	73A	76A
54G	73G	79G
54T	72T	81T
55G	72G	81G
53A	72A	79A
53C	71C	82C
53A	73A	82A
53A	72A	82A
55A	72A	81A
54A	73A	80A
54A	73A	80A
54A	73A	78A
54G	72G	78G
54G	73G	74G
53T	73T	76T

54G	73G	73G
53T	72T	75T
54A	71A	75A
53A	70A	77A
53T	68T	75T
54T	67T	76T
54A	67A	73A
53T	67T	79T
52A	67A	79A
51A	65A	81A
52A	64A	81A
53A	63A	78A
53T	61T	76T
52C	62C	75C
52A	64A	77A
51A	64A	77A
52A	64A	79A
51T	64T	76T
51T	64T	77T
51A	65A	78A
51A	64A	76A
50A	65A	77A
50A	65A	78A
48G	66G	78G
49G	66G	81G
48A	63A	82A
49G	63G	80G
49A	63A	77A
50G	65G	78G
50G	65G	33T_41G_1C
50T	64T	73T
54A	63A	70A
54T	64T	68T
55C	65C	68C
55G	61G	31T_36G
54G	62G	74G
54T	61T	77T
53G	62G	78G
53T	62T	78T
54C	61C	78C
56G	62G	80G
58T	62T	78T
57C	62C	75C

56T	63T	78T
56C	62C	80C
58C	63C	80C
57A	62A	79A
57T	63T	81T
57T	62T	81T
57G	62G	82G
58T	61T	82T
58C	64C	82C
59T	64T	80T
59C	64C	80C
59T	62T	80T
59G	62G	81G
60G	63G	80G
60A	61A	79A
60A	62A	79A
61G	63G	78G
62A	63A	74A
63C	63C	74C
63A	63A	76A
63A	64A	76A
63A	64A	76A
63C	64C	76C
64A	62A	77A
65A	59A	77A
65C	58C	77C
33A_33G	56A	77A
66C	55C	76C
68C	54C	73C
68T	57T	77T
68T	57T	75T
68C	55C	76C
69C	56C	79C
67T	57T	80T
67G	61G	78G
67T	61T	76T
67G	62G	76G
69T	62T	76T
67A	63A	76A
70T	63T	79T
69A	64A	79A
71T	62T	78T
70G	64G	77G

70G	64G	75G
72A	63A	79A
73A	65A	80A
73T	64T	80T
74T	64T	80T
75C	65C	80C
76T	65T	81T
75G	65G	81G
75G	64G	79G
75A	63A	80A
74G	66G	79G
70T	64T	79T
70T	63T	80T
69G	64G	79G
67T	63T	79T
66G	62G	79G
68A	62A	78A
69A	63A	77A
68T	61T	78T
69A	63A	79A
70G	63G	77G
70C	61C	78C
70A	64A	78A
70A	65A	79A
71T	64T	77T
72A	62A	79A
71T	61T	78T
69A	60A	77A
70G	56G	77G
70A	58A	80A
70T	58T	79T
69C	57C	78C
69A	57A	78A
69C	58C	81C
70A	59A	81A
74T	60T	79T
74T	61T	79T
74G	62G	79G
76C	63C	79C
75T	62T	80T
76G	63G	80G
77G	63G	79G
78G	63G	79G

77G	63G	80G
77A	63A	79A
76G	62G	82G
76G	64G	45A_37G
77A	62A	83A
77A	60A	81A
78A	60A	80A
77G	64G	82G
77C	64C	82C
78A	64A	84A
78G	63G	45A_39G
78T	63T	84T
79A	63A	87A
79A	63A	83A
80A	62A	85A
78G	62G	82G
79A	62A	78A
78T	61T	78T
78A	62A	74A
78A	64A	74A
75A	63A	77A
77C	63C	77C
73C	64C	78C
73C	65C	74C
72G	64G	74G
72G	64G	74G
73T	65T	75T
73T	67T	78T
74C	68C	76C
76C	68C	76C
76T	68T	75T
72G	68G	75G
70A	67A	74A
69A	67A	75A
71C	66C	75C
68C	66C	74C
66A	66A	72A
66A	65A	72A
66G	65G	71G
66C	65C	70C
66A	65A	71A
65G	64G	74G
65C	63C	74C

65C	63C	76C
65C	63C	75C
66T	64T	80T
66G	64G	79G
65G	63G	78G
65T	65T	76T
66G	67G	77G
66A	68A	77A
65A	70A	79A
64A	70A	76A
64C	70C	76C
65T	70T	75T
66T	72T	74T
68A	72A	71A
68C	72C	71C
67C	73C	71C
66C	73C	70C
64A	74A	69A
64G	75G	71G
62A	76A	72A
63C	74C	74C
63T	74T	74T
62G	74G	75G
61A	73A	73A
62A	73A	73A
62G	70G	73G
62A	70A	74A
62G	69G	73G
61A	68A	72A
63A	68A	71A
60T	67T	71T
60A	67A	72A
61A	66A	73A
62G	67G	74G
62G	66G	73G
61A	67A	73A
61A	66A	74A
62T	66T	74T
62T	65T	74T
61T	64T	71T
60C	63C	39T_33C
60A	64A	71A
60A	65A	72A

60G	65G	72G
60C	63C	72C
60A	63A	72A
59G	63G	70G
59A	62A	68A
60C	62C	67C
60A	62A	70A
59A	62A	71A
57A	61A	74A
59C	64C	74C
58T	63T	74T
60C	62C	74C
60C	64C	73C
59T	63T	73T
60G	62G	77G
59C	63C	78C
58T	63T	76T
59T	63T	77T
60G	64G	76G
59T	64T	77T
60C	63C	77C
60T	63T	78T
60A	65A	79A
60C	64C	79C
60T	64T	79T
59T	62T	76T
55G	61G	77G
55G	61G	74G
55A	60A	78A
53G	58G	73G
53G	59G	73G
52G	59G	71G
52A	59A	73A
51G	59G	74G
51T	59T	74T
51G	60G	74G
51C	60C	75C
51A	60A	75A
51A	60A	77A
51G	59G	78G
51T	60T	78T
51G	56G	78G
52A	56A	77A

51A	56A	79A
51G	56G	77G
50G	56G	76G
52A	56A	75A
52C	56C	74C
50C	57C	74C
53A	57A	75A
51G	57G	75G
51C	55C	75C
53T	55T	75T
52C	54C	72C
53T	54T	73T
52T	54T	75T
52G	53G	74G
51C	52C	74C
51G	52G	74G
51C	52C	73C
49C	51C	73C
48C	49C	73C
48G	49G	72G
47C	51C	40T_31C
48A	51A	69A
48G	50G	69G
48C	50C	71C
47A	48A	71A
47C	50C	70C
47C	49C	69C
47A	50A	70A
26A_20G	52G	29A_43G
46G	52G	74G
47T	52T	75T
47T	52T	78T
47G	55G	78G
46T	54T	78T
45G	57G	78G
45C	56C	80C
44A	53A	79A
44G	56G	80G
43G	57G	77G
44A	56A	73A
43T	54T	75T
42G	54G	73G
42G	52G	73G

43A	52A	75A
42C	54C	76C
42C	52C	76C
41C	52C	78C
39G	51G	79G
39G	51G	79G
39C	50C	80C
40T	50T	79T
40T	51T	78T
40G	50G	78G
40G	49G	77G
39G	49G	77G
42T	49T	74T
42G	49G	75G
43G	50G	79G
43C	50C	81C
43A	52A	81A
43A	53A	80A
43C	53C	79C
42C	52C	78C
42G	52G	77G
42T	52T	76T
44T	51T	74T
43G	52G	73G
43A	51A	69A
43G	51G	76G
43A	50A	78A
41G	50G	76G
40G	51G	76G
40T	51T	76T
44C	51C	76C
44C	52C	75C
45A	52A	76A
47C	52C	75C
48C	52C	78C
48T	53T	78T
49A	53A	78A
50G	53G	79G
50G	53G	80G
50T	53T	78T
50T	53T	78T
50G	53G	76G
51G	51G	76G

50G	50G	76G
50G	50G	76G
45A	50A	75A
47A	50A	77A
48G	50G	78G
46A	49A	81A
47A	49A	83A
48C	50C	83C
47A	51A	81A
47G	50G	84G
47A	50A	85A
46G	50G	88G
46A	49A	85A
47G	47G	85G
47G	47G	84G
50G	45G	81G
50G	46G	81G
49A	43A	80A
52G	45G	79G
50A	44A	74A
53T	45T	74T
53T	44T	74T
53C	45C	37G_39C
53A	46A	76A
53G	46G	75G
53G	46G	75G
53A	44A	75A
53C	45C	73C
54T	46T	71T
54A	47A	70A
54C	47C	67C
53C	47C	66C
53C	47C	65C
52A	48A	63A
54A	47A	63A
54C	47C	63C
54C	48C	64C
52T	49T	64T
52C	49C	65C
53G	46G	33A_33G
51T	48T	67T
52T	51T	63T
52G	50G	61G

50C	50C	62C
50C	51C	61C
49C	51C	61C
49T	53T	61T
49G	53G	62G
49G	52G	63G
50G	53G	63G
51G	54G	65G
51A	54A	65A
51A	54A	63A
53G	52G	63G
27G_26C	51G	63G
51G	51G	62G
50C	53C	61C
50C	53C	63C
25A_24G_1C	53A	28A_35G
51G	53G	64G
52C	52C	63C
52C	52C	61C
55A	49A	60A
55T	50T	60T
54C	50C	61C
54A	51A	60A
54G	50G	60G
56A	50A	60A
58A	48A	61A
58A	48A	59A
58G	48G	59G
56A	43A	60A
58C	45C	61C
58C	45C	60C
58T	46T	58T
59C	44C	57C
60C	46C	55C
61C	46C	54C
61A	46A	55A
61T	47T	53T
61C	46C	51C
61G	46G	20T_27G
60G	46G	48G
60A	46A	50A
59T	45T	52T
59G	44G	53G

59C	46C	27T_29C
61A	46A	60A
61G	48G	61G
59G	49G	63G
61G	50G	64G
60T	50T	64T
64T	49T	65T
64T	48T	68T
64T	47T	68T
64A	47A	70A
64A	48A	69A
64A	49A	73A
65T	48T	72T
65T	50T	76T
65T	48T	76T
64T	49T	75T
65A	49A	75A
64G	49G	76G
64A	50A	76A
64A	49A	77A
65T	50T	78T
62G	49G	78G
62T	48T	77T
62A	47A	76A
61A	48A	77A
64A	48A	75A
64T	47T	75T
63C	47C	75C
63T	47T	76T
63A	47A	75A
62G	47G	75G
63C	48C	76C
64A	48A	74A
65G	48G	75G
65T	48T	76T
65T	49T	74T
66C	51C	74C
65A	51A	75A
67G	52G	73G
67G	52G	73G
67G	53G	74G
68A	54A	73A
68C	53C	73C

67C	53C	71C
67A	53A	71A
65G	54G	71G
66A	55A	72A
65C	55C	74C
65A	54A	75A
62T	56T	75T
64T	56T	74T
64T	57T	74T
62T	57T	75T
62C	57C	74C
62T	57T	75T
62C	57C	75C
61C	58C	76C
61C	58C	75C
61T	59T	77T
61C	59C	74C
60T	61T	73T
60C	62C	72C
26A_33G	33A_29G	72A
58G	61G	70G
59G	60G	71G
59G	61G	71G
59A	60A	71A
60T	64T	68T
59G	64G	67G
58T	64T	69T
58G	63G	68G
58T	63T	69T
58G	63G	69G
57T	62T	68T
27A_32G	63A	69A
59T	63T	69T
59G	64G	68G
60T	64T	66T
60T	63T	66T
60T	63T	64T
29T_31C	36T_26C	65T
61G	62G	63G
30T_31C	37T_26C	65T
60G	63G	62G
60T	62T	63T
60A	62A	63A

59G	61G	65G
58G	61G	65G
57T	59T	68T
57T	58T	69T
59C	57C	70C
59C	57C	71C
59T	57T	73T
59C	57C	75C
58C	57C	76C
84A_42G_184C	75A_39G_170C	75C
68C	65C	77C
61T	58T	78T
63G	59G	77G
64G	60G	77G
64A	59A	76A
63C	59C	77C
61A	59A	77A
61A	60A	75A
61C	61C	75C
61A	61A	75A
61C	61C	74C
60C	62C	74C
62T	61T	76T
64T	60T	77T
64A	60A	78A
61C	60C	79C
61A	59A	79A
62A	59A	79A
60G	58G	79G
61A	58A	80A
61A	57A	78A
62T	59T	77T
62G	58G	76G
61G	27T_31G	77G
60C	58C	78C
59A	57A	78A
59C	56C	79C
59A	56A	80A
58A	54A	81A
60C	55C	80C
59T	55T	79T
60G	55G	80G
60G	55G	79G

59A	55A	77A
59A	55A	78A
60G	57G	78G
60G	57G	77G
60A	58A	79A
58C	59C	79C
28A_31C	59C	81A
59T	60T	81T
59A	61A	82A
60A	59A	83A
59A	60A	80A
58A	62A	79A
57A	61A	78A
57G	62G	79G
57T	63T	80T
55G	59G	78G
53T	59T	75T
52G	59G	76G
52C	59C	76C
52C	58C	76C
49T	57T	76T
51T	58T	75T
55T	57T	73T
55T	57T	72T
54T	56T	71T
55T	56T	70T
56G	56G	69G
55G	55G	67G
54A	56A	67A
53A	54A	63A
53A	55A	63A
53G	54G	64G
54G	53G	62G
53G	53G	61G
54G	53G	59G
54G	53G	60G
55C	52C	61C
56A	52A	61A
56T	52T	61T
56C	51C	61C
54C	53C	63C
56C	52C	64C
56G	54G	62G

56C	54C	63C
56A	54A	64A
55G	54G	65G
57A	48A	66A
57G	51G	67G
57G	51G	67G
57C	51C	66C
56C	50C	67C
55A	48A	67A
57C	49C	68C
57C	26T_22C	69C
57T	49T	71T
56G	49G	71G
55G	49G	70G
56C	49C	72C
54C	48C	72C
54A	48A	71A
55A	48A	69A
54A	47A	69A
54G	48G	69G
55T	49T	67T
55G	49G	66G
54G	49G	64G
53A	46A	63A
53T	45T	62T
52T	45T	62T
52C	45C	63C
52T	42T	63T
49C	42C	63C
49A	43A	64A
49T	43T	63T
49G	43G	62G
48T	43T	60T
48T	42T	60T
48A	43A	59A
48T	43T	60T
48C	43C	58C
48C	43C	58C
46A	43A	56A
46C	45C	58C
46C	44C	57C
46T	47T	55T
46C	47C	55C

19A_27G	25A_22G	55A
46G	48G	55G
46G	48G	53G
44C	47C	55C
43C	46C	55C
43G	46G	54G
44G	46G	55G
19A_25G	25A_21G	53A
45G	46G	54G
44A	45A	54A
48A	45A	55A
49G	47G	55G
52T	46T	55T
52C	47C	55C
50A	48A	53A
52C	49C	51C
55T	50T	51T
55C	50C	52C
53C	49C	52C
52A	50A	49A
52C	51C	49C
53T	51T	48T
51C	51C	46C
51C	51C	46C
51T	50T	44T
51G	50G	43G
50G	50G	43G
50A	50A	40A
49G_44C	49G_43C	40G_20C
5C	6C	18C
47G	44G	7A_20G
48C	45C	27C
30A_17G	46A	21A
47C	49C	31C
47G	49G	29G
48C	52C	31C
47C	51C	30C
47C	51C	30C
48A	52A	29A
49C	51C	32C
49C	53C	32C
49C	51C	32C
49A	52A	30A

32G_17C	23G_29C	30C
49C	54C	30C
48C	55C	30C
48T	55T	30T
47G	53G	31G
47A	54A	31A
47A	55A	32A
47T	55T	32T
47A	59A	34A
48T	60T	35T
49A	61A	36A
49T	29T_32C	36C
50G	61G	33G
50C	60C	36C
48C	59C	36C
45A	59A	38A
45C	59C	39C
45C	59C	41C
45C	59C	43C
45A	59A	42A
46C	60C	44C
47C	58C	47C
46T	58T	48T
46G	57G	48G
46C	57C	48C
44C	58C	47C
44C	59C	47C
43A	29A_30G	47A
42T	59T	47T
42A	59A	49A
42T	58T	48T
42A	61A	49A
43G	61G	48G
43A	31A_29G	49A
42T	60T	49T
43C	60C	48C
42G	30A_31G	49G
42T	62T	49T
42G	62G	54G
42C	31T_31C	56C
46T	61T	58T
48G	59G	57G
25A_23G	58G	56A

48C	62C	57C
48A	62A	59A
46G	63G	59G
47G	63G	59G
47A	62A	59A
47A	62A	59A
47G	62G	57G
48T	60T	58T
49G	62G	60G
48T	62T	61T
50G	62G	63G
50A	61A	60A
49C	63C	62C
26A_24G	63A	21A_40G
49T	64T	60T
50C	63C	64C
50A	61A	66A
51A	61A	67A
52T	62T	68T
52T	63T	71T
53G	65G	74G
54T	65T	73T
54G	65G	73G
54A	66A	72A
25T_28C	66C	45T_1G_28C
54G	67G	74G
55A	68A	74A
59T	67T	74T
59G	69G	76G
59G	71G	76G
59A	71A	75A
61G	70G	77G
62A	72A	77A
64A	73A	77A
66A	71A	79A
71T	72T	77T
71G	72G	78G
71A	74A	77A
71G	74G	77G
71C	74C	80C
72A	75A	77A
75C	37A_38C	79C
74T	75T	78T

75C	74C	78C
74G	74G	77G
75G	76G	75G
73A	76A	77A
73G	76G	77G
71C	77C	28T_45C
69T	78T	44T_28G
68C	77C	77C
68T	76T	76T
65C	75C	77C
65T	75T	79T
66C	74C	79C
66T	74T	80T
67C	72C	79C
65T	75T	78T
66T	76T	79T
66T	76T	80T
66A	76A	79A
64T	77T	80T
63C	74C	80C
64T	74T	78T
64A	73A	78A
63A	72A	75A
62G	71G	71G
63C	71C	73C
62A	71A	73A
62T	69T	73T
62C	70C	72C
62A	71G	39A_32G
60C	72C	72C
61T	72T	70T
61G	71G	71G
61G	71G	74G
61A	70A	75A
61T	69T	74T
58T	69T	59T
58A	68A	59A
58G	69G	58G
57A	69A	58A
58A	68A	58A
54G	1A_27G_36C	21G_36C
52C	62C	57C
53A	60A	56A

52A	26A_34C	21A_35C
53C	59C	58C
52C	33T_26C	34T_22C
59T	61T	71T
59T	61T	74T
59C	61C	74C
59T	61T	75T
59C	62C	75C
59T	62T	74T
60G	61G	76G
59G	60G	76G
60T	60T	74T
60C	59C	76C
61T	59T	76T
61A	61A	76A
64G	60G	76G
65C	60C	75C
65A	61A	74A
67A	61A	75A
67G	60G	74G
67G	62G	73G
66C	63C	74C
67C	46T_1G_83C	76C
67C	38C	75C
67T	36T	72T
67T	36T	75T
67C	57C	72C
67C	57C	73C
67T	59T	72T
67T	59T	73T
68A	61A	72A
64A	62A	70A
62C	62C	69C
62G	28A_34G	69G
62T	63T	70T
63A	29A_32G	70A
62T	61T	71T
61G	61G	69G
62A	61A	70A
63C	62C	70C
62T	33T_29C	69T
61A	60A	69A
62A	60A	68A

62G	60G	70G
62G	60G	69G
62T	61T	69T
63G	61G	69G
62C	61C	68C
61A	61A	65A
60C	61C	66C
60A	62A	66A
62T	62T	63T
61T	60T	65T
60T	60T	64T
62T	61T	64T
61T	62T	20A_65T_9G
61T	21A_59T	9A_43T_12G
61A	28A_33T	69A
63A	60A	66A
63A	59A	63A
59A	59A	60A
57A	59A	59A
57A	57A	60A
57A	55A	60A
57C	54C	18A_38C
56A	54A	57A
54G	55G	57G
53A	55A	57A
52T	56T	57T
49C	56C	56C
46T	57T	56T
46G	55G	55G
45T	55T	55T
46A	55A	54A
45T	53T	52T
44T	53T	52T
43C	53C	51C
43C	53C	54C
43T	53T	53T
44T	53T	49T
41C	28T_23C	51C
41T	51T	52T
41T	52T	53T
40T	50T	58T
40C	50C	58C
40C	51C	58C

40C	51C	57C
40A	51A	59A
39C	52C	57C
16A_23G	52G	22A_37G
39G	51G	60G
40C	50C	59C
39C	51C	58C
24T_16C	52C	59C
40C	52C	59C
40A	50A	58A
40G	51G	59G
39C	51C	60C
40T	51T	61T
40G	52G	62G
40T	52T	63T
39A	50A	63A
38C	52C	64C
39C	52C	65C
39T	51T	64T
39C	53C	63C
39C	52C	63C
38T	50T	63T
37G	52G	67G
38G	51G	69G
38C	53C	69C
37C	53C	69C
17G_20C	19A_33G	71G
37G	52G	71G
37T	52T	71T
37C	53C	70C
38A	54A	73A
38C	54C	73C
38C	53C	72C
38A	53A	73A
37G	52G	73G
38C	52C	73C
38A	52A	71A
38C	53C	71C
38C	53C	71C
38A	55A	70A
38T	55T	70T
38G	55G	69G
38C	55C	70C

37T	56T	70T
37G	55G	70G
37T	56T	70T
37G	57G	71G
36C	57C	72C
36T	56T	72T
33C	56C	70C
33C	56C	70C
33T	54T	71T
31T	53T	70T
31C	53C	71C
31C	53C	72C
32C	52C	74C
31A	53A	78A
30T	52T	78T
30A	51A	78A
31C	50C	79C
31T	49T	81T
30G	48G	78G
30C	47C	80C
30A	47A	81A
29C	46C	79C
30T	47T	78T
30T	47T	78T
29G	47G	73G
28G	47G	73G
29C	48C	73C
29G	48G	73G
29G	48G	73G
28A	47A	74A
28A	47A	73A
28G	47G	70G
28A	47A	73A
28T	48T	72T
28G	50G	72G
28T	50T	72T
26G	49G	71G
26G	50G	71G
25T	50T	69T
26C	50C	69C
27A	52A	67A
26A	50A	68A
26A	51A	66A

25A	51A	67A
25A	50A	66A
24C	49C	66C
25C	49C	65C
25A	48A	65A
24G	48G	65G
23C	48C	60C
24A	48A	58A
23G	46G	60G
23T	47T	61T
23T	47T	60T
23C	47C	59C
23G	49G	58G
23C	51C	58C
24T	49T	57T
24C	50C	56C
23C	49C	56C
24A	47A	54A
26G	46G	55G
26C	47C	54C
26A	47A	54A
26G	46G	55G
26T	46T	54T
26T	47T	53T
26C	47C	53C
26T	46T	54T
25G	46G	53G
25C	46C	53C
25A	46A	53A
25G	45G	53G
26C	47C	53C
27A	47A	54A
27G	51G	53G
27C	50C	55C
27T	50T	55T
27T	50T	53T
27C	50C	54C
27A	50A	53A
27T	53T	52T
26C	54C	51C
26T	53T	47T
26G	53G	47G
26G	52G	48G

27T	54T	47T
28C	54C	45C
28T	53T	46T
30C	52C	44C
31C	52C	44C
31G	53G	44G
32A	53A	44A
32C	53C	42C
31C	52C	43C
31T	53T	43T
30G	52G	44G
30G	53G	44G
30A	53A	42A
31A	51A	42A
30G	50G	41G
30A	50A	43A
30A	50A	42A
30G	50G	43G
30A	51A	42A
31A	49A	44A
31A	49A	47A
30G	48G	47G
31G	48G	49G
31C	47C	52C
31A	47A	51A
31G	47G	48G
30G	47G	49G
29G	46G	50G
30A	46A	50A
30G	46G	53G
31C	44C	54C
31A	45A	56A
31A	45A	57A
32A	22A_23C	57A
32G	44G	58G
33C	43C	56C
33C	43C	57C
33G	42G	56G
33G	44G	55G
33T	44T	57T
33G	44G	56G
32A	45A	58A
33G	46G	59G

34A	46A	59A
35A	47A	60A
35G	47G	59G
22G_12C	48C	33G_27C
34C	47C	62C
34G	46G	59G
34C	46C	60C
35T	45T	61T
37G	46G	62G
38G	47G	61G
38C	47C	63C
38C	46C	63C
38C	46C	66C
37T	46T	69T
38A	45A	67A
38C	47C	66C
38A	48A	66A
36C	47C	64C
14T_22C	25T_22C	27T_38C
36G	46G	66G
37C	48C	68C
36A	50A	74A
36A	50A	74A
37G	52G	75G
36G	51G	76G
36G	51G	78G
35C	50C	81C
36C	50C	82C
37A	51A	82A
37G	51G	83G
36A	50A	83A
36G	48G	82G
36G	48G	80G
35C	48C	83C
37T	48T	85T
37G	48G	84G
37C	48C	84C
36C	49C	83C
36A	49A	84A
36T	49T	84T
14A_22T	49T	34A_50T
35G	23A_26G	84G
35C	50C	82C

14A_21G	49G	32A_51G
37T	49T	82T
37G	48G	82G
37G	48G	79G
38A	46A	77A
38G	47G	78G
39A	47A	77A
39G	47G	74G
38C	47C	74C
36T	46T	76T
36T	45T	76T
36A	45A	76A
38A	45A	74A
38G	45G	72G
38G	45G	72G
38C	45C	71C
38A	45A	70A
38G	45G	73G
38A	44A	72A
38G	44G	73G
38G	44G	72G
37G	42G	72G
36G	43G	74G
36C	39C	72C
36G	39G	74G
36G	39G	72G
37G	39G	70G
32T	37T	70T
35C	39C	68C
36C	37C	69C
36C	36C	70C
35T	37T	69T
34C	36C	70C
36G	36G	71G
35G	35G	70G
35T	16T_19C	68T
35T	16T_19G	30T_39G
33T	35T	67T
32C	35C	65C
32A	34A	63A
31G	34G	60G
30A	35A	61A
31C	35C	64C

31C	35C	65C
31C	35C	66C
31A	36A	65A
31C	36C	28T_37C
30G	37G	64G
29C	37C	60C
29G	36G	61G
29C	37C	60C
29C	37C	62C
29G	35G	59G
28T	36T	58T
28T	37T	55T
28T	37T	58T
27T	37T	57T
27A	36A	58A
27T	36T	59T
27T	36T	60T
27T	35T	60T
27T	36T	58T
16T_11C	36T	58T
29A	36A	58A
28A	36A	59A
28A	34A	59A
28A	35A	28A_31C
28T	35T	60T
28A	34A	60A
28A	34A	59A
28G	14A_19G	60G
14T_14C	33C	32T_1G_30C
28G	31G	64G
28A	31A	65A
28C	31C	67C
28A	31A	65A
28T	30T	67T
27G	30G	68G
26T	29T	66T
26T	30T	66T
26A	29A	66A
26T	29T	64T
27T	29T	64T
27T	28T	66T
25T	29T	63T
24G	29G	62G

23G	28G	64G
23C	28C	62C
23C	28C	62C
23G	28G	61G
24C	27C	60C
23A	27A	59A
23A	26A	59A
23A	25A	58A
23C	25C	57C
23A	25A	56A
23A	25A	56A
22G	24G	56G
20A	22A	56A
22T	21T	56T
21G	20G	54G
21C	20C	55C
21G	18G	54G
21T	18T	53T
20G	18G	54G
19G	17G	55G
19T	17T	54T
19T	17T	54T
19G	17G	54G
19G	16G	55G
3A_11T	16G	31G
12A	16A	35A
17A_5T_5G_3C	15A	31A
12C	15C	34C
12A	5A	33A
12T	5T	33T
11C	5T	33C
20G	5G	49G
20C	5C	48C
9A_11G	8G	48G
20G	8G	48G
20C	8C	45C
20T	8T	45T
20C	8C	44C
20C	8C	46C
20C	3T_5C	21T_25C
20G	8G	46G
20G	8G	46G
20A	8A	46A

20A	8A	46A
20G	8G	47G
20C	8C	46C
19A	8A	47A
19T	8T	47T
19C	8C	48C
19A	8A	49A
19T	8T	49T
19T	8T	49T
19C	8C	50C
19C	8C	50C
20C	8C	49C
20C	8C	46C
20T	8T	46T
20A	8A	45A
20T	8T	44T
20T	8T	44T
20T	8T	42T
20A	8A	42A
20G	8G	41G
20T	8T	42T
19G	8G	41G
20A	8A	42A
21G	8G	42G
21G	8G	39G
8A_13G	8G	21A_18G
20G	8G	43G
20A	9A	42A
20G	9G	41G
20A	9A	41A
19A	9A	41A
19G	9G	42G
19A	8A	42A
19C	8C	39C
19A	8A	40A
19C	8C	39C
19A	8A	40A
19C	8C	41C
18A	7A	40A
19C	7C	22G_19C
20C	8C	42C
20A	8A	41A
21G	8G	40G

20C	8C	38C
20C	7C	39C
21A	7A	39A
21C	7C	38C
21A	7A	39A
21A	7A	39A
21G	7G	40G
21A	7A	39A
21A	7A	39A
21T	7T	38T
21A	7A_12T_12G	37A
9T_46G_34C	6G	57C
18G	12G	33G
22G	12G	33G
22G	12G	32G
11T_13G	12G	33G
21G	12G	34G
20G	12G	34G
20G	12G	19T_76G
20G	12G	13A_22G
17T	10T	34T
19G	11G	13T_22G
20C	11C	39C
21A	11A	40A
21T	11T	42T
21A	11A	44A
21T	11T	44T
20G	12G	46G
23G	12G	44G
22G	12G	44G
22C	12C	43C
23C	12C	44C
24C	11C	44C
16G_8C	11C	44C
25A	11A	45A
23C	11C	45C
24A	12A	45A
24A	12A	44A
24G	11G	44G
24T	11T	44T
24C	11C	46C
24A	10A	48A
24C	11C	48C

24C	11C	48C
23G	9A	43A
24G	11G	45G
25C	11C	45C
25C	11C	47C
25A	11A	48A
25C	12C	48C
25C	12C	49C
24C	13C	50C
25T	13T	49T
25T	13T	52T
26C	14C	54C
26C	13C	54C
28C	15C	56C
28T	15T	58T
28G	15G	58G
27A	16A	58A
27C	17C	57C
27C	17C	57C
17A_10G	17G	54G
27A	17A	56A
27G	17G	57G
27A	17A	57A
27A	18A	57A
27G	18G	58G
27T	18T	57T
27A	18A	58A
17A_10C	18C	57C
29A	18A	58A
29A	18A	60A
29A	18A	61A
29T	18T	61T
29C	18C	61C
29C	18C	61C
29A	18A	61A
30G	18G	60G
30A	18A	60A
30T	18T	61T
30C	18C	60C
30C	18C	30G_30C
12A_18G	18G	30A_30G
30A	18A	64A
30A	17A	63A

30C	18C	63C
30A	18A	63A
30G	30A_15T_30G_6C	62G
30A	15A	61A
30T	14T	61T
30G	14G	60G
30T	14T	60T
30G	14G	60G
29T	14T	58T
29A	13A	56A
29A	13A	56A
30A	13A	54A
30C	13C	55C
30A	13A	55A
30C	12C	56C
30A	11A	33A
31A	11A	34A
31A	11A	53A
31T	11T	54T
15T	11T	54T
15A	11A	55A
15A	11A	56A
15G	12G	55G
15G	12G	56G
13A	15G	55G
13A	15A	52A
13C	15C	50C
13G	15T	50T
13T	15T	51T
13C	16C	30G_22C
26C	16C	26T_30C
27G	16G	61G
27G	16G	62G
27T	15T	62T
27G	16G	62G
27A	16A	61A
27T	15T	61T
27G	15G	61G
27G	15G	61G
27A	14A	60A
27A	4A_10T	65A
27C	15C	65C
27A	14A	66A

27G	14G	67G
26A	14A	68A
26G	14G	70G
27C	14C	71C
27A	14A	72A
27T	14T	70T
27A	14A	70A
27T	14T	32T_38G
27T	14T	69T
27T	14T	70T
27T	14T	70T
27G	14G	70G
26G	14G	73G
26C	14C	71C
26T	14T	70T
26T	14T	71T
25C	14C	72C
23C	14T	31T_40C
24T	14T	72T
23A	14A	73A
23G	13G	75G
21A	13A	74A
21A	13A	74A
20A	12A	73A
20C	13C	74C
20T	13T	73T
20G	12G	74G
19G	12G	77G
19A	12A	77A
19G	12G	78G
19G	11G	76G
19G	11G	76G
19C	11C	74C
18G	11A	68G
18A	11A	67A
17C	11C	66C
16A	11A	66A
16C	10C	65C
16C	11C	67C
16A	11A	65A
16G	10A	65G
16G	10G	64G
16T	9T	64T

17A	9A	66A
17T	9T	64T
17A	8A	62A
18C	8C	35T_26C
18T	7T	59T
18T	7T	59T
18T	7T	59T
18G	7G	58G
17C	6C	59C
17A	6A	60A
17G	6G	57G
17C	6C	57C
17T	6T	55T
17G	6G	54G
17C	5C	50C
16C	5C	50C
16C	5C	49C
16A	5A	49A
16A	5A	48A
15A	4A	47A
15A	4A	48A
14T	4T	49T
14C	4C	48C
14T	4T	47T
13G	4G	45G
13A	4A	46A
12G	4G	44G
12G	4G	46G
12A	4A	44A
12A	3A	43A
11G	3G	42G
11G	3G	40G
11C	3C	40C
11T	3T	40T
11G	2G	40G
11G	3G	39G
11T	3T	39T
11C	3C	38C
11T	3T	36T
11A	3A	35A
10C	3C	34C
10A	3A	33A
10A	3A	32A

10A	3A	29A
10T	3T	29T
9C	3C	28C
9C	3C	27C
7A	2A	16T
6C	2C	16T
6C	2C	16T
6T	2T	20T
6T	2T	20T
6T	2T	20T
6G	2G	20G
6A	2A	18A
6A	2A	18A
6A	2A	17A
6C	2C	14C
6G	2G	14A
6G	2G	17G
6G	2G	17G
6G	2G	19G
6G	2G	20G
6G	2G	19G
5G	2G	19G
5G	2G	17G
5G	2G	17G
5G	2G	16G
null	2G	17G
null	2G	16G
null	2G	8A_12G
null	1A	4G
null	null	13A
4T	3T	13T
4A	3A	13A
8T	6T	14T
8T	6T	15T
8T	6T	18T
8A	6A	18A
8T	8T	19T
8T	8T	18T
8T	8T	18T
8T	8T	20T
9A	9A	20A
9T	11T	20T
9A	12A	21A

9T	12T	21T
9T	12T	21T
10T	12T	21T
10A	12A	21A
10T	12T	22T
10T	12T	22T
10T	12T	23T
10A	14A	23A
10T	14T	24T
11T	14T	23T
11T	14T	23T
12A	15A	21A
12T	15T	21T
12T	15T	19T
12G	16G	19G
12G	16G	20G
12A	16A	21A
12C	16C	21C
12T	17T	23T
13T	17T	23T
13A	17A	24A
13T	16T	24T
13A	17A	24A
13T	17T	26T
13A	18A	26A
14C	19C	28C
14C	19C	29C
15G	19G	30G
16C	19C	31C
18C	19C	32C
18C	20C	32C
18C	20C	32C
18A	20A	34A
17T	19T	34T
18A	20A	37A
19G	21G	37G
19C	21C	36C
19G	21G	36G
19C	20C	36C
19T	20T	36T
18A	20A	39A
18C	19C	39C
18A	20A	37A

17A	20A	39A
17G	19G	38G
15C	20C	38C
15A	20A	37A
15C	21C	39C
15T	19T	39T
16C	19C	40C
16T	20T	41T
16C	21C	41C
15C	22C	41C
16G	23G	41G
15G	23G	41G
16G	24G	43G
18C	23C	43C
18G	24G	42G
18G	24G	43G
18T	23T	42T
18T	23T	41T
18T	23T	41T
18A	22A	42A
17C	22C	40C
18A	22A	42A
19A	23A	40A_33T
19T	22T	45T
19T	23T	45T
19T	23T	46T
19A	22A	44A
19A	25A	45A
19T	26T	45T
18T	26T	46T
18A	28A	46A
18T	27T	47T
18A	27A	49A
18C	27C	50C
18A	27A	50A
18G	28G	49G
18G	28G	50G
19C	28C	51C
20T	29T	51T
20A	29A	52A
21C	30C	56C
21A	30A	54A
21C	30C	55C

21A	31A	53A
21T	31T	53T
20T	31T	52T
20G	31G	52G
20C	31C	53C
20C	31C	53C
20C	33C	53C
20C	34C	51C
20C	34C	51C
21C	35C	50C
21C	36C	50C
21A	35A	49A
21G	31G	50G
18C	39C	49C
18A	39A	48A
18A	39A	50A
18G	39G	49G
18C	38C	49C
18T	38T	47T
18G	38G	49G
18G	37G	49G
18G	38G	49G
19T	38T	47T
19A	38A	49A
19C	38C	47C
20T	38T	47T
20C	37C	47C
20A	38A	46A
20T	39T	46T
20T	39T	47T
20T	38T	48T
20T	39T	46T
20A	40A	46A
20C	40C	48C
20C	40C	49C
20G	41G	49G
20A	40A	48A
20C	40C	46C
20C	42C	46C
19T	39T	48T
20C	41C	47C
20G	40G	45G
20G	40G	47G

19A	39A	49A
19A	40A	49A
18G	41G	49G
17G	39G	48G
18A	41A	47A
18T	39T	47T
18G	39G	47G
17G	40G	47G
17A	42A	51A
17A	42A	51A
16G	42G	52G
16G	42G	52G
16C	42C	52C
16T	42T	52T
17G	42G	53G
17A	41A	50A
16G	39G	51G
15T	40T	50T
16C	40C	51C
15A	40A	51A
14A	40A	50A
14C	39C	51C
14C	39C	51C
14T	37T	51T
14T	38T	48T
13G	39G	48G
13A	38A	48A
13G	37G	47G
14C	38C	48C
13C	38C	48C
14A	30A_8G	28A_1T_19G
14G	38G	51G
14C	38C	52C
14T	38T	52T
14A	38A	52A
14C	38C	52C
14C	37C	53C
14T	37T	51T
14G	37G	51G
14G	37G	49G
13G	36G	49G
13A	38A	48A
13T	36T	47T

13T	38T	46T
13T	37T	46T
13G	37G	46G
12A	36A	45A
11A	35A	46A
11C	35C	47C
11C	33C	48C
10C	33C	46C
10C	33C	51C
10A	33A	51A
10G	33G	51G
10G	32G	50G
10T	33T	52T
9C	32C	50C
9G	29G	49G
9T	33T	49T
9G	33G	48G
9A	33A	50A
9G	30G	52G
9C	30C	52C
9A	23A	52A
8C	28C	52C
9A	27A	51A
8G	29G	49G
9T	28T	50T
9T	28T	50T
9T	28T	49T
9T	28T	47T
8A	29A	46A
7G	27G	44G
7C	27C	44C
6T	26T	44T
6T	26T	42T
6G	26G	45G
6C	25C	44C
6A	25A	43A
3A_3G	21G	43G
6T	23T	43T
6A	23A	42A
6C	23C	44C
6A	23A	43A
6G	22G	44G
6C	23C	43C

5G	23G	44G
5T	22T	44T
5T	24T	43T
5T	24T	44T
5T	21T	44T
5A	22A	44A
5A	22A	42A
5C	22C	41C
5C	22C	41C
5A	22A	41A
5C	21C	40C
5T	21T	39T
5G	21G	37G
5C	21C	37C
5G	21G	37G
5C	22C	39C
4C	22C	39C
4A	22A	38A
4C	20C	38C
4G	20G	39G
2A	20A	39A
1A_2G	20G	40G
22G	22G	57G
22C	20C	58C
23T	19T	58T
21G	17G	58G
21C	14C	59C
22T	14T	61T
22C	14C	61C
22T	14T	62T
22T	14T	64T
23G	15G	63G
23C	15C	63C
23T	15T	64T
23G	16G	63G
23A	16A	60A
23G	16G	58G
23C	16C	59C
23A	15A	58A
23C	17C	59C
23C	17C	58C
23C	17C	59C
23A	17A	60A

23G	16G	61G
23G	17G	63G
23T	16G	61G
23G	17G	60G
24A	17A	60A
24C	17T	61T
25G	17G	62G
25G	17G	62G
25C	17C	63C
25G	14G	65G
25G	17G	64G
25G	17G	66G
25C	17C	68C
25A	17A	68A
25A	17A	69A
25T	17T	69T
25G	17G	71G
25A	17A	70A
25G	17G	70G
26G	17G	36G_33C
26G	18G	71G
25T	18T	71T
25C	18C	75C
25C	18C	74C
25C	18C	74C
25A	17A	71A
25A	19A	71A
25G	19G	70G
25A	19A	72A
25G	19G	72G
25G	19G	71G
25G	19G	72G
25T	19T	71T
25C	19C	69C
26T	19T	67T
27A	15A	35A_33C
27C	19C	34G_33C
27A	19A	68A
28G	19G	67G
28G	19G	67G
28T	20T	67T
29A	20A	66A
29G	21G	64G

30G	21G	63G
29G	21G	63G
30G	21G	60G
30C	22C	58C
30T	17T_5C	55T
31C	22C	55C
31A	23A	55A
31C	23C	54C
31C	23C	59C
31C	24C	57C
32C	24C	58C
31C	24C	50C
30A	7A_17C	55A
30A	24A	55A
29A	24A	58A
31A	24A	61A
29A	25A	60A
29A	25A	60A
29G	25G	61G
30C	24C	63C
30A	24A	62A
30A	24A	63A
30A	23A	63A
29T	24T	64T
30T	23T	65T
29T	23T	64T
30T	23T	65T
30C	24C	64C
28A	24A	63A
28T	24T	64T
29G	24G	64G
29C	24C	63C
30A	24A	65A
29G	24G	66G
30G	24G	65G
29T	25T	62T
29C	23C	61C
30T	22T	61T
30C	23C	61C
30T	22T	32T_29C
30G	22G	61G
30A	22A	60A
29A	21A	63A

29A	22A	63A
29G	23G	61G
29A	23A	61A
27A	23A	63A
27A	23A	62A
27G	23G	62G
25T	23T	61T
26C	21C	60C
25C	22C	62C
25T	21T	64T
25A	21A	66A
24G	21G	64G
23C	22C	67C
23T	22T	66T
24C	21C	68C
24C	22C	68C
23A	22A	68A
24C	23C	69C
23C	24A	39A_32C
23A	24A	74A
23G	25G	75G
24C	25C	75C
23G	24G	38A_36G
23A	23A	71A
23C	24C	76C
23C	23C	76C
23T	23T	78T
23C	23C	78C
23A	24A	79A
23G	23G	81G
23A	22A	82A
24G	22G	81G
24T	22T	83T
24C	23C	82C
24A	23A	81A
25G	22G	79G
25G	22G	79G
27G	21G	80G
26G	21G	79G
26C	21C	77C
26C	12T_9C	76C
26G	9T_12G	1A_37T_38G
10T_16C	21C	74C

26C	22C	77C
26T	22T	75T
27G	24G	74G
27G	24G	74G
27T	24T	74T
27T	22T	75T
27C	24C	73C
27C	24C	74C
27C	24C	73C
28T	25T	73T
27T	25T	72T
27T	23T	71T
27T	24T	37T_34C
27C	24C	68C
26A	24A	66A
26G	24G	67G
27A	24A	65A
27C	23C	66C
27A	23A	68A
27G	22G	70G
27G	23G	71G
27C	23C	71C
27C	22C	72C
28T	24T	71T
27A	24A	71A
28C	26C	71C
27C	26C	68C
27G	26G	67G
27G	26G	69G
26T	26T	67T
26T	25T	66T
26C	25C	67C
23A	25A	66A
25C	24C	63C
24C	24C	64C
14A_10G	15A_9G	34A_28G
24A	24A	62A
24G	22G	61G
24A	23A	61A
23C	23C	58C
23T	22T	59T
23G	22G	58G
23G	22G	59G

23G	21G	59G
23G	21G	58G
26A	20A	55A
26A	21A	54A
27A	22A	53A
28A	22A	51A
26G	23G	54G
26T	23T	54T
26T	23T	51T
26A_11T_11C	23A	50A
28C	8T_24C	48C
29T	25T	48T_20C
28T	27T	45T
31T	27T	50T
34T	27T	49T
35T	29T	50T
34T	29T	51T
35T	29T	52T
35T	28T	52T
35T	27T	47T
35T	26T	26T_23C
34C	26C	48C
34A	26A	48A
34T	27T	48T
34C	27C	48C
34C	27C	48C
33T	28T	48T
33C	28C	51C
33A	28A	52A
33T	28T	51T
32C	29C	55C
32C	30C	54C
32T	30T	54T
32C	30C	54C
30A	30A	56A
30C	29C	57C
30C	33C	57C
33A	33A	58A
34T	34T	58T
34C	33C	57C
34T	34T	59T
34T	34T	62T
34A	34A	62A

34G	34G	64G
34A	34A	63A
34A	34A	63A
33C	35C	63C
32T	35T	62T
33G	35G	63G
33C	36C	63C
33A	36A	62A
33G	36G	61G
33A	37A	60A
33G	37G	60G
33C	35C	59C
33T	36T	31T_29C
33G	36G	59G
22G_11C	19T_15C	59G
32G	19A_15G	27A_33G
33A	34A	60A
33G	34G	60G
33G	34G	61G
33G	34G	61G
32A	33A	58A
32C	34C	29T_30C
32C	19A_15C	59C
32C	34C	58C
32T	34T	58T
33A	33A	57A
32T	34T	56T
32G	34G	57G
32G	36G	57G
32A	37A	56A
32A	36A	56A
33C	37C	55C
29A	37A	28A
29C	37C	28C
28A	36A	27A
27T	36T	45T
28C	35C	48C
28C	35C	47C
28G	35G	47G
28A	35A	44A
28G	35G	45G
27C	35C	45C
27G	35G	23A_1T_20G_1C

27C	35C	46C
27T	35T	46T
27T	34T	45T
27G	35G	45G
27C	36C	46C
28C	36C	20T_26C
28T	36T	45T
27T	36T	45T
27T	36T	45T
27C	37C	45C
27A	37A	43A
28C	38C	43C
27T	38T	41T
27T	38T	41T
27G	37G	40G
27C	37C	40C
27A	35A	39A
28T	37T	39T
28C	36C	37C
28T	35T	1A_18T_4G
29C	36C	24C
29C	36C	24C
28G	36G	24G
29T	36T	25T
37C	52C	22C
25C	19C	21C
26C	36C	4T_1G_17C
30C	36C	22C
31C	37C	23C
31C	36C	22C
31C	37C	21C
31C	37C	22C
36A_52C	37C	21C
16C	37C	22A_32C
4A_15C	17A_32C	39A_10C
15A	21A	7A
14A	36A	2A_1C
14A	35A	16A
26A	36A	17A
26A	36A	16A
26A	36A	16A
25A	37A	15A
27A	35A	14A

22A	35A	14A
12A_1T_7C	33C	4A_15C
21A	36A	37A
21A	36A	37A
23T	34T	40T
23C	34C	40C
23A	35A	40A
23A	35A	41A
22T	33T	42T
26A	34A	41A
25A	35A	42A
22G	36G	43G
21G	36G	43G
21A	36A	43A
21T	35T	44T
22A	37A	43A
21A	36A	43A
22T	36T	43T
22A	35A	44A
22A	34A	45A
20C	34C	48C
21A	35A	49A
21A	35A	49A
22C	35C	48C
22A	35A	48A
22T	35T	49T
22G	35G	51G
22C	35C	31A_20C
22T	35T	51T
22G	32G	53G
22G	33G	52G
22T	34T	51T
22C	32C	55C
22G	32G	56G
21G	32G	55G
22C	32C	55C
22A	32A	55A
22G	32G	56G
23G	32G	56G
23C	31C	55C
23T	31T	56T
23C	31C	55C
23T	30T	54T

25G	32G	52G
25T	33T	52T
25G	32G	52G
25A	33A	52A
25A	33A	52A
25A	33A	52A
25A	33A	51A
25C	33C	50C
25G	33G	51G
15T_10G	21T_12G	24T_27G
24G	33G	49G
25T	35T	48T
26G	36G	47G
26C	35C	50C
26G	36G	50G
26A	36A	52A
27T	36T	50T
27T	36T	47T
27C	36C	49C
28G	13A_23G	50G
28C	35C	51C
27C	34C	52C
27C	33C	53C
27C	33C	53C
29C	33C	56C
27T	33T	56T
27T	33T	58T
27C	33C	59C
26C	33C	59C
27C	33C	61C
27C	33C	61C
27A	33A	58G
27C	33C	59C
27A	32A	58A
27G	33G	57G
27G	33G	58G
27G	32G	59G
27C	32C	61C
28A	32A	61A
28G	32G	62G
28G	31G	66G
29G	31G	66G
29G	31G	67G

29T	32T	68T
29C	32C	68C
29C	31C	67C
31A	31A	68A
31G	30G	66G
31A	30A	68A
31G	21G_9C	68G
31G	30G	68G
30T	30T	67T
30C	31C	73C
29C	30C	72C
30A	30A	71A
30G	29G	72G
30A	30A	70A
15T_14G	31G	69G
29A	31A	68A
29G	31G	65G
28C	31C	65C
28A	33A	65A
27C	33C	67C
28G	33G	69G
30G	33G	68G
29C	33C	67C
29T	34T	66T
30G	34G	67G
29G	34G	70G
29C	34C	71C
30T	34T	71T
30G	33G	72G
30T	33T	70T
31C	33C	73C
32A	32A	73A
32A	31A	72A
33G	31G	76G
33C	32C	76C
33C	33C	76C
33A	32A	76A
33G	32G	76G
31G	33G	76G
31C	32C	76C
31T	31T	72T
31G	31G	45A_27G
31G	32G	70G

28A	31A	71A
28G	30G	70G
28A	30A	69A
28C	31C	70C
28C	31C	69C
27G	31G	69G
27C	31C	68C
9A_18C	32C	68C
26G	32G	66G
26G	31G	65G
26C	32C	65C
26T	33T	62T
26C	32C	64C
26C	31C	63C
26C	30C	62C
9T_17C	19T_13C	63C
27G	32G	63G
28C	32C	63C
28T	33T	63T
28C	35C	62C
28T	34T	62T
28A	34A	60A
28C	35C	58C
28C	35C	59C
28C	35C	55C
28C	35C	54C
28C	35C	52C
28T	35T	53T
29T	37T	55T
28T	38T	54T
29T	38T	53T
30T	38T	53T
30C	38C	50C
30T	38T	50T
30T	35T	51T
30C	35C	53C
30A	34A	49A
30A	33A	50A
30A	33A	50A
30T	33T	50T
30C	33C	50C
31A	33A	51A
31A	33A	51A

31G	33G	54G
32C	32C	54C
31A	30A	54A
31C	30C	52C
31C	31C	50C
32G	31G	51G
32C	31C	52C
31T	31T	53T
31T	31T	51T
31C	31C	49C
30C	31C	49C
30C	31C	50C
30A	32A	50A
31C	32C	49C
31A	32A	48A
31C	32C	50C
31G	33G	52G
31C	33C	49C
31A	33A	49A
31A	32A	49A
30A	33A	49A
30G	33G	49G
30G	33G	50G
30A	33A	50A
30A	35A	50A
30T	35T	50T
31G	36G	50G
32A	36A	52A
34G	37G	53G
32G	38G	54G
33G	38G	54G
34A	38A	56A
33G	38G	56G
33A	37A	56A
34T	37T	57T
35G	37G	57G
36T	36T	56T
34G	37G	57G
34C	37C	57C
35A	37A	57A
36A	38A	59A
38C	37C	59C
38T	36T	60T

38C	36C	60C
38A	36A	62A
37G	36G	62G
38C	36C	62C
38A	36A	61A
38G	36G	62G
38A	37A	64A
39G	37G	65G
39T	37T	64T
39C	35C	67C
39C	34C	69C
39G	34G	65G
37G	34G	67G
21T_16C	34C	68C
37A	33A	69A
36G	34G	70G
37G	34G	70G
38G	34G	71G
39G	35G	72G
40A	36A	69A
41A	36A	33A_38T
41C	37C	70C
42G	37G	71G
42C	37C	71C
43C	38C	72C
43A	37A	71A
41G	36G	72G
41A	36A	69A
41C	36C	70C
40C	36C	71C
40C	36C	71C
40A	36A	70A
41G	36G	70G
41G	35G	70G
41G	36G	70G
41C	36C	72C
41G	35G	71G
40T	34T	70T
41C	33C	69C
40C	35C	70C
41C	36C	70C
43C	35C	71C
45A	35A	69A

45G	35G	67G
46T	35T	67T
46C	34C	65C
46C	34C	66C
46G	33G	67G
46G	34G	70G
46C	32C	68C
45T	32T	68T
44C	32C	71C
44T	32T	72T
45T	30T	73T
45A	30A	70A
45T	29T	71T
45C	28C	70C
46T	29T	72T
46T	29T	73T
46C	29C	72C
45T	30T	70T
46C	29C	72C
45C	28C	71C
46C	28C	71C
46C	31C	70C
46G	31G	72G
48T	31G	33T_38G
48T	31T	70T
46C	31C	70C
46A	31A	69A
46A	30A	70A
46C	31C	69C
46T	31T	68T
46C	31C	69C
47C	32C	69C
47T	32T	67T
48G	32G	65G
48G	32G	65G
47G	32G	61G
48A	33A	61A
49C	33C	62C
49A	32A	63A
48G	33G	63G
48A	33A	62A
48G	33G	62G
48A	32A	62A

48A	33A	61A
48T	33T	60T
49C	34C	58C
48T	33T	58T
49C	33C	60C
49G	33G	57G
47C	32C	58C
48C	32C	59C
48T	32T	58T
48G	32G	57G
47A	32A	56A
47T	31T	55T
48T	32T	56T
48C	32C	56C
48T	32T	54T
48T	30T	56T
48A	30A	58A
48A	29A	61A
47C	29C	60C
45A	28A	59A
47G	27G	58G
46A	28A	57A
44G	28G	58G
43T	28T	57T
43G	28G	57G
43T	28T	58T
43C	28C	59C
42T	28T	60T
44G	28G	60G
45T	29T	59T
45T	30T	62T
44G	29G	62G
43G	30G	61G
42G	31G	62G
43C	30C	63C
43C	31C	62C
42A	30A	63A
42G	31G	63G
42G	31G	64G
42G	29G	36A_28G
42G	29G	63G
42A	29A	63A
43G	29G	60G

43G	29G	57G
43G	29G	56G
43A	29A	59A
42A	29A	57A
42A	29A	57A
42A	30A	58A
42C	28C	58C
41T	29T	56T
38G	28G	59G
37C	28C	56C
37C	27C	58C
14T_23C	20T_7C	58C
36C	26C	57C
34G	26G	57G
34T	26T	57T
32T	25T	59T
33G	25G	59G
34T	26T	59T
35T	26T	57T
36T	26T	60T
36G	25G	60G
36C	26C	61C
37C	26C	61C
36T	26T	61T
36G	25G	65G
36A	25A	65A
36C	25C	65C
35A	25A	65A
36A	27A	65A
36A	27A	66A
37A	25A	68A
37G	24G	68G
35A	26A	67A
33T	26T	66T
33G	26G	62G
33C	25C	63C
33A	26A	63A
34G	25G	68G
34T	28T	70T
35G	28G	68G
35T	28T	68T
34C	29C	69C
34T	29T	70T

34C	28C	68C
33C	28C	68C
33T	27T	68T
33A	29A	67A
33A	29A	68A
32G	27G	68G
32A	29A	69A
33T	29T	72T
33G	29G	71G
32C	31C	68C
32C	30C	69C
31G	31G	69G
31T	28T	69T
33T	28T	69T
34G	29G	68G
34T	29T	67T
35G	29G	66G
35G	28G	63G
37A	31A	63A
37T	30T	62T
38A	33A	63A
36C	35A_40T_28C	80A_82T_60C
39A	33A	68A
38T	33T	68T
38A	33A	67A
38T	33T	67T
67A_99T	74A_79T	67A
42T	32T	67T
39T	31T	57T
39T	33T	59T
41T	32T	58T
42T	32T	60T
42G	32G	60G
42C	32C	62C
42T	33T	63T
42C	33C	63C
42T	33T	63T
43C	34C	63C
43C	33C	64C
43C	34C	65C
43G	34G	64G
43G	34G	64G
42G	35G	64G

42A	35A	63A
42G	35G	62G
42A	35A	62A
42T	35T	60T
43A	34A	61A
44C	34C	60C
45G	34G	59G
45G	35G	59G
46T	35T	61T
44C	35C	63C
44C	35C	66C
44C	35C	67C
44T	34T	69T
43T	33T	69T
43C	33C	30G_39C
42C	32C	69C
43T	32T	68T
42C	32C	68C
41C	32C	69C
41G	33G	71G
41C	32C	74C
42C	34C	71C
43T	34T	68T
43T	34T	68T
42G	35G	67G
42T	36T	65T
42A	36A	65A
42A	36A	67A
42G	36G	67G
43A	37A	68A
43A	38A	70A
42T	37T	71T
42G	37G	70G
43C	37C	68C
43A	37A	67A
44G	36G	68G
43A	37A	69A
42T	37T	72T
42G	36G	71G
42G	37G	69G
42G	36G	71G
26A_18G	19A_18G	72A
44A	38A	73A

44A	38A	73A
44A	38A	76A
44A	38A	75A
45A	38A	76A
45A	38A	76A
45G	38G	79G
45A	40A	80A
45A	40A	81A
44G	40G	81G
43G	40G	82G
44G	41G	82G
43A	40A	82A
43G	39G	80G
43A	40A	78A
20T_21C	40C	78C
41G	38G	39A_37G
41G	39G	82G
41A	39A	79A
21T_20C	38C	82C
42T	40T	84T
42C	40C	87C
41C	39C	86C
21G_20C	18T_20C	86C
42T	39T	87T
42T	38T	86T
43T	38T	86T
43A	38A	84A
43A	40A	83A
43C	41C	81C
42C	40C	81C
43C	40C	80C
43C	40C	80C
44C	39C	80C
45T	40T	79T
46G	41G	79G
19G_27C	39C	78G
46G	40G	77G
46G	39G	74G
46A	39A	72A
20T_25C	39C	74T
43A	38A	70A
42A	40A	71A
42G	40G	72G

43A	39A	73A
44A	39A	73A
43A	40A	73A
43G	40G	70G
42A	40A	68A
42A	40A	69A
42G	40G	69G
42G	17A_23G	69G
40T	42T	70T
44G	41G	72G
45C	40C	71C
45T	40T	70T
44T	40T	69T
43C	38C	69C
23A_21G	38A	66A
43T	38T	65T
43T	38T	66T
42C	38C	68C
40T	38T	69T
39C	39C	69C
39C	37C	69C
39T	37T	65T
39C	37C	65C
38T	37T	66T
38C	38C	64C
38T	38T	63T
38G	37G	63G
38G	38G	62G
38G	38G	60G
38T	38T	60T
37T	37T	60T
37T	36T	59T
38T	37T	56T
38T	38T	55T
23A_15C	38C	58C
38T	39T	61T
38A	39A	61A
39C	38C	61C
38A	39A	62A
37G	40G	60G
37A	40A	59A
36A	40A	59A
36G	39G	59G

36G	39G	57G
37C	39C	58C
22A_15G	39G	56G
37C	39C	56C
37C	39C	54C
37A	39A	57A
37A	39A	59A
37G	39G	58G
35A	38A	62A
35T	38T	62T
35G	37G	64G
35C	37C	66C
35G	41G	68G
34T	40T	67T
34T	42T	68T
34T	42T	69T
35G	40G	70G
35G	42G	70G
36A	41A	69A
36G	43G	67G
37A	41A	67A
35C	43C	71C
36C	42C	71C
36C	42C	69C
37T	43T	70T
37G	44G	69G
36G	46G	68G
36T	45T	69T
36C	46C	28A_41C
36G	46G	69G
35T	47T	71T
36G	48G	72G
34T	49T	70T
35T	48T	73T
35T	47T	75T
34T	47T	73T
34G	47G	72G
33T	47T	71T
33T	46T	69T
34C	47C	71C
33A	46A	72A
16A_18G	46G	73G
34G	46G	72G

34C	45C	71C
34G	45G	72G
34G	45G	71G
34T	45T	71T
34C	44C	71C
33C	44C	70C
33C	45C	72C
33C	45C	26G_45C
33G	45G	70G
33C	45C	69C
32C	45C	69C
31G	45G	68G
33G	45G	68G
33G	45G	68G
32G	21G_23C	67G
32A	44A	69A
31T	45T	67T
32C	44C	69C
33A	44A	67A
33A	43A	65A
34G	43G	64G
34A	42A	63A
34C	43C	64C
33C	42C	64C
32G	41G	67G
30A	40A	67A
32G	40G	67G
32C	39C	69C
32A	39A	70A
32G	38G	69G
32A	40A	69A
32G	40G	69G
31C	39C	65C
33A	39A	65A
32G	40G	65G
33G	41G	62G
33T	41T	62T
32G	44G	61G
32G	44G	59G
31G	43G	58G
31T	44T	57T
30T	44T	59T
29G	42G	60G

29T	42T	59T
28T	42T	59T
27G	40G	57G
26T	40T	58T
27G	40G	57G
26A	41A	53A
26C	41C	53C
26C	42C	54C
26G	21A_21G	55G
26T	43T	54T
25G	42G	54G
24C	42C	53C
16A_8G	43G	53G
23C	43C	51C
7T_16C	43C	51C
23G	42G	24A_25G
23A	42A	49A
23G	42G	47G
22A	42A	45A
22A	41A	45A
22A	41A	45A
22G	41G	44G
22A	40A	44A
21T	41T	43T
20C	40C	42C
21T	39T	39T
21C	46C	39C
21C	38C	37C
11A_20C	38C	35C
16C	13A_51C	34C
15A_13C	10A_37C	34C
13C	25C	25A
13C	13A_12C	27C
13C	25C	27C
13C	25C	27C
11A	23A_68C	27C
13C	27C	30C
57C	27C	29C
17A	40A	33A
20A	40A	33A
21A	40A	33A
22A	41A	34A
22A	42A	34A

22A	42A	35A
22A	41A	35A
22T	42T	38T
22G	42G	38G
22C	42C	42C
22A	42A	42A
18A	42G	44G
22T	41T	43T
22C	42C	44C
22C	42C	46C
22A	42A	46A
22A	42A	44A
22A	41A	43A
22G	40G	44G
22T	40T	42T
22T	40T	43T
22C	39C	44C
22C	38C	44C
21A	38A	41A
21C	38C	41C
21C	38C	43C
21T	38T	45T
22G	38G	45G
22G	38G	45G
21C	39C	45C
21C	39C	43C
20A	38A	43A
20G	38G	43G
20G	38G	45G
21G	40G	45G
21G	40G	49G
21C	43C	51C
22T	44T	52T
22A	45A	53A
22C	45C	53C
22A	46A	52A
23G	44G	52G
25G	44G	54G
25G	46G	57G
25C	45C	56C
23C	44C	56C
23T	44T	58T
24A	44A	59A

24C	45C	59C
25T	44T	60T
24T	45T	61T
26T	43T	62T
26A	44A	63A
29C	43C	64C
30A	45A	64A
30G	45G	64G
30G	46G	64G
33G	47G	62G
33C	47C	62C
32T	29T_19C	62T
32G	49G	62G
32T	49T	61T
33T	50T	62T
33G	51G	63G
34T	51T	64T
34G	51G	65G
33C	52C	65C
35A	53A	66A
34A	53A	64A
35A	52A	64A
35T	50T	64T
35A	48A	32A_22G
35G	48G	22T_32G
35C	48C	22G_32C
35C	49C	59C
35A	50A	59A
34C	51C	59C
35A	50A	56A
36A	51A	56A
35C	51C	53C
35A	50A	54A
34T	50A	26A_27T
34G	29A_21G	53G
34G	50G	52G
34C	49C	51C
34A	49A	52A
35T	48T	52T
36G	49G	52G
36T	49T	52T
36G	50G	52G
35C	48C	28T_22C

36C	49C	50C
37C	49C	51C
38A	49A	51A
38A	49A	52A
37C	49C	51C
10A_27G	48G	51G
36C	49C	52C
36C	48C	51C
36A	47A	52A
34C	48C	54C
35A	47A	52A
35A	48A	54A
35A	46A	54A
35C	45C	55C
35G	44G	53G
35C	43C	51C
35T	44T	51T
35C	44C	49C
35T	44T	51T
35T	43T	52T
35G	43G	52G
35T	41T	50T
35A	43A	47A
35C	43C	47C
35G	25A_16G	49G
35A	42A	47A
36T	26T_16C	18T_28C
36G	42G	48G
36G	42G	47G
36C	42C	47C
36T	42T	47T
35T	42T	46T
34T	41T	48T
34T	40T	48T
35C	40C	49C
35A	40A	49A
36G	40G	50G
36G	40G	50G
36C	40C	51C
36C	41C	53C
37T	41T	53T
37T	41T	30T_22C
37G	41G	51G

37T	42T	51T
38G	41G	50G
38G	42G	48G
38G	42G	48G
38T	42T	48T
38C	43C	48C
38T	44T	50T
38G	44G	50G
39G	44G	49G
39G	44G	50G
40C	45C	50C
40A	46A	50A
40G	46G	50G
39C	46C	51C
40C	45C	51C
40C	45C	52C
40A	45A	51A
40T	45T	53T
40A	44A	53A
42C	44C	53C
41A	43A	53A
41A	43A	53A
41C	40C	53C
40G	39G	52G
41C	38C	52C
41T	38T	52T
42A	38A	52A
42G	38G	52G
41C	38C	51C
41T	35T	53T
41C	36C	52C
41C	37C	52C
41G	36G	49G
40G	37G	48G
40A	37A	50A
39T	37T	51T
38T	37T	50T
36C	37C	50C
37T	37T	48T
33G	38G	48G
33A	37A	47A
34G	37G	45G
34A	35A	48A

31G	35G	48G
32T	35T	50T
32G	34G	49G
32C	32C	48C
33T	33T	47T
32T	11A_32T	48T
20A_11T	31A	47A
30A	32A	48A
30A	31A	47A
30A	30A	47A
28A	29A	47A
57A_15G	29A	45A
12A	29A	45A
11A_10G	30A	47A
1A	27G	46A
27A	27A	48A
27A	27A	46A
27A	28A	46A
27A	27A	45A
26G	26G	45G
28A	28A	44A
29A	28A	44A
29A	28A	45A
28T	28T	43T
28A	28A	42A
28T	13A_16T	42T
28T	29T	42T
28A	27A	42A
29C	28C	40C
29C	31C	40C
29A	30A	39A
29A	31A	39A
28A	30A	38A
28T	30T	37T
28T	29T	37T
28C	30C	36C
29T	31T	38T
28A	31A	38A
27A	30A	39A
27T	30T	39T
25C	29C	38C
27T	30T	39T
28A	30A	39A

28A	29A	40A
28C	28C	41C
28A	28A	43A
29C	29C	43C
29C	29C	43C
28A	29A	42A
29A	29A	43A
29G	28G	44G
29A	29A	44A
29C	29C	46C
29G	29G	47G
29G	29G	48G
29T	29T	48T
28G	29G	46G
29C	29C	48C
30C	29C	48C
30C	26C	48C
29G	27G	51G
30G	28G	50G
30A	28A	48A
30G	28G	48G
28T	28T	48T
29T	27T	48T
30G	27G	46G
30G	28G	46G
30C	28C	17G_25C
13A_17G	28G	41G
29C	28C	42C
31T	27T	42T
31G	28G	42G
31A	27A	43A
32T	27T	43T
32T	28T	44T
32C	28C	44C
32C	28C	43C
30T	28T	42T
31G	14G_13C	43G
31G	27G	45G
31C	26C	45C
31A	25A	46A
31C	25C	26A_17C
16A_17G	25G	44G
33G	25G	43G

33G	24G	44G
33C	23C	44C
34A	23A	44A
33T	23T	44T
33C	23C	44C
32T	23T	45T
32A	23A	46A
32A	23A	47A
32A	23A	47A
32G	23G	49G
32G	23G	49G
32A	26A	49A
31A	26A	50A
31T	26T	49T
30C	26C	50C
30A	27A	50A
30A	26A	50A
29A	26A	50A
29G	26G	50G
28G	25G	50G
27T	26T	48T
26C	25C	48C
26A	24A	47A
26C	25C	47C
26T	25T	47T
26C	25C	46C
26A	25A	45A
26T	25T	43T
26T	25T	41T
26T	24T	39T
26C	23C	38C
26T	23T	38T
26G	23G	38G
24T	23T	37T
23G	23G	36G
12T_11C	23C	34C
23T	23T	33T
23C	23C	32C
23T	23T	31T
82A_60T_75G_120C	63A_40T_50G_80C	71A_40T_50G_80C
7G	13G	21G
6T	12T	18T
null	null	1C

null	null	null
null	null	null
null	null	null
null	null	null
null	null	null
null	null	null
null	null	null
null	null	null
null	null	null
null	null	null
null	null	null
null	null	null
null	null	null
null	null	null
null	null	1C
null	null	2T
null	null	2C
null	null	2T
19A	18A	18A
19G	18G	18G
19T	18T	18T
19C	19C	19C
19G	19G	4A_15G
19T	19T	25T
20T	20T	27T
20T	19T	27T
20C	21C	27C
20C	21C	28C
22C	21C	28C
22C	21C	28C
23A	21A	28A
23G	21G	28G
24C	21C	30C
25T	21T	30T
25C	21C	30C
25T	21T	30T
25G	21G	31G
25A	22A	32A
25C	22C	32C
25G	11A_11G	34G
25C	22C	36C
25C	22C	37C
25T	22T	38T

25G	22G	39G
25G	22G	41G
25C	22C	41C
25A	22A	43A
25A	22A	44A
25C	22C	45C
24T	20T	43T
24C	21C	23T_20C
24T	21T	43T
24G	20G	43G
24C	21C	43C
24C	21C	43C
23C	21C	41C
23C	21C	43C
23A	21A	43A
23C	21C	23T_18C
22G	21G	41G
22G	21G	40G
22C	21C	39C
21T	21T	40T
21C	21C	40C
21T	20T	40T
21G	20G	41G
21C	20C	41C
21C	20C	41C
21A	20A	39A
21C	20C	39C
21C	20C	40C
20C	20C	39C
20T	20T	39T
20T	20T	39T
20G	19G	38G
20C	19C	37C
20C	19C	37C
20A	19A	37A
20A	19A	37A
19A	19A	39A
19G	19G	39G
19G	19G	39G
19C	19C	38C
19A	19A	38A
17A	19A	38A
16C	19C	37C

16T	19T	37T
15G	18G	37G
15A	18A	37A
15G	17G	37G
15C	17C	37C
15A	17A	36A
15T	17T	36T
15T	17T	36T
15C	17C	34C
14C	17C	34C
14T	17T	34T
12T	17T	33T
12C	17C	32C
12C	17C	33C
12C	17C	33C
11T	16T	32T
11C	16C	31C
11C	15C	31C
11A	16A	31A
11C	16C	31C
11T	16T	30T
11T	16T	29T
10C	16C	29C
10T	16T	28T
10C	13C	26C
10A	12A	25A
10G	13G	23G
10C	13C	22C
10C	12C	20C
10C	10C	19C
10T	10T	18T
10C	10C	17C
10T	10T	16T
10G	10G	16G
5G_4C	10C	7G_9C
9T	10T	16T
9C	8C	17C
9T	8T	17T
9G	7G	17G
7G	7G	16G
6A	7A	15A
6A	7A	15A
4A	6A	15A

4T	5T	17T
4C	5C	19C
4A	4A	19A
3T_1G	3T	19T
4C	1G_2C	19C
4C	3C	18C
4C	3C	16C
4C	3C	16C
4C	3C	16C
4C	3C	16C
4C	3C	16C
4C	3C	14C
4C	3C	14C
4A	3A	15A
4C	3C	14C
4T	2T	15T
1T_3C	2T	16T
4C	1C	16C
4A	1A	15A
3A	1A	15A
3C	1C	15C
3C	1C	14C
2C	1C	13C
2A	1A	13A
1T	1T	13T
null	1C	13C
null	1A	12A
null	1G	6T_6G
null	1C	12C
null	null	12C
null	null	12T_30C
null	null	12C
null	null	12C
null	null	12C
null	null	12C
null	null	12C
null	null	12C
null	null	12G
null	null	12C
null	null	12T
null	null	13T
null	null	13C
null	null	13A

null	null	13A
null	null	13A
null	null	13C
null	null	13A
null	null	13C
null	null	12T
null	null	12G
null	null	12C
null	null	15C
null	null	15A
null	null	16C
null	null	16C
null	null	15A
null	null	14T
null	null	14C
null	null	14T
null	null	14G
null	null	14T
null	null	13A
null	null	13T
null	null	13T
null	null	13C
null	null	13T
null	null	13C
null	null	13T
null	null	11C
null	null	9A
null	null	9C
null	null	9G
null	null	9C
null	null	9A
null	null	9C
null	null	8C
null	null	8A
null	null	6T_2C
null	null	9G
1G	null	9G
1A	null	10A
1G	null	9G
1T	null	9T
1C	null	17C
1C	null	5C
1C	null	5C

1C	null	9C
1C	null	9C
1C	null	9C
1C	null	8C
1C	null	8C
1C	null	8C
1C	null	8C
1C	null	8C
1C	null	8C
1G	null	8G
1T	1T	9T
2T	1T_1G	10T
2C	2C	10C
2A	2A	10A
2C	2C	10C
2T	2T	10T
2A	2A	11A
2A	3A	11A
2C	3C	11C
2A	3A	11A
3C	3C	11C
3T	3T	11T
3T	3T	11T
4C	3C	11C
4C	4C	13C
4A	4A	13A
4T	4T	13T
4A	4A	12A
4G	4G	12G
4C	4C	12C
4G	2A_2G	6A_5G_1C
5C	4C	15C
5A	4A	15A
6A	6A	16A
6C	6C	16C
6A	6A	17A
7A	6A	16A
7A	6A	17A
7A	6A	16A
7G	6G	16G
6A	6A	17A
7G	6G	18G
7A	6A	19A

7G	6G	20G
7C	6C	21C
8C	6C	21C
8G	6G	22G
8G	6G	23G
8G	6G	23G
8A	6A	24A
8T	6T	24T
8G	7G	24G
8A	7A	25A
8T	7T	25T
8G	7G	25G
8A	8A	24A
8T	8T	26T
8G	8G	28G
9G	8G	27G
9A	8A	29A
9C	10C	31C
11A	10A	32A
11A	11A	33A
11T	11T	33T
11G	11G	33G
11G	10G	31G
12A	11A	30A
13G	12G	30G
13A	12A	31A
13T	12T	32T
14G	12G	33G
15C	12C	33C
15A	12A	34A
14T	13T	34T
15C	13C	35C
16A	13A	35A
16T	13T	37T
16A	13A	37A
16C	13C	38C
16A	13A	38A
16C	13C	38C
16A	12A	39A
17C	14C	39C
17G	18G	39G
17C	18C	39C
17A	19A	40A

17C	19C	39C
18T	19T	39T
18C	19C	39C
18C	19C	39C
18G	19G	39G
18A	19A	40A
19G	19G	42G
19C	19C	43C
19A	19A	41A
19C	19C	41C
19C	20C	42C
19C	20C	43C
20C	20C	43C
20T	19T	43T
21T	20T	43T
22A	21A	40A
22C	21C	40C
22A	21A	39A
22T	21T	39T
22A	21A	38A
22C	21C	38C
22A	22A	37A
22C	22C	36C
22A	22A	36A
22T	22T	34T
22A	23A	33A
11G_11C	23C	19G_14C
22C	23C	32C
22C	24C	31C
22T	25T	19T_13C
23T	25T	31T
24C	25C	30C
24C	25C	32C
24A	26A	32A
23C	27C	32C
23A	27A	33A
23C	27C	32C
23T	27T	33T
23T	27T	34T
24C	27C	34C
24T	27T	34T
25A	28A	32A
25C	27C	33C

25T	28T	35T
25G	27G	34G
25C	27C	33C
25C	30C	32C
26A	30A	32A
26T	30T	32T
26T	30T	32T
26G	30G	32G
26T	29T	32T
26G	29G	31G
26T	30T	32T
26G	30G	31G
26A	32A	30A
26G	32G	31G
25G	32G	32G
26C	31C	31C
26T	31T	31T
25C	33C	29C
26T	33T	29T
26T	33T	28T
26C	33C	28C
26T	33T	26T
25A	33A	26A
26A	33A	26A
25T	32T	26T
26G	32G	26G
26G	32G	24G
26A	31A	25A
26A	32A	25A
26A	33A	26A
26G	34G	26G
26A	33A	26A
26A	35A	26A
26G	36G	24G
26A	37A	23A
26C	37C	24C
25A	37A	26A
25G	37G	25G
25A	38A	25A
25T	38T	26T
26G	38G	26G
26T	37T	25T
26G	37G	27G

26G	39G	27G
26T	39T	27T
26G	40G	30G
27C	39C	32C
27T	41T	33T
26A	41A	34A
27A	41A	34A
28A	41A	34A
28A	39A	34A
26T	39T	35T
26A	39A	36A
27G	39G	36G
28T	39T	36T
28A	40A	35A
27A	40A	37A
26A	39A	39A
26C	39C	39C
26C	39C	39C
24A	39A	41A
23C	39C	40C
23A	40A	41A
23T	39T	40T
23G	40G	39G
22A	40A	39A
21T	40T_1G_26C	38T
22C	41C	38C
23C	42C	13T_37C
23C	42C	31C
23C	42C	31C
23C	42C	31C
19G	40G	null
12G_9C	37G	20C
21G	37G	37G
21G	37G	37G
22G	36G	38G
22A	37A	38A
23G	37G	40G
23A	37A	40A
24A	37A	43A
24A	37A	43A
24A	38A	43A
23A	37A	42A
23G	37G	42G

24G	37G	42G
24A	37A	41A
24A	37A	42A
9A_23G	37G	21A_42G
9A_14G	37A	21A_19G
21G	37G	34A_40G
20A	36A_12G	42A
20A	40A	43A
20A	40A	21A_22G
21A	40A	47A
21A	41A	49A
21A	40A	49A
21A	40A	49A
21A	41A	49A
21A	40A	49A
21G	42G	47G
21G	41G	47G
21A	41A	48A
19A	42A	48A
19T	42T	47T
20C	40C	45C
20A	41A	46A
19A	40A	47A
19G	40G	46G
19C	39C	45C
19A	39A	45A
20A	39A	46A
20T	40T	46T
20C	40C	44C
19A	40A	43A
19A	40A	41A
19G	40G	39G
18C	40C	39C
18A	38A	40A
18A	37A	40A
18T	37T	39T
19G	36G	40G
18C	36C	39C
18T	35T	39T
18T	35T	41T
18A	35A	41A
18T	35T	39T
18G	35G	37G

18G	35G	38G
18A	35A	36A
18C	35C	35C
17C	35C	35C
19A	33A	34A
20A	32A	34A
19G	32G	34G
20G	32G	32G
20A	32A	31A
20A	31A	31A
20G	30G	31G
20A	31A	31A
20A	31A	31A
20A	31A	29A
20A	32A	27A
20G	32G	28G
19A	31A	29A
18A	30A	27A
18A	28A	26A
19G	29G	26G
19A	29A	25A
19A	29A	24A
19G	29G	24G
19G	29G	25G
19T	29T	25T
20T	27T	24T
20C	27C	24C
20T	26T	23T
20G	27G	24G
20G	27G	24G
21G	27G	25G
20G	25G	26G
20A	26A	24A
20G	26G	26G
20G	26G	25G
20A_5G	25A	25A
20G	15A_10G	13A_15G
21G	24G	26G
21G	24G	27G
21G	23G	26G
20G	24G	27G
19G	24G	27G
19G	24G	28G

18G	24G	28G
17T	23T	30T
18T	24T	29T
17G	24G	33G
18G	24G	34G
20G	24G	37G
20G	25G	36G
19A	25A	37A
20G	25G	37G
19A	25A	43A
20G	25G	43G
20G	26G	43G
17T	26T	43T
20T	27T	44T
19T	28T	46T
19T	27T	47T
19A	28A	49A
20T	27T	48T
20G	28G	49G
21G	28G	49G
21C	28C	49C
22A	30A	50A
22T	31T	51T
21C	32C	53C
21T	32T	52T
21A	32A	54A
20T	31T	55T
20A	33A	52A
20G	33G	52G
19A	33A	52A
18G	33G	53G
19A	34A	54A
19G	34G	57G
19A	33A	57A
18G	33G	58G
18G	31G	60G
18A	32A	60A
18A	31A	60A
19G	31G	60G
20G	32G	60G
20C	32C	61C
20T	31T	62T
20A	31A	63A

19T	31T	63T
19G	29G	64G
20G	30G	68G
20G	30G	68G
20A	29A	68A
21T	29T	69T
21G	29G	69G
21G	29G	70G
21G	29G	72G
21A	30A	71A
21G	29G	73G
20T	29T	73T
20T	28T	73T
20C	29C	72C
19A	29A	73A
20A	29A	74A
20G	30G	74G
19A	30A	73A
19G	30G	73G
19A	29A	69A
19C	29C	70C
19T	29T	70T
19T	29T	69T
19G	29G	69G
19A	29A	71A
20G	28G	71G
20A	29A	71A
19A	29A	70A
19C	29C	73C
19A	29A	72A
19T	29T	72T
19T	29T	71T
19G	29G	66G
19A	28A	68A
19G	29G	67G
19T	29T	66T
19T	27T	66T
18G	28G	65G
18T	28T	66T
17T	28T	68T
17G	28G	67G
17C	27C	69C
17C	28C	70C

17A	28A	68A
17T	28T	68T
17T	29T	67T
17T	29T	67T
16T	30T	68T
16C	31C	66C
18A	30A	65A
18G	30G	63G
18G	30G	63G
19G	31G	63G
18A	32A	62A
19A	34A	63A
19T	34T	64T
19A	34A	64A
19A	34A	63A
18T	35T	62T
19C	34C	60C
19C	33C	61C
7A_13G	34G	61A
21A	35A	59A
21A	35A	59A
20G	35G	59G
20A	35A	59A
21G	36G	59G
21C	36C	61C
5T_5G_30C	1A_16T_16G_67C	60C
20G	38G	59G
20C	38C	60C
21C	41C	59C
21T	43T	55T
21C	45C	56C
20G	44G	56G
21G	44G	55G
22A	44A	53A
22T	43T	52T
22A	43A	50A
23A	41A	50A
23A	42A	50A
24G	42G	52G
22C	42C	51C
6A_16G	42A	25A_26G
22G	42G	49G
23C	39C	47C

25T	41T	47T
25C	40C	47C
26C	40C	48C
26C	41C	49C
26C	41C	46C
26A	41A	49A
26T	41T	51T
26C	42C	54C
25G	42G	53G
25T	41T	53T
24G	42G	54G
24G	42G	52G
23C	40C	50C
23T	39T	51T
23C	38C	51C
23A	38A	52A
24T	39T	53T
24G	39G	52G
24A	37A	53A
24T	37T	55T
25T	38T	52T
26C	38C	54C
27A	39A	53A
27C	39C	51C
27A	39A	52A
27C	39C	49C
27A	39A	47A
27A	41A	50A
29C	41C	51C
29T	40T	51T
28T	40T	51T
28C	43C	52C
28C	42C	52C
28C	42C	51C
28T	43T	54T
28G	43G	53G
27T	43T	52T
27A	43A	52A
28C	43C	51C
27T	44T	50T
17T_10C	43T	50T
27T	44T	53T
27G	45G	52G

27C	43C	53C
27A	43A	51A
28T	43T	52T
28T	43T	52T
28G	42G	54G
29T	42T	56T
29G	43G	56G
29T	42T	56T
29T	42T	55T
29T	43T	55T
30G	43G	55G
30C	42C	55C
29C	43C	57C
29T	23T_20C	57T
29G	43G	58G
29G	43G	58G
31G	44G	59G
31C	44C	60C
31A	45A	62A
31G	44G	65G
32C	45C	64C
32A	46A	64A
32A	47A	63A
32A	47A	61A
33C	48C	62C
32C	48C	64C
33T	48T	62T
33G	48G	64G
35G	49G	64G
35G	49G	65G
35C	49C	65C
35G	49G	66G
34G	47G	64G
34G	47G	62G
34G	47G	60G
40A	69A_20T_1G	105A_47T
32A	48A	70A
32T	48T	71T
32A	47A	71A
31T	48T	73T
32A	47A	74A
1A_7T_23G	44G	67T
30A	44A	66A

31C	43C	67C
33T	43T	68T
34C	44C	70C
35C	43C	72C
35T	43T	73T
35G	43G	74G
35C	44C	73C
35T	44T	75T
36G	43G	75G
35T	43T	74T
34T	43T	76T
34G	44G	76G
35C	43C	76C
35C	44C	75C
34A	42A	75A
34T	40T	76T
34T	41T	75T
34T	41T	77T
35T	42T	78T
34A	39A	79A
33C	38C	79C
33A	38A	79A
32G	38G	80G
32A	39A	77A
32T	40T	76T
33T	41T	78T
33A	40A	78A
32A	40A	77A
33T	42T	79T
32T	42T	81T
31A	42A	80A
32A	43A	80A
31C	42C	82C
30A	42A	82A
29G	43G	82G
30A	42A	81A
30G	43G	81G
30G	41G	78G
31T	41T	79T
31G	40G	78G
32G	40G	77G
33A	41A	78A
32G	41G	79G

33G	41G	78G
34G	42G	75G
34C	44C	77C
34A	45A	76A
36A	46A	76A
37A	46A	76A
25T_12C	46T	76T
37A	45A	78A
37G	45G	79G
36A	44A	81A
36C	45C	82C
35C	44C	85C
34G	44G	84G
35A	43A	84A
36T	43T	85T
37C	43C	86C
36A	41A	85A
36A	41A	83A
36A	42A	80A
34G	41G	80G
34C	39C	79C
36C	39C	80C
36C	39C	81C
38A	38A	81A
38A	39A	81A
38G	41G	82G
38C	40C	83C
37C	40C	83C
38A	40A	82A
37C	40C	81C
37C	38C	81C
37G	38G	81G
36A	39A	42A_41G
39G	40G	84G
38C	39C	83C
39C	41C	83C
39A	41A	82A
39G	41G	79G
40A	41A	78A
39A	41A	79A
40G	40G	78G
40G	39G	79G
39C	39C	78C

39T	40T	75T
39G	40G	74G
42C	40C	74C
42A	40A	74A
42A	41A	75A
43G	40G	76G
44G	41G	76G
43G	40G	76G
43A	40A	76A
44A	40A	74A
44G	21A_17G	75G
43C	38C	75C
43A	37A	73A
42A	37A	70A
43G	38G	70G
43T	38T	70T
43C	23T_17C	30T_38C
25A_18G	44G	67G
42G	44G	66G
41C	44C	66C
41T	44T	67T
41T	44T	65T
41C	44C	66C
41T	44T	68T
41C	44C	69C
41T	44T	68T
41G	43G	67G
42C	43C	67C
42T	43T	64T
42C	44C	65C
42T	43T	65T
41A	43A	64A
42G	43G	62G
43A	43A	62A
43C	44C	60C
41A	44A	59A
39A	44A	56A
39G	44G	56G
39G	43G	52G
40T	44T	52T
40A	43A	51A
40T	43T	50T
39A	43A	48A

40T	43T	49T
40A	44A	50A
40G	43G	51G
39A	44A	52A
39A	44A	51A
40G	45G	47G
40A	46A	48A
41G	45G	47G
44G	45G	46G
42A	45A	44A
42G	45G	43G
42G	45G	41G
43G	46G	41G
43C	46C	41C
43G	45G	42G
43G	45G	41G
41T	44T	37T
41G	44G	37G
41G	44G	36G
40T	42T	35T
41T	42T	34T_17G
12A_32G	34G	35G
41G	34G	44G
41G	34G	44G
43G	34G	44G
43G	64T_65G	46G
24T_62G	32G	48G
44G	33T	20T_79G
44G	38G	50G
44T	39T	43T
46G	39G	52G
44G	39G	52G
41C	39C	51C
41A	39A	52A
43C	39C	53C
44C	40C	52C
44T	40T	51T
44C	39C	51C
44T	39T	53T
43T	40T	53T
43C	40C	53C
42C	40C	54C
42T	39T	55T

42A	40A	58A
42G	42G	60G
42C	43C	60C
42C	43C	59C
42A	43A	59A
41A	44A	59A
40A	42A	59A
40A	42A	58A
40T	21A_20T	58T
38T	41T	61T
40G	41G	61G
39G	41G	25A_36G
39T	39T	61T
39T	40T	62T
39C	42C	62C
39T	43T	62T
40G	45G	63G
41T	46T	64T
41T	47T	65T
41G	50G	66G
42C	50C	65C
42C	50C	37G_26C
42A	50A	63A
43G	49G	66G
42A	49A	64A
42G	49G	66G
42C	49C	66C
42A	49A	68A
42G	49G	68G
41C	50C	66C
41C	49C	68C
41A	50A	71A
41A	50A	71A
38C	50C	73C
39C	50C	71C
39A	49A	72A
39G	49G	73G
40A	49A	70A
40T	49T	69T
40G	48G	71G
39C	47C	71C
39C	46C	71C
37C	47C	72C

38A	47A	73A
39C	48C	72C
39G	48A	74G
40G	48G	75G
41G	46G	74G
40A	44A	72A
41T	45T	71T
41G	45G	71G
41T	45T	71T
42T	45T	70T
42C	47C	72C
43C	48C	73C
44T	48T	74T
43A	49A	74A
44A	50A	70A
44G	51G	69G
44C	52C	71C
44A	52A	70A
44G	51G	70G
45G	51G	71G
45A	51A	71A
44T	51T	70T
45G	50G	69G
24T_19C	50T	70C
43G	51G	68G
44A	52A	71A
44G	52G	70G
44A	52A	70A
44T	53T	69T
43C	55C	69C
43A	55A	70A
44A	55A	69A
45C	54C	68C
45A	53A	67A
45A	53A	68A
46C	53C	69C
45A	53A	67A
45C	52C	68C
46G	53G	69G
27T_19C	54T	32T_38C
48T	53T	68T
48C	51C	69C
44A	51A	69A

44C	52C	69C
44T	52T	68T
44T	51T	68T
43T	50T	67T
43C	50C	66C
44C	51C	68C
44C	52C	67C
44C	52C	66C
44G	53G	67G
43G	53G	65G
43C	54C	65C
42A	54A	64A
18A_23G	52A	66A
41C	53C	67C
41T	52T	65T
40T	52T	62T
40G	52G	61G
39T	51T	57T
38A	51A	59A
37C	52C	57C
40T	54T	60T
40A	55A	60A
40T	55T	61T
40G	55G	60G
41T	58T	61T
41G	63G	61G
41A	62A	61A
44A	64A	62A
44G	65G	63G
43C	64C	60C
43T	63T	60T
44T	65T	62T
44C	65C	64C
45A	65A	64A
46A	65A	64A
46A	62A	64A
47C	64C	63C
47A	64A	61A
47C	64C	63C
49C	64C	63C
49A	65A	63A
50A	65A	65A
50A	65A	68A

50G	65G	68G
49G	63G	71G
50G	65G	70G
50C	65C	71C
51A	63A	74A
50T	62T	74T
50A	62A	76A
50G	60G	74G
48G	60G	74G
52A	61A	74A
54T	59T	75T
55A	60A	72A
54T	60T	71T
55C	61C	72C
54A	61A	72A
54T	61T	71T
54C	61C	71C
15G_39C	62C	71C
54T	63T	71T
54A	63A	72A
54T	63T	71T
54A	63A	70A
54A	62A	68A
55C	63C	69C
56C	63C	69C
55C	64C	69C
58T	64T	69T
59C	63C	69C
58C	62C	68C
58C	66C	69C
58A	66A	68A
60A	67A	70A
59A	67A	72A
59G	66G	72G
58C	68C	72C
58T	67T	70T
58T	67T	70T
57G	66G	71G
55C	66C	70C
55C	67C	70C
55T	67T	73T
55C	68C	75C
55T	69T	75T

55C	71C	74C
55C	69C	75C
55A	69A	73A
54C	70C	76C
54G	69G	75G
54A	68A	76A
54A	66A	76A
54G	66G	75G
55A	64A	74A
54A	64A	74A
54G	64G	72G
53C	64C	71C
54G	65G	71G
54T	66T	71T
57C	65C	73C
57T	64T	73T
41T_15C	64T	38T_34C
56C	65C	76C
57T	63T	75T
41A_16C	62C	76C
58T	62T	75T
59G	62G	73G
58A	63A	72A
42T_16G	27T_36G	35T_37G
58G	63G	72G
57T	63T	72T
56C	66C	68C
57T	68T	67T
59T	68T	65T
59T	68T	65T
60T	67T	65T
61T	68T	63T
60A	68A	64A
61A	68A	63A
64A	66A	65A
64A	66A	64A
64G	66G	64G
64C	65C	64C
64C	66C	65C
21A_44G	65G	66G
65C	64C	66C
66C	64C	65C
70A	68A	67A

70G	69G	68G
70G	68G	68G
71G	69G	67G
72C	70C	67C
72T	69T	67T
73G	67G	67G
74G	70G	68G
72C	68C	67C
74A	70A	68A
73G	69G	68G
74C	69C	70C
74G	68G	70G
75A	69A	72A
76T	70T	71T
76G	70G	71G
75A	68A	71A
73C	61C	69C
70G	28A_33G	69G
70G	62G	72G
71C	63C	73C
72T	63T	75T
73A	64A	78A
72T	63T	77T
72T	64T	75T
71T	65T	71T
70T	67T	70T
70G	67G	70G
69T	65T	72T
71C	66C	75C
70A	66A	77A
71G	66G	74G
72T	65T	75T
74T	64T	75T
74T	65T	74T
74T	65T	75T
73C	65C	77C
72C	66C	78C
72A	66A	78A
72A	66A	78A
72A	70A	79A
72A	71A	78A
72G	71G	76G
72A	69A	76A

71G	70G	75G
68T	69T	71T
67T	68T	70T
67C	67C	70C
65T	66T	71T
69T	65T	73T
68G	67G	74G
69A	67A	72A
69A	65A	73A
69G	65G	71G
68T	67T	73T
70C	67C	74C
70G	67G	74G
70T	67T	73T
69T	69T	75T
40A_28G	68A	76A
68G	68G	75G
66A	35A_34G	45A_32G
65C	69C	79C
64T	70T	79T
66G	67G	81G
66G	68G	82G
64A	69A	83A
65C	69C	82C
65T	69T	80T
65C	68C	81C
65G	33A_34G	35A_43G
65C	69C	78C
65C	70C	79C
65A	71A	80A
65T	69T	81T
66G	68G	81G
67G	69G	79G
68C	68C	80C
68A	66A	82A
68G	67G	81G
67A	65A	80A
67A	65A	79A
67G	63G	78G
67C	64C	76C
66A	65A	76A
66G	67G	77G
65C	68C	75C

66C	68C	76C
67A	68A	76A
68A	68A	73A
68G	67G	72G
70C	66C	70C
68T	66T	67T
68G	66G	68G
68A	66A	69A
71C	66C	70C
70T	69T	69T
69C	70C	69C
68T	69T	66T
68T	68T	63T
71C	68C	64C
70T	68T	67T
70G	68G	67G
71C	67C	69C
70A	65A	72A
71G	64G	73G
69C	64C	72C
67A	63A	73A
68A	63A	73A
66G	63G	73G
66A	62A	72A
66C	63C	73C
64C	63C	73C
65T	64T	71T
66G	65G	72G
67T	64T	73T
66C	65C	73C
65C	65C	71C
66T	65T	70T
65T	65T	67T
62C	61C	67C
62C	60C	67C
62A	57A	67A
62C	59C	67C
61A	58A	66A
62T	59T	64T
61C	59C	64C
59C	57C	66C
59T	57T	65T
59T	56T	64T

61G	56G	63G
59A	57A	63A
61T	56T	61T
60T	57T	63T
59G	57G	60G
59C	57C	59C
59C	57C	59C
59T	56T	58T
59C	56C	59C
25G_34C	57G	59G
59T	57T	58T
58C	60C	59C
57T	59T	58T
57T	58T	58T
58T	59T	58T
58T	59T	60T
58C	58C	60C
58A	59A	60A
59A	61A	58A
57G	60G	61G
56A	61A	63A
54T	60T	63T
23T_30C	60T	63T
54C	60C	65C
56C	62C	65C
56A	63A	66A
56C	63C	67C
57C	62C	68C
57A	63A	69A
56G	63G	69G
56T	61T	72T
56T	60T	72T
54T	60T	72T
56C	60C	73C
55C	59C	72C
55T	59T	74T
55T	58T	77T
55C	58C	77C
55A	59A	78A
52A	60A	78A
52A	61A	75A
51T	28T_33C	73T
53G	63G	72G

54G	63G	69G
53A	62A	65A
52C	62C	66C
52A	61A	65A
52T	61T	63T
53T	59T	63T
56A	60A	65A
57A	60A	65A
57G	59G	65G
56C	59C	65C
58T	58T	66T
58G	58G	65G
58G	57G	65G
57C	55C	66C
57C	55C	67C
57G	54G	67G
58G	55G	65G
58C	55C	66C
58C	53C	68C
58T	51T	68T
57C	50C	71C
58T	51T	70T
57A	51A	70A
56G	53G	68G
55T	54T	67T
57T	54T	70T
57T	54T	72T
57C	54C	73C
58C	54C	73C
60A	54A	72A
61G	54G	72G
61G	54G	73G
61A	53A	71A
61T	24T_28C	33T_38C
61C	52C	71C
61C	52C	70C
61C	52C	70C
62A	52A	69A
60C	53C	68C
60C	53C	70C
59C	53C	71C
58C	54C	71C
56T	52T	69T

56G	48G	69G
56G	49G	68G
57A	48A	68A
58C	48C	70C
56C	48C	68C
56C	48C	69C
57T	48T	69T
56T	48T	67T
55T	48T	67T
54C	48C	67C
55T	49T	69T
55G	49G	72G
55A	50A	73A
55A	50A	71A
56A	50A	69A
55A	50A	71A
54G	50G	72G
53G	49G	70G
52C	48C	69C
51T	49T	69T
51G	48G	67G
50G	49G	67G
51T	49T	64T
52G	49G	67G
56C	49C	68C
56T	49T	68T
56A	49A	69A
55C	50C	69C
56C	50C	71C
55C	48C	70C
55T	49T	69T
56G	49G	69G
55G	48G	69G
56C	47C	68C
55C	47C	69C
55G	47G	69G
55C	48C	70C
55T	47T	72T
56T	48T	73T
58T	49T	72T
58C	48C	73C
58C	50C	73C
57A	50A	73A

56G	48G	73G
55G	49G	73G
55A	47A	72A
57G	47G	72G
57A	46A	71A
56C	44C	71C
56A	43A	72A
55A	43A	75A
57G	42G	75G
56G	41G	74G
57A	41A	73A
57A	42A	74A
57G	43G	75G
56C	43C	75C
52A	42A	74A
53C	43C	73C
53A	42A	72A
52T	46T	72T
54G	46G	73G
57C	47C	73C
58A	47A	74A
58A	44A	74A
60A	46A	76A
60C	47C	75C
58A	47A	76A
60A	47A	78A
60A	47A	80A
60A	46A	81A
61C	47C	81C
60A	48A	80A
60C	46C	76C
60G	42G	78G
61A	46A	77A
61T	45T	79T
61T	46T	80T
61T	44T	84T
61G	46G	85G
61T	47T	81T
61A	46A	79A
61C	46C	79C
59A	48A	80A
58A	49A	83A
58C	49C	81C

58T	49T	80T
57A	50A	81A
57T	51T	80T
57C	51C	78C
58T	51T	80T
61T	52T	80T
61A	51A	82A
60T	51T	85T
60C	51C	85C
60C	51C	86C
60T	51T	85T
60G	51G	86G
36A_23G	52G	89G
59G	54G	88G
60C	51C	89C
61A	51A	90A
59C	52C	91C
59A	23A_29G	92A
60C	53C	90C
59T	53T	89T
57T	53T	90T
57T	53T	89T
57T	52T	87T
58C	52C	86C
57T	51T	86T
57G	52G	85G
57A	52A	84A
56A	52A	85A
56C	51C	87C
58C	52C	86C
14A_68T_31G_56C	26A_78T_53G_104C	44A_143T_92G_158C
61C	60C	103C
55T	57T	68T
55G	57G	68G
55T	56T	71T
55G	57G	72G
58C	57C	74C
24A_33T	35A_22T	77A
58T	57T	81T
58T	59T	82T
57T	59T	82T
58C	59C	83C
60A	59A	83A

35T_25C	59T	83C
61A	58A	83A
62G	58G	84G
62C	58C	87C
62C	60C	89C
63T	60T	91T
64T	60T	91T
65C	60C	92C
65T	61T	93T
63G	64G	40A_52G
63T	63T	91T
62T	65T	90T
64G	65G	89G
65C	67C	90C
64A	68A	88A
32A_29G	69A	38A_49G
60C	68C	86C
59C	40T_29C	86C
60A	70A	87A
60C	68C	87C
60C	69C	86C
60A	68A	86A
59A	68A	87A
59T	67T	86T
60G	66G	86G
59G	67G	86G
60A	67A	82A
59C	68C	81C
60C	66C	79C
59T	66T	80T
58G	66G	78G
58T	66T	76T
58T	66T	76T
58G	67G	76G
58G	67G	75G
59C	65C	73C
59T	66T	73T
59A	67A	73A
60C	67C	73C
59A	70A	75A
59G	68G	72G
61A	69A	68A
61C	69C	66C

61G	68G	68G
63A	68A	69A
62T	66T	69T
62G	67G	69G
62A	67A	68A
60C	65C	69C
61A	65A	70A
61A	65A	71A
60C	63C	70C
60A	63A	70A
59T	64T	70T
58T	64T	65T
59C	62C	64C
57G	62G	61G
57C	64C	62C
57A	64A	63A
57A	62A	63A
57C	63C	65C
57T	63T	62T
59C	61C	65C
58A	62A	64A
59A	62A	61A
58A	61A	58A
57G	59G	57G
56C	60C	59C
56C	60C	58C
56G	59G	60G
54T	59T	60T
55C	59C	61C
57C	59C	61C
59T	60T	60T
58T	60T	60T
58T	59T	61T
58A	59A	63A
58T	60T	63T
57T	59T	64T
57G	61G	64G
56G	61G	65G
56G	62G	67G
55C	61C	67C
56T	62T	66T
56G	61G	65G
57T	62T	67T

57A	61A	68A
55T	62T	66T
55T	62T	65T
55T	62T	66T
55T	61T	66T
55A	60A	68A
55T	60T	67T
28T_25G	25T_33G	21T_45G
29A_28G	24A_33G	68A
60T	56T	68T
60T	56T	68T
60T	57T	68T
62T	57T	68T
61A	55A	67A
60G	56G	67G
60A	56A	69A
59C	55C	70C
60A	55A	69A
61G	56G	67G
60T	56T	67T
58G	55G	67G
56A	56A	67A
57A	55A	66A
57G	55G	63G
57C	57C	64C
57C	57C	64C
57T	58T	64T
54G	58G	63G
55A	59A	63A
55T	59T	64T
57C	57C	64C
59C	57C	65C
60A	58A	65A
59C	58C	66C
59A	59A	65A
58G	59G	66G
57A	60A	65A
57A	60A	66A
57G	58G	67G
56A	60A	67A
55C	61C	69C
54T	61T	69T
54G	60G	70G

55A	57A	73A
55T	55T	73T
55T	55T	73T
53T	55T	72T
52A	54A	72A
52A	53A	73A
52G	52G	72G
52A	52A	70A
52G	52G	70G
52C	50C	70C
53A	51A	69A
53A	52A	71A
53G	52G	71G
53G	52G	72G
53A	52A	71A
52A	51A	73A
52A	53A	71A
29A_23T	17A_36T	71A
52G	54G	70G
51G	54G	72G
52A	54A	72A
52A	55A	73A
29A_23G	55G	72G
54G	55G	73G
54C	54C	76C
54T	53T	76T
54T	52T	75T
55T	53T	73T
55C	53C	75C
54T	52T	76T
55C	51C	76C
55C	49C	77C
54C	49C	75C
54T	50T	76T
55G	50G	75G
53A	50A	74A
52T	50T	78T
53A	49A	79A
52A	51A	79A
52A	52A	84A
51G	52G	84G
19G_32C	34G_18C	46G_39C
52A	52A	83A

52C	52C	83C
52T	50T	86T
51A	49A	87A
50T	50T	88T
50A	50A	88A
50G	48G	88G
50G	48G	88G
53A	48A	89A
53A	48A	90A
53G	48G	91G
55A	47A	91A
55A	48A	92A
55A	49A	91A
55A	49A	90A
56G	50G	91G
56G	50G	89G
55C	50C	88C
56A	50A	39A_49T
56G	49G	88G
56G	49G	88G
30T_24C	19T_30C	87T
52G	49G	88G
53A	48A	85A
55T	47T	89T
55C	49C	89C
57T	48T	88T
56T	49T	87T
56A	48A	85A
57G	48G	86G
57T	47T	86T
56G	47G	86G
57G	48G	85G
56G	49G	87G
57T	49T	87T
57T	48T	88T
57T	50T	88T
57T	50T	89T
57T	49T	88T
56C	49C	87C
55T	49T	86T
54A	49A	87A
57T	50T	89T
53G	51G	85G

52T	52T	86T
53T	52T	84T
53C	52C	83C
51A	53A	80A
52T	54T	80T
21G_31C	32G_23C	80C
52T	55T	79T
52T	55T	79T
52T	56T	77T
52A	56A	78A
53A	56A	78A
53T	56T	78T
31T_22C	24T_31C	32T_44C
53T	55T	78T
53C	55C	75C
53T	55T	76T
55T	56T	77T
55T	55T	76T
55G	55G	76G
56G	54G	77G
56C	55C	75C
54G	55G	75G
53T	55T	75T
52A	55A	73A
51T	55T	71T
51T	55T	72T
51A	55A	70A
51A	54A	69A
51G	54G	66G
55C	54C	67C
56C	53C	68C
57T	52T	67T
57T	52T	68T
57G	52G	68G
57A	53A	68A
57T	53T	67T
57T	51T	67T
57A	52A	67A
58C	53C	66C
58T	54T	69T
59T	54T	68T
59A	54A	70A
59A	55A	70A

59A	55A	71A
58T	55T	70T
58T	54T	70T
58G	53G	73G
58T	54T	73T
58A	54A	74A
58A	54A	74A
58T	53T	76T
57T	54T	75T
57A	52A	75A
57T	52T	74T
56G	51G	72G
56G	50G	73G
57C	50C	71C
55A	50A	28A_45G
54T	51T	72T
54A	51A	71A
54T	51T	71T
52C	51C	71C
51T	51T	74T
53T	50T	74T
52T	50T	74T
50T	50T	78T
51G	50G	79G
51G	50G	78G
51G	49G	75G
51T	48T	77T
53T	48T	76T
54G	48G	77G
54G	46G	78G
54T	44T	80T
55T	45T	82T
54T	47T	80T
52T	48T	84T
52G	48G	83G
52A	49A	82A
53A	49A	82A
54T	48T	84T
54T	48T	85T
54T	48T	84T
53A	48A	82A
51C	48C	83C
52T	50T	84T

52G	51G	84G
48G	53G	84G
48T	53T	82T
48G	53G	83G
48G	52G	83G
47G	51G	84G
47T	52T	84T
47G	52G	84G
47G	53G	82G
25T_21G	51T	83T
46G	53G	83G
46T	53T	81T
46G	53G	84G
44T	33T_21G	53T_31G
25T_39G	24T_30G	80T
19T	47T	76T
42T	53T	82T
42T	53T	82T
44T	52T	37T_45C
44T	52T	83T
43T	52T	83T
43T	52T	84T
43T	50T	84T
43C	49C	84C
42T	49T	86T
42T	49T	84T
41T	49T	84T
41T	50T	48A_34T
41C	51C	83C
41A	51A	84A
41A	51A	80A
41A	50A	80A
37A	51A	81A
37A	50A	80A
15A_23T	49T	78T
37G	49G	76G
37C	47C	76C
36T	47T	78T
36T	47T	77T
37T	46T	75T
37T	46T	74T
37T	46T	73T
37G	45G	73G

37T	44T	73T
37T	43T	70T
37T	44T	71T
37C	43C	69C
37T	45T	72T
37C	44C	73C
37C	43C	69C
38C	43C	69C
38T	44T	68T
36C	44C	69C
36C	43C	69C
36T	43T	69T
35C	42C	70C
34T	44T	72T
34A	44A	70A
34C	44C	68C
34C	44C	68C
34T	44T	64T
33G	45G	63G
34T	46T	66T
34G	47G	66G
34A	46A	67A
29T	46T	66T
28G	47G	68G
30G	47G	68G
30A	47A	68A
30T	47T	70T
29T	47T	69T
29T	46T	69T
30G	45G	69G
30A	44A	70A
14A_17C	44C	69A
31A	43A	69A
30A	43A	69A
31G	43G	71G
33A	43A	70A
1A_16G_16C	44G	70G
33G	45G	72G
32T	45T	71T
33G	45G	74G
32T	46T	73T
33T	45T	72T
33T	44T	71T

34T	44T	70T
34C	46C	72C
18A_16G	25A_1T_20G	34A_41G
34G	46G	74G
36C	47C	75C
36A	48A	74A
35T	48T	73T
35G	48G	71G
35G	48G	69G
35C	47C	71C
35G	47G	70G
34T	48T	69T
36G	48G	67G
36G	48G	66G
34T	48T	67T
35G	47G	67G
35A	48A	66A
36A	48A	67A
18T_15G	27T_20G	31T_36G
15A_18T	20A_28T	37A_31T
35A	50A	72A
36A	51A	71A
35A	52A	73A
35A	52A	74A
35C	51C	76C
35A	52A	76A
36G	52G	76G
36G	52G	77G
36A	53A	77A
37T	53T	78T
36T	54T	79T
37A	55A	76A
38A	56A	74A
38A	54A	76A
38A	53A	77A
39A	51A	77A
40T	49T	75T
40T	49T	73T
40A	49A	72A
40G	49G	71G
40C	47C	69C
40C	48C	71C
41A	49A	71A

43A	51A	71A
44C	51C	73C
44T	52T	76T
44C	51C	72C
44A	49A	73A
45C	50C	71C
44T	52T	72T
44A	53A	70A
43A	53A	69A
42A	57A	71A
42T	57T	69T
42G	58G	72G
43A	57A	72A
43G	58G	73G
43C	59C	69C
43A	57A	71A
43A	57A	70A
42C	56C	71C
43T	55T	70T
43G	54G	73G
43C	54C	74C
43T	53T	75T
44C	54C	75C
44A	54A	72A
45G	54G	73G
44A	54A	75A
44A	54A	76A
45A	54A	76A
44A	54A	74A
44G	53G	72G
44G	54G	73G
44A	55A	72A
44A	56A	71A
42G	56G	72G
42G	56G	71G
42C	56C	73C
41A	57A	71A
41G	57G	71G
41T	55T	72T
41C	56C	75C
41C	56C	74C
40A	56A	75A
40A	57A	74A

41T	57T	73T
41T	58T	71T
41C	59C	69C
41T	59T	69T
41G	58G	74G
41C	57C	75C
41A	57A	76A
42C	57C	78C
42T	59T	78T
42T	58T	79T
42T	60T	80T
42C	59C	83C
43C	60C	84C
44A	60A	83A
44G	59G	82G
44C	59C	82C
45A	58A	83A
46G	58G	82G
46A	60A	82A
46A	60A	87A
44A	61A	88A
44A	61A	87A
43T	62T	90T
43C	62C	92C
42C	62C	94C
42A	63A	92A
40A	63A	94A
40G	61G	96G
39T	62T	94T
40A	62A	91A
15G_25C	63G	94G
41C	36A_26C	94C
40T	62T	94T
40C	64C	94C
39T	63T	95T
41A	63A	94A
41A	38A_26C	94A
42G	64G	93G
40A	63A	90A
43T	61T	89T
44T	61T	89T
44T	61T	88T
44C	63C	88C

44C	64C	87C
43A	65A	87A
44A	64A	91A
43C	64C	89C
44C	65C	89C
44C	65C	88C
44A	65A	89A
44G	64G	91G
42G	64G	90G
42A	63A	89A
41C	64C	89C
42T	65T	93T
43T	63C	53T_40C
42T	63T	92T
43T	60T	91T
42T	63T	91T
21T_23C	63C	53T_39C
45C	63C	94C
46T	62T	94T
46C	62C	54T_39C
43G	62G	94G
22A_21G	61G	93G
43G	59G	95G
1T_22G_20C	60C	93C
24A_20G	59A	41A_55G
44C	62C	95C
42T	62T	95T
45T	63T	95T
45T	62T	94T
45T	64T	95T
45T	64T	93T
46T	66T	90T
46G	65G	90G
45T	65T	88T
45C	63C	86C
43T	63T	85T
43A	63A	84A
43G	63G	84G
43G	64G	80G
42A	63A	83A
42A	60A	82A
25T_16C	60T	33T_48C
41C	61C	83C

42A	59A	83A
43G	59G	85G
44C	57C	84C
43C	57C	83C
42A	59A	83A
42G	58G	83G
43G	58G	86G
44A	58A	85A
45A	56A	84A
45G	58G	86G
45A	62A	82A
46G	63G	81G
46A	64A	81A
45G	64G	83G
45A	64A	81A
44T	65T	79T
43T	65T	78T
45C	66C	77C
44G	66G	76G
45G	65G	76G
45T	66T	74T
45T	65T	75T
45C	64C	79C
45T	63T	79T
47T	63T	79T
47T	64T	80T
47T	64T	79T
48C	63C	80C
51A	66A	80A
52C	67C	76C
54A	66A	78A
55A	66A	79A
55A	67A	80A
55A	65A	81A
21T_33C	65T	82T
53C	65C	82C
54A	64A	83A
54G	64G	82G
54T	65T	79T
54A	65A	79A
54A	67A	80A
54T	66T	82T
54C	65C	82C

54T	65T	84T
54T	63T	83T
54T	63T	84T
53G	62G	86G
52G	62G	87G
52A	62A	90A
51C	64C	87C
50A	65A	89A
21T_28C	65T	87T
49G	64G	87G
49A	64A	91A
49T	66T	91T
49G	66G	91G
49A	65A	89A
49T	65T	90T
49C	65C	90C
49G	64G	89G
49C	63C	89C
51A	64A	92A
51C	63C	92C
51A	63A	93A
52G	62G	93G
51A	63A	93A
51C	65C	95C
51T	64T	95T
51A	64A	94A
50G	65G	93G
50A	64A	91A
51G	64G	93G
51C	65C	90C
48C	65C	91C
47G	65G	89G
48C	65C	90C
49G	65G	86G
48G	65A	49A_36G
48T	64T	85T
47A	64A	83A
47T	69T	84T
48C	69C	84C
48T	68T	85T
48G	67G	85G
48A	69A	87A
49C	68C	88C

49G	69G	90G
49C	68C	93C
48A	66A	93A
47G	65G	92G
47A	66A	94A
46C	66C	93C
46A	66A	91A
45A	67A	91A
44T	68T	90T
43T	68G	91G
43A	69A	94A
45A	70A	94A
45G	70G	94G
45T	71T	94T
44C	69C	94C
44C	72C	92C
44T	70T	92T
45T	71T	91T
42G	70G	88G
43G	72G	89G
43C	70C	87C
47A	69A	88A
47G	68G	88G
47C	67C	89C
47T	67T	89T
47A	67A	89A
47T	67T	91T
47C	65C	90C
48G	66G	89G
49C	65C	90C
51C	64C	90C
53A	66A	87A
53A	67A	85A
53G	67G	84G
52C	67C	83C
51G	67G	84G
50T	68T	85T
50C	69C	82C
50C	68C	82C
50T	68T	82T
50T	68T	82T
48C	68C	81C
48T	68T	82T

48G	68G	86G
48G	64G	86G
47C	64C	86C
48G	63A	34A_51G
48T	64T	83T
49T	64T	84T
49A	64A	84A
49A	65A	81A
49A	65A	81A
49G	65G	85G
49C	65C	85C
48T	65T	87T
49G	68G	88G
50A	68A	88A
51G	68G	89G
49G	69G	89G
50A	69A	89A
29A_21G	69G	91G
49A	69A	90A
47A	66G	56A_35G
46G	65G	91G
45G	65G	91G
44A	65A	91A
43T	63T	91T
45C	65C	91C
46G	65G	88G
47G	64G	90G
47G	64G	56A_35G
47A	64A	88A
47A	64A	93A
48C	64C	94C
49G	63G	93G
48T	64T	92T
47A	63A	91A
48A	63A	92A
50G	63G	93G
50A	63A	92A
49T	63T	89T
50C	62C	88C
99T_50C	112T_61C	157T_87C
52C	57C	90C
55C	59C	94C
56A	60A	94A

58C	61C	95C
58T	61T	94T
58G	61G	96G
58C	61C	96C
58C	63C	96C
58A	64A	94A
58A	64A	93A
56C	64C	92C
56T	65T	90T
56T	66T	90T
55T	67T	90T
55G	67G	88G
57G	65G	87G
57A	65A	87A
57G	65G	88G
57A	63A	89A
57C	62C	88C
56A	63A	81A
56C	62C	39T_42C
56A	61A	85A
56A	63A	85A
57A	62A	85A
56G	62G	86G
57C	61C	89C
57C	61C	89C
57A	62A	91A
56C	57C	85C
54A	58A	83A
54G	58G	83G
55G	56G	84G
55A	60A	83A
57A	60A	83A
57A	61A	84A
58A	60A	82A
59A	60A	82A
59G	61G	84G
60C	59C	87C
61A	61A	83A
59C	59C	81C
59A	57A	83A
59A	58A	82A
58A	58A	81A
58A	58A	80A

57G	59G	80G
57C	58C	78C
56T	59T	80T
57A	59A	81A
59T	57T	83T
59T	56T	83T
59C	55C	83C
60A	58A	85A
59G	56G	84G
60A	56A	83A
56T	57T	79T
55C	57C	81C
57T	58T	78T
57G	58G	80G
55C	35T_23C	79C
55C	55C	80C
55A	57A	83A
54T	57T	86T
53G	56G	84G
51T	55T	83T
49A	56A	80A
49G	54G	77G
49A	56A	79A
50C	54C	77C
50A	53G	75G
51C	53C	73C
51A	52A	74A
51C	52C	74C
51A	52A	74A
51G	53G	71G
51A	53A	73A
55C	53C	73C
54T	53T	74T
54G	53G	76G
54T	52T	77T
54T	53T	76T
54T	53T	77T
53G	52G	79G
53G	51G	79G
53G	50G	79G
72A_38C	50A	78A
32C	51C	80C
32A	53A	82A

31C	53C	80C
50A	50A	82A
49C	50C	83C
47A	50A	86A
50C	51C	87C
50A	50A	89A
24G_23C	49C	88C
47A	50A	85A
47C	50C	87C
70A_23G_23C	51A	85A
23G	24A_49G_26C	77G
23A	51A	77A
21A	51G	78G
47A	50A	78A
47T	49C	77C
47G	49G	40A_37G
47T	49T	81T
48A	49A	81A
49C	47C	80C
49A	48A	80A
49C	47C	82C
50A	46A	86A
50C	46C	88C
49T	46T	86T
48G	46G	86G
48A	47A	85A
48A	48A	86A
49C	46C	86C
48C	48C	86C
48T	48T	84T
48C	17A_29C	86C
48A	47A	84A
47G	46G	85G
46A	46A	86A
45G	46G	87G
45G	46G	87G
43G	46G	85G
42T	46C	87C
41T	44C	88C
42G	44G	86G
43C	44C	84C
44A	43A	85A
45C	44C	85C

45A	44A	84A
45C	43C	80C
45A	43A	81A
43A	40A	81A
46C	42C	80C
48C	45C	78C
48C	47C	79C
47C	47C	78C
45C	47C	78C
48A	49A	79A
48A	49A	79A
49G	48G	41T_38G
50C	48C	79C
50C	49C	79C
50T	49T	77T
51C	49C	78C
49T	47T	78T
49G	47G	80G
48C	47C	78C
49A	45A	83A
50T	46T	84T
51G	46G	84G
51T	48T	83T
51T	48T	82T
50A	49A	83A
50T	49T	80T
48C	48C	78C
48C	48C	79C
48T	48T	75T
47C	47C	76C
47T	48T	75T
46G	47G	74G
45A	47A	73A
45A	47A	75A
45T	45T	77T
45G	45G	73G
45A	46A	74A
46T	46T	75T
45T	47T	75T
45T	47T	74T
46T	49T	75T
46A	49A	76A
44T	51T	77T

45T	51T	77T
44T	50T	80T
42A	51A	81A
42A	50A	79A
41A	50A	78A
41A	51A	75A
41G	51G	76G
40G	51G	77G
40C	51C	77C
40C	51C	79C
41C	50C	78C
41T	50T	79T
41T	50T	80T
41T	48T	80T
42T	49T	80T
42A	49A	81A
42A	48A	80A
42A	49A	79A
41G	49G	78G
41G	49G	80G
39G	49G	81G
41G	49G	79G
41G	49G	81G
41C	50C	82C
41A	49A	83A
42A	51A	82A
38G	52G	81G
38A	53A	82A
39A	53A	83A
39G	52G	82G
39G	52G	83G
39G	52G	84G
39A	50A	83A
39A	49A	83A
38T	49T	84T
39C	48C	86C
39G	48G	87G
39T	46T	87T
37C	46C	87C
35C	46C	87C
35T	27T_20C	43T_46C
35G	47G	89G
35C	47C	87C

36G	46G	85G
36T	47T	81T
36T	47T	81T
37T	46T	77T
38C	46C	76C
40A	44A	75A
40C	46C	75C
40A	45A	76A
39C	46C	76C
38C	46C	76C
39T	46T	77T
40G	45G	76G
39T	47T	76T
40G	47G	77G
40G	46G	36A_1T_39G
40C	47C	76C
39T	47T	75T
41G	47G	74G
20T_20C	47C	72C
39T	46T	73T
38T	46T	73T
39T	46T	70T
39G	46G	71G
38T	47T	71T
38T	46T	72T
38C	47C	71C
38C	47C	71C
39G	46G	71G
38C	46C	68C
40T	45T	68T
40C	48C	70C
40T	46T	70T
39A	47A	72A
39A	46A	69A
41A	46A	67A
41A	46A	68A
41A	44A	66A
39T	46T	69T
40A	47A	68A
41A	47A	67A
41A	45A	67A
40A	45A	68A
40T	45T	66T

40A	45A	67A
40A	46A	66A
39A	46A	66A
37A	48A	66A
37T	47T	67T
22T_18G	47T	68T
40G	46G	66G
40G	46G	67G
41A	48A	67A
42A	48A	67A
40A	48A	68A
40C	46C	69C
39C	28T_18C	68C
39G	46G	69G
39T	47T	68T
39T	46T	69T
39T	46T	70T
39C	48C	69C
39T	50T	71T
22A_17G	49G	68G
39G	50G	67G
41G	50G	65G
41T	50T	66T
41C	52C	68C
41T	52T	66T
43G	52G	67G
43G	51G	66G
43G	53G	68G
42G	53G	70G
44G	53G	68G
44A	52A	70A
43A	51A	68A
42A	49A	68A
22T_20C	24T_23C	35T_34C
43G	48G	68G
43C	48C	70C
43T	48T	70T
47G	49G	70G
45T	49T	70T
47C	48C	71C
47C	48C	71C
46A	48A	70A
47G	49G	72G

47G	49G	73G
46A	49A	71A
47C	49C	71C
47C	49C	72C
47T	49T	71T
46G	49G	72G
46A	50A	72A
46A	50A	73A
46A	51A	71A
46C	52C	71C
46A	53A	73A
46G	52G	75G
45C	52C	75C
46C	53C	76C
46C	51C	74C
45T	52T	72T
45C	50C	69C
45T	49T	69T
45C	47C	69C
44C	85C	40A_147C
44C	28C	28C
43C	29C	25C
43A	29A	23A
45C	48C	69C
45C	48C	69C
45C	48C	68C
45A	45A	48A
46C	46C	52C
48C	46C	52C
47C	46C	51C
47C	46C	53C
22T_25C	47C	48C
46C	48C	51C
47A	50A	51A
46A	50A	54A
46A	51A	56A
45A	52A	56A
21T_24C	25T_27C	56T
44A	54A	59A
42G	54G	60G
41G	54G	60G
42G	54G	62G
42A	55A	65A

42A	55A	67A
43A	55A	70A
43T	55T	70T
43C	55C	70C
43A	55A	70A
43T	57T	74T
43G	58G	73G
43C	59C	71C
42C	58C	75C
42C	59C	77C
42T	57T	75T
42A	61A	73A
41T	61T	73T
43A	61A	72A
44A	61A	75A
44C	61C	75C
44A	60A	73A
46C	62C	74C
44G	61G	70G
46C	61C	72C
46A	64A	73A
46A	28A_34T	40A_32T
45T	62T	72T
46C	63C	71C
22T_23C	64C	70C
43T	64T	70T
43C	64C	73C
44A	64A	73A
44G	63G	73G
45G	62G	73G
45G	63G	71G
47G	63G	71G
47C	64C	72C
47A	63A	73A
46T	60T	73T
46G	61G	73G
46A	61A	73A
49G	60G	73G
48A	59A	71A
46T	57T	72T
46G	57G	71G
46G	56G	69G
46T	57T	66T

44C	57C	65C
45T	59T	64T
47T	59T	65T
47T	57T	64T
49T	57T	63T
51G	57G	65G
51A	57A	62A
53A	58A	64A
53G	57G	64G
53T	55T	63T
52T	55T	64T
52G	56G	65G
51T	54T	67T
51A	56A	67A
51A	56A	67A
51A	54A	70A
50G	53G	68G
50A	54A	68A
52A	54A	67A
51A	55A	66A
53A	55A	70A
54G	56G	68G
54A	56A	70A
55A	55A	68A
55T	55T	67T
53A	55A	66A
53G	56G	64G
52G	58G	62G
47A	58G	63G
48T	57T	62T
49T	58T	64T
49T	58T	63T
28A_22C	58A	60A
50A	58A	59A
50G	58G	60G
50G	59G	59G
48G	59G	59G
49T	59T	59T
49G	59G	59G
49C	58C	61C
22A_29G	60G	60G
51A	61A	58A
51G	60G	60G

51G	60G	61G
51T	59T	62T
51G	59G	62G
51G	60G	64G
50G	60G	64G
50T	59T	63T
50G	61G	63G
50A	63A	64A
51A	63A	65A
52C	65C	64C
52C	67C	61C
52T	68T	61T
53G	67G	60G
53G	67G	60G
51G	67G	59G
51A	66A	58A
51C	66C	59C
51C	67C	59C
50T	67T	59T
50C	68C	60C
52C	67C	60C
53T	67T	61T
52G	67G	61G
52C	66C	61C
52A	70A	62A
53T	69T	61T
54C	69C	63C
31T_23C	68C	64C
23T_31C	69T	25T_39C
55G	67G	64G
55T	67T	66T
55G	65G	69G
55G	67G	66G
55G	68G	66G
55C	68C	64C
53T	68T	66T
52G	70G	66G
53C	69C	65C
52A	69A	65A
52C	68C	63C
52T	65T	63T
52T	66T	65T
54T	66T	63T

54T	67T	63T
54T	67T	67T
54A	65A	68A
54C	64C	70C
23T_30C	64C	66C
52C	63C	69C
52A	64A	64A
52C	66C	67C
51C	64C	68C
52C	65C	68C
49A	66A	70A
50T	63T	70T
50T	63T	66T
50T	62T	65T
49C	64C	63C
49A	63A	63A
49A	62A	62A
49A	62A	62A
48G	63G	60G
48C	59G	31G_28C
47G	58G	57G
44G	59G	60G
42G	60G	62G
43A	59A	60A
43C	60C	61C
44A	60A	61A
44A	60A	59A
45A	61A	59A
43T	63T	58T
44A	63A	60A
44T	65T	58T
44G	66G	58G
46G	66G	59G
45G	65G	60G
45G	66G	58G
46C	64C	58C
45C	65C	60C
44C	65C	60C
44G	64G	61G
44C	65C	60C
44T	65T	60T
41G	64G	62G
44C	64C	63C

27T_18C	63C	63C
46T	63T	64T
46A	63A	63A
44G	62G	62G
45A	62A	62A
45C	62C	60C
45T	62T	60T
44G	62G	59G
44G	61G	58G
18G_24C	60G	58G
42C	59C	56C
39T	59T	55T
38T	57T	54T
38G	56G	54G
37C	56C	51C
37T	55T	48T
37C	55C	49C
38C	54C	49C
38T	53T	49T
38G	60A_40T_93G_60C	46G
42A_29T_28G_78C	48A_53T_32G_74C	72A_48T_48G_118C
39G	57G	68G
40C	57C	67C
40C	57C	68C
40G	47G	50G
40C	47C	51C
41C	47C	52C
40T	48T	52T
41G	48G	52G
42G	47G	53G
42C	49C	54C
41A	49A	58A
41G	50G	61G
41G	50G	61G
41C	50C	61C
41A	50A	61A
43G	49G	60G
42A	50A	60A
42A	49A	61A
43A	50A	60A
44T	49T	60T
44T	52T	60T
44G	52G	62G

43A	51A	62A
42A	51A	64A
41G	50G	63G
1T_17G_22C	50G	61G
41G	50G	62G
41G	46G	64G
41C	22T_24C	37T_28C
40G	46G	64G
40A	46A	67A
40T	45T	66T
39G	45G	67G
38G	45G	68G
38A	45A	65A
38C	46C	67C
38T	45T	67T
37T	45T	65T
38C	46C	65C
38C	45C	67C
38T	45T	67T
39A	44A	68A
39C	45C	69C
39C	47C	67C
39C	48C	66C
40T	48T	66T
41C	47C	68C
41T	47T	67T
41G	47G	68G
41A	45A	67A
40C	48C	67C
40C	47C	69C
40C	47C	70C
24T_16G	47T	41T_29G
41A	47A	70A
40A	47A	69A
41G	46G	69G
41C	46C	69C
41T	46T	65T
41C	46C	65C
40C	45C	65C
40A	46A	66A
42A	46A	66A
42A	45A	66A
41G	46G	66G

42C	46C	67C
42A	45A	67A
42T	44T	69T
42G	45G	67G
43G	45G	68G
43G	45G	64G
43T	45T	66T
43C	45C	65C
43C	42C	63C
43A	41A	63A
42C	41C	59C
42C	39C	59C
41C	38C	59C
41A	38A	58A
43G	18A_20G	25A_33G
43C	38C	56C
41C	38C	53C
42A	36A	54A
42A	37A	54A
41G	36G	56G
40T	36T	56T
64A_16T_72G_16C	116A_29T_94G_29C	40A_10T_76G_10C
19A	1C	32A
19C	null	31C
19T	null	33T
18A	null	33A
18G	null	33G
18A	1A	34A
18A	1A	36A
18G	1T_1G	36G
37G	36G	62G
36C	36C	62C
36T	38T	61T
35G	40G	62G
36G	40G	62G
36C	39C	62C
38A	41A	62A
38C	41C	61C
39A	41A	60A
40A	41A	27A_30G
39A	41A	59A
40C	42C	60C
39G	24A_18G	61G

40T	42T	63T
39T	42T	64T
40G	42G	64G
41A	43A	64A
39G	44G	64G
39A	44A	65A
39C	44C	66C
39A	45G	67G
39C	46C	66C
39C	47C	69C
40G	46G	66G
40A	47A	66A
40G	50G	68G
40G	51G	67G
39C	50C	66C
38T	49T	65T
38G	49G	68G
37C	49C	67C
37T	49T	67T
35G	50G	67G
36T	51T	66T
36A	50A	66A
34G	51G	64G
35T	50T	29T_33C
35A	51A	62A
35C	51C	63C
34A	52G	30A_30G
34A	52A	59A
34G	53G	59G
32A	53A	57A
81T_60C	110T_74C	94T_64C
20C	35C	29C
21T	39T	51T
23C	40C	49C
23T	40T	53T
22C	40C	50C
20T	41T	50T
20C	41C	49C
20T	61T	69T
20C	61C	70C
33T	62T	71T
33C	61C	73C
33T	61T	72T

32C	61C	71C
33T	64T	69T
33C	65C	68C
34T	65T	67T
34C	63C	62C
34T	64T	63T
34C	64C	62C
33T	64T	64T
33C	64C	61C
35T	65T	61T
30A_34T_42C	67C	33A_28C
36T	67T	65T
30G	67G	64G
30T	67T	64T
31T	67T	64T
32G	67G	65G
33C	66C	65C
33A	65A	66A
33T	66T	66T
34T	65T	65T
35T	66T	67T
35T	65T	67T
35C	66C	66C
35C	66C	68C
36A	65A	68A
36C	66C	68C
36A	66A	67A
36C	67C	67C
36A	66A	66A
36A	64A	65A
36T	64T	66T
38C	64C	67C
38C	63C	66C
38T	63T	68T
16T_39C	63C	68C
41T	64T	67T
39T	61T	66T
39T	63T	65T
39A	63A	63A
39A	62A	66A
39G	62G	65G
38A	62A	61A
38G	62G	61G

38G	61G	61G
20T_18C	1A_36T_22C	59C
37G	58G	59G
37A	57A	55A
37C	57C	54C
36A	59A	54A
37A	59A	54A
39C	58C	56C
39G	57G	53G
38A	57A	52A
37A	58A	52A
36T	58T	51T
37G	59G	53G
36C	60C	51C
36A	59A	53A
35A	60A	54A
35C	60C	56C
35A	63A	55A
35A	64A	51A
35T	63T	52T
36T	63T	50T
35G	64G	49G
35C	64C	48C
35T	65T	46T
34G	63G	45G
34T	63T	42T
35G	63G	43G
36T	64T	44T
36G	64G	47G
36T	64T	46T
36T	63T	47T
38T	65T	49T
38A	64A	50A
38G	65G	49G
38G	66G	49G
38G	66G	48G
37A	66A	48A
37G	67G	51G
35G	67G	49G
33G	67G	49G
33T	65T	50T
35G	67G	51G
35T	68T	50T

35G	70G	50G
35T	70T	51T
35G	70G	51G
36T	68T	51T
36G	69G	49G
36T	69T	50T
36G	70G	50G
35T	70T	48T
36C	71C	48C
36T	70T	49T
35A	69A	49A
36T	68T	49T
36G	68G	49G
36T	68T	48T
36G	69G	51G
36T	71T	50T
36G	72G	49G
36G	72G	49G
36A	72A	50A
36A	70A	55A
36C	71C	56C
36G	72G	56G
35G	72G	57G
34T	71T	56T
35G	70G	55G
36C	71C	54C
38A	71A	53A
38A	73A	54A
38T	73T	57T
39T	74T	57T
39A	74A	57A
39T	75T	58T
42T	74T	58T
41T	74T	57T
41G	74G	56G
39G	70G	57G
39T	72T	57T
37G	73G	57G
38A	74A	57A
39A	74A	55A
40G	75G	56G
40A	76A	54A
39T	76T	53T

38T	75T	54T
37G	75G	55G
37G	74G	55G
37A	75A	55A
38G	74G	55G
37G	75G	58G
37T	77T	57T
37G	77G	58G
39T	76T	57T
38C	77C	58C
38A	74A	59A
40C	75C	58C
40C	76C	55C
41C	75C	56C
41T	75T	55T
41G	75G	54G
41A	75A	51A
41C	75C	53C
41A	74A	54A
41A	73A	54A
42T	76T	55T
42T	76T	52T
42G	74G	50G
41A	75A	50A
40G	74G	52G
38A	73A	55A
40A	72A	56A
40T	72T	57T
39G	72G	55G
40A	72A	55A
40A	73A	57A
39G	75G	57G
41C	75C	56C
42A	76A	56A
42C	76C	56C
42G	76G	56G
42G	76G	56G
42G	76G	55G
42A	76A	56A
43G	74G	56G
43G	74G	57G
41A	73A	54A
41C	75C	55C

41C	75C	55C
41A	75A	54A
41G	75G	55G
42A	73A	50A
42A	73A	49A
42A	73A	49A
43T	73T	48T
43G	73G	49G
44A	70A	52A
44C	69C	52C
44A	71A	53A
45A	71A	55A
45T	69T	52T
45C	69C	54C
46A	70A	55A
46A	70A	56A
47A	67A	57A
46A	67A	57A
44G	69G	57G
45C	68C	56C
45T	68T	56T
45T	69T	57T
43C	69C	57C
43C	70C	56C
43A	69A	55A
43C	69C	55C
43C	71C	55C
44A	70A	52A
44G	70G	53G
44G	69G	52G
44G	70G	50G
44T	70T	52T
43A	69A	49A
43A	68A	49A
44A	66A	49A
44G	66G	50G
44T	66T	51T
42G	67G	49G
42T	67T	49T
43T	67T	50T
43A	67A	51A
43A	66A	52A
42T	65T	53T

41A	64A	53A
39A	63A	52A
40C	62C	50C
41A	62A	51A
41A	60A	50A
40C	61C	50C
40C	58C	50C
40T	57T	50T
43A	56A	47A
44A	55A	44A
45C	55C	43C
45T	55T	42T
45G	54G	42G
45C	52C	45C
45T	53T	43T
43T	53T	43T
43T	51T	42T
41C	51C	42C
41A	26A_22G	21A_20G
18G_23C	47G	20G_21C
40A	48A	41A
41T	47T	42T
41A	48A	41A
38C	48C	41C
38C	49C	41C
38A	50A	41A
38T	52T	40T
39G	50G	41G
39A	49A	40A
39T	48T	38T
39G	48G	38G
39A	47A	38A
39T	45T	38T
39G	47G	37G
38A	46A	35A
39T	45T	32T
39G	46G	32G
39A	45A	31A
37T	46T	29T
38G	44G	29G
37G	43G	27G
37C	42C	26C
35C	43C	24C

36T	41T	23T
36G	41G	22G
34C	40C	21C
18T_15C	39C	22C
33C	19A_20C	22C
33A	39A	18A_16C
33C	78C	41C
33C	18A_43C	9C
14A_34C	6C	9C
14A_5C	2C	5A_2C
33C	23C	7C
33C	36C	15C
34C	38C	14C
32C	38C	14C
32C	38C	14C
32C	38C	15C
32A	38A	15A
32C	39C	17C
33C	17T_22C	18C
31G	39G	18G
31G	39G	20G
31G	39G	19G_6C
31C	39C	19C
31C	39C	18C
31C	17T_22C	19C
31C	39C	18C
30C	40C	17C
29T	41T	14T
31T	41T	15T
31G	40G	14G
31C	40C	16C
31T	40T	18T
30C	41C	21C
30C	44C	21C
30C	44C	24C
18T_13C	44C	25C
30A	44A	27A
31G	44G	27G
29A	43A	29A
31A	44A	30A
30A	44A	30A
29G	43G	30G
29C	45C	29C

30T	45T	30T
30A	46A	30A
30A	46A	30A
30C	47C	31C
29A	47A	31A
30G	48G	31G
29A	45A	31A
27A	47A	33A
28G	46G	34G
27A	44A	34A
27C	45C	35C
28A	45A	35A
28A	45A	37A
29C	20T_25C	38C
29C	18T_26C	38C
29T	46T	39T
30C	45C	39C
30G	45G	40G
30C	45C	41C
30C	46C	42C
29T	44T	43T
29T	46T	44T
29C	46C	25T_22C
29T	45T	48T
29C	46C	48C
29A	45G	48A
29G	25A_18G	48G
28T	45T	49T
28T	45T	53T
28C	45C	54C
28A	44A	55A
28G	45G	56G
27C	44C	57C
27C	42C	57C
27A	43A	60A
27C	44C	61C
26T	43T	61T
26C	45C	62C
26A	45A	61A
26T	45T	63T
26A	45A	64A
26A	45A	65A
27T	44T	64T

25A	44A	62A
23T	44T	60T
21T	44T	62T
21C	46C	61C
21T	46T	60T
22G	46G	60G
23G	46G	60G
22C	46C	60C
21T	47T	60T
11G_10C	28G_19C	27G_34C
21T	49T	61T
21C	49C	61C
22T	48T	62T
21C	48C	62C
21T	48T	62T
21C	47C	60C
21C	47C	57C
22G	47G	58G
22C	46C	56C
21A	44A	56A
21T	44T	54T
21G	44G	53G
21G	43G	51G
21C	43C	51C
21G	42G	27A_24G
12A_8T_1C	25A_17T	25A_27T
21G	41G	54G
21C	41C	57C
21A	41A	59A
22T	41T	59T
22C	40C	59C
23C	41C	59C
25A	41A	60A
25G	42G	60G
25C	41C	59C
23T	39T	58T
23G	39G	58G
23C	39C	58C
23A	41A	58A
22G	41G	55G
22G	42G	56G
22G	42G	57G
21C	42C	56C

21C	41C	57C
14A_8G	25A_17G	57G
22G	44G	60G
22T	42T	60T
23T	42T	60T
24T	42T	59T
24T	42T	58T
24A	43A	58A
24A	42A	58A
23G	45G	58G
23G	44G	57G
23A	44A	59A
23A	44A	55A
23G	44G	54G
23A	46A	53A
23T	46T	54T
24C	45C	53C
24T	44T	54T
24A	44A	51A
23A	44A	50A
22A	44A	52A
9A_14G	44A	52A
23T	44T	52T
23T	44T	51T
25A	41A	51A
24A	41A	51A
23A	41A	52A
23T	40T	53T
22T	42T	54T
25G	43G	52G
24G	42G	52G
24A	24A_18C	50A
24A	43A	55A
24G	43G	55G
24C	42C	53C
24A	41A	53A
25G	43G	53G
24C	44C	53C
24T	44T	24A_53T
23A	42A	52A
24A	43A	61A
24A	44A	62A
24A	44A	63A

24A	45A	66A
24A	46A	65A
24A	44A	65A
25A	44A	65A
24T	20A_25T	66T
24G	44G	66G
23G	43G	67G
23A	43A	67A
24A	43A	67A
24A	43A	66A
24G	42G	63G
23T	42T	61T
24A	41A	60A
24T	41T	59T
24T	41T	58T
25C	41C	60C
25T	41T	62T
25A	40A	63A
26A	40A	64A
26T	40T	65T
27T	40T	64T
27A	40A	64A
27A	40A	65A
27C	41C	63C
27T	41T	63T
27G	40G	66G
27C	40C	66C
27C	41C	66C
31C	40C	65C
31C	39C	66C
31T	39T	65T
32G	39G	66G
32G	39G	65G
31G	40G	64G
31G	39G	66G
31C	40C	67C
31T	40T	67T
31C	38C	66C
31G	38G	67G
30T	38T	68T
31C	39C	68C
31A	39A	68A
31G	38A	69G

32T	38T	69T
33G	15T_21G	70G
33T	36T	71T
33C	37C	71C
18A_15G	15A_22G	37A_34G
34T	38T	71T
35C	40C	72C
36A	40A	73A
37T	41T	73T
38T	41T	73T
38T	41T	72T
38A	41A	73A
38T	41T	75T
38T	41T	75T
39C	41C	75C
39C	41C	74C
39A	42A	74A
39C	43C	74C
40C	43C	75C
40A	45A	70A
39G	45G	69G
39A	45A	70A
38G	44G	71G
36G	44G	73G
36A	43A	70A
36G	43G	69G
36C	43C	69C
37C	43C	70C
37A	42G	70A
37G	41G	71G
37A	40G	69A
37C	43C	69C
38T	43T	68T
39G	43G	69G
38G	43G	71G
38T	42T	71T
38T	40T	72T
38C	42C	73C
37A	42A	74A
36A	42A	76A
37A	42A	75A
37A	42A	77A
37C	42C	78C

38T	40T	77T
39A	40A	76A
39G	40G	76G
40G	40G	76G
41T	40T	76T
41A	39A	75A
42G	39G	75G
41G	39G	76G
41A	40A	77A
41T	40T	73T
41T	41T	73T
41C	41C	74C
40C	41C	72C
40C	41C	71C
40C	40C	71C
38A	40A	70A
38A	40A	72A
38A	40A	71A
39T	41T	69T
39G	40G	70G
37T	39T	71T
38C	41C	71C
38T	41T	70T
38C	40C	69C
38C	40C	71C
38T	40T	70T
37G	40G	69G
37C	39C	69C
37A	37A	68A
37G	37G	69G
37T	38T	69T
37T	38T	69T
38G	36G	68G
38C	36C	70C
38T	34T	67T
38T	34T	67T
37T	34T	67T
37G	34G	68G
38C	33C	68C
38T	32T	68T
39A	33A	69A
39A	33A	71A
38A	32A	71A

38A	32A	71A
38C	32C	71C
38T	32T	72T
39G	32G	72G
39A	33A	72A
39A	33A	72A
39A	33A	73A
39C	34C	71C
38T	35T	71T
37T	34T	69T
37G	36G	69G
37G	37G	66G
37G	37G	68G
38G	37G	66G
39A	37A	68A
39T	37T	68T
39A	38A	68A
39G	39G	68G
39A	38A	66A
35G	37G	68G
35T	37T	67T
35G	37G	65G
35C	37C	66C
35T	37T	64T
35C	38C	66C
35C	38C	65C
36T	38T	65T
36A	39A	66A
36C	39C	65C
36A	40A	65A
36T	41T	64T
35A	40A	64A
35T	40T	65T
34G	39G	64G
34G	39G	63G
32A	40A	64A
33G	41G	64G
32G	40G	66G
32T	40T	66T
32T	39T	64T
31C	39C	66C
30T	39T	68T
28G	38G	41G_29C

27T	38T	68T
27C	38C	69C
28C	39C	71C
29A	39A	72A
29G	39G	71G
29C	39C	71C
29C	38C	70C
29C	37C	70C
29T	37T	68T
28T	22A_15T	68T
29G	35G	67G
29T	35T	36T_30G
29G	35G	66G
29G	35G	64G
29A	35A	29A_24G
29C	35C	52C
58A_15T	93A_29T	140A_44T
13A	4A	5A
13A	3A	5A
11C	1C	null
27A	29A	46A
38A_11T_11G	29A	48A
16A	29A	48A
16C	29C	48C
16G	29G	56G
27C	30C	57C
28A	30A	57A
29C	30C	58C
30A	31A	59A
30G	32G	59G
31G	32G	60G
31G	32G	60G
32G	33G	61G
32C	33C	62C
33T	36T	63T
32A	35A	63A
32T	35T	62T
31A	35A	61A
32A	34A	63A
31A	34A	63A
31A	35A	65A
30A	34A	67A
29C	35C	66C

29C	35C	65C
28T	38T	65T
28A	39A	63A
29T	37T	63T
29T	38T	61T
29C	38C	62C
29T	39T	66T
29T	39T	69T
29C	41C	69C
30T	41T	69T
30C	41C	68C
30G	15A_26G	36A_34G
31G	41G	70G
30T	40T	70T
30T	39T	68T
30A	41A	67A
29G	39G	67G
29A	39A	67A
29A	39A	67A
29A	38A	67A
29G	38G	67G
30C	38C	68C
30A	38A	68A
30G	37G	66G
30A	38A	67A
30C	37C	65C
29T	37T	64T
30A	38G	64G
30A	38A	64A
31C	38C	62C
32C	38C	62C
32C	38C	63C
32T	38T	64T
32G	37G	61G
32T	37T	57T
31C	36C	61C
31C	36C	61C
31A	38A	62A
31C	38C	62C
31A	36A	63A
31C	37C	65C
30A	36A	64A
31C	36C	64C

31A	35A	64A
30C	36C	65C
31C	36C	66C
17T_15C	35C	66C
33G	35G	64G
33G	34G	62G
32C	33C	62C
33C	33C	63C
32G	33G	62G
32C	33C	63C
32C	34C	64C
32T	33T	64T
32T	32T	65T
32T	32T	65T
33G	34G	63G
33C	34C	64C
33T	34T	64T
32A	34A	63A
34T	34T	62T
34G	34G	64G
35A	33A	67A
34A	34A	67A
34G	33G	65G
36A	33A	66A
37A	32A	65A
37T	31T	64T
37T	31T	65T
37T	30T	66T
37G	30G	67G
38G	30G	68G
39A	28A	67A
38A	28A	71A
39G	28G	70G
39A	28A	68A
38T	28T	64T
39G	28G	64G
39C	28C	64C
38T	28T	65T
40A	28A	65A
40T	28T	65T
40T	27T	66T
37C	27C	67C
39T	27T	66T

38T	120A_47T_120G_120C	67T
39G	7G	66G
39A	null	68A
39A	null	67A
39A	null	68A
39C	null	70C
40A	null	71A
40A	null	71A
41C	null	71C
42C	null	30T_41C
42G	null	71G
42C	null	71C
42C	null	69C
42A	null	68A
20T_22C	null	67C
42G	null	67G
44G	null	65G
43T	null	65T
44G	null	66G
43G	null	64G
43T	21T	65T
44G	21G	64G
44G	21G	63G
44T	36T	61T
44G	36G	34A_27G
43G	36G	60G
43T	36T	63T
43G	36G	63G
42G	37G	61G
42G	37G	59G
42G	37G	63G
42A	37A	64A
42T	37T	65T
44A	37A	65A
46A	38A	66A
46T	14A_24T	65T
45G	40G	63G
45G	40G	64G
46G	40G	64G
47G	41G	63G
50A	41A	63A
50C	41C	65C
51T	41T	65T

51G	41G	64G
54A	41A	63A
54A	42A	64A
53G	42G	64G
53C	42T	61C
53C	42C	60C
53C	42C	60C
53C	42C	62C
53T	42T	62T
54A	43A	62A
54A	44A	60A
55A	44A	61A
54G	41G	61G
55A	40A	62A
55T	40T	62T
55A	40A	62A
55T	40T	62T
55C	39T	62C
53G	39G	61G
52T	37T	61T
52T	38T	61T
51T	38T	59T
51G	38T	50G
50T	38T	49T
49C	38T	47C
52C	40C	20T_28C
52C	38C	52C
52A	38A	52A
52A	40A	52A
52G	41G	52G
52C	43C	52C
52T	43T	51T
52T	42T	51T
52G	43G	50G
53G	44G	51G
53C	43C	50C
54T	45T	49T
24G_29C	46G	49G
50A	46A	50A
51G	46G	52G
53A	46A	53A
53A	46A	52A
53G	46G	51G

53T	47T	52T
53A	48A	53A
54T	48T	53T
54T	49T	53T
55C	49C	53C
53G	46G	52G
54C	46C	52C
56T	46T	55T
56G	46G	55G
54T	46T	55T
54T	46T	52T
53A	46A	52A
53G	46G	52G
53C	45C	53C
52C	45C	49C
51A	45A	49A
52C	45C	48C
52C	46C	49C
52C	46C	48C
53A	46A	49A
52G	46G	49G
51A	44A	47A
51G	44G	48G
51T	44T	49T
51A	44A	48A
50G	42G	48G
50A	42A	49A
50C	20G_22C	49C
49T	42T	51T
49T	41T	50T
50C	40C	48C
50G	40G	51G
49G	39G	52G
50T	39T	50T
50C	39C	50C
49T	39T	49T
49A	39A	48A
49A	39A	48A
49A	39A	48A
48T	39T	49T
47G	39G	44G
46G	38G	45G
46G	37G	42G

45C	36C	41C
45A	36A	39A
44G	35G	39G
43G	35G	38G
44A	37A	38A
46T	37T	39T
45A	36A	40A
44T	34T	38T
43A	33A	38A
41A	32A	39A
41G	34G	37G
42T	33T	37T
40T	33T	37T
40A	33A	37A
39G	32G	37G
37T	32T	37T
37T	31T	37T
37A	32A	37A
38G	31G	36G
39T	31T	36T
39T	31T	36T
39A	31A	36A
39G	31G	35G
39T	31T	33T
37T	31T	32T
38A	29A	32A
38G	30G	32G
39T	30T	31T
39T	30T	29T
38A	30A	29A
37G	18G_11C	29G
34T	29T	29T
34T	30T	30T
33A	30A	31A
31G	31G	30G
31T	31T	29T
31T	30T	29T
31A	31A	29A
31G	30G	29G
31T	29T	29T
32T	30T	31T
32A	30A	31A
32G	29G	30G

32T	28T	30T
32T	28T	30T
31A	27A	29A
31G	19G_9C	28G
30T	28T	28T
30T	28T	28T
30A	27A	26A
30G	27G	23G
30T	26T	22T
30T	26T	23T
30A	26A	22A
28G	25G	22G
29T	25T	20T
30T	26T	20T
30A	26A	20A
32G	26G	18G
32T	24T	16T
32T	25T	19T
33A	25A	18A
33G	24G	15G
33T	23T	15T
33T	21T	14T
33A	22A	13A
23G	20G	12G
22T	20T	12T
22T	21T	12T
22A	20A	12A
22G	19G	12G
23T	19T	12T
25T	20T	12T
27A	20A	12A
26G	20G	12G
26T	20T	11T
26T	20T	11T
26A	19A	11A
26G	18G	11G
24T	18T	10T
24T	18T	10T
24A	18A	10A
24G	17G	10G
25T	17T	10T
25T	17T	10T
25A	17A	10A

25G	17G	10G
24T	17T	11T
25T	17T	12T
25A	17A	12A
25G	17G	12G
25T	17T	12T
25T	17T	12T
27A	17A	12A
26G	19G	7G
28A	19A	7A
28T	20T	7T
28A	20A	7A
25G	20G	7G
28A	21A	7A
29T	21T	7T
29A	22A	7A
30G	22G	6G
30A	22A	6A
31T	22T	6T
31A	23A	6A
32G	23G	6G
32A	23A	6A
32T	23T	6T
32A	23A	6A
32G	23G	5G
32A	23A	5A
32T	23T	5T
32A	23A	4A
32G	22G	4G
32A	21A	4A
33T	21T	4T
32A	21A	4A
32G	21G	4G
32A	21A	4A
32T	21T	4T
32A	20A	3A
31G	21G	3G
31A	21A	3A
31T	21T	3T
31A	21A	3A
31G	19G	3G
31A	20A	3A
29T	20T	3T

29A	20A	3A
28G	19G	2G
27A	20A	2A
26T	20T	2T
26A	20A	2A
26G	20G	2G
26A	20A	2A
26T	20T	2T
25A	20A	2A
24G	20G	2G
23A	19A	2A
21T	20T	2T
22A	20A	2A
21G	20G	2G
21A	20A	2A
21T	20T	2T
21A	20A	2A
21G	20G	2G
21A	18A	2A
21T	18T	2T
21A	18A	2A
21G	16G	1G
21A	16A	1A
21T	15T	2T
21A	15A	2A
21G	14G	2G
21A	14A	2A
21T	14T	2T
21A	13A	2A
21G	13G	1G
21A	13A	1A
21T	13T	1T
21A	13A	1A
21G	13G	1G
21A	13A	1A
21T	12T	1T
21A	12A	1A
21G	12G	1G
21A	12A	1A
21T	12T	1T
21A	12A	1A
21G	12G	1G
21A	12A	1A

21T	12T	1T
21A	12A	1A
21G	12G	1G
21A	11A	1A
20T	11T	1T
20A	11A	1A
18G	11G	2G
18A	11A	2A
18T	11T	3T
17A	11A	3A
17G	10G	3G
17A	10A	3A
17T	9T	3T
17A	9A	3A
17G	8G	3G
17A	8A	3A
17T	8T	3T
17A	8A	3A
17G	8G	3G
16A	8A	3A
15T	7T	3T
14A	7A	3A
13G	7G	4G
14A	7A	5A
14T	7T	5T
14A	7A	5A
13G	7G	5G
14A	7A	5A
14T	7T	5T
14A	8A	5A
14G	7G	5G
13A	8A	5A
13T	8T	5T
13A	8A	5A
12G	7G	5G
14A	9A	10A
14T	9T	10T
15A	9A	10A
15A	9A	10A
15A	9A	10A
14T	9T	11T
12A	9A	11A
10A	7A	12A

10A	7A	13A
11T	6T	13T
11A	6A	13A
11A	5A	13A
12A	5A	13A
12T	5T	13T
13A	4A	13A
13A	3A	13A
14A	4A	14A
14T	4T	15T
15A	5A	17A
15A	5A	17A
15A	4A	17A
14T	5T	18T
14A	5A	20A
14A	5A	21A
15A	6A	21A
15T	6T	21T
15A	6A	21A
15A	6A	22A
15A	7A	22A
17T	7T	22T
17A	7A	25A
17A	9A	26A
17A	10A	28A
17T	10T	28T
17A	10A	32A
17A	10A	33A
18A	11A	33A
18T	11T	35T
18A	11A	35A
20G	12G	35G
21G	13G	35G
21G	13G	31G
22T	12T	34T
22A	14A	35A
21T	16T	34T
23G	16G	35G
23A	16A	36A
24T	16T	36T
26C	17C	39C
26C	17C	39C
26A	17A	40A

26G	17G	41G
26A	16A	42A
27T	17T	42T
26C	18C	44C
27A	19A	43A
27A	19A	43A
27G	19G	43G
27C	19C	43C
27C	19C	42C
27A	19A	45A
27G	21G	45G
28G	21G	44G
28G	21G	45G
28A	21A	45A
29A	22A	45A
29C	22C	45C
29A	22A	47A
30G	22G	52G
30A	22A	53A
31T	22T	53T
31G	22G	53G
31T	23T	53T
32T	23T	55T
32G	11G_12C	55G
32T	23T	54T
34C	23C	55C
34T	23T	57T
34T	24T	58T
34G	24G	58G
33G	24G	59G
34A	24A	59A
34A	24A	59A
34G	24G	59G
34A	24A	59A
34G	25G	60G
34A	25A	59A
34C	25C	58C
16A_19G	25G	56G
35C	25C	56C
35A	25A	60A
36G	25G	59G
35A	25A	59A
35C	25C	59C

36A	25A	60A
35A	25A	60A
36G	26G	60G
36C	25C	60C
36A	25A	61A
36A	25A	61A
38C	25C	60C
38T	25T	60T
38G	25G	57G
37T	25T	58T
39G	25G	56G
39G	25G	56G
38G	25G	56G
37A	25A	55A
38G	25G	55G
39G	25G	56G
40G	25G	57G
39A	25A	59A
38G	25G	57G
39A	25A	58A
39G	24G	60G
43G	25G	59G
43A	25A	59A
44G	26G	58G
43G	26G	56G
21A_22G	13A_13G	58A
41T	25T	57T
43G	28G	56G
44T	30T	54T
45T	30T	55T
45C	31C	55C
46C	32C	55C
46T	32T	55T
46T	32T	55T
47G	31G	56G
46C	32C	56C
46A	32A	56A
46A	32A	55A
45A	32A	57A
44C	32C	57C
44T	32T	57T
43A	32A	58A
44C	32C	58C

44G	33G	56G
44T	32T	52T
44C	31C	52C
44A	32A	52A
44T	31T	52T
44G	31G	51G
45C	31C	50C
44C	31C	50C
44T	30T	50T
44C	31C	50C
44G	31G	48G
43G	31G	46G
41G	30G	46G
42C	31C	46C
41A	28A	47A
41G	28G	47G
42A	29A	49A
42C	29C	50C
42C	30C	49C
42C	29C	49C
43A	30A	50A
43T	29T	50T
41C	29C	50C
40G	28G	49G
40C	30C	51C
40A	30A	49A
40G	30G	47G
39T	28T	48T
37G	28G	48G
37G	29G	49G
37G	29G	49G
35G	28G	48G
35C	28C	48C
36A	27A	47A
36G	29G	46G
37A	29A	49A
36G	28G	49G
36G	17A_11G	47G
36A	28A	48A
36G	28G	49G
36A	27A	50A
36A	28A	52A
36G	28G	54G

36A	29A	53A
36G	27G	52G
35G	27G	53G
35G	27G	51G
37G	26G	51G
35A	26A	50A
36A	26A	50A
36G	26G	49G
35C	26C	23G_26C
35C	25C	48C
35A	26A	48A
35A	26A	50A
36A	26A	50A
35G	27G	22A_27G
35A	27A	28A_22T
34A	27A	54A
33C	27C	53C
33C	27C	55C
33A	26A	54A
33G	26G	52G
33A	26A	54A
33A	26A	55A
33G	26G	54G
33C	27C	56C
32C	28C	56C
34C	26C	23T_33C
34C	27C	56C
34A	27A	58A
33C	27C	57C
33G	28G	57G
33T	28T	57T
32C	28C	58C
32C	28C	60C
32A	28A	62A
31G	28G	62G
31C	28C	63C
31A	27A	63A
32A	28A	63A
32C	16T_12G	25T_39C
33A	30G	40A_26G
31C	31C	65C
31A	31A	64A
31G	31G	64G

31C	31C	61C
30T	31T	61T
30G	31G	62G
30C	31C	64C
30A	31A	64A
30G	31G	64G
29C	31C	64C
29A	31A	61A
29G	31G	61G
29A	31A	62A
29G	31G	62G
29A	31A	62A
25G	30G	66G
25C	30C	66C
24T	29T	68T
24T	29T	69T
24T	29T	68T
24T	29T	69T
24C	27C	68C
22C	25C	66C
21C	25C	67C
21C	25C	66C
20C	24C	65C
20G	23G	66G
21G	25G	64G
21C	25C	63C
20T	25T	61T
20G	25G	62G
20C	25C	61C
20C	25C	59C
20A	25A	59A
20A	25A	58A
22A	25A	59A
22A	25A	60A
21A	23A	59A
21T	25T	61T
20A	25A	63A
20C	25C	65C
20T	26T	65T
20C	26C	63C
20T	26T	64T
19G	26G	63G
19G	26G	62G

19C	25C	62C
19A	25A	61A
19A	25A	60A
21G	26G	65G
21G	26G	65G
20A	26A	64A
20A	26A	64A
20T	27T	63T
19C	26C	62C
19A	26A	64A
18G	26G	64G
18G	26G	66G
17A	25A	66A
5A_12C	25A	65A
17G	25G	68G
17A	24A	68A
16G	23G	70G
16A	23A	69A
16G	24G	68G
16G	24G	68G
16A	24A	67A
16G	23G	69G
15A	23A	65A
15G	23G	67G
15G	23G	68G
14T_22G	23T	65T_26G
14G	23G	71G
13G	23G	71G
13G	23G	70G
13G	22G	71G
13G	22G	68G
13G	22G	69G
12T	19T	69T
13C	22C	69C
13A	22A	69A
13T	21T	67T
13G	21G	67G
13C	22C	68C
11T	22T	67T
11G	22G	64G
11A	22A	62A
11C	22C	62C
11C	22C	62C

10C	23C	63C
10A	23A	66A
11G	23G	66G
11A	22A	67A
11T	21T	65T
11C	21C	66C
11A	21A	66A
11A	22A	65A
11A	22A	65A
11A	22A	66A
11C	23C	67C
11C	23C	67C
11C	23C	67C
12G	22G	37A_30G
12A	22A	67A
12C	23C	66C
11G	21G	35A_30G
11A	21A	65A
11A	21A	63A
11A	21A	62A
11G	20G	64G
11G	20G	59G
12C	20C	60C
13A	20A	63A
13G	20G	61G
13G	20G	63G
14A	20A	64A
13T	20T	65T
14G	20G	65G
15A	19A	64A
15G	17G	63G
10T_5C	17C	61C
15G	17G	60G
16C	18C	62C
16T	18T	61T
16C	18C	62C
16A	19A	62A
18G	19G	60G
17C	19C	60C
17G	19G	59G
17G	20G	60G
17T	10T_10C	59T
17C	22C	61C

17C	22C	59C
17A	22A	58A
17C	22C	59C
17G	22G	60G
17T	22T	60T
17G	22G	59G
18A	24A	60A
18A	24A	61A
18G	24G	59G
18A	24A	57A
18G	24G	58G
18T	23T	59T
18C	24C	64C
18C	25C	63C
17C	26C	64C
17A	25A	63A
17G	26G	62G
17A	26A	62A
17G	26G	62G
17G	26G	62G
18G	27G	63G
18A	27A	65A
16A	27A	65A
16G	28G	64G
16A	29A	64A
16A	29A	63A
16A	27A	61A
16G	28G	60G
16A	29A	55A
17G	28G	59G
17G	28G	57G
17C	29C	57C
17A	30A	58A
17A	29A	58A
17G	29G	58G
18G	29G	57G
16G	29G	50A_57G
16A	28A	4A_1G
18G	29G	54G
13A_5C	30A	54A
18G	30G	54G
19A	28A	55A
19G	28G	53G

19A	28A	55A
19T	28T	57A
19G	28G	57G
19C	28C	59C
20T	11T_18C	59C
21A	29A	59A
21C	28C	59C
16G_5C	29G	62G
21G	29G	61G
21A	28A	58A
21A	28A	61A
21A	28A	61A
21C	28C	59C
21C	29C	59C
21C	30C	59C
21A	30A	57A
21G	29G	57G
21T	29T	58T
22G	29G	58G
17G	29G	58G
19G	30G	58G
20G	11T_19G	56G
21T	29T	59T
20G	30G	59G
21G	31G	58G
22C	30C	59C
22C	30C	62C
22C	31C	59C
22C	30C	57C
22A	30A	58A
22C	30C	58C
22G	30G	57G
22G	30G	56G
22C	30C	55C
21G	29G	52G
20G	18A_12G	52A
20A	30A	54A
20C	31G	56G
20G	30G	56G
20G	31G	59G
20A	31A	60A
20G	30G	57G
20G	30G	58G

19T	29T	58T
20G	29G	61G
20A	29A	61A
20G	29G	61G
20C	29C	64C
20T	29C	65T
20G	28G	65G
20G	18T_10G	62G
20G	29G	30A_33G
20C	18T_11C	64C
20C	33C	65C
20A	34A	66A
20G	34T	67T
21T	34T	67T
20C	34C	67C
20C	34C	64C
20C	34C	65C
20C	34C	65C
21A	34A	65A
21A	34A	65A
21A	35A	65A
21G	35G	64G
21A	35A	66A
21G	35G	69G
21G	36G	71G
19T	36T	70T
21G	36G	72G
21G	36G	72G
19A	37A	72A
21G	37G	74G
21T	38T	73T
21G	38G	74G
21G	39G	75G
21A	37A	75A
20T	37T	74T
20G	40G	74G
20T	40T	74T
20C	40C	75C
20G	41G	76G
20C	42C	77C
19A	40A	77A
20G	41G	77G
20C	41C	77C

20C	41C	80C
20C	42C	79C
20C	41C	79C
20A	41A	77A
20T	40T	76T
20A	39A	77A
21G	40G	74G
21A	39A	76A
22A	39A	78A
23G	39G	77G
23G	39G	76G
22G	38G	76G
23A	38A	77A
23G	39G	78G
23A	38A	78A
24T	37T	82T
25G	37G	81G
25C	37C	82C
26C	37C	84C
27C	36C	82C
26T	38T	81T
26C	38C	79C
26A	37A	79A
26C	36C	76C
26T	36T	74T
26C	36C	72C
25T	36T	71T
25G	36G	70G
25G	37G	67G
22A	36A	67A
23C	36C	67C
23A	37A	64A
22G	37G	63G
22A	37A	61A
22G	37G	61G
22C	38C	60C
22A	38A	59A
22A	38A	58A
21G	38G	57G
20G	38G	56G
20C	40C	56C
20C	1A_23T_16C	56C
20G	40G	56G

20T	40T	56T
20G	41G	56G
20G	41G	55G
20G	41G	55G
20G	40G	54G
20C	39C	54C
20T	40T	52T
1A	40C	2A
null	41T	null
null	41T	null
null	41C	null
null	41T	null
null	40G	null
null	39C	null
null	42C	null
null	40A	null
null	41C	null
null	41C	null
null	42C	null
null	42C	null
null	41T	null
null	42G	null
null	41G	null
null	40G	null
null	40C	null
null	40A	null
null	40A	null
null	39G	null
null	39C	null
null	14A_26G	null
null	40G	null
null	40G	null
null	40G	null
null	39G	null
null	39A	null
null	39T	null
null	39C	null
null	40C	null
null	40T	null
null	41C	null
null	42A	null
null	44G	null
null	41C	null

null	43G	null
null	24A_19G	null
null	46G	null
null	47G	null
null	45A	null
null	46A	null
null	47C	null
null	47C	null
null	47C	null
null	47G	null
null	47T	null
null	46C	null
null	45G	null
null	46A	null
null	46G	null
null	45A	null
null	46C	null
null	46A	null
null	46G	null
null	47A	null
null	47C	null
null	48A	null
null	47C	null
null	47A	null
null	47C	null
null	47A	null
null	43A	null
null	44G	null
null	44G	null
null	44G	null
null	44G	null
null	44C	null
null	43G	null
null	43A	null
null	44T	null
null	43G	null
null	43G	null
null	43G	null
null	43A	null
null	43A	null
null	42A	null
null	41A	null
null	43C	null

null	18A_25G	null
null	43C	null
null	44T	null
null	43C	null
null	42T	null
null	43C	null
null	42C	null
null	43A	null
null	42C	null
null	44A	null
null	44T	null
null	42G	null
null	42C	null
null	43T	null
null	43C	null
null	43A	null
null	43G	null
null	44A	null
null	44A	null
null	43A	null
null	43T	null
null	42A	null
null	41G	null
null	40T	null
null	40G	null
null	42T	null
null	41C	null
null	41C	null
null	41C	null
null	41C	null
null	41T	null
null	39T	null
null	38C	null
null	39G	null
null	43T	null
null	41T	null
null	42A	null
null	42T	null
null	43T	null
null	45G	null
null	45C	null
null	46T	null
null	45G	null

null	45A	null
null	45A	null
null	45T	null
null	44G	null
null	44C	null
null	43C	null
null	43A	null
null	44C	null
null	44G	null
null	44G	null
null	44A	null
null	44G	null
null	44C	null
null	44C	null
null	22A_23C	null
null	52A	null
null	52A	null
null	53A	null
null	55A	null
null	55G	null
null	55A	null
null	54G	null
null	57G	null
null	57T	null
null	22A_33G	2G
null	55C	3C
21T	56T	32T
21A	58A	34A
21C	58C	36C
21G	58G	39G
23A	57A	41A
23A	56A	42A
23A	54A	42A
23A	53A	43A
23T	50T	45T
23G	51G	47G
23T	49T	48T
23T	48T	50T
23C	47C	52C
23C	47C	54C
24T	47T	56T
24G	47G	58G
24G	21G_26C	61G

26A	47A	61A
26T	47T	62T
26T	47T	63T
26C	47C	63C
26A	47A	66A
26A	48A	29A_38G
27C	48C	67C
27C	47C	67C
27A	26A_21G	68A
27A	46A	69A
28C	45C	70C
28C	45C	70C
28T	44T	71T
28T	43T	72T
28G	43G	71G
27C	43C	71C
27A	43A	71A
26A	42A	68A
26A	43A	69A
25C	42C	67C
25A	42A	67A
25G	41G	66G
25A	40A	64A
25C	41C	64C
25C	41C	64C
25T	40T	63T
25C	40C	62C
25A	40A	62A
25T	40T	62T
25G	41G	61G
25T	41T	59T
25G	41G	60G
25T	42T	58T
26C	43C	58C
26T	43T	57T
26G	43G	56G
47T_7G_21C	1A_84T_14G_42C	54T
19C	29C	56C
21T	30T	55T
2G_4C	2G_6C	4G_2C
5T	8T	4T
5G	8G	4G
5T	8T	4T

5C	6C	1G_4C
12T	21T	5T
12C	21C	4C
12T	21T	4T
12G	21G	21G
12T	21T	21T
13G	21G	21G
13T	22T	21T
13G	22G	21G
13T	22T	21T
13G	22G	21G
6T_7C	18T	21T
7A_6G	14A_8G	21G
13T	22T	21T
13G	22G	21G
13T	22T	21T
13G	22G	21G
14T	22T	21T
14G	22G	21G
14T	21T	21T
14G	21G	21G
14A_21T_7G_14C	22T	21T
14G	24A_12T_22G_12C	21G
13T	21T	21T
13G	21G	21G
13T	21T	21T
13G	21G	20G
28A_13T_14G_28C	21T	20T
13G	21G	20G
13T	33A_20T_29G_26C	20T
13G	18G	19G
13T	19T	19T
13G	20G	19G
13T	20T	19T
13G	20G	18G
13T	19T	18T
13G	20G	17G
13T	20T	17T
13G	20G	17G
13T	20T	17T
13G	20G	15G
13T	20T	15T
13G	20G	14G

13T	20T	14T
13G	20G	14G
13T	20T	14T
13G	20G	13G
13T	20T	13T
13G	18G	13G
13T	18T	13T
13G	17G	12G
13T	17T	12T
12G	14G	12G
12T	14T	12T
12G	15G	12G
12T	15T	26T
12G	15G	30G
11T	13T	31T
11G	13G	31G
12T	13T	33T
12G	13G	35G
12T	13T	35T
12G	13G	35G
12T	13T	36T
12G	13G	36G
12T	13T	37T
11G	13G	40G
11T	13T	40T
11G	13G	40G
9T	13T	40T
9G	11G	41G
9T	11T	40T
9G	9G	41G
9T	9T	41T
9G	9G	36G
8T	9T	36T
9G	8G	38G
9T	9T	38T
11G	9G	43G
12T	9T	42T
13G	9G	44G
13T	9T	44T
13G	9G	46G
13T	9T	48T
13G	13G	49G
13T	2A_13T_5G_1C	50T

14G	13G	50G
14T	13T	50T
14G	13G	51G
14T	13T	53T
14G	13G	54G
13T	13T	55T
14G	13G	55G
14T	13T	55T
14G	13G	56G
15T	13T	57T
15G	13G	60G
15T	13T	61T
15G	13G	63G
16T	13T	63T
16G	13G	64G
16T	13T	64T
16G	13G	64G
16T	12T	64T
16G	11G	65G
16T	12T	65T
15G	12G	66G
15T	12T	66T
15G	12G	67G
15T	12T	67T
15G	12G	69G
15T	12T	70T
15G	12G	70G
15T	12T	72T
15G	11G	73G
15T	11T	74T
16G	11G	78G
16T	11T	80T
16G	11G	80G
16T	12T	81T
16G	12G	84G
16T	11T	85T
16G	11G	86G
16T	11T	86T
17G	11G	86G
17T	11T	56T
16C	9C	50C
16C	10C	49C
14G	3A_6G	38G

14T	6T_3C	41T
16G	9G	44G
16C	7C	45C
15A	7A	47A
15T	7T	47T
15C	7C	49C
15T	8T	49T
15C	9C	50C
15C	9C	51C
18T	9T	53T
18T	9T	55T
18C	9C	54C
18G	9G	56G
19G	9G	55G
19A	9A	54A
19C	8C	54C
19A	9A	55A
19A	9A	54A
19G	9G	54G
19C	9C	51C
19A	9A	51A
19A	9A	51A
19T	9T	50T
19T	10T	52T
19T	9T	52T
19A	10A	52A
18C	10C	52C
18A	10A	52A
18G	10G	50G
18C	10C	52C
18C	10C	53C
18G	11G	53G
18C	11C	53C
18C	11C	55C
18A	11A	57A
18G	11G	56G
18G	11G	56G
18A	10A	56A
17G	10G	56G
17C	10C	56C
17T	10T	56T
17T	8T	57T
16C	9C	56C

16C	9C	56C
16A	9A	56A
16T	9T	56T
16T	9T	56T
16T	8T	56T
16G	8G	58G
16C	8C	58C
10T_6C	8T	58T
16G	8G	56G
16G	8G	55G
16G	8G	56G
17G	8G	54G
17G	8G	54G
17C	8C	52C
17G	8G	49G
17A	8A	48A
17A	8A	49A
17A	9A	49A
17G	9G	46G
17T	8T	46T
19C	8C	45C
19A	9A	45A
18C	9C	46C
19T	9T	47T
18C	9C	47C
19G	9G	46G
19T	10T	45T
19A	11A	42A
18A	12A	42A
18T	12T	41T
17A	12A	39A
5A_18G_6C	12G	40G
16A	12A	38A
18A_27C	12C	29A_38C
17C	12A	31C
18C	13C	37C
18C	13C	36C
19C	13C	34C
19C	14C	35C
19C	16C	34C
18C	16C	34C
17C	16C	36C
17A	17A	34A

17C	18C	36C
17C	19C	36C
16T	19T	37T
16G	19G	38G
16C	19C	38C
17C	20C	40C
18A	21A	38A
18G	21G	39G
18A	21A	40A
18C	21C	40C
18C	21C	40C
18G	20G	40G
18C	21C	40C
18A	21A	37A
18G	21G	39G
19C	21C	37C
19C	21C	38C
20C	21C	36C
19C	21C	36C
19A	21A	34A
19C	21C	34C
19A	21A	35A
19C	21C	35C
19G	22G	37G
19C	21C	37C
19A	21A	37A
18C	23C	38C
18C	23C	39C
17T	24T	39T
18G	24G	41G
18C	24C	41C
18T	24T	41T
18T	23T	41T
18G	23G	41G
18G	23G	44G
18A	24A	47A
18T	24T	46T
18A	23A	47A
18T	24T	46T
18A	24A	49A
17C	23C	50C
16G	21G	50G
16G	24G	51G

16A	25A	51A
15G	26G	52G
16T	26T	51T
16T	24T	53T
18T	26T	53T
19A	26A	52A
18T	27T	53T
19T	27T	53T
20A	28A	55A
20A	29A	55A
20A	29A	55A
19T	28T	55T
20T	28T	56T
20A	30A	57A
21T	30T	56T
21G	33G	56G
20T	32T	56T
21T	32T	55T
21T	32T	55T
21T	31T	57T
22T	32T	58T
21T	32T	58T
21A	32A	57A
21A	33A	58A
21A	33A	58A
22G	34G	56G
22G	35G	54G
23C	36C	56C
23T	35T	55T
23G	36G	54G
23C	36C	53C
26A	36A	53A
27C	36C	52C
28T	38T	53T
28T	40T	52T
30G	39G	54G
31G	39G	54G
32A	43A	55A
33A	43A	54A
33A	42A	54A
33G	43G	54G
33G	43G	54G
34G	44G	54G

34C	43C	53C
34A	42A	52A
34G	45G	54G
34A	45A	54A
34A	45A	54A
35A	44A	52A
35T	44T	52T
35A	43A	50A
36G	43G	51G
34G	43G	51G
34C	43C	50C
35C	44C	50C
35A	43A	50A
33G	44G	48G
32A	45A	49A
32G	45G	48G
32T	46T	47T
33T	45T	47T
34T	44T	44T
34A	44A	44A
31A	44A	44A
31G	45G	43G
31G	45G	45G
31G	45G	43G
32C	43C	43C
34G	45G	42G
34A	45A	40A
33A	44A	38A
33A	46A	38A
34T	46T	37T
35C	44C	36C
36C	43C	36C
37C	46C	37C
37A	43A	37A
38G	45G	37G
38G	44G	36G
38G	43G	36G
38C	44C	35C
38A	43A	36A
38A	44A	35A
38T	43T	34T
39C	44C	34C
39T	44T	34T

39C	44C	34C
39T	43T	34T
39T	43T	34T
40C	45C	32C
41C	44C	31C
41A	45A	32A
41C	46C	32C
41G	47G	31G
40T	47T	31T
40T	49T	32T
40G	49G	31G
41G	50G	32G
41C	50C	34C
42T	51T	34T
42G	51G	34G
42C	51C	34C
42C	51C	34C
42A	50A	34A
42G	50G	35G
42A	48A	33A
41C	50C	32C
41T	49T	31T
41T	50C	33C
41T	50T	34T
41C	50C	33C
41C	50C	33C
41A	49A	33A
41G	49G	33G_10C
41T	49T	21T_14C
43C	49C	36C
43C	48C	37C
43C	49C	37C
42T	50T	35T
43G	49G	35G
43C	47C	35C
43A	47A	36A
42A	47A	37A
42A	47A	36A
39T	48T	36T
39C	48C	36C
40T	49T	36T
40T	49T	34T
39C	48C	33C

39T	47T	34T
39C	47C	35C
40C	47C	34C
40C	47C	32C
40A	45A	33A
40T	45T	33T
41C	42C	33C
40A	42A	32A
41G	42G	31G
41C	42C	31C
41C	41C	31C
41T	42T	31T
41T	41T	31T
41G	41G	32G
41A	40A	32A
40C	39C	32C
39A	41A	31A
39C	40C	31C
38T	40T	31T
39T	40T	30T
41C	39C	30C
42T	40T	34T
40T	40T	34T
38A	40A	33A
39T	39T	33T
38T	39T	34T
37T	39T	34T
36C	38C	35C
35A	36A	34A
34G	36G	34G
34C	36C	34C
34T	36T	35T
34C	35C	34C
33A	35A	36A
34C	35C	38C
34T	35T	38T
34T	34T	38T
34A	34A	38A
35G	34G	36G
33A	34A	35A
33G	34G	36G
34A	34A	34A
33G	34G	35G

34G	34G	35G
34G	34G	33G
33T	33T	33T
32A	34A	33A
33T	32T	34T
33T	32T	34T
33T	32T	33T
34A	32A	31A
33T	32T	32T
32G	32G	32G
32G	30G	32G
33G	31G	31G
32T	30T	30T
31G	30G	28G
31T	31T	25T
30C	31C	28C
28A	32A	30A
28T	30T	30T
28C	31C	30C
28T	30T	29T
27C	30C	28C
26T	30T	28T
25G	30G	29G
24A	28A	28A
25C	27C	28C
24C	27C	29C
24C	26C	29C
24T	27T	30T
24T	27T	29T
24C	27C	29C
25C	26C	30C
24T	26T	29T
23C	26C	29C
22T	26T	29T
23C	26C	29C
23C	26C	30C
21A	25A	26A
23C	24C	31C
22C	23C	31C
21T	23T	30T
22C	23C	30C
22G	21G	29G
22G	22G	29G

22A	20A	29A
21A	20A	30A
20C	18C	31C
20G	19G	31G
20C	18C	31C
20T	17T	27T
20T	17T	27T
20G	15G	27G
20G	16G	27G
20C	16C	28C
19C	16C	28C
19T	16T	27T
19G	16G	29G
19C	16C	29C
19T	16T	29T
19T	16T	28T
null	4A_16C	29C
9T	12A	27A
10C	24C	26C
19C	8C	12G_13C
19C	16C	24C
19C	16C	24C
19C	16C	25C
19C	14C	25C
19C	14C	24C
19C	12C	24C
10T_9C	13C	22T_20C
19T	14T	5T_2C
19T	14T	23T
19T	14T	25T
19T	14T	24T
19T	12T	22T
19T	11T	23T
18T	11T	22T
18T	11T	23T
18G	11G	24G
18C	11C	24C
17C	11C	24C
17A	11A	24A
17C	11C	24C
17A	11A	24A
16C	11C	13T_11C
16G	11G	24G

16A	11A	24A
16C	11C	22C
16T	11T	20T
16C	11C	21C
16C	11C	21C
15C	11C	22C
14T	11T	22T
16C	11C	22C
16C	12C	22C
16A	10A	22A
16T	10T	22T
15A	10A	22A
14A	10A	22A
14A	10A	21A
14C	10C	21C
13A	10A	21A
12A	10A	20A
12G	10G	19G
12C	9C	19C
12A	9A	18A
12C	9C	19C
12G	9G	18G
11G	9G	17G
12C	9C	17C
12T	9T	17T
12G	9G	17G
12C	1T_1G_8C	17C
12C	10C	17C
11C	9C	17C
10A	10A	16A
9G	10G	14G
9A	10A	14A
9C	10C	14C
8C	10C	15C
8C	10C	15C
8C	10C	15C
8A	10A	15A
7G	10G	15G
7G	10G	13G
7C	9C	13C
7C	9C	13C
6T	9T	11T
6C	8C	11C

6C	8C	11C
6C	8C	11C
6C	8C	10C
6C	8C	9C
6C	8C	9C
5C	8C	10C
5C	8C	11C
5C	6C	10C
5A	6A	10A
5C	6C	10C
4G_1C	6G	10G
5C	6C	10C
5C	6C	10C
4A_1C	6A	9A
5T	5T	9T
5A	5A	9A
5C	5C	8C
4A	5A	7A
4C	5C	7C
4A	5A	7A
4C	6C	7C
4C	6C	9C
4T	6T	9T
4C	6C	8C
3C	6C	10C
3G	5G	10G
4T	5T	10T
4T	5T	12T
4C	5C	13C
3T	5T	14T
3A	5A	14A
3T	5T	15T
3T	5T	15T
4A	5A	15A
4G	5G	16G
4T	5T	15T
4C	5C	15C
4T	5T	15T
4C	5C	16C
4G	5G	16G
3C	4C	16C
3C	null	16C
3T	null	17T

null	4T	14T
null	4T	17C
4C	4C	17C
4C	4C	17C
4C	4C	17C
4C	4C	17C
4C	4C	17C
4C	5C	18C
4C	5C	11A_8C
4C	5C	17C
4C	5C	18C
4A	5A	18A
4A	6A	18A
5G	6G	18G
5G	6G	20G
5C	6C	21C
5T	5T	21T
5G	6G	21G
5G	6G	22G
5C	6C	22C
5G	5G	23G
5G	5G	24G
5A	5A	24A
5G	5G	25G
5C	5C	25C
6C	5C	25C
6T	5T	25T
6G	5G	25G
7T	5T	24T
8A	5A	25A
9A	5A	25A
9T	5T	25T
9T	5T	26T
9C	5C	24C
10T	5T	25T
10C	5C	25C
10C	5C	26C
10C	5C	26C
10A	5A	26A
10G	5G	25G
10C	5C	26C
10G	5G	26G
10G	5G	26G

10C	6C	26C
9T	6T	28T
9G	5G	29G
9A	5A	27A
9C	5C	28C
9A	5A	30A
9C	5C	29C
9C	5C	30C
9A	5A	30A
9C	5C	29C
9A	5A	32A
9G	5G	34G
9G	5G	31G
9G	5G	31G
9A	5A	30A
9T	5T	31T
9G	5G	31G
9G	5G	31G
9C	5C	30C
9G	5G	30G
9G	4G	29G
9C	4C	30C
9A	3A	28A
9G	4G	28G
9G	4G	28G
9A	4A	27A
9C	4C	28C
9A	4A	28A
9T	4T	26T
9C	4C	27C
9A	4A	26A
9C	4C	26C
9T	4T	27T
9C	4C	27C
9T	5T	27T
10T	5T	26T
10C	5C	28C
11A	5A	28A
12G	5G	29G
12G	5G	29G
12G	5G	15A_1T_13G
12G	5G	28G
11G	5G	27G

12C	4G_1C	27C
12C	5C	26C
12C	5C	25C
12C	5C	25C
12C	5C	24C
12C	5C	23C
12C	5C	23C
12T	6T	24T
12T	6T	24T
12T	5T	24T
12C	5C	24C
14A_19C	4A_5C	16A_25C
5A	1A	9A
5C	5C	26C
4A_1C	5A	27A
12C	5C	28C
12A	5A	26A
12C	5C	26C
12A	5A	27A
11C	5C	26C
11A	5A	26A
11C	4C	28C
11A	5A	29A
11C	5C	29C
10A	5A	29A
10C	5C	29C
9A	5A	29A
7C	5C	31C
7A	4A_1C	14A_1G_14C
7C	5C	27C
7A	5A	28A
7T	5T	29T
7G	5G	28G
7G	5G	29G
7A	5A	29A
7T	5T	32T
7T	5T	32T
7G	5G	32G
7G	5G	32G
7C	5C	32C
7A	5A	30A
7G	5G	29G
7G	5G	28G

6C	4C	28C
7G	4G	27G
7A	4A	27A
7A	4A	27A
7G	5G	27G
7G	4G	27G
12T_13C	4C	27C
1T	4T	27T
null	4C	28C
null	4T	28T
6C	4C	29C
6T	4T	30T
6C	4C	31C
6G	5G	32G
8T	5T	30T
9T	5T	32T
9T	6T	32T
10G	6G	33G
10T	6T	33T
10C	6C	37C
10A	6A	38A
10A	6A	39A
10C	6C	38C
9G	6G	39G
9C	7C	40C
9C	7C	40C
9G	7G	18A_22G
10G	7G	39G
10A	7A	40A
9C	7C	40C
10T	7T	40T
10G	7G	40G
10A	7A	40A
9C	7C	41C
9A	7A	42A
10G	7G	42G
10A	6A	44A
10A	6A	45A
10T	6T	45T
7T_3G	6T	45T
10A	6A	42A
12C	6C	40C
12T	7T	41T

12C	8C	42C
12G	8G	42G
12C	8C	41C
12C	8C	41C
12C	8C	42C
12T	8T	40T
12C	9C	40C
12A	9A	40A
12A	9A	42A
12A	9A	45A
12T	10T	45T
12G	10G	47G
13A	10A	47A
13A	11A	46A
13A	11A	44A
13C	11C	46C
13G	11G	46G
14A	12A	46A
14T	13T	45T
14C	12C	43C
14T	12T	44T
14A	13A	18A_26G
14T	13T	46T
14C	13C	44C
14T	13T	47T
14G	13G	47G
14G	13G	49G
14G	12G	49G
14G	12G	49G
14G	11G	50G
14A	11A	50A
15G	11G	52G
15T	11T	53T
15T	12T	52T
14A	12A	56A
14A	12A	56A
14A	12A	52A
14C	12C	56C
14C	12C	55C
14T	12T	54T
14A	12A	53A
14T	12T	54T
14T	13T	54T

14T	13T	56T
14C	14C	55C
14C	14C	54C
14A	14A	51A
14G	14G	50G
14T	13T	49T
14G	14G	50G
14G	14G	50G
14A	14A	49A
14G	14G	49G
14C	14C	49C
14T	14T	49T
14G	14G	48G
14A	14A	47A
14G	14G	48G
14A	14A	48A
14A	14A	48A
14G	14G	47G
14A	14A	48A
14G	14G	47G
14A	14A	44A
6G_8C	14G	43G
14A	14A	43A
13G	14G	43G
13A	14A	43A
13G	14G	43G
13G	14G	23G
13G	14G	22G
13T	14T	22T
13T	14T	22T
13T	14T	22T
13T	14T	22T
5A_3T	15A	23A
3T_5G	15G	22G
8G	15G	22G
8G	15G	21G
12A_12T_32G_9C	15G	21G
10G	16G	32G
10G	16G	30G
10G	18G	30G
10A	18A	29A
10C	18C	31C
10C	18C	30C

10C	18C	30C
10A	18A	30A
10G	18G	30G
10C	18C	30C
10A	17A	30A
10A	17A	30A
9T	17T	28T
9G	17G	28G
8T	17T	25T
8C	17C	27C
8A	17A	27A
8A	17A	27A
8G	17G	27G
8A	18A	27A
8G	18G	25G
8C	17C	26C
8C	16C	26C
8C	16C	25C
8A	18A	23A
8A	18A	21A
8T	19T	21T
8T	19T	18T
8A	19A	18A
8A	19A	18A
8T	19T	18T
9T	19T	19T
7T	19T	18T
7G	19G	17G
7G	18G	17G
7G	20G	17G
7G	20G	16G
7C	20C	16C
3A_4G	20A	16G
7G	20G	16G
7G	19G	14G
7G	19G	14G
7T	19T	13T
7G	19G	16G
7G	19G	17G
7G	19G	16G
7G	18G	17G
7C	18C	18C
7A	18A	20A

8G	18G	23G
8G	18G	22G
8C	17C	18C
8C	18C	19C
8C	17C	20C
8C	17C	20C
8C	17C	20C
7C	16C	20C
8T	16T	18T
7T	15T	19T
7C	15C	19C
7C	15C	19C
7C	16C	21C
7C	15C	21C
7C	15C	21C
6A	16A	20A
7C	16C	22C
7C	16C	22C
7C	16C	15A_8C
7C	16C	24C
6A	15A	24A
7C	16C	24C
7C	16C	25C
7C	16C	25C
7C	15C	27C
7G	14G	27G
6T	15T	27T
7C	15C	28C
6A	15A	29A
6C	15C	29C
6A	14A	31A
6A	16A	31A
6G	16G	32G
6G	15G	32G
6G	15G	32G
6C	14C	18T_15C
6G	14G	33G
5A	14A	34A
5T	14T	34T
5G	14G	34G
6A	15A	34A
7G	15G	34G
6T	15T	34T

8C	16C	36C
8G	16G	36G
9G	16G	36G
9A	17A	40A
8T	17T	41T
8G	17G	41G
8T	17T	41T
8A	17A	41A
8T	17T	39T
8C	17C	41C
8C	17C	42C
8G	18G	43G
8A	18A	43A
7G	18G	42G
8A	18A	42A
8T	19T	42T
8C	19C	43C
9C	19C	43C
9A	19A	43A
9C	19C	45C
9C	20C	47C
9A	20A	51A
9A	20A	51A
9C	20C	52C
9T	20T	53T
9T	21T	53T
9T	21T	52T
9A	21A	50A
9T	20T	53T
9G	19G	52G
10G	17G	52G
10A	17A	52A
10C	17C	54C
10G	17G	55G
10G	18G	56G
10A	18A	57A
11G	19G	57G
11G	20G	58G
11A	20A	58A
12T	20T	55T
12A	21A	57A
12C	19C	56C
12C	20C	56C

13T	20T	56T
14T	21T	57T
16T	22T	58T
16A	23A	57A
16T	22T	57T
15A	22A	58A
16A	23A	59A
16G	23G	61G
16C	23C	59C
16C	22C	62C
16A	23A	62A
16A	21A	63A
17A	22A	63A
17A	23A	63A
17A	23A	61A
17C	23C	63C
17C	23C	63C
16T	23T	63T
16A	23A	63A
16T	23T	64T
17A	22A	63A
17A	22A	62A
17A	22A	62A
17G	24G	62G
18A	23A	61A
19G	23G	62G
20G	23G	62G
21G	25G	60G
22C	25C	60C
22C	25C	60C
22G	25G	57G
23A	26A	57A
21G	10A_26G	59G
22A	26A	60A
21A	26A	61A
21A	25A	60A
21A	25A	60A
21A	25A	58A
21A	25A	57A
21A	27A	57A
21A	9A_19C	55A_21C
22A	28A	54A
20A_6C	28A	56A

13C	28C	54C
22A	28A	55A
22C	30C	53C
22A	29A	53A
22G	29G	52G
23T	29T	48T
23G	29G	47G
22C	29C	46C
23A	29A	45A
25G	29G	46G
26G	29G	46G
26C	29C	45C
26T	29T	43T
26G	30G	43G
30A	30A	42A
31A	31A	41A
31A	31A	40A
32A	32A	39A
32G	32G	38G
33C	33C	38C
34A	33A	38A
34A	33A	37A
34A	33A	39A
34G	33G	40G
36T	33T	40T
36T	33T	40T
35C	33C	39C
36C	34C	38C
37T	34T	35T
38T	35T	22T_13C
38G	35G	35G
38G	35G	35G
38C	36C	33C
37A	36A	31A
36A	35A	30A
36A	34A	28A
36G	34G	26G
36T	35T	27T
35G	34G	26G
35C	35C	26C
35C	37C	27C
35T	38T	25T
35C	40C	25C

35T	40T	25T
35T	40T	25T
36T	39T	22T
36T	40T	30T_8G
35G	39G	22G
36G	40G	22G
36G	38G	22G
36G	39G	23G
36G	40G	24G
35G	40G	24G
34G	39G	24G
34T	38T	19T_5G
34G	38G	24G
34A	38A	24A
34C	38C	26C
34T	38T	26T
35A	38A	24A
34C	37C	24C
35A	37A	25A
35G	37G	24G
36A	37A	23A
36T	37T	24T
35C	36C	25C
35C	37C	27C
35C	36C	27C
35A	36A	27A
35G	36G	26G
35A	35A	24A
35A	35A	26A
36T	34T	25T
36C	34C	25C
35C	35C	25C
35C	35C	25C
36C	35C	24C
37C	35C	25C
36A	35A	24A
38G	34G	25G
37A	34A	24A
36G	34G	24G
35T	34T	24T
35C	34C	26C
36C	33C	27C
37A	33A	30A

37C	33C	30C
38A	34A	32A
38C	35C	33C
38A	36A	33A
39T	36T	33T
39A	36A	33A
40A	36A	34A
41G	36G	33G
41C	36C	31C
40G	36G	32G
41C	38C	33C
40T	39T	34T
40T	39T	35T
40G	39G	37G
40C	42C	37C
40C	42C	40C
40C	43C	40C
22A_17G	43G	41G
37T	43T	41T
36A	43A	42A
37T	44T	43T
37T	44T	42T
37T	44T	42T
36T	43T	42T
36G	43G	42G
37G	43G	45G
37A	44A	46A
37T	45T	46T
37T	46T	53T
37A	47A	52A
37G	47G	53G
38G	45G	53G
38C	44C	29T_24C
38A	44A	53A
37A	44A	53A
36A	44A	52A
37G	44G	53G
37T	43T	52T
38G	44G	54G
39G	45G	55G
38T	46T	57T
40C	47C	57C
41T	47T	56T

40G	48G	56G
39T	48T	56T
23A_16G	48G	54G
39G	48G	55G
39A	49A	57A
40C	48C	57C
37C	48C	55C
36A	48A	54A
36A	48A	56A
35C	46C	58C
20A_14C	47C	60C
34T	45T	60T
33T	47T	59T
34G	46G	60G
35A	46A	64A
35C	46C	65C
33T	46T	65T
33G	46G	66G
33G	47G	66G
34G	46G	68G
33G	45G	67G
32C	45C	68C
32G	45G	67G
32T	44T	68T
31C	43C	68C
31G	43G	68G
31C	43C	68C
33C	43C	66C
33C	43C	67C
33C	43C	67C
33T	43T	65T
33G	42G	66G
34G	41G	71G
33G	39G	69G
33T	36T	68T
33G	37G	69G
32C	37C	69C
32T	36T	69T
32G	35G	73G
32A	36A	74A
32A	36A	72A
32A	36A	72A
32T	35T	70T

32G	35G	72G
14T_18C	35C	42T_29C
32G	35G	71G
32G	34G	71G
32A	35A	71A
14A_18G	36G	41A_30G
32G	35G	70G
31G	37G	72G
31T	36T	72T
31G	37G	69G
31C	38C	69C
30C	38C	68C
31A	39A	66A
32A	39A	66A
32A	39A	66A
33A	39A	68A
34T	39T	69T
34A	39A	70A
34C	38C	71C
33A	39A	72A
32G	39G	71G
32A	39A	71A
34G	37G	70G
34G	37G	69G
33T	36T	26T_42G
32A	36A	67A
31A	37A	67A
30G	36G	67G
30T	34T	66T
31C	35C	68C
31T	35T	68T
31A	35A	68A
30C	35C	68C
30T	35T	67T
30C	35C	69C
29G	34G	68G
14T_15C	32C	64T
29G	32G	66G
28A	32A	65A
28G	31G	67G
12T_15C	31C	66C
11A_15G	31G	62A
28T	30T	62T

28T	31T	63T
28A	29A	63A
28A	28A	64A
28G	28G	64G
28A	27A	66A
28A	25A	67A
28A	24A	69A
29A	23A	69A
29T	23T	69T
29A	22A	69A
28C	21C	68C
28G	20G	72G
28T	19T	71T
28G	20G	70G
28G	20G	70G
27C	20C	70G
28A	20A	66A
15A_14G	19A	67A
29C	19C	67C
29A	18A	66A
29G	17G	66G
29C	17C	67C
28T	18T	64T
28C	18C	64C
28T	18T	63T
27C	18C	65C
13A_14G	18A	66G
26G	19G	65G
26T	18T	64T
26C	18C	63C
25C	17C	64C
25T	16T	63T
23G	15G	63G
21T	15T	63T
22T	14T	61T
22C	14C	63C
21T	14T	63T
21G	13G	63G
21T	12T	62T
20T	12T	59T
19C	12C	59C
19T	11T	61T
19T	11T	60T

19C	11C	23T_82C
19T	10T	61T
19T	10T	52T
18C	10C	55C
18C	10C	50T_51C
19T	10T	35T
18T_21C	10T_12C	34T_57C
21C	10C	61C
21C	10C	60C
22C	9C	60C
21C	8C	60C
21C	7C	62C
21C	7C	60C
21C	7C	58C
21C	7C	58C
21C	7C	58C
19A	6A	51A
20A	8A	52A
19A	7A	52A
19C	8C	51C
19C	8C	51C
17T	10T	51T
18A	10A	52A
20A	10A	52A
20A	9A	51A
21C	10C	50C
21T	10T	51T
21T	11T	49T
21C	12C	53C
20C	12C	49C
22T	13T	48T
23T	12T	48T
24T	14T	47T
24T	13T	42T
25T	14T	41T
25T	14T	44T
25C	13C	43C
26T	13T	43T
26T	14T	43T
25C	13C	42C
25A	13A	42A
15G_10C	13C	39G
25G	12G	39G

27G	13G	46G
26G	12G	47G
25T	12T	47T
27G	12G	49G
27G	12G	50G
25T	13T	49T
26A	14A	47A
27C	15C	46C
28C	16C	46C
28A	16A	46A
29G	17G	45G
31C	17C	45C
31T	16T	41T
32C	17C	46C
32C	18C	45C
31T	7A_12T	44T
33G	20G	43G
33G	20G	43G
32C	20C	44C
32A	22A	43A
32A	22A	45A
32A	21A	44A
32C	23C	42C
31A	26A	41A
31T	26T	42T
31T	26T	42T_47G
31G	26G	55G
31G	27G	55G
32G	27G	55G
32G	27G	55G
32G	27G	54G
31G	27G	54G
31G	27G	52G
31G	27G	51G
31C	28C	49C
30G	27G	51G
31C	31C	50C
31T	32T	50T
31C	32C	52C
32T	31T	52T
32T	32T	54T
32C	32C	54C
33C	31C	56C

33A	33A	57A
34C	32C	60C
36T	34T	60T
35C	33C	62C
37A	33A	63A
37C	34C	61C
39C	34C	60C
39T	35T	60T
41C	35C	60C
41T	36T	61T
42T	35T	62T
42C	36C	59C
42C	37C	61C
42C	38C	61C
43C	36C	60C
43A	38A	62A
43T	38T	32T_30C
43A	37A	62A
43G	37G	61G
42G	37G	25T_60G
42A	36A	63A
42A	37A	63A
42A	37A	62A
41A	37A	66A
42G	37G	64G
42G	37G	63G
43A	37A	59A
44T	36T	59T
45G	38G	57G
45C	38C	56C
46T	37T	24T_33C
45G	38G	58G
45G	38G	60G
44G	39G	62G
45A	39A	61A
45C	39C	63C
46T	39T	62T
46A	40A	61A
45G	40G	60G
46A	40A	59A
47G	42G	60G
47G	42G	61G
48G	43G	62G

47G	43G	61G
49G	43G	57G
49C	44C	59C
50C	45C	59C
51T	46T	59T
53A	46A	60A
54A	46A	60A
55T	48T	58T
57C	48C	58C
59C	46C	59C
60T	45T	59T
61G	46G	59G
61C	47C	57C
64A	46A	58A
62C	46C	59C
30T_32C	46C	59C
61A	45A	61A
62T	46T	61T
62A	46A	59A
63T	45T	65T
64T	44T	68T
63T	47T	65T
63C	46C	66C
62A	48A	67A
62T	46T	65T
63G	47G	63G
63C	50C	60C
62A	51A	62A
62T	51T	63T
64T	51T	62T
62C	51C	59C
62T	52T	58T
63T	52T	60T
64C	51C	62C
64C	53C	62C
65T	53T	61T
65G	54G	61G
66A	54A	59A
67C	55C	59C
67T	56T	59T
68G	55G	57G
68C	54C	57C
68C	52C	55C

67A	28A_26T	55T
67A	54A	55A
64C	52C	55C
62T	53T	57T
61T	52T	61T
61C	54C	63C
61T	53T	65T
59C	55C	61C
61T	55T	63T
61C	55C	65C
63T	54T	65T
63A	53A	65A
63A	54A	64A
63G	55G	62G
63C	54C	61C
63C	51C	65C
64A	27A_27G	64G
65G	46T_44G	65G
65G	56G	65G
64T	56T	65T
65G	57G	68G
66T	57T	71T
67G	58G	71G
67T	58T	72T
67G	57G	72G
67T	56T	71T
70G	58G	74G
69T	58T	74T
37A_32G	57G	74G
69T	56T	73T
67G	55G	74G
67C	56C	74C
67T	56T	73T
67T	28T_29C	75C
67T	56T	75T
67G	56G	74G
65T	56T	73T
66G	57G	74G
65G	57G	75G
66C	56C	76C
64T	55T	76T
63G	55G	74G
65T	55T	75T

68T	54T	74T
68T	53T	73T
69T	53T	74T
70A	54A	68A
71T	55T	64T
72G	55G	63G
74T	55T	66T
74C	56C	66C
74T	57T	67T
74C	58C	68C
75C	58C	68C
75A	59A	66A
77G	60G	33T_33G_1C
78T	59T	67T
77T	60T	66T
78T	60T	69T
78C	62C	68C
77C	61C	69C
75A	62A	70A
76G	62G	71G
76C	62C	71C
76C	62C	69C
77T	64T	71T
77T	65T	71T
77C	65C	74C
77T	68T	75T
77T	68T	75T
76T	68T	74T
76G	67G	73G
76T	67T	73T
74C	67C	72C
73A	65A	68A
74C	67C	67C
73T	69T	65T
73G	68G	66G
72G	69G	64G
73T	70T	62T
72T	69T	62T
71A	71A	61A
70T	71T	62T
68G	71G	62G
68C	70C	63C
66T	71T	61T

64G	70G	60G
64G	70G	59G
65G	69G	59G
62T	70T	59T
60A	70A	56A
61A	71A	55A
61A	71A	56A
61G	69G	55G
60G	71G	55G
61C	71C	55C
59A	68A	52A
59G	69G	54G
59A	68A	53A
59T	68T	55T
59G	68G	53G
59G	67G	55G
57G	66G	55G
155A_80T_80G_128C	63A	56A
43G	62G	55G
43C	63C	54C
35T	65T	55T
36G	64G	56G
35T	64T	57T
36G	63G	57G
36C	64C	57C
36T	65T	56T
36C	66C	56C
35A	64A	58A
35A	65A	58A
34A	65A	59A
34C	66C	57C
34C	67C	63C
34A	67A	63A
34C	67C	62C
34A	65A	61A
34T	67T	24T_38C
34C	64C	61C
33T	68T	63T
33G	68G	66G
35G	66G	63G
35A	64A	63A
35C	64C	65C
62A	64A	63A

63G	64G	63G
63C	63C	62C
65C	62C	61C
66T	62T	60T
67C	62C	60C
67A	62A	60A
68C	61C	60C
67T	60T	63T
65T	60T	60T
66C	60C	60C
66C	59C	61C
67C	59C	60C
68C	58C	61C
67T	59T	58T
66C	59C	60C
67C	59C	59C
67C	59C	60C
65A	59A	61A
67G	58G	63G
67T	57T	64T
68C	59C	64C
67A	59A	64A
68A	56A	65A
69A	56A	67A
67G	56G	67G
67C	56C	66C
67C	56C	64C
67T	55T	63T
67C	55C	61C
33T_35C	55C	61C
68C	55C	61C
69T	55T	61T
69A	56A	59A
71A	56A	57A
69A	56A	57A
69C	57C	57C
68C	55C	58C
67C	55C	58C
66A	55A	57A
66C	55C	57C
65C	56C	56C
66A	55A	57A
66T	56T	57T

66G	55G	56G
66C	55C	54C
67T	56T	55T
67T	56T	56T
66C	57C	55C
67C	59C	56C
67T	59T	54T
67T	57T	54T
67T	57T	55T
67T	57T	54T
66C	57C	54C
65T	58T	52T
64A	21A_37C	52A
66T	57T	53T
66C	55C	53C
64T	55T	51T
62C	51C	53C
62A	52A	52A
61T	52T	53T
61T	52T	52T
62T	51T	53T
62C	50C	50C
62C	51C	48C
64T	50T	49T
65C	49C	52C
63A	49A	49A
63C	47C	52C
64C	46C	52C
62T	47T	52T
62T	45T	53T
61T	45T	54T
63C	45C	54C
63C	45C	57C
62A	46A	56A
64C	47C	53C
62C	47C	54C
62T	48T	53T
62T	47T	52T
61A	47A	52A
60T	46T	52T
61T	46T	52T
61C	46C	52C
61C	46C	53C

61A	46A	54A
61T	46T	54T
62A	46A	51A
64C	46C	52C
63C	45C	53C
63T	44T	53T
62A	44A	53A
63A	45A	53A
60G	48G	52G
60C	49C	55C
62C	49C	57C
60A	47A	58A
59A	51A	58A
59C	51C	59C
57A	51A	59A
58C	51C	59C
60A	50A	61A
59G	50G	61G
58A	50A	61A
58G	49G	62G
58T	49T	61T
58G	49G	61G
58C	48C	60C
58T	47T	59T
58C	47C	60C
58C	47C	61C
58A	46A	59A
59C	44C	61C
59A	46A	62A
59A	46A	62A
55T	47T	62T
24T_31G	49T	32T_32G
53A	48A	63A
53A	49A	63A
53G	49G	62G
53C	48C	60C
52C	48C	61C
51A	48A	62A
50A	48A	62A
50T	46T	62T
49G	46G	61G
49C	46C	61C
21T_28C	45T	59C

29A_22G	44G	56G
51A	47A	57A
51G	48G	58G
51T	48T	59T
52G	47G	61G
53A	48A	61A
52G	49G	61G
52G	49G	61G
52G	48G	59G
53C	48C	62C
53A	48A	61A
52T	47T	61T
52T	47T	61T
52G	47G	61G
53C	47C	60C
53C	49C	59C
54T	50T	59T
54T	50T	58T
54G	50G	58G
54A	51A	57A
54T	51T	60T
52A	51A	61A
53C	51C	62C
54A	50A	62A
53G	50G	62G
53A	50A	61A
53C	50C	60C
53C	50C	58C
52T	51T	58T
50T	49T	58T
53C	50C	58C
52C	49C	58C
53T	50T	59T
53C	48C	29T_29C
54G	19A_30G	28A_28G
54G	48G	56G
52G	49G	54G
53G	48G	55G
54C	48C	24T_31C
53G	45G	58G
55C	46C	59C
55T	45T	59T
55T	47T	61T

55A	46A	59A
57A	45A	63A
57C	45C	63C
58G	45G	64G
57A	46A	65A
57C	45C	65C
58T	46T	65T
60G	47G	65G
60T	48T	64T
61C	49C	63C
61T	50T	62T
60T	52T	62T
61G	52G	60G
59C	52C	59C
59A	51A	60A
58G	53G	59G
59A	54A	59A
59A	54A	59A
58T	54T	59T
58G	54G	59G
58T	55T	60T
58C	54C	59C
57T	55T	59T
57G	55G	59G
58C	21T_30C	20T_32C
59C	53C	53C
60C	21T_32C	20T_33C
60T	33T_22C	33T_20C
62A	60A	52A
62C	61C	53C
63A	61A	53A
62G	61G	50G
62A	61A	50A
63T	62T	50T
62T	35T_29C	29T_21C
63G	35T_30G	21A_29G
62G	65G	58G
60A	66A	55A
60G	68G	58G
60G	68G	57G
60T	67T	60T
61G	69G	62G
61C	67C	64C

62C	65C	65C
61T	67T	65T
62T	68T	66T
61C	64C	66C
62A	64A	65A
62C	32A_1G_31C	64C
62T	63T	64T
60G	63G	64G
60G	63G	65G
60C	64C	65C
60C	64C	66C
60T	64T	68T
59C	63C	70C
60A	63A	70A
60A	63A	68A
60A	63A	68A
61G	62G	68G
60G	64G	67G
60C	64C	67C
30T_30G	63T	69T
60T	63T	68T
60T	61T	68T
59T	59T	68T
60C	59C	69C
59A	58A	67A
60G	59G	66G
61G	61G	65G
61G	59G	66G
61A	62A	62A
61G	62G	60G
61T	62T_27C	58T_56C
61C	62C	61C
61C	62C	60C
60A	61A	63A
58C	61C	63C
58C	60C	63C
57T	59T	63T
58G	60G	64G
57G	61G	66G
56C	61C	67C
57C	62C	65C
57T	62T	66T
56T	61T	66T

53C	63C	66C
53A	65A	67A
53G	66G	68G
53G	68G	65G
52T	67T	62T
52C	68C	62C
51T	66T	62T
53C	67C	63C
53T	66T	61T
52G	66G	61G
52T	64T	62T
52A	64A	61A
53A	66A	61A
53C	66C	63C
51T	65T	62T
51G	66G	63G
52C	66C	66C
52C	65C	65C
53C	65C	67C
53C	66C	66C
53T	68T	68T
51T	68T	67T
51C	69C	68C
50C	68C	68C
49T	68T	66T
48C	67C	66C
47C	67C	64C
47A	70A	63A
47A	69A	62A
46A	69A	62A
45T	70T	62T
47A	69A	62A
49C	69C	63C
48A	69A	62A
49C	70C	1A_17G_43C
47C	70C	62C
47C	69C	61C
47C	69C	60C
47A	70A	60A
49A	71A	58A
48A	71A	59A
47C	70C	59C
47A	70A	59A

47A	71A	61A
47C	69C	60C
46A	71A	59A
45C	69C	59C
47A	71A	59A
45A	70A	58A
45A	68A	57A
25T_19C	69C	57C
44G	68G	18A_37G
44C	68C	54C
44A	66A	35A_18C
44A	66A	55A
44A	68A	52A
43G	67G	52G
43T	68T	31T_19C
42G	67G	53G
40T	65T	53T
40T	65T	52T
38C	64C	56C
38T	64T	56T
37T	64T	55T
36C	64C	54C
36A	64A	52A
37T	63T	53T
37T	63T	54T
37C	61C	52C
36A	61A	51A
36T	61T	51T
36C	59C	52C
36C	59C	52C
36A	59A	52A
36C	58C	53C
36A	58A	54A
36T	58T	52T
36C	58C	53C
36C	28T_29C	54C
35T	56T	57T
35C	56C	57C
35A	28A_28G	59A
37G	57G	60G
36C	57C	58C
36T	56T	60T
36T	58T	59T

36T	58T	57T
36C	57C	57C
36A	57A	56A
36A	59A	55A
36A	58A	52A
36A	59A	55A
36A	60A	54A
35C	31T_29C	51C
35G	60G	51G
35A	61A	49A
35A	61A	51A
35T	62T	53T
35T	62T	26T_30G
36T	63T	56T
36T	64T	55T
37G	64G	57G
36T	62T	56T
36G	61G	57G
36C	62C	58C
36T	63T	57T
37C	63C	58C
37G	64G	58G
37C	66C	57C
37C	66C	59C
37A	64A	59A
37T	66T	58T
37G	66G	57G
37C	66C	56C
36T	66T	56T
36C	67C	56C
36T	67T	58T
36T	66T	59T
36G	67G	57G
38T	67T	59T
39G	67G	59G
40C	65C	58C
41A	64A	59A
41A	63A	60A
41T	64T	61T
40A	65A	63A
24T_16C	66T	31T_33C
38A	64A	66A
39G	63G	65G

39C	64C	65C
40T	64T	63T
39T	65T	63T
38C	66C	64C
38C	66C	64C
38A	66A	65A
37G	65G	65G
36C	65C	64C
38A	65A	64A
37T	66T	65T
37C	65C	66C
36T	66T	66T
37G	67G	64G
38C	66C	65C
38C	67C	65C
39A	67A	64A
40G	68G	63G
40C	67C	63C
40T	65T	61T
25A_15G	65G	62G
41G	65G	60G
41T	63T	59T
39G	63G	57G
37C	62C	57C
39A	62A	57A
39G	64G	56G
39C	64C	56C
39C	63C	56C
39A	64A	58A
38C	63C	60C
36C	62C	59C
24A_12G	60A	58A
38G	62G	59G
37A	62A	59A
37T	60T	58T
38C	61C	59C
38A	59A	59A
38G	60G	59G
36T	59T	58T
36T	58T	55T
37A	60A	55A
37T	60T	55T
38G	61G	53G

40T	61T	52T
40G	60G	52G
41A	61A	51A
41T	59T	50T
43T	59T	49T
43C	58C	49C
43T	58T	52T
43G	56G	50G
43G	54G	49G
42G	54G	49G
42A	55A	48A
42G	54G	49G
44C	55C	49C
44C	55C	50C
44C	55C	48C
43A	56A	50A
44G	55G	50G
44A	56A	51A
45A	55A	55A
45A	54A	55A
46A	55A	55A
47A	58A	56A
48A	59A	55A
47T	61T	53T
49A	61A	56A
48A	61A	57A
47A	59A	58A
49A	60A	61A
49C	62C	62C
48T	62T	61T
48T	61T	61T
48C	62C	65C
49T	62T	64T
50C	61C	68C
50C	63C	68C
31A_20G	64A	67G
51A	64A	69A
51A	62A	69A
51C	62C	70C
52A	61A	70A
31T_21C	60C	70C
51T	60T	71T
52G	60G	72G

52A	61A	75A
52A	62A	75A
52A	62A	75A
52A	62A	75A
51A	61A	74A
50G	63G	77G
50A	63A	78A
50G	64G	78G
49A	64A	78A
50G	62G	78G
52G	60G	78G
53G	62G	78G
53T	60T	78T
53C	59C	77C
55T	60T	78T
56G	61G	78G
56A	60A	77A
56C	58C	77C
56C	60C	76C
59T	59T	78T
59G	57G	80G
61A	58A	81A
60C	57C	80C
62A	58A	80A
61A	60A	79A
60A	59A	81A
59A	59A	82A
58C	59C	83C
58A	58A	84A
58A	57A	85A
58A	56A	85A
59G	55G	85G
59C	55C	85C
59A	57A	84A
59A	57A	84A
59T	59T	84T
59A	58A	85A
29A_30T	15A_41T_1C	42A_42T_1C
62C	58C	85C
64C	57C	85C
64T	59T	85T
64A	58A	85A
63C	62C	81C

64C	63C	82C
64C	63C	80C
64A	60A	77A
64G	61G	77G
66G	63G	76G
66C	62C	74C
62A	60A	72A
64C	61C	72C
65C	62C	69C
65C	60C	66C
66T	62T	63T
65C	62C	64C
65C	62C	63C
36A_29G	63G	35A_26G
65G	63G	61G
64C	63C	58C
64T	62T	58T
64C	62C	54C
65C	62C	54C
65T	62T	54T
67A	61A	55A
67T	62T	56T
67C	62C	55C
67A	63A	56A
67T	62T	55T
67T	62T	56T
66C	63C	56C
67C	62C	55C
68C	61C	56C
67A	64A	58A
67A	65A	56A
66T	65T	56T
66T	63T	53T
66G	63G	52G
64G	63G	52G
33T_31C	62C	52C
63G	62G	52G
62A	62A	52A
60C	63C	50C
61A	61A	50A
63C	63C	48C
28G_35C	63G	48C
62T	63T	47T

62C	63C	47C
62C	62C	48C
62T	63T	50T
61G	62G	49G
61G	63G	47G
60G	62G	49G
59G	61G	51G
27A_32G	63G	53G
58T	60T	55T
60G	62G	53G
61A	62A	53A
60T	61T	52T
60G	61G	50G
59G	61G	52G
59G	57G	52G
57A	57A	52A
22A_33G	56G	51G
55T	56T	50T
21T_33C	56T	26T_25C
54G	56G	51G
54T	57T	50T
55A	56A	51A
54G	57G	50G
54T	57T	50T
53T	57T	50T
51G	55G	51G
53G	54G	51G
53G	53G	52G
53C	54C	54C
55C	55C	53C
55C	55C	54C
56A	55A	56A
57T	55T	57T
57C	56C	57C
57T	54T	57T
57G	53G	58G
57G	52G	59G
56A	52A	60A
56A	52A	58A
55A	50A	59A
54G	49G	60G
56C	49C	61C
56A	49A	62A

57C	47C	62C
54C	49C	66C
54T	48T	66T
54G	48G	65G
55G	48G	63G
54T	48T	62C
53T	47T	63T
51A	47A	62A
54G	47G	61G
55G	47G	60G
53A	47A	59A
54G	46G	58G
52A	47A	58A
52G	47G	56G
51T	48T	56T
50C	46C	58C
50T	46T	57T
50T	46T	58T
50A	46A	57A
50G	46G	56G
51G	47G	57G
50A	47A	56A
51A	48A	57A
51G	48G	57G
50G	45G	57G
43A_50C	76A_1T_46C	93A_1T_57C
49G	42G	53G
50A	48A	64A
50G	47G	66G
48A	46A	64A
46G	45G	63G
46C	43C	57C
46G	41G	56G
45G	22G_19C	53C
45T	40T	54T
45C	39C	53C
45T	37T	51T
47C	37C	53C
43T	38T	51T
43C	38C	52C
43G	37G	55G
43T	37T	57T
42C	36C	57C

42C	36C	57C
42T	36T	58T
42A	35A	59A
41T	35T	59T
42C	33C	59C
42A	35A	59A
42C	36C	60C
41A	39A	60A
40A	41A	60A
39A	41A	60A
38A	41A	60A
38A	41A	59A
17G_19C	39G	59C
36T	39T	58T
36T	40T	57T
36C	38C	58C
37A	38A	58A
39A	38A	60A
39C	37C	60C
40A	36A	64A
39T	37T	66T
40C	37C	67C
40T	37T	66T
41C	37C	66C
44C	37C	66C
44A	38A	66A
45C	39C	62C
46G	38G	63G
46T	38T	63T
46C	39C	63C
46C	39C	62C
44G	38G	63G
44G	38G	63G
44A	37A	62A
44G	37G	63G
43T	37T	62T
43T	37T	63T
44C	37C	62C
45A	37A	64A
45C	36C	66C
44A	36A	65A
46A	37A	65A
46G	36G	65G

45G	36G	66G
44A	35A	67A
44C	35C	68C
43C	35C	70C
43A	36A	69A
45G	36G	68G
45C	36C	69C
44T	37T	73T
45C	35C	72C
45C	34C	72C
44G	35G	69G
44A	35A	66A
44G	35G	65G
44C	35C	65C
45C	37C	65C
43G	36G	65G
44T	37T	66T
44G	37G	66G
42A	36A	66A
42C	36C	69C
43C	36C	70C
41T	36T	72T
41T	33T	71T
40G	35G	71G
40A	34A	69A
42A	34A	68A
42A	34A	66A
43A	34A	64A
43C	34C	66C
43A	34A	65A
42G	34G	64G
42G	34G	64G
40T	34T	65T
40G	34G	66G
40C	33C	64C
40G	34G	64G
38G	34G	63G
37A	33A	64A
36A	32A	65A
23T_13C	20T_12C	41T_25C
35C	32C	66C
35C	32C	66C
35G	32G	64G

35C	31C	64C
34A	31A	61A
34C	32C	63C
33C	32C	61C
34C	32C	61C
34G	32G	60G
35C	34C	62C
34T	33T	62T
37C	34C	60C
37C	34C	59C
37C	35C	59C
36A	32A	59A
37C	36C	60C
36G	35G	63G
36C	35C	64C
36C	34C	67C
35C	34C	67C
35T	33T	67T
36G	34G	67G
36C	32C	66C
35T	35T	70T
35T	36T	70T
34T	35T	68T
35G	34G	68G
35C	36C	69C
38A	38A	70A
38A	39A	71A
38G	41G	70G
35C	41C	71C
35C	40C	70C
35G	40G	68G
37C	40C	69C
37T	40T	69T
37C	40C	67C
37T	39T	65T
37A	41A	66A
39A	42A	66A
41G	42G	66G
40C	43C	68C
41A	42A	70A
42G	42G	72G
42A	39A	73A
42C	38C	74C

42T	37T	73T
42G	37G	73G
41C	37C	72C
43T	37T	70T
43T	37T	69T
43T	37T	68T
45C	38C	68C
44T	38T	67T
44C	38C	68C
43A	37A	67A
40G	37G	67G
41T	37T	71T
42G	38G	69G
41G	40G	68G
41C	40C	70C
41T	40T	70T
40T	39T	70T
40T	38T	68T
40G	39G	69G
41C	41C	70C
41C	41C	69C
41A	42A	66A
42C	42C	68C
42G	42G	66G
42G	42G	66G
43G	43G	68G
43A	43A	69A
44A	44A	71A
43A	44A	72A
42G	43G	71G
44G	43G	68G
44A	44A	68A
43G	43G	68G
44G	45G	68G
44A	45A	67A
45C	44C	66C
47A	44A	70A
47A	44A	70A
48A	44A	71A
46A	44A	70A
46A	46A	68A
47C	46C	67C
46A	46A	62A

46G	47G	64G
48C	47C	64C
48A	47A	65A
48G	47G	65G
51A	47A	62A
53T	47T	64T
53T	47T	64T
53C	48C	62C
53C	48C	60C
54G	48G	59G
53T	48T	58T
53C	48C	58C
55T	50T	57T
56G	50G	57G
56G	51G	56G
56A	51A	56A
54G	51G	56G
54G	52G	57G
52T	52T	58T
52A	53A	59A
51T	53T	61T
52G	54G	64G
52A	55A	63A
52A	55A	61A
52A	55A	60A
52T	56T	59T
52G	54G	57G
52G	55G	61G
53T	54T	62T
53G	56G	63G
52G	56G	64G
53T	56T	64T
53T	56T	65T
53A	55A	65A
53T	55T	64T
53T	55T	66T
54G	53G	68G
52A	52A	69A
51T	51T	68T
51A	48A	68A
49A	48A	69A
50G	48G	69G
52A	48A	70A

51A	47A	70A
50A	47A	70A
52T	48T	70T
52A	48A	69A
52T	48T	72T
52T	48T	74T
52G	48G	72G
52T	48T	72T
53G	51G	72G
52C	50C	71C
53T	48T	71T
53G	51G	70G
53G	50G	68G
54G	50G	68G
54T	49T	70T
54G	48G	70G
53G	47G	68G
53C	46C	67C
53A	45A	69A
54G	44G	35G_35C
54A	46A	68A
54G	48G	68G
54G	49G	68G
55C	48C	69C
27A_28T	49T	69T
55G	49G	69G
55G	48G	71G
53A	47A	71A
52A	47A	72A
53A	46A	71A
52T	48T	72T
52A	47A	73A
52A	47A	72A
53A	46A	72A
54G	49G	72G
54A	50A	74A
54T	50T	76T
53C	48C	79C
29A_25G	50G	80G
54G	51G	82G
52G	50G	82G
53A	50A	83A
52A	51A	85A

52A	51A	86A
53A	51A	85A
53G	50G	85G
52C	49C	82C
55A	48A	81A
55T	48T	78T
53C	50C	79C
53A	51A	80A
51G	50G	81G
52C	51C	81C
52C	48C	80C
53A	48A	78A
53C	48C	78C
28T_25C	48T	40T_1G_35C
53G	47G	75G
55C	48C	78C
54G	47G	76G
54T	46T	79T
53T	46T	80T
27T_25C	22T_25C	79T
52G	47G	77G
51G	47G	76G
27A_26G	47G	74G
53G	47G	74G
24T_27C	45C	74C
51C	45C	74C
50C	46C	34T_38C
21A_27G	47G	76G
48A	46A	75A
48A	46A	74A
50G	48G	75G
50A	46A	72A
49G	45G	70G
50G	45G	70G
49T	43T	69T
49C	45C	70C
47A	45A	70A
48C	45C	69C
46C	45C	68C
21A_23C	42A	68A
45C	43C	68C
46G	42G	66G
46T	40T	64T

45C	42C	66C
45T	41T	66T
45G	42G	64G
43A	40A	64A
43C	42C	64C
43C	41C	66C
43C	41C	67C
43C	41C	66C
44A	40A	65A
44G	40G	66G
44G	41G	65G
44C	41C	63C
44T	40T	61T
44G	39G	63G
44A	39A	61A
23A_22G	39A	60A
45G	38G	60G
45C	38C	61C
46T	38T	61T
21T_24C	38C	61C
46G	37G	60G
46C	37C	58C
45T	38T	57T
46A	39A	55A
46G	39G	56G
47A	41A	53A
47G	42G	54G
45G	44G	53G
45A	44A	54A
47A	44A	52A
48T	44T	53T
47C	44C	51C
48T	44T	49T
45G	44G	47G
45C	45C	48C
45C	45C	46C
44C	44C	46C
44C	44C	45C
46C	46C	45C
48C	47C	47C
44A	42A	45A
48C	47C	48C
47C	44C	46C

48C	45C	47C
47C	43C	44C
47T	43T	42T
47C	45C	43C
47T	45T	44T
47G	43G	45G
50G	45G	47G
50C	46C	48C
50C	46C	47C
50G	45G	26A_19G
49A	45A	45A
53T	45T	41T
53A	43A	40A
52G	42G	43G
51C	42C	43C
51C	42C	44C
52C	42C	44C
53A	41A	47A
53G	43G	48G
53C	43C	50C
54T	43T	29T_23C
54G	41G	54G
54A	42A	55A
55G	42G	57G
54C	43C	55C
53C	42C	59C
54C	42C	60C
54T	43T	60T
54T	43T	61T
54T	43T	61T
54C	41C	60C
54T	44T	61T
54G	46G	60G
54T	45T	58T
54T	45T	57T
53C	45C	30T_28C
54A	45A	60A
54G	48G	61G
51C	47C	61C
50C	44C	61C
48A	44A	61A
49G	45G	61G
49G	45G	60G

49C	43C	60C
48C	45C	62C
47A	46A	61A
45A	46A	62A
46T	46T	62T
46A	46A	62A
44C	25T_21C	62C
45G	46G	62G
46T	46T	63T
46T	46T	64T
45A	44A	63A
46C	45C	64C
44T	45T	64T
43C	43C	64C
43C	44C	64C
45C	44C	65C
45A	44A	66A
45T	43T	68T
45T	42T	70T
45G	43G	70G
45A	43A	73A
43T	41T	74T
43T	41T	75T
41G	41G	73G
42C	40C	74C
42C	40C	77C
43A	40A	77A
44G	40G	75G
44T	40T	76T
43T	40T	77T
44G	40G	77G
43C	40C	76C
42C	40C	75C
41A	39A	75A
43C	40C	74C
43C	44C	75C
43C	44C	75C
43C	43C	77C
42C	42C	78C
42A	42A	79A
42C	43C	78C
42T	44T	78T
41G	43G	76G

41G	45G	77G
41T	44T	74T
41T	44T	72T
40T	44T	72T
41A	43A	70A
41T	44T	70T
39A	45A	70A
41T	45T	70T
42T	46T	69T
42G	46G	67G
42T	46T	67T
42C	47C	65C
43G	47G	66G
44C	45C	68C
44T	45T	67T
44C	45C	67C
43T	45T	65T
43C	44C	66C
43A	20A_21G	29A_37G
44A	42A	66A
43T	42T	64T
42G	42G	62G
41T	42T	62T
41T	42T	61T
41T	42T	60T
41T	41T	60T
41A	40A	60A
40G	39G	58G
40T	40T	58T
38G	38G	61G
37G	37G	60G
37G	37G	58G
37A	37A	56A
37C	37C	56C
37A	37A	56A
37A	37A	53A
37T	36T	52T
37A	36A	52A
37T	36T	51T
37A	36A	51A
37A	36A	51A
33A	35A	50A
43A_15C	75A_48C	64A_18C

56A_80C	56A_79C	62A_102C
14A	14A	30A
11C	13C	29C
14A	13A	29A
13C	12C	27C
20A	13A	25A
20C	13C	24C
19A	13A	23A
19C	13C	23C
19A	14A	21A
18C	13C	17C
30A	41A	34A
13A_18C	11A_30C	41C
35A	40A	42A
34C	12A_1T_27C	42C
34A	45A	33A
36C	44C	36C
35A	44A	32A
35C	43C	34C
35A	37A	33A
35C	18A_19C	34C
17A_18C	36A	11A_22C
36C	36C	34C
35A	36A	34A
34C	36C	36C
34A	36A	37A
34C	35C	37C
15A_19C	37A	1A_25T_11C
34C	37C	39C
33A	38A	41A
34C	37C	42C
34G	38G	43G
33G	38G	44G
33C	38C	44C
35A	39A	44A
36G	39G	46G
37G	40G	46G
37A	40A	49A
36G	39G	50G
36T	39T	52T
38T	39T	53T
39C	39C	53C
19A_20C	21A_18C	55C

39T	39T	54T
40T	39T	53T
39T	41T	54T
40A	41A	54A
40A	41A	54A
39C	41C	54C
39T	42T	53T
39C	43C	51C
40A	43A	52A
38T	44T	52T
40T	45T	50T
40C	45C	54C
40T	45T	53T
41C	45C	54C
40G	45G	55G
41G	45G	55G
41C	45C	56C
40A	45A	56A
40G	45G	58G
39G	45G	58G
40G	44G	58G
40C	44C	57C
39C	44C	56C
39C	43C	54C
39T	44T	53T
39T	44T	55T
39T	44T	53T
38T	44T	53T
39A	44A	52A
38C	44C	53C
20T_18C	21T_22C	29T_24C
40G	43G	52G
20T_20C	21T_1G_21C	24T_28C
41G	43G	54G
42C	43C	53C
42A	44A	54A
41A	44A	54A
41A	43A	54A
43G	41G	54G
44C	41C	56C
44A	42A	57A
46G	43G	56G
46G	41G	58G

47A	40A	58A
48G	41G	57G
48G	40G	57G
50A	38A	58A
50T	38T	57T
50G	39G	57G
49G	41G	55G
47G	41G	55G
48T	40T	53T
48T	38T	55T
47G	40G	57G
47G	41G	56G
48A	43A	56A
48A	42A	56A
48A	41A	56A
48G	43G	55G
48G	43G	55G
48A	42A	57A
47A	41A	58A
48G	42G	56G
49A	42A	56A
48G	17A_41G	53G
48A	43A	26A_28G
48A	41A	53A
48A	41A	55A
48A	42A	55A
47A	43A	54A
47G	43G	53G
47G	44G	54G
48A	44A	53A
48C	22T_21C	53C
48G	40G	53G
47A	40A	54A
46A	41A	55A
45A	41A	55A
46C	41C	55C
46A	42A	56A
46A	41A	57A
45G	40G	54G
44T	38T	54T
44C	37C	24G_31C
44T	36T	55T
44T	35T	55T

43T	34T	54T
43G	34G	54G
40C	33C	55C
39C	33C	57C
39A	32A	56A
38A	32A	57A
39G	32G	57G
37G	32G	57G
36A	32A	59A
37A	32A	59A
37A_17T	32A	55A_24T
38T	33T	58T
38T	33T	58T
38T	34T	59T
20A_37T	34T	58T
20A_17T	35A	30A_27T
38A	34A	58A
40A	34A	58A
40A	34A	59A
40A	33A	60A
41A	33A	60A
41C	34C	61C
42A	34A	62A
42C	34C	61C
39T	34T	61T
40C	34C	64C
39C	34C	60C
40T	33T	63T
40C	33C	63C
40T	33T	61T
39T	33T	62T
39T	34T	61T
39A	34A	62A
39T	34T	62T
38A	35A	63A
38A	35A	63A
38T	12T_21G	62T
38G	32G	62G
38T	31T	61T
38C	32C	60C
37A	30A	65A
37A	30A	65A
37G	29G	66G

36A	29A	65A
36G	28G	64G
35A	28A	66A
34T	28T	67T
34G	28G	69G
34G	28G	68G
34C	29C	68C
34C	29C	68C
34A	29A	66A
34C	29C	66C
35A	29A	69A
35C	29C	70C
34C	29C	72C
34T	29T	73T
34T	29T	74T
35T	27T	73T
35G	29G	74G
36T	31T	74T
36G	31G	75G
36G	31G	74G
35C	31C	45A_33C
33T	31T	77T
35G	33G	79G
36A	34A	79A
35T	33T	77T
35G	34G	77G
35G	10A_24G	77G
35G	36G	80G
33A	37A	77A
33T	37T	77T
33T	39T	78T
31T	38T	77T
31G	37G	77G
29C	36C	76C
11T_19C	39T	77T
31C	39C	74C
28C	39C	73C
28C	39C	73C
28A	40A	72A
28C	39C	72C
28C	37C	70C
29G	15A_22G	69G
29G	37G	70G

29A	36A	71A
30T	35T	72T
30C	34C	72C
30G	33G	72G
29A	33A	72A
28G	32G	71G
30G	32G	70G
31G	32G	69G
31A	32A	69A
31G	30G	67G
30G	31G	67G
30G	31G	66G
30C	31C	66C
30T	30T	67T
30T	30T	69T
29T	30T	70T
29C	29C	68C
30T	28T	68T
29A	27A	67A
29C	25C	68C
28G	26G	69G
28G	26G	67G
28C	26C	67C
28T	26T	69T
27T	25T	65T
27C	24C	65C
27C	24C	64C
28A	23A	64A
28A	23A	64A
28A	23A	63A
28G	23G	63G
28G	23G	64G
27G	23G	66G
27G	22G	68G
27A	23A	68A
27A	23A	69A
27C	23C	72C
27A	23A	68A
27G	7A_16G	36A_31G
1A_27G_14C	23G	67G
28C	22C	70C
28C	23C	70C
28C	23C	70C

28C	22C	70C
27A	23A	68A
27T	22T	67T
27G	22G	67G
27A	21A	62A
27T	21T	63T
27G	20G	65G
26G	20G	66G
24C	20C	67C
24A	20A	69A
23G	20G	68G
23G	19G	67G
22T	19T	68T
22G	19G	66G
22A	19A	66A
21A	18A	66A
22G	18G	69G
21C	18C	69C
22G	18G	71G
21T	18T	72T
22T	18T	73T
22G	17G	72G
21A	17A	72A
20T	17T	73T
20G	17G	73G
20G	16G	73G
20A	15A	74A
20C	16C	73C
21G	16G	73G
21C	16C	74C
22T	15T	72T
22C	15C	73C
22C	15C	72C
22A	15A	71A
22T	15T	72T
22G	15G	73G
22C	15C	74C
22G	15G	75G
22C	15C	77C
21A	15A	75A
21G	14G	76G
21A	14A	75A
21C	14C	75C

20T	14T	75T
21G	14G	76G
21A	13A	77A
21G	14G	76G
21T	14T	78T
21T	14T	79T
20G	14G	80G
20G	14G	78G
20T	12T	78T
20T	12T	78T
21G	12G	77G
21C	12C	76C
22T	12T	75T
20G	10G	74G
21A	9A	73A
21C	9C	72C
21T	8T	69T
21G	8G	66G
20C	6C	65C
19T	5T	64T
20A	5A	61A
20G	3G	61G
19G	3G	60G
24A	3A	59A
19A	3A	61A
19C	1C	61C
19A	1A	62A
19C	1C	61C
19A	1A	62A
19C	null	62C
19A	null	62A
18C	null	62C
18A	null	62A
18C	null	58C
18A	1A	56A
17C	1C	56C
17A	1A	53A
17C	1C	52C
17A	1A	53A
17C	1C	52C
15A	1A	52A
13C	1C	48A_5T_75C
14A	1A	31A

14C	1C	31C
14A	1A	37A
14C	1C	40C
13A	1A	43A
15C	1C	43C
15A	2A	44A
17C	2C	44C
18A	2A	44A
18C	2C	44C
17A	2A	44A
17C	2C	44C
17A	2A	45A
17C	1C	47C
17A	2A	47A
17C	2C	47C
17A	1A_1C	47A
18C	2C	47C
17A	null	47A
18A_5T_9C	null	60A_29T_30C
12A	null	3A
12T_4C	null	2T_1C
8C	2G_1C	2A_2C
19T	5T	39T
19C	6C	40C
20T	6T	41T
20C	6C	41C
21T	6T	40T
22T	7T	41T
23T	7T	41T
23T	6T	42T
23C	7C	45C
23T	7T	46T
22A	7A	48A
22G	7G	50G
22A	7A	51A
22C	7C	51C
22T	6T	52T
22T	7T	54T
22A	7A	54A
22A	7A	55A
22A	7A	55A
22A	7A	57A
24C	6C	58C

24C	7C	60T
24G	7G	62G
24T	7T	64T
24A	7A	64A
24G	8A_7G	65G
25A	10A	66A
25A	10A	66A
24A	10A	71A
24A	10A	71A
23A	10A	72A
23A	10A	72A
25T	11T	72T
26G	10G	71G
26T	11T	72T
27A	11A	75A
27A	11A	76A
27T	11T	75T
27C	11C	75C
9T_19C	5T_6C	77C
29A	11A	75A
29G	11G	75G
29G	11G	75G
29A	11A	75A
28G	11G	76G
28C	11C	80C
29C	11C	81C
29C	11C	80C
28C	11C	82C
29A	11A	84A
29T	11T	84T
30T	11T	85T
30C	11C	84C
30C	13C	87C
31G	13G	88G
31A	13A	84A
31A	13A	86A
30C	13C	87C
30C	14C	89C
30C	14C	87C
30G	14G	90G
29T	14T	89T
28G	14G	90G
28G	15G	90G

28C	15C	89C
28A	15A	90A
28A	16A	92A
28T	16T	91T
28T	16T	92T
27T	16T	92T
27C	17C	95C
26C	18C	94C
27G	20G	94G
27A	20A	94A
27A	18A	94A
27C	20C	93C
27C	20C	94C
27A	20A	96A
27C	21C	94C
27C	21C	94C
27T	21T	93T
27T	21T	93T
28C	21C	93C
28A	21A	94A
27A	20A	94A
27G	21G	94G
27G	21G	96G
26G	22G	93G
27C	22C	92C
27A	22A	93A
28G	22G	92G
28C	21C	94C
29C	22C	93C
29C	23C	93C
29C	23C	93C
29A	24A	94A
29G	25G	93G
28G	25G	92G
29G	26G	87G
29G	27G	87G
30A	27A	88A
31C	27C	88C
31A	27A	88A
12A_19G	12T_15G	87G
29G	27G	86G
28G	27G	83G
28A	26A	84A

28C	26C	83C
29G	26G	86G
27G	26G	85G
27C	26C	85C
27T	26T	83T
27C	26C	86C
27C	25C	90C
27G	25G	48A_41G
28T	25T	85T
28T	25T	85T
27A	25A	88A
27C	25C	86C
27A	24A	86A
26G	24G	87G
26T	24T	86T
25A	24A	87A
25G	25G	83G
26T	24T	84T
25C	24C	86C
24T	25T	87T
24A	25A	87A
24T	25T	86T
25G	25G	86G
25C	27C	85C
25T	27T	86T
25C	27C	86C
25G	27G	85G
24G	25G	83G
25G	27G	84G
25G	27G	82G
25C	28C	87C
25T	29T	87T
25T	29T	86T
10A_13G	29G	83G
24A	29A	82A
24A	29A	82A
24T	28T	85T
24C	27C	86C
24A	25A	86A
23G	27G	87G
23G	27G	84G
23G	27G	83G
23A	27A	85A

23G	27G	84G
24G	27G	83G
22T	26T	83T
22G	12A_14G	83G
21A_21T_19G_21C	27G	81G
13A	27A	79A
13A	27A	80A
13C	27C	82C
12T	27T	83T
12C	27C	80C
12T	27T	80T
12C	27C	76C
12T	28T	77T
12G	29G	77G
20T	29T	78T
20T	29T	78T
19T	14T_15C	77C
22A	29A	78A
22A	29A	78A
23A	29A	78A
23G	29G	75C
23G	30G	75G
23A	30A	77A
23C	28C	80C
22C	28C	80C
23A	28A	78A
23G	28G	77G
24C	29C	77C
25T	28T	73T
25T	28T	71T
25C	28C	71C
24A	28A	71A
25C	28C	71C
25A	27A	68A
25A	27A	68A
25G	32A_32T_65G_56C	67G
25A	17A	67A
25G	15G	63G
25G	15G	64G
25C	15T	61C
25G	15G	62G
24G	15G	61G
25C	13C	18G_34C

25T	13T	51T
25T	13T	16A_51T_64C
26C	14C	32C
26A	14A	32A
26C	14C	30C
26T	14C	30T
26C	14C	30C
27C	14T	51C
29A	14A	51A
28G	14G	53G
28A	14A	55A
28T	14T	54T
28T	14C	33T_21C
28G	22G	56G
28A	22A	53A
29A	22A	54A
29G	14T_8G	31G_24C
30G	24G	55G
30G	24G	55G
29G	24G	56G
29T	24T	57T
29C	24C	59C
29A	23A	59A
29G	23G	59G
29T	23T	58T
29C	22C	60C
29C	21C	59C
27C	21C	59C
27T	21T	57T
27C	21C	57C
27T	21T	57T
28C	21C	58C
27A	21A	54A
28C	22C	54C
28C	23C	52C
28C	23C	50C
28A	23A	49A
28G	23G	51G
27A	23A	50A
27C	23C	50C
28C	23C	50C
27C	23C	49C
26C	24C	47C

26A	23A	47A
26C	23C	47C
25G	22G	47G
25C	22C	45C
25C	22C	44C
25A	22A	43A
25A	22A	43A
24C	21C	43C
24G	21G	43G
24G	21G	43G
24C	21C	44C
24T	20T	43T
24G	13G	29G
24C	13C	30C
23C	13C	29C
24C	13C	30C
24C	13C	31C
24C	13C	30C
23C	13C	29C
23A	13A	31A
23G	13G	30G
23C	13C	31C
23G	13G	30G
22G	13G	30G
20T	14T	29T
20T	13T	30T
21C	13C	32C
21T	13T	33T
21G	13G	33G
21C	13C	33C
21A	13A	34A
22T	12T	36T
22T	12T	33T
22C	12C	36C
22T	12T	35T
21A	10A	36A
20G	10G	34G
20C	10C	32C
19T	10T	32T
20C	10C	34C
20T	10T	33T
20G	10G	33G
21C	9C	32C

23C	8C	32C
22C	9C	32C
21G	9G	32G
22C	9C	31C
22C	9C	29T
21T	8T	28T
21C	8C	29C
7T_14C	8C	28C
21C	8C	29C
21C	8C	27C
20C	8C	27C
20T	9T	27T
21C	9C	29C
21C	9C	29C
21T	9T	29T
21C	9C	30C
20A	9A	30A
19A	9A	29A
19G	9G	29G
18G	9G	28G
18A	9A	29A
18G	9G	28G
18A	9A	28A
19G	18G	38G
19C	18C	39C
19T	19T	38T
19A	19A	41A
20G	9G_10C	40G
20A	19A	40A
20A	19A	40A
21G	20G	40G
21G	20G	40G
22A	19A	40A
22G	20G	39G
22A	20A	39A
22G	20G	40G
21G	20G	40G
21C	9T_10C	13T_26C
20C	19C	39C
20T	19T	38T
21T	19T	38T
20T	19T	38T
18G	20G	37G

18T	20T	39T
18G	20G	40G
18G	20G	41G
17C	21C	39C
17A	21A	39A
17T	20T	41T
17A	21A	44A
17G	21G	44G
16C	21C	46C
16C	11T_10C	46C
16G	20G	43G
16T	19T	44T
16T	19T	43T
16G	19G	44G
16G	19G	46G
16A	19A	43A
16G	19G	46G
16A	17A	45A
17G	17G	45G
17C	17C	46C
19T	17T	47T
18G	17G	47G
18G	17G	46G
18G	17G	44G
18C	16C	46C
18T	15T	47T
18G	14G	47G
18A	14A	48A
18G	14G	48G
18A	14A	50A
18C	14C	50C
17T	14T	50T
17G	14G	50G
17G	12G	51G
17A	13A	50A
17G	13G	49G
17A	13A	51A
17G	13G	51G
16G	13G	51G
17G	13G	50G
17T	13T	49T
17C	13C	49C
17C	13C	48C

17A	13A	48A
17G	13G	32G_16C
18G	13G	47G
18T	13T	47T
18T	12T	48T
18T	13T	47T
18C	13C	49C
18T	12T	49T
18A	12A	50A
18G	11G	51G
18T	13T	50T
18C	13C	52C
18C	13C	52C
18T	12T	52T
18C	13C	52C
18A	12A	52A
18T	12T	52T
17G	12G	52G
17G	12G	54G
17A	10A	53A
16G	12G	53G
16G	12G	53G
16C	12C	53C
16C	12C	52C
17A	12A	53A
17A	12A	53A
17C	12C	53C
18T	12T	52T
13A_5G	5A_7G	51A
18C	12C	49C
18A	12A	49A
18C	12C	49C
18T	12T	47T
18T	12T	45T
15T	10T	45T
16G	10G	43G
16G	10G	43G
13G	9G	43G
13G	9G	42G
13T	8T	45T
13G	9G	44G
13G	9G	41G
12T	9T	41T

12G	8G	39G
12G	8G	39G
12A	6A	38A
12A	6A	37A
12A	6A	36A
11G	6G	35G
12G	6G	35G
9A_4G	6A	28A_7G
13G	6G	34G
13G	6G	31G
13G	6G	32G
13G	6G	31G
12C	6C	32C
12G	6G	30G
11T	6T	29T
11C	6C	28C
11C	5C	27C
11C	5C	26C
10T	5T	26T
10C	5C	26C
9A	3A_2C	24A
9C	4C	23C
9C	4C	23C
8C	4C	23C
8T	4T	24T
8T	3T	23T
7T	4T	24T
8C	4C	24C
8C	4C	24C
8C	4C	24C
8T	4T	23T
7C	4C	24C
7T	4T	24T
6A	3A	23A
7C	3C	23C
6C	3C	23C
7C	3C	22C
5A_2C	2A	21A
7C	2C	19C
7C	2C	19C
7C	2C	20C
7C	2C	20C
7T	2T	20T

7C	2C	20C
7C	2C	20C
7T	2T	18T
7C	2C	17C
7C	2C	17C
7A	2A	17A
6A	2A	17C
7C	2C	16C
8C	2C	16C
7A	2A	17A
7C	2C	17C
5C	2C	14C
5C	1C	15C
5A	2A	15A
5C	2C	15C
5C	2C	15C
5C	2C	15C
5A	1A_1C	14A
4T_2C	2C	13C
6C	2C	13C
6C	2C	13C
4A_2G	2A	3A_9G
6G	1G	9G_3C
6C	1C	11C
6C	1C	5G_7C
6A	1A	12A
6G	1G	12G
6C	2C	12C
5A	2A	12A
6C	2C	12C
6C	2C	11C
6C	3C	11C
8C	3C	12C
8T	3T	12T
8C	4C	12C
8C	4C	12C
8C	4C	12C
8C	4C	11C
8C	4C	11C
8T	4T	11T
8T	4C	11C
8C	4C	11C
8C	4C	11C

8T	4T	11T
9C	3C	11C
9T	3T	11T
9C	3C	11C
9A	4A	12A
9C	4C	12C
9C	4C	12C
9T	4T	12T
9C	4C	11C
9C	3C	11C
9A	4A	11A
9G	4G	11G
9C	4C	11C
9T	4T	10T
9C	4C	10C
8C	5C	10C
8T	5T	10T
10T	5T	10T
9G	5G	10G
9A	6A	8A
9A	5A	8A
9A	6A	9A
9T	6T	9T
9A	6A	9A
8A	6A	9A
8A	6A	10A
8T	6T	10T
8G	6G	10G
8C	6C	12C
9A	6A	12A
9G	6G	12G
9C	6C	13C
9T	6T	14T
9T	6T	14T
8C	6C	15C
9G	6G	14G
9G	6G	15G
9G	6G	15G
9C	6C	15C
9C	6C	15C
9C	6C	15C
9A	7A	15A
9C	8C	15C

9T	8T	15T
9C	8C	15C
9C	8C	15C
9A	8A	15A
9C	8C	15C
9A	8A	15A
9A	7A	15A
9T	8T	16T
9G	8G	15G
9G	8G	15G
9C	8C	16C
9G	8G	17G
9A	8A	16A
9A	8A	18A
9G	8G	18G
8A	8A	19A
8G	8G	19G
8G	8G	20G
8G	8G	20G
8T	8T	19T
8C	8C	20C
8T	8T	21T
7G	8G	21G
7G	8G	21G
7T	8T	21T
7C	8C	22C
8G	8G	22G
8G	8G	22G
8C	8C	23C
8C	8C	23C
7A	8A	23A
7T	9T	22T
7C	9C	22C
8C	8C	22C
8G	9G	21G
8G	9G	23G
10C	9C	24C
10A	9A	23A
10C	9C	23C
10A	9A	25A
10T	9T	25T
10G	9G	25G
10A	9A	25A

10G	9G	26G
10C	10C	26C
11C	10C	28C
11C	10C	28C
6A_5G	12G	19A_9G
11A	12A	27A
11A	12A	26A
11G	12G	27G
11G	12G	25G
11G	12G	25G
10G	12G	25G
11C	12C	25C
11T	12T	23T
11G	12G	23G
11A	12A	22A
12G	12G	22G
12C	12C	21C
12T	12T	22T
12T	11T	21T
12C	12C	21C
12T	12T	20T
12C	12C	20C
11G	12G	21G
10T	12T	21T
11G	11G	22G
11G	12G	23G
11C	12C	23C
11G	12G	23G
11G	13G	23G
11C	13C	23C
11C	13C	23C
11C	13C	23C
11T	13T	22T
11T	13T	24T
12G	13G	22G
12C	13C	22C
12T	13T	22T
13T	13T	21T
13T	12T	20T
14G	12G	20G
14C	12C	20C
14T	12T	20T
14C	12C	19C

14C	12C	18C
14T	12T	17T
14G	12G	17G
14C	12C	17C
14T	12T	17T
14G	12G	16G
14C	13C	16C
14A	13A	15A
13G	13G	15G
13G	13G	15G
14C	12C	15C
14A	12A	15A
13G	12G	15G
13G	12G	15G
13C	12C	16C
13C	12C	16C
13A	12A	17A
13G	12G	17G
13G	12G	17G
13A	12A	16A
12T	10T	15T
12G	11G	15G
13C	11C	15C
13G	11G	14G
13G	11G	14G
13C	11C	14C
13C	11C	14C
13C	11C	13C
13T	11T	13T
13T	11T	11T
13G	11G	11G
13A	11A	13A
12C	10C	13C
12C	9C	13C
12T	9T	13T
12G	9G	13G
12C	9C	14C
12G	9G	14G
12C	9C	14C
12C	9C	14C
12T	8T	14T
11C	9C	14C
11G	9G	14G

11T	9T	14T
11C	9C	13C
11C	9C	14C
11G	9G	14G
11G	9G	14G
11C	9C	14C
11T	9T	13T
11C	9C	13C
11C	9C	12C
11A	9A	11A
11G	9G	11G
11C	9C	11C
10T	9T	11T
10G	9G	11G
10C	9C	11C
10A	8A	11A
10G	9G	11G
9C	9C	10C
9A	9A	10A
9G	9G	10G
9C	9C	10C
9T	9T	11T
9T	8T	11T
9G	7G	11G
8A	7A	11A
8G	7G	11G
8A	7A	11A
8A	7A	11A
8T	7T	11T
8C	7C	11C
8A	7A	11A
8C	7C	11C
8G	7G	11G
8T	7T	11T
8C	6C	11C
7G	6G	11G
7G	6G	10G
7G	5G	11G
6G	5G	10G
6A	5A	11A
6T	5T	11T
6G	5G	10G
6T	4T_1C	10T

6C	5C	9C
6C	5C	9C
6G	5G	9G
6G	5G	9G
6C	5C	9C
5G	5G	9G
4C	5C	8C
3T_1C	5C	8C
4G	5G	8G
4T	5T	8T
4C	5C	9C
4G	5G	9G
4T	5T	9T
4G	4G	9G
4G	4G	11G
3T	4T	9T
3T	3T	9T
3G	3G	9G
3G	3G	9G
3G	2G	9G
3G	2G	8G
3A	2A	8A
3C	2C	14C
3C	2C	8C
3C	2C	8C
2T	2T	7T
2C	2C	9C
2C	1C	9C
2T	2T	9T
2C	1C	8C
1C	2C	8C
1T	2T	7T
1C	2C	7C
1C	2C	7C
1T	2T	7T
null	2C	7C
null	2C	7C
null	2A	3A_4T
1C	1C	7C
1C	1C	7C
1A	null	7A
1C	null	8C
1C	null	8C

1A	null	5A_3C
1C	null	8C
1C	null	8C
1A	null	8A
1C	null	9C
1C	null	9C
1C	null	9C
1C	null	9C
1C	null	8C
1T	null	8T
1C	null	8C
null	null	8C
null	null	8A
null	null	8C
null	null	8C
null	null	8C
null	null	8C
null	null	9C
null	null	2A_7T
null	null	9C
null	null	9C
null	null	9G
null	null	9C
null	null	9C
null	null	10C
null	null	10C
null	null	10C
null	null	10T
null	null	10C
null	null	10C
null	null	11A
null	null	13C
null	null	13C
null	null	12C
null	null	13C
null	null	13C
1A	null	13A
1C	null	13C
1T	null	13T
1G	null	11A_3G
1C	null	15C
1C	null	15C
2G	null	15G

2C	null	15C
2C	null	15C
2C	null	15C
2C	null	15C
2C	null	15C
2A	null	14A
2C	null	15C
2T	null	15T
2G	null	15G
2C	null	17C
2C	1C	17C
2C	1C	17C
2C	1C	18C
2C	1C	18C
2T	2T	18T
2C	2C	18C
2C	2C	18C
3G	2G	17G
3C	2C	19C
3C	2C	19C
3T	2T	19T
3G	2G	20G
3G	2G	20G
3G	2A_1G	20G
3T	3T	20T
3A	3A	19A
3A	3A	20A
3G	3G	21G
3C	3C	21C
3C	3C	21C
4T	4T	21T
4C	4C	20C
4G	2A_2G	21G
4G	4G	21G
4G	4G	21G
4A	4A	21A
4T	4T	21T
4A	4A	21A
3T_1C	2T_2C	21C
4G	4G	21G
4G	4G	21G
1A_3G	2A_2G	9A_11G
4T	4T	20T

4A	4A	20A
4A	2A_2G	9A_10G
5G	5G	18G
5C	5C	19C
5A	5A	9A_10G
5G	5G	19G
5G	5G	18G
5T	5T	7T_11G
5G	5G	18G
5C	5C	18C
5C	5C	18C
5G	5G	17G
5C	5C	18C
6C	5C	18C
6T	6T	18T
6C	6C	16C
6G	6G	16G
6T	6T	16T
6G	6G	17G
3G_3C	4A_2C	17C
6G	6G	17G
6G	6G	17G
6A	7A	17A
6T	7T	16T
6G	7G	15G
6C	7C	14C
6G	7G	14G
6G	7G	14G
6C	7C	14C
7C	7C	15C
7C	7C	15C
7A	7A	15A
7T	7T	15T
7A	7A	15A
7T	3T_4C	15T
7G	7G	15G
7G	7G	13G
7G	7G	12G
7T	7T	12T
7C	7C	11C
7T	7T	11T
7G	7G	11G
7G	7G	11G

7A	6A	11A
7A	7A	11A
7A	8A	10A
7C	8C	10C
7T	8T	10T
7C	8C	9C
7A	8A	9A
6G	8G	11G
6A	8A	12A
6C	8C	12C
6T	8T	11T
6T	8T	11T
6G	8G	12G
6A	8A	12A
6T	8T	12T
6G	8G	12G
6G	8G	12G
6C	8C	13C
6C	8C	13C
6C	8C	13C
6G	8G	13G
6G	8G	13G
6C	8C	13C
6T	9T	13T
7A	9A	13A
6G	9G	12G
8T	9T	12T
7G	9G	12G
7A	9A	12A
7A	10A	12A
7G	10G	11G
7G	10G	11G
7G	11G	11G
6G	11G	11G
6G	11G	11G
6G	11G	11G
6G	11G	11G
6T	9T	9T
6A	11A	13A
6G	12G	14G
6T	11T	14T
6G	12G	13G
6G	11G	14G

6T	11T	14T
6A	11A	14A
6G	11G	13G
5C	11C	13C
6C	11C	13C
6C	10C	13C
6G	10G	13G
6A	10A	13A
5G	10G	13G
5A	10A	11A
5G	10G	13G
5G	10G	13G
5G	10G	13G
5C	9C	12C
5C	9C	12C
5C	9C	13C
5C	10C	13C
5A	10A	7A_6G
5T	10T	14T
6G	10G	14G
6G	10G	14G
6C	9C	15C
6C	9C	16C
6C	9C	15C
6G	9G	17G
6C	9C	17C
6G	8G	17G
6G	8G	17G
6C	7C	17C
6G	7G	17G
6G	6G	17G
6C	7C	17C
6G	7G	18G
6G	6G	18G
6G	6G	17G
6G	6G	16G
5A	7A	14A
5C	7C	16C
2A_3G	3A_4G	3A_12G
5G	7G	14G
5G	7G	15G
5C	7C	15C
5A	7A	15A

5C	7C	14C
5G	3A_4G	13G
5G	7G	13G
5C	7C	13C
4C	7C	14C
4A	8A	13A
4G	8G	16G
4G	8G	17G
4G	8G	17G
4C	8C	18C
4G	8G	18G
4G	8G	18G
5G	8G	18G
5G	7G	18G
5G	7G	20G
5T	8T	17T
5C	8C	20C
5C	8C	20C
5C	8C	20C
5G	8G	21G
4T	8T	21T
4A	8A	20A
4G	8G	19G
4G	8G	18G
4G	8G	18G
1A_3G	8G	18G
4C	8C	17C
4T	8T	17T
4G	8G	17G
4C	9C	17C
4G	9G	15G
4C	9C	15C
4T	9T	16T
4C	9C	16C
3A_1G	5A_4G	11A_5G
4T	9T	16T
4A	8A	16A
4G	8G	17G
4T	9T	18T
4C	9C	18C
4C	9C	18C
4A	9A	20A
4C	9C	19C

4A	4A_5G	19A
4G	9G	19G
4G	9G	19G
4G	9G	19G
4G	9G	19G
4T	9T	18T
4C	9C	17C
4A	8A	17A
4T	9T	16T
4G	9G	16G
4G	9G	16G
4C	9C	16C
1A_3G	9G	10A_6G
4C	9C	16C
4C	8C	16C
3G	8G	15G
3C	9C	15C
2T	9T	16T
2G	8G	15G
2C	9C	16C
2T	7T	17T
2A	3A_5C	17C
2C	8C	18C
2C	6C	18C
2A	7A	10A_7G
2C	7C	18C
2T	7T	19T
2G	7G	7A_13G
2C	7C	21C
2C	7C	22C
2C	7C	22C
2C	7C	22C
2C	7C	23C
2T	7T	23T
2G	7G	22G
2C	7C	22C
2T	3T_4C	22C
2G	7G	22G
2G	7G	22G
2A	7A	20A
2G	7G	22G
2G	8G	20G
2G	7G	20G

2A	8A	20A
2G	8G	21G
2G	8G	21G
2C	8C	20C
2C	8C	20C
2C	8C	20C
2T	8T	20T
2T	7T	20T
2G	6G	20G
2G	7G	18G
1C	7C	20C
1T	7T	16T_4C
1C	7C	20C
1C	7C	20C
1C	7C	20C
1A	7A	19A
1C	7C	18C
1C	7C	18C
1C	7C	18C
1C	7C	17C
1G	7G	18G
1G	8G	17G
1G	8G	16G
1G	8G	16G
1T	8T	16T
1G	8G	14G
2G	8G	13G
2T	7T	13T
2G	7G	13G
2G	7G	13G
1T_1G	7T	13T
2G	7G	13G
2G	7G	14G
2G	7G	14G
2G	7G	14G
2A	7A	14A
2T	7T	14T
2G	7G	15G
2G	7G	15G
2G	6G	15G
3G	7G	15G
2A	7A	15A
2T	8T	15T

2G	8G	15G
2A	8A	14A
2T	8T	14T
2G	8G	13G
2G	8G	12G
2T	8T	12T
2G	7G	11G
2G	7G	11G
2T	7T	11T
2G	7G	11G
3G	6G	10G
3T	7T	9T
3G	7G	8G
1A_2G	7G	8G
3T	7T	7T
3G	7G	9G
3G	7G	8G
3T	7T	7T
3G	7G	9G
3G	7G	8G
3T	6T	5T_3G
3G	6G	11G
3G	6G	12G
3G	6G	13G
3G	6G	12G
3A	6A	13A
3T	6T	12T
3G	5G	13G
3A	5A	12A
3T	4T	13T
2G	4G	15G
2G	4G	15G
2T	4T	16T
2G	4G	16G
2G	4G	16G
2T	4T	16T
2G	4G	17G
2G	4G	17G
2T	3T	17T
2G	4G	17G
2G	4G	17G
2T	4T	17T
2G	4G	17G

1G	4G	17G
1T	4T	17T
1G	4G	17G
1A	4A	17A
1G	4G	18G
1C	4C	18C
1C	3C	18C
1A	3A	18A
1G	3G	18G
1G	3G	18G
1C	3C	18C
1C	3C	18C
1G	3G	18G
1T	3T	18T
1G	3G	19G
1G	3G	19G
1A	3A	18A
1G	3G	18G
1C	3C	18C
1G	3G	18G
1A	3A	17A
1G	3G	17G
1G	3G	17G
1C	3C	17C
1C	3C	17C
2G	3G	17G
2G	3G	15G
2C	3C	15C
2A	3A	15A
2G	3G	15G
2G	3G	14G
2C	3C	14C
2T	2T	13T
2C	2C	13C
2C	2C	13C
2C	2C	13C
2A	2A	13A
2T	2T	13T
2A	2A	13A
2G	2G	12G
2T	2T	11T
2C	2C	11C
2C	2C	9C

2C	2C	9C
2C	2C	9C
2T	2T	8T
2G	2G	8G
2C	2C	8C
2C	2C	8C
2T	2T	7T
2G	2G	6G
2G	2G	4G
2A	2A	4A
2C	2C	4C
2G	1G	4G
2G	1G	4G
2G	1G	4G
2G	1G	4G
2G	1G	4G
2A	1A	3A
1C	1C	3C
1A	1A	3A
1C	1C	3C
1G	1G	2A
1A	1A	2A
1T	1T	2T
1G	1G	2G
1G	1G	2G
1G	1G	2G
1G	1G	2G
1G	1G	2G
1G	1G	2G
1C	1C	2C
1G	1G	2G
1G	1G	2G
1A	1A	2A
1G	null	2G
1G	null	2G
1A	null	2A
null	null	2G
null	null	2G
null	null	2A
null	null	2G
null	null	2G
null	null	1G
null	null	1G

null	null	1G
null	null	2C
null	null	2G
null	null	2G
null	null	2T
null	null	3G
null	null	3G
null	null	4G
null	null	4G
null	null	5G
null	null	5C
null	null	5G
null	null	7G
null	null	7G
null	null	8G
null	null	8G
null	null	8C
null	null	8G
1G	null	8G
1G	null	8G
1G	null	8G
2G	null	7G
2G	null	8G
2C	null	8C
2C	null	8C
2C	null	8C
2G	null	8G
2T	1T	8T
2C	1C	9C
2G	1G	9G
2C	1C	9C
2G	1G	9G
2G	1G	10G
2G	1G	10G
2G	1G	10G
2G	1G	10G
2C	2C	10C
null	1T_1G_2C	null
null	1G	null
null	1C	2C
2C	2C	13C
2G	2G	14G
2T	2T	15T

2C	3C	15C
3T	3T	16T
3C	3C	18C
3C	2C	19C
4A	3A	21A
5G	3G	21G
5C	3C	21C
6T	3T	24T
6T	4T	25T
6G	4G	25G
6A	4A	27A
5A	4A	28A
6C	4C	29C
6C	4C	29C
6C	4C	30C
7G	4G	12A_20G
7T	4T	32T
7T	4T	33T
7G	4G	32G
7G	4G	32G
7C	4C	32C
7G	4G	32G
7C	4C	32C
7G	4G	32G
7G	4G	33G
7A	5A	33A
7T	5T	33T
9C	5C	33C
9A	6A	33A
9G	6G	34G
9G	6G	34G
9G	6G	34G
9C	6C	37C
10C	6C	38C
11T	6T	39T
11T	6T	38T
11C	6C	39C
11T	6T	39T
11T	6T	40T
11C	6C	40C
11T	6T	41T
11G	6G	42G
11C	6C	41C

11T	6T	42T
11G	6G	41G
11C	6C	43C
11T	6T	43T
11T	6T	44T
11C	7C	45C
11A	7A	45A
11G	8G	45G
11G	8G	45G
11G	8G	45G
11C	8C	45C
11G	8G	46G
11C	9C	47C
11G	9G	48G
12G	9G	49G
14T	9T	48T
14C	10C	49C
13C	10C	50C
14C	9C	51C
14G	10G	52G
14C	10C	53C
14T	10T	54T
14T	12T	55T
14G	13G	55G
14T	13T	57T
15A	13A	56A
15C	13C	57C
15A	13A	58A
15T	14T	57T
15C	15C	56C
15G	16G	55G
16G	15G	58G
16T	17T	59T
16C	17C	59C
16C	17C	57C
16G	17A	56G
15A	18A	55A
16A	18A	55A
16C	11T_7C	55C
16T	18T	54T
16T	18T	54T
16G	19G	53G
16T	21T	51T

16T	21T	52T
16G	21G	54G
17C	21C	54C
17G	20G	54G
17T	22T	54T
19C	22C	55C
19C	22C	54C
20G	23G	55G
21C	23C	54C
21C	23C	55C
21T	23T	55T
21C	23C	57C
19T	23T	57T
20C	24C	58C
20A	25A	59A
19T	25T	59T
19C	25C	58C
19C	25C	57C
19G	25G	56G
20G	25G	57G
20T	25T	55T
20C	25C	54C
20T	26T	55T
20G	26G	57G
19C	26C	57C
19G	27G	56G
20C	27C	56C
20G	27G	55G
20C	27C	56C
20A	27A	54A
20C	27C	54C
20G	27G	54G
20G	28G	54G
20C	28C	54C
20T	28T	55T
19G	28G	55G
19G	28G	54G
20A	29A	54A
19G	29G	56G
17G	29G	55G
17A	28A	56A
16G	29G	56G
16A	28A	57A

17G	28G	56G
17G	28G	55G
17A	28A	53A
17G	28G	55G
17A	28A	54A
17G	28G	53G
16A	28A	52A
16A	28A	51A
17G	28G	48G
17G	12A_16G	20A_28G
17G	28G	47G
17A	28A	47A
17A	27A	47A
17G	28G	46G
17A	29A	46A
17A	29A	41A
17G	28G	40G
17T	16T_11C	22T_17C
8T_7C	27C	17A_22C
16G	26G	41G
16G	27G	42G
16G	27G	42G
16G	27G	43G
16A	27A	44A
15G	27G	46G
14A	27A	48A
14A	27A	48A
14A	27A	46A
14G	27G	44G
14C	27C	43C
14A	28A	45A
14G	29G	46G
13A	28A	46A
14C	29C	48C
14C	27C	50C
14C	29C	53C
16T	29C	28T_25C
16T	29T	53T
16G	29G	52G
17G	28G	54G
19C	27C	52C
19G	27G	53G
20T	27T	54T

20C	27C	53C
20A	27A	56A
20G	28G	56G
20G	12A_15G	55G
20C	27C	56C
20C	27C	57C
18C	27C	57C
12A_7C	14A_12C	25A_28C
19G	27G	53G
19G	27G	53G
19G	28G	54G
20G	28G	56G
20T	23T	56T
20C	27C	57C
20T	27T	58T
20G	28G	58G
20T	28T	57T
20T	28T	57T
20C	28C	56C
20A	27A	57A
20C	26C	58C
20A	25A	58A
19C	25C	57C
19A	25A	58A
19A	25A	57A
19G	25G	56G
19C	25C	56C
19T	24T	56T
19G	24G	57G
33G	24G	59G
12G	24G	58G
11C	24G	31G_30C
11A	49A_78G_39C	62A
20G	8G	64G
20G	8G	63G
20C	8C	65C
20A	8A	65A
21G	8G	64G
21G	7G	64G
19C	7C	65C
19A	7A	66A
18G	6G	69G
17G	6G	70G

17C	6C	70C
17A	24A	70A
17G	24G	34A_36G
17G	19G	70G
17C	21C	70C
17A	20A	71A
17G	21G	71G
17G	22G	70G
17C	22C	71C
17A	22A	68A
17G	22G	72G
17G	22G	72G
17C	24C	73C
17A	24A	72A
17G	24G	75G
17C	24C	74C
17C	25C	71C
17T	25T	74T
17C	14T_11C	72C
18G	25G	69G
19G	25G	69G
18T	25T	70T
18G	25G	70G
18G	26G	70G
18G	26G	71G
18T	26T	68T
18T	26T	67T
18G	26G	66G
17G	27G	63G
17A	28A	64A
17G	28G	64G
17G	28G	63G
19C	28C	62C
19A	27A	61A
19G	28G	63G
19G	28G	64G
19A	28A	65A
19G	27G	64G
20G	27G	61G
20A	27A	61A
20G	26G	61G
21A	26A	60A
20C	26C	61C

21T	26T	62T
21T	26T	64T
21G	27G	65G
21G	27G	65G
21A	27A	67A
22A	27A	71A
22A	27A	70A
22A	27A	68A
22G	27G	71G
22G	28G	72G
23A	28A	70A
23T	28T	75T
23G	28G	37A_38G
23C	27C	75C
23T	28T	74T
23C	28C	73C
24C	28C	72C
27G	27G	71G
28T	26T	66T
29C	27C	72C
30T	29T	70T
31C	30C	74C
31C	30C	75C
31G	29G	76G
31G	30G	74G
31T	30T	71T
31T	30T	73T
30G	30G	75G
30C	30C	76C
30T	30T	36T_40C
31A	29A	76A
29G	30G	76G
29G	30G	78G
29C	30C	78C
28A	29A	80A
27A	29A	75A
27C	29C	79C
26C	29C	76C
26C	29C	77C
26C	30C	76C
26T	31T	73T
25C	31C	73C
26C	31C	73C

26C	32C	72C
26A	33A	74A
26A	34A	74A
26C	35C	75C
26C	35C	75C
26C	34C	76C
25C	33C	78C
23A	33A	75A
24T	33T	74T
24G	33G	75G
24C	32C	75C
23A	33A	76A
25G	32G	76G
25C	32C	75C
25C	32C	79C
25C	33C	79C
25T	31T	79T
25G	30G	81G
25G	30G	81G
24G	30G	82G
24G	30G	83G
24G	31G	83G
22A	30A	83A
23G	30G	84G
24G	30G	82G
24T	29T	80T
24C	30C	81C
24T	30T	80T
23C	29C	79C
23T	29T	81T
25T	30T	80T
25A	29A	76A
26G	30G	75G
26C	32C	76C
28A	31A	75A
29A	32A	73A
31C	32C	72C
31T	31T	70T
31T	31T	69T
31G	30G	69G
31G	31G	68G
31G	30G	68G
31G	30G	68G

31G	30G	69G
31A	28A	69A
35G	31G	34G_35C
36T	28T	70T
36G	29G	70G
36C	29C	68C
36T	29T	67T
36T	28T	64T
36C	28C	64C
36C	28C	65C
34T	29T	65T
33T	29T	63T
34T	29T	64T
33C	15T_1G_13C	63T
34G	13G_15C	31G_33C
34G	28G	62G
34C	28C	61C
35C	28C	60C
39C	28C	58C
39C	27C	58C
39T	26T	56T
39C	26C	57C
40T	26T	58T
24A_16G	26A	58A
41A	27A	59A
41G	27G	63G
42A	26A	62A
42T	27T	64T
43G	27G	61G
43A	28A	63A
44T	28T	62T
44C	28C	61C
43C	28C	58C
43T	28T	57T
43T	28T	58T
43G	28G	59G
43C	27C	60C
43A	27A	58A
43C	27C	61C
42T	28T	63T
43T	28T	65T
26T_18C	28C	29T_33C
45G	28G	62G

45G	27G	61G
44T	28T	61T
44G	28G	59G
46A	28A	60A
46G	28G	59G
46T	29T	58T
46T	29T	57T
46T	29T	58T
43C	29C	58C
43G	29G	58G
42G	29G	60G
40T	27T	60T
40G	26G	59G
40A	26A	57A
39A	27A	57A
41A	26A	57A
41A	26A	56A
40G	27G	55G
41T	27T	56T
41T	28T	55T
41C	27C	56C
40C	28C	57C
42T	29T	57T
42T	29T	57T
43C	29C	57C
43C	30C	28T_28C
43G	31G	56G
43G	29G	58G
43G	31G	57G
43A	31A	55A
43C	30C	55C
44C	30C	56C
44T	30T	55T
43T	30T	57T
43C	28C	57C
43T	28T	57T
43G	27G	56G
44C	26C	24T_56C
44T	26T	57T
44C	26C	55C
45C	26C	58C
45C	26C	59C
46A	29A	59A

48G	29G	61G
48A	29A	59A
48A	29A	63A
47T	29T	63T
47T	30T	66T
47C	30C	63C
48C	29C	63C
48C	29C	64C
48C	29C	63C
48A	30A	61A
49G	30G	61G
49C	32C	61C
49C	30C	61C
49A	32A	61A
49G	32G	61G
48G	31G	63G
47G	32G	62G
46T	32T	62T
47C	32C	62C
47A	32A	62A
47C	32C	63C
45C	32C	63C
45T	32T	62T
44G	33G	61G
44T	31T	61T
42G	33G	61G
41G	32G	61G
39G	32G	60G
40G	33G	60G
40C	32C	58C
41A	33A	59A
42T	33T	59T
18T_24C	14T_19C	59T
42C	33C	60C
42T	33T	30T_1G_28C
41C	33C	59C
37G	31G	59G
36G	32G	59G
36G	33G	59G
36G	32G	62G
37T	33T	62T
37C	33C	63C
37A	33A	65A

37T	33T	63T
38A	32A	66A
25T_14C	19T_13C	67T
39G	32G	69G
39C	32C	70C
40C	32C	70C
40C	32C	29A_67C
39A	32A	35A_35C
40A	32A	70A
36A	31A	70A
36A	32A	69A
36A	32A	68A
36A	32A	69A
31A	32A	71A
34G	31G	69G
33G	16T_13G_1C	34T_36G
32A	30A	70A
31A	29A	71A
33C	30C	72C
35T	29T	72T
34C	29C	73C
34G	29G	74G
33G	29G	75G
33C	30C	73C
33C	31C	72C
34A	31A	72A
35A	31A	73A
35G	31G	72G
36C	31C	71C
36C	31C	72C
36C	30C	71C
37T	31T	69T
36G	32G	71G
35C	31C	71C
34G	32G	70G
35A	31A	72A
35G	31G	72G
37G	31G	71G
35T	32T	73T
35T	32T	73T
34G	32G	76G
33G	32G	78G
33C	32C	77C

33C	32C	78C
34A	32A	80A
35G	33G	80G
35A	33A	83A
36A	34A	82A
36G	33G	82G
19T_17G	16T_18G	79G
35T	33T	78T
36T	33T	79T
36T	36T	79T
37T	36T	81T
38T	35T	83T
39C	35C	85C
39T	35T	86T
37C	35C	87C
37T	35T	87T
36G	34G	87G
36C	33C	86C
36A	33A	86A
36T	33T	89T
36C	33C	88C
36A	35A	89A
35G	35G	89G
35G	35G	84G
35G	35G	85G
36T	34T	83T
36C	36C	84C
36A	35A	84A
36G	36G	81G
37A	36A	82A
37G	36G	80G
37G	36G	81G
37G	36G	81G
37A	35A	82A
37C	35C	82C
36G	35G	81G
37G	35G	81G
37A	36A	79A
38C	35C	79C
38T	35T	79T
38C	35C	77C
37A	35A	75A
37T	35T	73T

37C	37C	31A_41C
37A	35A	72A
36G	17A_17G	72G
37C	33C	71C
37C	34C	70C
37C	34C	70C
37T	34T	68T
37T	33T	68T
37A	33A	68A
37A	34A	67A
37A	34A	68A
37C	34C	68C
20T_17C	17T_17C	68C
38C	33C	67C
38T	33T	68T
38G	31G	66G
38T	30T	67T
38A	31A	68A
38C	31C	67C
38A	30A	66A
37A	30A	68A
38A	32A	68A
37T	28T	65T
36T	30T	64T
36T	30T	62T
36G	29G	60G
36G	29G	63G
36A	29A	65A
36C	28C	63C
35T	29T	65T
34T	29T	64T
33C	30C	63C
34T	30T	63T
34C	12T_17C	65C
34C	29C	65C
34C	29C	65C
33C	29C	65C
32C	29C	63C
32C	29C	61C
33T	30T	61T
33C	32C	60C
33A	32A	57A
18A_15C	32C	57C

33C	32C	58C
34A	31A	59A
34C	34C	58C
34C	35C	56C
34A	35A	57A
33T	35T	57T
33T	16T_20C	57T
34C	36C	57C
34T	36T	57T
33G	36G	55G
31C	35C	57C
31C	35C	58C
31C	35C	56C
31A	36A	56A
31A	36A	56A
31A	35A	55A
29C	35C	53C
28C	35C	52C
28C	16T_19C	50C
27T	36T	51T
27C	36C	51C
27T	36T	52T
27A	36A	55A
28T	35T	55T
28G	35G	56G
28T	35T	56T
27G	35G	57G
28G	35G	57G
26G	35G	58G
25T	35T	58T
26C	34C	57C
26T	34T	56T
26C	36C	57C
27C	36C	59C
27C	36C	61C
26T	36T	61T
25A	35A	61A
25T	35T	59T
24A	34A	59A
25T	34T	58T
25G	33G	57G
25G	34G	57G
24G	34G	58G

24G	31G	58G
23G	31G	55G
23G	31G	54G
22C	31C	54C
22A	31A	52A
23T	31T	53T
23C	31C	52C
23A	31A	30A_22G
23G	31G	52G
23A	31A	50A
23A	30A	46A
23G	31G	45G
23G	30G	44G
23A	30A	40A
23A	29A	39A
23G_54C	11T_8C	16T_15C
5G	19G	30G
6C	47C	74C
14C	4A_4C	13C
8G_5C	3A_1G_5C	18G_25C
13C	9C	15C
21C	9C	15C
8A_13C	20C	28C
22C	20C	29C
22C	20C	29C
22C	20C	29C
20C	20C	29C
20C	11T_9C	16T_13C
20C	20C	25C
22G	20G	27G
22T	20T	27T
23T	20T	27T
23G	9G_11C	11G_17C
23G	22G	39G
23A	22A	40A
23C	21C	41C
23A	21A	42A
23T	13A_8T	42T
23C	25C	42C
24A	25A	44A
25G	25G	46G
25G	25G	47G
26C	25C	48C

27C	25C	51C
27C	25C	51T
28C	25C	51C
28C	25C	51C
28T	24T	52T
28C	25C	53C
28A	25A	55A
27A	9A_17G	16A_41G
28G	26G	58G
28C	26C	59C
28C	26C	59C
29C	26C	60C
30T	25T	59T
31T	27T	60T
31A	26A	60A
31A	27A	59A
31A	11A_17C	40A_21C
31C	28C	61C
30A	28A	61A
30A	28A	61A
29A	30A	60A
30G	31G	59G
30G	32G	59G
29C	31C	59C
30A	31A	61A
30G	30G	61G
30T	30T	62T
30T	30T	61T
30T	28T	62T
31C	30C	62C
31C	20T_11C	62C
31A	32A	65A
31A	32A	66A
31A	31A	67A
30C	32C	67C
30C	31C	67C
28A	26A	69A
30C	27C	70C
29A	26A	69A
30T	25T	70T
30T	25T	69T
30C	26C	71C
31A	26A	73A

33A	25A	72A
32G	27G	72G
31G	29G	72G
31G	29G	72G
30C	26C	72C
32A	28A	76A
32G	28G	74G
32A	28A	73A
32C	27C	75C
32C	28C	75C
33C	28C	74C
33C	28C	73C
34A	28A	74A
35G	28G	74G
34G	29G	74G
34A	30A	76A
33G	32G	75G
32G	32G	77G
32A	31A	76A
32C	31C	75C
32T	31T	75T
31C	31C	76C
31T	32T	77T
31G	32G	75G
31C	32C	73C
30T	30T	73T
32G	32G	72G
32C	32C	69C
33A	30A	69A
33G	30G	70G
33T	30T	69T
33A	30A	69A
34G	30G	68G
33T	30T	69T
33C	31C	67C
34T	32T	68T
34A	33A	67A
34T	31T	67T
34G	32G	65G
34C	32C	66C
34T	31T	66T
34C	32C	68C
33G	32G	68G

33G	32G	65G
33A	32A	64A
34C	32C	65C
33C	32C	65C
33G	31G	67G
33G	31G	68G
32A	31A	66A
32C	32C	63C
32C	32C	64C
33A	32A	64A
33G	32G	62G
32G	31G	62G
32G	31G	62G
33G	33G	66G
32G	33G	67G
32C	33C	65C
32T	32T	63T
31G	32G	64G
31A	32A	64A
31A	32A	64A
29T	32T	62T
29C	32C	60C
29A	33A	61A
29G	32G	62G
29G	31G	60G
29G	13G_19C	23G_38C
29A	33A	60A
29G	33G	60G
30G	33G	62G
30T	32T	63T
30T	32T	63T
29A	34A	63A
29A	33A	62A
28A	35A	62A
27C	36C	64C
27T	37T	63T
26G	37G	64G
26A	38A	63A
26G	38G	64G
26C	38C	63C
27T	37T	63T
26G	37G	64G
26A	37A	63A

26A	37A	65A
27G	37G	63G
27C	37C	64C
27T	37T	64T
26A	36A	63A
26A	36A	63A
26G	37G	63G
26A	37A	64A
26A	37A	66A
26T	37T	67T
27A	36A	69A
27C	36C	69C
28A	35A	68A
28T	34T	66T
30C	34C	68C
31A	35A	68A
32G	35G	69G
32G	38G	67G
35A	39A	68A
34T	39T	65T
35G	39G	66G
35T	38T	65T
35G	38G	66G
35T	38T	64T
34G	38G	63G
35T	39T	60T
35G	39G	61G
35G	39G	64G
32A	39A	62A
33A	40A	62A
32G	41G	65G
31G	41G	64G
32G	40G	64G
30G	40G	65G
29A	40A	65A
28T	38T	67T
29A	37A	65A
29C	16A_21C	41A_25C
28G	37G	66G
27T	38T	66T
27T	39T	63T
27C	38C	59C
27C	38C	58C

27T	38T	58T
26C	39C	58C
26T	40T	57T
25T	41T	56T
24T	41T	56T
24G	41G	57G
24C	39C	58C
23A	38A	57A
25G	38G	57G
25C	38C	57C
25C	38C	58C
25C	39C	58C
25C	39C	58C
25T	39T	60T
25C	39C	61C
26C	39C	59C
25G	39G	59G
25G	38G	59G
25A	38A	61A
25T	38T	61T
25T	38T	62T
25G	38G	62G
25G	39G	62G
24G	39G	61G
24C	39C	61C
22C	38C	62C
23C	37C	61C
23C	37C	60C
23T	38T	58T
23C	38C	60C
23T	38T	60T
23T	38T	59T
24C	37C	60C
23C	37C	59C
23C	38C	60C
23A	38A	57A
24G	39G	56G
24C	39C	54C
24T	39T	53T
23G	40G	51G
24C	41C	52C
24T	41T	49T
24G	40G	25G_26C

23T	40T	51T
23C	39C	51C
22A	39A	51A
22A	39A	49A
21G	37G	49G
21C	38C	50C
21A	38A	51A
21T	38T	49T
21C	39C	53C
21C	40C	56C
21A	40A	58A
21G	40G	55G
21T	40T	56T
21T	41T	55T
21G	41G	55G
21C	42C	56C
21T	41T	56T
21T	41T	55T
21A	41A	55A
20G	41G	52G
20A	41A	53A
19A	41A	54A
19A	40A	53A
19G	40G	54G
19C	38C	54C
19G	38G	24A_32G
19C	37C	56C
19C	38C	56C
19T	39T	55T
19C	40C	56C
19C	40C	58C
18A	41A	58A
18G	41G	57G
18C	41C	56C
18A	41A	56A
17G	41G	56G
16A	41A	56A
16T_8G	41T	55T
17G	41G	56G
17G	41G	56G
16G	40G	50G
16G	39G	52G
16G	38G	52G

15T	22T_16G	52T
15G	36G	52G
15G	37G	50G
14C	14G_23C	49C
14C	41C	47C
12C	41C	47C
11T	40T	46T
10T	40T	46T
7C	37C	45C
7C	36C	45C
7A	36A	45A
7G	35G	44G
7A	36A	45A
7C	35C	44C
7A	36A	45A
8T	36T	45T
8A	35A	44A
8G	35G	44G
8G	34G	44G
8A	35A	44A
8G	34G	44G
8A	34A	44A
8A	34A	44A
8A	34A	44A
8A	34A	44A
8G	34G	44G
7G	1T_16G_17C	43G
8C	34C	43C
8A	34A	41A
8G	34G	41G
8T	34T	41T
8C	33C	41C
8T_5C	32T_27C	40T
8C	33C	39C
8C	32C	39C
8C	32C	37C
8C	31C	34C
5A	27A	4A_16C
5C	27C	20C
5A_10C	27A_54C	3A_13T_5C
5C	27C	16C
5C	28C	22C
5C	28C	22C

5A	28A	22A
5C	28C	13T_10C
5C	28C	23C
5G	28G	24G
5C	28C	35C
5C	28C	35C
5T	28T	35T
5C	28C	35C
5C	28C	36C
5A	28A	37A
5A	28A	37A
5C	28C	37C
5A	28A	37A
5A	28A	37A
5C	28C	37C
5A	27A	37A
5A	26A	37A
5G	26G	36G
5G	26G	36G
4G	26G	35G
4G	26G	35G
4G	26G	36G
4A	25A	34A
3A	25A	34A
3C	24A	34A
3A	24A	33A
3A	24A	35A
3A	23A	34A
4C	22C	35C
4C	21C	34C
4C	21C	34C
4T	21T	34T
4C	21C	34C
4C	21C	35C
4C	20C	34C
3T_1C	20T	34T
4G	19G	34G
4G	19G	35G
4A	19A	35A
4C	19C	34C
4A	18A	34A
4T	18T	33T
4C	18C	32C

3A	18A	32A
3G	16G	32G
3G	16G	32G
3A	15A	32A
3C	15C	32C
3T	14T	33T
3G	14G	33G
3T	14T	31T
3T	14T	29T
3C	14C	28C
3G	14G	27G
3C	7G_7C	27C
3C	14C	28C
3T	13T	28T
3T	14T	28T
3T	13T	27T
3T	11T	27T
3T	10T	27T
3T	10T	27T
3A	9A	27A
3G	9G	26G
3A	9A	24A
3G	9G	24G
2G	9G	24G
2G	9G	21G
2T	9T	21T
2G	9G	21G
2C	9C	19C
2C	9C	18C
2A	9A	17A
2G	6G	14G
2G	4G	14G
1C	1G	2C
1A	null	2A
1A	null	2A
5C	null	2G_8C
1C	null	4C
1C	null	4C
1C	null	4C
1C	null	5C
1C	null	6C
1C	null	6C
3G	3G	13G

3C	3C	15C
3A	3A	15A
3G	3G	15G
3G	3G	17G
3A	4A	17A
3G	4G	17G
3G	4G	17G
3A	4A	17A
3G	4G	17G
3A	4A	17A
4A	4A	17A
4A	4A_1G	17A
4G	5G	17G
4G	5G	17G
4A	5A	18A
4G	5G	19G
4G	5G	19G
4A_2G	5A	20A
4G	5G	20G
4G	5G	21G
4G	5G	21G
4G	5G	23G
4G	5G	25G
4G	6G	25G
4T	6T	25T
4G	6G	25G
4A	7A	26A
4C	7C	25C
4T	7T	25T
4T	8T	25T
4T	8T	25T
4G	8G	25G
4C	8C	25C
4C	8C	26C
4A	8A	27A
4G	8G	25G
4C	8C	26C
4C	8C	26C
4A	8A	26A
5G	8G	26G
5C	8C	27C
5C	8C	27C
5T	8T	27T

5A	8A	25A
5G	8G	27G
5C	3A_5C	27C
5G	8G	27G
5A	8A	27A
5C	8C	28C
5T	8T	27T
5C	8C	28C
5C	8C	27C
5C	8C	28C
6A	8A	27A
6C	8C	28C
6A	8A	28A
6C	8C	28C
2A_3G	8G	13A_15G
5T	8T	29T
5C	8C	29C
5A	8A	30A
5G	8G	28G
5G	8G	28G
6T	8T	27T
6T	8T	27T
6G	8G	27G
6G	8G	26G
5G	8G	25G
5C	8C	25C
6G	8G	25G
6G	8G	23G
6A	8A	23A
6G	8G	23G
6G	8G	21G
6G	8G	21G
6A	8A	21A
6C	7C	21C
6C	7C	21C
6C	7C	21C
6A	7A	21A
6A	7A	21A
6A	7A	21A
6T	7T	20T
6C	7C	21C
null	7A	20A
6T	7T	19T

6G	6G	19G
6G	6G	19G
6G	6G	19G
6C	6C	19C
6T	6T	16T
6C	6C	15C
6A	6G	8A_7G
6C	6C	14C
6C	6C	14C
6T	6T	14T
6C	6C	14C
6C	6C	13C
6C	6C	13C
6A	6A	12A
6G	6G	12G
6G	6G	12G
6C	6C	12C
6A	6A	11A
6G	6G	11G
5G	6G	10G
5C	6C	10C
5A	6A	10A
5G	6G	10G
5C	6C	10C
5T	6T	9T
5G	5G	9G
4T	4T	9T
4C	4C	9C
4T	4T	8T
4T	4T	8T
4G	4G	8G
4G	4G	8G
4C	4C	7C
4C	4C	7C
4T	4T	7T
4G	4G	7G
4C	5C	7C
4A	5A	6A
4A	5A	6A
4A	4A	5A
4C	4C	4C
4A	4A	4A
4A	4A	4A

4G	4G	3G
3C	4C	2C
3T	4T	3T
3T	4T_1C	3T
3C	4C	2C
3T	3T_1C	2T
3C	1A_3C	3C
3C	1A_3C	1C
3C	4C	2C
3C	4C	2C
3C	4C	2C
3C	4C	2C
3C	5C	2C
3C	5C	2C
3C	4C	2C
3C	4C	2C
3A	2A_2C	2A
3C	4C	1T_1C
3A	3A	2A
3T	3T	2T
3G	3G	2G
3C	4C	1T_1C
3G	4G	2G
3C	4C	1T_1C
3C	4C	2C
3C	4C	2C
3T	4T	2T
3G	5G	2G
3A	5A	1A
3C	5C	1C
3T	4T_1G	1T
2C	5C	1C
2A	5A	1A
2G	5G	1G
2G	5G	1G
2T	5T	1T
2G	5G	1G
2A	5A	1A
2A	5A	1A
2G	6G	1G
2G	6G	1G
1A	6A	1A
1G	6G	1G

1A	6A	1A
1G	6G	1G
null	6G	1G
null	6G	1G
null	6G	1G
null	6T	1T
null	6A	1A
null	6C	1C
null	6A	1A
null	6A	1A
null	6A	1A
null	6C	1C
null	6C	1C
null	6G	1G
null	6G	1G
null	6C	1C
null	6T	1T
null	null	1C
null	null	1C
null	null	1C
null	null	1C
null	6C	1C
null	6C	1C
null	7C	1C
null	7C	1C
null	7C	1C
null	7C	1C
null	7C	1C
null	7G	null
null	7G	null
null	7T	null
null	7T	null
null	7T	null
1G	7G	null
1T	8T	null
1T	8T	1T
1C	8C	1C
1T	3A_6T	1T
3G	10G	1G
4T	10T	2T
4G	12G	6G
4T	12T	7T
4T	12T	7T

4T	13T	7T
4T	13T	8T
5G	13G	8G
6G	13G	8G
6C	13C	8C
6T	14T	9T
7T	14T	10T
7C	14C	11C
7G	14G	13G
7A	14A	13A
7T	14T	14T
7T	14T	15T
7T	14T	16T
8T	14T	18T
8C	14C	18C
8C	15C	21C
8C	15C	21C
8T	15T	22T
8A	15A	23A
8T	15T	24T
8C	15C	25C
8A	15A	25A
8G	15G	26G
8C	17C	26C
8T	17T	27T
8T	17T	28T
8G	17G	29G
9G	17G	32G
9A	17A	32A
9A	17A	32A
9G	15G	32G
9C	16C	33C
9C	16C	33C
9A	17A	34A
9A	17A	34A
9G	17G	35G
9C	17C	35C
9C	17C	35C
10G	17G	35G
11G	18G	36G
11A	18A	36A
11A	18A	37A
11A	18A	38A

11G	18G	38G
11A	18A	38A
11T	9T_8C	40T
11G	17G	40G
11A	18A	42A
12T	8A_10T	43T
12T	18T	44T
12C	18C	44C
12G	19G	44G
12G	19G	45G
13A	19A	45A
13T	19T	45T
13T	19T	46T
13T	19T	46T
14T	19T	48T
14A	19A	48A
14T	19T	48T
14G	19G	49G
15A	19A	49A
16A	20A	50A
16A	20A	52A
16C	20C	52C
17A	19A	54A
17C	19C	53C
17A	19A	50A
17C	19C	50C
17A	19A	50A
17C	19C	50C
17A	19A	49A
17C	19C	50C
17A	19A	50A
18C	19C	50C
18A	19A	49A
18C	19C	18T_30C
10T_8C	6T_12C	47C
18G	18G	46G
19G	18G	47G
19A	18A	45A
19A	18A	47A
19A	18A	47A
19A	17A	46A
20A	18A	46A
21A	19A	43A

21A	18A	44A
21A	18A	43A
22A	19A	43A
21A_10C	19A	40A
10C	18C	41C
22C	19C	42C
22T	19T	42T
22T	20T	41T
22C	20C	42C
23A	7A_13G	23A_18G
23C	20C	41C
24C	21C	41C
25A	21A	38A
25A	21A	38A
26G	21G	38G
27G	21G	38G
28A	21A	37A
28A	22A	39A
28G	25G	38G
29C	25C	40C
29T	25T	39T
29T	26T	39T
30A	27A	40A
31G	27G	40G
31G	27G	39G
30A	27A	37A
30G	26G	38G
30C	26C	39C
31A	26A	39A
31A	24A	38A
30A	24A	38A
30G	25G	39G
15G_14C	23T	18T_19C
29C	24C	37C
29C	24C	37C
29A	23A	39A
29G	23G	39G
28C	23C	38C
28T	24T	39T
27G	24G	39G
28C	24C	38C
27G	24G	38G
27A	24A	36A

28T	24T	36T
28T	24T	37T
28A	24A	38A
28T	24T	37T
28T	24T	36T
28T	24T	34T
27C	23C	34C
12T_15C	25C	23C
12T_15G	26G	25G
27G	26G	25G
27C	26C	25C
27C	26C	25C
27C	26C	26C
27C	26C	26C
27C	26C	26C
27C	24C	27C
12A_15G	25A	28A
27C	25C	26C
27C	25C	26C
27C	24C	26C
28G	25G	25G
26G	25G	25G
28C	25C	27C
29T	25T	26T
29C	24C	28C
29A	24A	28A
30A	24A	29A
31T	25T	30T
31C	25C	31C
31T	25T	31T
32A	25A	31A
30G	24G	30G
29A	15A_9G	30A
29G	24G	32G
28C	24C	31C
30C	25C	33C
30C	25C	33C
30A	23A	32A
30A	23A	32A
30G	22G	32G
29G	23G	34G
28G	24G	35G
29A	24A	34A

29T	24T	34T
29G	25G	33G
29C	25C	33C
30C	25C	31C
30C	24C	32C
30C	25C	32C
30A	25A	32A
29G	25G	32G
29G	24G	32G
29C	24C	32C
28T	24T	33T
28G	23G	34G
28A	23A	32A
28G	23G	34G
28T	23T	33T
27C	23C	33C
28T	11T_12C	34T
28C	23C	33C
30T	23T	32T
30C	23C	30C
31G	24G	30G
31C	24T	31C
31G	24G	31G
30G	24G	31G
30C	25C	39C
30G	26G	39G
31C	26C	38C
31G	25G	39G
32A	26A	38A
31A	26A	37A
31A	26A	37A
31A	26A	37A
31G	27G	38G
31C	26C	40C
31A	26A	39A
31C	26C	37C
31T	26T	36T
30G	26G	37G
30A	26A	37A
30G	28G	31G
31T	28T	39T
32T	27T	39T
32A	27A	38A

32T	27T	38T
32T	29T	38T
31T	28T	40T
30T	28T	40T
30T	29T	43T
31A	30A	44A
31T	30T	43T
30T	31T	43T
30T	31T	42T
31A	28A	44A
30T	28T	43T
31T	28T	41T
33C	27C	41C
32A	26A	41A
31T	27T	42T
31T	27T	43T
31G	27G	43G
32G	27G	34G
32A	26A	34A
31A	28A	34A
31C	15A_14C	34A
31A	29A	35A
31A	28A	37A
31A	28A	37A
31G	28G	34G
31A	28A	35A
31G	28G	34G
32T	28T	36T
32T	28T	36T
32C	27C	36C
33A	27A	35A
33A	27A	35A
33T	27T	35T
33T	13T_14G	34T
33T	27T	36T
33A	27A	38A
33T	28T	40T
33T	28T	39T
34T	28T	38T
34A	29A	38A
35C	28C	38C
35C	27C	38C
35G	27G	38G

36G	28G	38G
36A	28A	39A
37G	28G	39G
38C	27C	42C
38A	28A	42A
38C	27C	43C
38A	28A	43A
38T	28T	44T
39T	28T	61T
40C	31C	62C
40C	31C	62C
39A	32A	66A
39T	32T	66T
37A	34A	66A
36C	34C	68C
37T	34T	68T
38T	35T	67T
36T	35T	65T
35A	34A	67A
35C	34C	68C
35T	34T	70T
35G	34G	70G
36C	34C	29T_40C
37A	35A	69A
37A	36A	68A
37T	36T	71T
36G	35G	73G
36T	35T	72T
36A	35A	74A
36G	35G	73G
36G	35G	73G
36A	34A	69A
36A	33A	68A
36A	35A	71A
37G	34G	71G
37G	33G	69G
37A	33A	69A
36C	34C	69C
36T	35T	70T
38G	35G	70G
38T	36T	70T
40T	36T	68T
40C	38C	67C

26T_14C	38C	68C
40T	38T	70T
40T	39T	69T
40C	39C	69C
40A	39A	68A
41T	39T	69T
41T	39T	68T
40T	39T	68T
40C	40C	71C
38T	41T	69T
39G	41G	69G
38C	40C	67C
38G	40G	67G
38T	41T	67T
37A	40A	68A
38A	40A	68A
38T	40T	67T
38G	41G	66G
38G	41G	63G
37G	41G	60G
37G	41G	59G
37A	43A	61A
38G	43G	34G_28C
37A	43A	62A
38C	43C	64C
37A	43A	64A
38C	43C	65C
37A	42A	63A
37G	44G	51A_68T_113G_34C
36A	45A	47A
36A	43A	44A
36T	43T	45T
36G	43G	34G
36G	43G	35G
36T	42T	34T
37G	41G	35G
37C	42C	36C
37C	42C	37C
37T	41T	37T
36A	41A	39A
36T	40T	39T
36G	39G	66G
36A	38A	66A

35A	38A	68A
35T	38T	70T
34A	38A	74A
32T	38T	74T
32A	38A	75A
32C	37C	75C
33T	37T	75T
33T	37T	75T
32G	37G	75G
32G	37G	72G
32T	37T	73T
32T	17T_19C	76T
32T	36T	75T
31A	36A	75A
32T	36T	75T
32T	37T	76T
32T	40T	75T
33T	40T	72T
34T	40T	73T
34C	40C	73C
24T_9C	40C	76C
32G	40G	77G
32G	40G	75G
33A	40A	74A
33C	40C	76C
32A	40A	76A
32G	40G	78G
32C	39C	78C
32A	39A	78A
31T	38T	76T
31A	38A	77A
30A	38A	78A
30T	38T	79T
31T	37T	78T
33T	36T	78T
34C	36C	78C
33A	35A	80A
32A	37A	81A
32A	37A	83A
32A	37A	83A
32T	36T	84T
31G	37G	82G
32A	37A	81A

32A	36A	85A
32A	36A	87A
32C	37C	89C
33T	36T	90T
33T	35T	91T
34A	35A	89A
37A	36A	89A
36T	35T	89T
36T	35T	86T
38A	35A	89A
39T	35T	89T
39T	34T	88T
38A	34A	86A
37G	35G	86G
36T	35T	86T
37C	35C	87C
37C	35C	85C
36A	35A	84A
38T	35T	83T
38T	35T	83T
38T	35T	83T
39T	36T	82T
28T_10G	17T_18G	82G
39G	34G	83G
39C	32C	85C
38A	32A	90A
11G_27C	16G_16C	50G_40C
39G	31G	89G
39G	31G	89G
39C	30C	85C
37C	30C	86C
38A	30A	85A
36G	30G	84G
36C	28C	84C
36T	28T	84T
37A	30A	84A
38A	30A	85A
38G	30G	85G
38T	29T	82T
37G	31G	83G
38T	30T	82T
39T	32T	83T
39C	31C	84C

39T	30T	87T
38G	31G	86G
38G	31G	85G
38G	30G	84G
38A	30A	81A
39C	32C	82C
40T	32T	81T
39A	31A	79A
25T_13C	16T_14C	80C
39A	30A	79A
40G	30G	77G
40C	30C	79C
40T	29T	76T
39C	30C	78C
39G	28G	74G
38C	29C	74C
39A	30A	75A
40T	31T	77T
41G	31G	77G
43C	31C	79C
43T	31T	78T
42C	31C	78C
42C	31C	76C
42T	31T	76T
42T	29T	76T
42G	32G	76G
43A	32A	77A
43C	31C	76C
43C	31C	73C
44T	31T	72T
43C	30C	71C
43T	30T	70T
43G	30G	67G
43G	30G	66G
42C	28C	32T_29C
42A	28A	29A_32G
44G	30G	61G
45G	29G	60G
45G	30G	60G
45T	30T	60T
45T	30T	60T
45G	30G	61G
47G	30G	29A_30G

48G	30G	61G
48T	29T	63T
48G	30G	63G
48G	30G	62G
49G	31G	62G
50A	30A	61A
50A	30A	62A
51T	28T	60T
50G	28G	58G
48A	27A	53A
48T	28T	49T
49G	28G	54G
49G	28G	53G
49G	28G	54G
48A	28A	54A
48G	28G	55G
48T	28T	54T
49T	28T	54T
49C	28C	26T_29C
49T	29T	57T
49A	30A	56A
49G	30G	56G
49T	30T	56T
48C	30C	61C
49C	31C	62C
46C	31C	59C
46A	31A	59A
46A	33A	58A
46A	32A	59A
46C	32C	58C
47A	32A	57A
47A	32A	58A
48C	30C	58C
47T	30T	60T
46A	30A	61A
46G	31G	59G
45A	30A	59A
45A	30A	62A
45G	30G	60G
44G	30G	59G
40G	29G	59G
40T	28T	54T
40C	29C	60C

38C	29C	56C
37T	30T	55T
37G	31G	53G
37G	31G	52G
37G	31G	55G
36T	30T	54T
36C	29C	56C
36A	29A	56A
36C	29C	55C
36T	30T	57T
37T	31T	56T
37G	31G	55G
36C	30C	56C
35A	29A	57A
34G	31G	59G
34G	31G	59G
34T	31T	58T
34C	33C	60C
34T	33T	61T
34C	33C	61C
34T	33T	61T
34G	34G	61G
33G	34G	61G
33C	17A_34C	28A_64C
33G	32G	37A_29G
33G	35G	65G
32G	33G	66G
30A	15A_17G	65G
29A	32A	64A
29A	32A	63A
29A	32A	64A
28A	31A	65A
29T	31T	65T
30G	31G	66G
30G	31G	65G
30A	30A	66A
30T	31T	68T
32C	31C	68C
32C	31C	67C
32T	29T	67T
31G	29G	37A_29G
32T	29T	66T
32C	29C	66C

32C	29C	69C
32T	29T	68T
32C	29C	67C
32A	28A	67A
34C	14A_14C	67C
35C	29C	67C
35A	28A	66A
34G	28G	65G
34A	27A	67A
32T	27T	65T
32C	26C	66C
32C	26C	66C
33T	26T	65T
35T	26T	65T
36C	26C	69C
37T	25T	71T
40A	26A	70A
38G	26G	70G
39A	26A	70A
39A	26A	70A
38G	26G	70G
38G	26G	68G
37A	26A	69A
36G	25G	69G
37A	25A	69A
37T	25T	69T
37C	27C	70C
37A	26A	71A
37C	26C	74C
37G	26G	74G
37G	26G	72G
37G	26G	69G
37G	27G	71G
35A	27A	72A
34T	27T	72T
34T	27T	72T
34G	27G	70G
35A	26A	70A
34A	26A	71A
33C	26C	73C
34T	26T	73T
33G	26G	72G
33G	26G	74G

34C	28C	71C
34A	29A	72A
33A	28A	72A
32C	30C	72C
33C	31C	72C
33T	31T	73T
32T	32T	74T
32C	32C	71C
32C	32C	70C
32A	30A	71A
34C	31C	69C
34C	31C	69C
34C	34C	68C
35T	34T	68T
35C	33C	67C
35T	33T	67T
37C	34C	66C
36T	32T	66T
36A	32A	66A
36G	32G	67G
36C	32C	64C
36T	32T	67T
36T	34T	69T
35T	34T	69T
35C	32C	68C
36A	33A	69A
36T	31T	70T
37A	31A	70A
38T	31T	69T
39T	31T	69T
40G	32G	72G
41A	31A	70A
41T	33T	72T
41C	33C	71C
42C	33C	70C
42C	31C	70C
41T	32T	71T
43C	32C	70C
43T	32T	69T
43G	32G	69G
43A	32A	71A
44G	32G	68G
44G	32G	66G

43A	31A	68A
42G	30G	69G
42G	30G	70G
43A	31A	73A
43A	29A	74A
43G	30G	76G
43C	30C	77C
44T	28T	77T
45G	28G	78G
45T	28T	79T
45T	28T	77T
47C	28C	76C
47T	26T	75T
23A_24G	26G	47A_29G
47G	26G	76G
47G	26G	77G
47T	25T	75T
47G	25G	74G
47A	25A	74A
47A	25A	76A
47A	26A	76A
46A	26A	77A
46C	26C	78C
45T	26T	76T
44C	26C	74C
44C	26C	74C
45A	26A	73A
23A_22G	25G	73G
43T	24T	72T
44T	25T	73T
42G	25G	73G
43A	25A	75A
42T	26T	75T
42A	28A	75A
43C	29C	76C
42A	29A	76A
43A	29A	76A
43T	29T	76T
41G	29G	77G
42G	28G	78G
42G	28G	77G
42C	28C	77C
42T	28T	79T

42T	29T	80T
42C	28C	81C
42A	28A	80A
41A	28A	78A
40T	28T	79T
39G	28G	77G
39A	28A	74A
36T	27T	76T
37T	27T	78T
37T	27T	76T
37T	28T	75T
37T	28T	72T
39C	28C	74C
39C	28C	74C
40T	28T	71T
39C	28C	70C
39T	27T	71T
39T	27T	71T
38T	27T	70T
37G	27G	68G
39G	27G	68G
39C	27C	66C
39A	27A	67A
39T	26T	69T
39G	26G	68G
39A_15T	26A	69A
40T	26T	69T
40T	26T	66T
39A	25A	26A_39T
38A	26A	63A
38A	26A	59A
37A	26A	59A
37A	26A	59A
35G	26G	32A_25G
36A	24A	57A
36G	25G	56G
37A	25A	53A
36G	24G	54G
35G	23G	55G
35A	23A	53A
36A	22A	53A
37A	22A	53A
37C	23C	52C

36A	23A	52A
35A	23A	52A
36A	23A	50A
36G	21G	49G
35C	22C	48C
35A	22A	48A
36T	22T	46T
35C	21C	46C
35C	22C	46C
35A	22A	46A
16G_20C	8G_14C	22G_24C
36C	22C	46C
36T	22T	46T
37A	20A	45A
37C	20C	45C
36A	20A	43A
39A	20A	41A
38A	20A	40A
37C	20C	39C
37C	21C	39C
35C	22C	41C
34A	21A	41A
34C	22C	40C
35A	21A	41A
34C	22C	41C
34A	22A	41A
34G	22G	38G
34G	21G	37G
34A	22A	36A
35A	22A	36A
35C	7T_15C	25T_11C
34A	23A	35A
34T	23T	34T
34G	23G	34G
33C	23C	32C
52G	24G	30G
25G	24G	30G
22C	23C	24C
24G	69G	32G
35G	10G	31G
18T_71G	9G	32G
33G	9G	32G
35G	25G	32G

18T_16G	25G	31G
32G	25G	32G
33G	25G	32G
1T_14G_18C	25G	32G
34G	25G	32G
35G	25G	30G
34C	25C	27C
34T	25T	31T
34G	26G	32G
34G	26G	31G
34T	27T	29T
35T	27T	33T
36T	28T	32T
36T	28T	34T
37G	29G	35G
37G	27G	36G
37C	27C	38C
37T	27T	38T
37T	27T	39T
37C	27C	39C
37T	27T	40T
37T	18T_10C	13T_27C
36C	28C	39C
35T	27T	40T
33G	28G	40G
34A	27A	38A
34C	27C	40C
32T	26T	41T
32C	24C	41C
32C	24C	41C
33A	24A	40A
34A	24A	41A
34C	24C	43C
35A	24A	48A
35T	25T	47T
34G	25G	48G
35C	24T_37C	49C
34C	13C	50C
34T	12T	49T
33C	12C	50C
33C	25C	49C
34T	13T_12C	49T
34T	26T	49T

33C	25C	51C
33C	25C	51C
34T	26T	51T
33T	26T	52T
33T	26T	52T
33C	26C	51C
32C	26C	50C
33T	26T	50T
30A	26A	48A
30T	26T	48T
30G	27G	49G
30G	27G	50G
30C	27C	51C
30C	27C	49C
29T	28T	48T
29C	28C	48C
29T	27T	48T
29G	27G	48G
29G	28G	48G
29G	28G	47G
29C	28C	49C
29A	28A	48A
29G	28G	49G
29A	27A	51A
29G	29G	51G
28T	27T	52T
29G	29G	53G
29C	28C	55C
28T	29T	57T
27T	30T	58T
27C	30C	57C
26T	30T	57T
26G	29G	58G
26A	30A	58A
26A	30A	58A
25C	29C	58C
25A	29A	58A
24T	29T	59T
24G	29G	59G
24C	29C	60C
23A	29A	60A
23C	28C	61C
23A	27A	60A

23G	27G	62G
23A	27A	61A
23A	27A	61A
24T	26T	64T
7A_14G	16A_11G	63A
20C	27C	63C
20C	27C	28G_36C
20C	27C	64C
19T	27T	63T
19T	28T	65T
19C	28C	65C
19C	28C	64C
19T	27T	65T
19A	27A	67A
19G	27G	68G
19A	26A	68A
19A	26A	68A
19T	26T	34T_30C
19G	25G	64G
19A	25A	60A
19G	25G	62G
19C	24C	63C
19T	24T	63T
19T	24T	64T
18T	24T	67T
11G_6C	11G_13C	68C
17T	24T	70T
16G	23G	67G
16G	22G	67G
16C	22C	68C
16T	21T	68T
16T	21T	67T
16C	21C	68C
14T	21T	70T
14A	21A	73A
14G	21G	74G
13T	21T	72T
13C	21C	73C
13C	21C	73C
13A	21A	73A
12T	21T	74T
12A	21A	75A
12G	21G	78G

12G	21G	78G
6A_6G	12A_8G	41A_37G_1C
11T	20T	81T
11G	19G	82G
11C	19C	83C
10A	18A	86A
10T	18T	90T
10C	17C	90C
10T	17T	90T
10A	17A	90A
10C	17C	90C
10T	17T	88T
10T	17T	85T
10A	17A	85A
10G	18G	85G
10G	18G	84G
12C	17C	83C
12C	17C	84C
12A	17A	83A
12A	17A	84A
11T	18T	86T
12T	18T	84T
1A_3T_5G	5T_13G	82G
11T	18T	80T
10G	17G	80G
11T	33T_17G	77T
10G	15G	79G
9T	15T	73T
9G	15G	104G
9T	15T	104T
9G	15G	104G
9T	15T	100T
8G	14G	101G
8T	14T	101T
8G	14G	101G
8T	14T	99T
8G	13G	100G
8T	13T	99T
9G	13G	100G
9T	13T	100T
9G	13G	97G
8T	13T	96T
8G	13G	96G

7T	13T	95T
7G	13G	94G
7T	12T	93T
7G	12G	92G
7T	12T	91T
7G	12G	89G
7T	11T	88T
7G	11G	86G
7T	11T	86T
8G	11G	85G
8T	10T	83T
8G	10G	83G
8T	11T	84T
8G	11G	84G
10T	11T	84T
10G	13G	85G
11T	12T	84T
11G	14G	82G
12T	13T	82T
12G	14G	64G
13T	13T	78T
13G	13G	75G
13T	20T	77T
12G	21G	69G
13T	23T	69T
13G	21G	69G
13T	21T	66T
13G	22G	63G
13T	22T	57T
12G	22G	57G
12T	23T	56T
12G	23G	57G
12T	23T	57T
12G	24G	58G
11T	24T	59T
10G	24G	61G
11T	23T	62T
10G	24G	62G
10T	24T	63T
10G	24G	63G
10T	24T	64T
10G	25G	64G
10T	25T	64T

10G	23G	64G
11T	23T	66T
11G	24G	67G
12T	25T	69T
12G	26G	69G
12T	27T	72T
12G	27G	20T_52G
13T	27T	72T
13G	27G	73G
13T	27T	74T
13G	27G	74G
13T	27T	76T
13G	27G	45G
13T	27T	45T
13G	27G	47G
13A	17A_10T_7C	31A_16T_23C
7G_6C	20C	25C
13A	28A	48A
13C	28C	48C
13A	28A	48A
13C	29C	46C
13A	28A	50A
14A	28A	51A
14A	28A	29A_25C
14T	19T	54T
14C	19C	55C
15T	19T	56T
15G	19G	58G
15C	19C	61C
15A	19A	63A
15G	19G	63G
15G	19G	63G
15C	20C	65C
15T	21T	66T
15T	22T	65T
16C	23C	68C
16T	24T	68T
16G	24G	72G
19T	24T	71T
19T	24T	72T
19G	24G	72G
19A	24A	72A
20T	23T	72T

20C	23C	74C
20A	23A	74A
20C	23C	74C
20A	22A	74A
20G	23G	74G
19A	23A	73A
6G_13C	24C	72C
19C	24C	74C
21T	25T	74T
21T	25T	75T
21T	25T	76T
21T	25T	77T
21C	25C	76C
21T	25T	78T
21T	25T	79T
21T	26T	79T
21A	26A	80A
21A	26A	78A
21A	26A	77A
21A	26A	76A
21C	26C	74C
21A	26A	74A
21T	26T	76T
21T	26T	77T
21C	26C	77C
21G	27G	76G
22T	28T	76T
22C	28C	77C
22T	28T	78T
21C	29C	80C
20C	30C	78C
21T	30T	80T
21T	30T	78T
21C	30C	77C
21C	29C	79C
21A	30A	82A
21C	31C	84C
21A	31A	85A
20G	30G	84G
20C	30C	82C
20A	31A	81A
20A	32A	79A
20T	32T	79T

19A	32A	80A
19A	32A	79A
19A	32A	80A
19C	32C	80C
19T	34T	81T
19T	33T	76T
20T	32T	73T
20G	31G	72G
20A	30A	71A
19T	30T	73T
19A	30A	75A
18T	30T	74T
18A	30A	72A
18T	30T	70T
18T	30T	70T
18T	32T	69T
18A	32A	68A
19T	32T	67T
19A	31A	66A_26T
19A	31A	62A
19T	31T	87T
19T	29T	30T
19T	30T	56T
19T	30T	60T
19T	30T	60T
19T	31T	61T
20T	30T	79T_22C
21T_10C	31T	36T
9C	28C	30C
22C	31C	59C
23C	30C	61C
23C	30C	61C
23A	30A	61A
23T	30T	65T
23T	29T	65T
23T	30T	65T
22T	30T	64T
22G	31G	65G
22C	31C	67C
21T	30T	69T
21T	29T	69T
22T	28T	68T
21G	28G	68G

21G	28G	67G
22A	28A	69A
22T	29T	69T
22C	29C	67C
22T	31T	70T
24G	33G	70G
24T	34T	71T
23G	34G	72G
23A	33A	72A
23T	33T	73T
25C	13T_20C	34T_39C
27G	33G	74G
28A	32A	74A
29A	32A	76A
29G	32G	73G
28T	32T	74T
28T	32T	75T
28A	32A	73A
29G	32G	72G
28A	33A	73A
29T	33T	72T
29T	32T	69T
28C	32C	67C
29T	32T	68T
29G	31G	68G
29T	32T	68T
29T	32T	68T
29C	31C	67C
30T	30T	67T
30T	30T	68T
29T	28T	31T_36C
27T	30T	67T
27G	30G	66G
29A	30A	64A
29A	30A	65A
27A	30A	65A
28C	26A_30C	63C
28T	27A	59C
28A	27A	58A
28A	27A	56A
28G	27A	55A
28A	34A	55A
28A	34A	56A

27C	33C	21A_35C
27C	33C	58C
27C	34C	60C
27G	34G	60G
27A	33A	59A
27C	34C	62C
27A	34A	62A
27A	34A	60A
27T	33T	59T
27T	32T	12A_59T_4C
28C	33C	59C
27C	33C	44C
27T	33T	57T
27G	33G	44G
27T	32T	44T
27T	33T	44T
27G	12G_21C	46G
27T	33T	42T
26C	33C	46C
26C	32C	43C
26A	32A	71A
26C	31C	71C
26T	31T	71T
26C	31C	71C
25A	30A	73A
25A	31A	74A
25A	30A	74A
26A	29A	75A
26A	29A	75A
27G	29G	76G
28G	29G	78G
28G	19A_9G	79G
29G	27G	78G
30T	27T	79T
31G	27G	80G
31G	27G	81G
31A	8A_19G	83A
31A	28A	83A
32A	29A	84A
32A	29A	83A
31A	32A	85A
30C	31C	85C
30A	32A	84A

29A	31A	84A
31C	31C	86C
31C	31C	88C
32C	32C	87C
33G	33G	84G
34G	33G	84G
33A	32A	81A
34A	33A	79A
34A	33A	80A
33T	33T	81T
34A	33A	79A
34T	33T	79T
34C	33C	79C
34C	33C	79C
35A	33A	82A
36G	32G	81G
35A	32A	79A
35C	31C	82C
34C	17G_14C	81C
34T	31T	79T
33C	31G_14C	80C
32G	31G	80G
32A	30A	84A
32C	32C	84C
33A	32A	85A
33G	32G	85G
34T	31T	83T
34G	31G	83G
34C	31C	83C
34C	31C	80C
35G	31G	81G
35G	31G	82G
33A	30A	84A
33A	31A	84A
34A	32A	84A
33A	31A	84A
34A	32A	83A
34G	30G	83G
34C	26C	83C
33A	27A	82A
34T	28T	80T
34C	28C	81C
35C	28C	82C

35G	29G	76G
33G	29G	78G
33A	29A	78A
32T	29T	79T
31T	29T	81T
31T	30T	81T
31C	30C	84C
31A	30A	83A
31G	30G	81G
30C	30C	80C
30C	30C	82C
30A	30A	81A
30T	29T	80T
30G	30G	79G
30C	30C	77C
30A	29A	75A
31G	30G	75G
32C	29C	75C
32C	30C	75C
30T	30T	72T
30C	30C	72C
30A	29A	74A
29G	30G	71G
29T	30T	75T
27G	30G	73G
27T	31T	74T
27A	29A	73A
26A	26A	74A
27C	26C	73C
27C	25C	70C
27C	28C	70C
27C	28C	71C
27G	28G	72G
27T	28T	72T
27T	29T	73T
28C	29C	73C
28A	27A	73A
28T	29T	71T
28C	30C	75C
28T	30T	75T
28T	30T	75T
29T	30T	73T
29T	30T	74T

30T	30T	75T
30T	30T	73T
30T	30T	74T
30T_13C	30T	75T
16C	28C	41T_31C
31T	29T	73T
31T	29T	43T_32C
31C	29C	73C
31C	29C	72C
32A	28A	71A
33C	27C	71C
34C	28C	71C
34A	28A	70A
34A	28A	70A
34A	29A	72A
34C	29C	70C
33A	29A	69A
33A	30A	66A
33T	31T	65T
33T	32T	66T
31C	32C	68C
31G	32G	67G
31C	32C	67C
30T	33T	65T
29T	33T	63T
30T	35T	62T
31C	36C	64C
30A	36A	64A
29C	34C	63C
29T	32T	64T
30T	33T	67T
30T	32T	66T
30C	31C	64C
31T	31T	61T
30C	32C	62C
29T	32T	64T
32T	33T	64T
31A	32A	64A
31T	32T	64T
29T	32T	64T
29C	31C	63C
29A	31A	63A
29A	31A	63A

30G	31G	65G
28A	31A	64A
29C	32C	65C
29C	32C	63C
28C	33C	65C
28C	34C	65C
28C	34C	65C
28G	35G	33A_33G
30A	36A	64A
30A	35A	65A
30C	35C	65C
31G	36G	66G
31T	37T	65T
31T	37T	64T
31C	37C	67C
32A	37A	68A
32A	37A	66A
31G	38G	67G
31G	38G	66G
31A	38A	68A
31T	38T	69T
32T	40T	68T
34C	40C	71C
33C	40C	69C
33T	40T	42T_29G
34G	40G	73G
34C	39C	74C
33C	39C	74C
33T	39T	74T
34T	39T	73T
33T	39T	73T
32C	38C	76C
33T	39T	75T
33T	40T	76T
33C	39C	78C
33T	40T	79T
35C	28T_12C	80C
36C	39C	81C
36T	40T	80T
36C	40C	81C
36C	40C	84C
36G	40G	82G
37C	42C	82C

37C	42C	81C
38T	41T	83T
38G	42G	86G
38G	42G	83G
37A	41A	84A
37T	41T	86T
37T	42T	86T
36C	42C	89C
35A	41A	90A
35G	40G	90G
35C	40C	88C
35C	40C	87C
35A	40A	88A
35G	41G	87G
35C	41C	84C
35G	41G	84G
35A	42A	84A
19T_17C	28T_12C	84T
37C	39C	82C
38T	42T	81T
38C	14T_28C	81C
38T	44T	80T
38T	44T	79T
38T	44T	78T
38A	44A	78A
39T	44T	77T
39T	43T	80T
39T	44T	80T
38G	42G	81G
38C	43C	80C
38C	42C	81C
40C	42C	79C
37G	43G	79G
39A	43A	78A
40T	43T	78T
40T	43T	77T
40T	43T	75T
40G	43G	74G
40G	42G	72G
39G	42G	73G
39T	43T	74T
39T	42T	74T
39T	43T	73T

40A	43A	73A
39A	43A	75A
38G	43G	76G
38A	43A	75A
38A	43A	74A
38A	42A	75A
39A	42A	75A
40T	42T	75T
40A	42A	73A
40T	41T	70T
40A	40A	68A
43T	40T	68T
42T	40T	69T
42A	43A	72A
42G	42G	72G
45A	42A	71A
46T	42T	71T
46A	42A	70A
47T	42T	67T
47T	42T	67T
47C	42C	67C
46T	42T	64T
47C	42C	66C
48T	45T	66T
49C	45C	63C
49T	46T	66T
50T	46T	69T
48T	46T	69T
48T	45T_47C	65T_27C
48T	48T	62T
48T	48T	65T
48T	49T	67T
48A	49A	67A
48A	49A	65A
47A	50A	63A
47A	48A	66A
47C	49C	65C
47G	49G	66G
49C	49C	67C
27T_23C	49C	32T_32C
51A	48A	66A
51A	47A	65A
48A	48A	65A

48C	48C	65C
48A	47A	65A
47A	46A	65A
47A	47A	66A
48G	48G	67G
48A	47A	66A
47G	47G	67G
46T	47T	68T
48C	47C	69C
48A	48A	69A
48T	49T	66T
49T	49T	67T
48T	50T	66T
47A	51A	66A
47A	51A	66A
47A	52A	66A
46A	52A	63A
46C	53C	64C
46G	52G	64G
46A	54A	66A
45G	52G	68G
45G	53G	68G
47C	52C	69C
22A_24G	51A	36A_33G
47T	50T	67T
47T	50T	66T
45T	51T	68T
44A	51A	68A
44C	53T	35T_30C
44G	53G	64G
45C	52C	62C
45A	51A	63A
45T	50T	62T
44T	52T	61T
44G	52G	62G
19A_24G	53G	63G
43A	54A	63A
43G	54G	65G
43T	53T	67T
44T	53T	66T
44G	54G	62G
44T	56T	63T
44A	57A	65A

44A	57A	65A
44C	57C	65C
44T	56T	69T
44T	56T	71T
44T	55T	70T
44T	55T	72T
44G	54G	71G
44G	53G	71G
43T	52T	71T
42G	51G	70G
41C	51C	69C
44T	51T	68T
44G	50G	69G
45A	49A	69A
46T	50T	68T
46T	50T	66T
46T	50T	65T
46C	51C	65C
46C	51C	67C
47T	50T	66T
47G	50G	66G
47C	52C	65C
45A	51A	65A
45A	51A	67A
45A	51G	32A_33G
44G	51G	66G
46C	52C	66C
48A	53A	66A
50T	53T	65T
50G	54G	65G
51A	53A	63A
52T	53T	63T
68A_17T_18G	95A_43T_44G	84A_22T_23G
52A	57A	65A
51T	58T	65T
51G	58G	65G
53A	58A	68A
54T	58T	68T
54G	58G	68G
54A	59A	72A
53T	59T	72T
53G	59G	72G
53A	59A	70A

53T	59T	72T
54G	59G	71G
51A	58A	70A
51T	56T	69T
48A	53A	68A
48A	52A	68A
45C	53C	68C
46C	54C	69C
46A	22A_32G	70A
45T	53T	71T
46C	52C	69C
46A	50A	69A
45T	51T	70T
44G	52G	68G
42G	52G	72G
42G	52G	71G
45A	53A	71A
44G	52G	72G
44A	52A	71A
43A	52A	70A
44A	50A	72A
43G	50G	71G
46G	50G	71G
47A	52A	70A
46A	51A	72A
46G	51G	72G
45T	51T	68T
44G	51G	64G
44G	49G	63G
44A	51A	63A
45G	50G	63G
44A	50A	63A
45G	51G	62G
45A	50A	61A
45A	49A	61A
45A	50A	61A
46G	50G	61G
46C	32T_18C	63C
45C	2A_18T_29C	39T_25C
44G	49G	66G
43C	49C	65C
42A	48A	68A
21A_21T	46A	37A_28T

42A	48A	64A
41C	48C	64C
43G	48G	65G
43G	48G	63G
42G	48G	62G
42C	48C	61C
42T	48T	61T
42T	46T	65T
43G	46G	65G
43A	47A	65A
42A	48A	65A
42A	48A	65A
42T	48T	64T
41T	48T	64T
17A_34T_17G_58C	47C	20A_40T_20G_85C
42G	47G	68G
41T	44T	68T
43A	44A	69A
43C	45C	70C
42T	46T	69T
42T	46T	62T
42T	45T	62T
24T_19C	44C	62C
43A	42A	61A
43A	41A	60A
43G	42G	63G
43T	27T_15G	28T_35G
42G	42G	63G
43C	41C	64C
43T	39T	64T
43C	37C	64C
43T	38T	64T
43A	38A	66A
43A	38A	66A
46A	37A	68A
46A	37A	68A
46A	37A	67A
46C	38C	69C
46C	38C	70C
47C	38C	67C
47T	39T	67T
47G	39G	67G
46A	39A	66A

45T	39T	66T
44C	39C	69C
43C	39C	69C
44C	40C	69C
44G	41G	70G
44C	39C	71C
45T	40T	72T
45T	41T	71T
45T	41T	72T
46T	40T	71T
47C	18G_22C	72C
46C	41C	72C
45A	41A	73A
44C	41C	71C
43G	40G	71G
42A	39A	70A
40A	40A	68A
42A	39A	68A
42T	40T	65T
42A	38A	66A
26A_16G	38A	35A_31G
42T	38T	65T
40A	38A	64A
40A	38A	65A
41T	36T	66T
41A	36A	67A
41A	36A	68A
40T	35T	68T
40C	37C	67C
40A	36A	65A
40G	36G	65G
40G	17A_20G	64G
39G	36G	67G
39C	36C	67C
40T	37T	65T
39G	37G	67G
40T	37T	66T
40G	38G	66G
40A	36A	64A
39T	37T	66T
39T	39T	67T
37T	38T	67T
38G	39G	68G

38A	40A	70A
38G	40G	68G
39C	40C	67C
40C	39C	67C
40A	40A	66A
40G	39G	68G
40G	41G	69G
39C	39C	67C
39G	39G	70G
39C	39C	67C
39T	41T	66T
40T	41T	67T
40T	40T	67T
38G	41G	66G
39C	39C	65C
39A	39A	66A
39C	39C	64C
39A	40A	65A
39T	40T	65T
39G	41G	65G
39T	40T	64T
38T	39T	63T
37C	40C	65C
36A	40A	67A
35G	41G	71G
34G	41G	70G
34A	41A	71A
34A	41A	69A
34T	41T	68T
35A	42A	68A
35C	42C	67C
35T	42T	64T
36G	42G	64G
36T	43T	64T
36C	40C	65C
37T	39T	64T
16T_19C	39T	65T
35A	38A	64A
35G	37G	66G
35T	37T	67T
35G	37G	67G
35A	37A	69A
16T_19C	36T	70T

35C	36C	69C
35G	35G	69G
35G	36G	71G
20G_15C	36G	36G_32C
15T_20C	36C	32T_36C
35T	36T	70T
35A	36A	69A
20T_15C	36C	70C
34T	36T	70T
34C	34C	68C
34T	36T	68T
33A	21A_16G	34A_35G
33A	37A	71A
33G	39G	71G
33G	40G	73G
34T	41T	73T
34T	41T	71T
34G	41G	72G
35A	41A	72A
34A	41A	72A
36G	41G	71G
36G	41G	72G
36C	41C	72C
37T	41T	71T
37G	42G	69G
35G	42G	70G
35C	42C	69C
35T	42T	68T
36T	43T	68T
35T	44T	67T
36A	43A	69A
34T	43T	69T
35A	43A	68A
36C	43C	67C
37C	43C	67C
38G	45G	65G
38A	45A	64A
37A	43A	66A
38A	42A	66A
38G	42G	67G
38C	42C	67C
38C	41C	67C
38T	41T	68T

37G	40G	69G
37A	40A	68A
37G	38G	66G
37T	38T	67T
39A	38A	67A
39A	38A	68A
39C	37C	65C
38G	39G	64G
37G	39G	61G
38T	40T	61T
38A	40A	60A
38A	40A	60A
38T	40T	60T
38A	40A	60A
37A	40A	60A
35A	40A	60A
35T	40T	60T
36A	42A	58A
38A	42A	58A
38T	41T	58T
39A	41A	58A
39A	41A	59A
38T	41T	58T
39T	41T	59T
19A_20T_1G	40T	63A
40A	16A_25T	61A
40A	42A	61A
41A	43A	63A
41A	43A	62A
43A	43A	61A
43A	42A	63A
44G	13A_1T_25G	65G
44A	26A_13T	64A
43A	39A	65A
43T	40T	65T
43A	40A	67A
41T	15T_26C	68T
40A	41A	68A
40C	40C	68C
40T	39T	66T
40G	38G	68G
19T_21C	38T	66T
40A	38A	67A

39G	38G	67G
39A	36A	69A
39T	36T	68T
39C	36C	67C
39A	36A	67A
39G	36G	66G
39C	38C	66C
39T	38T	67T
39T	37T	68T
39T	37T	68T
41A	36A	68A
40G	36G	67G
40T	36T	69T
40T	37T	70T
40T	37T	68T
40C	36C	68C
43T	36T	69T
43C	35C	69C
43T	35T	69T
43T	35T	67T
42C	12T_23C	67C
42G	35G	67G
42A	35A	66A
41A	35A	64A
41G	34G	64G
41T	34T	65T
40G	34G	63G
40G	33G	64G
40T	35T	62T
40G	36G	62G
40G	36G	60G
40T	36T	60T
41T	37T	61T
41T	37T	60T
43G	37G	60G
43C	37C	60C
23A_20T	36A	60A
42C	36C	60C
42T	36T	60T
43C	36C	60C
43A	35A	60A
43G	36G	62G
43A	37A	65A

44A	38A	65A
44A	37A	65A
44G	36G	65G
44A	34A	63A
44A	33A	61A
44G	33G	62G
43G	31G	61G
43T	32T	59T
42C	32C	55C
43C	32C	55C
44C	33C	54C
41A	34A	52A
42G	35G	51G
42G	37G	52G
41G	38G	50G
41A	38A	49A
42A	38A	48A
42A	38A	47A
42T	38T	47T
41A	37A	47A
41T	36T	28A_19T
40T	37T	47T
40T	36T	48T
38T	37T	50T
38G	36G	47G
37T	37T	48T
37T	35T	47T
37T	35T	48T
37T	34T	44T
36T	35T	44T
36A	35A	45A
35C	34C	46C
35A	35A	48A
35A	36A	48A
35A	36A	48A
36G	37G	49G
36G	37G	48G
35T	37T	49T
33C	37C	51C
33T	38T	49T
33T	37T	47T
33C	37C	48C
34T	37T	48T

33T	37T	47T
33C	37C	47C
33C	37C	48C
33A	37A	51A
33T	37T	50T
33T	37T	53T
33A	38A	54A
33C	38C	54C
33T	36T	54T
31T	36T	53T
31T	36T	53T
30T	36T	51T
30C	36C	51C
30G	35G	51G
29G	35G	51G
29A	34A	51A
29G	32G	50G
30A	32A	51A
28A	31A	52A
28A	31A	51A
26G	31G	50G
27C	31C	51C
27T	31T	51T
28C	32C	52C
28T	32T	52T
31G	31G	48G
31A	33A	48A
16T_15C	14T_18C	48T
31C	32C	47C
32A	32A	47A
31G	32G	48G
31C	33C	48C
31C	34C	47C
31A	34A	47A
31T	34T	47T
31C	34C	49C
30T	35T	50T
30G	35G	50G
17A_13G	36G	19A_30G
29G	36G	49G
29G	34G	48G
29A	34A	50A
29A	37A	49A

29A	37A	49A
27A	38A	51A
28T	38T	52T
12A_16T	38A	29A_21T
28A	36A	50A
28A	35A	51A
28A	38A	50A
25T	38T	48T
25C	38C	48C
24T	38T	49T
25G	40G	49G
27G	39G	48G
27T	39T	49T
26A	39A	49A
26G	40G	48G
26G	42G	47G
27A	41A	45A
28A	42A	44A
28G	41G	43G
29A	42A	40A
30C	41C	41C
30C	40C	40C
30C	41C	38C
29A	40A	38A
29G	40G	38G
27G	40G	38G
27A	40A	38A
27C	40C	37C
27G	40G	37G
27C	40C	37C
26T	40T	35T
26A	40A	35A
26A	40A	33A
26A	39A	32A
27A	38A	32A
27A	38A	31A
27C	39C	32C
27G	39G	32G
25C	39C	33C
25C	38C	33C
25G	39G	34G
25C	38C	35C
25T	39T	34T

25T	40T	35T
25G	39G	35G
25G	39G	34G
25A	38A	34A
25G	36G	34G
25A	35A	33A
26G	34G	33G
25G	35G	31G
25C	35C	32C
25A	35A	33A
25G	33G	33G
25A	33A	33A
25T	33T	34T
25C	34C	34C
25T	34T	33T
26A	35A	33A
27A	35A	32A
27C	35C	33C
27A	35A	33A
27C	35C	31C
27G	35G	30G
27T	35T	32T
28C	35C	32C
28T	34T	32T
28G	34G	32G
27C	33C	32C
26A	34A	32A
26G	34G	32G
26G	34G	30G
26C	35C	29C
26T	34T	29T
26A	35A	29A
25T	34T	27T
25C	34C	27C
25G	33G	27G
25T	33T	27T
25C	33C	27C
25G	33G	29G
25G	32G	30G
25A	34A	32A
26A	33A	32A
26T	33T	31T
25A	33A	31A

25A	33A	32A
25C	34C	33C
25A	34A	33A
24G	33G	33G
25A	34A	33A
25G	34G	34G
24C	34C	34C
25A	34A	35A
25A	34A	36A
25T	34T	36T
25C	34C	36C
25T	35T	37T
24G	35G	39G
23A	35A	38A
22A	33A	37A
22G	34G	37G
19C	34C	38C
19G	32G	38G
19T	33T	38T
19G	33G	39G
18T	33T	38T
18T	33T	39T
19T	32T	39T
19A	31A	39A
19C	31C	39C
19T	31T	38T
19C	31C	38C
19G	30G	38G
19G	30G	39G
18A	29A	40A
18A	29A	43A
18G	29G	44G
18A	29A	45A
18A	26A	46A
18A	26A	49A
18A	25A	48A
17A	26A	46A
17G	26G	45G
18C	26C	45C
18G	26G	44G
18A	23A	44A
13A_6G	6A_17G	44A
19T	23T	43T

20A	23A	43A
20A	21A	43A
19C	21C	42C
19A	20A	44A
19A	20A	44A
19G	20G	42G
19C	19C	43C
19T	18T	42T
18C	19C	42C
17A	19A	42A
16A	18A	42A
15C	18C	43C
15A	18A	43A
16A	19A	43A
16G	19G	44G
16G	19G	44G
15A	19A	45A
16C	19C	44C
16T	19T	45T
16T	19T	46T
16T	20T	44T
16G	20G	42G
16A	20A	39A
17G	20G	38G
17G	20G	38G
17T	20T	37T
17C	20C	38C
17C	20C	37C
16C	17G	36C
17C	20C	36C
17T	20T	36T
17C	19C	36C
18T	19T	36T
18T	18T	36T
17C	18C	35C
17C	18C	34C
17T	17T	34T
17A	17A	34A
17A	17A	33A
17T	17T	30T
16T	17T	30T
16T	16T	30T
16C	16C	31C

16C	16C	30C
15A	15A	30A
16C	16C	30C
16T	15T	29T
16C	13C	30C
16T	13T	29T
15T	14T	28T
14G	14G	28G
14A	14A	28A
14G	14G	28G
14A	14A	27A
14C	14C	26C
14A	14A	25A
13C	14C	24C
13A	14A	21A
14A	13A	20A
14A	13A	19A
14G	12G	19G
14A	12C	6A_1G_10C
15G	13G	33G
15G	12G	20G
15G	12G	21G
14C	10G_11C	20C
15G	12G	22G
16G	12G	24G
16G	13G	22G
16G	13G	25G
16G	13G	25G
16G	14G	26G
16G	14G	26G
14G	13G	24G
13C	13C	24C
12G	13G	26G
16A	13A	25A
17A	12A	25A
18C	12C	23C
18G	12G	11G_13C
18A	11A	24A
18G	12G	24G
19A	12A	23A
20G	12G	23G
20T	12T	23T
19T	12T	20T

18C	12C	20C
18T	11T	20T
18A	11A	20A
19G	11G	20G
19C	11C	20C
18C	11C	21C
19A	11A	22A
19C	11C	22C
19G	10G	23G
19G	11G	23G
19G	11G	23G
19A	11A	25A
19G	11G	25G
19G	11G	24G
19T	11T	24T
19T	12T	25T
19T	12T	25T
19T	12T	26T
19G	12G	26G
20C	12C	26C
20A	13A	26A
20T	13T	26T
20T	13T	27T
20C	13C	27C
20A	13A	27A
20C	13C	27C
20A	12A	26A
20C	14C	26C
20G	14G	27G
20T	16T	28T
19G	16G	28G
19C	16C	28C
19A	16A	29A
18G	16G	29G
18G	16G	30G
17G	17G	30G
17A	17A	29A
17G	17G	30G
16G	17G	31G
17C	16C	31C
17G	17G	31G
17T	9T_8C	30T
16G	19G	29G

16T	19T	27T
16A	19A	27A
16T	20T	25T
16T	19T	26T
16T	19T	26T
15G	18G	25G
15G	19G	26G
15C	19C	27C
15T	19T	25T
15T	19T	24T
15T	18T	25T
15T	18T	26T
15C	18C	25C
6T_9C	10T_8C	11T_14C
15T	18T	24T
15T	18T	24T
15T	17T	23T
15T	17T	23T
15C	17C	23C
15T	17T	23T
15A	17A	23A
15C	17C	23C
15C	17C	23C
15A	17A	23A
15A	17A	23A
15G	17G	22G
15A	18A	22A
15C	19C	22C
15T	19T	22T
15G	20G	22G
15G	20G	23G
15G	20G	22G
15G	20G	23G
16G	20G	12T_11G
16T	19T	24T
17G	19G	25G
16G	19G	25G
8A_8G	19G	25G
16G	19G	23G
16T	19T	24T
15G	19G	28G
16G	19G	28G
16G	19G	29G

16G	19G	29G
16G	19G	29G
16A	19A	26A
16G	19G	29G
16G	19G	30G
16G	19G	31G
15A	19A	30A
14A	19A	30A
14G	19G	30G
14G	18G	30G
13A	18A	32A
13T	18T	32T
14T	18T	30T
14G	18G	28G
13A	18A	29A
13T	19T	30T
13G	18G	30G
13G	18G	30G
13A	19A	29A
13T	18T	28T
13C	19C	28C
13G	19G	30G
13G	19G	31G
13A	19A	31A
13G	19G	32G
13G	19G	30G
13T	17T	32T
13G	21G	33G
13C	21C	33C
13A	21A	33A
12A	21A	33A
11G	21G	33G
11A	22A	33A
11G	22G	32G
11T	22T	32T
10G	23G	33G
10A	24A	33A
9A	25A	32A
9G	26G	32G
10C	26C	32C
10A	26A	34A
10G	26G	34G
10C	26C	34C

10A	24A	34A
10A	25A	33A
10G	25G	34G
10C	25C	34C
10A	26A	35A
9A	26A	35A
9G	26G	35G
9G	26G	35G
9A	26A	35A
9A	26A	35A
8G	25G	34G
8C	25C	36C
8A	25A	36A
8G	25G	37G
9C	25C	37C
9C	24C	37C
8C	26C	37C
7T	26T	32T
7C	24C	32C
7C	24C	33C
7C	22C	34C
7C	23C	34C
7T	22T	34T
7T	23T	31T
7C	23C	30C
8C	23C	29C
9C	22C	28C
9G	22G	29G
9T	21T	27T
9C	21C	29C
9C	21C	30C
9C	20C	30C
9C	20C	29C
10C	18C	30C
10C	19C	31C
10A	19A	27A
10C	18C	32C
10C	19C	30C
10C	19C	31C
10C	19C	32C
10C	19C	32C
10G	19G	29G
10A	20A	26A

9A	20A	28A
9A	20A	30A
9G	20G	31G
9G	20G	14A_17G_1C
9C	20C	31C
9G	20G	29G
10C	20C	28C
9G	21G	28G
10G	21G	27G
10C	11A_10C	12A_15C
10C	21C	27C
10C	23C	27C
10T	22T	26T
10C	23C	28C
10C	23C	28C
10T	23T	28T
10G	23G	30G
10C	22C	30C
10G	22G	32G
10C	21C	31C
10C	21C	35C
10C	21C	35C
11C	21C	34C
10A	21A	35A
10C	21C	31C
10A	21A	33A
9C	21C	35C
9G	21G	36G
9G	23G	35G
9A	24A	35A
10G	24G	40G
10C	24C	39C
10T	24T	41T
10T	24T	41T
10G	24G	41G
11C	24C	42C
11T	25T	43T
11T	24T	44T
14A	25A	44A
14C	25C	44C
14G	26G	44G
14A	26A	42A
14T	26T	43T

14G	26G	42G
14G	26G	44G
15G	26G	44G
15A	26A	44A
15A	27A	43A
15A	28A	44A
15A	27A	44A
15C	27C	44C
16G	27G	45G
16G	26G	46G
16A	26A	47A
17A	25A	45A
17C	25C	45C
17C	25C	48C
17A	25A	47A
17C	15T_10C	48C
17A	26A	48A
17T	26T	47T
17T	24T	48T
17C	24C	48C
17G	23G	47G
17G	24G	48G
17G	24G	49G
17C	23C	48C
17A	25A	48A
16A	25A	50A
18A	24A	51A
18G	23G	51G
18A	22A	49A
19G	21G	49G
18G	22G	50G
18G	22G	50G
18G	22G	51G
18T	21T	52T
19G	23G	53G
17C	23C	54C
19A	23A	53A
19A	23A	52A
19G	22G	52G
19C	10A_21C	39A_52C
21A	21A	61A
22A	21A	60A
22A	21A	62A

22A	22A	60A
22A	21A	61A
23A	6A_15T	61A
23A	22A	61A
54A_33T	23A	99A_47T
21A	23A	52A
23A	24A	53A
23T	23T	49T
23C	24C	50C
24T	25T	51T
24T	25T	53T
25T	25T	53T
25G	26G	51G
25C	26C	52C
26T	26T	55T
27T	27T	56T
27G	27G	57G
27A	27A	55A
27A	27A	54A
27C	27C	54C
26G	27G	54G
26T	26T	55T
27A	27A	55A
30A	26A	55A
30A	27A	56A
29A	27A	54A
30C	30C	54C
30A	31A	54A
31G	31G	53G
32C	30C	56C
32A	31A	56A
32A	31A	56A
34T	32T	56T
34A	32A	56A
34C	32C	57C
35C	34C	59C
34C	34C	58C
34C	34C	59C
34T	34T	60T
34A	34A	62A
34A	35A	63A
34C	34C	62C
34G	34G	61G

34C	34C	61C
34T	34T	60T
34T	34T	59T
34A	31A	60A
33C	34C	60C
34A	33A	58A
34C	34C	57C
35A	36A	57A
35C	36C	57C
34G	36G	58G
34G	36G	59G
35A	38A	61A
35C	38C	62C
34C	38C	60C
35A	38A	59A
36G	38G	58G
36A	38A	58A
35A	37A	57A
36A	37A	57A
36C	37C	59C
36T	37T	59T
37G	36G	59G
36G	36G	59G
36A	37A	59A
36T	37T	58T
33G	36G	58G
33G	36G	57G
35G	36G	58G
35A	36A	55A
35G	18A_19G	54G
36C	37C	54C
36C	37C	53C
35C	36C	54C
35C	37C	56C
34T	37T	56T
34A	37A	55A
35G	37G	54G
35A	37A	53A
34A	37A	53A
34G	35G	54G
34G	34G	54G
33C	34C	51C
33C	34C	50C

33T	34T	50T
34C	34C	51C
34C	34C	53C
33C	34C	52C
34A	34A	52A
34A	34A	51A
34G	34G	51G
34G	35G	51G
34C	34C	51C
34T	34T	52T
34G	34G	54G
34T	35T	54T
33T	36T	53T
32G	35G	51G
32C	35C	52C
32C	35C	53C
31G	35G	54G
31G	35G	53G
31A	36A	52A
32G	36G	51G
32A	36A	51A
31T	36T	49T
31C	37C	50C
32G	36G	48G
32T	36T	47T
33C	36C	48C
33A	35A	50A
33C	36C	51C
34C	36C	51C
35C	37C	50C
35G	38G	51G
38T	39T	51T
37T	39T	51T
36C	40C	53C
38A	39A	54A
38C	39C	55C
37A	40A	54A
37C	39C	52C
37G	39G	50G
37T	39T	49T
36G	39G	51G
34C	39C	51C
34A	38A	52A

31A	36A	53A
33C	37C	52C
32C	37C	52C
32C	36C	51C
31C	37C	51C
31C	37C	54C
30G	36G	52G
30C	37C	52C
30G	37G	52G
30C	10A_28T	53C
30G	38G	54G
29G	38G	52G
30C	28T_12C	54C
29C	45C	55C
26C	44C	56C
26T	43T	56T
26C	45C	55C
26C	44C	53C
26A	41A	57A
25C	43C	57C
24C	43C	58C
24C	42C	59C
24A	42A	63A
22A	42A	64A
22C	42C	65C
22C	41C	64C
21C	39C	67C
22G	39G	66G
22G	39G	65G
23G	39G	63G
23A	38A	67A
23G	38G	67G
23T	37T	68T
29C	38C	70C
17C	38C	68C
25C	23T_38C	33T_74C
25T	13T	35T
25C	36C	72C
25C	35C	74C
26G	35G	75G
27C	36C	76C
27T	35T	77T
28C	35C	74C

27A	34A	75A
27C	34C	77C
26C	33C	76C
26T	31T	74T
27T	31T	75T
27C	31C	75C
27C	31C	74C
26A	31A	75A
26G	32G	76G
27C	32C	75C
27C	32C	76C
27T	31T	73T
26C	32C	74C
26A	32A	74A
26T	32T	76T
27G	33G	76G
27C	32C	74C
27C	32C	73C
28C	31C	39T_33C
28A	32A	71A
28C	32C	72C
27G	30G	72G
27G	31G	74G
28C	31C	73C
28C	30C	74C
29A	32A	75A
29G	31G	74G
29G	31G	74G
29C	31C	74C
29A	30A	73A
29C	32C	73C
29T	33T	72T
29T	33T	70T
29C	33C	70C
29T	33T	69T
30G	33G	70G
31G	33G	69G
30A	33A	69A
28A	34A	71A
28G	34G	72G
27C	35C	70C
28G	35G	71G
28G	36G	71G

29C	35C	70C
29A	34A	71A
30G	36G	70G
30T	35T	69T
30A	35A	70A
30G	35G	71G
32G	36G	69G
32G	36G	68G
33G	36G	68G
33C	34C	68C
32A	32A	67A
32C	32C	71C
32C	33C	70C
32T	33T	70T
32C	32C	69C
32T	32T	70T
31T	32T	68T
31G	32G	67G
30C	31C	67C
30G	32G	65G
30C	32C	65C
30T	33T	66T
26G	33G	66G
27C	32C	66C
27G	31G	68G
26T	31T	66T
26C	30C	69C
26T	31T	67T
27T	32T	67T
27G	31G	70G
28T	30T	70T
28C	30C	71C
28G	30G	70G
28A	30A	67A
28T	30T	70T
28C	31C	69C
28T	32T	71T
29T	33T	70T
29G	33G	71G
30C	34C	71C
30A	33A	72A
30G	34G	71G
30T	34T	73T

30T	33T	74T
30C	32C	77C
31T	32T	79T
31G	31G	79G
31G	30G	77G
31C	30C	77C
31T	27T	74T
31C	27C	79C
31T	27T	78T
31C	27C	76C
31G	26G	78G
32G	25G	78G
32T	25T	82T
32A	26A	83A
32C	26C	83C
32A	26A	82A
32C	26C	85C
32G	26G	86G
31T	26T	86T
30G	26G	86G
30T	26T	87T
30A	26A	87A
30A	27A	84A
30T	27T	82T
32G	28G	84G
32C	28C	85C
33T	30T	85T
34T	30T	85T
35G	30G	85G
36T	30T	85T
37T	30T	86T
37G	30G	85G
37T	31T	84T
36T	31T	82T
37C	31C	84C
36T	31T	80T
39G	32G	80G
39C	33C	79C
38A	33A	78A
38C	33C	81C
38G	33G	80G
39G	33G	80G
39T	33T	78T

39C	34C	81C
39C	34C	84C
39G	34G	82G
39C	33C	80C
39T	33T	81T
40T	34T	82T
41G	33G	82G
42A	33A	84A
42A	33A	79A
41G	33G	81G
41A	33A	84A
39A	35A	85A
41G	36G	85G
41C	36C	81C
40C	35C	81C
40C	36C	82C
39T	35T	82T
40G	35G	83G
39G	35G	82G
40A	35A	81A
42C	34C	80C
42G	34G	81G
41G	33G	81G
41G	32G	80G
40G	33G	79G
41A	33A	78A
40G	33G	77G
40A	31A	76A
41A	32A	74A
40A	31A	74A
41G	32G	74G
41C	31C	74C
42G	30G	75G
42A	29A	74A
42G	31G	71G
42C	31C	72C
42G	29G	74G
42A	29A	72A
11A_42G	28G	74G
44A	28A	71A
42A	28A	72A
41A	28A	75A
42A	29A	77A

42A	29A	78A
42A	28A	76A
44G	29G	76G
45A	29A	76A
45G	13A_16G	76G
45A	30A	75A
45T	30T	74T
45G	31G	76G
45G	31G	74G
46G	31G	73G
46G	32G	73G
46G	33G	73G
46A	31A	72A
46C	31C	71C
46T	31T	72T
48G	31G	71G
48A	31A	71A
50G	31G	71G
50A	30A	70A
49C	29C	73C
50A	28A	73A
49C	29C	75C
50G	29G	74G
51G	30G	71G
51G	30G	73G
53G	30G	74G
53A	29A	76A
53C	29C	73C
53G	29G	70G
53G	29G	70G
54A	28A	69A
54A	28A	68A
55G	27G	67G
55A	28A	66A
56A	27A	63A
56G	27G	64G
55G	27G	66G
56C	26C	62C
55C	27C	62C
56A	27A	62A
56C	28C	63C
56G	28G	65G
57G	28G	65G

57A	28A	64A
57T	28T	64T
56A	28A	62A
56T	16A_12T	28A_35T
81T_25C	15A_21T_9C	30A_53T_21C
56T	27T	67T
57C	28C	66C
58T	27T	67T
58C	28C	68C
58T	30T	68T
58C	29C	67C
59T	28C	32T_34C
60C	32C	66C
60T	30T	65T
66T_93C	32C	67C
58T	32T	66T
58C	33C	67C
57T	31T	69T
43T	30T	61T
46T	31T	68T
45T	31T	68T
42C	29C	65C
108T	31T	65T
49T	32T	66T
50T	32T	63T
50T	32T	63T
49T	32T	63T
50T	31T	62T
50T	31T	62T
49C	32C	63C
49A	32A	64A
50G	31G	63G
50T	30T	63T
50C	31C	63C
50G	30G	63G
50C	32C	64C
22A_50T_22G_22C	33T	64T
49A	33A	64A
49T	33T	64T
47T	32T	65T
46T	33T	64T
47G	34G	63G
46G	34G	63G

47A	34A	61A
47G	36G	62G
46A	35A	64A
47A	34A	60A
47A	34A	59A
48T	34T	58T
48G	34G	59G
48C	35C	60C
50A	35A	60A
51C	35C	59C
49T	35T	59T
50T	35T	59T
50T	35T	59T
50A	36A	60A
51T	36T	60T
25T_26C	37C	61C
51T	37T	62T
50A	37A	65A
50T	37T	65T
51G	37G	62G
51C	37C	61C
52T	37T	62T
53C	39C	61C
54A	40A	61A
54G	41G	63G
55G	41G	62G
54A	42A	63A
55A	42A	65A
54C	43C	62C
56A	43A	63A
54C	43C	63C
54G	41G	62G
52C	42C	62C
52T	42T	63T
53C	43C	65C
50G	43G	64G
50C	44C	64C
50C	44C	65C
50T	44T	65T
50T	43T	65T
49T	43T	64T
49T	42T	63T
49C	40C	64C

25T_24C	40T	66T
49C	41C	67C
47A	41A	68A
47G	41G	67G
46G	41G	65G
46C	29A_11C	65C
44G	41G	64G
43T	41T	62T
44G	41G	62G
44C	41C	60C
42T	41T	61T
42A	42A	61A
43T	43T	59T
24A_13T	43A	59A
13T_24C	42T	32T_26C
36C	42C	60C
36C	41C	60C
36C	41C	60C
36A	41A	59A
36G	41G	59G
36A	41A	61A
36G	41G	62G
36T	42T	62T
13A_23G	42G	61G
23A_13C	41A	61A
36A	41A	62A
36G	42G	63G
35G	42G	65G
13A_22G	41G	69G
35G	41G	67G
35T	40T	67T
39T	40T	70T
39G	40G	70G
39G	39G	72G
40T	38T	73T
40G	38G	71G
17T_23C	26T_11C	70C
39G	38G	69G
38G	37G	73G
39T	37T	69T
40C	39C	70C
39A	40A	71A
38G	40G	70G

37A	39A	69A
37T	39T	66T
37C	38C	65C
36A	38A	66A
38T	38T	68T
37C	37C	66C
37T	37T	65T
37T	36T	66T
36A	37A	66A
37G	37G	66G
37T	38T	63T
37C	38C	62C
36T	38T	63T
36C	37C	31G_33C
37T	37T	65T
35C	37C	67C
37T	36T	67T
36C	36C	67C
36G	36G	68G
36G	36G	68G
36T	35T	71T
37T	37T	70T
37T	38T	70T
38A	38A	71A
40A	39A	71A
40A	42A	70A
41C	42C	69C
41C	41C	66C
41C	41C	67C
42C	42C	67C
44T	42T	68T
45T	43T	67T
44A	42A	67A
45A	42A	70A
45T	43T	72T
46T	41T	72T
46G	41G	75G
44C	40C	74C
46T	40T	74T
46C	41C	74C
45T	41T	74T
46T	40T	77T
45T	42T	75T

43A	42A	77A
43A	42A	79A
43A	41A	81A
43A	42A	84A
44T	41T	83T
44A	42A	85A
43C	42C	88C
43C	42C	87C
43C	41C	88C
44T	42T	91T
44T	41T	90T
44A	41A	85A
45C	44C	84C
43A	43A	85A
42A	42A	85A
43T	42T	84T
43T	41T	85T
42A	41A	82A
41T	41T	80T
41T	40T	81T
40A	40A	85A
40A	42A	85A
40T	43T	81T
41T	42T	84T
41C	42C	84C
41A	41A	83A
40C	41C	84C
40C	40C	84C
39T	41T	84T
39A	42A	85A
39T	42T	89T
39T	44T	89T
39G	45G	90G
39A	46A	90A
39G	46G	88G
40T	46T	86T
40A	46A	84A
40G	46G	85G
40G	46G	86G
40T	46T	85T
40A	47A	86A
41G	47G	86G
40G	47G	83G

40T	47T	84T
40C	48C	83C
40A	47A	79A
39C	47C	82C
39T	47T	79T
39A	47A	79A
38A	48A	79A
38C	48C	79C
38G	48G	79G
36C	48C	82C
37G	48G	83G
38A	47A	85A
38G	47G	83G
38T	48T	82T
37A	48A	81A
39A	48A	84A
39T	48T	86T
39T	48T	83T
40T	51T	81T
40A	52A	85A
39A	52A	84A
39T	51T	85T
39T	52T	84T
42A	53A	84A
41T	53T	83T
41G	54G	82G
42C	55C	83C
42T	55T	83T
43A	55A	81A
43A	55A	80A
44T	56T	76T
44C	55C	77C
45G	53G	75G
45C	54C	73C
45C	53C	73C
46G	54G	71G
46C	54C	70C
48T	53T	71T
46T	53T	71T
48C	53T	70T
50A	53A	71A
50G	53G	70G
51C	52C	69C

50T	53T	71T
51A	52A	71A
51A	54A	71A
51C	54C	72C
51G	56G	68G
49A	56A	67A
50A	56A	67A
48G	56G	66G
47G	56G	66G
48G	57G	68G
47A	57A	67A
48A	57A	69A
47A	56A	69A
47T	56T	69T
46G	57G	68G
44G	57G	67G
44G	55G	68G
43C	54C	68C
43A	54A	68A
43T	55T	68T
43A	55A	70A
42A	55A	69A
41G	55G	68G
41A	56A	68A
41G	54G	68G
41G	54G	68G
40G	53G	67G
41G	53G	43A_23G
39T	53T	66T
39C	53C	69C
39T	55T	66T
39C	55C	66C
38C	55C	69C
38C	53C	68C
38T	53T	68T
38T	52T	70T
37T	54T	67T
37C	55C	66C
36A	55A	63A
36G	54G	63G
36G	53G	63G
36A	53A	62A
36A	53A	60A

11T_24C	53C	57C
35T	55T	57T
35C	56C	59C
34G	54G	53G
34G	53G	51G
33G	55G	50G
32G	54G	50G
32A	54A	50A
32A	53A	50A
32G	53G	49G
32G	52G	53G
32A	52A	51A
21G_11C	51C	46T_96G_138C
32G	50G	21G
31C	50C	20C
31C	51C	21C
31C	52C	21C
32C	52C	22C
31T	52T	22T
33C	51C	23C
33C	50C	23C
33G	49G	23G
33T	48T	23T
33G	22A_24G	50G
11A_22T	21A_24T	51T
33T	46T	53T
32C	46C	52C
32A	45A	52A
32C	47C	52C
32T	47T	53T
32T	47T	55T
32C	46C	55C
31A	48A	56A
31G	48G	56G
31A	48A	55A
31T	47T	56T
31G	46G	57G
29C	46C	57C
19A_10G	46G	27A_29G
29A	46A	57A
29A	46A	57A
29A	47A	57A
29G	47G	57G

29C	21A_26C	58C
29A	47A	58A
27A	47A	57A
28C	49C	59C
28G	48G	59G
28A	51A	58A
27G	51G	57G
28A	53A	59A
28C	52C	60C
28G	49G	59G
28G	48G	58G
28T	48T	57T
29T	48T	61T
29C	48C	60C
25C	48C	60C
25T	48T	60T
26G	47G	62G
25G	47G	64G
24G	47G	65G
25G	47G	62G
25C	46C	62C
24A	47A	63A
23T	48T	63T
22C	48C	62C
22G	49G	62G
23A	49A	63A
22T	48T	64T
22T	48T	63T
20A	47A	64A
20C	47C	65C
18G	46G	64G
16T	47T	64T
15G	48G	66G
15T	48T	65T
14T	22T_26C	42T_23C
13T	47T	65T
12T	46T	64T
12T	46T	62T
12T	45T	62T
12T	44T	65T
12T	44T	64T
12T	44T	65T
12A	43A	56A

13A	42A	59A
13A	97A	124A
13A	46A	67A
13C	45C	66C
13G	46G	65G
13T	45T	66T
13C	44C	66C
13G	43G	68G
12G	43G	67G
13A	43A	67A
13A	43A	68A
12A	42A	68A
12G	42G	68G
13A	40A	69A
13T	41T	70T
12T	41T	70T
12T	41T	68T
12T	39T	69T
11C	39C	70C
11C	40C	70C
11G	40G	70G
11C	40C	70C
11T	40T	69T
11T	38T	68T
11A	39A	67A
11A	39A	65A
11G	39G	64G
11C	39C	62C
11C	37C	62C
11G	38G	61G
9T_11G	37G	58G
11T	36T	58T
9T	35C	57C
10C	35C	57C
10G	35G	56G
10A	33A	55A
10A	32A	54A
9A	31A	30A_24G
10C	16T_15C	52C
10C	31C	49C
10C	31C	48C
9A	30A	47A
10A	31A	46A

10G	31G	44G
10C	30C	42C
9C	29C	42C
10G	30G	42G
10C	30C	42C
6A_7T_5G_16C	48A_56T_40G_78C	39C
9T	22T	38T
8C	22C	38C
8C	22C	37C
7A	20A	33A
7A	21A	32A
7T	20T	28T
7A	20A	28A
7T	19T	28T
7C	19C	28C
7T	18T	28T
7C	18C	24C
7A	19A	23A
7T	18T	23T
7C	7A_10C	22C
7T	16T	20T
7T	17T	19T
7G	17G	19G
7G	15G	17G
7A	15A	17A
7G	15G	16G
7G	15G	16G
7C	15C	17C
7G	14G	17G
7G	14G	13G
8C	12C	16C
8T	12T	17T
8C	12C	16C
9C	13C	17C
9A	13A	17A
9A	14A	18A
9T	14T	18T
9A	14A	18A
9T	15T	19T
9C	15C	19C
9T	16T	19T
9C	18C	19C
9A	18A	19A

9T	18T	17T
9C	10A_8C	19C
4T_5C	17T	19T
9T	18T	20T
9G	18G	21G
9G	18G	22G
9A	18A	23A
9G	18G	23G
9G	18G	23G
9C	19C	24C
9G	19G	24G
9G	19G	25G
10A	20A	25A
10T	23T	27T
10G	23G	28G
10A	23A	29A
10T	23T	30T
10G	24G	30G
10A	25A	31A
10G	25G	31G
10A	26A	31A
10G	26G	31G
10A	26A	31A
11T	26T	32T
11G	27G	32G
11T	28T	32T
11A	28A	32A
5T_11G	9T_28G	11T_32G
11T	28T	31T
6T_5C	41C	19T_11C
11C	13C	30C
13C	28C	29C
13C	28C	30C
14C	28C	31C
14C	28C	32C
14C	28C	33C
14C	27C	30C
16C	28C	31C
16C	28C	31C
16T	28T	32T
16T	28T	32T
16T	28T	32T
17C	28C	33C

17T	28T	31T
6A_12G	16A_12G	31A
19G	28G	29G
18C	28C	29C
18C	28C	29C
19A	26A	29A
19G	27G	28G
19C	28C	30C
19A	28A	30A
19C	27C	30C
19C	27C	30C
20C	27C	30C
20C	27C	32C
21T	27T	33T
21T	28T	35T
21A	28A	35A
21G	29G	36G
21G	30G	36G
21A	30A	37A
21G	30G	37G
21A	31A	37A
21C	32C	38C
22A	30A	39A
22G	31G	42G
21C	31C	42C
22T	30T	42T
22G	30G	44G
20C	30C	45C
20G	29G	46G
20T	30T	46T
20G	30G	46G
20G	30G	46G
20A	31A	45A
21G	31G	45G
21A	30A	45A
21C	31C	45C
21T	31T	45T
21T	32T	46T
21T	33T	45T
7A_14G	33G	45G
21C	34C	42C
21G	34G	42G
22C	35C	42C

21T	35T	40T
21C	35C	40C
22T	36T	39T
22G	36G	40G
21A	36A	41A
21T	35T	41T
21A	36A	42A
20C	35C	42C
20C	35C	42C
20C	35C	43C
21A	34A	44A
21C	35C	45C
24A	34A	46A
24G	39G	45G
24G	41G	45G
24A	41A	45A
25T	41T	44T
25C	41C	43C
25G	42G	45G
25G	44G	46G
24G	44G	47G
24A	44A	46A
24G	45G	47G
23T	44T	46T
24A	45A	24A_22G
24G	45G	46G
24C	45C	45C
24A	45A	46A
24T	44T	46T
25C	44C	45C
25C	44C	46C
25C	43C	46C
25C	43C	48C
25G	42G	48G
26G	40G	47G
26C	40C	47C
25G	40G	47G
26C	40C	44C
26T	41T	44T
26T	42T	41T
26C	42C	38C
26T	42T	38T
26T	42T	38T

27T	42T	38T
27T	42T	40T
28G	40G	39G
27C	41C	38C
27G	41G	38G
27C	41C	37C
27C	38C	36C
27G	37G	32G
27T	37T	33T
28A	37A	32A
28T	36T	33T
28C	35C	31C
28C	36C	31C
28T	37T	30T
28A	37A	30A
19A_9T	19A_18T	29A
29C	38C	31C
29G	36G	30G
29G	37G	29G
29C	37C	29C
29C	37C	28C
29A	37A	28A
26C	37C	27C
27G	37G	27G
26T	37T	28T
26T	37T	29T
26T	37T	30T
26A	38A	30A
24A	37A	30A
24A	37A	30A
24A	37A	31A
24C	37C	30C
25A	37A	31A
24C	37C	9A_19C
25A	37A	26A
8T_16C	37C	26C
23C	36C	7A_28C
23C	37C	27C
23G	37G	26G
22C	37C	27C
22A	36A	27A
22A	36A	27A
22T	36T	28T

22C	36C	27C
22T	36T	26T
21G	37G	26G
21G	37G	25G
19A	36A	25A
19G	35G	26G
19G	36G	25G
19A	37A	25A
18G	35G	25G
18A	35A	25A
14G	36G	24G
17A	36A	24A
17A	35A	23A
16A	36A	25A
15A	38A	25A
15A	37A	25A
14A	37A	25A
14C	38C	26C
14T	38T	27T
14C	39C	28C
14C	39C	29C
14G	39G	31G
14C	39C	31C
54T	39G	27G
1T	70T	26T
null	5T	29T
null	39T	30T
null	40T	30T
null	39T	28T
null	39T	28T
9A	38T	28T
9T	38T	27T
9T	37T	28T
9T	35T	30T
36A_9T	36T	7A_28T
14A	36A	44A
14A	36A	45A
14A	36A	43A
14T	36T	44T
14T	36T	44T
14G	37G	46G
14A	37A	45A
14T	38T	46T

14T	37T	45T
14T	37T	46T
14G	37G	47G
14A	40A	47A
14A	41A	52A
14T	41T	53T
14T	42T	56T
14A	38A	56A
14G	36G	58G
14G	36G	58G
15G	37G	57G
14C	36C	57C
14A	35A	58A
14A	35A	58A
14T	36T	58T
14T	35T	56T
14C	36C	58C
14G	37G	57G
14T	37T	57T
13T	37T	57T
8A_13T	37T	33A_57T
18A_19T	37T	23A_33T
13A	37A	58A
13A	38A	58A
13A	38A	60A
13A	40A	60A
13A	41A	60A
12A	42A	58A
12A	43A	58A
14A	43A	60A
14C	44C	54C
14T	43T	53T
14T	44T	52T
14T	44T	53T
13A	44A	54A
13G	44G	54G
13G	46C	24G_30C
13A	46A	55A
13A	47A	55A
14A	46A	55A
14C	45C	54C
14C	46C	54C
14T	44T	54T

14C	45C	54C
15C	45C	53C
15C	46C	52C
18C	46C	52C
19A	45A	51A
19A	47A	50A
19A	46A	49A
11A_7C	47A	48A
18G	48G	46G
18T	48T	45T
18A	47A	45A
18T	47T	45T
18G	46G	45G
18C	46C	44C
18C	45C	45C
18T	47T	46T
20A	47A	45A
20A	48A	43A
20A	48A	42A
21G	49G	42G
21G	49G	43G
21G	48G	44G
21T	48T	44T
20G	48G	45G
20T	48T	44T
19A	48A	44A
19A	48A	44A
20G	48G	44G
20C	46C	45C
20T	47T	43T
19C	47C	43C
20C	47C	40C
20T	47T	40T
19C	48C	41C
19G	48G	41G
19G	47G	41G
19G	47G	39G
20G	47G	40G
20C	46C	40C
19A	47A	39A
19A	46A	40A
19C	47C	42C
19A	47A	42A

19C	46C	43C
19C	44C	44C
18T	45T	44T
18T	44T	46T
18T	44T	46T
18T	44T	45T
18C	46C	46C
18C	44C	47C
18T	42T	45T
18A	42A	46A
18T	42T	46T
18T	41T	47T
17T	43T	47T
17G	42G	25A_21G
17T	42T	46T
17G	43G	48G
18C	43C	48C
19A	43A	47A
19T	40T	47T
19A	43A	47A
19G	43G	47G
19C	43C	47C
19T	43T	46T
19G	42G	49G
19C	42C	49C
19G	19A_23G	30G_19C
19G	41G	49G
19C	42C	49C
19G	42G	50G
19T	42T	50T
19G	42G	50G
12A_7G	42G	50G
19C	41C	51C
19A	41A	49A
19A	41A	49A
19T	43T	49T
19C	43C	51C
20A	42A	50A
20C	40C	52C
20A	40A	51A
20C	39C	51C
20A	40A	52A
19C	41C	52C

19C	42C	54C
19A	41A	55A
19C	43C	57C
19A	43A	57A
19C	42C	55C
19G	41G	55G
19A	41A	54A
19C	41C	55C
19T	40T	55T
19C	20T_19C	56C
18A	19A_20G	58A
18T	40T	57T
18T	42T	56T
19T	42T	54T
19A	42A	54A
19A	42A	54A
19A	41A	54A
20T	41T	52T
20A	40A	51A
20A	39A	50A
18T	38T	49T
17A	37A	49A
17G	37G	49G
17A	37A	49A
18C	37C	47C
18G	37G	46G
18C	36C	48C
18A	35A	45A
18A	35A	44A
18C	34C	43C
19G	66G	21T_36G
19G	18G	29G
19G	18G	49G
19G	17A_49G	48G
20G	37G	48G
19G	36G	48G
18G	37G	48G
15G	36G	48G
9A_22G	37G	42G
14G	36G	42A_58G_1C
11A	34C	48C
12A	34A	50A
12G	33G	53G

12C	33C	53C
13T	35T	52T
14G	35G	55G
14C	35C	56C
14T	35T	54T
14T	36T	54T
14T	35T	55T
14A	36A	58A
14T	36T	58T
14T	36T	57T
14C	36C	58C
15C	36C	58C
15A	36A	58A
16C	37C	57C
15A	37A	57A
16C	38C	60C
17C	37C	59C
17A	38A	59A
17G	20A_18G	60G
17T	38T	60T
17G	38G	61G
11T_6G	38G	61G
17C	37C	61C
17G	18A_19G	62G
17G	37G	59G
17G	37G	58G
16A	38A	58A
16C	38C	57C
16C	38C	59C
16T	38T	58T
17A	38A	58A
19A	37A	57A
19A	38A	58A
19G	39G	61G
19A	39A	58A
20A	40A	60A
20G	40G	61G
22T	39T	61T
22C	40C	61C
22C	41C	31G_31C
22T	41T	62T
23C	40C	64C
23G	40G	65G

23G	38G	68G
23A	38A	68A
24A	38A	69A
24G	38G	69G
9T_1G_14C	37T	69T
23C	36C	68C
22C	36C	68C
23C	36C	66C
23A	36A	68A_28G
24G	37G	36T
24A	37A	71A
24G	37G	68G
24A	37A	69A
24T	35T	71T
24T	33T	70T
24C	35C	72C
24C	35C	73C
24T	36T	71T
24C	37C	74C
25T	37T	73T
25G	38G	74G
26A	38A	72A
26G	38G	75G
26C	38C	75C
26A	38A	73A
26T	37T	71T
25G	37G	74G
25T	38T	73T
25A	37A	70A
25C	38C	71C
25A	38A	72A
25A	37A	72A
25A	35A	72A
26G	35G	74G
26C	35C	73C
26G	35G	74G
26C	35C	72C
26G	35G	70G
26C	36C	71C
26A	34A	68A
26G	35G	70G
27C	36C	70C
27A	36A	68A

13G_14C	35G	35G_34C
28G	34G	69G
27T	34T	70T
26C	35C	70C
28T	36T	69T
28T	36T	70T
27G	34G	70G
27G	33G	69G
27C	34C	68C
27C	34C	68C
27C	34C	68C
27C	33C	69C
27A	30A	65A
26C	32C	66C
14T_12C	31T	33T_31C
26C	32C	64C
26C	32C	63C
26G	32G	61G
26A	32A	55A
25G	32G	56G
24C	32C	56C
24C	32C	56C
24G	32G	26G_26C
24G	32G	52G
24C	30C	53C
23C	29C	52C
38A_75G_30C	51A_110G_44C	57A_30G_12C
7G	7G	42G
null	1A	24A
null	null	23A
null	null	25T
null	null	28C
null	null	29G
null	null	29G
null	null	29G
17A	24A	40A
17G	24G	39G
17A	24A	38A
17G	25G	37G
17G	26G	21A_13G
17A	26A	34A
17G	26G	34G
17C	26C	34C

18T	26T	34T
18G	26G	35G
18T	26T	20A_16T
18G	29G	37G
19A	29A	35A_15C
21C	29C	41C
21C	29C	43C
22C	29C	45C
22C	29C	21T_27C
23C	30C	48C
23T	30T	47T
23A	31A	48A
24A	31A	48A
25C	30C	48C
25T	31T	48T
25G	30G	49G
24G	30G	48G
24G	29G	48G
24G	30G	47G
24T	30T	47T
24G	30G	49G
24C	30C	23T_27C
24T	30T	50T
24G	31G	49G
24A	32A	48A
24G	32G	48G
25C	32C	25T_24C
25T	32T	49T
25C	31C	50C
25T	30T	53T
24A	31A	53A
24G	32G	54G
24A	31A	54A
24T	29T	56T
24C	30C	55C
23C	29C	56C
23C	29C	58C
22A	29A	57A
22T	27T	57T
22C	28C	55C
8A_14C	28C	58C
21T	27T	58T
21C	28C	58C

21C	28C	58C
21T	28T	58T
20C	28C	59C
20C	28C	60C
20G	27G	58G
13A_7G	28A	58A
20G	28G	59G
20C	28C	57C
20C	28C	58C
19A	27A	58A
19G	27G	57G
19C	27C	57C
19A	27A	55A
20G	27G	53G
36A_20G_24C	45A_27G_30C	60A_51G_40C
8G	24A_7G_12C	29G
8C	1C	28C
8C	null	27C
8A	null	27A
8A	null	29A
20G	14G	50G
19G	15G	51G
24C	6G_15C	57C
24T	22T	57T
24C	22C	58C
25A	22A	59A
25C	22C	61C
25G	22G	61G
25G	22G	61G
25C	22C	60C
25T	23T	58T
25C	23C	60C
25A	23A	61A
25T	23T	59T
25G	23G	58G
25G	22G	58G
25A	22A	58A
25A	22A	60A
25T	23T	59T
24C	24C	60C
26T	24T	58T
26G	25G	57G
26G	25G	57G

26A	25A	56A
28G	25G	57G
28T	24T	58T
28C	23C	56C
26C	23C	56C
26A	25A	57A
27T	25T	58T
26C	25C	59C
26A	25A	59A
26A	24A	59A
26C	23C	57C
26A	23A	57A
28T	22T	56T
28C	22C	56C
28T	22T	56T
28G	22G	55G
28G	22G	55G
28A	21A	55A
29G	21G	54G
29G	20G	52G
29G	20G	51G
28C	20C	19A_1T_30C
28A	20A	51A
27G	20G	51G
8G_19C	20C	49C
27A	20A	49A
29A	20A	47A
29T	19T	46T
29C	19C	45C
29T	19T	49T
29C	19C	49C
29A	19A	53A
29A	19A	53A
29A	19A	52A
29T	19T	50T
29T	19T	51T
28C	17C	50C
29T	17T	50T
29T	16T	49T
29T	16T	48T
28A	16A	51A
29A	16A	52A
28T	16T	49T

27T	15T	50T
26A	15A	50A
27A	15A	50A
27G	15G	48G
26G	15G	47G
26C	15C	47C
26T	14T	48T
26A	13A	47A
26C	14C	45C
25C	14C	44C
25T	14T	43T
25C	14C	43C
23T	13T_4C	43T_43C
26C	14C	71C
26C	14C	46C
26C	14C	46C
26C	14C	39C
25C	14C	40C
25C	7T_21C	40C
25C	14C	41C
7T_17C	13T	20T_15C
19T_5C	10T	20T_14C
19T_5C	12T	39T
24T	11T	40T
24T	11T	40T
24T	11T	40T
24T	11T	42T
24T	11T	43T
37T_32C	8T_2C	21T_25C
21C	10C	44C
21C	10C	48C
21T	10T	48T
21T	10T	48T
21T	10T	48T
21T	10T	48T
21G	10G	47G
21G	10G	46G
21T	10T	49T
20T	10T	52T
20T	10T	52T
20C	9C	54C
20A	11A	53A
20A	11A	56A

20A	11A	55A
20C	11C	54C
19T	3T_8G	55T
19G	11G	52G
19A	11A	48A
18T	11T	51T
17G	11G	53G
17T	11T	54T
17A	11A	52A
17T	11T	54T
16G	11G	51G
16C	11C	50C
16C	11C	46C
16T	11T	45T
15C	11C	46C
3G_12C	11G	46G
15A	11A	43A
14A	11A	44A
14A	11A	43A
14T	10T	43T
14A	10A	43A
14C	9C	41C
14C	10C	39C
12A	10A	39A
14A	10A	38A
14A	10A	38A
14A	9A	38A
14A	9A	38A
13A	7A	37A
2A	1T	1A
2A	7A_3T_1G_9C	null
2T	1T	1T
null	1G	1G
null	1G	2G
4G	4G	10G
4G	4G	10G
4G	4G	10G
5T	4T	11T
6T	4T	11T
6T	4T	11T
6A	4A	11A
6T	4T	11T
6G	4G	11G

6C	4C	11C
16G	4G	11G
16C	1G_3C	null
17G	4G	null
6G_11C	2G_2C	null
20G	7G	14G
21G	7G	15G
21G	7G	16G
21G	6G	16G
21G	7G	18G
21G	7G	19G
21G	15G	19G
9T_12G	5T_4G	19G
21T	7T	19T
22G	8G	19G
22A	8A	20A
22G	8G	20G
22C	8C	22C
22T	8T	24T
22G	3T_5G	24G
23C	8C	25C
23G	8G	25G
23T	8T	28T
23G	3A_5G	28G
25T	11T	29T
26T	11T	30T
27G	11G	32G
27G	13G	32G
27C	13C	32C
28C	13C	32C
28A	13A	33A
29T	14T	33T
29C	14C	33C
29C	14C	33C
29G	14G	33G
29T	15T	34T
16A_13G	11A_5G	34A
29G	16G	36G
30T	17T	37T
30T	18T	37T
30T	18T	39T
31A	18A	39A
31A	18A	39A

31C	18C	40C
31A	18A	41A
31G	19G	42G
31C	19C	42C
31T	20T	42T
31G	21G	42G
31G	21G	42G
31G	21G	44G
33A	21A	44A
33G	21G	43G
33G	21G	44G
33C	21C	46C
35C	21C	47C
36G	22G	46G
36A	22A	44A
36A	22A	46A
36C	22C	47C
36G	22G	46G
35A	23A	45A
36G	23G	47G
35G	23G	45G
35C	23C	47C
35C	21C	47C
36G	22G	47G
36A	22A	48A
36A	22A	48A
20A_16C	21C	26A_22C
35T	22T	51T
35G	22G	51G
33A	22A	54A
31A	22A	55A
30A	22A	58A
30A	22A	57A
31T	22T	57T
31C	22C	58C
33G	7A_15G	59G
33C	22C	58C
33C	22C	59C
33C	23C	61C
36C	23C	61C
36A	24A	62A
35A	24A	64A
36T	25T	64T

37A	25A	65A
36A	25A	63A
36A	25A	62A
36T	25T	59T
36T	25T	59T
35G	16T_9G	59G
37T	25T	55T
36C	26C	57C
37A	24A	57A
37A	24A	57A
38C	25C	55C
38G	25G	57G
38A	26A	57A
38C	27C	58C
38G	28G	58G
37T	28T	60T
36T	27T	62T
37T	27T	63T
37A	26A	65A
37A	26A	65A
36T	25T	65T
37A	25A	63A
35A	25A	63A
35A	25A	63A
35A	26A	62A
35G	26G	62G
36T	27T	61T
36T	27T	60T
36C	27C	65C
35C	27C	64C
34C	26C	64C
34A	28A	64A
34C	29C	64C
34A	29A	64A
33A	29A	63A
33G	28G	62G
33G	29G	62G
31G	29G	62G
31A	29A	60A
31A	28A	59A
31A	28A	58A
31G	29G	58G
27A_58T_27G	10A_39T_10G	36A_93T_36G

33G	31G	65G
33T	32T	65T
33A	32A	65A
29T	31T	48T_15G
33T	33T	64T
32A	34A	63A
32T	34T	63T
29G	32G	14T_55G_1C
33A	35A	66A
30C	33C	57C
30T	33T	58T
30G	33G	58G
30G	33G	59G
30G	32G	60G
30A	33A	60A
30A	34A	58A
30T	35T	58T
29G	36G	57G
28A	34A	58A
28C	33C	58C
28G	33G	60G
27T	33T	60T
29T	33T	59T
30T	32T	57T
30C	31C	58C
30C	31C	58C
29C	30C	57C
29T	30T	57T
29A	30A	57A
28G	30G	56G
28G	29G	56G
80A_26G	74A_24G	140A_45G
2A	2A	5A
2A	1A	5A
2T	1T	5T
28A	25A	50A
29A	25A	50A
29A	25A	50A
28T	25T	46T
33A	17A_9T	51A
34A	27A	53A
34A	27A	53A
34A	28A	54A

35G	8A_1T_19G	54G
36A	28A	54A
36A	29A	54A
37A	28A	54A
36G	29G	54G
35G	28G	55G
38G	30G	57G
38T	28T	57T
39A	29A	57A
39T	29T	56T
39T	29T	57T
38T	30T	56T
37T	30T	55T
37T	30T	51T
37A	30A	51A
36G	30G	50G
36T	30T	52T
36G	30G	52G
36G	30G	50G
36G	30G	50G
35G	29G	50G
35G	30G	50G
35A	30A	50A
36A	31A	50A
38G	31G	51G
39G	32G	52G
39T	32T	52T
37T	32T	53T
37T	32T	50T
38T	32T	49T
39G	33G	50G
38A	34A	49A
39G	33G	49G
39C	32C	48C
38A	32A	47A
37A	32A	46A
37T	32T	45T
38T	31T	45T
38G	32G	16A_25G
38C	34C	41C
38T	34T	39T
39A	33A	39A
39A	33A	39A

39A	33A	37A
40T	34T	37T
39A	33A	38A
38A	33A	41A
38G	32G	41G
38G	32G	40G
38A	32A	38A
37T	32T	40T
38A	31A	38A
38A	31A	38A
38T	31T	39T
37T	30T	39T
37A	32A	38A
36T	33T	38T
36T	34T	36T
36A	34A	38A
37G	34G	37G
38T	34T	37T
36G	34G	35G
36G	33G	36G
37G	34G	36G
37G	33G	36G
37G	33G	37G
38A	33A	39A
39G	32G	37G
39G	32G	37G
38T	31T	36T
39T	31T	37T
40T	32T	38T
40T	32T	40T
40G	32G	41G
41T	32T	41T
42T	32T	39T
44T	32T	38T
44A	33A	38A
44A	33A	37A
43T	31T	39T
43T	32T	38T
42G	31G	38G
42T	30T	38T
43T	29T	38T
43G	28G	38G
43C	28C	38C

44A	27A	36A
45T	30T	36T
45A	30A	37A
45G	28G	37G
45G	31G	36G
46A	31A	37A
47T	31T	41T
47A	32A	41A
47A	33A	42A
46T	33T	41T
46T	33T	42T
46A	33A	42A
46C	32C	42C
47T	32T	42T
47G	32G	44G
47G	32G	44G
48G	33G	45G
46G	32G	40G
46T	34T	27T_17G
46T	34T	46T
45T	33T	46T
43T	32T	46T
43T	34T	48T
43T	33T	49T
41A	30A	45T
42A	33A	49A
43T	34T	50T
43G	34G	51G
44T	34T	51T
44T	35T	50T
43T	34T	47T
42A	33A	46A
41A	32A	47A
41G	33G	46G
41A	32A	44A
40C	32C	46C
42A	31A	45A
43C	32C	44C
43T	32T	44T
42T	31T	45T
42C	32C	46C
40C	33C	46C
39G	33G	24A_22G

39C	33C	48C
41T	32T	48T
41T	33T	48T
41T	32T	47T
41C	32C	48C
41T	31T	47T
41C	32C	47C
41C	33C	45C
20T_21G	33T	45T
41C	34C	45C
41T	34T	44T
42G	34G	46G
42G	34G	45G
42C	34C	44C
43C	34C	42C
41T	34T	42T
39C	34C	42C
37A	33A	42A
37G	33G	44G
37T	33T	44T
37A	33A	43A
38G	33G	46G
38G	32G	46G
37G	31G	46G
36G	32G	48G
35G	31G	50G
34G	17A_15G	51A
34C	32C	52C
17A_17G	32A	52A
34T	31T	51T
34C	31C	51C
34C	29C	51C
33A	29A	51A
32G	29G	48G
33C	29C	49C
34C	29C	47C
33C	28C	48C
33T	28T	48T
34C	27C	47C
34T	29T	45T
34G	29G	45G
34C	30C	45C
34C	30C	47C

34C	30C	45C
33C	30C	45C
33A	30A	46A
33C	30C	46C
33A	30A	46A
34G	29G	46G
34T	28T	45T
34G	28G	43G
34G	28G	44G
33G	28G	45G
33C	28C	45C
33T	27T	44T
32C	27C	44C
32C	27C	45C
32T	27T	49T
31T	28T	49T
30T	29T	48T
30C	29C	49C
31T	29T	49T
31G	30G	48G
30C	29C	48C
30C	28C	48C
30C	29C	49C
29C	29C	50C
29A	29A	52A
28C	29C	52C
28G	29G	50G
28G	29G	49G
28G	29G	51G
28T	29T	51T
29G	29G	52G
29C	31C	52C
28A	31A	54A
28A	31A	53A
28A	31A	53A
28G	31G	54G
27T	28T	54T
27C	28C	54C
27C	28C	52C
27G	27G	52G
26C	27C	52C
26G	27G	51G
26C	25C	51C

26C	25C	51C
26C	25C	51C
26T	14T_10C	49T
26T	23T	50T
26T	23T	51T
25C	23C	49C
25C	23C	49C
25A	23A	49A
24C	22C	44C
26G	20G	46G
26C	21C	46C
26C	21C	46C
26T	21T	45T
26G	21G	45G
25C	20C	46C
25C	20C	46C
25A	20A	46A
25A	20A	46A
24G	19G	10A_35G
24G	19G	45G
23G	18G	45G
24A	18A	47A
24A	18A	46A
24G	8A_7G	46G
24A	6T_8G	45A
24A	14A	46A
23G	14G	44G
23A	14A	43A
21C	14C	44C
20A	14A	44A
19T	8T_6C	9T_34C
19C	14C	43C
19A	8A_6G	9A_34G
18A	13A	44A
19C	14C	43C
19C	14C	43C
17G	6T_8G	33T_10G
19G	15G	46G
19C	15C	46C
19A	15A	46A
19G	15G	47G
19G	15G	45G
19C	15C	46C

19T	14T	45T
19C	14C	45C
19C	9T_5C	44C
17G	14G	44G
18A	13A	44A
20G	14G	43G
20C	13C	40C
19G	13G	43G
20C	14C	43C
20C	14C	40C
20C	14C	40C
20A	13A	42A
20T	13T	41T
19C	13C	41C
18C	13C	41C
18A	12A	40A
18G	12G	40G
18A	12A	39A
18G	13G	38G
18A	13A	39A
18T	13T	39T
18G	13G	41G
18C	8T_5C	23T_18C
19T	13T	42T
19G	13G	43G
19C	13C	43C
20G	13G	44G
19G	13G	44G
19G	14G	46G
19G	14G	46G
18C	14C	47C
18G	14G	46G
18C	14C	46C
18A	13A	45A
18A	13A	45A
18A	13A	47A
18C	13C	47C
18C	14C	46C
19T	14T	50T
19G	13G	50G
17T	13T	49T
17T	13T	50T
17T	13T	51T

16G	13G	51G
16G	13G	52G
16A	13A	54A
16T	13T	53T
16G	13G	53G
16G	13G	50G
16C	12C	50C
16G	11G	50G
16C	11C	49C
16G	11G	47G
15C	10C	46C
15A	10A	47A
15A	10A	48A
15G	11G	47G
15G	11G	45G
15A	11A	45A
15G	11G	45G
14A	11A	43A
14A	10A	44A
14T	10T	45T
14C	10C	45C
6T_8G	10G	30T_20G
13T	9T	50T
14C	8C	49C
14C	8C	49C
14C	8C	48C
13A	6A_1G_1C	46A
13C	8C	47C
13C	8C	47C
13C	8C	48C
14A	8A	47A
14C	7C	48C
13T	8T	49T
15C	8C	49C
16T	8T	49T
16C	8C	48C
18G	8G	48G
17A	8A	48A
17A	7A	46A
18G	7G	47G
18G	7G	48G
18T	6T	47T
19G	7G	48G

17G	7G	47G
18A	7A	48A
18A	8A	48A
18C	8C	48C
18A	8A	48A
18A	8A	46A
18G	8G	47G
18C	8C	48C
17C	8C	48C
17G	8G	47G
17G	8G	47G
13T_4C	7C	46T
16C	8C	47C
16A	8A	46A
16G	8G	45G
16A	8A	44A
16T	8T	44T
16T	8T	44T
16A	8A	43A
16T	8T	41T
16T	8T	41T
15G	8G	53G
15G	8G	26G
15G	9G	38G
15G	9G	39G
15G	9G	38G
15G	9G	37G
15G	9G	38G
15G	10G	35G
16G	10G	12G_23C
17G	10G	34G
19C	10C	34C
18A	10G	11A_22G_1C
22C	9C	34C
22C	10C	36C
23C	10C	36C
25A	10A	36A
25G	10G	36G
26G	10G	36G
26T	10T	34T
25T	10T	34T
24T	10T	36T
24T	10T	37T

25T	10T	37T
25C	10C	36C
7T_18C	8T_2C	20T_17C
25C	10C	37C
25T	10T	37T
26C	10C	37C
26T	11T	37T
26T	11T	35T
26C	12C	33C
26C	12C	34C
26T	12T	35T
26T	12T	33T
26G	12G	35G
26G	12G	35G
27G	12G	34G
26G	11A	1A_21T_12G
26T	12T	31T
26G	13G	37G
24G	13G	35G
24G	13G	35G
24G	13G	35G
23T	13T	34T
25G	14G	35G
25G	14G	38G
25G	14G	38G
24A	14A	39A
26G	14G	39G
26T	14T	40T
28T	14T	39T
29A	14A	39A
28G	14G	40G
28G	16G	41G
30A	17A	40A
31A	17A	41A
31A	17A	41A
31A	17A	39A
31C	17C	39C
32A	17A	40A
33G	17G	39G
33G	16G	37G
33G	19G	37G
32T	18T	38T
33G	19G	38G

33A	19A	37A
33A	20A	38A
33G	20G	38G
33G	20G	39G
32C	19C	38C
33C	19C	40C
33A	19A	41A
33A	19A	41A
33C	19C	40C
33A	19A	41A
33C	19C	43C
33C	18C	42C
33C	18C	42C
33A	18A	42A
33T	19T	44T
32T	19T	44T
32T	19T	45T
33T	19T	45T
33A	19A	44A
33G	19G	45G
32G	19G	45G
31T	18T	46T
30T	18T	46T
31A	17A	49A
31A	17A	48A
29T	17T	48T
29T	17T	48T
30C	17C	48C
29C	17C	48C
30A	17A	47A
29T	18T	47T
29G	17G	46G
29T	17T	47T
28G	17G	46G
28G	17G	46G
28A	17A	45A
28C	17C	45C
28T	16T	45T
29G	16G	49G
29G	17G	48G
29G	17G	47G
30A	17A	48A
30T	17T	50T

30A	17A	50A
14A_16G	18G	30A_17G
30C	18C	48C
30A	18A	48A
30T	18T	48T
30T	18T	49T
30A	18A	48A
31A	19A	46A
32A	20A	45A
31A	20A	44A
18T_13C	20C	42C
31A	19A	41A
31A	20A	41A
31A	20A	40A
31A	20A	39A
31T	20T	38T
8A_21T_3C	21T	29A
25A	20C	27A
26A	20A	29A
29A	19A	28A_29T
29A	19A	30A
27A	19A	30A
26A	19A	32A
6A_20T	19T	33A
25A	20A	51A
25T	20T	51T
25A	20A	53A
23T	20T	53T
23T	21T	55T
23T	21T	56T
24T	21T	57T
24T	21T	58T
23C	21C	58C
24C	21C	60C
13A_10T	22A	60T
23G	22G	60G
23T	23T	61T
23T	24T	61T
23A	24A	63A
23A	24A	64A
23A	23A	63A
23A	23A	65A
23T	22T	65T

23A	22A	66A
23A	22A	65A
23G	22G	65G
24G	22G	65G
23G	22G	66G
23A	22A	68A
25G	25G	65G
25C	25C	65C
25A	24A	66A
25T	24T	65T
25A	24A	66A
26G	25G	64G
27A	25A	68A
28C	25C	67C
28G	25G	69G
28G	25G	69G
27A	25A	68A
27C	25C	67C
18T_8C	25T	31T_1G_35C
25C	24C	65C
24A	24A	67A
23A	24A	64A
23C	23C	65C
22A	22A	66A
14A_7G	21A	32A_36G
21C	22C	67C
21A	23A	67A
20A	23A	66A
20T	23T	66T
6A_13G	23A	66A
20C	24C	67C
20A	22A	67A
20G	22G	67G
21A	22A	66A
21A	22A	65A
22T	22T	63T
22T	23T	64T
22T	24T	63T
22T	24T	62T
22T	24T	58T
7A_16T	24T	30A_23T
23A	25A	52A
23A	24A	51A

23A	24A	51A
23G	25G	21T_28G
23C	25C	48C
24T	25T	43T
25T	25T	48T
18T_7G	25T	26T_22G
25T	25T	50T
25A	25A	52A
18T_7G	25T	26T_26G
25A	25A	57A
25A	25A	57A
25A	25A	58A
25G	26G	57G
25T	26T	57T
25T	26T	57T
25A	26A	56A
25A	26A	55A
24A	26A	55A
6T_18C	25C	30T_25C
24T	27T	56T
24A	27A	56A
24A	26A	58A
24G	27G	56G
24A	27A	55A
25T	27T	55T
26T	27T	54T
25T	27T	54T
9T_18C	27T	25T_27C
27C	28C	52C
26C	28C	51C
25C	27C	52C
26T	27T	51T
25G	28G	51G
25G	28G	52G
25G	28G	51G
25C	26C	50C
25G	27G	51G
25G	27G	51G
25G	27G	52G
25G	27G	51G
25G	26G	51G
24A	26A	51A
24A	25A	50A

24G	25G	51G
25A	25A	51A
25G	26G	51G
25A	28A	54A
25A	28A	53A
26C	27C	56C
11T_16C	27C	56C
26C	27C	55C
26G	27G	53G
26C	27C	54C
10G_15C	27G	55G
24T	27T	57T
25G	26G	57G
26A	28A	60A
26A	29A	58A
26A	28A	60A
25A	28A	59A
25T	28T	60T
26A	27A	60A
26T	26T	60T
26T	26T	62T
25A	26A	63A
25A	26A	62A
25T	26T	61T
25T	26T	61T
25C	25C	62C
26G	25G	31A_26G
26T	25C	54C
26A	24A	56A
27A	24A	58A
27A	24A	61A
26A	25A	63A
26A	25A	66A
26A	25A	65A
27A	25A	62A
26G	25T	62T
28T	25T	63T
27A	26A	64A
27G	27G	63G
27T	26T	63T
26T	24T	63T
7T_18C	24C	62C
26C	24C	62C

26C	25C	62C
26A	26A	62A
25T	25T	62T
24T	25T	63T
23A	26A	63A
23A	26A	61A
24A	27A	61A
24A	28A	60A
24G	28G	60G
24G	28G	61G
30T_24G	10T_28G	63G
24T	27T	65T
24T	30T	67T
24T	31T	68T
24T	31T	72T
24T	31T	71T
24T	31T	69T
30T	31T	68T
22C	31C	69C
22C	32C	68C
22C	32C	68C
22G	31G	67G
22A	31A	65A
22C	31C	67C
21G	31G	67G
22G	31G	66G
22G	30G	68G
22T	28T	69T
22C	29C	70C
21G	29G	71G
21C	32C	68C
21T	32T	68T
21A	31A	67A
21A	32A	67A
20A	32A	70A
21C	31C	69C
20C	31C	67C
21C	31C	66C
21A_35C	30A	67A
14C	30C	67C
14C	30C	65C
14C	14G_16C	66C
14A	30A	64A

14A	30A	67A
21A	31A	68A
22T	15T_15C	70T
15T_8C	30T	72T
24G	30G	70G
24G	30G	69G
24C	30C	70C
25G	30G	70G
24T	30T	69T
24T	30T	69T
23G	29G	71G
24T	30T	72T
24T	30T	72T
25T	29T	72T
26T	29T	70T
26A	29A	70A
26T	28T	70T
26T	28T	71T
26T	28T	72T
26C	27C	73C
27T	27T	72T
27A	26A	71A
27T	27T	71T
26A	27A	72A
26C	27C	71C
26T	28T	72T
25G	28G	73G
26A	27A	73A
27T	27T	72T
27T	29T	73T
26T	29T	72T
26T	30T	73T
18A_8C	30C	74C
26T	30T	77T
26T	30T	80T
26A	30A	80A
26T	31T	80T
26T	29T	81T
25T	29T	81T
25G	29G	80G
23A	29A	45A_34C
13T_23G	28G	74G
23T	29T	74T

24T	29T	76T
24T	29T	78T
17T_8C	29C	77C
25T	30T	76T
24T	29T	77T
25T	30T	78T
25T	30T	78T
24T	31T	78T
22C	31C	78C
21T	31T	77T
21T	31T	74T
21A	31A	73A
21A	31A	73A
22T	31T	73T
21C	31C	74C
22A	32A	76A
22T	32T	75T
23T	32T	77T
24C	30C	76C
24G	32G	76G
25G	32G	79G
25T	31T	79T
25C	30C	77C
24T	32T	77T
23C	32C	76C
24A	33A	75A
24T	34T	76T
24T	33T	74T
24T	34T	73T
26A	33A	71A
26T	34T	68T
26T	36T	69T
26T	36T	69T
26C	36C	68C
26T	36T	66T
26A	36A	69A
26A	36A	66A
24A	34A	67A
24C	35C	68C
24G	34G	68G
24G	33G	67G
24A	34A	69A
24C	34C	68C

24T	35T	67T
24A	35G	65G
24A	35A	65A
24G	33G	64G
24C	33C	65C
25G	33G	65G
25A	33A	67A
8T_17G	32T	67T
25C	32C	67C
25T	31T	68T
26C	31C	69C
26T	32T	71T
26A	32A	72A
26A	32A	72A
26A	33A	72A
25T	34T	72T
9A_16T	35A	72A
25A	35A	71A
25T	35T	71T
25A	35A	71A
25T	34T	70T
25G	34G	70G
25A	35A	67A
25C	36C	68C
27C	36C	68C
26G	36G	69G
28G	36G	70G
27G	36G	34A_34G
27A	36A	66A
27C	36C	67C
27A	38A	67A
27G	38G	66G
27T	40T	64T
27G	40G	63G
27G	41G	62G
25C	39C	63C
26T	38T	65T
25T	40T	67T
25A	40A	67A
25A	40A	67A
24T	40T	69T
25T	40T	68T
25T	41T	66T

25A	41A	65A
25C	41C	63C
25G	40G	62G
24C	40C	63C
23C	40C	60C
9A_14G	40G	33A_27G
23G	40G	59G
23A	40A	59A
23A	40A	59A
23G	40G	59G
22G	40G	58G
21A	40A	57A
21A	39A	57A
71T	55T	114T
2T	25T	47T
2T	40T	46T
7T	39T	41T
19T	38T	61T
19T	37T	62T
7A_12T	37T	59T
19A	40A	62A
19A	39A	61A
19A	40A	61A
19A	41A	61A
19A	40A	61A
19A	40A	59A
14A_21G	37A	59G
25A	38G	57A
49A	39A	102A
13A	39A	43A
13A	40A	41A
13G	40G	43G
26A	40A	70A
26A	40A	73A
26A	40A	73A
26A	43A	72A
27A	43A	71A
29A	43A	72A
26G	44G	71G
26A	43A	69A
27A	42A	71A
26C	42A	41A_29C
26A	42A	70A

26C	41C	71C
25G	41G	69G
26C	40C	69C
26A	41A	64A
26A	41A	63A
25T	41T	62T
27C	41C	62C
27C	41C	61C
27A	41A	62A
27G	41G	61G
28A	40A	60A
28T	40T	61T
27T	40T	59T
27C	42C	61C
28G	44G	61G
28G	44G	61G
13A_13G	43G	53A
26A	44A	52A
26C	44C	52C
25T	44T	53T
25T	45T	54T
25T	45T	57T
25G	45G	58G
25C	45C	58C
27A	43A	59A
28A	43A	61A
28A	43A	64A
28C	44C	63C
26T	45T	62T
26G	45G	63G
26A	46A	63A
26A	46A	67A
26G	45G	69G
26G	46G	69G
24A	46A	69A
24T	46T	69T
24A	45A	69A
24A	45A	69A
24C	45C	70C
24T	44T	65T
25G	45G	67G
25C	45C	68C
25C	44C	69C

25A	45A	70A
25A	47A	73A
25T	46T	74T
26C	46C	75C
26G	46G	78G
26A	45A	79A
27A	45A	78A
27G	44G	77G
27T	45T	78T
27G	44G	72G
26A	44A	71A
27C	44C	71C
27T	43T	69T
27C	43C	70C
28C	41C	69C
28G	40G	66G
29A	41A	66A
29A	40A	67A
29T	40T	68T
30T	40T	69T
30G	40G	69G
30C	38C	68C
30G	38G	69G
29G	37G	71G
29T	36T	70T
12T_1G_15C	39T	70T
29A	39A	71A
29G	38G	69G
29C	37C	68C
29T	37T	68T
29G	37G	71G
29C	37C	74C
29A	37A	77A
30C	37C	78C
30T	37T	77T
30T	37T	79T
30T	37T	82T
29G	37G	81G
14T_16G	37G	80G
31A	38A	81A
30T	38T	79T
31C	38C	79C
31C	39C	79C

31T	39T	78T
29T	39T	80T
30T	39T	80T
30G	40G	80G
30A	40A	79A
29A	39A	78A
28A	40A	77A
28G	40G	77G
28G	41G	75G
28A	41A	76A
14A_15G	39A	74A
29C	38C	76C
30C	39C	76C
29T	39T	72T
30C	41C	73C
30G	40G	68G
30G	41G	69G
31G	41G	71G
31A	40A	70A
30A	41A	69A
30A	42A	68A
30G	42G	68G
12G_17C	42C	67C
29G	42G	70G
17A_12G	45A	70A
31C	46C	70C
31C	43C	70C
31T	43T	70T
29A	43A	68A
31T	41T	68T
32A	43A	68A
32T	44T	65T
32T	45T	65T
33A	46A	66A
33C	46C	65C
33C	46C	65C
34A	46A	68A
34G	46G	68G
34C	47C	69C
35G	46G	70G
34G	46G	72G
34C	47C	72C
35A	47A	72A

35A	47A	73A
34G	46G	70G
33G	47G	70G
33T	46T	69T
33T	47T	68T
34T	46T	70T
33A	46A	71A
33T	46T	70T
33A	43A	71A
33T	45T	71T
32T	47T	73T
33C	46C	73C
33T	45T	75T
33C	44C	74C
33A	43A	72A
31C	41C	69C
31G	43G	66G
31G	43G	66G
30T	44T	66T
32C	42C	65C
32T	41T	64T
32T	41T	65T
32T	41T	69T
32C	41C	68C
32T	40T	69T
33C	40C	69C
33T	40T	70T
33A	41A	70A
33G	41G	70G
35A	42A	71A
35A	44A	72A
35C	44C	74C
35T	44T	72T
35A	45A	39A_33G
35C	45C	72C
36G	43G	73G
37G	43G	72G
36A	45A	71A
38T	44T	67T
39C	45C	73C
38T	46T	74T
38C	46C	75C
38C	45C	76C

39A	45A	73A
39C	44C	71C
39G	45G	72G
39A	45A	73A
40A	46A	73A
38A	46A	72A
40A	45A	72A
41A	46A	69A
41C	46C	67C
41C	47C	68C
39G	47G	70G
18A_21G	20A_25G	70G
39A	45A	69A
39T	45T	69T
18T_21C	20T_25C	69C
39G	45G	70G
40A	46A	69A
39C	46C	69C
39G	46G	69G
40C	46C	68C
41C	46C	65C
41G	46G	65G
41G	46G	64G
42A	47A	64A
41T	46T	61T
42T	47T	61T
42G	47G	62G
41C	46C	62C
42A	47A	61A
41G	46G	60G
40A	45A	62A
40T	45T	62T
39G	44G	60G
39T	45T	59T
39A	45A	61A
39A	45A	59A
37C	46C	60C
38C	44C	57C
39C	45C	58C
40T	45T	57T
40G	45G	58G
38G	45G	59G
39C	45C	59C

39G	45G	62G
38C	45C	61C
39C	44C	59C
38C	44C	60C
39A	44A	58A
38G	43G	57G
39A	43A	52A
38T	43T	53T
38T	43T	52T
38C	43C	51C
39G	44G	53G
38C	43C	54C
38T	42T	54T
38G	44G	54G
39G	45G	53G
39G	42G	55G
38A	41A	55A
39A	41A	55A
38C	41C	55C
39C	39C	56C
36A	40A	57A
35A	40A	57A
36A	39A	57A
35G	39G	55G
35C	38C	53C
35G	38G	56G
36C	38C	57C
33A	39A	58A
33G	39G	58G
34A	38A	58A
35A	38A	58A
35A	38A	59A
35T	37T	59T
34T	37T	61T
34T	37T	61T
34A	37A	62A
35G	38G	61G
35A	38A	58A
35G	36G	57G
35A	35A	58A
35A	35A	61A
35A	36A	61A
36T	36T	61T

36T	37T	61T
37T	35T	60T
37A	35A	60A
37A	35A	61A
38A	35A	60A
38A	35A	60A
38G	37G	63G
37A	37A	62A
37A	38A	63A
37G	36G	63G
36G	36G	61G
36C	36C	61C
38G	36G	62G
38A	37A	63A
38A	37A	61A
37G	37G	61G
37T	37T	62T
37C	38C	62C
38A	37A	60A
38A	36A	61A
37G	36G	62G
37A	34A	62A
37G	34G	64G
37T	33T	66T
37T	34T	65T
37A	34A	65A
36C	34C	66C
35T	33T	64T
35C	33C	63C
34C	32C	61C
33G	14A_19G	28A_33G
33G	32G	61G
33G	32G	60G
33A	32A	61A
33C	33C	61C
33T	32T	62T
32A	32A	63A
32A	31A	59A
31A	32A	58A
33T	32T	56T
33A	32A	57A
32A	31A	57A
32T	31T	53T

32A	31A	53A
32A	31A	53A
32C	31C	53C
32C	31C	53C
32T	31T	53T
31T	31T	53T
30C	18C	30C
18T_12C	18T	30T
30G	19G	29G
31T	20T	29T
30C	21C	30C
30T	21T	31T
30C	20C	31C
19A	21A	30A
20A	21A	30A
20T	21T	30T
19T	19T	30T
19C	20C	28C
19G	20G	27G
19A	20A	27A
19G	20G	27G
19A	20A	27A
19G	32G	51G
34G	32G	51G
34G	32G	52G
34C	31C	55C
34T	31T	55T
34C	31C	56C
34A	31A	56A
37A	31A	56A
37T	31T	57T
37T	32T	58T
37C	32C	61C
37G	32G	65G
37A	32A	65A
37G	31G	64G
37A	32A	64A
37G	32G	66G
37T	31T	67T
38C	31C	68C
37G	31G	67G
37A	31A	68A
37A	32A	66A

37A	33A	68A
38A	32A	67A
41A	33A	68A
41C	34C	68C
42C	33C	66C
42G	34G	68G
42G	34G	66G
42C	34C	64C
42T	34T	63T
43T	33T	62T
43C	33C	63C
42G	32G	63G
42A	33A	63A
41A	33A	63A
41A	34A	63A
43C	35C	63C
43G	35G	64G
43C	34C	63C
43A	34A	63A
43C	34C	60C
43G	34G	57G
44G	35G	60G
43A	35A	60A
43A	35A	59A
43A	35A	57A
43G	35G	56G
28T_14C	35T	1A_33T_26C
43C	34C	60C
43C	34C	60C
44C	35C	61C
46C	35C	59C
46G	35G	56G
46C	35C	56C
45G	35G	55G
46G	34G	56G
47G	34G	57G
46C	34C	25T_32C
46T	34T	56T
44C	35C	56C
43T	35T	56T
44A	34A	56A
44T	33T	56T
44T	32T	55T

46A	32A	57A
46G	32G	27A_29G
46T	33T	57T
46A	34A	59A
44T	33T	59T
44T	33T	60T
44A	34A	60A
44C	35C	61C
44C	34C	59C
43G	34G	56G
44C	35C	57C
43T	35T	57T
44G	35G	57G
44C	35C	58C
44T	37T	57T
44G	36G	58G
44C	36C	55C
44G	35G	50G
45G	36G	50G
44T	35T	50T
44T	34T	51T
44A	35A	50A
46A	36A	48A
46T	36T	49T
46A	35A	50A
45T	36T	52T
45T	36T	52T
45A	37A	50A
46A	38A	52A
47T	37T	51T
46A	38A	51A
46G	39G	52G
46C	39C	50C
45A	38A	54A
46T	39T	55T
47T	39T	56T
45G	41G	55G
45T	42T	54T
45A	43A	53A
45T	43T	52T
47T	42T	55T
47A	41A	54A
47A	41A	55A

47T	42T	54T
47A	41A	53A
46A	43A	54A
46T	42T	54T
46A	41A	54A
45A	41A	52A
47T	41T	53T
48G	42G	54G
48A	42A	56A
50C	42C	55C
49G	43G	51G
49A	44A	50A
50T	45T	50T
53G	44G	52G
52A	45A	52A
54T	45T	53T
55A	45A	54A
56A	46A	57A
56T	47T	59T
56A	47A	58A
54G	47G	58G
54C	47C	58C
54A	46A	61A
54G	46G	62G
53T	46T	61T
54A	46A	62A
55G	46G	62G
55C	46C	60C
55A	46A	60A
54G	46G	59G
53C	46C	58C
53G	46G	60G
55G	46G	60G
57C	46C	60C
57A	87A_21T_21G_21C	60A
57A	24A	60A
54C	23C	60C
54A	22A	60A
53G	22G	61G
53T	22T	62T
54A	45A	60A
55A	45A	62A
55C	43C	62C

55A	43A	63A
55G	44G	64G
55T	44T	65T
55A	44A	68A
55A	46A	67A
56T	45T	66T
54C	46C	67C
55G	46G	67G
56C	47C	70C
56A	47A	67A
57A	47A	67A
57C	46C	67C
55A	46A	65A
56G	46G	66G
57C	46C	68C
57A	46A	67A
57A	46A	68A
57T	46T	65T
56A	46A	66A
56G	46G	66G
29A_26C	46C	68C
53G	46G	68G
53G	45G	68G
52C	46C	68C
53A	46A	68A
52A	47A	71A
50G	47G	70G
52A	47A	70A
52A	47A	72A
52C	46C	73C
52G	46G	73G
52A	46A	75A
52A	46A	76A
55A	46A	76A
52A	48A	78A
52C	50C	79C
53G	50G	78G
54A	49A	82A
54T	50T	82T
55T	50T	82T
56T	49T	82T
57A	49A	82A
57A	49A	81A

57G	49G	80G
58C	49C	78C
60G	50G	74G
61T	51T	74T
59T	52T	74T
61A	49A	75A
61A	49A	73A
61G	48G	74G
59T	48T	77T
62G	49G	76G
62G	49G	76G
62C	50C	76C
64C	50C	42T_35C
62A	50A	77A
62C	50C	77C
62A	49A	77A
62A	48A	77A
62T	48T	77T
62T	46T	76T
61G	46G	77G
27T_34C	46C	77C
60C	45C	76C
59A	47A	75A
58T	49T	76T
59C	49C	78C
59A	48A	77A
59T	49T	77T
59T	47T	78T
60T	45T	77T
61C	45C	77C
61T	45T	74T
59G	45G	73G
61T	46T	74T
61T	46T	76T
61T	45T	75T
63A	45A	72A
63A	43A	73A
64T	42T	75T
64T	41T	74T
64A	42A	75A
62G	41G	73G
61A	42A	71A
60A	42A	73A

59C	42C	71C
58T	42T	69C
59C	41C	68C
58T	42T	67T
55T	43T	65T
55C	43C	67C
56C	43C	68C
55C	41C	69C
54T	41T	70T
55C	39C	69C
57C	40C	69C
56T	39T	74T
56G	39G	73G
57T	39T	72T
57A	38A	72A
57T	37T	70T
58C	37C	73C
57G	37G	73G
56T	36T	70T
56C	35C	68C
58T	36T	68T
58T	36T	65T
58A	37A	66A
57A	37A	67A
56A	36A	66A
56C	37C	65C
55C	34C	66C
55A	35A	67A
56A	34A	70A
56A	37A	70A
56G	36G	73G
56A	36A	73A
56G	36G	71G
55A	36A	68A
57G	36G	65G
57A	35A	65A
58G	35G	65G
58G	33G	66G
58G	33G	65G
57G	33G	35T_30G
57A	33A	31A_35T
56G	33G	69G
55G	32G	70G

56A	31A	70A
55A	32A	71A
54T	32T	72T
54C	33C	71C
53G	33G	73G
52C	33C	74C
52A	33A	73A
52T	34T	71T
52T	34T	71T
52T	36T	71T
52A	36A	70A
52T	36T	70T
52T	36T	70T
51T	36T	69T
52T	35T	70T
52A	35A	70A
52A	34A	70A
51A	33A	68A
50C	33C	70C
50G	33G	69G
50C	33C	70C
50G	34G	68G
50C	34C	68C
50G	33G	68G
47C	34C	72C
47C	33C	75C
47C	30C	74C
46G	31G	75G
45G	31G	77G
45C	31C	78C
45G	31G	80G
45T	31T	81T
44C	29C	79C
44A	28A	79A
44C	28C	79C
43C	28C	77C
40C	28C	76C
39A	28A	76A
39C	27C	72C
39C	27C	72C
41A	28A	75A
41T	29T	76T
41C	29C	77C

38A	28A	77A
38T	28T	79T
37A	28A	80A
37A	29A	79A
37A	30A	79A
37T	30T	79T
39A	30A	78A
39A	30A	79A
38T	30T	78T
39A	30A	77A
39A	29A	79A
40T	30T	80T
41T	30T	78T
42C	28C	77C
41G	26G	75G
40A	26A	75A
39T	26T	75T
39T	25T	77T
40T	26T	76T
39T	26T	75T
39A	27A	76A
40T	27T	74T
41T	27T	74T
39T	26T	74T
39G	25G	74G
38A	26A	75A
38A	25A	73A
38T	25T	76T
39T	26T	73T
39T	28T	73T
39C	28C	74C
39T	27T	73T
39C	26C	74C
40C	29C	75C
41C	29C	75C
42C	29C	73C
41G	30G	72G
41G	30G	72G
40G	29G	71G
40G	30G	71G
37G	30G	71G
37A	30A	69A
37G	31G	71G

36G	32G	70G
34G	32G	70G
35T	32T	67T
35T	33T	68T
34T	33T	68T
36G	34G	70G
36C	34C	70C
35T	35T	68T
36A	35A	65A
36C	35C	62C
36A	35A	62A
34T	35T	62T
34G	35G	60G
34G	34G	58G
33A	34A	57A
32T	34T	58T
32T	34T	58T
33A	35A	60A
33T	35T	59T
32G	35G	58G
34A	33A	58A
34T	33T	58T
33A	32A	58A
32A	33A	57A
34T	33T	57T
33G	33G	56G
33C	34C	55C
32C	34C	52C
32G	34G	49G
33C	34C	48C
33C	34C	49C
33A	14A_20G	51A
33G	34G	52G
33C	34C	52C
33A	34A	50A
34G	34G	50G
34C	20T_14C	51C
35G	33G	48G
34G	33G	47G
35C	33C	45C
35G	33G	48G
35A	33A	52A
35T	33T	53T

36T	31T	54T
37C	31C	53C
38T	30T	53T
38T	32T	52T
40T	33T	49T
40C	31C	50C
39T	34T	48T
39C	34C	49C
39C	34C	48C
38G	34G	48G
39G	34G	48G
40C	34C	46C
40A	33A	46A
40G	34G	47G
40C	34C	47C
40G	34G	47G
40G	33G	46G
40C	33C	46C
41G	33G	46G
44A	33A	47A
44A	33A	49A
44G	32G	48G
44G	32G	50G
44A	32A	53A
44G	32G	56G
44G	32G	56G
44C	33C	55C
44T	33T	54T
45G	33G	55G
45A	33A	55A
45G	34G	56G
43T	33T	57T
44T	32T	55T
44T	32T	57T
45T	31T	57T
46C	32C	58C
46C	31C	60C
46C	29C	60C
47G	31G	60G
47T	32T	62T
45C	32C	62C
45G	32G	63G
46G	32G	61G

46G	32G	61G
46T	30T	58T
44T	30T	57T
44C	31C	57C
43G	31G	59G
44G	32G	60G
45T	32T	61T
45G	34G	62G
44C	33C	58C
44A	34A	59A
45G	34G	59G
45G	33G	60G
43A	33A	61A
43T	32T	61T
45G	33G	62G
46G	33G	62G
46G	33G	61G
45T	32T	64T
46G	33G	63G
45C	32C	65C
45C	31C	66C
45T	31T	66T
46T	31T	66T
46T	31T	69T
44A	29A	66A
44A	29A	61A
43G	28G	61G
43C	28C	59C
43C	28C	59C
43T	28T	60T
43T	26T	60T
43T	27T	61T
23T_20C	11T_17C	61C
43C	27C	62C
43C	27C	61C
43C	27C	61C
43G	28G	61G
43G	28G	60G
43G	28G	60G
41G	27G	27A_60G
40G	27G	28A_29G
39A	26A	62A
40G	26G	62G

40A	26A	60A
41G	26G	63G
41G	26G	64G
42A	26A	65A
42A	26A	68A
43T	27T	68T
43T	27T	65T
45C	26C	64C
45C	25C	63C
45C	26C	63C
43C	27C	63C
43C	28C	62C
42A	28A	62A
43G	28G	62G
43C	28C	61C
17T_24C	15T_13C	34T_27C
42T	28T	60T
42G	27G	60G
42C	27C	60C
44G	27G	57G
43G	26G	56G
42A	26A	56A
42C	26C	53C
42C	26C	53C
45T	26T	52T
44A	26A	52A
43T	25T	52T
43T	27T	52T
42T	27T	52T
42C	29C	52C
42T	29T	50T
43T	28T	49T
42G	28G	48G
43G	28G	48G
42C	28C	47C
39G	28G	46G
40A	28A	44A
40T	27T	41T
37G	26G	42G
37G	26G	41G
6A_42T_42G_61C	5A_35T_35G_45C	1A_7T_7G_45C
25C	13C	1A
25T	13T	null

26G	13G	null
26G	13G	null
27C	13C	null
27G	13G	null
27G	12G	null
27C	13C	null
27T	13T	null
26T	11T	null
28A	13A	null
27G	13G	null
24G	12G	null
23T	12T	null
23G	12G	null
23T	12T	null
23T	12T	null
23T	12T	null
23C	12C	null
30C	16C	7C
29A	16A	7A
29T	16T	7T
30C	16C	7C
30T	15T	7T
30G	15G	7G
30T	15T	7T
30G	16G	7G
30T	16T	7T
30C	10T_16C	7T_7C
39T	15T	7T
31C	16C	7C
32C	15C	7C
32C	14C	8C
32C	14C	8C
32C	14C	8C
33C	5T_9C	8C
24G_9C	9G_5C	8C
9A_25C	5A_9C	9A
39A	15A	26A
39A	15A	27A
39C	15C	28C
38A	14A	29A
38A	15A	29A
38T	15T	30T
38A	15A	30A

37T	16T	30T
36T	16T	31T
35A	17A	31A
35A	17A	32A
35C	16C	33C
35A	17A	33A
36G	17G	33G
37G	17G	33G
37G	17G	34G
36G	17G	34G
35T	17T	35T
36C	16C	36C
36T	17T	36T
36G	18G	37G
36G	18G	39G
36G	18G	39G
37A	18A	39A
37T	18T	41T
36C	18C	41C
36A	18A	42A
36G	18G	42G
37C	19C	44C
37T	19T	44T
35G	17G	44G
34G	17G	44G
35G	17G	43G
35A	17A	42A
34T	17T	42T
34C	16C	43C
12T_22C	15C	42C
34T	15T	42T
11T_22G	15G	40G
34T	15T	40T
34G	15G	38G
33A	16A	38A
33T	15T	33T
32C	16C	34C
32C	16C	35C
32G	15G	34G
32C	15C	33C
32T	15T	32T
19G	15G	32G
21C	15C	32C

21C	14C	12T_20C
21T	12T	31T
21G	11G	32G
21G	11G	32G
23T	11T	31T
23G	11G	36G
23G	11G	36G
23G	11G	36G
23G	11G	36G
23A	11G	59G
21T	11T	32T
21C	11C	31C
21T	11T	31T
21C	11C	30C
21T	11T	30T
19C	7T_2C	28C
19T	13T_2G_2C	58T_19G_13C
19C	10T_5G_9C	32C
19C	7C	24C
19C	7C	24C
19C	7C	24C
19C	7C	24C
18C	7C	24C
18C	7C	24C
18C	7C	24C
17C	7C	23C
17G	7C	16G_6C
18T	7C	14T_1G_6C
18G	7G	22G
18C	7C	23C
18G	8T	7T_18G
18C	8C	33C
18G	8G	35G
18C	8C	35C
18T	8T	34T
17C	8C	35C
17G	8G	34G
17G	8G	34G
17A	8A	33A
31C	8C	32C
32T	8T	31T
33C	8C	30C
36C	8G	14G_18C

35T	8T	35T
35G	8G	34G
36C	9C	34C
37G	10G	34G
37T	10T	34T
37A	11A	36A
37T	11T	36T
37C	11C	35C
37T	11T	35T
37T	11T	35T
37T	12T	34T
37T	12T	34T
38G	11G	36G
39C	11C	39C
39G	11G	40G
39C	11C	39C
40C	11C	39C
40G	11G	37G
40G	11G	37G
39G	11G	37G
39C	12C	37C
39T	12T	38T
39G	12G	38G
40C	12C	39C
41G	12T	20T_19G
40A	12A	39A
40A	13A	44A
41A	13A	44A
41C	13C	44C
41G	13G	44G
41T	14T	43T
41T	13C	43C
41C	12C	44C
42C	12C	44C
42T	12T	44T
42C	12C	46C
42T	12T	46T
42G	12G	46G
42T	12T	47T
42T	12T	45T
41T	12T	45T
41T	12T	45T
41G	12G	47G

41G	11G	47G
42A	10A	45A
42T	10T	46T
42C	11C	47C
42C	11C	47C
44A	11A	47A
45A	11A	47A
45A	11A	47A
43A	12A	47A
43C	11C	48C
42A	11A	48A
42G	11G	47G
41A	11A	47A
41G	12G	47G
41G	12G	45G
41A	12A	44A
41A	12A	44A
41C	12C	45C
40G	12G	45G
41T	13T	46T
39T	13T	46T
38T	13T	47T
37T	12T	46T
37C	12C	47C
36T	12T	48T
37A	12A	48A
37G	12G	46G
36C	12C	44C
37G	12G	44G
36T	12T	44T
18T_18C	12C	43C
36G	12G	42G
36A	12A	41A
36T	42T	193T
17A_16C	6C	14T
83T	null	14T
19T	null	14T
18T	4T	14T
18T	6T	14T
16T	6T	12T
31T	13T	24T
32T	13T	42T
97T	13T	43T

19T	13T	42T
17T	14T	39T
18T	15T	39T
18T	15T	40T
18T	15T	40T
16C	8T_7C	43T
31T	16T	44T
31T	16T	45T
31C	16C	46C
31C	16C	46C
31T	17T	23T_24G_1C
31G	17G	48G
31G	18G	49G
31C	18C	49C
31G	18G	50G
31T	8A_10T	50T
31G	19G	50G
31G	19G	50G
31G	19G	47G
32C	20C	48C
33G	20G	48G
33A	20A	47A
33G	22G	48G
32G	21G	48G
32G	21G	49G
32A	22A	49A
32T	21T	48T
31C	22C	48C
31T	22T	48T
32C	22C	47C
32T	23T	48T
33T	23T	48T
33T	23T	46T
33A	23A	48A
33A	22A	47A
35G	24G	47G
34G	24G	48G
34G	24G	47G
31T	24T	47T
31C	24C	45C
31C	24C	47C
30G	24G	47G
29A	23A	46A

29T	22T	47T
30A	21A	46A
30G	21G	45G
30A	20A	46A
12T_18G	10T_10G	45T
30A	20A	44A
30A	20A	45A
32G	20G	44G
32A	19A	43A
31T	18T	43T
30T	20T	43T
30T	20T	44T
29T	22T	44T
29C	21C	44C
29C	21C	45C
29C	21C	43C
29C	20C	43C
29T	21T	41T
29T	20T	43T
29T	20T	42T
29T	20T	40T
28C	20C	40C
27A	19A	40A
25A	20A	39A
26A	19A	39A
27A	19A	37A
27G	19G	37G
27A	19A	36A
27G	20G	35G
27A	20A	35A
27G	20G	34G
27G	20G	36G
27C	21C	33C
27G	21G	34G
28C	22C	37C
28G	22G	37G
29C	22C	37C
30T	22T	36T
30G	22G	38G
30G	22G	40G
29T	11T_12C	41T
29G	23G	41G
28G	23G	44G

28T	23T	44T
28C	23C	45C
28T	23T	45T
25C	23C	45C
25T	24T	46T
25T	23T	46T
25G	23G	47G
25G	23G	46G
25C	23C	44C
25A	23A	45A
25G	23G	46G
25A	23A	47A
25G	22G	47G
25G	22G	48G
25T	22T	47T
23T	22T	48T
23G	21G	48G
23G	22G	45G
23G	22G	46G
23C	22C	47C
23A	22A	47A
22T	21T	48T
22T	21T	48T
21T	21T	48T
20T	21T	47T
20A	21A	47A
20G	21G	47G
20G	22G	47G
19G	22G	48G
19G	22G	50G
19C	22C	49C
19G	22G	49G
18T	23T	49T
18C	23C	48C
18C	23C	47C
18C	22C	48C
17T	21T	47T
17C	21C	47C
16G	21G	47G
17A	21A	45A
17G	20G	47G
18C	21C	45C
18A	21A	45A

18A	21A	45A
18C	21C	45C
18G	21G	44G
18C	20C	43C
19C	20C	43C
19G	20G	43G
19G	20G	43G
19G	19G	45G
19T	19T	46T
19T	18T	48T
18G	17G	48G
17T	17T	48T
17G	17G	48G
17G	15G	48G
17G	15G	49G
17G	15G	47G
17G	15G	46G
17A	15A	44A
17T	15T	42T
17C	15C	41C
16C	15C	41C
17C	14C	40C
16T	14T	38T
16T	14T	34T
16C	14C	35C
16C	14C	34C
14C	13C	31C
14T	14T	30T
14C	15C	30C
14C	15C	30C
14T	15T	28T
14C	16C	28C
13G	16G	27G
13A	16A	26A
13T	16T	27T
12C	16C	27C
12G	16G	25G
11G	16G	25G
11C	16C	24C
11C	16C	24C
11T	16T	23T
10C	16C	22C
10C	16C	21C

10C	16C	22C
10C	16C	24C
10C	16C	23C
11T	13T	22T
12G	14G	21G
12G	14G	21G
12C	14C	21C
11G	14G	21G
12C	14C	21C
12C	14C	21C
12C	14C	21C
12C	14C	20C
12C	14C	20C
12A	13A	18A
12A	15A	18A
10A	15A	18A
10G	15G	18G
10C	15C	18C
10A	15A	17A
10C	14C	16C
10G	13G	16G
10A	14A	17A
9C	14C	16C
9C	13C	16C
9C	13C	16C
8T	13T	16T
7C	13C	16C
5A_17T_20C	24T_7G_24C	15T
6C	14C	15C
6C	14C	15C
6T	14T	15T
6C	13C	15C
6T	13T	15T
6C	14C	15C
6A	12A	13A
6C	13C	12C
6C	13C	10C
5C	13C	5T_5C
5C	12C	11C
5C	12C	5T_6C
5C	12C	11C
5T	13T	5A_5T
5C	13C	10C

5C	13C	10C
5T	13T	4T_5C
5C	13C	9C
5C	13C	9C
5C	8T_5C	10C
4T_1C	13T	10T
5C	13C	11C
5C	13C	12C
5T	13T	12T
5C	13C	12C
5C	13C	13C
3T_2C	12T	6T_7C
5C	12C	14C
5C	12C	15C
5C	12C	15C
5T	13T	15T
5C	13C	15C
5C	13C	16C
5T	14T	18T
5C	14C	19C
5C	14C	20C
7C	14C	20C
7G	14G	20G
7G	13G	20G
7G	13G	20G
6G	13G	20G
6A	12A	20A
7T	13T	19T
7C	12C	18C
7C	12C	19C
7C	12C	19C
6A	12A	19A
6G	12G	19G
7G	12G	19G
7C	12C	18C
7C	12C	19C
8G	13G	19G
8G	13G	19G
8C	13C	19C
8T	14T	20T
8T	14T	20T
8C	14C	20C
8T	13T	21T

8G	13G	21G
8C	13C	22C
8C	13C	22C
8T	8T_5C	23T
8C	13C	23C
7T	13T	22T
8C	14C	22C
8C	14C	22C
8T	14T	21T
8G	14G	21G
8C	14C	21C
8C	14C	21C
8C	13C	21C
8G	13G	22G
8A	13A	21A
8C	13C	22C
8C	13C	22C
8C	13C	22C
8G	13G	22G
8T	13T	22T
8C	13C	22C
8G	13G	22G
8C	12C	22C
8G	12G	22G
8G	12G	22G
8G	12G	22G
9C	12C	22C
9T	13T	22T
9T	13T	22T
9A	13A	22A
8C	13C	21C
7C	13C	21C
6T	12T	21T
6T	13T	20T
6G	13G	20G
6C	13C	21C
7A	13A	21A
7G	13G	20G
7C	13C	20C
7T	13T	19T
7T	13T	18T
7T	12T	19T
7C	12C	20C

7G	12G	20G
7C	12C	19C
7A	13A	18A
7G	13G	18G
7G	13G	17G
7T	11T	17T
7G	5A_6G	17G
7A	11A	17A
7G	11G	18G
7G	11G	18G
7A	11A	18A
7G	11G	19G
7A	11A	17A
8C	11C	18C
8C	11C	18C
8G	11G	18G
8T	10T	18T
8A	10A	19A
8A	10A	19A
8T	10T	19T
8G	10G	19G
8G	10G	19G
8T	10T	19T
8A	10A	18A
8G	9G	18G
8C	9C	18C
8C	9C	17C
8G	9G	17G
8G	9G	16G
8A	9A	16A
8G	9G	15G
8A	9A	15A
8C	8C	16C
8C	8C	16C
8T	9T	16T
8T	9T	16T
8G	9G	16G
8T	9T	16T
8C	9C	16C
8C	9C	16C
8C	9C	15C
8C	9C	16C
8G	9G	16G

8C	9C	16C
8A	9A	16A
8G	9G	16G
8A	9A	17A
8C	9C	17C
8G	9G	17G
8G	8G	16G
8G	8G	16G
4A_4G	4A_4G	7A_10G
8C	7C	17C
8A	7A	18A
8G	6G	8G_10C
6A	6A	18A
6G	6G	18G
6C	6C	19C
6T	6T	19T
6C	6C	20C
6G	6G	20G
5T	6T	19T
5C	6C	17C
5C	6C	17C
5A	6A	17A
5G	7G	17G
5A	7A	18A
4T	7T	18T
4C	7C	18C
4C	7C	17C
4T	6T	17T
4C	6C	17C
4G	6G	17G
4T	6T	16T
4C	6C	17C
4G	6G	16G
4T	6T	16T
5A	6A	16A
5G	6G	15G
1A_4G	6G	15G
5A	6A	15A
5A	7A	14A
5T	7T	14T
4A	6A	13A
4A	6A	13A
4T_1C	6T	13T

5C	6C	12C
1A_4C	6C	11C
5A	5A	11A
4T_1C	5T	11T
5C	5C	11C
5C	5C	11C
5C	5C	11C
5C	5C	11C
5G	5G	11G
5C	6C	12C
5C	6C	12C
4G	6G	12G
4C	6C	12C
4C	6C	13C
4G	6G	13G
4G	6G	14G
4A	6A	13A
3C	6C	13C
3C	6C	15C

ZZ_6	ZZ_7	ZZ_8
172G	66G	78G
173T	68T	76T
172C	70C	77C
175C	71C	77C
172C	71C	77C
165A	71A	78A
176G	75G	81G
174C	77C	82C
175T	78T	83T
172C	80C	83C
172C	81C	84C
169T	82T	84T
168G	82G	84G
167C	85C	81C
162C	86C	84C
160A	87A	86A
158A	88A	85A
152C	82C	79C
156C	83C	84C
158T	91T	85T
159A	90A	85A
158G	91G	85G
156C	92C	86C
152A	91A	85A
148G	88G	84G
146T	89T	85T
151T	85T	79T
150C	88C	79C
145A	59A_28G	77A
149A	92A	80A
145A	89A	78A
141A	90A	77A
131G	87G	71G
135C	87C	70C
133A	95A	69A
133T	96T	67T
130G	96G	61G
113T	89T	56T
126A	95A	59A
121A	97A	56A
123A	97A	55A

121T	95T	52T
113G	91G	49G
106C	90C	46C
112G	90G	46G
111A	92A	48A
112G	90G	48G
113T	88T	46T
110A	90A	47A
112G	91G	45G
113A	90A	44A
111T	89T	44T
112A	89A	45A
112A	87A	44A
63A_49G	87A	34G
110T	86T	38T
114A	83A	38A
116G	80G	35G
114G	83G	34G
53T_58G	32T_44G	31T
111A	77A	33A
52G_58C	33T_45C	29G
119C	78C	37C
121A	81A	37A
118C	82C	38C
122C	78C	38C
116A	76A	38A
116T	77T	39T
117A	77A	38A
115G	77G	38G
116T	79T	38T
53A_60G	41A_33G	37G
115G	74G	37G
113G	75G	37G
115A	76A	38A
121A	76A	35A
120G	78G	34G
122G	78G	35G
124T	78T	36T
126A	78A	36A
129A	78A	37A
130T	41T_34C	38T
132G	74G	36G
131G	74G	36G

63T_66C	74C	34C
129G	73G	34G
130T	73T	35T
131T	75T	35T
132C	75C	36C
135T	38T_33C	35T
137A	71A	35A
137T	72T	34T
139G	72G	34G
141T	71T	34T
143C	72C	34C
145T	72T	32T
140A	75A	34A
140G	72G	35G
141T	73T	33T
142A	35A_31G	35A
142G	65G	35G
144A	66A	36A
146A	66A	36A
143A	66A	36A
146C	65C	34C
143T	64T	34T
143G	64G	36G
148T	65T	35T
147C	64C	35C
149C	64C	37C
146A	66A	35A
146C	65C	38C
151G	73G	41G
150G	76G	40G
148A	77A	41A
149C	78C	41C
150A	79A	41A
150A	80A	40A
150A	80A	42A
150T	76T	40T
149G	82G	44G
151C	70C	40C
148C	80C	44C
146G	84G	44G
145G	83G	45G
145C	81C	44C
141T	82T	46T

138C	80C	46C
139T	79T	46T
138A	78A	46A
138C	78C	45C
136A	77A	45A
144G	82G	44G
144C	80C	45C
146T	82T	45T
145T	85T	46T
146G	87G	46G
147G	85G	48G
147A	86A	48A
146G	83G	47G
150A	80A	49A
150T	84T	48T
150G	85G	51G
152G	85G	52G
154G	83G	51G
150G	87G	54G
149A	86A	57A
150T	88T	58T
147G	91G	59G
142A	89A	59A
142G	91G	22A_35G
144C	90C	45C
141A	87A	53A
148C	78C	54C
147C	87C	53C
150G	77G	53G
149C	89C	57C
146G	86G	56G
150C	93C	57C
148C	93C	59C
146C	93C	60C
143T	92T	60T
144A	92A	56A
139G	91G	59G
141A	93A	61A
141G	92G	60G
142T	91T	62T
140C	92C	58C
141G	91G	60G
140G	91G	61G

140A	94A	62A
137C	95C	62C
134A	95A	62A
135C	96C	63C
134A	93A	57A
135A	97A	65A
131C	98C	67C
132T	99T	65T
135G	101G	67G
134G	97G	62G
133A	94A	61A
136C	92C	62C
131T	88T	64T
130A	85A	61A
131A	85A	65A
132A	83A	66A
121T	54T_25C	68T
130G	24A_56G	68G
131T	82T	68T
132C	84C	65C
137A	83A	64A
136A	80A	65A
134G	81G	64G
139G	82G	64G
139G	86G	63G
140G	88G	63G
141A	88A	61A
141A	87A	67A
136C	87C	65C
139C	88C	62C
139T	87T	63T
132T	86T	64T
126T	87T	63T
125A	87A	65A
126C	84C	64C
125C	81C	62C
124T	79T	61T
124T	80T	59T
122T	80T	60T
113A	83A	60A
116C	84C	57C
114C	78C	58C
115T	79T	58T

114A	77A	55A
113T	75T	54T
105A	75A	53A
105T	73T	49T
41A_56C	71C	45C
99T	70T	47T
99G	70G	46G
98G	72G	46G
96C	72C	47C
97T	71T	47T
97A	73A	47A
97C	75C	48C
100T	74T	48T
99C	76C	50C
100T	79T	50T
100G	78G	50G
101C	79C	48C
99C	80C	47C
99A	80A	47A
105C	84C	47C
111C	85C	47C
109C	86C	19T_30C
109T	86T	49T
110C	87C	50C
111T	87T	51T
112C	87C	53C
115T	86T	54T
116C	82C	55C
117T	82T	56T
119A	84A	55A
120C	82C	55C
124C	83C	54C
123C	81C	56C
123T	86T	55T
122C	86C	55C
120C	85C	53C
120C	44T_41C	55C
119G	85G	56G
118G	82G	54G
119T	83T	53T
122G	83G	53G
122G	80G	53G
120G	79G	53G

122T	80T	50T
124G	78G	49G
122G	78G	52G
119A	75A	51A
112T	80T	54T
117T	80T	52T
114C	81C	55C
114T	82T	55T
113C	79C	57C
114G	77G	54G
112T	76T	55T
112A	76A	50A
110G	76G	52G
111T	75T	50T
111T	74T	53T
115C	74C	52C
116C	71C	51C
115T	69T	51T
115A	69A	49A
113C	67C	49C
114T	68T	50T
112C	67C	51C
111A	66A	49A
111C	63C	48C
108A	62A	50A
109C	61C	49C
107T	60T	49T
109G	59G	49G
104T	57T	48T
107C	56C	48C
107A	56A	48A
107A	58A	48A
106G	57G	49G
106T	57T	48T
107G	54G	50G
107A	54A	50A
107T	56T	49T
106A	54A	49A
105A	53A	49A
102T	53T	49T
102T	52T	48T
102C	49C	48C
102A	50A	49A

101T	48T	49T
102C	46C	49C
103C	47C	50C
101T	46T	51T
99T	47T	51T
100C	45C	50C
98A	46A	48A
99C	44C	48C
98A	44A	48A
99G	44G	48G
94T	43T	44T
92C	46C	44C
91T	47T	43T
88G	45G	42G
86A	44A	44A
83A	43A	43A
85T	45T	39T
85G	41G	39G
84C	41C	39C
82A	41A	38A
83A	43A	37A
84A	44A	37A
84G	45G	48G
82G	46G	17A_21G
43A_35G	14A_44G	19A_13G
104A	67A	36A
53A	27A	36A
77A	48A	37A
76A	48A	36A
76A	41A	35A
77A	51A	39A
38A_42T	25A_24T	34A_15T
75A	51A	39A
74A	53A	40A
74A	52A	41A
70A	50A	42A
74A	50A	42A
73T	51T	41T
77A	51A	40A
72A	51A	37A
79G	51G	39G
79G	52G	40G
78G	52G	40G

78C	53C	40C
79C	53C	41C
79A	55A	42A
80G	58G	45G
79C	61C	45C
81T	62T	45T
82C	64C	47C
84A	65A	46A
85C	67C	46C
87T	68T	47T
87G	69G	47G
88G	71G	48G
89T	72T	48T
88C	73C	50C
89C	73C	51C
88C	73C	48C
87C	72C	49C
86A	73A	50A
87A	75A	51A
87G	77G	52G
88C	79C	52C
88A	77A	54A
89G	78G	54G
88C	81C	54C
88A	82A	54A
87A	82A	53A
88A	81A	54A
90G	80G	54G
87A	80A	54A
86T	81T	53T
86A	81A	52A
86A	81A	53A
85G	82G	53G
85G	83G	51G
85A	84A	50A
86C	82C	52C
83T	82T	53T
84C	81C	54C
84G	80G	55G
85G	81G	53G
87C	79C	54C
88A	78A	55A
89A	79A	54A

88A	81A	54A
87G	80G	55G
88C	78C	53C
89G	79G	53G
87G	79G	53G
88G	79G	53G
89G	76G	53G
90A	73A	54A
92G	73G	54G
93A	72A	55A
93A	72A	54A
93A	72A	54A
93A	73A	53A
97C	74C	58C
98C	71C	54C
98C	72C	52C
98A	72A	49A
99C	69C	49C
102A	71A	49A
103C	71C	47C
103T	74T	49T
106T	75T	50T
102G	76G	51G
103A	75A	51A
100C	76C	52C
101T	77T	52T
99A	78A	53A
96T	77T	54T
97A	77A	53A
97A	76A	52A
98G	76G	49G
99T	73T	47T
96G	76G	48G
96A	75A	46A
95A	76A	50A
95T	75T	50T
94C	75C	49C
95G	76G	47G
92T	75T	47T
93A	75A	46A
93A	75A	46A
92A	72A	45A
93A	72A	45A

93T	73T	46T
93T	72T	43T
93T	72T	42T
91A	71A	41A
90T	70T	40T
91T	68T	40T
98G	69G	40G
99G	66G	39G
99A	66A	39A
101A	66A	41A
102T	67T	41T
101G	69G	41G
104T	67T	41T
107C	66C	40C
110T	65T	41T
109T	66T	40T
109C	65C	37C
109C	64C	40C
110A	61A	40A
109T	61T	41T
108T	61T	41T
106T	63T	41T
106G	64G	41G
105G	63G	42G
103T	62T	41T
102A	64A	40A
101A	64A	40A
103G	62G	42G
104A	61A	41A
106G	61G	43G
108A	61A	42A
106G	62G	43G
106C	62C	43C
105T	62T	42T
106C	63C	42C
105A	62A	42A
103T	63T	41T
105C	63C	42C
106T	64T	41T
107T	65T	42T
102A	64A	39A
100A	64A	39A
101C	62C	39C

100C	61C	40C
99C	59C	40C
95C	60C	39C
94C	62C	39C
92T	59T	37T
90C	59C	37C
89T	58T	35T
88G	58G	35G
90G	58G	35G
90G	58G	34G
92G	58G	34G
92A	58A	34A
93G	61G	34G
94T	63T	35T
94A	61A	35A
93A	61A	36A
94A	58A	36A
93A	61A	35A
93G	58G	34G
94G	59G	35G
94G	59G	35G
93A	58A	35A
93A	57A	38A
92A	57A	38A
91C	56C	38C
92A	57A	37A
93C	57C	37C
93T	59T	37T
92T	60T	39T
91A	59A	39A
92A	61A	41A
91G	60G	42G
91G	63G	41G
83A	61A	40A
82C	61C	41C
82A	59A	42A
80C	60C	40C
81T	60T	40T
79T	58T	39T
77T	59T	40T
75T	60T	40T
72T	60T	39T
71C	63C	40C

70G	63G	40G
69T	64T	38T
68T	65T	38T
70T	64T	38T
70C	64C	38C
69G	65G	35G
68A	66A	35A
69G	66G	34G
34A_35G	67A	18A_17G
72A	65A	36A
75A	67A	36A
76G	68G	37G
74A	69A	37A
72A	70A	36A
70A	71A	35A
72G	71G	34G
73G	69G	33G
74A	69A	33A
75A	68A	34A
74A	68A	33A
76A	69A	34A
75A	70A	34A
76C	70C	34C
76C	70C	35C
77A	70A	34A
78A	70A	34A
125A_55G	86A	63A_29G
80A	69A	37A
82G	69G	38G
84A	67A	39A
86G	66G	39G
88A	67A	39A
90G	68G	40G
92A	70A	40A
92G	70G	42G
95A	69A	42A
94G	69G	42G
91A	68A	41A
92G	68G	39G
89A	65A	42A
88G	63G	43G
87A	68A	45A
84T	49T	37T

87C	49C	39C
89A	48A	39A
91C	50C	40C
92A	68A	41A
90G	68G	39G
94A	68A	41A
94G	70G	41G
92A	71A	43A
93G	10A_5T_69G_10C	43G
93A	68A	43A
93G	63G	44G
96A	69A	44A
95G	68G	41G
101A	67A	40A
95G	65G	39G
117A	64A	39A
48A_29G	41A_22G	41A
100A	64A	40A
101G	64G	41G
98A	64A	41A
97G	65G	42G
96A	65A	43A
98G	66G	41G
98A	64A	40A
95G	64G	41G
96A	64A	42A
99G	63G	43G
100A	62A	42A
103G	61G	41G
100A	61A	41A
34A_34T_136G	62G	42G
64A	63A	42A
68G	68G	15T_49G
56A	51A	20A
105G	61G	20G
104T	18A_42T	19A
104G	71G	27G
103T	70T	28T
103G	70G	30G
107A	69A	30A
109C	68C	30C
108A	69A	29A
109C	69C	30C

110A	72A	34A
109T	71T	34T
113T	71T	33T
117C	72C	34C
115A	72A	35A
115A	71A	35A
113A	72A	38A
112C	68C	40C
111T	71T	40T
111A	74A	40A
110T	75T	40T
109G	75G	40G
107T	75T	39T
107T	75T	40T
104C	76C	40C
103T	77T	39T
104C	76C	42C
101C	76C	41C
101A	75A	40A
101G	76G	39G
100C	73C	41C
99A	75T	28A_16T
103A	76A	45A
103T	77T	45T
102T	76T	45T
103T	75T	44T
102C	75C	45C
101A	75A	43A
99A	75A	43A
101A	75A	44A
102C	76C	44C
101C	76C	43C
100A	76A	44A
99C	75C	43C
100T	73T	43T
103G	72G	43G
100C	74C	42C
102A	75A	40A
51T_50C	75C	40C
100T	75T	41T
99C	78C	41C
99A	79A	41A
98T	80T	41T

97G	82G	41G
100C	83C	40C
100A	84A	39A
103C	85C	41C
103G	83G	41G
102T	82T	40T
100C	81C	41C
99T	82T	42T
102A	82A	41A
103T	82T	39T
101G	82G	40G
99C	84C	41C
100C	85C	43C
97G	86G	43G
96A	85A	42A
95A	82A	43A
98C	82C	43C
102A	80A	44A
97G	73G	44G
99A	79A	43A
98G	76G	45G
98T	78T	45T
100C	79C	43C
100C	81C	43C
99A	80A	43A
99G	80G	43G
95A	80A	41A
96C	77C	38C
94G	78G	38G
93T	78T	38T
91C	77C	38C
91A	76A	38A
91C	76C	37C
93A	75A	35A
94G	75G	35G
94G	73G	35G
95A	70A	34A
95A	70A	35A
95C	72C	35C
98G	72G	18A_17G
98G	71G	35G
98A	69A	34A
101A	72A	35A

101A	74A	34A
101A	75A	33A
99A	75A	34A
99A	75A	34A
100G	76G	34G
98G	74G	31G
97T	73T	31T
95A	75A	30A
95G	76G	30G
95C	73C	30C
99A	75A	29A
102A	74A	29A
104C	74C	27C
104A	36A_37G	28A
102T	74T	29T
103G	74G	28G
101T	75T	28T
102T	77T	28T
103T	77T	28T
100G	77G	28G
104T	75T	27T
103G	75G	29G
106G	77G	30G
107A	77A	30A
108G	77G	30G
109G	77G	33G
109T	73T	35T
108G	71G	37G
107C	71C	38C
107A	71A	38A
107G	70G	37G
108A	70A	37A
108A	72A	37A
107A	74A	36A
108G	73G	38G
110C	72C	38C
109C	74C	40C
108A	74A	38A
112T	72T	38T
114T	71T	37T
115G	69G	36G
115C	70C	37C
117T	74T	36T

116G	74G	35G
115C	75C	36C
117G	39A_36G	36G
116G	69G	35G
116T	68T	35T
115G	67G	35G
112A	66A	35A
111C	64C	36C
109C	65C	36C
110C	65C	36C
111T	64T	37T
109T	66T	36T
114G	66G	36G
113A	67A	38A
111T	69T	39T
113G	67G	39G
117G	68G	38G
116C	68C	38C
115A	68A	37A
114C	68C	37C
113A	68A	37A
115A	69A	38A
116A	69A	39A
115A	73A	39A
109A	65A	40A
63A_50T	41A_28T	40A
108A	64A	36A
111G	68G	38G
112G	67G	42G
113G	69G	44G
111G	40A_30G	43G
111A	70A	45A
116T	70T	46T
116G	72G	46G
118G	73G	46G
118A	36A_37G	46A
119A	72A	45A
121A	72A	45A
117T	73T	46T
117G	72G	46G
115C	73C	47C
114T	73T	47T
115C	72C	48C

113C	72C	47C
114T	71T	48T
114G	34G_35C	49G
112A	69A	49A
113C	72C	49C
111A	72A	49A
114A	73A	48A
113A	74A	46A
114C	74C	47C
114C	71C	47C
113C	72C	44C
115A	73A	41A
119A	74A	42A
117C	74C	41C
119A	75A	41A
116C	76C	42C
115A	77A	42A
119A	77A	42A
121G	78G	43G
121G	80G	39G
118C	81C	38C
119A	81A	37A
120A	38A_42T	38A
117G	81G	37G
113G	81G	37G
114A	84A	40A
112G	84G	40G
112T	80T	41T
62A_49G	80A	25A_17C
112G	80G	42G
110A	80A	43A
107T	84T	44T
111C	41T_44C	43C
112C	83C	43C
118G	86G	44G
118C	87C	46C
121T	88T	47T
120G	87G	46G
121C	90C	45C
123A	89A	45A
120G	88G	44G
122G	88G	43G
123A	86A	44A

121A	90A	45A
116C	91C	47C
116A	93A	46A
116A	93A	49A
118C	94C	48C
119A	93A	48A
121C	92C	48C
119T	88T	48T
119G	87G	48G
117T	86T	48T
112C	88C	49C
112C	86C	48C
115A	86A	44A
116G	84G	41G
116C	86C	16G_24C
118C	86T	39C
117C	87C	40C
117A	85A	40A
116G	83G	41G
117T	82T	41T
120T	82T	42T
119C	83C	42C
119A	80A_27G	41A
123G	84G	43G
111G	83G	19A_37G
109A	84G	35A
111A	84G	35A
127A	79A	46A
128A	85A	46A
129A	51A_1T_34C	46A
131A	83A	46A
130A	85A	46A
133A	84A	46A
127A	83A	45A
124T	82T	46T
123C	83C	47C
122A	83A	47A
122C	86C	49C
123C	88C	48C
122T	87T	48T
123G	89G	48G
120T	87T	46T
123C	85C	47C

124T	85T	48T
121G	84G	48G
122A	83A	47A
124T	81T	47T
121C	81C	47C
125T	81T	47T
125A	84A	47A
125C	85C	48C
128A	85A	46A
127T	84T	46T
129T	84T	44T
129G	85G	47G
128C	85C	47C
125A	84A	46A
124C	83C	44C
126G	83G	43G
125T	84T	44T
124T	83T	43T
120T	84T	42T
117A	82A	43A
117T	82T	44T
118A	84A	44A
118G	81G	45G
117C	83C	46C
117C	84C	47C
115A	85A	46A
113T	86T	49T
114T	85T	49T
116T	83T	48T
115G	81G	48G
114A	84A	44A
112G	84G	48G
110A	82A	47A
110C	81C	47C
110C	82C	47C
109A	82A	47A
110C	80C	49C
55A_46T_1G	69T	43T
46T_57C	68T	19T_25C
102G	67G	46G
102A	69A	46A
49T_55G	70T	46T
113C	77C	49C

112A	77A	50A
115T	77T	50T
117A	81A	51A
118G	82G	53G
119C	84C	52C
119T	83T	50T
121C	86C	52C
120C	86C	52C
119A	86A	51A
119T	85T	52T
119T	82T	54T
114C	83C	53C
114C	84C	56C
119C	88C	55C
117C	86C	57C
118A	86A	56A
116A	87A	57A
116A	85A	57A
116A	85A	57A
118T	85T	55T
120C	86C	55C
119C	84C	54C
118T	84T	53T
116G	85G	52G
115G	80G	56G
118G	83G	56G
117A	84A	55A
115T	83T	56T
115T	84T	57T
114G	83G	59G
112G	81G	59G
113T	81T	59T
112A	83A	60A
111G	80G	60G
112A	78A	60A
111T	79T	59T
110A	79A	59A
107G	80G	59G
107G	79G	55G
108G	78G	57G
107G	80G	55G
109G	81G	57G
109T	80T	58T

108A	82A	57A
107T	80T	57T
109G	79G	56G
110T	79T	54T
108A	79A	55A
109G	79G	55G
110A	77A	54A
110A	78A	53A
112A	78A	54A
110T	75T	53T
113G	75G	50G
112T	73T	48T
114C	74C	51C
113T	74T	50T
115G	73G	51G
117C	71C	49C
118T	71T	49T
113A	72A	48A
112G	73G	50G
110C	72C	50C
111A	70A	47A
108A	70A	48A
106A	72A	50A
108G	73G	47G
107C	74C	49C
111C	75C	50C
112T	74T	51T
110C	75C	51C
108C	76C	53C
104C	74C	53C
106G	76G	52G
105A	75A	52A
107A	75A	55A
105C	71C	54C
107T	71T	53T
109C	75C	57C
111T	76T	58T
109G	77G	57G
109G	76G	58G
109C	72C	55C
105A	69A	56A
108G	72G	59G
109A	70A	58A

108G	71G	58G
107A	71A	58A
108A	71A	58A
106C	71C	59C
105T	69T	58T
104C	72C	58C
104T	72T	58T
61T_43G	74G	57T
106A	75A	55A
101A	73A	55A
100G	73G	55G
99C	74C	52C
98C	72C	51C
97C	72C	50C
95A	28A_44G	49A
94T	72T	49T
92C	71C	48C
106C	75C	60C
110C	75C	58C
106C	78C	57C
110A	78A	59A
112A	79A	61A
113A	79A	60A
114C	80C	60C
113T	82T	60T
113G	82G	58G
111C	81C	59C
112A	77A	58A
114A	77A	59A
114A	77A	61A
113T	36T_43C	60T
112T	79T	61T
114C	79C	62C
113C	78C	62C
112A	79A	61A
113A	79A	62A
112T	78T	59T
114A	80A	60A
112C	78C	62C
112T	80T	62T
108A	80A	61A
109G	81G	60G
108A	82A	58A

107A	81A	59A
107C	81C	62C
108A	81A	62A
110T	80T	61T
110G	80G	60G
112C	84C	60C
114C	84C	59C
114A	83A	59A
57T_56C	34T_47C	51T
111C	81C	49C
57A_52G	47A_33G	50G
115A	83A	51A
120G	84G	55G
125A	84A	55A
125T	83T	57T
126T	82T	59T
125T	83T	60T
122T	83T	59T
122G	83G	60G
121C	83C	59C
63T_59C	83T	58C
122A	85A	57A
126T	86T	56T
126A	87A	55A
127C	89C	56C
127A	87A	58A
125T	86T	55T
124A	48A_38G	56A
126C	86C	57C
129A	87A	58A
129A	88A	55A
128A	85A	55A
133T	86T	55T
129C	84C	55C
130C	82C	53C
129C	81C	54C
127A	83A	54A
131A	85A	53A
129G	84G	53G
131A	84A	53A
129T	86T	54T
133T	86T	54T
132T	86T	54T

129T	88T	53T
128G	86G	52G
125T	86T	55T
125T	85T	55T
125G	85G	55G
126A	84A	54A
125G	84G	56G
124A	84A	55A
128T	85T	57T
130G	86G	57G
131G	88G	58G
130A	91A	57A
127G	91G	54G
130C	91C	56C
129T	91T	57T
130G	92G	55G
130T	93T	54T
129A	94G	54G
129A	94A	56A
127T	92T	57T
128A	95A	58A
125A	93A	58A
130A	97A	59A
129T	95T	59T
130G	96G	60G
130G	95G	59G
131T	94T	61T
130A	96A	61A
127T	94T	56T
129C	94C	58C
127A	95A	57A
128A	96A	58A
127A	93A	55A
124C	90C	59C
122T	89T	57T
122G	89G	57G
121A	87A	56A
120C	89C	56C
116A	90A	56A
112C	91C	57C
107A	93A	58A
108A	93A	56A
106C	91C	53C

105C	91C	52C
109T	92T	52T
110G	93G	53G
108T	93T	54T
107G	91G	55G
106T	89T	56T
105C	87C	56C
103A	88A	61A
101T	86T	62T
100G	87G	60G
103T	87T	62T
106A	87A	61A
105G	87G	60G
101A	86A	60A
105G	84G	60G
104G	83G	59G
102G	83G	62G
101T	83T	63T
104C	83C	64C
103T	84T	62T
105C	83C	62C
102C	81C	64C
101A	79A	65A
99T	82T	66T
101C	84C	64C
97T	83T	64T
100C	82C	64C
100T	82T	68T
100C	83C	67C
102T	83T	66T
101T	82T	66T
101T	80T	65T
101C	78C	66C
100T	80T	66T
102T	79T	66T
98G	76G	67G
98A	76A	67A
98A	74A	69A
98T	72T	70T
99A	73A	70A
100T	73T	69T
98C	74C	69C
96C	73C	68C

97C	74C	69C
98A	72A	69A
97C	73C	69C
100C	75C	72C
98C	75C	71C
99A	75A	72A
95G	71G	70G
95T	70T	70T
99G	72G	69G
100G	74G	69G
98T	72T	66T
96T	70T	68T
99T	72T	70T
98C	72C	70C
99T	71T	70T
99C	70C	69C
97T	75T	70T
101C	76C	69C
103T	76T	70T
102T	78T	70T
103C	77C	70C
56T_48C	78C	41T_28C
104T	78T	70T
105T	76T	68T
106A	74A	69A
105C	75C	69C
104A	77A	69A
102C	44T_31C	70C
100A	75A	70A
100C	74C	70C
42A_57C	32A_42C	67C
101C	75C	67C
102A	77A	67A
101C	77C	65C
102A	76A	61A
104C	77C	60C
106A	78A	60A
103A	79A	59A
104A	79A	61A
48T_54C	80C	61C
104C	79C	23T_38C
100T	81T	61T
100G	81G	60G

99G	80G	60G
100T	82T	60T
101C	82C	60C
102T	80T	60T
102G	80G	61G
101G	82G	61G
101A	83A	59A
103G	82G	58G
102A	81A	62A
104G	83G	61G
103A	83A	61A
102A	82A	58A
102C	79C	59C
98G	82G	60G
100C	83C	61C
99A	83A	62A
96C	84C	61C
96T	86T	59T
95C	86C	59C
96T	85T	59T
97T	86T	60T
99G	86G	58G
54T_45C	50T_37C	59T
100T	89T	60T
101T	91T	63T
101T	88T	63T
101T	86T	64T
100A	88A	63A
100A	89A	62A
101A	88A	63A
99C	87C	59C
51A_49G	36A_51G	61G
101G	88G	61G
98T	89T	60T
99T	89T	60T
95G	87G	60G
92T	85T	59T
93G	86G	60G
32T_125G	37T_121G	24T_83G
91G	88G	58G
90T	89T	58T
84G	84G	51G
88T	88T	53T

87C	83C	23T_31C
86G	83G	54G
86G	83G	54G
89A	83A	33A_22T
91G	84G	59G
90G	85G	59G
91T	87T	58T
91T	88T	56T
91T	88T	56T
91T	87T	60T
90T	89T	63T
94A	89A	63A
94T	87T	64T
94G	88G	63G
96G	86G	64G
96A	88A	66A
99C	86C	66C
102C	87C	67C
101A	87A	67A
100G	88G	65G
97G	88G	66G
100A	94A	69A
100T	94T	67T
101T	93T	68T
100T	94T	68T
102G	94G	68G
100T	92T	68T
102A	92A	69A
99C	92C	68C
98A	96A	68A
99G	94G	66G
100C	94C	63C
99C	94C	65C
103T	94T	65T
102A	92A	64A
102T	91T	62T
103A	90A	60A
103G	90G	60G
104C	88C	62C
104A	91A	62A
103A	92A	59A
102A	89A	57A
104G	90G	58G

104A	91A	55A
109T	92T	58T
107G	89G	57G
104C	89C	58C
103T	89T	57T
99G	89G	55G
99C	86C	54C
100C	85C	53C
98T	86T	50T
102T	84T	49T
101C	86C	50C
102A	87A	50A
53T_51C	40T_45C	17T_33C
102G	84G	49G
100T	84T	47T
101C	81C	47C
101A	79A	47A
103A	79A	47A
104T	79T	47T
104G	76G	48G
102A	77A	47A
100A	76A	45A
99C	76C	45C
98A	77A	44A
101G	78G	44G
103A	77A	43A
103A	78A	45A
106A	77A	44A
104A	77A	44A
103G	76G	44G
101C	77C	44C
102A	75A	42A
100A	76A	44A
108C	75C	45C
107C	75C	46C
107T	73T	45T
106C	74C	44C
106T	72T	44T
104T	72T	43T
104C	76C	43C
106T	77T	44T
104A	81A	49A
100A	79A	47A

99A	79A	48A
97G	79G	46G
98C	78C	47C
98A	75A	50A
98C	77C	49C
95A	72A	47A
92T	73T	45T
38T_53C	36T_37C	18T_1G_27C
90T	74T	46T
91G	74G	44G
94T	74T	43T
93G	75G	45G
93C	77C	45C
93A	74A	44A
94C	74C	46C
94A	73A	46A
93T	72T	45T
36T_56C	70C	44C
91T	70T	44T
90G	71G	43G
89T	70T	44T
93G	70G	43G
94C	70C	41C
95A	67A	41A
98G	68G	41G
96G	68G	42G
95T	66T	40T
95G	65G	40G
91T	64T	40T
91T	63T	39T
90T	64T	42T
91G	66G	45G
92C	66C	45C
92A	65A	45A
91T	67T	47T
90T	65T	46T
87C	65C	46C
87A	64A	47A
87G	63G	47G
84T	63T	17A_45T_15C
86C	63C	50C
83A	63A	49A
83C	63C	49C

82A	63A	48A
79C	63C	49C
79A	65A	50A
81C	65C	49C
82A	63A	48A
85C	64C	50C
85A	63A	50A
85C	59C	51C
84A	57A	50A
82C	62C	51C
84A	54A	49A
25A_25G_106C	20A_20G_74C	51C
58A	35A	49A
56C	32C	51C
44G	27G	47G
77C	54C	49C
77A	55A	46A
73G	55G	47G
75A	57A	47A
75A	57A	44A
79G	57G	44G
80C	57C	45C
81C	57C	45C
80T	55T	43T
78A	55A	42A
77G	54G	36G
75A	54A	39A
76A	54A	39A
77A	50A	39A
77G	48G	41G
77G	48G	40G
78G	48G	40G
73T	48T	38T
82G	52G	41G
82G	52G	42G
81G	48G	44G
78C	49C	41C
80T	50T	17T_27G
78T	50T	45T
77G	50G	46G
77T	51T	48T
76A	51A	47A
71G	49G	47G

74A	49A	48A
72A	45A	46A
75C	20T_31C	17T_30C
76T	50T	47T
77G	52G	49G
73A	50A	48A
73T	53T	49T
72T	52T	48T
73C	53C	52C
71C	53C	51C
73A	53A	52A
71A	55A	52A
69A	54A	48A
74G	54G	47G
77G	55G	47G
77G	55G	45G
60T_18G	50T	42T
82G	54G	46G
87G	53G	43G
89G	53G	43G
92A	54A	45A
93G	55G	45G
93C	52C	45C
93A	53A	45A
93A	54A	43A
93A	56A	43A
93A	58A	44A
96G	22G_37C	20G_25C
97A	60A	46A
96G	60G	46G
96T	59T	47T
99C	63C	47C
99A	65A	46A
100G	67G	46G
101G	68G	45G
100G	66G	44G
101C	68C	43C
100T	69T	41T
103T	71T	43T
102T	73T	43T
104T	72T	43T
111A	72A	43A
111A	75A	42A

111A	75A	45A
113G	74G	45G
111C	73C	44C
108T	75T	45T
107G	77G	45G
108G	78G	44G
107T	77T	47T
110C	77C	47C
111C	79C	48C
112T	79T	49T
110A	78A	45A
112A	80A	43A
111A	81A	43A
111G	80G	42G
110A	82A	39A
109C	82C	41C
113C	85C	40C
110G	45A_41G	37G
108G	85G	37G
108G	85G	38G
109A	87A	40A
108G	87G	40G
110G	88G	40G
112A	85A	41A
110A	84A	39A
112T	82T	39T
112C	85C	41C
113G	86G	41G
114T	87T	42T
112C	86C	41C
118C	87C	42C
115A	85A	42A
116A	84A	41A
118C	85C	43C
117T	84T	43T
116C	85C	40C
117C	85C	37C
120C	87C	38C
123A	85A	39A
120T	84T	40T
121C	84C	39C
119A	84A	39A
120C	84C	40C

121C	85C	41C
121C	84C	40C
115T	86T	42T
111C	85C	41C
111A	84A	40A
111C	85C	43C
115C	85C	44C
114A	84A	45A
112G	84G	45G
113C	82C	45C
114C	81C	43C
111A	80A	43A
112T	80T	42T
111G	78G	41G
112T	79T	40T
108T	77T	40T
109G	38A_36G	40G
108G	73G	42G
107C	72C	42C
107T	71T	42T
105G	71G	43G
106A	70A	40A
107G	70G	40G
105G	67G	41G
102A	66A	41A
97C	66C	43C
95G	63G	43G
102A	62A	43A
100T	62T	42T
101G	62G	42G
101G	61G	42G
101G	60G	43G
100A	58A	41A
100G	60G	38G
99C	62C	39C
98T	60T	39T
98G	62G	39G
100T	62T	40T
98A	60A	40A
97G	61G	41G
95T	63T	41T
97G	63G	41G
98A	61A	41A

94G	60G	41G
93A	59A	41A
96A	61A	41A
98C	61C	38C
97T	59T	39T
97T	61T	39T
45T_51C	59C	38C
95G	61G	23A_13G
94G	63G	37G
97C	63C	36C
98A	61A	36A
97G	59G	36G
98G	61G	35G
98G	59G	36G
94T	62T	36T
97G	60G	35G
96C	64C	35C
96G	63G	35G
98A	65A	35A
95G	64G	35G
96G	63G	35G
93T	61T	35T
90T	61T	33T
91C	60C	33C
93A	59A	33A
92C	61C	33C
91C	60C	34C
91C	61C	34C
91A	62A	36A
94A	62A	36A
94G	61G	37G
95C	61C	36C
97G	62G	34G
96G	62G	33G
97C	62C	33C
97T	64T	33T
94T	65T	34T
98C	64C	33C
98A	63A	35A
98A	38A_24G	35A
101A	61A	36A
100A	63A	36A
99C	37T_26C	37C

99G	65G	37G
101A	65A	36A
104A	65A	37A
102A	66A	38A
102A	67A	39A
102C	67C	42C
101C	67C	43C
101C	68C	44C
102A	69A	44A
102A	67A	44A
104T	71T	22T_1G_21C
98G	72G	44G
99C	72C	43C
102A	73A	43A
103A	75A	42A
104C	74C	42C
104A	73A	42A
105C	72C	42C
104A	71A	42A
106G	72G	41G
106A	70A	41A
104G	72G	40G
107A	71A	40A
106G	71G	40G
109G	70G	40G
108G	36A_32G	40G
107A	70A	41A
107A	68A	41A
106C	70C	42C
104A	69A	41A
102T	68T	43T
102T	70T	41T
101T	67T	41T
99G	67G	43G
98T	66T	44T
99A	64A	42A
99C	66C	43C
97A	38A_32G	41A
94A	70A	41A
91A	69A	44A
91G	72G	42G
93A	70A	44A
91A	71A	45A

93G	69G	46G
94T	70T	47T
91C	70C	50C
93T	71T	49T
91G	72G	51G
91G	72G	53G
92A	73A	52A
91G	74G	53G
92G	75G	53G
95G	74G	54G
95A	73A	53A
94T	71T	52T
95C	73C	50C
94C	72C	49C
98C	73C	48C
97A	72A	49A
94A	70A	49A
93A	71A	48A
94G	72G	49G
95G	69G	51G
97C	69C	51C
96A	69A	52A
99G	71G	51G
99G	70G	50G
97G	70G	49G
49G_47C	72C	54C
97C	72C	53C
94C	74C	53C
91T	75T	53T
90A	29A_1T_47G	53G
91T	76T	51T
91T	75T	51T
92T	74T	48T
90G	75G	47G
90C	75C	46C
88T	77T	47T
88A	79A	46A
88A	76A	46A
88G	76G	45G
88G	76G	45G
83C	74C	47C
83T	72T	46T
84C	72C	48C

87A	75A	50A
86A	75A	48A
42A_44C	40A_36C	49A
84A	77A	47A
84A	78A	47A
85G	79G	49G
85A	79A	49A
88G	79G	49G
85G	78G	51G
84A	78A	51A
85G	76G	52G
87C	77C	51C
87T	76T	50T
86G	1A_38T_36G	51G
87C	77C	48C
90A	75A	49A
91G	74G	49G
94G	75G	47G
93A	75A	48A
95G	76G	48G
94A	74A	50A
96C	72C	50C
97A	76A	53A
100A	75A	50A
102A	72A	50A
100A	73A	49A
100C	73C	48C
97A	73A	47A
96G	73G	46G
98A	71A	44A
98G	72G	48G
96A	70A	48A
100G	68G	46G
99A	68A	46A
103A	68A	46A
101T	66T	48T
98C	67C	47C
102C	68C	46C
104A	33A_34T	22A_24T
103A	70A	47A
102A	71A	46A
102G	70G	47G
103G	69G	47G

106T	73T	48T
104A	73A	48A
103C	76C	48C
101A	76A	46A
104C	76C	45C
102A	77A	44A
99A	77A	45A
99T	77T	45T
98A	78A	46A
99A	77A	43A
102T	76T	43T
104A	74A	44A
106G	73G	45G
107C	71C	44C
107A	70A	44A
107T	70T	42T
109C	71C	45C
108C	71C	46C
112C	71C	46C
111A	70A	46A
111A	70A	47A
110C	68C	46C
110T	69T	47T
111C	68C	47C
113T	67T	46T
113C	67C	46C
115A	69A	45A
112G	69G	45G
110C	67C	49C
112G	35A_31G	49G
113G	65G	49G
113A	63A	49A
113G	60G	49G
109A	59A	49A
108C	58C	32T_16C
109T	60T	47T
111C	61C	46C
112T	62T	46T
111G	64G	46G
110G	63G	46G
111G	64G	46G
112A	63A	47A
112A	63A	47A

113G	63G	47G
110A	63A	47A
112G	63G	45G
113C	63C	43C
110G	63G	43G
109T	63T	43T
107G	60G	41G
107T	62T	42T
105G	61G	42G
106C	60C	41C
109T	59T	44T
109T	59T	44T
109C	57C	43C
107G	57G	42G
108A	59A	39A
114A	58A	38A
111G	57G	39G
111C	56C	40C
107A	56A	40A
108G	57G	39G
109A	56A	41A
106G	56G	41G
107T	56T	42T
111T	54T	42T
113T	56T	42T
110A	59A	43A
110A	58A	43A
109A	57A	42A
108A	57A	41A
109A	54A	40A
111G	53G	42G
109A	54A	41A
109A	52A	41A
109C	53C	41C
110G	53A	24A_18G
112G	51G	43G
113A	50A	43A
111G	50G	43G
109A	51A	42A
108G	52G	41G
106G	51G	40G
106A	52A	41A
107G	52G	44G

104G	51G	42G
106A	50A	41A
104C	52C	41C
106A	51A	41A
107G	51G	38G
107A	53A	39A
109A	52A	40A
110A	54A	41A
108G	55G	42G
109A	55A	44A
106C	53C	43C
108T	53T	44T
108A	52A	40A
107A	55A	41A
109A	52A	42A
107A	53A	42A
108G	53G	42G
109G	53G	42G
108G	54G	40G
107A	51A	40A
106G	52G	41G
105A	51A	41A
106G	49G	41G
105C	23T_26C	25T_17C
107T	49T	42T
106C	51C	43C
105G	50G	43G
106C	50C	42C
106T	51T	42T
107G	52G	42G
105G	49G	43G
105A	49A	42A
104A	50A	42A
104T	49T	41T
103T	46T	42T
104A	46A	42A
104G	46G	44G
102A	46A	42A
102G	48G	26G_16C
102T	48T	43T
103G	50G	43G
101T	48T	43T
97A	46A	43A

109A	52A	52A
110G	51G	51G
110C	52C	51C
108C	51C	50C
108A	50A	52A
110A	110A	103A
109A	38A	39A
105A	38A	38A
105A	35A	38A
106C	2A_9T_23C	38C
107A	57A	62A
106A	57A	62A
107A	58A	64A
108A	59A	65A
106C	49C	64C
105A	60A	64A
104A	61A	64A
105A	61A	63A
106A	61A	63A
105C	62C	62C
103A	63A	61A
103A	64A	61A
102A	63A	61A
102A	64A	60A
103C	66C	60C
102A	69A	59A
102A	68A	56A
101A	69A	56A
98A	61A	55A
97C	62C	55C
93A	57A	46A
96A	60A	46A
99A	60A	47A
93G	60G	44G
97G	61G	45G
98A	60A	45A
99A	63A	46A
99A	57A	42A
97A	59A	43A
99A	59A	44A
102A	31A_27C	13A_30C
100C	62C	41C
100A	62A	41A

99T	63T	39T
99A	64A	39A
102T	63T	40T
101A	63A	39A
106G	64G	41G
109G	66G	41G
106C	67C	41C
107T	67T	40T
108C	72C	42C
113A	72A	42A
111G	74G	43G
113C	75C	44C
110T	74T	41T
112C	74C	45C
115T	73T	44T
113G	75G	43G
115A	75A	42A
117A	76A	41A
117G	74G	42G
115C	76C	42C
117T	76T	41T
116C	79C	43C
113A	80A	41A
113A	83A	40A
113A	85A	41A
113G	87G	40G
114G	88G	39G
114G	87G	39G
115C	87C	39C
115A	86A	40A
116A	86A	39A
116A	82A	37A
115G	83G	37G
114A	85A	33A
115G	87G	32G
113A	87A	32A
114A	86A	30A
114A	84A	30A
116G	83G	30G
115A	84A	28A
113T	84T	27T
111C	85C	28C
112A	85A	28A

113T	86T	29T
112C	85C	31C
108C	85C	33C
108T	84T	32T
108A	84A	32A
109G	85G	14A_18G
110G	47A_38G	32G
110A	84A	32A
109T	83T	35T
111G	80G	34G
112T	80T	33T
116C	79C	36C
116C	79C	36C
119C	78C	35C
118C	79C	36C
113A	78A	36A
110A	80A	36A
107A	81A	35A
105A	81A	34A
105A	82A	33A
105C	79C	33C
103T	79T	34T
106G	77G	36G
103A	77A	34A
102G	77G	36G
102C	76C	36C
104A	77A	35A
104C	78C	36C
106T	77T	37T
107C	78C	37C
107A	78A	38A
101A	76A	37A
102G	76G	38G
102C	75C	41C
101A	74A	41A
101A	70A	40A
96A	69A	41A
94G	68G	40G
96G	66G	39G
95G	66G	38G
96G	66G	38G
94T	31T_35G	38T
96T	64T	38T

95T	64T	38T
96T	64T	39T
100T	65T	38T
100T	63T	38T
101T	61T	38T
101G	60G	37G
102A	58A	38A
102G	56G	38G
100A	54A	36A
100C	54C	36C
100C	55C	36C
99C	54C	37C
99C	54C	37C
97T	54T	36T
99G	57G	36G
98G	57G	39G
99C	58C	39C
41A_58G	29A_27G	39G
100G	55G	39G
100G	55G	38G
96A	57A	37A
97G	58G	38G
96A	59A	37A
100T	56T	39T
103A	55A	39A
105G	54G	38G
104A	53A	37A
104C	51C	34C
107A	51A	35A
108A	51A	33A
111A	52A	34A
112A	53A	34A
111G	51G	35G
110G	50G	36G
111G	51G	36G
110A	52A	31A
108T	53T	33T
106G	25A_27G	33G
103G	52G	31G
106G	51G	30G
104A	51A	31A
106G	49G	30G
109C	49C	31C

106A	47A	30A
105T	43T	29T
106C	48C	30C
105C	47C	32C
104T	47T	32T
103T	49T	31T
101C	49C	30C
100C	49C	29C
103C	49C	30C
103A	46A	30A
100G	46G	7A_23G
103C	45C	30C
102C	45C	29C
56A_47G_1C	44A	28A
105C	47C	27C
105A	47A	29A
103G	48G	27G
103A	51A	25A
105G	50G	25G
102G	52G	27G
105A	52A	26A
104A	52A	27A
103A	50A	27A
103T	51T	26T
104T	51T	26T
107A	48A	25A
105A	46A	25A
105G	48G	24G
102G	47G	23G
100A	46A_52G	23A_22G
102G	53G	25G
100G	52G	25G
100G	52G	25G
100G	53G	24G
100G	53G	24G
100G	52G	24G
95G_98C	50G	24G
104C	48C	22C
100A	49A	23A
100T	50T	23T
103A	48A	23A
103A	48A	20A
101T	48T	21T

99T	49T	21T
98T	49T	23T
99T	50T	25T
100C	46C	25C
102C	47C	25C
100C	48C	8T_17C
97T	50T	26T
95T	51T	26T
95A	52A	26A
97A	53A	26A
97G	54G	28G
95T	55T	27T
97G	55G	28G
94G	56G	25G
94T	56T	26T
94T	55T	27T
96G	55G	29G
95T	54T	29T
99G	52G	30G
100C	53C	30C
101C	56C	29C
102C	56C	31C
99A	57A	31A
98A	57A	30A
97G	58G	30G
96C	58C	30C
96T	58T	30T
95T	57T	31T
97T	56T	30T
95C	57C	31C
93C	57C	30C
95C	56C	30C
95C	57C	31C
95C	57C	30C
92T	59T	30T
92A	54A	31A
92T	61T	31T
91T	60T	31T
89T	58T	32T
87T	59T	32T
86T	57T	31T
88A	56A	30A
90T	58T	30T

89A	57A	31A
88G	54G	30G
90G	54G	30G
89C	54C	31C
89C	56C	30C
89A	56A	30A
88G	56G	31G
85A	58A	31A
82A	59A	31A
82G	58G	31G
82C	61C	32C
40A_43G	61G	32G
80G	57G	31G
79A	57A	29A
80C	57C	30C
79T	57T	31T
79G	57G	31G
78T	58T	31T
77T	57T	30T
77C	61C	32C
72A	61A	32A
74G	61G	32G
75G	61G	30G
74T	61T	30T
72A	61A	31A
72G	60G	31G
72T	61T	30T
72G	62G	31G
69A	62A	29A
68T	62T	28T
67G	61G	27G
63A	61A	28A
65T	60T	30T
65G	59G	27G
64A	56A	30A
63T	58T	30T
63G	61G	30G
63G	58G	28G
62C	59C	28C
59A	59A	27A
60G	57G	27G
59G	58G	26G
57A	57A	26A

55T	57T	23T
56G	57G	24G
51C	60C	23C
52T	60T	8A_23T
51G	59G	22G
47C	58C	23C
48G_72C	57G	16G_7C
46C	57C	22C
46C	57C	24C
49C	55C	25C
49C	55C	24C
50C	55C	23C
49C	56C	24C
49C	54C	23C
50C	55C	24C
46T	54T	22T
50T	54T	23T
49T	53T	24T
49T	50T	24T
49C	49C	24C
50A	49A	24A
50G	50G	23G
51G	51G	23G
51A	49A	23A
49G	50G	23G
49G	49G	22G
49C	46C	23C
48A	46A	22A
50A	47A	24A
55C	49C	24C
56C	48C	23C
54C	46C	24C
57A	47A	20A
57C	49C	23C
55C	48C	24C
54C	48C	24C
55C	47C	25C
56T	46T	24T
58T	47T	23T
57T	47T	24T
55G	46G	24G
59C	45C	24C
59C	45C	24C

59C	46C	25C
60T	46T	25T
59G	46G	25G
58A	43A	26A
58A	44A	26A
59C	45C	26C
60T	45T	26T
61G	46G	26G
61C	48C	26C
62T	49T	28T
62T	47T	29T
63C	46C	27C
63C	47C	28C
63T	47T	28T
64G	49G	28G
65A	49A	27A
68G	50G	27G
70A	49A	27A
69A	49A	25A
70T	48T	25T
70C	45C	26C
69G	45G	28G
69G	45G	27G
69C	44C	27C
70C	45C	28C
69T	44T	28T
70C	46C	29C
70C	48C	28C
68A	47A	28A
68G	47G	28G
68A	47A	27A
70C	47C	26C
71A	47A	27A
72A	46A	28A
73T	47T	29T
74C	47C	29C
70T	47T	27T
70T	47T	26T
70C	49C	28C
69C	48C	26C
69A	49A	27A
67A	50A	26A
69G	50G	26G

68G	49G	25G
71C	50C	24C
71A	50A	23A
74G	51G	24G
42A_2G_28C	27A_24C	24C
72C	51C	24C
71G	50G	25G
71C	50C	25C
72A	50A	27A
41T_30C	27T_23C	28T
72G	50G	28G
72G	51G	28G
70A	49A	26A
65G	50G	25G
64C	50C	26C
66C	49C	25C
63A	51A	26A
63A	49A	12A_14T
64G	48G	26G
65C	47C	25C
65C	50C	25C
66T	50T	26T
64C	51C	26C
64A	54A	27A
64C	54C	26C
62T	54T	26T
64C	54C	26C
65T	53T	26T
66T	52T	25T
67T	52T	26T
67C	52C	26C
67C	50C	29C
67A	49A	29A
66A	50A	28A
65A	48A	28A
67T	46T	28T
66G	45G	27G
66G	46G	26G
64G	45G	27G
66A	44A	27A
70A	45A	26A
69G	43G	27G
66G	42G	29G

62A	40A	28A
60A	41A	29A
61G	41G	29G
59G	42G	30G
59A	43A	28A
61A	43A	27A
63G	42G	27G
63G	42G	30G
63A	40A	30A
64A	41A	30A
65G	40G	31G
66G	38G	30G
65C	39C	30C
64C	37C	32C
64A	37A	32A
63A	17A_21G	33A
62C	39C	34C
60C	39C	33C
58T	38T	32T
57A	39A	31A
57C	40C	32C
58A	41A	33A
57A	38A	33A
56A	39A	33A
57G	37G	32G
57C	37C	33C
55C	38C	32C
54C	38C	33C
51T	38T	33T
50T	38T	35T
50C	39C	35C
50C	37C	34C
49A	38A	34A
48G	39G	34G
48T	39T	32T
46C	39C	31C
47C	39C	30C
47C	39C	14G_15C
50T	38T	29T
50C	37C	29C
50T	37T	30T
50G	37G	30G
51A	39A	30A

51G	36G	29G
52A	36A	30A
53A	36A	29A
52C	36C	29C
51A	33A	29A
49G	32G	27G
49G	31G	27G
49G	28G	28G
49A	28A	29A
19A_28C	29C	29C
44C	30C	29C
47A	32A	28A
46G	32G	28G
45A	31A	25A
46A	31A	25A
45A	31A	23A
43G	31G	23G
44A	30A	23A
45G	31G	23G
43A	31A	21A
43A	33A	22A
42G	31G	21G
46G	33G	21G
44C	32C	21C
40G	31G	21G
41C	32C	20C
43T	33T	18T
43A	33A	18A
44A	32A	18A
42G	32G	18G
42C	32C	17C
42C	31C	17C
40A	31A	17A
38A	33A	17A
40G	34G	15G
41G	34G	14G
40A	33A	14A
38A	32A	13A
38C	33C	13C
39A	32A	13A
41G	33G	12G
41C	32C	12C
40C	30C	12C

40A	31A	11A
1A_39G_45C	29G_15C	11G_5C
46C	17C	9C
47C	30C	12C
47C	31C	11C
47C	29C	11C
47C	32C	5A_15C
47C	31C	11C
45C	31C	13C
48C	31C	12C
41A	31A	12A
41A	31A	14A
43T	31T	14T
43G	31G	15G
43C	30C	15C
44A	34A	15A
42G	32G	16G
43A	32A	16A
42A	33A	17A
45G	33G	19G
45A	34A	20A
45G	35G	20G
45C	35C	21C
45A	35A	21A
46G	37G	21G
46G	37G	21G
46G	35G	21G
43T	35T	21T
46T	35T	21T
46G	35G	22G
46T	34T	22T
50T	35T	22T
50G	35G	22G
50G	37G	22G
49C	38C	23C
47A	37A	7A_18T
48C	38C	27C
49C	38C	26C
49C	37C	26C
51A	36A	26A
51C	35C	27C
22A_28G	16A_19G	27G
49G	19A_17G	26A

50T	42T	27T
52A	43A	27A
52T	43T	27T
55C	44C	28C
55G	46G	28G
55G	46G	28G
55C	45C	28C
55C	46C	27C
54T	46T	27T
54G	45G	27G
53C	46C	27C
53A	47A	18A_9G
54G	47G	27G
55C	47C	30C
55T	47T	30T
56T	47T	29T
55C	47C	30C
54T	47T	9T_21C
53T	47T	33T
54C	27T_21C	34C
55T	50T	35T
55G	49G	36G
53A	50A	35A
56G	53G	37G
57G	54G	36G
57G	54G	12A_25G
57C	54C	35C
55G	52G	35G
55G	54G	35G
60A	54A	35A
59C	55C	25T_1G_10C
62G	54G	37G
63C	56C	39C
64C	55C	39C
66A	56A	39A
72A	58A	38A
71G	59G	39G
38T_34G	58G	39G
72A	59A	37A
71T	58T	36T
74C	58C	36C
77C	59C	38C
76T	57T	36T

78G	56G	37G
78G	55G	37G
79G	54G	36G
79G	56G	35G
77C	57C	34C
77C	57C	34C
78A	57A	33A
76A	60A	33A
77C	59C	35C
77C	59C	35C
43A_34G	59G	35G
76C	60C	34C
76C	61C	36C
76T	61T	36T
76T	61T	35T
76G	61G	35G
76G	61G	34G
77A	58A	33A
78G	58G	35G
78G	58G	33G
76C	56C	32C
75A	57A	32A
75G	55G	32G
72G	54G	32G
72G	53G	30G
71C	51C	31C
73G	28A_23G	10A_22G
73G	50G	32G
72C	49C	32C
72C	48C	31C
70G	48G	32G
71T	44T	33T
71G	46G	34G
71A	46A	32A
74G	46G	34G
73G	46G	34G
76C	47C	34C
76A	46A	32A
76C	45C	33C
77T	45T	32T
76G	45G	31G
74C	46C	30C
73A	46A	30A

76C	46C	30C
77C	45C	31C
79C	45C	30C
79C	44C	29C
79C	42C	29C
80T	44T	28T
79T	43T	30T
78A	41A	29A
76C	41C	29C
75C	42C	29C
73C	43C	29C
72T	46T	29T
73G	44G	31G
67T	45T	29T
68G	46G	31G
69C	47C	29C
67C	47C	28C
68G	45G	29G
64T	45T	28T
60C	45C	31C
58T	45T	30T
59T	46T	30T
59G	46G	30G
59C	47C	30C
57C	48C	30C
54C	46C	30C
56C	45C	30C
53C	46C	29C
54T	44T	28T
52C	46C	28C
53C	45C	28C
48A	45A	29A
54G	48G	29G
53G	48G	29G
53G	46G	28G
53C	46C	29C
52C	45C	29C
52C	45C	30C
52A	46A	30A
52A	18A_29G	19A_11G
54T	47T	29T
53C	50C	30C
52C	52C	30C

51T	52T	30T
51C	33T_19C	11T_18C
51A	52A	28A
51A	52A	28A
52T	52T	26T
56C	52C	27C
56C	52C	27C
56C	52C	27C
55A	52A	27A
59C	52C	27C
57C	52C	26C
56A	52A	26A
58C	52C	26C
60C	52C	27C
60C	52C	28C
56A	49A	26A
61C	51C	11T_14C
60G	50G	25G
60C	51C	25C
60C	51C	26C
55T	50T	26T
57G	50G	26G
57C	50C	29C
56C	51C	30C
56C	50C	29C
57C	49C	30C
57A	49A	28A
58G	51G	30G
55T	51T	28T
54C	50C	29C
52T	50T	29T
53C	49C	30C
53T	49T	30T
57C	48C	28C
56T	47T	28T
57A	46A	29A
57C	45C	29C
57T	45T	28T
56A	41A	29A
58C	40C	26C
59C	41C	27C
58C	39C	26C
57A	38A	27A

57G	37G	28G
55A	35A	11A_15G_1C
56A	35A	27A
62G	33G	27G
62G	34G	26G
63G	33G	26G
64A	33A	26A
63C	31C	28C
66G	33G	28G
65G	33G	26G
65G	33G	25G
64A	30A_8G	25A
65G	32G	27G
66G	34G	27G
66G	34G	27G
67G	32G	27G
67G	29G	27G
68G	30G	27G
68G	31G	25G
70A	31A	27A
73G	33G	28G
77A	33A	25A
76G	33G	27G
80G	30G	26G
80G	30G	26G
78G	29G	24G
79C	29C	26C
82C	29C	26C
84C	30C	27C
85T	32T	27T
88T	34T	30T
89G	35G	30G
87T	36T	29T
91G	40G	29G
90C	41C	31C
95G	40G	31G
91C	41C	32C
90A	42A	33A
91A	27A_15T	33T
90G	43G	33G
88C	45C	33C
93G	46G	31G
93C	46C	33C

90A	46A	33A
89A	45A	34A
89C	48C	35C
90T	50T	35T
92C	51C	35C
94A	51A	35A
98A	50A	34A
97G	50G	34G
99T	50T	34T
99C	53C	34C
102C	55C	35C
101G	52G	34G
102C	52C	36C
103T	55T	36T
104T	56T	36T
103G	57G	36G
103G	58G	36G
101C	59C	37C
103A	61A	37A
105T	61T	37T
104G	62G	37G
105G	63G	38G
110A	66A	37A
111G	66G	38G
110C	67C	39C
111A	68A	39A
113T	67T	41T
114T	68T	39T
115T	69T	42T
113C	72C	42C
111G	75G	41G
114A	72A	43A
112T	74T	42T
112G	75G	41G
112A	74A	39A
111G	74G	38G
111C	76C	39C
112A	76A	40A
112A	77A	40A
113A	76A	41A
112T	75T	42T
114T	74T	42T
115G	75G	41G

114T	72T	39T
115T	75T	41T
117G	75G	39G
117C	76C	38C
114A	76A	37A
111A	77A	37A
113G	81G	38G
109G	81G	41G
108A	81A	42A
110A	81A	42A
110C	82C	44C
109C	85C	45C
107T	85T	45T
107C	83C	44C
103T	82T	43T
104C	83C	44C
103C	84C	44C
102G	81G	45G
103C	81C	45C
101T	82T	45T
102C	82C	44C
102A	81A	43A
101G	82G	43G
104G	81G	42G
104T	80T	45T
101G	79G	45G
100C	78C	47C
103T	79T	46T
105T	81T	46T
107G	80G	46G
109T	78T	47T
108G	78G	47G
107G	79G	48G
103T	79T	46T
103A	79A	45A
103C	79C	45C
102A	77A	44A
99G	77G	44G
99G	77G	43G
100T	78T	42T
99A	76A	42A
98C	75C	44C
99T	77T	45T

99C	76C	45C
100C	75C	46C
97T	73T	45T
95C	75C	45C
94G	75G	45G
94G	75G	47G
90C	74C	47C
88C	75C	47C
89T	73T	46T
91G	72G	46G
89C	72C	47C
88A	71A	49A
88T	70T	47T
89G	67G	47G
88C	67C	46C
88T	68T	46T
90G	68G	46G
90A	67A	46A
87G	68G	50G
88G	68G	49G
88G	67G	50G
86A	70A	51A
87C	72C	51C
85C	72C	52C
86G	71G	53G
86G	71G	55G
83A	71A	54A
85T	73T	58T
83G	72G	58G
83T	71T	56T
82C	72C	57C
82G	70G	58G
81G	70G	58G
79C	66C	56C
82C	66C	55C
83A	64A	53A
83G	63G	54G
81C	62C	52C
85C	60C	51C
87G	59G	51G
88G	59G	51G
89A	57A	51A
85G	55G	51G

85G	56G	53G
82A	55A	51A
83G	54G	51G
82C	54C	52C
81A	54A	52A
83G	54G	52G
88C	54C	54C
86T	60T	53T
85G	58G	53G
86C	59C	53C
85C	58C	51C
84G	59G	51G
84G	59G	51G
82A	57A	54A
81A	59A	52A
80T	59T	51T
81T	57T	51T
82T	59T	51T
86G	61G	54G
86T	61T	54T
86C	62C	55C
86C	60C	54C
88G	61G	53G
89T	60T	53T
90G	59G	55G
92G	60G	53G
89T	58T	51T
94G	62G	51G
93T	61T	49T
97G	60G	49G
100A	59A	48A
101G	59G	48G
102G	59G	46G
100A	58A	47A
100T	60T	46T
100A	60A	46A
98G	66G	45G
97T	66T	42T
97G	66G	40G
95G	66G	40G
97C	68C	41C
97A	65A	41A
95G	65G	40G

94A	64A	39A
94T	65T	39T
96C	65C	37C
97G	67G	37G
95T	67T	34T
98G	64G	33G
97T	66T	34T
100A	66A	34A
100C	66C	31C
97T	66T	30T
97C	64C	31C
97C	64C	30C
104A	63A	30A
102G	64G	33G
102C	63C	33C
102A	63A	34A
102G	62G	34G
105G	64G	35G
101G	62G	35G
99C	64C	36C
48A_51G	65G	38G
99G	66G	40G
95C	65C	40C
46A_51G	68G	24A_17G
97T	70T	41T
94T	74T	39T
97G	76G	39G
101G	76G	40G
101C	75C	40C
100T	75T	40T
100T	75T	42T
105T	76T	41T
105C	77C	43C
99T	79T	42T
99C	75C	42C
98C	75C	44C
96T	75T	44T
96G	77G	45G
96G	76G	45G
95G	77G	44G
98C	80C	41C
99G	80G	41G
99T	79T	42T

98C	79C	41C
100T	78T	44T
97T	76T	42T
99T	77T	42T
99C	75C	44C
101A	75A	44A
101C	74C	44C
100G	74G	45G
100A	75A	45A
100G	77G	46G
102G	77G	46G
99C	74C	47C
97T	74T	46T
95C	75C	47C
93G	76G	48G
94A	78A	48A
93G	76G	48G
94T	77T	51T
94T	75T	50T
93C	75C	52C
94T	72T	53T
96C	74C	54C
96C	75C	55C
93A	76A	58A
96C	74C	58C
95G	75G	59G
99T	76T	59T
99A	78A	62A
100T	79T	62T
99T	78T	62T
98T	76T	62T
99G	75G	62G
98A	75A	61A
98C	74C	62C
96A	74A	61A
97T	78T	61T
99C	79C	61C
99T	82T	58T
98G	81G	57G
92C	81C	23T_35C
92A	80A	56A
92A	79A	56A
95C	80C	35T_20C

97G	82G	55G
93A	80A	54A
93A	82A	53A
96A	76A	51A
66A_85T_66G_6C	60A_72T_60G_4C	24A_12T_57G_3C
60A	46A	34A
60A	45A	34A
56G	46G	32G
54G	45G	32G
52A_2G_33C	40A_2G_31C	30A_2G_13C
100A	82A	49A
97A	83A	47A
98G	84G	46G
102G	84G	48G
100G	83G	48G
98A	84A	50A
97G	83G	51G
99A	87A	50A
99G	86G	50G
102G	86G	49G
103C	86C	48C
105G	88G	50G
108G	85G	52G
108A	86A	51A
108G	84G	52G
106T	86T	52T
109G	86G	50G
109A	87A	51A
112G	86G	50G
112G	85G	51G
112C	86C	51C
115C	88C	56C
115T	89T	56T
118G	91G	52G
118A	92A	51A
120C	94C	53C
122A	94A	52A
120G	94G	53G
119G	94G	54G
119C	95C	29T_26C
117G	93G	56G
116A	89A	58A
116G	90G	58G

114A	88A	58A
115G	88G	54G
114G	89G	55G
115C	91C	55C
111G	92G	57G
112G	92G	55G
112G	92G	53G
114C	91C	29T_23C
111G	92G	53G
113G	92G	51G
113T	93T	51T
113T	91T	51T
113T	91T	49T
116C	96C	50C
49T_65C	96C	50C
110T	96T	51T
48A_61G	96G	50G
108T	96T	51T
110A	99A	49A
49T_62C	101C	52C
113G	102G	54G
113C	98C	26T_28C
117C	95C	56C
119A	96A	55A
121T	95T	56T
118C	94C	56C
117C	91C	56C
114A	90A	56A
114G	91G	56G
113G	90G	56G
109T	89T	54T
106G	87G	54G
106G	84G	54G
103T	82T	53T
104T	79T	54T
104T	78T	54T
105T	77T	53T
101T	76T	51T
101A	74A	51A
101A	73A	50A
97T	73T	49T
96T	72T	46T
97C	71C	46C

98T	70T	44T
97A	66A	43A
97A	66A	43A
94G	66G	43G
94A	66A	43A
92A	65A	41A
45C	2A_1G	3A_6G_21C
44G	1G	1A_3G
43A	1A	3A
44A	1A	3A
46A	1A	3A
47A	1A	3A
47A	1A	null
46A	1A	null
44A	1A	null
42G	1G	null
41A	null	null
36G	null	null
35C	null	null
34A	null	null
34T	null	null
35C	null	null
36T	null	null
36T	null	null
36T	null	null
36T	null	null
37C	null	null
38C	null	null
38T	null	null
39G	null	null
38A	null	null
38G	null	null
39G	null	null
45A	1A	5A
44G	1G	5G
44G	1G	8G
41G	1G	9G
41G	1A	9G
35A	8A	9A
42A	8A	9A
43A	8A	9A
43A	8A	9A
43A	8A	9A

44A	8A	9A
43G	8G	9G
43G	8G	9G
46G	8G	9G
45G	8G	9G
47A	8A	9A
46G	8G	9G
46T	8T	9T
45G	8G	9G
45T	8T	9T
44G	8G	9G
44G	8G	9G
44A	8A	9A
44A	8A	9A
45C	8C	9C
42C	null	null
41A	1C	null
46G	3G	null
47T	3T	null
47A	3A	null
49T	3T	null
49A	3A	null
50G	4G	null
48A	4A	1A
51C	4C	1C
51C	4C	1C
51A	4A	1A
53G	4G	1G
53A	4A	1A
55C	4C	1C
55G	5G	1G
55G	6G	1G
56G	7G	1G
55C	7C	1C
55G	8G	1G
54G	8G	1G
53G	8G	1G
51A	8A	2A
50T	8T	3T
49A	8A	3A
48C	8C	2T_1C
48A	8A	3A
48A	8A	3A

47A	8A	1A_2G
48T	8T	3T
47C	8C	3C
110A	8A	4A
33A	8A	4A
31A	8A	4A
30T	8T	4T
30A	8A	5A
30A	8A	5A
30A	8A	5A
29T	8T	5T
43A	8A	5A
43A	8A	5A
42A	8A	5A
42T	8T	5T
43A	8A	6A
42A	8A	6A
42A	8A	6A
42T	8T	6T
44A	8A	6A
45A	8A	6A
33A_12G	4A_4G	5A
47T	8T	6T
46A	8A	6A
45A	8A	6A
44A	8A	6A
44T	8T	6T
44A	8A	6A
45A	8A	6A
46A	8A	6A
10A_31T	4T	5T
31A	4A	5A
29A	4A	5A
30A	4A	5A
30T	4T	5T
30A	4A	5A
30A	4A	5A
30A	4A	5A
31T	4T	5T
30A	3A	5A
27A	3A	5A
26A	3A	5A
26A	3A	5A

26A	3A	5A
26T	null	5T
26A	null	5A
27A	null	5A
28A	null	5A
29T	null	5T
30A	2A	5A
30A	2C	5A
34A	2A	8A
37A	2A	9A
38C	4C	10C
37C	5T	5T
66G	38G	23G
66C	38C	24C
66A	41A	24A
70A	43A	24A
72A	43A	26A
33T_39C	46T	26T
76A	48A	29A
79G	50G	30G
80T	51T	30T
81G	51G	30G
84C	53C	30C
82T	53T	30T
83G	54G	31G
84T	54T	31T
84T	55T	32T
87C	55C	33C
87T	56T	32T
87C	56C	33C
86C	59C	1A_16T_1G_15C
86G	62G	33G
88G	61G	34G
89A	62A	35A
89T	63T	36T
89A	63A	36A
89A	64A	37A
90A	66A	38A
90T	66T	38T
91G	68G	39G
90T	66T	39T
91A	67A	40A
94A	70A	40A

92A	67A	41A
91A	68A	41A
91A	70G	41G
90T	69T	42T
90T	72T	41T
90C	72C	40C
88A	72A	41A
89A	74A	39A
90G	68G	39G
92A	75A	40A
93C	78C	41C
92A	78A	40A
93C	80C	40C
94A	80A	41A
94G	82G	41G
94G	83G	42G
95A	85A	41A
94A	84A	40A
94G	85G	41G
98C	86C	39C
103C	84C	40C
103T	84T	38T
107C	84C	37C
107C	84C	39C
106T	85T	38T
106A	86A	38A
107T	84T	40T
107T	84T	40T
107G	83G	40G
107A	83A	40A
110C	82C	44C
114T	82T	42T
113G	81G	45G
112T	81T	47T
113G	81G	46G
113G	81G	44G
114A	82A	42A
114G	82G	41G
114A	84A	41A
112T	83T	42T
63A_48G	84G	41G
110C	82C	42C
108A	82A	43A

105G	82G	43G
107A	80A	42A
102A	79A	41A
97G	77G	40G
101G	78G	41G
98A	81A	44A
96C	81C	43C
103C	84C	45C
103C	85C	46C
104T	85T	46T
106T	85T	45T
105T	87T	46T
105G	87G	49G
107C	89C	51C
111C	87C	51C
109A	85A	53A
109C	87C	52C
111T	90T	51T
112G	91G	52G
113G	91G	53G
113C	92C	55C
115A	90A	54A
116G	91G	54G
116A	92A	53A
115T	94T	54T
113T	93T	53T
111A	90A	52A
111G	91G	51G
113C	91C	51C
113T	88T	52T
113G	89G	52G
113G	88G	54G
114T	88T	54T
114G	89G	54G
114A	87A	55A
112T	90T	56T
112G	87G	56G
111C	85C	56C
117C	87C	55C
117A	85A	56A
116C	87C	55C
117G	85G	55G
115G	83G	55G

116A	82A	55A
118A	82A	54A
118T	81T	56T
116G	81G	56G
110T	82T	57T
112T	82T	59T
108C	83C	63C
108A	84A	62A
111C	82C	62C
112G	84G	62G
113A	84A	33A_26G_1C
115A	86A	59A
115C	84C	60C
116A	88A	60A
117T	89T	58T
115T	90T	56T
116T	88T	54T
118T	89T	55T
114A	90A	57A
109A	90A	59A
111T	92T	60T
112T	90T	62T
115T	89T	62T
116C	92C	61C
117C	92C	63C
118A	95A	63A
118A	35A_58G	40A_23G
122T	94T	61T
120G	98G	64G
121A	98A	63A
123G	97G	65G
119G	97G	65G
118T	95T	65T
116G	93G	65G
110G	89G	62G
111G	88G	62G
109G	88G	63G
111G	88G	64G
112A	90A	63A
113G	88G	61G
114A	88A	59A
111A	92A	60A
111A	91A	59A

111T	94T	58T
109C	89C	60C
110C	90C	58C
112C	41T_49C	57C
112T	89T	55T
112C	90C	56C
111C	88C	56C
110T	87T	55T
109C	85C	58C
112C	84C	58C
112T	85T	59T
116A	85A	60A
119A	86A	60A
119G	88G	59G
121C	86C	60C
122A	83A	59A
122G	83G	59G
126G	86G	59G
128A	87A	59A
129A	84A	59A
128A	85A	58A
127A	84A	57A
128A	84A	57A
125G	84G	55G
127G	86G	55G
127G	86G	56G
127G	85G	56G
130T	86T	56T
129T	86T	56T
129A	87A	54A
126G	87G	55G
54G_75C	89C	55C
128A	91A	52A
130C	90C	48C
130G	89G	50G
129G	90G	51G
127A	89A	50A
127A	92A	52A
123T	92T	52T
127A	93A	51A
125T	91T	51T
127T	43T_50C	52T
123C	96C	53C

121A	96A	53A
120C	96C	53C
119A	97A	53A
122A	97A	51A
119A	96A	53A
118C	95C	51C
115T	94T	51T
116G	92G	53G
116T	93T	50T
117C	90C	51C
121C	90C	49C
118T	93T	50T
117G	88G	47G
119T	88T	47T
121T	90T	45T
121T	91T	45T
125T	90T	43T
126C	90C	43C
126T	90T	44T
126A	90A	45A
123A	92A	45A
124A	91A	44A
119G	87G	43G
120A	88A	44A
119A	87A	44A
49A_67G	82G	43G
68G_50C	83G	42G
123C	79C	41C
123A	79A	42A
123A	79A	43A
123G	82G	45G
124T	81T	44T
123T	81T	44T
122A	82A	45A
49A_123G	94A_80G	51A_43G
129A	83A	45A
129A	83A	46A
128A	82A	45A
127A	82A	44A
122A	80A	45A
120A	78A	44A
119A	80A	45A
116A	80A	45A

109T	75T	43T
109C	75C	44C
106C	75C	44C
106T	73T	44T
105C	73C	43C
99A	71A	44A
100A	70A	44A
99G	67G	43G
98T	66T	43T
96G	65G	42G
94A	64A	42A
93C	64C	42C
81A	46A	23A
82G	1A_46G_20C	24G
81G	36G	23G
51A_32C	20A_16C	23C
32A_51G	16A_20G	23A
83A	37A	24A
82G	38G_14C	25G
84A	23A	26A
97G	50G	42G
99A	51A	43A
100G	52G	43G
100A	52A	44A
100A	54A	44A
99C	57C	44C
100A	63A	47A
99A	64A	46A
98G	64G	48G
98A	64A	51A
98C	67C	51C
99C	68C	52C
98C	67C	53C
97A	67A	52A
95T	68T	52T
95T	68T	53T
95A	69A	51A
93G	66G	54G
95C	67C	53C
97T	69T	55T
97G	67G	56G
96C	67C	55C
95A	66A	56A

96A	66A	56A
95A	67A	56A
95A	69A	57A
94C	70C	55C
95T	68T	55T
96C	69C	57C
98A	69A	55A
98T	69T	56T
100G	72G	57G
98T	73T	57T
96A	74A	57A
99G	79G	57G
99C	79C	55C
98C	79C	56C
95A	80A	54A
95T	82T	52T
97T	80T	51T
96G	81G	51G
98G	79G	49G
52T_46C	82T	51T
104T	84T	51T
104G	84G	52G
104A	85A	53A
108T	84T	54T
108G	86G	55G
108G	89G	54G
108G	87G	54G
109C	86C	53C
115T	84T	55T
117G	86G	54G
119T	85T	54T
121T	87T	53T
121A	90A	54A
122A	92A	52A
126T	92T	51T
126T	90T	51T
124C	90C	51C
125C	90C	50C
124A	90A	49A
125C	89C	48C
127T	90T	48T
124G	89G	48G
120A	89A	45A

119A	89A	44A
119A	90A	44A
120C	88C	43C
120A	88A	43A
118T	86T	42T
116C	87C	41C
116T	84T	42T
116C	86C	42C
118G	85G	42G
118T	83T	42T
117T	77T	39T
118T	77T	39T
119C	78C	39C
121T	79T	35T
122C	77C	38C
122A	78A	36A
124A	81A	35A
128G	81G	35G
130A	81A	36A
65A_66G	82G	37G
131A	81A	38A
130A	81A	35A
128A	79A	36A
130G	77G	36G
128T	77T	35T
130G	78G	35G
131G	80G	36G
130G	82G	38G
130A	84A	40A
130A	85A	39A
62A_68C	84A	39A
131G	87G	39G
68T_63C	88T	37T
134G	91G	37G
135T	90T	37T
136C	91C	36C
141A	90A	36A
140G	89G	36G
136A	82A	37A
135A	82A	39A
136C	85C	39C
141A	83A	39A
140T	82T	38T

138C	82C	38C
136A	81A	40A
135A	80A	40A
137C	80C	39C
133C	74C	39C
133G	40T_35G	37G
133A	77A	36A
129T	79T	36T
134A	80A	35A
135G	78G	34G
134C	79C	34C
134T	81T	36T
130C	81C	34C
129T	80T	35T
128T	80T	36T
126T	80T	39T
126C	80C	40C
128T	78T	41T
125C	79C	41C
124T	79T	41T
124T	82T	41T
124G	83G	43G
123G	84G	43G
121A	83A	44A
119T	84T	43T
121G	85G	42G
123A	85A	42A
123A	86A	45A
123T	85T	46T
122T	85T	46T
121A	41A_42G	45A
122A	84A	45A
122A	83A	45A
122T	39A_44T	20A_24T
123A	82A	43A
125G	84G	44G
127A	84A	45A
128T	85T	45T
126C	84C	45C
126A	86A	44A
125A	85A	19A_23C
126G	85G	21A_19G
123A	87A	40A

125C	86C	43C
122A	85A	44A
123A	86A	45A
122A	86A	44A
121G	87G	45G
122C	88C	45C
124A	89A	45A
117C	89C	44C
118A	90A	46A
117C	89C	47C
113A	89A	47A
113C	88C	45C
115A	85A	42A
113A	83A	43A
110A	83A	42A
109C	83C	43C
107C	82C	46C
109A	81A	46A
112C	81C	50C
113T	79T	50T
108G	79G	50G
110A	79A	51A
112T	79T	51T
113T	81T	50T
113T	78T	48T
109T	76T	25T_24G
109G	76G	49G
112A	77A	51A
113G	77G	49G
111G	76G	47G
107G	74G	48G
105G	74G	49G
106G	75G	51G
105T	78T	52T
105G	77G	51G
101C	78C	51C
101A	76A	51A
104C	77C	51C
106A	77A	51A
106A	77A	50A
110A	77A	49A
110T	41T_36C	48T
110A	75A	46A

110C	73C	44C
106A	73A	45A
106A	73A	46A
107C	74C	46C
110T	72T	46T
113G	71G	44G
114T	70T	43T
118T	71T	44T
119C	70C	45C
121C	70C	45C
123T	68T	47T
126G	67G	46G
125C	67C	45C
129T	70T	45T
129G	71G	45G
130C	70C	44C
130T	71T	46T
134T	73T	46T
132T	73T	47T
128C	70C	46C
125A	69A	45A
130T	71T	47T
133A	71A	48A
134T	70T	49T
135C	72C	50C
135C	72C	50C
135A	68A	52A
133T	65T	52T
132T	67T	52T
132G	66G	53G
133G	64G	53G
134C	66C	56C
132T	65T	56T
129G	66G	58G
132C	66C	60C
132T	66T	59T
133C	66C	58C
135A	64A	56A
136G	65G	57G
133C	67C	58C
133T	70T	60T
133G	71G	61G
132C	68C	59C

134T	68T	56T
131G	68G	58G
128A	67A	55A
130T	68T	56T
133G	68G	61G
134C	70C	59C
132C	70C	58C
132A	72A	59A
135C	75C	60C
135G	76G	60G
135G	76G	60G
134C	75C	59C
132A	75A	59A
131T	75T	59T
134G	77G	59G
136T	75T	56T
140T	76T	58T
141C	74C	57C
144A	78A	57A
145T	79T	57T
149A	81A	57A
145A	81A	58A
148A	80A	57A
146C	80C	59C
141A	80A	60A
140T	80T	63T
136T	80T	63T
137C	78C	66C
136A	78A	64A
137C	78C	63C
136A	77A	64A
131T	77T	64T
130T	77T	63T
131C	79C	63C
127C	80C	62C
126C	82C	66C
123A	81A	68A
120A	81A	67A
118C	84C	69C
118G	41A_42G	70A
118G	83G	70G
118C	82C	71C
116T	84T	71T

114T	83T	69T
111G	82G	67G
112G	81G	69G
115C	83C	69C
116A	87A	70A
111G	84G	69G
111A	85A	68A
110A	83A	68A
111T	81T	68T
111G	82G	70G
111G	81G	68G
111G	82G	69G
111A	81A	69A
108G	81G	68G
103A	81A	66A
103A	79A	63A
103A	80A	63A
103G	78G	63G
100A	78A	63A
99A	78A	61A
95G	78G	62G
93G	79G	64G
91A	76A	65A
89G	73G	62G
87C	67C	63C
86A	76A	65A
85C	78C	22A_65C
83A	76A	66A
65A	48A	67A
69A	49A	66A
65A	49A	65A
67A	50A	60A
70A	52A	60A
72A	52A	60A
69A	32A_16G	61A
73G	50G	58G
72C	52C	59C
74C	52C	59C
74A	52A	59A
76C	53C	59C
79C	54C	59C
80T	22A_33T	59T
80A	55A	59A

80C	56C	58C
82G	58G	58G
83T	57T	59T
87G	57G	58G
88A	58A	59A
90C	58C	59C
91C	60C	59C
92A	60A	58A
91T	29T_31C	56T
94A	60A	54A
95C	62C	54C
98C	29A_34C	52C
97A	64A	54A
96A	66A	55A
97A	66A	55A
97A	68A	55A
98A	69A	55A
99T	70T	55T
97C	68C	55C
97C	71C	50C
99C	72C	50C
101T	72T	54T
97C	72C	52C
96C	74C	53C
96A	74A	52A
94T	72T	52T
93T	74T	55T
92T	74T	55T
90T	74T	55T
92T	77T	54T
91G	77G	55G
94T	76T	55T
92T	78T	54T
91T	80T	54T
92G	81G	55G
92G	81G	52G
90A	81A	51A
89T	82T	52T
88C	84C	52C
91T	83T	52T
92G	85G	51G
90T	87T	52T
92G	86G	52G

88T	85T	51T
91G	85G	52G
94C	87C	51C
88A	85A	55A
87A	89A	53A
90A	90A	52A
94T	91T	52T
93A	92A	52A
92T	92T	51T
95G	96G	49G
94G	93G	48G
96G	96G	48G
94T	97T	46T
96G	97G	47G
97A	95A	47A
96A	96A	51A
96C	93C	53C
96T	94T	55T
96T	95T	53T
94C	95C	52C
95C	93C	54C
95T	92T	56T
96T	93T	56T
98C	94C	57C
95T	93T	57T
96C	95C	57C
97A	95A	57A
97A	96A	57A
97G	94G	57G
96A	93A	56A
95G	92G	56G
93A	91A	55A
92A	92A	57A
91G	91G	57G
89A	93A	55A
91G	95G	55G
88G	96G	58G
89G	93G	59G
87A	95A	59A
85G	95G	60G
83T	94T	59T
84G	95G	58G
85A	95A	57A

85G	93A	58A
84C	93C	55C
82T	94T	54T
86C	94C	56C
85A	94A	57A
84A	93A	52A
84T	91T	54T
86T	92T	52T
86G	91G	54G
87G	90G	57G
87G	89G	54G
88G	92G	55G
89C	91C	52C
87A	88A	52A
89T	43A_46T	53T
85A	91A	53A
88C	91C	52C
90A	89A	53A
90G	89G	53G
92G	88G	51G
94G	88G	53G
96A	88A	51A
96G	86G	49G
95G	86G	52G
95A	85A	53A
94A	84A	54A
96G	85G	54G
58A_43G	39A_46G	54G
98A	82A	53A
95G	84G	53G
96G	84G	51G
96C	81C	50C
93A	78A	53A
88A	79A	53A
90A	78A	55A
90G	78G	57G
87G	75G	53G
90A	76A	58A
91A	73A	58A
91C	72C	57C
90A	75A	60A
90T	76T	61T
90A	74A	60A

89G	76G	60G
91T	76T	57T
92A	77A	60A
96T	78T	61T
98G	77G	61G
97T	78T	59T
96A	78A	60A
94T	78T	60T
95T	79T	61T
95T	76T	60T
94G	76G	61G
96G	75G	62G
97G	74G	64G
97C	75C	63C
95A	77A	64A
96G	77G	63G
93G	78G	62G
93A	77A	64A
93T	76T	65T
91C	76C	66C
93T	77T	64T
96T	77T	62T
95G	75G	62G
95C	77C	60C
99C	77C	61C
97C	75C	61C
96C	75C	61C
95T	75T	61T
96T	74T	62T
94T	75T	63T
94A	76A	62A
95G	77G	64G
97A	76A	64A
97G	79G	61G
97C	76C	62C
98C	77C	62C
98A	79A	59A
100T	80T	58T
100G	78G	59G
99C	80C	57C
100A	83A	59A
99A	82A	60A
101A	83A	59A

99C	83C	62C
101T	83T	62T
100C	81C	62C
103C	86C	62C
101A	84A	63A
100G	85G	62G
101G	87G	62G
103G	89G	61G
104C	88C	59C
103C	87C	58C
103C	87C	57C
99A	86A	58A
100A	86A	58A
100A	84A	57A
100C	85C	56C
97A	85A	55A
97C	85C	53C
97G	84G	53G
101G	83G	50G
101C	82C	50C
100T	82T	50T
106G	81G	49G
104C	80C	49C
105A	81A	49A
104G	79G	48G
104T	79T	49T
102T	80T	50T
103C	78C	49C
102G	80G	51G
101T	79T	49T
100T	78T	48T
99G	81G	46G
101T	82T	46T
103T	83T	46T
105C	82C	46C
105C	82C	45C
104T	82T	43T
104C	82C	44C
109T	84T	45T
107G	83G	43G
109T	85T	46T
109A	84A	45A
108G	87G	46G

109A	86A	43A
110T	84T	46T
114G	84G	46G
113A	85A	45A
112T	84T	44T
107G	84G	45G
113G	40A_47G	44G
113A	86A	43A
110T	84T	43T
110G	87G	43G
110G	85G	41G
108A	87A	40A
108C	86C	45C
113A	84A	44A
113C	82C	44C
114A	79A	41A
113G	79G	41G
113A	79A	41A
58A_53T	79T	44T
109A	78A	44A
109C	78C	45C
108C	77C	46C
110T	76T	47T
110T	76T	47T
109T	74T	48T
108A	76A	47A
107A	75A	47A
107T	74T	44T
106C	75C	43C
106T	75T	42T
103C	76C	40C
101C	78C	41C
105C	82C	42C
104T	80T	43T
103G	80G	43G
53G_48C	29G_52C	43C
102C	82C	45C
100G	81G	45G
102T	84T	46T
101T	84T	46T
47T_59C	31T_51C	48C
105T	82T	47T
104C	82C	50C

105A	82A	53A
107A	83A	52A
101A	84A	54A
100G	85G	53G
99C	87C	54C
93A	88A	53A
94G	89G	52G
96C	90C	51C
96A	90A	50A
97A	90A	47A
99A	90A	42A
101C	90C	45C
99A	87A	44A
100G	86G	45G
101G	86G	44G
100C	83C	46C
99T	82T	45T
100C	80C	49C
100A	81A	50A
102G	81G	50G
106C	81C	51C
108T	81T	52T
102G	82G	52G
102A	82A	51A
103C	79C	52C
104A	78A	53A
105T	76T	52T
107G	78G	53G
107G	80G	51G
102A	80A	51A
106A	79A	50A
105A	78A	52A
105C	80C	52C
99C	79C	53C
101A	80A	53A
102T	80T	55T
105T	78T	56T
106A	78A	57A
107T	77T	59T
110C	78C	55C
107C	80C	56C
107C	81C	57C
106C	80C	56C

106A	81A	56A
106G	79G	55G
107C	81C	54C
110G	81G	52G
110C	81C	51C
109C	81C	49C
106C	82C	50C
108A	83A	49A
110G	82G	53G
109A	81A	52A
110G	82G	53G
109C	82C	53C
108C	81C	54C
109C	80C	58C
115T	75T	59T
121C	75C	59C
119T	69T	58T
121C	72C	58C
120C	71C	59C
123C	71C	61C
124T	71T	59T
123G	70G	59G
120G	68G	56G
118C	66C	52C
113G	67G	51G
113C	68C	49C
118G	73G	53G
122G	75G	52G
124G	79G	53G
130C	78C	53C
130A	78A	54A
132T	77T	53T
135G	76G	53G
135T	77T	52T
135T	76T	53T
135G	76G	54G
133T	78T	52T
131G	76G	58G
129G	78G	58G
131A	79A	58A
133A	79A	59A
130A	79A	59A
132A	79A	59A

131G	78G	57G
128A	79A	55A
125G	78G	55G
122G	78G	56G
121T	79T	54T
121C	78C	56C
120T	78T	53T
122T	80T	54T
123A	80A	55A
125G	80G	55G
124G	80G	54G
122A	77A	53A
122A	76A	53A
121C	76C	53C
125A	76A	53A
125C	76C	54C
126A	75A	53A
126A	74A	51A
126G	74G	53G
129G	74G	52G
127C	73C	52C
130G	77G	52G
130C	76C	52C
131A	75A	53A
130A	74A	54A
129A	73A	54A
129G	73G	54G
131G	73G	54G
132T	74T	55T
130T	73T	55T
128C	74C	55C
131T	73T	57T
135T	74T	57T
136T	74T	60T
137T	73T	61T
135G	77G	58G
135G	76G	59G
136T	75T	61T
135G	74G	59G
134C	73C	59C
133G	74G	57G
129A	77A	58A
125G	78G	58G

122A	79A	60A
122G	77G	60G
123G	78G	57G
119C	77C	57C
119C	78C	56C
124A	78A	57A
121T	82T	56T
125T	82T	56T
127A	82A	57A
127G	82G	57G
129C	81C	56C
127A	79A	56A
129G	76G	57G
124G	79G	57G
125T	78T	56T
126G	77G	57G
125T	79T	58T
123G	77G	58G
121A	76A	58A
124A	76A	57A
123G	75G	57G
125C	79C	55C
126C	81C	54C
125G	82G	53G
123G	82G	53G
125G	82G	53G
123G	82G	54G
125A	81A	55A
127G	81G	56G
131A	80A	54A
136T	79T	55T
133A	79A	54A
134A	80A	53A
135C	78C	52C
132C	77C	54C
131A	79A	51A
128G	80G	49G
129A	81A	51A
132T	81T	51T
130C	82C	51C
134C	82C	54C
71T_58C	84C	54C
135C	83C	52C

134T	82T	52T
133T	80T	54T
132T	84T	53T
128A	89A	53A
127A	89A	52A
121G	85G	52G
121C	84C	50C
118T	87T	48T
118G	86G	48G
117G	86G	47G
116C	85C	47C
114A	87A	48A
114G	86G	46G
115G	87G	48G
114C	91C	50C
116T	90T	49T
112G	89G	48G
111T	88T	45T
110T	88T	44T
114G	84G	45G
115T	83T	45T
118T	82T	43T
119G	83G	43G
120G	83G	44G
118G	82G	43G
118A	80A	16A_27G
118A	79A	41A
118A	78A	40A
117A	77A	41A
116A	76A	42A
116A	76A	41A
114G	76G	40G
113T	74T	39T
112G	70G	42G
107G	69G	40G
106G	69G	40G
108T	70T	40T
109G	70G	39G
109A	70A	41A
108T	71T	41T
109T	71T	41T
112T	74T	41T
110A	75A	41A

108T	74T	42T
109C	76C	45C
109C	76C	44C
106T	76T	44T
109C	75C	43C
110T	71T	43T
114A	68A	45A
117A	67A	46A
115T	69T	44T
116A	70A	47A
118G	70G	45G
119G	71G	47G
118C	73C	45C
112T	73T	45T
109T	75T	46T
111G	74G	46G
108A	77A	46A
107C	76C	48C
108A	76A	46A
110A	79A	46A
115C	78C	47C
118C	76C	46C
115C	76C	46C
117C	77C	48C
113C	78C	45C
114A	75A	45A
109G	77G	45G
109G	77G	22A_22G
110A	78A	43A
112G	75G	42G
111A	70A	42A
113G	70G	43G
115G	72G	42G
114A	72A	43A
116G	71G	42G
118C	71C	43C
118A	75A	42A
117A	77A	44A
121G	77G	45G
124C	79C	46C
126A	77A	44A
128G	73G	42G
124G	73G	42G

125C	73C	43C
125A	74A	45A
124A	72A	45A
122G	74G	42G
123T	76T	43T
119C	75C	43C
118A	75A	45A
115A	76A	42A
117G	79G	45G
117A	36A_42T	43A
116G	80G	44G
115C	83C	44C
117C	82C	44C
114C	83C	43C
117C	83C	42C
118G	82G	43G
115G	85G	43G
116A	85A	42A
116C	86C	42C
119T	86T	43T
120C	85C	42C
120T	85T	42T
121G	87G	41G
119G	87G	41G
117T	85T	39T
115T	85T	39T
117G	85G	38G
117A	85A	37A
116G	83G	36G
115A	81A	37A
116G	84G	38G
114G	83G	38G
113T	83T	38T
108C	82C	37C
103A	81A	37A
105C	78C	18T_19C
102G	77G	36G
103C	78C	35C
101G	78G	34G
100G	76G	32G
100A	76A	33A
102G	76G	33G
101C	76C	33C

99G	74G	34G
103G	77G	35G
103C	79C	35C
100A	75A	35A
93A	77A	35A
94C	76C	35C
95A	77A	35A
94T	75T	33T
94T	73T	35T
95A	74A	36A
98A	73A	38A
99C	72C	39C
99C	73C	40C
98C	75C	42C
101C	38T_37C	42T
102G	76G	43G
103G	78G	49G
102A	79A	49A
101A	79A	50A
99T	78T	52T
99G	76G	52G
104T	75T	50T
104T	76T	53T
98G	74G	51G
98A	78A	52A
97G	79G	54G
99G	80G	53G
99T	82T	54T
98T	82T	51T
99C	83C	52C
98C	81C	52C
96C	80C	52C
96C	80C	52C
97T	80T	51T
98T	79T	52T
102G	77G	53G
102A	76A	54A
101C	73C	56C
101A	72A	55A
100T	71T	53T
100T	73T	56T
99G	75G	58G
98A	74A	60A

99G	51A_21G	59A
97T	73T	59T
99C	76C	61C
97C	76C	60C
94A	75A	60A
92G	74G	60G
90T	74T	59T
90C	74C	60C
92G	73G	61G
91T	71T	63T
90G	70G	62G
91T	71T	61T
91C	70C	61C
93C	71C	60C
96G	70G	60G
98A	69A	61A
98C	69C	60C
98T	70T	58T
99C	72C	59C
97T	72T	60T
100A	71A	59A
97G	71G	59G
96G	70G	60G
96G	70G	60G
89C	50T_16G	29T_30C
86G	64G	29A_29G
86C	48T_16C	29T_1G_28C
30A_62G	16A_47G	61G
106G	71G	61G
107T	74T	61T
108G	73G	61G
114C	74C	62C
113T	75T	62T
115C	74C	62C
114A	74A	62A
119T	74T_42C	61T_21C
121C	74C	61C
116C	72C	59C
115C	25T_73C	58C
117G	71G	57G
119T	72T	54T
118C	72C	54C
116T	71T	50T

113C	70C	47C
115C	70C	46C
116A	73A	46A
116A	72A	45A
116G	73G	45G
112C	73C	43C
110C	76C	41C
110G	74G	43G
108T	74T	41T
109G	75G	42G
112G	74G	40G
64A_51G_1C	43A_1T_31G	40A
118G	76G	41G
118C	77C	41C
119C	76C	40C
120A	75A	40A
122G	77G	39G
122C	77C	39C
123G	77G	40G
121T	76T	37T
120T	77T	36T
124T	76T	36T
128T	76T	37T
128C	77C	36C
128C	74C	36C
74A_56G	40A_31G	19A_16G
133T	73T	35T
133A	74A	34A
137G	74G	37G
136A	74A	36A
134C	75C	37C
133A	77A	37A
133G	74G	37G
134T	75T	17T_20G
133T	76T	37T
130T	76T	36T
130T	75T	35T
129C	74C	35C
129G	75G	36G
131T	75T	36T
129G	74G	36G
118A	23A_38G	14A_19G
117T	62T	33T

118C	63C	33C
119A	63A	34A
116C	37T_26C	32C
117G	63G	32G
113T	62T	30T
116G	63G	32G
115G	37A_26G	31G
116C	62C	32C
118C	63C	32C
117A	65A	33A
117G	65G	36G
113T	29T_36C	15T_21C
115G	64G	37G
116C	66C	37C
116G	65G	38G
110A	65A	37A
110C	66C	39C
109T	66T	38T
110A	70A	40A
107G	73G	39G
103A	73A	40A
103C	74C	43C
102A	75A	43A
103C	77C	43C
103G	77G	45G
102G	79G	47G
103A	78A	48A
104A	79A	50A
105C	79C	51C
104G	77G	52G
105T	78T	51T
104C	78C	51C
105G	76G	52G
107T	73T	52T
106T	76T	51T
108A	78A	51A
109C	76C	50C
106C	76C	51C
99T	77T	51T
101T	76T	53T
105T	77T	52T
104C	79C	53C
102C	78C	53C

103C	79C	55C
104A	80A	55A
104C	81C	53C
104C	83C	20T_33C
103G	82G	50G
100A	82A	51A
97G	82G	51G
96G	83G	51G
95T	84T	52T
94G	85G	49G
93G	85G	50G
92T	86T	51T
88C	85C	51C
89C	84C	52C
89C	84C	53C
92T	86T	16A_77T_8G_16C
94A	88A	46A
90T	87T	46T
96T	87T	45T
99T	89T	45T
99A	91A	38A
100C	93C	40C
99C	92C	40C
99G	91G	40G
100T	91T	50T
102A	92A	50A
101T	92T	49T
101T	90T	49T
99T	88T	49T
100T	90T	50T
101T	91T	49T
101C	92C	40C
97G	52G_43C	50G
96C	96C	51C
97T	95T	49T
95C	94C	51C
95C	95C	52C
94A	95A	51A
95T	98T	51T
95A	100A	51A
94A	101A	50A
93G	100G	49G
93A	101A	48A

67T_24C	57T_45C	46C
79G	104G	51G
91C	105C	50C
89A	98A	51A
90C	98C	52C
90C	100C	51C
74T	102T	51T
74C	101C	50C
80T	100T	50T
80C	98C	49C
82C	95C	48C
84A	95A	48A
86T	98T	49T
85A	98A	47A
84A	99A	46A
85G	98G	44G
83A	98A	44A
83C	98C	44C
85G	95G	12A_30G
83C	94C	43C
80A	91A	44A
79C	91C	44C
79C	90C	43C
83A	91A	43A
82A	91A	43A
83A	92A	44A
85T	92T	45T
82T	91T	43T
84T	93T	43T
84T	93T	42T
83T	90T	42T
84A	94A	42A
65A_18G	95A	41G
88G	34T_61G	44G
90A	94A	44A
90G	94G	45G
89A	93A	45A
90A	93A	44A
22A_67G	30A_63G	18A_18G_1C
97A	95A	38A
100A	96A	39A
100A	95A	40A
102A	96A	40A

100C	91C	42C
102A	89A	42A
101G	91G	45G
103G	92G	45G
103A	93A	47A
105A	91A	45A
104A	89A	43A
104A	90A	45A
117A	92A	49A
120A	92A	52A
126T	92T	52T
126A	93A	53A
131A	95A	52A
123T	95T	51T
133C	94C	51C
136T	94T	50T
138G	92G	50G
138T	89T	49T
138T	91T	47T
139T	95T	48T
140T	94T	49T
140C	94C	49C
143T	91T	49T
144T	88T	46T
143C	90C	13T_32C
89A_55G	56A_33G	11A_33G
149C	92C	47C
151T	90T	46T
152C	89C	49C
153C	87C	50C
143A	88A	43A
155T	88T	49T
155A	86A	48A
157A	83A	51A
157G	84G	49G
158A	85A	49A
158C	84C	47C
163G	85G	48G
164C	88C	49C
162A	86A	48A
162C	84C	49C
164A	84A	50A
165G	85G	50G

166A	87A	49A
168C	91C	56C
170T	94T	47T
170T	95T	50T
174T	100T	52T
173C	99C	52C
176C	99C	53C
173A	98A	53A
170C	99C	53C
170C	98C	54C
167T	98T	53T
169C	97C	53C
169C	96C	52C
168C	95C	52C
166T	94T	51T
165G	89G	51G
163T	88T	50T
161T	87T	51T
158T	87T	49T
157T	86T	49T
156G	88G	51G
149T_45G	89T	52T
150G	87G	51G
150G	86G	50G
145G	39A_46G	49G
135A	85A	33G
137A	85A	33G
139A	87A	27A_12G
138A	88A	42A
137A	87A	43A
120A	89A	45A
120G	87G	48G
115T	86T	48T
119G	88G	48G
46T_63C	44T_44C	29A_1T_20C
108G	86G	51G
105T	86T	49T
109C	87C	50C
110T	86T	50T
110T	85T	51T
108A	85A	52A
107T	83T	52T
106A	84A	53A

108G	85G	54G
107T	88T	52T
108G	87G	56G
108C	85C	57C
108G	85G	55G
109A	84A	56A
108A	85A	55A
108A	86A	55A
108A	85A	54A
110A	86A	55A
106T	86T	55T
110C	85C	54C
112T	86T	55T
112A	87A	54A
113C	90C	53C
112T	89T	53T
114C	90C	59C
115G	89G	56G
116C	91C	57C
115A	90A	57A
116T	90T	57T
113T	88T	55T
111T	86T	53T
112T	86T	52T
112A	86A	51A
112A	85A	50A
116C	84C	51C
114A	85A	51A
112T	82T	51T
116G	81G	53G
113C	81C	51C
115T	81T	51T
115T	80T	51T
113T	82T	48T
113C	83C	51C
112T	81T	49T
114A	80A	51A
115A	81A	50A
114C	80C	51C
113T	82T	16A_83T_48G_16C
116G	81G	35G
117C	82C	30C
117T	82T	31T

117A	82A	30A
120G	82G	29G
121G	83G	30G
122T	83T	28T
122T	83T	48T
123G	81G	47G
124G	81G	47G
124C	81C	46C
123A	81A	47A
126G	82G	47G
128G	81G	48G
126A	79A	48A
127G	78G	50G
119G	79G	50G
126T	77T	48T
127G	76G	47G
130G	77G	45G
128G	76G	23A_23G
129A	77A	46A
130C	77C	46C
132A	77A	46A
130A	79A	45A
129G	36A_41G_1C	46G
129C	77C	44C
130G	75G	45G
131A	75A	44A
131T	75T	44T
128G	74G	43G
130G	71G	43G
129G	74G	42G
129A	72A	42A
129G	73G	44G
130C	72C	43C
128T	72T	44T
130G	71G	19A_24G
129A	71A	45A
132A	70A	46A
131T	71T	43T
136C	73C	43C
133T	75T	44T
131G	76G	44G
128T	73T	44T
127C	72C	44C

126A	73A	41A
128C	77C	44C
115G	74G	17A_27G
136T	82T	49T
137G	85G	49G
137G	88G	50G
135A	91A	49A
137T	92T	52T
140T	91T	49T
138C	94C	51C
137G	95G	51G
134A	93A	50A
133T	95T	52T
134C	94C	50C
131T	93T	52T
131T	90T	51T
133A	90A	52A
124C	86C	52C
133A	86A	49A
134A	87A	49A
122T	78T	41T
136G	91G	48G
138G	91G	49G
136C	91C	49C
136T	91T	49T
137G	91G	49G
136G	91G	49G
138T	93T	47T
137C	94C	49C
135T	92T	48T
136T	90T	48T
136C	90C	48C
131C	92C	50C
131G	93G	50G
131A	93A	49A
127T	91T	50T
130C	92C	51C
125T	93T	50T
128T	93T	51T
126G	94G	50G
127C	94C	50C
126A	94A	52A
130G	94G	53G

128C	95C	53C
129C	97C	56C
129C	97C	56C
128A	98A	57A
129G	100G	57G
129A	99A	55A
129G	98G	55G
130G	99G	56G
117C	91C	54C
126T	100T	55T
127T	99T	52T
121C	99C	54C
122T	99T	54T
121G	96G	56G
120C	44T_51C	56C
117G	94G	52G
114A	93A	53A
116T	95T	55T
114T	94T	57T
116T	94T	55T
114A	96A	54A
113A	94A	54A
112C	95C	54C
111C	93C	52C
113C	92C	52C
116G	91G	53G
116C	92C	53C
114A	90A	53A
113G	88G	51G
114C	88C	19T_32C
114G	88G	52G
116C	85C	51C
113C	82C	50C
114A	79A	50A
112C	77C	49C
109C	78C	48C
109T	74T	47T
50T_50G_158C	32T_32G_109C	14T_14G_61C
56G	43G	33G
57T	41T	32T
58C	41C	33C
114C	76C	53C
107G	74G	49G

108T	74T	49T
110C	79C	51C
110C	81C	51C
111C	80C	52C
112C	79C	56C
113C	80C	22T_33C
113T	82T	55T
116G	82G	55G
116G	85G	55G
119A	86A	55A
118A	87A	55A
120A	91A	55A
122G	91G	56G
124T	91T	56T
122T	92T	55T
123G	92G	55G
122A	94A	52A
122A	98A	53A
122C	98C	53C
124T	98T	52T
122A	95A	51A
123C	96C	53C
122C	96C	55C
121T	97T	54T
120C	99C	54C
119T	99T	54T
121C	102C	51C
119T	102T	50T
120T	101T	48T
122A	102A	47A
117A	103A	48A
117G	99G	49G
118G	95G	46G
116C	93C	46C
116A	92A	46A
121C	95C	45C
120T	94T	45T
118G	96G	46G
118G	96G	46G
116C	95C	45C
114A	95A	44A
114G	97G	46G
116G	95G	47G

114G	95G	46G
116A	94A	42A
116T	96T	21T_20C
117A	95A	42A
114G	93G	43G
116T	90T	43T
115A	92A	43A
112A	92A	42A
115G	91G	43G
111A	91A	43A
112G	91G	41G
108C	90C	43C
110T	44A_45T	43T
111A	89A	46A
112A	91A	45A
113A	92A	45A
113T	93T	45T
111T	95T	45T
112C	96C	43C
109A	94A	46A
110T	94T	46T
111C	91C	46C
112C	93C	47C
107T	95T	47T
105T	93T	44T
103G	95G	44G
103G	96G	44G
100G	98G	43G
106G	97G	43G
107A	99A	42A
109A	102A	42A
114T	102T	38T
115G	100G	39G
117T	97T	38T
116T	98T	38T
120G	97G	38G
119A	93A	36A
120A	94A	42A
119C	90C	42C
121C	89C	41C
119T	90T	42T
123C	90C	42C
124C	90C	43C

123A	87A	44A
122G	84G	44G
123C	85C	45C
122A	84A	47A
123T	88T	47T
123G	89G	46G
123G	89G	45G
126G	87G	44G
126G	86G	45G
125T	84T	44T
124G	83G	47G
126C	82C	48C
125A	83A	50A
126G	82G	50G
126C	81C	51C
126T	81T	50T
124A	82A	51A
124A	82A	52A
67T_56C	44T_40C	51C
55A_63G_1C	81G	53G
124G	82G	51G
125T	83T	51T
127C	83C	25A_1T_26C
130C	86C	54C
131C	87C	54C
132T	84T	51T
132C	86C	51C
132T	87T	52T
130G	87G	51G
130G	87G	53G
130T	87T	52T
132G	88G	49G
131C	88C	51C
133C	87C	51C
134A	87A	51A
132C	86C	51C
134T	86T	50T
130T	88T	51T
133C	91C	51C
130A	93A	52A
128C	94C	48C
62A_67G	55A_37G	26A_24G
128C	92C	49C

131T	92T	49T
133G	91G	50G
133C	90C	52C
133A	90A	51A
135G	93G	52G
135T	94T	50T
137T	94T	51T
136T	91T	50T
137A	96A	51A
137C	96C	52C
140A	96A	53A
141G	93G	53G
133C	90C	52C
131A	88A	55A
129T	86T	57T
122C	87C	58C
121T	87T	57T
121G	90G	57G
121C	93C	57C
115C	92C	58C
114T	93T	56T
111T	93T	53T
111T	93T	52T
108T	96T	52T
107T	94T	52T
112A	94A	53A
112A	95A	53A
111A	98A	55A
111C	100C	53C
112T	100T	53T
112G	102G	51G
53T_59C	98T	52T
111A	97A	52A
114T	98T	53T
113G	100G	52G
114A	101A	57A
115G	102G	58G
114C	102C	55C
113T	105T	55T
114T	107T	54T
112T	106T	53T
114G	107G	51G
116C	113C	53C

116A	114A	52A
120G	118G	52G
112C	118C	51C
112A	118A	51A
112G	117G	52G
112A	116A	52A
113G	115G	51G
111G	112G	23A_28G
111C	112C	29A_24C
115C	111C	53C
116T	111T	54T
119C	58T_52C	54C
119G	111G	55G
118C	109C	55C
121C	112C	55C
119T	113T	57T
121G	117G	57G
119T	117T	56T
117T	117T	57T
117A	117A	57A
115T	118T	56T
116C	116C	55C
118C	114C	53C
123C	113C	51C
69T_55C	111T	24T_28C
121G	111G	51G
121G	111G	53G
118A	110A	51A
118T	112T	52T
117C	112C	51C
116A	111A	50A
112A	110A	49A
112G	110G	50G
110C	109C	50C
45T_64C	110C	51C
108C	104C	51C
109C	102C	51C
62A_45G	102A	50A
107G	102G	51G
114G	102G	51G
115T	104T	48T
114G	103G	47G
116C	102C	47C

117A	101A	47A
116C	47T_53C	47C
49A_67G	98G	50G
116G	100G	50G
115A	100A	51A
117T	99T	51T
116A	99A	51A
114T	98T	52T
116T	99T	52T
109G	98G	51G
46A_63G	98G	54G
110G	96G	52G
113A	95A	52A
112T	95T	54T
112G	93G	53G
112G	93G	54G
114G	92G	54G
109A	45A_46G	55A
111T	89T	53T
112A	90A	52A
110A	89A	50A
109A	91A	51A
112A	90A	50A
111A	88A	50A
113C	89C	51C
112A	89A	52A
112G	84G	49G
114C	83C	50C
113A	79A	50A
117C	80C	50C
116C	80C	50C
118C	81C	50C
119A	81A	51A
120G	81G	51G
122T	81T	51T
121C	81C	48C
116C	82C	48C
114T	81T	46T
109T	83T	47T
112C	83C	46C
112A	83A	46A
110A	81A	47A
114A	80A	46A

110A	78A	47A
110G	76G	47G
112A	77A	46A
110C	79C	45C
111C	80C	45C
109A	79A	46A
106T	78T	46T
101C	79C	46C
102C	82C	46C
105A	84A	47A
105G	81G	44G
107C	82C	44C
110T	80T	44T
110T	79T	44T
111T	80T	45T
111G	80G	45G
108G	79G	44G
112G	79G	44G
111T	81T	43T
110T	79T	41T
111C	83C	43C
111A	82A	42A
110A	84A	43A
105T	84T	42T
104G	82G	43G
105G	83G	44G
104G	87G	43G
101A	87A	43A
100C	86C	42C
102A	83A	40A
102G	81G	39G
103A	80A	40A
101A	81A	41A
104G	82G	43G
109G	81G	43G
109C	82C	43C
110A	81A	42A
110G	81G	39G
110G	80G	38G
106C	79C	39C
105C	81C	39C
102A	82A	38A
104A	83A	40A

33T_69C	83C	16T_23C
103G	84G	39G
104C	83C	41C
101C	83C	41C
67A_33G	86A	24A_17G
104T	83T	41T
100C	82C	39C
98A	82A	40A
99A	82A	39A
99A	81A	40A
97C	80C	40C
95A	83A	40A
92G	85G	41G
89G	83G	42G
59A_33G	86A	24A_16G_1C
94T	83T	40T
91C	82C	39C
89T	83T	39T
86G	84G	38G
86T	84T	37T
84G	86G	38G
85G	85G	38G
84C	86C	38C
83T	86T	37T
82G	88G	37G
81G	89G	36G
77G	87G	35G
77C	86C	34C
76A	87A	34A
74T	87T	34T
73T	87T	34T
159A_116C	23A_86C	66A_52C
43A	65A	14A
42C	87C	14C
42A	86A	15A
40C	84C	4A_15C
45A	82A	19A
43C	82C	15A_19C
43A	83A	38A
30A_40C	83C	38C
78A	83A	37A
78C	82C	36C
79A	81A	36A

79C	83C	36C
76A	81A	33A
79C	82C	35C
79A	82A	35A
72C	82C	33C
58A	82A	29A
58C	80C	28C
59A	81A	29A
59C	83C	31C
43A_17C	84A	23A_9C
60C	80C	32C
62A	80A	31A
64T	80T	34T
21T_46C	81C	34C
70G	81G	34G
71C	80C	34C
71C	83C	34C
72A	82A	34A
72T	80T	35T
74A	80A	35A
76A	81A	35A
26T_52C	79C	34C
78A	78A	33A
78T	79T	35T
81T	77T	34T
82T	76T	35T
83G	74G	34G
81G	77G	36G
80T	76T	36T
80G	78G	33G
79T	78T	34T
28A_52T	76T	34T
80G	79G	31G
81A	78A	29A
81T_37C	77T	29T_22C
84C	76C	30C
84C	76C	30C
49T_33C	76C	30C
86C	76C	30C
88C	74C	31C
90C	72C	32C
89C	73C	32C
89C	30A_70C	30C

85A	73A	28A
86A	73A	29A
86A	74A	28A
88C	70C	28C
95T	70T	28T
95T	74T	28T
93G	75G	28G
92C	72C	29C
92C	70C	29C
94A	73A	31A
94G	73G	31G
92C	73C	31C
88T	73T	31T
93T	76T	31T
96G	74G	32G
96G	74G	32G
96G	73G	34G
94A	74A	35A
96A	75A	33A
98G	75G	33G
99G	79G	32G
98G	78G	32G
99A	78A	32A
95G	78G	33G
95T	81T	33T
95T	81T	33T
94C	79C	32C
93T	80T	33T
93C	80C	34C
94A	79A	34A
95T	78T	34T
93T	77T	36T
93T	78T	34T
94C	79C	33C
93A	78A	33A
92G	76G	32G
94G	76G	32G
94C	75C	33C
91A	75A	32A
91A	75A	33A
92G	74G	33G
94T	77T	35T
95T	76T	35T

96G	75G	35G
94T	77T	35T
95G	75G	36G
94A	75A	36A
95C	77C	37C
94A	77A	37A
93G	77G	37G
92A	77A	38A
92A	79A	37A
92T	80T	37T
88C	81C	36C
86C	80C	37C
88C	79C	37C
93C	78C	38C
96C	76C	37C
97T	75T	37T
99T	75T	37T
99T	77T	35T
99C	78C	37C
100T	78T	36T
100C	78C	39C
103A	78A	39A
100G	77G	41G
95A	76A	41A
101G	74G	41G
99T	72T	40T
98A	73A	40A
93A	72A	40A
91C	72C	39C
92A	71A	39A
91T	71T	39T
90T	71T	40T
87T	65T	40T
88C	66C	40C
87T	66T	41T
90G	66G	42G
89C	62C	42C
90A	62A	42A
91A	63A	43A
89A	66A	43A
86T	62T	45T
83C	61C	45C
83T	64T	44T

85T	63T	43T
86A	61A	41A
86T	61T	41T
84A	60A	41A
82T	56T	41T
82T	57T	39T
80T	57T	40T
82A	57A	40A
81T	54T	40T
83A	56A	41A
84G	55G	40G
86A	58A	41A
86A	57A	41A
88T	57T	41T
86A	58A	40A
87G	33A_25G	38G
93G	57G	39G
94G	57G	38G
94G	58G	40G
96A	56A	42A
94G	56G	42G
95G	57G	41G
97A	58A	41A
98A	57A	41A
98A	57A	41A
98T	54T	39T
97G	54G	40G
97T	53T	41T
98T	52T	42T
102A	52A	43A
104C	51C	45C
105C	52C	44C
104C	33T_20C	45C
105T	55T	45T
107C	56C	46C
105C	53C	46C
104C	52C	46C
105T	51T	44T
103C	28T_22C	45C
104C	50C	45C
99C	50C	43C
97A	49A	42A
96T	48T	41T

93T	47T	42T
92C	45C	40C
91G	44G	41G
91G	45G	41G
47A_46G	55A	17A_1T_23G
46A_45G	44A	40A
98A	44A	38A
97A	44A	39A
96A	44A	38A
96A	43A	38A
97A	123A	38A
97A	32A	38A
97A	30A	37A
94A	30A	38A
97C	30C	39C
95A	29A	38A
96A	34A	38A
96A	36A	40A
97A	37A	39A
98A	37A	38A
96A	37A	38A
91C	10A_1T_28C	38C
96A	48A	38A
96A	46A	38A
99A	47A	36A
99A	50A	36A
100C	50C	36C
100A	50A	36A
102C	51C	37C
102A	51A	37A
102A	51A	37A
102G	50G	38G
104C	51C	38C
104T	52T	39T
103A	52A	38A
102G	53G	39G
104G	53G	39G
104A	56A	39A
105G	56G	39G
102T	55T	40T
102T	57T	40T
99A	59A	41A
99C	60C	41C

97A	60A	42A
95T	61T	41T
97C	62C	41C
99G	62G	40G
98C	64C	41C
98T	63T	41T
98A	63A	39A
94A	62A	39A
93A	63A	39A
92T	63T	40T
89G	61G	41G
90T	59T	41T
89C	59C	39C
88A	61A	38A
89T	62T	36T
83A	62A	11A_26C
83T	63T	37T
83C	62C	38C
83T	61T	37T
84C	63C	39C
87T	65T	39T
89C	67C	39C
86T	68T	39T
86C	70C	40C
84T	72T	40T
84T	73T	39T
82C	74C	42C
83C	76C	41C
82A	76A	40A
84C	75C	42C
44A_40G	77G	43G
79C	76C	43C
79G	77G	43G
81G	78G	43G
83G	78G	44G
83C	80C	45C
84T	79T	45T
85T	83T	45T
87G	82G	46G
85A	80A	46A
84C	81C	46C
84A	80A	46A
84A	78A	48A

85T	79T	49T
87G	77G	48G
87A	75A	47A
87G	79G	49G
87G	81G	47G
86G	81G	48G
85C	79C	48C
85A	80A	48A
82G	81G	47G
83T	80T	48T
82T	78T	45T
82C	81C	47C
84A	80A	46A
83G	80G	47G
82C	82C	48C
82G	83G	48G
81C	84C	47C
80G	85G	46G
80A	87A	46A
79T	86T	46T
78G	86G	45G
78A	83A	43A
77A	83A	43A
76C	83C	44C
73T	81T	44T
79C	80C	45C
79A	80A	44A
80C	82C	43C
80C	83C	44C
78T	85T	45T
77G	84G	44G
77G	83G	41G
81T	82T	42T
79T	82T	43T
79T	80T	44T
80G	80G	45G
80G	79G	44G
80C	81C	44C
81A	81A	43A
82G	81G	44G
82A	82A	43A
80A	80A	42A
81G	79G	42G

82A	76A	43A
83A	77A	42A
82A	80A	40A
82C	78C	39C
82C	78C	42C
81C	77C	40C
81T	76T	43T
82A	75A	42A
81T	77T	42T
83T	75T	42T
83C	75C	42C
83A	77A	42A
85T	78T	43T
85G	78G	42G
87C	76C	42C
87T	77T	41T
86T	77T	42T
86T	76T	41T
86A	77A	40A
86A	78A	39A
86T	80T	40T
85T	76T	42T
83G	77G	41G
87A	80A	42A
87A	81A	42A
84T	83T	42T
84A	83A	41A
83T	82T	40T
82T	84T	40T
80T	84T	41T
81G	81G	38G
81A	78A	40A
81A	80A	40A
80T	81T	39T
81A	81A	41A
82A	79A	40A
81T	77T	43T
81C	79C	43C
79T	77T	44T
79T	77T	45T
80T	78T	43T
81T	78T	45T
84C	79C	44C

82A	79A	46A
83T	79T	47T
85T	78T	48T
85C	80C	50C
85C	80C	49C
84T	81T	51T
85G	79G	51G
87C	80C	51C
89G	80G	50G
87A	79A	50A
87A	79A	52A
87T	77T	54T
89G	75G	53G
89A	74A	52A
89A	75A	54A
88T	75T	54T
84G	76G	56G
86T	76T	56T
88T	78T	58T
89G	79G	57G
89T	79T	57T
92C	79C	55C
91C	79C	55C
91A	82A	55A
91A	81A	56A
90A	82A	56A
91T	84T	57T
90A	85A	55A
88A	85A	53A
87C	83C	55C
86A	83A	53A
82A	80A	55A
81C	80C	56C
84A	79A	56A
83T	78T	58T
85C	74C	57C
83G	74G	58G
84A	76A	56A
85C	74C	57C
83T	73T	57T
83C	73C	58C
84G	72G	57G
86C	71C	58C

84T	71T	59T
84C	71C	59C
84G	71G	59G
84C	71C	60C
86C	69C	59C
85G	68G	57G
85C	68C	58C
82A	67A	56A
81T	65T	58T
55A_83T_53G	67T	57T
102G	67G	58G
101A	66A	54A
101G	67G	56G
102A	67A	54A
102G	71G	59G
102A	71A	60A
104G	68G	60G
104A	67A	59A
106G	67G	56G
106A	69A	58A
108G	69G	56G
110A	66A	57A
110G	66G	57G
108A	68A	56A
108G	68G	57G
108A	66A	55A
106G	65G	55G
110A	65A	53A
111G	63G	52G
109A	61A	51A
100C	60C	50C
101C	64C	53C
101C	66C	54C
100A	68A	53A
97A	67A	52A
97G	68G	50G
98C	68C	49C
98A	67A	48A
97C	67C	48C
98G	67G	48G
96G	64G	47G
94C	63C	46C
91T	65T	45T

93G	66G	44G
94C	66C	43C
94G	66G	41G
93A	65A	41A
91A	65A	43A
90G	64G	42G
91A	65A	43A
90C	63C	45C
92T	61T	44T
93C	59C	45C
91C	59C	46C
94C	59C	46C
94T	60T	47T
98C	60C	45C
99C	61C	44C
99G	62G	42G
102A	62A	41A
95T	62T	40T
95G	63G	41G
94A	66A	41A
94C	67C	40C
94A	67A	41A
91A	68A	41A
90A	67A	40A
85A	70A	38A
88G	71G	37G
88C	71C	40C
87G	70G	39G
89C	71C	39C
87C	70C	40C
84G	69G	40G
82G	26A_42G	19A_20G
84G	69G	39G
87A	69A	39A
86G	71G	39G
86A	70A	38A
84A	66A	38A
80G	67G	38G
81G	66G	37G
81A	70A	35A
83G	70G	35G
84A	67A	34A
85G	66G	32G

83G	67G	32G
83A	67A	32A
80C	67C	32C
80A	66A	31A
80G	66G	32G
81C	66C	30C
82C	68C	30C
80A	68A	30A
84G	69G	29G
83A	70A	28A
85G	70G	31G
84G	68G	32G
80T	68T	34T
85G	70G	35G
85G	69G	35G
84G	66G	33G
85A	64A	32A
86G	63G	33G
90G	63G	34G
90G	65G	35G
89G	69G	34G
88G	71G	35G
87A	72A	34A
87C	71C	36C
87C	73C	37C
88G	76G	40G
89T	76T	42T
84T	74T	41T
91T	76T	42T
91T	77T	42T
94T	78T	44T
96C	78C	42C
97T	78T	42T
97A	77A	41A
100A	76A	40A
98G	79G	43G
101C	84C	44C
103C	85C	42C
103C	84C	44C
103C	83C	46C
101T	82T	46T
103C	80C	46C
102A	79A	50A

99A	81A	49A
100G	81G	49G
101T	82T	48T
102G	80G	48G
102T	78T	48T
104T	78T	47T
105C	77C	47C
108T	79T	47T
104T	76T	48T
105T	78T	50T
107G	77G	49G
111C	79C	51C
112T	78T	52T
115T	78T	51T
115T	78T	51T
115C	79C	50C
114A	80A	48A
110G	81G	49G
110C	80C	50C
108G	82G	50G
106C	83C	50C
107C	83C	51C
107G	83G	51G
108C	81C	54C
103T	81T	54T
106T	81T	53T
105T	84T	56T
105T	84T	55T
106G	84G	55G
105T	84T	56T
106T	85T	58T
107T	86T	60T
109G	90G	60G
108C	87C	60C
110C	86C	61C
108G	84G	60G
107G	81G	62G
106A	80A	60A
104G	83G	59G
104G	83G	57G
104C	83C	56C
104T	83T	56T
103T	83T	55T

103C	83C	55C
104T	84T	54T
101G	85G	53G
103A	82A	55A
103T	78T	54T
103A	77A	54A
103A	75A	52A
103A	77A	52A
105T	75T	51T
106T	74T	48T
105G	73G	46G
103C	73C	48C
102A	71A	48A
99A	70A	44A
99C	69C	45C
99C	71C	47C
100G	71G	47G
98C	72C	47C
96T	71T	47T
96T	71T	45T
96A	68A	45A
95A	66A	45A
94G	67G	44G
92G	67G	41G
91G	69G	39G
89A	69A	39A
90A	73A	39A
90C	71C	40C
92G	72G	40G
91C	69C	38C
89G	68G	38G
89A	66A	39A
89G	71G	40G
87G	69G	39G
86C	67C	39C
87G	68G	38G
87C	67C	36C
88A	69A	38A
88T	68T	35T
87A	68A	34A
85A	70A	34A
86A	69A	35A
84A	67A	35A

84G	66G	36G
84C	65C	36C
83C	64C	36C
83G	62G	36G
84C	61C	36C
83T	58T	35T
82C	59C	34C
82T	58T	32T
81T	59T	31T
81G	58G	31G
81C	55C	30C
80T	54T_24C	30T
80C	56C	30C
83C	57C	30C
82C	56C	28C
82C	56C	25C
78C	52C	25C
78C	54C	24C
77T	54T	23T
77C	54C	22C
79C	55C	23C
77C	54C	22C
78C	56C	23C
79T	55T	24T
79C	57C	24C
78G	59G	26G
78G	57G	27G
79A	59A	29A
80G	60G	29G
80T	59T	30T
80G	61G	27G
82G	61G	27G
82T	59T	26T
82T	58T	27T
82G	58G	28G
82T	56T	28T
83G	56G	28G
83T	56T	28T
83G	56G	26G
81A	56A	27A
80A	55A	27A
81T	53T	26T
79G	54G	25G

77G	53G	25G
78A	54A	25A
76A	52A	25A
78G	52G	26G
74T	49T	25T
74C	51C	25C
74A	51A	25A
76G	50G	26G
79G	49G	26G
77C	47C	25C
78G	45G	24G
78G	46G	23G
78G	45G	24G
79C	45C	24C
81G	45G	24G
80C	44C	23C
80A	44A	22A
79C	44C	23C
79G	44G	24G
78G	41G	24G
78G	39G	26G
75A	38A	24A
70G	40G	24G
69G	40G	24G
72C	40C	23C
71A	41A	23A
75G	42G	24G
75G	42G	23G
80A	41A	23A
82G	43G	23G
83G	44G	24G
82G	44G	25G
81G	45G	27G
81G	46G	28G
83T	45T	27T
84C	45C	28C
83A	44A	29A
84A	45A	27A
83T	45T	28T
84C	45C	28C
85C	44C	27C
89A	45A	28A
89T	47T	28T

88T	48T	28T
88A	46A	28A
90A	48A	28A
91G	49G	28G
91G	49G	29G
92T	50T	30T
91T	49T	29T
92C	50C	28C
91T	48T	29T
93A	46A	27A
95T	47T	26T
96T	45T	24T
96A	45A	24A
98A	46A	23A
98G	44G	24G
96T	47T	26T
98C	47C	26C
99C	48C	25C
99T	47T	24T
98C	47C	24C
99C	49C	24C
101T	51T	24T
101A	52A	25A
101G	52G	27G
104A	55A	27A
104G	55G	28G
104A	53A	27A
104A	54A	28A
104C	55C	28C
106A	55A	28A
107A	56A	28A
105A	57A	29A
110G	56G	29G
109C	61C	28C
106T	60T	28T
104A	61A	29A
104A	61A	29A
105C	61C	28C
104C	60C	29C
106T	60T	27T
105G	61G	28G
103T	60T	28T
104G	61G	28G

103C	60C	27C
103C	61C	27C
104A	61A	26A
103T	61T	27T
102C	61C	25C
102C	61C	25C
103T	59T	25T
106C	58C	26C
104A	58A	26A
104T	58T	26T
100A	60A	25A
100A	61A	26A
96A	62A	27A
94T	61T	29T
94A	62A	28A
94T	63T	27T
94T	63T	25T
95G	63G	23G
94C	63C	24C
95T	63T	24T
94G	62G	24G
94C	61C	24C
95A	62A	26A
91T	2A_36T_25C	27T
90G	64G	28G
86G	61G	27G
84A	59A	27A
84G	59G	28G
84G	61G	28G
82A	59A	28A
80G	58G	28G
79C	58C	27C
78C	56C	25C
79C	55C	26C
77C	55C	26C
74G	56G	26G
73A	56A	26A
71A	55A	24A
69T	54T	25T
67T	55T	25T
65T	54T	25T
63T	54T	25T
63A	52A	23A

60A	52A	23A
60T	51T	23T
58T	53T	23T
58A	53A	24A
57A	52A	24A
53A	51A	25A
54C	50C	24C
55C	49C	21C
51T	48T	21T
49C	50C	20C
49T	50T	20T
49T	50T	20T
49T	48T	20T
47A	48A	21A
45C	47C	20C
45C	46C	18C
42T	45T	18T
43G	41G	18G
44C	41C	18C
45T	40T	16T
44C	40C	17C
43A	41A	18A
43G	41G	17G
41G	41G	19G
41A	40A	20A
44G	41G	20G
43A	41A	21A
41T	40T	21T
42A	40A	21A
41G	39G	21G
41A	40A	20A
40G	41G	19G
41G	42G	20G
40G	42G	21G
37G	41G	21G
35G	41G	21G
34G	41G	21G
35A	38A	21A
35G	37G	21G
35G	36G	20G
36A	34A	18A
35G	33G	18G
35A	32A	18A

34G	32G	18G
32A	30A	18A
31A	31A	18A
29G	31G	18G
29A	31A	17A
49A_67G	32A	31A_43G
33G	31G	19G
33G	31G	18G
31A	30A	17A
30G	29G	17G
29G	30G	15G
31G	29G	16G
31A	27A	16A
32G	26G	18G
31G	28G	18G
31G	28G	19G
31A	28A	18A
31G	28G	20G
31G	27G	18G
31G	27G	19G
32A_1T_7G	27A	19A
35G	28G	19G
38G	28G	19G
37G	28G	19G
40A	31A	19A
41G	33G	18G
36A	33A	15A
39G	35G	15G
39A	35A	15A
42A	35A	16A
42A	36A	16A
42T	37T	17T
42C	38C	18C
42T	40T	19T
42A	40A	22A
46T	43T	22T
46T	41T	22T
47T	41T	22T
48A	42A	23A
48G	42G	25G
49A	42A	24A
50C	42C	24C
49T	43T	23T

48T	43T	22T
47G	20G_23C	22G
49A	44A	21A
50A	44A	22A
50C	44C	23C
51G	44G	24G
52T	44T	24T
52C	46C	24C
54G	24A_23G	25G
57G	46G	24G
58T	47T	24T
62C	49C	24C
62A	49A	25A
63G	50G	24G
63C	50C	24C
65T	51T	24T
65T	54T	25T
65C	56C	25C
66T	55T	26T
68T	55T	27T
69G	54G	26G
69T	57T	26T
68G	57G	26G
67C	57C	27C
70T	59T	24T
72C	61C	25C
72A	61A	25A
73C	61C	25C
72T	61T	23T
72C	60C	26C
72A	58A	26A
71C	59C	25C
71A	59A	24A
71C	59C	27C
72A	59A	27A
73C	60C	27C
74A	60A	28A
75C	60C	28C
74A	61A	29A
74C	61C	30C
73A	61A	28A
73C	59C	28C
73A	58A	30A

72C	59C	30C
74A	59A	29A
32A_41C	59C	8A_1T_21C
75A	58A	30A
67C	56C	29C
71A	56A	30A
28A_42C	59C	8A_22C
69A	59A	31A
67C	14A_54C	31C
65A	56A	31A
67C	45C	31C
42A_25G	56A	31A
66C	54C	31C
63A	53A	30A
63C	51C	32C
63A	50A	31A
65C	49C	32C
56A	47A	27A
61C	47C	23C
15A_40G_15C	18A_26G	20A
58C	44C	22C
30A	44A	22A
30C	43C	22C
16A_14G	45A	21A
10A_33T_12G_26C	43C	1A_14T_14G_22C
48G	8A_28G	23G
48T	27T_7C	23T
49G	7A_27G	24G
50T	42T	23T
50G	41G	24G
49T	42T	24T
49G	43G	26G_13C
46C	43C	12C
47A	43A	27A
27T_20G	42T	28T
49G	42G	30G
51T	42T	29T
51G	44G	31G
48T	43T	31T
49G	43G	32G
49T	42T	32T
49G	11A_10T_48G_10C	31G
48T	37C	27T

51G	39G	29G
51C	41C	15T_14C
50G	14A_27G	31G
50C	44C	34C
52G	45G	34G
52C	45C	34C
52A	45A	34A
51C	46C	35C
52G	46G	36G
53C	46C	35C
51A	47A	35A
51A	48A	34A
54C	50C	35C
54A	51A	35A
60G	53G	34G
61C	56C	34C
60A	57A	36A
61A	57A	35A
63T	56T	36T
65G	56G	37G
68T	58T	37T
69A	59A	38A
68C	58C	37C
68T	58T	40T
66G	59G	39G
69G	60G	37G
69G	61G	39G
71A	61A	42A
76T	61T	40T
74T	62T	38T
74G	62G	39G
76T	62T	39T
77G	62G	39G
77C	59C	39C
33A_1G_42C	58C	40A
74A	58A	39A
82C	56C	43C
84A	56A	42A
85G	54G	44G
86C	54C	43C
88C	54C	44C
89A	55A	45A
90G	55G	45G

91G	54G	46G
95A	54A	46A
95A	54A	47A
91A	53A	48A
91G	53G	47G
93A	55A	45A
94G	31A_1T_27G	46G
92G	59G	44G
94T	59T	46T
94G	58G	45G
94G	58G	44G
94T	57T	42T
94A	59A	46A
95T	59T	46T
96G	27A_33G	46G
97G	61G	44G
98G	62G	44G
97G	65G	43G
98A	65A	44A
97G	66G	43G
95A	66A	45A
94G	64G	44G
98G	65G	43G
98T	65T	42T
101G	63G	43G
100C	30A_32C	42A
100A	61A	44A
101A	60A	45A
102A	58A	46A
102T	59T	47T
103T	58T	44T
101C	57C	44C
101T	59T	46T
103C	59C	45C
105T	59T	44T
104A	59A	43A
105G	57G	42G
105T	56T	42T
107T	57T	43T
105A	56A	43A
107T	53T	45T
102T	53T	45T
103G	50G	46G

106G	50G	43G
107C	51C	44C
107A	54A	42A
107A	52A	42A
102T	51T	41T
104T	52T	43T
104A	53A	44A
105C	53C	44C
107A	54A	45A
105G	26A_27G	46G
106C	28A_25C	44C
104C	55C	44C
100A	57A	47A
103A	56A	47A
103C	55C	47C
98A	55A	47A
102C	56C	47C
105A	58A	47A
105C	59C	46C
109A	59A	46A
105A	60A	43A
103T	62T	45T
103A	62A	43A
102A	64A	43A
103A	64A	42A
103G	30A_32G	41G
103G	62G	41G
103C	63C	41C
99A	61G	40A
97A	63A	39A
97C	64C	39C
97A	64A	40A
97G	28A_33G_1C	40G
96T	65T	40T
96T	63T	39T
96C	64C	40C
94T	66T	39T
98C	69C	41C
99A	75A	43A
99G	74G	42G
97C	74C	42C
96A	73A	42A
95C	77C	44C

95C	77C	44C
49A_47G	74G	44G
95G	75G	44G
95A	75A	43A
96G	76G	43G
99G	79G	44G
95C	78C	44C
99T	79T	45T
97G	80G	45G
96T	79T	45T
101G	79G	45G
100A	79A	43A
103A	79A	42A
104G	80G	41G
103A	80A	43A
102T	81T	43T
100C	80C	41C
102A	82A	41A
101A	83A	42A
101A	84A	42A
102T	85T	42T
103C	45T_40C	43C
101A	82A	44A
100C	83C	44C
98T	84T	42T
97T	84T	42T
98C	84C	41C
96C	85C	43C
96T	84T	43T
94G	81G	42G
94G	82G	43G
96T	80T	43T
95G	80G	41G
95C	79C	39C
94A	79A	38A
92C	79C	39C
91C	80C	38C
89A	80A	38A
91A	78A	36A
91G	77G	33G
87T	78T	32T
88C	78C	33C
90A	78A	33A

86G	80G	33G
83G	79G	33G
84T	79T	31T
84G	81G	34G
84G	80G	34G
84A	79A	32A
83G	78G	32G
83G	78G	33G
83G	78G	32G
81G	77G	33G
80G	78G	33G
80C	77C	32C
80C	78C	34C
80A	79A	36A
82T	79T	36T
81G	79G	34G
81G	81G	33G
79G	78G	33G
81C	78C	35C
79C	78C	34C
80A	80A	36A
82T	78T	35T
79G	73G	33G
78A	74A	33A
76G	75G	33G
77C	74C	33C
78A	69A	31A
78C	69C	31C
76C	71C	33C
76A	68A	33A
76G	69G	33G
78T	69T	33T
76T	68T	32T
75G	68G	32G
74C	67C	31C
73A	67A	32A
75G	69G	32G
74C	70C	33C
75C	70C	33C
74T	69T	33T
72C	71C	33C
70C	72C	31C
72T	72T	31T

72T	73T	34T
76T	73T	36T
76G	71G	33G
78G	70G	35G
77T	70T	37T
76T	70T	35T
77T	71T	36T
78T	71T	38T
80C	73C	39C
79T	73T	42T
80C	75C	44C
79T	73T	42T
78G	73G	42G
78G	70G	41G
77G	73G	42G
80A	75A	42A
83A	75A	42A
86G	75G	42G
89G	78G	40G
90T	78T	41T
93C	78C	41C
95T	79T	42T
95G	78G	45G
95G	79G	47G
100T	81T	48T
101G	81G	48G
103A	81A	49A
101A	80A	50A
105A	80A	50A
103G	79G	50G
101A	77A	52A
99A	78A	53A
101G	76G	54G
104A	79A	54A
105A	78A	53A
107G	78G	54G
107A	77A	54A
109A	80A	51A
112C	82C	53C
114C	83C	54C
119A	84A	52A
117C	84C	54C
119C	85C	55C

118A	83A	57A
118T	83T	57T
119C	84C	54C
120A	84A	57A
122C	80C	57C
120C	79C	58C
119A	81A	59A
119G	78G	59G
119G	80G	60G
122T	81T	59T
124G	80G	60G
124G	82G	60G
125A	83A	59A
126T	84T	58T
128G	84G	57G
127G	84G	57G
129A	84A	57A
130A	86A	58A
129G	39A_49G	58G
129C	88C	55C
129T	87T	55T
129T	88T	55T
127T	89T	56T
127C	92C	55C
130T	91T	55T
130A	92A	57A
133A	93A	58A
134T	93C	55C
129A	91A	55A
126C	90C	56C
125A	91A	57A
124T	91T	57T
124G	92G	56G
124C	93C	56C
125A	92A	52A
125C	89C	53C
129A	89A	52A
129T	91T	50T
132G	92G	51G
133C	94C	53C
132A	95A	54A
132T	96T	55T
132C	97C	55C

129C	95C	55C
128A	95A	56A
126T	93T	57T
125G	93G	55G
123A	94A	56A
123A	92A	57A
123G	92G	55G
122A	92G	53G
117G	89G	54G
115A	91A	54A
115A	93A	55A
116A	96A	55A
111A	98A	54A
110G	101G	54G
114A	106A	54A
114A	108A	53A
111C	111C	52C
109T	109T	53T
111T	110G	53G
109T	111T	53T
107C	113C	53C
105A	113A	52A
102C	109C	53C
101T	109T	52T
98A	110A	52A
102A	109A	51A
101A	109A	51A
101T	108T	49T
101T	109T	50T
100T	108T	50T
101C	108C	50C
98T	109T	50T
98T	110T	50T
101G	108G	51G
101A	108A	49A
102T	107T	50T
98C	108C	49C
96T	107T	49T
99T	102T	51T
99G	50G_51C	50G
99A	102A	52A
97G	100G	52G
99T	99T	53T

96G	100G	52G
96T	100T	53T
96G	97G	55G
97A	96A	55A
97G	99G	21A_55G
96A	97A	59A
99A	95A	59A
99A	93A	62A
97A	92A	61A
99A	91A	61A
94A	39A_52T	29A_31T
96A	90A	61A
97A	91A	59A
97A	95A	60A
96A	95A	58A
83C	96C	57C
93A	98A	57A
94C	97C	58C
93A	98A	59A
95T	99T	58T
95G	99G	56G
91G	95G	58G
92A	95A	56A
90T	94T	56T
88T	93T	54T
90T	91T	54T
88T	90T	54T
88C	92C	55C
86T	96T	54T
89T	95T	54T
88T	96T	55T
89C	98C	52C
90C	98C	52C
90T	100T	51T
92T	97T	51T
96C	100C	51C
99A	98A	50A
97G	97G	51G
101C	93C	52C
105G	93G	53G
104C	89C	50C
107A	85A	51A
109A	83A	51A

109G	82G	52G
108A	82A	53A
107G	81G	53G
110T	82T	52T
112G	78G	55G
114A	76A	53A
114G	76G	56G
115A	73A	58A
117C	72C	59C
115A	75A	58A
113A	74A	58A
113G	74G	56G
114G	72G	56G
113A	73A	53A
114G	75G	54G
113C	75C	56C
112A	76A	55A
110A	77A	56A
110C	79C	57C
110T	78T	56T
110C	79C	57C
111T	80T	57T
111T	82T	57T
112G	83G	59G
111C	83C	59C
108T	82T	59T
107C	82C	58C
109C	80C	58C
108A	80A	55A
108G	80G	56G
107T	81T	57T
104C	79C	58C
102T	78T	55T
98C	79C	55C
97T	78T	52T
97G	78G	52G
97C	80C	51C
97A	79A	51A
95A	80A	50A
93T	79T	51T
91A	75G	50G
95A	39T_35C	50C
102C	74C	51C

104T	75T	52T
104T	75T	52T
107T	75T	52T
109G	74G	51G
107C	75C	54C
107A	73A	52A
107C	76C	52C
107A	77A	52A
106A	76A	53A
107A	78A	53A
109A	78A	53A
108C	76C	51C
109A	72A	52A
108T	39T_35C	52T
44G_63C	73G	51G
108A	71A	53A
106A	71A	52A
104C	71C	54C
103C	73C	55C
102C	72C	56C
99T	73T	56T
99T	73T	56T
98T	75T	58T
93T	73T	31T_26C
95C	77C	57C
90T	79T	57T
88C	79C	58C
88A	78A	58A
88G	80G	57G
86A	80A	57A
82G	79G	56G
81G	78G	55G
84A	79A	53A
86G	80G	54G
83C	80C	53C
82A	79A	51A
84A	76A	51A
86A	76A	52A
89G	77G	53G
90C	77C	53C
94A	77A	53A
93C	76C	51C
92A	75A	51A

95C	75C	51C
95A	73A	50A
95A	71A	48A
97G	71G	48G
97G	70G	51G
94G	72G	50G
95C	72C	50C
93A	71A	48A
95C	35G_38C	47C
97C	77C	49C
99C	78C	49C
99T	78T	48T
99C	77C	50C
100A	77A	50A
101A	77A	49A
104G	75G	48G
105C	77C	46C
109A	79A	46A
108G	80G	46G
106A	81A	46A
105G	78G	45G
107A	76A	46A
105A	75A	46A
112A	78A	45A
109G	80G	45G
106G	80G	45G
107C	77C	45C
108C	75C	44C
109G	75G	20A_24G
107G	74G	43G
107G	77G	44G
106T	76T	43T
107G	80G	43G
108A	79A	43A
108T	80T	43T
109T	79T	43T
109T	79T	43T
106T	80T	44T
107T	82T	43T
108C	83C	43C
109A	83A	45A
111A	83A	45A
111A	82A	45A

113A	81A	45A
114A	81A	44A
115G	81G	44G
114G	82G	42T
111T	82T	44T
113G	83G	40G
111C	80C	40C
111A	80A	40A
109A	78A	39A
110G	77G	42G
110C	76C	40C
111C	74C	40C
113A	73A	41A
115C	74C	40C
118C	75C	42C
119T	75T	44T
117G	77G	43G
119T	40T_39G	41T
118C	81C	41C
117A	81A	42A
116A	82A	42A
114A	85A	42A
113C	86C	42C
110A	87A	42A
112C	84C	22T_20C
113A	86A	42A
113C	86C	41C
112T	84T	40T
113T	86T	41T
115A	84A	41A
113A	85A	41A
114T	86T	39T
114C	83C	39C
117C	86C	40C
116T	86T	41T
115T	84T	41T
113A	80A	39A
113A	79A	40A
113A	81A	40A
113A	81A	39A
114G	81G	38G
116T	79T	38T
119C	81C	39C

121C	82C	40C
117A	81A	41A
120T	82T	41T
122G	81G	41G
122C	83C	41C
121A	83A	42A
120A	87A	42A
119C	84C	43C
120G	83G	45G
120A	83A	46A
122A	84A	46A
120A	85A	23A_23G
120C	88C	45C
120A	89A	47A
116G	86G	47G
117C	85C	48C
118A	84A	48A
115G	83G	50G
114C	82C	51C
113C	85C	51C
110A	84A	51A
114C	84C	51C
113T	82T	52T
112G	82G	49G
112T	82T	49T
109A	81A	51A
116A	80A	23A_26T
116T	82T	51T
118T	81T	51T
119T	83T	49T
118T	83T	49T
119T	82T	49T
120C	81C	50C
119A	84A	47A
125C	83C	48C
125G	82G	48G
126A	81A	46A
127G	83G	46G
125T	85T	47T
125A	85A	49A
124C	85C	49C
123C	84C	48C
122T	86T	48T

122T	84T	48T
122G	81G	48G
124G	79G	49G
126A	81A	47A
127T	82T	48T
126T	81T	49T
126T	80T	48T
128A	80A	48A
122G	80G	49G
121C	78C	49C
122A	80A	50A
121C	80C	52C
122C	80C	51C
117A	81A	52A
117C	83C	30T_23C
75G_42C	81G	54G
117C	79C	54G
114T	76T	53T
117C	80C	52C
119A	79A	52A
119A	81A	53A
118A	82A	51A
117A	80A	52A
117T	80T	52T
114T	80T	52T
111A	82A	53A
109G	81G	52A
106T	80T	52T
109A	80A	51A
108G	77G	51G
108G	77G	51G
108G	75G	51G
109A	78A	51A
107G	74G	49G
106C	74C	49C
106T	74T	47T
107T	76T	48T
104G	75G	48G
108C	73C	22T_27C
109G	69G	48G
110A	68A	45A
111G	68G	45G
107A	70A	44A

107G	74G	45G
110A	75A	43A
114G	73G	42G
116T	71T	42T
117G	73G	42G
114G	73G	42G
113G	76G	40G
113A	77A	40A
111A	75A	42A
112G	75G	40G
104A	75A	39A
104A	75A	38A
100G	74G	38G
97A	73A	39A
99T	71T	40T
100A	70A	39A
101A	70A	39A
101C	68C	40C
96C	68C	41C
96A	69A	40A
50A_246T_50G_75C	70T	41T
70G	69G	42G
62C	68C	39C
62C	68C	38C
60T	65T	38T
60A	66A	39A
59G	66G	36G
59C	65C	37C
59T	66T	38T
57T	65T	39T
56T	65T	39T
57T	64T	40T
57A	63A	38A
57T	62T	40T
93T	62T	40T
93G	63G	41G
92G	64G	40G
91T	65T	40T
93G	66G	38G
92G	64G	40G
91C	64C	38C
94A	62A	38A
95G	62G	36G

95G	65G	36G
96A	66A	35A
94A	63A	37A
92T	64T	38T
96A	66A	38A
99C	67C	38C
99C	71C	38C
100T	70T	39T
103T	72T	41T
105T	70T	40T
104A	72A	40A
110T	71T	39T
109G	73G	39G
111T	75T	40T
108T	76T	41T
107C	74C	41C
106T	73T	40T
108G	73G	40G
108A	43A_30T	40A
106G	72G	40G
104G	70G	39G
105A	71A	40A
104C	71C	39C
108C	70C	40C
107A	72A	42A
109A	72A	44A
111A	71A	22A_23G
109A	66A	45A
107C	65C	45C
104A	67A	44A
106C	66C	44C
109A	64A	44A
108A	62A	43A
115A	60A	43A
114A	58A	46A
114T	61T	47T
111C	62C	47C
112T	63A	47A
113G	62G	47G
118A	62A	45A
117G	62G	46G
115A	64A	46A
116A	65A	47A

111A	66A	46A
109T	66T	44T
109C	67C	44C
109C	66C	44C
108A	68A	44A
109G	69G	42G
106T	69T	43T
105T	69T	43T
105C	71C	43C
104T	73T	43T
106A	72A	43A
105G	72G	44G
106A	75A	44A
111A	74A	42A
112G	76G	43G
110A	75A	42A
109A	76A	43A
108G	77G	41G
107T	77T	40T
107G	78G	40G
108C	79C	40C
110T	75T	39T
109C	75C	39C
110T	76T	40T
111T	75T	40T
111T	75T	40T
109C	74C	40C
111A	70A	41A
111A	71A	41A
112G	71G	40G
112T	71T	40T
109G	70G	39G
109A	68A	40A
111C	65C	40C
109A	65A	39A
106A	64A	37A
104A	65A	37A
103A	62A	37A
101A	64A	39A
101A	61A	40A
101G	60G	41G
101G	60G	40G
101T	63T	41T

105G	64G	41G
106T	65T	41T
106A	67A	42A
108A	70A	41A
111T	71T	42T
112T	70T	41T
116A	70A	40A
113T	70T	40T
115A	69A	38A
114A	69A	36A
112A	68A	35A
111A	68A	34A
113T	69T	35T
114C	69C	35C
114A	69A	37A
111A	71A	37A
114A	72A	39A
109T	72T	40T
108T	72T	38T
108A	71A	36A
110A	74A	36A
109A	74A	36A
110A	75A	36A
106G	74G	36G
109G	73G	35G
109A	73A	33A
107G	70G	33G
110A	70A	33A
110G	72G	33G
104T	72G	12T_20G
101T	73T	33T
100A	73A	33A
99T	29T_43G	33T
105C	72C	33C
56T_53G	73G	13T_20G
120G	73G	35G
120T	72T	34T
121G	71G	35G
122T	73T	37T
124C	72C	37C
122G	43A_32G	37G
120T	74T	36T
122C	74C	38C

124T	73T	39T
73T_56C	74C	40C
129C	73C	41C
132A	71A	42A
131T	71T	42T
129T	71T	42T
129G	73G	44G
128T	74T	43T
130C	76C	45C
128T	76T	44T
130C	77C	47C
133T	78T	47T
132G	78G	46G
130G	76G	46G
131A	77A	46A
130A	75A	48A
131G	75G	48G
134A	75A	47A
136C	75C	47C
137A	74A	46A
135A	74A	48A
137A	76A	49A
137C	74C	48C
136A	75A	48A
134A	74A	49A
141C	75C	51C
143A	72A	51A
141C	72C	52C
141C	72C	53C
142T	71T	53T
140T	70T	53T
139C	70C	52C
136C	70C	52C
135T	72T	54T
134G	71G	54G
132T	72T	56T
134G	72G	56G
134T	71T	56T
135A	73A	56A
137T	71T	55T
136A	71A	55A
136T	71T	54T
137G	69G	54G

134G	66G	53G
133A	65A	53A
135A	66A	53A
134T	67T	54T
133T	65T	55T
133C	64C	55C
132T	62T	55T
130G	60G	55G
129G	62G	53G
128A	62A	55A
127G	63G	55G
123T	66T	56T
121T	67T	56T
119G	65G	56G
114T	63T	55T
114G	62G	55G
114A	61A	55A
112A	63A	57A
108T	63T	57T
108A	60A	58A
107G	60G	58G
109C	60C	58C
107A	58A	56A
106A	58A	55A
104T	56T	56T
106A	56A	59A
104T	56T	57T
103A	57A	55A
99G	57G	54G
98A	58A	54A
96T	58T	52T
100C	57C	53C
103A	59A	54A
104C	59C	52C
101A	57A	51A
102T	61T	49T
103T	62T	49T
101G	61G	46G
96C	63C	45C
95T	63T	45T
97G	64G	46G
96G	64G	42G
99G	64G	44G

96G	66G	43G
93A	66A	43A
93G	66G	42G
94G	66G	43A
94A	64A	43A
94A	64A	42A
91A	65A	42A
93G	66G	39G
93C	66C	38C
87A	65A	36A
89G	67G	35A
89T	66T	34T
86A	66A	33A
87A	68A	33A
88A	65A	33A
88G	64G	33G
87A	66A	33A
88T	64T	32T
88A	65A	33A
89A	66A	31A
89A	66A	31A
89C	68C	32C
88C	67C	33C
85C	68C	34C
45A_41G	68G	35G
88G	70G	36G
88T	70T	35T
91T	70T	35T
91C	70C	35C
93C	70C	36C
94T	70T	35T
92G	69G	35G
92A	67A	33A
90A	69A	36A
91C	72C	36C
88C	73C	37C
91A	72A	36A
91A	74A	37A
90G	71G	36G
89C	70C	36C
90A	73A	35A
94G	73G	36G
94C	72C	38C

95C	72C	37C
97C	71C	36C
102T	70T	36T
107G	71G	34G
106G	73G	35G
106T	74T	35T
106G	73G	35G
107A	76A	35A
107A	77A	33A
105A	78A	33A
105C	78C	33C
106T	78T	33T
105T	76T	33T
107A	77A	33A
108C	76C	33C
105C	76C	33C
103C	73C	32C
101A	73A	33A
102G	73G	33G
102A	67A	35A
102C	67C	36C
104T	67T	38T
104G	64G	38G
105A	66A	38A
103A	66A	36A
107G	66G	37G
106A	66A	37A
107G	64G	37G
106A	64A	36A
107A	66A	38A
107T	66T	39T
105A	67A	38A
105A	66A	39A
108G	66G	39G
111G	66G	40G
112A	71A	41A
113A	74A	42A
111T	72T	45T
107T	72T	46T
111T	72T	46T
108C	71C	46C
112A	73A	46A
110A	73A	45A

110G	73G	45G
110C	75C	43C
111A	74A	44A
112G	74G	45G
113A	74A	45A
113C	74C	45C
113A	73A	43A
114A	73A	41A
116A	73A	43A
114C	79C	45C
117T	80T	47T
117C	80C	46C
117C	80C	47C
114T	80T	45T
113G	79G	46G
115C	80C	45C
120T	82T	46T
120T	82T	45T
120G	80G	45G
121T	79T	46T
117C	82C	48C
118T	81T	49T
120A	81A	49A
121C	81C	50C
120T	78T	51T
118T	82T	50T
121G	84G	51G
124G	84G	53G
123A	84A	52A
123G	83G	52G
120G	84G	51G
122G	83G	51G
123A	86A	51A
127G	86G	53G
126T	83T	52T
125G	81G	54G
126C	81C	52C
126A	80A	52A
125A	81A	52A
125G	83G	53G
127T	83T	52T
128G	84G	51G
129A	84A	52A

128A	86A	51A
128G	87G	52G
130G	88G	52G
131A	89A	50A
134C	89C	48C
135C	91C	49C
135A	91A	50A
136G	89G	51G
136C	90C	52C
132T	89T	51T
131C	90C	51C
130T	90T	51T
131T	91T	52T
131G	90G	52G
131C	47A_43C	52C
133G	90G	53G
133C	92C	51C
132C	91C	50C
128C	90C	52C
127G	85G	52G
130C	83C	1A_20T_30C
128A	82A	51A
128G	85G	50G
129C	84C	49C
132A	82A	51A
134C	80C	51C
135C	82C	51C
134A	81A	52A
134G	79G	51G
132G	79G	52G
128T	81T	51T
130T	81T	52T
128G	82G	53G
128T	82T	55T
128G	83G	54G
128C	82C	53C
128A	75A	50A
124G	75G	49G
121G	77G	48G
121A	77A	50A
122T	77T	50T
122G	77G	50G
121G	79G	51G

118A	76A	49A
119C	79C	51C
122C	81C	51C
121C	81C	50C
121G	36A_44G	47G
123G	80G	44G
121C	80C	47C
120T	79T	46T
122T	79T	45T
121G	75G	45G
119G	72G	43G
115G	74G	42G
119T	75T	42T
117G	76G	43G
114G	78G	43G
114C	77C	43C
112A	73A	43A
111A	74A	42A
110C	73C	43C
111C	73C	43C
111G	74G	45G
113T	74T	46T
117T	74T	46T
118G	74G	47G
112A	74A	49A
111G	75G	49G
111A	79A	49A
112G	79G	51G
112G	78G	50G
109T	80T	51T
108C	80C	51C
106C	79C	50C
106A	80A	49A
110C	85C	48C
110C	86C	47C
114T	85T	46T
113A	87A	48A
115G	86G	52G
117G	44T_41G	52G
118T	86T	52T
119T	86T	55T
118G	87G	54G
119G	90G	52G

59A_61G	88G	53G
121G	92G	55G
121A	92A	54A
123A	96A	54A
120G	95G	55G
122A	99A	52A
122A	98A	51A
121C	98C	52C
120A	98A	53A
115G	98G	54G
116A	97A	56A
116G	99G	55G
117A	100A	56A
117G	99G	55G
117G	100G	54G
116G	99G	55G
117G	99G	55G
117A	98A	54A
119G	100G	55G
115A	101A	54A
113T	104T	55T
113T	103T	55T
57T_58C	101C	23G_30C
115A	102A	55A
117G	102G	56G
116G	102G	55G
117A	101A	55A
114C	101C	55C
113T	99T	55T
113A	100A	53A
113C	99C	53C
57A_55C	100C	54C
109C	98C	53C
113A	101A	51A
116A	101A	52A
122C	101C	52C
122C	103C	52C
122T	103T	50T
126C	103C	51C
126G	103G	22A_29G
126T	104T	51T
127T	101T	51T
126G	102G	51G

127C	104C	52C
125C	103C	50C
125C	105C	50C
126T	104T	49T
125G	103G	47G
121G	103G	46G
122G	105G	46G
123G	103G	45G
123A	102A	46A
126A	102A	47A
125G	99G	47G
125G	98G	45G
124G	99G	45G
127C	97C	44C
126C	98C	44C
66A_61G	49A_48G	25A_19G
125G	96G	44G
123C	92C	45C
121C	93C	45C
117A	94A	46A
119T	93T	44T
117C	92C	42C
119A	93A	42A
116G	93G	41G
115A	92A	37A
114A	93A	38A
112A	91A	39A
113G	92G	40G
108A	88A	40A
114C	88C	37C
111C	85C	39C
111T	87T	40T
110C	83C	39C
111C	84C	40C
110C	85C	39C
113A	86A	38A
111T	88T	37T
110C	89C	35C
111G	90G	34G
108G	90G	34G
107A	90A	34A
106T	88T	35T
104G	87G	33G

103C	86C	33C
105A	88A	32A
105G	86G	32G
107G	84G	34G
108G	87G	35G
110T	86T	35T
116T	90T	37T
114T	88T	38T
114T	88T	38T
114A	89A	38A
112A	89A	38A
112A	93A	38A
113T	94T	37T
111T	90T	37T
111T	92T	38T
113T	91T	38T
114A	91A	41A
113G	88G	43G
110A	91A	44A
104A	92A	44A
103T	92T	44T
53G_51C	96G	45G
99T	96T	45T
97A	95A	45A
97A	95A	44A
98A	94A	44A
100T	95T	44T
100C	94C	43C
100T	92T	44T
100A	90A	44A
99G	91G	44G
100C	91C	43C
99A	92A	43A
96G	90G	42G
98T	92T	43T
98T	93T	39T
97C	91C	39C
99A	91A	40A
101G	89G	40G
104G	90G	42G
101G	91G	43G
100A	90A	43A
104C	90C	44C

103C	92C	45C
103A	92A	44A
103G	94G	44G
103A	92A	48A
103C	91C	50C
102A	92A	50A
98T	92T	49T
103T	91T	50T
102T	91T	49T
102T	89T	50T
100C	88C	49C
98T	87T	48T
98C	87C	48C
93C	86C	49C
92C	87C	47C
92T	85T	48T
90C	85C	49C
89T	81T	47T
90C	83C	48C
87A	83A	48A
87G	83G	48G
87G	80G	45G
86G	79G	49G
86A	80A	50A
86T	80T	50T
85G	80G	51G
87T	79T	51T
88G	80G	52G
90T	78T	51T
89G	78G	54G
88T	79T	53T
89A	74A	52A
86T	74T	51T
79G	70G	49G
81T	73T	45T
81T	74T	45T
86T	73T	44T
89T	72T	45T
89G	68G	45G
88T	69T	45T
89G	69G	46G
90T	68T	48T
89A	67A	49A

91G	67G	47G
88G	67G	44G
91T	63T	45T
91T	61T	44T
92C	60C	45C
93C	61C	45C
94T	56T	44T
93C	59C	46C
91C	58C	46C
90C	98C	45C
46T_1G_41C	76C	45C
88T	63T	49T
88G	65G	48G
87G	65G	48G
87A	65A	49A
87C	69C	50C
87A	70A	52A
87A	71A	51A
88C	72C	51C
89A	71A	53A
89C	73C	52C
88C	72C	53C
86T	74T	53T
83T	74T	51T
85A	75A	51A
87C	75C	51C
83A	75A	51A
81A	75A	51A
82G	73G	51G
83A	69A	50A
83A	69A	46A
81T	71T	45T
82G	74G	43G
82G	74G	44G
79C	73C	44C
78A	75A	44A
80C	75C	43C
81A	74A	45A
82A	74A	43A
83C	73C	42C
83T	74T	42T
84G	74G	42G
87G	74G	40G

87A	74A	40A
86A	75A	41A
87G	73G	42G
85G	72G	44G
84A	39A_30G	46A
85C	72C	46C
87A	39A_31C	50A
89T	71T	51T
95A	72A	51A
96A	71A	51A
100A	73A	50A
100A	75A	49A
95A	75A	50A
95G	75G	48G
95T	74T	48T
96G	73G	49G
96T	73T	50T
99G	73G	52G
99C	73C	52C
102C	24T_70C	52C
98T	74T	55T
96T	73T	55T
97T	75T	56T
97T	75T	56T
97T	72T	57T
99T	72T	54T
99G	69G	56G
95G	71G	56G
99A	70A	56A
96A	69A	58A
97A	68A	58A
95G	68G	57G
95G	64G	56G
95G	68G	58G
96G	68G	56G
96G	67G	57G
98C	69C	58C
99A	70A	60A
100T	69T	57T
100C	72C	58C
101C	79C	57C
101C	76C	58C
101G	74G	58G

104C	73C	58C
104A	76A	58A
101G	78G	56G
99A	78A	56A
100G	77G	56G
100G	75G	55G
99C	75C	57C
101C	76C	57C
99A	75A	58A
98C	76C	58C
98C	77C	57C
101T	78T	57T
101G	78G	58G
100G	77G	59G
101C	79C	59C
103C	75C	58C
105A	75A	61A
111A	75A	60A
110A	77A	59A
110G	75G	59G
106T	76T	60T
111G	78G	59G
111G	80G	61G
110A	79A	60A
115T	80T	61T
113T	80T	62T
113C	82C	62C
114T	81T	62T
115C	81C	61C
114A	83A	61A
115T	83T	59T
116G	83G	58G
114T	83T	58T
113T	83T	54T
112A	83A	52A
106T	81T	52T
106C	82C	51C
101C	79C	51C
100A	79A	50A
101C	79C	49C
102C	79C	49C
102T	77T	48T
99C	80C	47C

99A	79A	46A
97G	78G	44G
96G	77G	44G
92C	77C	44C
92C	76C	42C
89G	77G	42G
88G	75G	41G
87A	73A	42A
91G	74G	41G
87A	73A	38A
88A	74A	37A
87G	74G	37G
84T	74T	36T
83C	74C	35C
80A	73A	35A
80C	73C	35C
79T	71T	36T
79C	71C	35C
80C	70C	35C
78A	71A	34A
76C	70C	34C
74T	68T	32T
71C	67C	30C
72C	64C	31C
71T	57T	31T
70G	57G	29G
69G	58G	27G
65A	57A	26A
66G_28C	51G_15C	1A_26G_17C
35C	34C	10C
48G	45G	20G
53C	48C	23C
14A_44G_5C	16A_32G	9A_13G_1C
46C	51C	19C
42G	50G	18G
45C	47C	19C
48C	47C	20C
48C	46C	20C
48A	44A	20A
49C	45C	20C
51C	45C	19C
50C	45C	20C
48A	47A	19A

47C	44C	19C
46C	45C	20C
44C	45C	19C
46T	44T	21T
42G	46G	22G
42A	43A	21A
44A	42A	26A
41T	43T	27T
41A	44A	25A
44T	43T	25T
44A	43A	26A
45C	44C	25C
21A_24G	43G	25G
45C	42C	24C
47C	41C	25C
49A	39A	26A
49C	42C	27C
48C	42C	27C
50C	44C	30C
50A	43A	28A
50C	44C	28C
50C	44C	30C
50T	44T	31T
51G	46G	31G
52C	48C	32C
53C	52C	34C
55C	52C	34C
57A	52A	34A
57T	57T	35T
58A	58A	36A
59T	58T	34T
60A	56A	35A
60G	54G	34G
61A	28A_26G	35A
64T	55T	35T
62C	54C	37C
61G	55G	36G
57T	53T	36T
58G	53G	36G
58C	28T_25C	36C
58T	53T	36T
61G	52G	37G
31A_30G	26A_29G	37A

65C	58C	37C
65A	57A	37A
69G	57G	36G
70G	57G	38G
71A	58A	40A
76A	62A	40A
77G	62G	40G
76T	62T	43T
78G	65G	44G
78T	66T	43T
77G	66G	41G
80A	68A	43A
81C	68C	42C
40A_42G	68G	45A
84T	68T	44T
86C	67C	46C
85A	69A	47A
86A	71A	48A
87T	71T	49T
88T	71T	49T
90G	74G	50G
92T	74T	52T
95G	78G	52G
96A	79A	52A
97C	39T_41C	51C
100G	80G	53G
100A	79A	54A
101T	79T	56T
101G	80G	56G
100G	80G	56G
99A	80A	53A
98G	78G	53G
98A	78A	54A
97A	78A	51A
99A	79A	49A
103T	79T	48T
99G	79G	49G
99A	79A	48A
102G	80G	48G
102C	81C	48C
100A	82A	48A
98C	42A_40C	47C
97T	83T	46T

98C	82C	44C
96G	82G	45G
96G	80G	44G
96A	80A	44A
96G	83G	44G
37T_56C	82C	38T
55T_37G_1C	82T	40G
101C	81C	44C
102T	79T	46T
105C	76C	47C
102T	77T	48T
102C	82C	49C
102T	80T	49T
103C	80C	48C
102T	83T	49T
103T	84T	50T
105T	85T	50T
106A	86A	48A
102T	86T	47T
104C	87C	47C
105T	86T	49T
105A	88A	49A
105A	88A	48A
105G	88G	49G
107C	89C	51C
104A	88A	52A
104T	86T	53T
102C	87C	54C
48A_54G	86G	56G
101C	85C	56C
99T	84T	54T
96G	85G	52G
93G	82G	52G
93A	81A	52A
93T	83T	49T
76T	81T	47T
76A	80A	49A
78G	80G	51G
75A	75A	51A
71A	76A	52A
21G_50C	1T_35G_31C	13G_27C
73C	66C	39C
77A	67A	39A

21A_56C	34A_33C	13A_1G_26C
78C	68C	39C
58T_21C	34T_35C	25T_13C
101T	76T	43T
102T	74T	44T
101C	77C	43C
101T	75T	44T
102C	76C	43C
102T	79T	44T
101G	80G	45G
104G	84G	43G
104T	84T	45T
106C	85C	46C
106T	87T	46T
107A	87A	47A
107G	87G	48G
107C	88C	46C
108A	88A	47A
107A	88A	49A
108G	88G	51G
104G	90G	51G
106C	90C	50C
104C	89C	50C
105C	87C	49C
103T	88T	50T
104T	88T	49T
103C	88C	49C
103C	89C	49C
102T	90T	48T
100T	92T	47T
101A	93A	47A
96A	90A	47A
102C	92C	49C
49A_52G	92G	21A_29G
100T	92T	53T
99A	93A	52A
96T	92T	51T
95G	92G	49G
96A	87A	50A
99C	86C	49C
100T	36T_54C	49T
100A	90A	49A
98A	92A	48A

96G	91G	48G
96G	90G	51G
95T	88T	53T
98G	89G	53G
97C	90C	54C
96A	89A	53A
99C	89C	52C
99A	90A	53A
98T	89T	51T
100T	89T	50T
104T	88T	50T
106T	39A_87T	49T
105T	75T	42A_46T_20G
100T	43A_48T	20A_21T_6G
98A	91A	56A
104A	90A	54A
105A	88A	56A
105A	87A	56A
106A	85A	57A
107A	85A	59A
107A	85A	62A
104C	81C	33A_25C
104A	80A	56A
105G	82G	56G
102A	86A	55A
101T	86T	55T
100C	87C	56C
97T	86T	57T
99G	85G	58G
101T	84T	57T
103A	84A	60A
104T	82T	60T
111T	83T	60T
115C	83C	61C
118C	80C	61C
118T	81T	59T
118T	78T	60T
119C	77C	60C
119T	75T	61T
118T	74T	60T
121T	77T	61T
121C	78C	58C
132C	77C	57C

132C	77C	57C
137A	77A	58A
138C	77C	58C
139G	77G	32A_1T_24G
138G	78G	57G
139C	80C	58C
139C	82C	58C
139C	82C	25T_33C
142C	84C	56C
142A	84A	56A
143G	82G	55G
144C	82C	58C
144T	83T	59T
143G	84G	60G
143T	84T	61T
141A	86A	59A
141C	88C	60C
142C	90C	59C
146T	89T	59T
150C	90C	59C
151C	91C	61C
150T	91T	61T
150G	92G	60G
147G	89G	60G
148C	85C	60C
150C	84C	59C
150G	83G	57G
149G	83G	57G
150T	82T	55T
149C	82C	57C
148A	81A	58A
147C	81C	55C
147C	80C	54C
144A	79A	54A
146G	76G	54G
145C	77C	54C
144A	77A	55A
140C	79C	52C
135C	79C	53C
133A	78A	53A
136T	82T	52T
136G	82G	51G
134C	83C	52C

136T	87T	53T
133G	90G	53G
134T	92T	52T
135G	91G	51G
137C	96C	51C
140T	94T	50T
138C	94C	51C
135C	96C	53C
135T	93T	55T
134T	94T	54T
138C	93C	54C
144C	96C	53C
142C	98C	51C
141A	98A	55A
141T	98T	53T
137A	99A	52A
133C	98C	50C
127T	97T	50T
124G	98G	51G
124C	98C	51C
127A	100A	51A
125C	99C	52C
124T	100T	50T
125T	100T	51T
121G	98G	51G
121G	97G	49G
116C	97C	49C
119G	98G	48G
117G	98G	48G
119A	96A	48A
119A	95A	49A
121G	93G	48G
120A	92A	48A
121T	91T	47T
120G	91G	47G
120T	89T	47T
121G	90G	50G
124G	88G	51G
122T	88T	49T
122C	87C	48C
122A	87A	46A
126A	88A	45A
127A	85A	45A

127A	84A	44A
127A	83A	43A
125C	82C	44C
122C	83C	45C
123A	84A	45A
123G	83G	46G
124C	81C	47C
122A	82A	48A
120G	85G	47G
121T	85T	48T
121T	86T	48T
124C	89C	48C
126G	89G	50G
125C	90C	49C
124T	89T	45T
123C	92C	48C
122C	93C	49C
125A	98A	48A
124G	99G	48G
125C	98C	47C
126A	100A	46A
127G	98G	46G
134T	99T	46T
134T	99T	46T
138C	96C	46C
139T	95T	45T
139G	93G	44G
136C	90C	43C
137A	87A	44A
135G	85G	44G
137C	86C	44C
137A	81A	43A
133G	81G	43G
135C	80C	42C
137T	78T	40T
138T	76T	38T
140C	75C	38C
136A	75A	39A
134T	72T	40T
134C	70C	40C
133T	68T	39T
131G	71G	40G
132G	73G	40G

130T	73T	39T
132C	74C	39C
132T	74T	38T
135C	74C	39C
132C	72C	40C
133G	72G	41G
133A	71A	40A
135C	71C	41C
136C	70C	41C
137T	69T	42T
135G	70G	42G
131G	70G	42G
129A	71A	43A
126A	71A	44A
124G	73G	45G
123A	75A	46A
122A	74A	47A
121G	75G	51G
120A	74A	52A
119A	74A	51A
116A	72A	49A
114G	71G	49G
114G	72G	48G
114C	71C	48C
113A	68A	48A
53A_58G	67G	48G
113G	66G	48G
112G	66G	50G
113A	68A	50A
112G	67G	49G
116C	68C	52C
116A	66A	49A
117A	65A	50A
118A	64A	49A
119G	66G	49G
120C	35T_29C	49C
118C	63C	47C
123G	63G	47G
119G	59G	45G
120T	59T	46T
123G	60G	46G
123A	61A	47A
123G	61G	47G

125A	60A	48A
123A	55A	50A
122G	53G	51G
57G_63C	55C	32G_19C
119C	56C	51C
119G	56G	54G
107C	53C	51C
111T	54T	52T
110G	55G	52G
108G	56G	50G
106C	57C	53C
106C	58C	52C
113C	59C	52C
113T	1A_34T_26C	32T_23C
113A	60A	51A
112C	61C	52C
114A	62A	52A
112C	63C	56C
114T	35T_28C	56C
117G	66G	56G
116C	70C	57C
116A	71A	56A
111A	70A	54A
118G	71G	55G
120G	71G	53G
121G	73G	53G
122C	72C	53C
120C	71C	54C
120A	73A	53A
117G	72G	54G
119A	73A	53A
119G	75G	53G
111G	76G	54G
118C	77C	53C
118T	75T	55T
119G	79G	55G
118C	80C	53C
116C	81C	55C
119A	79A	55A
119T	80T	54T
57A_60T	80T	54T
117G	81G	56G
116C	80C	56C

55A_61G	81G	24A_31G
119T	81T	51T
119G	80G	51G
118G	79G	51G
119A	81A	51A
119G	82G	51G
119A	80A	51A
120G	82G	51G
123C	82C	52C
123T	83T	53T
125T	85T	53T
128A	86A	54A
125A	85A	55A
127G	84G	57G
123G	83G	53G
121C	85C	55C
120A	86A	56A
120G	86G	56G
119A	83A	56A
120G	84G	56G
120G	86G	57G
117G	87G	58G
116G	88G	59G
117C	88C	57C
115G	85G	58G
117G	85G	57G
116G	85G	60G
114T	87T	60T
117C	87C	59C
118C	87C	59C
117C	85C	59C
121T	85T	58T
122C	88C	57C
120G	89G	58G
122G	88G	58G
124T	88T	59T
124T	43T_45G	59G
125T	87T	57T
122C	88C	59C
114A	88A	58A
117G	88G	57G
116A	91A	56A
115C	91C	59C

116C	91C	62C
117C	91C	59C
111A	88A	57A
111C	92C	65C
111G	89G	63G
110C	90C	63C
110G	92G	64G
107C	91C	65C
108C	89C	65C
108G	87G	68G
106T	86T	66T
104T	86T	67T
108T	83T	69T
111T	82T	69T
109A	82A	70A
107T	80T	69T
111T	80T	68T
111T	78T	69T
111T	78T	67T
112T	74T	32T_36C
113A	74A	68A
117A	72A	67A
115A	72A	67A
113A	71A	35A_31C
114T	69T	65T
114A	69A	62A
112A	66A	61A
117G	67G	62G
115C	65C	31T_32C
116G	66G	64G
118A	66A	65A
117C	65C	65C
117A	65A	64A
118T	65T	64T
118G	64G	63G
57T_59C	33T	63T
115T	33T	61T
111A	35A	59A
109T	35T	58T
110T	35T	56T
109T	35T	55T
110T	37T	55T
109G	36G	55G

110G	37G	53G
108C	39C	52C
108C	39C	52C
106G	40G	53G
105C	40C	53C
103A	40A	52A
101A	40A	51A
99A	40A	50A
98C	40C	50C
95A	40A	50A
94A	41A	46A
94G	39G	44G
91A	41A	44A
90T	42T	43T
90G	44G	43G
85C	42C	42C
83G	41G	41G
83T	40T	41T
80G	42G	40G
77G	42G	39G
77T	41T	39T
75T	41T	38T
75G	41G	39G
75G	41G	39G
72G	40G	25G
68A	40A	28A
68A	38A	27A
66C	38C	24C
1A_1G	38A	23A
1T	38T	22T
1C	37C	19C
16G	54G	30G
55C	53C	30C
56G	52G	30G
56G	53G	29G
59C	53C	28C
59T	55T	26T
59C	56C	26C
60C	58C	25C
60C	59C	11T_14C
60G	61G	26G
62G	61G	25G
64A	61A	26A

65A	61A	25A
65G	62G	26G
65C	63C	25C
65A	64A	27A
65T	64T	26T
67C	64C	28C
69A	66A	28A
70T	66T	28T
70T	67T	28T
72C	66C	28C
72C	68C	28C
73C	66C	29C
73C	67C	30C
71T	65T	28T
68A	63A	28A
68T	64T	29T
70T	63T	28T
70T	63T	28T
68A	61A	28A
68G	63G	28G
70T	63T	28T
68G	61G	27G
69A	60A	26A
71G	59G	25G
68G	58G	25G
68G	57G	10A_13G
67G	54G	25G
69A	55A	23A
69G	52G	25G
70A	52A	25A
68A	51A	24A
67G	51G	23G
68A	49A	22A
67C	50C	23C
67A	50A	23A
67C	50C	23C
67A	50A	23A
68C	49C	22C
66A	51A	22A
67C	50C	7G_15C
69C	49C	23C
68A	49A	24A
70G	49G	24G

69C	49C	24C
68C	49C	24C
68A	47A	23A
71C	48C	24C
70A	48A	23A
71A	49A	23A
72G	49G	23G
69A	49A	23A
67A	49A	23A
65T	49T	23T
63A_81C	49A	21A
18G_65C	50C	8A_12T_12G_39C
64G	53G	22G
64G	52G	21G
67G	52G	22G
68G	53G	22G
69G	22T_85G	22G
70G	52G	22G
72G	51G	22G
73G	47G	9A_14G
72T	51T	22T
76G	52G	8T_14G_1C
78C	50C	24C
79A	49A	24A
78T	50T	24T
77A	48A	25A
78T	49T	26T
80G	49G	26G
82G	50G	25G
82G	48G	25G
82C	48C	25C
85C	47C	23C
86C	46C	25C
87C	44C	26C
86A	43A	27A
87C	43C	28C
88A	43A	26A
90A	45A	26A
39A_53G	17A_29G	26G
93T	46T	26T
93C	47C	27C
93A	46A	26A
93C	46C	27C

94C	50C	27C
53A_41G	50G	12A_13G
97G	52G	25G
97C	51C	24C
97C	51C	26C
99A	51A	29A
99C	51C	29C
99C	52C	29C
99C	52C	29C
98T	53T	30T
98T	53T	28T
97C	53C	28C
100C	53C	28C
101C	53C	27C
98T	54T	28T
100G	55G	26G
99A	56A	25A
99C	55C	25C
99C	34T_20C	24C
97G	33T_20G_1C	24G
97A	56A	26A
99G	57G	26G
95A	57A	26A
94A	58A	27A
94G	61G	27G
92T	59T	28T
91A	61A	28A
44A_1T_46C	60C	28C
93A	61A	28A
93A	60A	28A
95A	60A	28A
94T	61T	24T
94C	59C	26C
93C	61C	25C
94A	61A	28A
94G	60G	28G
94A	61A	28A
95T	61T	28T
94C	58C	28C
92C	57C	29C
93G	58G	28G
93A	58A	28A
92A	59A	28A

92C	58C	28C
91A	60A	28A
89G	60G	28G
83A	61A	27A
83T	57T	27T
85G	55G	27G
84T	56T	27T
83G	56G	27G
84T	55T	26T
84A	58A	25A
83A	60A	24A
80A	59A	24A
80C	37T_1G_21C	25C
79A	60A	25A
78C	61C	25C
74A	60A	11A
72A	61A	13A
92A	61A	22A
92T	62T	23T
60T	62T	25T
62A	63A	26A
62A	61A	26A
63G	62G	26G
64G	62G	28G
64G	62G	13G_14C
64A	64A	27A
64C	64C	27C
39T_25C	60T	27T
65T	59T	28T
38G_25C	26G_34C	28C
67C	60C	13T_17C
68G	62G	36G
65G	62G	36G
65T	65T	36T
65G	64G	36G
65A	62A	36A
64T	63T	36T
64G	65G	36G
64G	65G	36G
64A	67A	36A
67A	68A	36A
67C	67C	38C
66A	67A	39A

68G	65G	39G
69A	65A	38A
69G	65G	39G
66C	65C	39C
65A	65A	40A
66T	64T	39T
64A	67A	42A
35T_30G	68T	19T_22G
69T	65T	41T
68T	64T	40T
69T	64T	40T
69G	64G	41G
71G	64G	39G
72C	63C	39C
70T	64T	37T
71T	65T	40T
70C	67C	41C
31T_39C	64T	19T_1G_22C
72T	62T	41T
75A	63A	42A
75G	63G	43G
74A	64A	41A
73A	64A	42A
75A	66A	44A
75C	65C	42C
73T	64T	43T
74G	64G	43G
73G	64G	43G
74A	63A	40A
74G	64G	45G
73G	63G	45G
73G	63G	42G
72C	64C	43C
30A_39G	63A	22A_21G
67A	63A	43A
66C	62C	43C
63A	59A	43A
63C	57C	41C
63C	58C	39C
61A	58A	38A
25A_36G	58A	19A_18G
61G	59G	37G
61T	59T	38T

61A	59A	39A
59T	58T	38T
59A	57A	37A
35T_22C	57C	18T_17C
57T	56T	34T
55T	55T	34T
53T	54T	34T
52G	53G	33G
51C	52C	33C
50A	54A	32A
49G	54G	32G
49C	54C	32C
47T	54T	31T
48G	51G	28G
47C	51C	29C
45C	50C	26C
45C	49C	25C
46A	46A	26A
47A	46A	26A
47A	47A	26A
47A	46A	26A
48T	44T	25T
47C	43C	26C
46T	41T	26T
44G	40G	25G
44A	40A	24A
45G	38G	23G
43G	38G	23G
44A	40A	24A
46A	40A	24A
46G	38G	24G
45G	38G	23G
42C	39C	22C
42T	36T	20T
41G	34G	20G
38G	34G	20G
38T	34T	18T
37C	33C	19C
37T	32T	18T
34A	31A	18A
32C	31C	18C
32A	29A	18A
31A	28A	17A

31A	27A	16A
30T	29T	15T
28C	29C	15C
25C	28C	14C
7A_12T	28T	4A
12T_7G	29T	2G_2C
13T_7C	28T	4C
18T	28T	4T
18T	28T	4T
18T	28T	4T
18G	29G	4G
18A	27A	4A
17A	27A	4A
17A	26A	4A
7T_10C	26C	2T_2C
17G	58G	4G
16G	14G	4G
15G	15G	4G
15G	15G	4G
15G	31G	4G
15G	34G	4G
15G	34G	4G
15G	33G	4G
15G	33G	4G
14G	33G	4G
14G	32G	4G
8G	30G	1A_1G
8G	32G	3G
22A	33A	3A
30A	37A	8A
33T	38T	8T
51A	38A	9A
62T	41T	13T
63T	41T	13T
70T	42T	13T
72A	41A	14A
73T	43T	14T
74T	43T	14T
74T	44T	14T
74T	42T	14T
76A	43A	15A
77T	43T	15T
78A	44A	15A

79T	45T	15T
79T	45T	15T
80T	45T	15T
79A	44A	15A
81T	48T	15T
81T	48T	15T
82T	49T	15T
83A	50A	17A
84T	51T	17T
85T	52T	17T
86T	52T	17T
86A	52A	17A
87T	51T	17T
89T	51T	17T
87G	52G	17G
88G	52G	17G
88A	51A	18A
88C	51C	18C
88T	53T	18T
80T	54T	18T
86A	54A	17A
87T	54T	17T
88A	54A	17A
87T	55T	16T
87A	55A	16A
86C	55C	15C
87C	57C	16C
90G	61G	17G
90C	58C	17C
90C	59C	17C
82C	60C	17C
89C	62C	17C
90A	65A	16A
87T	65T	16T
86A	67A	16A
86G	67G	16G
85C	66C	16C
81G	64G	1A_12G_3C
83C	65C	15C
83T	65T	15T
81A	64A	15A
82C	64C	15C
82A	65A	16A

78A	63A	17A
75G	64G	19G
74C	62C	18C
71A	60A	17A
70C	58C	17C
70T	58T	18T
65C	58C	18C
63T	56T	19T
58C	57C	19C
56C	54C	19C
50G	55G	19G
49G	54G	19G
47G	53G	20G
46C	52C	19C
42G	53G	18G
45G	52G	17G
42T	49T	17T
41T	50T	17T
41T	43T	18T
39A	50A	17A
38C	48C	16C
37A	49A	17A
37A	49A	17A
38T	60T	18T
35T	59T	19T
35T	59T	19T
22A	53A	19A
23A	54A	19A
23T	55T	19T
23T	55T	19T
21A	55A	19A
24T	56T	19T
25A	57A	19A
26C	57C	18C
26A	58A	19A
26G	58G	19G
27G	62G	19G
28C	64C	20C
28T	64T	20T
28A	64A	21A
27C	64C	21C
23A	65A	22A
25C	65C	22C

26A	65A	21A
26T	67T	21T
26T	64T	21T
26G	64G	21G
31C	66C	21C
33C	67C	21C
34C	66C	20C
33C	66C	21C
32C	68C	21C
33C	68C	22C
32C	68C	21C
27A	64A	20A
26G	66G	18G
26C	66C	20C
26A	64A	20A
26A	61A	20A
26G	61G	20G
26C	61C	20C
27T	59T	19T
27G	61G	19G
27G	62G	20G
29G	64G	21G
30T	65T	21T
31A	63A	22A
31C	66C	22C
31T	68T	22T
31C	69C	21C
32A	69A	20A
33T	69T	18T
33T	71T	18T
33T	71T	18T
33T	70T	18T
33A	73A	14A
33C	74C	15C
35C	75C	15C
34G	74G	15G
34A	75A	15A
34C	74C	16C
35C	73C	16C
36T	71T	14T
35C	74C	14C
37G	74G	15G
37G	74G	15G

38A	75A	15A
38A	76A	14A
41G	76G	15G
45G	73G	14G
46A	75A	15A
47T	73T	16T
49G	73G	16G
48G	70G	15G
49A	53A_17G	15A
50A	71A	15A
53G	72G	15G
54G	72G	15G
53C	74C	15C
52T	74T	15T
56G	75G	15G
56A	72A	15A
56G	74G	15G
55T	73T	15T
56C	75C	13C
56A	75A	13A
55A	71A	14A
56C	71C	13C
57C	71C	13C
58T	73T	11T
57T	75T	11T
60G	76G	11G
60A	77A	13A
59G	76G	13G
59C	78C	13C
59C	81C	13C
28A_28G	62A_18G	13A
56G	85G	13G
57C	87C	14C
58T	88T	16T
58A	87A	16A
60C	87C	15C
62C	90C	14C
62T	92T	15T
62G	93G	16G
63G	91G	16G
63G	92G	17G
64A	96A	17A
64T	98T	16T

65T	98T	17T
65T	97T	16T
64G	97G	16G
65A	96A	16A
65A	96A	17A
63C	92C	17C
62C	95C	16C
61C	95C	16C
62C	96C	16C
62A	94A	16A
61G	93G	16G
60G	93G	16G
59T	90T	16T
62C	90C	16C
62G	91G	15G
63T	90T	16T
63G	88G	16G
64A	88A	16A
62G	86G	15G
61C	90C	15C
60A	69A	15A
61C	91C	14C
60A	92A	14A
60G	92G	14G
62T	91T	14T
60T	91T	14T
61T	93T	14T
60T	95T	14T
59A	96A	14A
56G	96G	13G
52C	98C	13C
52T	98T	13T
55T	98T	11T
55G	98G	11G
56C	100C	11C
55A	100A	10A
55G	96G	1A_8G_1C
53T	99T	10T
51A	101A	10A
52C	98C	10C
53A	101A	10A
51G	101G	10G
50C	102C	10C

49G	101G	10G
49T	101T	10T
48T	100T	10T
48T	99T	10T
47T	99T	9T
47A	98A	9A
45A	97A	9A
45C	96C	9C
45C	96C	9C
45A	93A	9A
46C	91C	7C
48T	93T	7T
49G	91G	7G
50C	88C	7C
47G	87G	7G
48C	83C	7C
48C	82C	6C
49A	81A	4A
49C	81C	4C
47G	82G	4G
44A	81A	2A_1C
44G	79G	3G
64G	76G	5G
63C	75C	6C
66T	76T	5T
66G	75G	4G
68C	74C	5C
65T	74T	6T
66C	75C	6C
69T	76T	7T
71T	75T	7T
73G	76G	10G
74C	76C	9C
74T	72T	10T
75G	76G	10G
72A	75A	10A
73G	74G	10G
73C	72C	10C
72A	73A	11A
73C	73C	11C
72C	75C	13C
73C	75C	13C
73A	76A	14A

74G	76G	13G
72G	76G	14G
72G	75G	14G
75G	68G	14G
74A	69A	14A
32T_44C	68T	15T
78G	66G	15G
78G	66G	15G
30T_45C	67C	16C
78G	67G	6A_10G
78G	65G	17G
78G	62G	16G
79C	63C	17C
83A	61A	19A
84A	60A	19A
81T	59T	19T
80G	59G	20G
77A	56A	18A
78G	57G	19G
42G_36C	58G	9G_10C
77G	57G	19G
79T	57T	18T
79C	58C	19C
76C	25T_34C	19C
76C	57C	19C
74A	55A	19A
74A	54A	19A
75G	55G	19G
79A	54A	19A
79G	56G	19G
81G	56G	20G
81G	54G	20G
80T	53T	21T
82C	54C	21C
80T	55T	22T
44A_35C	54A	10A_12C
78C	53C	22C
77A	52A	23A
76G	50G	24G
74G	49G	24G
73T	51T	24T
72A	51A	24A
71G	52G	25G

72G	54G	25G
70G	54G	25G
70G	55G	25G
71C	57C	24C
34T_39C	58T	25T
70C	59C	24C
70A	58A	25A
70C	57C	24C
66C	55C	25C
66C	58C	24C
67C	58C	26C
66C	57C	24C
67A	29A_28C	11A_13C
67A	57A	25A
64A	57A	25A
64A	56A	22A
65A	57A	23A
66A	58A	24A
62G	56G	24G
67C	59C	25C
67A	59A	26A
67A	59A	27A
68A	57A	28A
68T	60T	26T
68T	58T	26T
71T	58T	25T
69T	58T	26T
68C	58C	28C
69A	59A	28A
70T	61T	28T
71G	61G	29G
70C	63C	29C
71A	64A	29A
71G	64G	29G
74G	64G	30G
75T	63T	29T
74C	64C	31C
73T	64T	30T
75C	64C	30C
77T	62T	30T
76G	66G	28G
73A	68A	30A
75A	70A	30A

77A	68A	32A
74G	68G	32G
77A	68A	33A
77A	66A	33A
78A	67A	33A
76G	70G	33G
78T	70T	34T
80C	69C	34C
82C	70C	34C
83T	71T	34T
83A	71A	34A
84G	72G	35G
82C	36A_37C	36C
80T	72T	38T
80C	72C	40C
40T_39C	73C	40C
86A	74A	39A
89C	75C	40C
90A	76A	40A
90A	78A	40A
89G	79G	40G
93C	79C	39C
93G	78G	23A_15G
95A	79A	37A
98C	33T_44C	36C
98C	77C	39C
99T	78T	38T
99C	78C	40C
102A	78A	40A
101G	78G	41G
98A	76A	42A
100G	75G	43G
101T	76T	43T
101C	76C	43C
101A	76A_34T	43A
102G	75G	42G
101G	74G	41G
100G	73G	41G
98G	74G	40G
101C	71C	40C
103C	72C	39C
102G	72T	24T_16G
101C	75C	39C

101C	75C	40C
102T	72T	39T
106G	72G	39G
105G	72G	38G
105T	73T	37T
105T	72T	36T
104C	74C	35C
104C	70C	35C
104C	73C	36C
101T	76T	36T
101T	75T	36T
100T	75T	35T
96T	37T_39C	14T_1G_21C
96C	76C	36C
94A	75A	36A
91G	73G	36G
91A	72A	35A
90C	72C	36C
88A	71A	37A
86G	70G	37G
85G	69G	35G
85C	71C	35C
84C	75C	35C
84T	76T	35T
83A	70A	35A
82C	68C	34C
81C	69C	36C
80G	71G	33G
80G	71G	33G
79T	71T	32T
80T	73T	33T
79C	69C	34C
80A	69A	35A
48C	68C	34C
47C	70C	34C
45G	69G	34G
46A	67A	34A
46G	67G	34G
45A	67A	33A
43C	69C	33C
44T	69T	31T
45G	70G	29G
47G	68G	29G

47G	70G	29G
51G	69G	29G
52G	69A	28A
53A	69A	29A
55A	70A	29A
53A	72A	30A
53G	72G	30G
52T	72T	31T
51T	71T	32T
51A	71A	30A
50C	70C	31C
52T	69T	28T_20C
56T	71T	29T
56T	72T	29T
57T	70T	30T
55T	71T	29T
56T	73T	30T
57T	72T	31T
58T	73T	31T
77T	74T	31T
80T	74T	16T_15C
79C	75C	32C
81A	76A	32A
79T	78T	33T
78C	79C	34C
78C	80C	35C
80T	78T	35T
81C	77C	35C
84A	78A	33A
83T	81T	34T
84C	82C	36C
84C	82C	37C
88T	84T	37T
88C	84C	36C
89A	84A	36A
92C	82C	36C
95C	82C	35C
96A	82A	39A
95T	91T	38T
96C	91C	38C
96T	90T	38T
100T	90T	39T
101A	90A	39A

100G	91G	39G
100A	91A	37A
101A	92A	39A
106C	92C	36C
108T	92T	36T
107G	90G	37G
107C	89C	36C
106A	84A	37A
107G	84G	36G
109A	84A	35A
109G	82G	36G
107C	81C	36C
110T	35T_43C	22T_13C
111G	77G	34G
108G	76G	34G
112G	41A_34G	11A_22G
112A	73A	31A
112G	75G	32G
109G	74G	32G
109G	73G	32G
110A	73A	32A
110C	72C	32C
109C	71C	32C
112C	69C	31C
50A_62T	67T	31T
109A	67A	31A
106T	66T	31T
103G	64G	31G
99G	62G	31G
97A	61A	30A
97A	60A	29A
96C	78C	28C
100A	41A	15A
102C	41C	14C
100A	43A	12A
100T	42T	23T
99C	41C	24C
101C	40C	23C
99G	42G	22G
92A	40A	22A
95G	41G	23G
97C	40C	23C
96G	22A_17G	22G

96C	40C	21C
89T	37T	21T
98T	40T	21T
96G	40G	20G
98C	39C	20C
101C	22T_17C	12T_8C
102T	38T	20T
102T	38T	20T
102T	36T	20T
107C	38C	20C
103A	37A	20A
103C	38C	22C
101T	38T	22T
100T	37T	21T
102G	36G	20G
104C	35C	20C
104A	35A	19A
103T	33T	18T
102C	30C	18C
101T	13T	7T_3G_1C
98C	14C	10C
97C	12C	9C
97G	11G	10G
97T	11T	10T
97C	1A_1T_8C	3T_7C
89C	9C	10C
89C	9C	3T_7C
90C	9C	10C
89C	9C	10C
89C	8C	11C
86C	10C	11C
204C	10C	11C
32A_11C	10C	11C
38C	11C	11C
38C	10A_11C	27A_11C
34A	7A_3C	8A
81A	11A	8A
84A	12A	7A
85A	12A	6A
86A	12A	6A
84A	12A	7A
84A	13A	7A
85A	13A	13A_6C

83A	13C	1A
84C	13C	4A
85A	41A	27A
86A	43A	28A
84T	44T	29T
84C	45C	30C
84A	47A	30A
85A	47A	30A
84T	48T	30T
86A	50A	30A
87A	52A	32A
86G	52G	33G
84G	54G	33G
85A	54A	34A
86T	54T	35T
87A	54A	34A
88A	54A	35A
84T	56T	37T
86A	56A	37A
86A	56A	37A
84C	56C	38C
83A	56A	39A
80A	58A	38A
79C	59C	37C
82A	59A	37A
82T	59T	38T
83G	58G	37G
81C	39A_21C	22A_14C
80T	58T	36T
79G	60G	37G
78G	60G	38G
77T	58T	38T
78C	60C	38C
79G	61G	37G
77G	61G	35G
78C	61C	37C
76A	61A	37A
72G	63G	37G
69G	63G	35G
47C	61C	34C
45T	63T	33T
44C	62C	31C
44T	63T	31T

46G	61G	30G
46T	61T	30T
45G	63G	31G
45A	62A	30A
45A	60A	31A
44A	61A	31A
44A	60A	31A
44C	60C	30C
45G	60G	31G
46G	23T_35G	12T_19G
45G	60G	32G
49T	60T	32T
49G	60G	32G
49C	61C	14T_1G_20C
50G	60G	36G
48A	59A	39A
49T	61T	38T
50T	61T	38T
50C	63C	38C
50G	62G	13A_23G
50C	61C	37C
50C	59C	36C
52C	58C	34C
51C	58C	36C
53C	60C	36C
54T	61T	37T
53T	63T	36T
53C	65C	36C
53C	63C	36C
58C	65C	36C
55C	65C	36C
94G	64G	17A_21G
95C	65C	38C
95A	63A	38A
97G	64G	38G
99G	64G	39G
97G	62G	39G
96C	59C	40C
95A	60A	39A
97G	61G	40G
99G	61G	40G
99G	63G	39G
100G	64G	39G

100T	64T	39T
102C	62C	38C
105C	65C	38C
106A	65A	38A
106G	67G	38G
109A	70A	37A
112C	41G_28C	37G
113G	67G	37G
110T	65T	36T
112C	66C	37C
112C	66C	37C
113A	66A	38A
113G	66G	38G
111A	64A	37A
112G	63G	37G
113A	64A	37A
114G	64G	38G
113C	63C	40C
114A	27A_36C	40A
115C	63C	41C
112G	63G	41G
113G	62G	41G
116C	62C	41C
115T	62T	41T
113G	60G	42G
111G	61G	43G
114C	60C	43C
112T	61T	43T
111G	62G	44G
111T	59T	43T
112C	59C	43C
113A	61A	44A
112A	59A	42A
112G	60G	42G
110C	60C	42C
107C	60C	43C
107A	60A	45A
106G	58G	44G
105G	58G	45G
105C	59C	46C
105T	59T	43T
104G	58G	23A_18G
103G	60G	40G

106A	59A	42A
104G	57G	42G
105A	53A	44A
109C	57C	44C
110C	57C	44C
107G	57G	43G
99C	58C	43C
100C	23G_36C	42C
99G	58G	40G
98G	56G	39G
98C	54C	39C
99T	54T	38T
100C	55C	39C
95C	55C	40C
98C	53C	39C
51T_48C	54C	37C
98G	53G	36G
103C	56C	37C
100T	57T	38T
99C	56C	38C
100T	57T	37T
101A	57A	35A
101C	57C	35C
98C	54C	35C
97C	56C	34C
97C	54C	35C
97C	54C	35C
93T	55T	33T
94T	56T	35T
98T	56T	34T
96T	57T	35T
100T	55T	34T
97C	53C	35C
98T	53T	35T
98T	54T	34T
98C	55C	36C
94A	56A	35A
93A	57A	35A
91A	55A	34A
94T	57T	34T
94C	56C	34C
91A	57A	34A
91A	57A	33A

90G	57G	32G
93C	57C	31C
94A	58A	30A
90C	57C	31C
93C	59C	31C
92G	57G	30G
90C	58C	33C
89T	60T	34T
89T	61T	33T
92C	61C	34C
90C	62C	34C
93C	62C	34C
96A	64A	33A
95C	64C	33C
95A	65A	32A
95C	64C	33C
94G	65G	33G
94C	64C	34C
93A	66A	34A
97A	66A	33A
96A	67A	31A
97G	69G	32G
96G	71G	30G
101A	70A	31A
99A	68A	31A
102T	70T	33T
103G	69G	31G
99A	68A	28A
102G	70G	32G
104G	70G	30G
101G	68G	30G
103A	69A	28A
103G	68G	29G
102A	70A	29A
99T	70T	29T
103G	69G	29G
103T	68T	31T
104G	69G	32G
104C	68C	31C
104A	68A	32A
105A	66A	31A
104C	68C	31C
105T	69T	28T

104C	71C	29C
100A	68A	30A
99G	67G	31G
100C	68C	30C
100A	67A	32A
100G	68G	32G
101A	66A	33A
103G	69G	32G
103T	71T	30T
103C	74C	32C
103C	73C	33C
105G	74G	33G
104G	74G	31G
100T	37T_36C	14T_18C
101A	75A	33A
101G	76G	34G
101G	76G	35G
99G	75G	35G
97G	75G	35G
100A	72A	33A
98A	73A	33A
100C	74C	34C
100G	75G	36G
98C	73C	35C
98C	74C	35C
101A	74A	37A
102G	72G	37G
103A	72A	35A
102C	73C	35C
100C	71C	37C
101C	75C	38C
100A	76A	37A
103G	78G	39G
100G	77G	37G
103G	79G	35G
105C	82C	35C
103G	83G	35G
102T	84T	35T
101C	85C	36C
100C	81C	36C
99C	83C	37C
100C	82C	37C
101A	81A	36A

106G	81G	38G
104T	82T	37T
107C	79C	37C
106C	81C	39C
108G	82G	40G
105G	81G	39G
107C	79C	39C
103T	78T	39T
103C	80C	39C
103T	77T	40T
101T	78T	40T
102A	78A	41A
100T	78T	40T
99C	78C	40C
100T	78T	43T
100T	77T	43T
102C	77C	40C
105T	76T	43T
106C	74C	43C
103C	75C	42C
101C	75C	41C
41T_62C	76C	40C
104G	75G	41G
45T_62G	57G	1A_17T_24G
104T	57T	43T
104C	22T_33C	44C
103A	33A_21G_1C	45A
103A	31A_22C	47A
103C	54C	48C
105T	55T	46T
104C	56C	45C
105C	56C	44C
104T	56T	46T
103G	56G	47G
104G	68G	47G
103G	66G	45G
105A	65A	44A
109C	69C	46C
110A	72A	48A
110G	73G	49G
109A	73A	50A
108G	74G	51G
106A	72A	50A

106A	74A	50A
107T	77T	49T
107C	79C	49C
107T	79T	50T
110C	80C	51C
113G	81G	50G
115C	80C	49C
114C	84C	50C
116T	84T	51T
116G	84G	53G
116A	86A	53A
114T	88T	53T
113T	86T	53T
114C	88C	53C
115T	91T	51T
116T	90T	51T
117A	91A	52A
119A	90A	54A
118C	90C	55C
116A	87A	55A
118G	85G	56G
122A	86A	55A
121G	85G	55G
120T	84T	56T
120G	86G	56G
119T	86T	54T
120C	87C	57C
117T	87T	56T
116G	86G	55G
113T	86T	55T
113T	87T	55T
113G	86G	54G
113G	87G	54G
114G	86G	54G
114C	86C	54C
113C	86C	55C
114A	87A	53A
113G	87G	54G
111G	86G	53G
110G	89G	31A_22G
112G	87G	51G
110A	85A	51A
108G	88G	49G

105G	86G	49G
105G	86G	51G
108A	87A	50A
106A	91A	51A
107A	91A	52A
107A	91A	52A
106C	89C	51C
106T	89T	51T
108G	90G	51G
107C	90C	54C
112C	93C	55C
111C	95C	54C
117C	98C	53C
119G	100G	52G
122T	99T	51T
119T	100T	51T
119G	99G	49G
117T	95T	47T
116T	98T	48T
117T	97T	48T
117G	97G	48G
112C	94C	48C
112C	91C	46C
112T	92T	46T
113G	93G	45G
113A	95A	45A
113C	93C	45C
111A	92A	44A
110A	90A	44A
111A	90A	45A
111A	89A	47A
111G	88G	47G
107A	87A	48A
106T	88T	49T
104G	83G	46G
106C	83C	48C
104A	82A	45A
104G	81G	45G
105T	83T	45T
106G	82G	46G
107T	81T	46T
105C	82C	46C
105T	82T	46T

104C	79C	45C
100C	78C	42C
102T	78T	41T
103A	77A	43A
100A	75A	43A
97G	73G	44G
97A	70A	43A
95T	74T	44T
94G	75G	43G
95C	76C	44C
93C	76C	42C
92G	77G	42G
90T	79T	44T
91T	79T	44T
91G	79G	44G
89T	78T	42T
86G	75G	42G
86G	77G	40G
87A	77A	41A
87T	78T	41T
88A	76A	42A
65A_70T_82C	77C	40C
100A	78A	41A
101T	81T	43T
99A	81A	43A
98T	80T	44T
144A_82T	125A_81T	72A_78T
100T	79T	51T
37A_6T_22C	2A_54T_21C	41T
61T	78T	41T
62T	75T	39T
62T	78T	43T
64G	81G	44G
65C	84C	47C
66T	86T	47T
68C	86C	49C
71T	88T	49T
73C	87C	50C
74C	87C	50C
76C	87C	50C
76G	89G	50G
78G	90G	51G
83G	90G	49G

84A	91A	50A
83G	91G	50G
84A	93A	49A
87T	94T	47T
88A	94A	47A
85C	91C	47C
86G	94G	49G
84G	94G	48G
84T	92T	47T
83C	90C	47C
83C	89C	48C
81C	91C	47C
81T	89T	44T
83T	89T	45T
81C	87C	27G_19C
80C	88C	46C
79T	88T	46T
81C	89C	47C
79C	87C	46C
79G	87G	46G
80C	87C	48C
79C	87C	48C
78T	89T	50T
82T	89T	49T
83G	87G	48G
83T	86T	48T
84A	84A	46A
84A	84A	46A
82G	83G	47G
82A	84A	47A
83A	85A	47A
83T	86T	45T
82G	85G	45G
79C	83C	44C
79A	85A	45A
78G	82G	46G
78A	79A	46A
77T	76T	44T
77G	77G	42G
83G	79G	43G
81G	77G	43G
75G	32A_44G	32A_11G
85A	77A	44A

83A	78A	44A
83A	77A	44A
87A	78A	45A
91A	76A	46A
89A	76A	45A
89G	71G	46G
90A	70A	47A
90A	70A	45A
90G	71G	47G
91G	71G	47G
91G	72G	45G
91A	73A	44A
88G	74G	43G
86A	73A	44A
47T_40C	40T_1G_33C	16T_30C
94G	71G	45G
96G	68G	46G
97A	64A	45A
98C	66C	43C
99T	65T	40T
98C	64C	41C
94C	66C	41C
48G_47C	67C	40C
98T	67T	40T
96T	62T	40T
97T	62T	39T
96A	61A	39A
91A	60A	39A
92C	63C	41C
96C	61C	40C
96C	60C	41C
95C	60C	40C
92C	60C	42C
92T	57T	42T
90G	57G	41G
42G_47C	37G_19C	42G
89G	55G	41G
92G	1A_18T_35G	42G
92A	54A	42A
46T_47C	36T_18C	43T
99A	65A	43A
99A	65A	42A
99G	66G	40G

99A	68A	40A
99A	71A	40A
99A	73A	40A
100G	74G	43G
101A	73A	42A
100A	75A	42A
102G	74G	39G
104G	77G	41G
104T	78T	43T
103G	76G	41G
105C	77C	44C
104T	79T	47T
107T	81T	46T
108C	79C	48C
54A_50G	40A_39C	49A
105T	78T	48T
56T_51C	78T	49T
108C	80C	50C
108T	77T	50T
109C	76C	49C
108C	78C	48C
107T	78T	50T
108C	77C	50C
109T	76T	50T
111C	76C	50C
54T_47C	38T_39C	50T
102G	79G	48G
104G	77G	51G
103G	77G	52G
102T	77T	50T
105T	77T	51T
105T	77T	51T
106T	78T	49T
104T	79T	47T
103C	77C	49C
103T	77T	49T
103A	78A	49A
103C	78C	49C
99A	78A	49A
96G	77G	48G
89A	39A_39T	48A
89A	76A	49A
89G	77G	49G

89G	77G	47G
91C	77C	47C
94G	76G	46G
92C	39G_37C	47C
93C	77C	49C
94A	77A	50A
91A	76A	51A
41T_51G	78G	50G
93A	78A	50A
94T	78T	53T
94G	75G	53G
93C	75C	52C
94G	78G	51G
91T	80T	51T
93T	82T	53T
93T	85T	53T
95G	84G	53G
95G	84G	53G
98A	87A	54A
99G	82G	53G
96A	80A	51A
92C	82C	56C
92C	83C	55C
94C	84C	55C
93T	85T	55T
92G	86G	56G
94G	86G	56G
95T	84T	57T
96C	82C	36A_22C
96G	80G	57G
96T	82T	57T
96G	83G	59G
98T	83T	61T
97T	84T	60T
95T	81T	58T
90T	81T	57T
90G	81G	57G
86T	80T	56T
37T_47C	77T	54T
45A_37C	77C	52C
83A	77A	52A
85G	77G	50G
88G	79G	49G

88C	80C	49C
88G	80G	49G
89G	80G	50G
88T	79T	50T
91C	80C	50C
93C	80C	50C
90C	80C	51C
46T_43C	79C	29G_22C
88G	77G	50G
88C	77C	50C
86C	74C	50C
84G	76G	45G
84G	75G	46G
82G	74G	46G
78G	73G	45G
79A	74A	45A
79T	73T	45T
80C	72C	45C
80A	72A	44A
79A	70A	44A
79G	69G	45G
78A	68A	45A
78C	67C	44C
78C	67C	44C
77G	65G	44G
76A	67A	43A
75G	66G	41G
76C	36G_27C	43C
74A	62A	43A
72G	62G	43G
70A	61A	41A
69G	61G	40G
68C	60C	39C
67A	59A	38A
63G	58G	38G
62G	57G	38G
61T	57T	34T
61G	57G	36G
61G	57G	37G
59G	52G	37G
57T	49T	35T
55T	47T	33T
54G	45G	32G

50T	45T	31T
51T	44T	31T
49G	41G	30G
48T	41T	29T
50G	41G	28G
52A	39A	27A
51C	38C	27C
50C	37C	26C
49G	35G	26G
51T	36T	27T
50G	34G	25G
48C	35C	24C
46G	34G	22G
44C	33C	22C
44C	32C	19C
44G	32G	11A_6G
41A	31A	15A
41G	30G	15G
40A	29A	14A
40A	29A	14A
40A	29A	14A
39G	29G	15G
37A	27A	15A
37T	27T	14T
36C	26C	15C
30T	26T	15T
25C	24C	15C
23C	23C	15C
22C	21C	14C
10A_32C	21C	12C
21C	18A_54C	11C
15C	4A_9C	8A
10T_5C	13C	8C
15C	13C	8C
15C	13C	8C
19A_57C	13C	8C
15C	25C	9C
17C	25A_16C	9C
14A_8C	35A	15A
36A	35A	15A
36A	37A	16A
37A	36A	16A
37A	37A	17A

38A	39A	17A
38A	42A	18A
38T	46T	18T
38G	48G	18G
39C	50C	18C
39A	52A	18A
39G	52G	18G
40T	51T	17T
40C	53C	18C
41C	54C	17C
43A	57A	19A
45A	62A	18A
45A	62A	19A
47G	62G	21G
47T	63T	21T
49T	61T	21T
48C	62C	21C
51C	63C	21C
54A	65A	21A
54C	66C	23C
55C	66C	26C
55T	66T	25T
54G	66G	24G
54G	68G	24G
55C	68C	25C
52C	69C	25C
53A	70A	26A
54G	70G	26G
54G	73G	26G
56G	73G	27G
53G	74G	27G
52C	74C	27C
52T	73T	27T
54A	73A	27A
54C	74C	28C
53A	74A	30A
53G	72G	30G
55G	70G	30G
55G	71G	30G
57C	73C	29C
59C	72C	29C
58T	72T	29T
57A	70A	29A

60C	71C	30C
59T	71T	31T
60T	71T	32T
62T	71T	32T
63A	71A	34A
63C	72C	35C
68A	72A	34A
68G	72G	34G
67G	73G	34G
65G	74G	34G
65C	75C	34C
67T	73T	36T
68G	72G	36G
69T	70T	36T
72T	70T	37T
74G	70G	37G
76T	71T	36T
77G	70G	37G
77C	69C	36C
76A	70A	36A
72A	70A	36A
68A	71A	37A
70T	71T	37T
69A	71A	36A
70G	69G	36G
29T_40C	70C	36C
69C	71C	36C
67A	70A	36A
69C	68C	35C
70A	66A	36A
70A	65A	36A
72C	65C	34C
73A	63A	36A
30A_45T	61A	37A
74G	63G	37G
75G	63G	39G
75C	63C	39C
74A	61A	38A
73T	57T	38T
74G	56G	37G
74T	57T	35T
74G	57G	35G
72C	57C	37C

73C	56C	38C
74C	57C	38C
71A	56A	37A
70A	54A	36A
70C	55C	33C
29A_42G	29A_26G	33G
72C	55C	33C
74C	53C	33C
74A	54A	30A
73C	56C	32C
72A	56A	31A
71A	57A	33A
70A	56A	36A
71C	57C	19T_17C
70G	55G	35G
72C	55C	37C
74T	54T	35T
73C	54C	37C
74T	55T	38T
76T	55T	36T
80G	56G	36G
79T	58T	35T
79A	59A	36A
79C	57C	37C
77G	59G	37G
76A	59A	37A
77T	35T_25C	18T_19C
76G	59G	36G
77G	59G	36G
77C	58C	35C
79T	59T	34T
78T	57T	34T
79T	56T	33T
75T	54T	31T
75C	57C	34C
75A	56A	35A
75G	56G	35G
74G	57G	36G
74C	56C	34C
73C	57C	34C
72T	59T	33T
71T	59T	17T_16C
70G	59G	33G

69T	60T	34T
67G	60G	31G
66G	61G	33G
64G	61G	34G
65T	60T	32T
65C	61C	34C
64T	60T	35T
65G	60G	35G
63G	60G	35G
64G	60G	35G
64C	60C	35C
64A	59A	35A
63G	59G	35G
63C	59C	33C
62C	61C	35C
60C	59C	34C
59A	60A	34A
57T	61T	34T
57A	59A	36A
56C	60C	33C
55A	61A	36A
56A	60A	36A
56C	59C	35C
55G	59G	35G
53C	60C	35C
53T	59T	35T
53A	59A	35A
51G	59G	34G
50C	58C	33C
50T	57T	32T
49C	57C	37C
50C	57C	38C
49G	57G	37G
49G	56G	35G
48A	56A	38A
47T	55T	38T
47T	52T	38T
48C	51C	40C
48T	49T	37T
49G	47G	36G
48A	46A	34A
48G	47G	34G
46A	48A	34A

45G	49G	35G
46T	51T	35T
46G	50G	33G
44C	50C	33C
41T	51T	32T
14A_40T	20A_49T	33T
40A	51A	33A
38A	51A	34A
39A	50A	35A
40A	51A	35A
41A	51A	38A
41A	53A	55A
40A	53A	24A
39A	55A	24A
34G	56G	21A
35A	57A	33A
37A	58A	32A
38A	57A	32A
41A	54A	31A
42G	55G	31G
42A	54A	19A_12G
42A	52A	29A
45A	50A	31A
45T	48T	31T
44A	49A	31A
19A_28T	48T	32T
47T	48T	33T
46A	47A	32A
49C	47C	31C
49C	48C	31C
49A	48A	31A
52A	47A	31A
52A	47A	16A_14G
52T	47T	30T
54T	47T	29T
57C	47C	31C
58T	49T	31T
58A	50A	30A
59A	50A	32A
59T	50T	32T
62C	48C	32C
63T	48T	31T
63A	48A	32A

68A	49A	32A
69C	47C	31C
69A	50A	32A
71C	49C	31C
71C	49C	30C
71A	49A	30A
73A	49A	29A
73G	46G	29G
75A	46A	29A
77C	48C	30C
78G	49G	29G
78G	49G	30G
77T	49T	30T
79G	49G	32G
79C	49C	31C
79C	51C	30C
78C	51C	30C
79G	52G	28G
80G	52G	26G
79A	52A	27A
79G	54G	28G
79T	53T	27T
78T	54T	27T
79G	57G	28G
78G	56G	28G
79C	56C	28C
82G	52G	29G
81C	50C	30C
78T	50T	28T
79G	50G	30G
78A	27A_24G	28A
78T	53T	30T
78T	53T	30T
79C	53C	29C
79C	54C	28C
78T	53T	27T
81G	28G_24C	27G
79G	50G	27G
79C	49C	26C
79A	49A	25A
80C	49C	14A_13C
36A_44G	48G	27G
79G	47G	27G

76G	46G	27G
75C	46C	27C
73A	45A	27A
72T	44T	26T
70C	45C	26C
70T	47T	28T
70A	46A	28A
70A	47A	28A
71A	47A	28A
68G	47G	27G
68G	47G	28G
69A	47A	29A
67A	47A	30A
68T	45T	29T
67C	46C	31C
65A	45A	32A
65A	44A	32A
64A	45A	31A
61G	44G	31G
58G	44G	29G
57T	42T	28T
57C	40C	28C
56A	39A	27A
54C	39C	27C
52T	38T	25T
51C	36C	26C
51A	35A	25A
46T	34T	25T
45T	33T	25T
44T	32T	24T
43C	30C	24C
41T	30T	24T
40G	30G	24G
38T	30T	23T
38G	30G	23G
34C	30C	23C
34T	29T	21T
33C	28C	21C
32T	27T	20T
80A_48T_60G_96C	55A_28T_35G_56C	47A_28T_35G_56C
19G	19G	11G
18T	19T	11T
null	1C	null

null	null	null
null	null	null
null	null	null
null	null	null
null	null	null
null	null	null
null	null	null
null	null	null
null	null	null
null	null	null
null	null	null
null	null	null
null	null	null
null	null	null
1C	null	1C
2T	null	1T
2C	1C	1C
4T	3T	1T
33A	23A	11A
33G	24G	11G
33T	25T	12T
35C	25C	12C
36G	25G	11G
37T	25T	13T
37T	26T	13T
39T	28T	13T
39C	29C	14C
39C	29C	14C
39C	30C	15C
41C	31C	15C
41A	32A	16A
41G	32G	16G
41C	32C	15C
41T	32T	16T
42C	32C	16C
43T	32T	18T
43G	31G	18G
44A	32A	18A
44C	34C	18C
45G	16A_18G	19G
45C	34C	19C
46C	34C	20C
47T	34T	20T

49G	35G	20G
49G	34G	20G
51C	34C	19C
50A	34A	17A
49A	35A	18A
51C	36C	18C
52T	36T	18T
55C	20T_17C	18C
55T	37T	18T
56G	38G	18G
58C	39C	18C
58C	39C	17C
55C	41C	18C
56C	40C	18C
54A	40A	19A
55C	40C	19C
56G	39G	18G
56G	39G	17G
55C	39C	16C
54T	40T	16T
55C	41C	15C
55T	41T	15T
54G	40G	15G
59C	39C	13C
59C	39C	14C
59A	38A	13A
60C	38C	13C
61C	38C	13C
63C	37C	13C
64T	36T	13T
64T	35T	15T
64G	35G	16G
61C	35C	17C
60C	35C	17C
57A	35A	16A
56A	35A	16A
55A	35A	16A
54G	35G	16G
57G	37G	16G
58C	36C	16C
57A	35A	16A
57A	35A	15A
56C	35C	16C

56T	34T	15T
54G	32G	18G
53A	28A	19A
53G	28G	19G
53C	27C	18C
52A	27A	18A
52T	27T	18T
22T_29C	26T	17T
51C	26C	17C
49C	23C	18C
49T	23T	17T
49T	23T	17T
49C	22C	14C
48C	20C	16C
48C	20C	15C
48T	20T	15T
48C	20C	14C
48C	19C	16C
47A	21A	15A
47C	23C	14C
47T	23T	13T
47T	23T	14T
47C	21C	14C
48T	22T	13T
48C	23C	14C
48A	24A	13A
47G	24G	12G
45C	23C	13C
45C	23C	13C
46C	23C	13C
46T	22T	13T
47C	22C	13C
45T	21T	13T
44G	20G	13G
41G	21C	3G_10C
41T	22T	14T
40C	21C	15C
38T	19T	15T
38G	18G	15G
38G	16G	16G
36A	16A	16A
37A	16A	15A
35A	16A	15A

35T	16T	16T
35C	16C	14C
35A	15A	15A
35T	9T_5G	10T_2G_1C
36C	9G_3C	7G_6C
36C	13C	13C
36C	13C	13C
30C	13C	13C
31C	11C	12C
30C	13C	13C
30C	13C	2T_1G_10C
29C	13C	13C
26C	12C	12C
26A	12A	12A
26C	12C	12C
26T	12T	12T
26T	12T	11T
26C	12C	11C
27A	12A	11A
26A	12A	10A
25C	12C	11C
23C	12C	10C
23C	13C	11C
23A	12A	11A
22T	12T	11T
22C	12C	11C
21A	12A	10A
12T_9G	12G	10G
21C	12C	9C
20C	12C	7C
20T_44C	12T_47C	7T_1G_17C
21C	12C	7C
20C	12C	7C
20C	12C	7C
19C	12C	7C
19C	12C	7C
19C	12C	7C
18G	12G	7G
18C	12C	7C
18T	12T	7T
19T	12T	7T
19C	12C	6C
19A	12A	6A

20A	9A	5A
19A	9A	6A
19C	9C	6C
18A	9A	5A
17C	8C	5C
16T	7T	5T
15G	6G	5G
15C	6C	5C
15C	6C	5C
14A	6A	5A
15C	6C	5C
14C	6C	5C
14A	6A	5A
13T	6T	5T
13C	6C	5C
13T	5T	4T
13G	4G	5G
12T	4T	5T
12A	4A	4A
12T	4T	4T
12T	4T	4T
13C	4C	3C
11T	3T	3T
12C	3C	3C
12T	3T	3T
10C	3C	3C
11A	3A	3A
11C	3C	3C
11G	3G	3G
10C	3C	3C
11A	3A	3A
11C	3C	3C
10C	2C	3C
8A	2A	3A
9T	2C	2T_2C
8G	1G	3G
8G	1G	3G
8A	1A	3A
8G	1G	3G
8T	1C	3T
8C	1C	7C
8C	2C	1C
8C	3C	1C

7C	3C	3C
7C	3C	3C
7C	2C	3C
7C	2C	3C
7C	2C	3C
6C	2C	3C
8C	2C	3C
8C	2C	3C
8C	2C	3C
6G	2G	3G
6T	2T	3T
7T	2T	3T
7C	2C	4C
8A	2A	4A_1T
8C	3C	5C
8T	4T	5T
8A	5A	6A
8A	5A	5A
8C	7C	6C
10A	7A	7A
10C	7C	7C
10T	7T	8T
11T	7T	8T
11C	9C	8C
12C	10C	8C
11A	10A	8A
13T	10T	9T
13A	11A	9A
11G	12G	8G
12C	12C	9C
4A_9G	4A_8G	7A_2G
13C	13C	9C
13A	13A	9A
13A	14A	9A
14C	14C	9C
14A	14A	10A
14A	14A	10A
16A	14A	10A
16A	14A	10A
16G	14G	10G
16A	14A	11A
19G	14G	11G
21A	14A	11A

24G	14G	11G
24C	14C	12C
24C	15C	12C
25G	15G	12G
27G	15G	12G
27G	15G	12G
26A	15A	12A
26T	9T_6G	11T
26G	15G	12G
15A_11T_1G	16A	12A
27T	16T	12T
27G	15G	14G
29A	15A	13A
29T	15T	13T
29G	15G	13G
28G	15G	13G
28A	16A	14A
28C	16C	15C
28A	17A	14A
29A	16A	14A
30T	16T	14T
32G	16G	15G
32G	18G	15G
33A	18A	15A
33G	18G	15G
33A	18A	14A
33T	19T	15T
33G	19G	15G
34C	19C	15C
34A	19A	15A
34T	19T	16T
34C	21C	16C
34A	21A	17A
34T	22T	16T
35A	22A	17A
35C	22C	18C
35A	23A	18A
36C	23C	18C
35A	24A	18A
36C	24C	18C
36G	24G	17G
36C	23C	18C
36A	22A	18A

36C	22C	17C
36T	23T	17T
36C	21C	17C
34C	21C	16C
34G	22G	16G
34A	22A	14A
34G	23G	15G
34C	21C	15C
33A	20A	15A
33C	20C	15C
32C	20C	15C
33C	21C	16C
33C	20C	16C
33T	20T	16T
32T	22T	18T
33A	22A	18A
34C	22C	18C
35A	21A	18A
35T	21T	18T
36A	21A	18A
36C	22C	18C
35A	22A	19A
35C	23C	19C
36A	23A	19A
37T	23T	18T
34A	23A	18A
32C	10T_13C	9T_9C
29C	24C	18C
29C	25C	17C
29T	24T	9T_8C
29T	25T	17T
27C	26C	18C
28C	28C	18C
28A	28A	18A
28C	28C	17C
29A	28A	18A
29C	27C	18C
29T	27T	18T
31T	27T	16T
29C	28C	16C
29T	28T	16T
30A	29A	16A
30C	29C	16C

30T	28T	17T
30G	29G	16G
30C	30C	16C
30C	30C	16C
30A	31A	16A
29T	33T	15T
29T	30T	15T
28G	34G	15G
29T	36T	16T
30G	36G	17G
30T	35T	18T
29G	35G	17G
31A	34A	9A_1G_8C
32G	35G	19G
32G	35G	19G
32C	33C	19C
32T	32T	19T
32C	32C	18C
31T	32T	18T
31T	31T	19T
31C	31C	20C
30T	30T	20T
32A	30A	20A
30A	30A	19A
30T	30T	20T
30G	30G	19G
30G	29G	17G
31A	30A	18A
31A	29A	21A
34A	30A	21A
34G	32G	21G
35A	31A	20A
38A	32A	21A
37G	30G	21G
38A	30A	14A_1T_7G
39C	31C	23C
39A	32A	23A
40G	32G	23G
40A	32A	20A
40T	32T	23T
40G	33G	23G
41T	32T	20T
40G	31G	21G

39G	31G	22G
39T	30T	21T
38G	31G	21G
38C	32C	21C
38T	31T	21T
39A	32A	20A
39A	31A	21A
38A	32A	20A
37A	32A	21A
37T	32T	21T
41A	34A	21A
42G	32G	21G
42T	32T	22T
43A	32A	22A
42A	32A	21A
43A	31A	21A
43C	29C	20C
45C	29C	21C
45A	30A	21A
45C	31C	21C
45A	31A	22A
46T	31T	23T
43G	32G	22G
43A	31A	23A
44T	32T	23T
44C	32C	24C
44C	32C	25C
45C	33C	25C
47C	34C	26C
49C	34C	26C
49G	36G	27G
48G	34G	15G_12C
47G	33G	28G
48G	34G	27G
51G	31G	28G
19A_33C	29A	30A
51G	30G	30G
53A	31A	29A
56A	31A	29A
58A	31A	29A
62A	31A	29A
63A	31A	27A
63G	32G	27G

63G	33G	26G
62A	34A	26A
62A	34A	26A
62G	34G	29A_24G
61A	34A	12A_1T_8G
64G	34G	8A_13G
64A	34A	21A_8G
62A	36A	24A
64A	36A	11A_14G
67A	37A	27A
69A	39A	27A
69A	38A	27A
69A	38A	27A
68A	38A	26A
69A	36A	27A
68G	37G	27G
66G	37G	28G
66A	39A	28A
65A	40A	28A
65T	40T	28T
66C	42C	27C
66A	42A	27A
66A	42A	27A
66G	42G	26G
67C	41C	24C
67A	43A	25A
66A	41A	25A
67T	43T	25T
69C	44C	24C
69A	45A	25A
67A	44A	26A
70G	45G	25G
70C	43C	26C
70A	44A	25A
69A	43A	26A
70T	43T	28T
70G	43G	27G
67C	43C	27C
68T	43T	27T
68T	44T	27T
68A	44A	26A
67T	45T	26T
67G	45G	26G

68G	46G	27G
68A	45A	28A
67C	46C	29C
67C	45C	27C
35A_31G	45A	27A
67A	45A	27A
67G	45G	27G
65G	44G	25G
66A	44A	23A
65A	44A	23A
67G	45G	22G
67A	44A	20A
67A	45A	20A
64A	43A	20A
63A	41A	19A
67G	41G	18G
67A	41A	18A
66A	40A	17A
65A	40A	19A
62G	40G	18G
62A	40A	18A
60A	40A	18A
61G	39G	18G
58G	40G	18G
54T	39T	18T
54T	40T	17T
53C	39C	18C
54T	41T	18T
54G	40G	18G
55G	40G	16G
54G	40G	17G
53G	40G	17G
52A	40A	16A
53G	39G	17G
53G	39G	15G
51A	39A	15A
43A_30G	19A_36G	15G
51G	22G	16G
52G	39G	17G
52G	40G	17G
50G	41G	17G
49G	42G	16G
50G	42G	16G

46G	37G	15G
49T	40T	16T
49T	38T	16T
52G	44G	17G
53G	45G	16G
54G	45G	17G
57G	45G	19G
56A	47A	18A
56G	47G	20G
58A	50A	21A
58G	49G	23G
57G	49G	23G
58T	51T	23T
58T	53T	24T
62T	51T	25T
64T	51T	25T
63A	51A	25A
67T	54T	27T
68G	55G	26G
69G	55G	25G
68C	55C	27C
68A	55A	28A
69T	57T	28T
70C	56C	28C
70T	57T	28T
69A	54A	28A
69T	55T	29T
68A	54A	29A
72G	56G	28G
71A	57A	28A
72G	58G	29G
70A	57A	29A
73G	58G	29G
73A	57A	29A
74G	59G	30G
76G	58G	29G
76A	59A	29A
78A	59A	33A
77G	60G	33G
79G	60G	32G
79C	60C	34C
77T	60T	34T
82A	61A	35A

81T	62T	37T
82G	62G	37G
83G	62G	36G
84G	62G	36G
82A	64A	36A
82T	62T	37T
80G	63G	38G
78G	64G	37G
78G	64G	37G
79A	64A	37A
77G	65G	39G
81T	64T	39T
80T	64T	40T
83C	61C	43C
86A	62A	44A
87A	62A	44A
89G	64G	43G
91A	66A	45A
93G	69G	45G
95A	72A	43A
96C	72C	45C
94T	74T	45T
94T	73T	46T
93G	73G	46G
94A	72A	46A
94G	71G	48G
94A	70A	46A
97A	71A	50A
98C	72C	50C
99A	72A	50A
100T	74T	52T
98T	74T	54T
96G	74G	53G
95A	75A	51A
97G	74G	53G
99T	72T	57T
100T	70T	57T
100G	70G	55G
98T	71T	55T
100T	70T	55T
101G	69G	56G
98C	67C	53C
98C	70C	55C

97A	70A	55A
93T	69T	53T
92T	68T	54T
91T	67T	53T
91T	69T	54T
90C	68C	53C
93A	66A	54A
94G	66G	54G
96G	66G	56G
97G	70G	54G
95A	69A	55A
98A	72A	53A
95T	71T	55T
95A	69A	54A
98A	70A	52A
99T	71T	53T
97C	74C	51C
96C	75C	52C
94A	74G	17A_34G
93A	74A	48A
90A	74A	51A
89G	73G	50G
89A	73A	50A
87G	74G	50G
89C	75C	51C
44T_43G_175C	74C	53C
101G	73G	52G
101C	72C	50C
100C	72C	50C
99T	75T	49T
94C	76C	50C
97G	42A_34G	50G
99G	76G	49G
105A	75A	48A
105T	78T	46T
106A	80A	48A
106A	80A	48A
104A	80A	46A
101G	81G	48G
100C	83C	47C
100A	45A_37G	18A_27G
99G	80G	44G
98C	79C	44C

98T	79T	45T
99C	77C	42C
100C	74C	44C
59T_38C	71C	46C
97C	73C	45C
99A	72A	44A
99T	73T	42T
56T_41C	74C	42C
98G	73G	41G
99T	74T	41T
99G	75G	43G
97G	76G	39G
99C	74C	40C
100T	75T	38T
101C	73C	38C
100A	73A	37A
100T	71T	37T
101G	69G	36G
102A	71A	36A
99T	74T	32T
98T	77T	32T
100C	79C	32C
100A	77A	31A
100C	77C	31C
101A	77A	32A
102C	78C	33C
105A	79A	33A
105A	36A_42C	34A
106C	78C	36C
106T	78T	37T
106T	78T	36T
107C	76C	35C
105C	78C	34C
103C	79C	34C
102T	81T	33T
100G	80G	32G
100T	77T	33T
99A	78A	33A
97C	74C	34C
96T	75T	33T
48T_46C	37T_39C	33T
91T	77T	34T
93G	77G	34G

92C	74C	33C
94A	73A	36A
97T	74T	36T
98T	77T	37T
98G	78G	37G
102T	80T	38T
102G	79G	38G
100T	78T	40T
99T	77T	38T
99T	80T	38T
100G	81G	39G
99C	82C	38C
92C	82C	39C
53T_43C	80T	38T
103G	81G	39G
105G	80G	38G
103G	81G	40G
102C	83C	39C
102A	79A	40A
102G	78G	40G
102C	80C	41C
105A	79A	41A
106A	79A	40A
107A	78A	43A
107C	79C	42C
104C	79C	43C
103T	79T	43T
103G	38T_39G	42G
101G	77G	41G
99G	77G	43G
99C	78C	41C
101G	74G	41G
101G	74G	42G
100G	74G	42G
100G	73G	41G
178A_80T	99A_26T	71A_34T
110A	76A	45A
113T	76T	45T
110A	78A	45A
111T	79T	47T
109A	79A	47A
59T_44G	31T_39G	43T
103A	69A	43A

103C	70C	43C
103T	71T	43T
104C	68C	41C
106C	68C	43C
108T	67T	45T
110G	67G	48G
109C	69C	49C
108T	67T	49T
107G	66G	49G
108T	66T	50T
107T	66T	47T
106G	66G	48G
106C	65C	47C
106C	67C	46C
106A	65A	47A
110T	64T	50T
110T	63T	49T
112T	67T	49T
111T	69T	50T
115A	72A	49A
116C	72C	49C
118A	71A	49A
116G	72G	47G
120A	73A	49A
119T	72T	50T
121T	70T	52T
121A	72A	52A
122A	71A	53A
119T	70T	53T
117T	70T	54T
117A	67A	56A
119A	67A	57A
123C	68C	57C
123A	68A	58A
122G	71G	57G
121A	70A	55A
124G	68G	58G
122G	69G	57G
120T	69T	58T
122G	70G	58G
121G	72G	57G
123A	71A	56A
123G	70G	56G

125G	70G	54G
125G	69G	56G
123C	70C	55C
122A	68A	56A
123A	69A	56A
123A	69A	54A
124T	69T	53T
124A	70A	54A
127G	70G	54G
126A	70A	53A
126C	71C	53C
57T_70C	71C	54C
127G	72G	54G
125A	72A	54A
122T	72T	53T
120C	72C	55C
121A	72A	54A
123A	70A	54A
124A	68A	59A
126G	69G	60G
122C	73C	61C
119C	74C	60C
124C	74C	61C
123A	72A	60A
123A	73A	60A
125G	72G	59G
126C	75C	60C
126C	49T_26C	60C
126A	76A	61A
127C	77C	61C
59T_68C	79C	61C
125G	79G	58G
124A	78A	27A_1T_28G
126G	81G	54G
126C	82C	55C
128C	81C	55C
126A	81A	53A
127G	33G_48C	53G
130A	82A	53A
130A	83A	54A
130G	85G	54G
134G	87G	57G
132C	88C	55C

131T	88T	55T
130G	85G	55G
130C	82C	56C
130A	80A	58A
129A	80A	60A
131G	80G	61G
134G	80G	62G
128G	77G	61G
128A	76A	60A
127A	76A	58A
124G	73G	55G
123C	73C	55C
123A	72A	53A
127A	71A	52A
124G	73G	49G
123T	73T	47T
120C	71C	23T_1G_20C
120G	71G	45G
118G	68G	45G
112C	68C	47C
112T	68T	44T
115T	66T	45T
112C	67C	45C
110T	67T	43T
109C	65C	45C
108T	65T	45T
108G	66G	45G
109C	73C	45C
110T	73T	48T
109C	72C	48C
109T	73T	48T
107A	71A	48A
106G	71G	48G
107A	71A	46A
106C	72C	48C
104A	71A	48A
105A	74A	49A
104G	73G	49G
107G	75G	44G
106T	73T	47T
108A	72A	47A
110T	74T	46T
107A	74A	44A

106T	74T	45T
106A	74A	45A
105G	73G	42G
102A	73A	41A
101A	69A	42A
99G	67G	41G
96A	64A	40A
95G	64G	41G
93G	63G	41G
92A	62A	36A
90G	60G	39G
89G	59G	39G
89G	58G	39G
57T_31C	34T_23C	39C
86G	55G	41G
86G	55G	42G
86T	55T	42T
86G	51G	41G
85G	50G	40G
80T	50T	39T
81T	50T	37T
52G	12A_28G	32G
56G	40G	35G
44T_82G	40T_58G	37G
61G	35G	36G
51T_58G	20T_15G	34T_51G_1C
66G	25T_60G	34G
24T_45G_1C	22T_24G	1A_21T_17G
96G	65G	42G
93T	66T	41T
96G	67G	44G
98G	67G	42G
100C	72C	43C
99A	73A	41A
105C	73C	43C
105C	74C	44C
106T	75T	45T
105C	77C	45C
103T	79T	45T
105T	78T	47T
106C	81C	46C
105C	82C	46C
106T	83T	48T

107A	85A	48A
107G	86G	51G
110C	87C	50C
108C	85C	50C
109A	84A	49A
109A	85A	47A
109A	85A	48A
108A	86A	48A
107T	79T	46T
108T	80T	45T
107G	81G	27A_18G
105G	81G	43G
107T	83T	43T
107T	84T	44T
107C	85C	45C
105T	85T	46T
105G	85G	46G
104T	83T	44T
104T	83T	45T
102G	81G	45G
45T_54C	84C	48C
47G_51C	86C	17G_31C
100A	84A	46A
100G	85G	48G
101A	85A	44A
99G	87G	47G
99C	88C	46C
100A	88A	44A
99G	88G	42G
101C	87C	44C
102C	87C	46C
101A	86A	45A
99A	85A	45A
99C	84C	46C
95C	86C	45C
94A	85A	45A
95G	84G	46G
96A	84A	45A
96T	85T	46T
94G	82G	49G
93C	83C	49C
91C	82C	50C
92C	83C	50C

87A	84A	53A
86C	80C	51C
42A_43G	81G	52G
85G	80G	53G
85G	83G	52G
82A	82A	52A
81T	84T	52T
82G	83G	51G
79T	82T	51T
80T	80T	53T
80C	80C	53C
81C	79C	53C
82T	79T	52T
86A	76A	53A
87A	79A	50A
88G	80G	27T_23G
88C	80C	52C
89A	80A	52A
86G	81G	53G
89G	80G	53G
89A	78A	53A
88T	79T	54T
92G	79G	54G
97T	80C	52C
99G	80G	51G
100A	78A	52A
99G	77G	50G
99A	76A	52A
100T	77T	50T
100C	79C	53C
101A	79A	55A
100A	79A	54A
101C	77C	55C
101A	78A	54A
102A	79A	53A
102C	79C	55C
105A	79A	56A
106C	77C	54C
107G	76G	56G
57T_49C	76C	55C
106T	75T	55T
106C	72C	54C
108A	70A	54A

113C	70C	57C
113T	69T	57T
112T	69T	57T
111T	69T	58T
113C	68C	55C
116C	71C	55C
119C	72C	57C
120C	71C	57C
120G	71G	58G
120G	70G	58G
123C	70C	60C
126A	69A	61A
126A	67A	61A
123C	68C	61C
120T	68T	61T
121T	69T	61T
121G	70G	61G
117T	43T_28C	56T
123A	70A	56A
124C	71T	56C
135T	70T	60T
136A	71A	60A
137T	71T	61T
134G	73G	60G
134T	73T	59T
135G	74G	58G
130A	74A	57A
134A	72A	55A
133G	72G	55G
132C	70C	56C
134T	71T	53T
132T	66T	53T
129C	68C	54C
133A	67A	53A
132A	70A	53A
131A	69A	53A
132C	69C	48C
130A	67A	50A
129C	70C	51C
129C	69C	50C
127A	70A	47A
122A	67A	47A
122A	72A	47A

125G	71G	45G
124G	69G	45G
125G	71G	45G
122C	73C	44C
121A	74A	43A
123T	72T	43T
119A	71A	45A
113G	72G	45G
113G	73G	46G
114A	75A	48A
114T	75T	49T
114A	75A	46A
113T	74T	46T
113C	73C	44C
114A	76A	44A
114T	75T	43T
115C	76C	44C
117C	38A_39C	46C
119T	77T	45T
117A	77A	45A
117T	78T	45T
117A	79A	45A
116A	80A	43A
120C	78C	43C
119C	79C	43C
119C	80T	44C
118T	79T	43T
113C	82C	42C
112C	81C	43C
115C	83C	44C
116A	83A	44A
117A	82A	44A
113A	80A	44A
109G	80G	43G
111C	82C	44C
110T	83T	45T
114T	84T	43T
112G	83G	42G
110C	83C	41C
108C	83C	40C
110T	84T	39T
112C	90C	39C
106T	89T	37T

108C	91C	36C
107C	90C	38C
102A	92A	38A
102C	92T	39C
102G	92G	39G
101A	93A	38A
102A	94A	39A
103G	94G	39G
101A	97A	38A
100A	95A	40A
101G	97G	41G
47T_53C	98C	39C
101G	99G	21A_18G
101T	99T	41T
102C	99C	44C
103T	99T	44T
53T_49C	98C	24T_21C
101C	101C	46C
106T	101T	47T
108C	102C	45C
109T	102T	46T
110G	103G	45G
111A	101A	44A
109T	103T	44T
108G	102G	43G
105T	45A_58T	44T
52A_56C	100C	46C
106T	100T	47T
105T	100T	43T
107T	98T	45T
108T	98T	45T
107T	98T	45T
106A	100A	44A
107A	100A	43A
107A	102A	44A
105A	99A	42A
97G	97G	43G
104C	99C	42C
106C	101C	41C
107G	100G	29G
106C	101C	29C
104C	100C	29C
106A	102A	29A

105G	101G	29G
104G	101G	29G
104G	96G	29G
105C	99C	30C
103T	96T	31T
104G	95G	33G
107G	94G	33G
104C	95C	33C
104A	96A	33A
106G	94G	34G
106C	95C	33C
107G	93G	34G
107A	94A	36A
108T	93T	36T
108G	95G	35G
104A	95A	34A
106C	94T	36C
107G	93G	36G
105G	96G	37G
105C	96C	35C
100T	96T	34T
36A_65G	97A	35A
101T	96T	35T
103T	97T	35T
105T	95T	35T
105T	92T	32T
104G	94G	33G
106T	93T	33T
106C	31G_62C	32C
105A	89A	30A
103G	90G	31G
110T	89T	30T
109T	89T	31T
108T	88T	32T
111T	87T	31T
114C	84C	31C
114C	85C	30C
115A	83A	29A
117A	86A	29A
119A	85A	29A
122A	85A	30A
118G	84G	27G
119A	88A	27A

121G	86G	27G
121T	85T	27T
116T	81T	24T
116C	84C	25C
115T	85T	25T
116T	84T	25T
118G	82G	25G
120A	80A	26A
121A	83A	26A
121G	83G	25G
119T	80T	24T
121C	81C	26C
121G	81G	26G
118T	82T	26T
118T	80T	26T
118A	79A	27A
117G	77G	27G
117G	77A	26G
117C	75C	25C
117T	75T	25T
116G	75G	23G
118G	74G	23G
115A	74A	24A
45T_1G_50C	74C	25C
93T	73T	25T
44T_50C	72C	26C
51A_44G	73A	26A
94C	45T_29C	26C
111C	74C	26C
115A	76A	26A
117T	76T	25T
116G	76G	24G
117G	78G	23G
119C	79C	23C
119A	79A	23A
122G	77G	23G
121A	78A	23A
124A	78A	23A
124G	80G	23G
123C	81C	21C
122A	82A	36A
122G	82G	39G
122C	81C	41C

124C	84C	40C
124A	87A	39A
122A	85A	38A
123G	84G	39G
125C	82C	40C
124T	81T	40T
125G	80G	39G
123A	80A	39A
124C	34T_46C	19T_21C
122T	80T	39T
123C	79C	40C
120T	78T	40T
121T	77T	41T
121C	79C	42C
121T	77T	40T
114G	77G	42G
117C	78C	45C
118A	77A	45A
115G	78G	46G
112C	78C	49C
115A	77A	49A
116A	77A	51A
115G	73G	51G
113A	75A	53A
111C	73C	51C
113C	74C	52C
114T	73T	52T
114G	74G	53G
112T	75T	52T
113C	78C	52C
115C	76C	53C
114T	78T	53T
113T	78T	54T
111C	79C	55C
110C	81C	54C
109A	78A	56A
109C	78C	57C
110A	79A	56A
109T	80T	55T
110C	80C	55C
114C	82C	55C
115T	81T	57T
114T	82T	56T

117G	82G	56G
117A	83A	55A
117T	80T	54T
116T	80T	53T
118G	81G	53G
116C	80C	51C
116C	79C	49C
117T	80T	49T
117C	77C	51C
116G	78G	49G
112T	75T	48T
112C	76C	51C
113T	75T	50T
111T	74T	50T
109T	72T	49T
112T	71T	50T
113C	73C	48C
112A	72A	47A
114A	73A	47A
114G	74G	46G
116A	75A	46A
113T	77T	44T
112T	74T	45T
113C	72C	45C
112C	72C	46C
112A	71A	45A
114C	71C	44C
113C	67C	45C
113A	63A	46A
116G	63G	46G
116T	62T	46T
116T	63T	45T
122T	24T_40G	23T_23G
125C	64C	45C
127C	64C	45C
127T	63T	45T
126T	61T	45T
126C	59C	46C
126A	60A	47A
127A	61A	45A
129A	60A	45A
55T_79G	63C	26T_22C
134G	63G	49G

132G	61G	47G
133A	62A	47A
132C	60C	45C
135A	60A	45A
133T	60T	45T
135T	63T	44T
137A	62A	45A
136A	62A	44A
135G	62G	44G
136C	59C	43C
136T	58T	44T
135G	42G	43G
134G	41G	24G_20C
137C	41C	43C
135C	40C	44C
135G	38G	44G
136G	38G	45G
136C	38C	44C
135C	37C	45C
134T	36T	43T
134C	36C	44C
133T	36T	44T
133A	34A	45A
130G	34G	45G
127T	33T	47T
125T	33T	47T
128T	33T	46T
126C	33C	47C
128C	32C	47C
129A	32A	47A
128G	32G	47G
125G	31G	46G
125A	31A	44A
76T_49C	32C	47C
122C	33C	46C
123C	34C	46C
123C	35C	47C
128A	36A	47A
128C	35C	46C
132C	36C	46C
131C	35C	45C
132C	37C	44C
129T	38T	45T

129G	38G	45G
128G	41G	46G
128A	40A	46A
126C	42C	45C
123C	40C	44C
126C	39C	46C
127T	40T	48T
128T	41T	45T
130T	42T	46T
130C	42C	45C
130T	43T	45T
132G	43G	45G
134A	44A	43A
131A	42A	46A
137A	43A	46A
136A	44A	47A
130G	45G	22A_26G
124G	47G	48G
123C	47C	47C
121T	47T	47T
123G	47G	51G
122G	47G	51G
121T	47T	51T
124G	46G	50G
124C	47C	51C
124T	45T	49T
124A	46A	48A
126C	47C	48C
125C	75C	45C
127C	76C	48C
127T	76T	49T
125G	77G	48G
122G	77G	51G
122C	79C	50C
123C	77C	48C
122G	79G	50G
124C	80C	52C
124T	82T	51T
124T	85T	52T
123T	84T	52T
119C	85C	52C
120C	86C	55C
121A	86A	56A

119G	87G	56G
119G	90G	55G
121A	96A	55A
122G	97G	55G
124A	98A	51A
127C	98C	53C
126A	98A	56A
125A	98A	56A
126G	97G	54G
127G	98G	53G
126A	99A	55A
125A	99A	55A
122G	101G	55G
121C	100C	53C
121A	100A	54A
126C	100C	55C
127A	101A	55A
129T	100T	53T
130G	101G	52G
132C	98C	53C
133A	96A	53A
130A	97A	53A
130A	98A	53A
127C	99C	53C
130A	99A	56A
129A	99A	55A
130A	99A	54A
129A	99A	55A
131C	97C	56C
129A	96A	55A
134C	97C	55C
136G	101G	55G
135A	101A	53A
133T	100T	51T
135T	100T	51T
135T	99T	52T
137G	99G	53G
134T	99T	54T
130A	99A	54A
130C	97C	53C
132A	99A	53A
125A	101A	52A
127C	95C	54C

130T	94T	53T
132A	94A	54A
129T	95T	53T
131C	96C	57C
131T	96T	53T
132T	100T	53T
134A	100A	51A
131T	101T	51T
129C	94C	50C
124C	92C	49C
124T	92T	51T
125G	91G	50G
126G	92G	50G
126G	92G	48G
123C	92C	50C
124A	95A	23A_27G
126C	93C	47C
124A	94A	47A
124C	95C	47C
129T	94T	47T
125T	95T	47T
122T	94T	47T
121T	90T	45T
120C	91C	43C
119T	91T	42T
118G	90G	40G
65A_31T	89A	39A
96A	89A	39A
93C	87C	38C
92C	81C	38C
55A_169T_111G_195C	25A_105T_53G_101C	16A_54T_32G_64C
117C	94C	50C
89T	76T	30T
90G	76G	30G
96T	78T	33T
99G	78G	33G
100C	79C	34C
102A	40A_40T	35A
105T	80T	37T
106T	83T	36T
109T	85T	39T
111C	84C	40C
112A	84A	42A

114C	41T_46C	42C
117A	86A	42A
122G	86G	44G
124C	87C	46C
125C	89C	47C
128T	90T	49T
130T	89T	51T
131C	88C	52C
135T	91T	53T
69A_66G	91G	25A_28G
134T	91T	55T
135T	92T	56T
133G	93G	58G
134C	93C	57C
137A	91A	60A
76A_63G	90A	29A_31G
137C	92C	61C
141C	89C	60C
140A	90A	59A
142C	91C	59C
140C	90C	58C
143A	89A	58A
141A	91A	57A
140T	92T	58T
138G	88G	58G
136G	88G	58G
136A	87A	59A
137C	88C	59C
137C	90C	59C
135T	89T	57T
135G	87G	57G
134T	89T	58T
133T	91T	61T
136G	91G	61G
134G	89G	61G
131C	88C	61C
130T	90T	59T
133A	90A	59A
129C	91C	57C
128A	91A	59A
128G	92G	59G
128A	94A	60A
129C	48T_45C	61C

132G	93G	61G
133A	90A	59A
131T	92T	60T
128G	92G	60G
128A	95A	62A
127C	93C	63C
129A	94A	62A
130A	94A	61A
77A_53C	93C	60C
126A	94A	60A
131T	45A_51T	63T
131T	95T	63T
132C	96C	63C
131G	98G	64G
132C	95C	64C
131A	94A	65A
129A	95A	63A
132C	95C	63C
128T	94T	59T
127C	36A_93C	60C
126A	93A	59A
124A	91A	58A
124A	94A	56A
127G	92G	57G
124C	90C	56C
72T_50C	95C	29T_1G_25C
124G	92G	54G
123T	93T	54T
122C	91C	55C
123C	91C	56C
124T	90T	55T
124T	86T	54T
122T	89T	51T
122A	90A	50A
123T	89T	49T
118T	88T	48T
120G	88G	50G
123G	88G	48G
119G	89G	51G
120C	90C	50C
120T	91T	50T
117G	90G	47G
117T	91T	48T

116A	92A	49A
114T	91T	49T
114T	91T	50T
116T	89T	52T
116T	88T	51T
114A	90A	52A
114T	90T	50T
56T_54G	92G	22T_24G
110A	42A_49G	46A
118T	90T	47T
119T	92T	48T
119T	92T	50T
123T	93T	50T
126A	94A	50A
126G	94G	51G
125A	93A	50A
127C	91C	47C
125A	88A	46A
124G	87G	46G
125T	86T	46T
122G	86G	46G
122A	86A	46A
122A	87A	48A
122G	85G	48G
122C	87C	48C
122C	88C	47C
123T	89T	48T
121G	93G	47G
119A	91A	45A
119T	91T	49T
121C	89C	48C
121C	85C	46C
122A	85A	47A
120C	84C	47C
120A	86A	50A
121G	87G	50G
121A	85A	48A
119A	84A	46A
119G	84G	44G
119A	84A	44A
121C	85C	42C
121T	84T	44T
123G	85G	43G

123A	82A	43A
123T	85T	44T
122T	85T	44T
122T	86T	42T
125A	87A	46A
126A	90A	47A
125G	91G	49G
120A	89A	48A
122G	91G	48G
121C	90C	49C
120A	89A	47A
121A	95A	48A
122G	98G	47G
119G	98G	43G
123A	99A	46A
125A	99A	47A
125A	99A	47A
124A	98A	48A
123G	48G_53C	49G
122G	103G	48G
123A	104A	51A
126A	103A	51A
124G	103G	48G
131G	101G	50G
132C	99C	51C
134T	98T	53T
131T	98T	52T
134T	98T	50T
134C	101C	53C
133T	101T	51T
127C	101C	49C
125C	104C	50C
125C	101C	49C
125T	51T_51C	50T
125G	101G	51G
124A	101A	52A
123T	101T	53T
121A	101A	52A
119A	101A	50A
120A	101A	50A
117G	100G	50G
61G_57C	100G	1T_33G_18C
118A	100A	53A

118C	105C	54C
118T	104T	55T
120A	106A	56A
121T	107T	57T
124A	54A_53G	57A
125G	105G	55G
122G	104G	56G
123A	104A	55A
123A	102A	56A
125G	99G	56G
124A	100A	56A
123A	96A	54A
129A	97A	54A
130A	99A	54A
130G	100G	54G
132G	100G	53G
130C	100C	48C
66A_64T	98C	17A_34T
131G	45A_51G	49G
132G	97G	50G
132T	98T	51T
129G	101G	52G
127A	100A	51A
129T	98T	54T
127C	98C	53C
124T	99T	55T
126T	99T	56T
125A	101A	55A
126G	99G	55G
125T	100T	59T
124G	101G	60G
123G	98G	55G
123G	96G	56G
124T	93T	55T
129T	95T	56T
130T	95T	55T
130T	93T	54T
130T	93T	56T
127C	92C	55C
126T	89T	58T
127A	89A	58A
122T	88T	55T
119G	86G	58G

120T	88T	57T
119T	88T	57T
120C	84C	56C
124A	81A	54A
121T	79T	52T
115C	80C	52C
116T	80T	54T
112T	83T	53T
111T	83T	51T
109A	85A	50A
109A	84A	50A
107T	82T	48T
106T	41T_40C	27T_21C
107T	81T	48T
110C	82C	49C
110T	80T	51T
114T	80T	52T
115T	82T	51T
114G	83G	51G
113G	85G	52G
112C	89C	51C
113G	88G	52G
114T	88T	51T
112A	88A	53A
112T	89T	51T
109T	89T	53T
111A	87A	54A
109A	81A	54A
107G	85G	52G
105C	84C	52C
103C	82C	50C
106T	82T	54T
103T	83T	56T
108G	84G	54G
109A	84A	51A
110T	87T	54T
108T	87T	56T
112A	85A	56A
112C	86C	56C
110T	86T	56T
108T	84T	57T
105A	82A	57A
105A	83A	57A

106A	85A	59A
108T	84T	59T
105T	85T	58T
103G	85G	58G
105T	87T	57T
110A	87A	57A
108A	87A	56A
109T	87T	54T
111T	86T	58T
112A	85A	58A
110T	83T	57T
111G	81G	56G
108G	83G	57G
108C	83C	55C
105A	49A_34G	30A_24G_1C
106T	84T	55T
108A	85A	54A
104T	84T	52T
104C	84C	53C
102T	82T	52T
105T	81T	53T
102T	82T	52T
103T	77T	51T
105G	78G	49G
104G	79G	48G
103G	80G	50G
104T	81T	48T
101T	81T	48T
103G	81G	48G
105G	82G	50G
102T	85T	51T
101T	85T	51T
103T	87T	51T
102T	85T	50T
103G	83G	50G
103A	85A	50A
103A	82A	50A
103T	83T	50T
102T	84T	50T
103T	85T	49T
101A	84A	48A
99C	83C	48C
98T	82T	47T

93G	84G	46G
91G	80G	48G
92T	77T	47T
94G	74G	45G
97G	70G	44G
99G	73G	44G
98T	72T	44T
98G	73G	43G
97G	70G	43G
97T	74T	41T
93G	75G	42G
60T_63G	75T	20T_16G
90G	72G	36G
88T	42T_31G	33T
54T_27G	37T_33G	20T_16G
74T	62T	31T
106T	74T	41T
108T	75T	41T
113T	75T	26T_16C
110T	76T	42T
106T	73T	42T
108T	74T	43T
110T	74T	44T
105C	70C	45C
105T	72T	45T
105T	74T	47T
105T	74T	49T
105T	74T	19A_29T
108C	73C	47C
109A	72A	50A
108A	72A	52A
104A	71A	52A
101A	68A	51A
102A	68A	51A
99T	68T	53T
100G	69G	51G
100C	71C	50C
97T	73T	51T
101T	74T	51T
103T	71T	51T
104T	73T	51T
106T	73T	51T
106G	74G	52G

106T	76T	51T
107T	76T	53T
108T	76T	53T
106C	76C	54C
108T	79T	53T
108C	78C	53C
109C	78C	53C
108C	78C	53C
107T	77T	54T
109C	79C	54C
111C	79C	54C
111T	78T	55T
110C	80C	56C
110T	79T	57T
110A	81A	57A
109C	39T_1G_41C	59C
110C	79C	58C
111T	81T	58T
112G	81G	57G
113T	82T	54T
56A_56G	86G	57G
113A	86A	58A
111T	85T	58T
112G	86G	60G
115G	86G	60G
116A	86A	61A
118T	86T	61T
121T	84T	61T
122T	88T	59T
122G	88G	60G
118A	91A	60A
116A	94A	61A
114A	91A	62A
113A	91A	63A
115G	91G	62G
114A	91A	61A
110G	89G	62G
107G	92G	62G
103T	93T	58T
105G	93G	60G
103T	93T	61T
100T	92T	57T
99T	93T	60T

98T	92T	59T
96C	93C	58C
54A_42G	94A	26A_33G
97G	93G	59G
97C	93C	60C
95A	93A	58A
91T	94T	58T
92G	95G	57G
94G	97G	56G
93C	97C	53C
92G	96G	53G
91T	95T	55T
92G	95G	56G
91G	96G	54G
96T	95T	53T
95G	97G	52G
94A	98A	50A
93A	98A	54A
48T_41G	99T	53T
42A_46T	98T	52T
91A	97A	51A
93A	95A	52A
94A	95A	52A
97A	96A	54A
99C	94C	54C
97A	93A	53A
95G	91G	50G
96G	90G	51G
97A	91A	51A
95T	92T	51T
95T	94T	53T
93A	91A	54A
95A	91A	53A
96A	93A	54A
101A	93A	52A
101A	93A	55A
100T	91T	53T
100T	91T	53T
99A	89A	53A
100G	88G	50G
99C	87C	50C
99C	87C	48C
98A	88A	48A

99A	88A	47A
101C	87C	48C
101T	87T	49T
102C	86C	49C
102A	84A	50A
102C	84C	50C
103T	85T	52T
102A	83A	50A
101A	85A	50A
101A	85A	49A
100T	85T	48T
98G	86G	48G
97A	83A	48A
96G	84G	51G
95C	83C	52C
99A	84A	53A
99A	84A	51A
100C	83C	53C
99T	82T	56T
103G	83G	57G
107C	83C	56C
108T	80T	53T
113C	78C	57C
113A	80A	58A
112G	80G	56G
112A	82A	57A
114A	82A	57A
119A	81A	57A
119A	80A	58A
120G	83G	59G
118G	81G	63G
121A	81A	61A
125A	80A	62A
126G	80G	60G
125G	80G	63G
123C	77C	62C
124A	77A	63A
126G	80G	64G
121T	82T	61T
125C	84C	62C
124C	83C	62C
122A	82A	62A
126A	81A	63A

125T	81T	63T
124T	32T_48C	63T
125C	79C	62C
124T	82T	63T
128G	81G	63G
127C	81C	64C
127A	82A	63A
123C	81C	62C
121T	82T	63T
126T	82T	62T
130T	83T	64T
132C	80C	65C
130C	79C	66C
133A	80A	65A
136G	80G	63G
136C	81C	60C
134A	81A	62A
132G	80G	62G
127A	78A	64A
127A	80A	64A
126A	82A	64A
126A	81A	64A
130T	81T	63T
127C	80C	62C
131C	81C	63C
130A	77A	64A
133A	76A	62A
133G	77G	63G
133T	79T	64T
132A	79A	65A
136G	78G	66G
136C	78C	65C
134T	78T	62T
136C	78C	62C
135T	78T	63T
133A	77A	62A
133A	78A	61A
133G	77G	61G
133A	76A	62A
134T	76T	61T
133T	74T	64T
136T	73T	62T
134C	71C	61C

135C	72C	62C
134A	72A	59A
136A	72A	58A
136C	71C	57C
138C	72C	57C
138C	73C	57C
135A	73A	55A
135G	70G	54G
137G	69G	53G
134A	68A	53A
136C	69C	53C
135T	72T	53T
66T_1G_68C	38T_33C	52T
131T	69T	50T
131T	70T	50T
132T	72T	51T
62T_65C	40T_34C	50T
130C	75C	50C
132T	74T	49T
137C	74C	22T_27C
137G	74G	47G
134G	72G	47G
135G	72G	45G
131C	70C	44C
70A_61G	70G	42G
129C	70C	42C
119T	67T	40T
128T	69T	43T
129T	69T	44T
129T	69T	43T
130T	67T	42T
124T	68T	42T
125G	67G	42G
124T	68T	41T
127C	70C	42C
126T	68T	42T
121A	69A	42A
118G	69G	42G
116G	69G	42G
115A	70A	42A
115A	68A	43A
65T_49C	68C	43C
114C	68C	44C

112A	67A	43A
113G	67G	44G
117C	66C	43C
119C	65C	42C
121A	63A	43A
120G	62G	46G
119G	63G	46G
120A	65A	47A
118A	64A	47A
57A_67G	64G	47G
122A	65A	48A
125G	63G	48G
122A	64A	47A
121G	63G	45G
122A	63A	44A
124T	65T	45T
124T	66T	44T
124C	67C	45C
123G	67G	44G
124G	66G	44G
127T	64T	44T
129T	64T	44T
132C	66C	47C
133T	66T	46T
135T	68T	42T
132T	68T	42T
130T	69T	41T
134C	69C	43C
134A	70A	43A
130C	70C	42C
130A	70A	43A
127A	71A	43A
126A	71A	44A
129A	71A	44A
127T	75T	42T
126C	74C	47C
128A	75A	47A
129G	74G	47G
125T	72T	48T
126A	73A	48A
128A	72A	47A
130T	75T	44T
128C	75C	45C

128T	73T	45T
129T	71T	45T
127T	71T	45T
122G	73G	45G
121G	70G	45G
123A	71A	47A
126C	69C	51C
126A	72A	51A
132T	72T	50T
133G	72G	50G
133A	72A	49A
132T	73T	50T
130G	74G	49G
133A	75A	50A
134T	75T	49T
136C	75C	49C
137G	76G	50G
137C	75C	50C
134A	73A	49A
135C	73C	50C
137A	73A	50A
137G	72G	49G
135A	73A	51A
135C	74C	50C
136T	75T	50T
137A	76A	50A
138G	77G	53G
136A	77A	55A
137G	78G	55G
135C	78C	56C
130C	83C	55C
131G	86G	54G
45T_81C	86C	52C
124G	85G	52G
82A_44G	83G	50G
129T	85T	56T
135A	85A	56A
135T	83T	54T
133C	84C	54C
134T	82T	57T
135G	84G	57G
133A	83A	57A
131C	84C	57C

132G	83G	57G
130C	81C	58C
131A	82A	58A
132G	82G	58G
127A	83A	57A
129C	83C	57C
128A	83A	55A
130A	83A	55A
129T	80T	56T
59T_71G	81T	27T_27G
130A	81A	56A
127A	81A	55A
127G	80G	55G
131T	81T	54T
131C	80C	54C
130C	79C	54C
130T	79T	54T
126T	81T	55T
127G	76G	55G
127G	78G	53G
124C	77C	54C
124A	76A	55A
123G	76G	55G
122C	75C	54C
123T	75T	55T
125A	71A	55A
121T	71T	56T
124C	72C	56C
121G	72G	29A_26G
122C	71C	56C
124C	70C	58C
123A	74A	56A
125A	74A	56A
122G	77G	54G
126C	75C	57C
121G	75G	57G
121T	76T	57T
118C	74C	58C
123C	75C	58C
125T	76T	56T
122T	76T	56T
129C	76C	55C
131T	77T	56T

133G	76G	55G
133G	75G	55G
138C	77C	54C
71A_68G	34A_44G	51G
138T	77T	52T
138T	76T	53T
140A	78A	55A
139A	74A	55A
137A	74A	54A
135G	72G	55G
136C	69C	52C
138T	69T	51T
138G	69G	50G
139A	69A	55A
139G	65G	55G
139G	62G	52G
138A	63A	51A
138G	63G	49G
138A	63A	50A
72A_66G	37A_26G	23A_25G
132G	64G	50G
132G	65G	51G
134A	67A	51A
133T	65T	50T
130C	67C	51C
130G	67G	52G
134G	64G	55G
133G	63G	26A_31G
136A	64A	55A
134A	63A	56A
135C	64C	56C
136G	65G	55G
134T	67T	54T
134A	65A	54A
131A	66A	55A
133G	68G	53G
133A	71A	53A
132T	70T	54T
131C	69C	54C
235T_1G_130C	56T_69C	98T_54C
131C	67C	58C
133C	69C	59C
136A	70A	59A

141C	70C	60C
141T	67T	60T
141G	67G	60G
140C	67C	59C
140C	68C	58C
139A	66A	59A
137A	66A	58A
135C	66C	60C
136T	64T	58T
138T	63T	63T
137T	63T	61T
139G	64G	62G
137G	65G	63G
138A	64A	62A
134G	64G	62G
133A	62A	62A
130C	63C	61C
122A	61A	56A
52T_68C	59C	25T_31C
124A	60A	58A
129A	58A	59A
130A	56A	59A
130G	55G	60G
130C	55C	60C
127C	55C	60C
123A	55A	60A
122C	53C	61C
121A	55A	62A
115G	56G	62G
116G	56G	63G
114A	59A	62A
116A	58A	61A
119A	60A	60A
121A	60A	61A
118A	61A	63A
119G	63G	64G
117C	62C	62C
117A	65A	63A
119C	65C	63C
119A	64A	58A
121A	64A	59A
123A	66A	58A
123A	67A	59A

123G	67G	59G
122C	67C	59C
123T	67T	60T
119A	66A	59A
121T	65T	58T
118T	65T	57T
125C	65C	58C
126A	66A	58A
122G	63G	57G
124A	63A	56A
121T	64T	54T
123C	66C	54C
122T	69T	56T
124G	69G	56G
122C	68C	56C
123C	66C	55C
124A	64A	58A
125T	65T	59T
126G	65G	61G
129T	63T	62T
128A	63A	57A
128G	63G	58G
126A	63A	55A
126C	66C	54C
124G	40A_27G	53G
124C	66C	52C
121A	68A	52A
124C	69C	51C
120A	69A	51A
122G	69G	50G
124A	69A	48A
125C	70C	49C
124T	70T	49T
127G	70G	47G
124T	70T	46T
129T	72T	47T
129T	75T	43T
128G	76G	43G
128G	76G	45G
128G	76G	44G
132A	75A	44A
133C	75C	44C
136A	76A	44A

132C	77C	44C
132A	77A	46A
134C	77C	46C
133A	76A	43A
131C	76C	43C
132A	79A	44A
131C	78C	44C
130A	78A	46A
133C	79C	46C
133A	79A	66A_20G_20C
130G	28G_53C	17G_5C
129A	79A	21A
131G	76G	17G_5C
132A	75A	42A
133C	51T_23C	28T_1G_17C
136G	23A_51G	46G
136T	73T	46T
135A	72A	46A
137C	72C	45C
139A	71A	49A
141C	72C	51C
143A	72A	53A
142C	73C	53C
144T	75T	55T
143G	75G	55G
141A	73A	53A
140A	73A	55A
140C	75C	55C
139C	75C	56C
138T	75T	55T
141C	76C	55C
140A	76A	55A
140G	75G	54G
135A	73A	19A_35G
137G	73G	54G
135G	72G	55G
133G	73G	54G
134C	71C	53C
134C	68C	50C
135G	64G	53G
133C	65C	53C
136A	64A	55A
138C	65C	55C

138A	66A	54A
139C	64C	54C
135A	62A	55A
133A	63A	52A
135C	63C	52C
135C	64C	50C
139C	64C	49C
138C	63C	48C
138C	62C	49C
137A	62A	51A
140A	61A	52A
137G	59G	25T_27G
137C	60C	53C
139C	61C	54C
133T	62T	53T
134C	32T_30C	52C
138T	63T	53T
135G	64G	53G
135C	63C	52C
130A	61A	52A
129T	59T	53T
130G	59G	53G
129T	59T	54T
133T	58T	55T
129A	58A	55A
126T	56T	54T
124C	56C	50C
125C	54C	52C
124T	54T	52T
124C	55C	52C
124T	57T	53T
120G	60G	53G
125A	57A	52A
127A	62A	53A
127T	63T	50T
125G	61G	52G
126A	63A	53A
127T	60T	53T
127T	61T	55T
126T	62T	56T
129T	60T	57T
132A	62A	56A
132T	64T	57T

131T	65T	58T
131T	66T	57T
132A	65A	55A
129A	65A	54A
126A	65A	52A
124A	62A	51A
124G	62G	50G
123G	61G	49G
122C	61C	49C
121C	60C	49C
122C	61C	47C
121T	61T	51T
121T	61T	49T
123T	60T	51T
125T	60T	49T
125A	60A	50A
125A	61A	52A
124A	63A	53A
123G	64G	51G
126G	67G	56G
129G	66G	58G
129G	68G	58G
128G	68G	60G
131C	68C	61C
133A	68A	61A
133A	70A	59A
131G	72G	61G
130A	71A	64A
130A	72A	65A
129G	74G	64G
129G	75G	62G
129G	75G	64G
130A	74A	64A
129A	74A	66A
128T	78T	63T
126C	75C	65C
124G	75G	64G
120T	75T	64T
119C	76C	64C
117C	75C	64C
69T_1G_47C	73T	64C
119G	73G	64G
116C	73C	65C

121G	73G	64G
122T	72T	65T
126T	71T	65T
128T	72T	65T
128C	73C	64C
130A	74A	64A
132C	73C	63C
128A	74A	64A
129C	75C	62C
131C	75C	63C
131T	75T	64T
131G	78G	62G
133T	78T	58T
135G	78G	61G
135G	77G	59G
135C	75C	60C
127T	75T	61T
125G	71G	59G
127C	70C	60C
126T	70T	60T
126T	68T	59T
124T	68T	57T
125G	67G	57G
123T	67T	54T
118T	65T	51T
115C	65C	50C
114C	65C	48C
114G	63G	29A_18G
115C	66C	48C
112T	66T	46T
115C	66C	48C
114T	65T	46T
113A	66A	46A
113A	65A	45A
113A	64A	46A
114A	65A	46A
117A	64A	46A
116T	62T	45T
117A	61A	44A
120A	62A	42A
118A	63A	42A
114A	61A	42A
114T	61T	43T

114A	60A	41A
113A	58A	41A
113A	56A	42A
112A	53A	38A
108T	53T	38T
110T	52T	39T
109G	52G	37G
105G	53G	36G
106A	54A	35A
102A	52A	35A
94A	50A	33A
103C	50C	32C
100C	49C	32C
102G	47G	32G
102T	48T	32T
103T	48T	31T
101T	47T	31T
99C	46C	32C
101T	41T	33T
101G	41G	32G
102G	40G	35G
102G	41G	36G
103T	40T	33T
105C	42C	36C
106T	44T	35T
105G	43G	35G
107G	43G	35G
104G	43G	35G
102G	43G	37G
99G	43G	37G
96A	41A	36A
96A	42A	39A
93A	41A	39A
53T_41C	41C	20T_19C
97G	42G	39G
96C	40C	41C
96T	41T	41T
97G	42G	43G
96T	38T	41T
93C	38C	43C
92C	38C	42C
91A	37A	43A
93G	36G	43G

93G	36G	43G
93A	37A	43A
92C	37C	42C
93C	38C	42C
95T	39T	42T
96G	40G	44G
95A	41A	44A
96A	40A	43A
98A	42A	43A
98C	41C	44C
99A	40A	44A
97G	40G	46G
98C	36C	45C
100C	38C	43C
100C	38C	44C
98T	37T	44T
99C	37C	44C
98T	38T	42T
96C	38C	45C
154C	38C	25A_92C
59C	38C	17C
58C	38C	17C
52A	38A	16A
86C	37C	45C
85C	38C	44C
84C	38C	43C
71A	38A	28A
81C	41C	32C
80C	41C	34C
79C	41C	31C
80C	40C	30C
76C	19T_23C	30C
74C	42C	32C
76A	42A	32A
80A	45A	33A
80A	44A	34A
80A	45A	34A
43T_37C	24T_21C	35T
79A	44A	37A
77G	47G	36G
79G	46G	36G
81G	47G	37G
82A	46A	37A

82A	47A	37A
83A	48A	37A
84T	49T	37T
84C	51C	37C
84A	51A	37A
83T	51T	36T
86G	53G	36G
86C	52C	36C
86C	50C	34C
88C	53C	34C
87T	52T	35T
86A	53A	16A_17G
84T	53T	32T
85A	55A	32A
84A	56A	31A
83C	55C	31C
85A	55A	29A
85C	56C	29C
85G	56G	29G
85C	56C	32C
84A	56A	31A
81A	56A	32A
77T	57T	33T
78C	59C	31C
77C	60C	35C
76T	63T	32T
76C	63C	34C
76A	64A	34A
79G	63G	34G
78G	65G	35G
80G	67G	38G
78G	66G	36G
81C	66C	39C
81A	68A	39A
82T	71T	39T
81G	70G	38G
79A	69A	37A
80G	72G	37G
81A	73A	39A
85T	73T	41T
84G	74G	41G
82G	73G	42G
82T	73T	40T

80C	73C	40C
79T	72T	41T
80T	73T	40T
80T	72T	41T
83T	73T	40T
84G	73G	42G
85A	72A	43A
84A	73A	42A
86G	71G	43G
87T	73T	42T
85T	73T	44T
85G	70G	41G
84T	71T	44T
87A	74A	45A
90A	77A	44A
93A	76A	44A
95G	75G	44G
97A	75A	44A
96A	72A	43A
97A	75A	42A
97A	75A	45A
97G	76G	46G
95A	77A	45A
98A	77A	46A
100T	77T	47T
99A	74A	48A
101G	75G	49G
57A_47G	75G	50G
47A_57G	42A_33G	50G
106T	74T	50T
105T	72T	51T
107T	73T	52T
109A	72A	52A
107A	70A	52A
106G	70G	51G
106G	70G	51G
105G	68G	51G
106T	68T	50T
106G	68G	53G
106C	40T_30C	53C
107G	69G	54G
108A	68A	54A
110G	69G	55G

109G	69G	54G
109T	67T	55T
110G	67G	56G
109G	69G	53G
110G	68G	53G
112T	67T	53T
111G	66G	53G
116A	66A	52A
116A	66A	50A
118C	64C	49C
119C	63C	49C
119T	62T	49T
116G	63G	48G
116G	62G	45G
112G	59G	46G
111A	59A	43A
109C	58C	45C
111C	56C	47C
109T	52T	45T
109C	53C	49C
108C	53C	50C
110T	51T	50T
109G	50G	48G
104C	50C	47C
100A	48A	49A
104T	33A_17T	51T
106C	50C	51C
106C	50C	52C
107C	49C	25T_26C
110G	48G	51G
110T	47T	49T
106G	49G	50G
109G	50G	50G
110G	50G	49G
112C	50C	50C
109T	50T	50T
110G	50G	51G
112C	49C	50C
109A	48A	47A
111C	49C	48C
110T	47T	51T
107T	45T	52T
103T	45T	54T

101T	46T	56T
98T	47T	56T
98A	46A	56A
97C	47C	57C
100C	46C	54C
102C	45C	52C
102A	46A	51A
100C	44C	51C
97C	47C	51C
97C	48C	50C
95A	48A	50A
96T	49T	49T
96T	49T	49T
92T	48T	49T
93C	49C	47C
93A	50A	46A
92A	50A	46A
91A	32A_14G	45A
90G	47G	45G
81G	14G_33C	20G_24C
81G	34A_14G	42G
89G	53G	45G
90G	54G	44G
95A	53A	44A
98C	52C	46C
101A	52A	46A
100A	53A	46A
102A	53A	48A
102T	53T	46T
103A	56A	45A
103T	56T	44T
102G	57G	43G
101G	58G	42G
101G	59G	42G
96G	58G	41G
100C	58C	42C
99C	59C	43C
98C	60C	44C
98G	60G	43G
99C	60C	45C
98T	60T	47T
100G	62G	46G
49G_48C	64C	49C

96C	35T_31C	48C
98T	66T	46T
100A	67A	47A
102G	66G	46G
103A	67A	45A
102C	67C	45C
103T	68T	45T
104G	68G	44G
104G	67G	42G
49G_53C	31G_39C	38G
100C	71C	38C
97T	70T	37T
97T	71T	38T
93G	72G	37G
92C	72C	38C
92T	69T	38T
89C	70C	36C
86C	69C	35C
84T	69T	35T
84G	69G	35G
120A_80T_80G_202C	39A_27T_26G_107C	54A_36T_36G_88C
94G	90G	52G
95C	91C	53C
94C	91C	56C
16A_43G	77G	40G
78C	78C	41C
82C	82C	42C
84T	85T	43T
86G	84G	44G
86G	82G	44G
88C	85C	47C
88A	87A	47A
89G	88G	47G
91G	87G	18A_29G
95C	87C	47C
95A	86A	47A
95G	85G	47G
99A	85A	50A
99A	83A	48A
100A	83A	47A
99T	85T	47T
98T	87T	45T
95G	86G	46G

95A	85A	47A
91A	85A	46A
93G	84G	46G
90G	45G_39C	47G
90G	83G	47G
87G	84G	46G
88C	85C	50C
89G	85G	53G
92A	86A	53A
91T	85T	52T
92G	85G	53G
93G	85G	52G
90A	86A	52A
94C	86C	50C
95T	88T	48T
94T	87T	44T
95C	88C	43C
98C	89C	45C
95T	90T	44T
95A	89A	47A
95C	91C	48C
94C	90C	49C
97C	87C	47C
96T	88T	46T
99C	87C	48C
102T	88T	48T
103G	88G	48G
103A	86A	48A
103C	87C	48C
103C	89C	48C
100C	89C	47C
101T	89T	17T_30G
101A	87A	46A
101A	87A	46A
99G	89G	46G
98C	90C	46C
97T	86T	45T
96C	86C	45C
99C	87C	46C
98A	85A	46A
98A	84A	45A
96A	84A	46A
95G	81G	46G

98C	82C	45C
97A	83A	45A
97T	85T	45T
98G	85G	44G
99G	86G	45G
98G	81G	44G
100T	80T	43T
100C	79C	40C
100C	77C	40C
101A	76A	39A
99C	73C	38C
100C	70C	39C
99C	71C	37C
100A	72A	39A
102G	71G	36G
102C	69C	36C
100C	67C	36C
101A	66A	36A
100A	67A	36A
102G	70G	37G
105T	71T	37T
103G	84A_21T_114G_21C	36A_9T_53G_9C
104A	45A	21A
104C	44C	21C
102T	45T	21T
104A	44A	21A
106G	45G	21G
106A	46A	20A
108A	46A	20A
111G	48G	20G
113G	83G	38G
114C	82C	41C
113T	84T	42T
113G	84G	42G
114G	86G	40G
111C	84C	42C
108A	85A	39A
108C	84C	40C
107A	84A	41A
108A	83A	22A_16G
105A	81A	38A
43A_3T_41C	82C	38C
39A_44T_3G	87G	37G

86T	89T	39T
88T	90T	41T
87G	89G	41G
88A	89A	40A
84G	89G	39G
87A	89A	39A
82C	91C	42C
35A_45G	44A_44G	43G
35T_45C	89C	42C
82C	87C	44C
36A_46G	43A_42G	44G
99A	86A	45A
99G	87G	43G
99G	87G	43G
99C	86C	44C
96T	86T	46T
96G	87G	46G
99C	86C	44C
101T	83T	44T
104G	83G	46G
101T	83T	45T
104A	82A	44A
102G	80G	45G
103T	80T	29T_1G_15C
101A	77A	43A
103C	77C	44C
102G	30A_42G	16A_28G
98A	71A	44A
98G	70G	44G
96A	67A_39T_39C	44A
100T	105T_37C	95T_76C
50A_46C	66C	18C
100T	66T	33T
94C	64C	33C
98T	63T	32T
97C	60C	32C
97T	77T	32T
98C	77C	31C
96T	77T	44T
96C	75C	44C
100T	74T	44T
98C	74C	45C
99T	74T	46T

99C	73C	45C
99T	70T	45T
99C	71C	44C
178T_20G_80C	68T	44T
64C	61C	41C
66T	60T	42T
67C	58C	42C
67T	59T	43T
67C	59C	39C
65T	60T	40T
48A_16C	23A_36C	16A_1G_22C
64T	60T	39T
1T_48G_16C	51G	39G
84T	51T	39T
82T	52T	39T
81G	53G	40G
81C	59C	42C
84A	59A	44A
83T	60T	46T
83T	60T	46T
83T	62T	46T
82T	62T	45T
81C	64C	47C
83C	66C	46C
83A	66A	46A
87C	66C	46C
84A	65A	44A
85C	68C	44C
87A	66A	45A
86A	65A	46A
86T	64T	45T
86C	65C	45C
89C	64C	44C
88T	63T	44T
85C	63C	44C
90T	63T	44T
90T	63T	43T
92T	63T	44T
92A	61A	43A
96A	60A	42A
94G	60G	41G
94A	58A	39A
93G	57G	37G

94G	56G	39G
43T_1G_52C	54C	37C
91G	54G	37G
89A	52A	37A
92C	55C	36C
87A	56A	36A
90A	56A	35A
89C	57C	34C
92G	56G	35G
91A	54A	34A
91A	53A	32A
91T	53T	32T
91G	57G	30G
92C	59C	31C
92A	59A	31A
88A	58A	31A
89C	60C	33C
89A	62A	33A
89A	64A	34A
85T	66T	34T
85T	68T	33T
84G	67G	34G
83C	69C	34C
37A_46T	72T	33T
86G	72G	33G
85T	71T	33T
86G	71G	34G
86T	72T	34T
85G	76G	35G
82T	75T	34T
83T	77T	35T
84T	78T	33T
83A	78A	34A
83G	79G	33G
83G	81G	34G
82G	82G	35G
83A	83A	35A
85G	45A_38G	35G
85G	82G	36G
83G	83G	35G
81T	85T	37T
82G	88G	36G
85T	87T	35T

87G	84G	33G
88T	84T	32T
86G	85G	31G
85T	86T	31T
85G	84G	31G
86T	84T	31T
89G	83G	29G
87T	79T	28T
86C	77C	30C
83T	78T	30T
86A	78A	30A
84T	75T	30T
83G	74G	30G
82T	74T	30T
85G	75G	30G
87T	73T	30T
84G	74G	30G
83G	76G	32G
81A	73A	32A
79A	75A	33A
79C	75C	34C
79G	77G	35G
77G	76G	35G
76T	78T	35T
79G	80G	36G
78C	80C	36C
78A	83A	36A
79A	82A	35A
82T	86T	35T
82T	87T	36T
82A	88A	35A
81T	87T	37T
82T	85T	38T
82T	87T	37T
81G	87G	36G
79G	89G	36G
78T	89T	36T
77G	91G	37G
76A	90A	36A
78A	86A	36A
79G	88G	37G
78A	89A	38A
78T	90T	37T

77T	86T	36T
78G	84G	37G
76G	85G	37G
78A	87A	37A
78G	86G	38G
76G	86G	38G
75T	85T	39T
75G	86G	41G
71T	88T	40T
71C	87C	41C
72A	90A	41A
72C	89C	42C
71C	86C	40C
73C	87C	42C
70T	85T	42T
69G	86G	42G
69A	86A	41A
70C	88C	44C
72A	86A	43A
72A	87A	42A
73T	85T	41T
71T	86T	42T
71G	86G	41G
73A	86A	40A
73G	85G	39G
74A	82A	40A
73A	83A	40A
73T	82T	41T
71G	81G	39G
72A	80A	40A
73A	79A	41A
73G	82G	43G
72C	84C	43C
71A	85A	45A
72C	85C	45C
74G	85G	43G
74G	84G	43G
71G	85G	43G
71A	85A	43A
72G	88G	41G
73G	89G	42G
70A	87A	41A
71C	87C	41C

72C	86C	42C
74A	88A	40A
75G	93G	40G
77A	92A	41A
76A	91A	40A
76A	92A	39A
77T	92T	40T
76G	91G	38G
72A	93A	39A
75C	93C	39C
77A	94A	38A
76A	95A	39A
73T	93T	38T
73C	94C	38C
74A	95A	39A
73A	94A	36A
74A	96A	37A
73A	94A	38A
74G	93G	38G
74C	94C	37C
73T	95T	37T
76T	94T	37T
77C	91C	35C
75C	91C	36C
73A	91A	34A
73C	90C	34C
74C	90C	34C
75A	90A	33A
75G	45G_46C	32G
76G	92G	32G
74G	87G	32G
74T	87T	31T
74A	87A	30A
75A	88A	28A
76A	86A	27A
76G	86G	26G
77T	88T	25T
76G	86G	23G
77T	87T	23T
77T	86T	23T
75A	85A	23A
76A	88A	23A
76T	89T	23T

74A	89A	23A
72A	87A	20A
70C	87C	20C
70A	85A	20A
68A	88A	20A
70C	87C	20C
72C	86C	20C
70T	85T	20T
70A	85A	20A
68A	84A	20A
66C	82C	20C
65T	82T	20T
63G	84G	20G
63C	84C	20C
63T	85T	19T
61T	84T	17T
63T	81T	16T
61C	82C	15C
62A	82A	7A_9G
1T_41G_18C	43G_41C	9G_7C
59A	85A	15A
59T	85T	16T
57A	86A	16A
58C	85C	16C
57C	86C	16C
59A	86A	16A
57T	87T	17T
56G	86G	16G
55A	83A	16A
54T	79T	15T
52G	79G	14G
51A	79A	14A
49T	79T	13T
48G	78G	12G
47A	77A	12A
47T	74T	11T
45G	74G	10G
43A	70A	10A
42T	69T	11T
40G	69G	10G
40G	67G	10G
39C	67C	9C
40C	66C	8C

37T	65T	8T
36G	65G	7G
35C	65C	7C
34C	31T_29C	7C
34C	57C	7C
28A	54A	5A_12C
28C	56C	9C
13A_40C	56C	5C
15C	25A_53C	5C
7A_10C	21A_6C	6A
26C	51C	6C
24C	52C	7C
23C	52C	7C
23C	48C	7C
23C	48C	8C
23C	47C	8C
21A	46A	8A
21C	46C	8C
22C	24G_22C	8C
22G	43G	8G
22G	47G	8G
6G_18C	46G	9G
25C	20A_25C	11C
26C	44C	11C
25C	46C	12C
28C	46C	13C
32C	47C	13C
32T	45T	15T
33T	44T	16T
35G	44G	16G
36C	45C	17C
35T	48T	17T
35C	53C	18C
37C	53C	21C
38C	54C	23C
38C	55C	22C
39A	56A	23A
39G	58G	23G
41A	58A	23A
43A	61A	23A
45A	60A	24A
46G	61G	24G
48C	66C	24C

46T	67T	24T
47A	66A	24A
46A	67A	25A
47C	69C	25C
52A	67A	25A
52G	68G	25G
54A	68A	26A
54A	67A	31A
54G	67G	32G
55A	66A	33A
37T_21C	34T_32C	33C
61A	71A	32A
62A	70A	32A
61C	71C	32C
62C	72C	34C
61T	71T	34T
62C	75C	34C
62G	78G	35G
64C	75C	35C
66C	78C	36C
64T	76T	36T
65T	78T	37T
66C	79C	36C
68T	83T	38T
70C	82C	38C
28A_42G	41A_42G	39A
74G	84G	39G
77T	84T	38T
77T	85T	39T
78C	87C	39C
78A	87A	39A
79G	88G	39G
82C	89C	39C
84C	90C	39C
82A	90A	38A
86C	91C	41C
87T	91T	41T
88C	94C	41C
89A	94A	41A
90T	96T	41T
92A	97A	40A
97A	99A	42A
99T	99T	42T

100A	100A	43A
100T	102T	43T
100T	105T	43T
103C	104C	41C
106T	101T	42T
107G	103G	41G
109G	102G	43G
109C	103C	44C
111T	102T	45T
43G_66C	103C	23G_21C
106T	102T	43T
105C	103C	43C
104T	103T	43T
104C	101C	43C
104T	100T	45T
103C	98C	45C
104C	94T	46C
104G	91G	43G
104C	91C	41C
106A	92A	41A
103T	90T	42T
104G	91G	42G
103G	91G	42G
102C	89C	41C
100G	90G	17A_26G
102A	87A	26A_15T_1G
101G	82G	43G
105C	80C	45C
105A	78A	45A
105T	77T	45T
105C	76C	45C
100C	75C	45C
99A	74A	46A
98G	74G	45G
100C	76C	40C
100T	73T	39T
100G	75G	38G
97C	76C	39C
95A	71A	39A
92G	70G	39G
94G	71G	39G
93G	70G	38G
93C	69C	38C

93C	66C	39C
94G	38A_26G	38G
91G	64G	39G
92T	63T	35T
94T	65T	38T
95T	66T	38T
95T	67T	40T
94A	63A	39A
92A	63A	39A
91G	61G	38G
89G	38A_23G	37G
88A	60A	39A
89A	62A	39A
89G	60G	39G
90A	61A	39A
90T	58T	40T
88C	61C	40C
87T	61T	41T
87A	62A	41A
86A	61A	39A
86A	60A	38A
86A	24A_35G	37A
84T	61T	38T
85T	61T	37T
85A	62A	37A
81A	59A	35A
82A	60A	37A
81T	58T	35T
83T	58T	36T
84G	55G	37G
82G	54G	38G
79A	54A	37A
76A	54A	37A
72G	54G	36G
72C	54C	35C
71A	55A	34A
71G	59G	33G
74C	60C	32C
73T	56T	9A_32T
73A	30A_27T	31A
73A	59A	34A
73A	59A	32A
74A	62A	34A

74A	66A	33A
73A	68A	33A
75A	71A	35A
77A	70A	35A
78T	70T	33T
78G	70G	33G
83G	69G	34G
85A	72A	36A
86A	72A	34A
85A	75A	34A
84G	75G	33G
81T	76T	33T
83A	77A	35A
86T	78T	34T
85T	80T	34T
85C	80C	32C
88T	82T	33T
92A	82A	33A
91A	80A	32A
90T	80T	32T
91T	78T	32T
92A	81A	31A
94A	82A	31A
96C	84C	34C
94T	84T	33T
95G	84G	34G
98C	85C	34C
100C	88C	34C
101C	88C	35C
103C	89C	34C
103T	88T	35T
102G	87G	33G
103G	89G	31G
103G	90G	31G
103G	90G	32G
103C	89C	34C
104T	91T	35T
49T_52C	40T_50C	34C
100G	90G	34G
100T	87T	33T
99C	88C	34C
98A	88A	33A
91A	39A_48G	32G

90T	87T	34T
46A_44T	85G	33G
92T	86T	35T
94C	86C	38C
45A_50G	86G	14A_25G
100T	84T	40T
101C	83C	42C
104A	81A	44A
103T	81T	44T
104T	82T	45T
102T	84T	45T
103A	85A	45A
101T	83T	43T
100T	83T	42T
101C	84C	42C
101C	85C	44C
101A	83A	42A
103C	85C	42C
104C	86C	42C
104A	87A	42A
106G	82G	41G
103A	81A	40A
104G	83G	42G
105G	83G	42G
107A	80A	42A
107G	81G	42G
107C	77C	41C
109C	73C	40C
56A_49G	69G	12A_26G
103G	67G	37G
53A_48G	66G	10A_1T_26G
102C	67C	38C
102T	68T	40T
100G	69G	40G
99G	67G	38G
98T	66T	36T
98T	63T	36T
100C	67C	37C
102A	68A	37A
102A	68A	37A
99A	67A	38A
101A	66A	41A
103C	68C	41C

104T	67T	41T
102A	66A	41A
102G	67G	41G
105G	68G	41G
107T	69T	41T
112A	68A	41A
110G	66G	40G
109G	67G	39G
109A	67A	40A
108T	67T	39T
104T	68T	38T
104C	67C	38C
105C	64C	38C
102C	64C	38C
55T_46C	64C	37C
101A	66A	39A
101A	63A	41A
100A	64A	40A
99T	66T	40T
98G	67G	39G
96T	67T	41T
99C	67C	42C
101T	68T	41T
100C	70C	41C
101C	69C	44C
101T	70T	44T
102G	71G	45G
102C	73C	42C
101A	74A	41A
103G	74G	43G
103T	74T	40T
104T	76T	38T
104G	77G	36G
105C	75C	36C
104T	74T	35T
107T	74T	35T
108T	73T	34T
110G	74G	33G
116C	75C	33C
117T	75T	33T
118A	77A	33A
119A	77A	33A
119A	78A	31A

119A	79A	32A
118C	78C	32C
117T	77T	32T
115G	75G	32G
113A	75A	32A
115A	76A	31A
116A	75A	31A
114C	78C	32C
113T	78T	32T
114T	78T	34T
114G	79G	33G
109G	80G	34G
111G	81G	34G
112G	81G	36G
114A	83A	37A
114T	84T	36T
116A	84A	35A
117G	82G	36G
118A	84A	37A
118G	84G	37G
120T	85T	37T
120G	85G	37G
117C	82C	37C
116T	82T	37T
117C	83C	37C
116C	81C	35C
116T	80T	35T
110A	79A	36A
107C	78C	37C
108A	81A	38A
107T	81T	38T
107A	80A	39A
103T	80T	40T
103G	81G	39G
103G	83G	39G
104A	83A	38A
107G	85G	38G
108G	84G	41G
109T	82T	41T
107T	82T	40T
108C	82C	40C
109T	83T	39T
109G	82G	38G

108T	83T	35T
110C	80C	37C
113C	78C	37C
115A	78A	36A
115G	77G	36G
112C	77C	35C
110C	77C	35C
111C	76C	34C
110T	78T	32T
114T	76T	33T
111G	75G	32G
110T	73T	32T
110G	72G	32G
108G	71G	30G
105A	70A	20A_10G
103C	69C	30C
171A	174A_53T	85A_28T
63A	15A	1A
59A	19A	1A
41C	4C	null
74A	62A	30A
111A_37T_37G	61A	30A
39A	64A	31A
39C	64C	31C
49G	70G	39G
94C	72C	40C
94A	72A	42A
94C	73C	42C
97A	73A	42A
98G	76G	42G
97G	78G	42G
98G	79G	43G
99G	81G	44G
103C	84C	45C
103T	85T	45T
104A	84A	46A
102T	86T	45T
104A	86A	46A
104A	86A	46A
106A	87A	44A
104A	89A	43A
104A	89A	44A
107C	86C	44C

107C	89C	44C
106T	87T	44T
106A	87A	42A
107T	86T	44T
110T	85T	43T
109C	86C	43C
108T	84T	45T
109T	83T	45T
109C	81C	44C
107T	81T	47T
106C	44T_39C	47C
105G	41A_43G	19A_27G
107G	88G	44G
108T	88T	44T
109T	88T	43T
111A	90A	45A
114G	91G	45G
114A	90A	44A
115A	90A	44A
112A	90A	42A
110G	88G	44G
111C	89C	44C
113A	87A	44A
113G	87G	44G
111A	86A	46A
116C	85C	45C
115T	85T	45T
62A_57G	38A_49G	46G
124A	89A	47A
124C	90C	47C
127C	89C	47C
128C	88C	48C
127T	89T	48T
125G	91G	49G
127T	89T	48T
127C	91C	43C
127C	89C	46C
127A	88A	45A
126C	90C	46C
127A	88A	45A
124C	89C	45C
123A	87A	44A
122C	89C	44C

123A	92A	43A
122C	91C	43C
124C	90C	42C
121C	91C	41C
119G	86G	41G
121G	86G	41G
119C	86C	41C
119C	85C	39C
117G	84G	41G
114C	84C	40C
115C	83C	37C
116T	84T	39T
114T	86T	40T
113T	82T	40T
116G	82G	40G
116C	49G_34C	41C
115T	80T	40T
112A	80A	39A
113T	79T	38T
114G	79G	38G
112A	79A	38A
110A	79A	37A
109G	42T_34G	37G
107A	73A	39A
107A	72A	39A
106T	30T_42C	40T
105T	72T	39T
103T	73T	41T
102G	74G	41G
99G	76G	41G
98A	76A	40A
93A	78A	39A
93G	78G	39G
91A	78A	39A
89T	78T	40T
87G	78G	40G
88C	75C	39C
89T	74T	40T
89A	73A	38A
86T	72T	40T
84T	72T	41T
82C	73C	42C
82T	73T	41T

60A_89T_60G_60C	72T	40T
68G	71G	40G
46A	71A	40A
44A	71A	40A
46A	71A	37A
47C	69C	39C
46A	68A	39A
42A	66A	36A
43C	65C	37C
40C	67C	1A_16T_20C
39G	68G	39G
38C	67C	39C
39C	66C	39C
34A	66A	40A
37C	33T_34C	39C
35G	69G	40G
34G	69G	39G
34T	68T	38T
35G	68G	40G
35G	67G	40G
45T	66T	18A_39T_18G
46G	66G	21G
47G	35A_29G	21G
61T	64T	41T
61G	64G	40G
64G	65G	39G
64T	63T	39T
63G	61G	38G
63G	64G	39G
62G	62G	40G
64G	61G	39G
66A	61A	39A
66T	61T	39T
66A	59A	39A
69A	59A	38A
69T	58T	39T
68G	59G	39G
70G	61G	40G
71G	60G	39G
70G	58G	38G
70A	57A	39A
71C	59C	41C
76T	57T	41T

77G	57G	40G
79A	56A	41A
79A	55A	40A
81G	54G	41G
40T_43C	52C	41C
82C	53C	42C
85C	54C	40C
87C	55C	39C
88T	55T	40T
86A	58A	40A
83A	58A	39A
85A	60A	40A
84G	59G	39G
84A	57A	41A
86T	57T	40T
85A	55A	39A
83T	54T	39T
39T_45C	54C	18T_21C
81G	26A_27G	39G
83T	53T	39T
84T	51T	36T
83T	51T	35T
41T_40G	48G	15T_19G
82T	48T	33T
39T_44C	48C	14T_18C
85C	53C	33C
87C	53C	33C
91A	55A	32A
91A	54A	32A
91G	53G	32G
91C	53C	32C
91T	55T	32T
90T	55T	32T
89G	56G	31G
91G	56G	30G
91C	55C	31C
92T	54T	28T
91G	55G	28G
87A	55A	27A
87G	55G	27G
88A	55A	26A
87A	55A	25A
86G	54G	24G

88T	53T	24T
88A	53A	24A
90T	54T	22T
91T	54T	22T
92C	54C	22C
91G	56G	22G
90C	29T_26C	22C
91T	56T	20T
90G	56G	20G
89T	54T	21T
90T	56T	21T
90A	56A	21A
93G	53G	20G
93C	52C	20C
91C	53C	20C
86A	53A	20A
88C	53C	20C
89C	53C	20C
87C	54C	20C
88A	54A	20A
88G	54G	20G
86A	52A	19A
84G	53G	19G
85T	54T	19T
85A	53A	19A
83G	52G	16G
78A	54A	16A
78C	54C	16C
79T	53T	16T
78T	53T	15T
76C	54C	14C
74G	56G	16G
75G	55G	15G
73T	52T	15T
71C	51C	15C
70T	51T	16T
71A	47A	14A
71A	46A	13A
71A	46A	13A
71T	47T	13T
70G	46G	12G
69G	47G	12G
71G	49G	11G

71C	49C	11C
74A	49A	11A
74G	49G	12G
74G	49G	13G
72A	49A	13A
69T	49T	13T
68A	52A	14A
66T	53T	14T
65A	53A	14A
65A	53A	15A
65G	54G	15G
62T	52T	14T
66T	59T	15T
66A	59A	15A
65G	58G	15G
64T	56T	15T
63T	55T	15T
62A	56A	15A
58G	55G	15G
56T	55T	14T
56T	54T	14T
56A	52A	14A
54G	52G	14G
54T	55T	14T
52T	55T	14T
52A	54A	13A
52G	53G	14G
49T	53T	14T
48T	54T	13T
45A	53A	14A
21G_22C	52G	14G
42T	52T	14T
42T	49T	14T
42A	51A	14A
41G	50G	14G
41T	50T	14T
39T	50T	14T
38A	49A	15A
38G	49G	15G
35T	49T	15T
35T	49T	16T
35A	48A	16A
35G	47G	16G

34T	48T	16T
31T	48T	16T
31A	50A	15A
19G_11C	50G	16G
30T	47T	16T
31T	49T	16T
32A	49A	16A
31G	46G	16G
31T	47T	14T
31T	48T	15T
31A	45A	14A
30G	46G	15G
27T	46T	15T
27T	47T	15T
27A	46A	15A
26G	45G	13G
27T	43T	12T
27T	44T	13T
27A	44A	13A
27G	44G	11G
26T	45T	11T
26T	45T	12T
25A	44A	12A
18G	43G	12G
18T	42T	12T
17T	43T	12T
16A	43A	12A
16G	44G	12G
15T	44T	12T
15T	42T	11T
15A	42A	10A
13G	41G	10G
14T	41T	10T
14T	37T	9T
14A	35A	9A
12G	35G	10G
11T	33T	8T
11T	32T	8T
10A	32A	8A
7G	26G	8G
8T	25T	8T
8T	26T	8T
8A	26A	8A

7G	25G	8G
8T	24T	9T
8T	24T	9T
8A	24A	9A
9G	24G	10G
9T	25T	10T
9T	26T	10T
8A	24A	10A
7G	26G	10G
7A	27A	10A
7T	27T	10T
7A	27A	10A
7G	25G	10G
7A	25A	10A
7T	25T	10T
7A	25A	10A
7G	24G	10G
7A	23A	10A
7T	22T	10T
7A	21A	10A
7G	21G	9G
7A	20A	8A
7T	19T	8T
7A	18A	8A
7G	18G	6G
7A	18A	6A
7T	18T	6T
7A	18A	6A
7G	17G	6G
7A	17A	6A
7T	17T	1A_4T
7A	17A	5A
6G	16G	5G
5A	16A	5A
5T	16T	3T_2G
5A	16A	5A
5G	16G	5G
5A	16A	5A
5T	15T	4T
5A	14A	4A
4G	13G	4G
4A	13A	4A
4T	12T	4T

4A	12A	4A
2G	12G	4G
2A	12A	4A
2T	12T	4T
2A	11A	4A
2G	10G	3G
2A	11A	3A
2T	10T	3T
2A	15A	3A
2G	14G	3G
2A	12A	3A
2T	12T	3T
2A	12A	3A
2G	12G	3G
1A	12A	3A
1T	12T	3T
1A	12A	3A
1G	12G	3G
1A	13A	3A
1T	13T	2T
1A	13A	2A
1G	13G	1G
1A	13A	1A
1T	13T	1T
1A	13A	1A
1G	12G	1G
1A	12A	1A
1T	12T	1T
1A	12A	2A
1G	12G	2G
1A	12A	2A
null	12T	1T
null	12A	1A
null	10G	1G
null	9A	1A
1T	6T	1T
1A	5A	1A
1G	5G	1G
1A	5A	1A
1T	6T	1T
1A	6A	1A
1G	7G	1G
1A	7A	1A

1T	7T	1T
1A	7A	2A
1G	7G	2G
1A	7A	2A
1T	7T	2T
1A	7A	2A
1G	8G	2G
1A	8A	2A
1T	9T	2T
1A	10A	2A
1G	10G	2G
1A	10A	2A
1T	11T	2T
1A	11A	2A
1G	11G	2G
1A	11A	2A
1T	11T	2T
1A	11A	2A
1G	11G	2G
1A	10A	2A
1T	11T	2T
1A	11A	2A
1G	11G	2G
1A	11A	2A
1T	11T	2T
1A	11A	2A
1G	11G	2G
1A	10A	2A
1T	11T	2T
3A	11A	2A
2A_1G	11G	2G
3A	11A	2A
3T	11T	2T
3A	11A	3A
2A_1G	11G	1A_1T_1G
6A	8A	5A
6T	10T	7T
6A	11A	7A
6A	10A	7A
7A	11A	8A
7T	11T	7T
7A	12A	7A
7A	12A	7A

7A	13A	7A
7T	14T	8T
7A	14A	9A
7A	16A	10A
8A	16A	10A
8T	17T	10T
10A	19A	10A
10A	21A	10A
10A	21A	10A
10T	21T	11T
11A	21A	11A
11A	21A	11A
11A	21A	12A
11T	22T	12T
14A	23A	12A
14A	23A	12A
14A	23A	12A
16T	23T	12T
16A	23A	13A
17A	23A	14A
17A	23A	14A
19T	23T	14T
21A	24A	15A
22A	23A	15A
23A	23A	15A
25T	22T	15T
26A	22A	15A
27A	22A	15A
28A	22A	16A
29T	23T	17T
31A	23A	18A
31G	24G	18G
32G	24G	17G
33G	26G	18G
35T	27T	20T
36A	26A	22A
38T	9T_18C	22T
40G	28G	23G
41A	29A	23A
43T	29T	23T
43C	30C	22C
43C	30C	23C
45A	30A	23A

46G	30G	26G
45A	30A	27A
48T	30T	27T
48C	33C	27C
48A	34A	28A
48A	35A	28A
50G	35G	28G
51C	34C	28C
53C	35C	28C
52A	35A	28A
54G	35G	31G
54G	35G	29G
54G	36G	30G
52A	36A	30A
53A	37A	31A
54C	37C	31C
54A	38A	31A
54G	39G	32G
54A	38A	32A
54T	40T	32T
55G	39G	30G
55T	38T	29T
55T	38T	30T
56G	37G	29G
56T	40T	30T
56C	39C	30C
56T	41T	30T
56T	40T	29T
56G	40G	29G
57G	41G	29G
59A	40A	28A
58A	40A	28A
58G	40G	28G
56A	39A	30A
56G	38G	30G
57A	38A	29A
59C	39C	30C
61G	42G	31G
61C	42C	31C
63A	42A	31A
63G	42G	31G
61A	41A	31A
62C	42C	31C

63A	42A	31A
63A	41A	31A
64G	42G	31G
63C	42C	30C
65A	42A	30A
62A	42A	31A
62C	43C	31C
61T	42T	32T
61G	43G	33G
60T	43T	33T
60G	43G	33G
59G	43G	31G
58G	44G	29G
57A	45A	29A
56G	46G	29G
56G	47G	28G
55G	46G	28G
57A	43A	28A
56G	43G	27G
55A	46A	25A
55G	45G	25G
53G	44G	25G
52A	43A	24A
50G	44G	27G
50G	43G	27G
33A_17G	43A	27A
49T	43T	27T
49G	43G	23G
49T	43T	23T
47T	44T	24T
47C	42C	25C
49C	42C	23C
49T	42T	25T
47T	43T	25T
46G	43G	25G
43C	45C	26C
43A	45A	26A
41A	45A	24A
42A	47A	26A
42C	46C	25C
42T	20T_26C	25T
42A	45A	23A
41C	45C	13T_12C

44G	44G	25G
44T	44T	24T
46C	45C	24C
46A	45A	24A
45T	45T	25T
45G	45G	25G
45C	44C	25C
46C	46C	25C
46T	43T	25T
46C	44C	24C
52G	43G	26G
52G	42G	26G
53G	42G	27G
53C	40C	26C
52A	40A	26A
53G	40G	25G
53A	38A	25A
55C	38C	26C
55C	37C	27C
55C	36C	26C
55A	36A	28A
52T	36T	27T
53C	36C	27C
51G	37G	27G
53C	37C	29C
53A	37A	29A
52G	39G	29G
52T	40T	30T
51G	41G	30G
50G	41G	28G
49G	42G	29G
47G	42G	29G
48C	42C	29C
47A	40A	28A
48G	40G	27G
48A	40A	25A
47G	40G	26G
46G	40G	26G
49A	42A	27A
50G	41G	27G
50A	39A	27A
50A	39A	27A
52G	37G	26G

53A	38A	27A
51G	38G	28G
52G	37G	29G
53G	36G	29G
52G	37G	30G
51A	37A	29A
53A	35A	30A
54G	35G	28G
55C	12G_21C	8G_19C
56C	34C	27C
56A	35A	27A
57A	36A	27A
57A	35A	26A
59G	11A_25G	9A_17G
59A	24A_11T	17A_8T
56A	40A	25A
58C	41C	25C
60C	41C	25C
61A	41A	25A
61G	39G	24G
62A	39A	25A
60A	39A	23A
62G	38G	24G
62C	40C	24C
63C	41C	23C
62C	17T_24C	8T_15C
64C	42C	23C
65A	44A	23A
66C	43C	24C
64G	44G	24G
65T	44T	26T
66C	44C	25C
67C	44C	15A_11C
68A	45A	27A
68G	46G	27G
69C	47C	25C
70A	47A	27A
66A	47A	27A
36T_30C	48C	28T
65G	50G	29G
67C	51C	29C
68A	52A	30A
67G	53G	32G

68C	53C	32C
65T	54T	31T
66G	56G	30G
65C	57C	30C
63A	59A	28A
63G	57G	26G
65C	58C	27C
66A	58A	27A
65G	60G	26G
64A	59A	26A
66G	57G	27G
65A	56A	29A
65G	55G	30G
66C	55C	30C
66T	55T	30T
66T	55T	30T
65T	55T	29T
66T	54T	31T
64C	55C	31C
67C	56C	33C
69C	58C	36C
69C	58C	36C
65C	59C	36C
68G	60G	36G
70G	60G	36G
70C	60C	36C
69T	60T	38T
67G	61G	39G
70C	61C	39C
69C	62C	39C
69A	61A	38A
68A	60A	38A
67A	60A	37A
66A	61A	37A
67A	61A	37A
68T	60T	37T
70A	60A	37A
70C	59C	38C
70T	61T	38T
71C	59C	38C
70T	62T	39T
70G	63G	40G
70G	62G	39G

70C	61C	40C
69A	61A	40A
67A	62A	39A
67G	65G	42G
67G	66G	42G
66A	67A	40A
66A	66A	40A
65T	63T	40T
65C	66C	41C
65A	65A	43A
65G	66G	42G
63G	67G	45G
60A	66A	45A
61A	66A	44A
62G	66G	42G
63A	63A	42A
65G	66G	43G
61A	65A	42A
63G	64G	42G
63G	65G	43G
63A	64A	42A
64G	62G	41G
63A	61A	41A
67G	59G	38G
66G	62G	38G
1A_62T_34G	60T_46G	39T
67G	61G	37G
69G	62G	38G
69G	61G	40G
70G	60G	40G
71G	60G	42G
71G	59G	42G
72T	56T	44T
73C	57C	46C
73A	56A	45A
73T	53T	45T
75G	53G	45G
76C	54C	45C
74T	54T	43T
75G	54G	44G
77A	55A	43A
77C	56C	45C
78C	57C	45C

77C	60C	48C
78A	60A	47A
78G	58G	45G
78A	59A	44A
77T	57T	42T
77C	62C	44C
74A	60A	45A
73A	62A	45A
75A	63A	46A
75A	64A	46A
76C	63C	45C
76C	62C	44C
74C	62C	43C
74G	29A_33G	21A_21G
73A	62A	42A
74C	62C	43C
76G	61G	22A_21G
77A	63A	43A
75A	63A	44A
73A	62A	44A
73G	59G	45G
72G	61G	44G
71C	60C	44C
70A	61A	45A
70G	60G	45G
68G	60G	45G
68A	63A	45A
69T	64T	48T
72G	64G	48G
72A	64A	48A
73G	63G	47G
75C	64C	47C
77G	63G	47G
80C	63C	48C
78T	63T	47T
82C	63C	47C
83A	63A	46A
82G	63G	47G
84C	26T_35C	43C
83G	61G	46G
84G	61G	46G
85T	62T	42T
82C	64C	46C

86C	66C	46C
88A	65A	45A
87C	65C	47C
86G	66G	44G
84T	66T	47T
86G	27G_39C	47G
86A	68A	48A
86A	68A	48A
86G	66G	48G
85A	69A	47A
86G	72G	46G
85T	71T	44T
84C	72C	44C
85C	73C	44C
84C	71C	43C
84A	72A	44A
82G	76G	41G
83A	76A	40A
82G	76G	40G
42G_40C	79G	40G
79G	79G	38G
82A	79A	38A
81A	82A	37A
81G	83G	37G
80A	82A	36A
84A	82A	35A
83A	81A	34A
85G	79G	32G
84A	79A	31A
82G	79G	33G
80G	78G	35G
79C	79C	35C
41A_39C	39A_35C	32A
80A	74A	31A
78G	76G	31G
77G	76G	30G
67A_79G	31A_75G	24A_31G
9A_4G	44A	4A_3G
85G	76G	32G
85A	77A	33A
85G	76G	31G
83A	78A	32A
77G	77G	32G

79A	78A	33A
81A	77A	35A
82G	78G	36G
82C	80C	37C
85C	81C	37C
84A	81A	38A
86C	82C	40C
86G	80G	40G
87G	81G	40G
87A	81A	41A
86A	79A	40A
87A	78A	39A
88C	76C	39C
86C	75C	37C
85C	76C	39C
88A	76A	41A
86G	77G	40G
86T	76T	41T
86G	77G	43G
82G	74G	42G
82G	76G	42G
81G	76G	43G
80T	75T	43T
81G	77G	42G
81G	80G	44G
83C	76C	43C
81C	79C	43C
79C	78C	43C
79C	76C	43C
78A	78A	41A
33T_39C	78C	43C
69G	78G	41G
68G	79G	42G
68C	78C	41C
37A_28G	41T_32G	40G
28A_36G	30A_42G	39A
67A	71A	41A
67G	71G	41G
65G	71G	42G
68G	71G	44G
69A	71A	45A
70G	72G	46G
70G	71G	47G

71T	70T	45T
71G	72G	45G
69A	69A	45A
71G	67G	47G
71C	69C	47C
38T_24C	70T	49T
63G	70G	49G
22T_39G	69G	50G
41A_22G	39A_27G	50G
22T_41C	65C	24T_27C
75C	67C	51C
76A	65A	52A
78T	69T	54T
77T	69T	57T
78C	69C	58C
77C	70C	54C
79C	71C	53C
78C	72C	53C
79A	71A	53A
79A	70A	53A
81A	72A	52A
81G	72G	50G
80A	72A	50A
79G	72G	51G
79G	72G	51G
79T	72T	50T
80G	32A_39G	48G
82G	71G	47G
83A	71A	45A
85G	39A_32G	45G
85T	71T	44T
82G	70G	44G
81G	70G	42G
79A	70A	42A
79T	70T	41T
79G	66G	40G
79T	65T	40T
83C	67C	41C
82G	69G	41G
82C	68C	41C
82A	66A	41A
83G	65G	39G
83C	63C	37C

87C	63C	36C
89C	62C	35C
89C	63C	34C
91A	63A	35A
93T	62T	35T
94A	62A	36A
93G	62G	36G
94A	64A	37A
98A	62A	37A
96G	63G	37G
98G	62G	39G
96G	59G	39G
99A	59A	37A
100G	57G	38G
99A	58A	37A
103T	61T	38T
103G	60G	38G
103C	61C	39C
106C	61C	40C
107C	63C	40C
105T	65T	41T
105C	65C	43C
105A	65A	42A
102C	64C	42C
101T	66T	41T
100C	65C	40C
96T	64T	40T
93G	64G	40G
92G	64G	40G
94A	63A	40A
92C	61C	38C
91A	61A	38A
90G	60G	36G
87A	61A	36A
84G	62G	37G
83C	62C	37C
84A	63A	36A
84A	63A	36A
83G	64G	35G
83G	60G	33G
83C	60C	30C
82C	60C	33C
84G	60G	33G

84T	59T	33T
84G	57G	34G
83G	56G	34G
84G	55G	35G
83G	54G	35G
82C	55C	35C
83T	52T	33T
51C	48C	26C
51T	47T	27T
54T	48T	27T
56C	47C	27C
55T	48T	27T
54G	49G	27G
53C	49C	27C
53C	49C	27C
53A	48A	28A
56C	50C	28C
57C	49C	28C
57C	50C	27C
57C	50C	28C
58T	50T	27T
57G	51G	27G
58G	49G	27G
58G	49G	27A
57C	50C	26C
56A	50A	26A
55A	50A	26A
52G	49G	27G
51C	49C	27C
51A	49A	27A
51G	49G	27G
52G	49G	27G
50G	50G	25G
51G	51G	22G
51A	48A	22A
48T	48T	22T
50C	47C	23C
50C	47C	24C
50T	46T	24T
50C	46C	24C
49A	45A	24A
50G	44G	24G
50T	42C	24T

50G	43G	24G
51G	41G	22G
49G	41G	21G
50G	39G	21G
51A	38A	19A
52A	37A	18A
52C	38C	17C
51C	39C	16C
51C	37C	15C
52G	37G	16G
53T	38T	15T
53C	37C	16C
55G	37A	16G
52A	39A	15A
52G	39G	16G
53A	40A	17A
53C	39C	17C
53A	40A	16G
53G	39G	16G
53A	40A	17A
53C	39C	17C
52A	38A	17A
49C	37C	18C
49A	37A	19A
50C	37C	19C
50A	35A	15A
49A	35A	16A
47G	35G	19G
48G	35G	18G
49G	34G	17G
45G	34G	17G
46C	32C	17C
46G	34G	17G
46A	32A	16A
46T	29T	18T
46G	30G	18G
46G	28G	18G
42G	29G	18G
44A	27A	20A
44A	27A	19A
47A	27A	20A
48A	28A	20A
48C	29C	20C

45A	27G	20G
45C	28C	20C
46T	27T	22T
48C	27C	23C
47T	28T	23T
48C	27C	23C
48C	27C	23C
49A	27A	23A
50C	27C	23C
50A	28A	23A
49T	28T	23T
50G	28G	23G
49C	28C	23C
49T	27T	23T
51C	27C	23C
50A	27A	24A
51G	26G	25G
51A	26A	24A
51A	26A	25A
52A	26A	25A
53T	26T	24T
52A	26A	22A
53G	26G	22G
50T	26T	22T
50G	27G	21G
51T	31T	22T
50C	31C	23C
53C	30C	23C
52C	31C	23C
52C	31C	23C
52T	31T	23T
53T	31T	22T
52C	31C	23C
52G	30G	23G
52T	30T	23T
51T	31T	23T
53A	32A	22A
52T	31T	23T
54T	32T	22T
52G	32G	22G
53C	30C	22C
54T	30T	22T
53G	28G	21G

54A	27A	21A
54A	27A	21A
54T	27T	22T
54G	25G	22G
53C	23C	20C
53C	24C	20C
54A	24A	18A
51C	25C	18C
52G	25G	18G
54G	25G	18G
53A	24A	18A
54G	24G	15G
55C	25C	15C
53C	25C	15C
57A	26A	17A
55A	26A	17A
55A	23A	18A
54A	23A	18A
56A	24A	17A
56G	26G	17G
54A	26A	17A
55G	25G	18G
54G	25G	16G
53T	24T	16T
54G	29G	15G
54C	28C	15C
83T	48T	28T
83A	49A	27A
83C	49C	28C
83G	50G	26G
83A	51A	26A
86A	50A	28A
86A	52A	28A
84A	51A	29A
84T	53T	29T
85G	52G	30G
87T	52T	31T
88T	53T	32T
87C	54C	31C
90C	55C	32C
93T	58T	33T
92G	58G	34G
92G	58G	33G

91A	60A	35A
97T	61T	36T
97T	63T	36T
96C	64C	37C
99A	65A	38A
99A	65A	19A_20G
98C	65C	40C
101C	65C	39C
53A_48G	64A	40A
99A	60A	42A
102C	62C	41C
100C	62C	42C
101T	61T	43T
103T	62T	43T
104G	63G	44G
107C	63C	45C
109A	63A	46A
108A	61A	46A
111A	64A	47A
110C	62C	46C
109A	62A	46A
108G	62G	45G
105A	60A	43A
104C	59C	44C
103C	58C	44C
101T	56T	43T
101C	57C	41C
99A	57A	41A
100T	60T	43T
100G	60G	41G
99T	59T	41T
94G	58G	39G
95T	52T	38T
94C	53C	39C
94T	51T	39T
94G	51G	39G
94T	66T_5G_15C	36T
90C	43C	37C
89T	46T	37T
6G_7C	27C	5G_6C
13T	28T	11T
13G	27G	9G
10T	27T	6T

3G_7C	29C	6C
11T	33T	6T
10C	33C	7C
20T	34T	7T
34G	46G	21G
34T	45T	21T
34G	46G	21G
34T	46T	21T
34G	46G	21G
34T	46T	21T
34G	45G	21G
34T	45T	21T
34G	40G	21G
34T	45T	21T
34G	43G	21G
34T	44T	21T
34G	43G	20G
34T	43T	20T
34G	42G	20G
34T	42T	20T
34G	41G	20G
33T	39T	20T
33G	37G	19G
33T	36T	20T
31G	36G	20G
30T	36T	20T
29G	36G	19G
29T	35T	19T
28G	32G	19G
28T	3A_32T_2G_3C	19T
24G	30G	19G
24T	32T	19T
23G	31G	16G
23T	30T	16T
23G	28G	15G
22T	29T	15T
22G	27G	13G
22T	28T	13T
22G	26G	13G
22T	28T	13T
21G	27G	12G
21T	26T	12T
20G	25G	11G

20T	24T	11T
19G	20G	11G
19T	21T	11T
19G	20G	10G
19T	21T	11T
19G	21G	10G
19T	21T	10T
19G	21G	7G
19T	21T	6T
19G	21G	5G
18T	21T	6T
18G	19G	6G
26T	31T	14T
24G	32G	15G
26T	32T	15T
24G	33G	14G
24T	33T	14T
25G	33G	17G
28T	33T	18T
29G	33G	20G
32T	34T	20T
34G	35G	20G
35T	36T	20T
35G	37G	20G
36T	38T	20T
38G	40G	23G
40T	40T	23T
40G	39G	23G
40T	40T	23T
41G	39G	22G
44T	36T	24T
43G	29G	22G
49T	31T	23T
52G	32G	23G
54T	33T	23T
55G	33G	23G
56T	35T	24T
59G	35G	25G
59T	36T	25T
61G	36G	26G
60T	36T	26T
62G	37G	27G
62T	38T	27T

64G	38G	27G
65T	38T	27T
67G	37G	29G
69T	39T	29T
69G	39G	30G
70T	39T	30T
71G	38G	32G
73T	39T	33T
74G	40G	33G
75T	40T	34T
76G	40G	36G
77T	41T	37T
79G	40G	40G
79T	42T	41T
83G	42G	42G
84T	45T	44T
85G	44G	45G
88T	46T	46T
88G	46G	46G
90T	48T	47T
93G	48G	49G
95T	48T	48T
95G	46G	49G
96T	48T	49T
97G	48G	51G
97T	49T	51T
97G	48G	52G
97T	49T	52T
99G	48G	52G
100T	48T	53T
101G	48G	53G
100T	50T	53T
103G	47G	53G
104T	50T	53T
104G	50G	53G
104T	52T	52T
104G	53G	53G
105T	53T	53T
105G	53G	54G
84T	38T	36T
71C	31C	32C
68C	30C	33C
33A_22T_40G_33C	18A_12G	2A_15G_10C

32T_11C	22T_8C	25T
49G	32G	28G
50C	30C	28C
41A	27A	26A
42T	27T	28T
43C	27C	29C
41T	28T	30T
56C	29C	30C
56C	29C	31C
57T	30T	28T
58T	28T	29T
17T_40C	29C	32C
61G	29G	33G
61G	28G	33G
59A	29A	32A
60C	30C	32C
61A	29A	32A
62A	29A	33A
64G	30G	32G
65C	29C	33C
65A	29A	32A
62A	29A	30A
63T	30T	31T
62T	30T	31T
62T	33T	31T
65A	34A	29A
42G_24C	33C	31C
67A	36A	31A
68G	36G	31G
69C	37C	29C
69C	37C	29C
70G	37G	28G
69C	38C	30C
69C	38C	29C
68A	38A	28A
69G	38G	28G
70G	38G	27G
69A	38A	26A
67G	38G	25G
69C	38C	25C
73T	38T	24T
72T	38T	27T
72C	38C	24C

72C	38C	23C
71A	39A	24A
71T	38T	23T
70T	39T	24T
71T	39T	24T
71G	39G	25G
71C	40C	26C
71T	40T	26T
71G	39G	25G
71G	39G	25G
72G	40G	25G
32A_44G	41G	24G
76G	41G	25G
75C	42C	26C
44A_32G	41G	26G
76A	41A	27A
76A	41A	27A
76A	41A	28A
79G	40G	27G
79T	39T	28T
79C	38C	28C
33A_44C	36A	28A
78C	37C	24C
75T	35T	28T
75C	23T_12C	28C
76G	34G	28G
76T	33T	27T
74A	33A	27A
73A	32A	28A
71T	32T	28T
72A	31A	28A
72G	31G	27G
72A	31A	31A_10C
29A_70C	19A_29C	17A_24C
39A_34C	26C	24C
76C	28C	26C
76C	28C	26C
79C	28C	27C
80C	28C	26C
77C	28C	25C
76C	28C	25C
79C	26C	25C
71A	24A	24A

75C	25C	25C
71C	25C	26C
67T	25T	25T
68G	25G	26G
71C	25C	26C
70C	25C	25C
67A	26A	26A
67G	24G	27G
66A	21A	25A
69C	22C	28C
75C	21C	28C
74G	19G	29G
74C	21C	29C
75A	21A	28A
78G	20G	27G
78C	19C	27C
81C	20C	29C
82C	20C	29C
83C	20C	29C
82A	20A	31A
81C	20C	30C
79A	18A	30A
82C	20C	29C
77C	20C	27G
78C	20C	27C
72A	19A	27A
78C	20C	26C
79C	24C	27C
78T	22T	27T
79G	20G	25G
80C	19C	26C
80T	21T	26T
79T	21T	25T
78G	21G	25G
78G	21G	24G
77A	22A	24A
74T	21T	23T
73A	20A	21A
70T	22T	21T
71A	23A	21A
71C	24C	21C
70G	25G	20G
69G	25G	21G

67A	25A	22A
65G	28G	22G
67T	28T	23T
68T	29T	23T
72T	30T	23T
72A	32A	23A
72T	33T	22T
72T	33T	23T
72A	34A	22A
72A	34A	24A
72A	33A	24A
72T	33T	23T
73T	36T	24T
73A	36A	24A
73T	38T	26T
71G	39G	26G
69T	40T	24T
70T	40T	23T
70T	40T	23T
67T	41T	23T
67T	41T	23T
64T	41T	22T
64A	41A	22A
64A	43A	22A
64A	43A	24A
63G	43G	24G
61G	43G	24G
61C	42C	22C
63T	41T	21T
63G	41G	21G
63C	41C	22C
63A	40A	24A
64C	40C	24C
65T	39T	25T
64T	39T	24T
65G	39G	23G
64G	39G	22G
59A	38A	22A
59A	38A	22A
58A	37A	22A
58G	37G	23G
59G	37G	24G
58G	38G	24G

56C	38C	23C
55A	39A	24A
55G	40G	24G
56A	39A	22A
58A	40A	22A
61A	40A	23A
61T	40T	23T
62A	40A	24A
63G	40G	25G
62G	39G	25G
62C	39C	25C
62C	35C	25C
64A	36A	25A
64G	36G	26G
68A	38A	26A
66G	36G	26G
67T	37T	27T
68T	40T	26T
70T	40T	28T
71A	41A	29A
72A	41A	29A
71G	41G	30G
71G	41G	30G
72G	41G	29G
73C	40C	29C
74G	38G	30G
77A	40A	30A
78A	39A	30A
77A	37A	30A
77T	38T	28T
77C	37C	29C
73C	38C	30C
74C	36C	30C
74A	35A	30A
74G	35G	29G
72G	36G	29G
72G	37G	28G
74C	38C	28C
76A	38A	28A
73A	36A	28A
73T	36T	28T
75C	34C	27C
75T	34T	27T

77C	34C	27C
77T	34T	28T
78T	34T	28T
79C	33C	29C
80C	33C	29C
79A	33A	29A
80C	34C	30C
81G	14A_18G	29G
82T	32T	27T
79T	32T	28T
81G	32G	28G
82G	32G	28G
80C	33C	28C
82T	32T	29T
83G	32G	29G
81C	33C	27C
82C	34C	27C
81A	35A	26A
82G	35G	26G
81A	36A	25A
83C	36C	27C
83T	36T	27T
83C	36C	26C
83T	36T	25T
80C	20T_16C	24C
80C	40C	24C
81A	39A	24A
81G	39G	24G
46T_38C	39C	19T_5C
83C	38C	24C
82C	37C	24C
80C	38C	24C
77T	38T	23T
78G	36G	23G
79C	35C	22C
78A	37A	21A
81A	37A	21A
81A	37A	21A
82T	38T	22T
81C	37C	21C
82T	37T	21T
76T	35T	21T
76C	35C	20C

74T	34T	20T
77C	32C	20C
76C	32C	19C
75C	30C	18C
73A	30A	17A
75T	30T	17T
75C	28C	17C
73A	27A	17A
72G	27G	17G
74C	28C	16C
71C	27C	15C
72T	27T	15T
72T	29T	15T
70G	29G	15G
70A	29A	15A
72C	28C	14C
71A	28A	13A
70C	29C	14C
72T	30T	14T
72T	28T	14T
71C	28C	13C
69T	28T	13T
67T	29T	13T
67A	29A	12A
67T	29T	13T
66T	29T	12T
68T	28T	12T
66C	27C	12C
65A	27A	11A
63G	29G	11G
62C	29C	10C
60T	29T	10T
59C	29C	11C
57A	28A	10A
59C	29C	11C
58T	29T	11T
58T	29T	11T
57A	29A	12A
56G	29G	12G
57A	29A	12A
56G	29G	11G
55A	29A	10A
55G	28G	10G

54G	26G	10G
54G	26G	9G
56T	26T	10T
56A	25A	10A
53T	25T	10T
52T	25T	10T
52T	25T	10T
50A	25A	10A
51T	24T	9T
52G	20G	9G
51G	20G	9G
51G	20G	9G
48T	19T	9T
49G	19G	10G
49T	20T	11T
49C	20C	11C
49A	20A	11A
48T	20T	11T
46C	21C	11C
45T	20T	12T
42C	21C	12C
41T	22T	12T
41G	21G	11G
38A	21A	12A
38C	21C	12C
38C	21C	12C
39C	21C	12C
39T	22T	11T
37T	22T	11T
39C	23C	11C
42C	23C	11C
43T	23T	11T
42C	24C	12C
42T	23T	12T
42C	24C	13C
43C	24C	12C
40A	22A	12A
40C	22C	12C
39C	22C	12C
39T	20T	13T
39C	19C	13C
16G_24C	10G_10C	13G
38G	20G	14G

39A	22A	14A
40A	21A	14A
38C	21C	14C
38G	9A_2T_9G_1C	14G
38C	21C	15C
37T	20T	15T
37T	19T	15T
38G	18G	15G
38G	19G	14G
36C	19C	14C
34C	19C	14C
33T	19T	14T
32G	19G	14G
32C	17C	14C
32T	17T	14T
32T	17T	14T
25A_2T_51C	13C	13C
5C	9A_7C	13A
7C	16C	20C
9T_21C	17C	4C
32C	17C	11C
32C	17C	11C
33C	17C	11C
32C	16C	11C
31C	16C	10C
32C	16C	11C
30C	16C	9C
26C	17T	9T
31T	17T	12T
31T	18T	11T
33T	18T	10T
33T	18T	10T
33T	18T	10T
33T	18T	11T
34T	19T	11T
34G	19G	11G
34C	19C	11C
34C	20C	13C
34A	20A	13A
32C	21C	13C
34A	21A	13A
33C	21C	13C
33G	21G	12G

32A	22A	12A
34C	22C	11C
35T	21T	11T
36C	20C	11C
35C	21C	12C
35C	20C	12C
37T	20T	12T
37C	20C	12C
36C	20C	13C
35A	20A	12A
36T	21T	12T
36A	21A	12A
36A	20A	12A
35A	19A	12A
34C	19C	12C
35A	18A	12A
33A	18A	12A
29G	18G	11G
30C	18C	11C
30A	18A	11A
30C	18C	11C
30G	17G	11G
29G	18G	11G
29C	18C	11C
29T	18T	11T
29G	16G	10G
27C	15C	10C
28C	14C	10C
27C	14C	9C
27A	13A	9A
26G	13G	9G
25A	13A	9A
25C	10T_3C	9C
25C	12C	8C
24C	12C	8C
25C	12C	2G_6C
25A	12A	8A
24G	12G	8G
24G	12G	8G
24C	12C	8C
24C	12C	8C
23T	12T	8T
23C	12C	8C

23C	12C	8C
22C	12C	8C
23C	12C	9C
22C	11C	8C
22C	11C	9C
23C	11C	9C
15A_7C	8A_3C	9C
22C	11C	9C
22A	11A	9A
22C	11C	9C
15A_7G	11G	8G
22C	10C	9C
22C	10C	10C
24A	10A	10A
23T	10T	10T
20A	10A	10A
20C	10C	11C
20A	10A	10A
19C	9C	10C
19A	9A	10A
20C	8C	11C
20C	9C	9C
20T	8T	9T
21C	7C	9C
21C	7C	9C
23G	6G	9G
25T	6T	9T
18T_8C	5T	9T
25C	5C	9C
24T	6T	10T
24A	6A	10A
24T	5T	9T
24T	5T	8T
22A	5A	9A
22G	5G	9G
22T	5T	9T
22C	5C	10C
21T	4T	10T
22C	3C	10C
21G	3G	10G
22C	3C	10C
22C	3C	10C
21T	3T	10T

20T	3T	9T_4C
20C	3C	11C
20C	3C	11C
19C	3C	11C
20C	3C	10C
21C	3C	10C
20C	4C	10C
20C	4C	10C
21C	5C	7A_3C
20C	5C	10C
13A_9C	5C	11C
20A	5A	11A
21A	5A	12A
22G	5G	10G
22G	5G	10G
22C	5C	13C
21T	5T	13T
23G	5G	12G
23G	7G	14G
27C	7C	15C
27G	7G	15G
27G	8G	17G
27A	11A	16A
30G	13G	16G
33C	13C	18C
33C	13C	19C
36T	13T	19T
37G	13G	19G
37T	13T	18T
36A	13A	18A
39A	14A	19A
41T	16T	20T
41T	17T	20T
41C	18C	20C
40T	18T	20T
41C	18C	20C
42C	17C	20C
43C	19C	20C
42A	19A	20A
42G	19G	19G
41C	19C	7T_11C
43G	20G	17G
42G	20G	18G

42C	20C	20C
44T	20T	19T
44G	21G	20G
43A	23A	19A
44C	24C	19C
45A	24A	19A
46C	24C	20C
45C	24C	20C
45A	25A	20A
44C	25C	20C
45A	25A	20A
45G	25G	20G
46G	25G	19G
45G	24G	19G
47A	25A	17A
46T	25T	19T
46G	25G	19G
47G	26G	18G
48C	26C	17C
49G	26G	17G
50G	27G	17G
50C	27C	16C
51A	28A	16A
50G	28G	16G
52G	28G	17G
52A	28A	18A
54C	28C	18C
54A	28A	18A
56T	28T	19T
58C	30C	19C
57A	32A	19A
58C	32C	19C
58T	32T	19T
58C	31C	19C
57T	31T	19T
60T	31T	19T
61C	31C	19C
60A	31A	18A
61G	34G	18G
63G	28G	18G
64G	31G	19G
64G	15G_15C	20G
63G	15G_15C	19G

63C	12G_18C	11G_8C
63C	30C	19C
64C	30C	17C
60C	30C	17C
61C	15T_29C	16C
64C	28C	15C
61C	26C	15C
61T	25T	15T
58T	23T	14T
58T	23T	13T
55C	23C	13C
76A_92C	18A_32C	7A_13C
16A	13A	6A
16C	13C	13C
13A	12A	12A
53C	28C	11C
52A	26A	11A
51C	26C	11C
51A	26A	12A
51C	27C	12C
50A	27A	12A
51C	27C	12C
50A	28A	12A
51C	27C	12C
53A	26A	12A
52C	26C	12C
52A	26A	8A_3C
50C	21C	9C
51A	21A	8C
52C	21C	8C
52A	21A	9A
53T	21T	10T
53G	21G	10G
54G	21G	10G
55A	21A	10A
58T	21T	12T
59T	21T	12T
59G	21G	12G
59G	21G	12G
58C	21C	15C
57A	23A	15A
55G	23G	16G
55G	24G	16G

53C	25C	17C
54G	26G	16G
52A	26A	16A
52A	26A	17A
53G	26G	17G
52G	26G	17G
51C	27C	16C
51T	28T	18T
50C	28C	18C
56T	27T	18T
56C	27C	18C
59T	29T	17T
59C	32C	18C
59G	34G	18G
57T	36T	15T
59T	36T	18T
58T	35T	19T
58G	33G	19G
60T	32T	19T
60C	31C	20C
61A	31A	20A
62A	31A	21A
63C	30C	21C
60G	30G	22G
60C	31C	22C
60C	31C	21C
62G	31A	14A_6G
60G	32G	21G
57A	31A	22A
59C	31C	23C
60T	32T	23T
63G	32G	23G
65A	29A	23A
67C	30C	24C
68A	30A	24A
67G	30G	26G
66A	32A	27A
66A	33A	27A
67T	34T	27T
28T_38G	35T	27T
66A	36A	28A
67C	38C	29C
67T	38T	29T

69C	39C	29C
70G	39G	28G
71C	39C	28C
71C	38C	28C
72C	38C	28C
72T	39T	27T
72C	39C	28C
73A	39A	32A
71A	39A	33A
74A	40A	34A
75T	41T	34T
75G	42G	34G
75A	43A	34A
76A	43A	36A
78A	43A	35A
77C	44C	34C
81G	45G	34G
81A	46A	35A
80T	46T	36T
78C	49C	34C
79T	49T	35T
81A	51A	36A
82T	50T	35T
82C	51C	33C
84T	50T	34T
86G	52G	33G
89G	51G	33G
91G	52G	35G
90G	51G	36G
95G	51G	36G
94A	51A	35A
95G	52G	36G
96T	53T	35T
96T	53T	37T
96A	53A	36A
98A	53A	36A
89A	54A	36A
93C	54C	35C
91C	52C	36C
91T	50T	35T
92A	50A	35A
94T	48T	35T
92T	50T	32T

91T	53T	32T
89C	55C	32C
88C	56C	32C
90A	57A	32A
88G	58G	32G
86T	59T	31T
87G	58G	30G
86G	59G	30G
86A	58A	30A
86G	58G	31G
84C	59C	31C
42T_43C	58T	31T
85G	59G	30G
85A	58A	29A
85G	56G	30G
83A	56A	31A
81A	57A	31A
80G	57G	30G
79A	56A	30A
78G	56G	28G
77A	53A	27A
76G	27G_26C	27G
75A	52A	28A
78G	52G	28G
77A	51A	27A
76G	49G	26G
77G	49G	26G
77G	48G	27G
75T	48T	27T
75T	47T	28T
77T	49T	28T
76T	48T	28T
76A	26A	28A
76G	26G	28G
74G	28G	24G
75G	29G	23G
72G	28G	22G
73G	42G	22G
70G	40G	22G
69G	40G	22G
65A	39A	19A
65C	40C	20C
64C	41C	22C

61C	41C	22C
62A	41A	21A
61G	41G	20G
60C	41C	21C
63A	41A	20A
61A	40A	19A
60T	39T	19T
60G	39G	19G
57T	35T	18T
57C	39C	18C
54A	39A	16A
51A	39A	15A
51G	38G	14G
49A	37A	15A
49G	38G	15G
49C	37C	15C
47C	39C	15C
50C	38C	14C
48A	38A	14A
47A	39A	13A
46T	38T	13T
45T	39T	13T
43A	39A	13A
39A	38A	13A
42T	38T	13T
40T	41T	13T
36T	36T	13T
40G	38G	13G
40G	38G	11G
39G	38G	12G
38G	35G	12G
36C	34C	12C
25A_13G	31A	8A_4G_1C
40G	34G	13G
41G	33G	14G
41G	33G	14G
36T	32T	12T
42G	32G	14G
41G	31G	12G
41G	30G	14G
36G	30G	14G
42C	31C	13C
42A	31A	12A

42G	31G	13G
41G	30G	13G
33C	26C	11C
38C	29C	12C
41C	29C	12C
41C	30C	12C
41C	30C	12C
38C	28C	13C
31T	29T	11T
29T	26T	11T
35C	30C	12C
92C	32C	12C
8C	33C	13C
8C	33C	14C
6C	32C	16C
5A_1G_2C	27A	13A
33C	33C	15C
32C	33C	16C
34C	33C	7A_9C
33C	32C	17C
30A	31A	17A
32C	34C	18C
32C	35C	17C
32C	36C	18C
34C	35C	18C
35G	33G	18G
36T	33T	16T
37C	34C	18C
37A	35A	19A
38C	35C	19C
38A	34A	20A
39A	34A	20A
41G	36G	20G
42G	37G	21G
43G	38G	22G
44C	40C	12T_14C
41G	42G	27G
42A	43A	29A
42T	44T	30T
47G	45G	32G
48A	46A	34A
50G	47G	34G
48T	47T	34T

50C	47C	36C
48G	46G	36G
50G	46G	36G
53A	46A	36A
55T	46T	36T
57G	45G	36G
55T	47T	36T
58A	48A	36A
62T	51T	35T
63C	50C	36C
64C	51C	36C
64G	51G	37G
64A	52A	39A
64G	53G	40G
63A	57A	42A
66T	58T	42T
68C	57C	41C
72C	56C	42C
72A	58A	41A
75C	59C	41C
78C	61C	42C
78A	64A	42A
77A	64A	42A
80C	64C	42C
80T	64T	42T
81T	64T	45T
81T	64T	45T
81A	63A	43A
84T	63T	44T
84G	64G	45G
87G	63G	44G
87A	64A	46A
87C	65C	46C
87G	68G	46G
90G	68G	46G
91A	67A	46A
93G	69G	46G
94G	40A_30G	45G
94A	67A	46A
94T	68T	45T
95A	69A	44A
95C	71C	43C
96C	70C	44C

97T	71T	43T
99T	73T	44T
100T	75T	45T
103A	74A	44A
105T	73T	43T
108A	73A	45A
109A	32A_40G	44A
109G	71G	45G
110C	74C	43C
110C	73C	44C
110A	73A	44A
115A	73A	44A
117A	72A	43A
118A	72A	43A
121A	74A	41A
119C	75C	42C
117C	72C	42C
117T	76T	44T
118A	76A	44A
117T	74T	40T
117A	73A	39A
117A	72A	37A
120A	72A	36A
117G	72G	34G
117A	72A	31A
117G	71G	31G
117G	71G	30G
117G	70G	30G
118C	71C	30C
116C	70C	30C
113G	70G	31G
112A	70A	32A
112G	71G	32G
114A	69A	33A
113A	68A	35A
110A	64A	35A
110A	62A	35A
110A	61A	35A
111A	61A	34A
113A	60A	33A
105A	60A	28A_1G_15C
53A_103C	57A	30A
104A	55A	33A

105C	54C	33C
107A	55A	33A
110C	55C	33C
109A	55A	34A
107G	53G	32G
106T	52T	32T
105G	50G	32G
104C	52C	32C
103A	52A	29A
103G	54G	30G
104G	54G	28G
104C	56C	28C
103T	57T	29T
103G	59G	27G
102A	55A	25A
99A	57A	26A
98A	54A	26A
95A	54A	25A
95G	53G	25G
95C	54C	25C
93A	54A	27A
92A	52A	28A
92A	49A	28A
90G	50G	28G
89T	50T	29T
88T	50T	28T
87C	50C	29C
86C	49C	28C
84T	47T	27T
81T	48T	11T_1G_15C
81G	48G	28G
78G	48G	27G
75C	47C	27C
74A	47A	26A
73A	47A	26A
73A	48A	26A
72G	45G	26G
70T	48T	25T
69G	48G	25G
65C	48C	25C
64C	48C	25C
62T	47T	26T
61C	48C	25C

62T	48T	24T
62T	50T	23T
62T	47T	22T
78T_23G	45T_22G	57T_25G_1C
49G	26G	21G
53G	46G	21G
54G	46G	21G
54G	46G	22G
53G	46G	24G
53G	47G	23G
54G	48G	23G
46T	1A_23T_23G	21G
52G	45G	22G
63A	45A	20A
66C	44C	22C
66T	45T	23T
65A	45A	24A
66C	46C	25C
65A	47A	26A
65G	47G	26G
64A	46A	26A
66T	46T	27T
66C	46C	25C
68C	45C	26C
67C	45C	26C
69A	45A	26A
68G	47G	26G
65A	47A	25A
70A	46A	26A
70T	47T	26T
70C	47C	24C
72C	49C	26C
77C	50C	26C
77C	49C	25C
76C	48C	25C
77A	47A	25A
78G	48G	24G
77A	53A	26A
80G	52G	27G
78T	53T	27T
81C	50C	26C
82C	49C	27C
82A	48A	28A

81C	49C	26C
83A	49A	25A
85C	51C	25C
86A	51A	24A
86T	52T	24T
87A	51A	24A
85A	50A	23A
87G	54G	24G
91C	56C	24C
92G	56G	26G
93C	56C	25C
87T	55T	24T
92T	57T	26T
96G	58G	26G
96C	58C	28C
97C	58C	30C
95C	58C	30C
96G	57G	30G
93T	58T	30T
96A	60A	32A
96T	60T	31T
99T	59T	30T
98T	60T	28T
96T	58T	29T
95G	60G	30G
97G	61G	30G
98A	60A	30A
101T	60T	30T
99T	59T	32T
99A	57A	33A
101G	58G	32G
100G	58G	33G
97C	57C	33C
98A	58A	32A
96A	58A	31A
95A	60A	30A
97G	58G	30G
97T	59T	31T
97G	59G	31G
97G	59G	31G
98T	59T	31T
100C	62C	34C
100T	62T	34T

103G	63G	34G
104T	62T	33T
104G	62G	33G
104G	62G	31G
103A	64A	31A
105C	65C	33C
108C	66C	34C
107A	68A	33A
108A	67A	33A
107C	70C	35C
104C	69C	34C
108T	70T	34T
106T	70T	34T
104G	70G	35G
104A	68A	34A
102C	69C	33C
98T	69T	35T
100G	71G	36G
104G	73G	37G
102G	73G	37G
106G	72G	38G
103C	68C	37C
102G	70G	36G
102T	69T	36T
100C	73C	36C
53A_48G	72G	38G
101C	71C	37C
99C	71C	38C
99C	70C	39C
99C	69C	39C
98T	70T	38T
98G	68G	37G
97G	68G	38G
98G	71G	38G
101T	70T	37T
98G	69G	36G
97C	69C	35C
102T	69T	35T
103G	68G	36G
105A	68A	38A
101A	68A	39A
101A	68A	36A
101T	67T	36T

102G	67G	36G
52T_50C	68T	29C
102G	70G	36G
101G	67G	35G
101A	66A	35A
97G	31A_1T_35G	37G
102G	67G	39G
104G	67G	39G
104T	68T	37T
103G	69G	37G
105C	69C	36C
103C	68C	37C
104A	69A	36A
106A	69A	39A
103A	70A	37A
106A	70A	39A
107T	72T	41T
104A	75A	41A
105C	79C	41C
107A	77A	41A
105G	78G	42G
104A	77A	41A
106G	76G	42G
107G	77G	43G
104T	44T_32G	42T
106A	75A	41A
106A	74A	40A
104G	77G	40G
102T	78T	42T
104C	79C	43C
107T	78T	43T
108A	77A	42A
106C	76C	42C
102T	74T	39T
103C	74C	39C
102G	74G	41G
56T_44C	71T	39T
100G	73G	40G
98A	71A	40A
98G	72G	41G
98T	71C	13T_29C
56A_42G	72A	42A
104T	69T	42T

104T	70T	42T
107A	67A	41A
102A	65A	40A
103G	65G	40G
102A	66A	40A
102A	66A	42A
101A	65A	42A
99A	64A	43A
97T	62T	43T
97A	64A	41A
97C	66C	42C
98G	67G	41G
98T	68T	41T
98G	67G	41G
101G	66G	40G
53G_46C	66G	41G
98A	65A	42A
98A	65A	43A
97C	67C	42C
96A	66A	43A
99G	66G	42G
95C	66C	43C
95T	65T	42T
92C	66C	40C
94T	67T	39T
95C	67C	38C
48A_44G	31A_36G	38G
91G	68G	39G
91T	67T	38T
92C	66C	39C
93C	70C	39C
91T	72T	39T
91G	72G	37G
92T	71T	36T
90T	70T	34T
91C	69C	36C
91T	66T	36T
90G	66G	36G
88T	71T	35T
88T	58T	35T
88C	58C	31C
88T	63T	34T
86T	65T_23C	31T

86C	64C	18T_47C
88T	58T	32T
87T	56T	23T
84C	57C	28C
28T_84C	46T_57C	29T_23C
89T	35T	20T
90T_29C	33T_41C	15T_6C
81C	63C	28C
89C	64C	29C
85C	64C	29C
85C	64C	29C
85C	65C	31C
87C	64C	30C
85C	65C	31C
85C	64C	29C
85C	62C	28C
80A	61A	28A
83A	61A	27A
83A	61A	28A
81C	60C	28C
80C	62C	27C
79T	62T	28T
81A	62A	28A
79A	61A	26A
79A	62A	27A
79C	64C	26C
78T	66T	26T
77T	67T	25T
75C	67C	25C
80C	65C	25C
81T	63T	24T
82T	62T	25T
86T	63T	24T
86T	65T	23T
85T	67T	23T
88T	66T	22T
89C	66C	20C
89T	66T	20T
89T	63T	19T
87C	64C	19C
84A	61A	18A
77G	56G	17G
79G	57G	17G

86G	60G	18G
88G	60G	18G
84T	60T	18T
90G	63G	20G
90G	59G	20G
88T	59T	19T
91A	60A	21A
93C	61C	20C
92C	62C	22C
92A	60A	21A
92G	61G	21G
92C	60C	21C
91T	58T	19T
90C	59C	21C
94C	59C	20C
95T	57T	20T
95G	56G	20G
93G	56G	21G
91C	36G_23C	20C
92A	61A	21A
92A	61A	21A
92A	62A	20A
92C	63C	19C
88A	61A	20A
87T	64T	20T
88T_50G	63T_19G	19T_12G
96G	67G	25G
96G	67G	25G
95G	68G	25G
93G	65G	25G
95G	64G	26G
49A_47G	63G	25G
97G	65G	26G
96G	63G	25G
86C	62C	24C
88G	62G	25G
87C	64C	25C
87T	61T	24T
90C	61C	24C
90T	60T	24T
91T	59T	24T
93C	60C	23C
92C	61C	12T_13C

90A	61A	28A
90C	62C	30C
90T	60T	30T
92C	62C	31C
90A	64A	33A
92C	66C	33C
90C	64C	33C
90T	65T	32T
90C	64C	32C
93T	64T	33T
94T	63T	34T
92C	64C	34C
90C	63C	34C
90C	66C	34C
93C	67C	34C
92A	68A	34A
91T	69T	17T_17C
92A	65A	34A
91G	67G	34G
82T_88G	62T_63G	1A_31T_33G
90A	62A	34A
97A	66A	38A
99A	67A	40A
97A	68A	39A
97G	66G	37G
93G	66G	38G
98A	68A	37A
97T	69T	38T
100G	67G	37G
99C	66C	38C
52T_1G_46C	37T_30C	35C
99G	65G	34G
101G	66G	34G
97G	67G	32G
99A	66A	34A
98C	62C	34C
98T	64T	34T
98A	64A	33A
97G	65G	32G
96A	67A	33A
95G	68G	33G
96G	69G	32G
94G	69G	33G

95G	68G	31G
94G	65G	34G
95C	64C	35C
95C	64C	38C
99T	63T	39T
100A	64A	39A
103A	65A	38A
102T	65T	38T
100C	62C	40C
101C	62C	39C
100T	62T	41T
100G	62G	40G
56T_43C	63C	41C
97A	63A	40A
95C	64C	42C
95C	64C	42C
96A	64A	43A
97T	64T	43T
98A	65A	42A
98T	64T	43T
98T	63T	43T
100T	66T	43T
99C	66C	42C
101A	65A	45A
99T	65T	46T
100G	64G	45G
105C	64C	41C
107A	64A	40A
107T	66T	41T
106T	66T	40T
108C	64C	38C
110T	65T	39T
113T	66T	37T
112C	67C	40C
112C	69C	41C
111T	67T	40T
110G	66G	39G
114A	69A	40A
114C	69C	41C
114T	67T	41T
110G	66G	41G
116C	65C	40C
117C	65C	39C

118T	65T	41T
116A	67A	47A
118C	67C	48C
117T	66T	46T
118T	67T	46T
118C	69C	46C
112T	67T	44T
113C	69C	43C
112T	69T	44T
111C	70C	43C
109T	68T	46T
109A	67A	46A
109A	67A	47A
109G	69G	45G
108C	70C	45C
106C	70C	47C
64A_38G	69G	50G
102G	69G	50G
61A_38G	68G	48G
39T_60G	69T	49T
104G	71G	51G
106T	71T	54T
105G	75G	54G
104T	75T	54T
104G	74G	54G
104T	74T	55T
108G	72G	57G
107T	71T	59T
111G	71G	56G
110T	72T	53T
110G	73G	52G
106C	71C	50C
106T	71T	49T
61T_42C	71C	20T_28C
102T	73T	48T
100G	73G	48G
100T	74T	45T
101G	77G	46G
100G	77G	46G
101C	77C	46C
105T	79T	46T
105G	79G	44G
106T	77T	43T

107T	79T	42T
107T	79T	44T
107T	77T	45T
108A	77A	45A
108T	78T	45T
107G	74G	44G
109T	75T	45T
105C	77C	44C
106T	78T	44T
108C	75C	43C
108C	80C	44C
107A	79A	43A
107G	77G	25T_19G
106T	79T	45T
104T	78T	44T
102T	76T	44T
101C	76C	45C
100C	74C	45C
101A	70A	44A
100G	73G	45G
99C	72C	45C
95C	70C	44C
96T	70T	46T
98T	70T	46T
96C	71C	46C
94T	71T	47T
93T	70T	47T
92T	69T	48T
88G	71G	43G
86T	69T	40T
84C	68C	43C
82A	68A	43A
83C	68C	42C
83T	69T	42T
84G	67G	41G
90G	67G	43G
90T	67T	42T
92T	66T	42T
92A	67A	41A
94T	68T	41T
95G	66G	42G
96C	67C	42C
95T	68T	39T

94G	69G	39G
94G	69G	39G
94G	70G	39G
97T	69T	36T
98A	70A	36A
98A	72A	33A
102A	67A	33A
104G	67G	34G
104G	69G	34G
104C	69C	34C
98A	70A	32A
99G	73G	31G
96A	74A	32A
95T	73T	32T
96G	74G	34G
98G	74G	34G
101G	78G	34G
101A	76A	35A
102G	74G	35G
102C	75C	34C
104T	74T	36T
104G	72G	38G
103T	74T	37T
106G	76G	37G
102C	75C	37C
101T	74T	38T
100C	74C	41C
101A	74A	41A
99A	75A	39A
100A	77A	40A
100C	76C	39C
98C	78C	39C
97A	78A	40A
96C	78C	39C
96A	77A	40A
95T	76T	22T_1G_15C
92C	79C	38C
91T	80T	40T
93G	82G	43G
93G	84G	40G
96A	81A	41A
96C	81C	41C
96A	80A	43A

98G	79G	43G
102C	82C	43C
106C	82C	43C
105T	81T	41T
109C	83C	42C
110A	85A	42A
107C	84C	42C
106T	85T	42T
106T	86T	42T
105C	88C	41C
107C	87C	40C
105C	85C	41C
106C	85C	41C
105T	87T	41T
107C	87C	41C
107C	87C	41C
106C	88C	41C
106A	86A	42A
106G	87G	44G
100T	87T	41T
100C	87C	42C
98A	88A	43A
98A	88A	43A
93A	87A	42A
94G	87G	9A_9T_49G_15C
92C	85C	40C
94C	85C	40C
94T	84T	25T
97C	85C	26C
97C	84C	26C
93C	85C	27C
93T	84T	28T
92A	82A	28A
91A	83A	27A
91A	84A	22A_5G_1C
91C	82C	34C
93C	80C	34C
94C	81C	34C
94A	79A	35A
93C	80C	36C
94C	79C	36C
95A	76A	35A
95T	77T	35T

92G	75G	35G
92C	75C	34C
90T	77T	35T
93T	75T	34T
93C	77C	35C
93C	76C	36C
96T	74T	39T
93T	71T	39T
92T	72T	40T
92T	72T	40T
96C	71C	37C
99T	70T	38T
103A	70A	36A
101T	70T	38T
99C	72C	38C
101T	70T	39T
101C	70C	39C
101A	70A	39A
100T	70T	38T
100T	72T	39T
100T	71T	40T
100C	67C	39C
100C	66C	38C
101T	64T	41T
100C	66C	42C
99A	68A	42A
97C	69C	42C
96C	69C	42C
92T	67T	41T
89T	67T	42T
89T	67T	42T
87C	67C	41C
84C	66C	41C
85A	66A	40A
88C	67C	39C
90C	69C	40C
88T	67T	42T
86T	68T	43T
86A	69A	43A
86T	67T	41T
89T	66T	41T
89C	67C	41C
89C	70C	41C

91A	68A	39A
92T	69T	39T
95A	71A	37A
96C	72C	38C
99C	71C	40C
99T	71T	41T
99A	72A	42A
101A	74A	42A
103G	74G	43G
105C	75C	42C
104C	75C	46C
104A	75A	46A
101A	74A	46A
101C	74C	47C
100A	73A	46A
101C	74C	46C
100A	75A	46A
100G	74G	45G
100A	72A	45A
102G	72G	46G
99T	74T	47T
98G	76G	47G
99C	76C	48C
101T	72T	47T
101C	77C	49C
100C	79C	50C
99A	80A	50A
97C	79C	52C
98A	81A	52A
100A	78A	53A
97T	78T	54T
96T	32T_46G	2A_24T_27G
97A	78A	53A
94A	78A	51A
94G	78G	51G
95C	76C	24G_28C
97C	76C	52C
95A	76A	53A
91A	77A	54A
88T	77T	54T
87G	76G	53G
87C	76C	53C
48T_35C	27T_46C	52C

83G	74G	53G
84A	74A	54A
85G	75G	54G
86T	75T	52T
86G	75G	53G
87A	74A	52A
84G	77G	51G
86G	80G	48G
84G	80G	47G
88C	77C	47C
90A	78A	46A
91T	78T	47T
46T_48C	77T	49T
96G	78G	46G
95C	78C	48C
95C	77C	46C
95T	77T	48T
93T	78T	48T
93G	77G	49G
94A	74A	48A
96T	78T	48T
100A	77A	46A
103C	76C	44C
104A	78A	45A
103G	79G	45G
106A	79A	44A
107C	77C	45C
106C	77C	44C
102T	77T	45T
101T	75T	45T
100C	74C	45C
99C	75C	42C
99T	74T	41T
100C	75C	40C
102G	41A_32G	23A_16G
104G	73G	40G
103G	72G	42G
106G	70G	41G
108C	71C	1A_22T_19C
109G	71G	44G
108C	74C	43C
108T	74T	45T
113T	73T	46T

114A	73A	46A
111A	77A	49A
112C	81C	53C
110G	81G	52G
110A	79A	52A
115C	76C	51C
114T	75T	49T
110G	75G	48G
109T	73T	49T
107C	74C	19T_1G_28C
106T	74T	46T
107T	74T	47T
107G	72G	47G
106C	76C	47C
104A	77A	45A
104G	76G	44G
104A	76A	45A
102A	75A	44A
102T	77T	42T
101G	78G	41G
100T	78T	39T
99C	79C	39C
57T_28C	80T	22T_10C
28A_56G	80G	10A_22G
84C	79C	18T_10C
83C	77C	29C
136A_198T_204G_136C	78C	25T
30C	81T	28C
44A	81A	38A
45C	43G_39C	40C
47A	82A	41A
48G	83G	41G
49A	84A	41A
49T	81T	41T
51C	41T_41C	41C
51G	80G	19A_22G
52G	82G	48G
53A	80A	48A
53G	80G	50G
54G	82G	51G
55T	85T	51T
56G	87G	2A_25T_25G
56C	86C	51C

56C	84C	52C
58T	83T	52T
109T	85T	51T
110C	85C	51C
110A	83A	51A
111C	84C	52C
115T	86T	52T
114G	85G	52G
113G	87G	51G
114C	87C	52C
116C	87C	53C
116T	86T	51T
121C	88C	53C
123A	87A	52A
123A	89A	53A
124A	92A	53A
127G	93G	52G
126G	92G	53G
125C	92C	54C
121T	93T	51T
123T	94T	52T
122T	94T	50T
120T	94T	51T
119C	96C	50C
117A	94A	50A
120G	98G	48G
116G	96G	48G
120G	98G	47G
117A	94A	43A
115G	89G	41G
111T_2G_102C	88T_84C	38T_1G_37C
117C	93C	43C
115C	93C	47C
114A	90A	47A
117C	92C	50C
118C	92C	50C
115T	93T	50T
114G	93G	50G
116G	94G	51G
113C	91C	47C
111C	92C	49C
108T	93T	49T
106T	95T	48T

104C	98C	44C
103A	97A	42A
101G	97G	47G
101G	100G	47G
101T	102T	44T
102C	100C	44C
106T	99T	41T
104C	100C	40C
102T	100T	41T
101G	100G	42G
101T	97T	43T
100A	99A	43A
100A	98A	41A
100C	97C	40C
99T	98T	41T
98G	97G	41G
97C	95C	41C
94C	94C	42C
98C	96C	43C
97C	96C	43C
99T	97T	45T
101T	95T	45T
100C	95C	45C
98C	91C	48C
100T	91T	49T
101C	90C	47C
102C	93C	49C
102A	94A	54A
101A	92A	56A
100A	93A	56A
99T	95T	57T
97A	95A	57A
95C	93C	57C
99A	96A	58A
100C	92C	58C
98C	92C	57C
97C	91C	56C
97C	94C	54C
95A	96A	56A
95A	97A	56A
97A	95A	55A
98C	91C	56C
97A	90A	56A

99A	88A	54A
100C	89C	53C
100A	87A	58A
100C	86C	57C
103A	87A	58A
104A	85A	58A
104A	84A	57A
105C	85C	59C
102G	83G	59G
104C	88C	59C
100A	85A	58A
100A	86A	59A
98A	88A	60A
98G	91G	60G
99T	92T	60T
96G	91G	62G
97T	92T	58T
99T	91T	55T
96C	90C	55C
95T	92T	55T
96T	91T	57T
98C	89C	56C
99A	90A	57A
98T	91T	58T
97T	87T	57T
99C	87C	57C
100A	85A	59A
102T	84T	59T
105C	84C	60C
106C	82C	57C
109A	80A	62A
110C	77C	62C
110A	80A	62A
107T	80T	62T
107C	80C	62C
107C	79C	62C
107T	79T	61T
109C	78C	60C
111A	78A	63A
110G	80G	63G
109C	82C	65C
109T	80T	67T
109T	81T	67T

111T	82T	68T
112C	81C	67C
112A	79A	69A
112A	80A	69A
109A	80A	68A
108A	81A	65A
110A	82A	64A
114C	84C	62C
114G	87G	60G
114A	87A	60A
115A	86A	61A
114T	84T	57T
119T	86T	55T
121T	88T	54T
121T	85T	53T
122G	86G	53G
122T	89T	55T
56A_62G	85G	54G
117C	88C	55C
118T	92T	56T
116C	93C	59C
118G	38A_51G	59G
117C	88C	59C
115C	88C	59C
116A	89A	60A
115T	90T	60T
115G	90G	60G
116C	91C	61C
116T	91T	61T
119C	92C	58C
118T	95T	57T
116T	94T	57T
115G	98G	56G
115T	99T	56T
113G	101G	56G
114C	100C	56C
115A	97A	55A
115A	98A	55A
115T	99T	58T
115A	97A	55A
64T_54C	96T	57T
118A	95A	57A
118G	94G	55G

117C	94C	57C
122T	94T	57T
122T	96T	56T
128C	95C	55C
126C	94C	54C
123A	94A	55A
121G	93G	56G
123C	94C	56C
123A	95A	56A
123T	95T	56T
124C	96C	54C
122T	93T	56T
119G	93G	57G
119C	96C	55C
118C	96C	51C
117A	98A	51A
117G	96G	52G
115C	96C	52C
115T	97T	51T
116G	55A_1T_41G	54G
117G	97G	53G
117T	97T	55T
116G	96G	53G
116C	95C	52C
116A	92A	53A
115G	92G	51G
113C	91C	52C
114C	91C	51C
115A	91A	50A
113C	92C	51C
115C	92C	53C
56A_59G	91A	54A
115G	90G	56G
114A	89A	56A
115T	85T	57T
114C	83C	61C
115A	84A	62A
113G	84G	61G
112T	84T	63T
107T	84T	65T
105A	82A	67A
109T	82T	68T
109G	79G	67G

109T	80T	65T
109G	80G	65G
108A	78A	64A
108T	73T	64T
109T	75T	62T
108C	76C	62C
108T	77T	62T
108G	76G	61G
103G	75G	60G
108G	75G	61G
107A	77A	61A
103G	77G	60G
48T_53C	77C	60C
99C	77C	60C
104C	75C	60C
105A	75A	62A
106G	72G	62G
106A	71A	60A
107A	72A	62A
109A	71A	62A
106A	72A	63A
105A	71A	63A
106A	70A	62A
105T	70T	62T
104A	69A	60A
61A_43T	69A	58A
103A	71A	57A
106A	69A	60A
100C	70C	60C
102T	69T	60T
101T	69T	60T
101C	70C	60C
100T	70T	59T
100C	70C	56C
101C	72C	56C
101A	72A	56G
98A	75A	56A
98A	75A	58A
100C	80C	58C
102A	79A	56A
103C	79C	59C
102T	78T	61T
102G	76G	62G

104A	76A	61A
106A	76A	63A
110A	77A	65A
110A	77A	63A
110A	80A	64A
107G	80G	63G
104A	81A	62A
102G	41A_40G	63G
101A	80A	61A
104G	81G	61G
107G	82G	64G
105G	81G	61G
102T	79T	65T
102C	80C	63C
99T	81T	62T
98G	83G	61G
96A	84A	59A
94C	89C	60C
91C	89C	61C
92T	89T	58T
92G	89G	58G
93A	89A	58A
95C	91C	56C
97A	92A	55A
97A	90A	54A
94A	90A	53A
94A	89A	53A
94C	91C	53C
94A	92A	53A
93A	94A	52A
94A	95A	52A
94G	91G	52G
94C	91C	51C
93A	90A	52A
97A	89A	52A
99T	90T	54T
98A	90A	55A
97A	88A	55A
98C	89C	55C
98C	89C	55C
98T	91T	55T
94A	92A	55A
93C	92C	56C

93C	92C	54C
93C	91C	59C
93A	86A	58A
92G	88G	58G
94G	87G	56G
100C	88C	57C
99A	88A	56A
99C	88C	57C
98C	87C	57C
97C	86C	58C
95T	82T	55T
98C	83C	55C
99C	82C	56C
100G	81G	57G
97G	80G	58G
97C	81C	57C
98T	82T	58T
98C	81C	59C
100C	79C	59C
99T	78T	58T
99A	77A	58A
99T	78T	56T
97C	75C	55C
100A	77A	57A
98T	76T	54T
97T	78T	55T
99C	80C	52C
98C	79C	55C
96C	80C	25T_29C
91A	80A	52A
91A	82A	51A
92T	80T	51T
94T	81T	51T
97G	81G	51G
97G	81G	50G
100C	83C	48C
101G	85G	49G
97A	84A	45A
96C	82C	45C
96A	84A	43A
55T_40C	87C	47C
101G	42G_43C	23G_22C
107T	80T	44T

108C	84C	44C
110C	79C	42C
112T	79T	42T
111G	79G	43G
110G	80G	44G
114G	80G	45G
114G	82G	45G
113G	82G	44G
115T	83T	44T
114G	83G	44G
115A	87A	46A
115T	83T	48T
114G	84G	49G
115G	82G	49G
115G	81G	51G
115A	81A	50A
116G	82G	52G
116T	81T	51T
116T	47T_34C	27T_25C
115G	84G	51G
114T	85T	48T
116A	86A	48A
117G	85G	47G
117T	86T	48T
116T	84T	47T
118G	83G	46G
118G	83G	46G
118G	85G	45G
121C	85C	43C
119C	89C	43C
119C	89C	43C
117A	89A	45A
113T	89T	46T
112C	89C	45C
113T	90T	45T
113G	89G	46G
116G	91G	45G
116A	93A	45A
114A	94A	46A
114A	93A	45A
112G	92G	45G
115C	91C	48C
114A	90A	47A

115C	88C	47C
115C	88C	46C
113T	88T	48T
114G	89G	48G
115G	88G	48G
46T_70C	89T	24T_24C
117T	90T	48T
114A	90A	47A
115G	90G	47G
115G	88G	44G
116A	87A	45A
119G	88G	41G
119A	88A	42A
120G	87G	43G
119T	85T	43T
119C	85C	42C
116T	83T	43T
116T	84T	42T
[truncated: 344,230 more chars]
