# Supplementary material for: A near-complete genome assembly of the bearded dragon Pogona vitticeps provides insights into the origin of Pogona sex chromosomes
Source: Gigascience. 2025 Aug 19;14:giaf079. doi: 10.1093/gigascience/giaf079 (PMC12360845; doi:10.1093/gigascience/giaf079)
Supplement: giaf079_Authors_Response_To_Reviewer_Comments_Original_Submission [file giaf079_authors_response_to_reviewer_comments_original_submission.pdf]

Author's Response To Reviewer Comments

Point to point response for Reviewers' Comments:

In this report, we have addressed all the questions and comments from the two reviewers (marked in italic black), by our point by point responses (marked in blue). All references cited in this document have been placed at the end.

Point to point response for Reviewer #1
Reviewer #1: Guo et al. present a new reference genome for Pogona vitticeps, a widespread reptile model organism that is also common as a domestic animal worldwide. The genome assembly shows much improvement over an older assembly from 2017. There are two points that make this manuscript outstanding from common genome assembly papers:
1. The authors find a new sex determination locus in this species.
2. the authors use a new nanopore sequencing technology ("CycloneSEQ"), which has so far only described in a preprint:
(https://www.biorxiv.org/content/10.1101/2024.08.19.608720v1).

In my opinion this deserves a publication in Gigascience, but both points must be focused more on a revised manuscript.

Response
We are sincerely grateful for your summary of our work and your recognition of its significance. We also appreciate the numerous constructive suggestions provided, which have significantly enhanced our manuscript. We have carefully revised the manuscript based on your comments to ensure that it includes sufficient evidence and details, and all the revised parts in the manuscript have been highlighted in dark red. We believe that the revised version represents a substantial improvement.

Below, I will explain these points:
1-1 The authors have sequenced a male individual (ZZ), which means the long-read reference assembly is missing the W-chromosome. PAR and SDR regions are deduced from the Z sequence, by analysis of sequencing coverage of only a few sexed samples (2 females and 4 males). It is unclear if these individuals are from the same family, which could mean that the newly found SD-region could just be a family specific variation. To make the whole story more intriguing and statistical sound the authors should at least test 15 males and 15 females from different P. vitticeps populations for W-specific markers near the proposed AMH deletion. The authors should also show that the prior proposed SD locus (nr5a1) does not carry W-specific mutations in these 15+15 individuals. Furthermore, a phased assembly of a female (ZW) Pogona vitticeps individual, could enable the assembly of the missing W-chr and should be included, it would even improve analysis of W-specific sequences in the proposed additional individuals.

Response
Thank you for this constructive suggestion. Based on your comment, I have broken them down into the following four points and will address each one in turn:

Point 1: PAR and SDR regions are deduced from the Z sequence, by analysis of sequencing coverage of only a few sexed samples (2 females and 4 males). To make the whole story more intriguing and statistical sound the authors should at least test 15 males and 15 females from different P. vitticeps populations for W-specific markers near the proposed AMH deletion.
Response
We sincerely appreciate your insightful suggestion to expand the sample size for validating the PAR/SDR regions. While logistical constraints (e.g., difficulties in obtaining specimens from diverse wild populations due to import restrictions) limited our ability to reach the proposed 15 males and 15 females, we made significant efforts to enhance our cohort.
We collected an additional 14 individuals and rigorously validated their genetic sex using W-specific molecular markers, as described in the Methods section (Sex identification). These markers were analyzed

via agarose gel electrophoresis, where ZZ males exhibited a single band and ZW females showed two distinct bands (Figure R1.1; Supplementary Fig. S13) [1]. Combined with the original 6 samples, our final dataset now includes 8 ZZ males and 12 ZW females (Table R1.2).
Although this sample size falls short of the reviewer's recommendation, we note that it aligns with established practices in sex chromosome studies [2]. For instance, recent work on Hippocampus erectus sex determination demonstrated robust PAR/SDR demarcation with comparable sample size (10 females + 10 males) [2]. Notably, our re-analysis incorporating the expanded dataset confirmed consistent PAR and SDR boundaries (see updated Fig. 5C), reinforcing the reliability of our initial findings.
We acknowledge that larger, population-diverse cohorts could further strengthen statistical confidence. However, the current evidence – supported by methodological rigor, consistency across analyses, and congruence with published precedents – provides and compelling foundation for our conclusions regarding Z chromosome architecture in P. vitticeps.

Figure R1.1 The agarose gels for the additional 14 individuals.

Table R1.2 8 ZZ male and 12 ZW female individuals' information used for PAR/SDR demarcation.

Sample ID	Genotype	Total clean data (Gb)	Genome coverage (X)
ZW_1	ZW female	63.19	35.3
ZW_2	ZW female	66.05	36.9
ZZ_1	ZZ male	68.56	38.3
ZZ_2	ZZ male	112.23	62.7
ZZ_3	ZZ male	100.60	56.2
ZZ_4	ZZ male	98.27	54.9
ZW_3	ZW female	246.48	137.70
ZW_4	ZW female	149.29	83.40
ZW_5	ZW female	153.76	85.90
ZW_6	ZW female	165.04	92.20
ZW_7	ZW female	167.01	93.30
ZW_8	ZW female	205.85	115.00
ZW_9	ZW female	185.62	103.70
ZW_10	ZW female	176.49	98.60
ZW_11	ZW female	148.75	83.10
ZW_12	ZW female	147.85	82.60
ZZ_5	ZZ male	161.82	90.40
ZZ_6	ZZ male	257.04	143.60
ZZ_7	ZZ male	187.41	104.70
ZZ_8	ZZ male	121.36	67.80

Point 2: PAR and SDR regions are deduced from the Z sequence, by analysis of sequencing coverage of only a few sexed samples (2 females and 4 males). It is unclear if these individuals are from the same family, which could mean that the newly found SD-region could just be a family specific variation.
Response
We thank you for highlighting this critical concern. To preclude familial bias in PAR/SDR analysis, our cohort integrates: (1) five geographically/temporally diverse samples from public datasets (Supplementary Table S13), (2) one newly collected sample with no lineage overlap to published data, and (3) 14 additional samples collected from different locations. All individuals were verified as unrelated through breeder records and genomic validation. Besides this, to empirically rule out familial relatedness, we conducted two complementary genomic analyses:
1. Principal Component Analysis (PCA) was performed using Plink v1.07 (RRID:SCR_001757) [3] on genome-wide SNPs called using FreeBayes v0.9.21 (RRID:SCR_010761) [4]. The resulting PCA plot (Figure R1.3) revealed no clustering patterns indicative of shared ancestry, with samples broadly dispersed across genetic space.
2. Kinship analysis was conducted using PLINK's pairwise PI_HAT estimates, which quantify the proportion of alleles shared identical-by-descent (IBD). All pairwise values fell within a narrow range of 0.012 ~ 0.100 (Figure R1.4), well below the threshold for even distant relatedness (PI_HAT > 0.1) [5]. Critically, no sample pairs approached values suggestive of first- or second-degree relationships (PI_HAT > 0.25), confirming the absence of parent-offspring, sibling, or avuncular connects.

The combination of samples from different sources, PCA-based population structure validation, and kinship coefficient analysis provides robust evidence that our cohort is devoid of familial relatedness. This ensures that the observed Z-W coverage patterns and the identification of the SDR region reflect species-level genomic architecture rather than lineage-specific artifacts.

Figure R1.3 PCA analysis of 20 samples.

Figure R1.4 kinship analysis of 20 samples.

Point 3: The authors should also show that the prior proposed SD locus (nr5a1) does not carry W-specific mutations in these 15+15 individuals.
Response
We conducted a comprehensive analysis to evaluate potential W-specific mutations in the nr5a1 locus. First, we compared genetic diversity patterns between the nr5a1 region and autosomes (Chr1~Chr15). Comparative analysis of SNP density (per Mb) and average sequencing coverage between the nr5a1 gene region and autosomes (Chr1~ Chr15) (Figure R1.5 and Figure R1.6) revealed no evidence of a two-fold depth reduction across all 20 samples.
Building on these global patterns, we performed detailed nucleotide-resolution analysis of sequencing coverage across all sites within the nr5a1 region (Supplementary Table S17). Among 26,763 interrogated loci, 24,072 (89.95%) exhibited consistent homozygosity across all samples. None of the 2691 retained loci exhibited mutations specific to ZW individuals, with any observed mutation being consistently shared across all ZW individuals. This systematic evaluation conclusively excludes W-linked mutational signatures in nr5a1.
Collectively, our multilayered analysis – spanning regional diversity metrics, depth profiles, and allele-specific patterns – confirms the absence of W-specific mutations in nr5a1. These findings support its characterization as a conserved sex-determining gene on the Z chromosome, rather than a W-derived element.

Figure R1.5 SNP density per Mb between nr5a1 region and Autosomes (Chr1~Chr15).
Figure R1.6 Average sequencing coverage (X) between nr5a1 region and Autosomes (Chr1~Chr15).

Point4: Furthermore, a phased assembly of a female (ZW) Pogona vitticeps individual, could enable the assembly of the missing W-chr and should be included, it would even improve analysis of W-specific sequences in the proposed additional individuals.
Response
We fully acknowledge the importance of resolving the W chromosome to strengthen our conclusions. However, due to limitations in project funding, achieving W chromosome assembly would require collecting and sequencing data from parents and F1 ZW females, as well as performing haploid assembly. Such a task is beyond the scope of the current project. Nevertheless, we hope to address this in future work.

1-2 A technology aware reader would like to see more information on the specifics of the CycloneSEQ data quality and handling and maybe a comparison to competing technologies.
Response
We thank you for emphasizing the importance of comparing emerging sequencing technologies with other sequencing platforms. However, since we did not sequence data from the PacBio and ONT platforms in this study, direct comparisons are not feasible. For more detailed sequencing performance metrics of this sequencing technology, please refer to the preprint article “A single-molecule nanopore sequencing platform” [6].

1-3 Which enzymes and buffers were used to prepare the library? In the sections on the methods, there are only superficial descriptions such as (DNA repair buffer/enzyme, DNA clean beads, wash buffer for long fragments). Is it a kit or were the enzymes and buffers purchased individually?
Response
We thank you for highlighting the need for methodological transparency. The CycloneSEQ library was prepared using the CycloneSEQ Universal DNA Library Prep Set kit (H940-000013), a commercially available kit optimized for long-read sequencing. The full protocol and reagent specifications are available

in the MGI Technical Documentation:
(https://en.mgitech.cn/Home/Products/instruments_info/id/65.html).

1-4 I cannot find the procedure for preparation and sequencing of the long-read cDNA libraries.
Response
We apologize for any ambiguity in the original manuscript and appreciate your request for clarification. The long-read cDNA libraries were prepared using the MGI CycloneSEQ RNA Library Prep Kit, following the manufacturer's optimized protocol. For specific and detailed methodological descriptions, please refer to the Methods section "CycloneSEQ long-read RNA-seq library preparation and sequencing". (Lines 587-596)

1-5 How many flowcells were needed to generate the different datasets?
Response
We thank you for raising this important technical detail. The number of flowcells used to generate each dataset has been thoroughly documented in Table S1 of the Supplementary Materials (Table R1.7).

Table R1.7 The number of flowcells for different datasets.
Data type Sample/Tissue Flowcell number
CycloneSEQ long-read WGS data muscle and lung 36
CycloneSEQ based Pore-C data liver 6
CycloneSEQ long-read RNA-seq data eye 3
kidney 4
live 3
lung 3
muscle 4
testis 3
brain 5
heart 3

1-6 How do the read-length distributions look like (statistics over all reads not only selected 40Kb+)?
Response
We thank you for requesting clarity on read-length distributions. As show in Figure R1.8, the full distribution of all raw CycloneSEQ reads (prior to size selection), with a N50 of 32.4 kb and over 40% of reads exceed 40 kb.

Figure R1.8 Frequency distribution of read lengths in all data.

1-7 How was the variability between those runs, especially cumulative output over time?
Response
We thank you for the highlighting the importance of sequencing run consistency. The sequencing for this study were primarily conducted using the WT platform of CycloneSEQ, a platform optimized for moderate-throughput applications. For the majority of run, the WT platform consistently generated 15~20 Gb of data per flowcell, with minimal variability across experimental batches. The WT platform's reliability in delivering stable yields within the 15~20 Gb range ensured robust data generation for the core assembly and sex chromosome analyses.

1-8 What hardware was needed to run the basecalling and what was the runtime?
Response
Basecalling for CycloneSEQ runs was performed in real-time during sequencing using the integrated computational module of the WT instrument. The process utilized a dedicated hardware configuration comprising an Intel i7/i9 CPU (24 cores), 64 GB DDR4 RAM, a NVIDIA RTX 4090D GPU, and a 512 GB NVMe system drive, which collectively enabled simultaneous signal acquisition and basecalling without delaying the sequencing workflow. Each run on the WT platform completed within 24 hours, including both sequencing and Basecalling. This integrated approach ensured rapid data availability while maintaining high accuracy.

1-9 How is the Q-Value distribution of the reads?
Response

The Q-value distribution of raw CycloneSEQ reads in shown in Figure R1.9.

Figure R1.9 Frequency count distribution of Q-value for reads in all data.

1-10 Why is the consensus accuracy of the assembly low (Q36.4)? can it be improved? Typically reference quality genomes should have Q40+.
Response:
We thank you for raising the critical question regarding the consensus accuracy (QV=36.4) of our assembly. The observed QV value primarily stems from the challenges inherent to assembling this species' highly heterozygous genome, which exhibits extensive allelic divergence between homologous chromosomes. During the assembly process, regions of high heterozygosity often led to erroneous merging of divergent haplotypes into single contigs. These artifacts, misinterpreted as sequencing errors during quality assessment, disproportionately inflated base-level discordance and reduced the overall QV score.

1-11 Which regions of the genome display lower consensus accuracies (is it random or sequence specific)?
Response:
By assembly-specific 21-bp k-mers distribution feature, we find that the assembly errors are random rather than sequence specific. As demonstrated in Figure R1.10, we systematically mapped assembly-specific 21-bp k-mers (entirely absent in raw WGS data) across the genome. Their uniform spatial distribution provides definitive evidence that assembly errors are randomly distributed. This pattern strongly argues against locus-specific technical biases in genome reconstruction.

Figure R1.10 The frequency distribution of assembly-specific 21-bp kmers (absent in raw WGS data) within 500-kb windows (Right) and the repeat density (Left).

Minor comments:
1-12 L.900: PRJNAxxxxxx looks like a placeholder, insert the true number,please.
Response
Thanks. We have replaced the placeholder "PRJNAxxxxxx" with PRJNA1184386 in Line 923. Morever, all sequencing data of 14 additional samples used for sex determination analysis have been deposited in public databases (NCBI: PRJNA1184386; CNSA: CNP0005509) to ensure transparency and reproducibility.

Point to point response for Reviewer #2
Reviewer #2: Impressive work but needs major revision to be accepted. The authors compressed everything in the result section and did not put enough effort into the other sections. Introduction and discussion need major changes and more details regarding many aspects of the study that comes in the results. Methods need rearrangement. It's common to keep the order of methods such as first DNA extraction, then sequencing and so on. The data availability needs to be completed. Biosamples for each sequenced tissue, all the reads, and even intermediate assemblies need to be submitted to the database and reported in the manuscript. More specific comments are on the copy of the manuscript attached for the authors.
Response
We sincerely thank you for the positive assessment of our work and for the thoughtful, detailed feedback that has significantly strengthened the manuscript. We will proceed to reply to each comment marked by the reviewer in the manuscript one by one. Finally, all the revised parts in the manuscript have been highlighted in dark red.

2-1 Software and database versions are missing throughout the manuscript.
Response
Thank you for highlighting the importance of software and database version transparency for reproducibility. We have rigorously updated the Methods section to include full version details for all software and databases used in this study, aligning with GigaScience's reporting standards.

2-2 The background description is too poor.
Response
Thank you for your feedback. Upon review, we agree that it was insufficient. We have now rewritten the

background section to provide a more comprehensive and detailed description.

2-3 Lines 29 “N50 of 202.5 Mb and all contigs anchored”: Explain if it's any better than previous genomes published for this organism.
Response
We appreciate your suggestion. We have added key assembly improvement metrics in the Abstract: “The new reference genome is ~1.8 Gb, shows a 5,700-fold improvement in contiguity (N50 = 202.5 Mb vs 35.5 kb) and complete chromosomal anchoring (16 scaffolds vs. 13,749).” (Lines 30-32)

2-4 The conclusions are too short.
Response
Thank you for your feedback. Due to word count limitations (not exceed 250 words), the conclusions were kept concise. However, we will expand on the key findings and implications in the revised manuscript to provide a more comprehensive summary.

2-5 Introduction does not include many info that is needed.
Response
Thank you for your feedback. As per your suggestion, we have added more relevant information and removed unnecessary details. We have also reorganized the paragraphs accordingly, with significant revisions made to the first paragraph of the Introduction to better align with your expectations.

2-6 Lines 46 “In the wild, P. vitticeps has a natural distribution in the eastern and central Australia.” Ambiguous
Response
Thank you for your feedback. Following your suggestion regarding the Introduction, we have reviewed the sentence in Line 46 and agree that it is ambiguous and not essential to the main content. Therefore, we have removed it from the revised manuscript.

2-7 Lines 48-50 “It is one of the most popular reptile pets nowadays, owing to ease of breeding in captivity, presence of morphs varying in scale coloration, and docility to their owners.” Unrelated
Response
Thank you for your feedback. We have removed the sentence in the revised manuscript to ensure the content remains focused and aligned with the main theme.

2-8 Lines 54-56 “as it possesses a ZZ/ZW genetic sex determination system whereas the final sex is also influenced by environmental temperature during embryonic development.” Break it into a separate sentence
Response
Thank you for your feedback. We have addressed the sentence in Lines 54-56 by splitting it and reorganizing the content for better clarity and flow.

2-9 Lines 109-114 “probably due to the well-known challenge of assembling the highly repetitive telomeric regions [7] which was also evidenced by the prevalent absence of telomeric sequences in the chromosomal ends of most squamate genomes published so far (Supplementary Table S9). However, we were able to find many CycloneSEQ reads containing TRUs before assembling, suggesting that the missing of telomeres are mainly due to computational drawback rather than sequencing omission.” move to discussion
Response
We thank you for the constructive suggestion. As recommended, the highlighted text has been relocated to the Discussion section (now Lines 438-446) to better contextualize telomere assembly challenges within broader genome assembly limitations. This adjustment improves the manuscript’s logical flow by positioning technical limitations within comparative biological interpretations.

2-10 Lines 132-133“our Pvit2024 assembly outperformed all other squamate genomes reported so far in terms of continuity” add discussion
Response
We appreciate this suggestion. The statement regarding assembly continuity has been incorporated into the Discussion (Lines 436-438).

2-11 Lines 152 “7,480 Sauropsida conserved genes” more details. did you find them?
Response
Thank you for raising this critical point. We have clarified (Lines 152-153) that the 7480 conserved genes derive from the Sauropsida_odb10 BUSCO dataset, representing single-copy orthologs shared across birds, squamates, and turtles.

2-12 Lines 162 “good agreements” What does this mean? "Good" needs to be replaced.
Response
We sincerely thank you for the precise suggestion to improve terminology clarity. In response, the phrase “good agreements” has been replaced with “high consistency” to objectively describe the Hi-C validation results.

2-13 Lines 169 cite more studies that published microchromosomes in lizards.
Response
We thank you for emphasizing the importance of contextualizing our findings within the broader literature on reptilian microchromosomes. In response, we have added three key references to the revised manuscript that explicitly discuss microchromosomes in squamates [8-10].

2-14 Lines 174 “GC content, lower repeat content, and more frequent inter-chromosomal interaction” Why do we care?
Response
We thank you for raising this important point. The three microchromosomal features highlighted in Lines 174 — GC content, repeat content, and inter-chromosomal interaction patterns — serve as established quality metrics in Sauropsida genome assembly studies. Here, we report these parameters to objectively validate the completeness and accuracy of our assembly, rather than to explore their biological implications.

2-15 Lines 175-176 “These multiple lines of evidence together highlight the reliability of the Pvit2024 chromosomal assembly.” Move to discussion
Response
We thank you for this suggestion. The sentence highlighting assembly reliability has been moved to the Discussion (Lines 447-450) and expanded with supporting validation data. This revision consolidates evidence for assembly quality within the broader genomic context.

2-16 Microchromosome background info: add background for any concept that comes to result section.
Response
Thank you for your suggestion. Detailed background information on “Microchromosome” has been added to the Introduction section to enhance clarity and provide context.

2-17 Lines 439-445 “P. vitticeps possesses a ZZ male/ZW female genetic sex determination (GSD) system. However, the default sexual fate of ZZ embryos can be overwritten by high incubation temperature, leading to sex reversal of ZZ males to ZZ females [11]. The capacity of a ZZ embryo to develop as a normal female without the help of W chromosome suggests that sexual fate is most likely determined by a dosage-sensitive gene on the Z chromosome [12]. Therefore, the achievement of a complete Z chromosome sequence represents a particularly important beginning for disclosing the sex-determining cascade in P. vitticeps. " This needs to go to introduction.
Response
Thank you for your suggestion. We have incorporated the content from Lines 439-445 into the first paragraph of the Introduction to provide a more comprehensive background and context for the study.

2-18 Lines 516-517 BioSample ID?
Response
Thank you for your attention! The BioSample information for the corresponding samples is presented in the table below (Table R2.1). Additionally, we have included this information in the supplementary materials for readers’ reference. Please note that there are some issues with the CycloneSEQ data upload to NCBI, which are currently being resolved. However, the corresponding data are fully accessible and available for

download in the CNGBdb database.
BioSample ID information have been added to Supplementary Table S1.

Table R2.1 BioSample ID for all uploaded datasets.
Data type Instrument/Platform Sample/Tissue NCBI
Biosample No. NCBI
Accession No. CNSA
Accession No.
CycloneSEQ long-read RNA-seq data CycloneSEQ eye SAMN44670089 *** CNS1198628
kidney SAMN44670090 *** CNS1198630
live SAMN44670091 *** CNS1198631
lung SAMN44670092 *** CNS1198632
muscle SAMN44670093 *** CNS1198633
testis SAMN44670094 *** CNS1198634
brain SAMN44670095 SRR31361862 CNS1198627
heart SAMN44670096 SRR31361861 CNS1198629
DNBSEQ short-read RNA-seq data DNBSEQ brain SAMN44670097 SRR31361884 CNS1065805
eye SAMN44670098 SRR31361883 CNS1065806
heart SAMN44670099 SRR31361882 CNS1065807
kidney SAMN44670100 SRR31361881 CNS1065808
liver SAMN44670101 SRR31361880 CNS1065809
lung SAMN44670102 SRR31361879 CNS1065810
muscle SAMN44670103 SRR31361878 CNS1065811
testis SAMN44670104 SRR31361877 CNS1065812

2-19 Lines 533 Keep the order of the sections in a rational order. First extraction.
Response
We thank you for emphasizing the importance of methodological clarity. The Methods section has been restructured to follow a logical workflow, beginning with Sample collection, followed by different kinds of library preparation and sequencing (include CycloneSEQ long-read WGS data, CycloneSEQ based Pore-C data and CycloneSEQ long-read RNA-seq data). This reorganization aligns with best practices in genomics reporting and enhances reproducibility.

2-20 Lines 614 All reads and intermediate assemblies need their database information. (SRR?)
Response
We thank you for emphasizing the importance of open data access. All sequencing data have been deposited in NCBI and CNSA. The detail information can be found in Table R2.1 and Supplementary Table S1. And the intermediate assemblies have been deposited in GigaDB database.

Table R2.2 The accession ID of NCBI and CNSA for all uploaded datasets
Data type Instrument/Platform Sample/Tissue Accession (NCBI) Accession (CNSA)

CycloneSEQ long-read WGS data CycloneSEQ muscle and lung SAMN44670087 CNS1198625
DNBSEQ short-read WGS data DNBSEQ muscle of neck SRR31361869/SRR31361868 CNS1065816
muscle of tail SRR31361871/SRR31361870 CNS1065815
CycloneSEQ based Pore-C data CycloneSEQ liver SRR31361885
CNS1198626
Hi-C data DNBSEQ muscle (Hi-C_1 data) SRR31361875/SRR31361876
CNS1065813
muscle (Hi-C_2 data) SRR31361872/SRR31361873
CNS1065814
CycloneSEQ long-read RNA-seq data CycloneSEQ eye SAMN44670089 CNS1198628
kidney SAMN44670090 CNS1198630
live SAMN44670091 CNS1198631
lung SAMN44670092 CNS1198632
muscle SAMN44670093 CNS1198633
testis SAMN44670094 CNS1198634

brain SRR31361862 CNS1198627
heart SRR31361861 CNS1198629
DNBSEQ short-read RNA-seq data DNBSEQ brain SRR31361884 CNS1065805
eye SRR31361883 CNS1065806
heart SRR31361882 CNS1065807
kidney SRR31361881 CNS1065808
liver SRR31361880 CNS1065809
lung SRR31361879 CNS1065810
muscle SRR31361878 CNS1065811
testis SRR31361877 CNS1065812

2-21 Lines 681 "homologous" more details.
Response
We thank you for highlighting the need for methodological precision. The revised manuscript (Methods – Repetitive element annotation, Lines 698-702) now provides comprehensive details on the homology-based annotation pipeline.

2-22 Lines 709 "winnowmap -u b -K 4G -G 135k -ax splice ref fastq." No italic
Response
Thanks. We have revised it accordingly (Line 729).

2-23 Lines 900 PRJNAxxxxxx
Response
Thanks. We have replaced the placeholder "PRJNAxxxxxx" with PRJNA1184386 in Line 923.

References:

1. Quinn, A.E., et al., Extension, single-locus conversion and physical mapping of sex chromosome sequences identify the Z microchromosome and pseudo-autosomal region in a dragon lizard, Pogona vitticeps. Heredity, 2010. 104(4): p. 410-417.
2. Long, X., et al., Independent evolution of sex chromosomes and male pregnancy–related genes in two seahorse species. Molecular Biology and Evolution, 2023. 40(1): p. msac279.
3. Purcell, S., et al., PLINK: a tool set for whole-genome association and population-based linkage analyses. The American journal of human genetics, 2007. 81(3): p. 559-575.
4. Garrison, E. and G. Marth, Haplotype-based variant detection from short-read sequencing. arXiv preprint arXiv:1207.3907, 2012.
5. Manichaikul, A., et al., Robust relationship inference in genome-wide association studies. Bioinformatics, 2010. 26(22): p. 2867-2873.
6. Zhang, J.-Y., et al., A single-molecule nanopore sequencing platform. bioRxiv, 2024: p. 2024.08. 19.608720.
7. Bresler, M.a., et al., Telescoper: de novo assembly of highly repetitive regions. Bioinformatics, 2012. 28(18): p. i311-i317.
8. Pinto, B.J., et al., A lizard is never late: squamate genomics as a recent catalyst for understanding sex chromosome and microchromosome evolution. Journal of Heredity, 2023. 114(5): p. 445-458.
9. Waters, P.D., et al., Microchromosomes are building blocks of bird, reptile, and mammal chromosomes. Proceedings of the National Academy of Sciences, 2021. 118(45): p. e2112494118.
10. Lisachov, A.P. and P.M. Borodin, Microchromosome polymorphism in the sand lizard, Lacerta agilis Linnaeus, 1758 (Reptilia, Squamata). Comparative Cytogenetics, 2016. 10(3): p. 387.
11. Whiteley, S.L., et al., Two transcriptionally distinct pathways drive female development in a reptile with both genetic and temperature dependent sex determination. PLoS Genetics, 2021. 17(4): p. e1009465.
12. Quinn, A.E., et al., Temperature sex reversal implies sex gene dosage in a reptile. Science, 2007. 316(5823): p. 411-411.

Cookie Preference Center

We use cookies which are necessary to make our site work. We may also use additional cookies to analyze, improve and personalize our content and your digital experience. For more information, see our Cookie Policy.

You may choose not to allow some types of cookies. However, blocking some types may impact your experience of our site and the services we are able to offer. See the different category headings below to find out more or change your settings.

Allow all

Manage Consent Preferences

Strictly Necessary Cookies

Always active

These cookies are necessary for the website to function and cannot be switched off in our systems. They are usually only set in response to actions made by you which amount to a request for services, such as setting your privacy preferences, logging in or filling in forms. You can set your browser to block or alert you about these cookies, but some parts of the site will not then work.

Functional Cookies

These cookies enable the website to provide enhanced functionality and personalisation. They may be set by us or by third party providers whose services we have added to our pages. If you do not allow these cookies then some or all of these services may not function properly.

Performance Cookies

These cookies allow us to count visits and traffic sources so we can measure and improve the performance of our site. They help us to know which pages are the most and least popular and see how visitors move around the site.

Targeting Cookies

These cookies may be set through our site by our advertising partners. They may be used by those companies to build a profile of your interests and show you relevant adverts on other sites. If you do not allow these cookies, you will experience less targeted advertising.

Cookie List

Clear

Apply Cancel
Consent Leg.Interest

Confirm my choices

Powered by onetrust
