# Supplementary material for: A near-complete genome assembly of the bearded dragon Pogona vitticeps provides insights into the origin of Pogona sex chromosomes
Source: Gigascience. 2025 Aug 19;14:giaf079. doi: 10.1093/gigascience/giaf079 (PMC12360845; doi:10.1093/gigascience/giaf079)
Supplement: giaf079_Authors_Response_To_Reviewer_Comments_Revision_1 [file giaf079_authors_response_to_reviewer_comments_revision_1.pdf]

Author's Response To Reviewer Comments

Reponse to Reviewer1: Thank you for your positive feedback and understanding regarding the scope of our manuscript. We appreciate your valuable comments and suggestions.

Point to point response for Reviewer2' Comments:
Reviewer #2: Though the authors provided more details, still more elaborated background review is needed for introduction. For example, a major part of analysis is focused on Telomeres but there is no mention of their importance in introduction.
Response
We appreciate your suggestion. We have added the details of telomeres in introduction. (Lines 71-73)

Writing format and structure needs improvement. Please go through your manuscript and edit the structure by considering these examples:
Line 77: Result details belong to result section.
Response
Thank you for your feedback. We have removed the sentence in the revised manuscript.

Data format/Tables are impossible to follow. Please reformat into a readable version.
Response
We suspect that the issue might be due to the fact that you downloaded a consolidated PDF file containing all the content, which may have compromised the readability of the tables. In reality, we have submitted the data in highly readable Excel file formats. If you need to access the original files, you can download the corresponding excel files through the GigaScience system's File Inventory.

Choose a format for abbreviation and stick to it. For example: line 112 versus line 151, either use capital for the start of the letters or don't.
Response
We have carefully reviewed the abbreviation formats and have clarified the capitalization as follows: BUSCO in line 151 is capitalized as it is a proper noun and widely used in this format in the literature [1]. TRUs in line 112 is not capitalized as it is not a proper noun. Finally, we have ensured consistency in the manuscript.

All tables and figures have to be referred in a numeric order in the text. In the result section you jumped from S1 to S7.
Response
We have carefully reviewed and reorganized all tables and figures to ensure they are numbered sequentially in the order they are mentioned in the manuscript. This should now provide a clear and logical flow for the reader.

1. Huang, N. and H. Li, miniBUSCO: a faster and more accurate reimplementation of BUSCO. BioRxiv, 2023: p. 2023.06. 03.543588.

Cookie Preference Center

We use cookies which are necessary to make our site work. We may also use additional cookies to analyze, improve and personalize our content and your digital experience. For more information, see our Cookie Policy.

You may choose not to allow some types of cookies. However, blocking some types may impact your experience of our site and the services we are able to offer. See the different category headings below to find out more or change your settings.

Allow all

Manage Consent Preferences

Strictly Necessary Cookies

Always active

These cookies are necessary for the website to function and cannot be switched off in our systems. They are usually only set in response to actions made by you which amount to a request for services, such as setting your privacy preferences, logging in or filling in forms. You can set your browser to block or alert you about these cookies, but some parts of the site will not then work.

Functional Cookies

These cookies enable the website to provide enhanced functionality and personalisation. They may be set by us or by third party providers whose services we have added to our pages. If you do not allow these cookies then some or all of these services may not function properly.

Performance Cookies

These cookies allow us to count visits and traffic sources so we can measure and improve the performance of our site. They help us to know which pages are the most and least popular and see how visitors move around the site.

Targeting Cookies

These cookies may be set through our site by our advertising partners. They may be used by those companies to build a profile of your interests and show you relevant adverts on other sites. If you do not allow these cookies, you will experience less targeted advertising.

Cookie List

Clear

Apply Cancel
Consent Leg.Interest

Confirm my choices

Powered by onetrust
