# Supplementary material for: A near-complete genome assembly of the bearded dragon Pogona vitticeps provides insights into the origin of Pogona sex chromosomes
Source: Gigascience. 2025 Aug 19;14:giaf079. doi: 10.1093/gigascience/giaf079 (PMC12360845; doi:10.1093/gigascience/giaf079)
Supplement: giaf079_Reviewer_1_Report_Original_Submission — Heiner Kuhl -- 11/15/2024 [file giaf079_reviewer_1_report_original_submission.pdf]

Reviewer Report

Title: A near-complete genome assembly of the bearded dragon Pogona vitticeps provides insights into the origin of Pogona sex chromosomes

Version: Original Submission **Date:** 11/15/2024

Reviewer name: Heiner Kuhl

Reviewer Comments to Author:

Guo et al. present a new reference genome for Pogona vitticeps, a widespread reptile model organism that is also common as a domestic animal worldwide. The genome assembly shows much improvement over an older assembly from 2017. There are two points that make this manuscript outstanding from common genome assembly papers:
1. The authors find a new sex determination locus in this species.
2. the authors use a new nanopore sequencing technology ("CycloneSEQ"), which has so far only described in a preprint (https://www.biorxiv.org/content/10.1101/2024.08.19.608720v1).
In my opinion this deserves a publication in Gigascience, but both points must be focused more in a revised manuscript.
Major comments:
1) The authors have sequenced a male individual (ZZ), which means the long-read reference assembly is missing the W-chromosome. PAR and SDR regions are deduced from the Z sequence, by analysis of sequencing coverage of only a few sexed samples (2 females and 4 males). It is unclear if these individuals are from the same family, which could mean that the newly found SD-region could just be a family specific variation. To make the whole story more intriguing and statistical sound the authors should at least test 15 males and 15 females from different P. vitticeps populations for W-specific markers near the proposed AMH deletion. The authors should also show that the prior proposed SD locus (nr5a1) does not carry W-specific mutations in these 15+15 individuals. Furthermore, a phased assembly of a female (ZW) Pogona vitticeps individual, could enable the assembly of the missing W-chr and should be included, it would even improve analysis of W-specific sequences in the proposed additional individuals.
2) A technology aware reader would like to see more information on the specifics of the CycloneSEQ data quality and handling and maybe a comparison to competing technologies.
Which enzymes and buffers were used to prepare the library? In the sections on the methods, there are only superficial descriptions such as (DNA repair buffer/enzyme, DNA clean beads, wash buffer for long fragments). Is it a kit or were the enzymes and buffers purchased individually?
I cannot find the procedure for preparation and sequencing of the long-read cDNA libraries.
How many flowcells were needed to generate the different datasets?
How do the read-length distributions look like (statistics over all reads not only selected 40Kb+)?
How was the variability between those runs, especially culmulative output over time?
What hardware was needed to run the basecalling and what was the runtime?
How is the Q-Value distribution of the reads?

Why is the consensus accuracy of the assembly low (Q36.4)? can it be improved? Typically reference quality genomes should have Q40+.
Which regions of the genome display lower consensus accuracies (is it random or sequence specific)?
Minor comments:
L.900: PRJNAxxxxxx looks like a placeholder, insert the true number,please.

Methods

Are the methods appropriate to the aims of the study, are they well described, and are necessary controls included? Choose an item.

Conclusions

Are the conclusions adequately supported by the data shown? Choose an item.

Reporting Standards

Does the manuscript adhere to the journal's guidelines on minimum standards of reporting? Choose an item.

Choose an item.

Statistics

Are you able to assess all statistics in the manuscript, including the appropriateness of statistical tests used? Choose an item.

Quality of Written English

Please indicate the quality of language in the manuscript: Choose an item.

Declaration of Competing Interests

Please complete a declaration of competing interests, considering the following questions:

- Have you in the past five years received reimbursements, fees, funding, or salary from an organisation that may in any way gain or lose financially from the publication of this manuscript, either now or in the future?
- Do you hold any stocks or shares in an organisation that may in any way gain or lose financially from the publication of this manuscript, either now or in the future?
- Do you hold or are you currently applying for any patents relating to the content of the manuscript?
- Have you received reimbursements, fees, funding, or salary from an organization that holds or has applied for patents relating to the content of the manuscript?

- Do you have any other financial competing interests?
- Do you have any non-financial competing interests in relation to this paper?

If you can answer no to all of the above, write 'I declare that I have no competing interests' below. If your reply is yes to any, please give details below.

I declare that I have no competing interests

I agree to the open peer review policy of the journal. I understand that my name will be included on my report to the authors and, if the manuscript is accepted for publication, my named report including any attachments I upload will be posted on the website along with the authors' responses. I agree for my report to be made available under an Open Access Creative Commons CC-BY license (http://creativecommons.org/licenses/by/4.0/). I understand that any comments which I do not wish to be included in my named report can be included as confidential comments to the editors, which will not be published.

Choose an item.

To further support our reviewers, we have joined with Publons, where you can gain additional credit to further highlight your hard work (see: https://publons.com/journal/530/gigascience). On publication of this paper, your review will be automatically added to Publons, you can then choose whether or not to claim your Publons credit. I understand this statement.

Yes Choose an item.
